# Integrated transcriptomics- and structure-based drug repositioning identifies drugs with proteasome inhibitor properties

**DOI:** 10.1038/s41598-024-69465-6

**Published:** 2024-08-13

**Authors:** Peter Larsson, Maria Cristina De Rosa, Benedetta Righino, Maxim Olsson, Bogdan Iulius Florea, Eva Forssell-Aronsson, Anikó Kovács, Per Karlsson, Khalil Helou, Toshima Z. Parris

**Affiliations:** 1 Department of Oncology, Institute of Clinical Sciences, Sahlgrenska Academy, University of Gothenburg, Gothenburg, Sweden; 2 Sahlgrenska Center for Cancer Research, Sahlgrenska Academy, University of Gothenburg, Gothenburg, Sweden; 3 Institute of Chemical Sciences and Technologies “Giulio Natta” (SCITEC)-CNR, Rome, Italy; 4 Gorlaeus Laboratories, Leiden Institute of Chemistry and Netherlands Proteomics Center, Leiden, The Netherlands; 5 Department of Medical Radiation Sciences, Institute of Clinical Sciences, Sahlgrenska Academy, University of Gothenburg, Gothenburg, Sweden; 6 Department of Medical Physics and Biomedical Engineering, Sahlgrenska University Hospital, Gothenburg, Sweden; 7 Department of Clinical Pathology, Sahlgrenska University Hospital, Gothenburg, Sweden; 8 Department of Oncology, Sahlgrenska University Hospital, Gothenburg, Sweden

**Keywords:** Transcriptomic signature, Molecular docking, Drug screening, Drug discovery, Drug mechanism-of-action, Antineoplastic agents, Undescribed proteasome inhibitor, Cancer genomics, Chemotherapy

## Abstract

Computational pharmacogenomics can potentially identify new indications for already approved drugs and pinpoint compounds with similar mechanism-of-action. Here, we used an integrated drug repositioning approach based on transcriptomics data and structure-based virtual screening to identify compounds with gene signatures similar to three known proteasome inhibitors (PIs; bortezomib, MG-132, and MLN-2238). In vitro validation of candidate compounds was then performed to assess proteasomal proteolytic activity, accumulation of ubiquitinated proteins, cell viability, and drug-induced expression in A375 melanoma and MCF7 breast cancer cells. Using this approach, we identified six compounds with PI properties ((-)-kinetin-riboside, manumycin-A, puromycin dihydrochloride, resistomycin, tegaserod maleate, and thapsigargin). Although the docking scores pinpointed their ability to bind to the β5 subunit, our in vitro study revealed that these compounds inhibited the β1, β2, and β5 catalytic sites to some extent. As shown with bortezomib, only manumycin-A, puromycin dihydrochloride, and tegaserod maleate resulted in excessive accumulation of ubiquitinated proteins and elevated HMOX1 expression. Taken together, our integrated drug repositioning approach and subsequent in vitro validation studies identified six compounds demonstrating properties similar to proteasome inhibitors.

## Introduction

The de novo anticancer drug discovery process is time-consuming (can take about 12–15 years from discovery to approval), cost ineffective (costs around $2.5 billion per drug), and high-risk (around 95% anticancer drug attrition rates)^1–4^ for pharmaceutical companies^5,6^. Drug repurposing or drug repositioning (DR), i.e. the process of identifying novel clinical indications for drugs that have already been approved by the US Food and Drug Administration (FDA) and/or the European Medicines Agency (EMA), has therefore become an attractive alternative to the de novo drug discovery process in oncology. DR is not only comparatively cheaper (1/3 of the cost) and quicker (can take around 3–9 years)^5^, but can also potentially discover treatments for cancer forms currently lacking effective therapeutic options or rare cancers^6^. Two main DR approaches utilize (1) the desirable or undesirable side effects of a particular drug to treat another disease and (2) high-throughput screening of cancer cell lines with large compound libraries to discover potent cytotoxic drugs for a specific cancer type^7^. Notably, new clinical indications for acetylsalicylic acid (aspirin), tamoxifen, and sildenafil were found using the “drug side effect” approach^8^. Computational DR based on drug structure or cellular responses to drug treatment has also become increasingly popular due to the vast amount of publicly available data from medicinal chemistry and drug-associated genomic/transcriptomic profiling (e.g. The connectivity map [CMap] or library of integrated network-based cellular signatures [LINCS])^9^. Virtual screening using ligand-based^10,11^, structure-based^12,13^, or combined strategies^14,15^ has therefore emerged as a powerful tool to identify new therapeutic candidates among drugs that were approved for different indications^16^.

The ubiquitin-proteasome system (UPS) is responsible for the degradation of 80–90% of defective, misfolded, and unneeded proteins. The UPS is found in all eukaryotic cells and consists of three enzymes (ubiquitin activation enzyme [E1], ubiquitin-conjugating enzyme [E2], and ubiquitin-protein ligase [E3]) responsible for tagging proteins with ubiquitin molecules (ubiquitination) that will ultimately be recognized and degraded by the 26S proteasome^17^. The UPS is therefore pivotal for intracellular protein homeostasis, regulation of cellular processes (e.g. cell cycle, DNA repair, and drug resistance), recycling amino acids to produce new proteins, and cellular adaptation to different conditions^18,19^. The 26S proteasome consists of one core particle (20S) and one or two regulatory particles (19S) that form a barrel-like structure containing three pairs of catalytic sites (β1 [caspase-like], β2 [trypsin-like], and β5 [chymotrypsin-like]) responsible for the degradation process^19^. Elevated proteasome activity is relatively common in cancer cells, thereby often playing a pivotal role in tumorigenesis and tumor cell survival^18^. The proteasome has therefore become an attractive target for cancer therapy with proteasome inhibitors (PI), as PIs will disrupt protein homeostasis and lead to apoptosis^18,20^.

In 2003, bortezomib (VELCADE®, formerly PS-341) was the first PI to be approved by the FDA for use in the treatment of multiple myeloma; bortezomib later became first-line treatment in 2008^20^. Due to problems with treatment resistance, second generation PIs were subsequently developed and approved by the FDA and EMA for clinical use in the treatment of multiple myeloma and mantle cell lymphoma (carfilzomib [Kyprolis®, formerly PR-171; FDA approved in 2012] and ixazomib [Ninlaro®, formerly MLN-9708; FDA approved in 2015])^19,21^. These PIs mainly target the β5 chymotrypsin-like activity, but can also bind to the β1 and β2 catalytic sites at high doses^22^ either reversibly (bortezomib and ixazomib) or irreversibly (carfilzomib)^23,24^. Although PIs can effectively penetrate most tissues, problems have arisen with crossing the blood-brain barrier^23,25^. Therefore, there is a need for novel PIs that can bind to one or more of the proteasome catalytic sites with high affinity at low doses, cross the blood-brain barrier, overcome treatment resistance, and improve treatment efficiency. In recent years, Virtual Screening (VS) has gained much attention for hit identification because it can be implemented quickly and at a low cost. While ligand-based VS methods do not necessitate a 3D representation of the biological target^26,27^, structure-based VS does^28,29^. Here, we used an integrated DR approach, based on transcriptomic data and structure-based VS, to identify compounds that induce similar transcriptomic profiles as proteasome inhibitors (bortezomib, MG-132, and MLN-2238), bind to the β5 proteasome catalytic site, and are cytotoxic to cancer cells.

## Results

### Proteasome inhibition induces dysregulation of a 12-gene signature

**Figure 1:**
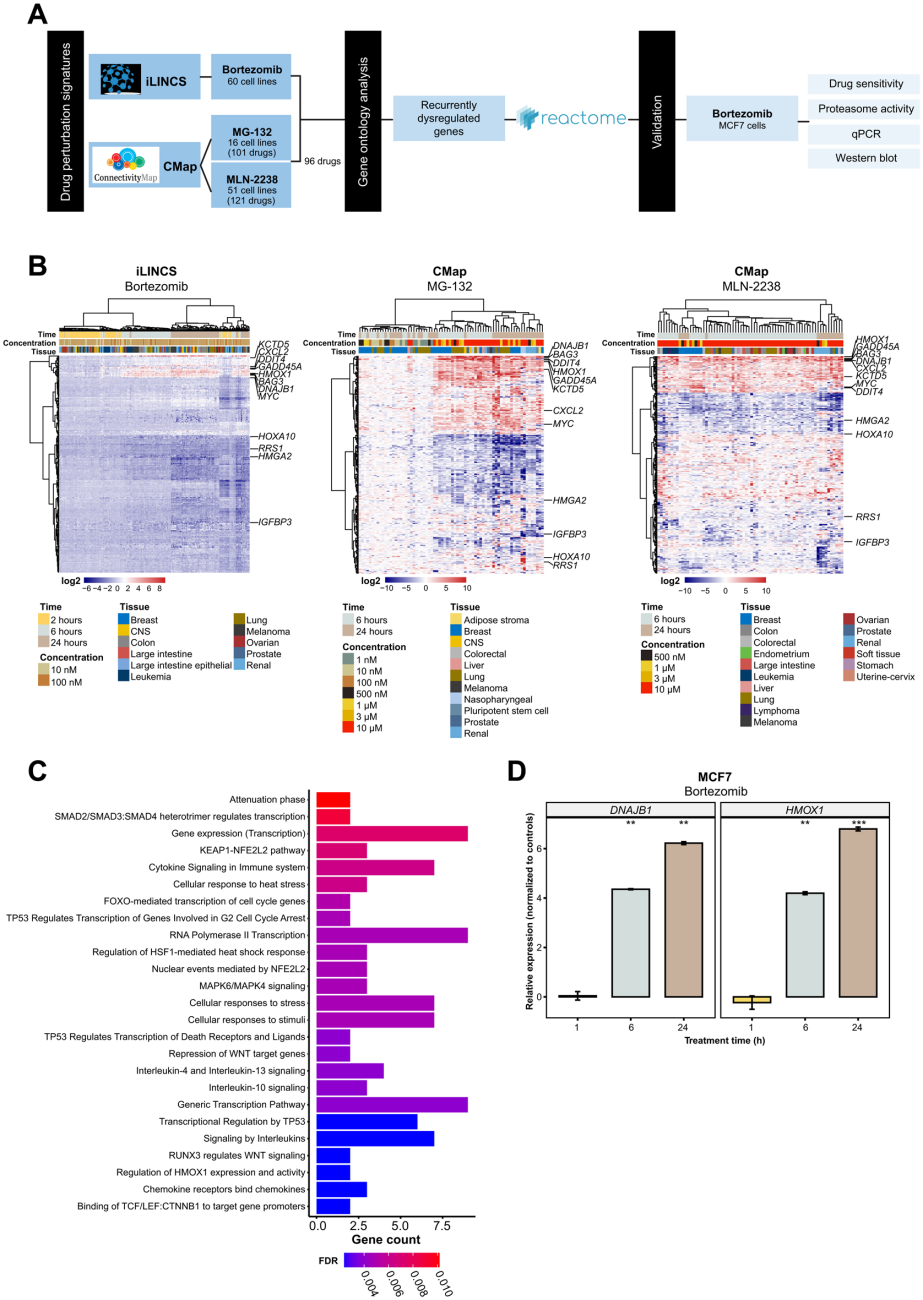
Proteasome inhibition induces time- and dose-related changes in gene expression. (**A**) Overview of the analysis pipeline to identify and validate recurrently dysregulated genes following proteasome inhibition. Perturbation-induced gene signatures were curated for bortezomib, MG-132, and MLN-2238 from the Library of Integrated Cellular Signatures (LINCS) Consortium (iLINCS and Connectivity Map [CMap]). (**B**) Heatmaps illustrating hierarchical clustering (Manhattan distance metric and Ward’s minimum variance method [Ward.D2]) of the top 250 differentially regulated genes for cell lines treated with proteasome inhibitors using datasets from iLINCS (bortezomib) and CMap (MG-132 and MLN-2238). The 12 recurrently dysregulated genes from the three drugs are shown. (**C**) Gene Ontology and Reactome enrichment analysis for the 12 recurrently dysregulated genes. The top 25 most significant pathways are shown. (**D**) Quantitative real-time PCR analysis confirms time-dependent expression of *DNAJB1* and *HMOX1* in MCF7 breast cancer cells treated with 10 µM bortezomib for 1-, 6-, and 24 h. Error bars depict the standard error of the mean. T-test was used to calculate statistical significance (Benjamini–Hochberg adjusted p-values) between the 1 h treatment time and the other time points. ns = not significant (*P* > 0.05); *P ≤ 0.05; **P ≤ 0.01; ***P ≤ 0.001; ****P ≤ 0.0001.

To identify transcriptomic signatures induced by proteasome inhibition, drug perturbation signatures were retrieved from the iLINCS and CMap web-based tools for cell lines treated with bortezomib (iLINCS), MG-132 (CMap), and/or MLN-2238 (CMap). An overview of the workflow is shown in (Fig. 1A). Subsequent analysis of the iLINCS dataset identified 5,448 differentially regulated genes between bortezomib-treated (10 and 100 nM) cell lines and controls, of which 11 genes (*ATF3*, *BAG3*, *DDIT3*, *DNAJB1*, *DNAJB4*, *GABARAPL1*, *GADD45A*, *HMOX1*, *HSPA6*, *HSPH1*, *PPP1R15A*) were consistently upregulated in cells treated for 6- and 24 h. In contrast, the 2 h bortezomib time point, drug concentration (10 or 100 nM), and cell line tissue of origin (breast, CNS, colon, large intestine, large intestine epithelial, leukemia, lung, melanoma, ovarian, prostate, renal) had little to no effect on the transcriptome (Fig. 1B and Supplementary Table 1). Although the 2 h exposure time was not tested in the CMap dataset, the inclusion of drug concentrations higher than 100 nM (up to 10 µM) revealed a clear dose–response effect on gene expression patterns, particularly following treatment with MG-132 (Supplementary Table 2, Supplementary Table 3). Despite differences between the two datasets (differences in PIs, treatment time, drug concentration, and cell lines), 12 genes (upregulated: *BAG3*, *CXCL2*, *DDIT4*, *DNAJB1*, *GADD45A*, *HMOX1*, *KCTD5*, *MYC*; downregulated: *IGFBP3*, *HMGA2*, *HOXA10*, *RRS1*) were consistently dysregulated. Gene ontology analysis showed that the 12 dysregulated genes play a pivotal role on immune response, transcriptional regulation by *TP53*, WNT signaling, regulation of the cell cycle, and cellular responses to stress (Fig. 1C).

To validate these findings, MCF7 cancer cells were treated with 10 µM bortezomib for 1, 6, and 24 h. Our previous work shows that 24 h treatment with bortezomib resulted in near complete suppression of proteasome activity at doses ≥ 100 nM, while the 50% inhibitory concentration (IC50) after treatment was approximately 229 nM in MCF7 cells^3^. Here, subsequent treatment of MCF7 cells with 10 µM bortezomib confirmed the time-dependent upregulation of *DNAJB1* and *HMOX1* expression (*P* < 0.05) in treated cells, with progressively higher expression of each gene over time (Fig. 1D).

### CMap data reveals drug-drug similarity with proteasome inhibitors

**Figure 2:**
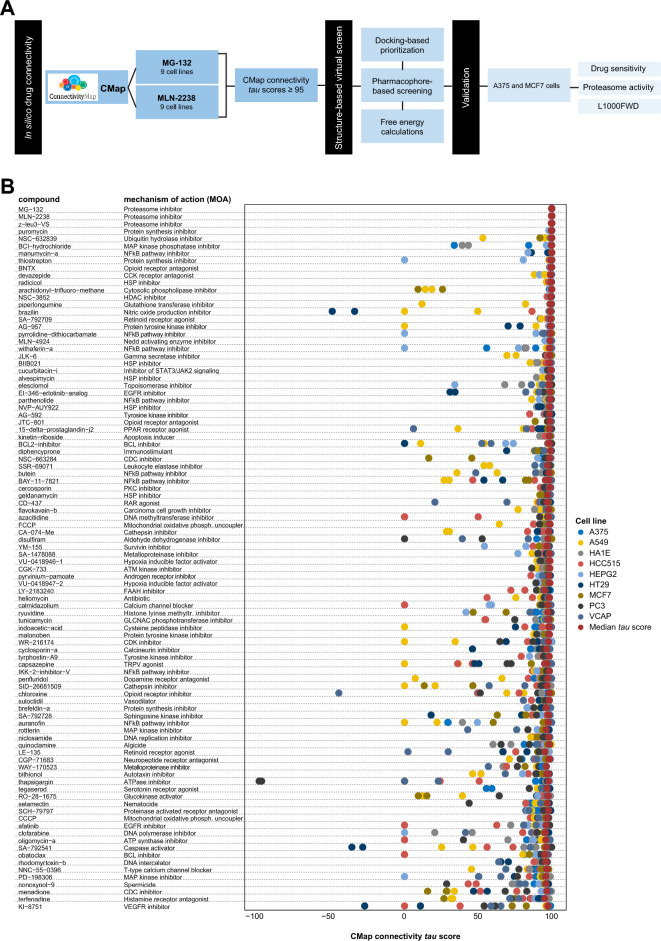
Integrated transcriptomics- and structure-based drug repositioning of proteasome inhibitors. (**A**) Overview of the integrated drug repositioning pipeline to identify compounds with proteasome inhibitor properties. (**B**) Dot plot depicting CMap connectivity *tau* scores for 96 common compounds with connections to MG-132 and MLN-2238 (median *tau* score ≥ 95).

**Figure 3:**
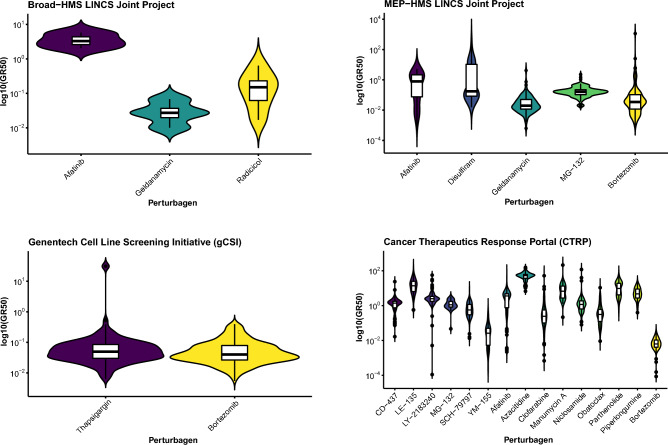
Dose-dependent sensitivity data (log10 GR50) from four datasets (retrieved from the GR metrics calculator and browser web-based tool) for nonmalignant and cancer cell lines treated with 18/96 identified compounds. Bortezomib has been included as a reference.

Given the similarity in gene expression patterns in cell lines treated with bortezomib, MG-132, and/or MLN-2238, we then used a CMap touchstone^30^ query for MG-132 and MLN-2238 (bortezomib not available) to identify other compounds with perturbagen-driven gene expression signatures similar to PIs and a median *tau* score ≥ 95 (Fig. 2A and Supplementary Table 4, Supplementary Table 5). This analysis subsequently identified 113 perturbagens (101 compounds and 12 gene knock-down) for MG-132 and 152 perturbagens (121 compounds and 31 gene knock-down) for MLN-2238, of which 107 (96 compounds and 11 gene knock-down) were common for both drugs (Table 1). A number of the 96 common compounds also had a similar mechanism-of-action (MOA) as PIs, *e.g.* apoptosis inducers (kinetin-riboside), BCL inhibitors (BCL2-inhibitor and obatoclax), endoplasmic reticulum stress inducers (thapsigargin), NFkB pathway inhibitors (auranofin, BAY-11-7821, butein, IKK-2-inhibitor-V, manumycin-A, parthenolide, pyrrolidine-dithiocarbamate, withaferin-a), protein synthesis inhibitor (puromycin), and ubiquitin hydrolase inhibitor (NSC-632839; Fig. 2B). Not surprisingly, 7/11 gene knock-downs showing strong connectivity (*tau* score ≥ 95) involved proteasome subunits (*PSMA1*, *PSMA3*, *PSMB2*, *PSMB5*, *PSMD1*, *PSMD3*) or ubiquitin genes (*UBC*). The remaining gene knock-downs included *EIF2S2* (Eukaryotic Translation Initiation Factor 2 Subunit Beta), *HSPA5* (Heat Shock Protein Family A [Hsp70] Member 5), *PHB2* (Prohibitin 2), and *VCP* (valosin containing protein). Evaluation of the *tau* scores revealed several compounds with relatively low *tau* scores (*tau* score < 75) in some cell lines, thereby implying diverse transcriptomic responses to drug exposure (Fig. 2B). In contrast, proteasome inhibitors and a few other compounds (*e.g.* puromycin, BNTX, radicicol, NSC−3852, BIIB021, NVP−AUY922, and AG−592) clearly showed very little variance in *tau* scores. To assess whether the identified compounds have antineoplastic activity, drug sensitivity data (GR50 values) for the 96 compounds were retrieved from the GR Metrics Calculator and Browser web-based tool. Data for only 18/96 compounds were available. Nevertheless, this analysis demonstrated that the 18 compounds indeed have antineoplastic activity (Fig. 3). Using bortezomib as a reference, the potency of geldanamycin, radicicol, and thapsigargin were found to be in line with bortezomib, whereas the other 15 compounds had higher GR50 values.

**Table 1:** Common CMap perturbagens (compounds or gene knock-down) with *tau* score ≥ 95 for MG-132 and MLN-2238.

Rank	Score	Type	ID	Name	Mechanism of action (MoA)	Clinical phase
1	99,98	Compound	BRD-K60230970	MG-132	Proteasome inhibitor	Preclinical
3	99,89	Compound	BRD-K78659596	MLN-2238	Proteasome inhibitor	
4	99,89	Compound	BRD-K15935639	z-leu3-VS	Proteasome inhibitor	
6	99,79	Compound	BRD-A28970875	puromycin	Protein synthesis inhibitor	Preclinical
8	99,75	Compound	BRD-K74402642	NSC-632839	Ubiquitin hydrolase inhibitor	Preclinical
10	99,68	Compound	BRD-K33551950	radicicol	HSP inhibitor	
11	99,65	Compound	BRD-K78599730	manumycin-A	NFkB pathway inhibitor	
12	99,65	Compound	BRD-K07303502	arachidonyl-trifluoro-methane	Cytosolic phospholipase inhibitor	
14	99,65	Compound	BRD-A11007541	BCI-hydrochloride	MAP kinase phosphatase inhibitor	
13	99,65	Compound	BRD-A55484088	BNTX	Opioid receptor antagonist	Preclinical
15	99,61	Compound	BRD-A20697603	thiostrepton	Protein synthesis inhibitor	Launched
20	99,44	Compound	BRD-K80970344	pyrrolidine-dithiocarbamate	NFkB pathway inhibitor	Preclinical
22	99,33	Compound	BRD-K51290057	SA-792709	Retinoid receptor agonist	
24	99,29	Compound	BRD-K36737713	AG-957	Protein tyrosine kinase inhibitor	
25	99,19	Compound	BRD-A28105619	cucurbitacin-i	Inhibitor of STAT3/JAK2 signaling	
26	99,08	Compound	BRD-K31238592	devazepide	CCK receptor antagonist	Preclinical
29	98,87	Compound	BRD-U08759356	EI-346-erlotinib-analog	EGFR inhibitor	
31	98,78	Compound	BRD-K14821540	FCCP	Mitochondrial oxidative phosphorylation uncoupler	
32	98,76	Compound	BRD-K89930444	AG-592	Tyrosine kinase inhibitor	
33	98,73	Compound	BRD-K44432556	VU-0418946-1	Hypoxia inducible factor activator	
37	98,7	Compound	BRD-K24132293	piperlongumine	Glutathione transferase inhibitor	
40	98,7	Compound	BRD-K13169950	NSC-3852	HDAC inhibitor	Preclinical
39	98,7	Compound	BRD-K17705806	JTC-801	Opioid receptor antagonist	Phase 2
38	98,7	Compound	BRD-K22010301	JLK-6	Gamma secretase inhibitor	Preclinical
41	98,7	Compound	BRD-A52193669	withaferin-a	NFkB pathway inhibitor	
36	98,7	Compound	BRD-K76907295	VU-0418947-2	Hypoxia inducible factor activator	
42	98,67	Compound	BRD-A83326220	brazilin	Nitric oxide production inhibitor	
43	98,66	Compound	BRD-A50737080	CGK-733	ATM kinase inhibitor	Preclinical
45	98,62	Compound	BRD-K05396879	15-delta-prostaglandin-j2	PPAR receptor agonist	
46	98,59	Compound	BRD-K83988098	alvespimycin	HSP inhibitor	Phase 2
47	98,59	Compound	BRD-K31912990	CGP-71683	Neuropeptide receptor antagonist	Preclinical
49	98,48	Compound	BRD-K73395020	SA-1478088	Metalloproteinase inhibitor	
50	98,48	Compound	BRD-K26669427	WR-216174	CDK inhibitor	
52	98,45	Compound	BRD-K17075857	chloroxine	Opioid receptor antagonist	Launched
54	98,41	Compound	BRD-K51730347	diphencyprone	Immunostimulant	Phase 2
55	98,41	Compound	BRD-K03109492	NSC-663284	CDC inhibitor	Preclinical
56	98,38	Compound	BRD-K38477985	malonoben	Protein tyrosine kinase inhibitor	
59	98,31	Compound	BRD-A78360835	cercosporin	PKC inhibitor	
58	98,31	Compound	BRD-K64517075	heliomycin	antibiotic	
62	98,27	Compound	BRD-K74305673	IKK-2-inhibitor-V	NFkB pathway inhibitor	Phase 1
63	98,24	Compound	BRD-K28907958	CD-437	RAR agonist	Preclinical
66	98,07	Compound	BRD-K40255344	tyrphostin-A9	Tyrosine kinase inhibitor	Preclinical
67	98,06	Compound	BRD-M86331534	pyrvinium-pamoate	Androgen receptor inhibitor	Launched
68	98,06	Compound	BRD-A38030642	cyclosporin-a	Calcineurin inhibitor	Launched
69	97,99	Compound	BRD-K17497770	butein	NFkB pathway inhibitor	Preclinical
71	97,96	Compound	BRD-K51967704	BIIB021	HSP inhibitor	Phase 2
72	97,96	Compound	BRD-K20755323	SA-792728	Sphingosine kinase inhibitor	
74	97,94	Compound	BRD-K67844266	MLN-4924	Nedd activating enzyme inhibitor	
76	97,85	Compound	BRD-K10573841	tunicamycin	GLCNAC phosphotransferase inhibitor	
77	97,83	Compound	BRD-K98548675	parthenolide	NFkB pathway inhibitor	Phase 1
78	97,78	Compound	BRD-K03406345	azacitidine	DNA methyltransferase inhibitor	Launched
80	97,74	Compound	BRD-K35960502	niclosamide	DNA replication inhibitor	Launched
81	97,74	Compound	BRD-K15616905	CCCP	Mitochondrial oxidative phosphorylation uncoupler	
79	97,74	Compound	BRD-K39120595	bithionol	Autotaxin inhibitor	Withdrawn
82	97,67	Compound	BRD-K15409150	penfluridol	Dopamine receptor antagonist	Launched
84	97,64	Compound	BRD-K72895815	SSR-69071	Leukocyte elastase inhibitor	
85	97,6	Compound	BRD-K82135108	elesclomol	Topoisomerase inhibitor	Phase 3
86	97,6	Compound	BRD-K39111395	BCL2-inhibitor	BCL inhibitor	
87	97,57	Compound	BRD-K88677950	PD-198306	MAP kinase inhibitor	Preclinical
88	97,5	Compound	BRD-K88868628	iodoacetic-acid	Cysteine peptidase inhibitor	
90	97,5	Compound	BRD-K21672174	RO-28-1675	Glucokinase activator	Preclinical
89	97,5	Compound	BRD-K32744045	disulfiram	Aldehyde dehydrogenase inhibitor	Launched
91	97,38	Compound	BRD-A34205397	suloctidil	Vasodilator, Adrenergic receptor antagonist	Withdrawn
92	97,29	Compound	BRD-K66792149	quinoclamine	Algicide	
93	97,29	Compound	BRD-A58564983	selamectin	Nematocide	Launched
95	97,15	Compound	BRD-K17140735	SCH-79797	Proteinase activated receptor antagonist	
97	97,11	Compound	BRD-K41859756	NVP-AUY922	HSP inhibitor	Phase 2
99	97,08	Compound	BRD-A98283014	calmidazolium	Calcium channel blocker	
101	96,93	Compound	BRD-K94325918	kinetin-riboside	Apoptosis inducer	
102	96,91	Compound	BRD-K15600710	obatoclax	BCL inhibitor	Phase 3
106	96,9	Compound	BRD-K06426971	ryuvidine	Histone lysine methyltransferase inhibitor	Preclinical
105	96,9	Compound	BRD-K08417745	SID-26681509	Cathepsin inhibitor	
104	96,9	Compound	BRD-K78122587	NNC-55-0396	T-type calcium channel blocker	Preclinical
107	96,86	Compound	BRD-K78126613	menadione	CDC inhibitor	Launched
109	96,83	Compound	BRD-A62809825	thapsigargin	ATPase inhibitor	
108	96,83	Compound	BRD-K47150025	KI-8751	VEGFR inhibitor	Preclinical
112	96,55	Compound	BRD-K21806131	tegaserod	Serotonin receptor agonist	Withdrawn
111	96,55	Compound	BRD-K74133369	oligomycin-a	ATP synthase inhibitor	Preclinical
114	96,5	Compound	BRD-A79465854	auranofin	NFkB pathway inhibitor	Launched
115	96,49	Compound	BRD-A56020723	CA-074-Me	Cathepsin inhibitor	
116	96,31	Compound	BRD-K06593056	LE-135	Retinoid receptor agonist	Preclinical
119	96,01	Compound	BRD-K03816923	rottlerin	MAP kinase inhibitor	
120	95,98	Compound	BRD-A17065207	brefeldin-a	Protein synthesis inhibitor	Preclinical
122	95,95	Compound	BRD-K24681473	YM-155	Survivin inhibitor	Phase 2
126	95,81	Compound	BRD-A82371568	clofarabine	DNA polymerase inhibitor	Launched
129	95,67	Compound	BRD-A08003242	rhodomyrtoxin-b	DNA intercalator	
130	95,64	Compound	BRD-K15025317	BAY-11-7821	NFkB pathway inhibitor	
131	95,63	Compound	BRD-K37865504	LY-2183240	FAAH inhibitor	
132	95,62	Compound	BRD-K30296925	flavokavain-b	Carcinoma cell growth inhibitor	
134	95,5	Compound	BRD-K66175015	afatinib	EGFR inhibitor	Launched
137	95,38	Compound	BRD-K36198571	WAY-170523	Metalloproteinase inhibitor	Preclinical
138	95,36	Compound	BRD-A19500257	geldanamycin	HSP inhibitor	Preclinical
139	95,35	Compound	BRD-K88625236	nonoxynol-9	Spermicide	Launched
142	95,1	Compound	BRD-K44849676	capsazepine	TRPV agonist	Preclinical
144	95,07	Compound	BRD-K68143200	SA-792541	Caspase activator	
145	95,07	Compound	BRD-A06352418	terfenadine	Histamine receptor antagonist	Withdrawn
27	98,98	Gene knock-down	CGS001-5682	PSMA1	Proteasome subunits	
61	98,27	Gene knock-down	CGS001-5684	PSMA3	Proteasome subunits	
64	98,18	Gene knock-down	CGS001-5707	PSMD1	Proteasome subunits	
73	97,95	Gene knock-down	CGS001-5690	PSMB2	Proteasome subunits	
94	97,25	Gene knock-down	CGS001-3309	HSPA5	Heat shock proteins / HSP70	
103	96,9	Gene knock-down	CGS001-7316	UBC	-	
121	95,95	Gene knock-down	CGS001-5693	PSMB5	Proteasome subunits	
127	95,77	Gene knock-down	CGS001-7415	VCP	ATPases / AAA-type	
128	95,77	Gene knock-down	CGS001-11331	PHB2	–	
136	95,45	Gene knock-down	CGS001-5709	PSMD3	Proteasome (prosome, macropain) subunits	
141	95,23	Gene knock-down	CGS001-8894	EIF2S2	Serine/threonine phosphatases/protein phosphatase 1, regulatory subunits	

*AKT* serine-threonine protein kinase*, ATM* ataxia-telangiectasia mutated serine/threonine protein kinase, *ATP* adenosine triphosphate, *BCL* B-cell lymphoma, *CCK* Cholecystokinin, *CDC* cell division cycle, *CDK* cyclin-dependent kinase, *EGFR* epidermal growth factor receptor, *FAAH* fatty acid amide hydrolase, *GLCNAC* N-Acetylglucosamine*, HDAC* histone deacetylases, *HSP* heat shock protein, *JAK* janus kinase, *MAP* mitogen-activated protein, *NFkB* nuclear factor kappa-light-chain-enhancer of activated B, *PFMRK* plasmodium cyclin-dependent protein kinases, *PI* proteasome inhibitors, *PKC* protein kinase c, *PPAR* peroxisome proliferator activated receptor, *RAR* Retinoid receptor, *TRPV* transient receptor potential cation channel subfamily V, *UPS* ubiquitin–proteasome system, *VEGFR* vascular endothelial growth factor receptor.

### Molecular docking-based drug repositioning

**Figure 4:**
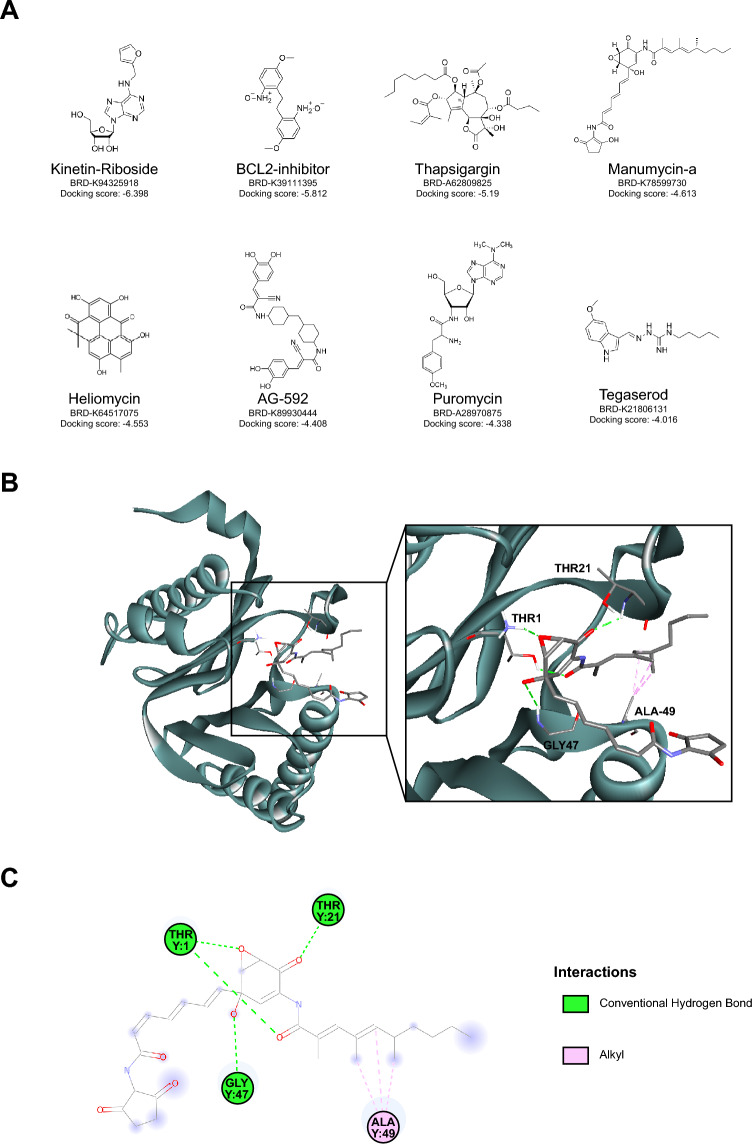
Candidate compounds based on the drug repositioning virtual screen. (**A**) 2D structure of the selected compounds including the corresponding code and the docking score. (**B**) 3D structure of the complex between β5 catalytic site and manumycin-A, as determined by molecular docking calculations. The β5 subunit is shown as lead blue ribbons. Key residues and the manumycin-A molecule are colored as atom type. In the zoomed-in section, hydrophobic interactions and hydrogen bonds are shown as dashed lines in light pink and green, respectively. (**C**) 2D ligand interaction diagram generated by discovery studio–BIOVIA software.

The 96 selected compounds were screened by means of molecular docking calculations *vs* the β5 subunit of human 20S proteasome. Proteasomes are classified as a family of N-terminal nucleophilic (Ntn) threonine proteases, where the N-terminal Thr1 of a catalytically active β-subunit acts as a nucleophile in peptide bond hydrolysis^31,32^. The 5LF3 crystallographic structure, where bortezomib is covalently bound to the catalytic O atom of Thr1 was used for virtual screening with the aim to identify reversible and less cytotoxic inhibitors^33^. Among non-covalent inhibitors, TMC-95 and other peptides form hydrogen bonds and hydrophobic interactions with the conserved proteasomal residues Thr1 (active site), Thr21, Ala49, and Gly47, suggesting a common mode of inhibition^34,35^. The results of docking validation on yeast 20S proteasome bound to TMC-95 indicated that the predicted binding conformation determined by Glide match well with that of the co-crystallized ligand. The ligand docked pose was in close agreement with the crystallographically determined position with a RMSD of the heavy atoms of only 0.16 Å. Following validation of the docking protocol, the dataset of 96 selected drugs was screened against proteasome subunit β5 using Glide SP mode and all the generated states were subsequently screened using the XP docking mode. All good scoring states from this last docking stage were analyzed and filtered by choosing only those compounds forming a hydrogen bond with the catalytic oxygen atom of Thr1. Eight compounds (AG-592, BCL2-inhibitor, heliomycin, kinetin-riboside, manumycin-A, puromycin dihydrochloride, tegaserod maleate, and thapsigargin) were identified on the basis of the docking score (Fig. 4A and Supplementary Table 6) and processed for further studies.

Molecular dynamics simulations of bortezomib, heliomycin, manumycin-A, puromycin, tegaserod maleate, thapsigargin, and kinetin-riboside bound at the β5 subunit binding site were performed to evaluate the stability of the ligands within the predicted site. In these systems, the distance between the center of mass of the ligands and the center of mass of the proteasome subunit β5 site remained constant over the simulation time with the exception of tegaserod maleate and puromycin, which exhibited the lowest docking scores among the identified virtual hits (Supplementary Fig. 1 and Supplementary Table 6). Notably, bortezomib and MLN-2238 had comparable docking scores (-6.387 and -6.728, respectively), indicative of predicted binding affinity for the β5 site (Supplementary Table 6). Evaluation of the eight candidate compounds using L1000 fireworks plots confirmed a similarity with known PIs (bortezomib, MG-132, and z-leu3-VS; Supplementary Fig. 2).

### Proposed proteasome inhibitors disrupted the proteasomal catalytic activity and caused accumulation of ubiquitinated proteins

**Figure 5:**
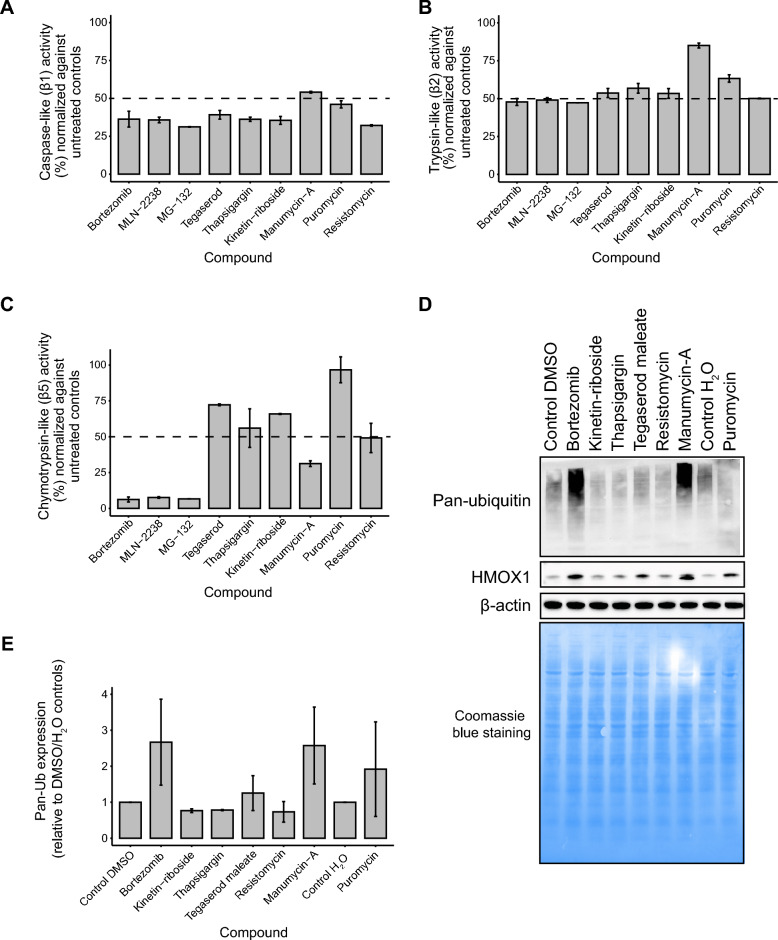
Analysis of proteasome activity inhibition using the six compounds with proposed proteasome inhibitor properties and three known proteasome inhibitors (Bortezomib, MG-132, and MLN-2238 were used as reference). (**A**–**C**) The six candidate compounds inhibited the three catalytic sites (caspase-like activity [β1 catalytic site], and trypsin-like activity [β2 catalytic site], and chymotrypsin-like activity [β5 catalytic site]) to different extents. Error bars depict the standard deviation. (**D**–**E**) Quantification of polyubiquitination and HMOX1 expression relative to Beta-actin and solvent controls (DMSO and H_2_O). Of the three candidate compounds (manumycin-A, puromycin dihydrochloride, and tegaserod maleate) displaying accumulation of polyubiquitinated proteins (between 40 kDa and higher molecular weight proteins) and elevated HMOX1 levels in MCF7 cells, manumycin-A was most efficient in line with bortezomib.

We then evaluated whether the six candidate compounds (reference: bortezomib, MG-132, and MLN-2238) inhibited the β1 (caspase-like), β2 (trypsin-like), and/or β5 (chymotrypsin-like) catalytic sites of the 20S proteasome. Both the known PIs and candidate compounds inhibited all three catalytic sites to some extent at 10 µM. Although the known PIs inhibited the catalytic activity the most (bortezomib: β1 = 63.7%, β2 = 52.2%, β5 = 93.8%; MG-132: β1 = 68.8%, β2 = 52.7%, β5 = 93.4%; and MLN-2238: β1 = 64.2%, β2 = 50.9%, β5 = 92.4%), > 50% suppression of proteasome activity was achieved by all of the candidate compounds for one or more of the three catalytic sites ((-)-kinetin-riboside: β1 = 64.5%, β2 = 46.6%, β5 = 34.1%; manumycin-A: β1 = 45.9%, β2 = 15.0%, β5 = 68.8%; puromycin dihydrochloride: β1 = 53.9%, β2 = 36.7%, β5 = 3.3%; resistomycin: β1 = 67.8%, β2 = 49.9%, β5 = 59.9%; tegaserod maleate: β1 = 60.8%, β2 = 46.3%, β5 = 27.7%; and thapsigargin: β1 = 63.8%, β2 = 43.2%, β5 = 44.0%, with manumycin-A displaying the highest levels of inhibition for the β5 site (Figs. 4B,C, 5A,C and Supplementary Fig. 3–Supplementary Fig. 9). In line with bortezomib, three of the candidate compounds (manumycin-A, puromycin dihydrochloride, and tegaserod maleate) caused accumulation of ubiquitinated proteins and induced elevated HMOX1 levels (Fig. 5D,E and Supplementary Fig. 10).

### Puromycin dihydrochloride was the most potent drug with proposed proteasome inhibitor properties

**Figure 6:**
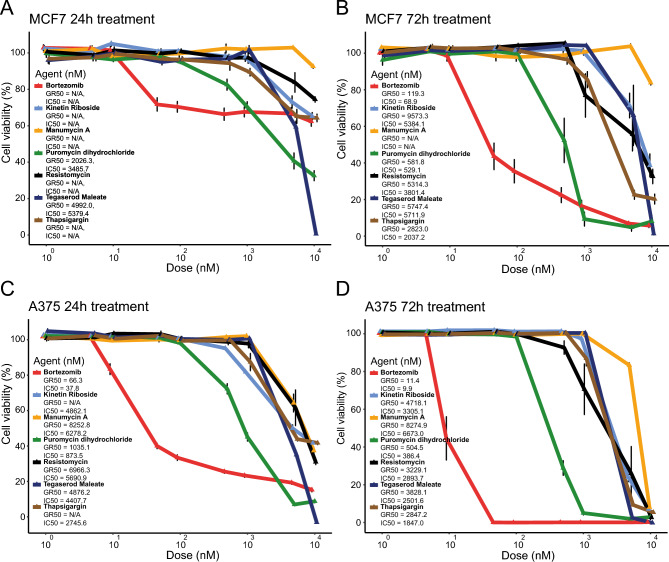
Potency of the identified compounds. MCF7 and A375 cells were exposed for 24 h or 72 h with the candidate compounds and bortezomib as a reference. (**A**,**B**) After 24 h, the most potent drugs for MCF7 cells were puromycin dihydrochloride and tegaserod maleate, and after 72 h all drugs were potent except for manumycin-A. (**C**,**D**) For A375 cells, the drug potency varied for the candidate compounds after 24 h treatment, whereas all compounds were potent but often at high concentration after 72 h. Error bars depict the standard deviation.

Of the eight compounds predicted to have high binding affinity for the β5 proteasome subunit and interaction with the hydroxyl group of Thr1, only six (heliomycin [resistomycin], kinetin-riboside, manumycin-A, puromycin dihydrochloride, tegaserod maleate, and thapsigargin) were available for purchase. Using bortezomib as a reference, the potency of each compound was then determined in A375 melanoma and MCF7 breast cancer cells treated for 24 or 72 h. A375 cells were significantly more sensitive to treatment with the tested compounds, with lower IC50 and GR50 values than MCF7 cells (Fig. 6A,B. After 72 h, bortezomib showed the lowest IC50 (A375: IC50 = 0.01 µM, SD = 0.002; MCF7: IC50 = 0.07 µM, SD = 0.04) and GR50 values in both cell lines (A375: GR50 = 0.01 µM SD = 0.002; MCF7: GR50 = 0.1 µM, SD = 0.005), followed by puromycin dihydrochloride (A375: IC50 = 0.4 µM, SD = 0.06; GR50 = 0.5 µM, SD = 0.05; MCF7: IC50 = 0.5 µM, SD = 0.1, GR50 = 0.6 µM, SD = 0.07). In MCF7 cells, IC50 and GR50 values could only be calculated for puromycin dihydrochloride and tegaserod maleate after 24 h exposure, while IC50 values could be calculated for all of the tested drugs in A375 cells at that time point (Fig. 6C,D).

## Discussion

In the current study, we used an integrated DR approach to identify compounds with proteasome inhibitor properties, i.e., the ability to inhibit the proteasomal degradation process. This approach was performed in three steps to identify compounds (1) displaying similar treatment response (transcriptomic signatures) as known proteasome inhibitors (bortezomib, MG-132, and MLN-2238), (2) potentially binding to the β5 proteasome subunit, and (3) inhibiting the activity of the catalytic sites, causing accumulation of ubiquitinated proteins, displaying cytotoxic effects, and inducing HMOX1 expression. Computational screening revealed 113 compounds with similar induced gene expression patterns as PIs, which was subsequently narrowed down to 8 compounds based on binding properties to the β5 catalytic site. Subsequent in vitro evaluation showed that the 6 tested compounds not only inhibited the β5 catalytic site, but also the β1 and β2 sites. Although these compounds inhibited the β1 and β2 catalytic sites as well as the 3 known PIs, manumycin-A was the best inhibitor of the β5 site among the test compounds. Furthermore, only manumycin-A, puromycin dihydrochloride, and tegaserod maleate led to a significant accumulation of ubiquitinated proteins and elevated HMOX1 levels.

Pharmacogenomics has previously been used to correlate the induced transcriptomic profile of a compound with its MOA^36,37^. Despite differences in the chemical properties of bortezomib, MG-132, and MLN-2238, treatment with these PIs led to the recurrent dysregulation of 12 genes (e.g. *BAG3*, *DNAJB1*, *HMOX1*) in cell lines representing multiple cancer types. Some of these genes (e.g. *BAG3* and *HMOX1*) were also identified by Mofers et al.^38^. Here, we show that upregulation of *DNAJB1* and *HMOX1* in MCF7 breast cancer cells was time-dependent. Notably, MCF7 cells required at least 6 h exposure to bortezomib at concentrations ≥ 100 nM to induce changes in transcriptomic profiles. *BAG3* (regulates cellular proteostasis and cell viability), *DNAJB1* (associated with ER stress and the ubiquitin-proteasome pathway), and *HMOX1* (involved in oxidative stress and cell defense)^39–41^ play a crucial role in stress and cell survival, possibly explaining their consistent expression patterns in cells treated with the known proteasome inhibitors and the six identified compounds. Although the integrated transcriptomics- and structure-based drug repositioning approach used here may provide the opportunity to repurpose drugs for specific diseases and/or identify drugs that may have fewer side effects, candidate drugs still need to be validated in vitro and/or in vivo to validate their potency at optimal conditions.

Using the CMap touchstone dataset for 9 cell lines treated with MG-132 and MLN-2238, we were able to identify 96 compounds and 11 gene knock-downs that were proposed to have similar MOA as proteasome inhibitors and could therefore be assumed to be compounds with proteasome inhibitor properties. Despite differences in filtering the CMap data (*i.e.*, differences in input compounds and median tau score cut-off), we and Mofers et al.^38^ identified a relatively comparable list of compounds showing similar drug-induced signatures with PIs. As a proof of concept, 7 of the 11 gene knock-downs involved genes encoding for components of the proteasome complex (*PSMA1*, *PSMA3*, *PSMB2*, *PSMB5*, *PSMD1*, and *PSMD3*) and a ubiquitin gene (*UBC*). Knock-down of these genes would ultimately have a similar effect on the proteasome-mediated degradation process as suppression of the proteasome with PIs, *i.e*. an accumulation of ubiquitin-tagged proteins that in turn would cause cellular instability and apoptosis^42,43^. Of the remaining gene knock-downs, inactivation of heat shock protein *HSPA5* that plays a pivotal role in refolding misfolded proteins would have a similar effect on cellular homeostasis as proteasome inhibition^44^. Moreover, 18 compounds (missing data for 78 compounds) were further evaluated for their antineoplastic activity using GR Metric Calculator, thereby showing that geldanamycin, radicicol, and thapsigargin had a cytotoxic effect on cancer cells in line with bortezomib. These findings were consistent with our in vitro validation in A375 melanoma and MCF7 breast cancer cells, since bortezomib and thapsigargin had a similar cytotoxic effect at > 5 µM. Of the identified candidate compounds, puromycin was the most potent. However, suitable working doses in vivo might differ between compounds and need to be tested in animal studies.

We then used molecular docking^36^ to evaluate the binding affinity of the 96 compounds for proteasome β5 subunit and their ability to form hydrogen bonds and hydrophobic interactions with the proteasomal residue Thr1. Of the 96 compounds, we identified 8 compounds (e.g., manumycin-A, kinetin riboside, and puromycin dihydrochloride) fulfilling these criteria, several of which included antibiotics and plant hormones. To evaluate their MOA further, we used purified proteasome lysate to evaluate suppression of the 20S proteasome after 6 h treatment at a concentration of 10 µM with 6/8 candidate compounds and 3 known proteasome inhibitors (bortezomib, MG-132, and MLN-2238) as references.

Tegaserod maleate is currently used to treat irritable bowel syndrome, but has also been shown to have an inhibitory effect on the growth of breast cancer xenografts in mice when used in combination with anti-PD1/anti-TIGIT (immunotherapy)^45,46^. Manumycin-A is a natural antibiotic that has an anti-tumoral effect in triple-negative breast cancer^47^. Puromycin is another natural antibiotic that is toxic to both eukaryotic and prokaryotic cells by affecting the protein synthesis negatively^48^. It has also been shown to induce apoptosis in the MCF-7 breast cancer cell line^49^. Here, we also confirm that tegaserod not only affects cell survival in the MCF-7 cell line, but also the A375 melanoma cell line. Manumycin-A and puromycin were also cytotoxic to A375 cells. These findings demonstrate that compounds can be used for multiple diseases.

The integrated DR approach described here provides the opportunity to identify new drugs for rare diseases and new indications for old drugs, while at the same time developing a better understanding of how different drugs work and cellular response to treatment. This information can therefore be used to improve treatment by identifying effective drug combinations (e.g., one drug that induces DNA damage, while the other targets DNA repair). By analyzing drug-induced transcriptomic responses, it could be possible to identify resistance genes. However, the limitation of this work was that we were unable to examine all of the 8 identified compounds since 2 were not available for purchase. Potentially potent proteasome inhibitors might have also been missed during the compound selection process. In addition, we only used two cell lines to investigate drug potency. Although the transcriptomics analysis did not show a significant difference in drug-induced expression due to tissue of origin, the drug-of-interest should be investigated in other cell lines representing the desired disease model and in animal models.

In summary, our integrated DR approach identified six candidate compounds with proteasome inhibitor properties (e.g., puromycin dihydrochloride, manumycin-A, and tegaserod maleate), which was confirmed using in vitro assays to assess the proteasome activity, cell viability, and protein expression. However, identifying novel compounds based solely on transcriptomic profiling should be used with caution. Therefore, additional in vitro and in vivo testing is warranted to determine whether the drug affects the desired target or not.

## Materials and Methods

### Curation of proteasome inhibitor-induced gene expression signatures from iLINCS and CMap

To identify gene signatures associated with proteasome inhibition (bortezomib, MG-132, and MLN-2238 [also known as ixazomib]) and other perturbagens inducing similar gene expression patterns in cell lines, we retrieved two pharmacogenomics datasets from the Library of Integrated Cellular Signatures (LINCS) Consortium, *i.e.* iLINCS-Pharmacogenomics transcriptional signatures^50^ and broad institute connectivity map^51^ (CMap 2.0 version 1.2 build 1.44 December 17, 2020, level 5 gene expression data; Table 2). The iLINCS dataset was comprised of 351 signatures from the NCI Transcriptional Pharmacodynamics Workbench (NCI-TPW; Affymetrix GeneChip Human Genome U133A microarray)^52^ and the Plate-seq project (RNA sequencing)^53^ datasets for 60 cell lines (derived from breast, central nervous system [CNS], colon, large intestine, large intestine epithelial, leukemia, lung, melanoma, ovarian, prostate, renal) treated with 10 and 100 nM bortezomib for 2, 6, and 24 h and corresponding controls. Using the iLINCS data portal, the dataset was filtered to include the top 100 differentially expressed genes per signature, resulting in a total of 5,448 unique genes. Differential expression data (log2) for the top 250 genes were then downloaded for further analysis. CMap utilizes L1000 technology, a Luminex bead array-based platform that infers the expression patterns for 11,350 genes by measuring the expression of 978 landmark genes^51^. CMap contains gene expression data for nine cell lines (A375, A549, HA1E, HCC515, HEPG2, HT29, MCF7, PC3, and VCAP) exposed to 0.0016-20 µM MLN-2238 and MG-132 for 6 and 24 h. Transcriptomic signatures for cells treated with MLN-2238 and MG-132 were retrieved from CMap and filtered for the 250 most variable genes using the transcripTools R package (version 0.0.0.9000)^54^.

**Table 2:** Data sources used in the study.

Data source	Dataset name	Data type	Drug	Treatment time	Drug concentration	Number of cell lines	Cell Lines and tissue type
iLINCS Data Portal	Pharmacogenomics transcriptional signatures	Drug perturbation-associated gene expression	Bortezomib	2, 6, 24 h	10 and 100 nM	60	**Breast** (BT-549, HS-578 T, MCF7, MDA-MB-231, MDA-MB-435, MDA-MB-468, T-47D), **CNS** (SF-268, SF-295, SF-539, SNB-19, SNB-75, U251), **Colon** (COLO-205, HCC-2998, HCT-15, KM12, SW-620), **Large intestine** (HT29), **Large intestine epithelial** (HCT-116), **Leukemia** (CCRF-CEM, HL-60, K-562, MOLT-4, RPMI-8226, SR), **Lung** (A549, EKVX, HOP-62, HOP-92, NCI-H226, NCI-H23, NCI-H322M, NCI-H460, NCI-H522), **Melanoma** (LOX, M14, MALME-3 M, SK-MEL-2, SK-MEL-28, MEL-5, UACC-257, UACC-62), **Ovarian** (IGR-OV1, NCI-ADR-RES, OVCAR-3, OVCAR-4, OVCAR-5, OVCAR-8, SK-OV-3), **Prostate** (DU-145, PC-3), **Renal** (786-0, A498, ACHN, CAKI-1, RXF-393, SN12C, TK-10, UO-31)
Connectivity Map (CMap)		Drug perturbation-associated gene expression	MG-132	6 and 24 h	1, 10, 100, 500 nM; 1, 3, 10 µM	16	**Adipose stroma** (ASC), **Breast** (MCF7, SKB), **CNS** (NEU), **Colorectal** (HT29), **Liver** (HEPG2, PHH), **Lung** (A549, HCC515), **Melanoma** (A375), **Nasopharyngeal** (NPC), **Pluripotent stem cell** (FIBRNPC), **Prostate** (PC3, VCAP), **Renal** (HA1E, NKDBA)
		Drug perturbation-associated gene expression	MLN-2238	6 and 24 h	500 nM; 1, 3, 10 µM	51	**Breast** (MCF7), **Colon** (CL34, HCT116, HT115, RKO, SW480, SW948), **Colorectal** (HT29, LOVO, MDST8, SNUC4, SNUC5, SW620), **Endometrium** (HEC108, SNGM), **Large intestine** (NCIH508, SNU1040), **Leukemia** (NOMO1, PL21, SKM1, THP1, U937), **Liver** (HEPG2), **Lung** (A549, CORL23, DV90, H1299, HCC15, HCC515, NCIH1694, NCIH1836, NCIH2073, NCIH596, SKLU1, T3M10), **Lymphoma** (WSUDLCL2), **Melanoma** (A375, SKMEL1, SKMEL28), **Ovarian** (COV644, EFO27, OV7, RMGI, RMUGS, TYKNU), **Prostate** (PC3, VCAP), **Renal** (HA1E, NKDBA), **Soft tissue** (A673), **Stomach** (AGS), **Uterine-cervix** (JHUEM2)
		Drug perturbation similarity	MG-132			9	**Breast** (MCF7), **Colorectal** (HT29), **Liver** (HEPG2), **Lung** (A549, HCC515), **Melanoma** (A375), **Prostate** (PC3, VCAP), **Renal** (HA1E)
		Drug perturbation similarity	MLN-2238				
L1000 fireworks display (L1000FWD)		Drug perturbation similiarity					

Hierarchical clustering of the 250 dysregulated genes in the iLINCS (bortezomib) and CMap (MG-132 and MLN-2238) datasets was performed with the pheatmap R package (version 1.0.12)^55^ using the Manhattan distance metric and Ward’s minimum variance method (Ward.D2). To identify putative predictive biomarkers for proteasome inhibition, gene ontology analysis was performed with Reactome (https://reactome.org/)^56^ for common dysregulated genes in both datasets.

### Curation of perturbagen-driven gene expression signatures for drug repositioning

The CMap Touchstone tool^37^ (data version Beta) was used to identify other perturbagen types (compounds or gene knock-down) that induce similar transcriptomic signatures as MG-132 and/or MLN-2238. CMap connectivity *tau (τ)* enrichment scores range from -100 to 100, with negative/positive scores indicating opposing/similar gene signatures between a compound of interest and other perturbagens in the CMap Touchstone database. Perturbagens with median *tau* scores ≥ 95 were chosen for further analysis; a *tau* score of 95 indicates that only 5% of other compounds in Touchstone are more similar to the queried transcriptomic profile^57^. The L1000 fireworks display (L1000FWD)^58^ tool was then used to generate L1000 fireworks plots for candidate compounds using known proteasome inhibitors (bortezomib, MG-132, and z-leu3-VS) and chemotherapeutic agents (Docetaxel, Etoposide, and Tamoxifen) as references.

### Curation of dose-response data for candidate compounds

Dose-dependent sensitivity data for cell lines (nonmalignant and cancer) treated with 18/96 candidate compounds identified using CMap Touchstone and bortezomib (as a reference, when available) were retrieved from the GR Metrics Calculator and Browser web-based tool. Box plots were generated using growth rate inhibition (GR50) data from four datasets, *i.e.* Broad-HMS LINCS Joint Project, MEP-HMS LINCS Joint Project, Genentech Cell Line Screening Initiative (gCSI), and Cancer Therapeutics Response Portal (CTRP). Cell lines with missing GR50 data were removed from the plots.

### Structure-based virtual screening

The structure of the human 20S proteasome at 2.1 Å (PDB code: 5LF3)^59^ was obtained from the Protein Data Bank^60^. The Glide program^61^ was used for virtual screening and the library of the selected approved drugs was docked into the β5 subunit of the proteasome after removal of the covalently bound bortezomib inhibitor. The protein preparation process of β5 included correcting mislabeled elements, adding hydrogen atoms, assigning bond orders and performing restrained energy minimization using the OPLS4 force field^62^ and was carried out using the Protein Preparation Wizard of Maestro (Schrödinger Release 2022–3: Maestro, Schrödinger, LLC, New York, NY, 2021). The library of approved drugs was prepared using LigPrep (Schrodinger Release 2022–3, LigPrep, Schrodinger, LLC, New York, NY, 2021) and all possible stereoisomers, tautomers, and protonation states at pH 7.0 ± 2.0 were generated using the Epik module^63^. Finally, drug-molecules were energy minimized using OPLS4 force field. All ligands were docked into the active site of the β5 subunit using inner and outer receptor grid boxes of 10 and 23 Å, respectively, centered on the co-crystalized bortezomib. A ligand-flexible docking was performed in two steps, *i.e*. SP (standard precision) and XP (extra precision) mode using the GlideScore scoring function to rank compounds. Validation of the docking protocol was performed using the crystal structure of yeast 20S proteasome bound to the non-covalent inhibitor TMC-95A (PDB code: 1JD2). The co-crystalized ligand was re-docked into the active site of yeast proteasome and the RMSD between the crystallographic pose and the docked pose structure calculated.

### Molecular dynamics calculations

All-atom molecular dynamics simulations were performed using the Desmond-6.8 module of Schrödinger software package (Schrödinger Release 2021–4: Desmond Molecular Dynamics System, D. E. Shaw Research, New York, NY, 2021) as implemented in Maestro. Docked complexes were placed in an orthorhombic box at a buffer distance of 10 Å and solvated with SPC water models. A 0.15 M NaCl salt concentration was added and additional Na^+^/Cl^−^ ions were added to neutralize the systems. The particle-mesh Ewald method was used to calculate the long-range electrostatic interactions. A cut-off radius of 9.0 Å was applied for short-range van der Waals and Coulomb interactions. Each solvated system was minimized and equilibrated using the default protocol of Desmond in Maestro which includes 2 NVT and 2 NPT restrained short simulations. All equilibrated systems were then subjected to a MD run with periodic boundary conditions in the NPT ensemble using OPLS4 force field 73 for 50 ns. The temperature of 300 K and the pressure of 1 atm of the systems were maintained by the Nosè-Hoover chain thermostat and Martyna-Tobiase-Klein barostat methods, respectively. The analysis of MD trajectories was performed using Desmond suite of programs and VMD software^64^.

### Cell culture and drug treatment

To validate the findings from the publicly available datasets, human melanoma (A375) and breast (MCF7) cancer cell lines were used. The cell lines were cultured in dulbecco modified eagle’s medium (DMEM) supplemented with 2 mM L-glutamine, 4 g/L D-glucose, and 10% FBS (ThermoFisher Scientific) and maintained at 37 °C in a humidified 5% CO_2_ environment. Cell authentication was performed using the Eurofins Genomics Human Cell Line Authentication service. Candidate compounds were purchased from Sigma-Aldrich ((-)-kinetin-riboside, manumycin-A, puromycin dihydrochloride, tegaserod maleate, and thapsigargin) or Cayman Chemicals (resistomycin [heliomycin]), while known PIs (bortezomib, MG-132, and MLN-2238) were purchased from Selleckchem. Stock solution concentrations of 1–2 mM were prepared using DMSO (bortezomib, (-)-kinetin-riboside), manumycin-A, MG-132, MLN-2238, tegaserod maleate, and thapsigargin), or Milli-Q water (puromycin dihydrochloride). A drug sensitivity screen was performed using cells seeded on 96-well clear, flat-bottom microplates at a density of 4.0 × 10^3^ (A375) or 7.5 × 10^3^ (MCF7) cells/well and incubated for 24 h. The cells were then exposed to the candidate compounds and bortezomib (control) at 9 concentrations (1–10,000 nM) and matched drug solvent (dimethyl sulfoxide, DMSO) concentration vehicle controls for 24 h or 72 h, as described elsewhere^3^. Cell viability was determined using the resazurin cell viability assay and growth rate metrics assessed (IC50 and GR50) with the GRmetrics (version 1.16.0 package^65^ in R/Bioconductor version 4.0.3). Mean values of IC50 and GR50 and standard deviation thereof were determined.

### β1, β2, and β5 catalytic activity of the 20S proteasome

Purified 20S proteasome substrate (Enzo Life Sciences, Cat. BML-PW8720-0050) was used to investigate whether compounds with proposed proteasome inhibitor properties inhibit one or more of the 20S proteasome catalytic sites. The proteasome substrate was diluted in reaction buffer (according to suppliers’ protocol) to 0.004 mg/mL in 96-well black, flat-bottom microplates. Proteasome activity of the β1 (caspase-like), β2 (trypsin-like), and β5 (chymotrypsin-like) catalytic sites were then evaluated after 2 h drug exposure with known proteasome inhibitors (bortezomib, MG-132, and MLN-2238) used as controls and candidate compounds (manumycin-A, (-)-kinetin riboside, puromycin dihydrochloride, resistomycin, thapsigargin or tegaserod maleate) at a concentration of 10 µM. After drug exposure, Z-Leu-Leu-Glu-AMC (Caspase-like; Enzo Life Sciences, Cat. BML-ZW9345), Ac-Arg-Leu-Arg-AMC (trypsin-like; Enzo Life Sciences, Cat. BML ZW9785), Suc-Leu-Leu-Val-Tyr-AMC (chymotrypsin-like; Enzo Life Sciences, Cat. BML-P802) or substrate were added to reach a concentration of 20 µM and incubated with the purified proteasome substrate for 40 min before measuring the fluorescence intensity (excitation 355 nm and emission 460 nm) using a Wallac 1420 VICTOR2 microplate reader (Perkin Elmer).

### Western blot

MCF7 cells were treated for 6 h with the candidate compounds (manumycin-A, (-)- kinetin riboside, puromycin dihydrochloride, resistomycin, thapsigargin or tegaserod maleate) at 10 µM or drug solvent (DMSO or Milli-Q H_2_O) and harvested, washed with PBS (Gibco), and lysed in Qproteome Mammalian Lysis Buffer (Qiagen, Hilden, Germany) supplemented with Benzonase® Nuclease, as well as protease and phosphatase inhibitors. The lysates (20 µg) were separated on NuPAGE™ 4–12% Bis-Tris gels (ThermoFisher Scientific) and transferred to nitrocellulose membranes. The membranes were stained with Imperial Protein Stain (ThermoFisher Scientific) to determine the total protein content per lane (loading control), followed by overnight incubation in 5% non-fat dry milk (NFDM; Semper) solution at 4 °C. The membranes were then incubated with primary antibodies for mouse anti-ubiquitin (pan; 1:1000 dilution; Sigma-Aldrich, Cat. MABS486), rabbit anti-HMOX1 (dilution 1:500 dilution; Abcam, Cat. AB68477) or mouse anti-Beta actin (1:2000 dilution; Abcam, Cat. Ab6276) at room temperature (RT) for 2 h, followed by secondary horseradish peroxidase-linked anti-mouse (1:2000; Amersham, Cat. NA931V) or anti-rabbit (1:2000; Amersham, Cat. NA934V) IgG antibodies at RT for 1 h. Proteins were detected using the SuperSignal™ West Femto Maximum Sensitivity Substrate (ThermoFisher Scientific) and images acquired with a Fujifilm LAS-1000 Luminescent image analyzer.

### Quantitative real-time PCR

MCF7 cells were seeded at a density of 5 × 10^5^ cells per T25 flask and treated with 10 µM bortezomib for 1, 6, and 24 h. Total RNA was extracted from MCF7 cells using the RNeasy Lipid Tissue Mini Kit (Qiagen), followed by evaluation of RNA concentration and integrity using Qubit (ThermoFisher Scientific) and TapeStation (Agilent), respectively. Complementary DNA was synthesized with the Superscript III First-Strand Synthesis for qRT-PCR kit (ThermoFisher Scientific). Quantitative real-time PCR (qPCR) was performed using predesigned TaqMan Gene Expression Assays for *HMOX1* (Hs01110250_m1) and *DNAJB1* (Hs00428680_m1) expression. Relative gene expression patterns were determined using the ΔΔCt method after normalizing the data with the geometric mean of three endogenous controls (*HPRT1* [Hs02800695_m1], *PPIA* [Hs99999904_m1], and *PUM1* [Hs00472881_m1]) and DMSO-treated controls.

### Statistical analysis

Statistical analyses were performed in R/Bioconductor (version 4.0.3); *P* < 0.05 was considered to be statistically significant. The Shapiro-Wilk normality test was performed using the dplyr R package (version 1.0.8)^67^ to determine whether the data were normally distributed. The parametric T-test was used if *P* > 0.05 (normally distributed) or the non-parametric Wilcoxon test was used if *P* < 0.05 (not normally distributed). Bar plots were constructed using the ggpubr R package (version 0.4.0)^67^ to compare different groups with Benjamini–Hochberg adjusted *P*-values, while dot and violin plots were generated using ggplot2 (version 3.3.6)^68^.

### Supplementary Information

Supplementary Figure S1.

Supplementary Figure S2.

Supplementary Figure S3.

Supplementary Figure S4.

Supplementary Figure S5.

Supplementary Figure S6.

Supplementary Figure S7.

Supplementary Figure S8.

Supplementary Figure S9.

Supplementary Figure S10.

Supplementary Table S1.

Supplementary Table S2.

Supplementary Table S3.

Supplementary Table S4.

Supplementary Table S5.

Supplementary Table S6.

## Data Availability

All data used in this study are included or referred to within this work.
