# Supplementary material for: Integrated transcriptomics- and structure-based drug repositioning identifies drugs with proteasome inhibitor properties
Source: Sci Rep. 2024 Aug 13;14:18772. doi: 10.1038/s41598-024-69465-6 (PMC11322189; doi:10.1038/s41598-024-69465-6)
Supplement: Supplementary file 1 — Supplementary Figure S1. [file 41598_2024_69465_MOESM1_ESM.pdf]

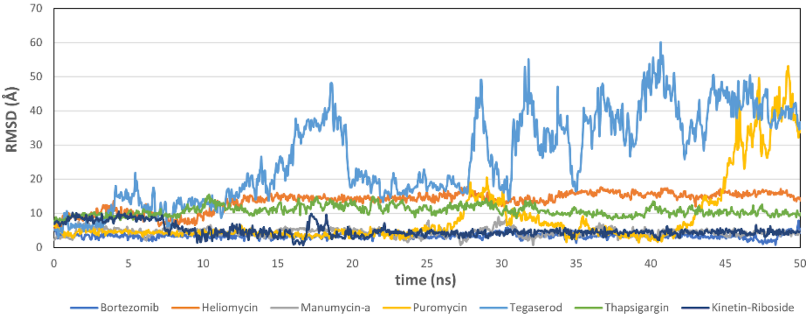

Supplementary Figure 1. Time evolution of the distance between the center of the mass of the proteasome β5 site and the center of the mass of bortezomib, ((-)-kinetin-riboside, manumycin-A, puromycin dihydrochloride, resistomycin [heliomycin], tegaserod maleate, and thapsigargin.

Supplementary Fig. 1. Larsson *et al*. (2024)
