# Supplementary material for: Integrated transcriptomics- and structure-based drug repositioning identifies drugs with proteasome inhibitor properties
Source: Sci Rep. 2024 Aug 13;14:18772. doi: 10.1038/s41598-024-69465-6 (PMC11322189; doi:10.1038/s41598-024-69465-6)
Supplement: Supplementary file 2 — Supplementary Figure S2. [file 41598_2024_69465_MOESM2_ESM.pdf]

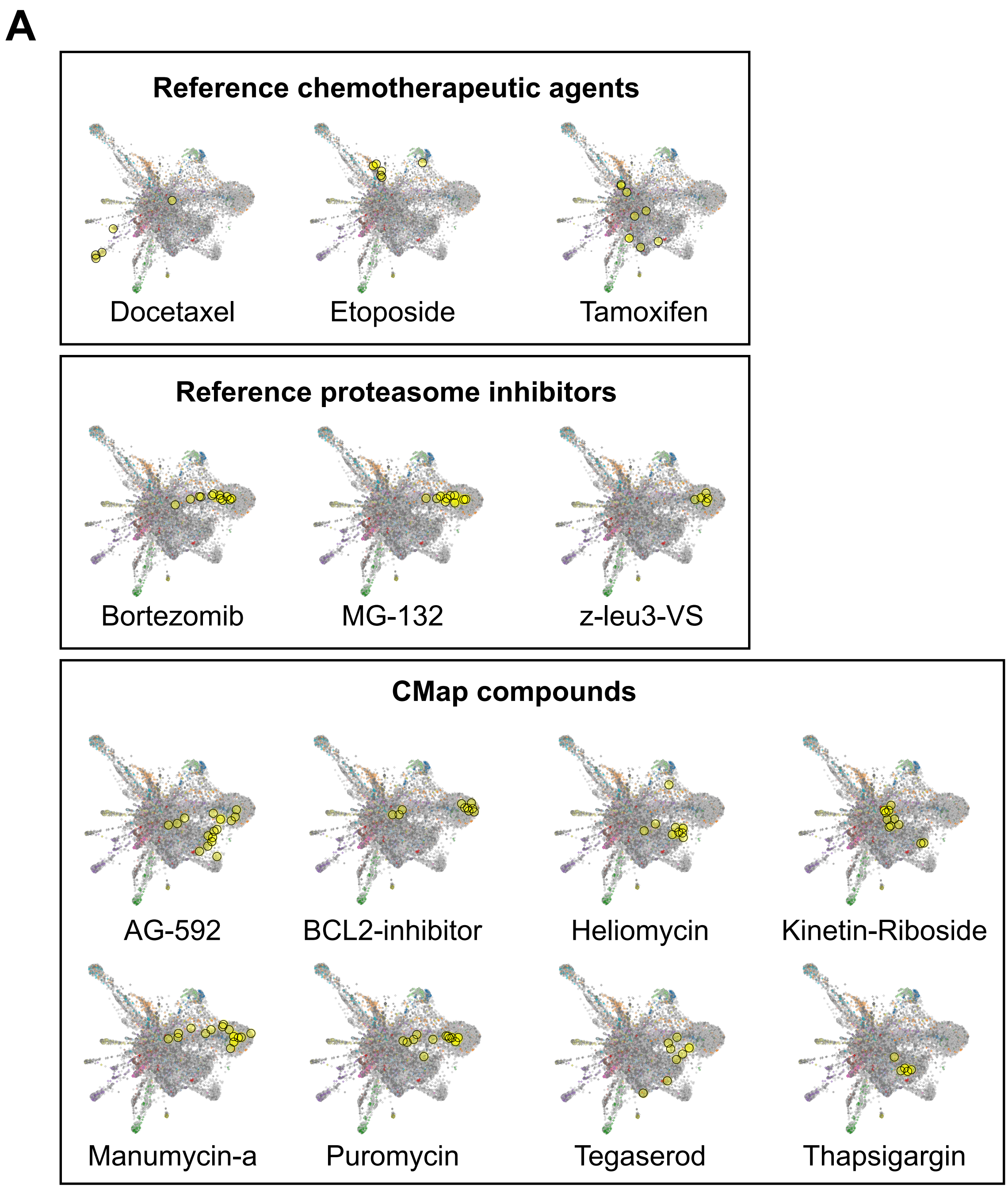

Supplementary Figure 2. L1000 fireworks plots shows compounds similarity to Reference proteasome inhibitors.

Supplementary Figure 2. Larsson *et al*. (2024)
