# Supplementary material for: Integrated transcriptomics- and structure-based drug repositioning identifies drugs with proteasome inhibitor properties
Source: Sci Rep. 2024 Aug 13;14:18772. doi: 10.1038/s41598-024-69465-6 (PMC11322189; doi:10.1038/s41598-024-69465-6)
Supplement: Supplementary file 3 — Supplementary Figure S3. [file 41598_2024_69465_MOESM3_ESM.pdf]

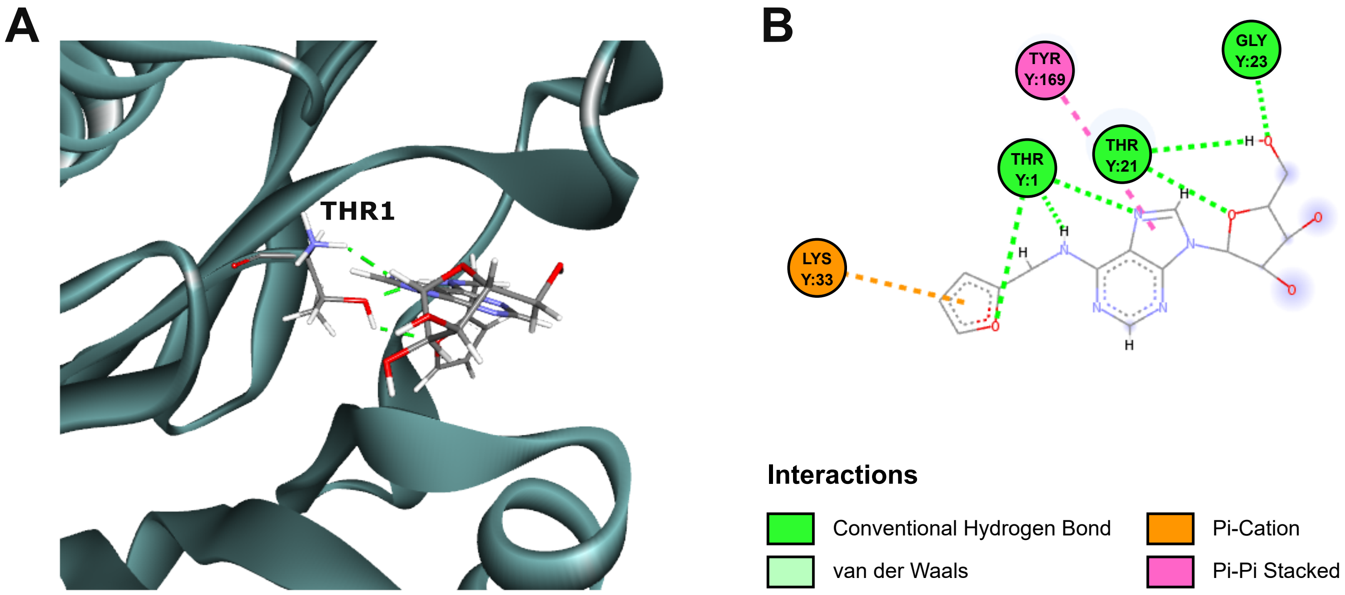

Supplementary Figure 3. (**A**) Zoomed-in section of the complex between the β5-proteasome and compound Kinetin riboside as determined by molecular docking calculations. Only the Thr1 residue of the β5 protein subunit and the hydrogen bonds between Thr1 and compound Kinetin riboside are shown. (**B**) 2D Ligand Interaction Diagram generated by Discovery Studio – BIOVIA software.

Supplementary Fig. 3. Larsson *et al*. (2024)
