# Supplementary material for: Integrated transcriptomics- and structure-based drug repositioning identifies drugs with proteasome inhibitor properties
Source: Sci Rep. 2024 Aug 13;14:18772. doi: 10.1038/s41598-024-69465-6 (PMC11322189; doi:10.1038/s41598-024-69465-6)
Supplement: Supplementary file 5 — Supplementary Figure S5. [file 41598_2024_69465_MOESM5_ESM.pdf]

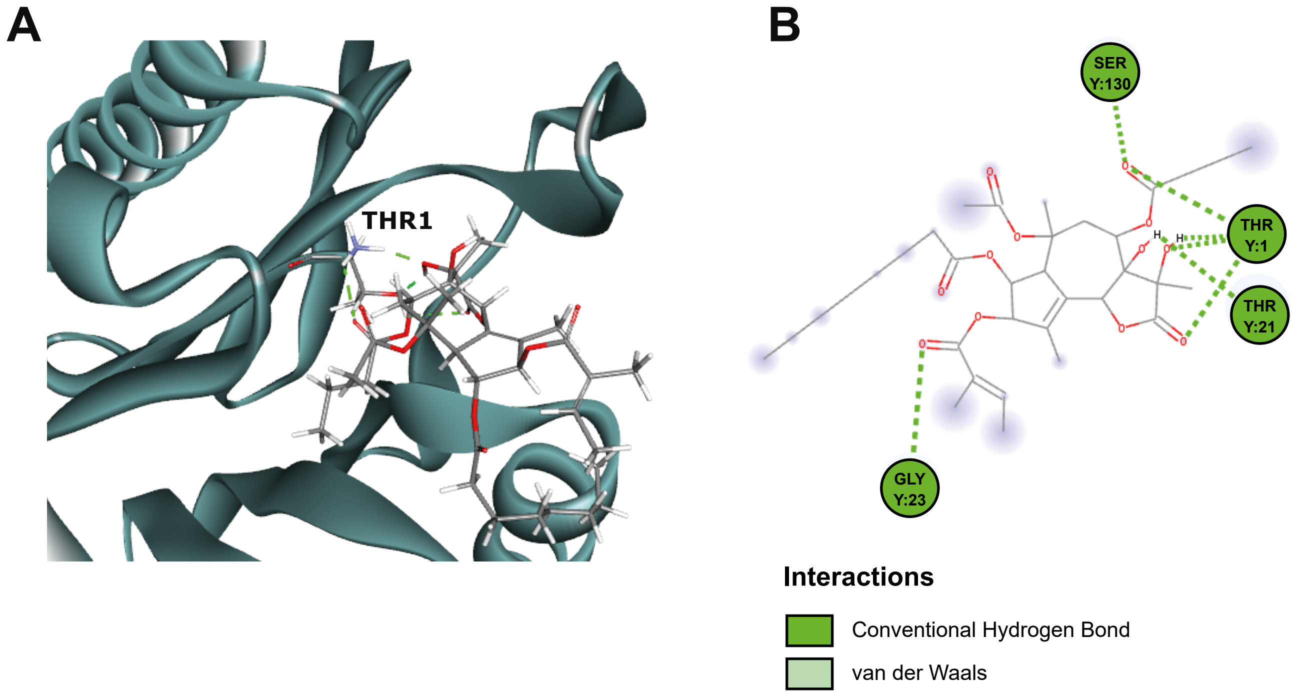

Supplementary Figure 5. (**A**) Zoomed-in section of the complex between the β5-proteasome and compound Thapsigargin as determined by molecular docking calculations. Only the Thr1 residue of the β5 protein subunit and the hydrogen bonds between Thr1 and compound Thapsigargin are shown. (**B**) 2D Ligand Interaction Diagram generated by Discovery Studio – BIOVIA software.

Supplementary Fig. 5. Larsson *et al*. (2024)
