# Supplementary figures and images for: Integrated transcriptomics- and structure-based drug repositioning identifies drugs with proteasome inhibitor properties
Source: Sci Rep. 2024 Aug 13;14:18772. doi: 10.1038/s41598-024-69465-6 (PMC11322189; doi:10.1038/s41598-024-69465-6)

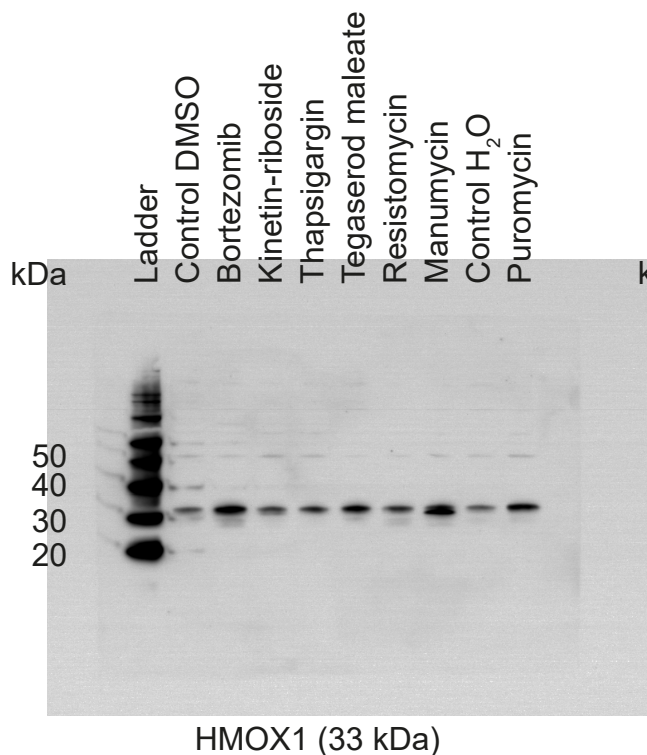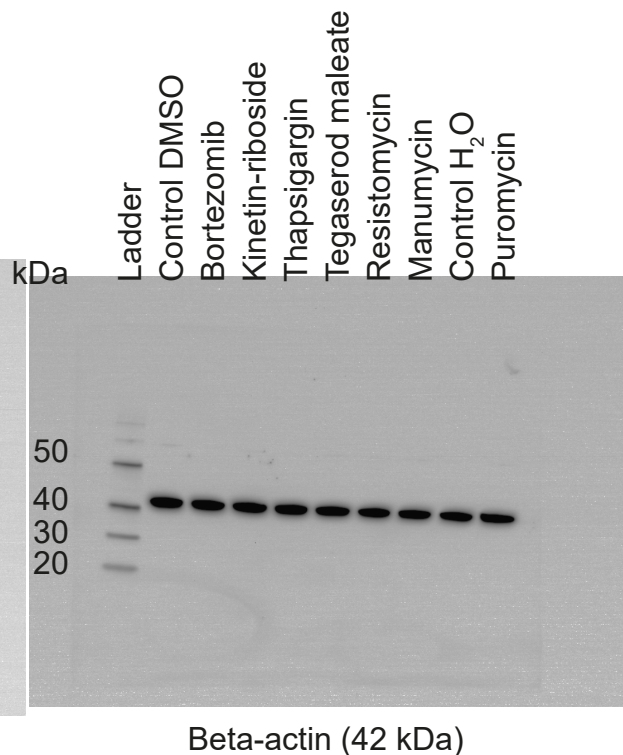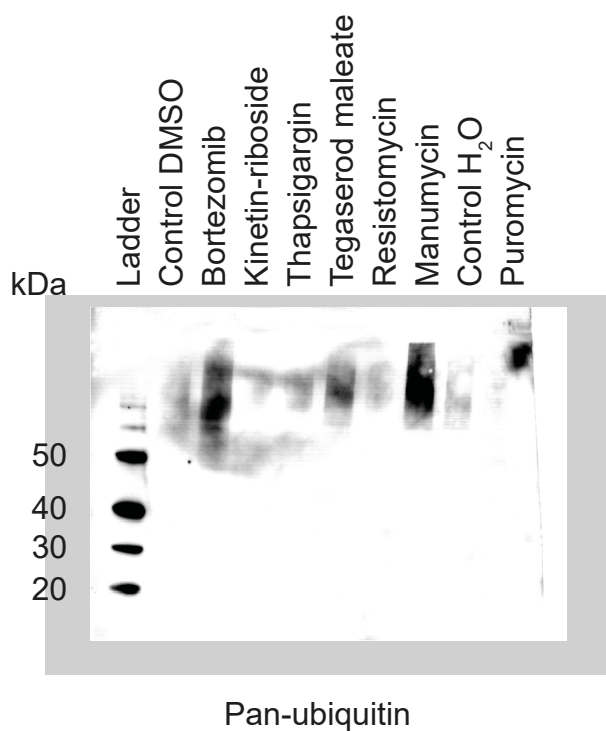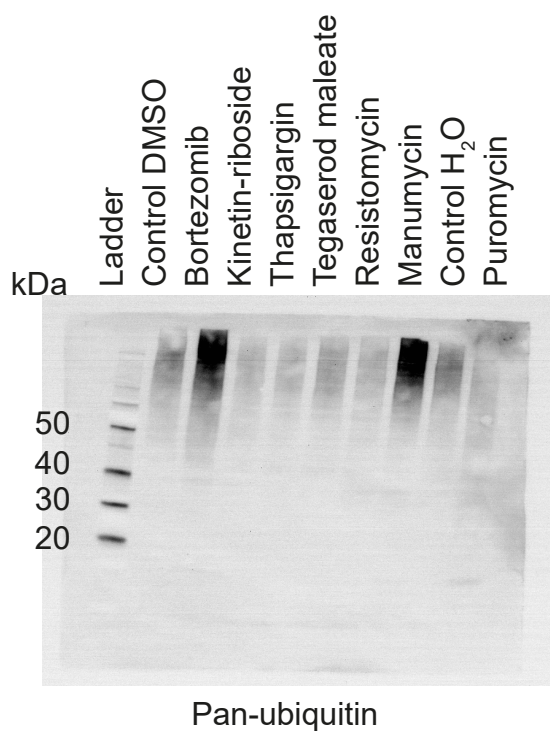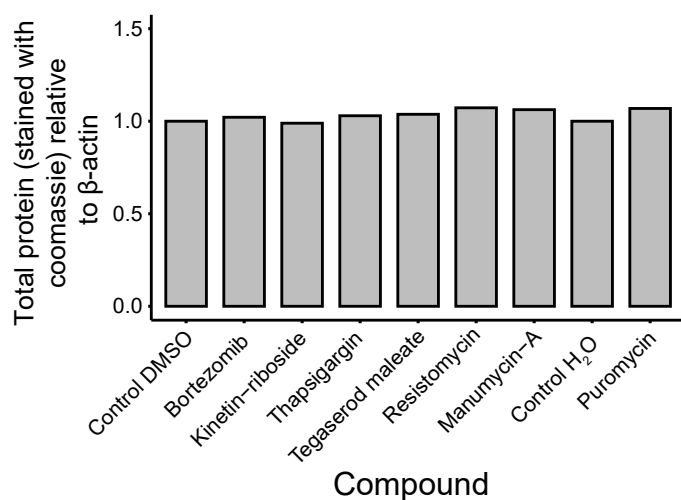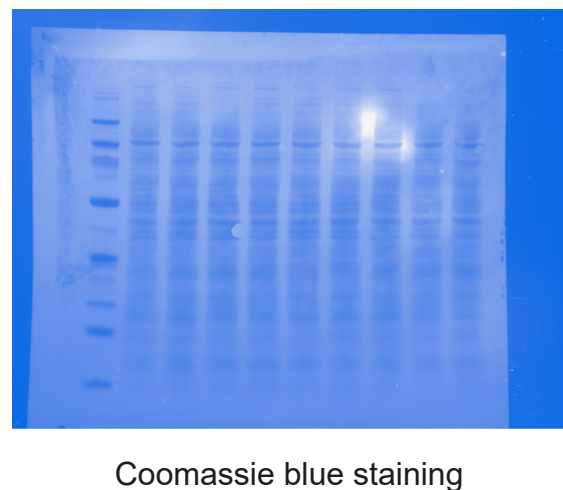

Supplement: Supplementary file 10 — Supplementary Figure S10. [file 41598_2024_69465_MOESM10_ESM.pdf]
