# Supplementary material for: Integrated transcriptomics- and structure-based drug repositioning identifies drugs with proteasome inhibitor properties
Source: Sci Rep. 2024 Aug 13;14:18772. doi: 10.1038/s41598-024-69465-6 (PMC11322189; doi:10.1038/s41598-024-69465-6)
Supplement: Supplementary file 11 — Supplementary Table S1. [file 41598_2024_69465_MOESM11_ESM.pdf]

## Supplementary Table 1. Top 250 differentially regulated genes (log2) for bortezomi

| Gene Symbol | PG_292 | PG_293 | PG_294 | PG_295 | PG_296 | PG_297 | PG_298 | PG_299 |
|---|---|---|---|---|---|---|---|---|
| *ADGRA3* | -1.662872 | -0.877233 | -2.754764 | -1.314133 | -1.895286 | -2.050113 | -0.318038 | -1.161311 |
| *ADNP* | -2.688425 | -1.451636 | -2.069496 | -1.945150 | -1.981272 | -1.482007 | -0.269544 | -0.252585 |
| *AHNAK2* | -0.579105 | -0.232580 | -2.620483 | -0.175001 | -0.274184 | -2.435455 | -0.643936 | -0.562972 |
| *AIDA* | -4.911614 | -1.604641 | -2.863989 | -3.705121 | -3.849426 | -1.285345 | 0.0567034 | -0.003495 |
| *ALDH18A1* | -3.415314 | -1.269744 | -2.127290 | -2.275247 | -2.149322 | -0.921904 | 0.1018500 | 0.1186629 |
| *ALG6* | -1.620819 | -1.059104 | -2.369194 | -0.827612 | -1.841583 | -1.632561 | -0.398135 | -0.880053 |
| *AMD1* | -1.125032 | -1.477807 | -0.881279 | -0.892241 | -2.351917 | -0.443052 | -0.462277 | -1.445082 |
| *ANXA4* | -1.505078 | -0.614477 | -2.593857 | -0.880778 | -1.389232 | -2.012897 | 0.4368141 | 0.2663989 |
| *AREL1* | -1.772219 | -0.547536 | -1.005738 | -0.104155 | -1.307564 | -0.883607 | -1.114915 | -0.610588 |
| *ARL4C* | -0.445242 | -1.411037 | -2.806271 | -0.670249 | -2.438755 | -2.197430 | 0.0446147 | -0.564745 |
| *ARPC1A* | -2.815796 | -0.926734 | -1.829620 | -1.715217 | -2.112576 | -1.136794 | -0.052687 | 0.0219050 |
| *ARPC5* | -2.769393 | -1.736292 | -2.570985 | -2.333733 | -2.922536 | -1.839256 | 0.0888889 | -0.056076 |
| *ASPM* | -2.956055 | -1.025887 | -2.022406 | -1.454415 | -2.211276 | -1.623165 | -0.026303 | -1.119677 |
| *ATF3* | -0.704770 | 3.0989475 | 2.6872175 | -0.919951 | 3.5510221 | 3.5022309 | 0.8599777 | 2.7063123 |
| *B3GNT2* | -2.275790 | -2.184804 | -2.004393 | -1.869655 | -2.103531 | -1.808662 | 0.1268484 | -0.128185 |
| *BAG3* | -0.623455 | 2.3277217 | 2.8904574 | -0.111634 | 2.9001216 | 3.4370167 | -0.461336 | 1.5149835 |
| *BCOR* | -1.646552 | -2.269230 | -0.741997 | -1.407717 | -2.370141 | 0.1396644 | -1.402765 | -1.303939 |
| *BDNF* | -0.769166 | -1.313679 | -1.139483 | -1.009431 | -1.407597 | 0.2454282 | -2.092136 | -2.261907 |
| *BHLHE41* | -1.001076 | -1.645435 | -3.035132 | -1.129704 | -2.422624 | -2.685957 | -1.106076 | -1.681248 |
| *BIRC3* | -1.531890 | -2.244137 | -4.499211 | -1.951934 | -1.896267 | -4.606056 | -1.661768 | -0.368097 |
| *BLMH* | -3.400625 | -0.954626 | -1.416634 | -2.202791 | -2.104331 | -0.462267 | -0.201755 | 0.3298225 |
| *BRINP1* | -0.135653 | -1.520719 | -3.172430 | -0.292011 | -1.885943 | -2.686770 | -0.570792 | -0.528740 |
| *BSDC1* | 0.8880815 | 1.6242040 | 1.3952960 | 0.8514468 | 1.5325352 | 1.1593996 | 0.0376801 | 0.5297608 |
| *C1GALT1C1* | -0.765580 | -1.283825 | -3.330701 | -1.102203 | -2.700745 | -3.463449 | -0.302629 | -1.194906 |
| *C6orf120* | -1.239017 | -1.450613 | -3.248837 | -1.336932 | -2.553620 | -3.116720 | -0.258084 | -0.924397 |
| *CAMK2G* | -0.365954 | 1.0321865 | 1.0328829 | 0.1357868 | 0.6135661 | 0.8214001 | -0.064572 | 0.4618942 |
| *CAPRIN1* | -2.987828 | -2.286683 | -2.329170 | -2.623869 | -2.778462 | -1.330621 | 0.2367567 | 0.0921513 |
| *CASD1* | -3.419209 | -1.492795 | -2.763536 | -2.472726 | -2.283884 | -1.551423 | -0.402179 | -0.959702 |
| *CAV1* | -1.470195 | -0.765697 | -2.240705 | -0.621797 | -1.691695 | -2.060599 | -0.291901 | -0.608751 |
| *CD164* | -2.529345 | -1.254554 | -2.757201 | -1.722691 | -2.118980 | -2.225889 | 0.0147926 | -0.191054 |
| *CD24* | -0.776330 | -0.477384 | -4.093165 | -0.351207 | -1.497044 | -3.839247 | -0.144942 | -0.487905 |
| *CD70* | -0.016650 | -0.276722 | -2.425969 | 0.1000043 | -0.410099 | -2.688539 | 0.7912077 | 0.1943354 |
| *CDC6* | -2.020858 | -1.371228 | -0.249468 | -1.784647 | -1.326448 | 0.5461514 | -0.800980 | -1.137304 |
| *CDCA3* | -1.524264 | -0.732784 | -1.926189 | -0.993262 | -1.103909 | -1.641481 | -0.302429 | -0.525147 |
| *CDH6* | -1.449736 | -0.725643 | -3.907020 | -0.937089 | -1.305268 | -3.365188 | 0.3371661 | -0.669049 |
| *CDKN1B* | -2.427688 | -0.915183 | -2.234173 | -2.094159 | -1.850108 | -1.349091 | -0.585380 | -1.092434 |
| *CENPA* | -1.269245 | -1.270594 | -1.453561 | -1.405162 | -1.461974 | -1.416125 | -0.043622 | -0.514328 |
| *CHAC1* | 0.5871371 | 4.2826638 | 1.8144757 | 1.8209633 | 4.1588507 | 0.5095787 | 1.7609132 | 2.5590175 |
| *CHST11* | -3.009830 | -1.321759 | -0.721075 | -1.391458 | -2.204833 | 0.1347485 | -0.588080 | -0.040734 |
| *CITED2* | -1.842463 | -2.013333 | -2.043202 | -2.014699 | -2.583374 | -1.416243 | -0.479414 | -2.090461 |
| *CLPB* | 0.4144647 | 0.8661703 | 2.0056015 | 0.6262069 | 0.9734907 | 1.7963828 | -0.338410 | -0.292123 |
| *CMC4* | -0.436921 | -0.515330 | -1.960001 | -0.158498 | -1.157011 | -2.032435 | 0.1573111 | -0.634184 |
| *CNN3* | -2.417955 | -0.706980 | -1.767463 | -1.324604 | -1.477786 | -0.378176 | -0.338952 | -0.380111 |
| *COMMD10* | -3.950838 | -1.575060 | -3.336274 | -2.947428 | -2.759405 | -2.222969 | 0.0051631 | -0.445752 |
| *CRY2* | 1.0150533 | 0.8422802 | 1.7695869 | 0.9096990 | 1.0635256 | 1.1376711 | -0.077943 | 0.8607121 |
| *CRYAB* | 0.0522492 | 0.4584177 | 3.1199857 | 0.0096613 | 1.6717065 | 5.4039490 | -0.281257 | 0.0662168 |
| *CSNK1E* | 1.7268113 | 0.4046127 | 0.6482079 | 0.7913330 | 0.2593029 | -0.143862 | -0.307686 | -0.302165 |

| | | | | | | | | |
|---|---|---|---|---|---|---|---|---|
| *CSNK1G3* | -2.7893249 | -1.3327491 | -1.2935718 | -2.3859677 | -2.1629735 | -0.2657268 | -0.1221158 | 0.4713601 |
| *CTSC* | -2.6202902 | -1.0897915 | -2.5827468 | -1.4169105 | -1.5780545 | -1.6699984 | -0.5073802 | -0.7611125 |
| *CUL1* | -2.8172728 | -0.7375149 | -0.9917114 | -1.6828989 | -1.6092357 | -0.2997360 | -0.2044485 | 0.3972462 |
| *CXCL2* | -0.9288250 | 1.2439816 | -0.9470072 | -0.5603904 | 0.6003060 | -0.2239039 | -1.3613862 | 0.8286886 |
| *DACT1* | -1.4214956 | -2.1579490 | -1.7172353 | -1.3519094 | -1.8562584 | -0.6989947 | -0.3815156 | -0.1704860 |
| *DAPP1* | 1.7942568 | 0.1441391 | 0.8777672 | 0.9157457 | 0.9453061 | 0.6472654 | -0.4074101 | -0.3104381 |
| *DBF4* | -1.8569616 | -0.4473880 | -1.2893780 | -0.9969223 | -1.8743184 | -0.5427739 | -0.5546311 | -1.0059897 |
| *DDIT3* | 0.3022796 | 1.4740091 | 2.1263620 | 0.6527424 | 1.7137155 | 1.9179164 | 0.0783247 | 1.3596899 |
| *DDIT4* | -1.1026378 | 3.0359086 | -0.5416495 | -0.4914316 | 3.1504246 | -1.8782990 | 0.9277628 | 1.1091962 |
| *DENND4C* | -2.1904029 | -0.6589186 | -0.7544916 | -1.3516003 | -0.9821658 | -0.8864387 | -0.3130601 | -0.2093744 |
| *DNAAF2* | -1.7254674 | -1.6837272 | -1.9370707 | -1.6119956 | -2.4411281 | -1.9189477 | -1.1995722 | -1.6430817 |
| *DNAJB1* | -1.3561404 | 1.9237220 | 2.8779410 | -0.6740207 | 2.7552373 | 3.6383901 | 0.2339213 | 2.0459915 |
| *DNAJB4* | -1.6317447 | 0.6550476 | -0.3327655 | -1.2410915 | 0.5336830 | 1.0472092 | 0.1850493 | 1.6946822 |
| *DPY19L1* | -2.3309517 | -1.2120694 | -1.5615318 | -1.3132936 | -2.2578844 | -0.8002818 | -0.1012254 | -0.5022705 |
| *E2F5* | -2.7920181 | -1.9749058 | -2.0631617 | -2.0944270 | -2.2080701 | -1.5996254 | -0.5147530 | -0.6204645 |
| *EGLN1* | -0.6261858 | -1.1709308 | -0.9417514 | -0.7686067 | -1.8056053 | -0.8060680 | -0.3334202 | -0.5779019 |
| *EID1* | -1.7940903 | -1.1596933 | -3.7328946 | -1.4918701 | -3.1348111 | -3.4473902 | -0.1008501 | -0.4044042 |
| *EIF2B1* | -2.0510546 | -0.9178330 | -1.3033126 | -0.9865409 | -1.9427118 | -1.2234085 | -0.3660396 | -0.6120096 |
| *ELP5* | -2.3249250 | -1.7339680 | -2.4710283 | -1.9411915 | -2.0069583 | -2.0813202 | -0.4249312 | -0.8483727 |
| *EML4* | -2.6394347 | -2.6230032 | -2.9105780 | -2.1936107 | -2.3468028 | -2.7821848 | -0.0128808 | -0.3169083 |
| *ERAP2* | -1.7103010 | 0.1530562 | -2.9050283 | -0.7222793 | -0.6690894 | -2.2114893 | 0.2664607 | -0.1486700 |
| *ERCC6L* | -0.8551523 | -1.2492610 | -1.5213896 | -1.3139323 | -1.9324456 | -1.4595833 | -0.6517125 | -1.6038787 |
| *EXOC3* | 1.4449519 | 0.4617329 | 1.2141890 | 0.7292937 | 0.2780679 | 0.7398105 | 0.2641840 | 0.1663235 |
| *EXOC5* | -2.6458253 | -3.1982090 | -3.9538008 | -3.4412895 | -3.5706970 | -2.8468763 | 0.4365039 | 0.8342965 |
| *FAIM* | -2.9043218 | -1.3578826 | -2.9161844 | -2.1151638 | -2.0913044 | -1.8558490 | -0.5056675 | -0.7845179 |
| *FASTKD3* | -1.6702749 | -1.0176376 | -0.7971652 | -0.8823082 | -1.6361806 | -0.1235777 | -0.0165547 | -0.1987596 |
| *FJX1* | 0.3755079 | -1.8842751 | -1.5335519 | -0.2276872 | -2.3429379 | -2.4745707 | -1.7261172 | -1.9143386 |
| *GABARAPL1* | -0.2137644 | 1.2230897 | 1.9469479 | -0.2345111 | 1.1152302 | 1.9331875 | 0.8784284 | 1.9808112 |
| *GADD45A* | -0.8770146 | 1.0708769 | 1.4335428 | -0.5928740 | 1.3886422 | 2.3635374 | 0.1587144 | 1.3921193 |
| *GALNT7* | -0.5875770 | -0.4175255 | -2.0522348 | -0.5504105 | -0.9060899 | -1.9181505 | -0.0584675 | -0.1546814 |
| *GCNT1* | -0.5657643 | -1.3570476 | -2.0897189 | -0.5938424 | -1.6012022 | -1.1635191 | -0.3540867 | -1.1860835 |
| *GDE1* | -4.9648634 | -2.4718013 | -3.6922480 | -4.3542253 | -3.6526046 | -2.2620231 | 0.1006079 | -0.2497635 |
| *GEMIN6* | -0.9842428 | -1.6579520 | -1.5082244 | -1.2702952 | -1.8126380 | -1.3882731 | -0.8434278 | -1.3024923 |
| *GET1* | -3.1854158 | -1.0380433 | -3.1240125 | -2.1930755 | -2.8100372 | -1.0191430 | -0.1367355 | -0.9840822 |
| *GLA* | -0.5315155 | 0.9208648 | 1.4665030 | -0.2630098 | 0.6932038 | 1.3518745 | -0.4105468 | -0.2244035 |
| *GNG11* | -0.9113924 | -0.4531403 | -2.4798189 | -0.6459110 | -1.8022322 | -2.1634164 | 0.0906215 | -0.2580272 |
| *GRB14* | -1.9596750 | -0.8223840 | -2.9766556 | -1.9479719 | -1.7274538 | -2.3737921 | -0.4143015 | -0.8047419 |
| *GTF2H5* | -1.1905137 | -0.8580407 | -2.5429669 | -1.1002848 | -2.2617471 | -2.4943321 | 0.2933477 | -0.3329718 |
| *HGSNAT* | 0.1325378 | -0.0018926 | -2.6791609 | 0.5107113 | -0.8985017 | -3.0036792 | -0.2027571 | -0.4234596 |
| *HILPDA* | 0.2334623 | -1.3177448 | -2.5370544 | -0.2115802 | -1.6745693 | -2.7333655 | -0.5045983 | -1.2752719 |
| *HMG20A* | -2.1333062 | -0.7989263 | -1.5113518 | -1.1557054 | -1.1278856 | -1.0439170 | -0.6117412 | -0.6834011 |
| *HMGA2* | -1.2100245 | -2.4149962 | -0.2753270 | -0.4903000 | -2.2991383 | 0.4438918 | -1.6701075 | -2.1013065 |
| *HMOX1* | 0.3864693 | 4.0033202 | 4.4214709 | 1.1177733 | 4.7248602 | 4.4200708 | -0.2317880 | 1.4158551 |
| *HNRNPA2B1* | -2.1201854 | -0.4233795 | -0.8858157 | -1.1339258 | -1.1987031 | -0.3633121 | 0.0001353 | -0.0357529 |
| *HNRNPF* | -2.4050378 | -1.7463482 | -2.2565445 | -2.5865532 | -2.8546142 | -2.1919895 | 0.0156309 | -0.1512740 |
| *HOXA10* | -0.2973615 | -1.4715744 | -2.7047650 | -0.8394096 | -2.1000096 | -2.1775148 | -0.6291371 | -1.2134804 |
| *HSPA6* | 0.2900737 | 5.8748479 | 7.4349865 | 0.3988816 | 7.2895224 | 8.8478397 | -0.1762168 | 3.5398061 |
| *HSPH1* | -0.5171360 | 1.0314801 | 1.7778597 | -0.5010943 | 1.7985881 | 2.4181805 | -0.5670840 | 1.4026085 |
| *ID1* | -3.0453685 | -2.2868920 | -5.1556998 | -2.5811104 | -2.3596017 | -3.5771581 | 0.2569479 | -0.2444400 |
| *IDH1* | -1.1934579 | -0.3421618 | -2.6576827 | -0.9789415 | -1.0109685 | -2.3003373 | -0.0171722 | 0.0289039 |

| | | | | | | | | |
|---|---|---|---|---|---|---|---|---|
| *IGFBP3* | -2.277483 | -1.370182 | -4.447870 | -1.857801 | -2.263797 | -3.181954 | 0.5629424 | 0.4848231 |
| *IK* | -0.689099 | -1.192526 | -0.598933 | -1.299173 | -0.638484 | -0.322418 | -0.004401 | -0.288896 |
| *IL10RB* | -1.359897 | -0.438611 | -0.801148 | -0.590386 | -1.304348 | -0.752046 | 0.2843786 | 0.0711097 |
| *ILF2* | -2.007725 | -0.602141 | -1.162586 | -1.263076 | -1.173354 | -0.494094 | 0.0342677 | -0.076358 |
| *IMP3* | -1.218971 | -1.851333 | -2.908269 | -1.626811 | -2.849754 | -3.416245 | -0.727156 | -1.612606 |
| *INSIG1* | -1.379807 | -0.691418 | -3.152438 | -1.230873 | -1.070437 | -2.495460 | -0.413741 | -1.332822 |
| *IREB2* | -2.841985 | -1.784219 | -2.038147 | -2.613312 | -1.797234 | -1.346417 | 0.0693516 | 0.0993893 |
| *KCNQ1DN* | 1.3914679 | -0.029177 | 0.0472248 | 0.1267479 | -0.245705 | 0.2424765 | -0.399854 | -0.326809 |
| *KCTD17* | 1.2620778 | 0.2632574 | 0.8901353 | 0.3551994 | 0.2546985 | 0.2178854 | -0.277128 | -0.241029 |
| *KCTD5* | -1.662818 | 0.7622834 | 0.9604508 | -0.273109 | 0.4969174 | 1.4185277 | 0.1075238 | 1.0517054 |
| *KDELR3* | -1.091403 | -0.816848 | -2.704204 | -0.734705 | -1.442929 | -1.863825 | -0.061214 | -0.353026 |
| *KIF18A* | -3.188150 | -1.396601 | -1.880323 | -2.610870 | -2.204227 | -1.013000 | 0.0256371 | -0.558315 |
| *KIF20A* | -0.355552 | -0.203244 | -1.855924 | -0.219109 | -0.318413 | -1.874827 | 0.1213159 | -0.815532 |
| *KIF23* | -1.918441 | -0.522020 | -1.205352 | -0.954906 | -1.143795 | -0.717484 | 0.2086743 | -0.328089 |
| *KIFBP* | -1.704254 | -0.726127 | -1.269640 | -1.281746 | -1.583161 | -1.067969 | -0.126836 | -0.134205 |
| *KLK15* | 1.1353280 | -0.186531 | -0.099941 | 0.1176739 | 0.1696832 | -0.093432 | 0.0268039 | -0.170740 |
| *KMT5B* | -1.722628 | -1.607586 | -2.411650 | -1.545426 | -1.996483 | -1.795408 | -0.114909 | -0.312674 |
| *KRT33B* | 1.1642879 | -0.135743 | 0.0645171 | 0.4155944 | 0.1048239 | -0.079493 | 0.0039558 | -0.277318 |
| *LIG4* | -0.280794 | -1.312451 | -0.818221 | -0.637675 | -0.240359 | -1.258438 | -0.036902 | -0.597992 |
| *LIN37* | 0.7527729 | 1.0879725 | 2.5363441 | 0.3703070 | 1.9401732 | 1.7652861 | -0.114788 | 0.3022985 |
| *LIPA* | -1.862810 | -0.636422 | -1.859577 | -0.680379 | -1.257531 | -2.097156 | -0.345617 | -0.509908 |
| *LMBRD1* | -2.777509 | -1.204672 | -2.108719 | -1.742103 | -2.339530 | -1.405309 | 0.4204578 | -0.124817 |
| *LRRC17* | -0.630765 | -0.856231 | -4.178595 | -0.480047 | -2.424273 | -4.112206 | 0.2040416 | 0.1207152 |
| *LRRC8D* | -1.903770 | -1.091950 | -1.928669 | -1.267152 | -1.671273 | -1.892237 | -0.245235 | -0.859419 |
| *LYPD1* | -0.888481 | -1.577660 | -1.686669 | -0.686213 | -1.590015 | -1.958251 | -0.177696 | -1.135313 |
| *LYRM2* | -2.118449 | -1.062447 | -2.590264 | -0.941817 | -2.347841 | -2.164133 | -0.014849 | -0.488968 |
| *M6PR* | -1.818188 | -0.603047 | -0.816883 | -0.945096 | -1.194329 | -0.248011 | -0.012734 | -0.321589 |
| *MAPKBP1* | 1.6813472 | 0.6328332 | 1.0843019 | 0.8564346 | 0.8393547 | 0.5180210 | -0.119948 | 0.1976467 |
| *MARCKS* | -0.758414 | -0.884003 | -2.985124 | -0.220911 | -2.177130 | -2.079628 | 0.1069640 | -0.659968 |
| *MCM10* | -3.101264 | -0.920722 | -0.699074 | -1.584704 | -1.778391 | 0.1523796 | -0.855510 | -0.926795 |
| *MEIS2* | -0.425176 | -1.125472 | -2.124855 | -0.183927 | -1.341129 | -1.404795 | -0.766642 | -1.968966 |
| *MINPP1* | -2.215826 | -1.254083 | -2.115314 | -1.741011 | -2.313814 | -1.868313 | -0.370258 | -0.480773 |
| *MITF* | -1.588550 | -0.563312 | -0.445743 | -1.038299 | -1.274293 | -0.323116 | -0.821832 | -1.389442 |
| *MPDU1* | -2.017196 | -0.312721 | -1.226301 | -0.979575 | -1.111955 | -0.500755 | 0.1412579 | 0.0748450 |
| *MPV17* | -1.640057 | -0.512010 | -2.323392 | -0.541362 | -1.078661 | -1.660963 | -0.550892 | -0.580156 |
| *MRFAP1L1* | -2.229893 | -1.133655 | -2.758180 | -1.643835 | -2.530352 | -2.471718 | -0.123489 | -0.607866 |
| *MRPS28* | -1.916445 | -1.312125 | -1.662880 | -1.906003 | -1.720910 | -1.049631 | -0.282362 | -0.815629 |
| *MSH2* | -4.792150 | -1.751057 | -3.480538 | -3.169699 | -3.521607 | -1.497253 | -0.350964 | -0.792094 |
| *MTHFSD* | -0.717845 | -0.878007 | -0.978025 | -1.007333 | -1.005305 | -0.855210 | -0.509365 | -0.470314 |
| *MYBL1* | -1.318002 | -1.081759 | -0.488724 | -0.794958 | -1.781891 | -0.007887 | -0.790196 | -1.215229 |
| *MYC* | -3.435046 | -0.577439 | 0.5570234 | -2.551386 | -1.426609 | 1.5038196 | 0.2972356 | 0.1781994 |
| *NCAPG2* | -2.775540 | -1.055174 | -1.628139 | -1.717444 | -1.404014 | -0.043942 | 0.0104884 | -0.200162 |
| *NDC1* | -3.034459 | -1.468885 | -1.555931 | -2.123098 | -2.967772 | -1.297755 | -0.053522 | -0.299801 |
| *NEDD4* | -2.387528 | -0.594291 | -1.533293 | -1.283698 | -1.399370 | -1.328836 | 0.2255735 | 0.1240409 |
| *NEFL* | -0.912945 | -0.358994 | -3.125682 | -0.715942 | -1.471304 | -2.532762 | 0.3680431 | 0.0694072 |
| *NFE2L2* | -3.041698 | -0.191872 | -1.182815 | -1.410089 | -0.738327 | -0.375851 | -0.109416 | 0.1835164 |
| *NFKB1* | -2.780739 | -1.044924 | -1.472540 | -1.555270 | -1.701057 | -1.065537 | -0.605442 | -0.295586 |
| *NRBF2* | -2.752385 | -0.466552 | -1.123446 | -1.799473 | -1.118708 | -0.349994 | 0.1138144 | 0.7631441 |
| *NREP* | -1.147376 | -1.333920 | -3.482882 | -1.127316 | -2.243384 | -3.034696 | 0.2213448 | -1.318498 |
| *NUBPL* | -2.411992 | -1.389351 | -2.473133 | -1.719933 | -1.579055 | -2.113173 | -0.986227 | -0.948780 |

| | | | | | | | | |
|---|---|---|---|---|---|---|---|---|
| *NUDT21* | -2.797511 | -1.725838 | -1.761562 | -2.382299 | -2.439343 | -0.755517 | 0.0005401 | -0.253012 |
| *NUDT6* | -2.283693 | -1.929959 | -2.440861 | -2.227213 | -2.347267 | -2.676939 | -1.063803 | -1.980859 |
| *NUP42* | -1.793251 | -0.699457 | -0.790751 | -0.982850 | -0.908485 | -0.076115 | -0.599374 | -0.412672 |
| *NUPR1* | -0.088833 | 1.5886739 | 1.6189674 | 0.0007640 | 1.6580766 | 1.3865189 | 0.6792178 | 1.1229883 |
| *OSGEPL1* | -1.614383 | -1.574799 | -1.958464 | -1.777506 | -1.418954 | -1.928979 | 0.2314392 | -0.753874 |
| *OSGIN1* | 0.2176074 | 1.4162251 | 1.3994576 | 0.5645214 | 1.5289758 | 0.6152127 | -0.015808 | 0.4803455 |
| *OXA1L* | -2.427280 | -1.237467 | -1.961749 | -1.516481 | -2.052155 | -1.526939 | -0.546625 | -0.529479 |
| *OXSM* | -1.746887 | -0.898301 | -2.403958 | -1.167759 | -1.589248 | -1.905349 | -0.754514 | -1.053913 |
| *PAGR1* | -1.111067 | -1.122355 | -1.542081 | -0.709236 | -1.828399 | -1.520337 | -0.457385 | -1.223331 |
| *PANX1* | -1.563487 | -1.440143 | -1.080941 | -1.304350 | -1.780249 | -1.053146 | -0.667382 | -0.455674 |
| *PAXBP1* | -2.098836 | -0.560513 | -0.242562 | -0.905424 | -1.022525 | 0.1357713 | -0.315042 | 0.2227008 |
| *PCTP* | -1.673128 | -1.211668 | -2.292483 | -0.890805 | -1.946111 | -1.373733 | -0.344411 | -0.766271 |
| *PDGFC* | -0.950856 | -1.241352 | -1.587930 | -0.794843 | -1.738147 | -0.865041 | -0.086287 | -1.063456 |
| *PDZD8* | -2.348425 | -1.069626 | -1.302089 | -1.569828 | -1.971318 | -0.374542 | -0.149514 | -0.255539 |
| *PDZK1* | -1.405770 | -0.222787 | -5.022506 | -0.406555 | -1.923206 | -3.632903 | -0.702836 | -0.789000 |
| *PEG10* | -1.923899 | -1.403128 | -3.738327 | -1.470023 | -1.846900 | -2.741643 | 0.0244604 | -0.385015 |
| *PELP1* | 2.1010498 | 0.6739345 | 1.5120974 | 1.1176856 | 1.1291441 | 0.4839754 | -0.756627 | -0.828962 |
| *PEX11B* | -1.333665 | -0.602075 | -2.806880 | -0.957512 | -1.236466 | -2.546789 | -0.355823 | -0.857234 |
| *PEX12* | -0.869898 | -1.227600 | -1.603269 | -0.860323 | -1.333891 | -1.464656 | -0.337919 | -0.744764 |
| *PEX2* | -3.839768 | -1.706446 | -3.574295 | -2.295502 | -3.272350 | -1.792115 | -0.261384 | -0.836054 |
| *PGF* | 0.9160058 | 0.4298477 | 2.6339068 | 0.5222969 | 1.5098957 | 2.6366106 | -0.277362 | -0.075585 |
| *PGGT1B* | -1.090016 | -1.505794 | -1.562206 | -1.428861 | -1.655610 | -1.317492 | -0.059882 | 0.3258054 |
| *PGRMC2* | -0.696540 | -1.206001 | -3.021473 | -0.830203 | -2.019985 | -2.278927 | -0.144281 | -0.845172 |
| *PIGN* | -1.621642 | -0.941378 | -1.731464 | -1.283101 | -1.622299 | -1.273642 | -0.124807 | -0.305849 |
| *PIGV* | -0.070384 | -0.721714 | -2.408474 | -0.452761 | -0.815177 | -2.143105 | -0.208703 | -1.042173 |
| *PJA2* | -1.440209 | -0.634918 | -2.495527 | -1.067251 | -1.820508 | -1.083444 | -0.045267 | -0.171936 |
| *PNMA1* | -2.076650 | -1.591861 | -2.864081 | -1.705442 | -2.032786 | -3.140276 | -0.577965 | -0.671856 |
| *PNRC2* | -1.488400 | -1.222627 | -1.883514 | -1.001328 | -1.708583 | -1.185880 | -0.760066 | -0.774389 |
| *POLI* | -2.238539 | -1.047642 | -2.273724 | -1.473976 | -1.770306 | -1.611098 | -0.471167 | -0.478433 |
| *POLR3F* | -1.881934 | 0.5941309 | 0.4104864 | -0.836022 | 0.0123575 | 1.4105432 | -0.121486 | 0.8661485 |
| *PPP1R15A* | 0.6775072 | 2.0151328 | 3.9433976 | 1.2853232 | 2.5543811 | 4.2379099 | 0.1830903 | 1.1406103 |
| *PPP4R3B* | -0.253950 | -1.412641 | -0.681558 | -1.291655 | -1.596146 | -0.556682 | -0.739261 | -0.391413 |
| *PRMT5* | -1.835728 | -0.359304 | -0.075374 | -0.705753 | -0.654336 | -0.148775 | -0.330770 | -0.334155 |
| *PSMC3* | -2.625194 | -0.602010 | -0.563033 | -1.411634 | -1.368817 | -0.103133 | 0.1518810 | 0.2427855 |
| *PSMD3* | 0.7738769 | 1.2206891 | 1.8183854 | 1.0260445 | 1.3590345 | 1.4267652 | 0.1099724 | 0.4875498 |
| *PTER* | -2.411264 | -1.055961 | -2.141496 | -1.395114 | -2.371324 | -1.208997 | -0.304218 | -0.584539 |
| *R3HCC1L* | -1.088714 | -1.759914 | -1.699596 | -1.476070 | -1.526331 | -1.547995 | 0.6017077 | 1.0686880 |
| *RABGGTB* | -3.392932 | -1.316448 | -1.155626 | -2.288474 | -1.589466 | 0.0474659 | -0.234835 | 0.5110455 |
| *RBM7* | -2.994365 | -1.216280 | -1.292274 | -1.883131 | -2.022689 | -0.338989 | 0.0116295 | 0.1795607 |
| *RDX* | -2.884610 | -1.661871 | -2.340313 | -2.031525 | -2.237190 | -1.621153 | -0.087504 | -0.129452 |
| *RIN2* | -0.446005 | -1.371335 | -1.282497 | -0.625820 | -1.617368 | -1.431525 | -0.842715 | -1.547063 |
| *RINT1* | -2.042984 | -0.106309 | -0.193017 | -1.426966 | -1.129800 | 0.7887861 | -0.560631 | 0.1834973 |
| *RIT1* | -2.560438 | -0.730568 | -1.157185 | -2.130055 | -2.300771 | 0.0835557 | 0.4264505 | 0.5107845 |
| *RPAP3* | -2.573289 | -0.674624 | -0.895063 | -1.494951 | -0.920072 | -0.607326 | -0.056962 | 0.1428415 |
| *RPL36AL* | -1.259362 | -1.065898 | -2.145649 | -1.074121 | -1.971311 | -1.995507 | -0.473781 | -0.997415 |
| *RPP14* | -2.385496 | -0.996616 | -0.508308 | -1.508291 | -1.388805 | -0.062018 | -0.054308 | 0.1291477 |
| *RRAGA* | -1.341342 | -1.035263 | -3.413518 | -1.112577 | -1.956559 | -3.350640 | -0.461490 | -0.862026 |
| *RRS1* | -1.966080 | -1.809389 | -2.069457 | -1.601957 | -2.325597 | -1.921076 | -0.916832 | -1.224363 |
| *RTL8C* | -0.148402 | -0.391657 | -2.634448 | 0.0171139 | -1.261335 | -2.905033 | -0.147674 | -0.467591 |
| *SELENOT* | -4.213248 | -1.399502 | -2.607655 | -2.571337 | -3.226362 | -1.333395 | -0.142054 | -0.356887 |

| | | | | | | | | |
|---|---|---|---|---|---|---|---|---|
| *SGPP1* | -3.6382341 | -3.2125284 | -3.4110614 | -3.2766301 | -3.5384304 | -2.8770014 | 0.1892072 | 0.4750583 |
| *SH3YL1* | -1.9069716 | -0.8510031 | -1.5457605 | -1.7867890 | -1.3054056 | -0.9823443 | -0.1171098 | -0.0928080 |
| *SKA1* | -2.6351973 | -0.6163013 | -0.6906212 | -1.4189539 | -0.9109861 | -0.2899201 | -0.4884007 | -0.5212280 |
| *SLC26A2* | -1.5857668 | -1.3072696 | -4.0095398 | -1.0486569 | -2.5165054 | -2.9026406 | -1.1606123 | -1.7122273 |
| *SLC35A5* | -1.8635839 | -1.3456246 | -1.9263448 | -1.7193021 | -2.4938249 | -2.2460475 | -0.1355221 | -0.9338325 |
| *SMIM10L1* | -1.2123799 | -0.8405740 | -3.1326195 | -0.7353026 | -1.2125212 | -2.5357515 | -0.1382745 | 0.1315893 |
| *SQSTM1* | -0.2803072 | 1.7295375 | 1.5374185 | 0.3299731 | 1.2827299 | 0.7728100 | 0.1062360 | 0.7696016 |
| *STAG3L3* | 1.6408823 | -0.0239825 | 0.2311362 | 0.5230482 | -0.3883405 | -0.2236027 | -0.3110084 | -0.4842119 |
| *TBL1XR1* | -4.1570592 | -2.7656159 | -4.7250291 | -4.5381485 | -4.2748945 | -2.5395889 | 0.2136777 | -0.1302909 |
| *TDRD7* | -2.3275954 | -0.7008461 | -0.8351421 | -1.0299049 | -1.1270561 | -0.8580467 | -0.4250145 | -0.3500506 |
| *TGIF1* | -1.8072190 | -0.9637795 | -2.0951102 | -1.7474149 | -1.9970132 | -1.4484251 | -0.4101716 | -1.0488986 |
| *THAP1* | -2.1845028 | -1.6223950 | -1.6761816 | -2.2468341 | -2.3524326 | -0.9274158 | -0.7588536 | -0.8565805 |
| *THBS2* | 1.0978773 | -0.0389619 | 0.1286385 | 0.3375961 | 0.0401985 | 0.2949903 | -0.6411632 | -1.2954249 |
| *TM9SF1* | -0.6945823 | -1.3872906 | -1.2448324 | -0.5657677 | -1.3156309 | -1.3658044 | 0.0827789 | 0.0383819 |
| *TMEM14A* | -0.9321949 | -0.6364375 | -2.4269004 | -0.7045763 | -1.5458957 | -2.1493419 | 0.1025346 | -0.3278760 |
| *TMEM185B* | -0.9783892 | -0.9916681 | -2.4419747 | -0.8313911 | -1.3004145 | -2.5544198 | -0.1803038 | -0.2297215 |
| *TMEM230* | -5.3446064 | -3.5934511 | -5.1364284 | -4.3667136 | -4.2823696 | -3.9810649 | 0.0417856 | -0.2467138 |
| *TMEM243* | -1.1114911 | -1.0498855 | -2.6883425 | -1.2486441 | -1.7364065 | -1.8466125 | -0.0434820 | -0.2844857 |
| *TMEM251* | -1.3793657 | -1.9314859 | -3.2937038 | -1.0274183 | -2.9190261 | -3.0147532 | -0.5561511 | -1.2501118 |
| *TMOD3* | -2.0534510 | -2.0555575 | -1.2292754 | -1.6224364 | -2.0015161 | -1.2047468 | 0.4716107 | 0.8635623 |
| *TNFAIP8* | -1.6326589 | -1.2845550 | -3.2069173 | -1.5902226 | -2.0735694 | -2.6776324 | 0.0556365 | 0.0310652 |
| *TNFSF10* | -0.6253785 | -1.5869355 | -5.1791135 | -0.3859275 | -2.5653262 | -4.7012996 | -1.2109218 | -2.0834278 |
| *TNS3* | -0.7009138 | -0.9578157 | -2.5485226 | -0.4266483 | -1.5745215 | -2.1969187 | -0.5603719 | -1.2624178 |
| *TPRKB* | -3.0514289 | -1.0316152 | -0.5455317 | -2.1127457 | -2.1952332 | 0.2200706 | -0.1605615 | -0.2040185 |
| *TRDMT1* | -1.8941355 | -1.0596214 | -1.6477786 | -1.3640588 | -1.5130028 | -1.3778194 | 0.0838317 | -0.0229524 |
| *TRIT1* | -1.9481713 | -0.6062400 | -0.2912440 | -0.8038753 | -0.7704926 | -0.4461874 | -0.5137195 | -0.5401852 |
| *TRMT5* | -1.2856316 | -0.4335283 | -1.8078689 | -0.8739983 | -1.5796398 | -1.3051116 | -0.3424701 | -0.3710291 |
| *TUG1* | -1.8128013 | -0.9326192 | -2.1971969 | -1.1530392 | -1.7745284 | -1.8729881 | -0.4733831 | -0.4227146 |
| *TVP23B* | -2.2941639 | -0.2386103 | 0.2250623 | -0.9018012 | -0.9544660 | 0.6509315 | 0.1407483 | 0.3519416 |
| *UBE2H* | -2.4562218 | -0.9352155 | -0.4808382 | -1.6954431 | -1.4393053 | 0.2992168 | 0.2349455 | 0.5063597 |
| *UBQLN2* | -0.4182598 | -0.3105704 | -3.1253164 | -0.4412167 | -0.8821616 | -2.3344620 | -0.4029382 | -0.2938987 |
| *UGP2* | -4.0209271 | -1.6676555 | -2.8594375 | -2.8703963 | -2.5830949 | -1.5085587 | -0.1586081 | -0.3738950 |
| *UPP1* | 0.4391301 | 0.3970246 | 3.5618848 | 0.0369377 | 1.2752102 | 2.6286092 | -0.8226221 | -0.3209386 |
| *UQCR10* | -1.0073818 | -0.5469263 | -2.2203301 | -0.8664752 | -1.1779039 | -1.8659914 | -0.3757415 | -0.6082433 |
| *USP25* | -1.4009702 | -0.2569194 | -0.6710690 | -0.1969365 | -0.7363882 | -0.7975051 | -0.2078093 | 0.0370879 |
| *UXS1* | -3.1219602 | -0.6889703 | -2.0317898 | -1.4714030 | -2.6457995 | -1.2269655 | -0.1234437 | -0.5501776 |
| *VANGL1* | -1.1422547 | -1.2142388 | -2.5462700 | -0.8833574 | -2.3803114 | -2.2628606 | -0.5528127 | -0.9450581 |
| *VCAM1* | 0.2574288 | -0.4677933 | -4.4008242 | -0.2138621 | -1.1222979 | -5.0646631 | -1.4581651 | -0.8072136 |
| *VIPAS39* | 0.1021040 | 0.7689386 | 0.8225849 | -0.0011555 | 0.8332483 | 0.4444287 | -0.2641325 | -0.0829897 |
| *WASHC3* | -2.7275370 | -0.5181787 | -1.0469966 | -1.4020197 | -1.5019448 | -1.1415429 | -0.2773188 | -0.6531035 |
| *YIPF5* | -4.1077853 | -2.2308645 | -3.4642508 | -3.4867075 | -3.8012321 | -1.5956531 | 0.6288577 | 1.1955567 |
| *YWHAH* | -3.3061101 | -1.4689478 | -3.6000398 | -2.1921152 | -2.8804212 | -2.1870047 | -0.0013721 | -0.7200488 |
| *ZBTB25* | -0.4482331 | -1.0001203 | -0.8253864 | -0.6305563 | -1.1175598 | -0.9797728 | -0.3291946 | -0.6247914 |
| *ZBTB38* | -2.0323767 | -1.1329115 | -3.0827639 | -1.3717072 | -1.9301646 | -1.9826767 | -0.7109501 | -0.7817172 |
| *ZC3H7A* | -3.4247534 | -0.5841088 | -0.6630630 | -1.2155474 | -0.8366794 | 0.3356395 | 0.0727242 | -0.1618154 |
| *ZFYVE21* | -4.0936402 | -2.2963182 | -3.0047411 | -3.1337981 | -3.3160772 | -2.3619231 | -0.1338470 | -0.2220669 |
| *ZMYM6* | -1.0996104 | -1.2625435 | -1.9415068 | -1.1374555 | -1.4441070 | -1.6743545 | -0.0710806 | -0.4305451 |
| *ZNF217* | -1.4975457 | -1.4785241 | -3.1920452 | -1.4213543 | -1.7482722 | -2.4916377 | -0.8064395 | -0.9809386 |
| *ZNF273* | -2.0351445 | -1.6368321 | -1.3503366 | -1.3137031 | -0.9233938 | -0.5355427 | -0.2417845 | -0.1928971 |
| *ZNF318* | -1.0898533 | -1.3377307 | -1.6049578 | -1.2437976 | -1.7320474 | -1.3524491 | -0.6255299 | -0.6121569 |

| | | | | | | | | |
|---|---|---|---|---|---|---|---|---|
| *ZNF430* | -1.4195303 | -0.8754670 | -0.5274969 | -0.3815197 | -0.8344374 | 0.5096519 | -0.3177187 | -0.1580960 |
| *ZNF84* | -1.5039265 | -0.5480500 | -0.3552198 | -0.3559915 | -0.1443921 | 0.2009584 | 0.2106891 | 0.5881671 |
| *ZNHIT3* | -2.0169693 | -0.5811013 | -0.4704258 | -1.1159753 | -1.2375037 | -0.3372239 | -0.2722268 | -0.2415426 |

## ib in the iLINCS dataset

| PG_300 | PG_301 | PG_302 | PG_303 | PG_304 | PG_305 | PG_306 | PG_307 | PG_308 |
|---|---|---|---|---|---|---|---|---|
| -1.608479 | -0.491764 | -1.631986 | -1.195476 | -0.369524 | -0.261827 | -1.067026 | -0.401505 | -0.676124 |
| -1.049246 | -0.274036 | -0.287341 | -1.191817 | -0.438914 | -0.834528 | -0.851515 | -0.561123 | -0.901131 |
| -1.616568 | -0.404326 | -0.507946 | -1.920607 | -0.133627 | -0.813682 | -0.403030 | -0.042749 | -0.896270 |
| 0.4097335 | 0.0450483 | -0.124801 | 0.0424517 | -0.172490 | -0.064493 | 0.0890111 | -0.001387 | -0.116785 |
| -0.148291 | -0.008621 | 0.4113524 | 0.0273028 | -0.066731 | -0.408582 | -0.625400 | -0.091198 | -0.346894 |
| -1.862802 | -0.260531 | -1.224590 | -1.619458 | -0.531541 | -0.558687 | -1.005365 | -0.789407 | -0.840508 |
| -1.677796 | -0.700565 | -2.074411 | -1.397865 | -0.206241 | -0.777474 | -0.378510 | -0.232523 | -1.120034 |
| -1.372343 | 0.2365781 | 0.2812834 | -1.459736 | 0.2213785 | 0.2355058 | -0.319964 | 0.1956128 | 0.1885885 |
| 0.5283365 | -0.604843 | -0.245542 | 0.8393028 | -0.236651 | -0.127328 | -0.358391 | -0.443864 | -0.315555 |
| -1.784496 | -0.159589 | -1.195870 | -1.875220 | -0.518043 | -0.948876 | -0.516441 | -0.619338 | -1.323126 |
| -0.356138 | -0.025404 | -0.062625 | -0.228041 | -0.217682 | -0.300475 | -0.075129 | -0.229364 | -0.279011 |
| -0.701248 | 0.0480484 | -0.230123 | -0.896796 | -0.229029 | -0.361699 | -0.233812 | -0.226681 | -0.522806 |
| -2.979098 | -0.285404 | -2.054189 | -3.672273 | -0.431118 | -0.990871 | -2.406550 | -0.322596 | -1.207686 |
| 2.1785916 | 1.6167475 | 3.2965112 | 2.1903006 | -0.911379 | 2.4130890 | 2.8202390 | -0.525444 | 3.5478801 |
| 0.9622045 | 0.3973455 | 0.2210721 | 0.9735288 | -0.091435 | -0.321763 | -0.356795 | 0.1438056 | -0.174582 |
| 2.0615070 | 0.2073458 | 1.8115621 | 1.9385908 | -0.098904 | 1.9306002 | 1.5676259 | 0.2976724 | 2.5119502 |
| -1.016249 | -1.416608 | -1.353902 | -1.149771 | -1.137381 | -2.636061 | -2.268165 | -1.370299 | -2.590196 |
| -2.502216 | -2.152866 | -2.408496 | -2.146996 | 0.0559485 | -1.119366 | -0.001326 | -0.530006 | -0.824996 |
| -1.463183 | -1.836526 | -1.792253 | -1.373426 | -0.351726 | -0.930951 | -0.380436 | -0.572065 | -0.277268 |
| -0.461607 | -1.736336 | -1.133140 | -2.376084 | -0.138139 | -0.207685 | -0.790793 | -0.327784 | -1.539089 |
| 0.6453948 | -0.167241 | 0.5326563 | 0.6672489 | -0.264028 | 0.1193649 | 0.1458736 | -0.125213 | 0.2368586 |
| -0.339081 | -0.478080 | -0.609062 | -0.364670 | -0.447929 | -0.662625 | -0.230864 | -0.520341 | -0.392362 |
| 0.3896794 | 0.1785407 | 0.5371251 | 0.1073299 | -0.318621 | 0.6344335 | 1.2649783 | -0.084082 | 0.3401033 |
| -2.146481 | -0.265494 | -1.671253 | -2.391415 | -0.303811 | -0.515187 | -1.032570 | -0.220195 | -1.368031 |
| -1.947863 | -0.204903 | -1.197849 | -2.153785 | -0.608398 | -1.102913 | -1.454397 | -0.681023 | -1.924992 |
| 0.9314693 | -0.045233 | 0.4686405 | 0.6537462 | -0.093459 | 0.4606584 | 0.8195567 | -0.007011 | 0.3373355 |
| -0.021778 | 0.2531824 | 0.2463198 | -0.033954 | 0.1849070 | -0.068410 | -0.713806 | 0.2105474 | -0.044078 |
| -1.839027 | -0.489816 | -1.080528 | -1.961588 | -1.130732 | -1.108837 | -1.283387 | -1.209314 | -1.665611 |
| -1.961802 | -0.396079 | -1.064928 | -2.282577 | 0.4715189 | -0.411061 | -0.876640 | 0.4304105 | -0.916016 |
| -0.059080 | -0.031449 | -0.617276 | 0.0267696 | -0.316740 | -0.223476 | -0.123386 | -0.165082 | -0.542399 |
| -4.328263 | -0.315195 | -0.824328 | -4.229306 | 0.7975899 | 0.5857988 | -0.008123 | 0.7960202 | -0.054104 |
| -0.395069 | 0.7606181 | 0.2487544 | -0.568152 | 0.5897886 | 0.8240777 | 0.4904675 | 0.7438802 | 0.5208571 |
| -1.876506 | -0.860652 | -1.147062 | -2.144804 | -0.211416 | -0.336251 | -1.452279 | -0.308081 | 0.2325201 |
| -2.801204 | -0.560157 | -1.281532 | -3.371563 | 0.0334043 | -0.350869 | -3.489701 | 0.0397590 | -0.787080 |
| -3.223559 | 0.2058019 | -0.627821 | -2.857385 | -0.086165 | -0.149311 | -0.169617 | -0.079475 | -0.141515 |
| -1.234244 | -0.982385 | -2.147772 | -1.158404 | -1.026707 | -0.989522 | -0.665293 | -1.384911 | -1.664648 |
| -1.548370 | -0.546281 | -1.045978 | -2.076985 | 0.0565169 | -0.418971 | -1.975311 | -0.041634 | -0.496600 |
| 0.0003964 | 2.1975683 | 2.2942086 | -0.218339 | 0.1022770 | 2.5487275 | 0.4352928 | 0.5298147 | 2.9180456 |
| -0.437837 | -0.523473 | 0.3301633 | -0.287310 | -0.191507 | 0.1316127 | -0.784084 | 0.0378489 | 0.4239233 |
| -0.643056 | -1.426363 | -2.469963 | -1.332600 | 0.1003378 | -0.869901 | 0.4225530 | -0.405698 | -0.685028 |
| -0.126503 | -0.445503 | -0.462782 | -0.349043 | 0.2005262 | 0.5395414 | 0.7375651 | 0.1920171 | 0.6205274 |
| -2.989835 | -0.081836 | -0.729140 | -3.186268 | -0.340008 | -0.928901 | -1.262641 | -0.461356 | -1.060929 |
| -1.183560 | -0.335848 | -0.623816 | -1.240961 | 0.0549321 | -0.160817 | -0.551714 | 0.0229538 | -0.178868 |
| -0.928178 | -0.101651 | -0.380116 | -0.918219 | 0.0520264 | 0.2072573 | 0.1376890 | 0.0782958 | 0.2653120 |
| 1.0726925 | 0.0747425 | 0.6755654 | 0.9976289 | -0.122640 | 0.1483173 | 0.8866144 | -0.151230 | 0.4937959 |
| 2.3388389 | -0.215893 | 0.4244550 | 2.3127281 | -0.652314 | -0.583349 | 2.5369900 | -0.456071 | -0.118664 |
| -0.500364 | -0.056162 | 0.0589919 | -0.051927 | 0.2748831 | -0.299172 | 0.1143991 | -0.038245 | 0.2913373 |

| | | | | | | | | |
|---|---|---|---|---|---|---|---|---|
| 0.5292467 | 0.2158173 | 0.8169842 | 0.5112796 | -0.151962 | 0.2465042 | 0.1529997 | -0.188876 | 0.3887682 |
| -2.570308 | -0.475423 | -0.796168 | -2.790200 | -0.678205 | -0.732921 | -1.559915 | -0.589940 | -0.750170 |
| 0.1656847 | -0.202584 | 0.4620346 | 0.3711188 | -0.303265 | -0.077204 | 0.1409801 | -0.190836 | -0.022408 |
| 0.1061081 | -0.159706 | 1.4398017 | -0.076763 | -0.253200 | 1.6486922 | 2.6623972 | -0.592700 | 1.2729434 |
| 0.0288244 | -0.315740 | -0.215628 | -0.030053 | -0.642906 | -0.702352 | -0.163464 | -0.583492 | -0.546250 |
| -0.187353 | -0.217981 | -0.163508 | 0.3399920 | -0.285319 | -0.401390 | -0.249926 | -0.250265 | -0.328401 |
| -1.128824 | -0.890001 | -1.828420 | -1.116000 | -0.633142 | -1.059072 | -1.861302 | -0.787313 | -1.494206 |
| 1.9947198 | 0.6481117 | 1.6883804 | 1.6628954 | -0.608043 | 1.9809432 | 1.6749350 | 0.0093446 | 2.8292510 |
| -0.091272 | 1.3462687 | 1.0293562 | -0.719595 | -0.750668 | 1.9446755 | 0.4782241 | 0.7383201 | 1.5138697 |
| 0.4744220 | -0.556858 | -0.173105 | -0.014547 | -1.174021 | -0.361385 | -0.162609 | -0.904132 | -0.573974 |
| -1.830599 | -1.108263 | -1.425232 | -1.828236 | -0.133895 | -0.985203 | -1.549435 | -0.471033 | -1.026257 |
| 2.6447986 | 0.9260571 | 2.5258052 | 2.5952124 | -0.037436 | 2.1773021 | 1.4693301 | 0.4689474 | 2.6817563 |
| 1.4737240 | 0.7566744 | 1.6959349 | 1.3078121 | -0.288339 | 1.7029019 | 1.3036762 | 0.2085005 | 1.8370886 |
| -1.812590 | -0.404879 | -0.958840 | -1.733452 | -0.276358 | -0.650323 | -0.889069 | -0.241842 | -0.764841 |
| -0.660672 | -0.728451 | -0.910025 | -0.800798 | -0.401315 | -1.150269 | -1.315209 | -0.649603 | -1.586085 |
| -0.725092 | -0.368307 | -0.800418 | -0.607842 | -0.126232 | -0.738022 | -0.723139 | -0.284814 | -0.875347 |
| -1.692451 | -0.056362 | -0.992979 | -1.777140 | -0.468568 | -0.438505 | -1.351814 | -0.490537 | -1.320239 |
| -0.407988 | -0.446767 | -0.729574 | -0.381333 | -0.297661 | -0.451565 | -0.491274 | -0.255149 | -0.869660 |
| -0.674367 | -0.682320 | -1.091373 | -0.440359 | -0.208657 | -0.311067 | -0.355808 | -0.294994 | -0.445259 |
| -0.529195 | -0.311852 | -0.645362 | -0.773181 | 0.2442881 | -0.219587 | -0.657202 | 0.1429994 | -0.083144 |
| 0.7499517 | 0.2157919 | -0.481988 | 0.3633534 | -0.266798 | 0.1576828 | -0.469214 | -0.254133 | -0.251301 |
| -2.700266 | -1.072232 | -1.977845 | -2.362354 | -0.398668 | -0.997171 | -1.863756 | -0.494496 | -1.031555 |
| 1.1065961 | 0.4385831 | 0.4571567 | 1.0028035 | 0.1284054 | -0.133842 | 0.2522788 | 0.0224072 | 0.0480411 |
| 0.9294407 | 0.5199481 | 0.9067838 | 0.6750352 | 0.4740322 | 0.8836521 | 0.8002895 | 0.5206243 | 0.7366340 |
| -1.448075 | -0.335809 | -0.754315 | -1.471660 | -0.508251 | -0.344663 | -1.808584 | -0.392949 | -0.793778 |
| -0.156896 | 0.3403321 | -0.125407 | -0.150227 | -0.296261 | -0.434638 | -0.541898 | -0.137620 | -0.605063 |
| -1.960815 | -1.927296 | -2.063590 | -1.889737 | 0.0876591 | -1.688093 | -1.913589 | -0.149203 | -1.909399 |
| 3.0058232 | 0.9902423 | 1.9931009 | 3.0065211 | 0.1837414 | 2.0418784 | 3.8539611 | 0.4825031 | 2.4737765 |
| 1.4705055 | 0.5740500 | 1.4555339 | 1.4473852 | 0.3972340 | 1.1968252 | 2.1527345 | 0.6376958 | 1.5152198 |
| -1.161042 | -0.037206 | -0.231182 | -1.003339 | -0.279399 | -0.237683 | -0.895888 | -0.207535 | -0.436156 |
| -0.309893 | -0.742302 | -0.778236 | -0.226189 | 0.0625840 | -0.141555 | 0.1576081 | 0.3290288 | 0.2046476 |
| -0.166895 | -0.034515 | -0.248249 | -0.073595 | 0.1321534 | -0.061738 | -0.012417 | 0.1303347 | -0.222314 |
| -1.423863 | -1.084380 | -1.556835 | -1.369429 | -0.123285 | -1.086594 | -1.113780 | -0.289713 | -1.603578 |
| -1.626508 | -0.102414 | -1.383154 | -1.551016 | -0.549036 | -0.622550 | -0.658626 | -0.506394 | -0.957802 |
| -0.016717 | -0.285743 | -0.159513 | 0.2246732 | -0.000035 | 0.1817033 | 0.4099814 | -0.023740 | 0.2184193 |
| -1.373868 | 0.0583467 | -0.605799 | -1.598593 | 0.2119325 | 0.1574115 | -0.604231 | 0.2545089 | -0.183504 |
| -2.601471 | -0.309098 | -1.205910 | -2.602004 | 0.1353756 | -0.303888 | -1.964424 | -0.044116 | -0.829761 |
| -2.480890 | 0.2676341 | -0.886981 | -2.860645 | -0.200069 | -0.316613 | -1.015346 | -0.204419 | -0.890879 |
| -1.497714 | -0.215413 | -0.688909 | -1.906824 | -0.227714 | -0.441673 | -0.704775 | -0.302276 | -0.858784 |
| -1.586580 | -0.876251 | -1.644295 | -1.583619 | -0.400856 | -1.440793 | -1.800100 | -0.663063 | -1.261768 |
| -1.134659 | -0.491983 | -0.734690 | -1.441701 | -0.722998 | -1.005549 | -1.279911 | -0.830204 | -1.119009 |
| -1.732996 | -2.057405 | -2.383261 | -1.216217 | -0.047186 | -1.635785 | -1.245873 | -0.131961 | -1.890808 |
| 2.9667767 | 0.7568908 | 2.8423285 | 3.4899275 | 1.2389986 | 1.4549382 | 0.3834503 | 1.6323627 | 2.1002670 |
| -0.469669 | -0.009782 | -0.159701 | -0.615279 | -0.080215 | 0.0663241 | -0.408567 | -0.067868 | 0.1413561 |
| -0.598472 | -0.187409 | -0.159289 | -0.611132 | 0.0232576 | -0.622004 | -1.056156 | -0.147965 | -0.476095 |
| -2.000760 | -1.358279 | -1.675530 | -1.528470 | -0.716151 | -1.033677 | -0.847111 | -0.714508 | -1.149076 |
| 7.1378604 | 0.8468324 | 5.6185983 | 7.8089886 | -0.047968 | 5.9025332 | 4.5410288 | 0.8311873 | 7.2128659 |
| 2.3444794 | -0.273269 | 1.8664704 | 2.3805281 | -0.173831 | 1.4673305 | 1.0402341 | -0.035192 | 2.0480417 |
| -1.389710 | 0.3674666 | 0.2270856 | -0.652987 | 0.2918424 | 0.3042614 | -0.322319 | -0.015401 | 0.5706516 |
| -0.092471 | -0.173246 | -0.069389 | -0.250898 | -0.270581 | 0.1553754 | 0.4411046 | -0.232472 | 0.0624410 |

| | | | | | | | | |
|---|---|---|---|---|---|---|---|---|
| -1.965525 | 0.5250285 | 0.4019226 | -2.119145 | 0.0436783 | -1.364501 | -3.254700 | -0.156765 | -1.513840 |
| 0.4724415 | -0.236611 | 0.0322962 | 0.5285249 | -0.131230 | -0.034284 | 0.1268556 | -0.066060 | 0.0451017 |
| 0.3516604 | 0.1338986 | 0.0696165 | -0.076094 | -0.316066 | -0.256785 | 0.1342127 | -0.413404 | -0.750267 |
| -0.055210 | 0.0356734 | -0.014425 | 0.1460118 | -0.046783 | 0.0737101 | -0.588677 | -0.118314 | -0.102379 |
| -1.556207 | -1.366456 | -2.084335 | -1.546581 | -0.062333 | -1.199526 | -1.323873 | -0.242999 | -1.650718 |
| -1.304457 | -0.444620 | -1.717300 | -1.359691 | -0.793479 | -0.976758 | -1.068352 | -0.762418 | -0.851592 |
| 0.2306415 | 0.1538535 | -0.093939 | 0.2375722 | -0.478546 | 0.1020850 | -0.698446 | -0.663271 | -0.066999 |
| 0.0128074 | -0.405918 | -0.161508 | -0.092955 | 0.1075988 | -0.247413 | -0.205395 | 0.2120457 | -0.316349 |
| -0.163694 | -0.348746 | -0.298698 | -0.277302 | -0.034844 | 0.1227796 | -0.381042 | -0.178354 | 0.1443088 |
| 1.4107599 | 0.2690623 | 1.1143259 | 1.4475615 | -0.231851 | -0.048649 | -0.553017 | -0.038512 | 0.1986337 |
| -1.069003 | -0.087098 | -0.483013 | -0.754290 | -0.388118 | -0.453323 | -0.236018 | -0.318497 | -0.498164 |
| -0.672312 | -0.324894 | -1.737751 | -0.829480 | -0.153403 | -0.361484 | -1.264564 | -0.487426 | -0.602961 |
| -4.780214 | -0.143673 | -1.885991 | -5.316289 | -0.017073 | -0.255404 | -2.724614 | -0.013902 | -0.677882 |
| -1.937041 | 0.1992690 | -1.118595 | -2.543875 | -0.109505 | -0.367481 | -1.936141 | -0.109773 | -0.598879 |
| -0.252265 | -0.018901 | -0.061094 | -0.441251 | -0.126726 | -0.252449 | -0.276464 | -0.103729 | -0.586674 |
| -0.457502 | -0.063074 | 0.0101688 | -0.150516 | -0.207140 | -0.080469 | -0.011305 | 0.1324326 | 0.0954250 |
| -0.638513 | -0.013666 | -0.367576 | -0.615879 | -0.885381 | -1.221692 | -1.103406 | -1.186024 | -1.199872 |
| -0.105419 | -0.330734 | -0.158577 | 0.0277378 | -0.135506 | -0.127684 | -0.315532 | -0.251990 | -0.007302 |
| -0.946385 | -0.309981 | -0.402863 | -0.967262 | -0.520880 | -0.447556 | -0.258557 | -0.475618 | -0.174161 |
| 1.7402880 | 0.3039568 | 0.9043164 | 1.7493222 | -0.035960 | 0.5790105 | 1.4882571 | -0.190686 | 0.9922832 |
| -0.038430 | -0.411539 | -0.607589 | -0.073029 | -0.236874 | -0.309938 | 0.0534841 | -0.359153 | -0.582173 |
| -0.239631 | 0.1980956 | -0.223725 | -0.317512 | -0.290555 | -0.533240 | 0.3205107 | -0.297782 | -0.414044 |
| 0.1022419 | -0.245043 | -0.163877 | -0.101295 | -0.003345 | -0.216439 | -0.042115 | -0.102495 | -0.003097 |
| -1.444909 | -0.444269 | -1.006949 | -1.192039 | 0.0156901 | -0.711192 | -1.360647 | -0.210210 | -0.858696 |
| -2.430009 | -0.623256 | -1.577443 | -2.668284 | -0.011777 | -1.390680 | -1.749405 | -0.765445 | -1.402242 |
| -0.937165 | -0.172267 | -0.728044 | -1.057512 | -0.147173 | -0.028120 | -0.350994 | -0.088322 | -0.539362 |
| 0.7311794 | -0.137196 | -0.236913 | 0.6999020 | 0.1628463 | -0.160400 | 0.1841864 | 0.0738900 | -0.209627 |
| 0.3148900 | -0.178288 | 0.4884144 | 0.6031904 | -0.101759 | -0.202158 | 0.0269607 | -0.322524 | -0.057968 |
| -1.385247 | -0.184783 | -1.373515 | -1.364267 | -0.228977 | -1.286222 | -1.297105 | -0.311711 | -2.116587 |
| -1.680593 | -0.742419 | -1.107576 | -2.368399 | -0.335239 | -0.588792 | -2.119586 | -0.430223 | -0.511814 |
| -1.946345 | -0.831913 | -2.478278 | -1.635623 | -0.293245 | -1.225555 | -1.813452 | -0.222618 | -1.255735 |
| -0.551007 | -0.441840 | -0.591431 | -0.647195 | -0.200024 | -0.298585 | -0.061587 | -0.228375 | -0.685919 |
| -1.295928 | -1.104759 | -1.452728 | -0.963736 | -0.764899 | -2.171500 | -0.795993 | -0.938866 | -1.645761 |
| -0.113138 | 0.2328956 | 0.0386560 | -0.111558 | 0.4988414 | 0.3794511 | 0.0734297 | 0.5254342 | 0.4793654 |
| -2.146938 | -0.451994 | -0.673148 | -2.508898 | -0.132818 | -0.325812 | -0.997919 | -0.221836 | -0.266862 |
| -0.478284 | -0.100506 | -1.033424 | -0.851082 | -0.419911 | -0.799796 | -0.542035 | -0.700919 | -1.178456 |
| -0.631074 | -0.312675 | -1.031457 | -0.464679 | -0.018697 | 0.0750415 | -0.030917 | -0.092874 | -0.070537 |
| -0.768193 | -0.268517 | -0.725027 | -0.762543 | -0.202251 | -0.155363 | -0.724000 | -0.060027 | -0.116744 |
| -0.797414 | -0.615331 | -0.700694 | -0.727327 | -0.148571 | -0.289418 | -0.685603 | -0.407855 | -0.357713 |
| -0.295227 | -0.613516 | -1.247936 | -0.494255 | -0.053251 | -0.768514 | -1.330284 | -0.263830 | -1.174127 |
| 0.7544284 | -0.250729 | -0.006850 | 0.7467348 | -0.049520 | -0.401306 | 0.2512968 | -0.649966 | -0.045327 |
| -1.407201 | -0.010930 | -0.147956 | -1.804337 | -0.112886 | -0.235871 | -1.316062 | -0.132306 | -0.211761 |
| -0.500691 | 0.0209087 | -0.603032 | -0.498261 | 0.1859484 | 0.2058528 | -0.447009 | 0.3099014 | 0.0366467 |
| -0.991381 | 0.2375939 | 0.0772972 | -1.068258 | 0.2937552 | -0.090919 | -0.567507 | 0.3111882 | -0.218054 |
| -2.253127 | 0.3754431 | 0.2402452 | -2.730434 | -0.282557 | -0.476758 | -0.317325 | -0.547566 | -0.488548 |
| -0.178424 | 0.0207105 | 0.1412452 | -0.295492 | -0.478734 | -0.023371 | -0.362396 | -0.445200 | -0.080943 |
| -0.995823 | -0.882495 | -0.402273 | -1.231446 | -0.218807 | -0.665250 | -0.896486 | -0.216157 | -1.047360 |
| 0.6130874 | 0.4098068 | 1.1614012 | 0.6806093 | -0.445224 | 0.3007340 | 0.3250856 | -0.225291 | 0.4301702 |
| -2.946102 | -0.159568 | -2.192227 | -2.786402 | -0.764108 | -0.497663 | -1.023216 | -0.762398 | -0.564784 |
| -1.292050 | -1.055333 | -1.019742 | -1.563808 | -1.099893 | -0.525965 | -1.534117 | -0.670270 | -0.931462 |

| | | | | | | | | |
|---|---|---|---|---|---|---|---|---|
| -0.0984931 | -0.0114817 | -0.1009794 | -0.0498163 | 0.0506702 | 0.0298213 | -0.0865331 | -0.0390327 | 0.0270359 |
| -2.0421452 | -1.4464145 | -2.0722109 | -2.1948278 | -0.7191291 | -1.2759800 | -1.2357093 | -0.8377769 | -1.3755771 |
| -0.2532138 | -0.5714920 | -0.4361704 | 0.0944903 | -0.4635974 | -0.8870401 | -0.4945613 | -0.7275680 | -0.6458075 |
| 1.0691269 | 0.8765084 | 1.4977504 | 0.9566438 | 0.0056185 | 1.2236418 | -0.3927645 | -0.2088329 | 1.9944455 |
| -1.3410854 | -0.0302205 | -0.7537153 | -1.1848987 | -0.0512463 | -0.3498619 | -0.9440439 | -0.2043845 | -0.8821010 |
| 0.1527719 | 0.2006111 | 0.8528940 | -0.1886880 | 0.1662676 | 0.6204732 | -0.8120483 | 0.5257100 | 0.8022476 |
| -0.5761091 | -0.5906916 | -0.6330950 | -0.6452803 | -0.2029624 | -0.2095607 | -0.2128118 | -0.2720799 | -0.2832451 |
| -1.4862085 | -1.1026908 | -1.3342664 | -1.3262308 | -0.5714344 | -0.8109333 | 0.1023913 | -0.7200260 | -1.1474763 |
| -2.2779059 | -0.6281122 | -1.3677698 | -1.8615556 | -0.1479276 | -1.3005784 | -2.2283922 | -0.4145976 | -1.6034631 |
| 0.1694269 | -0.5167575 | -0.3624657 | 0.0088811 | -0.6563529 | -0.0105653 | 0.1197019 | -0.6353012 | -0.4987160 |
| 0.5291251 | 0.0218592 | -0.0349982 | 0.4356184 | -0.4196268 | -0.3500869 | -0.3765543 | -0.4497529 | -0.2066965 |
| -1.4758320 | -0.4853885 | -1.0700350 | -1.6613906 | -0.7094401 | -0.7888686 | -0.5852421 | -0.6177081 | -0.7482861 |
| -1.1624127 | -0.1837439 | -1.6145698 | -0.8431625 | 0.1833398 | -1.0243085 | 0.3311108 | 0.2930787 | -0.3357292 |
| -0.2370000 | -0.0383648 | -0.3151781 | 0.0615280 | -0.4176762 | -0.8993529 | -1.3254748 | -0.5254900 | -1.2567520 |
| -2.8802026 | -0.8835104 | -1.0991410 | -2.4970568 | -1.1552533 | -1.0056983 | -0.5764119 | -1.2413623 | -1.0686493 |
| -2.1508320 | -0.1029457 | -0.5240541 | -1.5704488 | 0.4981783 | -0.3299770 | -2.4161732 | 0.5313520 | -0.7108632 |
| -0.3522480 | -0.7556358 | -0.9836118 | -0.3265161 | -0.2476465 | -0.5891180 | -0.8138514 | -0.2847858 | -0.2820017 |
| -1.0288489 | -0.6820826 | -1.5454052 | -1.8572027 | -0.5297528 | -1.2454510 | -0.2169502 | -0.9750072 | -1.8067444 |
| -0.7256973 | -0.4590802 | -0.9807099 | -0.9404816 | -0.7239946 | -0.9174432 | -0.2365894 | -1.0168192 | -0.8467221 |
| -1.4708500 | -0.3406725 | -1.2700035 | -2.1406925 | -0.4253852 | -0.3455318 | -0.4503174 | -0.5200428 | -0.8416990 |
| 0.4205619 | -0.1936642 | 0.0991666 | 0.3697432 | -0.4129186 | 0.2284289 | 0.2193799 | -0.2936517 | 1.2631157 |
| -0.1857852 | 0.0188480 | 0.4459485 | -0.0765972 | 0.0920673 | 0.3652932 | 0.2884808 | 0.2617701 | 0.5878848 |
| -2.1267545 | -0.3117785 | -1.3900206 | -1.9978571 | -0.3927949 | -1.2049565 | -1.6503462 | -0.5512549 | -1.9218675 |
| -0.4826967 | -0.2433041 | -0.4471864 | -0.5986185 | -0.6564086 | -0.6764622 | -0.3124077 | -0.5566534 | -0.6099568 |
| -1.8728913 | -0.5429880 | -1.0726273 | -1.7508427 | -0.2522123 | -0.8594429 | -0.8955843 | -0.3790586 | -0.9241640 |
| -0.7253172 | -0.2783127 | -0.3820297 | -1.0582520 | -0.8888933 | -0.9763684 | -0.2783720 | -0.8931502 | -1.3641911 |
| -1.5866690 | -0.4800228 | -0.8073861 | -1.8407875 | -0.3827247 | -0.6026175 | -0.4141644 | -0.4107025 | -0.6283285 |
| -1.3597741 | -0.9102150 | -0.9678142 | -1.4246229 | -0.3500587 | -1.0534876 | -0.8283732 | -0.7712005 | -1.2824515 |
| -1.5277759 | -0.1763350 | -1.0346400 | -1.7741372 | -0.4513580 | -0.0962160 | -0.0696957 | -0.8579549 | -0.5966022 |
| 1.7440359 | 0.4147304 | 1.0167056 | 1.7585840 | -0.1576297 | 0.9619715 | 0.9867916 | -0.0139521 | 1.0100941 |
| 2.3629351 | 0.4204260 | 1.3636538 | 2.4744990 | -0.4029579 | 0.9271003 | 2.2895553 | -0.1631723 | 1.9354416 |
| -0.6606686 | -0.1650336 | -0.4359853 | -0.8685337 | -1.2508715 | -1.4275000 | -1.3415381 | -1.4538100 | -1.5182547 |
| 0.3248337 | -0.2451579 | -0.2380892 | 0.2374035 | 0.1075809 | 0.1449382 | -0.0738700 | 0.0012581 | 0.1777600 |
| 0.7154982 | 0.1579150 | 0.3755002 | 0.5416654 | 0.2747094 | 0.5591654 | 1.0450797 | 0.2849964 | 0.6327872 |
| 0.5352107 | 0.0215911 | 0.5293408 | 0.5487264 | 0.3301844 | 0.6595191 | 0.5506660 | 0.4794889 | 0.6886419 |
| -0.1474053 | -0.1228266 | -0.5002618 | -0.6455491 | -0.4590308 | -0.1916600 | -0.4785242 | -0.3581968 | -0.6033354 |
| 1.5809441 | 0.8573101 | 1.5252078 | 0.9904251 | 0.1717076 | 0.4264666 | 0.0560310 | 0.5449487 | 0.7294688 |
| 0.0677242 | 0.0768414 | 0.7379119 | 0.5264098 | -0.4522211 | -0.0782003 | -0.5072572 | -0.3895871 | 0.3149944 |
| -0.6145669 | 0.1171907 | 0.1830727 | -0.6797311 | -0.5417285 | -0.1361533 | -0.2419624 | -0.6508975 | 0.1099679 |
| -1.1910793 | -0.1354391 | -0.2319481 | -1.4519471 | 0.1117801 | -0.2590491 | -0.6101734 | 0.1320177 | -0.3026254 |
| -1.1615371 | -1.2011915 | -1.8580047 | -0.8326548 | -0.1742495 | -1.0648204 | -0.4351127 | -0.4222240 | -0.7289303 |
| 1.0214731 | -0.1252447 | 0.3757087 | 0.9078600 | -0.6227857 | 0.0815917 | 0.1511204 | -0.4595429 | -0.1387750 |
| 0.5476722 | 0.5466684 | 0.5824699 | 0.7356996 | -0.1646686 | 0.5095437 | 0.4530357 | -0.0526775 | 0.8494612 |
| 0.8768955 | 0.2592542 | 0.1960862 | 0.9235038 | -0.1128854 | 0.7154784 | 0.6300804 | 0.0034333 | 0.5044430 |
| -1.2162747 | -0.4366782 | -1.1821639 | -0.9952096 | -0.0798531 | -0.3758363 | -0.6132817 | 0.0139454 | -0.5098075 |
| 0.3458510 | -0.2184420 | 0.0180207 | 0.2024427 | -0.0299524 | -0.1042343 | -0.0011778 | -0.2562544 | -0.0019856 |
| -1.9072002 | -0.7268273 | -1.3072940 | -2.1177050 | -0.3129092 | -0.7152616 | -0.6829520 | -0.2820030 | -0.9359262 |
| -1.0442604 | -1.0344454 | -1.2549567 | -0.7633853 | 0.0213249 | -0.8390971 | -1.4757679 | -0.2402769 | -0.9871912 |
| -2.0554134 | -0.2788032 | -0.8636418 | -1.9417862 | 0.0278522 | -0.2197740 | -0.5341337 | 0.0126902 | -0.6779064 |
| -0.4536648 | -0.1244427 | -0.2590440 | -0.3279381 | -0.0986164 | -0.0529446 | 0.0109634 | -0.1813672 | -0.1380257 |

| | | | | | | | | |
|---|---|---|---|---|---|---|---|---|
| 0.4263742 | 0.4074635 | 0.3279368 | 0.5080284 | 0.1208672 | 0.3594471 | 0.2596344 | 0.1723373 | 0.2061454 |
| -0.430581 | 0.2123881 | 0.0195108 | -0.943043 | -0.919730 | -0.019012 | -0.483750 | -0.593743 | -0.013286 |
| -1.493331 | -0.598905 | -0.736697 | -2.156528 | -0.739861 | -0.381799 | -1.611672 | -0.841243 | -0.528848 |
| -3.039766 | -1.149827 | -1.937892 | -2.916457 | 0.0288091 | -0.806592 | -1.125887 | -0.252922 | -0.939354 |
| -1.644573 | -0.341975 | -1.454600 | -1.696941 | -0.585223 | -0.717364 | -0.632543 | -0.526868 | -1.405995 |
| -0.906986 | -0.029150 | -0.070662 | -0.966809 | -0.096109 | 0.1976595 | -0.158121 | -0.156582 | -0.145391 |
| 1.5301848 | 0.0203101 | 1.0158782 | 1.2924290 | 0.0848509 | 0.4474778 | 1.0567919 | 0.2429767 | 0.3827534 |
| -0.048902 | -0.151397 | -0.503529 | -0.228138 | -0.121319 | -0.400716 | -0.038790 | 0.1553101 | -0.380256 |
| -0.521591 | 0.1531329 | -0.525228 | -0.674985 | 0.0151515 | -0.519949 | -1.482492 | 0.2522295 | -0.572062 |
| 1.3114141 | -0.567000 | -0.166305 | 0.8829141 | -0.255516 | -0.487064 | 0.1266426 | -0.327533 | 0.0179833 |
| -1.132691 | -0.579517 | -1.033107 | -1.236141 | 0.0167548 | -0.324693 | -0.111912 | -0.299373 | -0.587152 |
| -0.854051 | -0.836945 | -0.863399 | -1.207323 | -0.921730 | -1.013130 | -0.674547 | -0.657754 | -0.603037 |
| -1.981454 | -0.847341 | -1.188628 | -1.695065 | -0.661957 | -0.416182 | -0.568072 | -0.973325 | -0.336227 |
| -0.466698 | 0.1730409 | -0.148633 | -0.592457 | 0.2309501 | 0.0735595 | 0.1686430 | 0.4212779 | -0.044796 |
| -2.121635 | 0.1002989 | -0.750940 | -2.159759 | -0.277014 | -0.304014 | -1.213774 | -0.274206 | -0.769383 |
| -1.200244 | -0.176731 | -0.620774 | -1.110888 | -0.023551 | -0.280407 | -0.634924 | 0.0816847 | -0.438542 |
| -1.642149 | -0.012075 | -0.408738 | -2.111188 | 0.0220162 | -0.155655 | -0.925349 | -0.069308 | -0.319228 |
| -1.108382 | 0.0187194 | -0.294743 | -0.977165 | -0.519948 | -0.957053 | -0.536280 | -0.502366 | -1.046853 |
| -2.930046 | -0.686625 | -1.729524 | -3.156542 | -0.344473 | -0.694400 | -1.521436 | -0.375757 | -1.264411 |
| 0.4933961 | 0.5130851 | 0.6302899 | 0.2670343 | 0.4968464 | 0.5034235 | -0.029635 | 0.5429045 | 0.4073059 |
| -1.883940 | 0.1273633 | -0.181495 | -2.020748 | -0.603908 | -0.574735 | -0.572444 | -0.824673 | -0.818849 |
| 0.1521669 | -1.227797 | -2.099564 | -1.431367 | -0.217667 | -0.272051 | -0.346321 | -0.310581 | -0.119719 |
| -2.286777 | -0.649146 | -1.464200 | -1.989837 | -0.034631 | -1.420691 | -2.830553 | -0.232462 | -1.972073 |
| 0.2976897 | -0.223979 | -0.343098 | 0.1689000 | -0.184301 | 0.0067261 | 0.2111557 | -0.133552 | -0.136792 |
| -0.394901 | 0.0643690 | -0.484414 | -0.374054 | -0.313740 | 0.0219743 | -0.645831 | -0.337578 | -0.259556 |
| -0.255014 | -0.539846 | -0.464321 | -0.164144 | -0.281301 | -0.685608 | -0.640897 | -0.401525 | -0.549806 |
| -2.605591 | -0.384455 | -0.695601 | -2.607066 | -0.392026 | -0.450658 | -1.429691 | -0.422511 | -0.601713 |
| -1.130112 | -0.499779 | -0.717432 | -1.077796 | -0.529861 | -0.933114 | -1.466453 | -0.611422 | -1.347441 |
| 0.3430838 | 0.3521275 | 0.3023121 | 0.3592141 | -0.205622 | 0.3682502 | 0.1918429 | -0.161202 | -0.012435 |
| 0.6088369 | 0.1702789 | 0.6360959 | 0.4634872 | 0.1884317 | 0.0221195 | 1.1834917 | -0.015586 | 0.2890489 |
| -0.884397 | -0.254581 | -0.504740 | -1.201991 | -0.780413 | -0.489906 | -0.940593 | -0.539255 | -1.135523 |
| -0.595419 | -0.159366 | -0.552656 | -0.444723 | -0.175590 | -0.142555 | -0.295739 | -0.170485 | -0.236658 |
| 1.7611892 | -0.684995 | -0.125409 | 1.6960602 | -0.403270 | -0.432100 | 0.1355347 | -0.455905 | -0.305413 |
| -2.134048 | -0.365993 | -0.933247 | -2.127155 | -0.412367 | -0.300233 | -0.635302 | -0.358771 | -0.819293 |
| -0.040107 | -0.190695 | -0.062892 | -0.028189 | 0.0272612 | -0.161132 | -0.602206 | 0.0627074 | 0.0119125 |
| -0.901694 | -0.231221 | -0.711904 | -1.061690 | -0.347590 | -0.531229 | -0.712153 | -0.188628 | -0.795073 |
| -1.480198 | -0.834664 | -1.399228 | -1.630167 | -0.690005 | -1.833782 | -2.043697 | -0.536713 | -2.003069 |
| -2.230264 | -1.490196 | -1.627565 | -2.815122 | -0.187539 | -0.067421 | -0.166240 | 0.2513208 | 0.110353 |
| 0.1249410 | -0.381742 | 0.1300942 | 0.1093021 | -0.251801 | 0.7752336 | 0.9904910 | -0.224793 | 0.9885357 |
| -0.958929 | -0.331970 | -0.499938 | -1.531549 | -0.357255 | -0.148830 | 0.2164945 | -0.452641 | -0.391739 |
| 0.9581772 | 0.6483944 | 1.2841280 | 0.9812818 | 0.0763839 | 0.3306070 | 0.8808260 | -0.106215 | 0.3523998 |
| -1.562971 | -0.088099 | -0.997665 | -1.415103 | -0.012331 | -0.692406 | -1.215362 | -0.069112 | -1.023083 |
| -0.717499 | -0.363725 | -0.706726 | -0.760982 | -0.310048 | -0.637383 | -0.435500 | -0.460891 | -0.668519 |
| -0.490754 | -0.846506 | -0.460999 | -0.733883 | 0.0437547 | -0.457454 | -0.365113 | -0.063391 | -0.856180 |
| 0.0213635 | 0.1345497 | -0.121432 | 0.0921626 | -0.428917 | -0.388221 | -0.737617 | -0.634895 | -0.437102 |
| -0.698953 | -0.032864 | -0.494387 | -0.941407 | -0.367500 | -0.135226 | -0.619933 | -0.394686 | -0.382734 |
| -0.896329 | -0.019387 | -0.636046 | -1.012559 | -0.230592 | -0.378091 | -0.064635 | 0.0724266 | -0.516710 |
| -1.357096 | -1.112738 | -1.374696 | -1.842072 | -0.961774 | -1.252466 | -1.451914 | -1.526460 | -1.705650 |
| -0.005503 | -0.191672 | -0.166903 | 0.0119150 | -1.016727 | -0.374367 | -0.936355 | -0.666723 | -0.457788 |
| -0.573507 | -0.745806 | -0.777079 | -0.896296 | -0.662998 | -0.909918 | -1.417422 | -0.803262 | -1.028031 |

| | | | | | | | | |
|---|---|---|---|---|---|---|---|---|
| 0.4882981 | -0.2370695 | 0.0677320 | 0.6188459 | -0.6648189 | -0.9291978 | -0.7633359 | -0.2273199 | -0.9223393 |
| 0.4613275 | 0.5042654 | 0.5055063 | 0.2053111 | -0.3752595 | -0.2154843 | -0.0774705 | -0.2792163 | 0.0011952 |
| 0.2079604 | -0.0296396 | -0.2210250 | 0.1660353 | -0.0095932 | 0.0684010 | 0.1812706 | -0.0809413 | 0.1291282 |

| PG_309 | PG_310 | PG_311 | PG_312 | PG_313 | PG_314 | PG_315 | PG_316 | PG_317 |
|---|---|---|---|---|---|---|---|---|
| -1.3132087 | -0.2050323 | -1.0797610 | -2.6487299 | -0.4186644 | -1.9182269 | -2.5668332 | -0.7178269 | -0.8569734 |
| -1.1836859 | -0.5072352 | -0.4466825 | -1.1843532 | -0.5874382 | -0.1751883 | -1.2620393 | -0.7326128 | -0.4728018 |
| -1.7626813 | -0.4697028 | -0.5360986 | -2.6112664 | -0.5289313 | -0.5188478 | -3.2834271 | -0.6360022 | -1.3165070 |
| 0.0602938 | -0.1660877 | -0.4087236 | -0.3425667 | -0.2162702 | -0.6048882 | -0.2861188 | -0.311299 | -0.4229380 |
| -0.5230769 | -0.1484452 | -0.2386082 | -0.4795517 | -0.0576583 | -0.2084387 | -0.0395551 | -0.3861804 | -0.4930416 |
| -1.7458800 | -0.4301174 | -1.1861946 | -2.4267372 | -0.7960575 | -1.5623761 | -2.8063300 | -0.3096555 | -1.0480606 |
| -0.2224794 | -0.4880039 | -0.8142740 | -1.4753347 | -0.5417039 | -1.1653901 | -0.9822720 | -0.2201727 | -0.9990653 |
| -0.5624869 | -0.1455501 | -0.1300551 | -0.7228357 | -0.0538572 | -0.2891517 | -1.3940383 | 0.0422139 | -0.1786270 |
| -0.0785989 | -0.1451498 | -0.3186185 | -0.2608701 | -0.2997493 | -0.4427979 | 0.3112788 | -0.4659515 | -0.1238704 |
| -0.6380454 | -0.3032785 | -0.7586015 | -2.0906048 | -0.5887911 | -1.1636877 | -2.4622656 | -0.2839301 | 0.2102573 |
| -0.1930174 | 0.0986995 | -0.1557598 | -0.0971459 | 0.0580372 | -0.2661069 | -0.0614369 | -0.2698383 | -0.3700235 |
| -0.2712518 | -0.2934150 | -0.5754584 | -0.8584044 | -0.3719168 | -1.2081385 | -0.8601649 | 0.0252673 | -0.2340837 |
| -3.2169428 | -0.3788710 | -0.7540044 | -3.6831812 | -0.6526440 | -1.1204372 | -4.7919168 | -0.6526990 | -0.8823609 |
| 4.0514867 | 0.0599958 | 3.0163776 | 3.3443384 | 0.9270814 | 4.2196638 | 3.3171679 | -0.6675292 | 1.7147743 |
| -0.1572829 | 0.1618989 | -0.7501825 | -0.9194269 | -0.2280205 | -0.8277909 | -0.5109809 | -0.3606483 | -0.0736496 |
| 1.7846100 | -0.4894261 | 1.9879508 | 3.5124868 | 0.1452939 | 3.0640546 | 3.4123000 | -0.3182585 | 1.6269056 |
| -2.6525359 | -0.7175171 | -1.5994927 | -1.9272658 | -0.6840632 | -1.2807344 | -1.7153566 | -0.9129250 | -1.6469456 |
| 0.0326023 | 0.1063496 | 0.1628123 | -0.7033904 | 0.1008098 | 0.2073558 | -0.5291503 | -0.4205789 | -0.8355587 |
| -0.5475950 | -0.6295272 | -0.3971789 | -0.0399614 | -0.8108788 | -0.3798161 | 0.2404659 | -0.7746991 | -0.3931078 |
| -2.6688493 | -1.0987953 | -0.0743561 | -2.1485900 | -0.8326824 | -0.0434141 | -2.6450057 | -0.4465980 | -0.6841434 |
| -0.1596623 | -0.5169150 | -0.0350846 | 0.1282651 | -0.3472169 | 0.0090506 | 0.0982312 | -1.1470686 | -0.6220583 |
| -0.2164929 | -0.6439911 | -1.9147477 | -2.3780757 | -0.8460803 | -1.8490188 | -2.3417610 | -0.1705722 | -0.1688842 |
| 0.9928194 | -0.0579964 | 0.7981214 | 1.5688066 | 0.0945137 | 1.1876742 | 1.5324229 | -0.1457791 | 0.5407278 |
| -1.3109555 | -0.3291859 | -0.6778291 | -1.9998346 | -0.4032172 | -1.6594075 | -2.1478582 | 0.1848030 | -0.6072720 |
| -2.2271644 | -0.0638455 | -0.6844599 | -1.1503701 | -0.3567576 | -1.2222186 | -1.8193849 | -0.2600090 | -0.9458424 |
| 0.7100908 | -0.1153955 | 0.5984375 | 0.8098637 | 0.2033334 | 0.8100571 | 0.8644738 | -0.5085885 | 0.0397401 |
| -0.7459818 | 0.0037383 | -0.2575979 | -0.3100898 | -0.0858295 | -0.2879134 | -0.3701395 | -0.2552256 | -0.2400535 |
| -1.7561243 | -0.3755446 | -0.6373389 | -0.8161797 | -0.3478750 | -0.7174162 | -0.7722065 | -0.9324621 | -1.2693927 |
| -1.3941330 | -0.0457260 | -0.3220638 | -1.4139230 | -0.1018663 | -0.7572506 | -2.2516681 | -0.2084608 | -0.5410673 |
| -0.0827782 | -0.0859803 | -0.1909050 | -0.3387379 | -0.0802561 | -0.7360128 | -0.3649025 | -0.0599799 | -0.3351420 |
| -1.2348294 | 0.1641954 | -0.0651779 | -3.0878772 | 0.1108764 | -0.5809598 | -3.7724735 | 0.4053416 | -0.1415939 |
| 0.0719114 | -0.0957021 | -0.2357715 | -1.1982982 | 0.2205309 | 0.0324882 | -1.3210406 | 0.3933326 | 0.1914250 |
| -0.7977792 | -0.2456576 | -0.2603237 | -1.6963174 | -0.2537482 | -0.1175033 | -0.8418448 | -0.7190306 | -0.1945467 |
| -3.5450640 | -0.1496128 | -0.2956251 | -4.4821763 | -0.2511620 | -0.5946155 | -4.7663192 | -0.7141200 | -1.1815625 |
| -0.0385609 | -0.2626312 | -0.2654090 | -0.2050938 | -0.3282046 | -0.2092546 | -0.2125458 | -0.7284142 | -0.8251507 |
| -0.7159752 | -0.2676392 | 0.1283246 | -0.3175161 | -0.8862253 | 0.4249876 | -0.4262521 | -0.6526449 | -0.4743277 |
| -2.5384518 | -0.0166725 | -0.3165121 | -1.9103951 | -0.3901955 | -0.6043780 | -2.3067293 | -0.4998040 | -0.3425920 |
| 0.2981789 | -0.0590162 | 2.7884994 | 1.2233868 | 1.3027901 | 2.7243459 | 1.2049240 | -0.3083228 | 2.8575844 |
| 0.0986009 | 0.3766547 | 0.6031542 | 0.4102395 | 0.2714778 | 0.9530866 | 0.8605230 | 0.1444749 | -0.0448992 |
| 0.3019204 | -0.4500854 | -0.8679758 | -2.0420525 | -1.4524044 | -0.8828663 | -1.8487395 | 0.0918476 | 0.1898172 |
| 0.8482694 | -0.3544284 | 0.4797622 | 0.5310982 | 0.2551658 | 0.7636840 | 0.2469728 | -0.1213294 | -0.0140992 |
| -1.6604626 | -0.0977431 | -0.8345495 | -2.0068738 | -0.4470052 | -1.0812480 | -1.9985416 | 0.0180248 | -0.4676372 |
| -0.4338243 | -0.6599464 | -0.4581796 | -0.9852388 | -0.5502255 | -0.6268597 | -0.7455533 | -0.5762475 | -0.7864161 |
| -0.1892621 | -0.0396726 | -0.0249079 | -1.0490661 | -0.1044682 | -0.2508604 | -1.3422871 | -0.3711238 | -0.4663701 |
| 0.7514659 | 0.1420721 | 0.7740679 | 1.2838417 | 0.4545209 | 1.2581592 | 1.0770209 | -0.1976457 | 0.1154514 |
| 4.7455848 | 1.6990372 | 1.4298883 | 4.1396512 | 1.7224828 | 0.9400512 | 4.4869540 | -0.7320374 | -0.1724379 |
| -0.0171280 | 0.0257828 | 0.0243789 | 0.1274852 | 0.2813665 | 0.0245170 | -0.1273763 | -0.1469421 | 0.0733297 |

| | | | | | | | | |
|---|---|---|---|---|---|---|---|---|
| 0.2850086 | -0.3961801 | -0.4251369 | 0.3156170 | -0.5718001 | -0.1368013 | 0.5647320 | -0.4607323 | 0.0985317 |
| -1.2093688 | -0.1854263 | -0.3027635 | -0.7077063 | -0.2088196 | -0.1705596 | -0.9750695 | -0.4871191 | -1.1885629 |
| 0.0709561 | -0.0184584 | 0.2154648 | 0.3075958 | 0.0010799 | 0.2351565 | 0.5061671 | -0.4832412 | -0.4140152 |
| 1.7032635 | 0.8159843 | 2.3465552 | 1.9890358 | 1.6236166 | 2.6147057 | 2.5098686 | 0.0934448 | 2.7602348 |
| 0.1051419 | -0.4751535 | -0.3919222 | -0.0943620 | -0.3480556 | -0.3601919 | -0.3190571 | -1.6867187 | -1.6165233 |
| 0.0587708 | -0.3245964 | -0.2436791 | -0.1517701 | -0.1074825 | -0.2079266 | 0.0512598 | -0.2041234 | -0.4129640 |
| -2.2142829 | -0.3591795 | -0.3066229 | -1.4051990 | -0.6352358 | -0.6230409 | -1.3647210 | -0.1235555 | -0.5414760 |
| 2.7016527 | -0.2825317 | 2.2680358 | 2.8764735 | 0.7330997 | 2.3067728 | 3.0218490 | -0.7569404 | 1.4425485 |
| -0.4758615 | 0.1801169 | 3.4008771 | 1.5639074 | 2.4194691 | 3.4835893 | 0.7887239 | 0.2796733 | 1.7152957 |
| -0.8114649 | -0.7224033 | -0.4640238 | 0.4265425 | -0.7495606 | -0.0338492 | 0.1732497 | -0.5094174 | -0.0581082 |
| -1.7005800 | -1.1063820 | -1.3129259 | -1.4553928 | -1.1731204 | -1.8889158 | -1.1883030 | -0.4920351 | -1.1330149 |
| 2.1002303 | -0.2708805 | 2.1662299 | 4.0881988 | 0.7244953 | 3.2025638 | 4.1672230 | 0.2378259 | 3.1366477 |
| 1.6119977 | -0.5132394 | 1.3718419 | 2.4282641 | -0.0006840 | 2.3968707 | 2.5063859 | 0.0295969 | 1.3497114 |
| -0.9630563 | -0.2046241 | -0.4141478 | -0.7961819 | -0.1281115 | -0.5601685 | -0.4066192 | -0.7442622 | -1.3564282 |
| -1.8614204 | -0.8871916 | -1.3761394 | -1.5092737 | -1.1435941 | -1.3304315 | -1.3201777 | -0.6384027 | -1.7633704 |
| -0.4250831 | -0.4559454 | -0.9146497 | -0.9790029 | -0.5700131 | -0.9969552 | -0.8257136 | -0.3359988 | -0.8673766 |
| -2.0187681 | -0.2615922 | -0.7252843 | -1.5343262 | -0.3058688 | -1.6100782 | -1.8852387 | -0.2951420 | -0.9050288 |
| -0.7888926 | -0.0926452 | -0.4321347 | -0.4103164 | -0.1569674 | -0.7411068 | -0.4211241 | -0.5356709 | -0.7076083 |
| -0.4961063 | 0.1477609 | -0.6776419 | -1.1637399 | -0.0570222 | -1.3338170 | -1.3210574 | -0.1362995 | -0.4371535 |
| -0.8723897 | 0.1822134 | -0.2487434 | -0.7767701 | -0.0064122 | -0.4369575 | -0.7298053 | -0.4044507 | -0.3893093 |
| -0.3578327 | 0.1526361 | -0.1802143 | -0.1241764 | -0.0792231 | 0.1535083 | -0.1592327 | -0.8633967 | -0.4667154 |
| -2.1044733 | -0.6537717 | -0.9996434 | -3.0309781 | -0.8162659 | -1.0089586 | -3.6280009 | -0.7446373 | -0.9742601 |
| 0.3536268 | -0.0460704 | -0.3862640 | 0.4709570 | -0.1162739 | -0.1052891 | 0.3333909 | -0.1342719 | 0.0535273 |
| 0.5571301 | -0.0356504 | 0.1112216 | 0.8701595 | 0.2612171 | -0.1046969 | 0.7872740 | 0.0287741 | 0.4518576 |
| -1.5405432 | -0.8337088 | -1.0035781 | -1.5780891 | -0.7167221 | -1.0952461 | -1.9698593 | -0.7056601 | -1.7167416 |
| -0.5550277 | -0.3419138 | -0.5951192 | -0.3399612 | -0.5229850 | -0.8473106 | -0.0011780 | -0.1068967 | -0.4062425 |
| -2.1875917 | -0.5340311 | -1.9050522 | -3.3390929 | -0.6641984 | -2.7570909 | -3.4534152 | -0.1380954 | -1.3458611 |
| 4.3619185 | 0.5721354 | 1.5192125 | 3.6386442 | 0.8548559 | 1.6987808 | 3.7710232 | -0.2132988 | 1.3334834 |
| 2.9982376 | -0.4191230 | 2.0692037 | 2.5610384 | 0.4655172 | 2.6901275 | 2.6056718 | -0.1877487 | 0.7054370 |
| -1.0837099 | -0.4026219 | -0.4337783 | -1.5344639 | -0.4889171 | -0.6999738 | -1.9204945 | -0.4762368 | -0.7651489 |
| 0.2689049 | 0.1713432 | -0.4785018 | -0.6807092 | -0.0782840 | -0.3420296 | -0.5325681 | -0.2590031 | -0.5771450 |
| 0.0594944 | 0.3475199 | 0.0335101 | 0.3019484 | 0.2756937 | -0.2350951 | 0.3456301 | -0.1494325 | -0.3850578 |
| -1.2394965 | -0.4272583 | -1.5998681 | -1.8172426 | -0.8213570 | -2.2484416 | -1.7875310 | -0.7445613 | -1.6186729 |
| -0.8254290 | -0.5887472 | -0.8031381 | -0.9506307 | -0.6203271 | -1.6753670 | -0.8134646 | -0.7220152 | -1.0181493 |
| 0.4348072 | -0.2623730 | 0.7478143 | 1.6280345 | 0.2247274 | 1.2280465 | 1.7576311 | -0.1808695 | 0.0821687 |
| -0.5773755 | 0.3384956 | -0.0203642 | -1.1789278 | 0.2984415 | -0.7878207 | -1.2270920 | 0.6290091 | 0.4760369 |
| -1.9704798 | 0.2475936 | -0.6673044 | -2.6993636 | 0.0660008 | -0.4517812 | -2.9316613 | -0.0567781 | -0.4787644 |
| -1.3159582 | -0.1524733 | -0.9420847 | -1.9344208 | -0.3256260 | -1.4964395 | -2.0197717 | -0.2998520 | -0.8239634 |
| -1.7490440 | -0.3614011 | -0.6116723 | -1.0948818 | -0.2287025 | -0.7508695 | -1.4185376 | -0.0217992 | -0.4659073 |
| -1.8558298 | -0.5492442 | -1.0831503 | -1.8958708 | -0.5715751 | -1.4909156 | -1.9667878 | -0.7334807 | -0.4391736 |
| -1.3903663 | -0.8455766 | -0.6309566 | -0.8488050 | -0.8775270 | -0.7026751 | -0.7080834 | -0.7334954 | -0.8494949 |
| -0.8526688 | -0.4537681 | -1.9063857 | -2.2853798 | -0.6410122 | -3.3579449 | -1.9671185 | -1.0335726 | -1.4331492 |
| -0.4319281 | -0.1563435 | 2.7830322 | 5.5006991 | 1.4717539 | 3.8108062 | 5.2859536 | 0.2285912 | 2.8554477 |
| -0.4860250 | 0.1606502 | 0.0510604 | -0.5391738 | 0.2009997 | 0.0935361 | -0.2021923 | -0.1718724 | -0.2845763 |
| -1.1409141 | -0.0832387 | -0.5307038 | -1.1901992 | -0.0338635 | -0.4459084 | -1.2907806 | -0.2269033 | -0.5169878 |
| -0.8641182 | -0.4509556 | -1.6481278 | -3.0434755 | -0.7658524 | -1.3965583 | -2.3232096 | -1.2264417 | -1.8250957 |
| 7.4313642 | -0.1530860 | 2.2892947 | 7.1884101 | 0.0376776 | 5.1860480 | 7.5598229 | -0.0827060 | 5.1686246 |
| 1.3013768 | -0.1831586 | 0.9802715 | 2.5311154 | -0.0803411 | 1.7712021 | 2.4459131 | 0.1938294 | 1.5753896 |
| 0.1608519 | -0.5578191 | -1.1447476 | -2.8171908 | -1.2056065 | -1.0646449 | -1.6461164 | -0.7915978 | -1.0238040 |
| -0.1429521 | -0.2251173 | 0.0205121 | 0.8722268 | -0.2220294 | 0.0202462 | 0.7384073 | -0.2292483 | -0.5387113 |

| | | | | | | | | |
|---|---|---|---|---|---|---|---|---|
| -2.8129943 | 0.2096568 | 0.0703303 | -2.0105021 | -0.0550361 | -0.1094083 | -1.8436212 | -0.7826382 | -1.7949327 |
| -0.0026164 | -0.1222927 | -0.4672507 | 0.6478721 | -0.1786819 | -0.4024148 | 0.5819036 | -0.0657434 | -0.2147639 |
| -0.1800290 | -0.1735188 | -0.5712018 | 0.4570997 | -0.4530329 | -1.0280578 | 0.6397129 | -0.2646859 | -0.7173199 |
| -0.2656462 | 0.0740089 | -0.0765772 | -0.1851273 | 0.0557225 | -0.1182957 | -0.0045468 | -0.5099150 | -0.5415555 |
| -1.6904999 | -0.3515375 | -1.3721356 | -1.8796035 | -0.5410272 | -1.9924696 | -1.8744401 | -0.6527932 | -1.6575297 |
| -0.9598400 | -0.1214742 | -0.2987187 | -0.6245346 | -0.1481066 | 0.0342549 | -0.5915059 | 0.0812359 | 0.0140917 |
| -0.5511192 | -0.4581183 | 0.0897259 | -0.2108391 | -0.2744997 | -0.2432579 | -0.0063606 | -0.2903965 | -0.3320614 |
| -0.3360718 | -0.2766684 | -0.1293509 | -0.1100876 | -0.0667128 | -0.2267683 | -0.0420531 | -0.0040720 | 0.0142171 |
| -0.2996355 | 0.0348975 | 0.0012694 | -0.1222986 | 0.1131245 | 0.1703333 | -0.1894918 | -0.4627840 | -0.4456193 |
| 0.0782967 | -0.2331289 | 1.0523458 | 1.0807764 | 0.1615012 | 1.2095562 | 1.5337206 | -0.1355351 | 0.2208658 |
| 0.1971185 | -0.1070641 | -0.3035835 | -1.1859956 | -0.1669670 | -0.4375345 | -1.0498216 | -0.4681193 | -0.7854202 |
| -1.9592199 | -0.1170911 | -0.2881230 | -1.3554747 | -0.4072930 | -0.4150089 | -1.7921299 | -0.8524011 | -0.5412818 |
| -3.6529134 | -0.1876703 | -0.5594234 | -3.2761577 | -0.4211275 | -0.7064905 | -4.8510049 | -0.4588740 | -0.8279665 |
| -1.9528152 | -0.2924542 | -0.4770888 | -2.2978204 | -0.4807465 | -0.5858039 | -2.6682553 | -0.5477987 | -0.8611669 |
| -0.6240466 | -0.3862448 | -0.6624325 | -0.8603178 | -0.4492092 | -0.7425550 | -1.0968345 | 0.0969976 | -0.1067946 |
| -0.2164164 | 0.1739646 | -0.1319043 | -0.0010909 | 0.0528087 | -0.1413288 | 0.0190697 | -0.0351232 | -0.1613712 |
| -1.2406259 | -0.5389569 | -0.7323155 | -0.9550963 | -0.4969686 | -0.8151771 | -1.2133705 | -0.6831151 | -0.4657892 |
| 0.6732577 | -0.2686426 | -0.2752739 | 0.0651360 | -0.1379094 | -0.2220240 | 0.6357825 | -0.2051412 | -0.2983174 |
| -0.4632428 | -0.4737013 | -0.9469932 | -0.0452269 | -0.5577279 | -0.8538103 | -0.5204878 | -0.6530230 | -0.8666724 |
| 1.6233789 | 0.3470653 | 0.5212123 | 2.6063949 | 0.0310694 | 1.2289188 | 2.3964316 | 0.2103808 | 0.5829871 |
| -0.3435096 | -0.2050597 | -0.2228731 | -0.5956341 | -0.2959682 | -0.4451591 | -1.0489100 | 0.4028974 | 0.3356669 |
| 0.4271254 | -0.2893409 | -0.5278571 | 0.0895918 | -0.3529368 | -0.7962679 | 0.0881470 | -0.1747047 | -0.5783834 |
| -0.3039960 | -0.0608345 | -0.0893511 | 0.2628392 | -0.2048230 | -0.0207655 | -0.0852188 | -0.7763031 | -0.6124600 |
| -1.1756343 | -0.3374133 | -0.5580496 | -0.9113154 | -0.4216395 | -0.5252369 | -0.9629357 | -0.4022473 | -0.6398267 |
| -0.9108851 | 0.0465005 | -0.7795201 | -1.0258164 | 0.1604036 | -0.9365911 | -0.8368503 | -0.3466275 | -0.2681955 |
| -0.8067173 | 0.2407120 | -0.1390377 | -0.0876762 | 0.0320823 | -0.4452149 | -0.2009933 | 0.0391047 | -0.2423330 |
| 0.4789297 | 0.1575105 | -0.0877363 | 0.7710354 | 0.2412035 | -0.4713544 | 0.7572444 | -0.1531587 | -0.5260981 |
| 0.0278505 | -0.3893665 | -0.1865040 | -0.2713241 | -0.6250796 | 0.0165638 | -0.0360243 | -0.5137403 | -0.1685087 |
| -1.3793330 | -0.3560739 | -0.9990110 | -2.1072682 | -0.5885939 | -1.6300327 | -2.0664653 | -0.0303181 | -0.3318249 |
| -1.4445312 | -0.4887465 | -0.5493267 | -2.9647365 | -0.8386332 | -0.1602952 | -3.0394884 | -0.5107477 | -0.2766661 |
| -1.6515042 | -0.5819882 | -0.9848894 | -2.0348504 | -0.7148950 | -1.4853194 | -1.3498074 | -1.2425282 | -0.9817836 |
| -0.4604715 | -0.3497985 | -0.6372834 | -0.4687917 | -0.4111390 | -1.3278016 | -0.5534206 | -0.3535796 | -0.9371260 |
| -0.9794775 | -0.8156226 | -1.6506234 | -1.2987298 | -1.0634093 | -1.3716052 | -1.4024684 | -0.5540390 | -0.5372826 |
| -0.0436358 | 0.2606621 | 0.2496661 | 0.0956429 | 0.2770347 | -0.0680646 | -0.0099204 | 0.3021185 | 0.1695113 |
| -1.3882119 | 0.1643926 | -0.1153559 | -0.7918001 | 0.1118903 | -0.2376803 | -0.9053185 | -0.3916246 | -0.8320773 |
| -1.1435039 | -0.3056750 | -1.0421747 | -1.0080057 | -0.5656358 | -1.7999163 | -1.2577731 | -0.7053757 | -0.9131411 |
| 0.0869002 | -0.2011172 | -0.5567691 | -0.7100318 | -0.2151346 | -0.7777741 | -0.7014668 | -0.0219737 | -0.3949997 |
| -0.6366112 | -0.1232891 | -0.4551401 | -1.8301926 | -0.2806355 | -0.5189860 | -1.4532266 | -0.3793983 | -0.5242887 |
| -0.5938878 | -0.4722107 | -0.4020154 | -0.6616650 | -0.5774535 | -0.6103079 | -0.6733689 | -0.6681211 | -0.5922069 |
| -1.3522479 | -0.3001209 | -1.0500255 | -1.8536549 | -0.3371410 | -1.1805300 | -2.3233434 | 0.3717870 | -0.1286467 |
| 0.4701581 | -0.0978818 | 0.4664104 | 0.2086696 | 0.4301122 | 0.4379168 | 0.3103060 | -0.4188660 | 0.3615491 |
| -1.2014793 | 0.1294003 | 0.0357266 | -1.5603267 | 0.1258827 | 0.1661766 | -1.8606728 | -0.1060632 | -0.0983228 |
| -0.5035047 | -0.1286619 | -0.0293818 | -1.4700409 | -0.1688988 | -0.4554326 | -1.3483139 | -0.1910965 | -0.3520803 |
| -0.9144014 | -0.0682596 | -0.1741966 | 0.4841444 | -0.3282467 | -0.1926291 | 0.2554111 | -0.3850383 | -0.9861842 |
| 0.1042267 | 0.2665014 | -0.3474094 | -1.4331188 | -0.0512883 | -0.3355807 | -1.6598156 | -0.1356190 | -0.2002983 |
| -0.5780026 | -0.3614287 | 0.2670952 | -0.3033231 | -0.2111085 | 0.5445020 | -0.2638064 | -0.5575156 | 0.2559912 |
| -1.4402870 | -0.1750055 | -0.4684826 | -0.9312322 | -0.2163575 | -0.1570653 | -1.1929545 | -0.7778841 | -0.5517430 |
| 0.5450047 | -0.2100953 | 0.5792496 | 1.1611496 | -0.0835936 | 0.9329901 | 1.5381482 | -0.3530659 | 0.0071798 |
| -1.1162688 | -0.1846667 | -1.0045008 | -1.3772255 | -0.6438390 | -1.1152797 | -1.3028715 | 0.0086929 | -1.0117937 |
| -1.5802495 | -1.0015911 | -0.7987611 | -1.8065484 | -1.0286696 | -0.9501138 | -2.2014703 | -1.1146986 | -0.9829130 |

| | | | | | | | | |
|---|---|---|---|---|---|---|---|---|
| 0.1231446 | 0.1419842 | 0.0423649 | 0.0186223 | 0.0711747 | -0.522668 | 0.2514025 | -0.195607 | -0.074903 |
| -2.022343 | -0.388852 | -0.467677 | -0.698727 | -0.553437 | -0.331006 | -0.659507 | -0.464819 | -1.100734 |
| -0.591229 | -0.401894 | -0.357462 | -0.412923 | -0.408936 | -0.129026 | -0.088719 | -0.448158 | -0.878569 |
| 0.6538293 | 1.2651527 | 2.0438770 | 3.1035510 | 1.4544263 | 2.2148947 | 2.8176496 | -0.344357 | 1.1235288 |
| -1.331054 | -0.220988 | -0.607509 | -1.755638 | -0.352735 | -1.044815 | -1.446465 | -0.514593 | -0.942978 |
| -0.305202 | -0.136256 | 2.9524010 | 2.8464003 | 2.0497207 | 3.4597597 | 2.6538690 | -0.187185 | -0.080649 |
| -0.404519 | -0.204066 | -0.425912 | -0.106903 | -0.310941 | -0.525435 | -0.121164 | -0.411551 | -0.547426 |
| -0.632352 | -0.388790 | -0.276012 | -0.658222 | -0.400242 | -0.358897 | -1.003227 | -0.534994 | -0.873901 |
| -1.624922 | -0.026160 | -1.207476 | -2.741715 | -0.366021 | -1.420540 | -2.609710 | -0.564275 | -0.823207 |
| 0.2083729 | -0.476574 | -0.753105 | -0.772418 | -0.492285 | -0.800444 | -0.584289 | -0.951939 | -0.884365 |
| -0.110842 | -0.364218 | -0.134426 | -0.429251 | -0.132723 | -0.175104 | -0.324778 | -0.276283 | 0.0731516 |
| -0.745772 | -0.615599 | -1.112153 | -0.644108 | -0.791249 | -1.224137 | -0.754313 | -0.588917 | -1.167163 |
| 1.8417528 | -0.316064 | -0.780162 | -1.029189 | -0.376897 | -0.942403 | -1.133428 | -0.911012 | -0.820373 |
| -0.892553 | -0.384232 | -0.467629 | -0.002381 | -0.386117 | -0.572899 | 0.3562234 | -1.017171 | -0.787794 |
| -0.678746 | -0.622943 | -1.080887 | -3.537818 | -0.977692 | -1.672532 | -3.822127 | -0.415695 | -0.421787 |
| -2.306266 | 0.1628592 | 0.0170246 | -1.296541 | 0.0943121 | -0.174197 | -1.282935 | -0.611269 | -1.004253 |
| -0.582600 | -0.261947 | -0.209278 | -0.406502 | -0.135621 | 0.1166012 | -0.612510 | -0.060145 | -0.197788 |
| -1.256050 | -0.441754 | -0.802918 | -0.024864 | -0.767500 | -1.150897 | -0.386110 | -0.230096 | -0.649700 |
| -0.882459 | -0.650520 | -0.787534 | -0.274387 | -0.987426 | -0.991046 | -0.535305 | -0.819023 | -0.462633 |
| -0.928899 | -0.453484 | -1.127380 | -0.667392 | -0.664143 | -1.860150 | -1.064258 | -0.601491 | -0.952192 |
| 1.0603971 | -0.286216 | 0.0343706 | 1.1061354 | -0.045902 | 0.2862094 | 1.2828404 | 0.1786791 | 0.5202705 |
| 0.0154412 | -0.033337 | 0.0453100 | 0.5131598 | -0.155814 | 0.2807322 | 0.4078927 | -0.210715 | 0.1299114 |
| -2.190579 | -0.607089 | -1.001969 | -2.406818 | -0.707385 | -1.495527 | -2.424129 | -0.425091 | -0.999313 |
| -0.800911 | -0.453042 | -0.276588 | -0.422602 | -0.727769 | -0.521203 | -0.440543 | -0.415035 | -0.759693 |
| -1.380169 | 0.0230115 | -0.629751 | -1.056301 | -0.219144 | -1.084609 | -1.400728 | -0.243492 | -0.382997 |
| -0.801463 | -0.777937 | -0.931622 | -0.161780 | -0.829709 | -1.436168 | -0.086565 | -0.329603 | -0.354866 |
| -0.894100 | -0.387625 | -0.682763 | -0.573104 | -0.543950 | -0.810137 | -0.956732 | -0.543896 | -0.674319 |
| -0.761679 | -0.334389 | -0.817582 | -0.722215 | -0.551157 | -1.084837 | -0.669454 | -0.089635 | -0.350352 |
| -0.879121 | -0.203431 | -0.810208 | -0.638257 | -0.218347 | -0.891539 | -0.675357 | -0.434051 | -0.931002 |
| 1.0691069 | -0.156092 | 0.6571036 | 0.8745311 | -0.093792 | 0.7244041 | 1.2897430 | 0.0809218 | 0.4130234 |
| 3.3280019 | -0.084536 | 1.5720621 | 2.7360941 | 0.4977201 | 2.4814526 | 2.9042667 | 0.2946700 | 1.4723645 |
| -0.939477 | -0.652681 | -0.719116 | -0.962026 | -0.898258 | -0.613894 | -1.125785 | -0.650672 | -0.741129 |
| -0.212874 | -0.100031 | -0.101789 | -0.027340 | -0.134939 | -0.125145 | -0.048983 | -0.253067 | -0.322389 |
| 0.9086581 | 0.3417813 | 0.3215020 | 0.9470249 | 0.3338166 | 0.2763139 | 1.1013186 | -0.507542 | -0.220936 |
| 0.0354638 | 0.5533044 | 0.7550483 | 1.3565159 | 0.5586297 | 0.9348468 | 1.3062526 | 0.1045877 | 0.4901659 |
| -0.578716 | -0.435194 | -0.721902 | -0.740589 | -0.384836 | -0.820451 | -0.886472 | -0.348519 | -0.608380 |
| 0.9823103 | 0.0921904 | 0.8458902 | 1.5223893 | 0.2493205 | 0.8688182 | 1.4477069 | 0.2490362 | 0.2550213 |
| 0.2038600 | -0.244783 | 0.3723984 | -0.010584 | 0.0661891 | 0.5445688 | 0.4198463 | -0.487782 | -0.245467 |
| -0.147239 | -0.284706 | -0.029125 | -0.200595 | -0.397673 | -0.091246 | 0.1410858 | -0.808658 | -0.651133 |
| -0.931031 | -0.042484 | -0.184339 | -1.055787 | -0.214912 | -0.248258 | -0.883315 | -0.621314 | -0.486932 |
| -0.587940 | -0.757793 | -0.996079 | -1.790563 | -0.751279 | -1.209407 | -1.282643 | -0.123504 | -0.878834 |
| 0.3834437 | -0.374023 | 0.0551693 | 0.6885712 | -0.293338 | 0.1817262 | 0.9263131 | -0.333567 | -0.077113 |
| 0.7121491 | -0.455082 | 0.7437910 | 1.8887206 | 0.0212746 | 0.8570602 | 2.0179740 | 0.0710891 | 0.2724161 |
| 0.5501320 | -0.239424 | -0.030160 | 0.3445239 | -0.364806 | 0.0660155 | 0.4743429 | -0.551358 | -0.317219 |
| -0.543616 | -0.081359 | -0.643129 | -1.100117 | -0.139499 | -1.160852 | -1.178311 | -0.274949 | -0.790655 |
| 0.0047103 | -0.109371 | -0.222484 | -0.012026 | -0.215824 | -0.324354 | 0.2359287 | -0.371762 | -0.078735 |
| -1.126460 | -0.431820 | -0.911669 | -1.277443 | -0.623216 | -1.297795 | -2.034673 | 0.1368098 | -0.291476 |
| -1.620175 | -0.575944 | -1.096219 | -1.644816 | -0.653151 | -1.376430 | -1.864651 | -0.116570 | -0.798739 |
| -0.980032 | 0.0131593 | -0.475242 | -1.542627 | 0.0025891 | -0.880780 | -1.799309 | -0.048468 | -0.506675 |
| -0.183930 | -0.228167 | -0.211887 | 0.1530506 | -0.189981 | -0.440989 | 0.4055043 | -0.297143 | -0.504361 |

| | | | | | | | | |
|---|---|---|---|---|---|---|---|---|
| 0.3771744 | -0.073818 | -0.507882 | -0.473093 | -0.271605 | -0.944940 | -0.066074 | -0.508110 | -0.426390 |
| -0.240653 | -0.313977 | -0.329553 | -0.054638 | -0.508311 | -0.289110 | -0.517218 | -0.819824 | -0.542841 |
| -1.335551 | -0.569334 | -0.060748 | -1.999577 | -0.576244 | 0.1909512 | -2.097779 | -0.916228 | -0.465418 |
| -1.896580 | -0.776060 | -1.168642 | -3.490178 | -0.799876 | -1.515795 | -2.974844 | -1.179499 | -1.000276 |
| -1.251822 | -0.442896 | -0.488824 | -0.858884 | -0.605620 | -1.034422 | -0.943387 | -0.592546 | -0.967559 |
| -0.427865 | -0.086911 | 0.0957260 | -0.592583 | -0.049870 | 0.0861371 | -0.603004 | -0.536905 | -0.559622 |
| 0.3506067 | -0.137173 | 0.7409400 | 2.1359004 | 0.2784525 | 1.2144450 | 1.5427876 | -0.128913 | 0.6518390 |
| -0.267225 | -0.024126 | -0.041752 | -0.085692 | -0.068282 | -0.147551 | -0.122020 | -0.174128 | -0.621608 |
| -1.480300 | 0.3621532 | -0.044732 | -1.468067 | 0.2536179 | -0.196362 | -1.471110 | -0.494279 | -0.350510 |
| 0.1340738 | -0.327923 | -0.663261 | -0.504466 | -0.114122 | -0.317652 | -0.796438 | -0.031996 | -0.118144 |
| -0.060696 | -0.002469 | 0.0258617 | -0.647064 | -0.405834 | 0.0401341 | -0.858226 | -0.597692 | 0.5684805 |
| -0.795066 | -0.409414 | -0.866055 | -0.913471 | -0.788936 | -0.693232 | -0.934291 | -0.641938 | -0.748588 |
| -0.553905 | -0.100068 | -0.143661 | -0.139064 | 0.0056737 | -0.153670 | -0.113719 | -0.262025 | -0.215910 |
| -0.016982 | -0.154492 | -0.479409 | -0.848713 | -0.301077 | -0.845872 | -0.812366 | 0.1217274 | -0.119012 |
| -1.473614 | -0.225868 | -0.845115 | -2.410151 | -0.294659 | -1.282382 | -2.335994 | -0.320907 | -0.797271 |
| -1.021239 | -0.208059 | -0.627188 | -0.842427 | -0.207065 | -0.667791 | -0.921159 | -0.334698 | -0.424673 |
| -1.302538 | -0.220254 | -0.586010 | -1.655523 | -0.402122 | -1.003709 | -2.029890 | -0.486082 | -0.391474 |
| -0.843036 | 0.0641178 | -1.042813 | -1.274036 | -0.153256 | -1.439217 | -1.085885 | -0.386600 | -0.833222 |
| -2.070278 | -0.545912 | -1.328806 | -1.282305 | -0.657107 | -1.952436 | -1.447910 | -0.161582 | -0.766878 |
| 0.1311203 | 0.2638240 | 0.3511642 | 0.2095527 | 0.2982025 | 0.0733187 | 0.2743432 | -0.091857 | 0.1207717 |
| -1.415045 | -0.642922 | -0.719676 | -1.565492 | -0.721138 | -0.749651 | -1.860252 | -0.124902 | 0.4311708 |
| -0.362030 | -0.285133 | -0.875671 | -1.217183 | -0.561854 | -1.111355 | -1.140888 | -0.254757 | -0.202817 |
| -3.850203 | -0.377044 | -0.757544 | -3.994811 | -0.439951 | -0.999488 | -4.350814 | -0.272446 | -1.117337 |
| 0.2500327 | -0.276637 | -0.611697 | -0.000221 | -0.279712 | -0.811923 | 0.1850844 | -0.376313 | -0.459132 |
| -1.010758 | -0.171704 | -0.030572 | -1.081419 | -0.171883 | -0.517468 | -0.941544 | -0.386270 | -0.231907 |
| -0.449739 | -0.697782 | -0.398059 | 0.2173911 | -0.597391 | -0.106231 | 0.3944169 | -0.387872 | -0.618108 |
| -1.427876 | -0.191742 | -0.304196 | -2.007116 | -0.243644 | -0.818588 | -2.287560 | -0.019295 | -0.297676 |
| -1.654084 | -0.246301 | -0.744257 | -0.781477 | -0.285560 | -0.661424 | -1.088280 | -0.386473 | -0.592342 |
| 0.3838189 | -0.121427 | 0.0717954 | 0.6699665 | 0.0848706 | 0.0493364 | 1.1043295 | -0.397209 | -0.266251 |
| 1.2530399 | 0.0319550 | 0.4179999 | 1.8881146 | -0.009855 | 0.7664448 | 1.6819774 | 0.0573213 | 0.6036130 |
| -1.707996 | -0.942010 | -0.389032 | -0.808742 | -0.774175 | -0.094472 | -1.251329 | -0.718569 | -0.803228 |
| -0.198291 | -0.094914 | -0.358208 | -0.655543 | -0.227498 | -0.442228 | -0.537135 | -0.224143 | -0.543248 |
| 0.2910772 | -0.162739 | 0.3759515 | 2.8886570 | 0.0348655 | 0.7022618 | 2.5473414 | -0.621666 | -0.331133 |
| -0.890635 | -0.113713 | -0.296389 | -1.730986 | -0.189541 | -0.816509 | -1.958791 | -0.377835 | -0.829323 |
| -0.504023 | -0.064562 | 0.0055362 | -0.070593 | -0.081997 | 0.3609534 | -0.249391 | -0.471379 | -0.221657 |
| -0.972781 | -0.005692 | -0.225431 | -1.465449 | -0.111931 | -0.456621 | -1.549525 | -0.837530 | -1.272969 |
| -2.528947 | -0.714214 | -1.447473 | -2.286420 | -0.731612 | -1.641309 | -2.577617 | -0.631456 | -1.347088 |
| 0.0636155 | -0.185895 | -0.470271 | -2.987076 | -0.643674 | -0.538967 | -3.33163 | -0.086057 | -0.488810 |
| 0.7633446 | -0.460608 | 0.4984248 | 1.5073565 | -0.383337 | 0.9584979 | 1.2199996 | -0.326092 | 0.1883623 |
| -0.331226 | -0.226798 | -0.417562 | 0.4083537 | -0.268315 | -0.608720 | -0.107277 | -0.307792 | -0.927434 |
| 0.9015553 | 0.1442679 | 0.1557578 | 0.7535171 | 0.0547577 | -0.008489 | 1.0464244 | -0.346373 | -0.036838 |
| -1.342732 | -0.219836 | -0.495887 | -1.558351 | -0.214007 | -0.523820 | -1.934213 | -0.509025 | -0.861836 |
| -0.621143 | -0.077130 | -0.295252 | -0.314473 | -0.514126 | -0.288672 | -0.465030 | -0.448661 | -0.571758 |
| -0.856908 | 0.0209888 | 0.2334392 | -0.795392 | -0.106810 | -0.080180 | -1.209779 | -0.476700 | -0.648729 |
| -0.637465 | -0.287717 | -0.154812 | 0.1173292 | -0.234718 | 0.0190269 | 0.2181762 | -0.722943 | -0.372530 |
| -0.532290 | -0.099674 | -0.418935 | -0.850333 | -0.242317 | -0.637465 | -0.936210 | -0.918819 | -0.792223 |
| -0.639348 | -0.250874 | -0.287405 | -0.613952 | -0.285327 | -0.473513 | -0.531315 | -0.433857 | -0.687034 |
| -2.258077 | -1.264756 | -1.304956 | -2.552839 | -1.481474 | -1.188550 | -2.821589 | -0.684515 | -0.290166 |
| -0.154487 | -0.168961 | 0.3274800 | -0.065080 | -0.057916 | 0.9832234 | 0.6072821 | -0.539352 | -0.497214 |
| -1.398048 | -0.760072 | -1.079665 | -0.984398 | -0.758041 | -1.389666 | -0.976841 | -0.540506 | -0.949289 |

| | | | | | | | | |
|---|---|---|---|---|---|---|---|---|
| 0.0811822 | -0.1539704 | 0.2657288 | -0.0387433 | 0.1546003 | 0.7833153 | 0.6842885 | -0.3698166 | -0.3566515 |
| 0.5280080 | -0.1647919 | -0.0246095 | -0.3046108 | 0.0699623 | 0.5112308 | -0.1557841 | -0.3964394 | -0.2833664 |
| 0.1423648 | -0.3439541 | -0.1329215 | -0.1440604 | -0.3090386 | -0.4579385 | -0.1466184 | -0.5981124 | -0.6210640 |

| PG_318 | PG_319 | PG_320 | PG_321 | PG_322 | PG_323 | PG_324 | PG_325 | PG_326 |
|---|---|---|---|---|---|---|---|---|
| -0.5189324 | -0.8159312 | -0.9840737 | -0.4748456 | -0.1321537 | -0.8110469 | -2.3691723 | -0.1435265 | -0.8146137 |
| -0.8784209 | -0.7787351 | -0.2638412 | -1.3743949 | -0.5507826 | -0.8307158 | -1.4101950 | -0.8266892 | -0.9200190 |
| -3.4290023 | -0.5481460 | -1.3323457 | -3.1967672 | -0.5220820 | -1.0954941 | -1.8005998 | -0.8032397 | -1.0304113 |
| -0.0820359 | -0.9908164 | -0.5864841 | -0.4927634 | -0.0111032 | -0.1469550 | 0.0989058 | 0.0034866 | 0.1462230 |
| -1.1627536 | -0.9358446 | -0.4626701 | -1.1951496 | 0.0446216 | 0.0113856 | -0.8855691 | 0.0169942 | 0.0113122 |
| -2.1089438 | -0.4246579 | -0.9885590 | -2.6265349 | -0.4164938 | -0.4947503 | -1.8846661 | -0.2647328 | -0.7044659 |
| -0.7655297 | -0.7000988 | -1.2413864 | -0.8191071 | -0.2919452 | -0.9963446 | -0.6022201 | -0.3078153 | -1.1700128 |
| -1.4264201 | -0.2805532 | -0.1275879 | -1.7517397 | -0.6213522 | -0.6510413 | -2.1949449 | -0.5221344 | -0.9299757 |
| -0.3890360 | -0.2071204 | -0.4142815 | -0.6965287 | -0.5589994 | -0.2382437 | -0.3207409 | -0.7524919 | -0.2811761 |
| -0.0040581 | -0.7523216 | -0.0150927 | -0.009469 | 0.1605316 | -0.6255867 | -2.5588688 | -0.0837150 | -0.7466554 |
| -0.7201917 | -0.3279221 | -0.5253600 | -0.8685374 | 0.3502001 | 0.3732235 | 0.0459760 | 0.3132018 | 0.4602789 |
| -0.5384428 | -0.3085625 | -0.3511536 | -0.7730314 | -0.0154693 | -0.0790141 | -0.4043729 | 0.0187757 | -0.1749740 |
| -1.9757080 | -0.601945 | -1.6295857 | -2.6758525 | -0.2047483 | -1.0295407 | -2.9793856 | -0.3762459 | -1.0443892 |
| 2.3337636 | -0.4538198 | 1.9588555 | 2.2916550 | -0.3126484 | 1.8888061 | 3.6299357 | 0.0136826 | 1.8697232 |
| 0.0129385 | -0.6685926 | -0.0382298 | 0.3758862 | -0.2745357 | -0.9267997 | -0.6653139 | 0.0733211 | -0.9970198 |
| 1.9661825 | -0.1826008 | 1.9829485 | 1.6866515 | -0.3500386 | 2.2323649 | 2.8938948 | -0.1043357 | 2.4778255 |
| -1.6858702 | -0.7819311 | -1.5840156 | -0.9660416 | -1.2976037 | -2.0787402 | -1.6008282 | -1.7578820 | -2.0400881 |
| -0.8050220 | -0.7066472 | -1.3353140 | -0.9260632 | -0.8209759 | -2.2641364 | -2.1198862 | -1.254222 | -2.5614559 |
| -0.0409811 | -0.4333689 | -0.5363994 | 0.0711414 | 0.5563503 | -0.3937444 | -1.1298595 | 0.4055737 | -0.2666680 |
| -0.9488126 | -0.8720293 | -0.8820916 | -0.8402170 | -0.1220764 | 1.5088130 | -0.9245223 | -0.4850450 | 1.6563494 |
| 0.3870606 | -1.0654196 | -0.3651722 | 0.2847396 | -0.8743479 | -0.1443743 | -0.2645897 | -0.8788197 | -0.4388035 |
| -0.4083194 | -0.0986502 | -0.2061169 | -0.2782898 | -0.6996885 | -0.9880764 | -1.0322051 | -0.7655166 | -0.7402675 |
| 1.2536602 | 0.0215650 | 0.7142579 | 0.8522103 | 0.1903391 | 1.1817914 | 1.4057162 | -0.2047722 | 1.1972696 |
| -1.8061860 | 0.0199828 | -1.1337464 | -2.0995020 | -0.1459665 | -0.4459938 | -2.2300090 | -0.1831353 | -0.6838752 |
| -1.4061294 | -0.9595009 | -1.2725536 | -1.6636270 | 0.7503986 | 0.3400980 | -0.8039904 | 0.3579134 | 0.2191008 |
| 0.8298182 | -0.5608633 | -0.0247172 | 0.6342221 | -0.0447347 | 1.0737109 | 1.4912038 | 0.1720787 | 1.0750786 |
| -0.0047051 | -1.0661287 | -0.1074620 | -0.2913114 | -0.0831256 | -0.3625300 | -0.4671197 | -0.1819622 | -0.4311477 |
| -1.9862563 | -0.6477929 | -1.7391114 | -2.3406897 | -0.5817753 | -0.5586416 | -1.4672693 | -1.0319199 | -0.3798694 |
| -1.7890353 | -0.2771739 | -0.9106849 | -1.8229619 | -0.2588876 | -0.6138676 | -1.9930007 | -0.2432432 | -0.6494362 |
| -0.5427789 | -0.6687712 | -0.5652523 | -0.7621390 | 0.5306366 | 0.2371905 | -0.2513282 | 0.3700732 | 0.3974364 |
| -1.5827238 | 0.0618743 | -0.2797846 | -1.6419445 | -0.1663150 | -0.6229919 | -3.6380771 | -0.3301824 | -0.6665577 |
| -0.9162475 | -0.1230628 | 0.5052117 | -0.0207125 | 0.9919180 | 0.5591272 | -1.5237030 | 0.6737945 | 0.4515615 |
| 0.5166771 | -1.3977167 | 0.2148903 | 0.4314036 | -1.0260414 | -1.4983763 | -2.0934832 | -1.0801766 | -1.7134568 |
| -2.3287654 | -1.6246367 | -1.7169804 | -2.6892561 | 0.0357074 | -0.7255365 | -3.5744208 | 0.0528374 | -0.9662015 |
| -1.2033920 | -0.8230289 | -0.7836060 | -1.1869643 | -1.8343660 | -2.1226535 | -4.2202845 | -1.8365173 | -2.2009427 |
| -0.9858135 | -0.7659659 | -0.9353862 | -1.2346966 | -0.5214437 | -1.1120983 | -1.3882709 | -1.1529010 | -1.0246483 |
| -0.9204404 | -1.2121275 | -0.6728467 | -1.2305860 | -0.1794026 | -1.1193551 | -1.9786393 | -0.2858948 | -1.1856574 |
| -0.0349386 | 0.2839048 | 3.0373067 | -0.4014155 | -0.2749335 | 2.2039881 | 0.9474274 | -0.0031732 | 1.9475663 |
| 0.2595843 | -0.9900191 | 0.2718673 | 0.4175723 | -0.6462856 | -0.8376433 | -1.4945233 | -0.9522414 | -1.3956409 |
| -0.5819521 | -0.7950605 | 0.1241821 | -0.3777928 | -0.1169229 | -1.2055048 | -1.0521274 | -0.8287819 | -1.3143732 |
| 0.4950365 | -0.4257505 | 0.1372576 | 0.7368376 | -0.0876862 | 0.8110340 | 0.0647959 | 0.1043118 | 0.0077418 |
| -1.6639699 | -0.2356149 | -0.6375358 | -1.3318134 | -0.3976576 | -0.9258163 | -2.6687894 | -0.3991616 | -1.2202939 |
| -1.2186701 | -1.0408652 | -0.8517615 | -1.3113647 | -0.6276083 | -0.8656181 | -1.3572270 | -0.5465029 | -0.6065787 |
| -0.6322207 | -1.3512831 | -0.4015089 | -0.6519688 | 0.4169895 | 0.0496472 | -0.5905311 | 0.3514816 | -0.0002191 |
| 0.5654269 | 0.1750836 | 0.5013355 | 1.0225074 | -0.2316661 | 0.8485463 | 1.6600480 | 0.0183374 | 0.9098726 |
| 1.9031924 | -0.3626514 | -0.1338761 | 1.0809908 | -1.1774666 | -0.3749302 | 3.0451838 | -0.9137690 | -0.9700492 |
| -0.3091114 | -0.0166945 | 0.0216664 | 0.3131614 | -0.3550285 | -0.0768636 | -0.2755390 | -0.0639117 | -0.1458508 |

| | | | | | | | | |
|---|---|---|---|---|---|---|---|---|
| -0.0747943 | -0.5744584 | 0.2385324 | -0.4374101 | -0.0648504 | 0.2817537 | 0.3629360 | -0.1486757 | 0.0777539 |
| -2.1744495 | -0.5086888 | -1.1816319 | -2.6540529 | -1.2683615 | -1.3492875 | -2.7152927 | -1.2272463 | -1.4092652 |
| 0.2401501 | -0.8162565 | -0.3740613 | 0.2928761 | -0.6273942 | 0.0938831 | 0.3249764 | -0.6294458 | 0.0380783 |
| 2.5977828 | -0.0814945 | 3.1895039 | 2.8913893 | 0.6468630 | 3.5718491 | 4.4145738 | 1.5718052 | 3.3465468 |
| -2.1013683 | -1.3090837 | -1.3864603 | -1.7697125 | -0.2378797 | -1.1734521 | -0.6682850 | -0.9880523 | -0.9687735 |
| 0.1124117 | -0.0207164 | -0.2686401 | 0.2833440 | -0.4442164 | -0.3436551 | -0.1413053 | -0.1575637 | -0.0100990 |
| -0.7723305 | -0.2724792 | -0.9523549 | -0.9884279 | -0.2454140 | -0.8670303 | -1.1591972 | -0.8240259 | -0.6499997 |
| 1.6350960 | 0.1040462 | 1.6823557 | 1.6167029 | -0.0297820 | 2.1694276 | 3.1194849 | 0.2802339 | 2.0347820 |
| 0.3100220 | 1.3598966 | 1.5755870 | -0.1822459 | -0.6484920 | 2.4132397 | 0.4160769 | -0.5255006 | 2.5566279 |
| 0.3203656 | -0.6013731 | -0.2065445 | -0.0010035 | -1.0348016 | -0.5067127 | 0.2405129 | -1.7811749 | -0.3282706 |
| -0.7841142 | -0.6349048 | -1.3831260 | -0.9730896 | -1.1236279 | -2.1344680 | -2.6251613 | -1.2613421 | -1.6624616 |
| 3.4251129 | 0.3552706 | 3.3631892 | 3.1990433 | -0.0883827 | 2.4615095 | 2.5082340 | 0.1747609 | 2.4010996 |
| 1.9294577 | -0.3261763 | 1.3089865 | 1.6484750 | 0.6301614 | 2.4956848 | 2.7563070 | 0.5366443 | 2.5902920 |
| -1.7174313 | -0.6978065 | -1.7079595 | -1.9015146 | -0.1968582 | -0.5840652 | -1.2074554 | -0.2298163 | -0.0724597 |
| -1.2241389 | -1.2852004 | -1.5788951 | -1.6728261 | -0.8810732 | -1.8344777 | -1.8345734 | -1.3432048 | -2.1301715 |
| -1.4188206 | -0.7174913 | -1.0671616 | -1.4840587 | -0.3297906 | -0.8176974 | -1.3647954 | -0.4923457 | -0.7990526 |
| -1.9014588 | -0.4211976 | -1.3150477 | -1.9781046 | -0.1314934 | -0.4176157 | -1.5171963 | -0.2529419 | -0.2080547 |
| -0.7067552 | -0.5895397 | -0.7663067 | -1.0565410 | -0.3239320 | -0.4295585 | -0.5367858 | -0.4221233 | -0.4589831 |
| -0.4224000 | -0.6153084 | -0.7385783 | 0.0115662 | -0.0157127 | -1.0355607 | -1.2085338 | -0.3254809 | -1.5487356 |
| -0.0743578 | -2.0452467 | -0.2228110 | -0.5453099 | -0.2889514 | -1.3129840 | -1.4730639 | -0.5348456 | -1.0936072 |
| -0.3783367 | -0.3548907 | -0.3702293 | -0.6587880 | 0.0160618 | 0.3969423 | -0.3771613 | -0.3603691 | 0.1483754 |
| -1.0571057 | -1.0719311 | -1.1058731 | -1.0041542 | -1.1179620 | -1.4538012 | -3.8534213 | -1.1431724 | -1.8802346 |
| 0.5795335 | -0.4506581 | 0.1713680 | 0.6447655 | -0.0122773 | 0.1499244 | 0.8305397 | -0.0274657 | -0.2142176 |
| 0.6478384 | -2.1503184 | 0.4773704 | -0.0696562 | 0.2862570 | 0.5757697 | 0.9206920 | 0.1048615 | 0.3413503 |
| -1.4108937 | -1.0569808 | -1.7016657 | -1.5792339 | -1.6476141 | -2.2590805 | -2.3578489 | -1.4984528 | -2.2006311 |
| -0.4850497 | -0.3133560 | -0.7381721 | -0.4624604 | -0.3859085 | -0.7233952 | -0.4245421 | -0.5721789 | -0.8330478 |
| -1.8255221 | -0.3170727 | -1.6343835 | -1.3114233 | -1.4673956 | -2.7930777 | -3.3227612 | -1.9518453 | -2.7254823 |
| 2.9489109 | 0.1422036 | 1.3004946 | 2.4171315 | 1.2407116 | 2.6072172 | 3.3119693 | 1.3391590 | 2.6321181 |
| 1.8845422 | 0.0009955 | 0.7038978 | 2.2039781 | 0.4956409 | 1.5685159 | 2.9783800 | 0.6578129 | 1.7014874 |
| -1.9801490 | -0.4911321 | -0.9110278 | -2.3187680 | -0.5018424 | -0.7115648 | -1.7858934 | -0.4354887 | -0.7637230 |
| 0.1132026 | -0.5959902 | -0.4442565 | 0.2615280 | -0.1620132 | -0.9577241 | -0.8694006 | -0.3670703 | -0.8022852 |
| -0.4801290 | -1.5150638 | -0.3863924 | -0.6163824 | 0.0617399 | -0.1764841 | -0.3714034 | -0.0029362 | -0.3763063 |
| -1.4336650 | -1.0167469 | -1.7520237 | -1.1959589 | -0.7718201 | -1.3385388 | -1.8261511 | -0.7480230 | -1.4874114 |
| -2.0291421 | -0.8484112 | -1.2191263 | -2.1395371 | -0.3263295 | -0.6997438 | -0.9186031 | -0.3719662 | -0.4774415 |
| 0.7758726 | -0.2375729 | 0.2484078 | 0.6125071 | -0.3660594 | 0.9595695 | 1.4569938 | -0.0586602 | 0.6850152 |
| -1.6315745 | 0.8687383 | 0.2413008 | -1.2055831 | -0.4201879 | -0.6770060 | -2.4074332 | -0.2879503 | -0.7940903 |
| -0.3292206 | -0.0467248 | -0.4163669 | -0.4828937 | -0.6775827 | -1.6008648 | -2.7653437 | -1.5524822 | -1.3227600 |
| -1.8173107 | -0.0144550 | -1.0318586 | -1.7568908 | -0.2167995 | -0.5921207 | -2.2579873 | -0.1462852 | -0.6008776 |
| -1.7480906 | -0.6851856 | -0.9388382 | -2.4344884 | 0.1592188 | 0.3288965 | -1.2486659 | 0.1470569 | -0.1046848 |
| -1.2820349 | -0.5493822 | -0.4844307 | -1.6480299 | -0.5286286 | -1.1664064 | -2.2314493 | -0.5390846 | -1.1802268 |
| -1.5379920 | -0.6964749 | -0.9425416 | -1.4462734 | -0.6340694 | -1.4115589 | -1.1379679 | -1.7980828 | -0.7688513 |
| -0.7343331 | -2.2772222 | -1.7599111 | -1.1288618 | -0.5026608 | -1.2288419 | -3.2405116 | -0.7734772 | -1.8694149 |
| 3.6744196 | 0.9093471 | 3.5420030 | 3.4122137 | -0.3148740 | 2.7282341 | 3.7375274 | 0.6878210 | 2.2689731 |
| -0.2862582 | 0.0033992 | -0.3071283 | -0.4932058 | -0.2082742 | -0.2106996 | -0.6693875 | -0.2134694 | -0.0958377 |
| -0.3094989 | -0.8161167 | -0.2536589 | -0.5484548 | -0.5339775 | -0.7907340 | -1.4670185 | -0.3704241 | -1.0636571 |
| -1.5867874 | -1.3228330 | -1.9853383 | -1.4648886 | -0.5484222 | -1.4770467 | -2.6780878 | -0.7456151 | -1.3444586 |
| 7.9192958 | 0.1644540 | 6.7360700 | 8.3832297 | -0.0555527 | 4.8266635 | 5.4306618 | -0.0328998 | 4.9429810 |
| 2.1765259 | -0.0097696 | 1.8058621 | 1.8908059 | -0.0585413 | 1.5686276 | 2.3294379 | -0.0427203 | 1.6933805 |
| -1.5482041 | -1.0887163 | -0.7850717 | -0.9566353 | -0.3136581 | -1.2962056 | -4.0376964 | -0.6482605 | -1.5333428 |
| -0.9359745 | -0.1953398 | -0.1333921 | -1.6589482 | 0.2113397 | 0.3566408 | 0.2790625 | 0.1024511 | 0.3016618 |

| | | | | | | | | |
|---|---|---|---|---|---|---|---|---|
| -2.2187087 | -1.2188586 | -2.2176385 | -2.2331922 | 0.3662103 | 0.0437519 | -1.4024113 | 0.1178860 | 0.0599428 |
| 0.4019460 | 0.0088952 | -0.1510893 | 0.3284285 | -0.1916137 | -0.0645624 | 0.4661641 | -0.0446696 | -0.1602406 |
| -1.4237754 | -0.6700040 | -0.6763560 | -1.6893269 | 0.0753971 | 0.0655158 | 0.4535753 | 0.0909202 | 0.0709532 |
| -0.5056078 | -0.5456848 | -0.5890518 | -0.2362599 | -0.2193937 | -0.2789464 | -0.3033256 | -0.4886615 | -0.4154875 |
| -1.4628767 | -1.1863797 | -1.6622732 | -1.2326254 | -0.5435295 | -1.1144759 | -1.6490409 | -0.6541889 | -1.3049902 |
| -0.8040148 | 0.1443105 | 0.1323703 | -0.8801844 | -0.9641800 | -1.0630756 | -2.1882130 | -0.9778266 | -0.9537723 |
| 0.0639955 | -0.9046737 | -0.5703169 | 0.084174 | -0.6360242 | -0.4127630 | -0.3644927 | -0.5306714 | -0.0955852 |
| 0.3384996 | -0.0449985 | -0.0587699 | 0.2194555 | -0.1430906 | -0.0198958 | -0.0970105 | 0.1801307 | 0.1830304 |
| -0.5771476 | -0.3702789 | -0.3943316 | -0.1925091 | -0.0827233 | 0.0388873 | -0.2764869 | -0.1288830 | -0.0385510 |
| 0.6402888 | -0.6592247 | 0.2656999 | 0.6837321 | -0.0235146 | 1.0878582 | 1.7616870 | 0.0956490 | 1.0607166 |
| -1.3319485 | -0.8641681 | -0.8882244 | -1.6651381 | 0.1726797 | -0.0769078 | -1.4050209 | 0.0467341 | -0.0626149 |
| -0.5953400 | -1.1193593 | -1.1282881 | -1.1105194 | -0.7235288 | -1.1038635 | -2.0284691 | -1.4064284 | -1.0782859 |
| -3.1629778 | -0.6160921 | -1.3268246 | -2.9847784 | 0.3507319 | -0.6103816 | -3.2759925 | 0.2821710 | -0.7645063 |
| -1.1841400 | -0.8994691 | -1.5205769 | -1.6474027 | -0.3336378 | -1.2554034 | -2.1871107 | -0.7463676 | -1.0103272 |
| -0.4909518 | -0.0617317 | -0.1832114 | -0.8111474 | -0.0799954 | -0.1527830 | -0.2251121 | -0.1481338 | -0.3728397 |
| -0.3496484 | -0.1849977 | -0.1896754 | -0.1022196 | -0.0851153 | 0.1467245 | -0.0130623 | -0.0482982 | -0.1297615 |
| -1.3346535 | -0.9477053 | -0.8367958 | -1.7956729 | -0.9828185 | -1.1770882 | -1.4071898 | -0.9450996 | -1.1279943 |
| -0.1946131 | 0.1355164 | -0.2265802 | -0.1795729 | 0.2084966 | 0.2830814 | 0.4453333 | 0.5392809 | -0.0122589 |
| -0.3085827 | -1.4129117 | -0.9492407 | -0.8521124 | 0.1429132 | -0.3748181 | 0.1668748 | 0.2122603 | -0.6841712 |
| 1.6429740 | -0.3099226 | 0.7855390 | 1.7917754 | -0.4568909 | 0.4153014 | 2.0513829 | -0.4193599 | 0.0157801 |
| -1.5129865 | 0.2124762 | 0.2119609 | -1.8438004 | 0.2808296 | 0.4062504 | -0.8792299 | 0.3218671 | 0.1663222 |
| -1.2432389 | -0.2533388 | -0.7623701 | -2.0512480 | 0.4803641 | 0.6525860 | 0.6999413 | 0.6369761 | 0.2663154 |
| -0.7289716 | -0.4152517 | -0.3028873 | -0.6607727 | -0.1720989 | -0.4291395 | -0.023164 | -0.2474136 | -0.2136583 |
| -1.3839983 | -0.3348263 | -0.3181099 | -1.4481624 | -0.6964098 | -1.1118614 | -1.7956550 | -0.7397964 | -1.2231144 |
| -0.3525355 | -0.2939305 | -0.3683358 | -0.4979650 | -0.9700811 | -1.6666698 | -1.9237741 | -1.3984981 | -1.5631061 |
| -0.3072353 | -0.9993735 | -0.5652083 | -0.5838403 | 0.5570765 | 0.1989359 | 0.0675893 | 0.3015991 | -0.0359161 |
| -0.1822935 | -1.0594808 | -0.5223241 | -0.3382189 | 0.1056329 | -0.0522984 | 0.4948248 | -0.0566093 | -0.0442065 |
| -0.1708126 | -0.3829930 | -0.2360137 | 0.0849189 | -0.4794875 | -0.3946084 | -0.0210498 | -0.1308253 | -0.4952774 |
| -0.7098919 | -0.5507991 | -0.5459721 | -0.7688175 | 0.3535109 | -0.6437694 | -1.5049090 | 0.2530019 | -0.5922528 |
| 0.5535683 | -0.9190356 | -0.0039372 | 0.4896114 | -1.6232524 | -1.6030099 | -2.3900263 | -1.5634594 | -1.4428826 |
| -0.5638191 | -1.2009442 | -1.1807228 | -0.8789636 | -0.7281963 | -2.2411986 | -3.1324070 | -0.9794828 | -1.7382922 |
| -0.7001509 | -0.7157056 | -0.6865267 | -1.3553307 | 0.2756352 | 0.0982507 | -0.4879883 | 0.0943048 | -0.2282984 |
| 0.0744817 | -0.5696372 | -0.0571250 | 0.5260023 | -0.8174128 | -0.9272093 | -1.2472095 | -0.9191629 | -0.8738379 |
| -0.2547818 | -1.1759083 | -0.0675912 | -0.4931537 | 0.5792288 | 0.5084845 | 0.5053587 | 0.6237106 | 0.0559526 |
| -1.7099548 | -1.1992491 | -0.8521089 | -2.0140686 | 0.0778565 | 0.0977866 | -1.2005916 | 0.1916779 | -0.1850128 |
| -0.8442980 | -0.7391681 | -1.0790742 | -1.1614267 | -0.1564770 | -0.2075418 | -0.4862865 | -0.2178465 | -0.3029852 |
| -0.2551868 | -0.2625856 | -0.5496455 | -0.3559315 | -0.6588868 | -0.7491773 | -0.8312248 | -0.6393027 | -0.8027140 |
| 0.0206544 | -0.8130995 | -0.2790960 | -0.2647971 | -0.7932757 | -0.9842547 | -1.8809401 | -0.9538657 | -1.0855868 |
| -0.6205544 | -0.7719164 | -0.5933153 | -0.4971375 | -0.4710083 | -1.2111316 | -0.4710472 | -0.6062581 | -0.6112250 |
| -0.5696358 | 0.3314168 | -0.3278518 | -0.7062582 | -0.1515458 | -0.9480981 | -1.5735461 | -0.4710049 | -0.8471932 |
| 1.2305258 | -1.1426963 | 0.3419531 | 1.2397073 | -0.1333196 | 0.1258534 | -0.2382235 | -0.2452550 | -0.0777414 |
| -0.0496445 | -0.2728913 | -0.1847591 | -0.1389771 | -0.1127908 | -0.1010496 | -1.6185922 | -0.2066303 | -0.5175567 |
| -0.2492754 | -1.0143103 | -0.5708518 | -0.7400098 | -0.4496158 | -0.5772644 | -1.8080845 | -0.3726688 | -1.1988912 |
| -1.2593029 | -0.6325712 | -1.0388289 | -1.9387818 | -0.8462629 | -0.7888682 | -0.7787443 | -0.6808369 | -0.8851284 |
| -0.1193290 | -0.1369154 | -0.1496568 | -0.1971514 | -0.0172349 | 0.1098733 | -0.6646671 | 0.0555597 | -0.1276575 |
| 0.2552917 | -0.4258495 | 0.1863273 | 0.2094499 | 0.1083755 | 0.1875974 | 0.1150724 | 0.1165036 | 0.4519158 |
| -0.8729367 | -0.9351391 | -0.6013048 | -0.6838815 | -0.9488385 | -0.7509595 | -1.1501175 | -1.2832566 | -0.7188871 |
| 0.4318754 | -0.3575179 | 0.3331270 | -0.1179656 | -0.1856598 | 0.4443292 | 0.8007395 | 0.0210387 | 0.5316376 |
| -2.4850975 | -0.9462334 | -1.5979687 | -2.5350332 | -0.0753044 | -1.3580094 | -3.2108872 | -0.2512276 | -1.1634895 |
| -1.4705526 | -1.3801760 | -1.2522757 | -1.5077002 | -0.8627572 | -1.0507106 | -1.7960415 | -0.9502875 | -0.7701743 |

| | | | | | | | | |
|---|---|---|---|---|---|---|---|---|
| 0.5817617 | -1.2547222 | -0.0537934 | 0.5260488 | 0.1760399 | -0.0476991 | -0.1973384 | 0.0458009 | -0.4947950 |
| -2.0022185 | -1.8842936 | -1.2967928 | -2.1194579 | -0.3746701 | -1.1278713 | -1.2636838 | -0.6399236 | -1.0836242 |
| -0.5096363 | -0.7652874 | -0.6324487 | -0.4977539 | -0.3157076 | -0.0796446 | -0.1462870 | -0.1430708 | -0.4260603 |
| -0.0162988 | 0.0470901 | 1.5814479 | 0.8928485 | 0.5397810 | 1.3669956 | 0.8466590 | 0.4866985 | 1.1476693 |
| -1.2514853 | -0.8765054 | -0.5736205 | -1.5143543 | 0.3254343 | -0.4297554 | -1.2167166 | -0.1169765 | -0.6591333 |
| 0.5214005 | -0.3686716 | 0.4004451 | 0.5333268 | 0.4229978 | 2.2661543 | 2.5443595 | 0.9780115 | 2.0181837 |
| -0.6213456 | -1.1018619 | -0.7392816 | -0.7639666 | -0.3393008 | -0.3848048 | -0.2626706 | -0.5291178 | -0.7110055 |
| -1.3730763 | -1.1015059 | -1.2363226 | -1.2528367 | -0.4459994 | -0.1335559 | -0.8916068 | -0.6766296 | -0.3125686 |
| -1.2796535 | -1.2349043 | -0.8589633 | -1.0517375 | -0.9765299 | -1.6388277 | -3.2501893 | -1.3899421 | -1.7589984 |
| -0.3622843 | -0.9978149 | -0.7707691 | -0.6582352 | -0.5599123 | -0.8303794 | -0.7903315 | -0.5933322 | -1.3732554 |
| 0.9865882 | -0.7275738 | 0.3790111 | 1.2251051 | -0.5460360 | -0.2468779 | 0.0678111 | -0.4254297 | -0.4997764 |
| -1.0041169 | -0.6035979 | -0.8133787 | -0.8578614 | -0.2156355 | -0.6333439 | -0.5199767 | -0.6311628 | -0.6387139 |
| -1.0504653 | -0.6863765 | -0.8558832 | -1.3626532 | -0.3838789 | -1.3266701 | -1.6817589 | -0.4565994 | -1.5454092 |
| -0.6157329 | -1.4519159 | -1.1396904 | -0.8225912 | -0.6034130 | -0.6659601 | -0.2944406 | -0.9266782 | -0.5193117 |
| -0.8885281 | -0.2876637 | -0.5000174 | -0.7955534 | -2.6572192 | -3.8187509 | -4.5716817 | -3.7923860 | -3.4778618 |
| -1.8370387 | -0.8786574 | -0.8482673 | -1.5967045 | -0.3337015 | -0.8792444 | -2.7635084 | -0.3661835 | -0.8720458 |
| 0.7955177 | 0.5778309 | -0.0093610 | 1.0141732 | -0.9510442 | -0.6636294 | -0.3911909 | -0.9699124 | -0.3497717 |
| -0.7476928 | -0.9226371 | -1.0292090 | -1.8148924 | -0.4391473 | -0.6568105 | -0.1211597 | -0.3924510 | -0.0937156 |
| -0.5660435 | -0.7815215 | -0.8773142 | -0.5964010 | -0.5242623 | -0.6558447 | -0.2817272 | -0.4981750 | -0.5696527 |
| -1.6034745 | -1.1823160 | -1.1816890 | -1.9426918 | 0.0274692 | -0.3102994 | -0.6569589 | -0.0806771 | -0.3795398 |
| 1.3760883 | 0.6902866 | 0.9269627 | 1.6560776 | -0.3645014 | -0.3387496 | 0.1750194 | -0.2291947 | -0.1537461 |
| 0.1181464 | -1.3845317 | 0.1268289 | -0.4121336 | -0.1440939 | 0.1697094 | 0.2994058 | -0.0271471 | -0.0922043 |
| -1.5871724 | -0.3649878 | -1.2912182 | -2.3908591 | -0.1883775 | -0.9676006 | -2.0867409 | -0.5142857 | -0.9295141 |
| -1.2619461 | -0.5080269 | -0.5487281 | -1.8325080 | -0.0770807 | -0.3739038 | -0.3587062 | -0.2872545 | -0.4208284 |
| -1.2589425 | -0.2994228 | -0.5500727 | -1.4779867 | 0.6305918 | -0.0033552 | -0.8828682 | 0.4207688 | -0.2208665 |
| -0.6594984 | -0.6447254 | -0.3467160 | -1.1269815 | 0.3609581 | 0.1137095 | 0.2623942 | 0.3514934 | 0.1099069 |
| -1.1494036 | -0.4984402 | -0.8057278 | -1.3811983 | -0.4238709 | -0.8826565 | -0.9115286 | -0.6913348 | -0.9375087 |
| -0.8386040 | 0.0054292 | -0.6166464 | -0.9698641 | 0.0961207 | -0.3595787 | -0.0257452 | -0.0690828 | -0.2843232 |
| -0.6092514 | -0.7823814 | -1.2857721 | -1.2538696 | -0.8079614 | -1.1701807 | -1.4780818 | -0.8911663 | -1.2447836 |
| 1.0448599 | -0.2572598 | 0.4262437 | 1.0285910 | -0.4619532 | 0.4075235 | 1.3102480 | -0.3602745 | 0.5930918 |
| 2.7102519 | 0.3824923 | 1.9912480 | 3.0701925 | -0.0984585 | 1.3964629 | 3.2152133 | 0.3211926 | 1.2953874 |
| -0.3553869 | -1.0452381 | -0.8228887 | -0.8570868 | -0.6764783 | -0.9276733 | -0.3170390 | -0.7697631 | -0.7334784 |
| 0.5048055 | -0.7684348 | -0.3749001 | 0.3397091 | -0.5039921 | -0.2124492 | 0.1240670 | -0.5847652 | -0.3529098 |
| 0.0420186 | -1.0862750 | -0.2588613 | -0.0315782 | 0.0107549 | 0.0624035 | 0.3479837 | 0.0385620 | -0.1714348 |
| 1.1924215 | 0.0137396 | 0.3922904 | 1.3160113 | 0.0628628 | 0.6225712 | 0.8338392 | 0.0228073 | 0.6001378 |
| 0.0394817 | -1.4248168 | -0.1488174 | -0.5979522 | -0.5016650 | -0.7835903 | -0.6517142 | -0.5516452 | -0.5252271 |
| 0.5945060 | -0.8948653 | 0.7304913 | 0.9397778 | 0.5442420 | 0.2953947 | 1.6235138 | 0.0173564 | 0.5851557 |
| 0.3546142 | -0.3120495 | -0.3122804 | 0.3355638 | -0.8847584 | -0.2957057 | 0.2845819 | -0.9171664 | -0.4222085 |
| -0.4822153 | -0.8757739 | -0.8409379 | -0.6017639 | -0.6779611 | -0.6690808 | -0.6317515 | -0.9593509 | -0.3428022 |
| -1.1208111 | -1.5932651 | -0.5253182 | -1.6521401 | -0.5306265 | -0.8098643 | -1.2066643 | -0.7720415 | -0.7271529 |
| -1.5199121 | -0.0562799 | -0.7847829 | -1.8773993 | -0.5323848 | -1.2693599 | -1.9993786 | -0.7837760 | -1.2776379 |
| 0.7277513 | -0.1755740 | -0.1310541 | 0.6827506 | -0.4466714 | 0.3001280 | 1.4437377 | -0.5109431 | 0.4070114 |
| 0.4448312 | -1.0165059 | 0.3603996 | 0.4777719 | 0.4496778 | 1.3314178 | 1.4593725 | 0.7736560 | 0.9041069 |
| 0.5646695 | -0.5035317 | -0.4296609 | 0.1091235 | -0.4615270 | 0.2834733 | 0.4703988 | -0.4707155 | 0.2117789 |
| -0.7414840 | -0.2146638 | -1.0962666 | -0.4732294 | -0.4279868 | -0.7044773 | -1.2186839 | -0.4661220 | -0.7441741 |
| -0.2013721 | -0.6568581 | -0.2019477 | -0.3048444 | -0.2594624 | -0.5379029 | 0.1452375 | -0.2919353 | -0.7369176 |
| -1.5215431 | -0.0426675 | -0.5494245 | -1.5014216 | -0.6177866 | -0.9775094 | -1.8655130 | -0.5593948 | -0.9196083 |
| -0.2525692 | -0.3696019 | -1.1204564 | -0.0960324 | -0.8880674 | -1.2229468 | -1.7926279 | -0.8879370 | -1.5536535 |
| -1.8964792 | -0.2891182 | -0.9685366 | -1.5221132 | 0.2940367 | 0.1244578 | -1.3842868 | 0.3027515 | 0.1114688 |
| -0.8656428 | -1.0991547 | -0.7137419 | -1.1784688 | 0.0972116 | 0.2535468 | 0.3826223 | 0.1267510 | -0.0969159 |

| | | | | | | | | |
|---|---|---|---|---|---|---|---|---|
| -0.336747 | -1.504425 | -0.546964 | -0.669082 | -0.172632 | -0.243136 | 0.1762093 | -0.456069 | -0.365913 |
| -1.316366 | -1.041949 | -0.557277 | -1.440261 | -0.174874 | -0.593474 | -0.622459 | -0.354155 | -0.533342 |
| 0.0191793 | -1.542179 | -0.439255 | -0.712678 | -1.033431 | -0.823296 | -2.063416 | -0.903829 | -0.812084 |
| -1.412515 | -1.073127 | -1.164717 | -1.259937 | -0.854282 | -1.389687 | -3.309923 | -1.136821 | -1.464573 |
| -1.415311 | -1.004698 | -1.307495 | -2.255541 | 0.0279225 | -0.343884 | -1.004429 | -0.230310 | -0.389275 |
| -0.326952 | -0.588691 | -0.723110 | -0.209764 | -0.009140 | 0.0734478 | -0.663694 | -0.061938 | 0.1173580 |
| 1.7721522 | 0.0419937 | 0.8697778 | 1.2517408 | 0.9968176 | 2.2843829 | 2.6528090 | 0.8855812 | 2.3915896 |
| -0.534218 | 0.1869829 | -0.541914 | -0.341059 | -0.349753 | -0.509024 | -0.051221 | -0.279088 | -0.161884 |
| -0.785667 | -1.195379 | -0.750297 | -1.032483 | 0.0299112 | -0.587819 | -1.190130 | -0.405386 | -0.035439 |
| -0.283443 | -0.000685 | -0.072237 | -0.470840 | -0.208040 | -0.458332 | -0.377274 | -0.434061 | -0.885337 |
| 0.7429141 | -0.535279 | 0.7616895 | 0.7557737 | -0.678560 | -0.855634 | -0.886234 | -1.141151 | -0.795446 |
| -0.644759 | -0.585152 | -1.034567 | -1.111292 | -1.024134 | -1.209088 | -0.704690 | -1.533717 | -0.973521 |
| -2.947816 | 0.1547163 | -0.243762 | -3.018247 | 0.7941787 | 0.3715758 | -2.145737 | 0.7959800 | 0.2827611 |
| -0.640054 | -0.912751 | -0.313887 | -0.936087 | -0.221089 | -0.251340 | -0.926944 | -0.344324 | -0.740539 |
| -2.604575 | -0.238678 | -0.976320 | -2.793341 | 0.0746698 | -0.155440 | -2.123156 | 0.0860782 | -0.297864 |
| -0.334826 | -0.752768 | -0.701989 | -0.518989 | 0.0404337 | -0.261405 | -1.248613 | -0.132858 | -0.418779 |
| -1.387555 | -1.567356 | -0.542822 | -1.908379 | 0.3371533 | 0.1683969 | -0.784462 | 0.2079709 | -0.098046 |
| -0.677551 | -0.360531 | -0.868221 | -0.507294 | 0.1084527 | 0.0171502 | -1.030844 | 0.0673718 | 0.0077152 |
| -1.476562 | -0.543070 | -1.120094 | -1.622230 | -0.489334 | -1.477112 | -2.360590 | -0.458270 | -1.534271 |
| 0.3660702 | -1.754216 | 0.0238097 | 0.0011420 | 0.5067538 | 0.0977071 | 0.1160715 | 0.1393558 | 0.1647499 |
| -0.512532 | -0.208140 | 0.1477645 | -0.609095 | -0.310327 | -0.423197 | -2.205101 | -0.521710 | -0.504279 |
| -0.624383 | -0.111398 | -0.235043 | -0.559241 | -0.660100 | -1.761775 | -2.383955 | -1.311144 | -1.456371 |
| -3.306704 | -0.110051 | -1.219010 | -2.884684 | 0.1693663 | -0.648569 | -2.867479 | 0.0473420 | -0.529600 |
| 0.0699794 | -0.512570 | -0.587675 | -0.056061 | -0.496551 | -0.491002 | -0.135073 | -0.525469 | -0.584452 |
| -0.548270 | -0.146730 | -0.411602 | -0.873273 | -0.709639 | -0.635405 | -0.823005 | -0.577664 | -0.721992 |
| -0.469894 | -0.271672 | -0.656107 | -0.301986 | -0.865540 | -0.654197 | -0.029921 | -1.037867 | -0.900048 |
| -0.655928 | -0.245616 | -0.508928 | -0.858389 | 0.0310858 | -0.059820 | -1.767100 | 0.0465290 | -0.000798 |
| -1.000754 | -0.775067 | -0.702835 | -1.210941 | -0.623450 | -0.502624 | -0.574992 | -0.573939 | -0.481689 |
| -0.094031 | -0.322876 | -0.314405 | -0.232343 | -0.064681 | 0.4715923 | 0.7375724 | 0.2067667 | 0.4711563 |
| 0.3322630 | -0.724000 | 0.8707523 | 0.2974533 | 0.9384358 | 1.0922850 | 1.3558440 | 0.8916355 | 0.7282221 |
| -0.777382 | -0.315620 | -0.940290 | -1.088730 | -0.435001 | 0.0612343 | -0.563330 | -0.488901 | 0.2244153 |
| -1.029610 | -0.479970 | -0.609182 | -1.532905 | 0.0044695 | -0.330880 | -0.885704 | -0.041152 | -0.506165 |
| 0.8531869 | -0.023539 | -0.007674 | 0.8077248 | -0.358392 | -0.339545 | 2.6469897 | -0.509886 | -0.464033 |
| -1.211542 | -0.517131 | -1.009365 | -1.062291 | -0.161481 | -0.261857 | -1.594104 | -0.344150 | -0.279060 |
| -0.374366 | -0.617469 | -0.193584 | -0.729953 | -0.429764 | -0.639432 | -0.768798 | -0.322167 | -1.183265 |
| -2.494941 | -1.067704 | -1.543629 | -2.337283 | -0.453506 | -0.468537 | -1.332516 | -0.458797 | -0.739733 |
| -1.572560 | -0.792065 | -2.002359 | -2.172972 | -0.934894 | -2.435137 | -2.904135 | -1.326514 | -1.593149 |
| -0.330958 | -0.116733 | -0.246906 | -0.147647 | 0.4492163 | 0.5282400 | -3.837938 | 0.4682915 | 0.6040482 |
| 0.3827354 | -0.455355 | 0.1521407 | -0.375842 | -0.093532 | 1.0593736 | 1.2813801 | 0.0095534 | 0.7590012 |
| -1.840034 | -0.602962 | -0.884917 | -2.207011 | 0.0167726 | -0.121013 | -0.173663 | 0.1010843 | -0.195897 |
| 0.3333750 | -1.448111 | -0.085569 | -0.028653 | 0.4941837 | 0.6591505 | 1.0123485 | 0.4519093 | 0.5666322 |
| -1.142386 | -0.786451 | -1.033567 | -1.066204 | -0.359041 | -1.074551 | -2.052906 | -0.505693 | -1.115906 |
| -0.831388 | -0.609494 | -0.600939 | -1.105151 | -0.530502 | -0.743750 | -0.819540 | -0.859151 | -1.098270 |
| 0.1951966 | -1.401020 | -0.587011 | -0.184592 | 0.3243511 | -0.100260 | -0.195304 | 0.1898389 | -0.179652 |
| 0.0835098 | -0.416428 | -0.326639 | 0.0566056 | -0.335131 | -0.289848 | 0.1321592 | -0.392381 | -0.474164 |
| -1.511056 | -1.812042 | -1.135542 | -1.542694 | -0.454840 | -0.684666 | -1.111204 | -0.675697 | -0.676048 |
| -0.947340 | -0.998554 | -0.781688 | -1.453615 | -0.031953 | -0.589981 | -0.747974 | -0.123307 | -0.569261 |
| -1.572579 | -0.620238 | -0.448626 | -2.044996 | -0.446152 | -0.719852 | -0.954812 | -0.986122 | -0.464431 |
| -0.326948 | -1.294486 | -0.587896 | 0.2851220 | -0.229208 | -0.220827 | 0.0776656 | 0.0867691 | -0.243270 |
| -1.162356 | -0.545135 | -1.146851 | -1.285637 | -0.776497 | -1.354800 | -1.309034 | -1.463153 | -1.089710 |

| | | | | | | | | |
|---|---|---|---|---|---|---|---|---|
| -0.2177075 | -0.8124853 | -0.3849878 | 0.2830377 | -0.2944765 | -0.6981535 | -0.3817825 | -0.5138375 | -0.4684199 |
| -0.3331949 | -0.6372666 | -0.1065038 | -0.5356442 | -0.3976188 | -0.5265997 | 0.0012285 | -0.3768895 | -0.7065133 |
| 0.0962574 | -0.7584439 | -0.7531052 | 0.1590748 | -0.3320230 | -0.1046876 | -0.1634893 | -0.1930226 | -0.6076493 |

| PG_327 | PG_328 | PG_329 | PG_330 | PG_331 | PG_332 | PG_333 | PG_334 | PG_335 |
|---|---|---|---|---|---|---|---|---|
| -1.7303359 | -0.5541680 | -0.4216447 | -1.3711192 | -0.3229227 | -0.3838331 | -0.5349348 | -1.0682448 | -2.4126253 |
| -1.5416977 | -0.3899498 | -0.1996769 | -0.7647230 | -0.3260149 | -0.1178978 | -0.4797594 | -0.8719489 | -0.6610247 |
| -2.0738231 | -0.6380047 | -0.2119122 | 0.0866457 | -0.5007055 | -0.4157139 | -0.4867014 | -0.2989219 | 0.7326270 |
| 0.0712790 | -0.3885138 | -0.5835012 | -0.7862002 | -0.3162963 | -1.0568112 | -0.1111644 | -0.3292119 | 0.3384475 |
| -0.7171486 | 0.1214406 | -0.1053015 | -0.3639979 | 0.1430669 | -0.3511080 | 0.0308616 | -0.0587126 | -1.4520335 |
| -1.3432476 | -0.2525249 | -0.4254199 | -0.7853995 | -0.1677758 | -0.1586472 | -0.2764251 | -0.5634760 | -1.1712534 |
| -0.3470008 | -0.1765286 | -0.6811677 | -0.6342060 | -0.0570528 | -0.8848019 | -0.0126523 | -0.2800150 | -0.4346091 |
| -2.4164157 | -0.6778874 | -0.6651167 | -0.8903525 | -0.6041654 | -0.6814359 | 0.1797236 | -0.1300204 | -2.6620851 |
| -0.1029438 | -0.2712404 | -0.3401955 | -1.3305844 | -0.2324498 | -0.3308697 | -0.3866711 | -0.2412101 | -0.1159034 |
| -2.3122917 | -0.0803729 | -0.6555688 | -0.0524966 | -0.3592694 | -1.3364596 | -0.4921244 | -1.0539593 | -2.2321156 |
| 0.0399416 | -0.1755975 | -0.3110801 | -0.3663968 | -0.3136331 | -0.5351813 | -0.2857411 | -0.3566524 | 0.0610246 |
| -0.5753619 | -0.0579604 | -0.3747342 | -0.4346985 | -0.0040813 | -0.6872626 | -0.1846676 | -0.0993528 | -0.0508106 |
| -3.3387518 | -0.0947959 | -0.1822021 | -0.2322294 | -0.0326434 | -0.0783986 | -0.5844618 | -0.7140333 | -1.8380326 |
| 3.8292883 | -0.0508572 | 2.6661103 | 1.6108708 | 1.3553383 | 3.6398699 | -1.0016711 | 1.8888382 | 4.0050338 |
| -0.2322180 | 0.0162871 | -0.3230587 | -0.6542747 | -0.0872202 | -0.3333535 | -0.0981800 | -0.3657217 | -0.1719918 |
| 2.7441018 | -1.2832025 | 0.0631869 | 0.0924707 | -0.6150502 | 1.0028925 | -0.1570012 | 2.7981473 | 2.5657285 |
| -2.3656990 | -0.9317339 | -1.8890431 | -1.7136092 | -1.0427898 | -2.3316204 | -0.9484499 | -2.5504631 | -1.1950029 |
| -2.3712347 | -0.6844896 | -0.4130352 | -0.5165831 | -0.5482501 | -0.6268328 | -0.3999435 | -0.3217482 | -0.2342334 |
| -1.6158945 | -0.4813247 | 0.0967948 | 0.7982731 | -0.1982553 | 0.2630559 | -0.8025929 | -0.7802901 | 0.0148224 |
| -1.9225140 | -1.2565034 | -1.1896488 | -1.1631761 | -1.2792153 | -1.5669820 | -1.3623897 | -1.6341924 | -1.4791845 |
| 0.0240688 | 0.1146739 | 0.2264157 | -0.2911433 | 0.1144641 | 0.3331348 | -0.3201491 | 0.9122839 | 0.6076777 |
| -0.6682740 | -0.1228130 | -0.3474308 | -0.3719429 | -0.3817625 | -0.3479423 | -0.5303509 | -0.2162425 | -0.3503061 |
| 1.3131188 | -0.1460144 | 0.9947135 | 0.0498249 | 0.0415379 | 0.8599765 | -0.1506825 | 1.1695010 | 1.8461187 |
| -2.2837995 | -0.4944213 | -1.0070861 | -2.0709953 | -0.7472890 | -1.4162465 | -0.2139199 | -0.8085536 | -1.6966707 |
| -1.1454011 | -0.2726788 | -0.7217288 | -0.7452051 | -0.5825786 | -0.8289796 | -0.6369835 | -1.5162939 | -1.7130650 |
| 1.5912275 | -0.1202655 | 0.4683799 | -0.1547844 | 0.0777678 | 0.1922461 | 0.1870493 | 0.4983287 | 1.2545528 |
| -0.4666055 | 0.0984297 | -0.0805141 | -0.0819952 | 0.0207298 | 0.0267721 | 0.2178812 | 0.1332608 | -0.6243447 |
| -1.6402863 | -0.4068153 | -0.7025732 | -0.8078739 | -0.3244263 | -0.5112088 | -0.6044147 | -1.1025176 | -1.4297847 |
| -2.4044988 | -1.6131767 | -0.7319244 | -1.5880031 | -1.5022569 | -1.3553110 | 0.1700860 | -0.6281239 | -1.4605770 |
| -0.2954366 | -0.2919272 | -0.2511521 | -0.5823066 | -0.3399578 | -0.3730656 | 0.1726433 | -0.5818012 | -0.6965700 |
| -3.4082495 | -1.2380661 | -0.2478710 | -0.2376150 | -0.9918989 | -0.4181009 | 0.1300889 | -0.5674461 | -2.0034510 |
| -1.5971490 | -0.0037797 | -0.0250779 | 0.3777211 | -0.4823439 | -0.2772371 | -0.0870568 | -0.0942268 | 0.0376429 |
| -1.5603153 | -0.4934208 | -0.2302481 | -1.5038829 | -0.3054288 | 0.0614690 | -0.0781539 | 0.0468431 | -0.7734619 |
| -3.2822931 | 0.2244329 | 0.0458469 | -0.5512792 | 0.1053472 | 0.0256470 | -0.3097704 | -0.2155759 | -1.6281925 |
| -4.1110152 | -0.0722360 | -0.1091754 | 0.2685390 | -0.0894013 | -0.1565327 | -0.4968043 | -0.3743592 | -0.5026960 |
| -1.4252665 | -0.5384200 | -0.3799711 | -0.4899830 | -0.6952526 | -0.6945234 | -0.5108994 | -0.9962053 | -0.4671948 |
| -2.1284256 | 0.3045296 | 0.2878077 | -0.7389748 | 0.4932824 | 0.4330565 | 0.0845499 | -0.7240030 | -2.1064272 |
| 0.8864779 | 0.2110938 | 2.9420523 | 0.7555124 | 2.7618457 | 3.4886706 | -0.3226962 | 2.6668989 | 0.5002919 |
| -1.3126890 | -0.0186577 | 0.0092912 | -0.5104868 | 0.0414894 | 0.0800760 | -0.1624430 | -0.0825335 | -2.2483408 |
| -1.2415731 | -0.3396976 | -0.1689575 | -1.1569918 | -0.3167239 | -0.7538436 | -0.5349603 | -0.4798923 | -0.1534489 |
| 0.2280321 | 0.3415807 | 0.3862336 | -0.3575624 | 0.0784545 | 0.4327442 | -0.2900554 | -0.1256402 | 0.3243290 |
| -2.7607356 | -0.0765014 | -0.6942836 | -0.8987412 | -0.1955565 | -0.9549884 | -0.4624812 | -1.2334226 | -1.9333336 |
| -1.5198481 | -0.5184752 | -0.6393534 | -1.7077950 | -0.6738623 | -0.5184575 | -0.4577905 | -0.3636737 | -0.6685962 |
| -0.7527408 | -0.0241357 | 0.0533560 | -0.6044386 | 0.1446367 | -0.0476763 | -0.0309759 | -0.1644030 | -0.5500775 |
| 1.5476032 | -0.0088453 | 0.2159377 | 0.2311939 | 0.1134871 | 0.2807347 | -0.1064759 | 0.8600985 | 0.9955143 |
| 3.1399596 | -0.1856849 | -0.2691995 | 0.6463176 | -0.3392267 | -0.4677601 | -0.5751369 | -0.2613578 | -0.2797137 |
| -0.3457883 | -0.3959135 | 0.1903288 | -0.3133524 | -0.3651521 | 0.1582481 | -0.4405775 | -0.0491216 | -0.2875786 |

| | | | | | | | | |
|---|---|---|---|---|---|---|---|---|
| 0.3563301 | -0.5492543 | -0.2969196 | -0.4216888 | -0.4081880 | 0.0212661 | -0.3269994 | -0.1591005 | -0.0883483 |
| -2.5302813 | -0.2424709 | -0.6212845 | -0.4948515 | -0.3171486 | -0.9944463 | -0.3568731 | -0.6691305 | -2.2146812 |
| 0.2889743 | -0.5794283 | -0.2368452 | -1.0983770 | -0.4588600 | -0.3469150 | -0.8957398 | 0.3946457 | 0.3370821 |
| 4.3817415 | -0.5000711 | -0.4952651 | 0.7406152 | -0.2820061 | 0.2103076 | -1.7175626 | 1.6728661 | 1.4350268 |
| -0.2626511 | -0.0542402 | -0.1265188 | -0.2819699 | -0.3467481 | -0.3320939 | -0.2021298 | -0.2371704 | -0.2741655 |
| 0.0977349 | -0.3125696 | -0.1773291 | 1.7872875 | -0.2552668 | -0.2479849 | -0.2559503 | -0.1338322 | -0.0161940 |
| -1.3340944 | -0.1276198 | -0.3742972 | -0.7617246 | 0.0704223 | -0.6756782 | -0.3570487 | -0.5407660 | -1.4082123 |
| 2.7929009 | -0.4946529 | 1.3696690 | 0.6154138 | 0.2851175 | 1.3683505 | -0.4402815 | 1.7284027 | 2.4175781 |
| -0.3627720 | -0.6650243 | 0.5816196 | -1.1314430 | -0.1982091 | 1.2458647 | -0.6062828 | 3.8964413 | 3.0557980 |
| -0.3583989 | -0.8129302 | -0.3915398 | -0.7538520 | -0.5225186 | -0.8080572 | -0.7559558 | 0.0057714 | 0.5775294 |
| -2.4703151 | -0.5292597 | -0.9879155 | -1.0578011 | -0.8094345 | -1.0250327 | -0.8374452 | -1.1143645 | -2.0265914 |
| 2.5410713 | 0.6296646 | 2.8898494 | 2.2225486 | 1.7684387 | 3.6674522 | 0.5331030 | 2.4071028 | 2.2669739 |
| 2.8021267 | 0.1233695 | 1.8781800 | 0.7990643 | 1.1173924 | 2.5403759 | 0.1287220 | 3.1675943 | 2.4200002 |
| -0.9718679 | -1.0074119 | -0.8643545 | -1.8378953 | -1.2141710 | -1.4528177 | -0.4595403 | 0.0219425 | -1.1140481 |
| -1.9426402 | -0.6196514 | -1.0837945 | -1.7464428 | -0.7982065 | -1.2087631 | -1.3258147 | -1.1010413 | -1.5104338 |
| -1.5021192 | -0.8257822 | -0.4510568 | -1.6169278 | -0.3493495 | -0.8376723 | -0.6936593 | -0.4130767 | 0.3368086 |
| -1.6591682 | -0.2812655 | -0.7789489 | -0.8604111 | -0.4079841 | -1.2368790 | -0.1179328 | -0.6894881 | -1.5563461 |
| -0.2815087 | -0.0221949 | -0.5160447 | -0.7200982 | -0.1767145 | -0.6667210 | -0.2932878 | -0.5049752 | -0.4750186 |
| -1.3304667 | 0.0231345 | -0.3279303 | -0.2602522 | -0.1585464 | -0.4655522 | -0.1541334 | -1.0426047 | -1.7109640 |
| -1.0513687 | -0.3032345 | -0.2385077 | -1.4213598 | -0.0319549 | -0.2958126 | 0.1008694 | -0.2013741 | -0.9359704 |
| -0.5617648 | 0.4183620 | 0.1055541 | -0.2442547 | 0.3084397 | -0.2564855 | -0.3235518 | -0.6435810 | 0.3944418 |
| -2.7649756 | -0.4414161 | -0.6398711 | -1.0481367 | -0.4331617 | -0.8217427 | -0.5785837 | -0.6620147 | -1.2433859 |
| 0.5832746 | 0.3428502 | 0.1480816 | -0.5628117 | 0.1443131 | -0.0740869 | -0.2605052 | -0.1127033 | 0.0910672 |
| 0.8398644 | 0.2320550 | 0.4622589 | -0.3327863 | 0.3838692 | 0.4934734 | 0.6951139 | 0.5072647 | 1.3726893 |
| -2.5230659 | -0.3660791 | -1.0121424 | -1.7763078 | -0.5329824 | -1.2482393 | -0.5070385 | -1.3212301 | -1.4896517 |
| -0.1635794 | -0.1114833 | -0.3685875 | -0.1722860 | -0.1571633 | -0.0705755 | -0.0229675 | -0.3398269 | -0.2483314 |
| -3.0941747 | -0.7978622 | 0.2962720 | -0.6626178 | -0.7170723 | -0.4737110 | -0.3265189 | -0.5289785 | -0.6441953 |
| 3.4100910 | 0.3172299 | 2.1626439 | 2.2355658 | 0.4422537 | 2.1529707 | 0.4319765 | 2.9160438 | 4.4876926 |
| 3.1058604 | -0.6694068 | 0.3370508 | 1.9412812 | -0.1747683 | 0.4681033 | -0.1249330 | 0.7576856 | 1.9348383 |
| -1.5133645 | -0.1977464 | -0.4561840 | -0.6147529 | -0.4028042 | -0.8691689 | -0.2229560 | -1.0487732 | -1.7141369 |
| -0.9721877 | -0.1405564 | -0.1147568 | 0.0924292 | 0.2382684 | -0.1680907 | -0.5753246 | -0.6834816 | -0.8568086 |
| -0.4873659 | -0.0867679 | -0.0028797 | -0.3797119 | -0.1898576 | -0.3169910 | 0.1261529 | -0.2307461 | 0.0061360 |
| -1.7542846 | -0.4318819 | -0.8759724 | -0.9253623 | -0.6920967 | -0.8620543 | -0.7337731 | -0.9561818 | -1.2585469 |
| -0.9790703 | -0.3087153 | -0.4401159 | -0.9794605 | -0.2512611 | -0.9332406 | -0.4774357 | -0.7569404 | -1.0049316 |
| 1.3642659 | -0.3056291 | 0.3889162 | 0.1263033 | -0.2802363 | 0.7310171 | -0.2463183 | 1.3567019 | 1.5505906 |
| -2.2583961 | -0.5620869 | -0.2165867 | 0.5945344 | -0.2950146 | 0.3638082 | -0.2236086 | -0.2478401 | -0.3790624 |
| -2.2872324 | -0.5967972 | 0.1637387 | 0.0844090 | -0.4787136 | -0.2648950 | -0.7447584 | -0.9470916 | -1.2031449 |
| -2.4854099 | -0.0919238 | -0.6944624 | -0.7661446 | -0.2944987 | -1.4277364 | -0.3132275 | -1.1479230 | -2.1026299 |
| -1.4563672 | -0.2054103 | -0.5574351 | -1.0104855 | -0.2188969 | -0.4430825 | -0.3230709 | -0.4340689 | -1.1208638 |
| -2.2116580 | -0.2775241 | -0.4947381 | -0.8244202 | -0.3518780 | -0.8479261 | -0.4151528 | -0.9524310 | -1.6536312 |
| -0.9922543 | -0.9544734 | -0.8943490 | -1.6711618 | -0.8185988 | -0.9984077 | -0.4204054 | -0.9965131 | -0.8810691 |
| -2.9422836 | -1.1538908 | -0.8104761 | -0.0360290 | -0.9375256 | -1.0319846 | -1.1216683 | -1.8978226 | -2.8101073 |
| 3.2221674 | -0.8710533 | -0.5843951 | -0.5900251 | -0.8138506 | -0.3182116 | -0.0093915 | 3.0812000 | 1.6648118 |
| -0.4663206 | -0.1049479 | 0.0519669 | -0.1878827 | -0.1890613 | -0.2966041 | -0.1215441 | -0.2366183 | -0.4823111 |
| -1.3103517 | 0.0097789 | -0.0981831 | -0.0282740 | -0.0948483 | -0.0039327 | -0.0895209 | -0.5186039 | -1.2637184 |
| -2.3734068 | -0.6317864 | -0.8318975 | -0.0922521 | -0.8579659 | -0.8957874 | -0.6784390 | -1.9268676 | -1.5112398 |
| 6.1915318 | -0.1469661 | 0.2264118 | 2.1591434 | -0.1701654 | 0.8234984 | -0.0630275 | 6.4896521 | 5.6927044 |
| 2.4168022 | -0.0594245 | 1.6440929 | 1.3674400 | 0.3380275 | 2.2415929 | -0.1338329 | 2.5693146 | 1.7753230 |
| -2.2263763 | -1.1981393 | -1.7145451 | -1.3749247 | -1.6777289 | -2.2456663 | -0.7688965 | -1.4211540 | -3.5309586 |
| 0.1265558 | -0.5979879 | -0.4222842 | -0.3444323 | -0.5909699 | -0.3636189 | -0.1401414 | 0.0324936 | 0.2007123 |

| | | | | | | | | |
|---|---|---|---|---|---|---|---|---|
| -1.3296337 | -0.9905811 | -0.2173748 | -0.9524181 | -0.9559978 | -0.3315965 | -0.7194377 | -3.3440337 | -2.7545929 |
| 0.6005995 | -0.0815897 | 0.0199467 | 0.1956105 | -0.1400699 | -0.1631250 | -0.3917218 | -0.5302593 | 0.1096168 |
| 0.3877786 | -0.2475260 | -0.6872685 | -1.0707566 | -0.6541118 | -1.0863374 | -0.2909330 | -0.6987143 | 0.0866017 |
| -0.0775464 | 0.1500034 | -0.0760360 | -0.1429262 | 0.1773222 | -0.0251406 | -0.1408419 | -0.2533949 | -0.8258892 |
| -1.8519709 | 0.0682135 | -0.2284613 | -0.1896432 | -0.1211733 | -0.4947447 | -0.4497711 | -0.9082849 | -1.7803003 |
| -2.1518814 | -0.0783605 | -0.3037115 | -1.2333738 | 0.1055929 | -0.4601197 | -1.0651030 | -0.2283868 | 0.3900116 |
| -0.2094836 | -0.9074059 | -0.4374398 | -0.9278409 | -0.6274758 | -1.1432205 | 0.5257466 | 0.1224510 | 0.3232929 |
| 0.1823591 | -0.2056979 | -0.1163265 | -0.2355918 | 0.0543600 | -0.1454402 | -0.0270201 | -0.1751414 | 0.0141008 |
| -0.4466191 | -0.0407849 | 0.1994226 | 0.2000337 | -0.1057414 | 0.0258700 | -0.1246301 | -0.6641956 | -0.6492304 |
| 1.8155048 | 0.0148575 | 0.0061497 | -0.7464164 | 0.2926037 | 0.2216497 | 0.2194838 | 0.9568663 | 1.2473737 |
| -1.1779076 | -0.4730496 | -0.5653484 | -0.2628884 | -0.5437223 | -0.1782745 | -0.1801615 | -0.2542368 | -1.6793875 |
| -2.1958804 | -0.0509014 | -0.2417397 | -0.5178570 | -0.0927780 | -0.4136727 | -0.2396793 | -0.2353756 | -0.9583046 |
| -4.0270090 | 0.0991002 | 0.0312813 | -0.3454194 | 0.1354688 | 0.2491112 | -0.2851630 | -1.2695290 | -3.2502856 |
| -2.4365011 | -0.1635757 | -0.6045208 | -0.9371817 | -0.4716223 | -0.5913016 | -0.0225627 | -0.5243742 | -1.4169143 |
| -0.5013658 | -0.2395825 | -0.5047343 | -0.5823264 | -0.1924565 | -0.4084622 | -0.4637131 | -0.6016988 | 0.1342647 |
| 0.0341714 | -0.1889719 | 0.0213683 | -0.0299248 | -0.2564593 | -0.0128294 | -0.0421586 | -0.4594602 | -0.3591811 |
| -1.5459460 | -0.6723833 | -1.0191697 | -1.4330264 | -0.4100364 | -1.0519910 | -0.2172456 | -1.6880703 | -1.4635758 |
| 0.4907524 | -0.1582726 | -0.3849883 | 0.0706939 | -0.2537246 | -0.3135816 | -0.1762222 | -0.1420183 | -0.0722452 |
| -0.4676455 | -0.2063196 | -1.1466965 | -0.8794148 | -0.2485202 | -1.4869239 | -0.3801036 | -0.8088606 | -0.2604405 |
| 1.9456878 | -0.0981064 | -0.2024166 | -0.0699334 | -0.9075023 | 0.1766762 | -0.7646509 | -0.2418471 | 1.0975996 |
| -0.9300560 | -0.1869951 | -0.5029981 | -0.3912645 | -0.1765867 | -0.7463814 | -0.2584288 | -0.4360602 | -0.9057416 |
| 0.6419888 | -0.0087688 | -0.1844010 | -0.2395975 | -0.1842092 | -0.1727108 | -0.6660766 | -0.7030320 | -0.0728874 |
| -0.1665104 | -0.3345862 | -0.2118264 | 0.4468238 | 0.0124309 | -0.0235656 | -0.2756447 | -0.1518951 | -0.0891262 |
| -1.8961845 | 0.0119902 | -0.0581635 | -0.4756880 | -0.2542218 | -0.0467387 | -0.3674279 | -1.0833578 | -2.6429054 |
| -1.8956054 | -0.3329141 | -0.3029615 | -0.1610833 | -0.4557999 | -0.2217338 | -0.5930088 | -0.8154373 | -0.3869634 |
| -0.0292634 | -0.1452486 | -0.4615169 | -0.3127777 | -0.5051551 | -0.9633312 | -0.2016060 | -0.6002609 | -0.2426132 |
| 0.6252413 | 0.1068450 | -0.2770419 | -0.1768361 | -0.0119765 | -0.3895141 | -0.0134915 | 0.0127129 | 0.3452969 |
| 0.2549849 | 0.2955638 | 0.0893952 | -0.4626672 | 0.1897663 | 0.3452217 | -0.1709092 | -0.3010441 | -0.3368444 |
| -1.4981692 | -0.7874210 | -1.0244421 | -1.2258470 | -0.8045434 | -1.3301176 | -0.5333141 | -1.4833248 | -0.3911102 |
| -1.7742463 | -0.5368076 | -0.5206373 | -1.3608641 | -0.5202384 | -0.2338940 | -0.2519069 | -0.4014816 | -1.4701801 |
| -2.4304440 | -1.8854426 | -1.2151809 | -1.1093951 | -1.0379895 | -1.2715198 | -0.7395321 | -2.6679495 | -3.5788741 |
| -0.6233163 | -0.4417892 | -0.8412738 | -1.1670263 | -0.3586622 | -1.1210773 | -0.5391808 | -0.4311177 | -1.0616096 |
| -1.1567528 | -0.5036281 | -0.3958726 | 0.6756657 | -0.8558126 | -0.0794890 | -0.2839466 | -0.1855852 | -0.0344035 |
| 0.4342967 | 0.6393949 | 0.2270877 | -0.6678225 | 0.3974847 | 0.0982691 | 0.2901137 | 0.1759805 | 0.0232682 |
| -1.3835779 | -0.1796660 | -0.4590806 | -1.6316363 | -0.3160982 | -0.8684640 | -0.2795735 | -0.7416331 | -1.4283750 |
| -0.7734061 | -0.2886632 | -0.5136602 | -0.5082945 | -0.3519898 | -0.7795458 | -0.4458912 | -0.6800357 | -0.8341669 |
| -0.9636998 | -0.0406903 | -0.2960447 | -0.5243391 | -0.1772686 | -0.5362001 | -0.2423789 | -0.3464514 | -0.2978052 |
| -1.5996379 | -0.2329344 | -0.2420181 | -0.5128751 | -0.1858045 | -0.2257539 | -0.1693643 | -0.2772255 | -1.0388279 |
| -0.4342234 | -0.5724893 | -0.8746910 | -1.1716992 | -0.6676113 | -0.8126404 | -0.4568419 | -0.3177827 | 0.2864664 |
| -1.5832231 | -0.5966213 | -0.8789127 | -1.3147284 | -0.7251357 | -1.0058594 | -0.4372547 | -0.6675100 | -0.8002415 |
| -0.0674313 | 0.0724763 | 0.2910453 | -0.2539768 | 0.0328713 | 0.1676277 | 0.0993443 | 1.0566351 | -0.7016570 |
| -1.2016414 | -0.0362294 | 0.0995203 | -0.0990363 | 0.1022076 | 0.2348588 | -0.0278222 | 0.3273082 | -0.7595302 |
| -2.0425858 | -0.0774356 | -0.0473838 | -0.7071062 | 0.0078947 | 0.0491977 | -0.1011921 | -0.0393342 | -0.9739454 |
| -0.7416281 | -0.3784976 | -0.4675266 | -1.0664243 | -0.5809370 | -0.2931693 | -1.0309800 | -0.4888023 | -0.8540905 |
| -0.6323186 | -0.2782646 | 0.0315422 | 0.1548804 | -0.1627869 | -0.0409055 | -0.2018132 | -0.2750756 | -0.0204879 |
| 0.0636041 | -0.2520985 | 0.2180437 | -0.2253137 | 0.0170004 | 0.4469501 | -0.1348699 | 0.3241106 | 0.2689102 |
| -1.6052265 | -0.2828809 | -0.3962036 | -0.6680879 | -0.3010864 | 0.0654043 | -0.8833420 | -1.1281438 | -1.2149592 |
| 1.0339712 | -0.2689046 | -0.3829838 | -0.8307875 | -0.3760887 | -0.7128433 | -0.0957694 | -0.0513842 | 0.3569167 |
| -3.0119143 | 0.9044520 | 0.1792619 | 0.5628686 | 0.8709116 | -0.5048280 | -0.2509096 | -0.2774716 | -0.5064119 |
| -2.0491172 | -0.5625988 | -0.4887991 | -1.1951568 | -0.5616644 | -1.6290643 | -0.7433282 | -0.6796392 | -2.0760515 |

| | | | | | | | | |
|---|---|---|---|---|---|---|---|---|
| -0.053905 | 0.3240771 | 0.2556639 | 0.0102740 | 0.2995813 | 0.2541521 | 0.1818156 | 0.2248965 | -0.089507 |
| -1.214351 | -0.225869 | -0.697237 | -1.418329 | -0.548670 | -1.299635 | -0.825397 | -1.418336 | -2.009971 |
| 0.0096383 | -0.310319 | -0.484119 | -0.944777 | -0.054111 | -0.129408 | -0.439543 | -0.061294 | -0.559821 |
| 0.6719449 | -0.578499 | -0.483362 | 0.1752640 | -0.551090 | -0.419789 | -0.167298 | -0.195853 | 0.0415165 |
| -1.244341 | 0.0233282 | 0.1103383 | -0.732880 | 0.0078138 | -0.630857 | -0.717520 | -1.084891 | -1.476070 |
| 2.2324995 | -0.671847 | 0.6259686 | 0.2319305 | -0.197099 | 0.9971983 | 0.8301054 | 2.8907947 | 2.3528001 |
| -0.330299 | -0.151311 | -0.255306 | -0.460973 | -0.260286 | -0.089354 | -0.293675 | -0.577845 | -0.398516 |
| -1.166309 | -0.424486 | -0.686144 | -1.257488 | -0.588033 | -1.322804 | -0.561058 | -0.592734 | -0.618559 |
| -2.921336 | -0.274004 | -0.807570 | -0.804846 | -0.329489 | -1.188497 | -0.773174 | -0.676391 | -1.606683 |
| -0.381331 | -0.310930 | -0.145285 | -0.585642 | 0.0579458 | -0.166572 | -0.931671 | -0.437824 | 0.1544009 |
| 0.3465582 | 0.0458075 | -0.015288 | -0.096267 | 0.2484120 | 0.2612346 | -0.049938 | 0.0105841 | -0.023064 |
| -0.479861 | -0.431737 | -0.746307 | -1.104177 | -0.701726 | -1.468094 | -0.526337 | -0.831947 | -0.682259 |
| -1.788347 | -0.955550 | -1.042406 | -0.424867 | -0.773401 | -0.741544 | -0.662038 | -0.649342 | -0.627032 |
| -0.089812 | -0.617654 | -0.496339 | -1.252224 | -0.694450 | -0.509919 | -0.419960 | -0.406727 | -0.781820 |
| -4.514482 | -0.138368 | -0.271957 | -0.080648 | -0.332397 | -0.221368 | -0.273055 | -0.465039 | -4.295094 |
| -2.784456 | -0.813360 | -0.025536 | -0.674083 | -0.901666 | -0.434675 | -0.460970 | -0.505916 | -0.563012 |
| -0.555087 | -0.359749 | -0.251769 | -0.666546 | -0.524901 | -0.169642 | -0.624412 | -0.513638 | -0.559480 |
| -0.579435 | -0.443784 | -0.745008 | -2.055629 | -0.425547 | -1.196805 | -0.473856 | -1.307859 | -0.557140 |
| -0.823391 | -0.681257 | -1.107963 | -0.967109 | -0.636491 | -0.865874 | -0.532233 | -0.943940 | 0.0637929 |
| -0.733898 | -0.312359 | -0.652546 | -1.048276 | -0.230118 | -1.021153 | -0.247336 | -0.720130 | -0.914037 |
| 0.1852544 | -0.208146 | -0.206386 | 0.9372460 | -0.253285 | 0.1332109 | -0.304054 | 0.1746224 | 0.0645504 |
| 0.1499431 | -0.041330 | -0.081190 | -0.864309 | -0.137606 | 0.0506699 | -0.267754 | 0.0899788 | 0.0557784 |
| -2.307443 | -0.758830 | -1.036030 | -1.655682 | -0.847260 | -1.387834 | -0.402466 | -1.072414 | -2.111416 |
| -0.430047 | -0.334306 | -0.509260 | -1.577610 | -0.215448 | -0.171795 | -0.453279 | -0.399399 | -0.449362 |
| -1.204190 | -0.338115 | -0.958894 | -0.630823 | -0.548419 | -0.804754 | -0.435903 | -1.051310 | -1.330762 |
| -0.166975 | -0.833065 | -1.020201 | -1.618605 | -0.724894 | -1.214636 | -0.427950 | -0.506392 | -0.051735 |
| -1.408521 | -0.632912 | -0.873211 | -0.856558 | -0.906913 | -0.957119 | -0.673725 | -1.890116 | -0.649319 |
| 0.1516220 | 0.1205200 | -0.206566 | 0.0543875 | 0.0239169 | -0.067018 | -0.230486 | -0.578250 | -0.663782 |
| -2.018125 | -0.336856 | -0.959213 | -1.909378 | -0.796773 | -1.238048 | -0.612486 | -1.251567 | -1.130032 |
| 1.2195701 | -0.125522 | -0.133327 | -0.482706 | -0.104914 | 0.3260335 | 0.3435420 | 0.7937703 | 1.3863823 |
| 3.3197546 | 0.1609284 | 1.1942548 | 1.9515172 | 0.7478369 | 2.4173398 | -0.093261 | 1.8448963 | 3.3270716 |
| -0.702853 | -0.521945 | -0.913807 | -1.350733 | -0.352893 | -0.757618 | -0.668246 | -1.251994 | -1.328907 |
| 0.0999725 | 0.0994054 | -0.049915 | -0.464560 | 0.0063869 | -0.107170 | -0.072896 | 0.0825450 | 0.0286124 |
| 0.4550115 | 0.7542348 | 0.4312771 | 0.4224120 | 0.6044176 | 0.3649806 | 0.0364112 | 0.4170383 | 0.6696174 |
| 0.7226134 | 0.3435399 | 0.8355337 | 0.4709135 | 0.3853010 | 0.8823883 | 0.5634550 | 1.0602060 | 0.8409692 |
| -0.716914 | -0.681675 | -0.915821 | -1.414153 | -0.482572 | -0.835276 | -0.161771 | -0.773965 | -1.226579 |
| 1.9564341 | 0.3578999 | 0.8573378 | 0.1551595 | 0.3305618 | 1.1243896 | 0.2010999 | 0.6210383 | 0.7734604 |
| 0.6214920 | -0.016369 | 0.2981775 | 0.1533156 | 0.1837169 | 0.8234225 | -0.344943 | 0.2855329 | -0.413092 |
| -0.531083 | -0.195372 | -0.099771 | 0.1010003 | -0.078954 | 0.0909504 | 0.2374224 | -0.039823 | 0.0845213 |
| -1.325385 | -0.697930 | -0.385948 | -1.349864 | -0.869354 | -0.498346 | -0.392088 | -0.133415 | -0.136046 |
| -1.821965 | -1.171290 | -0.600093 | -0.314013 | -0.878950 | -0.614602 | -0.436085 | -2.616308 | -2.636034 |
| 1.3646550 | -0.559772 | -0.546374 | -0.874385 | -0.459035 | -0.301914 | -0.282621 | 0.4094837 | 0.9918024 |
| 1.0284417 | 0.2355422 | 0.6271092 | 0.0114921 | 0.2839104 | 0.8652108 | -0.482092 | 1.1521639 | 1.9355713 |
| 0.5988325 | -0.354317 | -0.026972 | -0.230722 | -0.249695 | 0.0236169 | -0.236415 | 0.8471870 | 0.7544266 |
| -1.262625 | -0.165906 | -0.379400 | -0.250671 | -0.281513 | -0.404462 | -0.401774 | -0.768975 | -1.003809 |
| 0.3832577 | 0.0561558 | 0.0015120 | -0.254641 | -0.084857 | 0.0594959 | -0.270761 | -0.545399 | -0.115600 |
| -2.272106 | -0.578066 | -0.723515 | -0.507049 | -0.552355 | -0.750214 | -0.592932 | -0.942738 | -1.184747 |
| -1.356389 | -0.402006 | -0.514929 | -0.994090 | -0.315704 | -0.421371 | -0.584984 | -0.763759 | -2.365468 |
| -1.430090 | -0.600403 | -0.239279 | -0.217314 | -0.505368 | -0.356785 | -0.345149 | -0.054991 | -0.112753 |
| 0.1495758 | -0.201558 | -0.046886 | -0.170291 | -0.204927 | 0.1078397 | -0.250940 | -0.041614 | 0.3247265 |

| | | | | | | | | |
|---|---|---|---|---|---|---|---|---|
| 0.2978241 | -0.3483224 | -0.0332695 | -0.8791685 | -0.3158263 | 0.6956257 | -0.3880489 | 0.2196161 | 1.0526628 |
| -0.7145135 | -0.0609284 | -0.4861772 | -1.1359562 | -0.1237618 | -0.7008005 | 0.0265061 | -0.1690149 | -0.4773816 |
| -1.7890581 | -0.6705048 | -0.2471570 | -0.6277124 | -0.5960836 | -0.1265724 | -0.5571385 | -0.2367234 | -0.6582455 |
| -3.0041341 | -0.8677729 | -0.6402007 | -1.9738947 | -0.8063647 | -0.8406027 | -0.2404857 | -0.9475618 | -1.6951730 |
| -1.5864949 | -0.7351105 | -0.8025111 | -0.7238247 | -0.6066304 | -0.9013836 | -0.5701872 | -0.6438382 | -0.7239601 |
| -0.7130514 | 0.2013731 | 0.5468643 | 0.3738557 | 0.0941723 | 0.2151300 | 0.1755757 | 0.1739740 | -0.4374561 |
| 2.0835073 | -0.3363755 | 0.4756803 | -0.3756137 | -0.4678660 | 0.6579081 | -0.1008331 | 1.1814102 | 2.9185342 |
| -0.1838950 | -0.1198266 | -0.3280402 | -0.1714432 | -0.1019214 | -0.1625225 | -0.4363534 | -0.6485523 | -0.6054926 |
| -1.4627250 | -0.1395492 | -0.0670736 | -1.2627106 | -0.0750507 | -0.1066082 | 0.2652717 | -0.6762819 | -1.0743731 |
| -0.0241724 | -0.3583622 | -0.3896646 | -0.9256433 | -0.5393220 | -0.5183976 | -0.4471367 | -1.0097639 | 0.6006278 |
| -0.9881170 | -1.1701224 | -0.6489155 | 0.2264164 | -1.3481545 | -0.4579504 | -0.2194943 | -0.3600458 | -0.9153263 |
| -0.9002335 | -0.7901949 | -0.7457288 | -1.2610361 | -0.6707144 | -0.7943146 | -0.9786954 | -1.4744841 | -1.3169681 |
| -1.7141286 | 0.0434844 | -0.2454069 | -0.1856051 | 0.0058427 | -0.4289191 | -0.1348016 | 0.0486479 | 0.1812232 |
| -0.7812949 | -0.0126778 | -0.2820310 | -0.6838950 | -0.3710996 | -0.7598350 | 0.1541325 | -0.0205001 | -0.1063710 |
| -2.0011064 | -0.3647878 | -0.7754197 | -0.9147648 | -0.3201384 | -1.2606471 | -0.0472985 | -0.6962665 | -0.8031430 |
| -1.1971007 | -0.8823324 | -0.5464383 | -2.2780092 | -0.9796216 | -0.4610791 | -0.3433755 | -0.5362579 | -1.2696467 |
| -1.0912855 | 0.0771300 | -0.1849056 | -0.4192538 | -0.0924311 | -0.4587504 | 0.0105028 | -0.1566925 | -0.4982696 |
| -0.8146831 | -0.4943816 | -0.6462764 | -0.8468161 | -0.4423124 | -0.8647687 | -0.3615455 | -1.1728387 | -1.7670030 |
| -2.6732124 | -0.4380616 | -0.9641145 | -1.9340752 | -0.6711122 | -1.1973009 | -0.1718413 | -1.1979897 | -1.8863240 |
| -0.1219104 | -0.8228630 | -0.1141520 | -1.5029277 | -0.6957830 | -0.0851001 | 0.6842018 | 0.5019713 | 0.7449682 |
| -2.3686485 | -0.4373944 | -0.5940704 | -0.6154856 | -0.3668947 | -0.5100113 | -0.2243510 | -0.6957297 | -0.6005171 |
| -2.5402677 | -0.5850964 | -0.7702539 | -0.5952721 | -0.5161474 | -1.0300342 | -0.1831883 | -1.9377163 | -2.0460196 |
| -2.7831520 | -1.1538698 | -0.3977549 | -0.5500465 | -1.1015455 | -0.3035774 | -0.8733485 | -3.7267091 | -3.7231620 |
| -0.0348947 | -0.2293979 | -0.2534828 | -0.2759461 | -0.3391284 | -0.3651454 | -0.1938983 | -0.1822800 | 0.2352413 |
| -1.4462137 | -0.0367009 | -0.7122760 | -1.4594260 | -0.3154598 | -0.4619548 | 0.2918274 | -0.3854715 | -0.2954338 |
| -0.0486240 | -0.3729264 | -0.3454319 | -0.7616156 | -0.3268328 | -0.3403809 | -0.4143149 | -0.6412975 | -1.0106878 |
| -1.4841416 | 0.0964979 | -0.1634355 | -0.0159677 | 0.0586084 | -0.3345635 | -0.0886625 | -0.3425164 | -1.0728620 |
| -0.7392687 | -0.3058105 | -0.4506088 | -0.6079793 | -0.2665267 | -0.2042634 | -0.5005177 | -0.8861835 | -0.8596062 |
| 0.8375967 | -0.8079047 | -0.3564325 | -0.5923292 | -0.6778390 | -0.1295391 | -0.1051445 | 0.1690342 | 0.1548319 |
| 1.2911560 | -0.3108869 | 0.1361198 | 0.0646441 | -0.1794340 | 0.3066567 | 0.1033718 | 0.1898381 | 1.4261277 |
| -0.9977544 | -0.8386040 | -0.7389132 | -1.1335951 | -0.7366737 | -0.8948700 | -0.6116467 | -0.1579703 | -0.4408137 |
| -0.8980772 | -0.1573903 | -0.4016832 | -0.3928995 | -0.2123698 | -0.4538490 | -0.0718882 | -0.2329628 | -0.7529421 |
| 2.3994242 | -0.7691197 | -0.5766602 | -0.2355417 | -0.6039870 | -0.6446940 | -1.1276442 | -0.7908341 | 3.9479327 |
| -1.5593676 | -0.0675752 | -0.4746026 | -0.4788352 | -0.1383972 | -0.9392663 | -0.1947223 | -0.4461281 | -1.5677510 |
| -0.7314888 | -0.1002129 | -0.1428701 | -0.5230927 | -0.0668573 | 0.2653003 | -0.4814603 | -0.8305473 | -0.6419020 |
| -1.3418274 | -0.6728943 | -0.6286792 | -0.9712480 | -0.8660324 | -0.6275865 | -0.5674721 | -0.5043461 | -0.5182554 |
| -2.7520049 | -0.5812466 | -0.8001440 | -1.9206550 | -0.4150459 | -1.1970870 | -0.7918464 | -2.0994734 | -2.3480639 |
| -4.1204899 | -0.3846377 | 0.0282754 | 0.1062196 | -0.4700350 | -0.1216346 | -0.2744632 | -0.1325821 | -0.2556289 |
| 0.8568087 | -0.0264637 | 0.2580228 | -0.1199554 | 0.0109151 | 0.5981095 | -0.5617840 | 0.5959048 | 1.0420762 |
| -0.7167323 | 0.0512249 | -0.3702974 | -0.2803370 | -0.0635521 | -0.6335703 | -0.2718840 | -0.4543124 | 0.1510784 |
| 1.2108125 | -0.1397397 | 0.0816665 | -0.7465870 | -0.1490089 | -0.2668744 | -0.0570158 | 0.0398690 | 0.6490746 |
| -2.5176995 | -0.4562235 | -0.3509402 | -0.5999594 | -0.4298501 | -0.5476951 | -0.6284036 | -1.0756469 | -1.0056426 |
| -1.0005884 | 0.0153825 | -0.4136330 | -1.0308617 | 0.0218969 | -0.6186191 | -0.6003903 | -0.7766112 | -0.2005438 |
| -0.3696521 | -1.0042731 | -0.5655763 | -1.4284279 | -1.0911984 | -0.7706778 | -0.3715134 | -0.5695752 | 0.6104013 |
| -0.0268318 | -0.2533552 | -0.1416268 | -0.8778872 | -0.3269694 | 0.0022122 | -0.4562138 | -0.8482572 | -0.9191122 |
| -1.2428374 | -0.0240773 | -0.4333534 | -0.8875446 | -0.2413829 | -1.2202847 | -0.7076200 | -0.3054676 | -1.1200882 |
| -0.9520309 | -0.0196005 | -0.2935670 | 0.0058704 | -0.2800314 | -0.1994615 | -0.1688560 | -0.7090011 | -0.3831223 |
| -1.7863751 | -0.8950852 | -1.4391411 | -1.2550622 | -1.4999330 | -1.8807355 | -0.9651096 | -0.5849218 | -0.6610504 |
| 0.5216882 | -0.4038210 | 0.2361879 | 0.1761235 | -0.0414501 | 0.2922623 | -0.0453285 | -0.1634975 | 0.2552306 |
| -1.2394011 | -0.8013428 | -0.9530200 | -1.0375269 | -0.6399135 | -0.9878664 | -0.6106347 | -1.2103169 | -2.0449188 |

| | | | | | | | | |
|---|---|---|---|---|---|---|---|---|
| 0.5466574 | -0.2890650 | -0.0835112 | 0.0963364 | -0.2067822 | 0.2525293 | -0.3951602 | -0.0224358 | 0.7718424 |
| -0.2550601 | -0.1935053 | -0.1143785 | -0.2248926 | -0.1994011 | 0.0071280 | -0.3257471 | -0.6201223 | -0.0320377 |
| -0.2088168 | -0.1346382 | 0.0694101 | -0.1956812 | -0.0104214 | 0.1646886 | -0.2963906 | -0.0745354 | 0.2779744 |

| PG_336 | PG_337 | PG_338 | PG_339 | PG_340 | PG_341 | PG_342 | PG_343 | PG_344 |
|---|---|---|---|---|---|---|---|---|
| -0.531638 | -1.100435 | -2.158755 | -0.366726 | -0.545288 | -0.989343 | -0.508878 | -0.667567 | -1.241587 |
| -0.568794 | -0.674683 | -0.881084 | -0.511026 | -0.945579 | -1.014035 | -0.613996 | -1.053397 | -0.956201 |
| -0.220701 | -0.639706 | 0.3424034 | -0.487558 | -0.346341 | -1.650360 | -0.443556 | -0.750036 | -2.114958 |
| -0.191549 | -0.671833 | 0.1672050 | 0.1380092 | 0.0452343 | 0.2724538 | 0.1942008 | 0.1843795 | 0.1442154 |
| 0.0966880 | 0.1760545 | -1.506210 | -0.011428 | -0.087585 | -0.347364 | 0.0666697 | -0.340663 | -0.046059 |
| -0.397685 | -0.432150 | -1.632464 | -0.092828 | -0.336867 | -1.652503 | -0.352857 | -0.625785 | -1.700418 |
| 0.0621513 | -0.421133 | -0.633465 | -0.558625 | -0.581682 | 0.1813381 | -0.391147 | -0.243470 | 0.6450287 |
| 0.2106734 | -0.379311 | -3.118450 | -0.332153 | -0.309403 | -1.511185 | -0.295151 | -0.459544 | -2.209257 |
| -0.250000 | 0.2794935 | -0.021573 | 0.0139606 | -0.003139 | 0.3897649 | -0.090245 | -0.097400 | 0.2829283 |
| -0.796173 | -1.652494 | -2.341907 | -0.465932 | -0.276353 | 0.5143772 | -0.514919 | -0.473645 | 0.7462006 |
| -0.180698 | -0.206438 | 0.0530267 | -0.210620 | -0.058356 | -0.470974 | -0.195512 | -0.313644 | -0.793580 |
| -0.079721 | -0.167486 | -0.041262 | -0.134214 | -0.346405 | -0.247887 | -0.193869 | -0.589437 | -0.355266 |
| -0.246593 | -0.912345 | -2.223874 | -0.118068 | -0.369993 | -2.188479 | -0.295372 | -1.158646 | -2.294091 |
| -0.717142 | 3.5294996 | 4.8120919 | -0.758095 | 0.9525835 | 3.6212075 | -1.072537 | 2.7673782 | 4.8429795 |
| -0.104637 | -0.319585 | 0.1157707 | 0.1852175 | 0.1905505 | 0.4679988 | 0.2019597 | 0.3067390 | 0.2662329 |
| 1.1179116 | 3.2727445 | 2.8334977 | -0.342109 | 0.9406490 | 1.6116091 | -0.380806 | 2.1445397 | 1.6193046 |
| -1.464258 | -2.679144 | -1.962306 | -0.737341 | -1.450057 | -1.233288 | -0.974235 | -1.479453 | -0.982341 |
| 0.0586401 | -0.345179 | -0.343268 | -0.364555 | -1.924236 | -2.189239 | -0.409385 | -2.608998 | -2.675233 |
| -0.945823 | -0.835094 | 0.7596785 | -0.713619 | -0.778523 | -0.174900 | -0.583077 | -0.708230 | -0.455977 |
| -1.689718 | -1.485637 | -1.335308 | 0.2585488 | -0.965512 | -3.229459 | 0.1426995 | -1.940110 | -4.291617 |
| -0.276037 | 1.2274534 | 0.6217563 | -0.386278 | 0.2321098 | -0.031694 | -0.118033 | 0.1950755 | 0.0313356 |
| -0.318345 | 0.1429853 | -0.216832 | -0.795950 | -0.408699 | -0.275189 | -0.269021 | -0.263036 | -0.304890 |
| -0.055823 | 1.0739885 | 1.9317506 | 0.1145133 | 0.6998647 | 1.0421918 | -0.070242 | 0.9846649 | 1.1768995 |
| -0.364646 | -1.558878 | -2.114606 | -0.355725 | -0.549671 | -1.527690 | -0.513717 | -1.264548 | -1.885911 |
| -1.244827 | -1.723539 | -1.616646 | -0.234852 | -0.713902 | -1.763685 | -0.169966 | -1.457054 | -2.717361 |
| 0.3797426 | 0.4659319 | 1.1393929 | -0.046204 | 0.6776460 | 0.2680093 | 0.1286110 | 0.2191604 | 0.2284496 |
| 0.1294133 | 0.1574537 | -0.646291 | 0.0767499 | 0.0604233 | -0.288765 | 0.2416450 | -0.072773 | 0.0171329 |
| -0.290098 | -1.101076 | -1.244826 | -0.098410 | -0.476354 | -0.326557 | -0.191815 | -0.255372 | -0.972924 |
| 0.0947925 | -0.750037 | -0.924279 | -0.022682 | -0.424746 | -3.215693 | -0.100718 | -0.911002 | -3.395624 |
| 0.0286245 | -0.717430 | -0.678302 | -0.085604 | -0.292582 | -0.031504 | -0.114361 | -0.152210 | -0.064352 |
| 0.0175210 | -1.450374 | -2.185154 | -0.247742 | -0.745422 | -2.069481 | -0.319836 | -1.464213 | -3.341245 |
| -0.283799 | 0.5246894 | 0.2108778 | 0.2186193 | 0.4775352 | -0.326699 | 0.4611856 | 0.2591161 | -0.954510 |
| -0.123610 | 0.4143409 | -0.697655 | -0.228507 | -0.538824 | 0.2681829 | -0.489695 | 0.3820408 | 0.2953993 |
| -0.436608 | -0.223163 | -1.982509 | -0.401789 | -0.374649 | -3.477983 | -0.398175 | -1.168179 | -4.539611 |
| -0.463782 | -0.236852 | -0.527835 | -0.099719 | -0.175040 | -0.090995 | -0.112231 | -0.153478 | -0.080438 |
| -1.381192 | -1.364155 | -0.663762 | -0.235713 | -1.117270 | -0.581848 | -0.619942 | -0.378935 | 0.5983151 |
| -0.491626 | -1.010813 | -1.675604 | -0.116827 | 0.0525389 | -1.927694 | -0.393018 | -0.897159 | -2.429820 |
| 1.2319140 | 2.0090988 | 0.0799161 | -0.270326 | 3.2068835 | 2.0102579 | 0.2574871 | 3.7152659 | 0.9073022 |
| -0.671125 | -0.377470 | -2.748186 | -0.207152 | 0.3084187 | 0.4328880 | -0.131669 | 0.5339701 | -0.247121 |
| -0.741444 | 0.0462483 | -0.037978 | -0.618022 | -1.595571 | 0.8102261 | -1.319440 | -0.787091 | 1.9653981 |
| -0.540888 | -0.356411 | 0.0869161 | -0.342204 | 0.0671292 | -0.137893 | -0.406362 | 0.0643961 | 0.7300216 |
| -0.771130 | -2.117085 | -2.006912 | -0.644097 | -0.531530 | -1.744230 | -0.648452 | -0.993157 | -1.567533 |
| -0.340927 | 0.0652955 | -0.172090 | -0.012117 | 0.0087943 | -0.256941 | -0.196523 | -0.044205 | -0.202112 |
| -0.245195 | -0.333639 | -0.784328 | -0.085324 | -0.116078 | -0.662935 | 0.0403489 | -0.157414 | -1.225106 |
| 0.2754769 | 0.6727545 | 1.2642215 | 0.0462391 | 0.2511242 | 0.2339589 | -0.198375 | 0.1321459 | 0.4563747 |
| -0.112216 | 0.1919501 | 0.1037931 | -0.389319 | -0.073260 | -0.432859 | -0.250574 | -0.380529 | -0.089689 |
| -0.186148 | -0.268500 | -0.388143 | -0.091868 | -0.368897 | -0.707841 | -0.174346 | -0.391684 | -0.484057 |

| | | | | | | | | |
|---|---|---|---|---|---|---|---|---|
| -0.5657902 | -0.2699947 | -0.6397632 | -0.0769475 | -0.0575727 | -0.0288724 | -0.2432052 | 0.2264642 | 0.0996521 |
| -0.3014025 | -0.6322203 | -2.4230399 | -0.6093171 | -0.7302899 | -1.7365055 | -0.5342528 | -0.8963374 | -2.7247265 |
| -0.3432793 | 0.3018250 | 0.3796017 | -0.5025805 | -0.1943393 | -0.1822592 | -0.5602396 | 0.0560200 | -0.0069054 |
| -1.5318955 | 2.4768678 | 2.2475305 | 0.2001919 | 0.8758694 | -1.1078960 | -0.9579757 | 1.0145204 | -1.5198734 |
| -0.1543421 | 0.1731108 | -0.0933709 | -0.2751995 | -0.2872278 | -0.3159093 | 0.0103139 | -0.3187450 | -0.1576410 |
| -0.0598641 | 0.2032849 | 0.3432681 | -0.2034378 | -0.2975845 | -0.2563823 | -0.2369552 | -0.2794947 | -0.0530372 |
| -0.6579048 | -0.9225705 | -2.2195699 | -0.1798001 | -0.4775441 | -1.8506627 | -0.3829252 | -1.3272708 | -1.9495188 |
| 0.7771781 | 2.8322147 | 2.5342739 | -0.1446626 | 1.6050127 | 2.8995721 | 0.2688512 | 2.8843429 | 3.3067091 |
| 2.1420764 | 4.1818822 | 3.0980716 | -0.1306801 | 3.4237226 | 2.2617949 | 0.4062291 | 4.1925318 | 1.7966230 |
| -0.2265220 | -0.2936835 | 0.5923243 | -0.6306705 | -0.5030185 | -0.2036837 | -0.5644173 | 0.1807875 | 0.0949626 |
| -0.8151210 | -1.4861100 | -2.4378293 | 0.0131395 | -0.9460489 | -1.1853178 | -0.3311940 | -1.0101895 | -1.6508988 |
| 1.6096546 | 2.5374913 | 2.4536046 | -0.2044817 | 1.8167705 | 2.8030470 | 0.2117745 | 2.8166197 | 2.7852649 |
| 2.0796628 | 3.5469119 | 2.4019931 | -0.0478309 | 1.0029180 | 1.4409889 | 0.0541189 | 2.1848512 | 1.5091683 |
| -0.6132872 | -0.6326720 | -1.1859183 | -0.4511151 | -0.4332385 | 0.0325354 | -0.4170580 | -0.4914934 | 0.3397266 |
| -1.1588448 | -1.1475256 | -1.3792046 | -0.4593460 | -1.4320475 | -2.3500950 | -0.9009287 | -1.7102904 | -2.6675528 |
| -0.8673196 | -0.9390590 | 0.1930657 | -0.3117631 | -0.6968610 | -0.6621842 | -0.3105009 | -0.4699151 | 0.0132224 |
| -0.3075897 | -1.4761499 | -1.9380259 | -0.0874455 | -0.1411582 | -1.1230066 | 0.0374519 | -0.3902185 | -1.8853906 |
| -0.3772257 | -0.7504526 | -0.4394255 | -0.2998441 | -0.3092788 | -0.3664896 | -0.0757841 | -0.9556860 | -0.0447535 |
| -0.6595789 | -0.9301966 | -1.8872839 | -0.4057639 | -0.2083792 | -0.7998668 | -0.2718677 | -0.5422804 | -0.5129999 |
| -0.0258176 | -0.4075573 | -1.4894798 | -0.0366073 | -0.1891093 | 0.3485640 | -0.2421917 | 0.1858017 | 0.3375626 |
| -0.2065166 | -0.6567351 | 0.4406932 | -0.2808185 | -0.4510847 | -0.9783400 | -0.3933796 | 0.1253653 | -1.3932949 |
| -0.4903009 | -1.1037984 | -1.5554082 | -0.8500201 | -0.8023999 | -2.0667767 | -0.6705792 | -1.4041012 | -2.7047373 |
| -0.7183502 | -0.5411738 | -0.3453318 | -0.2836477 | -0.3272574 | -0.3255835 | -0.1897122 | -0.4382792 | -1.3853989 |
| 0.3684706 | 0.3123571 | 0.7278707 | 0.3196850 | 0.5566631 | 1.0296979 | 0.6444217 | 0.8064630 | 0.7738459 |
| -0.5840912 | -2.2528308 | -1.5242980 | -0.3703952 | -0.5238048 | -1.5157828 | -0.3173931 | -0.8711090 | -2.3323389 |
| -0.0934381 | -0.5957002 | -0.4331377 | -0.2091653 | -0.4605336 | -0.1736180 | -0.1504177 | -0.6166658 | -0.1865675 |
| -0.7588297 | -0.5085894 | -0.5816322 | -0.5847972 | -2.1266855 | -3.0985572 | -0.7240918 | -3.3843915 | -3.8384029 |
| 0.9796202 | 3.0676370 | 4.5739334 | 0.0171582 | 1.3000933 | 3.3100129 | 0.2588305 | 1.9413661 | 3.9308938 |
| -0.4204880 | 1.3200153 | 2.5199199 | -0.4033302 | 0.3937634 | 1.6250408 | -0.6869818 | 0.8541680 | 2.4683477 |
| -0.4060701 | -1.4371008 | -1.6241405 | -0.2907267 | -0.3832585 | -1.3080997 | -0.2099866 | -0.4535573 | -1.9254867 |
| -0.4570612 | -0.4700164 | -0.3085453 | 0.5332327 | 0.0072079 | 0.2748859 | 0.0169479 | 0.2474650 | 0.0313013 |
| -0.1086848 | -0.5145522 | -0.3400555 | -0.1974354 | -0.1386507 | -0.1304743 | -0.1511870 | -0.3878103 | 0.4147036 |
| -0.8978888 | -1.1880462 | -1.0734281 | -0.4954192 | -0.7669026 | -1.5540720 | -0.4915752 | -1.9403990 | -1.9707207 |
| -0.4992762 | -1.0593659 | -1.2237293 | -0.1151737 | -0.3825076 | -0.5148630 | -0.2029239 | -0.6232511 | -0.6228131 |
| 0.3153956 | 1.7706388 | 1.4929117 | -0.2662201 | 0.5543013 | 1.0503427 | -0.1560963 | 0.8309737 | 1.2428206 |
| 0.0250530 | -0.4681047 | -0.2953100 | 0.1364945 | 0.0301521 | -0.3007703 | 0.0234358 | -0.2814673 | -0.2932656 |
| -0.3351776 | -0.7356476 | -1.2301091 | -0.3553503 | -0.5319245 | -0.2274920 | -0.4412000 | -0.6039338 | 1.3512010 |
| -0.5269329 | -1.9611196 | -2.1116600 | -0.0654790 | -0.1562750 | -1.3940501 | 0.0683569 | -0.8220765 | -1.5670548 |
| -0.5051250 | -0.9079332 | -1.2098513 | -0.0891844 | -0.2989504 | -1.7068957 | -0.1434055 | -1.1151670 | -2.1525137 |
| -0.3516973 | -1.2387020 | -1.2213050 | -0.4953064 | -1.2133069 | -0.6839634 | -0.6227195 | -1.5202756 | -1.1399428 |
| -0.8298452 | -1.3020139 | -1.1214116 | -0.4096823 | -0.3930259 | -0.0914117 | -0.6759061 | -0.1191439 | -0.0953893 |
| -1.3237887 | -2.7974592 | -2.8737219 | -0.2303266 | -1.5836379 | -0.2134562 | -0.6529236 | -1.7336109 | -0.6566528 |
| 1.6687118 | 3.8062026 | 1.9778933 | 0.1881032 | 1.3746914 | 0.9279573 | 0.4905530 | 2.4419921 | 0.6927411 |
| -0.2322787 | -0.1680516 | -0.4316218 | 0.0545409 | 0.0438952 | -0.0213126 | 0.1631919 | 0.2453055 | 0.0456507 |
| -0.3537734 | -0.6443447 | -1.5536705 | 0.1295427 | -0.4015478 | -0.5030771 | 0.0784513 | -0.3663569 | -0.5499738 |
| -1.1766257 | -1.8734313 | -1.5324153 | -0.5124293 | -1.2476132 | -1.0373393 | -1.0302575 | -1.4508029 | -1.1024475 |
| 2.7915996 | 7.3288537 | 7.0014621 | 0.0884403 | 3.1469361 | 6.2952469 | -0.0244536 | 6.3775304 | 6.8219676 |
| 0.6512206 | 2.9198006 | 1.8995846 | -0.2080551 | 1.1511806 | 2.2169336 | 0.0579445 | 2.3620980 | 2.3414775 |
| -2.3372014 | -1.2184678 | -4.0671969 | 0.0121415 | 0.3986804 | -0.8347783 | 0.2608969 | 0.1945337 | -1.4037129 |
| 0.0393612 | 0.5143679 | -0.1499059 | -0.2039434 | -0.1922601 | 0.0087312 | -0.1963710 | -0.2262827 | -0.1771338 |

| | | | | | | | | |
|---|---|---|---|---|---|---|---|---|
| -1.491613 | -2.804455 | -2.512710 | -0.717136 | -1.253200 | -1.907318 | -0.694970 | -1.541782 | -1.543700 |
| -0.550565 | -0.392169 | 0.2212744 | -0.269716 | -0.119006 | 0.1303039 | -0.095001 | -0.170385 | 0.2631836 |
| -0.583899 | -1.155214 | -0.270473 | -0.257795 | -0.327372 | -0.072171 | -0.288854 | -0.809699 | 0.0158518 |
| -0.143949 | -0.160277 | -0.772556 | 0.0727496 | 0.0189438 | -0.077233 | -0.016297 | 0.0840049 | 0.0786093 |
| -0.548475 | -1.139338 | -1.627001 | -0.447374 | -0.992398 | -1.466873 | -0.432691 | -2.051521 | -2.106517 |
| -1.056712 | -0.427650 | 0.7153493 | -0.092241 | -0.334761 | 0.5921197 | -0.088796 | 0.2737265 | 0.8502578 |
| 0.3424892 | -0.311019 | 0.4404746 | -0.334409 | -0.141974 | -0.017275 | -0.156531 | 0.2347831 | -0.464885 |
| -0.264963 | -0.100163 | -0.087435 | -0.132148 | 0.0011678 | -0.148613 | -0.044806 | 0.0957401 | -0.138910 |
| -0.381428 | -0.403398 | -0.234367 | -0.050336 | -0.059623 | -0.099775 | -0.223407 | -0.115041 | -0.276707 |
| 0.1966890 | 1.2420624 | 1.2722087 | -0.078012 | 0.3216603 | 0.3742686 | -0.148339 | 0.4903775 | -0.064645 |
| -0.144843 | -0.586704 | -1.486553 | -0.016622 | -0.222207 | -0.209809 | 0.0364285 | -0.490940 | -0.568287 |
| -0.503296 | -0.381039 | -1.928964 | -0.222983 | -0.644615 | -1.789978 | -0.163170 | -1.047576 | -2.374870 |
| -0.573475 | -1.628666 | -4.915488 | -0.333761 | -0.057835 | -3.733574 | -0.143849 | -0.850467 | -4.198202 |
| -0.239732 | -0.777021 | -2.952350 | -0.222518 | -0.342281 | -2.079376 | -0.279097 | -0.638899 | -2.655888 |
| -0.510299 | -1.262743 | -0.259418 | -0.147376 | -0.306295 | -0.707721 | -0.063732 | -0.594931 | -0.626713 |
| -0.250055 | 0.0580563 | -0.347096 | -0.040184 | -0.177157 | 0.1301415 | -0.102795 | -0.064044 | -0.056011 |
| -0.680243 | -1.490459 | -1.463079 | -0.518895 | -1.388570 | -0.985551 | -0.641659 | -1.562368 | -0.695419 |
| -0.002183 | -0.276805 | 0.4278328 | -0.299519 | -0.283227 | -0.053420 | -0.179435 | -0.396529 | 0.1986686 |
| -0.605638 | -1.066169 | -0.575389 | -0.206001 | -0.266891 | -0.378996 | -0.059703 | -0.688796 | -1.017652 |
| -1.477930 | -1.137475 | 0.4931604 | -0.605325 | 0.0900289 | 1.4653355 | 0.0168803 | 0.5942441 | 1.4332883 |
| -0.082383 | -0.562777 | -0.903710 | -0.356841 | -0.356257 | -1.043867 | -0.306555 | -0.383383 | -1.281989 |
| -0.647853 | -0.609799 | 0.0222080 | -0.216979 | -0.348983 | 0.1304501 | -0.196717 | -0.159599 | 0.2075714 |
| 0.0676867 | 0.1672061 | -0.036343 | -0.100957 | -0.130691 | -0.113234 | -0.222467 | -0.105708 | -0.154134 |
| -0.297656 | -1.097907 | -2.736727 | -0.583943 | -0.424615 | -1.665859 | -0.454148 | -0.635181 | -2.338893 |
| -0.532171 | -0.271337 | -0.244140 | -0.247616 | -0.289586 | -0.363125 | -0.551302 | -0.482263 | -0.188493 |
| -0.799359 | -2.024698 | -1.240904 | -0.279343 | -0.115427 | -0.389196 | -0.039457 | -0.411246 | -0.866295 |
| -0.142515 | 0.1167290 | 0.2036745 | -0.115548 | 0.1732008 | 0.0608212 | 0.0438657 | -0.171921 | -0.070885 |
| 0.0868743 | -0.229416 | -0.412427 | -0.450675 | -0.088845 | 0.3694781 | -0.335910 | -0.151539 | 0.0447313 |
| -0.979792 | -1.729113 | -0.696435 | -0.541392 | -0.687963 | 0.4372530 | -0.651559 | -0.370695 | 0.1738226 |
| -0.596870 | -0.286201 | -1.952154 | -0.098438 | -0.392003 | -0.644372 | -0.241164 | 0.0553504 | -0.871034 |
| -0.863754 | -3.060934 | -3.104833 | -0.285799 | -0.731521 | -1.535704 | -0.553756 | -0.440783 | -1.005231 |
| -0.866408 | -0.839172 | -1.190878 | -0.147116 | -0.366506 | -0.376663 | -0.161792 | -0.289434 | -1.097669 |
| -0.123697 | -0.108650 | 0.7054300 | -0.359267 | -0.743041 | -0.935182 | -0.267770 | -1.342333 | -0.546737 |
| -0.167410 | -0.276860 | -0.512337 | 0.3306868 | 0.3295419 | 0.0069040 | 0.2712957 | 0.2273277 | -0.226952 |
| -0.502060 | -1.033008 | -2.094640 | -0.299893 | -0.136039 | -1.354889 | -0.233282 | -0.520364 | -2.256182 |
| -0.321414 | -0.757760 | -0.598656 | 0.0624874 | -0.209012 | -1.050343 | -0.180843 | -1.059417 | -1.620535 |
| -0.224910 | -0.562381 | -0.075271 | -0.121770 | 0.0492024 | -0.017947 | -0.151276 | -0.026112 | -0.149314 |
| -0.150236 | -0.736967 | -1.033442 | -0.047439 | -0.326966 | -0.013673 | 0.0757602 | -0.335590 | -0.204491 |
| -0.204290 | -0.142948 | -0.142331 | -0.244618 | -0.287968 | -0.565230 | -0.234334 | -0.646216 | -0.671450 |
| -0.654869 | 0.1986643 | -0.629357 | 0.1510790 | -0.603287 | -2.275148 | 0.1652754 | -0.683871 | -1.586776 |
| 0.4236608 | 0.9514211 | -0.363852 | -0.313238 | -0.933448 | 0.3755578 | -1.196717 | -0.402901 | 0.7143598 |
| -0.095249 | 0.2624097 | -1.399410 | -0.058702 | 0.0178269 | -0.398835 | -0.091164 | 0.0629962 | -0.767251 |
| -0.160411 | -0.376296 | -1.431143 | -0.118009 | -0.001966 | -0.705831 | 0.0595869 | 0.0145439 | -0.972192 |
| -0.878898 | -0.719609 | -0.748327 | 0.2050847 | -0.086496 | -0.624173 | 0.2044901 | -0.238675 | -0.501042 |
| 0.0221608 | -0.062592 | -0.260057 | -0.097511 | -0.186897 | -0.889698 | -0.231465 | -0.154141 | -0.606585 |
| -0.009237 | 0.6069789 | 0.1575254 | -0.352605 | 0.1449128 | 0.1015241 | -0.098674 | 0.6148514 | 0.2501148 |
| -1.350976 | -1.515237 | -1.173449 | -0.130029 | -0.383688 | -1.249002 | -0.342545 | -0.817818 | -1.350335 |
| -0.188501 | -0.761499 | 0.7481714 | -0.377174 | 0.2025966 | 0.9000238 | -0.074599 | 1.1150397 | 0.8257115 |
| -0.242295 | 0.0839070 | -0.456420 | -0.219411 | -0.530706 | -0.465745 | -0.338813 | -0.535601 | -0.403213 |
| -0.684382 | -1.395957 | -2.136965 | -0.503090 | -0.689875 | -2.145573 | -0.685397 | -0.690365 | -2.836873 |

| | | | | | | | | |
|---|---|---|---|---|---|---|---|---|
| -0.173251 | -0.039554 | -0.018356 | -0.014351 | 0.2541664 | 0.6865292 | 0.3146946 | 0.4017107 | 0.7008588 |
| -0.683578 | -1.350293 | -2.044290 | -0.329683 | -0.658918 | -1.824016 | -0.485653 | -1.589796 | -1.718285 |
| -0.502246 | 0.0127438 | -0.726747 | -0.292009 | -0.176187 | -0.394369 | -0.371748 | -0.351618 | -0.570372 |
| -0.340464 | -0.014051 | -0.105517 | -0.297132 | -0.196125 | -0.406661 | -0.370822 | -0.226221 | -0.374926 |
| -0.603368 | -1.875764 | -2.047412 | 0.0824389 | -0.646777 | -1.430846 | 0.1566146 | -1.015681 | -1.432882 |
| 1.3688067 | 2.4674607 | 1.8100978 | -0.738100 | 1.1999749 | 0.3878541 | -0.252871 | 1.4261758 | -0.185375 |
| -0.342653 | -0.571447 | -0.427312 | -0.317135 | -0.282726 | -0.554592 | -0.367276 | -0.718134 | -0.975642 |
| -0.463332 | -1.017366 | -0.553914 | -0.464564 | -0.298174 | -0.522663 | -0.684907 | -0.742360 | -0.747849 |
| -1.055135 | -1.678639 | -1.982187 | -0.507282 | -0.587895 | -1.763578 | -0.296505 | -1.262579 | -1.465520 |
| -1.121679 | -0.723054 | -0.192265 | -0.369542 | -0.852415 | -0.412769 | -0.553319 | -1.014795 | -0.373392 |
| 0.0563389 | 0.1916723 | 0.2229455 | 0.1401577 | 0.0254622 | 0.2174377 | 0.0629385 | 0.0897188 | 0.8802260 |
| -0.362191 | -0.655572 | -0.079296 | -0.733282 | -0.757149 | -1.269016 | -0.577311 | -1.152228 | -1.469330 |
| -0.548721 | -0.290857 | -0.358685 | -0.580014 | -0.688875 | 0.5302492 | -0.752570 | -1.127757 | 0.5627246 |
| -0.726601 | -0.940432 | -0.397284 | -0.558708 | -0.534075 | 0.2853489 | -0.390114 | -0.103078 | 0.7064928 |
| 0.0209157 | -1.106589 | -3.806806 | -0.191373 | -0.216865 | -0.291926 | -0.275720 | -0.201333 | -0.344340 |
| -0.222586 | 0.0352545 | -0.225381 | 0.3656160 | 0.2092208 | -1.821111 | 0.2504929 | 0.0616090 | -0.822110 |
| -0.590341 | -0.240279 | -0.653975 | -0.462818 | -0.672311 | -0.843949 | -0.708004 | -0.712461 | -0.862637 |
| -0.589418 | -1.564147 | -0.693293 | -0.217090 | -0.853326 | -1.312349 | -0.689445 | -2.027182 | -1.944184 |
| -0.509758 | -1.354138 | -0.099067 | -0.382743 | -0.754679 | -0.685618 | -0.615580 | -0.868464 | -0.655670 |
| -0.210394 | -0.731418 | -1.350429 | -0.353114 | -0.513932 | -0.196398 | -0.520310 | -0.764555 | -0.522772 |
| -0.069811 | 0.6105033 | 0.4738951 | -0.095008 | -0.019736 | 0.9793985 | -0.068840 | 0.5285343 | 1.6940949 |
| -0.234544 | -0.361594 | 0.0656716 | -0.042790 | 0.0475599 | 0.2781287 | -0.082788 | 0.4541694 | -0.277914 |
| -1.278177 | -2.211810 | -2.478203 | -0.650569 | -1.113175 | -1.293988 | -0.632175 | -1.538146 | -1.354745 |
| -0.597202 | -0.413302 | -0.640413 | -0.311620 | -0.243529 | -0.814680 | -0.401435 | -0.451049 | -1.071384 |
| -0.590393 | -1.311338 | -1.469225 | -0.063625 | -0.555434 | -1.487801 | -0.258489 | -1.113894 | -1.453477 |
| -0.441664 | -1.145417 | 0.2791106 | -0.286696 | -0.375692 | 0.0158695 | -0.245105 | -0.675924 | 0.3021807 |
| -1.209807 | -2.088610 | -0.844694 | -0.326713 | -0.441548 | -0.310221 | -0.317914 | -0.579299 | -0.897920 |
| -0.366662 | -0.857732 | -0.538239 | -0.370059 | -0.685909 | -0.564391 | -0.625466 | -0.850550 | -0.408212 |
| -0.943279 | -1.857723 | -1.367075 | -0.426507 | -0.650237 | -1.062314 | -0.294703 | -0.864151 | -1.491259 |
| 0.6343519 | 0.9846063 | 1.1934389 | 0.0024855 | 0.4732076 | 0.6634447 | 0.2931440 | 0.8219471 | 0.5582962 |
| -0.016584 | 3.4340683 | 3.1942150 | -0.196116 | 0.4269748 | 1.6314270 | -0.614265 | 1.5524742 | 2.2437051 |
| -0.702973 | -0.867417 | -1.102582 | -0.662462 | -0.671129 | -1.237579 | -0.587367 | -0.933578 | -0.885911 |
| -0.130917 | 0.1135978 | -0.130465 | 0.1484853 | 0.1551910 | -0.035432 | 0.2131270 | 0.0689747 | -0.216656 |
| 0.0895161 | 0.5413710 | 0.7004091 | -0.060356 | 0.3141093 | 0.7153832 | 0.0978903 | 0.3236908 | 0.4106650 |
| 0.3478721 | 0.9480864 | 0.3776570 | 0.2216007 | 0.6413796 | 1.1039256 | 0.1116748 | 0.8802987 | 0.7476943 |
| -0.317894 | -1.023878 | -1.673119 | 0.1176998 | -0.307475 | -0.977060 | -0.229576 | -0.436894 | -1.034326 |
| 0.1557566 | 0.1109636 | 0.9718872 | 0.1706414 | 0.6736823 | 1.2580222 | 0.3691672 | 1.0750546 | 1.7374486 |
| -0.076210 | 0.7495989 | -0.035804 | -0.407087 | 0.1594140 | -0.053151 | -0.097193 | 0.6253242 | 0.0813539 |
| 0.2889818 | 0.0227628 | -0.040614 | -0.279645 | -0.088694 | 0.0084225 | -0.207552 | 0.2040988 | 0.2582745 |
| -0.697261 | -0.221380 | -0.372436 | -0.322230 | -0.280152 | -0.419326 | -0.265151 | -0.184652 | -0.459998 |
| -1.224399 | -3.450181 | -3.392769 | -0.658453 | -1.831443 | -1.499983 | -0.785719 | -2.390587 | -1.864627 |
| -0.357075 | 0.5478918 | 0.7026451 | -0.399826 | -0.209540 | 0.2179423 | -0.399628 | 0.3244894 | 0.1334905 |
| 0.3537282 | 0.7542813 | 1.7595899 | -0.022236 | 0.6329257 | 1.0374304 | 0.1296185 | 0.8644281 | 1.4092515 |
| 0.0205525 | 0.8539176 | 0.8781077 | 0.0413520 | 0.0806155 | 0.3741164 | 0.1514644 | 0.3883461 | 0.6136967 |
| -0.282738 | -0.901471 | -0.562847 | 0.0808306 | -0.143688 | -0.247884 | 0.1215869 | -0.259304 | -0.107378 |
| -0.313999 | -0.448308 | 0.0855366 | -0.062751 | 0.0456242 | 0.1291930 | -0.082648 | 0.1391776 | 0.1911280 |
| -0.756693 | -1.264242 | -1.585458 | -0.522198 | -0.728492 | -1.488625 | -0.679250 | -1.102737 | -1.483230 |
| -0.827502 | -1.106001 | -2.352529 | -0.523245 | -1.219417 | -1.524752 | -0.635162 | -1.621336 | -1.809962 |
| -0.610195 | -0.095330 | 0.3962033 | -0.105587 | -0.082617 | -1.058647 | 0.1026729 | -0.400994 | -0.952969 |
| -0.325904 | -0.203031 | 0.1845378 | -0.254276 | -0.096131 | -0.019803 | -0.273373 | -0.222107 | 0.0526359 |

| | | | | | | | | |
|---|---|---|---|---|---|---|---|---|
| -0.115001 | 0.6048811 | 0.9534348 | 0.3588897 | 0.2524044 | 0.6723447 | 0.4205760 | 0.5076894 | 0.8092898 |
| -0.201245 | -0.186838 | -0.613147 | -0.521942 | -0.592589 | -0.971827 | -0.416667 | -0.657914 | -1.468018 |
| -0.992968 | -0.003642 | -1.035870 | -0.284595 | -0.090070 | -1.057375 | -0.497356 | 0.0510751 | -1.737234 |
| -0.844841 | -1.541331 | -1.210102 | -0.182761 | -0.663110 | -2.050536 | -0.430553 | -0.859864 | -2.349451 |
| -0.971398 | -0.987609 | -1.098154 | -0.268557 | -0.481867 | -0.968783 | -0.219919 | -0.545388 | -1.013219 |
| 0.2392413 | -0.317600 | -0.711291 | -0.516580 | 0.2699812 | -0.291815 | -0.141811 | 0.2495385 | -0.783741 |
| 0.1482529 | 1.4373801 | 2.6981198 | -0.109441 | 0.6600820 | 0.2422483 | 0.0797244 | 0.6521861 | -0.244384 |
| -0.411423 | -0.596031 | -0.404458 | -0.203402 | -0.079934 | -0.405804 | 0.0685082 | -0.033427 | -0.173546 |
| 0.3760567 | -0.896713 | -1.425010 | 0.6151354 | 0.1221009 | -0.246452 | 0.2552057 | 0.1393771 | -0.192094 |
| -1.108833 | -0.902779 | 0.8719194 | -0.300574 | -0.476512 | -0.500986 | -0.189858 | -0.805981 | -0.416464 |
| -0.471210 | 0.3270032 | -1.334687 | -0.033679 | -0.709992 | 0.0399543 | -0.521152 | -0.264222 | 0.2795004 |
| -1.768148 | -0.885937 | -0.827792 | 0.0460555 | -0.802706 | -0.414602 | -0.097236 | -0.324191 | -0.380074 |
| -0.146648 | -0.229486 | -0.126840 | -0.356700 | -0.072999 | -0.167434 | -0.195740 | -0.103742 | -0.144598 |
| -0.408309 | -0.616182 | -0.387341 | 0.0790865 | 0.0543259 | 0.0875652 | 0.2811224 | 0.0525813 | 0.3470705 |
| -0.109260 | -1.183316 | -0.891063 | 0.0558655 | -0.157173 | -1.374477 | -0.024158 | -0.742340 | -1.753119 |
| -0.923555 | -0.839071 | -1.827483 | -0.083998 | -0.180759 | -0.056227 | -0.165473 | 0.0448347 | -0.874020 |
| 0.0586426 | -0.445599 | -0.886128 | -0.290715 | -0.157713 | -1.073321 | -0.028546 | -0.217585 | -1.287715 |
| -0.593308 | -1.235226 | -1.879207 | -0.232022 | -0.982935 | -0.492611 | -0.070881 | -1.176349 | -0.517035 |
| -0.616004 | -2.245270 | -2.242364 | -0.189579 | -0.508422 | -0.869300 | -0.048182 | -1.117321 | -1.118218 |
| 0.2659973 | 0.3000240 | 0.6013356 | 0.4515022 | 0.0179746 | 0.0325520 | 0.2086899 | 0.2824711 | -0.018450 |
| -0.199709 | -0.997442 | -0.807010 | -0.393335 | -0.777546 | -0.273948 | -0.397176 | -0.560701 | -1.370125 |
| -1.147006 | -1.820403 | -1.692041 | -0.438373 | -0.439004 | -0.304881 | -0.493920 | -0.396214 | -0.318100 |
| -1.143214 | -3.386537 | -2.600963 | -0.665337 | -1.195356 | -3.664707 | -0.731649 | -1.959307 | -4.445717 |
| -0.371031 | -0.225585 | 0.1822692 | -0.194595 | -0.072804 | 0.2798032 | 0.0106254 | -0.124219 | 0.2355371 |
| 0.1726623 | -0.870162 | -0.433330 | -0.154834 | -0.262624 | -1.161018 | 0.0867046 | -0.185171 | -1.159791 |
| -0.421719 | -0.460194 | -1.038802 | -0.497094 | -0.750682 | -0.052188 | -0.489148 | -0.650537 | 0.0558540 |
| 0.0452870 | -0.363179 | -0.903516 | -0.284212 | -0.232799 | -1.393940 | -0.325680 | -0.502658 | -0.946806 |
| -0.317994 | -0.993410 | -0.912848 | -0.340345 | -0.430893 | -1.131386 | -0.620115 | -0.759165 | -1.354782 |
| 0.0781551 | 0.1378287 | 0.3464150 | 0.0543638 | 0.2412051 | 0.4833839 | 0.2643055 | 0.4287626 | 0.5615990 |
| -0.510995 | 0.3437505 | 1.4071063 | -0.229207 | -0.104120 | 0.7644412 | -0.134616 | 0.0602936 | 0.8917689 |
| -0.794751 | -0.841582 | -0.928155 | -0.401728 | -0.494469 | -0.734103 | -0.465256 | -0.148634 | -0.697855 |
| 0.0012426 | -0.412785 | -0.673500 | -0.060632 | -0.086912 | -0.264800 | -0.115552 | -0.289950 | -0.169284 |
| -0.907684 | 1.1262385 | 4.2557777 | 0.0911155 | -0.123402 | 0.9142124 | -0.158125 | 0.2178711 | 0.1001826 |
| -0.215614 | -0.889891 | -1.489599 | -0.124997 | -0.314754 | -1.009907 | -0.324277 | -0.685831 | -0.951199 |
| -0.572302 | -0.761101 | -1.003239 | -0.102235 | -0.000082 | -0.286621 | -0.148779 | -0.110246 | -0.555963 |
| -0.719767 | -0.958539 | -0.842001 | -0.178984 | -0.417195 | -1.417809 | -0.408734 | -0.859395 | -3.132250 |
| -1.399604 | -2.789912 | -3.004497 | -0.393951 | -0.783402 | -2.228510 | -0.413078 | -1.835858 | -3.037685 |
| -0.238403 | 0.0901930 | -0.210689 | -0.087959 | -0.212967 | -0.051411 | 0.0100853 | -0.228502 | -0.177749 |
| -0.495009 | 0.7686398 | 0.7917068 | -0.028454 | 0.7470540 | 1.1258049 | -0.191024 | 0.9485911 | 0.4942048 |
| -0.434147 | -0.944511 | -0.351868 | -0.161609 | -0.052204 | -0.699551 | -0.133733 | -0.426725 | -1.382783 |
| 0.0788792 | -0.064349 | 1.0520490 | -0.187340 | 0.0403910 | 0.8204419 | -0.056846 | 0.0860284 | 0.8000589 |
| -0.690615 | -1.442980 | -1.030198 | -0.011553 | -0.384189 | -1.193473 | -0.217948 | -0.648829 | -1.767432 |
| -0.517576 | -1.143547 | -0.768201 | -0.666675 | -0.512855 | -1.098825 | -0.598907 | -0.942554 | -1.404399 |
| -0.591182 | -0.137509 | 0.5508716 | -0.102860 | 0.0358273 | -0.287465 | -0.331655 | -0.206335 | -0.452393 |
| -0.422592 | -0.533092 | -0.912756 | -0.294183 | -0.295415 | -0.395724 | -0.416590 | -0.268983 | -0.280014 |
| -0.747383 | -0.936137 | -1.291370 | -0.297137 | -0.367392 | -0.702164 | -0.111700 | -0.452306 | -1.735056 |
| -0.195422 | -0.420819 | -0.675517 | -0.029323 | -0.166461 | -0.403369 | -0.065030 | -0.489735 | -0.735202 |
| -0.983195 | -0.340311 | -0.695533 | -0.418091 | -1.123323 | -1.302031 | -1.015890 | -0.893369 | -0.668683 |
| 0.0009985 | -0.243184 | 0.9176838 | -0.415996 | -0.181101 | -0.141841 | -0.120154 | 0.0334964 | 0.0081589 |
| -0.746611 | -0.796627 | -1.564557 | -0.474097 | -0.953923 | -1.903101 | -0.707025 | -1.486191 | -1.920265 |

| | | | | | | | | |
|---|---|---|---|---|---|---|---|---|
| -0.233749 | 0.7159581 | 1.0926509 | -0.222685 | 0.1133081 | 0.2502229 | -0.067053 | 0.2776837 | 0.9919515 |
| -0.136291 | -0.362779 | 0.1583589 | -0.530399 | -0.563185 | 0.2187454 | -0.126177 | -0.388606 | 0.9900433 |
| -0.334034 | -0.105752 | 0.2880972 | -0.163201 | -0.169142 | 0.0163998 | -0.225321 | -0.134535 | -0.042721 |

| PG_345 | PG_346 | PG_347 | PG_348 | PG_349 | PG_350 | PG_351 | PG_352 | PG_353 |
|---|---|---|---|---|---|---|---|---|
| -0.717466 | -0.736865 | -1.202567 | -0.666404 | -0.785101 | -1.233335 | -0.812016 | -1.018354 | -2.403511 |
| -0.555489 | -0.752246 | -1.203534 | -0.642612 | -0.740412 | -1.402679 | -0.557403 | -0.199227 | -1.129431 |
| -0.861757 | -1.049229 | -1.903468 | -0.890414 | -1.536096 | -2.267119 | -0.536327 | -0.675713 | -0.400409 |
| -0.316241 | -0.163458 | -0.376561 | -0.473574 | -0.738276 | -0.421437 | -0.043726 | 0.3002358 | 0.3172015 |
| -0.271329 | -0.155448 | -0.719160 | -0.179485 | -0.114961 | -0.483215 | -0.054057 | -0.168316 | -1.052493 |
| -0.322837 | -0.898135 | -1.467475 | -0.299829 | -0.979397 | -2.210469 | -0.278989 | -0.522071 | -1.936225 |
| -0.393283 | -1.024256 | -0.381702 | -0.437433 | -1.184736 | -0.335445 | -0.264365 | -0.256664 | -0.616831 |
| -0.096582 | -0.396748 | -2.083361 | -0.122196 | -0.459192 | -2.500320 | 0.2061533 | 0.1529797 | -1.796550 |
| -0.630091 | -0.516660 | -0.073176 | -0.710283 | -0.598733 | 0.2338708 | -0.673835 | -0.412956 | -0.590099 |
| -0.818896 | -1.803945 | -1.292215 | -1.060541 | -1.917956 | -1.332915 | -0.614008 | -0.490504 | -0.915710 |
| -0.380331 | -0.515711 | -0.496772 | -0.419910 | -0.729559 | -0.787476 | -0.160551 | -0.221308 | -0.474253 |
| 0.0886617 | -0.463567 | -0.377597 | 0.0959081 | -0.717670 | -0.700674 | -0.112575 | -0.221707 | -0.119290 |
| 0.6177746 | 0.0959062 | -1.820086 | 0.7445982 | -0.549474 | -3.804783 | -0.551396 | -0.514698 | -0.371111 |
| 0.0612572 | 3.2850552 | 2.9830767 | 0.2533963 | 4.7161725 | 4.3044102 | -0.306294 | -0.658205 | 4.0342325 |
| 0.1146671 | -0.573175 | -0.516722 | 0.1476319 | -0.590987 | -0.160629 | -0.326956 | -0.077606 | 0.0608778 |
| -0.219449 | 2.6375765 | 2.6418098 | -0.192370 | 3.2032922 | 3.1835386 | 0.1144200 | -0.350296 | 3.0277620 |
| -1.404747 | -2.498283 | -2.161807 | -1.238476 | -2.315942 | -2.524210 | -1.502400 | -1.593895 | -1.579925 |
| -0.305649 | -1.778914 | -0.498372 | -0.671090 | -1.819802 | -0.656571 | -0.263821 | -0.317421 | -0.129487 |
| -0.222668 | -0.334778 | -0.095201 | 0.0540517 | 0.2496807 | 0.1397004 | 0.0332989 | -0.328623 | 0.0588066 |
| -0.573714 | -1.563340 | -0.681931 | -1.153617 | -3.210825 | -1.305157 | -0.633982 | -0.579569 | -2.392514 |
| -0.068028 | 0.3876231 | 0.4689622 | -0.160029 | 0.6275709 | 0.3526033 | -0.464580 | -0.501188 | -0.093640 |
| 0.3883435 | -1.189368 | -2.580057 | 0.0769632 | -1.631271 | -2.512457 | 0.0562388 | -0.541760 | -0.328279 |
| -0.163306 | 0.9512257 | 2.0591766 | -0.225784 | 1.0549602 | 1.8497642 | -0.422161 | -0.336148 | 1.0270095 |
| 0.1140022 | -0.464269 | -1.106259 | 0.0392375 | -1.041112 | -1.801454 | -0.106341 | -0.400795 | -1.431929 |
| -0.439725 | -1.242473 | -1.452931 | -0.729313 | -1.821753 | -2.291767 | -0.311272 | -0.185817 | -1.670947 |
| -0.047258 | 0.5148592 | 1.0199552 | 0.0001677 | 0.6035647 | 1.0708482 | -0.222369 | -0.488049 | 0.1429915 |
| 0.0634196 | 0.0651239 | -0.452552 | 0.0457232 | -0.072202 | -0.389047 | 0.2379905 | 0.1142254 | -0.142091 |
| -1.216422 | -0.777435 | -1.529608 | -1.046560 | -1.365910 | -1.611430 | -1.555072 | -0.740109 | -1.786807 |
| -0.072431 | -1.065163 | -1.749073 | -0.213639 | -1.530280 | -2.693065 | -0.496937 | -0.850115 | -0.306734 |
| -0.343474 | -0.139321 | -0.369550 | -0.241866 | -0.756978 | -0.377859 | -0.135945 | 0.0065669 | -0.187390 |
| -0.161736 | -0.710250 | -1.090342 | -0.229251 | -1.328509 | -1.371434 | 0.2234802 | 0.1329907 | 2.0209080 |
| 0.4651485 | 0.1195857 | -0.946944 | 0.0832834 | -0.029244 | -0.896426 | -0.030187 | -0.461794 | 0.2912666 |
| -0.198931 | -0.540008 | -1.961538 | 0.1493772 | -0.388398 | -2.264673 | -0.363480 | -0.539110 | 0.6516495 |
| 0.7481621 | -0.181968 | -2.762991 | 0.6813701 | -0.430638 | -4.664352 | -0.480588 | -0.714831 | -0.156035 |
| -0.066023 | -0.055369 | -0.072029 | -0.018547 | -0.055915 | -0.046745 | -0.064715 | -0.067763 | -0.161833 |
| -0.334860 | -0.117966 | -0.033759 | -0.644535 | -0.264303 | 0.1359177 | -0.346957 | -0.242183 | -0.000659 |
| 0.9664068 | 0.6542769 | -1.789484 | 0.5847542 | -0.289909 | -2.498509 | -0.092240 | -0.242281 | -0.118441 |
| -0.226189 | 4.0271029 | 1.6468767 | 0.7205205 | 4.3543432 | 1.6488655 | 0.0070903 | 0.0476292 | 2.1911845 |
| -0.465317 | -0.795854 | -0.019530 | -0.459734 | -0.178428 | -0.014394 | -0.449858 | -0.892189 | 0.0492297 |
| -0.636946 | -1.583911 | 0.3953092 | -1.412544 | -0.911385 | 0.0274520 | -0.132280 | -0.204127 | -0.130836 |
| -0.187899 | 0.0555858 | 0.3571201 | -0.083596 | 0.2371004 | 0.7820685 | -0.099114 | -0.425141 | 0.0877470 |
| 0.2907301 | -0.521180 | -1.149112 | 0.1968467 | -0.559862 | -1.592996 | -0.431306 | -0.636850 | -1.936709 |
| -0.460020 | -0.586877 | -0.851201 | -0.423385 | -0.905863 | -0.964454 | -0.679971 | -0.126271 | -0.408167 |
| 0.2551691 | -0.107313 | -0.256369 | 0.0878505 | -0.089447 | -0.381114 | 0.0031716 | -0.178818 | -1.098106 |
| -0.277354 | 0.7072713 | 1.5769504 | 0.0231859 | 0.8508614 | 1.9842057 | -0.664530 | -0.294434 | 0.3345287 |
| 0.8572026 | 0.8154265 | 3.2352338 | 0.9217904 | 1.2442943 | 4.5102263 | -0.371606 | -0.432391 | 0.1296189 |
| -0.500220 | -1.105345 | 0.0702492 | -0.622050 | -0.907896 | 0.0348509 | 0.0712482 | 0.0312894 | 0.4734400 |

| | | | | | | | | |
|---|---|---|---|---|---|---|---|---|
| -0.206799 | 0.0367835 | -0.048234 | -0.165472 | 0.0616194 | 0.1802105 | -0.353802 | -0.266201 | -0.358832 |
| -1.288299 | -1.366445 | -2.641503 | -1.222154 | -1.609744 | -2.947675 | -0.577218 | -0.527134 | -2.604128 |
| -0.559268 | 0.1330584 | 0.2287886 | -0.417614 | 0.2862949 | 0.4038757 | -0.473066 | -0.679309 | -0.467942 |
| -0.589919 | -0.142525 | 0.3770228 | -1.250005 | -0.283024 | 0.3190415 | 0.1662079 | -0.840852 | -0.120824 |
| -0.174720 | -0.024716 | -0.235164 | -0.348776 | -0.046799 | 0.0342760 | -0.059542 | -0.271678 | -1.565363 |
| -0.131468 | 0.2160095 | -0.013447 | 0.1245350 | -0.159833 | 0.3383508 | -0.150629 | -0.166652 | 0.1169700 |
| -0.061536 | -0.568155 | -1.775157 | -0.106548 | -1.172164 | -2.544887 | -0.229574 | 0.0705591 | -0.635086 |
| -0.147675 | 2.2358382 | 2.2368573 | -0.088518 | 2.5902779 | 2.7509216 | -0.373910 | -0.270334 | 3.2431488 |
| -0.644963 | 1.1449818 | -0.374147 | 0.2714821 | 0.9023196 | -0.699812 | -0.687276 | -0.605212 | 2.1372315 |
| -1.064005 | -0.105441 | -0.083374 | -1.044414 | -0.476855 | -0.695169 | -0.571904 | -0.617191 | -0.922248 |
| -0.963588 | -0.851630 | -1.284589 | -0.626652 | -1.552284 | -1.546228 | -0.488665 | -0.257949 | -1.531896 |
| -0.068941 | 2.7607160 | 2.3663605 | 0.2863210 | 3.1537208 | 2.8649028 | 0.2584549 | -0.432989 | 2.1167236 |
| -0.211701 | 1.9624924 | 1.4504610 | -0.267943 | 2.0747624 | 1.7182198 | 0.0505348 | -0.286637 | 3.1807957 |
| -0.947145 | -1.020057 | -1.291762 | -0.851213 | -1.438548 | -1.237180 | -0.391704 | -0.435509 | -0.619292 |
| -0.765843 | -1.813818 | -1.552217 | -1.005949 | -2.125916 | -2.574369 | -0.582062 | -0.908639 | -2.428701 |
| -0.672873 | -0.852041 | -0.320240 | -0.620182 | -0.923428 | -0.241518 | -0.351339 | -0.625070 | -1.563768 |
| -0.456758 | 0.0238619 | -1.120965 | -0.335863 | -0.886307 | -1.431965 | -0.133929 | 0.0445819 | -1.359755 |
| -0.197111 | -0.405169 | 0.2834865 | -0.250049 | -0.651400 | -0.425844 | -0.301166 | -0.540262 | -0.736121 |
| -0.259455 | -1.348804 | -0.907798 | -0.325290 | -1.076077 | -1.423938 | -0.390114 | -0.861165 | -1.767873 |
| -0.066455 | -0.208494 | -0.906479 | -0.066220 | -0.344056 | -0.920329 | 0.1235939 | 0.1782856 | -1.378134 |
| -0.200900 | -0.175860 | 0.0442517 | -0.297776 | -0.184934 | -0.005746 | -0.719287 | -0.498083 | 0.3999099 |
| 0.1750902 | -0.676387 | -2.458741 | 0.0682295 | -1.214683 | -2.852031 | -0.767820 | -0.701135 | -0.725925 |
| -0.112664 | -0.685822 | 0.5649508 | -0.439431 | 0.0481955 | 0.6606747 | 0.3593945 | -0.257369 | 0.2734880 |
| 0.2528893 | 1.0798447 | 0.7423015 | 0.3691573 | 0.6682505 | 0.7818223 | 0.5589112 | 0.5282851 | 1.2967537 |
| -1.451648 | -1.238826 | -1.855791 | -0.986444 | -1.232868 | -2.663988 | -0.993770 | -0.729603 | -2.581611 |
| -0.224302 | -0.618249 | -0.530207 | -0.401304 | -0.462655 | -0.175584 | 0.1018195 | 0.1927002 | -0.479073 |
| -0.332938 | -1.365984 | -1.461115 | -0.604931 | -1.490309 | -1.595061 | -0.461561 | -0.581798 | -2.227493 |
| 0.8107379 | 2.3114122 | 3.6206642 | 1.0644430 | 2.4772860 | 4.1741104 | -0.105153 | -0.032398 | 4.2727100 |
| -0.341478 | 0.6277877 | 1.5164446 | -0.334919 | 0.7517009 | 2.2571795 | -0.327601 | -0.069607 | 1.7233084 |
| -0.371341 | -0.974605 | -2.001659 | -0.653832 | -1.113247 | -2.101297 | -0.103091 | -0.230269 | -1.156088 |
| -0.106956 | -0.152469 | -0.275577 | 0.4202587 | -0.050784 | -0.004668 | -0.147040 | -0.117205 | 0.0274948 |
| -0.210674 | -0.494380 | -0.079826 | -0.172962 | -0.591998 | -0.076021 | -0.153309 | -0.232060 | -0.811621 |
| -0.201894 | -0.831937 | -0.495253 | -0.249070 | -0.890150 | -0.723625 | -0.609394 | -0.690048 | -1.565034 |
| -0.103851 | -0.299875 | -0.668259 | -0.305774 | -0.852448 | -1.023068 | -0.387671 | -0.286409 | -1.964617 |
| -0.082068 | 1.0008098 | 1.4639059 | 0.1840568 | 1.3790676 | 1.4716307 | -0.384028 | -0.546419 | 1.7046068 |
| 0.2236963 | 0.0936944 | -1.009045 | 0.0781047 | -0.341429 | -1.494908 | -0.094188 | -0.053740 | 0.1692530 |
| -1.137443 | -1.465945 | -2.638313 | -1.061938 | -1.792682 | -2.348809 | -0.077748 | -0.568394 | -2.228176 |
| -0.310439 | -0.830634 | -1.030664 | -0.446811 | -0.961733 | -1.312713 | -0.198103 | -0.472820 | -2.149361 |
| 0.2954209 | -0.723622 | -0.637111 | 0.1219212 | -0.755957 | -1.298268 | -0.189233 | -0.812983 | -1.248757 |
| -0.341706 | -1.408987 | -1.512287 | -0.436677 | -1.425516 | -1.862935 | -0.525649 | -0.582429 | -1.623973 |
| -0.707524 | -0.784139 | -1.087454 | -0.877492 | -0.732369 | -1.107396 | -0.798358 | -0.723165 | -1.230365 |
| -0.590803 | -0.674287 | -0.671481 | -0.694436 | -0.930804 | 0.0488646 | 0.3106715 | -0.270477 | -4.129060 |
| 0.2933308 | 1.7379996 | 1.4583874 | 1.2312897 | 2.7974520 | 1.2957096 | -0.005310 | 0.3645572 | 1.5807490 |
| -0.061278 | 0.2032050 | -0.490424 | -0.090212 | -0.192403 | -0.533676 | -0.242724 | -0.047851 | -0.369141 |
| 0.0885674 | -0.443927 | -0.871671 | -0.154707 | -0.431493 | -0.808442 | -0.064355 | -0.143945 | -0.687239 |
| -0.680645 | -1.402033 | -1.445326 | -0.841570 | -1.718647 | -1.242775 | -1.139547 | -0.874597 | -1.697747 |
| 0.1593581 | 5.3417723 | 5.7071614 | 0.0225716 | 6.6797263 | 7.7529224 | -0.075962 | -0.067675 | 3.8308989 |
| -0.061669 | 2.5664198 | 1.8009051 | 0.0804649 | 2.9601536 | 2.2608603 | 0.2579669 | -0.007976 | 1.2380641 |
| -0.200804 | -0.239484 | -1.561584 | -0.219723 | -0.211912 | -0.911698 | -0.605522 | 0.0378455 | -4.187570 |
| -0.253612 | -0.287078 | 0.2259087 | -0.225727 | -0.370365 | -0.160220 | 0.1198142 | 0.1120363 | -0.401657 |

| | | | | | | | | |
|---|---|---|---|---|---|---|---|---|
| -0.048840 | -0.761733 | -2.869363 | -0.137489 | -1.140178 | -2.556463 | -0.102424 | -0.105069 | -1.844682 |
| -0.159347 | -0.097021 | 0.4406306 | -0.437839 | 0.0570185 | 0.6354387 | -0.411179 | -0.609542 | 0.2050943 |
| -0.224977 | -0.905854 | 0.2391373 | -0.671795 | -1.177734 | -0.625385 | -0.376155 | -0.604606 | -0.432668 |
| 0.4033259 | 0.5225432 | 0.036223 | 0.4152261 | 0.5057639 | 0.0468081 | -0.202980 | -0.374176 | 0.2039742 |
| -0.250600 | -1.235728 | -1.038765 | -0.451161 | -1.502958 | -1.391568 | -0.234331 | -0.301155 | -1.441279 |
| -0.573066 | -1.042968 | -1.105210 | -0.741365 | -0.745554 | -0.680130 | -0.008500 | 0.3750646 | 0.8690151 |
| -0.600904 | 0.3568726 | -0.165137 | -0.658512 | -0.117472 | -0.201520 | -0.474463 | -0.001274 | -0.213212 |
| -0.095630 | -0.076569 | 0.2753082 | -0.052591 | 0.0022815 | 0.1902706 | 0.2026709 | -0.241799 | 0.4168617 |
| -0.123080 | -0.168405 | -0.306680 | -0.029466 | -0.141412 | -0.041501 | -0.290210 | -0.506017 | -0.290618 |
| -0.495814 | 0.3530059 | 0.4668059 | -0.057313 | 0.6015199 | 0.7290598 | -0.235211 | -0.215351 | 0.7373651 |
| -0.644440 | -0.863708 | -1.366477 | -0.492378 | -1.004146 | -1.280729 | -0.003669 | -0.060078 | -0.113347 |
| 0.4699431 | 0.1914398 | -2.105653 | 0.1053306 | -0.256805 | -3.226312 | -0.369187 | -0.087661 | 0.2770327 |
| 1.2388924 | 0.5018670 | -2.618653 | 0.7127541 | -0.156971 | -5.014209 | -0.095339 | -0.432483 | -0.161987 |
| 0.5628064 | 0.3193754 | -2.037241 | 0.3991264 | -0.370928 | -2.956847 | -0.227003 | -0.056425 | -0.638612 |
| -0.167823 | -0.365716 | -0.541812 | -0.322816 | -0.402381 | -0.855998 | -0.090501 | -0.222672 | -0.054623 |
| 0.1048604 | -0.125114 | 0.0528886 | -0.101350 | 0.0231795 | -0.141620 | 0.0091747 | 0.0188713 | 0.2085490 |
| -0.749346 | -0.471017 | -0.589336 | -0.816124 | -0.699001 | -0.808080 | -0.839683 | -0.758125 | -0.986526 |
| -0.195624 | -0.129990 | -0.144570 | -0.199514 | 0.0557738 | 0.0981177 | -0.110664 | -0.083061 | -0.091292 |
| -0.428079 | -0.825601 | -0.890113 | -0.590860 | -1.067306 | -0.658775 | -0.489844 | 0.0024323 | 0.2583810 |
| -0.692041 | -0.345395 | 1.3084927 | -0.434232 | 0.3554484 | 1.3984564 | -0.304511 | -0.806887 | 1.1668359 |
| -0.393729 | -0.887150 | -0.813612 | -0.341532 | -0.703604 | -1.243567 | -0.195584 | -0.439204 | -1.095902 |
| -0.347083 | -0.839150 | -0.450762 | -0.475671 | -0.825392 | -0.485064 | -0.254317 | -0.376581 | 0.1708073 |
| -0.326639 | 0.0128502 | -0.052261 | -0.286765 | 0.0649959 | 0.0823257 | -0.006681 | -0.089588 | -0.199720 |
| -0.391195 | -1.062828 | -1.613853 | -0.376316 | -0.888316 | -1.680736 | -0.223838 | -0.582329 | -1.938336 |
| -0.962006 | -1.224379 | -2.414752 | -1.061671 | -1.234058 | -2.169611 | -0.432842 | 0.0110836 | -0.365099 |
| -0.604501 | -0.584111 | -0.046962 | -0.478022 | -0.727119 | -0.337964 | -0.377427 | -0.366215 | -0.744371 |
| 0.0114447 | -0.344266 | 0.1455320 | 0.0270339 | -0.433665 | 0.1367793 | 0.1313774 | -0.026524 | -0.034354 |
| -0.239735 | -0.139567 | 0.3352006 | -0.428812 | 0.3222437 | 0.4053038 | -0.506114 | -0.711982 | 0.0526787 |
| -0.400419 | -1.269246 | -1.914234 | -0.628418 | -1.979047 | -2.101473 | -0.060555 | -0.069929 | -0.149377 |
| -0.102003 | -0.244330 | -2.370656 | 0.1052965 | -0.529408 | -3.111579 | -0.510771 | -0.521984 | -0.999123 |
| -0.580111 | -1.874497 | -2.940709 | -0.667047 | -1.956546 | -2.921718 | -0.500290 | -0.414860 | -1.276501 |
| -0.277062 | -0.491621 | -0.341125 | -0.329670 | -0.639787 | -0.604175 | 0.1439507 | -0.457370 | -0.840633 |
| -0.360692 | -0.604824 | -0.542959 | -0.568305 | -0.742058 | -0.528940 | -0.171669 | 0.0108973 | 0.5127383 |
| 0.0788589 | -0.298731 | 0.0437318 | 0.2202387 | -0.058138 | -0.187885 | 0.2048016 | -0.140956 | -0.425434 |
| -0.188664 | -0.846421 | -1.045548 | -0.234452 | -0.875536 | -1.544485 | -0.474959 | -0.560081 | -1.842051 |
| -0.175810 | -0.004940 | -0.289324 | -0.533913 | -0.494022 | -0.631050 | -0.451793 | -0.272977 | -0.889708 |
| -0.276228 | -0.374870 | -0.075127 | -0.357837 | -0.508326 | -0.164598 | -0.419958 | -0.415559 | -1.109911 |
| 0.1965532 | -0.124982 | -1.389902 | 0.2359710 | -0.144407 | -1.482396 | -0.385550 | -0.528173 | -0.189536 |
| -0.771478 | -0.567136 | -0.201799 | -0.705697 | -0.676487 | -0.578364 | -0.655643 | -0.444689 | -0.820053 |
| 0.0748054 | -1.347044 | -2.507650 | 0.0274879 | -1.211620 | -2.849029 | -0.826134 | -1.594240 | -1.631123 |
| -0.489950 | -0.716694 | 0.6116709 | -0.737043 | -0.724361 | 0.3649370 | -0.691539 | -0.227721 | -0.294611 |
| 0.4137183 | 0.1261489 | -1.743514 | 0.1943355 | 0.3655702 | -2.341199 | -0.169556 | -0.266021 | -0.258346 |
| 0.0804722 | 0.0236759 | -1.003943 | 0.1814199 | -0.284921 | -1.526195 | -0.131541 | -0.047864 | -0.596061 |
| -0.080789 | 0.0609919 | -0.804519 | -0.080204 | -0.382784 | -1.332706 | -0.011483 | -0.113072 | -1.239618 |
| -0.112292 | -0.292544 | -0.032132 | -0.037307 | -0.158764 | -0.075568 | -0.168721 | -0.251677 | -0.119411 |
| -0.298154 | 0.6154945 | -0.105092 | -0.123456 | 0.3992654 | -0.009524 | -0.359410 | 0.2386340 | 0.5660644 |
| -0.513728 | -0.784767 | -0.877492 | -0.506928 | -0.581570 | -0.910269 | -0.295122 | -0.188939 | -1.290624 |
| -0.719939 | 0.4065237 | 0.3380746 | -0.344489 | 0.5264227 | 0.7609442 | -0.327465 | -0.262257 | 0.6954154 |
| 0.2270789 | -1.108284 | -2.681020 | 0.3232124 | -1.631929 | -2.910812 | -0.345660 | -0.694880 | -0.358675 |
| -1.084719 | -0.913704 | -1.213920 | -1.150912 | -1.037657 | -1.539319 | -1.479860 | -0.958086 | -2.605508 |

| | | | | | | | | |
|---|---|---|---|---|---|---|---|---|
| 0.1352347 | -0.032380 | 0.0963826 | 0.2236427 | 0.0773281 | 0.3019331 | 0.3670440 | 0.1690407 | -0.201847 |
| -0.650584 | -1.604119 | -2.343501 | -0.988616 | -1.986905 | -2.958075 | -1.086762 | -0.940564 | -2.529630 |
| -0.478557 | -0.467293 | -0.522297 | -0.576885 | -0.260474 | -0.349465 | -0.365119 | -0.168762 | -0.276803 |
| -0.160080 | 0.8562776 | 0.2322669 | -0.022065 | 2.1507425 | 0.4728622 | -0.419862 | -0.276292 | 0.4698715 |
| -0.158276 | -0.974400 | -1.769929 | -0.092064 | -0.999561 | -1.930406 | -0.227508 | -0.398988 | -1.203321 |
| -0.259836 | 0.9790639 | 1.2624057 | 0.1992402 | 1.6069725 | 1.1672028 | -0.239432 | -0.384728 | 1.7285379 |
| -0.441239 | -0.713230 | -0.462131 | -0.487217 | -0.552650 | -0.578933 | -0.314646 | -0.298930 | -0.752762 |
| -0.217058 | -0.228957 | 0.5296426 | -0.286750 | -0.155082 | -0.147172 | -0.327372 | -0.471242 | -1.042372 |
| -0.204099 | -1.150062 | -1.943187 | -0.386817 | -1.291666 | -2.216965 | -0.352092 | -0.790449 | -1.575785 |
| -0.691073 | -1.050131 | -0.346439 | -0.492418 | -0.611720 | -0.004086 | -0.693714 | -0.534150 | 0.1300866 |
| 0.0272677 | 0.0679124 | 0.3003471 | 0.1024910 | 0.3959644 | 0.4463443 | -0.002794 | -0.287205 | 0.5052376 |
| -0.775955 | -1.339044 | -1.276623 | -1.127327 | -1.557316 | -1.249240 | -0.757992 | -0.617826 | -1.328160 |
| -0.205910 | -1.019291 | -1.448861 | -0.255515 | -1.272278 | -1.254837 | 0.5320179 | 0.3058445 | -2.374949 |
| -0.821979 | -0.990123 | -1.023028 | -0.723697 | -0.958069 | -0.498179 | -0.140222 | -0.045288 | -1.399379 |
| 0.0177797 | -0.075548 | -0.157875 | -0.143080 | -0.182205 | -0.197702 | -0.268586 | -0.288152 | -0.438277 |
| 0.7753509 | 0.0924331 | -1.913018 | 0.7812096 | 0.0038411 | -1.972045 | -0.639195 | -0.721099 | -1.011188 |
| -0.127940 | -0.645338 | -0.165071 | -0.422275 | -0.283087 | -0.103780 | -0.664179 | -0.765238 | -0.648297 |
| -0.316605 | -0.873139 | -0.070391 | -0.539479 | -1.233756 | -0.571927 | -0.372206 | -0.389088 | -1.851206 |
| -0.608483 | -0.870337 | 0.2083708 | -0.913305 | -1.058320 | -0.205095 | -0.664864 | -0.341568 | -0.233707 |
| -0.478994 | -0.664893 | -1.030365 | -0.567080 | -1.022936 | -1.103707 | -0.456490 | -0.232526 | -0.881620 |
| -0.094962 | 0.2673844 | 0.5689418 | -0.283786 | 0.1832776 | 0.9046805 | -0.384688 | 0.0745412 | 0.3247888 |
| -0.397028 | 0.2805197 | 0.0790942 | -0.275813 | 0.4391705 | 0.2771959 | -0.324225 | -0.004654 | -0.202323 |
| -0.239584 | -0.824035 | -1.178068 | -0.293502 | -1.294472 | -1.445030 | -0.696857 | -0.551837 | -1.409744 |
| -0.344154 | -0.474010 | -0.642266 | -0.548650 | -0.872096 | -0.874603 | -0.476463 | -0.577822 | -0.938013 |
| -0.329725 | -1.111850 | -1.285907 | -0.506697 | -1.396536 | -1.854155 | -0.123849 | -0.321996 | -1.557372 |
| -0.329545 | -0.416239 | -0.152921 | -0.447246 | -0.837817 | -0.592195 | -0.369645 | -0.450787 | 0.7491120 |
| -0.790447 | -0.964662 | -0.514707 | -0.675381 | -1.044991 | -0.728209 | -0.948815 | -0.935992 | 0.5052969 |
| 0.0273972 | -0.698995 | -1.076305 | -0.336234 | -0.804594 | -0.823358 | -0.113354 | 0.0839056 | -0.482882 |
| -0.391997 | -1.345737 | -0.590904 | -0.659043 | -1.214456 | -1.397822 | -0.483466 | -0.946428 | -1.321805 |
| -0.155593 | 1.3097804 | 1.1660286 | 0.0409646 | 0.9891805 | 1.3348781 | -0.452294 | -0.048712 | 0.3829881 |
| -0.191424 | 1.0908781 | 1.8633394 | 0.1625403 | 1.9898978 | 3.1607439 | -0.388435 | -0.284886 | 2.5565269 |
| -0.453971 | -0.866325 | -1.365186 | -0.690148 | -1.176031 | -1.162303 | -1.022051 | -0.320984 | 0.0083022 |
| -0.296983 | -0.090698 | 0.3772296 | -0.260492 | 0.0360196 | 0.2874717 | -0.159523 | -0.386238 | 0.1361665 |
| 0.0790718 | 0.2650827 | 0.9058008 | 0.1555364 | 0.5508029 | 0.7924688 | 0.0089464 | -0.285357 | 0.7170516 |
| 0.1335506 | 0.6893540 | 1.0533753 | 0.3455804 | 0.9265889 | 0.9109082 | 0.1782628 | 0.0175858 | 0.7403302 |
| -0.636657 | -0.792122 | -0.900644 | -0.606156 | -0.921188 | -0.786671 | -0.186015 | -0.042710 | -1.193483 |
| 0.0616362 | 0.7069029 | 0.3552517 | 0.0251037 | 0.8895983 | 0.6673796 | -0.022720 | 0.3523327 | 0.8805245 |
| -0.668239 | 0.2152376 | -0.044769 | -0.384068 | 0.5370775 | 0.2386149 | -0.126571 | -0.288606 | -0.274973 |
| -0.099523 | 0.0942887 | -0.369037 | -0.066062 | -0.120898 | -0.280239 | -0.631875 | -0.383790 | 0.2782300 |
| -0.467328 | -0.251116 | -0.835351 | -0.464948 | -0.508717 | -0.948388 | -0.667108 | -0.219643 | 0.4700070 |
| -0.780175 | -1.640432 | -1.111018 | -0.699819 | -1.269853 | -1.758406 | -0.223809 | -0.232419 | -2.092701 |
| -1.134293 | 0.0306914 | 0.5026616 | -0.988165 | 0.2673036 | 0.8668695 | -0.796819 | -0.433663 | 0.6680290 |
| -0.127478 | 0.4017432 | 1.8459252 | 0.0990278 | 0.9017396 | 1.8163186 | -0.367912 | -0.528518 | 2.5490522 |
| -0.140430 | 0.5596310 | 0.6757641 | -0.159915 | 0.1751527 | 0.7121070 | -0.341385 | -0.055714 | 0.0234011 |
| -0.338496 | -0.754154 | -0.521513 | -0.375723 | -0.687883 | -0.472274 | -0.189576 | -0.114475 | -0.281036 |
| -0.305897 | -0.069776 | -0.136122 | -0.345030 | -0.055833 | 0.0642857 | -0.450940 | -0.131500 | -0.615807 |
| -0.345690 | -0.881168 | -0.447885 | -0.346085 | -0.920227 | -0.748059 | -0.381031 | -0.411631 | -0.943727 |
| -0.438492 | -1.249658 | -1.314773 | -0.487364 | -1.298423 | -1.423077 | -0.528760 | -0.776586 | -1.712893 |
| 0.1456658 | -0.332124 | -0.775213 | 0.0963166 | -0.665744 | -0.999526 | 0.1645379 | -0.043460 | -0.176192 |
| -0.381853 | -0.405643 | -0.105169 | -0.286884 | -0.460524 | -0.386374 | -0.323328 | -0.282258 | -0.295182 |

| | | | | | | | | |
|---|---|---|---|---|---|---|---|---|
| -0.3120559 | -0.0336526 | 0.3218449 | -0.1477847 | -0.3852243 | 0.7223519 | -0.2599748 | -0.0437670 | 0.8850319 |
| -0.4233842 | -0.6349720 | -1.0186738 | -0.4061025 | -0.4612849 | -1.0104844 | -0.0506409 | -0.2248049 | -0.8324055 |
| 0.0829731 | 0.1983315 | -1.7583154 | 0.1774931 | 0.0452708 | -2.8462205 | -0.8818480 | -0.3713713 | -0.4343127 |
| 0.3722076 | -0.1342695 | -0.6041037 | 0.2355934 | -0.8582352 | -0.7141308 | -0.6625676 | -0.7184666 | -0.3233085 |
| -0.4303392 | -0.8083712 | -1.0083714 | -0.5033498 | -1.0979684 | -1.4568277 | -0.4340197 | -0.2095145 | -0.9746297 |
| -0.0197956 | 0.4718543 | 0.5312430 | -0.0452927 | 0.3427222 | 0.2271084 | -0.5340797 | -0.4285866 | -0.1007384 |
| -0.6675876 | 1.1617193 | 2.4537099 | -0.3565150 | 1.2373626 | 2.0043403 | -0.2881186 | -0.0769699 | 1.9475053 |
| -0.0856661 | 0.0981987 | 0.0125858 | -0.1814712 | 0.1016520 | -0.1094425 | -0.7306412 | -0.3760171 | -0.5409225 |
| -0.4189232 | -0.2826286 | -1.5430742 | -0.3649046 | -1.2780132 | -1.6500537 | -0.5524533 | 0.2802822 | -1.2044309 |
| -0.1720902 | -0.8683478 | -0.5216712 | -0.4870428 | -0.3602505 | -0.1781249 | -0.2191815 | -0.5470428 | 0.2913269 |
| -0.2321803 | -0.1459112 | -0.2839615 | -0.1361922 | -0.0378646 | 0.0181461 | -0.3329677 | -0.4332297 | 0.0296660 |
| -0.8553924 | -0.8119220 | -0.5889197 | -1.3152227 | -0.8977339 | -0.7126928 | -0.7529813 | -0.6923336 | -1.1095913 |
| 0.0772805 | -0.1693570 | 0.1043112 | 0.0714161 | 0.0867992 | 0.0807524 | 0.1422162 | -0.1672648 | 0.1140580 |
| -0.3577287 | -0.9529772 | -0.4297813 | -0.3344677 | -0.6752382 | -0.5115325 | 0.3039698 | -0.2894851 | 0.2529179 |
| 0.0406985 | -0.5658925 | -1.6608341 | -0.1790522 | -1.0349165 | -2.2411397 | -0.0133686 | -0.2478281 | -1.8842797 |
| -0.2496010 | -0.7184170 | -0.9175491 | -0.2623205 | -0.6694870 | -0.6934509 | -0.1724835 | -0.2394521 | -0.5195134 |
| -0.2332469 | -0.3692495 | -0.8886273 | -0.1285070 | -0.6402431 | -1.5022766 | -0.1595063 | -0.1776336 | -0.5497890 |
| -0.0913112 | -0.6548412 | -1.1789057 | -0.0954807 | -0.4761989 | -1.4615696 | -0.3059899 | -0.2937775 | -0.9028883 |
| -0.1920557 | -1.0382944 | -1.3741230 | -0.2044900 | -1.7485315 | -1.6755425 | 0.0955028 | -0.0652801 | -1.1200859 |
| -0.2248341 | 0.5538012 | 0.0880931 | -0.0222254 | -0.1479859 | 0.2669709 | -0.3192557 | 0.4215929 | 0.4303373 |
| -0.6746939 | -0.6402317 | -0.9473708 | -1.0125936 | -0.8085156 | -0.7752986 | -0.9707670 | -0.8232845 | -1.0741184 |
| 0.2962256 | -0.2589750 | -0.4320449 | 0.0902959 | -0.4306909 | -0.3804367 | -0.3350094 | -0.2513119 | -0.4052929 |
| -0.6679453 | -1.5556526 | -3.3208845 | -0.6724945 | -2.0861447 | -3.7681608 | -0.4495036 | -0.6007168 | -4.1350859 |
| 0.0156505 | 0.1249298 | 0.4095301 | 0.1220819 | 0.2050008 | 0.4158573 | -0.2734324 | -0.2368634 | -0.2143288 |
| -0.4019832 | -0.2472530 | -0.4371929 | -0.1792422 | -0.4302117 | -0.5643053 | -0.2730454 | -0.2578009 | -0.1673091 |
| -0.3131265 | -0.4567212 | -0.3502856 | -0.4950103 | -0.4187135 | -0.2757149 | -0.6492240 | -0.5669396 | -1.5879758 |
| -0.0209578 | -0.2098760 | -1.9120737 | -0.0964118 | -0.5797381 | -2.0240966 | -0.1109993 | 0.0780764 | -0.9293421 |
| -0.4979925 | -0.5037572 | -0.7650928 | -0.4293826 | -0.5412097 | -0.7442965 | -0.4991378 | -0.4471814 | -0.5152113 |
| -0.3646554 | 0.3416358 | 0.4469017 | -0.3848313 | 0.1122109 | 0.8261883 | -0.1018989 | 0.1011388 | 0.6457827 |
| -0.2995096 | -0.3505512 | 0.8535470 | -0.3942350 | -0.0061451 | 1.0413884 | -0.0207881 | -0.2488814 | 1.5163631 |
| -0.4103324 | -0.3017043 | -0.3966102 | -0.7288326 | -0.5698069 | -0.8029930 | -0.5219004 | -0.6202976 | -1.2512211 |
| 0.0499707 | -0.2206454 | -0.4396580 | 0.0556754 | -0.1956540 | -0.6370244 | -0.0434663 | -0.1497149 | 0.0732743 |
| -0.9119384 | -1.2816861 | 0.1503044 | -1.0263203 | -1.0683434 | -0.1175751 | -0.6471309 | -0.3776013 | 1.8975639 |
| -0.6557128 | -0.5737937 | -0.9276221 | -0.5175864 | -0.9955166 | -1.2050512 | -0.2761823 | -0.1737358 | -1.3263446 |
| -0.1637223 | -0.5063843 | -0.5207623 | -0.0564071 | -0.1102452 | -0.4565643 | -0.2329413 | -0.5714747 | -0.5515593 |
| -0.6701139 | -1.0631544 | -0.7331367 | -0.6313742 | -1.1302927 | -1.0756003 | -0.6794968 | -0.9066729 | -0.8852407 |
| -0.3000199 | -0.8213848 | -1.7211333 | -0.4961853 | -1.5718372 | -2.3672527 | -0.9233238 | -1.1380467 | -1.8351278 |
| -0.1404564 | -0.3237962 | -0.1101809 | -0.2192591 | -0.1731066 | -0.2000768 | -0.0681278 | -0.2001621 | -0.2150294 |
| -0.4064410 | 0.1629498 | 1.3830415 | -0.4477575 | 0.7070699 | 0.9815193 | -0.2335336 | -0.8460838 | 0.4513309 |
| -0.1547490 | -0.8296397 | 0.2185621 | -0.0917738 | -0.5888208 | -0.3046306 | -0.1999326 | -0.4406162 | -1.3388509 |
| -0.2876117 | 0.1003098 | 0.4167907 | -0.2155406 | 0.1155753 | 0.8883341 | -0.2771508 | -0.1998844 | 0.9774854 |
| -0.2852935 | -1.2050664 | -1.9294191 | -0.3732043 | -1.5267442 | -2.0831759 | -0.3111988 | -0.1685524 | -0.6237691 |
| -0.3664659 | -0.9697820 | -0.4002602 | -0.5520536 | -0.7130771 | -0.7504413 | -0.5264117 | -0.4341168 | -0.6127280 |
| -0.2052520 | -0.6904317 | -0.6431197 | -0.5117632 | -0.9820984 | -0.8381063 | -0.1855700 | -0.1016627 | -0.4016196 |
| -0.6844348 | -0.4226149 | -0.4544498 | -0.7512984 | -0.1843387 | -0.2866570 | -0.7341324 | -0.6329820 | 0.1064573 |
| -0.3290820 | -0.9342334 | -0.9734286 | -0.5649127 | -1.0567024 | -0.8657204 | -0.3799782 | -0.6246507 | -0.9687736 |
| -0.5862032 | -0.6850902 | -0.3143459 | -0.1470293 | -0.5581670 | -0.2772950 | -0.1807277 | -0.3713377 | -0.3535433 |
| -0.5193878 | -0.6248598 | -1.0493687 | -0.6084666 | -1.0079852 | -1.2541133 | -1.4177795 | -0.5374255 | -0.5022351 |
| -0.2607152 | 0.0518050 | -0.6021519 | -0.0602430 | -0.5210657 | -0.3231800 | -0.5110196 | -0.4663742 | 0.0772974 |
| -0.8369550 | -1.0798351 | -1.3268614 | -0.8500769 | -1.0250674 | -1.4110060 | -0.8398511 | -0.8493378 | -2.0850892 |

| | | | | | | | | |
|---|---|---|---|---|---|---|---|---|
| -0.2612050 | -0.4003301 | -0.7562817 | -0.2837250 | -0.1023116 | -0.1579706 | -0.7507649 | -0.4137038 | 0.3899295 |
| -0.6081044 | -0.6123547 | -0.1775736 | -0.2641743 | -0.0345983 | 0.1469723 | -0.9229493 | -0.4622084 | -0.9430068 |
| -0.1940513 | -0.4506074 | 0.2716458 | -0.2323534 | 0.2358784 | 0.2353350 | -0.5743506 | -0.4947338 | 0.2626043 |

| PG_354 | PG_355 | PG_356 | PG_357 | PG_358 | PG_359 | PG_360 | PG_361 | PG_362 |
|---|---|---|---|---|---|---|---|---|
| -0.644062 | -1.080962 | -3.127780 | -0.168098 | -0.326362 | -2.269610 | -0.416757 | -0.678304 | -2.319723 |
| -0.521477 | -0.854114 | -1.460140 | -0.583040 | -0.302209 | -1.118347 | -0.485620 | -0.318255 | -1.374452 |
| -0.820343 | -0.954958 | -0.199151 | -0.310213 | -0.623201 | -1.278909 | -0.640416 | -0.682870 | -1.392884 |
| -0.015183 | -0.372906 | -0.561370 | -1.281186 | 0.0171225 | -0.311135 | -0.181626 | -0.252913 | -0.894294 |
| 0.0767187 | -0.106941 | -1.636128 | -0.660911 | 0.0098069 | -0.489977 | -0.099170 | -0.068740 | -0.242872 |
| -0.306519 | -1.093026 | -2.521396 | -0.465769 | -0.042466 | -2.012231 | -0.325345 | -0.275549 | -1.845903 |
| -0.292535 | -0.643899 | -0.826590 | -0.494346 | -0.048236 | -0.186074 | -0.034421 | -0.626981 | -0.025961 |
| 0.2413115 | -0.050441 | -2.094573 | -0.330610 | 0.0380119 | -0.873106 | 0.0052657 | 0.0689422 | -1.355738 |
| -0.480528 | -0.166076 | -1.191637 | -0.326091 | -0.244929 | -1.062855 | -0.262555 | -0.003175 | -1.870751 |
| -0.684311 | -1.347963 | -0.733783 | -0.386580 | -0.418451 | -0.726128 | -0.349696 | -0.592467 | -0.495063 |
| -0.132972 | -0.395850 | -0.830534 | -0.494945 | -0.129213 | -0.693283 | -0.098533 | -0.291998 | -0.691141 |
| -0.143329 | -0.407159 | -0.261471 | -0.525356 | -0.195376 | -0.455213 | -0.179862 | -0.702359 | -0.576602 |
| -0.677524 | -1.647040 | -2.739254 | -0.661734 | -0.755963 | -1.540520 | -0.547775 | -0.981493 | -1.545788 |
| 0.2195892 | 4.6111463 | 4.5925963 | -0.167318 | 0.7959330 | 3.6569204 | 0.8442744 | 3.0569823 | 3.5738896 |
| -0.171851 | -0.084214 | 0.1510060 | -0.774036 | -0.301485 | -0.238237 | -0.149418 | -0.291309 | -0.193519 |
| -0.175971 | 3.3675085 | 3.1512981 | 0.0447110 | 0.6069394 | 1.3185193 | 0.2837026 | 2.2592066 | 1.9806124 |
| -1.845354 | -2.758213 | -1.825964 | -0.772323 | -1.247165 | -1.834539 | -1.067777 | -1.917485 | -1.797642 |
| -0.055560 | -0.116523 | 0.3660271 | -0.678339 | -1.134048 | -2.668084 | -0.799282 | -1.028252 | -2.838238 |
| -0.444172 | -0.289032 | 0.4443447 | -0.344666 | -0.571839 | -0.619011 | -0.712472 | -0.685572 | -0.570052 |
| -0.592796 | -2.026430 | -2.859217 | -1.079946 | -1.096123 | -0.568326 | -0.598568 | -0.643192 | -0.815687 |
| -0.487795 | 0.1058084 | -0.195337 | -0.698720 | -0.379952 | 0.2757059 | -0.379067 | -0.118656 | 0.0457051 |
| -0.155968 | -0.473914 | 0.2900852 | -0.279352 | -0.224696 | -0.508138 | -0.414202 | -0.584553 | -0.514220 |
| -0.362359 | 0.7460352 | 0.5492730 | 0.1967286 | 0.0504826 | 1.5610859 | -0.071555 | 0.6015258 | 1.0875574 |
| -0.175162 | -1.153737 | -2.562065 | -0.459535 | -0.104868 | -2.134192 | -0.421972 | -1.954875 | -2.104150 |
| -0.462682 | -1.411400 | -2.223388 | -0.358661 | -0.690142 | -2.146744 | -0.779054 | -1.401574 | -1.949962 |
| -0.359482 | 0.1598589 | -0.025653 | 0.0159769 | -0.030900 | 0.8672951 | -0.051422 | 0.4001624 | 0.6581475 |
| 0.2927537 | -0.097835 | -0.273527 | -0.911454 | 0.3432585 | -0.418346 | 0.1540400 | 0.1108877 | -0.389676 |
| -1.407806 | -1.586957 | -2.699669 | -1.152947 | -1.109321 | -1.356145 | -0.797647 | -1.122901 | -1.859749 |
| -0.891415 | -1.162882 | -0.177403 | -0.349272 | 0.0206533 | -1.816649 | -0.225109 | -0.760684 | -1.619277 |
| -0.146136 | -0.602805 | -0.441711 | -0.825147 | 0.1346814 | -0.528180 | 0.0446157 | -0.677282 | -0.902854 |
| 0.1430143 | 0.0238026 | 1.3601327 | -0.153032 | -0.157686 | -1.482423 | -0.196015 | -0.346414 | -1.181751 |
| 0.2052399 | -0.193268 | 0.4750533 | -0.369585 | 0.1523984 | 0.1130685 | -0.118181 | -0.593939 | -0.008051 |
| -0.443173 | 0.5801192 | 0.1361987 | -0.534558 | 0.0701857 | 0.1437010 | -0.059465 | 0.7802086 | 0.3821046 |
| -0.525372 | -0.453976 | -1.221655 | -0.803167 | -0.269241 | -3.176323 | -0.407590 | -1.056844 | -2.848689 |
| -0.105683 | -0.071725 | 0.2852708 | -0.241892 | -0.298389 | -0.176504 | -0.306675 | -0.316406 | -0.113237 |
| -0.626677 | -1.622107 | -0.912819 | -0.479563 | -1.154381 | -0.335636 | -1.042817 | -1.080827 | -0.912551 |
| -0.342979 | -1.311488 | -1.584182 | -0.545071 | -0.196319 | -1.886541 | -0.106858 | -0.472580 | -1.464889 |
| 0.0035182 | 4.1705504 | 1.4969316 | 0.3656723 | 1.3020867 | 1.4261993 | 1.3354446 | 2.6649830 | 0.7221674 |
| -0.249745 | -0.537288 | 0.4167293 | -0.844048 | 0.0631029 | -0.708335 | -0.497060 | -0.101382 | -0.345619 |
| -0.446932 | 0.0587100 | -0.186252 | -0.586901 | -0.373109 | 0.3484632 | -0.784581 | -1.068793 | -0.209399 |
| -0.404200 | -0.050678 | -0.930426 | -0.041159 | -0.168936 | -0.219297 | -0.057880 | 0.3191759 | 0.0381404 |
| -0.415829 | -1.086369 | -2.056256 | -0.087220 | -0.759242 | -1.540397 | -0.776813 | -1.097770 | -1.481037 |
| -0.354620 | -0.159184 | -0.093289 | -0.610891 | -0.147291 | -0.581628 | -0.338309 | -0.347759 | -0.505920 |
| -0.198299 | -0.392112 | -1.744854 | -0.694791 | 0.3641815 | -1.102625 | 0.1268679 | -0.227512 | -0.950651 |
| -0.296100 | 0.2779813 | 0.7111534 | 0.2447929 | -0.248463 | 0.2327578 | 0.0689322 | 0.3066176 | 0.7864132 |
| -0.186813 | -0.443996 | 0.8408626 | -0.159043 | -0.379856 | 0.1801441 | -0.234236 | -0.233933 | 0.9306781 |
| -0.186193 | 0.3152104 | 0.7430786 | 0.0368275 | -0.074272 | -0.259318 | -0.059724 | -0.027211 | -0.012252 |

| | | | | | | | | |
|---|---|---|---|---|---|---|---|---|
| -0.334627 | -0.409942 | -1.515358 | -0.939443 | -0.312251 | 0.0170716 | -0.278112 | -0.171552 | -0.189380 |
| -0.612634 | -0.771683 | -3.985281 | -0.888025 | -0.565110 | -2.593302 | -0.734999 | -0.897539 | -2.221134 |
| -0.524032 | -0.035470 | -0.326600 | -0.639629 | -0.367209 | -0.317787 | -0.570778 | 0.1105918 | -0.295220 |
| -0.432092 | 1.3442045 | 0.3776485 | -0.353572 | -0.505251 | 0.4856200 | -0.549873 | -0.143174 | 1.1719998 |
| -0.228588 | -0.724744 | -1.517238 | -0.555944 | -0.693973 | -1.307862 | -0.691333 | -1.123240 | -1.109585 |
| -0.309911 | 0.0597524 | 0.2904457 | -0.012858 | -0.281243 | 0.1618136 | -0.383544 | -0.333393 | 0.3228776 |
| -0.111236 | -1.313469 | -1.769579 | -0.117346 | -0.334853 | -1.556614 | -0.245238 | -0.934030 | -1.370252 |
| -0.075652 | 2.7180695 | 3.1298461 | -0.285518 | 0.1686974 | 3.3417317 | 0.5873436 | 2.1686424 | 3.3697976 |
| -0.481791 | 3.5323111 | 1.0996598 | 0.0752132 | 1.4342979 | 2.7875533 | 1.7606182 | 3.3119540 | 2.2263075 |
| -0.617546 | -0.941027 | -1.006644 | -0.995687 | -0.978538 | -0.650747 | -0.936664 | -0.697965 | -0.629707 |
| -0.881708 | -1.973553 | -1.979167 | -0.731669 | -0.732623 | -1.915832 | -0.757429 | -1.378746 | -1.970957 |
| 0.0746712 | 2.7016131 | 2.2927157 | -0.343088 | 1.1219404 | 1.7387094 | 1.3124047 | 2.5684650 | 2.3683982 |
| -0.144535 | 3.3991025 | 2.9061418 | -1.027767 | 0.4901415 | 1.1215397 | 0.4431043 | 2.0246331 | 0.8313964 |
| -0.448019 | -0.353280 | -0.829943 | -0.388917 | -0.697045 | -1.724573 | -0.626984 | -0.595969 | -1.747360 |
| -0.799507 | -2.655539 | -3.752573 | -1.147941 | -0.821449 | -2.309410 | -0.757405 | -1.734521 | -2.093767 |
| -0.320395 | -1.655059 | -2.097641 | -0.465879 | -0.531114 | -1.771282 | -0.485107 | -1.165298 | -2.086572 |
| -0.144509 | -0.718741 | -1.803299 | -0.627213 | -0.278663 | -2.429376 | -0.296304 | -1.311238 | -2.152135 |
| -0.344526 | -0.765521 | -1.027597 | -0.551902 | -0.168528 | -0.234654 | -0.280616 | -0.741389 | -0.508283 |
| -0.380423 | -1.218853 | -2.600377 | -0.441743 | -0.141348 | -0.877798 | -0.121683 | -0.738415 | -0.816173 |
| 0.1876435 | -0.416079 | -1.964918 | -1.187888 | 0.2084034 | -0.825356 | -0.011861 | 0.2330391 | -0.788103 |
| -0.545687 | -0.451484 | -0.145881 | -0.233754 | -0.180301 | -0.459236 | -0.344944 | -0.129882 | -0.423072 |
| -0.552630 | -1.197748 | -1.584091 | -0.436117 | -0.639949 | -1.985562 | -0.602431 | -1.149999 | -2.024823 |
| 0.2974211 | 0.2048874 | -0.405182 | 0.4835752 | -0.014965 | -0.276536 | -0.321195 | -0.059000 | -0.201296 |
| 0.4632791 | 0.4889500 | 0.4939019 | -1.421680 | 0.7657790 | 0.5394179 | 0.6049959 | 0.1823825 | -0.020361 |
| -0.900692 | -1.488423 | -2.819138 | -0.852688 | -0.534482 | -2.763958 | -0.579479 | -0.980733 | -2.114116 |
| 0.0946427 | -0.783579 | -1.321534 | -0.226501 | -0.468576 | -0.344764 | -0.218574 | -0.905054 | -0.321718 |
| -0.256676 | -1.332311 | -1.761673 | -0.449564 | -0.579106 | -1.849538 | -0.878741 | -1.543172 | -1.548244 |
| -0.033592 | 1.9102968 | 3.8296135 | 0.2074778 | 0.3624979 | 3.8161229 | 0.8540300 | 1.8031905 | 3.5245235 |
| -0.152292 | 2.3862980 | 2.2073451 | 0.1066599 | 0.4928340 | 3.0813054 | 0.6271892 | 1.7459047 | 3.0715258 |
| -0.118441 | -0.400324 | -1.687923 | -0.397171 | 0.0975512 | -2.232947 | -0.213440 | -0.757404 | -2.206699 |
| 0.0902024 | -0.099251 | -0.116273 | -0.160547 | -0.224979 | -0.017479 | -0.234380 | -0.552660 | 0.3619140 |
| -0.058816 | -0.584253 | -1.287535 | -1.180932 | 0.0380643 | -0.197013 | -0.008963 | -0.434733 | -0.535030 |
| -0.544106 | -1.658498 | -1.598291 | -0.613317 | -0.727524 | -1.344301 | -0.680085 | -1.665690 | -1.372939 |
| -0.294266 | -1.055435 | -2.764316 | -0.753788 | -0.272932 | -0.852464 | -0.285873 | -0.611774 | -1.258008 |
| -0.312479 | 1.1704726 | 1.3538433 | -0.322069 | -0.081349 | 1.5256685 | 0.0150463 | 0.9010235 | 1.4035096 |
| 0.3194247 | -0.239745 | 0.3988944 | 0.0731459 | -0.251281 | -0.107948 | -0.190038 | -0.284043 | -0.245293 |
| -0.600232 | -1.033745 | -2.331413 | -0.494228 | -0.325455 | -1.471128 | -0.174942 | -0.391765 | -1.117633 |
| -0.250996 | -1.546169 | -2.666477 | -0.333461 | -0.176398 | -1.819931 | -0.229018 | -1.056028 | -1.674859 |
| -0.482277 | -0.659569 | -1.543808 | -0.500336 | -0.419889 | -1.287479 | -0.209887 | -0.954472 | -1.407166 |
| -0.513093 | -1.262403 | -1.689232 | -0.426395 | -0.642885 | -1.797029 | -0.848246 | -1.419822 | -1.713550 |
| -0.770890 | -1.505869 | -2.143270 | -0.388448 | -0.738742 | -1.204864 | -0.631514 | -0.725416 | -1.554377 |
| 0.0801043 | -1.964210 | -4.260454 | -0.199390 | -0.183327 | -2.554359 | -0.064143 | -1.641913 | -2.231277 |
| 0.4565940 | 2.2047714 | 0.8162382 | 0.4435350 | 1.0333687 | 1.6775606 | 0.8547140 | 3.0449919 | 0.6384309 |
| -0.152009 | -0.272515 | -0.423393 | -0.340126 | -0.190611 | -0.376140 | -0.028104 | -0.219838 | -0.286199 |
| -0.059394 | -0.378849 | -0.621344 | -0.400734 | 0.1522223 | -0.980641 | -0.032313 | -0.120335 | -0.740635 |
| -0.937688 | -1.452715 | -1.462692 | -0.523859 | -0.832203 | -1.930584 | -1.042990 | -2.138865 | -1.9009463 |
| -0.121822 | 5.2330678 | 5.5809597 | -0.046103 | -0.022175 | 0.0765836 | -0.158630 | 1.3783374 | 1.2090130 |
| 0.2120165 | 1.5102040 | 1.2738876 | -0.180092 | 0.6785989 | 0.5726233 | 0.2526310 | 1.2094653 | 1.2950365 |
| -0.662199 | -1.008909 | -4.048001 | -1.232354 | -0.122310 | -2.499227 | -0.521580 | -0.616087 | -2.9686498 |
| 0.1478064 | 0.1618279 | -0.972756 | -0.275505 | -0.448370 | 0.1780937 | -0.184216 | 0.1162952 | -0.211634 |

| | | | | | | | | |
|---|---|---|---|---|---|---|---|---|
| -0.1162407 | -0.6131385 | -1.9293828 | -0.5519052 | -0.4762797 | -0.4998127 | -0.6407548 | -0.5167473 | -0.1553286 |
| -0.3514285 | -0.4677408 | 0.3631844 | -0.4094581 | -0.2723009 | 0.3611539 | -0.2468673 | -0.4432454 | 0.1349171 |
| -0.3575660 | -1.1491668 | -1.1229902 | -0.7300703 | -0.4819537 | -0.2538147 | -0.5509015 | -1.0983105 | -0.5171292 |
| -0.2281338 | -0.1181158 | -0.2584522 | -0.3608823 | 0.0357438 | -0.0117083 | -0.0191992 | -0.0391286 | 0.1317180 |
| -0.1840982 | -1.2612985 | -1.7632737 | -0.3864683 | -0.2991399 | -1.6894630 | -0.4984535 | -1.6407289 | -1.8614832 |
| 0.1516769 | 0.7270525 | 0.5942212 | -0.7563346 | -0.9634738 | -0.0670932 | -0.5984619 | -0.6057331 | -0.2921332 |
| -0.5301175 | -0.4074401 | -1.0429040 | -1.5792562 | -0.2344852 | -0.2188195 | -0.2768937 | 0.0925359 | -0.5810203 |
| -0.1418450 | 0.2049322 | 0.8333600 | 0.4150912 | 0.0213571 | 0.6168147 | 0.0560502 | 0.4951444 | 0.2970170 |
| -0.4098015 | 0.0238342 | 0.3158030 | 0.3114031 | -0.0334887 | -0.3487932 | -0.0798118 | -0.1016562 | -0.1910700 |
| -0.1083679 | 0.9508366 | 0.5269678 | -0.1491211 | 0.0898759 | 0.2404125 | 0.2077087 | 0.5971301 | 0.2268172 |
| -0.0101487 | -0.2845957 | -0.6274238 | -0.5883108 | -0.0173877 | -1.3841876 | -0.0500525 | -0.6666027 | -0.9206800 |
| -0.5209530 | -1.4243685 | -2.5104208 | -1.0931394 | -0.4988038 | -1.7042673 | -0.2520282 | -0.5759565 | -2.0071984 |
| -0.1458729 | -1.1438551 | -2.3729218 | -0.3288244 | -0.1572064 | -2.6522031 | -0.3703000 | -0.7873118 | -1.8938537 |
| -0.3115452 | -0.8755603 | -2.5007723 | -0.4576519 | -0.3683755 | -1.4384081 | -0.1269292 | -0.2434472 | -1.1640435 |
| -0.1572179 | -0.4205295 | -0.6070924 | -0.3808321 | -0.2854903 | -0.6989801 | -0.2775826 | -0.8873254 | -0.9674599 |
| -0.2668906 | -0.1980395 | -0.0332502 | -0.1435378 | -0.0877822 | 0.0262015 | -0.1084097 | -0.0230859 | -0.1246551 |
| -0.6982532 | -1.5483934 | -1.3950172 | -0.5303050 | -0.9955039 | -0.6395641 | -0.9606033 | -1.3563467 | -0.7092087 |
| 0.1800602 | -0.1400162 | 0.5792095 | 0.2930944 | -0.1083375 | 1.1340281 | -0.3609472 | -0.3341159 | 3.1475984 |
| -0.5716331 | -1.0964354 | -0.4231397 | -0.7555019 | -0.6683000 | -0.9909028 | -0.4868715 | -0.9488987 | -1.2659005 |
| -0.8136414 | -0.6691825 | 1.1676100 | -0.2395126 | -0.6408490 | 0.9571722 | -0.2850284 | 0.0752975 | 0.8610947 |
| -0.1662487 | -0.4400191 | -1.4802975 | -0.6263830 | -0.3993945 | -1.1356621 | -0.4235901 | -0.6263677 | -1.3799884 |
| -0.4301476 | -0.5337071 | -0.0239854 | -0.4581440 | -0.4294331 | 0.0088050 | -0.5500327 | -0.5910745 | -0.3139734 |
| -0.0071504 | 0.0881178 | 0.3334855 | -0.3342345 | -0.0340421 | 0.0260756 | -0.2137080 | -0.1872806 | -0.2425677 |
| -0.3202785 | -0.9101901 | -1.8532079 | 0.0152719 | -0.1249519 | -2.0452678 | -0.2705793 | -0.7566794 | -1.8251499 |
| -0.3372361 | 0.1749519 | 0.1050466 | -0.6296967 | -0.3008867 | -0.1526135 | -0.5186913 | 0.3718696 | -0.0792411 |
| -0.5646796 | -0.9450793 | -0.8989241 | -0.6591641 | -0.4255023 | -0.8168556 | -0.0961639 | -0.9204378 | -1.0286989 |
| 0.1241435 | -0.2302766 | -0.0020717 | -0.7596230 | 0.2598609 | -0.3159733 | 0.0372871 | -0.4560157 | -0.4879182 |
| -0.4885443 | -0.2270443 | 0.5163406 | 0.0187646 | -0.4241545 | 0.0593347 | -0.2596115 | -0.1786115 | -0.3376229 |
| -0.1232598 | -1.9138920 | -0.7866419 | -0.6436255 | -0.3775018 | -0.7861567 | -0.3833325 | -1.1525688 | -1.2881506 |
| -0.3394494 | -0.0580496 | -1.2552692 | -0.7711839 | -0.3737972 | -0.9966998 | -0.1883350 | -0.0752702 | -0.6659831 |
| -0.8384510 | -1.5170318 | -2.1421716 | -0.5897827 | -0.4138401 | -1.5296096 | -1.0117420 | -0.9466842 | -0.8250576 |
| -0.1317992 | -1.2553949 | -2.1059165 | -0.1831643 | -0.0891832 | -2.0684242 | -0.1201858 | -1.0280431 | -2.6555559 |
| -0.2833210 | 0.1870421 | -0.0215589 | -0.3728373 | -0.8297454 | -0.2162960 | -0.8180118 | -0.6638421 | -0.1582441 |
| 0.2270976 | -0.0971737 | -0.7307594 | -0.7638718 | 0.7067422 | 0.0034799 | 0.2848453 | 0.4401498 | -0.1457149 |
| -0.3795794 | -0.7104201 | -2.7776500 | -0.8751052 | -0.0900626 | -1.1853308 | -0.2363480 | -0.5206170 | -1.1343066 |
| -0.4580265 | -1.1465315 | -1.1038295 | -0.5973592 | -0.5495493 | -0.9843351 | -0.3232247 | -1.3527598 | -1.2790893 |
| -0.4113927 | -0.6488258 | -2.0024995 | -0.1495027 | -0.0682064 | -0.6952834 | -0.1594196 | -0.3451079 | -0.6449330 |
| -0.3101342 | -0.4393866 | -0.1909206 | -0.6987994 | -0.2321167 | -0.8325789 | -0.1852525 | -0.2200524 | -0.6928390 |
| -0.5446728 | -0.8411713 | -1.0550414 | -0.4389400 | -0.4157407 | -1.2790888 | -0.4140578 | -0.5184409 | -1.1930228 |
| -1.1671115 | -1.0688921 | -1.7496479 | -0.4818663 | -0.5093749 | -1.7004130 | -0.4095485 | -0.5713625 | -0.9851254 |
| -0.3790587 | -0.7420325 | -0.3988962 | -0.3316525 | -0.1944575 | 0.1535770 | 0.1300263 | -0.0586539 | -0.2518566 |
| -0.1309673 | 0.0298988 | -0.9349250 | -0.3407592 | -0.0071838 | -0.6274403 | -0.1879641 | 0.1954738 | -0.3361715 |
| -0.0620466 | -0.2436451 | -1.7897872 | -0.6440657 | 0.1957708 | -0.9003327 | 0.0343876 | -0.3447738 | -0.6201958 |
| 0.0882346 | -0.7295058 | -2.5210643 | -0.5287944 | -0.4994829 | -2.0800602 | -0.3954236 | -0.7738367 | -2.3391477 |
| -0.1883077 | -0.1351789 | 0.0597568 | -0.1284223 | -0.2899287 | 0.0008961 | -0.3718452 | -0.2604642 | -0.0823469 |
| -0.4783624 | 1.0323029 | 0.4556830 | -0.3744770 | -0.2090686 | -0.2607933 | -0.1486038 | 0.0971472 | -0.4932129 |
| -0.1543462 | -0.6571066 | -1.2775497 | -0.5720729 | -0.2845883 | -1.1466122 | -0.4230802 | -0.7240456 | -0.9683619 |
| -0.4125512 | -0.1917304 | 0.2081031 | -1.0143872 | -0.4010005 | 0.4911946 | -0.2856511 | 0.1134103 | 0.5007607 |
| -0.6013289 | -0.9360323 | -0.3986254 | -0.6779052 | -0.2807564 | -1.5279163 | -0.6717193 | -1.0083951 | -1.3318022 |
| -1.3701809 | -1.1158500 | -2.4538681 | -1.3874156 | -0.8842132 | -1.5276493 | -0.9732323 | -1.0772829 | -1.8899277 |

| | | | | | | | | |
|---|---|---|---|---|---|---|---|---|
| 0.0881343 | 0.162088 | -0.5894131 | -0.9229343 | 0.6448360 | 0.3174652 | 0.5701662 | 0.2335194 | 0.3472580 |
| -1.0334327 | -2.2557005 | -2.3843321 | -1.4339746 | -1.2250868 | -1.9866882 | -1.0134920 | -1.4926985 | -2.0556035 |
| -0.1561094 | -0.2621608 | -0.3530648 | -0.8694296 | -0.3370339 | -0.2388271 | -0.4800465 | -0.5181837 | -0.3614964 |
| 0.0060251 | -0.0484942 | 0.2183788 | -0.4847413 | -0.4882632 | -0.0465752 | -0.4742973 | -0.0960983 | -0.0463432 |
| -0.3989037 | -1.0391099 | -0.9313952 | -0.5682779 | -0.0226663 | -1.7511507 | -0.3784491 | -1.1781371 | -1.2080564 |
| -0.1048043 | 2.2097126 | 1.0704786 | 0.2078675 | 0.8803337 | 1.6515905 | 1.3161044 | 2.2505766 | 1.3502784 |
| -0.2831173 | -0.4692666 | -0.8864745 | -0.7635360 | -0.2350076 | -0.6942517 | -0.2305316 | -0.4085110 | -0.8072799 |
| -0.5207723 | -1.4457841 | -1.7211708 | -0.5514336 | -0.6269455 | -1.1576268 | -0.7301421 | -1.4135039 | -1.5919375 |
| -0.3076299 | -1.4860280 | -1.7388587 | -0.3029219 | -0.2794136 | -1.9784386 | -0.3131862 | -1.6452608 | -1.9942087 |
| -0.4647335 | -0.4881878 | -0.1198171 | -0.8675311 | -0.6561763 | -0.4866480 | -0.6845674 | -0.6627703 | -0.5130681 |
| -0.1394074 | 0.3873778 | 0.4651682 | -0.1518911 | 0.0999046 | 0.5901138 | -0.0511349 | 0.1985380 | 0.4689560 |
| -0.6717440 | -0.9086671 | -2.2534995 | -0.4794338 | -0.8632096 | -1.2298959 | -0.4779822 | -0.8791678 | -1.6265974 |
| 0.4224733 | -0.8470287 | -2.2764096 | -0.3948057 | -0.4363546 | -1.3831037 | -0.5716868 | -1.1088147 | -1.2898123 |
| -0.5745490 | -0.9420060 | -1.4467687 | -0.5194545 | -0.6536840 | -0.8739412 | -0.2915007 | -0.3898773 | -0.9130323 |
| -0.2753717 | -0.2633493 | -0.0690850 | -0.1236605 | -0.3142218 | -0.1552162 | -0.1758418 | -0.1731441 | -0.0854433 |
| -0.5114361 | -0.3382561 | -0.2784874 | -0.3189347 | -0.3783810 | -2.0298213 | -0.3595889 | -0.2186052 | -1.7119867 |
| -0.8134611 | -0.4923815 | -1.0666225 | 0.5774335 | -0.2254514 | -0.4442069 | -0.3590006 | -0.3171731 | -0.5166452 |
| -0.7093997 | -1.9711031 | -3.1458229 | -0.7555692 | -0.6138768 | -1.2791985 | -0.7059879 | -2.2130178 | -2.2110571 |
| -0.5065846 | -0.7063497 | -0.5001960 | -0.6117392 | -0.8067487 | -0.5619209 | -1.0793612 | -1.1749265 | -0.6431386 |
| -0.3542118 | -0.8731988 | -1.6559784 | -0.8629790 | -0.3799605 | -0.9491884 | -0.2337587 | -0.8175656 | -1.0244041 |
| -0.2654806 | 0.0260361 | 0.7098447 | -0.2983176 | -0.1528289 | 0.2476156 | -0.0259098 | 0.7422211 | 0.6569762 |
| -0.2935652 | -0.2887227 | -1.1196052 | -1.0064274 | -0.0078604 | -0.0082463 | 0.2954163 | -0.1304249 | -0.0705165 |
| -0.5138118 | -1.3672122 | -1.7731680 | -0.1534414 | -0.5063396 | -2.1530543 | -0.4000079 | -1.4433883 | -2.0634726 |
| -0.3283453 | -0.7311537 | -1.6805561 | -0.8313898 | -0.0437079 | -0.4367093 | -0.4419852 | -0.2203799 | -0.2716917 |
| -0.2173578 | -1.2394871 | -1.8030141 | -0.2012229 | 0.1055498 | -1.5989757 | -0.3028878 | -0.5656895 | -1.5322486 |
| -0.2987421 | -1.1580557 | 0.1111766 | -0.8750092 | -0.4614732 | -0.1656595 | -0.4974368 | -0.7040187 | -0.2447515 |
| -1.4655087 | -1.4446196 | 0.8220100 | -0.6713450 | -0.8796278 | -2.5973654 | -0.8759972 | -1.2216278 | -2.4153591 |
| -0.2079360 | -0.3992379 | -0.4070308 | -0.0882754 | -0.1557252 | -0.0116441 | -0.2155340 | -0.5739626 | 0.2203631 |
| -1.0154664 | -2.0694043 | -2.0414485 | -0.9395484 | -0.4930758 | -1.5165836 | -0.6707139 | -1.4038003 | -1.8649689 |
| -0.3838711 | 0.2106987 | 0.0697713 | -0.2555390 | 0.0315394 | 0.4991657 | 0.2216139 | 0.6201779 | 0.7847079 |
| -0.0086929 | 2.7330275 | 3.1324699 | 0.4947140 | 0.7506818 | 3.0137098 | 0.4677055 | 2.4923519 | 3.3268272 |
| -1.1164448 | -0.5652356 | -0.8657676 | -0.6866515 | -0.9238403 | -1.0578089 | -0.6810805 | -1.5971638 | -1.3013342 |
| -0.0801862 | -0.3812405 | -0.0403887 | -0.5234919 | 0.1737422 | 0.1424579 | 0.1002401 | -0.0066858 | 0.1359306 |
| 0.1261326 | 0.3389436 | 0.5972705 | -0.5742105 | 0.1325035 | 0.2940127 | 0.0447868 | 0.2128562 | 0.3073221 |
| 0.1621874 | 0.8041479 | 0.8381655 | 0.6048417 | 0.2360980 | 0.3994093 | 0.1494751 | 0.5169656 | 0.2741211 |
| -0.2249603 | -0.4785356 | -1.6470237 | -1.2773462 | -0.0288054 | -1.1155544 | -0.1562297 | -0.6476228 | -1.1677373 |
| -0.2309302 | 0.5528168 | 0.6095003 | -0.5647577 | -0.3413864 | 1.1331334 | 0.5146494 | 0.6146035 | 1.4452529 |
| -0.2336492 | 0.3365807 | -0.2654947 | -0.3697883 | 0.0583868 | 0.4522365 | -0.1504745 | 0.3834656 | 0.6689766 |
| -0.5648333 | -0.2000141 | -0.3484688 | -0.3947455 | -0.3926338 | 0.3238637 | -0.0411361 | -0.1050362 | 0.4880737 |
| -0.7178760 | -0.9238508 | 0.1141704 | -0.8728375 | -0.2435960 | -0.9670919 | -0.2919028 | -0.3317625 | -1.2924553 |
| -0.1567290 | -2.0746720 | -2.8508630 | -0.4823096 | -0.8179965 | -0.3317093 | -0.4890967 | -0.3380253 | -0.4242655 |
| -1.1488050 | 0.3299932 | 0.5033886 | -0.6392364 | -0.1521469 | 0.4184672 | -0.2662900 | -0.0998538 | 0.4259209 |
| -0.4045405 | 0.5223486 | 2.0693887 | -0.6499824 | -0.2620653 | 0.3366822 | 0.3625214 | 0.7081736 | 1.7919023 |
| -0.2391568 | 0.1561214 | 0.1184282 | -0.3640874 | -0.0739283 | 0.2189532 | -0.0658062 | -0.1410553 | 0.1643390 |
| -0.1642948 | -0.5590446 | -0.3460090 | -0.1142917 | -0.2220815 | -0.4405365 | -0.2872636 | -0.7019889 | -0.3282719 |
| -0.3642412 | -0.5513773 | -0.9098447 | -0.7958484 | -0.6073438 | -0.0046955 | -0.3189609 | -0.3716692 | -0.1751759 |
| -0.3778740 | -0.9149639 | -1.5546794 | -0.3526630 | -0.4277788 | -1.8502894 | -0.6322795 | -1.3471817 | -2.5401796 |
| -0.6030548 | -1.3035702 | -1.8049871 | -0.5697235 | -0.4866583 | -1.9010848 | -0.7812097 | -1.0761144 | -1.6007532 |
| 0.2615153 | -0.6321143 | -0.1181681 | -0.0805429 | -0.0915272 | -1.9541966 | -0.2663500 | -1.3153633 | -1.8836350 |
| -0.2917526 | -0.3119588 | -0.4711293 | -1.1393258 | -0.1904893 | -0.2718675 | -0.2068687 | -0.3192072 | -0.4426168 |

| | | | | | | | | |
|---|---|---|---|---|---|---|---|---|
| -0.106662 | -0.733214 | -0.102749 | -1.646398 | -0.364174 | -0.243423 | 0.0359181 | -0.648645 | -1.024759 |
| -0.081126 | -0.396468 | -1.031246 | -0.430385 | -0.384584 | -0.860065 | -0.305245 | -0.077707 | -0.349207 |
| -0.966773 | -0.303050 | -2.002579 | -1.148104 | -0.411404 | -0.710852 | -0.450038 | 0.1793501 | -0.524393 |
| -0.508519 | -1.054808 | -1.096006 | -0.737761 | -0.803683 | -2.398765 | -1.055769 | -1.099518 | -2.445982 |
| -0.528515 | -1.156715 | -1.558435 | -1.045329 | -0.587292 | -1.441553 | -0.464507 | -0.899168 | -1.378706 |
| -0.388793 | -0.186005 | -0.139634 | -0.294009 | -0.163960 | -0.847897 | -0.231718 | -0.158916 | -0.720330 |
| -0.234509 | 1.4728665 | 1.7553516 | -0.068441 | 0.2036451 | 0.9218091 | 0.2910966 | 1.1363696 | 0.2117507 |
| -0.935330 | -0.868027 | -1.093278 | 0.3479230 | -0.528152 | -0.052334 | -0.357544 | -0.629994 | -0.050040 |
| -0.471546 | -1.030463 | -2.460574 | -0.942613 | -0.084148 | -1.807342 | -0.138595 | -0.297345 | -1.916614 |
| -0.507708 | -1.102812 | -0.419026 | -0.347447 | -0.627007 | -0.213972 | -0.437093 | -0.356758 | -0.575837 |
| -0.295286 | -1.096817 | -0.474199 | -0.267127 | -0.069141 | -1.370259 | -0.034047 | -0.772791 | -1.959459 |
| -0.643283 | -1.902925 | -1.872231 | -0.930826 | -0.805687 | -0.761519 | -0.789538 | -0.628193 | -0.933765 |
| 0.0841582 | 0.2296687 | 0.2991309 | -0.386844 | -0.535451 | -0.439775 | -0.388698 | -0.273413 | -0.203831 |
| 0.2136821 | -0.203635 | -0.579962 | -0.521371 | -0.090291 | -0.948150 | -0.198961 | -0.804043 | -1.049214 |
| -0.172713 | -1.116899 | -2.918292 | -0.276552 | -0.272262 | -1.288273 | -0.080974 | -0.649981 | -1.371579 |
| -0.210623 | -0.501852 | -1.061968 | -0.726646 | -0.033728 | -2.241793 | -0.366822 | -0.494830 | -1.840601 |
| -0.043558 | -0.526869 | -1.650272 | -1.446502 | 0.2955823 | -1.118243 | 0.0441048 | -0.127544 | -1.037197 |
| -0.301025 | -0.672153 | -1.437106 | -0.459695 | -0.489634 | -0.643576 | -0.355568 | -0.868102 | -0.554134 |
| -0.093863 | -1.363338 | -2.192215 | -0.376470 | -0.217931 | -2.537620 | -0.269916 | -1.500487 | -2.045860 |
| 0.0732163 | 0.1373429 | -0.463767 | -1.506076 | 0.3524212 | -0.009359 | 0.5467743 | 0.3782293 | -0.067750 |
| -1.052110 | -1.007871 | -1.680661 | -0.514710 | -0.539296 | -1.598936 | -0.610404 | -0.585800 | -1.833549 |
| -0.339357 | -0.484028 | -0.419958 | -0.320594 | -0.322444 | -0.488934 | -0.438521 | -0.206920 | -0.339752 |
| -0.370652 | -1.866598 | -4.022098 | -0.599711 | -0.653619 | -2.613662 | -1.191627 | -1.264045 | -2.749871 |
| -0.371350 | -0.573375 | -0.453650 | -0.354148 | -0.175706 | -0.111842 | -0.153688 | -0.430782 | -0.022823 |
| -0.315963 | -0.449340 | -0.012913 | -0.416655 | 0.0413126 | -1.365751 | 0.1607819 | -0.238262 | -1.220665 |
| -0.635132 | -0.753618 | -1.770166 | -0.259552 | -0.407604 | -0.781355 | -0.323706 | -0.559171 | -0.917426 |
| -0.161044 | -0.303376 | -0.916766 | -0.412169 | -0.167895 | -1.123086 | -0.197209 | -0.404462 | -0.663862 |
| -0.537680 | -0.345199 | -0.311830 | -0.703017 | -0.396753 | -1.200853 | -0.467423 | -0.485823 | -0.835173 |
| -0.402973 | 0.1525428 | 0.1004750 | -0.399585 | -0.190498 | 0.7333074 | 0.0441826 | -0.017518 | 1.0579073 |
| -0.021569 | 0.2668941 | 1.4993092 | -0.803515 | 0.1815674 | 1.1127642 | 0.1329198 | 0.5061186 | 0.7407789 |
| -0.356253 | -0.231962 | -1.696046 | -0.157148 | -0.510067 | -1.998549 | -0.550259 | -1.042910 | -2.844120 |
| -0.105842 | -0.577307 | -0.534901 | -0.363430 | -0.016196 | -0.076486 | -0.245177 | -0.388445 | -0.388193 |
| -0.566965 | -0.276919 | 1.9569937 | -0.633332 | -0.358937 | 2.8424272 | -0.811412 | 1.3409479 | 2.5603185 |
| -0.222412 | -0.598324 | -1.516612 | -0.483826 | -0.226772 | -0.926260 | -0.225779 | -0.746780 | -0.876823 |
| -0.388564 | -0.784886 | -1.093915 | -0.405317 | -0.067630 | -0.758949 | -0.160718 | -0.110504 | -1.150166 |
| -0.545137 | -1.443105 | -1.130938 | -0.362726 | -0.759885 | -1.910070 | -0.334417 | -0.837842 | -2.009462 |
| -1.187583 | -2.106800 | -2.130815 | -0.481689 | -0.759865 | -2.555983 | -0.971994 | -1.582850 | -2.472109 |
| -0.112091 | -0.120959 | -0.199706 | -0.405611 | -0.746970 | -0.537865 | -0.650667 | -0.633199 | -0.563928 |
| -0.564280 | -0.200977 | -0.013435 | -0.359019 | 0.0401803 | 0.1562996 | -0.377075 | 0.3089034 | -0.133577 |
| -0.264346 | -0.878834 | -1.971001 | -0.447546 | -0.539289 | -1.018734 | -0.465237 | -0.832765 | -1.049767 |
| -0.214308 | -0.081571 | 0.8200968 | -1.598137 | -0.024409 | 1.0782739 | 0.1462733 | 0.0844370 | 1.2051334 |
| -0.449485 | -0.348976 | -0.735891 | -0.546088 | -0.043431 | -1.357977 | -0.256680 | -0.265661 | -1.343895 |
| -0.696440 | -1.208660 | -1.109361 | -0.762343 | -0.528135 | -1.568619 | -0.776554 | -0.806986 | -1.378684 |
| -0.439452 | -0.372678 | -0.935417 | -0.944663 | -0.347000 | -0.748827 | -0.246433 | -0.496917 | -0.690057 |
| -0.744638 | -0.403338 | -0.287621 | -0.103974 | -0.511044 | 0.1525981 | -0.485245 | 0.1741330 | 0.1818170 |
| -0.272594 | -0.903483 | -1.546626 | -1.585410 | -0.361136 | -1.396819 | -0.463706 | -1.372625 | -2.448587 |
| -0.233181 | -0.570092 | -0.573879 | -0.618825 | -0.460231 | -0.608327 | -0.266017 | -0.512306 | -0.572682 |
| -1.092667 | -0.767800 | -1.263981 | -0.796122 | -1.156406 | -1.570951 | -0.815181 | -1.687225 | -2.345923 |
| -0.592135 | -0.463466 | -0.074518 | -1.423383 | -0.298839 | 0.1343156 | -0.047009 | 0.0712668 | 0.4241374 |
| -0.791708 | -1.550304 | -1.923316 | -0.680478 | -0.952954 | -1.615833 | -0.984304 | -1.335601 | -1.624071 |

| | | | | | | | | |
|---|---|---|---|---|---|---|---|---|
| -1.0644104 | -0.6991194 | 0.7154083 | -0.8433329 | -0.4587410 | 1.0061521 | -0.2613775 | -0.0935896 | 1.2412955 |
| -0.5817839 | -0.9245595 | 0.2881326 | -0.5034308 | -0.4389428 | -0.3402951 | -0.1428963 | -0.2740800 | 0.3462517 |
| -0.5321140 | -0.5377590 | -0.0784640 | -0.4202410 | -0.3204673 | 0.0192271 | -0.2356255 | -0.1855758 | -0.2514519 |

| PG_363 | PG_364 | PG_365 | PG_366 | PG_367 | PG_368 | PG_369 | PG_370 | PG_371 |
|---|---|---|---|---|---|---|---|---|
| -0.1544786 | -0.3505235 | -1.2585608 | -0.3716139 | -0.6305322 | -1.5117039 | -0.5757263 | -1.2168793 | -0.8570224 |
| -0.4255827 | -0.7411616 | -0.5265534 | -0.4845720 | -0.6249833 | -0.8763420 | -0.5125149 | -0.6901792 | -0.6467556 |
| -0.0247867 | -0.1148089 | -0.1097644 | 0.2037632 | -0.1235858 | 0.0041030 | -0.4931618 | 0.0641844 | 0.4850515 |
| -0.0417047 | -0.0792881 | 0.1388490 | 0.1690585 | -0.2953459 | -0.0550711 | -0.2543852 | -0.2375545 | -0.6760860 |
| -0.1734210 | 0.1015550 | -0.5000850 | -0.1076492 | 0.0621738 | -0.3029696 | 0.3377126 | -0.2175305 | -0.4637830 |
| -0.4267810 | -0.5877559 | -1.6156894 | -0.3669218 | -1.0469979 | -1.5507254 | -0.6345645 | -0.5309316 | -1.0021550 |
| -0.2528730 | -0.6026521 | -0.5800889 | -0.0124085 | -0.9117823 | -0.1106808 | 0.1106993 | -0.3293805 | -0.2078007 |
| -0.2450842 | -0.3484775 | -1.1168698 | -0.5264116 | -0.3749714 | -1.7293033 | -0.4803520 | -0.7454378 | -0.3307335 |
| -0.2046953 | -0.1201669 | -0.4220340 | -0.1583480 | 0.1334347 | -0.9255739 | -0.2700612 | -0.4678800 | -0.7149666 |
| -0.4076605 | -0.8982823 | -1.2685406 | -0.7903489 | -1.3466913 | -1.6444548 | -0.7131994 | -0.3493039 | -0.0503698 |
| -0.1266011 | -0.2134470 | -0.1176518 | -0.2142255 | -0.1851389 | -0.3617699 | -0.3778444 | -0.5624525 | -0.8302689 |
| -0.2096702 | -0.4196573 | -0.2110853 | -0.2151060 | -0.6355356 | -0.1103062 | -0.2978792 | -0.4593936 | -0.4475144 |
| -0.5658459 | -0.4957051 | -1.3522013 | -0.3138296 | -1.1140881 | -2.1679448 | -0.5422265 | -0.5930103 | -0.7211810 |
| -0.4307819 | 2.2848876 | 3.6243664 | -0.6186616 | 5.0690933 | 4.4471437 | -1.0996518 | 2.5593265 | 2.7720335 |
| -0.1983914 | -0.3183285 | -0.0878465 | -0.1687713 | -0.8293224 | 0.0305760 | -0.2480244 | -0.5336153 | -1.0301005 |
| -0.6272214 | 1.3515656 | 2.3806151 | -0.0129776 | 3.0104053 | 3.1817953 | -1.1022747 | 2.1193004 | 1.6551036 |
| -0.6476627 | -2.1738662 | -1.2513973 | -0.9920251 | -2.8014455 | -1.8855630 | -1.2050791 | -3.1508128 | -2.0549472 |
| -0.2324199 | -0.4189979 | -0.9847192 | -0.6960409 | -0.6748740 | -1.4350037 | -0.3121015 | -0.5346173 | -0.2199513 |
| -0.1188920 | -0.3996340 | -0.1948457 | -0.2585504 | -0.3177965 | -0.2469525 | -0.6391811 | -0.8173736 | -0.0538664 |
| -0.6032890 | -0.6586754 | -0.5627168 | -0.6382682 | -0.7324348 | -0.7610512 | -1.0639976 | -1.1946578 | -0.5388645 |
| -0.2370271 | 0.1156782 | 0.0924926 | -0.2034470 | 0.3998535 | 0.4667666 | 0.1543086 | 0.3810611 | -0.1460305 |
| -0.3966264 | -0.6202606 | -0.2719988 | -0.3008325 | -0.4490062 | -0.5360442 | -0.5450428 | -0.5775672 | -0.0944411 |
| -0.0062303 | 0.3368004 | 1.4831808 | -0.0361091 | 0.8532471 | 1.8922350 | -0.2740082 | 0.9899964 | 0.2725875 |
| -0.3082398 | -0.6634815 | -1.3515100 | -0.6007563 | -1.4777826 | -1.5789111 | -0.2762624 | -0.9335199 | -0.5237731 |
| -0.2626968 | -0.8989076 | -1.9569665 | -0.6715806 | -1.3644973 | -2.3567795 | -0.0815743 | -0.8192896 | -0.7367832 |
| -0.1772072 | 0.4743164 | 0.6270064 | -0.2236291 | 0.5926014 | 0.6829355 | -0.2358267 | -0.1001371 | -0.2341564 |
| 0.0384207 | -0.1919196 | -0.4591852 | 0.1505183 | -0.1278093 | -0.5696904 | 0.3080220 | 0.2072015 | -0.1396119 |
| -0.6363842 | -0.9397445 | -1.0481718 | -0.4092927 | -1.2487008 | -1.1647028 | -1.1820815 | -1.1462322 | -2.3114471 |
| -0.3169976 | -0.7914107 | -1.9212195 | -0.4652374 | -1.3809202 | -2.8366824 | -1.4017475 | -0.7012351 | -0.3829002 |
| 0.0550071 | -0.1539697 | -0.2837461 | 0.1178283 | -0.4079307 | -0.2419527 | 0.0577721 | 0.0458912 | -0.4646060 |
| -0.1834040 | -0.4925999 | -0.2714435 | -0.4029829 | -0.6986418 | -1.1704804 | -0.9631837 | -0.6738332 | -0.1507653 |
| 0.1364041 | 0.3580073 | -0.2715612 | 0.2841173 | -0.0960838 | -0.2285150 | -0.6365235 | -1.5862217 | -1.7120539 |
| -0.1532531 | -0.1128324 | -0.1615881 | -0.2514950 | 0.3820056 | 0.0292415 | -0.0455088 | 0.0834243 | -0.9619312 |
| -0.1628079 | -0.1961713 | -1.5714396 | -0.6268531 | -0.5268774 | -2.0926061 | -0.5126929 | -0.8223088 | -0.6902014 |
| -0.1460469 | -0.1020152 | -0.1492506 | -0.0770645 | -0.1177092 | -0.0775197 | -0.2607453 | -0.2578852 | 0.1321218 |
| -0.1818802 | -1.3851865 | -0.1311362 | -1.2185821 | -1.3306167 | -0.2578022 | -0.5112423 | -0.6457963 | -0.8033757 |
| -0.0939502 | -0.3776409 | -0.9508857 | -0.8623429 | -0.5440990 | -1.4393604 | -0.1121412 | -0.3922206 | -0.4854070 |
| 0.0975212 | 2.2257510 | 2.6394590 | 0.2782918 | 4.2261299 | 0.3932820 | 0.0086833 | 3.4132104 | 0.6995587 |
| -0.1609290 | 0.0276186 | -0.095114 | 0.2077125 | -0.2545355 | 0.0306424 | 0.3761273 | -0.1874638 | -0.8500775 |
| -0.6020660 | -2.4518631 | -1.8170253 | -2.3311528 | -1.9398807 | -1.3080819 | -0.6310561 | -0.7935570 | -0.6150338 |
| 0.0037787 | 0.3173125 | 0.9528940 | 0.0167426 | 0.6640436 | 0.8897174 | 0.0220284 | -0.2039207 | -0.6171996 |
| -0.5058644 | -0.5703154 | -1.5948898 | -0.8022478 | -1.1202702 | -1.7966438 | -0.6852155 | -1.2317445 | -0.9526795 |
| -0.3124722 | -0.5171765 | -1.1063084 | -0.2769097 | -0.6961241 | -1.4487609 | -1.7182395 | -0.9302380 | -0.6142270 |
| -0.1539858 | -0.3764063 | -1.1945138 | -0.5521117 | -0.1378700 | -1.0535958 | -0.2031236 | -0.2301415 | -0.3961893 |
| -0.1133437 | 0.2661082 | 0.5556573 | -0.1960259 | 0.4666253 | 0.4372879 | -0.0474761 | 0.0350463 | 0.0646749 |
| -0.4445174 | 0.0472032 | 3.1179723 | -0.1263363 | 0.2539963 | 5.8912883 | -0.1657171 | -0.3029537 | 0.1640923 |
| -0.1995713 | -0.2365055 | -0.3423107 | -0.0673277 | 0.0514787 | -0.0566041 | -0.1987486 | -0.0162950 | 0.0924484 |

| | | | | | | | | |
|---|---|---|---|---|---|---|---|---|
| -0.347048 | -0.490912 | 0.1148648 | -0.127693 | 0.1019814 | 0.2078527 | -0.046253 | 0.1812790 | -1.805080 |
| -0.474029 | -0.472832 | -1.920366 | -0.639729 | -0.615820 | -1.610171 | -0.448912 | -0.986513 | -0.733729 |
| -0.462831 | -0.354648 | -0.282815 | -0.375067 | -0.106884 | -0.113922 | -0.276496 | -0.457406 | -0.272632 |
| -0.433554 | 0.0770599 | 0.4250783 | -0.499250 | 0.0687451 | 1.3945567 | -0.708632 | 1.0078247 | 2.4685403 |
| -0.086301 | -0.235540 | -0.210071 | -0.113639 | -0.029460 | -0.034724 | -0.562659 | -0.317883 | -0.260046 |
| -0.172445 | -0.187682 | -0.081489 | -0.186186 | -0.147282 | 0.2334164 | -0.714782 | -0.633225 | 0.7071503 |
| -0.089971 | -0.592861 | -1.378077 | -0.102299 | -0.912100 | -1.520906 | -0.482617 | -0.551710 | -0.625303 |
| -0.387051 | 1.0295340 | 2.9493283 | 0.1309148 | 2.9701310 | 3.2941132 | -0.509059 | 1.4057087 | 1.0457032 |
| -0.603449 | 2.9529767 | 3.4463278 | -0.358628 | 4.8766495 | 2.5974519 | 0.2827130 | 2.8747498 | 1.0715644 |
| -0.663762 | -0.562104 | -0.554312 | -0.182341 | -0.488559 | -0.208945 | -1.020150 | -0.403149 | -1.131855 |
| -0.474712 | -1.059812 | -1.352294 | -0.581102 | -1.564343 | -1.912010 | -0.763713 | -1.695759 | -1.301912 |
| -0.097490 | 2.1075210 | 2.1332834 | 0.6642608 | 3.2703687 | 2.8706227 | 0.2558920 | 2.9087988 | 1.1414986 |
| -0.511127 | 0.6148382 | 1.9706477 | -0.179077 | 2.5840267 | 1.9948711 | -1.146369 | 2.4058098 | 1.3960872 |
| -0.263282 | -0.292089 | -1.020505 | -0.175701 | -0.764583 | -1.143387 | -0.797910 | -1.045440 | -0.601828 |
| -0.751689 | -1.401208 | -2.344587 | -0.970459 | -1.719203 | -3.070502 | -0.705957 | -0.544499 | -0.580921 |
| -0.393428 | -0.663825 | -0.855594 | -0.538211 | -1.060395 | -1.049782 | -1.027315 | -1.045894 | -1.179587 |
| -0.093602 | -0.217315 | -1.359118 | 0.1594472 | -0.632768 | -1.623655 | -0.193793 | -0.518327 | -0.531446 |
| -0.254091 | -0.260111 | -0.396133 | -0.478041 | -0.625058 | -0.174049 | -0.172504 | -0.717190 | -0.378020 |
| -0.109317 | -0.330493 | -1.099228 | -0.776028 | -0.498327 | -1.025042 | 0.3076446 | -0.082542 | -1.037403 |
| -0.042381 | -0.155508 | -0.800756 | 0.0721647 | -0.264368 | -0.748733 | -0.032097 | -0.320607 | -0.644007 |
| -0.376221 | -0.321522 | -0.166998 | -0.577741 | -0.150475 | -0.603306 | -0.398284 | -0.494786 | -0.989081 |
| -0.562860 | -0.566231 | -1.122520 | -0.523238 | -0.713058 | -1.434192 | -1.200529 | -1.259072 | -1.082796 |
| -0.120743 | -0.096234 | 0.0789588 | -0.399339 | -0.008374 | 0.2174867 | 0.4203335 | -0.199260 | -0.198799 |
| 0.1122770 | 0.1611336 | 0.4541600 | 0.3695055 | 0.2770535 | 0.4134710 | 0.2216172 | 0.5034868 | -0.167060 |
| -0.475627 | -0.735164 | -1.701525 | -0.719261 | -1.295923 | -2.444190 | -1.014123 | -0.819338 | -0.206451 |
| -0.199880 | -0.653493 | -0.332020 | -0.421360 | -0.691845 | 0.1850455 | 0.2071049 | -0.345525 | -0.267214 |
| -0.302446 | -1.173266 | -1.556746 | -1.005627 | -1.361311 | -2.506609 | -1.433356 | -0.747075 | -0.087633 |
| 0.6230113 | 1.2762209 | 3.5949162 | 0.1413846 | 1.7143433 | 3.4953074 | -0.239296 | 1.8649407 | 2.5573127 |
| -0.432958 | 0.3327833 | 2.5965796 | -0.694387 | 1.2680657 | 2.6135319 | -0.902788 | 2.1393652 | 1.6047980 |
| -0.412686 | -0.622091 | -0.979865 | -0.223097 | -1.093125 | -1.666724 | -0.424928 | -0.971212 | -0.687700 |
| 0.1452530 | -0.846039 | 0.0796004 | -0.630608 | -0.340279 | 0.5028697 | -0.334630 | -1.273480 | -1.184587 |
| -0.023268 | -0.025911 | -0.233969 | -0.266037 | -0.555799 | -0.548572 | 0.2075207 | -0.182612 | -0.314801 |
| -0.607495 | -1.185060 | -1.394020 | -0.672469 | -1.658229 | -1.406703 | -0.514377 | -0.944436 | -0.591022 |
| -0.424964 | -0.576139 | -0.799356 | -0.233641 | -0.997986 | -0.985756 | -0.666317 | -0.781124 | -0.795617 |
| -0.307902 | 0.3138744 | 1.7129209 | -0.056660 | 0.7809908 | 1.5707698 | -0.092948 | -0.205091 | -0.190116 |
| 0.1884563 | 0.0830029 | -0.035232 | 0.3266587 | -0.067546 | 0.0021968 | -0.626732 | -0.116671 | 0.0485957 |
| -0.135492 | -0.509678 | -1.696731 | -0.622292 | -0.179037 | -1.566170 | -0.244019 | -0.035560 | -0.100678 |
| -0.144800 | -0.224238 | -0.864003 | -0.349348 | -1.007142 | -1.168216 | 0.0127625 | -0.373614 | -0.276967 |
| 0.1375802 | -0.308680 | -0.979241 | -0.589927 | -1.110026 | -1.754591 | -1.046172 | -0.642729 | -0.844233 |
| -0.407094 | -0.558977 | -1.245444 | -0.697649 | -1.150836 | -1.959064 | -0.700385 | -1.876920 | -1.335787 |
| -0.365000 | -0.650467 | -1.084034 | -0.498998 | -0.536767 | -0.993182 | -0.587220 | -0.837314 | -1.289514 |
| -0.487023 | -2.002883 | -3.429793 | -1.182437 | -3.910676 | -4.097076 | 0.6638934 | -0.955094 | 0.6825600 |
| -0.028406 | 0.8564941 | 2.7447033 | -0.128404 | 2.9766250 | 1.0327689 | -0.404194 | 1.0577981 | 4.1391799 |
| 0.0851097 | -0.000132 | -0.079600 | -0.063799 | -0.123096 | -0.159586 | 0.0471785 | -0.093958 | -0.104415 |
| 0.0547981 | -0.146687 | -0.848841 | -0.182137 | -0.141701 | -1.412784 | 0.2369084 | -0.109099 | -0.125220 |
| -0.906407 | -1.856885 | -1.695035 | -1.303408 | -2.195608 | -1.980133 | -1.330033 | -2.377186 | -1.882282 |
| -0.080764 | 3.5842343 | 4.0974383 | 0.1855336 | 7.5941289 | 5.7676895 | 0.0085869 | 0.2760959 | -0.049263 |
| -0.025178 | 0.6264002 | 1.1389782 | 0.2346961 | 1.9371951 | 1.6241119 | 0.2080898 | 1.6974017 | 0.9844972 |
| -0.433553 | -0.671438 | -2.695909 | -1.478047 | -1.025585 | -2.605546 | -1.221162 | -0.436734 | -0.419909 |
| -0.243317 | -0.323520 | -1.069695 | -0.151835 | -0.367042 | -1.003774 | -0.991992 | 0.0583738 | -0.085671 |

| | | | | | | | | |
|---|---|---|---|---|---|---|---|---|
| -0.2493178 | -0.2417749 | -0.3897049 | -0.0875151 | -0.4051390 | -0.0845571 | -1.3400205 | -0.6910889 | -0.2744206 |
| -0.0888773 | -0.0893922 | 0.2849674 | -0.2687286 | -0.2209191 | 0.6175105 | 0.4392715 | 0.0569720 | -0.0101453 |
| -0.0353844 | -0.3850093 | -0.3637063 | -0.2073148 | -0.9208173 | -0.4045862 | -0.2473539 | -0.9564656 | -1.1854187 |
| 0.0250770 | 0.0609424 | 0.2382858 | -0.2730300 | -0.0052889 | 0.5593279 | 0.0086836 | -0.1012472 | -0.5547698 |
| -0.2272542 | -0.5963122 | -1.8148489 | -0.5450811 | -1.4027744 | -2.1185098 | 0.1787425 | -0.9491947 | -0.2685231 |
| 0.0221773 | -0.2506911 | -0.3650910 | -0.0613146 | -0.3268138 | -0.5165534 | -0.8572497 | -1.2101192 | -1.3136458 |
| 0.0679470 | 0.2097295 | -0.2273055 | 0.1787671 | 0.2981582 | 0.0917138 | -0.5297669 | -0.2807517 | -1.1487302 |
| -0.0844132 | -0.0098861 | 0.0594563 | -0.2793666 | 0.1299174 | 0.2088639 | -0.3576004 | -0.0855257 | -0.1905855 |
| -0.1809441 | -0.1602631 | 0.1359452 | -0.3132959 | -0.3312592 | -0.1681362 | -0.3528796 | -0.2696781 | 0.3227703 |
| -0.1233001 | 0.3813001 | 0.4745567 | -0.2495411 | 0.4714488 | 0.5287153 | -0.2582595 | 0.0511751 | -0.2576082 |
| -0.1465531 | -0.1215272 | -1.3793515 | -0.1952152 | -0.2983280 | -1.5279715 | -0.8216665 | -0.3022626 | 0.0057085 |
| -0.5326685 | -0.4602010 | -1.0080651 | -0.6459387 | -0.7631166 | -1.7643514 | -1.1616618 | -0.4966899 | -1.1480820 |
| -0.3220740 | -0.3807941 | -1.9983048 | -0.4705334 | -0.8232641 | -2.5306894 | -0.5218132 | -0.7171610 | -1.0403607 |
| 0.0150520 | -0.0515694 | -0.9067878 | -0.2111911 | -0.4440819 | -0.6924137 | -0.7242983 | -0.4465134 | -1.1357330 |
| -0.2214721 | -0.3144857 | 0.0412236 | -0.1480754 | -0.1873811 | 0.2088882 | -0.7400673 | -0.6682781 | -0.4731333 |
| -0.2485828 | -0.0410567 | -0.0042017 | 0.0081401 | -0.0201464 | -0.0969305 | 0.0061380 | -0.0563032 | -0.0907663 |
| -0.6943167 | -1.2662295 | -0.1540190 | -0.6269478 | -1.8651115 | -0.5552024 | -0.5450766 | -0.9310840 | -1.0804416 |
| -0.3667968 | -0.4779643 | 0.0418940 | -0.0883355 | 0.0304573 | -0.0419560 | -0.3493936 | 0.1555705 | -0.0594398 |
| -0.4315349 | -0.5149742 | -0.0411697 | -0.1682222 | -0.6786045 | -0.5865545 | 0.4900898 | 0.1677813 | -0.0429844 |
| 0.3213870 | 0.1147668 | 1.7513653 | -1.0811800 | 0.8225975 | 1.9317533 | -0.1516541 | 0.3172743 | 0.1094487 |
| -0.3403519 | -0.4759276 | -0.8544148 | -0.3369836 | -0.4796667 | -1.1283232 | -0.4058435 | -0.7561286 | -0.7347010 |
| -0.3034572 | -0.4257981 | 0.5128262 | -0.3823734 | -0.4235513 | 0.3232396 | 0.1340938 | -0.3483692 | -0.2218572 |
| -0.2066082 | -0.1266667 | -0.1573541 | 0.1206952 | 0.1734790 | -0.2082398 | -0.2348019 | -0.1722371 | -0.1214962 |
| -0.3121295 | -0.6790662 | -1.2373846 | -0.4404879 | -0.6970239 | -1.2590617 | 0.0105271 | -0.3362580 | -0.3197480 |
| -0.1444960 | -0.2384925 | -0.1875727 | 0.0046332 | -0.3338352 | 0.1224669 | -0.4966677 | -0.7759156 | -0.0251665 |
| 0.1474345 | -0.1224696 | 0.1251409 | -0.4784990 | -0.5539256 | 0.0922309 | 0.0387644 | -0.3672191 | -0.0354793 |
| 0.0921276 | -0.0797736 | -0.0478621 | -0.2380481 | -0.3691664 | -0.0109309 | 0.1461537 | -0.2426787 | -0.0635419 |
| 0.0062868 | 0.0498231 | -0.0078928 | -0.4440516 | 0.0668481 | -0.2418532 | -0.4656176 | -0.3757549 | 0.2130068 |
| -0.0873581 | -0.5932685 | 0.4464306 | -0.4524556 | -1.5573551 | -0.2775213 | -1.5194480 | -0.9253313 | -0.7477431 |
| -0.3864975 | -0.4094992 | -0.7319860 | -0.2169439 | 0.0525508 | 0.1101681 | 0.0327756 | -0.4398792 | -0.9952519 |
| -0.8667343 | -0.6899100 | -1.2418143 | -0.2715201 | -0.4390648 | -1.0411362 | -0.1310145 | -0.7759357 | -0.3871024 |
| 0.0545057 | -0.2521758 | -0.8770163 | -0.0833954 | -0.3691167 | -1.3704569 | -1.5145440 | -1.2495615 | -1.4139190 |
| -0.5014042 | -0.7168188 | 0.4569654 | -0.5380203 | -0.1124007 | 0.7593104 | -1.0077998 | -1.0998746 | 0.3961140 |
| 0.2598922 | 0.1928333 | -0.0287780 | -0.3764640 | 0.1546748 | -0.0398707 | 0.3311554 | 0.3157951 | -0.1616709 |
| -0.2370229 | -0.0893566 | -1.1386394 | -0.7453358 | -0.5447970 | -1.7932340 | -0.2628612 | -0.5256327 | -0.8342210 |
| -0.2320944 | -0.3976233 | -0.7585757 | -0.2726879 | -0.8094724 | -0.9882130 | -0.3574132 | -0.3773405 | -0.4475426 |
| -0.3031666 | -0.2238814 | -0.4636531 | -0.2382260 | -0.2498172 | -0.5837294 | -0.1566981 | -0.3364701 | -0.5297540 |
| -0.1464902 | -0.1636715 | -0.2752496 | -0.1999794 | -0.2848051 | -0.3289141 | 0.0155016 | -0.2085892 | -0.3765360 |
| -0.3212545 | -0.0919924 | -0.6835717 | -0.5059742 | -0.3846543 | -0.6414817 | -0.4170571 | -0.5459745 | -0.2007239 |
| -0.2065529 | -0.7207224 | -0.8314540 | -0.2822048 | -0.6257269 | -0.2386382 | -1.1626603 | -0.8195363 | -0.5009908 |
| 0.2976167 | -0.4010584 | -0.1378988 | -0.8480321 | -0.8121235 | -1.4302926 | 0.0648665 | -1.3107373 | 0.0466476 |
| -0.1371673 | -0.0685288 | -0.5911892 | -0.3818225 | 0.1279100 | -0.2677566 | -0.2489572 | -0.0812008 | -0.3672730 |
| 0.0202378 | -0.0754566 | -0.5751013 | -0.1134254 | -0.1492645 | -0.3536921 | 0.5869828 | 0.2456977 | -0.3486026 |
| -0.0672605 | -0.4267138 | -1.1244804 | -0.2214310 | -0.3509908 | -1.7620470 | -0.1252913 | -0.3515009 | -0.0169181 |
| -0.1334821 | -0.0423093 | -0.0911441 | -0.0789272 | -0.0644689 | -0.1152294 | -0.5490638 | -0.5280168 | -0.0155918 |
| -0.3352741 | 0.0442602 | 0.5618578 | 0.1236350 | 0.6277250 | 0.6144775 | -0.7002053 | -0.0193008 | -0.4299218 |
| -0.2677276 | -0.4041832 | -0.4370879 | -0.4048189 | -0.6422062 | -1.0850678 | -0.3679777 | -1.0431084 | -0.3850243 |
| -0.2174186 | 0.1666327 | 0.9906077 | -0.1154741 | 0.4070784 | 1.0061311 | -0.0577380 | -0.1306704 | -1.0542191 |
| -0.1741127 | -0.4358973 | -0.7481251 | -0.0590850 | -0.8081576 | -0.7746081 | -0.5853176 | -1.3662450 | -1.0529421 |
| -0.8882865 | -1.2179324 | -1.8190816 | -1.1021595 | -0.9112460 | -2.0746401 | -0.8321606 | -1.3634312 | -1.4482988 |

| | | | | | | | | |
|---|---|---|---|---|---|---|---|---|
| 0.2377764 | 0.1013267 | 0.1577558 | -0.032877 | 0.4126220 | 0.5768793 | 0.4320377 | 0.4834587 | -0.004725 |
| -0.832492 | -1.290473 | -2.179409 | -1.468673 | -2.344251 | -2.310993 | -0.793324 | -1.652251 | -1.567908 |
| -0.489688 | -0.460939 | -0.707416 | -0.423586 | -0.211322 | -0.536284 | -0.535555 | -0.576955 | -0.626102 |
| -0.466243 | -0.306058 | -0.245236 | -0.348304 | -0.594422 | -0.123002 | -0.234346 | -0.356997 | -0.136773 |
| -0.324552 | -0.738192 | -1.533619 | -0.523468 | -1.107116 | -1.523860 | -0.164823 | -0.708077 | -0.183610 |
| -0.004564 | 1.2993455 | 2.3833831 | -0.166997 | 2.2545001 | 2.4463908 | -0.396399 | 1.0628385 | 1.4500464 |
| -0.240691 | -0.340371 | -0.813897 | -0.435069 | -0.527958 | -0.913965 | -0.050178 | -0.436490 | -0.317028 |
| -0.344474 | -0.661189 | -0.659186 | -0.800171 | -0.990314 | -1.000070 | -0.052677 | -0.447584 | -1.200796 |
| -0.061767 | -0.682876 | -1.277821 | -0.732231 | -1.420826 | -2.116904 | -0.122639 | -0.765724 | -0.540585 |
| -0.461376 | -0.648241 | 0.1499001 | -0.565409 | -0.326810 | 0.2231686 | -0.710494 | -1.022872 | -0.559530 |
| -0.193098 | -0.282443 | 0.1342099 | -0.150136 | 0.3070930 | 0.5886561 | -0.101290 | 0.1900720 | -0.189376 |
| -0.738653 | -0.855411 | -0.957150 | -1.044935 | -0.700448 | -0.226883 | -1.332492 | -1.564239 | -1.643457 |
| 0.0759489 | -0.813114 | -0.618476 | 0.1496906 | -0.481679 | -0.234194 | -1.880575 | -1.612694 | -0.698411 |
| -0.155816 | -0.489137 | -0.891842 | -0.091921 | -0.533591 | -0.933451 | -0.959909 | -0.321041 | -0.994918 |
| -0.366935 | -0.214128 | -0.140899 | -0.073957 | -0.277539 | -0.118653 | -0.216744 | -0.107210 | -0.467899 |
| -0.232819 | -0.318760 | -0.399841 | -0.175033 | -0.383056 | -0.205814 | -0.725165 | -0.510990 | -0.289939 |
| -0.246091 | -0.284746 | -0.337261 | -0.546213 | 0.0244357 | -0.287974 | -0.532075 | -0.790005 | -0.448967 |
| -0.601291 | -0.817748 | -0.710298 | -0.663198 | -1.604827 | -1.227215 | -0.737892 | -1.038791 | -0.750490 |
| -0.535044 | -0.783747 | -0.383267 | -0.822166 | -0.568667 | -0.256981 | -0.648335 | -0.377934 | -0.625600 |
| -0.332487 | -0.500026 | -0.513078 | -0.437705 | -0.900361 | -0.546270 | -0.451599 | -0.433159 | -0.963511 |
| -0.152836 | -0.165718 | 0.0708740 | 0.0136979 | 0.0756857 | 0.1299461 | -0.234179 | -0.240427 | 1.0536226 |
| -0.232338 | -0.233594 | 0.1060634 | -0.034921 | 0.2308195 | 0.5669370 | 0.1337558 | 0.1821921 | -0.622959 |
| -0.248107 | -0.746148 | -1.649289 | -0.438261 | -0.967156 | -2.023847 | -0.799455 | -1.646965 | -1.086561 |
| -0.377378 | -0.636382 | -1.061987 | -0.172237 | -0.354288 | -1.064970 | -0.680591 | -1.061608 | -1.445799 |
| -0.246937 | -0.667670 | -1.076636 | -0.478929 | -1.005306 | -1.382512 | -0.781464 | -0.915970 | -0.842449 |
| -0.362963 | -0.519405 | 0.1624988 | -0.267414 | -0.834353 | -0.198396 | -0.608811 | -0.785957 | -1.036559 |
| -0.364407 | -0.798270 | -0.852066 | -0.748096 | -1.391721 | -1.613946 | -1.677821 | -1.408145 | -0.971121 |
| -0.127080 | -0.328141 | -0.157951 | -0.004257 | -0.511387 | 0.0377396 | 0.1221318 | 0.0028149 | -0.292984 |
| -0.598370 | -1.024396 | -0.564876 | -1.455777 | -1.195230 | -1.024120 | -0.729374 | -0.982023 | -1.331444 |
| 0.2481958 | 0.2692309 | 0.5417428 | 0.2124700 | 0.6253584 | 0.7426917 | -0.114116 | 0.4023566 | -0.387797 |
| 0.1326121 | 1.0904367 | 3.2561132 | -0.392797 | 2.4139151 | 3.9362215 | -0.119308 | 1.4675918 | 0.8242152 |
| -0.754009 | -0.858712 | -0.582649 | -0.676076 | -0.773767 | 0.0362021 | -0.178575 | -0.830034 | -1.149996 |
| -0.015616 | -0.155543 | -0.176072 | -0.248683 | -0.262290 | -0.150984 | 0.1204017 | -0.333249 | -0.204091 |
| 0.0322858 | 0.1914943 | 0.3378275 | -0.245927 | 0.2489682 | 0.5638602 | 0.3803411 | 0.5401601 | 0.1681207 |
| 0.2948406 | 0.8678809 | 0.9745082 | 0.0649088 | 0.7653437 | 1.1767029 | 0.7247339 | 0.9766137 | 0.6247140 |
| -0.585565 | -0.850720 | -1.212629 | -0.116405 | -1.103931 | -1.712744 | -0.526715 | -0.738034 | -0.864579 |
| -0.068064 | 0.2457548 | 0.7691236 | 0.1780007 | 0.7636258 | 1.6066191 | 0.2919880 | 0.7599136 | -0.454262 |
| -0.346494 | -0.121116 | -0.206365 | -0.456845 | 0.3456109 | 0.4460481 | 0.0111888 | 0.3361036 | -0.200132 |
| -0.225647 | -0.259835 | 0.8648040 | -0.071616 | 0.0504464 | 1.2056967 | -0.025733 | -0.113401 | -0.486180 |
| -0.165433 | -0.369380 | -0.384183 | -0.244593 | -0.544496 | -0.492578 | -0.643078 | -0.437840 | -0.706817 |
| -0.457546 | -0.649846 | -0.993849 | -0.146873 | -0.629357 | -0.657546 | -1.133805 | -0.817714 | -0.040236 |
| -0.115767 | -0.218800 | 0.4382060 | -0.195530 | 0.2314316 | 0.6582265 | -0.330288 | -0.234312 | -0.842104 |
| -0.256788 | 0.1169498 | 1.8808922 | -0.871332 | 0.2668347 | 1.7279628 | 0.0894898 | 1.7800341 | 0.7872989 |
| -0.416680 | -0.168422 | 0.3910316 | 0.1152433 | 0.2198650 | 0.8465938 | -0.161758 | 0.0925072 | -0.361396 |
| -0.035546 | -0.159096 | -0.339149 | -0.208617 | -0.485684 | -0.364955 | -0.263937 | -0.687276 | -0.510336 |
| -0.313096 | -0.176285 | 0.1874588 | -0.458533 | -0.221092 | 0.5904077 | -0.100503 | -0.154621 | -0.580231 |
| -0.311555 | -0.407264 | -0.838885 | -0.572270 | -0.862127 | -1.491380 | -0.677864 | -1.050189 | -0.756028 |
| -0.343240 | -0.716160 | -2.027030 | -0.573192 | -0.961064 | -2.669430 | -0.385711 | -1.030054 | -0.218204 |
| -0.299648 | 0.0194702 | 0.0485268 | 0.6809625 | 0.1657065 | 0.4459208 | -0.665526 | -0.551278 | -0.270216 |
| -0.280048 | -0.397070 | -0.281840 | -0.555317 | -0.481105 | -0.123214 | -0.334360 | -0.171812 | -0.484089 |

| | | | | | | | | |
|---|---|---|---|---|---|---|---|---|
| 0.0392486 | -0.0823300 | -0.0129764 | -0.0501152 | -0.0727828 | -0.2137167 | -0.9596826 | -0.2572722 | -0.9052830 |
| -0.4606728 | -0.4481984 | -0.7008701 | -0.3305149 | -0.1882293 | -0.6070739 | -0.7239846 | -0.6724904 | -1.5816168 |
| -0.5396063 | -0.5503574 | -0.6215322 | -0.5261585 | -0.1574992 | -0.3679598 | -0.3582522 | 0.0826279 | -0.8232336 |
| -0.3892912 | -0.7462112 | -1.2454155 | -0.0507450 | -1.1694856 | -2.1245783 | -1.0582472 | -0.9133057 | -0.8209989 |
| -0.3487740 | -0.7213833 | -0.6986462 | -0.5699006 | -1.1046277 | -0.9817965 | -0.6468823 | -0.7314812 | -1.4439354 |
| -0.1351640 | 0.1866566 | -0.1329784 | -0.2579090 | 0.0019373 | -0.2305950 | -0.1980021 | 0.0346592 | 0.0649674 |
| -0.1487100 | 0.9379124 | 2.7990193 | 0.0282345 | 1.5404438 | 2.3475897 | -0.5542892 | 1.2644498 | 2.6800297 |
| -0.3583190 | -0.5660256 | -0.9245126 | -0.4345950 | -0.8453999 | -0.6264885 | -0.4100979 | -0.2913777 | -0.1217063 |
| -0.1322921 | -0.2382806 | -1.1176561 | 0.3834042 | -0.5993328 | -1.4884780 | -0.1808609 | -0.2736370 | -0.9344460 |
| -0.2857645 | -0.4482316 | 0.4047680 | -0.6464132 | -0.1457967 | 0.4857890 | -1.0403122 | -1.7669517 | -1.1300230 |
| -0.0005969 | -0.6162184 | -0.3812239 | -1.1358472 | -0.8874830 | -1.4420786 | -1.0409154 | 0.0195719 | 0.0751627 |
| -0.5573182 | -0.9642452 | -0.0968218 | -0.5547777 | -0.5243566 | -0.1051386 | -0.5985393 | -0.9611121 | -1.8359492 |
| -0.0588360 | 0.0917058 | 0.1125889 | 0.1045296 | 0.1961573 | 0.3075433 | -0.3589578 | -0.3854934 | 0.1116839 |
| 0.2057451 | -0.0399925 | -0.0501262 | -0.4373685 | -0.1094793 | -0.3169159 | -0.5399917 | -0.3146910 | -0.8476316 |
| -0.2655634 | -0.4027790 | -1.1711373 | -0.2436958 | -1.0397492 | -1.2710833 | -1.0934154 | -1.1635893 | -1.4067780 |
| -0.0568585 | -0.2986911 | -0.8632121 | -0.3373231 | -0.1707755 | -1.7509625 | -1.0644092 | -1.0415317 | -1.2423295 |
| -0.1476996 | -0.1975674 | -0.5558915 | -0.1933117 | -0.4021798 | -0.9980923 | 0.1093152 | -0.0659379 | -0.2677693 |
| -0.2198713 | -0.6327234 | -0.7549846 | -0.2419141 | -0.9517284 | -1.2451157 | -0.3982062 | -1.1759017 | -0.9406519 |
| 0.1395103 | -0.4734213 | -1.2662516 | -0.1839617 | -1.2198565 | -1.3521319 | -0.3301407 | -1.6784101 | -1.1465344 |
| 0.3287454 | 0.2486692 | 0.5341824 | 0.0876286 | 0.2476167 | 0.7381450 | 0.1027201 | 0.4656181 | -0.8569477 |
| -0.4036657 | -0.8298061 | -0.5455698 | -0.5269205 | -0.4781873 | -1.1643808 | -0.7064975 | -0.6900268 | -1.0955988 |
| -0.2757329 | -0.2519930 | -0.2338054 | -0.2654186 | -0.1616649 | -0.2460395 | -1.1547010 | -1.7657892 | -0.9938516 |
| -0.7155695 | -1.4277146 | -4.2169361 | -0.7067442 | -3.3733873 | -4.9097042 | -1.2280702 | -2.0980341 | -0.8547360 |
| -0.0557310 | -0.0292143 | 0.0131649 | -0.0601712 | -0.2528863 | 0.3506731 | -0.1430046 | -0.1416300 | -0.1832984 |
| 0.0902613 | -0.2399737 | -0.6287429 | -0.2343344 | -0.2522633 | -0.6967375 | 0.1601544 | -0.5691059 | -0.8029923 |
| -0.5619305 | -0.5385085 | -0.7160710 | -0.4994761 | -0.6400671 | -0.6338375 | -0.3856826 | -0.6890659 | -0.6520345 |
| -0.0788745 | -0.1209397 | -0.5409007 | 0.0574806 | -0.3915001 | -0.3732901 | -0.1326346 | -0.1741549 | -0.3877317 |
| -0.2646816 | -0.2768379 | -0.8739194 | -0.4131073 | -0.6982769 | -0.9319678 | -0.4192488 | -0.6192176 | -0.6952190 |
| -0.0475318 | 0.0460339 | 1.2192981 | -0.0525464 | 0.4177662 | 1.6653538 | -0.9621700 | -0.1527517 | -0.7184762 |
| -0.0307646 | -0.0451338 | 1.8093809 | -0.4939263 | 0.2721565 | 1.4837857 | -0.3806922 | -0.0226565 | 0.0580593 |
| -0.5426696 | -0.4830721 | -1.0968774 | -0.1767821 | -0.5357138 | -1.8781988 | -0.8626992 | -0.3929703 | -0.9099356 |
| -0.1301634 | -0.3222182 | -0.2115764 | -0.1337369 | -0.3239902 | -0.5395188 | -0.2217916 | -0.4525947 | -0.4096008 |
| -0.5281100 | -0.6900847 | 1.8623765 | -0.9706838 | -0.2140031 | 0.7851946 | -0.8083872 | -0.8883297 | -0.4226272 |
| -0.2480324 | -0.1806688 | -0.8731168 | -0.0614444 | -0.7400473 | -0.8331008 | -0.1606930 | -0.4340068 | -0.4906243 |
| -0.1570023 | -0.6998940 | -1.2498191 | -0.3924005 | -0.9849856 | -1.5667922 | 0.1307774 | -0.2583469 | -0.4115805 |
| -0.0469131 | -0.3708713 | -0.5705279 | -0.3623329 | -0.9013921 | -1.0088700 | -0.8651542 | -1.5009998 | -1.2085351 |
| -0.4365125 | -0.9557394 | -1.9553748 | -0.4763836 | -2.0385427 | -2.2405244 | -1.2663119 | -1.7803182 | -1.8120377 |
| -0.2097840 | -0.1269571 | -0.2142241 | 0.1008061 | -0.1251222 | -0.0453244 | -0.7350919 | -0.7155981 | -0.2804449 |
| -0.5397291 | -0.0267731 | 0.7570707 | -0.4563115 | 0.5113072 | 0.6451637 | -0.0806957 | 0.5393452 | -0.2280007 |
| -0.2781551 | -0.2764109 | -0.5988233 | -0.3840877 | -0.5356831 | -1.0425007 | -0.7157441 | -0.6840715 | -0.6016552 |
| -0.1170940 | 0.1321159 | 0.8652494 | -0.2812926 | 0.1077218 | 1.3912152 | -0.0513228 | -0.0138727 | -0.5530633 |
| -0.0679339 | -0.3522404 | -1.0081973 | -0.2932903 | -0.9642107 | -1.3959196 | -0.1054151 | -0.7704359 | -0.3634971 |
| -0.5368042 | -0.8151422 | -1.1910971 | -0.9967161 | -1.1031552 | -1.4983849 | -0.4818899 | -0.1745223 | -0.3480611 |
| -0.0656682 | -0.4879961 | -0.4791310 | -0.2853780 | -0.4699836 | -0.6389146 | -0.3665964 | -0.6287107 | -1.0295823 |
| -0.4256902 | -0.4336977 | 0.0001416 | -0.3929601 | -0.1330916 | 0.5661295 | -0.2236536 | -0.3712772 | -0.6751152 |
| -0.3250493 | -0.3047443 | -0.8410153 | -0.7219839 | -0.7044989 | -0.9729382 | -0.7303565 | -1.1290311 | -0.9918957 |
| -0.1174051 | -0.3934432 | -0.0830857 | -0.2540425 | -0.5487312 | -0.2135405 | 0.0502202 | -0.4615916 | -0.3703659 |
| -0.6999946 | -1.6332920 | -1.0059105 | -1.5417231 | -1.4386270 | -1.6476020 | -1.3073199 | -1.2016190 | -1.4547291 |
| -0.3839976 | -0.3677818 | -0.0049597 | -0.1801164 | 0.2561488 | 1.0157769 | -0.4251084 | -0.2283530 | -1.1971696 |
| -0.4573433 | -0.7771462 | -1.4410753 | -0.6276277 | -1.2159924 | -1.5457254 | -0.7223257 | -1.4118209 | -1.3404549 |

| | | | | | | | | |
|---|---|---|---|---|---|---|---|---|
| -0.2550225 | -0.3245305 | 0.7326230 | 0.1271932 | 0.4141567 | 1.2094041 | -0.1095721 | -0.2356250 | -0.7813631 |
| -0.4188835 | -0.3701689 | -0.4233633 | -0.3400687 | -0.2445094 | 0.5991849 | -0.4393788 | -1.0031650 | -0.4540256 |
| -0.2221945 | -0.2178804 | -0.1499138 | -0.5441678 | -0.3186713 | 0.2378045 | 0.0004666 | -0.2080400 | -0.4514790 |

| PG_372 | PG_373 | PG_374 | PG_375 | PG_376 | PG_377 | PG_378 | PG_379 | PG_380 |
|---|---|---|---|---|---|---|---|---|
| -0.354062 | -1.269474 | -0.517312 | -0.335693 | -0.790474 | -0.211791 | -0.239489 | -1.077730 | -0.384755 |
| -0.548474 | -0.557955 | -0.171472 | -1.262128 | -1.039886 | -0.713707 | -1.000073 | -1.438176 | -0.540546 |
| -0.421296 | -0.342038 | -0.385198 | -0.787899 | -1.019903 | -0.560596 | -1.298060 | -0.590084 | -0.958680 |
| -0.286423 | -0.718323 | 0.2991564 | -0.878555 | -0.757892 | -0.929361 | -1.081922 | -1.233408 | 0.1502948 |
| 0.2719853 | -0.428716 | 0.0526044 | -0.414606 | -1.156062 | -0.530249 | -0.569282 | -0.637268 | 0.0216007 |
| -0.542742 | -0.373290 | -0.212589 | -0.390479 | -0.673891 | -0.476809 | -0.407357 | -1.308092 | -0.256476 |
| 0.1391245 | -0.312157 | -0.104214 | -1.105171 | -0.352859 | -0.512987 | -1.319385 | -0.508194 | -0.148355 |
| -0.580197 | -0.898893 | -0.067821 | -0.006372 | -0.866731 | -0.030917 | 0.0363989 | -1.071457 | -0.185214 |
| -0.275672 | -0.241640 | -0.212023 | 0.0699076 | -0.010284 | 0.0444503 | 0.3031652 | 0.1217845 | -0.121467 |
| -0.770757 | -0.447215 | -0.449444 | -1.100606 | -0.795360 | -0.767638 | -0.983722 | -0.712198 | -0.437490 |
| -0.502006 | -0.603605 | 0.0679106 | -0.332234 | -0.390297 | -0.354397 | -0.358061 | -0.759810 | -0.067061 |
| -0.369588 | -1.009274 | -0.198127 | -0.533490 | -0.580741 | -0.763013 | -0.950103 | -1.007347 | -0.127997 |
| -0.530534 | -0.524267 | -0.204813 | -0.235385 | -0.257989 | -0.120803 | -0.604940 | -1.573994 | -0.440320 |
| -0.961708 | 3.2547085 | -1.023956 | 0.5287735 | 1.8360421 | -0.769783 | 2.3676123 | 3.6734657 | -0.623680 |
| -0.003920 | -0.331449 | -0.323749 | -1.189337 | -0.122598 | -1.168052 | -1.431989 | -0.218132 | 0.0150176 |
| -0.723869 | 2.7256807 | -0.549154 | 2.5750117 | 1.6529653 | 0.4812288 | 3.1318548 | 2.9076095 | -1.018797 |
| -1.602365 | -3.263100 | -1.043290 | -1.904771 | -0.680743 | -0.480037 | -1.093461 | -0.888139 | -0.636104 |
| -0.448584 | -0.471014 | -0.918125 | -2.083528 | -0.747052 | -1.031118 | -2.492976 | -1.673487 | 0.2841651 |
| -0.700188 | -0.850854 | -0.152082 | -0.675571 | -2.534243 | -0.689463 | -0.850354 | -2.992207 | -1.161615 |
| -1.204945 | -1.230636 | -1.756113 | -1.012340 | 0.7164849 | -1.950105 | -0.936157 | -2.136027 | -1.260212 |
| 0.0937727 | 0.3450862 | -0.342205 | 0.1392698 | 0.2248712 | -0.431612 | 0.2506101 | 0.5777071 | -0.099745 |
| -0.318164 | -0.680994 | -0.317755 | -0.476396 | -0.438334 | -0.516279 | -0.461781 | -0.350284 | -0.209341 |
| -0.015146 | 0.7155729 | -0.246872 | 1.2297311 | 1.5986449 | 0.4214827 | 1.3542209 | 1.6432816 | -0.136198 |
| -0.272652 | -1.547199 | -0.659878 | -0.698315 | -0.977112 | -0.562627 | -1.150228 | -1.879179 | -0.343219 |
| -0.201448 | -0.990965 | -0.269781 | -0.590917 | -1.883384 | -0.298231 | -1.067213 | -2.628608 | -0.154993 |
| -0.215380 | -0.325199 | 0.1188247 | 1.2008334 | 1.5646420 | 0.3230099 | 1.1278447 | 1.4640228 | -0.369995 |
| 0.3629055 | 0.2912204 | 0.1503401 | -1.011324 | -1.280058 | -1.125297 | -1.050168 | -1.226428 | 0.0948006 |
| -1.189058 | -1.231816 | -0.633385 | -1.612870 | -1.442626 | -0.706486 | -1.266671 | -2.273710 | -0.555564 |
| -1.259921 | -1.096080 | -0.240426 | -0.554015 | -1.308274 | -0.336414 | -0.914138 | -2.925510 | -0.150033 |
| -0.007497 | -0.307399 | 0.1455209 | -0.916469 | -0.265290 | -0.932706 | -1.092172 | -0.306091 | 0.0070887 |
| -0.827040 | -0.626335 | 0.2362399 | -0.344730 | -1.368179 | 0.0814092 | -0.349429 | -1.123338 | 0.5964628 |
| -0.951783 | -1.288559 | -0.017886 | 0.3170611 | 0.2036411 | -0.037477 | 0.0355372 | 0.0696710 | 0.7270481 |
| 0.0821510 | 0.0408861 | -0.390386 | -0.695380 | -0.073355 | -0.760842 | -0.233803 | 0.7275734 | -0.163924 |
| -0.618891 | -0.964634 | 0.0202750 | -0.099189 | -0.746651 | 0.0089280 | -0.416408 | -2.163338 | -0.263929 |
| -0.376406 | -0.235016 | -1.357688 | -1.660794 | -2.306998 | -1.457339 | -1.742650 | -2.225893 | -0.098542 |
| -1.264014 | -1.662894 | 0.1765503 | -1.096879 | -0.381606 | -0.928109 | -1.130740 | -0.352764 | -0.287364 |
| -0.546087 | -0.231845 | 0.3805548 | 0.0898018 | -0.578566 | -0.102889 | -0.279443 | -1.782140 | -0.133031 |
| 1.5204674 | 3.4136628 | 0.0961194 | 1.5021204 | -0.769388 | 0.1016454 | 3.7283080 | 0.2712611 | 0.0507265 |
| 0.2032895 | -0.014256 | -0.641202 | -0.821571 | -0.132405 | -0.762657 | -0.720207 | 0.0639644 | -0.264536 |
| -0.186086 | -0.634712 | -0.295040 | -1.053309 | 0.1966568 | -1.104488 | -0.906634 | -0.776033 | -0.699457 |
| 0.0943241 | 0.0969942 | -0.233233 | 0.2374208 | 1.0165881 | -0.289167 | 0.5162381 | 0.7121837 | -0.234260 |
| -0.792900 | -1.387784 | -0.630542 | -0.770340 | -2.058250 | -0.514411 | -0.916437 | -1.955568 | -0.445655 |
| -1.620188 | -0.923056 | -0.201329 | -0.427708 | -0.588245 | -0.396009 | -0.504667 | -0.986138 | -0.621717 |
| -0.111811 | -0.273988 | -0.024207 | -0.604733 | -1.235561 | -0.762073 | -1.022012 | -2.068742 | 0.1331562 |
| 0.1509006 | 0.2930824 | -0.005371 | 0.7847277 | 1.1863495 | 0.5671830 | 0.8036642 | 1.8629519 | -0.372654 |
| -0.485019 | -0.367539 | -0.395946 | -0.625024 | -0.328605 | -0.374610 | -0.396649 | -0.023069 | -0.317472 |
| 0.0230647 | 0.0735504 | -0.244175 | -0.732799 | -0.125235 | -0.260478 | -0.526659 | -0.348190 | -0.214416 |

| | | | | | | | | |
|---|---|---|---|---|---|---|---|---|
| -0.179255 | 0.4470825 | -0.336727 | -0.949327 | -0.649404 | -0.677751 | -0.655655 | -0.363826 | -0.183142 |
| -0.414324 | -1.345414 | -0.483028 | -0.663415 | -1.775639 | -0.399524 | -0.703364 | -2.400273 | -0.585218 |
| -0.292430 | -0.234623 | -0.495895 | -0.161162 | 0.1580797 | -0.597445 | -0.143981 | 0.1903467 | -0.526043 |
| -0.223652 | 2.7565006 | -1.626268 | 0.4861782 | -0.797708 | -1.428034 | 1.6903091 | -0.313770 | -0.751861 |
| -0.551770 | -0.499914 | -0.856552 | -1.105244 | -0.649320 | -1.164697 | -0.699612 | -0.049090 | -0.774022 |
| -0.430564 | -0.006926 | -0.284733 | 0.1502537 | 0.1514263 | -0.107260 | 0.1041742 | 0.1778128 | -0.569287 |
| -0.344549 | -0.547303 | -0.153567 | -0.423712 | 0.1541093 | -0.302106 | -0.816533 | -0.500991 | -0.390648 |
| 0.4600745 | 1.7919375 | -0.732557 | -0.205952 | 0.9532087 | -0.727099 | 0.7286029 | 2.6060300 | -0.622627 |
| 1.8394316 | 2.8200084 | -0.431996 | 1.2401142 | -2.726134 | -0.666195 | 3.6888190 | -1.413975 | 0.1082819 |
| -0.728467 | -0.572150 | -0.860494 | -0.517047 | -0.099267 | -0.869930 | -0.592566 | -0.054885 | -0.711346 |
| -0.856233 | -1.455045 | -0.623941 | -1.215642 | -1.207793 | -0.967185 | -1.346011 | -1.627532 | -0.626939 |
| 1.1197011 | 3.5490552 | -0.269313 | 2.5675682 | 0.6750356 | 0.6912521 | 2.9782230 | 3.1673057 | -0.235625 |
| 0.2570150 | 2.3965769 | -0.898049 | 0.7095552 | 0.5904010 | -1.245888 | 0.8841603 | 1.1650353 | -0.332516 |
| -0.773561 | -1.711218 | -0.572497 | -0.364352 | -0.296648 | -0.489723 | -0.530030 | -0.544170 | -0.554945 |
| -0.661544 | -1.378691 | -0.707706 | -1.879695 | -1.106530 | -1.119878 | -2.237595 | -2.334932 | -0.363175 |
| -0.617630 | -1.219898 | -0.635912 | -1.373883 | -1.055225 | -1.049871 | -1.195906 | -0.778676 | -0.474736 |
| -0.248187 | -1.316388 | 0.0654459 | -0.398280 | -1.458254 | -0.153060 | -1.039044 | -2.420091 | -0.181761 |
| -0.332833 | -0.879820 | -0.377279 | -0.669173 | -0.883610 | -0.498206 | -0.859783 | -0.827565 | -0.237510 |
| 0.2275566 | -0.126725 | -0.273600 | -1.144504 | -1.254926 | -0.944709 | -1.640508 | -1.483310 | -0.074102 |
| -0.038446 | -0.206776 | 0.3102964 | -1.719787 | -1.433224 | -2.287739 | -1.747932 | -1.796246 | 0.0115905 |
| -0.478301 | -1.484721 | -0.204774 | 0.0870820 | 0.3215727 | -0.147365 | -0.077862 | -0.467478 | -0.128424 |
| -1.667151 | -2.099073 | -0.654359 | -0.575411 | -0.353730 | -0.516503 | -1.005871 | -1.242493 | -0.611454 |
| 0.2435319 | 0.0942635 | -0.408874 | 0.1672479 | 0.4066161 | 0.1271493 | -0.149465 | 0.2087551 | -0.021702 |
| 0.1453208 | 0.6268127 | 0.1981130 | -1.606138 | -1.337014 | -1.998650 | -1.813380 | -2.104225 | 0.5271146 |
| -1.189986 | -0.961611 | -0.598369 | -0.799565 | -1.735812 | -0.533307 | -1.037546 | -2.190046 | -0.300648 |
| 0.0600301 | -0.256874 | -0.509321 | -0.580744 | -0.002766 | -0.387342 | -0.644716 | -0.013532 | 0.1541641 |
| -1.334721 | -0.859783 | -0.710747 | -2.204604 | -1.962025 | -0.576297 | -2.774540 | -2.661400 | -0.437129 |
| -0.051398 | 2.0534426 | -0.027389 | 1.7870279 | 2.8420937 | 0.5693970 | 1.7992591 | 3.2009690 | 0.1261055 |
| -0.018273 | 2.6931363 | -0.346425 | -0.143969 | 1.2982991 | -0.617527 | 0.2223133 | 1.4016830 | -0.182269 |
| -0.390649 | -1.240404 | -0.307047 | -0.435927 | -1.506811 | -0.325746 | -0.550398 | -1.958614 | -0.121086 |
| -0.381121 | -1.225682 | -0.192516 | -0.842526 | -0.986248 | -0.406189 | -1.194229 | -1.114495 | 0.0287941 |
| 0.2286478 | -0.411848 | -0.282001 | -1.281258 | -0.744852 | -1.616441 | -1.713740 | -1.165227 | -0.036010 |
| -0.558575 | -1.249334 | -0.542842 | -1.107331 | -1.054805 | -0.739327 | -1.437350 | -1.286945 | -0.448412 |
| -0.610986 | -1.379879 | -0.248075 | -0.648827 | -0.879806 | -0.496744 | -0.701600 | -1.151159 | -0.215458 |
| -0.200592 | -0.585454 | -0.355434 | 0.9423663 | 1.7437923 | 0.2714046 | 1.2846672 | 1.4762416 | -0.423749 |
| -0.727829 | -0.458763 | -0.104908 | -0.057853 | -0.602388 | 0.3456027 | -0.281799 | -1.002955 | -0.345030 |
| -0.224833 | -0.304892 | -0.437208 | 0.0419156 | -0.114188 | -0.328841 | -0.333889 | -0.281311 | -0.263844 |
| -0.146807 | -1.177524 | -0.010762 | -0.127362 | -1.133052 | 0.0708529 | -0.462591 | -1.771991 | 0.0104591 |
| -0.919236 | -0.865484 | -0.370472 | 0.0148276 | -1.533910 | -0.219841 | -0.434629 | -2.338524 | -0.350762 |
| -0.932751 | -2.312842 | -0.258750 | -1.051085 | -1.106847 | -0.192830 | -0.438488 | -1.403454 | -0.299126 |
| -0.664570 | -0.900073 | -0.403036 | -0.392497 | -0.639895 | -0.467963 | -0.324951 | -0.522417 | -0.723575 |
| 0.4505041 | -1.578664 | -0.461162 | -2.065932 | -1.237741 | -0.743837 | -2.466261 | -2.273844 | -0.584153 |
| 0.1419163 | 2.5616772 | -0.441339 | 1.8704071 | 0.7083726 | 0.5952525 | 2.5250789 | 0.6214395 | -0.215013 |
| 0.0822935 | -0.287324 | -0.015631 | -0.196665 | -0.267703 | -0.110743 | -0.362524 | -0.411622 | -0.046922 |
| 0.4049813 | 0.0229532 | -0.188275 | -1.156709 | -0.999228 | -1.142418 | -1.078376 | -0.752223 | -0.099931 |
| -1.316097 | -2.608666 | -0.426792 | -0.797596 | -1.143999 | -0.418333 | -0.694772 | -0.892955 | -0.802465 |
| -0.045641 | 1.7822720 | -0.181479 | 6.2617143 | 2.6678002 | 0.7516196 | 6.8392113 | 8.1125016 | -0.118118 |
| 0.2685926 | 2.4331418 | 0.0497657 | 0.9893501 | 0.3139401 | -0.259815 | 1.3214584 | 1.5133555 | -0.140002 |
| -1.133826 | -0.409077 | 0.1134366 | -0.621827 | -2.575611 | -0.200876 | -0.454912 | -2.690978 | -0.470361 |
| -0.861905 | 0.2650264 | 0.0224695 | 0.2741276 | -0.522629 | 0.0529814 | 0.3745465 | -0.365369 | -0.618377 |

| | | | | | | | | |
|---|---|---|---|---|---|---|---|---|
| -1.228530 | -0.719663 | -1.341737 | -1.803619 | -1.298383 | -1.466089 | -1.820427 | -1.147849 | -0.527191 |
| 0.2553636 | -0.062949 | -0.357404 | -0.207281 | 0.2135231 | -0.155387 | -0.573935 | 0.3307627 | -0.081162 |
| -0.408953 | -1.316885 | -0.514733 | -0.142892 | 0.3255169 | -0.405403 | -0.322570 | 0.2886233 | -0.580785 |
| 0.0619678 | -0.184563 | 0.0702284 | -0.004396 | -0.013333 | -0.145264 | -0.013941 | 0.1415112 | 0.1146695 |
| 0.0614382 | -1.039170 | -0.244892 | -1.145464 | -1.046501 | -0.695605 | -1.559533 | -1.882318 | -0.246918 |
| -0.855280 | -1.157357 | -0.352751 | -0.303592 | -1.270182 | -0.503449 | -0.277420 | -0.715181 | 0.1016547 |
| -0.534596 | -0.782708 | -0.000925 | -0.462768 | -0.243846 | -0.926678 | -0.613230 | -0.742038 | -0.607553 |
| -0.096142 | -0.041887 | -0.200315 | 0.0790312 | -0.064716 | 0.0362753 | 0.1587580 | -0.246643 | -0.503806 |
| -0.511214 | -0.396576 | -0.239445 | -0.578212 | -0.248704 | 0.0674269 | -0.301196 | -0.111210 | -0.035252 |
| 0.0285736 | 0.0372328 | -0.157798 | 0.8017660 | 1.2357330 | 0.2322322 | 0.9713105 | 1.2822274 | -0.270502 |
| -0.593499 | -0.349087 | -0.269620 | -0.550217 | -1.032001 | -0.638997 | -0.568378 | -0.322790 | -0.127983 |
| -0.453421 | -0.442908 | -0.364214 | -0.688617 | -0.224080 | -0.923833 | -1.040586 | -1.407521 | 0.0751612 |
| -0.745883 | -0.713752 | 0.5182245 | 0.5586669 | -0.181073 | 0.6208509 | 0.3127407 | -2.049152 | -0.475307 |
| -0.852511 | -0.312960 | 0.0189624 | -0.152535 | -0.180190 | 0.0239993 | -0.482805 | -1.49905 | -0.079425 |
| -0.612855 | -1.192747 | -0.280259 | -0.536531 | -0.652194 | -0.415093 | -0.842221 | -0.709697 | -0.122708 |
| -0.196502 | -0.118824 | -0.141180 | -0.162647 | -0.177510 | -0.088412 | -0.266994 | 0.0333687 | 0.0093026 |
| -0.519976 | -0.912281 | -0.717683 | -0.750025 | -1.585617 | -0.568708 | -1.071560 | -1.665657 | -0.908024 |
| -0.186334 | 0.0195585 | -0.595971 | -0.589591 | -0.276281 | -0.353588 | -0.667881 | -0.139054 | -0.343557 |
| 0.4032617 | 0.0863737 | -0.607172 | -1.129689 | -0.893133 | -1.102158 | -1.068957 | -1.437740 | 0.0153339 |
| 0.1376285 | 0.4989049 | -0.996109 | -0.666809 | 1.0216474 | -0.135816 | -0.187351 | 1.2135608 | 0.0468490 |
| -0.607665 | -0.872205 | -0.279614 | -0.174068 | -0.534474 | -0.155706 | -0.308077 | -1.033237 | -0.228252 |
| 0.0074118 | -0.581185 | -0.647853 | -0.883122 | -0.304617 | -0.628320 | -0.611431 | -0.346767 | -0.214568 |
| -0.244843 | -0.073894 | -0.011612 | -0.232649 | -0.330328 | -0.109471 | -0.140476 | -0.268072 | -0.248216 |
| -0.221478 | -0.257301 | -0.471864 | -0.691827 | -1.192169 | -0.440322 | -0.704991 | -1.585320 | -0.103637 |
| -0.656952 | -0.694315 | -0.241430 | -0.677519 | -0.933208 | 0.1850831 | -0.591120 | -1.148039 | -0.035361 |
| -0.132768 | -0.750239 | -0.657728 | -0.666339 | -0.751505 | -0.598822 | -0.946305 | -1.074948 | 0.0146669 |
| 0.1387821 | -0.415901 | -0.136667 | -0.603718 | -0.247727 | -0.614920 | -0.903190 | -0.433832 | -0.143617 |
| -0.580841 | -0.434682 | -0.434689 | 0.1901250 | 0.5748080 | -0.091705 | 0.2035112 | 0.9030670 | -0.523700 |
| -1.365460 | -0.707613 | 0.1575569 | -0.454188 | -1.497642 | 0.0513885 | -1.148077 | -2.352248 | -0.067659 |
| 0.0199890 | -0.389370 | -0.598170 | -0.736651 | 0.1853076 | -0.837839 | -0.333267 | 0.5011007 | 0.0568680 |
| -0.361611 | -0.728490 | -0.108496 | -1.094291 | -1.666536 | -0.177325 | -1.281769 | -1.226185 | -0.380466 |
| -1.610030 | -1.850050 | -0.331581 | -0.378655 | -0.710691 | -0.297254 | -0.498090 | -1.113037 | -0.156497 |
| -1.208243 | -0.703522 | 0.2208023 | -0.808879 | 0.0912501 | -0.351069 | -0.558446 | 0.1130741 | -0.279905 |
| 0.4186572 | 0.0032657 | -0.200503 | -0.096834 | -0.440546 | -0.341096 | -0.064196 | -0.775035 | 0.5363591 |
| -0.314495 | -0.741062 | -0.534251 | -0.566051 | -1.463711 | -0.501715 | -0.471039 | -1.925558 | -0.149168 |
| -0.281739 | -0.674311 | -0.124625 | -0.541441 | -0.944461 | -0.493001 | -1.033092 | -1.108169 | -0.096565 |
| -0.093806 | -0.708741 | -0.200710 | -0.429710 | -0.081273 | -0.584967 | -0.396736 | -0.283970 | 0.3393697 |
| 0.0176301 | -0.402119 | -0.413406 | -0.866583 | -0.424184 | -1.075388 | -0.864431 | -0.290068 | -0.060657 |
| -0.565247 | -0.560855 | -0.112747 | -0.091448 | -0.129795 | -0.556985 | -0.104195 | -0.435099 | -0.598867 |
| -1.239094 | -1.266716 | -0.679965 | -0.872598 | -0.366790 | -0.349757 | -1.121920 | -0.055658 | -0.275756 |
| 0.4869915 | -0.711659 | -1.006787 | -1.552800 | -0.155162 | -2.103349 | -1.470000 | -1.746207 | -0.639347 |
| -0.308328 | -0.190839 | -0.155776 | -0.067191 | 0.0103631 | 0.1501401 | 0.0306937 | -0.291707 | 0.1740251 |
| 0.4899310 | 0.3084473 | -0.075506 | -0.604440 | -0.209131 | -0.733835 | -0.791391 | -1.246425 | 0.2763328 |
| -0.173432 | -0.003054 | -0.788099 | -0.952963 | -0.475013 | -0.734963 | -0.821979 | -0.506963 | -0.115902 |
| -0.475624 | -0.532049 | -0.718370 | -0.356755 | -0.440136 | -0.315721 | -0.559585 | -0.357683 | -0.500865 |
| -0.511611 | 0.2248223 | -0.160711 | 0.1006107 | -0.002574 | -0.228524 | 0.1422503 | 0.2906964 | -0.395489 |
| -0.370445 | -0.948992 | -0.480835 | -0.776923 | -0.563084 | -0.838647 | -0.485803 | -0.590052 | -0.632441 |
| -0.293213 | -0.329277 | -0.763769 | -0.246388 | 0.1264940 | -0.809022 | 0.2163418 | 0.3905232 | -0.267763 |
| -0.510173 | -1.867602 | -0.938241 | -1.841890 | -3.202813 | -1.395808 | -2.307763 | -3.198062 | -0.106053 |
| -1.097489 | -1.506994 | -0.924816 | -0.707247 | -1.384591 | -1.088664 | -0.737447 | -1.975109 | -0.608269 |

| | | | | | | | | |
|---|---|---|---|---|---|---|---|---|
| 0.5390208 | 0.3306984 | 0.1827148 | -1.1272148 | -0.2983662 | -1.1135436 | -1.0114701 | -0.2146405 | 0.3442200 |
| -1.3001210 | -1.8212561 | -1.0512173 | -1.3686663 | -1.7550482 | -1.1233078 | -1.6102431 | -2.1897222 | -0.6434336 |
| -0.4436260 | -0.7240094 | -0.7402900 | -0.4242129 | -0.1805546 | -0.8287524 | -0.4175842 | -0.1307755 | -0.4881502 |
| -0.9459275 | -0.5281599 | -0.3103808 | -0.3282152 | -0.3602596 | -0.6398640 | 0.1254773 | -0.1170898 | -0.5518653 |
| -0.4319394 | -0.8727348 | -0.5469032 | -1.3806558 | -1.9085485 | -1.1026600 | -1.6274928 | -2.1120257 | 0.2058280 |
| -0.1877406 | 1.4475228 | -0.2283318 | 2.7155316 | 1.5483206 | 1.4760057 | 2.7214111 | 1.7737432 | -0.3429617 |
| -0.2029239 | -0.3180664 | -0.5197034 | -0.7445436 | -0.4974169 | -0.8823202 | -0.9191284 | -0.6392200 | -0.3026098 |
| -0.1717558 | -1.2641224 | -0.5675726 | -0.0676848 | -0.3742033 | -0.3961070 | -0.4115156 | -0.9569880 | -0.5235768 |
| -0.3735311 | -1.1774168 | -0.4784649 | -0.6128059 | -1.4627184 | -0.4757553 | -1.1621056 | -1.3201038 | -0.3333821 |
| -0.7123642 | -0.9378733 | -1.4545190 | -0.9949085 | -0.3374393 | -1.1386591 | -0.8573210 | -0.1591660 | -0.4348498 |
| 0.1016647 | 0.1955320 | -0.2533074 | -0.0970366 | 0.1773472 | -0.3804250 | -0.0194069 | 0.5365036 | -0.3411673 |
| -0.9825930 | -1.9766661 | -0.1400708 | -0.6056784 | -0.8303991 | -0.4704918 | -0.7928947 | -0.9256241 | -0.4044354 |
| -1.7052412 | -1.5297525 | -0.2426464 | -1.0069135 | -1.0318881 | -0.2994591 | -1.3418989 | -1.2841636 | -0.1104986 |
| -0.2876470 | -0.1511502 | -0.2674628 | -0.6140800 | -0.4567402 | -0.0229631 | -0.5881036 | -0.0527420 | -0.2921553 |
| -0.1608220 | -0.2869305 | -0.1938275 | -0.2058404 | -0.2586290 | -0.1724416 | -0.1364298 | -0.0687672 | -0.0239631 |
| -0.9175557 | -0.8592158 | -0.0316041 | -0.7491913 | -1.5855805 | -0.5248575 | -1.0642846 | -1.8287252 | 0.5435430 |
| -0.4106973 | -0.6671897 | -0.6762366 | -0.0923885 | 0.5082195 | 0.3042669 | 0.3842891 | 0.7581235 | -0.6487253 |
| -0.6407244 | -1.6248399 | -0.4692376 | -1.0113305 | -0.6279007 | -0.7839275 | -1.4965688 | -1.4267499 | -0.4402440 |
| -0.7468012 | -0.7626976 | -0.4805426 | -0.7113560 | -0.8139221 | -0.9926733 | -0.7903764 | -0.5142275 | -0.5066549 |
| -0.4401059 | -0.8542073 | -0.4192471 | -0.6504868 | -1.1371924 | -0.7866047 | -0.8232109 | -1.4508340 | -0.2051174 |
| -0.3356532 | 0.0859175 | 0.0088491 | 0.2160908 | 0.5220201 | 0.1569383 | 1.2519273 | 1.6161874 | -0.2536059 |
| -0.2705450 | 0.0254321 | -0.3210982 | -0.9004595 | -0.7991202 | -1.1070533 | -1.1584895 | -0.5972335 | 0.0642193 |
| -0.9404277 | -2.4978629 | -0.6667956 | -1.1316350 | -1.2614133 | -0.5092782 | -1.3050186 | -1.2537073 | -0.3630913 |
| -0.6306454 | -0.5667774 | -0.8935245 | -0.4213016 | -0.4308643 | -0.5200082 | -0.6948091 | -0.5883336 | -0.2613369 |
| -0.6818266 | -1.0932984 | -0.3461609 | -0.6785170 | -1.2201974 | -0.3291086 | -0.7759876 | -1.9635961 | -0.2773993 |
| -0.7691383 | -1.1393561 | -0.1754398 | -0.7230807 | -0.3137841 | -0.3392549 | -0.8072291 | -0.2099589 | -0.4579353 |
| -1.4998036 | -1.8774708 | -0.6722619 | -0.9255388 | -1.2983219 | -0.5719116 | -1.1739653 | -2.0496373 | -0.4431381 |
| 0.0237143 | -0.1644445 | -0.0498679 | -0.4118417 | -0.6277508 | -0.2839035 | -0.7657528 | 0.0056819 | -0.0369246 |
| -0.9684872 | -1.3607711 | -0.4333441 | -0.9095913 | -1.4180645 | -0.4530166 | -1.1890843 | -1.1277558 | -0.3384100 |
| -0.1764652 | 0.5591051 | -0.1093405 | 0.6219992 | 1.0460775 | -0.0048062 | 0.6333066 | 1.5170793 | -0.0370822 |
| 0.0182405 | 2.0292994 | -0.6925340 | 0.7305249 | 2.1647548 | 0.1870583 | 1.1230370 | 3.1970325 | -0.3143939 |
| -0.1047723 | -0.8684013 | -0.6527861 | -1.3673414 | -0.9799014 | -0.6778328 | -1.1918467 | -0.6431946 | -0.4893484 |
| -0.0218655 | -0.2438881 | -0.0287649 | -0.1100612 | 0.0112922 | -0.2204848 | -0.0906857 | 0.0462469 | 0.2013125 |
| 0.4078167 | 0.4817347 | -0.1347108 | 0.0680080 | 0.3502021 | -0.4366086 | -0.1371241 | -0.0375620 | 0.2846107 |
| 0.6452193 | 1.0391854 | 0.1994826 | 1.3702934 | 1.7989420 | 0.8577606 | 1.597451 | 1.4066787 | 0.1017265 |
| -0.2093691 | -0.3885476 | -0.2730656 | -0.4644028 | -0.9565541 | -0.4812028 | -0.6496423 | -1.0995858 | -0.2777224 |
| 0.6273910 | 1.1032912 | 0.1660206 | -1.4273325 | -1.1879328 | -1.2376446 | -0.8707882 | -1.0585971 | 0.5713875 |
| 0.2755516 | 0.7278905 | -0.5903201 | -0.3845533 | -0.5922648 | -0.9023062 | -0.3256979 | 0.1248225 | 0.0641472 |
| -0.1771800 | -0.1997977 | -0.5534029 | -0.8970922 | -0.7503803 | -0.8448020 | -0.8903215 | -0.2422560 | -0.0387185 |
| -0.6931336 | -0.3113729 | -0.4851434 | -1.2400337 | -1.7258017 | -1.2545766 | -1.4702482 | -2.3839417 | -0.2103778 |
| -1.3727014 | -0.8748672 | -0.4131859 | -1.1880884 | -0.5335272 | -0.5421880 | -1.2539496 | -1.5732229 | -0.2449179 |
| -0.4540893 | -0.1809600 | -0.6151393 | -0.1138650 | 0.3444411 | -0.3678610 | 0.1583336 | 0.5679315 | -0.3428151 |
| 0.8355009 | 1.6547953 | -0.5643977 | -0.0770362 | 0.3150356 | -0.9139242 | -0.1376676 | 0.5858476 | -0.1131615 |
| -0.0117931 | -0.0646725 | -0.1863680 | -0.0935094 | 0.5735911 | -0.5107582 | 0.0261069 | 0.6263125 | 0.0413550 |
| -0.3125441 | -0.9224881 | -0.2694733 | -0.3885065 | -0.5011706 | -0.3731313 | -0.6804433 | -0.5097753 | -0.0245379 |
| 0.0308472 | -0.2292589 | -0.2046855 | -0.5270163 | -0.1523257 | -0.6846115 | -0.4154875 | 0.1243026 | 0.2663135 |
| -1.0501393 | -1.3951296 | -0.4547008 | -0.4301809 | -0.6235863 | -0.4252690 | -0.8216825 | -1.2618112 | -0.7119887 |
| -0.3806481 | -0.7063075 | -0.2674617 | -1.2359935 | -1.2771488 | -0.7931662 | -1.4028747 | -1.7125194 | -0.3048605 |
| -0.9968125 | -0.6429148 | 0.0742114 | -0.1249570 | -1.3700542 | -0.2836241 | -0.4720299 | -1.9043894 | -0.1878187 |
| -0.4070539 | -0.3643061 | -0.5198009 | -0.7940890 | -0.3070332 | -1.0696024 | -0.9594116 | -0.9042473 | -0.1460878 |

| | | | | | | | | |
|---|---|---|---|---|---|---|---|---|
| -0.800498 | 0.3014519 | -0.272758 | -1.899728 | -1.158235 | -2.004964 | -1.900247 | -1.133833 | 0.3029170 |
| -0.589239 | -0.437513 | -0.855411 | -0.334515 | -1.426680 | -0.370566 | -0.023211 | -0.974941 | -0.397209 |
| -0.519077 | -0.474348 | -0.506324 | -0.700082 | -0.323465 | -0.791449 | -0.268334 | -0.940442 | -0.510581 |
| -1.059539 | -0.915061 | -0.319885 | -0.639811 | -0.786620 | -0.383323 | -0.657961 | -1.815771 | -0.759262 |
| -0.948643 | -1.240567 | -0.865324 | -0.804108 | -1.293468 | -0.878734 | -1.279302 | -1.581608 | -0.176341 |
| -0.201115 | -0.057516 | -0.248734 | 0.2451926 | -0.443316 | -0.074531 | 0.3322125 | -0.557220 | -0.142418 |
| 0.2339788 | 1.5549964 | -0.035616 | 1.5096810 | 1.7327409 | 0.4849595 | 1.5584210 | 1.3569080 | -0.521883 |
| 0.0905441 | -0.484486 | -0.202808 | -0.383680 | 0.1075226 | 0.0623322 | -0.190359 | 0.0595452 | -0.127704 |
| 0.0941721 | -0.468844 | 0.5129271 | -0.850387 | -1.566868 | -0.745446 | -1.020221 | -2.329282 | 0.0424780 |
| -1.149936 | -1.596713 | -0.942759 | -0.432379 | -0.018289 | -0.627848 | -0.357580 | 0.0566222 | -0.416727 |
| -0.655242 | 0.4696193 | -0.371406 | -1.162250 | -0.107788 | -1.087853 | -1.151819 | -0.696299 | -0.535919 |
| -0.609088 | -1.134330 | -0.493497 | -0.924630 | -0.719388 | -1.045808 | -0.521057 | -0.639946 | -0.634669 |
| -0.373667 | -0.239664 | -0.339124 | 0.0940212 | -2.331412 | 0.2748097 | -0.271927 | -2.664882 | 0.1100860 |
| -0.522587 | -0.574403 | -0.585920 | -0.825089 | -1.085353 | -0.613314 | -0.771631 | -1.068801 | 0.3699673 |
| -1.092118 | -1.966539 | -0.151384 | -0.535523 | -1.540796 | -0.271506 | -0.658055 | -1.866815 | 0.1033608 |
| -0.748702 | -0.491542 | -0.179104 | -0.395491 | -0.432269 | -0.135451 | -0.309146 | -0.729463 | 0.0453396 |
| -0.029610 | -0.405887 | 0.0598191 | -1.174539 | -2.500185 | -1.556970 | -1.637395 | -3.303142 | 0.0395765 |
| -0.578577 | -1.598383 | -0.237147 | -0.993013 | -0.200106 | -0.254632 | -0.426352 | 0.3486894 | -0.205892 |
| -0.513623 | -2.453951 | -0.528500 | -0.965693 | -1.468697 | -0.345887 | -1.214393 | -1.895598 | 0.0556314 |
| 0.4064818 | 0.3719029 | 0.4097267 | -1.204423 | -0.647997 | -1.062680 | -1.434069 | -1.408680 | 0.3297943 |
| -0.737731 | -0.441402 | -0.842001 | -1.092754 | -1.785217 | -0.942468 | -0.805690 | -1.692311 | -0.232307 |
| -1.368879 | -1.543675 | -0.541763 | -1.157217 | -2.030480 | -0.763353 | -1.267088 | -2.101132 | -1.082389 |
| -1.366207 | -2.351949 | -0.294009 | -0.713781 | -2.872641 | -0.188689 | -1.212147 | -3.388173 | -0.719330 |
| -0.102747 | -0.350849 | -0.201619 | -0.409505 | -0.113786 | -0.363033 | -0.500931 | 0.0101518 | -0.023305 |
| 0.0160460 | -0.157395 | -0.192315 | -0.470902 | -0.644388 | -0.113176 | -0.595171 | -1.212963 | 0.8036704 |
| -0.464801 | -0.837260 | -0.515186 | -0.331251 | -0.237840 | -0.320838 | -0.443517 | -0.175016 | -0.431172 |
| -0.201708 | -0.442530 | 0.2996457 | -0.120932 | -0.907046 | -0.076573 | -0.195325 | -0.874222 | -0.253908 |
| -0.586386 | -0.596899 | -0.556072 | -0.431158 | -1.462931 | -0.671019 | -0.699180 | -1.616420 | -0.578915 |
| -0.650802 | -0.116900 | -0.419691 | -0.044986 | 0.2318039 | -0.258619 | -0.260004 | 0.4550572 | -0.327623 |
| -0.442812 | 0.0536085 | -0.341296 | -1.033885 | -0.108210 | -1.311930 | -0.791051 | -0.069610 | -0.172243 |
| -0.609327 | -1.224478 | -0.395348 | 0.1654848 | -0.082445 | -0.072614 | 0.0113865 | -0.552421 | -0.758915 |
| -0.351814 | -0.531647 | -0.061333 | -0.728961 | -0.878041 | -0.547048 | -0.808263 | -0.846475 | -0.140551 |
| -0.798430 | -0.806163 | -1.220757 | -1.071871 | -0.568768 | -0.672513 | -0.647832 | -0.761597 | -0.431461 |
| -0.273755 | -1.119811 | -0.018646 | -0.210310 | -0.914211 | -0.204601 | -0.462499 | -1.242571 | -0.262253 |
| 0.1350401 | 0.2005605 | -0.734351 | -0.822412 | -1.212092 | -0.524088 | -0.740775 | -1.601093 | -0.251103 |
| -1.130475 | -1.679425 | -0.300317 | -0.335555 | -0.162412 | -0.294614 | -0.477976 | -0.919227 | -0.543110 |
| -1.476583 | -1.636368 | -0.171496 | -0.470960 | -0.776366 | -0.210738 | -0.645845 | -1.361055 | -0.411759 |
| -0.730917 | -0.481661 | -0.438825 | -1.325289 | -3.472591 | -0.601146 | -1.916219 | -4.770833 | 0.1720216 |
| -0.169642 | 0.4685353 | -0.774280 | 0.6735793 | 0.6228871 | -0.407294 | 0.7957091 | 0.3089172 | -0.838877 |
| -0.779694 | -0.975567 | -0.502041 | -0.571968 | -0.744029 | -0.581425 | -0.871185 | -1.407865 | -0.361351 |
| -0.057745 | -0.510401 | 0.3582542 | -1.192995 | -1.289775 | -1.249059 | -1.248543 | -1.296904 | 0.0925805 |
| -0.173545 | -1.033227 | -0.539759 | -1.320003 | -1.283930 | -1.005443 | -1.837892 | -2.143982 | -0.176053 |
| -0.446079 | -0.237046 | -0.519410 | -0.595018 | -0.721693 | -0.598443 | -0.678402 | -0.632043 | -0.260058 |
| -1.310542 | 0.0069944 | -0.585430 | -1.156684 | -0.993708 | -0.967904 | -1.240179 | -1.310939 | 0.0581138 |
| -0.359384 | -0.150438 | -0.465479 | -0.312348 | -0.067774 | -0.440336 | -0.107377 | 0.1817582 | -0.341113 |
| -1.197362 | -1.307109 | -0.499445 | -1.298331 | -1.005750 | -1.306872 | -1.792926 | -1.314508 | -0.179933 |
| 0.0491604 | -0.363452 | -0.636859 | -1.178688 | -1.012639 | -0.819430 | -1.137129 | -1.189922 | -0.174661 |
| -1.846522 | -1.909777 | -0.745849 | -1.267270 | -0.601598 | -1.152183 | -1.489329 | -1.633312 | -0.708862 |
| -0.183757 | -0.199321 | -0.225152 | -0.826696 | -0.123484 | -1.079267 | -0.708758 | 0.7453445 | -0.128412 |
| -1.055062 | -1.479511 | -0.573438 | -0.920592 | -0.882604 | -0.828059 | -1.058503 | -1.018691 | -1.042896 |

| | | | | | | | | |
|---|---|---|---|---|---|---|---|---|
| -0.3081639 | 0.0986814 | -0.2668153 | -0.3949037 | 0.1829566 | -0.6360459 | -0.3526281 | 0.9623694 | 0.0416141 |
| -0.1473517 | -0.6868308 | -0.4105438 | -0.5904814 | -0.3883602 | -0.3424580 | -0.4129699 | 0.6497457 | -0.4830056 |
| 0.0079870 | -0.4366241 | -0.5031344 | -0.2175114 | 0.2572062 | -0.3885383 | -0.3304695 | 0.3163595 | -0.1403559 |

| PG_381 | PG_382 | PG_383 | PG_384 | PG_385 | PG_386 | PG_387 | PG_388 | PG_389 |
|---|---|---|---|---|---|---|---|---|
| -0.8972335 | -0.7035911 | -0.2049286 | -0.7252734 | -0.9078218 | -0.4037961 | -0.2195786 | -1.0129386 | -0.2864584 |
| -0.7768928 | -1.0981063 | -0.7155171 | -0.8839080 | -1.3662212 | -0.4811878 | -0.8326345 | -1.9661860 | -0.6357764 |
| -1.5788924 | -2.7895152 | -0.8572833 | -1.7944576 | -3.5655605 | -0.3644041 | -0.8562544 | -1.9090685 | -0.4531226 |
| -0.0636555 | 0.3789679 | 0.1962095 | -0.1076379 | 0.1216968 | -0.2573999 | -0.1009118 | -1.0729787 | -0.3261937 |
| -0.1641842 | -0.4057260 | -0.0430897 | 0.1686329 | -0.5090685 | -0.0936781 | 0.1012092 | -0.1818698 | -0.0837553 |
| -0.0946288 | -0.6292212 | -0.3692769 | -0.4240131 | -0.7277860 | -0.5793314 | -0.9002666 | -2.1146257 | -0.2861193 |
| -0.2108430 | -0.1673524 | -0.0233193 | -0.6953501 | -0.0717841 | -0.5486601 | -0.9685161 | -1.3852008 | -0.3924859 |
| -0.5852860 | -1.4532993 | -0.3174407 | -0.6623142 | -1.5770631 | -0.1335615 | -0.0578225 | -1.6567756 | 0.0332772 |
| -0.0078629 | -0.0625825 | -0.2213128 | -0.1158170 | 0.4194324 | -0.0933881 | -0.0427124 | -1.0655369 | -0.3651950 |
| -1.1438562 | -2.1476535 | -0.7518498 | -1.5646052 | -2.2022085 | -0.1569914 | -1.1252797 | -1.8295252 | -0.5609045 |
| -0.0548165 | -0.1583343 | -0.0579964 | -0.0631770 | -0.4367876 | -0.0092125 | -0.1476701 | -0.9387715 | 0.0575563 |
| -0.2102492 | -0.2383055 | -0.2377712 | -0.3803078 | -0.2884048 | -0.1863021 | -0.2619598 | -0.7599935 | -0.2086989 |
| -0.6900619 | -0.8038781 | -0.5685390 | -2.2997223 | -1.7083256 | -0.4218576 | -0.8825546 | -2.7484050 | -0.5074225 |
| -0.0529373 | 1.7933945 | -0.8519754 | 1.0260208 | 2.3731987 | -0.5549821 | 1.3456332 | 4.4326933 | -0.1197557 |
| -0.2503380 | 0.7714423 | 0.1602740 | -0.3739322 | 1.2342201 | -0.0827269 | 0.3143008 | 0.3906190 | -0.0403895 |
| 1.8087963 | 1.7647569 | -0.1621801 | 2.4017938 | 1.9957550 | -0.0398074 | 2.3874682 | 2.5722644 | 1.0808369 |
| -2.6511659 | -1.6053112 | -0.5418904 | -2.8501345 | -2.2362327 | -1.3981112 | -1.8141729 | -1.8293305 | -1.5032578 |
| -0.5937104 | -0.4905345 | 0.1542606 | -0.6808154 | -0.2901538 | -0.633721 | -1.3612420 | -0.2235974 | -0.7614223 |
| -1.5810064 | -2.4005679 | -1.4409116 | -1.3926735 | -2.1743219 | -0.7897979 | -1.3500710 | -2.1585198 | -0.8831601 |
| -1.0521174 | -2.3730304 | -1.1545496 | -2.1466027 | -2.5987722 | -0.7131940 | -1.1423558 | -1.0502013 | -0.8537906 |
| 0.8776091 | 0.6829793 | -0.0617682 | 0.7307332 | 0.7770925 | -0.1614409 | 0.0781596 | 0.0918345 | -0.1348478 |
| -0.6522240 | -0.1477313 | -0.5525487 | -0.4460516 | -0.4959080 | -0.4978696 | -0.3414246 | -0.5542451 | -0.4955167 |
| 0.6395349 | 0.9072150 | -0.0106718 | 0.6433362 | 1.2263817 | -0.0077731 | 0.6788734 | 1.7733526 | 0.1885374 |
| -0.8663898 | -2.1543551 | -0.3595284 | -1.3275746 | -2.2040468 | -0.3828797 | -0.4180369 | -1.3781352 | -0.4692069 |
| -0.6645167 | -2.2838400 | -0.5017714 | -1.2468154 | -2.7602113 | -0.3797085 | -1.1530738 | -3.1152497 | -0.6913983 |
| 0.9235621 | 1.0736209 | -0.0030314 | 1.1184580 | 1.0748966 | -0.2003034 | 0.3106625 | 1.0363999 | -0.1478492 |
| 0.2177119 | 0.0358333 | 0.0889316 | 0.2064664 | -0.0089268 | -0.0149706 | -0.1201727 | -0.4376618 | -0.1859571 |
| -0.8717837 | -1.8080925 | -0.6325602 | -1.2165956 | -2.2898264 | -0.9513534 | -0.9891315 | -2.3333507 | -0.8834837 |
| -0.5645515 | -2.0547551 | -0.2121933 | -1.0333181 | -2.7666597 | 0.0999667 | -0.4082894 | -1.5770891 | 0.0507332 |
| -0.0199152 | -0.2763181 | 0.1142759 | -0.2813082 | -0.1605612 | -0.3758859 | -0.4335853 | -1.1392539 | -0.3039611 |
| 0.3513238 | -0.4007997 | 0.8132684 | -0.0127284 | -0.6808489 | -0.9988386 | -1.0878535 | -1.7065815 | -0.9154964 |
| 0.3634049 | 0.1334596 | 0.4859192 | -0.0609220 | 0.0339123 | -0.1650901 | 0.0978039 | 0.0636954 | 0.0502038 |
| -0.3020040 | 0.6230866 | -0.0311430 | 0.5286712 | 1.0935837 | -0.4539319 | -0.4253807 | 0.7966672 | -0.4840778 |
| -0.3306828 | -0.8965941 | -0.2382474 | -0.6210513 | -2.1537176 | 0.1042825 | -0.2219617 | -1.8441473 | 0.1037898 |
| -0.0578010 | -0.1476240 | -0.1414775 | -0.0469264 | -0.1106273 | -0.3457021 | -0.4065163 | -0.9262957 | -0.3720649 |
| -0.7694587 | -0.7331043 | -0.7613038 | -1.1989349 | -1.1899754 | -0.7654446 | -2.1677110 | -0.9936443 | -1.3823365 |
| -0.3825908 | -0.7302667 | -0.7082940 | -1.4192334 | -1.9230240 | 0.1057828 | -0.3089150 | -1.7164751 | 0.1224229 |
| 1.5361677 | 1.0466268 | 0.1976802 | 3.3370408 | 0.6860310 | -0.3400396 | 2.4094754 | -0.1721399 | 0.8704004 |
| -0.9345561 | -0.4568241 | -0.1972949 | -0.2125375 | -0.2280299 | -0.5902327 | -0.8081921 | 0.0566849 | -0.9598964 |
| -1.4311195 | -0.7001216 | -1.7604504 | -1.4364170 | -1.1691706 | -0.9073118 | -1.3304702 | -0.0235068 | -1.1685211 |
| -0.2016067 | 0.6014561 | -0.2470115 | 0.2289054 | 0.5494427 | 0.1878396 | 0.2984995 | 1.5946436 | 0.1158096 |
| -0.6140824 | -2.5340566 | -0.5158498 | -0.7210058 | -2.8607071 | -0.2509850 | -0.5668718 | -1.7503994 | -0.2565361 |
| -0.5887284 | -0.9681454 | -0.2571986 | -0.6785484 | -0.9217915 | -0.3504855 | -0.6854312 | -0.5734422 | -0.2507978 |
| -0.1070876 | -0.5434696 | 0.0824972 | -0.0861064 | -0.7864328 | -0.0573800 | 0.0336844 | -1.0907429 | 0.0283976 |
| 0.2367262 | 1.0540581 | 0.0220728 | 0.4448254 | 1.0730596 | -0.1910759 | 0.5792628 | 0.7179701 | -0.2148783 |
| -0.2090266 | 1.4390402 | -0.1497758 | 0.2306669 | 3.2533792 | -0.0900606 | -0.1594010 | 2.0485403 | 0.1476895 |
| -0.4748927 | -0.7252212 | 0.0498791 | -0.0890832 | -0.5605311 | -0.0038694 | 0.0548465 | 0.0368652 | -0.0986738 |

| | | | | | | | | |
|---|---|---|---|---|---|---|---|---|
| 0.0032641 | 0.3373755 | -0.097542 | 0.1944359 | 0.154032 | -0.310289 | -0.288032 | -0.542495 | -0.371852 |
| -0.737636 | -1.746076 | -0.503391 | -0.881591 | -2.393390 | -0.477429 | -0.703491 | -1.438271 | -0.540268 |
| -0.108546 | 0.0539588 | -0.431009 | 0.1322595 | -0.038321 | -0.458914 | -0.017128 | 0.2141300 | -0.341354 |
| 0.1945258 | 0.5157933 | -0.971628 | 1.1574050 | 0.6661201 | -0.267500 | 1.4158383 | 1.7514889 | -0.098299 |
| -0.432730 | -0.188059 | -0.898770 | -0.355547 | -0.257235 | -0.403111 | -2.107748 | -2.741615 | -0.536673 |
| -0.232895 | -0.096253 | -0.453584 | -0.146896 | 0.2134440 | -0.311825 | -0.153504 | 0.2400672 | -0.468333 |
| -0.436656 | -0.205561 | -0.519134 | -0.924671 | -0.851841 | -0.564693 | -1.000511 | -1.931368 | -0.712096 |
| 0.7588738 | 2.0099937 | -0.434218 | 1.6531128 | 2.2107331 | -0.166293 | 0.1458136 | 0.8951047 | 0.0886171 |
| 0.1400019 | -1.842173 | 0.0387850 | 0.7153110 | -2.707832 | -0.179616 | 1.8657041 | -1.015198 | 0.9075849 |
| 0.1627856 | -0.255403 | -0.842326 | -0.052666 | -0.150469 | -0.820105 | -0.539209 | -0.660264 | -0.740371 |
| -0.809253 | -1.571719 | -0.692624 | -1.165566 | -1.812286 | -1.228048 | -1.415536 | -1.179413 | -1.164320 |
| 3.3915082 | 2.8319532 | 0.7969066 | 3.8794484 | 3.2689589 | 0.4123364 | 3.2209792 | 3.2356876 | 1.9989096 |
| 2.1242740 | 1.6885385 | 0.4976741 | 2.3481309 | 1.9953910 | -0.189767 | 1.5611359 | 1.7719596 | 0.4044521 |
| -0.536300 | -0.819663 | -0.476462 | -0.756579 | -0.964914 | -0.301797 | -0.435024 | -1.754631 | -0.360589 |
| -0.750007 | -0.258426 | -0.571636 | -1.391669 | -1.501872 | -0.532235 | -1.184520 | -1.577585 | -0.939801 |
| -0.853433 | -1.012218 | -0.567910 | -0.995142 | -1.080136 | -0.958232 | -0.985650 | -0.738510 | -0.912583 |
| -0.462116 | -1.678704 | -0.186310 | -0.982904 | -2.257310 | -0.466107 | -0.528571 | -1.883991 | -0.420592 |
| -0.196313 | -0.867276 | -0.252634 | -0.638537 | -0.694452 | -0.292792 | -0.422576 | -0.593486 | -0.249638 |
| -0.084358 | -0.944195 | -0.106968 | -0.323157 | -1.071380 | 0.0637493 | -0.370538 | -0.461700 | -0.242294 |
| -0.183547 | 0.0603286 | 0.0759888 | -0.499024 | -0.062073 | -0.088738 | -0.191916 | -2.004698 | -0.169935 |
| -0.060655 | 0.3203129 | 0.3510864 | 0.0367386 | 0.1708315 | 0.2926499 | 0.4088666 | -0.866198 | 0.4967902 |
| -0.692405 | -0.621926 | -0.980237 | -1.146716 | -1.468977 | -0.595576 | -1.021087 | -1.257137 | -0.516549 |
| -0.082306 | -0.083801 | -0.148313 | 0.0681300 | -0.193241 | 0.2491353 | 0.0230752 | 0.4934690 | 0.1970140 |
| 1.2673151 | 1.3890225 | 0.6667605 | 1.2416875 | 1.2685673 | -0.052715 | 0.4648721 | -0.340416 | 0.3472554 |
| -0.771655 | -1.423820 | -0.199290 | -0.879992 | -1.506440 | -0.320885 | -0.822713 | -1.603634 | -0.420044 |
| 0.0518937 | 0.2089453 | 0.1772337 | -0.032396 | 0.2493329 | -0.377323 | -0.534479 | 0.0090280 | -0.422630 |
| -1.183758 | -2.139396 | -0.606195 | -1.908192 | -2.732324 | -0.839358 | -1.528613 | -0.835627 | -0.714646 |
| 1.1347414 | 2.5942621 | 0.2213002 | 1.2513694 | 2.8286633 | -0.501269 | -0.273631 | 1.3131215 | -0.388741 |
| 0.1707936 | 0.9966423 | -0.441219 | 0.0963010 | 1.1999982 | -0.174318 | -0.053344 | 1.7179827 | -0.110441 |
| -0.207231 | -0.965421 | -0.236091 | -0.595877 | -1.257481 | -0.290048 | -0.021964 | -2.103491 | -0.225404 |
| -0.300385 | -0.019746 | 0.0961140 | -0.322830 | 0.0670872 | -0.422400 | -0.445815 | -0.957906 | -0.342333 |
| -0.128792 | -0.299652 | -0.047333 | -0.171502 | -0.355266 | -0.025886 | -0.040010 | -0.242274 | -0.014841 |
| -0.492224 | -0.984606 | -0.680036 | -0.717199 | -0.923791 | -0.218009 | -0.947781 | -0.777200 | -0.536206 |
| -0.491490 | -1.055624 | -0.349311 | -0.607194 | -1.231881 | -0.376211 | -0.646191 | -1.627522 | -0.521721 |
| 1.1419606 | 1.7665364 | -0.153796 | 1.4004912 | 1.9465233 | -0.140133 | 1.0389627 | 2.0974681 | 0.2644985 |
| -0.565151 | -1.127423 | -0.274505 | -0.783422 | -1.140960 | 0.2558326 | -0.251207 | -0.231656 | 0.2090817 |
| -0.409971 | -0.157052 | -0.425877 | -0.234736 | -0.226418 | -0.282728 | -0.540111 | -0.223090 | -0.087192 |
| -0.214235 | -1.081827 | -0.005945 | -0.656718 | -1.519212 | -0.144209 | -0.486985 | -1.388200 | -0.283797 |
| -0.498416 | -1.583469 | -0.444444 | -0.899752 | -1.810974 | 0.1373519 | -0.221801 | -2.967105 | 0.0707516 |
| -0.009849 | -0.605406 | -0.513794 | -0.187572 | -0.797493 | -0.236256 | -0.442433 | -1.875673 | -0.213867 |
| -0.997628 | -1.664992 | -0.913075 | -1.208274 | -1.645388 | -0.982415 | -1.207831 | -1.464156 | -0.859074 |
| -0.780327 | -1.845268 | -0.582765 | -1.332720 | -2.237800 | -0.441245 | -1.771168 | -1.930005 | -0.860563 |
| 1.1966970 | 1.8714934 | -0.148611 | 2.1311817 | 1.4031448 | 0.9444487 | 3.2488694 | 2.6966286 | 2.5613901 |
| 0.0424275 | -0.233535 | 0.0029423 | -0.084347 | -0.451676 | -0.101026 | -0.144940 | -0.148307 | -0.239733 |
| 0.1788358 | -0.205909 | -0.005391 | 0.3949761 | -0.336729 | -0.236010 | -0.230137 | -0.101986 | -0.431679 |
| -0.977437 | -1.002800 | -0.971015 | -0.957008 | -0.983123 | -1.198686 | -2.012904 | -1.668235 | -1.393713 |
| 4.1491593 | 4.0804636 | 0.2591360 | 5.5436849 | 6.9399621 | 0.1626861 | 6.9204688 | 7.6222737 | 3.9749043 |
| 1.7566688 | 1.9126533 | -0.141910 | 2.3942124 | 2.2350435 | -0.109099 | 1.7317283 | 2.0581202 | 0.4792035 |
| -1.593369 | -2.736775 | -0.994422 | -2.157667 | -3.658624 | -0.209462 | -1.605526 | -1.450427 | -0.493710 |
| -0.476410 | -0.286702 | -0.663940 | -0.372336 | -0.502390 | -0.196051 | -0.250451 | -1.559519 | -0.124477 |

| | | | | | | | | |
|---|---|---|---|---|---|---|---|---|
| -0.959682 | -1.062639 | -0.751506 | -1.278758 | -1.063988 | -0.100492 | -0.714874 | -1.678798 | -0.236466 |
| -0.073629 | 0.0769504 | -0.103388 | -0.065827 | 0.1796624 | -0.060625 | -0.134876 | 0.6167937 | -0.059168 |
| -0.757234 | -0.500510 | -0.411690 | -0.969475 | -0.852059 | -0.176695 | -0.403722 | -0.781942 | -0.320535 |
| -0.057213 | 0.4370372 | 0.1385165 | 0.0074138 | 0.5535640 | -0.066242 | -0.037897 | 0.3732617 | -0.174685 |
| -0.734936 | -1.968959 | -0.501973 | -1.294689 | -2.190009 | -0.567151 | -1.357732 | -1.575067 | -0.758624 |
| -0.816785 | -1.176319 | 0.0945904 | -1.522134 | -1.198683 | -0.729366 | -0.691524 | -1.653270 | -0.563684 |
| -0.518333 | -0.160188 | -0.495660 | -0.876695 | -0.172869 | -0.476706 | -0.479174 | -0.575775 | -0.590774 |
| 0.0344968 | -0.073692 | -0.350188 | 0.1648343 | 0.0790319 | -0.110897 | 0.0831910 | 0.2937695 | 0.0209975 |
| -0.193712 | -0.355608 | -0.095041 | -0.250469 | -0.060762 | 0.0450479 | 0.0009564 | -0.123641 | -0.178435 |
| 0.5419855 | 0.6800487 | 0.0066487 | 0.4280268 | 0.9359303 | -0.068582 | 0.5385575 | 1.0569207 | 0.1563101 |
| -0.367078 | -1.265301 | -0.189111 | -0.430692 | -1.185040 | 0.2291271 | -0.263809 | -0.950858 | 0.1696051 |
| -0.577678 | -0.253119 | -0.755181 | -1.375392 | -1.330777 | -0.174621 | -0.772515 | -2.376594 | -0.603405 |
| -0.715092 | -1.360086 | -0.720298 | -2.265024 | -3.752155 | 0.0545305 | -0.440078 | -3.355210 | -0.045367 |
| -0.728489 | -0.360277 | -0.415577 | -1.356986 | -1.328643 | -0.238570 | -0.725942 | -1.980735 | -0.384125 |
| 0.0191790 | -0.293836 | -0.173455 | -0.234580 | -0.349464 | -0.161695 | -0.377158 | -0.627426 | -0.042385 |
| -0.247742 | -0.397642 | -0.111943 | -0.094308 | -0.155710 | -0.012953 | -0.071823 | -0.090918 | -0.057067 |
| -1.540535 | -1.885266 | -0.977286 | -1.796150 | -1.806579 | -0.531925 | -1.046810 | -1.697737 | -0.978171 |
| -0.053471 | 0.1359060 | -0.526562 | 0.0045300 | -0.289451 | 0.0291606 | -0.099492 | -0.331018 | -0.078437 |
| 0.1068068 | -0.275924 | -0.031247 | -0.446741 | -0.202062 | -0.513983 | -0.622937 | -0.849284 | -0.617449 |
| -0.021825 | 0.9279307 | -0.261463 | 0.0949283 | 1.2466527 | 0.1245368 | 0.1869390 | 1.6712311 | 0.0450684 |
| -0.402044 | -1.057852 | -0.337261 | -0.565573 | -1.064959 | -0.357437 | -0.674884 | -2.090888 | -0.517094 |
| -0.432504 | -0.708900 | -0.352717 | -0.560919 | -0.811560 | -0.500641 | -0.467345 | -0.748733 | -0.333051 |
| -0.388522 | -0.858884 | -0.333305 | -0.775900 | -1.071952 | -0.329845 | -0.211863 | -0.324859 | -0.331661 |
| -0.291144 | -0.518949 | -0.291426 | 0.2079926 | -0.886586 | -0.404560 | -0.597126 | -0.986518 | -0.393356 |
| -0.430311 | -0.347962 | -0.438685 | -0.174461 | -0.245499 | -0.087305 | -0.494179 | -0.328489 | -0.239990 |
| -0.138349 | -0.719733 | -0.079595 | -0.671268 | -0.997235 | -0.300147 | -0.690022 | -0.438181 | -0.586701 |
| 0.0229918 | 0.3796241 | -0.062627 | -0.006099 | 0.4033963 | -0.008363 | 0.0159856 | -0.125297 | -0.100289 |
| -0.374550 | -0.145544 | -0.740497 | -0.112250 | -0.143675 | -0.113567 | 0.1432569 | 0.4298225 | -0.028284 |
| -0.479059 | -0.481276 | -0.073650 | -0.915271 | -0.855490 | 0.1252501 | -0.562560 | -1.656223 | 0.0108342 |
| 0.0969794 | 0.5273789 | -0.168730 | 0.4441166 | 0.6033380 | -0.706673 | -0.601594 | -0.466287 | -0.707886 |
| -1.286320 | -1.743537 | -0.639913 | -1.258695 | -1.369796 | -1.082495 | -1.830279 | -2.042290 | -0.996190 |
| -0.065358 | -1.227992 | -0.318960 | -0.145955 | -1.255044 | -0.410665 | -0.168695 | -0.852454 | -0.158922 |
| -0.897332 | -1.246813 | -0.673199 | -1.119841 | -1.054047 | -0.436812 | -0.509922 | -0.184735 | -0.521672 |
| 0.3339073 | 0.3307653 | 0.6062696 | 0.4696509 | 0.1897232 | 0.4226934 | 0.3435011 | -0.077043 | 0.4265154 |
| -0.384089 | -1.311714 | -0.255183 | -0.589841 | -2.166989 | 0.3089538 | 0.0329508 | -0.838804 | 0.0950457 |
| -0.110565 | -0.821828 | -0.143622 | -0.588300 | -0.887941 | -0.419360 | -0.343266 | -0.841184 | -0.447927 |
| 0.0392534 | 0.3670541 | 0.2680362 | -0.105625 | 0.1894701 | -0.100385 | -0.241266 | 0.0703919 | -0.042113 |
| -0.052882 | -0.012632 | -0.029306 | 0.0117312 | 0.0058110 | -0.398000 | -0.431549 | -0.046947 | -0.348029 |
| -0.321263 | -0.473167 | -0.791596 | -0.674098 | -0.686495 | -0.450610 | -0.412419 | -0.451751 | -0.416979 |
| -0.464609 | -1.350790 | -0.206256 | -1.083501 | -1.411398 | -0.247423 | -1.193231 | -1.367196 | -0.182515 |
| 0.8938271 | 0.4510139 | -0.844539 | 0.8742107 | -0.001001 | -0.073771 | -0.206447 | 0.7627038 | -0.391899 |
| 0.1454585 | 0.1120149 | 0.1771703 | 0.0147439 | -0.206966 | -0.056802 | -0.204298 | -0.794828 | 0.0731721 |
| 0.3631278 | 0.7003307 | 0.3651800 | 0.2691975 | 0.1059714 | -0.030101 | -0.072887 | -0.343687 | 0.0231457 |
| -0.274382 | -0.982659 | -0.061927 | -0.679079 | -1.064218 | -0.369772 | -0.567745 | -1.398538 | -0.382895 |
| -0.426854 | -0.116681 | -0.426357 | -0.277048 | -0.177427 | 0.0815039 | 0.1194602 | 0.1349889 | 0.2723305 |
| -0.121860 | -0.133528 | -0.251298 | -0.209085 | 0.0354766 | -0.444478 | -0.155379 | -0.111392 | -0.230431 |
| -0.479549 | -0.641817 | -0.798090 | -0.296850 | -0.697753 | -0.118413 | -0.406426 | -0.463439 | -0.294828 |
| 0.5125524 | 0.6594091 | -0.078846 | 0.6461340 | 0.7574884 | -0.534275 | 0.1328492 | 0.5349843 | -0.386727 |
| -1.093953 | -3.790647 | -0.405348 | -2.452909 | -4.192517 | 0.0335960 | -0.604283 | -3.338734 | 0.0316283 |
| -0.687048 | -1.729353 | -0.740074 | -0.941099 | -2.424907 | -1.053125 | -0.908458 | -1.531226 | -0.822712 |

| | | | | | | | | |
|---|---|---|---|---|---|---|---|---|
| 0.3187617 | 0.4761211 | 0.3854071 | 0.2703594 | 0.4682379 | -0.051985 | 0.0761488 | 0.4451829 | -0.058587 |
| -1.179210 | -1.871143 | -0.872636 | -1.490653 | -1.976861 | -0.853348 | -1.478295 | -1.749511 | -1.084259 |
| -0.199062 | -0.029849 | -0.496532 | -0.040143 | 0.1513256 | -0.751763 | -0.374515 | 0.2384610 | -0.613038 |
| -0.254790 | 0.4402398 | -0.194380 | 0.7772646 | 0.2540953 | 0.6575378 | 0.5387035 | 1.0728031 | 0.6549047 |
| -0.700750 | -1.970714 | -0.404738 | -0.856303 | -1.857275 | 0.1344810 | -1.102360 | -1.841122 | -0.022980 |
| 1.0063907 | 0.5161685 | -0.074759 | 0.8834191 | 0.6785365 | 0.2170231 | 1.7483609 | 2.0320145 | 0.9404872 |
| -0.226492 | -0.673775 | -0.382066 | -0.165976 | -0.608014 | -0.293235 | -0.413478 | -0.545785 | -0.191058 |
| -0.190419 | -1.341212 | -0.842448 | -0.728440 | -1.542862 | -0.645868 | -1.008130 | -0.849536 | -0.757651 |
| -0.726793 | -1.396591 | -0.512130 | -0.966306 | -1.693019 | -0.292681 | -0.889469 | -1.852388 | -0.278347 |
| -0.873617 | -0.454169 | -0.534330 | -0.957960 | -0.628727 | -0.333400 | -0.405462 | -0.639715 | -0.263869 |
| 0.1682257 | 0.7066978 | -0.162840 | 0.1291622 | 0.7553521 | -0.453644 | 0.1561880 | 0.6268153 | -0.089226 |
| -1.000882 | -1.184660 | -0.613951 | -0.860908 | -1.146422 | -1.099905 | -1.095376 | -1.644373 | -1.066415 |
| -1.018007 | -1.351000 | -0.198422 | -1.534367 | -1.650218 | 0.0707876 | -0.681255 | -1.478059 | 0.1669875 |
| -0.528978 | -0.163233 | -0.220979 | -0.325092 | -0.181220 | -0.531592 | -0.495824 | -1.101979 | -0.678803 |
| -0.285550 | -0.300705 | -0.232438 | -0.182678 | -0.226965 | -0.364693 | -0.243803 | -0.345569 | -0.324195 |
| 0.3711978 | -1.402620 | 0.6447764 | 0.2484308 | -1.297813 | 0.1341527 | -0.116314 | -0.841570 | 0.1026949 |
| -0.747899 | -0.726933 | -0.537812 | -0.991178 | -0.925073 | -0.351707 | -0.257276 | 0.6731580 | -0.405893 |
| -0.457245 | -1.535664 | -0.861082 | -1.346060 | -1.967710 | -0.759723 | -1.807261 | -2.902244 | -1.161472 |
| -0.359421 | -0.833575 | -0.757490 | -0.751047 | -0.627926 | -0.708672 | -0.904015 | -0.883208 | -0.933934 |
| -0.541338 | -1.068634 | -0.578596 | -0.736725 | -1.523152 | -0.444599 | -0.883404 | -1.352841 | -0.662529 |
| 0.6818896 | 1.3535879 | -0.315892 | 1.5709540 | 1.7458288 | 0.1032547 | 0.3995438 | 1.5828264 | -0.108830 |
| 0.3343516 | 0.4128445 | 0.2855212 | 0.7201210 | 0.2467171 | -0.030173 | 0.1271198 | 0.0843611 | -0.029240 |
| -1.052525 | -1.737867 | -0.589390 | -1.741254 | -2.278358 | -0.632179 | -1.241487 | -1.964789 | -0.782615 |
| -0.219077 | -0.259396 | -0.331651 | -0.479691 | -0.646831 | -0.415474 | -0.375503 | -2.163809 | -0.041451 |
| -0.440165 | -1.851378 | -0.448477 | -0.745701 | -1.931754 | -0.156132 | -0.519689 | -1.068444 | -0.152582 |
| -0.616429 | -0.836835 | -0.558286 | -1.269953 | -0.563935 | -0.712942 | -0.558132 | -0.759708 | -0.919328 |
| -0.850502 | -1.686019 | -0.533114 | -1.437470 | -1.893595 | -0.235987 | -0.704627 | -1.819008 | -0.292593 |
| -0.720730 | -0.806528 | -0.322053 | -0.965385 | -0.369948 | -0.412900 | -1.040460 | -0.124870 | -0.616038 |
| -1.040297 | -1.496767 | -0.690283 | -1.538349 | -1.599116 | -0.569156 | -1.160023 | -1.936428 | -0.676541 |
| 0.8013816 | 1.4238605 | 0.4047816 | 0.8383867 | 1.5929708 | -0.074478 | 0.4420525 | 0.9739076 | 0.1106989 |
| -0.070154 | 1.2955651 | 0.0028119 | 0.4186913 | 2.0290654 | 0.3517226 | 0.6087777 | 3.0081748 | 0.4258505 |
| -1.254335 | -1.298255 | -0.602556 | -1.567282 | -1.051963 | -0.593085 | -0.740706 | -0.630455 | -0.680936 |
| 0.3415119 | 0.3502984 | 0.1550165 | 0.2059968 | 0.3914286 | -0.207322 | -0.277490 | 0.3450076 | -0.274423 |
| 0.4829725 | 0.6340976 | 0.3244506 | 0.5381016 | 0.5447249 | 0.2447355 | 0.4009525 | 0.6851549 | 0.3305851 |
| 0.5183192 | 0.7065234 | 0.2656248 | 0.7646714 | 0.7053129 | 0.3467711 | 0.7698561 | 1.3745678 | 0.4254541 |
| -0.735512 | -0.930248 | -0.531739 | -0.674457 | -0.909258 | -1.332425 | -1.030135 | -1.433245 | -1.458366 |
| 1.0063923 | 1.4244726 | 0.5303879 | 1.1852533 | 1.9385459 | 0.5096381 | 0.5095340 | 1.1399271 | 0.0827131 |
| 0.2723927 | 0.4056714 | 0.0982531 | 0.5134111 | 0.6322515 | -0.176853 | -0.076746 | 0.4074002 | -0.239929 |
| -0.116247 | -0.390711 | -0.132422 | -0.074341 | -0.274383 | -0.490447 | -0.377817 | 0.5392248 | -0.390596 |
| -0.040311 | -0.341738 | -0.171461 | -0.077508 | -0.454738 | -0.206963 | -0.293287 | -0.913360 | -0.234376 |
| -0.565991 | -2.142374 | -0.446604 | -0.731606 | -2.683401 | -0.385655 | -1.233138 | -1.871022 | -0.385584 |
| 0.1807276 | 0.8547994 | -0.283213 | 0.3590958 | 0.9498935 | -0.746132 | -0.128631 | 0.0958178 | -0.416695 |
| 0.8974126 | 1.1886580 | 0.1610716 | 0.8867582 | 1.6136989 | -0.200771 | 0.5492193 | 1.8610562 | 0.0105019 |
| 0.4686066 | 1.1348159 | 0.0322183 | 0.6116070 | 1.1693455 | -0.628240 | -0.128703 | 0.1903074 | -0.342565 |
| -0.146020 | -0.550444 | 0.0220356 | -0.469735 | -0.412873 | -0.091577 | -0.305537 | 0.0660624 | -0.206687 |
| -0.012236 | 0.1869140 | 0.2328570 | 0.0168133 | 0.2651713 | -0.067104 | -0.006593 | 0.0977675 | -0.064810 |
| -0.855888 | -1.801214 | -0.802598 | -1.250535 | -2.300590 | -0.226944 | -0.755238 | -1.617413 | -0.115797 |
| -0.537591 | -1.176174 | -0.459754 | -0.750613 | -1.298512 | -0.491066 | -0.984605 | -1.077949 | -0.629647 |
| -0.422318 | -2.329389 | -0.285025 | -0.658636 | -2.344523 | 0.3174664 | -0.056142 | -1.246387 | 0.4153829 |
| -0.008522 | -0.030331 | -0.130170 | -0.019321 | -0.044339 | -0.043121 | -0.087708 | -0.020248 | -0.029076 |

| | | | | | | | | |
|---|---|---|---|---|---|---|---|---|
| 0.3212459 | 0.5995113 | 0.3564442 | 0.2554760 | 0.6940194 | -0.2236407 | -0.1015616 | 0.1235502 | -0.1780308 |
| -0.4652575 | -1.6461815 | -0.4492051 | -0.2830119 | -1.3708377 | -0.6563190 | -0.4774571 | -2.3074531 | -0.6410712 |
| 0.0469029 | 0.0781341 | -0.2291102 | -0.0761046 | -0.6425633 | -0.5310137 | -0.2420048 | -1.2274689 | -0.5144084 |
| -0.9237564 | -1.6781950 | -0.8196834 | -1.8038704 | -1.5313252 | -0.8597582 | -1.0479304 | -2.8457778 | -0.8780731 |
| -0.3482728 | -1.2022922 | -0.4202350 | -0.6272279 | -1.5784215 | -0.2286160 | -0.6240672 | -1.8853094 | -0.1562043 |
| -0.1781558 | -0.7155510 | -0.1223371 | -0.1337742 | -0.9654614 | -0.4279266 | -0.0491240 | -0.0341280 | -0.2899712 |
| 0.8814102 | 1.5033638 | -0.3728272 | 1.0857618 | 1.4713779 | 0.1430763 | 0.7159694 | 1.9674595 | 0.0982177 |
| -0.1660715 | -0.4583031 | -0.3212488 | -0.3238084 | -0.3052347 | -0.0139879 | -0.3225729 | 0.0067767 | -0.2425109 |
| -0.2280013 | -0.4650159 | 0.1354022 | -0.6284695 | -0.7774757 | -0.1465858 | -0.5622577 | -2.0647357 | -0.4115344 |
| -0.7668626 | -0.0256728 | -0.5507873 | -0.4881416 | -0.0505199 | -0.4659656 | -0.3969861 | -0.1328851 | -0.6200373 |
| -0.9616180 | -0.3948092 | -0.9306126 | -1.1079557 | -0.7473973 | -0.0054300 | -0.8449307 | -0.5081287 | -0.3272870 |
| -0.3507719 | -0.8309611 | -0.7731174 | -0.4917727 | -0.3235907 | -0.6654955 | -1.0604821 | -0.4254686 | -0.8927412 |
| -1.0557169 | -1.4832676 | -0.1055099 | -0.7395260 | -1.6818597 | -0.3353069 | -0.3550676 | -0.8255758 | -0.2340690 |
| 0.1073690 | -0.7710864 | 0.2689874 | 0.0564684 | -0.6056929 | 0.1786559 | -0.0971296 | -0.2654919 | 0.0714504 |
| 0.0398874 | -1.5376080 | -0.0140876 | -0.2598110 | -1.9043476 | 0.0086729 | -0.2447219 | -1.7289747 | -0.0153080 |
| -0.4641141 | -1.0497241 | -0.0961403 | -0.8145163 | -1.2564455 | -0.0585513 | -0.2516897 | -0.8886333 | -0.2955126 |
| -0.0261805 | -1.3137592 | -0.0057857 | -0.0890823 | -1.8652095 | 0.2003082 | -0.0143848 | -0.7671050 | 0.1992095 |
| -0.5406260 | -0.7964020 | -0.2732150 | -0.5655935 | -0.8608807 | -0.4541711 | -0.6085806 | -0.4624213 | -0.4257966 |
| -0.7758272 | -2.2470862 | -0.0605429 | -1.4551471 | -2.5271915 | -0.3323811 | -0.6661969 | -1.7342056 | -0.3823036 |
| 0.3502982 | -0.1357788 | 0.4566758 | -0.1715787 | -0.2466971 | -0.1228533 | -0.0015499 | -0.6276211 | -0.1092004 |
| -0.7969207 | -0.5949720 | -0.4823924 | -0.7912527 | -0.9386318 | -0.9400049 | -1.1003502 | -2.9846588 | -0.9310482 |
| -1.7966151 | -2.2657857 | -1.3236698 | -2.1784883 | -2.2870520 | -0.3345778 | -0.3179703 | -0.1219004 | -0.2310661 |
| -1.4555699 | -3.4197656 | -1.0047158 | -2.5626926 | -3.5124698 | -0.5542850 | -1.2179537 | -3.1691248 | -0.5370647 |
| 0.0808052 | 0.6701117 | 0.0826329 | 0.1143191 | 0.5876387 | -0.1914169 | -0.0880747 | 0.0643070 | -0.1879243 |
| 0.5772610 | -0.4048801 | 0.5564717 | 0.0773209 | -0.8514318 | 0.2193766 | -0.5280456 | -1.4250826 | 0.4755754 |
| -0.2985042 | -0.1983219 | -0.5626658 | -0.4050064 | -0.1573147 | -0.4316952 | -0.4192192 | -0.3876328 | -0.4589133 |
| -0.1768065 | -0.9623862 | -0.1617040 | -0.4340537 | -1.1404933 | 0.0107116 | -0.1363739 | -0.8702863 | -0.0807780 |
| -0.5770585 | -1.4608037 | -0.5188780 | -0.7991450 | -1.3740060 | -0.3716377 | -0.5108204 | -0.7182162 | -0.4339681 |
| 0.2361324 | 0.4939469 | 0.0322448 | 0.3926877 | 0.9472134 | -0.2684567 | 0.0714805 | 0.9278393 | -0.1361217 |
| 0.3144177 | 0.1543883 | -0.182916 | 0.5632243 | 0.2920101 | 0.0267423 | 0.3213316 | 0.8391319 | 0.2331042 |
| -0.2354171 | -0.8405016 | -0.6881647 | -0.4303888 | -1.1166121 | -0.9234961 | -0.6647137 | -1.3100051 | -0.6949718 |
| -0.4715985 | -0.5358296 | -0.3134912 | -0.5394381 | -0.5048340 | -0.0952944 | -0.4052401 | -1.0338568 | -0.1091384 |
| -0.8639390 | 0.8326457 | -0.4817606 | -0.5988749 | 0.7020394 | -0.1023496 | -0.2187571 | -0.1234228 | -0.0682116 |
| -0.1467383 | -1.0853996 | -0.1534485 | -0.4507054 | -1.3917823 | -0.1311742 | -0.2424155 | -0.3197370 | -0.1398018 |
| -0.2937310 | -0.2716867 | -0.2909268 | -0.3206763 | -0.3947964 | -0.2108154 | -0.4205764 | -0.5788941 | -0.0720038 |
| -0.5663572 | -0.8186307 | -0.4495411 | -0.9141202 | -1.1525011 | -0.4054866 | -0.5190054 | -1.5055117 | -0.4675792 |
| -1.3842164 | -1.4948593 | -0.7540257 | -1.8711032 | -2.0310097 | -0.5886199 | -0.9949171 | -2.8158249 | -0.4110817 |
| -0.3680865 | -3.8522860 | -0.1076285 | -0.667028 | -4.7872722 | -0.0889857 | -0.0203771 | -0.4752474 | -0.2088170 |
| 1.0310684 | 0.9048368 | -0.7322081 | 1.2811931 | 0.7809738 | -0.2655739 | -0.2118033 | -0.4821554 | -0.2399847 |
| -0.3472361 | -0.7865874 | -0.2942456 | -0.8068353 | -1.3906567 | -0.0527326 | -0.1557529 | -1.1274644 | -0.0213678 |
| 0.3343424 | 0.2037177 | 0.1740005 | 0.3801855 | 0.5711021 | 0.2493109 | 0.3087915 | 0.4822711 | 0.0120616 |
| -0.4117296 | -1.2096255 | -0.0190924 | -1.0308513 | -1.4940410 | -0.0493437 | -0.2889893 | -1.3874073 | -0.1121828 |
| -0.8689102 | -1.3602762 | -0.3385723 | -0.8445946 | -1.3986943 | -0.1227269 | -0.6987367 | -0.8623386 | -0.336037 |
| -0.2899127 | -1.6069506 | 0.0358262 | -1.0547836 | -1.7686230 | 0.2333140 | -0.4419990 | -0.7965906 | -0.1344257 |
| -0.7473135 | -0.3102682 | -0.3134360 | -0.8135940 | -0.1401634 | -0.5242371 | -0.6856407 | -0.6233256 | -0.6720138 |
| -0.2707970 | -0.5980054 | -0.4303455 | -0.7582830 | -0.4186796 | -0.4080038 | -0.4592949 | -1.6950698 | -0.4229648 |
| -0.0947510 | -0.5542931 | -0.0478483 | -0.4692875 | -0.9998807 | -0.2310980 | -0.5258309 | -0.7511426 | -0.3325845 |
| -1.3417444 | -1.2564028 | -1.3023805 | -1.7807147 | -1.4396501 | -1.1098138 | -1.5039166 | -2.1691383 | -1.5028645 |
| 0.0067884 | 0.8515286 | 0.0533702 | 0.0594239 | 1.0930967 | -0.7985498 | -0.6060829 | -0.2959054 | -0.7294871 |
| -1.1485655 | -1.1593383 | -0.9421033 | -1.3331035 | -1.3487002 | -0.8097243 | -1.1247505 | -1.4494575 | -0.7208170 |

| | | | | | | | | |
|---|---|---|---|---|---|---|---|---|
| -0.2702839 | 0.3448730 | -0.1679465 | -0.1265972 | 0.6490789 | -0.4935885 | -0.8353949 | 0.7630288 | -0.5320219 |
| -0.4468306 | -0.4387484 | -0.3801161 | -0.1976509 | 0.1473337 | -0.3994777 | -0.0106017 | -0.3830692 | -0.2929739 |
| -0.1262294 | 0.2509852 | -0.1618164 | -0.1286331 | 0.2711689 | -0.3979600 | -0.2960931 | -0.0892573 | -0.3504655 |

| PG_390 | PG_391 | PG_392 | PG_393 | PG_394 | PG_395 | PG_396 | PG_397 | PG_398 |
|---|---|---|---|---|---|---|---|---|
| -0.333080 | -1.191612 | -0.085957 | -0.812318 | -2.627214 | -0.263404 | -0.927546 | -2.020051 | -0.791344 |
| -0.962036 | -2.206868 | -0.301718 | -0.693836 | -1.542269 | -0.506689 | -0.683149 | -1.293343 | -1.073862 |
| -1.230327 | -2.096098 | -0.591380 | -0.799525 | -0.472496 | -0.429043 | -0.315548 | 0.1926657 | 0.7345743 |
| -0.324967 | -1.432159 | -0.598338 | -0.507390 | -0.190695 | -0.298637 | -0.640011 | -0.171798 | -1.760076 |
| 0.2174240 | -0.167912 | -0.253806 | -0.361459 | -1.709796 | 0.0660591 | -0.250114 | -1.467100 | -0.920328 |
| -0.822884 | -2.403614 | -0.418028 | -0.852856 | -1.405406 | -0.555076 | -0.634281 | -1.354717 | -0.848648 |
| -1.083159 | -1.156676 | -0.429660 | -0.915351 | -0.665850 | -0.177572 | -0.991937 | -0.358433 | -0.946219 |
| -0.092347 | -1.632442 | -0.201447 | -0.534061 | -1.876794 | -0.294290 | -0.536405 | -1.587490 | -0.075461 |
| -0.036047 | -0.702526 | -0.416949 | -0.142580 | -0.073497 | -0.506956 | 0.0223616 | 0.0689958 | -0.706630 |
| -1.683827 | -2.075236 | -0.474544 | -1.785324 | -1.812246 | -0.688981 | -1.778506 | -1.816970 | -0.121771 |
| -0.251968 | -1.104932 | -0.288141 | -0.390893 | -0.853069 | -0.324226 | -0.555669 | -0.847921 | -1.205720 |
| -0.449277 | -1.038652 | -0.389455 | -0.732205 | -0.299200 | -0.458982 | -0.751124 | -0.456827 | -1.003872 |
| -1.735137 | -2.843784 | -0.096021 | -0.885029 | -1.618444 | -0.352634 | -0.924275 | -1.234314 | -1.144982 |
| 2.1710650 | 4.3724974 | -0.164283 | 3.5931953 | 4.3283245 | 0.2809451 | 3.9939726 | 4.6658080 | 0.5248877 |
| 0.8100985 | 0.0321473 | -0.538144 | -0.553984 | 0.0750229 | -0.274610 | -0.278282 | 0.2819495 | -2.864561 |
| 2.5951836 | 2.7575292 | -0.374028 | 3.3372207 | 3.1897268 | 1.0307471 | 3.4769464 | 3.5311556 | 0.0747467 |
| -1.891526 | -1.855271 | -0.814702 | -2.379824 | -2.394371 | -1.788102 | -2.200943 | -2.476535 | -1.493483 |
| -1.588478 | -0.503775 | -0.590516 | -0.799879 | -0.186640 | -0.902301 | -0.656784 | -0.385069 | -0.145715 |
| -1.704389 | -1.968549 | -0.003023 | -0.709551 | -2.746959 | -0.494167 | -1.150722 | -3.365120 | 0.4034707 |
| -1.591365 | -1.083721 | -1.235276 | -1.105967 | -1.911568 | -1.552836 | -1.808420 | -1.660901 | -0.975131 |
| 0.0624327 | -0.285892 | -0.589430 | 0.5021969 | 0.4759871 | -0.506888 | 0.5500418 | 0.3264249 | -1.075158 |
| -0.420753 | -0.056644 | -0.404922 | -0.124766 | -0.130388 | -0.314672 | -0.509720 | 0.1006128 | 0.0938674 |
| 0.9180298 | 1.7875754 | -0.102991 | 1.2214866 | 2.0667283 | -0.297194 | 1.0437256 | 1.8674037 | 0.4549733 |
| -0.555780 | -1.598553 | -0.370994 | -1.317400 | -2.338768 | -0.574751 | -1.616614 | -1.915469 | -0.907866 |
| -1.753477 | -3.179642 | -0.119495 | -1.309610 | -2.717394 | -0.691739 | -1.534623 | -1.923512 | -1.020908 |
| 0.1441749 | 0.5507418 | 0.3575523 | 0.3488502 | 0.6157179 | -0.010952 | 0.2256279 | 0.3640596 | -0.036433 |
| -0.178533 | -0.847906 | -0.225283 | -0.468212 | -0.299656 | 0.2996207 | -0.223883 | -0.240179 | -1.842332 |
| -0.790751 | -2.970957 | -0.994603 | -1.893559 | -1.474158 | -0.953278 | -2.108140 | -1.159308 | -2.485295 |
| -0.520824 | -1.601898 | -0.157734 | -1.049578 | -2.237236 | -0.592916 | -1.322764 | -1.191014 | -0.200066 |
| -0.319708 | -1.152296 | -0.508231 | -0.514053 | -0.167502 | -0.089961 | -0.661045 | -0.122997 | -1.804409 |
| -1.141983 | -1.637429 | -0.512030 | -0.894818 | -1.809548 | -0.438200 | -0.792618 | -1.466715 | -0.098932 |
| 0.1115112 | 0.2839525 | -0.191710 | -0.381045 | 0.4051134 | 0.5988351 | -0.045227 | -0.040611 | 0.5492772 |
| 0.4817078 | 0.7506230 | -0.471883 | 0.4198591 | -0.367122 | -0.242659 | 0.8110098 | -0.244219 | -1.041172 |
| -0.628808 | -2.191343 | -0.533182 | -0.713609 | -1.499017 | -0.502887 | -0.671507 | -2.039498 | -0.709894 |
| -0.359705 | -0.763358 | -0.125513 | -0.111269 | 0.0261798 | -0.024233 | -0.102211 | -0.100215 | -0.195256 |
| -2.300116 | -1.184834 | -0.546499 | -0.936850 | 0.3063095 | -1.931889 | -1.336973 | 0.3258256 | -0.923829 |
| -0.784829 | -2.076673 | -0.370069 | -0.947815 | -1.691078 | -0.429306 | -0.498106 | -1.347353 | -1.147260 |
| 3.3956314 | -0.527191 | 0.9101613 | 4.0553809 | 0.3460497 | 1.4500084 | 3.7483708 | 0.0030002 | -0.371191 |
| -0.546958 | -0.170185 | -0.776077 | -0.714155 | -0.813409 | -0.309088 | 0.1345123 | -0.861186 | -0.416191 |
| -1.122970 | -0.447391 | -0.590691 | -0.419248 | 0.7793628 | -0.976028 | 0.3026352 | 1.1558178 | -0.851825 |
| 0.1472321 | 1.4884928 | 0.1695130 | 0.4755164 | 0.3602813 | -0.390590 | 0.4873741 | 0.3944921 | 0.3883011 |
| -0.704589 | -1.611533 | -0.517905 | -1.478748 | -2.359939 | -0.950341 | -1.849825 | -2.318666 | -0.507760 |
| -0.564525 | -0.752652 | -0.886588 | -0.463092 | -0.043515 | -0.667147 | -0.562057 | -0.028217 | -0.410998 |
| -0.273605 | -1.311047 | -0.572000 | -0.393429 | -0.759208 | -0.385262 | -0.374158 | -0.959559 | -1.464139 |
| 0.2076888 | 0.6464350 | 0.2335746 | 1.1594006 | 1.6556550 | -0.164460 | 1.0200041 | 1.3966177 | 0.2557117 |
| 0.2152615 | 2.9544117 | 0.2349341 | -0.273277 | 0.8382114 | -0.434152 | -0.080076 | 2.1381810 | -0.129576 |
| -0.213217 | 0.1070937 | 0.2825662 | -0.020111 | 0.2264559 | -0.168766 | 0.0033367 | -0.410113 | 0.9150605 |

| | | | | | | | | |
|---|---|---|---|---|---|---|---|---|
| 0.1513508 | -0.6704594 | -0.6026252 | 0.1142685 | 0.2475207 | -0.6547423 | 0.2781527 | 0.4586826 | -0.8194694 |
| -0.7872262 | -1.6339273 | -0.1622755 | -0.5883622 | -2.9555786 | -0.3485615 | -0.6018961 | -2.4783354 | -0.6964719 |
| -0.0204006 | 0.1416122 | -0.3522143 | -0.0454186 | 0.0603564 | -0.2945112 | -0.2183200 | -0.1931962 | -1.0929064 |
| 2.2603876 | 2.4757784 | -0.5693309 | 2.2223740 | 1.6250041 | -0.9531430 | 1.3130018 | 1.7487626 | 0.0279521 |
| -1.8642444 | -2.7319055 | -0.3618274 | -0.2874382 | -0.2194363 | -0.4132077 | -0.3851725 | -0.1798561 | -0.3952566 |
| -0.5553331 | 0.5456556 | -0.2421988 | -0.3221639 | 1.2182500 | 0.2719951 | 0.1352380 | 1.5289599 | 0.3532809 |
| -1.6097903 | -1.7204045 | 0.0666956 | -0.5080635 | -1.3276208 | 0.0564990 | -0.8700985 | -1.3565380 | -1.0682243 |
| 0.1586870 | 0.8810513 | -0.1309309 | 2.0899860 | 2.8755536 | 0.3283279 | 2.0052522 | 2.6602531 | -0.3547067 |
| 2.1238201 | -0.3956057 | 1.1919157 | 4.5940878 | 1.1661627 | 2.6164290 | 3.9810293 | 1.0372248 | 0.5263628 |
| -0.3721990 | -0.9450155 | -1.0590258 | -0.5441992 | 0.4522531 | -0.7176217 | -0.7989180 | 0.2657001 | -1.4921743 |
| -1.4302688 | -1.3344977 | -0.4140788 | -1.5510397 | -2.7081560 | -0.8113033 | -1.7486151 | -2.0657953 | -1.4203709 |
| 3.6079522 | 3.5407456 | 0.0724616 | 3.1870886 | 2.5944653 | 1.6486172 | 3.4835091 | 3.3473260 | -0.1362969 |
| 1.9480742 | 1.7105466 | -0.0810206 | 3.6400529 | 2.7001583 | 1.4215763 | 3.7964698 | 3.0259201 | -1.1691875 |
| -0.5249361 | -1.8862221 | -0.3387715 | -0.5124753 | -1.3751792 | -0.6847734 | -0.7678881 | -1.2567589 | -0.5319994 |
| -1.4351457 | -1.9057913 | -0.9800411 | -1.6071344 | -4.0438636 | -0.7865561 | -2.1919749 | -3.7327245 | -1.3077395 |
| -0.7824841 | -0.4497233 | -0.874536 | -1.2195764 | -1.2189720 | -0.3484633 | -1.4866275 | -1.2970477 | -0.7879631 |
| -0.9177358 | -2.0006407 | -0.3305468 | -0.9171146 | -2.3861211 | -0.2805812 | -1.6292367 | -2.0920903 | -1.0828093 |
| -0.7162297 | -0.6814223 | -0.2147440 | -0.8548260 | -1.0167104 | -0.3563952 | -1.2004284 | -1.0403830 | -0.9215819 |
| -0.4435651 | -0.6747662 | -0.5826439 | -0.7026058 | -1.4576424 | -0.6298586 | -0.8325720 | -1.3937162 | -1.2049964 |
| -0.3488367 | -2.2994354 | -0.2103907 | -0.4932429 | -1.5894276 | 0.5610694 | -0.4069046 | -1.6308958 | -3.4169027 |
| 0.3546728 | -0.8944124 | -0.6677585 | -0.3155799 | -0.8086654 | -0.4942905 | -0.6757355 | -0.9843169 | -0.4722011 |
| -0.9997052 | -1.4228564 | -0.5366462 | -0.9443170 | -2.0781343 | -0.4761199 | -1.1354336 | -2.0298013 | -0.6231947 |
| -0.2355237 | 0.1503510 | -0.0866327 | -0.5098363 | 0.1637885 | -0.4960300 | -0.1551663 | 0.0569808 | 0.7171913 |
| 0.3916067 | -0.8927789 | -0.5870019 | 0.2461640 | 0.6702515 | 0.2357673 | 0.2728721 | 0.4083376 | -3.6321290 |
| -0.7590663 | -1.7142496 | -0.7322042 | -1.3877894 | -1.4020809 | -1.2312243 | -1.3061390 | -1.1595559 | -1.4999399 |
| -0.6947438 | -0.3626260 | 0.2044261 | -0.8593122 | -0.5130855 | -0.1013148 | -1.2228701 | -0.0520281 | -1.1387856 |
| -1.7449123 | -1.3655993 | -0.3473665 | -1.5168323 | -2.2091369 | -1.0142953 | -1.6913881 | -2.2922838 | -0.4419934 |
| -0.1658033 | 0.3105155 | -0.3904948 | 3.1639783 | 5.8756622 | -0.0471742 | 3.1267843 | 5.6221036 | 0.0599461 |
| 0.3025267 | 1.3058886 | -0.2499085 | 0.9636654 | 2.9762916 | -0.4618369 | 0.9935059 | 3.2882826 | -0.6208986 |
| -0.1343596 | -2.4646489 | -0.3650024 | -0.9655765 | -1.9430030 | -0.3657526 | -1.2126976 | -2.1052899 | -0.6914014 |
| -0.9077121 | -1.0200772 | 0.0368069 | -0.1057798 | 0.2643806 | 0.0388277 | -0.1064671 | 0.4696023 | -0.4539000 |
| -0.1390369 | -0.4294027 | -0.4637801 | -0.7336102 | -0.3844731 | -0.1801904 | -0.8492672 | -0.7249758 | -2.1163497 |
| -1.1685048 | -0.9915707 | -0.6747046 | -1.6797003 | -1.7127291 | -0.5682507 | -1.6786809 | -1.4147287 | -0.7455243 |
| -0.5747112 | -1.8908894 | -0.4448423 | -1.3765973 | -1.9926349 | -0.5845413 | -1.5690186 | -1.7256317 | -1.7971762 |
| 1.0611969 | 1.9785636 | -0.3479805 | 1.2025828 | 1.5456520 | 0.0971532 | 1.3334788 | 1.4740723 | 0.0602278 |
| -0.2817786 | -0.0722842 | -0.1007867 | 0.0450617 | -0.1481149 | -0.2785791 | -0.2882641 | -0.1274159 | 0.8627893 |
| -0.2868282 | 0.0893803 | -0.3783436 | -1.7217136 | -2.6965745 | -0.5993412 | -1.2718559 | -1.9184577 | -0.8423231 |
| -0.7096536 | -0.9650213 | -0.2256131 | -1.1791976 | -1.6485027 | -0.5404754 | -1.5072082 | -1.3823121 | -0.4080390 |
| -0.6659244 | -3.3102719 | -0.1522872 | -1.0657608 | -0.8472083 | -1.0554979 | -0.9905773 | -0.6354654 | 0.3454183 |
| -0.7206767 | -2.2520806 | -0.0406998 | -1.3302756 | -2.7424399 | -0.1138143 | -1.5078944 | -2.5311659 | -0.1434586 |
| -1.0523595 | -1.7310861 | -0.4931258 | -1.1002568 | -0.7360253 | -0.3657911 | -1.1226659 | -1.0308942 | -1.2253453 |
| -2.6050886 | -3.1590749 | -0.3122390 | -3.1603713 | -4.6531841 | -1.0630998 | -3.4918325 | -4.0913693 | -0.0020956 |
| 3.8100202 | 2.7901594 | 0.0557073 | 3.3091736 | 1.7243833 | 0.9040500 | 3.5066384 | 1.4478307 | 0.1905716 |
| -0.0384287 | -0.1706933 | -0.0468229 | -0.1829977 | -0.3845275 | -0.0727863 | -0.2580400 | -0.3994077 | -0.7698205 |
| 0.0223659 | 0.1151288 | -0.2369285 | -0.4680953 | -1.3215942 | -0.1577397 | -0.5001935 | -1.4827495 | -1.4151449 |
| -2.0487224 | -1.7246157 | -1.1257405 | -2.7646565 | -2.7268690 | -1.5872962 | -2.9951021 | -3.0191614 | -0.3402701 |
| 7.7889232 | 8.6280068 | 0.1610631 | 8.1614570 | 6.8238948 | 5.0451400 | 8.3964245 | 8.4680403 | 0.2594982 |
| 2.1864546 | 2.2015118 | -0.2021505 | 2.1255674 | 1.6985112 | 0.4508155 | 2.3023350 | 1.9360107 | -0.9980188 |
| -1.6235169 | -0.1459155 | -0.8147871 | -0.9421203 | -3.0020587 | -0.4689419 | -1.4542343 | -2.5798756 | -1.2937512 |
| -0.1819342 | -1.7837886 | -0.5564158 | 0.2057363 | 0.3122206 | -0.4420469 | 0.5018492 | 0.4034282 | -0.4677136 |

| | | | | | | | | |
|---|---|---|---|---|---|---|---|---|
| -0.8288043 | -1.0666919 | -0.6317731 | -0.7489300 | -0.2637172 | -0.5570641 | -0.9336549 | -0.3629498 | -0.5638879 |
| -0.2597148 | 0.4537081 | -0.2738619 | -0.5505330 | 0.3078351 | -0.4593803 | -0.5003837 | -0.0023700 | -0.7361163 |
| -0.7739366 | -1.4820211 | -0.6429328 | -1.7046207 | -0.2718877 | -1.5349574 | -1.7049048 | -0.2557966 | -0.8683527 |
| 0.0019737 | 0.4057989 | -0.2901046 | -0.2696824 | 0.0348629 | -0.1437405 | -0.1420004 | 0.3284075 | -0.5049920 |
| -1.7848630 | -2.2155395 | -0.6068933 | -1.2606894 | -2.3744607 | -0.5021772 | -1.5982039 | -2.2592044 | -0.7625584 |
| -0.6751341 | -1.6039552 | -0.9657914 | -0.5617782 | 1.5576766 | -0.7866210 | -0.8555084 | 1.8325340 | -0.9571568 |
| -0.6993524 | -1.4444471 | -0.4614503 | -0.0875890 | 0.0593457 | -0.0948592 | 0.2386669 | 0.3563877 | -2.2243279 |
| 0.0315937 | 0.1888975 | 0.0077324 | 0.1130765 | -0.1658972 | 0.2049573 | -0.0759344 | -0.0395421 | 0.2851717 |
| 0.0001144 | -0.1966014 | -0.2826990 | -0.1688727 | -0.2431840 | -0.0952223 | -0.2419439 | -0.2369590 | 0.6374444 |
| 0.4752405 | 0.5981771 | -0.0258598 | 0.6332704 | 1.0024272 | 0.0876742 | 0.7569244 | 1.0075799 | -0.5306164 |
| -0.2484220 | -0.9292948 | -0.7073217 | -1.3935107 | -1.8786519 | -0.7639982 | -1.4554273 | -1.2919841 | -0.6545141 |
| -1.3710093 | -2.9265497 | -0.3419572 | -0.9792609 | -1.1831989 | -0.5272353 | -1.2103202 | -1.2609001 | -2.1359907 |
| -0.7854103 | -3.5267550 | -0.2958713 | -1.1807559 | -2.3881330 | -0.5702444 | -1.1097977 | -2.5674758 | -0.3240555 |
| -0.6972193 | -2.4916702 | -0.0640230 | -0.1954488 | -0.9433748 | -0.2656146 | -0.0968921 | -1.0155627 | -0.8124468 |
| -0.2989919 | -0.8883919 | -0.2136214 | -0.6508035 | -0.5149472 | -0.4320914 | -0.7427750 | -0.8566882 | -0.9387638 |
| -0.2898365 | -0.0846269 | -0.1768422 | -0.1659415 | -0.2126196 | 0.6402741 | -0.0203281 | -0.3042599 | 0.3023758 |
| -1.4191753 | -1.5273630 | -0.5700623 | -1.3802631 | -0.4823975 | -0.4991648 | -2.0306710 | -0.4919816 | -1.5363811 |
| -0.2572694 | 0.0903607 | -0.0283733 | -0.2049768 | 0.0510505 | 0.0338335 | -0.1040744 | 0.3145610 | 0.0705635 |
| -0.5904257 | -0.9502382 | -0.5787895 | -0.8638241 | -0.8976083 | -0.4180111 | -0.8234565 | -1.3813003 | -0.8722423 |
| 0.0612841 | 1.5587375 | 0.0299688 | -0.8576931 | 1.3542588 | -1.6393031 | -0.4386585 | 1.6006016 | 0.4915309 |
| -0.7295102 | -2.6558732 | 0.0598572 | -0.3421304 | -1.1113375 | -0.1341277 | -0.3855771 | -0.9154233 | -0.5502555 |
| -0.4371910 | -1.2392351 | -0.5275123 | -1.0434555 | -0.1139066 | -0.4427590 | -1.2344477 | -0.3826797 | -0.6102873 |
| 0.5614318 | -0.4459691 | -0.6925480 | -0.3095627 | -0.1699398 | -0.8363654 | -0.8059729 | -0.1164199 | 0.0646314 |
| -0.3865682 | -1.2372832 | -0.3693868 | -0.9515637 | -2.4514424 | -0.3919391 | -0.7980209 | -2.5178217 | -0.5104526 |
| -0.4866103 | -0.7511516 | -0.5227251 | -0.2325550 | -0.3447032 | -0.2586429 | -0.2480120 | -0.3019212 | -0.0007621 |
| -0.8120836 | -0.5804136 | -0.3709200 | -1.0323912 | -0.1258150 | -1.0753117 | -1.2193622 | -0.3832565 | -1.8440334 |
| -0.0800935 | -0.1890982 | -0.4120542 | -0.4035771 | 0.2291647 | -0.0410135 | -0.3855106 | 0.2872193 | -0.7449717 |
| 0.4661077 | 0.1126133 | -0.0254792 | 0.1295890 | 0.8042052 | -0.0197465 | 0.2752651 | 0.5046230 | 0.5903414 |
| -0.8045309 | -1.7627144 | -0.4067241 | -2.2247720 | -1.2911413 | -0.9096158 | -2.0477432 | -1.3087270 | -1.2020721 |
| -0.2543256 | -1.2903620 | -0.4799616 | -0.3241144 | -0.9456852 | -0.3401262 | -0.4611538 | -0.6494216 | -0.9457239 |
| -1.5430640 | -1.3601624 | -0.5135179 | -2.6425318 | -2.6842673 | -0.7296045 | -2.1631490 | -2.0321050 | -1.1440594 |
| -0.0962426 | -1.0098677 | -0.0726783 | -0.6832720 | -2.3542693 | -0.5059652 | -0.8319975 | -2.1486121 | -1.0315777 |
| 0.3841274 | 0.2239114 | -0.3555525 | -0.3526844 | 0.2104789 | -0.2949903 | -0.0214843 | 0.0651950 | -0.6735227 |
| 0.2936754 | 0.0164528 | 0.1494685 | 0.1592815 | -0.0127852 | -0.0225343 | 0.1237137 | 0.0585900 | -0.0869250 |
| -0.0229795 | -0.7466546 | -0.3756671 | -0.8601117 | -2.3838577 | -0.6880295 | -1.2112182 | -2.0253249 | -0.4454663 |
| -0.5730065 | -0.9308234 | -0.5202828 | -1.3254726 | -1.1179262 | -0.5142504 | -1.4243917 | -0.6619671 | -0.9594319 |
| -0.3068989 | -0.2455795 | -0.3635369 | -0.8104628 | -0.5300387 | -0.3437231 | -0.9073039 | -0.7633709 | -0.9004530 |
| -0.0538594 | -0.5700369 | -0.2657454 | -0.7371175 | -1.2046252 | -0.0924608 | -0.7432907 | -1.1253740 | -1.5260531 |
| -0.4949406 | -0.8608054 | -0.5844836 | -0.5350719 | -0.2774132 | -0.2483979 | -0.9670174 | -0.3755637 | -0.8214892 |
| -1.0461909 | -1.0726868 | -0.0055555 | -1.6893119 | -1.3299781 | -0.7688646 | -1.1282060 | -0.1462061 | -0.6311030 |
| -0.5891739 | -0.1801937 | 0.0363109 | -0.1786154 | -1.5110966 | 0.3184107 | -0.3318799 | -1.7132594 | -0.9316797 |
| -0.2248412 | -1.0726232 | -0.0826335 | 0.2245220 | -0.6749550 | -0.2246410 | 0.1782130 | -0.4007544 | -0.7392357 |
| -0.1529122 | -0.9416404 | -0.2265657 | -0.2534013 | -0.6027923 | 0.0105129 | -0.2307516 | -0.5502715 | -1.2670766 |
| -0.7162905 | -1.3061659 | -0.2378690 | -1.2117317 | -2.6249477 | -0.7437754 | -1.1700525 | -2.5343115 | -1.1203955 |
| -0.0710694 | 0.1496234 | -0.1032261 | -0.3321127 | -0.1185922 | -0.2346959 | -0.1722499 | -0.1386961 | -0.2094503 |
| -0.0983813 | -0.3792299 | -0.0784020 | 0.7846259 | 0.4843376 | 0.0434638 | 0.4427776 | 0.7366583 | -0.8169545 |
| -0.4175017 | -0.3282118 | -0.4536769 | -1.1099156 | -2.6749018 | -0.3397875 | -1.0309535 | -2.4363651 | -0.6007013 |
| 0.5136937 | 0.4940716 | -0.2724751 | -0.1303947 | 1.0873441 | -0.6014726 | 0.0302940 | 1.4078340 | -1.5780482 |
| -1.3603173 | -3.4919294 | -0.6398497 | -0.8063507 | -0.0887894 | -0.5146334 | -0.6018012 | -0.0999355 | -1.3611652 |
| -0.8209236 | -2.0067597 | -1.0605330 | -1.0617516 | -2.2659218 | -1.0386910 | -1.1577535 | -2.4270795 | -0.9659927 |

| | | | | | | | | |
|---|---|---|---|---|---|---|---|---|
| 0.2778520 | 0.2799206 | -0.4491483 | -0.1463080 | 0.4126671 | 0.1379806 | -0.0544290 | 0.5142220 | -1.7728182 |
| -1.3327539 | -1.7805542 | -0.3909734 | -1.8388907 | -1.5623168 | -1.6689992 | -1.2883604 | -1.6630827 | -1.8913116 |
| -0.6483717 | 0.0780480 | -0.4105720 | -0.6805864 | -0.5381794 | -0.4717047 | -0.6431240 | -0.8885967 | -0.5076028 |
| 0.7479750 | 1.3577912 | -0.0674618 | -0.3634925 | 0.1366040 | -0.1746307 | -0.0993230 | 0.2624027 | 0.7775061 |
| -1.2582147 | -1.9991153 | -0.4208731 | -0.9233912 | -1.2709181 | -0.4830779 | -1.2999972 | -1.1663604 | -1.2270228 |
| 1.9363755 | 1.8144651 | 0.6542072 | 2.8454581 | 1.8784363 | 0.8548524 | 3.1978091 | 1.2970463 | 0.2988168 |
| -0.5369745 | -0.6017374 | -0.4502547 | -0.7113090 | -0.7225880 | -0.4345750 | -0.9043404 | -0.7908098 | -0.4648277 |
| -1.3319659 | -1.4854091 | -0.1935540 | -0.7574611 | -0.9986870 | -0.1105262 | -1.4452630 | -0.8629368 | -0.5906736 |
| -1.3839450 | -1.4210483 | -0.3371553 | -1.4388410 | -1.8104825 | -0.8304060 | -1.4623720 | -2.0623339 | -0.9342845 |
| -0.5804619 | -1.1234375 | -1.0002933 | -0.7451162 | -0.1591697 | -0.8456388 | -0.4915399 | -0.3079100 | -0.5024433 |
| 0.4589220 | 0.7450815 | -0.1569928 | -0.0837274 | 0.3530648 | -0.0593370 | -0.1622412 | 0.3046213 | -0.6713758 |
| -1.3436799 | -1.8361121 | -0.5903654 | -0.9701319 | -1.0923778 | -0.7383728 | -0.4357720 | -0.6750503 | -1.0115690 |
| -0.9363420 | -1.6752874 | -0.7126803 | -2.2206883 | -1.0963492 | -1.2258290 | -2.7154585 | -1.1787420 | -2.4696164 |
| -0.2523185 | -0.7905028 | 0.2253481 | -0.4332058 | -1.0677399 | -0.0720543 | -0.5240210 | -0.7691290 | -0.6650086 |
| -0.2219070 | -0.2883569 | -0.5534530 | -0.5044205 | -0.1887913 | -0.0556273 | -0.4940838 | -0.1382713 | -0.3781180 |
| -0.1099427 | -0.5557905 | -0.0210125 | -0.3033580 | 0.0229409 | 0.0776949 | -0.1050789 | -0.1307944 | -0.6355963 |
| -0.4949850 | 0.8273037 | -0.1454352 | -0.2284818 | -0.7214323 | -0.9098287 | -0.6886675 | -0.6775692 | 0.5380062 |
| -2.6253934 | -3.7652280 | -0.8352098 | -2.2545023 | -2.5095220 | -0.6781565 | -3.0861008 | -2.1156813 | -1.0531327 |
| -0.6852930 | -0.9010645 | -0.3360959 | -1.0838287 | -0.3077920 | -1.0454610 | -1.0753703 | -0.3211241 | -0.7877901 |
| -0.9161343 | -1.3947382 | -0.4105602 | -0.8619311 | -0.6854121 | -0.3464930 | -1.1678750 | -0.8228249 | -1.8000744 |
| 0.9084374 | 1.9811166 | -0.1520135 | 0.2189613 | 0.3197137 | 0.1017673 | 0.4367910 | 0.6804223 | 0.2529360 |
| 0.1453986 | 0.0218186 | -0.2970420 | -0.2286337 | -0.0820000 | 0.0264862 | -0.1708137 | 0.0324329 | -1.5089759 |
| -1.1369852 | -2.0425625 | -0.3843838 | -1.2321635 | -2.1819492 | -0.6722679 | -1.6331799 | -1.7573343 | -0.7997545 |
| -0.3597891 | -2.7603929 | -0.7407934 | -0.6527172 | -0.3581180 | -0.7572953 | -0.4962827 | -0.9325971 | -1.2621449 |
| -0.7494208 | -1.1928002 | -0.2083539 | -1.3370805 | -1.6009410 | -0.6768261 | -1.3730708 | -1.5120107 | -0.1024985 |
| -0.5561415 | -0.8763179 | -0.5628599 | -1.4526514 | 0.5454270 | -1.0631178 | -1.2812779 | 0.4891520 | -0.8856664 |
| -0.7798131 | -2.0545339 | -0.5829580 | -1.0533560 | -1.1613030 | -1.1657377 | -1.0370543 | -1.0598394 | -0.9366649 |
| -1.2093693 | -0.2732820 | -0.0057400 | -0.1735802 | 0.1947535 | -0.1164382 | -0.2424577 | 0.3303021 | -0.5531838 |
| -1.4309220 | -2.2538624 | -0.6430637 | -0.9140160 | -1.5249302 | -1.1671645 | -1.3557707 | -1.0811601 | -1.3248518 |
| 0.3231047 | 0.4753348 | 0.1142226 | 0.6688874 | 1.6313730 | -0.0741871 | 0.4064011 | 1.7146521 | -0.9215872 |
| 0.8289854 | 3.2171223 | 0.1321041 | 2.9188697 | 3.9524388 | 0.2299834 | 3.3554757 | 4.0981937 | 0.4680191 |
| -0.9449132 | -0.8616233 | -0.2601730 | -0.4219899 | -0.5388680 | -0.5192298 | -1.0455837 | 0.1525829 | -1.0748537 |
| -0.2019979 | 0.1321551 | -0.3678492 | -0.2414502 | -0.1975079 | -0.0861092 | -0.1999281 | -0.2437580 | -0.4230087 |
| 0.3728811 | 0.6904591 | -0.0483605 | 0.2065365 | 0.4846553 | 0.0087438 | 0.2596250 | 0.5139560 | -0.7109769 |
| 0.6544553 | 1.2788944 | 0.4082184 | 0.7569539 | 0.2634574 | 0.0405623 | 0.6009764 | 0.1461687 | 0.6738803 |
| -0.8047370 | -1.3410089 | -0.5354195 | -1.7728015 | -1.6778539 | -0.6295065 | -1.5138105 | -1.4754015 | -1.7805623 |
| 0.9772615 | 0.8385489 | -0.4539817 | -0.0673331 | 1.3900953 | -0.0118421 | 0.1461818 | 1.8369810 | -1.1059249 |
| -0.0174884 | 0.5347663 | -0.6406593 | 0.2841622 | 1.0468003 | -0.3079887 | 0.4686763 | 1.3586134 | -1.2800115 |
| -0.2578670 | 0.4482982 | -0.1831892 | -0.3021833 | 0.5482663 | -0.2913449 | -0.5144083 | 0.7351097 | -1.1879565 |
| -0.3262089 | -1.3571367 | -0.4081379 | -0.7318585 | -1.3206488 | -0.3520267 | -0.6873561 | -1.2787683 | -1.7246908 |
| -0.6422010 | -1.4461740 | -0.4760155 | -3.1947947 | -1.8678195 | -1.1801634 | -3.0230610 | -1.8589768 | 0.1577562 |
| 0.1838229 | -0.0082360 | 0.0835382 | 0.4059639 | 1.2176972 | -0.1164403 | 0.5312538 | 1.2060275 | -1.2472543 |
| 0.7638804 | 1.6308972 | -0.3765803 | 1.1690669 | 2.7895419 | -0.1189033 | 1.2896033 | 2.5261715 | -1.4498045 |
| 0.2463477 | -0.1035917 | -0.2303797 | 0.3974319 | 0.8299572 | -0.0086007 | 0.2827084 | 0.7292133 | -1.2041025 |
| -0.5211813 | 0.2874041 | -0.2904254 | -1.0770357 | -0.8282942 | -0.3742271 | -1.0291180 | -0.3730570 | -0.4965141 |
| -0.0953484 | 0.0203366 | -0.3579473 | -0.6507491 | 0.0657683 | -0.2612495 | -0.4714360 | 0.0353280 | -0.9063673 |
| -0.8015985 | -1.6552471 | -0.4595520 | -1.2738723 | -2.4611328 | -0.8457438 | -1.4557678 | -2.3620730 | -0.4451224 |
| -1.2319005 | -1.0323619 | -0.2996580 | -1.4037358 | -2.3540823 | -0.5863232 | -1.3874675 | -2.0863230 | -1.0469084 |
| -0.1867310 | -1.1068761 | -0.5872035 | -0.4292775 | 0.0235475 | 0.7521656 | -0.9076772 | -0.2626823 | -0.2436427 |
| -0.1557069 | -0.4598270 | -0.4098254 | -0.4578973 | -0.1071215 | -0.3414777 | -0.5206061 | -0.3522492 | -1.9044015 |

| | | | | | | | | |
|---|---|---|---|---|---|---|---|---|
| 0.2971333 | -0.4006012 | -0.8258914 | -1.0472206 | -0.0048138 | -0.6581384 | -0.8797792 | 0.4080861 | -3.8767590 |
| -0.6827704 | -1.7617902 | -0.1316704 | -0.3727485 | -0.6726894 | -0.2943182 | -0.3095054 | -0.6350459 | -1.5653171 |
| -0.2671571 | -1.6969892 | -0.8619141 | -0.0664451 | -0.2932063 | -0.9589141 | 0.4107236 | -0.2235927 | -1.4682687 |
| -1.1593471 | -2.3957442 | -0.5335748 | -0.6463032 | -0.4732999 | -0.6508571 | -0.4861383 | 0.1527811 | -0.3601995 |
| -0.4763306 | -2.3460312 | -0.6281414 | -0.8746569 | -1.1892150 | -0.9263139 | -1.2109259 | -1.2351008 | -1.8179218 |
| -0.2974891 | -0.1011232 | -0.0947272 | 0.0941084 | -0.5196964 | -0.3047447 | -0.1881585 | -0.5096489 | -0.5540342 |
| 0.7088982 | 1.2764928 | -0.1691275 | 1.9107878 | 1.7566725 | 0.2111846 | 1.9037272 | 1.3346936 | 0.4305238 |
| -0.3163562 | 0.2907380 | -0.1163407 | -0.5226313 | -0.1982888 | -0.1512915 | -0.4959499 | -0.0710027 | 0.0467744 |
| -0.5346913 | -2.7960666 | 0.0567102 | -0.2239262 | -1.3488127 | 0.3114612 | -0.1736913 | -0.9988077 | -2.3943341 |
| -0.3017000 | -0.7767870 | -0.6104223 | -1.7283542 | 0.5322893 | -1.3156392 | -1.2892629 | 0.8262283 | -0.0634876 |
| -0.9156534 | -0.6972126 | 0.0505805 | -0.4234983 | -0.5531614 | -0.1088840 | -1.2418444 | -0.9933033 | -0.3576307 |
| -0.5714436 | -0.3996276 | -0.7949039 | -1.4398732 | -0.8041556 | -1.0023649 | -1.7150428 | -0.7889400 | -2.1121353 |
| -0.2340665 | -0.7232880 | -0.1946506 | -0.2754439 | -0.1031421 | -0.5376455 | -0.0372518 | -0.0626120 | 0.1149108 |
| 0.0405827 | -0.3840689 | -0.3932406 | -0.7681340 | -0.8509252 | -0.4688289 | -1.0346381 | -0.4316688 | -0.5254501 |
| -0.3617333 | -1.6639199 | -0.4396488 | -1.2918007 | -2.4778357 | -0.4827747 | -1.7659644 | -1.9299564 | -0.7471305 |
| -0.1746942 | -1.0088577 | -0.2640675 | -0.6147342 | -2.0110119 | -0.5498260 | -0.8422393 | -1.8269475 | -0.1993092 |
| -0.1263022 | -1.0239153 | -0.5525335 | -0.5807237 | -1.3308336 | -0.1048542 | -0.6504874 | -1.2440166 | -2.5925371 |
| 0.1282951 | -0.2734956 | -0.3195824 | -1.0567622 | -1.5380478 | -0.3361301 | -0.9879326 | -1.3209109 | -1.2468240 |
| -1.1739725 | -2.2058415 | -0.3872185 | -2.0174850 | -2.8407393 | -0.6310014 | -1.8094879 | -2.3175195 | -0.4660516 |
| 0.1011792 | -1.2477486 | -0.3933507 | 0.2601304 | 0.8181094 | -0.0253867 | 0.4245367 | 0.5660718 | -2.3201494 |
| -1.3349352 | -2.8680845 | -0.2275163 | -0.6262211 | -1.7091731 | -0.4957304 | -0.5833328 | -1.6079455 | -1.2713198 |
| -0.3422371 | -0.1289946 | -0.9687151 | -1.4137783 | -0.8062046 | -1.1978130 | -1.4392819 | -0.6726868 | -0.1168877 |
| -1.6241180 | -3.2749823 | -0.4696944 | -2.3865797 | -3.9175807 | -0.8084083 | -2.8676716 | -3.4476985 | -0.1579730 |
| -0.1567616 | 0.2641416 | 0.0111459 | -0.3462354 | 0.4253257 | -0.2214763 | -0.3960890 | 0.3723137 | -1.5296221 |
| -0.2714976 | -1.4703781 | -0.0358908 | -0.4028855 | -1.1261622 | -0.1345397 | -0.1268100 | -1.4158341 | -0.3612262 |
| -0.5043921 | -0.5187304 | -0.2899054 | -0.5411471 | -0.6770512 | -0.5318718 | -0.6539261 | -1.0286121 | -0.8829378 |
| -0.2488266 | -0.8390135 | -0.1959612 | -0.2296825 | -0.7315908 | 0.1212683 | -0.3817627 | -0.5227633 | -0.3082217 |
| -0.5603360 | -0.7325717 | -0.3469717 | -0.5092111 | -0.8951819 | -0.4976264 | -0.8186488 | -0.6371718 | -0.9420719 |
| 0.4678092 | 0.9317462 | -0.0523609 | 0.1313140 | 0.9197163 | -0.0326595 | 0.0389162 | 1.1029531 | -0.9079933 |
| 0.4941486 | 0.6608337 | -0.0710348 | 0.2798976 | 1.1671139 | -0.0030224 | 0.3211165 | 1.0438670 | -1.2682237 |
| -0.4850776 | -1.7749346 | -0.3948159 | -0.0708892 | -1.4719034 | -0.6636871 | -0.7003865 | -1.7474723 | -0.3510374 |
| -0.3960188 | -1.1609959 | -0.1810049 | -0.6028053 | -0.5110256 | -0.2359025 | -0.7457134 | -0.6073333 | -1.0663275 |
| -0.3522441 | 0.0138043 | -1.3340862 | -0.6049415 | 1.0598538 | -1.1313428 | -0.4886478 | 0.5126961 | -0.9809155 |
| -0.4641217 | -0.3101691 | -0.2620161 | -0.6204301 | -1.2094961 | -0.1797935 | -0.9697264 | -1.1442327 | -0.5912842 |
| -0.3771481 | -0.5583281 | -0.0016218 | -0.1371228 | -1.7461344 | -0.6124247 | -0.0951188 | -1.0932240 | -0.2944242 |
| -0.5815287 | -1.7115339 | -0.1292732 | -0.5662146 | -0.9511508 | -0.4445912 | -0.6790774 | -0.9053879 | -0.8380366 |
| -1.6375425 | -3.1270169 | -0.6864633 | -2.3900460 | -2.5544335 | -0.7361045 | -2.2447183 | -2.4711772 | -0.6792822 |
| -0.1733421 | -0.2572266 | -0.2537492 | -0.2103030 | -0.3818756 | -0.1976429 | -0.4505010 | -0.2533800 | -0.0416657 |
| -0.0533403 | -0.9518475 | -0.4958933 | 0.9982122 | 0.6074409 | -0.9991330 | 0.8560875 | 0.5417054 | -0.1146633 |
| -0.2707286 | -1.1033999 | -0.5303537 | -0.6526969 | -0.3924883 | -0.6354882 | -0.5480875 | -0.6908619 | -0.2446534 |
| 0.5472867 | 0.5375005 | -0.6967254 | -0.3940121 | 1.5074939 | -0.0828820 | -0.4917072 | 1.5338203 | -2.2120211 |
| -0.3492601 | -1.3892965 | -0.4827640 | -0.8430956 | -1.9153725 | -0.3301728 | -0.6156262 | -1.5454257 | -1.1630893 |
| -0.8148101 | -0.6766695 | -0.5017915 | -0.9174016 | -1.1474220 | -0.5973932 | -1.1049203 | -1.3104379 | -1.2541644 |
| -0.5220858 | -1.0911156 | -0.4099975 | -0.2386300 | -0.2204164 | -0.4978123 | -0.6157048 | -0.4114338 | -0.5144694 |
| -0.8063654 | -0.5054200 | -0.3185536 | -0.7098880 | -0.1227533 | -0.4500909 | -0.6065389 | -0.1016918 | -1.1178336 |
| -0.6001702 | -1.8882056 | -1.0937820 | -1.3241241 | -2.1216881 | -0.5161988 | -1.8681150 | -1.8178400 | -2.1904341 |
| -0.6938781 | -0.7131281 | -0.3989535 | -0.8276788 | -0.7307393 | -0.4148203 | -0.9037575 | -0.7491622 | -0.8371330 |
| -1.8673215 | -2.9008907 | -0.6794762 | -0.9667227 | -0.7523971 | -1.3311974 | -0.8857070 | -0.7111226 | -1.7229872 |
| -0.4247150 | -0.3200692 | -0.7913245 | -0.4943140 | 1.2540384 | -0.3365408 | -0.2314368 | 0.9309185 | -1.9071868 |
| -1.3368632 | -1.6212232 | -0.5455155 | -1.4208006 | -1.4321048 | -0.6223682 | -1.2019856 | -0.9206483 | -0.8298392 |

| | | | | | | | | |
|---|---|---|---|---|---|---|---|---|
| -0.0608549 | 1.1695955 | -0.3784133 | -0.3491376 | 2.0013174 | -0.4046442 | -0.0650601 | 2.4088494 | -1.4263412 |
| -0.1642887 | -0.0785745 | -0.1585683 | -0.7557154 | -0.2776079 | -0.6634709 | -1.3485433 | 0.0236500 | -0.5996454 |
| -0.3275264 | -0.3876022 | -0.1665191 | -0.2542946 | 0.1838432 | -0.3326923 | -0.2173601 | 0.0222979 | -0.7140783 |

| PG_399 | PG_400 | PG_401 | PG_402 | PG_403 | PG_404 | PG_405 | PG_406 | PG_407 |
|---|---|---|---|---|---|---|---|---|
| -0.718416 | -1.024411 | -1.012572 | -0.825592 | -0.450507 | -0.824681 | -1.240709 | -1.020675 | -0.652832 |
| -1.621310 | -1.497896 | -1.878211 | -1.290515 | -0.713890 | -0.689658 | -0.701630 | -0.712206 | -0.821314 |
| 0.3576175 | -0.235771 | 0.6621888 | 0.5949497 | 0.6804082 | -0.222390 | -0.102181 | -0.774457 | -0.432734 |
| -2.343944 | -2.774736 | -2.481685 | -1.766051 | -0.467914 | 0.0199767 | -0.069727 | -0.060180 | -0.093410 |
| -1.501545 | -1.699389 | -1.346124 | -1.258843 | -0.959415 | 0.1515637 | -0.276376 | -0.562974 | 0.1098387 |
| -1.293715 | -2.389433 | -0.961036 | -1.483553 | -1.302073 | -0.081056 | -0.390941 | -1.085977 | -0.233141 |
| -1.089266 | -0.920350 | -0.946084 | -0.909817 | -0.601527 | -0.197986 | -1.037669 | -1.104802 | -0.200245 |
| -0.190169 | -2.842089 | -0.125541 | -0.287667 | -1.127337 | -0.369841 | -0.976242 | -0.395161 | -0.383568 |
| -0.624096 | -0.133976 | -0.778623 | -0.307252 | -0.103075 | -0.625614 | -0.210698 | -0.962794 | -0.511663 |
| -0.369762 | -0.878511 | -0.414872 | -0.389296 | -0.392186 | -0.252306 | -0.488940 | -0.411733 | -0.399820 |
| -1.509850 | -1.904761 | -1.408627 | -1.440527 | -0.984691 | -0.317150 | -0.201463 | -0.421452 | -0.224714 |
| -2.001155 | -1.949150 | -2.121249 | -1.417129 | -0.872824 | -0.317883 | -0.415748 | -0.356536 | -0.329601 |
| -1.682015 | -2.180596 | -1.687507 | -1.706109 | -0.552345 | -0.357490 | -0.376113 | 0.0076561 | -0.304896 |
| 0.0719371 | 4.1448404 | 0.3359540 | 3.2535300 | 3.3599676 | 0.1865414 | 2.6244242 | 3.6200990 | 1.5247072 |
| -2.982328 | -2.537793 | -2.598893 | -2.490251 | -0.340491 | 0.0439485 | -0.862499 | -0.516767 | -0.241507 |
| 0.8733506 | 2.6276994 | -0.020113 | 2.3542758 | 1.8214184 | -0.619626 | 1.8692858 | 1.3625497 | -0.003654 |
| -1.524492 | -1.052442 | -1.422403 | -2.085308 | -0.405016 | -0.625893 | -1.934253 | -1.218224 | -0.717869 |
| -0.661157 | -1.450369 | -0.389644 | -0.392804 | -0.501672 | -0.866515 | -0.959804 | -0.757333 | -1.053980 |
| 0.3802045 | 0.0710704 | 0.6459049 | 0.4341124 | -0.209678 | -0.227123 | -0.134681 | 0.0994281 | -0.329671 |
| -0.884711 | -3.394467 | -0.928853 | -0.985893 | -0.965054 | -0.456325 | -0.725555 | -1.002645 | -0.734480 |
| -1.276786 | -0.790968 | -2.138062 | -0.560546 | 0.2935970 | 0.1432361 | 0.2488953 | 0.6833955 | 0.2343237 |
| -0.474892 | -0.307169 | -0.236821 | -0.339024 | -0.361903 | -0.526585 | -0.601130 | -0.383856 | -0.347302 |
| 0.9123821 | 1.6865349 | 0.4506262 | 1.2825784 | 1.3081145 | -0.073566 | 1.2762751 | 0.9986344 | 0.1600266 |
| -1.462628 | -1.964614 | -1.035075 | -1.506917 | -1.370084 | -0.372067 | -0.831835 | -1.125133 | -0.457445 |
| -1.715652 | -4.008118 | -1.503575 | -2.289083 | -2.436776 | -0.098140 | -0.661707 | -1.926762 | -0.363829 |
| 0.9000840 | 1.9652401 | 0.1072597 | 0.7721551 | 1.3703333 | -0.102266 | 0.7383063 | 0.8617395 | -0.040078 |
| -2.468313 | -2.778054 | -2.773096 | -1.966744 | -0.925258 | 0.2420686 | -0.145425 | -0.056958 | 0.1533645 |
| -2.003273 | -2.484768 | -2.208406 | -2.214553 | -1.304814 | -1.240205 | -0.732283 | -1.670488 | -1.071255 |
| -0.125188 | -1.319536 | -0.169405 | -0.611428 | -0.207857 | -1.512773 | -1.586292 | -1.463785 | -1.670047 |
| -1.954057 | -2.288259 | -2.174339 | -1.729478 | -0.984148 | -0.398722 | -0.766204 | 0.0379409 | -0.508491 |
| -0.383261 | -2.024433 | -0.313656 | -0.525318 | -0.602015 | -1.330254 | -1.923651 | -0.687130 | -1.502367 |
| -0.259368 | 0.0161222 | -0.383149 | 0.2071210 | -0.061647 | -0.079299 | -0.565395 | -0.386082 | -0.476460 |
| -1.536245 | -0.521247 | -1.441056 | -0.640729 | -0.509082 | -0.189661 | -0.109394 | -0.268325 | -0.113407 |
| -0.785697 | -2.630779 | -0.612885 | -1.132465 | -1.312023 | -0.294248 | -0.660947 | -0.617124 | -0.418990 |
| -0.117660 | -0.000344 | -0.004234 | -0.185328 | -0.127803 | -0.128821 | -0.287558 | -0.112836 | -0.184221 |
| -1.333116 | -1.054575 | -1.322952 | -0.926416 | -0.547931 | -0.373021 | -0.469570 | 0.0120539 | -0.835948 |
| -1.009432 | -1.411866 | -1.475958 | -1.628887 | -1.046473 | 0.1125714 | -0.472370 | -0.346556 | -0.163477 |
| 1.4072189 | 2.3534361 | 0.5442225 | 4.1907122 | 1.8189393 | 1.2736781 | 3.4203496 | -0.103131 | 2.3178125 |
| -0.550699 | 0.3037891 | -0.725659 | -0.365513 | -0.366504 | 0.0541213 | -0.508823 | -0.250262 | -0.112632 |
| -1.806840 | 0.4729033 | -1.522507 | -0.425808 | 0.5484666 | -0.303172 | -0.674838 | -0.811999 | -0.507371 |
| 0.3465942 | 0.6477567 | 0.5371248 | 0.3595553 | 0.2595107 | -0.170623 | 0.0482636 | 0.6220508 | -0.138656 |
| -0.758175 | -2.384147 | -0.526784 | -1.336240 | -2.007116 | -0.298848 | -0.504762 | -1.137705 | -0.392035 |
| -0.933393 | -0.763164 | -0.779484 | -0.582055 | -0.162221 | -1.297098 | -1.839229 | -1.407572 | -1.422969 |
| -1.999984 | -2.454133 | -2.057104 | -1.757192 | -1.215313 | -0.060724 | -0.280878 | -0.923605 | -0.257894 |
| 0.8985763 | 2.1802197 | 0.4348041 | 1.2946095 | 1.1621521 | -0.442403 | 0.8842484 | 0.4643832 | -0.050784 |
| -0.136605 | 3.6696406 | 0.0667452 | -0.244687 | 0.6397824 | 0.0632630 | -0.100071 | 0.1212166 | -0.162111 |
| 0.6037678 | -0.317106 | 1.0760840 | 0.3629297 | -0.408306 | -0.105732 | -0.501652 | -0.224093 | -0.071878 |

| | | | | | | | | |
|---|---|---|---|---|---|---|---|---|
| -0.9488927 | -0.6291604 | -1.6601172 | -0.9163631 | -0.2153046 | -0.6676369 | -0.4818402 | -0.0658290 | -0.5158242 |
| -0.7665847 | -2.5709006 | -0.9645497 | -0.7671928 | -1.6835963 | -0.3072676 | -0.9129425 | -1.6412717 | -0.4550548 |
| -1.2678403 | -0.1863219 | -1.5010389 | -0.8614975 | -0.1510586 | -0.1468523 | -0.1232100 | 0.3749566 | -0.0648624 |
| -0.5629303 | 0.0536163 | -0.1205307 | -0.4610240 | -0.4700889 | -0.7203050 | 1.4864763 | 0.6501694 | 0.0514953 |
| -0.2081511 | -0.1980002 | -0.3468458 | -0.2318334 | -0.1412432 | -0.5462794 | -0.3271380 | -0.2756871 | -0.4570536 |
| 0.3989776 | 0.4760616 | 0.3567604 | 0.1255552 | 0.3106856 | -0.3021638 | -0.5071153 | 0.2256324 | -0.2907105 |
| -1.5123656 | -1.4512992 | -0.8926416 | -1.3101340 | -0.7379375 | -0.0922177 | -0.6600596 | -0.7256928 | -0.2621252 |
| 0.6890582 | 2.9467017 | -0.1217578 | 2.2416677 | 2.3964015 | -0.2295620 | 0.9025167 | 2.7892710 | 0.5202561 |
| 0.6178861 | 0.2047851 | 0.6983779 | 1.5411596 | 0.7897533 | 1.7460292 | 3.6313985 | 1.7529917 | 3.0561022 |
| -0.8733738 | -0.5777772 | -1.9568852 | -0.7767050 | -0.1690455 | -1.5933747 | -0.5100393 | 0.0892669 | -1.3159083 |
| -1.6911002 | -1.7669154 | -1.3795164 | -2.2397078 | -1.4126420 | -0.9731593 | -1.3195775 | -1.6710196 | -1.3757919 |
| 0.7581527 | 2.9204770 | -0.3425118 | 2.6314496 | 1.6120044 | -0.0043445 | 2.9192093 | 1.6351292 | 1.2956058 |
| -0.4880856 | 1.2200641 | -0.8810431 | 1.6374577 | 1.8302903 | -0.0388437 | 2.3430799 | 2.5042987 | 0.6300385 |
| -0.5661456 | -0.9630655 | -0.9408973 | -0.6173913 | -0.1391823 | -0.5435947 | -0.5761270 | -0.7342927 | -0.4192809 |
| -2.6374017 | -2.7607658 | -2.5050576 | -2.0843623 | -1.6492430 | -0.7805360 | -1.4282376 | -1.7221172 | -0.8578330 |
| -1.4242090 | -0.7238863 | -1.1007300 | -1.3374360 | -0.6122479 | -0.6497109 | -1.0553247 | -1.0185733 | -0.8923423 |
| -1.0224131 | -2.8615960 | -1.2421678 | -1.3304549 | -1.6737612 | -0.2156819 | -0.6214180 | -1.8810819 | -0.3471048 |
| -1.1303818 | -0.9609185 | -1.1355172 | -1.6059226 | -0.7599814 | -0.0357074 | -0.3766313 | -0.7024578 | -0.0953897 |
| -2.0593394 | -2.3976420 | -2.3098367 | -2.6485588 | -2.0901889 | -0.0543900 | -0.6003002 | -0.9067572 | -0.1793875 |
| -3.3134687 | -2.9506612 | -2.6879214 | -3.1359409 | -1.2412507 | 0.0402535 | -0.1747279 | -0.3630915 | -0.0512675 |
| 0.2225534 | -1.4062423 | -0.4787687 | 0.0209660 | -0.4926724 | -0.3745359 | -0.6880060 | -0.1774872 | -0.1359933 |
| -0.9322294 | -1.3928971 | -0.8286605 | -1.1256452 | -0.8688773 | -0.7330601 | -0.6014224 | -0.6144022 | -0.6017251 |
| 0.3447163 | 1.0477858 | 0.6569413 | 0.1265224 | 0.7933878 | -0.0913242 | -0.2645229 | 0.3959566 | -0.1910496 |
| -2.6587325 | -2.5288676 | -3.3679886 | -2.3595589 | -0.8281593 | 0.4917203 | 0.5569208 | 0.9448665 | 0.3111734 |
| -2.4690730 | -2.3715623 | -1.7764023 | -1.6543683 | -1.8135268 | -0.9990071 | -1.2525914 | -1.7533228 | -1.1630425 |
| -1.1004949 | -1.1505480 | -1.3351926 | -0.9277126 | -0.2858674 | -0.0340764 | -0.5386794 | -0.4296300 | -0.1925256 |
| -1.2770714 | -1.7658944 | -0.6961657 | -1.8392505 | -1.6225465 | -0.9132458 | -1.3662207 | -1.2875486 | -0.9099331 |
| 0.7703407 | 3.5661966 | 0.2544545 | 0.9764449 | 3.1524535 | -0.1121164 | 1.7812859 | 3.7316534 | 0.0168741 |
| -0.3790925 | 1.8208917 | -0.8742025 | 1.0160695 | 1.6951247 | -0.2159220 | 0.3212966 | 0.8063728 | -0.2812230 |
| -0.6972730 | -1.6326489 | -0.9287249 | -0.9166779 | -0.8386504 | -0.1177714 | -0.7696406 | -1.0775592 | -0.0401134 |
| -0.1899342 | 0.8118872 | 0.2266984 | -0.1274581 | 0.6711879 | -0.1510618 | -0.4765324 | -0.2632585 | 0.1068476 |
| -3.2708560 | -2.5936226 | -3.0851216 | -2.0742044 | -1.3710383 | -0.0002962 | -0.6050363 | -0.1149935 | -0.1149520 |
| -1.7911974 | -2.0415712 | -1.3808216 | -1.8891442 | -1.6999856 | -0.4087595 | -1.1417314 | -1.2718349 | -0.7192726 |
| -1.7559598 | -2.6889261 | -2.2962984 | -1.8008925 | -0.8960405 | -0.1524474 | -0.2786510 | -1.2682894 | -0.2376797 |
| 0.2792332 | 0.9586958 | -0.2469886 | 0.6424820 | 1.0240041 | -0.2845423 | 0.4040918 | 1.7329280 | -0.1698288 |
| -0.0034925 | -0.6489409 | -0.0835422 | 0.8182319 | -0.5487202 | -0.5871231 | -0.8491994 | -0.6079376 | -0.9013475 |
| -1.5086436 | -1.7506133 | -0.6390207 | -1.0692534 | -1.4488927 | -0.1254034 | -0.3522720 | -0.1430779 | -0.1548308 |
| -0.8051583 | -2.4753398 | -1.0454246 | -1.2543905 | -1.4318136 | 0.0506083 | -0.1262825 | -0.7086523 | -0.0144180 |
| 0.1257315 | -0.8597141 | 0.4692977 | -0.4116515 | -0.8215609 | -0.2070458 | -0.6273431 | -0.5251341 | -0.5918083 |
| -0.7371766 | -1.4365688 | -0.2581764 | -0.8216736 | -1.4140554 | -0.3654106 | -1.0787592 | -1.6352495 | -0.4605583 |
| -1.4115908 | -1.0361108 | -1.5511023 | -0.9939022 | -0.7752649 | -0.5000733 | -0.6034801 | -1.0981141 | -0.5258766 |
| -0.4471022 | -1.538934 | -0.1387838 | -0.6853870 | -0.8231618 | -0.9547079 | -1.4394497 | -1.1751413 | -0.8916737 |
| 1.8125033 | 3.8328624 | 1.0609102 | 2.8278989 | 4.0694489 | -1.1694129 | -1.1681423 | -0.7970101 | -0.6850729 |
| -1.0172206 | -1.0095799 | -1.1020157 | -0.6725792 | -0.1315611 | -0.0353878 | -0.0916039 | -0.0589569 | -0.0758365 |
| -2.3991783 | -2.0923760 | -1.8610070 | -1.8434786 | -1.1288229 | -0.0103928 | -0.1571171 | -0.2480981 | -0.0172722 |
| -0.1861894 | -0.4682266 | -0.1761133 | -0.5661598 | -0.5857911 | -0.8860684 | -0.8903868 | -1.0664353 | -0.7087762 |
| 2.1678610 | 8.2591487 | 0.1847651 | 6.5426614 | 4.1457234 | -0.2076027 | -0.1005918 | -0.3239092 | -0.2189852 |
| -0.2685624 | 1.4190760 | -0.8916227 | 1.1307060 | 1.0004974 | -0.1730710 | 1.0241792 | 1.1265431 | -0.0400618 |
| -1.5595760 | -1.3254640 | -1.9072701 | -1.4684549 | -1.4972003 | -0.6799112 | -0.7497088 | -1.3370331 | -0.9637924 |
| -0.3628870 | 0.2519521 | -0.6713055 | -0.3311103 | 0.2949574 | -0.3887012 | -0.1716040 | -0.0244848 | -0.6392394 |

| | | | | | | | | |
|---|---|---|---|---|---|---|---|---|
| -0.9955915 | -1.6575586 | -1.2675964 | -0.8119540 | 0.0919876 | -1.2737905 | -1.3887072 | -1.4256898 | -1.3391892 |
| -1.1948494 | -0.2709769 | -0.5960530 | -0.4582659 | 0.1228845 | -0.0431624 | -0.0050040 | 0.5757772 | 0.0254812 |
| -0.4138644 | -0.6698483 | -0.4141577 | -0.4003566 | -0.8798338 | -0.4024138 | -0.3626265 | -0.1061464 | -0.5699783 |
| -0.8532204 | -0.4630841 | -0.9414027 | -0.5145395 | -0.1633854 | 0.0120750 | 0.0436116 | -0.1001295 | -0.0034355 |
| -1.3214925 | -2.1292570 | -1.1185698 | -2.2749333 | -1.9113205 | 0.0222267 | -0.9268856 | -1.7138907 | -0.0251231 |
| -0.5492136 | 0.0937580 | -0.7776817 | -0.1454651 | 0.2444065 | -0.7737847 | -1.2875218 | -1.2944085 | -0.7098307 |
| -1.7412761 | -1.5750621 | -2.4089263 | -1.5786459 | -1.0090654 | -0.2395636 | 0.0769018 | -0.0992158 | -0.1467529 |
| -0.0754970 | -0.0085992 | 0.1221768 | -0.3051371 | -0.1414521 | -0.1832513 | -0.1132428 | 0.1081768 | -0.1160360 |
| -0.0685619 | -0.4306478 | 0.6181430 | 0.0655928 | -0.5540358 | -0.1100453 | -0.0372172 | -0.4099048 | -0.0012604 |
| 0.0513141 | 1.0153353 | -0.4338492 | 0.3838492 | 0.7532602 | -0.1604903 | 0.2274767 | 0.2378535 | -0.0342093 |
| -0.8302194 | -1.6236728 | -1.1832932 | -0.9202546 | -1.0623155 | -0.2120065 | -0.4527181 | -0.1000973 | -0.4365420 |
| -2.1619246 | -2.7711738 | -3.5825931 | -1.6569397 | -0.5855974 | -0.4053014 | -0.3723443 | 0.5694432 | -0.4790746 |
| -0.2812159 | -3.0425214 | -0.2888742 | -0.6194626 | -0.9456780 | -0.1659784 | -0.7713649 | -0.2699638 | -0.5357516 |
| -0.9264504 | -1.9050185 | -1.1225651 | -1.0375008 | -0.7726589 | -0.5526439 | -0.9695500 | -0.5610825 | -0.7793606 |
| -1.0189590 | -0.7809320 | -1.0893943 | -1.0165121 | -0.0422589 | -0.4479485 | -0.3262822 | 0.0647990 | -0.6509935 |
| -0.0361268 | -0.1248767 | -0.0096729 | -0.3187733 | -0.1358112 | 0.0403918 | 0.1560500 | -0.0982834 | -0.0383354 |
| -1.1704578 | -1.3681241 | -2.0018216 | -1.5649500 | -0.5520472 | -0.7874015 | -1.0140513 | -0.5731436 | -1.2555410 |
| -0.3692291 | 0.2797299 | 0.0265868 | -0.1097021 | -0.1201288 | -0.5356376 | -0.2571170 | -0.3920339 | -0.4606298 |
| -0.8830568 | -1.6408837 | -1.9177610 | -1.0832445 | -0.7558457 | -0.1485210 | -0.9280901 | -0.7471161 | -0.4025769 |
| 0.3956549 | 1.5746215 | 0.5045040 | 0.6728776 | 1.4136842 | -0.3297440 | 0.3487726 | 1.9605363 | -0.4110653 |
| -0.5641574 | -1.4611776 | -0.7555302 | -0.6863087 | -0.5742284 | -0.6995596 | -1.2011862 | -1.7064104 | -0.6702321 |
| -1.2753903 | -0.8868236 | -1.2914379 | -1.1461759 | -0.4302614 | -0.1234443 | -0.2710333 | -0.1602481 | -0.1787145 |
| -0.2219848 | -0.2456258 | 0.1436405 | -0.1152437 | -0.2714730 | -0.1686820 | -0.1260366 | -0.1813815 | -0.3111708 |
| -1.5203337 | -1.4881174 | -1.1496587 | -1.1875599 | -0.4039031 | -0.7086312 | -1.0770665 | -0.2148488 | -0.6224208 |
| -0.4320939 | -0.1480284 | -0.0565324 | -0.3913335 | -0.2389215 | -0.1198365 | -0.5436776 | -0.2651162 | -0.3661351 |
| -1.7156328 | -1.8651551 | -1.6848461 | -1.9151063 | -0.7789045 | -0.2617269 | -0.3669958 | -0.5616186 | -0.2756384 |
| -0.9923690 | -1.5045405 | -1.2884575 | -1.0256991 | -0.5000560 | -0.0716761 | -0.5624406 | -0.6832897 | -0.1670897 |
| 0.9584460 | 1.4483482 | 0.9710304 | 1.1707957 | 1.0062285 | -0.5445624 | -0.3550379 | 0.3705854 | -0.2892404 |
| -0.8970860 | -0.7892047 | -1.0294552 | -0.8542085 | -1.3684849 | -1.0193397 | -0.7103732 | -0.9739706 | -0.9992291 |
| -1.5360875 | -1.6480312 | -1.5692173 | -0.6878159 | -1.4944538 | -0.2430196 | -0.0940533 | -0.8059588 | -0.1342500 |
| -1.0231698 | -1.7456912 | -0.9310014 | -1.2838568 | -1.6966738 | -0.5591151 | -0.7669849 | -1.3582604 | -0.5021222 |
| -1.7731405 | -2.5553801 | -1.7348127 | -1.9671149 | -1.4132075 | -0.1993599 | -0.3182195 | -1.6806462 | -0.3984238 |
| -1.3004924 | -1.0427890 | -0.6430515 | -0.8331675 | -1.2320867 | -0.4765371 | -0.4216165 | 0.2991215 | -0.4811639 |
| -0.1971863 | -0.7720782 | -0.3910761 | -0.4287950 | -0.4972452 | 0.5346511 | 0.0975777 | -0.4381257 | 0.5669835 |
| -0.3377531 | -2.2475572 | -0.5725742 | -0.6361786 | -1.8941636 | 0.0989917 | -0.0298074 | -0.7329703 | 0.1088164 |
| -1.3925468 | -1.5007902 | -1.7755954 | -1.3174861 | -0.7790114 | -0.3482528 | -0.3950039 | -0.8928029 | -0.4045601 |
| -1.6232312 | -0.8876172 | -1.5137160 | -1.1942594 | -0.6254185 | -0.2609375 | -0.2818308 | -0.5918867 | -0.2832985 |
| -2.3774388 | -2.2703679 | -2.3279670 | -1.4560232 | -0.8374644 | 0.0564583 | 0.1332990 | -0.3666998 | 0.0088786 |
| -0.6034290 | -0.9180358 | -0.9621815 | -0.8070506 | -0.4964523 | -0.2794845 | -0.3739627 | -0.6738238 | -0.3966569 |
| -1.2350194 | -0.6820453 | -0.4315383 | -0.5074249 | -1.7894922 | -1.0444121 | -0.6651768 | -1.0900066 | -0.8600734 |
| -1.0362362 | -0.8906748 | -1.4704688 | -0.4330722 | 0.3606341 | -0.2275965 | -0.5480974 | 0.2833804 | -0.2872483 |
| -1.5715730 | -1.3635322 | -1.2826041 | -0.5782842 | -0.6981305 | -0.1814945 | 0.0961690 | 0.1617053 | -0.0992273 |
| -1.5043672 | -1.5439487 | -1.8841161 | -0.8769751 | -0.4108371 | 0.1049304 | -0.2638810 | -0.0230191 | -0.0734858 |
| -1.4238862 | -1.0676734 | -0.7488250 | -0.5372582 | -0.1689499 | -0.7923814 | -0.2654920 | -0.6171120 | -0.6755033 |
| -0.0080186 | -0.1628605 | 0.0486694 | -0.0885181 | -0.1207170 | -0.2112355 | -0.1646002 | -0.1573551 | -0.1827302 |
| -0.8448155 | -0.3617200 | -1.1248401 | -0.2230637 | 0.1129264 | -0.3124583 | 0.1432782 | 0.3333699 | -0.0104971 |
| -1.5019079 | -0.6681298 | -1.2976970 | -1.4247097 | -0.6022517 | -0.4061774 | -0.7251592 | -0.8406355 | -0.4217833 |
| -0.4794465 | 0.5547495 | -1.4832590 | 0.0207883 | 0.5035819 | -0.3866854 | -0.2503139 | 0.2995857 | -0.2479461 |
| -1.3493217 | -2.9711098 | -1.4409515 | -1.8107405 | -2.3832521 | -0.6726007 | -0.7977830 | -0.9915007 | -0.4888905 |
| -1.3497593 | -1.4465593 | -1.4235036 | -1.3565957 | -1.2584245 | -0.8358255 | -0.8732109 | -1.7553974 | -0.9243642 |

| | | | | | | | | |
|---|---|---|---|---|---|---|---|---|
| -1.5387278 | -0.7497776 | -2.2646788 | -1.1510380 | -0.0891731 | 0.3409609 | 0.1053614 | 0.0734024 | 0.1667186 |
| -1.9993541 | -1.7083599 | -0.9049908 | -1.7589868 | -1.5106546 | -0.4888329 | -0.9026141 | -1.1405348 | -1.0005095 |
| -1.4143919 | -0.4834524 | -0.8715113 | -0.8878572 | -0.5436793 | -0.4921442 | -0.5639359 | -0.8098519 | -0.5046574 |
| -0.2538350 | 0.2576395 | -0.2693281 | -0.1188796 | 0.0875472 | -0.6799350 | -0.3836794 | -0.2398135 | -0.9960156 |
| -2.0022449 | -2.8465346 | -2.2836691 | -2.0372686 | -2.3812180 | -0.2869097 | -0.4104939 | -0.5906725 | -0.0552812 |
| 1.7542419 | 2.3742468 | 0.9034351 | 2.2352930 | 1.5090727 | 0.0502285 | 1.8920245 | 2.3213353 | 0.5012578 |
| -1.2663413 | -1.5244429 | -1.1783387 | -0.8976997 | -0.8252261 | -0.2387093 | -0.4762899 | -0.5926947 | -0.3236087 |
| -0.8212804 | -0.6620045 | -0.9331367 | -1.1008696 | -0.6096859 | -0.1108065 | -0.1820409 | -0.4596711 | -0.3604993 |
| -1.5163037 | -1.9880194 | -1.3637193 | -1.7968983 | -1.5665015 | -0.2808776 | -0.8302179 | -0.9319192 | -0.1400315 |
| -1.1090442 | -0.4613110 | -0.8503253 | -0.6924133 | -0.3663702 | -0.6005516 | -0.9626228 | -0.6737345 | -0.7294301 |
| -1.0562787 | 0.0472478 | -0.7534028 | -0.4848373 | 0.0815914 | -0.2121674 | -0.0850765 | 0.2661357 | 0.0407546 |
| -1.2199148 | -1.3444211 | -1.1936195 | -1.6680843 | -1.1055096 | -0.2729933 | -0.6713146 | -1.3063716 | -0.5614018 |
| -2.4810705 | -0.8864163 | -1.8409393 | -2.9040651 | -1.7690017 | -1.1281584 | -1.3465503 | -0.2385639 | -1.3503760 |
| -0.9581727 | 0.4033073 | -0.9677919 | -0.2157319 | 0.2640301 | -0.4578730 | -0.8710116 | -1.1716307 | -0.3473335 |
| -0.3822148 | 0.4011899 | -0.4349690 | -0.3450124 | 0.0634996 | -0.0256040 | -0.5730722 | -0.2286679 | -0.0207105 |
| -1.3119356 | -2.6349385 | -1.2056219 | -0.8491602 | -1.2940199 | -0.8292015 | -1.1357465 | -1.2608409 | -0.8948632 |
| 0.5708008 | 0.8697128 | 0.7928989 | 0.4457568 | 0.0126757 | -0.7188894 | -0.1852181 | -0.6136099 | -0.4578746 |
| -1.6849663 | -1.9936472 | -0.7460927 | -3.1099399 | -0.9459492 | -0.5702718 | -0.5975903 | -1.3625674 | -0.8662649 |
| -0.9907423 | -0.5420020 | -1.1864143 | -1.0800591 | -0.3692815 | -0.3551294 | -0.3399384 | -0.8383719 | -0.7670139 |
| -2.2320761 | -2.5480807 | -2.5841148 | -1.8862288 | -1.2058578 | -0.4017664 | -0.3053452 | -0.4302875 | -0.5148439 |
| 0.5953628 | 1.6084790 | 0.5391123 | 1.0211116 | 0.3940546 | -0.0350207 | -0.0719743 | 0.0272468 | -0.1347076 |
| -1.7958105 | -1.2718372 | -1.4205373 | -1.2032031 | -0.4423862 | -0.2125534 | -0.0145980 | 0.1980385 | -0.2817478 |
| -1.1184046 | -1.9211515 | -0.5552785 | -1.1814345 | -1.1556663 | -0.5481457 | -1.2581273 | -1.9031888 | -0.7678669 |
| -1.810861 | -1.0630662 | -0.7374077 | -0.8033875 | -0.1936106 | -0.4198152 | -0.5975688 | -0.1700736 | -0.5575126 |
| -0.2479660 | -1.2456132 | -0.2515298 | -0.7730541 | -1.0075510 | -0.1039096 | -0.3004936 | -0.9056074 | -0.2400706 |
| -0.9914265 | -0.5434279 | -0.8219417 | -0.9513227 | -0.1442400 | -0.4012978 | -0.4426440 | 0.2891582 | -0.4786112 |
| -1.3619401 | -1.0264714 | -1.1686488 | -1.3800861 | -0.2092426 | -0.7436773 | -0.8490975 | -1.3144878 | -0.7941602 |
| -0.9934688 | -1.0820252 | -0.8919601 | -0.8080405 | -0.5653171 | 0.0069959 | -0.2594923 | -0.6008060 | 0.0050861 |
| -1.6498908 | -2.5494072 | -1.8903182 | -1.7174234 | -1.3451585 | -0.7936234 | -1.1009668 | -0.6959880 | -0.7160226 |
| -0.1794283 | 0.8939149 | -0.4957345 | -0.0575351 | 0.7054870 | -0.4067226 | 0.1853368 | 0.3512377 | -0.5143233 |
| 1.4724034 | 3.3533877 | 1.0872861 | 2.1792737 | 1.5621877 | -0.0076056 | 0.8593730 | 1.6211610 | 0.1625063 |
| -1.2499735 | -1.1342716 | -1.3265812 | -1.1288724 | -0.9642090 | -0.6417662 | -0.7933708 | -1.8664235 | -0.8111285 |
| -0.2186642 | -0.1174284 | -0.4635757 | -0.3702025 | 0.0583148 | -0.0634021 | -0.2043363 | -0.0080662 | -0.1008413 |
| -0.6241826 | -0.1298172 | -0.9211242 | -0.5919112 | 0.1870260 | 0.2287551 | 0.2107883 | 0.7046944 | 0.2232736 |
| 1.0418616 | 1.6387852 | 0.8585594 | 1.0172729 | 1.3753008 | 0.5835426 | 0.9033770 | 1.0802811 | 0.7382787 |
| -1.6208275 | -1.3295621 | -1.1664168 | -1.8680898 | -0.6508940 | -0.4411371 | -0.6068004 | -1.2859904 | -0.5601845 |
| -1.2857129 | -0.9415535 | -1.3538075 | -1.0491041 | -0.3904549 | 0.3811276 | 0.0933318 | 0.8574256 | 0.2259495 |
| -1.6065375 | -0.0366206 | -1.8987483 | -0.3361214 | 0.1534139 | -0.4130937 | -0.1423319 | -0.5129626 | -0.4438333 |
| -1.2065210 | -0.7949646 | -1.4015301 | -0.8299449 | -0.1654195 | -0.5223730 | -0.3297072 | -0.2233434 | -0.5350825 |
| -1.5702440 | -1.9483005 | -1.6449130 | -1.5764608 | -0.7643722 | -0.4157275 | -0.3054896 | -0.0817283 | -0.3420934 |
| -0.3758515 | 0.1244703 | -0.0385084 | 0.1782497 | -0.0351254 | -1.1135448 | -0.8056358 | -0.8258763 | -1.0287360 |
| -0.7399388 | 0.4951775 | -0.8282454 | 0.2584861 | 0.7756176 | -0.3246172 | 0.0070401 | 0.4336282 | -0.2441218 |
| -1.4562015 | -1.3895950 | -1.9368452 | -1.1356180 | -0.8333243 | -0.1305570 | 0.4223160 | 0.9179759 | -0.1385211 |
| -0.9455524 | -0.2532033 | -1.8407789 | -0.8328245 | 0.2805048 | -0.1888269 | 0.0183671 | 0.3421886 | -0.3318923 |
| -0.6042580 | -0.5822898 | -0.6164994 | -1.0352697 | -0.3019897 | -0.2252464 | -0.5347910 | -0.9494515 | -0.3706227 |
| -1.1927778 | -0.7165698 | -1.2350514 | -0.9116935 | -0.5862210 | -0.2561645 | -0.2911655 | -0.1422219 | -0.3910456 |
| -0.8153851 | -1.4031708 | -0.6499626 | -0.9778379 | -0.5473076 | -0.6744367 | -0.7360025 | -0.9680113 | -0.6047253 |
| -1.6369688 | -1.8187961 | -1.5468175 | -1.6111410 | -1.2643949 | -0.6991996 | -1.5814780 | -1.8553313 | -0.8455333 |
| -0.1268979 | -1.6558322 | -0.0172024 | -0.6306829 | -1.1762111 | -0.0662466 | -0.2446790 | -1.5256988 | -0.1933951 |
| -2.2264385 | -2.0224441 | -2.3174933 | -1.8854857 | -0.8203599 | -0.3272899 | -0.4609737 | 0.0234249 | -0.3150273 |

| | | | | | | | | |
|---|---|---|---|---|---|---|---|---|
| -3.606111 | -3.224570 | -4.079487 | -3.365609 | -0.600602 | -0.465522 | -0.474441 | -0.148090 | -0.670181 |
| -1.012556 | -1.546524 | -1.187285 | -1.546774 | -1.945612 | -0.281449 | -0.514181 | -1.428065 | -0.721063 |
| -1.468835 | -1.267556 | -1.890025 | -1.112577 | -0.716821 | -0.608212 | -0.258764 | -0.315950 | -0.741823 |
| -1.192841 | -2.272974 | -0.872410 | -0.849831 | -0.543628 | -0.998654 | -0.932681 | -0.407873 | -0.514328 |
| -1.622034 | -2.107062 | -1.210531 | -1.688170 | -1.070974 | -0.577168 | -0.562075 | -0.593993 | -0.999791 |
| -0.070884 | -0.620703 | -0.492095 | -0.267156 | 0.1813865 | -0.291704 | -0.088121 | -1.088200 | -0.191548 |
| 0.5891827 | 1.8784123 | 0.1502623 | 0.9911367 | 1.9326834 | -0.355600 | 1.5210881 | 2.7846851 | 0.1836751 |
| -0.071851 | 0.8448246 | -0.237249 | -0.181527 | -0.024335 | -0.083154 | -0.453128 | -0.124276 | -0.088068 |
| -3.500090 | -3.737149 | -3.922982 | -2.331120 | -0.827165 | 0.1729702 | -0.173811 | -0.279582 | -0.104519 |
| -0.873621 | 0.0194606 | -0.869097 | -0.507108 | 0.6080414 | -0.618679 | -0.756855 | 0.5124829 | -0.829981 |
| -0.570252 | -0.077790 | -0.550336 | -0.559613 | 0.3163823 | -0.747218 | -0.066868 | 0.5615283 | -0.784747 |
| -2.454157 | -1.840793 | -1.944279 | -2.347787 | -0.600230 | -1.088558 | -1.294414 | -0.995077 | -1.430362 |
| 0.3600270 | 0.2283839 | 0.3732465 | 0.0695360 | -0.144320 | -0.132054 | -0.269592 | -0.140526 | -0.110486 |
| -0.507972 | -0.942695 | -0.581232 | -1.289417 | -0.973664 | -0.253410 | -0.720161 | -0.164067 | -0.214059 |
| -0.843239 | -2.067301 | -0.873809 | -1.520453 | -1.016161 | 0.0258153 | -0.384805 | -1.615510 | -0.040762 |
| -0.661443 | -1.158161 | -0.451069 | -0.930244 | -0.863577 | -0.487628 | -0.771263 | -1.461576 | -0.822522 |
| -3.640399 | -4.749507 | -5.006061 | -2.569451 | -1.818585 | -0.102166 | -0.201123 | -0.115489 | -0.013019 |
| -1.235906 | -1.023307 | -1.193697 | -0.902904 | -0.988450 | -1.242532 | -1.139055 | 1.1396278 | -1.007944 |
| -1.359530 | -2.009564 | -1.328665 | -1.357553 | -1.241023 | -0.312616 | -1.166408 | -2.209748 | -0.680231 |
| -1.634800 | -1.330450 | -2.105564 | -1.514748 | -0.447137 | -0.177548 | -0.134460 | 0.2144589 | -0.270646 |
| -1.493897 | -1.961337 | -1.507058 | -1.236657 | -1.169474 | -0.356564 | -0.502523 | -1.126472 | -0.559971 |
| -0.078921 | -0.108925 | -0.053876 | -0.161135 | -0.270386 | -0.245778 | -0.517017 | -0.372925 | -0.341697 |
| -0.995584 | -3.649390 | -0.163814 | -1.641723 | -2.199515 | -0.935141 | -1.818148 | -1.199230 | -1.203290 |
| -1.530185 | -1.136954 | -2.188311 | -1.038940 | -0.321407 | -0.125033 | -0.234379 | 0.0661902 | -0.198491 |
| -0.577973 | -1.007126 | -0.290159 | -0.400773 | -0.880296 | -0.190618 | -0.134195 | -0.793999 | -0.424963 |
| -1.049798 | -0.349985 | -1.683097 | -0.879589 | -0.130518 | -0.331992 | -0.288375 | -0.513502 | -0.282102 |
| -0.664069 | -2.038875 | -0.562321 | -0.520502 | -1.549622 | -0.081961 | -0.168076 | -0.587704 | -0.135249 |
| -1.159802 | -1.162165 | -1.125093 | -0.811465 | -0.686626 | -0.556351 | -0.061389 | -0.785259 | -0.411168 |
| -0.511318 | 0.2817947 | -1.034000 | -0.000635 | 0.4782546 | -0.956100 | -0.235536 | 0.0653808 | -1.050911 |
| -1.420834 | -0.678383 | -1.660116 | -0.797771 | 0.2296662 | -0.440454 | 0.0022933 | 2.4576691 | -0.286675 |
| -0.418747 | -0.588293 | -0.533840 | -0.144546 | -0.242717 | -0.792278 | -0.677781 | -1.607610 | -0.712791 |
| -2.052376 | -1.745710 | -2.077766 | -0.952233 | -0.242389 | -0.074028 | -0.403470 | -0.223538 | -0.185206 |
| -0.390503 | 3.4191289 | -0.453025 | -0.561056 | 1.7674865 | -0.979668 | -1.024237 | 1.9976119 | -1.140232 |
| -0.545573 | -1.486117 | -0.609627 | -0.789770 | -1.048917 | -0.093568 | -0.374873 | -1.419085 | -0.208904 |
| -0.329909 | -0.742863 | -0.318031 | -0.323594 | -0.406790 | -0.293507 | -0.260366 | -0.488062 | -0.023969 |
| -1.204649 | -1.102151 | -1.188136 | -0.999352 | -0.120298 | -0.620899 | -1.225024 | -1.497089 | -0.688126 |
| -1.493778 | -0.917553 | -1.090585 | -1.288484 | -0.750756 | -0.688577 | -1.101953 | -0.756986 | -0.769600 |
| -0.235978 | -0.003457 | -0.150262 | -0.245478 | -0.261821 | 0.0383712 | 0.0110139 | -0.195573 | -0.236051 |
| -0.027453 | 0.3754628 | 0.1091862 | 0.1466760 | 0.6986299 | -0.551172 | 0.4067861 | 1.1050471 | -0.513658 |
| -0.416602 | -2.226786 | -0.528937 | -0.239142 | -0.410422 | -0.223703 | -0.174180 | 0.5809781 | -0.085496 |
| -3.060089 | -2.310603 | -3.578694 | -2.105415 | -0.269927 | -0.026984 | 0.0093035 | 0.5705503 | -0.294740 |
| -1.765006 | -2.520833 | -1.654028 | -1.912038 | -1.258409 | -0.287520 | -0.690292 | -1.362580 | -0.299327 |
| -1.527602 | -1.602360 | -1.685808 | -1.295977 | -0.782797 | -0.426592 | -0.552602 | -0.406999 | -0.360599 |
| -0.969560 | -0.478490 | -0.123489 | -0.962598 | -0.284043 | -1.153560 | 0.1077327 | 0.9387868 | -0.901668 |
| -0.980047 | -0.758429 | -1.428941 | -1.055208 | -0.409961 | -0.438227 | -0.184553 | 0.1356831 | -0.432630 |
| -2.777646 | -3.031175 | -3.210578 | -2.292968 | -1.283435 | -0.561545 | -1.033550 | -0.610464 | -0.797338 |
| -0.708647 | -1.670704 | -0.759618 | -0.950719 | -0.761134 | -0.126890 | -0.450668 | -0.288541 | -0.208743 |
| -1.980829 | -1.479755 | -1.710732 | -1.024982 | -0.477718 | -1.275169 | -1.131339 | -1.219061 | -1.549271 |
| -1.937042 | -0.790803 | -2.019542 | -1.216816 | -0.460193 | -0.391640 | 0.2193377 | 0.4241931 | -0.432197 |
| -1.374069 | -1.404230 | -1.383420 | -1.307184 | -1.128347 | -0.671348 | -0.818443 | -0.770046 | -0.597412 |

| | | | | | | | | |
|---|---|---|---|---|---|---|---|---|
| -0.9695924 | 1.0265214 | -0.7056145 | -0.3006895 | 0.2696341 | -0.5291647 | -0.3721845 | 0.0965223 | -0.3618720 |
| -1.1732180 | -0.6834460 | -1.1045173 | -0.7350868 | -0.2548660 | -0.7640338 | -0.8548041 | -0.8759051 | -0.4135497 |
| -0.5632642 | -0.4569919 | -1.0261477 | -0.5709216 | -0.1372181 | 0.0048573 | -0.1972720 | -0.0182664 | -0.0708350 |

| PG_408 | PG_409 | PG_410 | PG_411 | PG_412 | PG_413 | PG_414 | PG_415 | PG_416 |
|---|---|---|---|---|---|---|---|---|
| -1.187366 | -0.339694 | -0.374849 | -1.794787 | -0.421499 | -0.778419 | -2.197833 | -0.480021 | -0.487461 |
| -1.033394 | -0.375756 | -0.806055 | -0.904564 | -0.542235 | -0.834340 | -0.873386 | -0.562968 | -1.075289 |
| -0.361379 | -0.070036 | -0.864716 | -0.149567 | 0.0037090 | -0.967231 | -0.684668 | -0.137903 | 0.0350059 |
| -0.261727 | -0.320907 | -0.369640 | -0.373179 | -0.438959 | -0.827740 | -1.175344 | 0.4736442 | 0.0774035 |
| -0.947802 | 0.1392800 | 0.0259963 | -0.985829 | 0.1359503 | -0.052208 | -0.819305 | -0.313246 | -0.258554 |
| -0.781684 | -0.353298 | -0.703337 | -1.815074 | -0.567029 | -0.808519 | -1.920232 | -0.279248 | -0.434056 |
| -1.121194 | -0.390927 | -0.585323 | -0.757267 | -0.394196 | -0.691887 | -0.297086 | 0.0485438 | -0.672951 |
| -0.816490 | -0.203632 | -0.311184 | -2.587402 | -0.244571 | -0.425145 | -2.765626 | -0.143201 | -0.140819 |
| -0.720151 | -0.096169 | 0.0823030 | -0.236024 | 0.0002181 | -0.058848 | -0.657523 | -0.272274 | -0.040983 |
| -0.327451 | -0.685594 | -0.963288 | -0.667765 | -0.699980 | -0.667668 | -1.069231 | -0.040984 | -1.405870 |
| -0.434216 | -0.249418 | -0.120278 | -0.161297 | -0.304594 | -0.157334 | -0.337568 | -0.017334 | -0.072636 |
| -0.316542 | -0.209281 | -0.391168 | -0.556122 | -0.271868 | -0.616372 | -0.507791 | -0.310797 | -0.372316 |
| -0.318745 | -0.627558 | -0.794644 | -0.529967 | -0.491196 | -0.745434 | -0.976536 | -0.406428 | -0.675232 |
| 3.6175822 | -0.747188 | 1.7459747 | 2.7935095 | -0.067125 | 2.3386468 | 2.8322274 | 0.0406250 | 0.4824298 |
| -0.270768 | 0.1511182 | -0.435118 | 0.0901389 | -0.025682 | -0.420446 | 0.3296374 | -0.192009 | -0.359823 |
| 1.5759710 | -0.668215 | 2.7255570 | 1.5955537 | 0.7138331 | 2.7661051 | 2.6216058 | -0.098147 | 2.1550577 |
| -1.967864 | -1.143153 | -1.739626 | -1.835764 | -1.511202 | -1.810080 | -2.249300 | -0.438860 | -1.400794 |
| -1.019808 | -0.092997 | -0.190938 | -0.173829 | -0.169530 | -0.063192 | -0.385395 | 0.4696209 | -1.241556 |
| -0.047610 | -0.086366 | -0.830666 | -1.177131 | -0.507155 | -1.040158 | -1.096566 | -0.396933 | -0.259268 |
| -0.945447 | -1.219140 | -1.303352 | -1.011905 | -1.515637 | -1.083868 | -0.962042 | 0.0204503 | -0.753246 |
| 0.3019329 | -0.377718 | 0.7434343 | 0.1050069 | -0.136675 | 0.7336028 | -0.405865 | -0.380964 | 0.1911103 |
| -0.178573 | -0.354005 | -0.147122 | -0.492939 | -0.382729 | -0.360982 | -0.189406 | -0.408576 | -0.291326 |
| 1.1675954 | 0.0600095 | 0.9814528 | 1.7339055 | -0.019762 | 0.9513976 | 1.4460426 | -0.118785 | 0.7171058 |
| -1.557421 | -0.259314 | -0.533766 | -1.574405 | -0.279604 | -1.207302 | -1.532236 | -1.208200 | -1.402890 |
| -1.616751 | -0.418961 | -1.126185 | -1.743644 | -0.900804 | -0.912703 | -0.880130 | -0.244395 | -0.578281 |
| 1.1244881 | -0.079003 | 0.6644690 | 1.2245888 | -0.052086 | 0.6289698 | 0.6218466 | -0.256802 | 0.6151022 |
| -0.314631 | 0.1244217 | -0.040435 | -0.507469 | 0.1305432 | 0.1177617 | -0.161917 | 0.3870651 | 0.1326808 |
| -1.845029 | -0.499157 | -1.315190 | -0.819194 | -0.862690 | -1.353687 | -0.966381 | -0.374178 | -0.202337 |
| -1.626548 | -0.557469 | -0.947762 | -0.530491 | -0.672357 | -0.774021 | -0.381861 | 0.0151149 | -0.137171 |
| -0.517946 | -0.051463 | -0.292797 | 0.0823430 | -0.223583 | -0.293715 | 0.1143029 | 0.2014171 | -0.080219 |
| -1.539256 | -0.102836 | -0.573468 | -1.229112 | -0.197514 | -0.857351 | -1.048799 | -0.488604 | -0.550024 |
| -0.666752 | -0.684971 | -0.466304 | -1.204621 | -1.024039 | -0.197527 | -1.387654 | 0.1674573 | 0.2876035 |
| -0.291563 | -0.045286 | 0.3978622 | 0.5062603 | -0.047763 | 0.7566120 | 0.7386049 | -0.183402 | 0.1810593 |
| -0.898933 | -0.685370 | -0.103689 | -1.242308 | -0.614106 | 0.1999189 | -1.815009 | -0.300121 | -0.732091 |
| -0.062767 | -0.291811 | -0.194501 | -0.456707 | -0.272791 | -0.130435 | -0.261358 | -0.214722 | -0.188818 |
| -0.450346 | -0.661219 | -0.254024 | 0.5166474 | -1.578937 | -0.070483 | 0.4976800 | -0.251485 | -1.315085 |
| -0.667726 | -0.470546 | -0.380739 | -0.858513 | -0.686927 | -0.401751 | -1.361993 | -0.067013 | -0.773425 |
| -0.178323 | -0.022904 | 2.0832102 | 0.5327472 | 0.5106072 | 2.6909625 | 0.5728213 | -0.168412 | -0.394071 |
| -0.669300 | -0.957623 | -0.548027 | -0.253184 | -0.877778 | -1.277959 | -0.610046 | -0.211798 | -0.091439 |
| -0.704156 | -1.105583 | -0.466645 | 0.2952985 | -1.364766 | -0.346870 | 0.8869698 | -0.060454 | -0.773841 |
| 0.2202282 | 0.0774696 | 0.2995618 | 0.4440163 | 0.1526624 | 0.2695786 | 0.4923242 | -0.405269 | -0.123661 |
| -1.084749 | -0.348596 | -1.014136 | -2.567227 | -0.321326 | -1.220952 | -2.138711 | -0.639263 | -0.730829 |
| -1.475248 | -0.580076 | -0.324707 | -0.429929 | -0.536390 | -0.388645 | -0.647955 | -0.097395 | -0.006342 |
| -1.007351 | -0.183557 | -0.138604 | -0.868439 | -0.042773 | -0.086238 | -1.244818 | -0.038108 | -0.110201 |
| 1.0375759 | 0.0530610 | 0.6520032 | 0.7787587 | -0.081781 | 0.3348840 | 0.8195860 | -0.375684 | 0.8300964 |
| 1.1355616 | -0.764590 | -0.566525 | -0.426442 | -0.445705 | -0.468836 | 0.3302080 | -0.342044 | -0.506829 |
| 0.1861030 | -0.172927 | 0.1051534 | -0.039291 | -0.090189 | 0.1903152 | -0.191471 | 0.1984454 | -0.185187 |

| | | | | | | | | |
|---|---|---|---|---|---|---|---|---|
| -0.3546947 | -0.0791825 | 0.0505434 | 0.4835409 | -0.1817504 | 0.4319868 | 0.5380500 | 0.0413194 | -0.0349153 |
| -1.7416973 | -0.4542311 | -0.3704881 | -1.9743539 | -0.3855272 | -0.6217208 | -2.1242426 | -0.0040098 | -0.3408984 |
| 0.4613493 | -0.3774118 | -0.0147386 | -0.0252877 | -0.3662769 | -0.1638856 | -0.2071293 | -0.6725936 | -0.4804079 |
| 0.8643775 | -0.7383698 | 0.3544167 | 1.3785366 | -0.5746057 | 1.1300862 | 0.7565615 | 0.4258938 | 0.4306981 |
| -0.2055617 | -0.2755782 | -0.1383127 | -0.2825604 | -0.1540868 | 0.0745920 | -0.1852762 | -0.5627141 | -0.4072704 |
| 0.5841735 | -0.3194054 | -0.2814778 | 0.0030167 | -0.3525130 | -0.2230101 | 0.1542527 | -0.2011190 | -0.3105007 |
| -0.6190230 | -0.4582794 | -0.5538407 | -0.8198969 | -0.5706322 | -0.6887694 | -1.4786840 | -0.1877441 | -1.0349282 |
| 2.0770029 | -0.2155299 | 1.2067739 | 2.9947408 | 0.5713470 | 1.7398417 | 2.7669125 | -0.1936192 | 0.3016177 |
| 0.2775458 | -0.3888356 | 1.8388931 | 1.0681299 | 0.9795632 | 1.5912177 | 1.0106550 | -0.1633347 | 0.0992320 |
| 0.0440545 | -0.3854521 | -0.4893472 | 0.1167854 | -0.5562317 | -0.5948683 | -0.2112322 | -0.3192436 | -0.8292874 |
| -1.6473748 | -0.7057650 | -1.3082873 | -1.9297442 | -0.9140545 | -1.6069766 | -2.0306169 | -0.9201964 | -2.0220612 |
| 2.1129989 | 0.1377394 | 2.7511781 | 1.6626933 | 1.9727084 | 3.0822511 | 2.7672956 | -0.0817969 | 3.0992495 |
| 2.4198921 | 0.1811380 | 3.1813097 | 2.6026585 | 1.8038304 | 3.3094899 | 2.9623808 | 0.3901140 | 2.2166543 |
| -1.0911400 | -0.5908141 | -0.2646058 | -0.4063065 | -0.4258719 | -0.3991591 | -0.9097653 | -0.7522996 | -0.3991191 |
| -1.6330963 | -1.2094430 | -1.2802374 | -2.6154977 | -1.1952917 | -1.5985583 | -3.4951353 | -0.2840631 | -0.9140437 |
| -1.2591446 | -0.6488655 | -1.3750796 | -1.3452080 | -0.8851001 | -1.5628590 | -1.5960365 | -0.1436205 | -0.6445359 |
| -1.7839848 | -0.2743701 | -0.8060769 | -1.7344305 | -0.3275559 | -1.1786488 | -1.6419542 | 0.1255759 | -0.1907048 |
| -0.7325654 | -0.3009161 | -0.4514887 | -0.5353050 | -0.3552747 | -0.6939306 | -0.6932483 | -0.3548014 | -0.4314108 |
| -1.2484874 | -0.3449635 | -0.4812814 | -1.8283327 | -0.2961580 | -0.6801834 | -1.6492405 | -0.1181657 | -0.5439388 |
| -0.3367641 | 0.2337375 | -0.1437221 | -0.9794519 | 0.1338406 | -0.3272213 | -1.3170353 | 0.2366788 | 0.0243479 |
| -0.4851548 | -0.0062533 | 0.2249309 | -0.8591553 | 0.2226957 | -0.5483389 | -1.5618885 | 0.3326051 | 0.2549750 |
| -0.5519880 | -0.5427078 | -0.8593167 | -1.1244584 | -0.6075652 | -1.1380692 | -1.6742496 | -0.5781456 | -0.2982265 |
| 0.3942566 | 0.0850555 | -0.4764609 | -0.3403589 | 0.1959975 | -0.1879727 | -0.2540001 | -0.1978934 | -0.7678632 |
| 0.5239882 | 0.4065572 | 0.4559944 | 0.7429131 | 0.1874299 | 0.6380150 | 0.6340997 | 0.8861459 | 0.5349345 |
| -1.6832379 | -0.6405938 | -1.3246371 | -2.0188439 | -0.9246630 | -1.6259365 | -2.1838193 | -0.4532355 | -0.8096219 |
| -0.2164433 | -0.2270676 | -0.4610447 | -0.7949358 | -0.2256973 | -0.6906883 | -0.3304454 | -0.2938216 | -0.8692985 |
| -1.0655001 | -0.7744717 | -0.5658547 | -0.9387158 | -0.7643461 | -0.5281218 | -0.9798306 | -0.0971824 | -1.8650200 |
| 3.8746863 | -0.0241236 | 1.5703297 | 3.9764049 | 0.5425113 | 1.8548267 | 3.440187 | 0.4252806 | 2.1300934 |
| 1.5803552 | -0.6151498 | -0.1848969 | 1.8926850 | -0.6566959 | 0.2431792 | 2.5449160 | 0.3315749 | 0.4052561 |
| -1.0075548 | -0.4916497 | -0.9505261 | -2.0746291 | -0.4937866 | -1.2513163 | -2.5043797 | -0.4897825 | -0.6229240 |
| -0.2864480 | -0.1785567 | -0.7959980 | -1.3183066 | -0.6263365 | -1.0777452 | -1.0117571 | -0.4769227 | -0.3141239 |
| 0.0027903 | 0.0675975 | -0.3356319 | -0.3131402 | -0.0077653 | -0.6929542 | -0.3143582 | -0.2128355 | -0.1441748 |
| -1.4347392 | -0.6111765 | -0.9978303 | -1.9249735 | -0.8711273 | -1.4031916 | -1.8323176 | -0.2902748 | -0.9843873 |
| -1.2297424 | 0.0267929 | -0.5015749 | -1.0755384 | 0.0716825 | -0.7429007 | -1.0248053 | 0.1129122 | -0.1839948 |
| 1.7566915 | -0.1678156 | 0.8109026 | 1.8101467 | 0.0688905 | 1.0574549 | 1.4825522 | -0.4693850 | 1.0893167 |
| -0.5544896 | -0.2743724 | -0.5781047 | -0.8764378 | -0.5115901 | -0.3929945 | -0.9052266 | -0.0505568 | 0.1442806 |
| -0.2112218 | -0.5159098 | -0.9154050 | -0.9835149 | -0.7352552 | -0.8852451 | -1.2133040 | -0.2450195 | -0.5482192 |
| -0.7512732 | -0.0607245 | -0.5074794 | -1.6383584 | -0.1938688 | -1.0160429 | -1.5868799 | 0.1743012 | -0.2610953 |
| -0.5837702 | -0.0191221 | -0.3068338 | -0.8740933 | -0.2766459 | -1.3008958 | -0.8344789 | -0.0558055 | -0.2561979 |
| -1.1297946 | -0.5455424 | -0.8722483 | -1.4797784 | -0.6947483 | -0.8767421 | -1.9429208 | -0.3510864 | -1.6299365 |
| -1.4862567 | -0.5030243 | -0.7602540 | -0.8939980 | -0.5373168 | -0.8701824 | -1.0127037 | -0.3206736 | -0.5677219 |
| 0.1780901 | -0.7228715 | -2.2112103 | -2.0487297 | -1.4699256 | -2.6397206 | -1.9084815 | 0.4381594 | 0.0325792 |
| -0.4714979 | 0.0090034 | 2.5216160 | -0.0806996 | 1.5769615 | 2.8192734 | -0.4495848 | -0.6195197 | 2.4162091 |
| -0.2068606 | -0.2386943 | -0.0498867 | -0.2269126 | -0.1472657 | -0.1285918 | -0.2340915 | -0.0226447 | -0.1150762 |
| -0.5251167 | -0.0563317 | -0.2126593 | -0.8782321 | -0.0268354 | -0.0512673 | -0.8964165 | -0.0445362 | -0.3768372 |
| -1.2627097 | -1.1177893 | -2.1342825 | -2.3858249 | -1.6258756 | -1.8372318 | -2.2308302 | -0.9864863 | -1.3917949 |
| -0.1714333 | -0.0739361 | 5.5897656 | 4.5233647 | 1.7696473 | 6.6801356 | 7.0796428 | -0.1424687 | 5.8660827 |
| 1.1671384 | -0.2280002 | 1.8239150 | 1.1270757 | 0.7091827 | 2.2531082 | 2.4287000 | 0.1591328 | 1.3041159 |
| -1.8102234 | -0.6609690 | -1.2608069 | -3.2344308 | -1.0821588 | -1.5996612 | -3.6714490 | 0.1328715 | -0.1447575 |
| -0.4878513 | -0.3939565 | -0.1702106 | 0.3373746 | -0.3301553 | 0.2012229 | 0.0940048 | -0.5161823 | -0.1725855 |

| | | | | | | | | |
|---|---|---|---|---|---|---|---|---|
| -0.7670683 | -0.5398573 | -1.7038675 | -2.5469052 | -0.7952781 | -1.5023449 | -1.9519957 | -0.1739677 | -0.3049726 |
| 0.4964275 | -0.2114743 | -0.1992096 | 0.1468519 | -0.3434057 | -0.2245550 | 0.2997289 | -0.0633601 | -0.2161953 |
| -0.4807317 | 0.2550892 | -0.8803645 | -0.0976181 | -0.1035549 | -0.9502307 | -0.1329982 | -0.0601938 | -0.4203138 |
| -0.1971937 | -0.1758699 | -0.0923369 | -0.0672964 | -0.1424770 | -0.1529126 | 0.1777558 | 0.0510562 | -0.0369506 |
| -1.0245402 | -0.3103083 | -0.9196804 | -2.0921771 | -0.4628790 | -1.2250804 | -1.9408168 | -0.1732870 | -1.3767227 |
| -1.3799247 | -0.8251073 | -0.2015181 | 1.3961617 | -0.8727794 | 0.4118188 | 1.7904232 | -0.5776105 | -0.4625412 |
| -0.5677587 | -0.3607472 | -0.3614780 | 0.1619520 | -0.0888715 | -0.2071448 | 0.2253429 | -0.2800609 | -0.1432198 |
| 0.1295974 | -0.4774429 | -0.2207262 | -0.4535815 | -0.2934107 | -0.2497717 | 0.0884453 | 0.1001026 | -0.0138353 |
| 0.0092372 | -0.4779091 | -0.0897214 | -0.0657782 | -0.2067001 | -0.0100669 | -0.2782895 | -0.2299469 | -0.3227765 |
| 0.2857883 | 0.2458345 | 0.6194564 | 1.1712375 | 0.1960682 | 0.7238848 | 0.8566621 | -0.1763060 | 0.3819415 |
| -0.6008635 | 0.0566854 | -0.4332253 | -0.3788697 | 0.0583151 | -0.2743367 | -0.1908661 | 0.0771259 | -0.2293992 |
| -0.0556488 | -0.4064668 | 0.0768357 | 0.1294468 | -0.3937978 | -0.2415933 | -0.6350994 | 0.5121277 | -1.0662005 |
| -0.6393030 | -0.3700078 | -0.2181775 | -1.1345671 | -0.3826484 | -0.3203418 | -1.8408376 | -0.1338375 | -0.4313587 |
| -0.7587602 | -0.4279512 | -0.4096054 | -1.0457301 | -0.4807427 | -0.4110429 | -1.2380156 | -0.0426337 | -0.8638068 |
| -0.3399225 | -0.3608196 | -0.5192145 | -0.0492260 | -0.4250244 | -1.0183046 | -0.3117010 | -0.1006742 | -0.3866583 |
| -0.2583848 | -0.2790650 | -0.3526759 | -0.3204948 | -0.2057109 | 0.0581755 | -0.2199592 | -0.0949412 | 0.0840015 |
| -1.0521172 | -0.3448958 | -1.1937214 | -0.8158109 | -0.6741830 | -1.6668547 | -0.5921659 | -0.3100044 | -0.8329137 |
| -0.0741259 | -0.3656034 | 0.0654801 | 0.0272196 | -0.1477315 | -0.3762958 | 0.0215854 | -0.1178272 | -0.0892876 |
| -1.4822523 | -0.0190479 | -0.4567408 | 0.1244828 | -0.9443377 | -0.8847252 | -0.3999909 | 0.2078927 | -0.1390252 |
| 1.5537626 | -0.2785908 | 0.2125510 | 1.1454898 | -0.3640631 | 0.0832508 | 1.4563321 | -0.2308903 | -0.1995396 |
| -1.5114428 | -0.3068587 | -0.5720575 | -1.1802410 | -0.3327541 | -0.7599774 | -1.4264846 | -0.6286038 | -0.4538181 |
| -0.1140764 | 0.0068121 | -0.2728421 | 0.6716551 | 0.0424021 | -0.3947313 | 0.6352346 | -0.2878280 | -0.4421798 |
| -0.2126680 | -0.0339494 | -0.1045252 | -0.2123869 | -0.3573537 | -0.0288427 | -0.2978331 | -0.2393518 | -0.1007129 |
| -1.3342139 | -0.1681033 | -0.6236528 | -1.9776980 | -0.3431487 | -0.7136420 | -2.0498833 | -0.3045999 | -0.7925719 |
| -0.4199022 | -0.5422427 | -0.5589613 | -0.6180679 | -0.7633615 | -0.7461319 | -0.7600831 | -0.5992329 | -0.3244354 |
| -0.4682109 | -0.1278405 | -0.4460813 | -0.4094584 | -0.1207626 | -0.8252507 | -0.1705202 | 0.1077228 | -0.2851009 |
| -0.5911982 | -0.0932080 | -0.2296027 | -0.2733993 | -0.1936664 | -0.3217078 | -0.1447555 | 0.3019094 | 0.0950966 |
| 0.1097533 | -0.4018333 | -0.2582078 | 0.3802742 | -0.1568169 | -0.4027905 | 0.4929168 | -0.4401438 | -0.1898024 |
| -1.2363940 | -0.4407131 | -0.7394660 | 0.6051608 | -0.7579850 | -0.6860509 | 0.4937383 | 0.5350299 | 0.3817948 |
| -0.6584839 | -0.1586788 | -0.2874093 | -0.9083693 | -0.1332879 | -0.2457953 | -0.7146892 | -0.1759104 | -0.0098434 |
| -1.2723181 | -0.3312851 | -1.4144653 | -1.2031092 | -0.4848797 | -1.1276865 | -1.3300370 | -0.1030265 | -0.6752663 |
| -2.4223865 | -0.2653386 | -0.5108563 | -1.1185945 | -0.6768795 | -0.8690122 | -1.4672515 | -0.0841229 | -0.7500621 |
| -0.5796479 | -0.7490257 | -0.7047746 | -0.3391676 | -0.7613605 | -0.1382962 | -0.3654217 | -0.3449611 | -0.3840786 |
| -0.4200498 | 0.2561643 | 0.3246879 | -0.5091401 | 0.1798323 | 0.0829636 | -0.5284218 | 0.4198691 | 0.2322388 |
| -0.8472213 | -0.2057636 | -0.3830088 | -1.3488347 | -0.3530243 | -0.5121466 | -1.3177367 | -0.2594211 | -0.1144190 |
| -0.5617399 | -0.3730611 | -0.8375638 | -0.9742250 | -0.5034524 | -0.9990005 | -0.6724093 | 0.0289093 | -0.1753007 |
| -0.4188281 | -0.0994526 | -0.2126408 | -0.4369474 | -0.0389277 | -0.4312410 | -0.7625978 | 0.0015533 | -0.1750989 |
| -0.4214072 | -0.1600734 | -0.2887680 | -0.8848153 | -0.1227166 | -0.3747065 | -0.6180963 | -0.1517600 | -0.2040939 |
| -0.6835189 | -0.4618226 | -0.7108686 | -0.5364961 | -0.9056753 | -0.7194117 | -0.5907573 | -0.3512017 | -0.6652786 |
| -0.6882660 | -0.8467766 | -1.1146653 | -1.7522103 | -1.3400957 | -0.7945165 | -1.8039415 | -0.0515494 | -0.6459663 |
| -0.2150791 | 0.0322803 | 0.9135605 | -0.2341222 | 0.7298923 | 0.7050698 | -0.5756376 | -0.2932331 | -1.9067886 |
| 0.1631044 | -0.1118135 | 0.0959969 | 0.0247557 | 0.1662107 | 0.1495664 | -0.1134341 | -0.1617794 | 0.0013802 |
| -0.3121951 | 0.0235220 | 0.0687022 | -0.6401267 | -0.0689654 | -0.0738428 | -0.6101469 | 0.1612831 | -0.0659594 |
| -1.5874549 | -0.0903796 | -0.2529331 | -0.9382080 | -0.3665763 | 0.1140230 | -1.0378987 | 0.3873507 | 0.1524866 |
| 0.0212685 | -0.4087955 | -0.5249431 | -0.6075479 | -0.4854258 | -0.3932288 | -0.5830672 | -0.3640095 | -0.1884785 |
| 0.4003389 | -0.3272419 | 0.0546977 | -0.0833769 | -0.2798842 | 0.0519534 | -0.3698697 | -0.1374330 | 0.0918993 |
| -1.1040401 | -0.4708726 | -0.6590712 | -1.2584604 | -0.5218047 | -1.0989482 | -1.1195501 | -0.6194203 | -0.7951308 |
| 0.4347489 | -0.1300928 | -0.3844051 | 1.0971235 | -0.2927444 | 0.0983137 | 1.7524373 | -0.1874718 | -0.1005466 |
| -0.7156380 | -0.4147211 | -1.1113029 | -2.0282745 | -0.5634030 | -1.1860664 | -1.9370186 | -0.4772448 | -0.6346261 |
| -1.5525554 | -0.5804118 | -0.8871090 | -1.8326589 | -0.6946815 | -1.2784185 | -1.7825880 | -0.5579180 | -0.6856123 |

| | | | | | | | | |
|---|---|---|---|---|---|---|---|---|
| -0.042725 | 0.1702605 | 0.1038235 | 0.0405728 | 0.2086069 | 0.3235492 | 0.5375934 | 0.4899379 | 0.0967252 |
| -1.385119 | -0.681218 | -1.297827 | -2.401306 | -0.899311 | -1.306845 | -2.414110 | -0.692271 | -1.277893 |
| -0.372880 | -0.724700 | -0.570690 | -0.731497 | -0.667453 | -0.616425 | -0.447053 | -0.441284 | -0.466644 |
| -0.294846 | -0.343972 | 0.1629582 | -0.444150 | -0.371489 | 0.2085740 | 0.0515573 | -0.357737 | -0.125587 |
| -0.612260 | -0.308268 | -1.022505 | -1.199733 | -0.566608 | -0.842289 | -0.865022 | -0.061260 | -0.583375 |
| 2.3907638 | 0.0801094 | 1.9501598 | 1.2616450 | 1.0981617 | 2.1392678 | 0.7923091 | -0.364094 | 2.2645073 |
| -0.657180 | -0.338424 | -0.434800 | -0.785108 | -0.400163 | -0.449179 | -0.574883 | -0.307579 | -0.426468 |
| -0.525626 | -0.488610 | -0.775145 | -0.730318 | -1.211885 | -1.198474 | -0.930078 | -0.679357 | -1.024459 |
| -1.278506 | -0.397066 | -1.086203 | -1.610458 | -0.651130 | -1.418509 | -1.954506 | -0.331618 | -1.114620 |
| -0.511981 | -0.605113 | -0.415403 | 0.0410595 | -0.590379 | -0.138062 | 0.3401735 | -0.354168 | -0.734345 |
| 0.1296793 | 0.0480944 | 0.1365911 | 0.4303508 | 0.0155731 | 0.0889051 | 0.6259535 | 0.0508456 | -0.007979 |
| -1.324851 | -0.594165 | -0.677203 | -1.243358 | -0.582171 | -0.458863 | -0.963978 | -1.285331 | -1.048836 |
| -1.136538 | -0.465125 | -0.590027 | -0.796091 | -0.839349 | -0.779519 | -1.119997 | 0.3325448 | -0.302323 |
| -1.648933 | -0.462980 | -0.583878 | -0.597891 | -0.469659 | -0.627852 | -0.704195 | -0.418225 | -0.479658 |
| -0.386322 | -0.330909 | -0.414411 | -0.509153 | -0.507359 | -0.560559 | -0.444672 | -0.022521 | -0.345686 |
| -1.435285 | 0.0658539 | -0.342867 | -1.611000 | 0.0226433 | -0.453553 | -1.149712 | 1.0087406 | 0.8760115 |
| -0.555671 | -0.696797 | -0.664806 | -0.990803 | -0.627712 | -0.702535 | -1.448538 | -1.070224 | -0.374736 |
| -2.049055 | -0.296553 | -1.198281 | -1.941422 | -0.646992 | -1.975136 | -2.510743 | -0.475988 | -1.281345 |
| -0.504347 | -0.406862 | -0.991709 | -0.531495 | -0.842207 | -1.047917 | -0.388423 | -0.373931 | -0.430186 |
| -0.418457 | -0.252448 | -0.854210 | -0.984747 | -0.456336 | -0.793118 | -0.550862 | -0.046723 | -0.370574 |
| 0.4185162 | -0.423608 | 1.0599008 | 0.7095502 | -0.369293 | 1.9168908 | 1.5629402 | -0.421931 | 0.2791694 |
| 0.1117300 | 0.0303649 | 0.2727638 | 0.3890173 | 0.0925856 | 0.2407691 | 0.5484447 | -0.120626 | -0.160704 |
| -1.803716 | -0.493194 | -1.249329 | -1.951530 | -0.672058 | -1.309430 | -2.535967 | -0.695612 | -0.956281 |
| -0.536199 | -0.253069 | -0.560625 | -0.844268 | -0.534876 | -0.553884 | -1.324189 | -0.360118 | -0.084762 |
| -1.154862 | -0.309867 | -0.911871 | -1.757342 | -0.496028 | -1.063229 | -1.759266 | -0.131481 | -0.338065 |
| -0.662362 | -0.288247 | -0.709394 | -0.232601 | -0.557578 | -1.286718 | -1.079028 | -0.345621 | -0.425132 |
| -1.393971 | -0.639430 | -1.030007 | -0.112092 | -0.883047 | -0.926469 | -0.579889 | -0.281306 | -0.372598 |
| -0.356570 | -0.161568 | -0.619711 | -0.085454 | -0.416223 | -0.472380 | 0.6091867 | -0.029162 | -0.426386 |
| -0.766874 | -0.565592 | -1.021762 | -0.712529 | -0.823663 | -1.213460 | -0.942776 | -0.434923 | -1.150755 |
| 0.6336150 | 0.1640047 | 0.4786728 | 0.8756132 | -0.010574 | 0.3358165 | 1.0294228 | 0.1776753 | 0.4429924 |
| 2.0311126 | 0.0202113 | 1.6842727 | 3.5737483 | 0.5224094 | 2.5638597 | 3.9090016 | 0.1618024 | 0.9237124 |
| -1.753465 | -0.322795 | -0.739102 | -0.791554 | -0.737090 | -0.550259 | 0.4127255 | -0.366184 | -1.031231 |
| -0.249028 | 0.1473846 | 0.1321076 | -0.041000 | 0.1081980 | -0.004018 | -0.229755 | 0.0616205 | 0.0719086 |
| 0.5924516 | 0.1175753 | 0.4458537 | 0.5162424 | 0.2289053 | 0.4302329 | 0.5987196 | 0.1488391 | 0.3269209 |
| 0.8229187 | 0.3002381 | 0.9405538 | 1.1445706 | 0.3238022 | 0.6358369 | 0.8975951 | 0.1463299 | 0.9281184 |
| -0.838609 | -0.126526 | -0.951577 | -1.278703 | -0.281602 | -1.065103 | -1.316111 | -0.303536 | -0.948198 |
| 0.4115376 | 0.4224662 | 0.4280715 | 1.4641140 | 0.7672644 | 0.6853840 | 1.8988886 | 0.8779318 | 1.0012380 |
| -0.196051 | -0.551608 | -0.013362 | 0.0215177 | -0.353067 | 0.3503121 | 0.6653988 | -0.017254 | -0.029348 |
| -0.274198 | 0.0952554 | -0.135758 | 0.7595456 | 0.0467645 | 0.0152260 | 1.2575304 | -0.191682 | -0.435985 |
| -0.379062 | -0.688550 | -0.671745 | -0.890667 | -0.724723 | -0.520289 | -0.791375 | -0.034446 | -0.032842 |
| -0.696047 | -0.811262 | -1.629363 | -2.470823 | -1.073799 | -1.900725 | -2.295992 | 0.6289969 | -0.530375 |
| 0.5271385 | -0.318283 | -0.027502 | 0.7018335 | -0.263073 | 0.0300233 | 0.6398833 | -0.122441 | -0.193406 |
| 1.0353074 | 0.1463024 | 0.7295864 | 0.9417370 | 0.5331471 | 0.9087021 | 1.4774047 | 0.2939864 | 0.8574094 |
| 0.5193384 | 0.0522623 | 0.4297724 | 0.6822623 | 0.0511058 | 0.4373582 | 0.7006755 | 0.1968376 | -0.023538 |
| -0.751332 | -0.157858 | -0.383381 | -0.479722 | -0.172176 | -0.453729 | -0.256317 | -0.364668 | -0.301707 |
| -0.177666 | -0.047760 | -0.381313 | 0.1944371 | -0.137902 | -0.175856 | 0.3007323 | -0.114198 | -0.317542 |
| -1.045128 | -0.468540 | -1.130099 | -1.614655 | -0.771775 | -1.400641 | -1.665195 | -0.524861 | -0.628187 |
| -1.175623 | -0.917251 | -1.026517 | -2.862060 | -0.993395 | -1.273056 | -2.330313 | -0.264218 | -1.321443 |
| -1.432152 | -0.244497 | -0.029261 | -0.487698 | -0.236074 | -0.235966 | 0.1928166 | 0.1072057 | 0.0644145 |
| -0.153615 | -0.201819 | -0.119973 | 0.1912474 | -0.171840 | -0.295596 | 0.2930000 | -0.196079 | -0.324874 |

| | | | | | | | | |
|---|---|---|---|---|---|---|---|---|
| -0.0117464 | 0.2633803 | 0.2315740 | 1.2130764 | -0.0771687 | 1.5218184 | 1.1693954 | 0.4204692 | 0.2046008 |
| -1.3581281 | 0.0767200 | -0.0192260 | -0.6008293 | 0.0101231 | 0.0840969 | -0.4443083 | -0.3887180 | -0.8892659 |
| -0.5399287 | -0.6874321 | -0.1258399 | -0.1519017 | -0.6857643 | 0.1430926 | -0.1134120 | -0.3839299 | -0.2997869 |
| -0.9208306 | -0.6492573 | -1.9242187 | -1.6970481 | -0.9835559 | -1.7954714 | -1.2743888 | -0.5818370 | -0.7443303 |
| -1.1544757 | -0.2377396 | -1.0670639 | -0.6502780 | -0.3976501 | -0.5519788 | -0.7501345 | -0.2092970 | -0.5179046 |
| -0.4402557 | -0.2404771 | -0.0413623 | -0.6184467 | -0.2556050 | -0.0472270 | -0.1052618 | -0.0469847 | 0.3681790 |
| 2.7247425 | -0.1225604 | 1.1583426 | 1.7279291 | 0.0653669 | 1.1233756 | 0.8607385 | -0.1196585 | 1.5416613 |
| -0.1183297 | -0.1713274 | -0.5058103 | 0.0951740 | -0.2316498 | -0.1235346 | -0.2040035 | -0.1237107 | -0.0830147 |
| -0.5267791 | 0.0920192 | -0.1798124 | -0.7308567 | 0.1290390 | -0.3774343 | -1.6639230 | 0.1390643 | 0.0870803 |
| 0.1762019 | -0.5947211 | -1.0724834 | -0.0811363 | -0.5421779 | -1.2687865 | 0.0116535 | -1.1681357 | -1.0470447 |
| 0.3417104 | -0.5047873 | 0.2047977 | -0.0049177 | -0.1706085 | -0.1504261 | -0.5895669 | -0.4306918 | -2.4426690 |
| -1.1524664 | -0.6339961 | -0.5414839 | -0.3118343 | -0.7053552 | -0.1582889 | -0.2540618 | -0.1728561 | -0.6912005 |
| -0.0353855 | 0.0047951 | -0.0458698 | -1.7894602 | 0.0779455 | -0.3225382 | -2.1089431 | -0.0657460 | 0.3205328 |
| -1.0166945 | 0.2287000 | -0.1678020 | -0.2637337 | 0.1214902 | -0.4698799 | -0.3189741 | 0.0923309 | 0.1686246 |
| -1.5593557 | -0.1511322 | -0.5663016 | -1.2293789 | -0.1779047 | -1.0073814 | -1.3291879 | -0.0978504 | -0.1881880 |
| -1.6099526 | -0.3682191 | -0.6437729 | -1.5936295 | -0.5543270 | -0.7147767 | -1.7772781 | -0.0715160 | -0.2105470 |
| -0.5274580 | -0.2221940 | -0.4288764 | -1.0400347 | -0.1982657 | -0.5184988 | -0.7437321 | 0.1766574 | 0.0731266 |
| -0.3403995 | -0.8141971 | -0.8847597 | -1.2056245 | -0.5962762 | -0.5024915 | -1.4317507 | -0.1042544 | -0.3505433 |
| -1.7843223 | -0.0565159 | -0.7654115 | -1.5816394 | -0.1751373 | -1.0747707 | -1.0131256 | -0.2235890 | -0.9826988 |
| -0.3277045 | 0.3074873 | 0.2880024 | 0.8862467 | 0.3449200 | 0.5933335 | 0.6900274 | 0.5489515 | 0.1249748 |
| -1.3210536 | -0.1239746 | -0.3566544 | -0.4683822 | -0.1690341 | -0.1155809 | -0.7276975 | -0.8497332 | -0.8039806 |
| -0.3356955 | -0.3988358 | -0.6992827 | -0.7438854 | -0.5609154 | -0.6272528 | -0.7455156 | -0.3247070 | -0.4083018 |
| -1.5984751 | -0.8374123 | -1.8027919 | -3.8671435 | -0.9157672 | -3.3410513 | -4.2895667 | -1.1305598 | -1.6428136 |
| 0.0342687 | -0.2149249 | -0.3205113 | -0.1062471 | -0.2113076 | -0.3605095 | 0.2669090 | 0.0229476 | -0.1690504 |
| -0.9232826 | 0.0333112 | -0.3264395 | -0.4186923 | 0.1798271 | -0.2679383 | -0.6029076 | 0.0749419 | -0.3838865 |
| -0.4642974 | -0.5025554 | -0.5317164 | -1.0005273 | -0.6255128 | -0.6543439 | -1.0203283 | -0.3276648 | -0.3930744 |
| -0.5746551 | -0.1789182 | -0.3838611 | -1.0636753 | -0.3990474 | -0.5514032 | -0.5567946 | -0.1722297 | -0.2954257 |
| -0.6041952 | -0.2155996 | -0.4890288 | -0.0036075 | -0.3337269 | -0.5330442 | 0.4556899 | -0.5289505 | -0.4313880 |
| 0.3940765 | -0.1096792 | 0.1826986 | 1.1812238 | -0.0401898 | 0.7641567 | 1.6680882 | 0.2262770 | 0.0760277 |
| 1.7575964 | 0.0769248 | 0.1771273 | 1.7262873 | 0.1177218 | 0.5490998 | 1.9541270 | -0.1508452 | 0.2353930 |
| -1.8746304 | -0.4754933 | -0.4569149 | -0.9355552 | -0.5541725 | -0.6207765 | -1.1809066 | -0.4688350 | -0.3811803 |
| -0.5007689 | -0.1355456 | -0.4482741 | -0.2743210 | -0.2150303 | -0.5376412 | -0.3049062 | -0.1093661 | -0.2021690 |
| 1.4500732 | -0.7474709 | -1.1065730 | 0.3948415 | -0.7228212 | -0.6413901 | 0.3307765 | -0.0172916 | -0.0382429 |
| -1.1079835 | -0.1327056 | -0.1541180 | -0.9760336 | -0.0522373 | -0.6076638 | -1.0328264 | -0.5179934 | -0.3986953 |
| -0.5007578 | 0.1631310 | 0.0681433 | -0.5088884 | -0.2096064 | 0.1198273 | -1.0970486 | -0.0725716 | -0.0955255 |
| -1.2349470 | -0.3794650 | -0.6851173 | -0.9710716 | -0.5303772 | -0.8273356 | -1.3073924 | -0.7150496 | -0.7200630 |
| -0.9266154 | -0.8728081 | -1.6238009 | -1.8785137 | -1.1064242 | -1.5690227 | -2.2562495 | -0.8427501 | -1.5776996 |
| -0.2603841 | -0.6454569 | -0.3981100 | -0.5824680 | -0.5487438 | -0.6370185 | -0.7424025 | -0.1016522 | 0.0429106 |
| 0.8311955 | -0.4318065 | 0.8767733 | 0.6629983 | -0.3240592 | 1.0713218 | 0.1752551 | -0.2356173 | -0.0074555 |
| 0.1380696 | -0.2108098 | -0.3185868 | -0.2099554 | -0.1968705 | -0.5164254 | -0.4558377 | -0.2233535 | -0.1429040 |
| 0.2448319 | 0.0893109 | -0.0031087 | 1.3842525 | 0.0230716 | 0.2851678 | 2.0998849 | 0.1319787 | 0.1264844 |
| -1.2699913 | -0.1773867 | -0.6823087 | -0.7737382 | -0.2317158 | -0.8772749 | -1.0748755 | -0.6101272 | -0.8361736 |
| -0.5899242 | -0.6136640 | -0.6756366 | -0.5934091 | -0.9917209 | -0.7255792 | -0.7190143 | -0.5043052 | -0.9421204 |
| 0.5978619 | -0.1891627 | -0.9216506 | -0.1904952 | -0.0751905 | -0.2395153 | 0.0211263 | 0.4525319 | -0.1395796 |
| -0.1892326 | -0.3975426 | -0.2051960 | 0.1992054 | -0.3003781 | -0.2909039 | 0.4455533 | -0.3450613 | -0.2595853 |
| -0.9194992 | -0.5161783 | -0.8655111 | -0.7218163 | -0.6050991 | -0.7199221 | -0.9954813 | -0.2139837 | -0.6054210 |
| -0.4898744 | -0.0065132 | -0.6982091 | -0.4963864 | -0.1252281 | -0.5973900 | 0.0192674 | -0.2995597 | -0.1321971 |
| -1.4905840 | -0.6367145 | -0.4305682 | 0.0587136 | -1.1115904 | -0.0926193 | -0.3220116 | -0.1270479 | -1.3611905 |
| 0.5414811 | -0.3851528 | -0.5024227 | 0.9676282 | -0.2666950 | 0.3356418 | 1.7136291 | -1.2034764 | -1.0430049 |
| -1.0873461 | -0.6708555 | -1.0537636 | -1.6971578 | -1.1031959 | -1.3088319 | -1.9677836 | -0.7780699 | -0.9562717 |

| | | | | | | | | |
|---|---|---|---|---|---|---|---|---|
| 0.2042363 | 0.1630050 | 0.7471322 | 2.9520514 | 0.5134475 | 1.2715469 | 3.4648166 | -0.3596469 | 0.2465235 |
| -0.3618334 | -0.1248089 | -0.1328633 | 0.3671120 | -0.1390445 | -0.0551176 | 0.9231967 | -0.1802027 | -0.3523777 |
| 0.2393430 | -0.1356501 | -0.1249717 | 0.1017762 | -0.2060903 | -0.2400724 | -0.2707224 | -0.4920744 | -0.3605119 |

| PG_417 | PG_418 | PG_419 | PG_420 | PG_421 | PG_422 | PG_423 | PG_424 | PG_425 |
|---|---|---|---|---|---|---|---|---|
| -2.8440643 | -0.4166083 | -0.5717627 | -3.7232719 | -0.7052551 | -0.9484683 | -2.0613912 | -0.3426840 | -0.8494979 |
| -1.6805801 | -0.6957470 | -1.5831783 | -4.0778205 | -1.2119535 | -1.1804698 | -1.8631941 | -0.9818905 | -0.8358180 |
| -1.1883319 | -0.1162406 | -0.7774414 | -1.9509451 | -0.1893686 | -0.5443063 | -0.8765737 | 0.2213383 | -0.5174046 |
| -1.1208512 | 0.2643347 | 0.0283790 | -3.8372029 | -2.2136799 | -1.4928776 | -1.2778022 | -1.0026312 | -1.1941695 |
| -1.5690512 | -0.0581362 | -0.4069122 | -2.2308921 | -1.4758955 | -0.9425178 | -2.0614158 | -0.8568862 | -0.6998170 |
| -1.4717376 | -0.3913728 | -0.4303022 | -2.7382676 | -0.8796719 | -0.9353342 | -3.1716619 | -0.8626400 | -1.1323278 |
| -1.1126914 | 0.0598105 | -0.5795912 | -1.5478575 | -1.0250267 | -2.2415216 | -1.6953218 | -1.0466941 | -2.6166532 |
| -0.5173287 | -0.1718685 | -0.0931148 | -2.2097836 | -0.6575210 | -0.4622877 | -1.5718746 | -0.4267817 | -0.5146288 |
| 0.6163930 | -0.3153422 | -0.2180166 | -1.1773533 | -0.1520577 | 0.1825083 | 0.1914095 | -0.2676069 | 0.6101245 |
| -1.6417915 | -0.1640623 | -1.3939110 | -2.0553808 | -0.4578033 | -0.5650358 | -0.6961641 | -0.6060454 | -0.6244928 |
| -0.6522409 | 0.0555961 | -0.0864110 | -2.3815994 | -0.9726228 | -0.7205017 | -1.4834996 | -0.4103498 | -0.8331642 |
| -0.4318245 | -0.3177096 | -0.3648240 | -1.6911081 | -1.6462388 | -0.9415457 | -1.5044810 | -0.8422984 | -0.9341737 |
| -2.7709764 | -0.3462524 | -0.8325355 | -4.2853714 | -0.9971597 | -1.2624683 | -4.2413137 | -0.7287626 | -1.5058369 |
| 2.8989006 | 0.2875076 | 0.9393687 | 2.8217167 | -0.9259341 | 2.1378845 | 3.6808915 | -0.2276021 | 3.6316478 |
| -0.6475966 | -0.0051126 | -0.1385796 | -3.2527530 | -2.6119872 | -1.4630201 | -2.3763617 | -1.0910536 | -1.1757059 |
| 1.8870636 | 0.0790330 | 2.7818977 | 1.9414241 | -0.3662060 | 2.2295720 | 2.9884081 | 0.0863722 | 2.6335955 |
| -1.7391974 | -0.8083054 | -1.2468816 | -1.7446564 | -0.8083486 | -1.8839751 | -2.4422077 | -0.6282715 | -1.8843877 |
| -1.5366262 | 0.0427383 | -1.3955644 | -2.2356804 | -0.0399219 | 0.0188457 | -0.3511236 | 0.1961908 | 0.0353142 |
| -0.3066502 | -0.3557566 | -0.2060807 | 0.5129930 | 0.0700345 | -0.1493187 | -0.0587934 | -0.0855864 | -0.5162055 |
| -2.0702850 | -0.5118175 | -1.7078848 | -2.8717505 | -0.5445350 | -0.7662916 | -0.7108682 | -0.5584885 | -0.5453143 |
| 0.4065564 | -0.3310755 | 0.0347691 | -1.7090869 | -1.3140529 | -0.4555956 | -0.9838323 | -0.9233065 | -0.3558440 |
| -0.4916625 | -0.4204083 | -0.5537737 | -0.5588647 | -0.3691238 | -0.6721875 | -0.3849391 | -0.4319834 | -0.2693171 |
| 2.4529935 | -0.1684211 | 0.9569468 | 1.9183200 | 0.4587701 | 1.4635016 | 2.4130661 | 0.4378595 | 1.3499404 |
| -1.2062498 | -1.4078595 | -1.1852147 | 0.3227608 | -1.1223983 | -1.3307980 | -1.5576099 | -1.1117205 | -1.6850466 |
| -1.7236911 | -0.2889959 | -1.2040761 | -2.5708736 | -0.9830687 | -1.4015930 | -2.0643026 | -1.1864725 | -1.6330182 |
| 1.4797927 | -0.2528575 | 0.6020585 | 1.2518059 | 0.2298876 | 1.1577327 | 1.5052780 | 0.2690513 | 0.8432887 |
| -1.1870663 | 0.3683378 | -0.0119390 | -2.9644886 | -2.4917212 | -1.7781085 | -2.3791096 | -1.5798863 | -1.3049382 |
| -0.9891187 | -0.3786215 | -0.7048854 | -2.6034566 | -1.1557567 | -1.1772992 | -2.0668250 | -0.9359806 | -1.1861161 |
| -1.4486308 | -0.0359313 | -0.2142027 | -2.5890948 | 0.0524417 | -0.5648215 | -1.4007552 | 0.1408872 | -1.0149444 |
| -0.3464878 | 0.1098375 | 0.0530748 | -1.9581052 | -1.5404327 | -1.5173328 | -1.0846779 | -1.1728513 | -1.2798906 |
| -0.4946796 | -0.6094565 | -0.7728071 | 0.3422954 | -0.8206475 | -0.5885058 | -0.8248505 | -0.7248387 | -0.6381985 |
| -0.5677519 | 0.2613831 | 0.4536031 | 0.0439057 | 0.2048543 | -0.0676068 | 0.7397407 | -0.0623163 | 0.1850632 |
| -1.7986776 | -0.2564288 | 0.4213751 | -2.5180700 | -1.1830338 | -0.6281811 | -1.4258847 | -0.7782553 | 0.2143854 |
| -3.3927562 | -0.1339227 | -1.0912017 | -2.6914076 | -0.5648567 | -0.6426801 | -3.2085747 | -0.5661029 | -0.7689672 |
| -0.1165742 | -0.2458167 | -0.2147233 | 0.1318365 | -0.0802059 | -0.1121226 | -0.3156277 | -0.1073687 | 0.0061262 |
| 0.4337530 | -0.9509321 | -0.7425731 | -0.8708700 | -1.0675339 | -1.0324728 | -0.2664701 | -0.8799989 | -1.4546308 |
| -1.9270376 | -0.2349481 | -0.9663822 | -2.2546926 | -0.7373312 | -0.9953663 | -1.9837187 | -0.9601723 | -1.0202733 |
| -0.0918562 | 0.0450148 | 0.6459008 | 0.4157397 | 0.7527856 | 3.5854612 | 1.4006214 | 0.9997963 | 4.3120466 |
| -1.4969306 | -0.1043931 | -0.1798959 | -2.6789584 | -0.9501551 | -0.2544907 | -1.2991897 | -0.1470678 | -0.1023569 |
| 1.0607921 | -0.3441280 | -0.3372398 | -0.4751673 | -0.7736965 | -1.3244705 | -0.8214475 | -1.3452503 | -0.7642310 |
| 1.2027313 | -0.3385437 | -0.2782557 | 0.6441727 | 0.4770721 | 0.6601540 | 0.6872800 | -0.0130534 | 0.5715547 |
| -1.8977609 | -0.6095150 | -1.0052246 | -1.7266151 | -0.5142232 | -0.9444584 | -2.1698092 | -0.7249890 | -1.2001990 |
| -1.0315961 | 0.0691758 | 0.1290997 | -1.6151995 | -0.7695113 | -0.5661493 | -1.0226681 | -0.3133987 | -0.5284121 |
| -1.3688870 | 0.1284144 | -0.0699195 | -2.5029248 | -2.4066639 | -1.5758424 | -1.8959088 | -1.904696 | -1.4297574 |
| 2.2711122 | -0.3441729 | 0.5598650 | 1.6808157 | 0.2250871 | 1.0635125 | 2.0217870 | 0.1412949 | 0.5106132 |
| 0.5263582 | -0.2183140 | 0.4084981 | 1.2814535 | 0.1120646 | -0.2568904 | 0.8794883 | -0.4008694 | -0.3764286 |
| 0.1105777 | 0.0624891 | -0.4006924 | -0.1595435 | 0.2288595 | -0.0887495 | -0.1520151 | -0.5766683 | -0.1872154 |

| | | | | | | | | |
|---|---|---|---|---|---|---|---|---|
| -0.5592665 | 0.0617086 | -0.0284047 | -2.3839007 | -2.0597659 | -1.1356857 | -0.7347707 | -1.1244806 | -0.5152763 |
| -1.9189697 | -0.2557221 | -0.3212324 | -2.2133627 | -0.5311158 | -0.8984001 | -3.0948690 | -0.3869384 | -0.9384044 |
| -0.6662313 | -0.5768647 | -0.5754166 | -1.6189157 | -1.3388187 | -0.4326921 | -0.3477279 | -0.5488789 | -0.2701465 |
| 0.5810294 | 0.0218363 | 0.1200442 | -0.4614918 | -0.1717519 | 0.0262043 | -0.1428065 | 0.2735029 | -0.4013788 |
| -0.3838643 | -0.3459679 | -0.4549510 | -0.2124171 | -0.2509399 | -1.2711949 | -2.6328396 | -0.6322425 | -1.5978309 |
| 0.3590864 | -0.2406291 | -0.1596362 | 2.2467325 | 0.0443240 | -0.0156501 | 0.4863217 | 0.2016242 | -0.2513338 |
| -1.9163736 | -0.4407031 | -1.2549897 | -2.5788569 | -0.5297502 | -1.0225586 | -2.1499474 | -0.5434466 | -1.1041000 |
| 3.1786977 | 0.0142816 | 0.9554400 | 3.4405657 | 0.1043203 | 1.1658152 | 2.1504720 | -0.2052981 | 1.6028914 |
| 0.9091631 | 0.2033117 | 0.6738496 | 1.0245408 | -0.2587354 | 1.3264990 | -0.7030761 | 0.0504190 | 1.5383711 |
| 0.1641490 | -0.6394601 | -0.6042073 | -0.8594200 | -1.1438283 | -0.4118132 | -0.1759838 | -0.8139066 | -0.5314336 |
| -2.3308463 | -1.2943886 | -2.4275274 | -3.3707624 | -1.4039902 | -1.4468508 | -1.8503831 | -0.8481895 | -1.7817215 |
| 2.0987742 | 1.0027219 | 3.8600091 | 2.3459858 | -0.8517206 | 2.3756330 | 2.6550243 | -0.0689672 | 3.0108222 |
| 2.5403652 | 0.8004448 | 2.9728309 | 1.4449983 | -1.2114854 | 1.4045267 | 1.4281832 | -0.7088745 | 2.4291475 |
| -1.4914374 | -0.5242438 | -0.3636839 | -3.3046739 | -0.9538873 | -0.5658176 | -1.5874303 | -0.5238278 | -0.7857200 |
| -2.4941264 | -0.1990294 | -1.7190734 | -3.2814006 | -1.1870218 | -1.3021445 | -1.5896710 | -1.3280326 | -1.4428646 |
| -1.1093521 | -0.6110506 | -0.8992181 | -2.4979952 | -1.2873402 | -1.1097458 | -0.9747310 | -1.2352055 | -1.1702539 |
| -1.5241711 | 0.0812510 | -0.3069885 | -3.1081125 | -0.7910572 | -1.1308813 | -1.6098764 | -0.5941132 | -1.5014958 |
| -0.5264406 | -0.2787518 | -0.6573133 | -1.6416171 | -0.4527122 | -0.3747806 | -0.4963832 | -0.5377300 | -0.7543598 |
| -1.6273072 | -0.0808132 | -1.0278939 | -2.9704976 | -0.9016550 | -0.5562806 | -0.6321350 | -0.5052952 | -0.6495027 |
| -1.8671470 | 0.3226688 | -0.153706 | -3.1225607 | -2.9083152 | -2.0013878 | -2.4073686 | -2.3658211 | -1.5189852 |
| -0.2341515 | 0.0932422 | 0.2078305 | -1.1014957 | -0.4124312 | -0.1793013 | -0.3299711 | -0.2622342 | -0.1402229 |
| -2.3856785 | -0.3911129 | -0.6627883 | -2.6067497 | -0.7283306 | -0.6866809 | -1.6113974 | -0.7154873 | -0.7146078 |
| 1.0518229 | -0.4343819 | -0.6101153 | 0.0331945 | 0.5244435 | 0.4176745 | 0.8620630 | 0.0721924 | 0.5796909 |
| -1.2417664 | 0.7094459 | 0.8154579 | -3.8741723 | -2.5516756 | -2.1347766 | -2.9542308 | -2.7899440 | -2.1004341 |
| -1.8481512 | -0.8185491 | -0.9454777 | -2.0707241 | -1.2015628 | -1.4497783 | -2.1521599 | -0.9477897 | -1.0369937 |
| -0.8756619 | -0.3991401 | -1.2321163 | -2.4608052 | -0.7889090 | -0.7472671 | -0.7068145 | -0.7321189 | -0.8669788 |
| -1.9920228 | -0.2591911 | -2.6786101 | -3.6173600 | -0.2303904 | -0.7116293 | -0.4395548 | -0.8539354 | -0.8452846 |
| 4.2548435 | 0.8130447 | 2.5414566 | 2.8007249 | 0.1607313 | 1.5135726 | 3.0898569 | 0.4381459 | 1.6018007 |
| 2.6315276 | 0.1602864 | 0.4965301 | 1.9222731 | -0.4325925 | 1.0270803 | 3.4998982 | 0.0578012 | 2.0246580 |
| -1.7029429 | -0.5247022 | -0.6917177 | -2.9824675 | -0.4126015 | -0.5122587 | -0.8320772 | -0.3333600 | -0.4422849 |
| 0.0189812 | -0.1660881 | -0.4228128 | -0.1005790 | -0.0771071 | 0.1902023 | 0.1946481 | 0.2757466 | 0.1341555 |
| -0.4526821 | -0.2581833 | -0.3741771 | -3.5963033 | -2.9590611 | -1.8212852 | -1.6837049 | -1.5864191 | -1.4611739 |
| -1.6466095 | -0.3230302 | -1.3133078 | -1.7704038 | -1.0941064 | -1.2131712 | -0.9861120 | -1.1125023 | -1.4078516 |
| -0.3878751 | -0.1700149 | -0.2460292 | -2.7686308 | -1.1543681 | -0.8959554 | -1.4757065 | -0.5848346 | -1.1010866 |
| 2.2113292 | -0.1290335 | 1.2304979 | 1.9946645 | -0.1933787 | 0.5162081 | 1.2174903 | -0.1436021 | 0.3458174 |
| 0.2660125 | 0.1776080 | -0.0430837 | 0.7449455 | -0.4678164 | -0.3910282 | -1.4865105 | -0.1716188 | -0.7888359 |
| -0.6177685 | -0.4915246 | -0.3947183 | -0.6401306 | -0.2716053 | -0.2439127 | -0.2768500 | -0.3154272 | -0.0542451 |
| -0.8489981 | -0.0177446 | -0.4000007 | -1.9362172 | -0.5346236 | -1.0377423 | -2.2334487 | -0.4108346 | -1.5995404 |
| -0.9153999 | -0.2108161 | -0.7654169 | -2.3573129 | -0.1601274 | -0.1678298 | -1.2763694 | -0.5948602 | -0.9145247 |
| -1.6260899 | -0.5787894 | -2.0830761 | -1.7603215 | -0.2118600 | -0.7287487 | -1.6447729 | -0.3932258 | -0.7311797 |
| -0.6755656 | -0.4465179 | -0.8700450 | -2.9879395 | -0.7617608 | -0.6896622 | -1.4211852 | -0.5108825 | -0.5538485 |
| -2.3007828 | 0.4538442 | -0.1412530 | -2.6999498 | -0.4998542 | -0.6216333 | -0.3234981 | -0.6359497 | -1.0665078 |
| 2.9802907 | 0.4090969 | 2.9875809 | 2.2863539 | 0.2618373 | 1.9846820 | 1.3112314 | 0.4060420 | 3.1522333 |
| -0.5661467 | -0.0873253 | -0.0767916 | -1.1969168 | -0.6341966 | -0.4847894 | -0.8630517 | -0.1154255 | -0.4293342 |
| -1.8521737 | 0.0594090 | -0.4942324 | -3.1761067 | -1.7907971 | -1.4850534 | -2.5827269 | -1.3371002 | -1.0450685 |
| -1.5110984 | -0.9299716 | -1.4010252 | -1.1172966 | -0.2549637 | -0.7069396 | -0.8214527 | -0.8625242 | -0.3538704 |
| 4.0148860 | 0.5769918 | 7.9183335 | 7.1548145 | -0.1100415 | 1.9462380 | 2.7130250 | -0.1256633 | 4.0832660 |
| 1.0005119 | 0.2434639 | 2.1299732 | 1.2682013 | -0.7079603 | 1.0297232 | 1.6150739 | -0.6148894 | 1.7799992 |
| -3.2628834 | -0.0528824 | -0.5458234 | -3.4755520 | -1.6372830 | -1.9883963 | -3.3766093 | -1.5460899 | -1.8784759 |
| -0.0403135 | -0.3197662 | -0.1016333 | -0.7630400 | -0.1823444 | -0.0740297 | -0.7006928 | -0.0111696 | -0.0781498 |

| | | | | | | | | |
|---|---|---|---|---|---|---|---|---|
| -3.509234 | -0.181375 | -0.954753 | -3.189411 | -0.766429 | -0.792074 | -3.347312 | -0.785403 | -0.798723 |
| 0.3980905 | 0.1189505 | -0.100420 | -0.777262 | -0.448499 | -0.281048 | 0.6520410 | -0.549072 | -0.045506 |
| 0.2370747 | -0.016224 | -0.623687 | -0.433034 | -0.372067 | -0.360110 | 0.5894063 | -0.494956 | -0.950334 |
| -0.498452 | -0.006722 | -0.043036 | -1.322822 | -0.789681 | -0.347352 | -0.098455 | -0.277999 | -0.104695 |
| -2.195832 | -0.215939 | -2.337296 | -3.004966 | -0.900947 | -1.563502 | -1.440836 | -0.970952 | -1.963827 |
| -1.463442 | -0.628312 | -0.506086 | -1.876888 | -0.824661 | -0.735462 | -0.850955 | -0.930569 | -0.366347 |
| -0.741316 | -0.415304 | -0.134213 | -0.330958 | -1.890280 | -1.010855 | -1.217089 | -0.762669 | -0.626996 |
| 0.1128330 | 0.0737361 | 0.5575201 | 0.2466642 | -0.165463 | -0.024323 | -0.333890 | -0.084707 | -0.030742 |
| -0.348581 | -0.222281 | -0.034439 | -0.140168 | 0.2046383 | 0.0959124 | -0.094379 | -0.276418 | 0.0606741 |
| 0.6922616 | -0.059310 | 0.3273853 | -0.177824 | -0.091511 | 0.4686072 | 1.2700103 | 0.0963923 | 0.505033 |
| -1.264119 | -0.103683 | -0.417072 | -1.274292 | -0.564899 | -0.657476 | -1.234777 | -0.588763 | -0.635031 |
| -2.701434 | 0.2960469 | -0.705989 | -3.942924 | -2.174556 | -1.761170 | -3.005482 | -1.058395 | -1.341424 |
| -2.542076 | -0.154907 | -0.565608 | -1.677487 | -0.175064 | -0.592521 | -4.387948 | -0.382764 | -0.836223 |
| -3.095954 | -0.151589 | -1.276224 | -2.786657 | -0.551545 | -1.049179 | -1.715334 | -0.247457 | -0.737550 |
| -0.381427 | -0.179984 | -0.423897 | -1.699608 | -0.939302 | -0.547463 | -1.041807 | -0.515110 | -0.484812 |
| 0.1671690 | -0.171301 | -0.093373 | 0.1944406 | -0.236680 | -0.199650 | -0.299097 | 0.0700026 | -0.065098 |
| -0.697992 | -0.373489 | -1.427221 | -2.086557 | -1.337896 | -1.803469 | -0.974230 | -1.323826 | -1.951854 |
| 0.5029771 | -0.638228 | -1.121908 | 1.3754400 | 0.0182982 | 0.0061682 | -0.208425 | -0.234145 | -0.122400 |
| -0.758644 | 0.2430903 | -0.513608 | -2.101990 | -1.594027 | -2.156111 | -1.344517 | -1.151410 | -0.995965 |
| 1.8787097 | -0.389076 | 0.0123594 | 2.0505296 | 0.4111287 | 0.6249918 | 1.7327356 | -0.183773 | 0.4336212 |
| -0.421342 | -0.641731 | -0.527271 | -1.894821 | -0.734233 | -0.566647 | -1.082110 | -0.472758 | -0.645228 |
| 0.0164179 | -0.248861 | -0.500345 | -2.321136 | -0.965428 | -0.698985 | -0.835888 | -1.290633 | -0.808705 |
| 0.2339933 | -0.327883 | -0.347069 | -0.052305 | 0.0548715 | -0.018217 | -0.054424 | 0.1155009 | -0.102544 |
| -1.938407 | -0.358619 | -0.902709 | -2.838354 | -0.512611 | -0.853678 | -1.743528 | -0.558986 | -0.623920 |
| -0.255295 | -0.120127 | -0.449510 | 0.4231978 | -0.657923 | -1.130522 | -3.829002 | -0.733834 | -1.744930 |
| -0.316597 | -0.054674 | -0.327144 | -2.202711 | -1.184990 | -1.476087 | -0.757291 | -1.341536 | -1.521917 |
| -0.810562 | 0.3749180 | -0.100711 | -1.416050 | -0.877118 | -1.370973 | -0.651509 | -1.013855 | -0.960600 |
| 0.9138624 | -0.573734 | -0.310676 | 1.0004404 | 0.8292790 | 0.7865573 | 0.8880765 | 0.1922672 | 0.5099538 |
| -1.259572 | 0.3848512 | -0.039244 | -1.888216 | -0.247588 | -0.716191 | -1.698072 | -0.008539 | -0.959986 |
| -2.601169 | -0.229369 | -0.055982 | -3.713251 | -1.184985 | -0.613941 | -2.363805 | -1.058151 | -0.145404 |
| -1.615971 | -0.414127 | -0.568451 | -2.067147 | -0.374625 | -0.897117 | -0.432736 | -0.440196 | -0.998164 |
| -1.445304 | -0.182006 | -0.870016 | -2.337973 | -0.932638 | -0.877418 | -1.665465 | -0.818794 | -0.713755 |
| -0.050210 | -0.285907 | -0.400976 | 1.0807589 | -0.147607 | -1.178894 | -3.004658 | -0.674943 | -1.671643 |
| -0.200121 | 0.4406734 | 0.3499981 | -1.579234 | -1.008573 | -0.439786 | -0.937747 | -0.497761 | -0.201269 |
| -1.199394 | -0.061868 | -0.218720 | -1.911929 | -0.208870 | -0.445624 | -1.157405 | -0.575728 | -0.558644 |
| -0.819404 | -0.113499 | -0.707358 | -2.636780 | -1.136289 | -1.071968 | -0.855949 | -1.008720 | -0.815581 |
| -0.882680 | -0.054794 | -0.178601 | -2.579039 | -1.260095 | -0.832429 | -0.825954 | -0.898046 | -1.125027 |
| -1.948920 | -0.149916 | -0.214033 | -4.096265 | -1.698362 | -1.116920 | -2.149927 | -1.023921 | -0.708749 |
| -0.354510 | -0.248556 | -0.519052 | -0.563980 | -0.621480 | -0.488609 | -0.575865 | -0.612711 | -0.617178 |
| -2.436811 | -0.156436 | -0.532507 | -3.325687 | -1.006091 | -0.784709 | -2.764295 | -0.965561 | -0.682058 |
| -0.398950 | -0.938815 | -1.763691 | -1.865017 | -1.556638 | -1.187303 | -1.322881 | -1.697384 | -0.824836 |
| -1.386813 | -0.217415 | -0.091920 | -1.651487 | -0.841232 | -0.208271 | -1.161279 | -0.463372 | 0.1980588 |
| -2.132089 | 0.0713883 | 0.0450480 | -1.972523 | -1.433604 | -1.024212 | -0.845806 | -0.906466 | -1.082723 |
| -0.901046 | 0.3694363 | 0.1752151 | -2.266832 | -0.745364 | -0.933711 | -1.380888 | -0.705530 | -0.691962 |
| -0.231638 | -0.208695 | -0.250827 | 0.0928857 | -0.178163 | -0.244475 | -0.043593 | -0.319326 | -0.150072 |
| 0.1815337 | -0.080111 | 0.2512856 | -0.475720 | -0.754379 | -0.144581 | 0.0210032 | -0.289922 | 0.3012934 |
| -1.338464 | -0.714049 | -1.125043 | -1.481394 | -1.022326 | -0.909927 | -1.472258 | -0.930559 | -0.964151 |
| 0.3343005 | -0.500267 | 0.0104204 | -1.370474 | -0.946599 | -0.333311 | 1.1247640 | -0.517332 | 0.2782511 |
| -1.363664 | -0.371922 | -0.909383 | -1.644274 | -0.464052 | -0.817784 | -1.804942 | -0.707265 | -0.946293 |
| -1.508491 | -0.558253 | -0.428215 | -1.227883 | -0.945195 | -1.467371 | -1.888868 | -1.494005 | -1.141075 |

| | | | | | | | | |
|---|---|---|---|---|---|---|---|---|
| -0.603703 | 0.4637948 | 0.4347672 | -2.394144 | -1.840409 | -1.216407 | -0.198748 | -1.699381 | -0.974672 |
| -1.327132 | -0.770310 | -1.473353 | -1.478747 | -1.205296 | -1.790148 | -2.255155 | -1.305584 | -1.955321 |
| -0.815210 | -0.686895 | -0.825898 | -1.958753 | -0.625488 | -0.425504 | -0.034006 | -0.783819 | -0.373278 |
| -0.111132 | 0.0140721 | 0.1142212 | 0.8896632 | -0.424455 | 0.0148129 | -0.153871 | -0.338834 | 0.2946278 |
| -1.697850 | -0.146130 | -0.854319 | -1.310659 | -1.315619 | -1.296464 | -2.058538 | -1.380833 | -1.644170 |
| 2.2982243 | 0.1979717 | 2.4615051 | 2.2849247 | 0.5900077 | 1.6592866 | 1.5541687 | 0.2282822 | 1.4976690 |
| -0.660013 | -0.339469 | -0.614844 | -1.680286 | -1.054718 | -0.963872 | -1.452468 | -0.818449 | -0.901919 |
| -1.071459 | -0.717110 | -1.517087 | -1.739446 | -0.413531 | -0.405309 | -0.548957 | -0.923109 | -1.218453 |
| -2.441609 | -0.362450 | -1.384958 | -2.344285 | -0.672543 | -0.947339 | -1.740651 | -0.863567 | -1.398194 |
| -0.722105 | -0.320088 | -0.862192 | -1.445960 | -0.911581 | -0.782439 | -0.323353 | -1.077614 | -0.806335 |
| 0.2151238 | -0.092641 | -0.028934 | -1.167439 | -0.542181 | -0.330968 | -0.061936 | -0.391373 | 0.1388299 |
| -0.503981 | -1.143287 | -1.532545 | -0.691564 | -1.065163 | -1.072581 | -0.844647 | -1.043611 | -1.078536 |
| -0.775068 | 0.3124699 | -0.419948 | -1.428231 | -0.143135 | -0.621829 | 0.4046573 | -0.973665 | -0.164216 |
| -0.479261 | -0.237115 | -0.416820 | -2.110195 | -0.942016 | -0.617108 | -0.299947 | -0.731333 | -0.063877 |
| -0.106145 | -0.313541 | -0.376701 | 0.2890498 | -0.745527 | -0.874928 | -0.637321 | -0.806059 | -0.848386 |
| -1.179238 | 1.0520763 | 0.6699743 | -2.247548 | -0.690061 | -0.702026 | -2.148957 | -0.795164 | -0.927751 |
| 0.0756858 | -0.864780 | -0.532753 | 0.5649315 | 1.0457828 | 0.7885718 | 1.1012071 | 0.5549010 | 0.5135148 |
| -0.291483 | -0.778047 | -2.314505 | -2.932246 | -0.652099 | -0.837335 | -1.046491 | -1.038300 | -1.848314 |
| 0.0165387 | -0.795308 | -0.667792 | -1.705252 | -0.915432 | -0.896867 | -0.969859 | -0.530463 | -1.059078 |
| -0.808013 | -0.198719 | -0.532871 | -2.716219 | -2.002650 | -2.136780 | -2.231394 | -1.587518 | -1.663373 |
| 0.6456819 | -0.290302 | 1.3895989 | 1.8928128 | 0.2992391 | 0.4201855 | 0.7980861 | 0.0198592 | 0.8963468 |
| -0.380218 | -0.241320 | -0.425016 | -1.262671 | -1.423914 | -0.851041 | -1.038109 | -0.986722 | -0.702769 |
| -1.799773 | -0.666489 | -1.101123 | -2.775318 | -0.405924 | -0.970557 | -1.511419 | -0.502937 | -1.925860 |
| -0.163771 | -0.320826 | -0.069257 | -1.539502 | -1.387614 | -0.730471 | -1.250571 | -0.954471 | -0.907150 |
| -1.055425 | -0.337953 | -0.480147 | -1.785564 | -0.360702 | -0.891031 | -1.416233 | -0.705412 | -1.243687 |
| 0.4344132 | -0.237817 | -0.631064 | -2.037848 | -1.240165 | -0.927393 | -1.141366 | -0.838419 | -0.850955 |
| -0.539504 | -0.280833 | -0.816131 | -1.835063 | -1.111269 | -0.561051 | -0.457783 | -1.084488 | -0.942276 |
| -0.084667 | -0.127769 | -0.502696 | -0.612702 | -0.382319 | -0.562635 | 0.1701470 | -0.054164 | -0.476494 |
| -1.263634 | -0.670805 | -1.158630 | -2.169579 | -0.996476 | -1.563132 | -1.561699 | -1.181285 | -1.614533 |
| 1.5311895 | 0.0222097 | 0.6193329 | -0.186623 | -0.657563 | 0.9914207 | 1.4523272 | 0.0683889 | 0.9303715 |
| 2.7700505 | 0.2593336 | 1.0376775 | 3.1003729 | 0.7653352 | 1.6671922 | 3.4594036 | 0.8014162 | 2.4994945 |
| 0.1100311 | -0.682511 | -1.280194 | -1.353837 | -0.903666 | -1.108028 | -0.641144 | -0.927911 | -0.883356 |
| 0.0016646 | 0.1138142 | -0.119628 | -0.753975 | -0.701964 | -0.234908 | -0.039815 | -0.295419 | -0.031912 |
| 0.6050160 | 0.2007051 | 0.3459613 | -0.330466 | -1.286858 | -0.362062 | -0.511352 | -0.638605 | -0.299039 |
| 1.4255671 | 0.2554589 | 0.8024493 | 1.4325202 | 0.7432857 | 1.1664511 | 0.8015979 | 0.8712909 | 1.0755861 |
| -1.675747 | -0.663073 | -0.875900 | -2.753241 | -1.458308 | -1.521305 | -2.531457 | -1.139643 | -1.274879 |
| -0.466082 | 0.6053312 | 1.0559404 | -0.800240 | -1.591937 | -1.768191 | -1.655098 | -1.673259 | -1.481688 |
| 0.3543527 | -0.015550 | 0.3632548 | -0.719920 | -1.422893 | -0.360698 | -0.058769 | -0.641460 | -0.007907 |
| 0.0134973 | -0.252757 | -0.324232 | -1.287210 | -1.356048 | -0.587225 | -0.012484 | -0.901850 | -0.437385 |
| -1.037394 | -0.035308 | -0.243917 | -2.218067 | -2.113319 | -1.592467 | -2.191108 | -1.447551 | -1.326704 |
| -1.905985 | 0.5388222 | -0.343542 | -1.904158 | 0.8331888 | -0.824810 | -1.795347 | 0.1524139 | -0.801856 |
| 0.7775364 | -0.427620 | 0.2265302 | 0.1037146 | -0.640159 | 0.0522957 | 0.6718491 | -0.266214 | 0.1771738 |
| 2.0538184 | 0.5086609 | 1.0302296 | -0.158765 | -1.935980 | -0.872234 | 0.9319464 | -1.416640 | -0.249006 |
| 0.5202818 | 0.0080322 | 0.1573686 | -1.300793 | -1.256944 | -0.129076 | 0.4992907 | -1.009312 | -0.032847 |
| -0.432235 | -0.285453 | -0.465969 | -0.386759 | -0.903465 | -0.714268 | -0.152659 | -0.528866 | -0.855336 |
| -0.591416 | -0.182021 | -0.472798 | -1.475942 | -1.322693 | -0.707774 | -0.383816 | -1.223832 | -0.473717 |
| -0.550081 | -0.416667 | -0.928505 | -1.405937 | -0.389437 | -0.32315 | -0.630266 | -0.298240 | -0.597206 |
| -2.276702 | -0.268214 | -1.632694 | -2.707476 | -1.288631 | -1.469066 | -1.548177 | -0.989821 | -1.327757 |
| -0.996359 | 0.1157885 | -0.184458 | -0.854089 | -0.121946 | -0.394639 | -1.408618 | -0.147297 | -0.858272 |
| -0.411563 | -0.247502 | -0.306328 | -1.956848 | -2.249795 | -1.140787 | -0.529106 | -1.255868 | -0.984987 |

| | | | | | | | | |
|---|---|---|---|---|---|---|---|---|
| -0.823923 | 0.4119415 | 0.0957503 | -3.084524 | -2.010234 | -1.872485 | -1.857822 | -1.900170 | -0.824975 |
| -1.217359 | -0.515349 | -0.860795 | -0.622518 | -1.016805 | -0.902188 | -1.630257 | -1.516523 | -0.851904 |
| -2.366145 | -0.734164 | -0.369640 | -2.709419 | -1.015624 | -0.552761 | -2.106587 | -1.079901 | 0.1336796 |
| -1.468784 | -0.570689 | -0.950788 | -0.729888 | -0.707613 | -1.449009 | -2.298257 | -0.511132 | -1.598968 |
| -1.209682 | -0.265145 | -0.581732 | -3.270931 | -0.734599 | -0.888893 | -1.938164 | -0.832960 | -1.036969 |
| 0.0734542 | 0.1793524 | 0.3102488 | -0.273325 | 0.0408100 | -0.327714 | -0.238559 | -0.095315 | -0.527640 |
| 2.6476205 | 0.2339118 | 1.4002691 | 1.2573403 | 0.1889209 | 0.7161749 | 1.5477499 | 0.1956089 | 0.9358267 |
| -0.038735 | 0.0103783 | -0.140954 | 0.9936729 | 0.4482829 | -0.299415 | 0.3901947 | -0.051834 | -0.460727 |
| -2.469342 | -0.074397 | -0.353549 | -3.641204 | -3.558711 | -2.164827 | -3.181095 | -1.235847 | -1.307477 |
| 0.3348857 | -0.935735 | -1.141289 | -0.639233 | -0.677377 | -1.284180 | -1.546675 | -0.514525 | -1.394743 |
| -0.755739 | -1.053508 | -2.687836 | -2.165329 | -0.950426 | -0.213520 | -0.089485 | -0.605995 | 0.0885556 |
| 0.3245355 | -0.594995 | -0.653852 | -2.007179 | -1.367384 | -1.543335 | -1.023894 | -1.067589 | -0.853278 |
| -0.918392 | 0.3089505 | 0.2269087 | -0.634086 | 0.3421141 | 0.2046957 | -2.327146 | -0.209589 | -0.048747 |
| -0.322836 | 0.2293679 | -0.011589 | -1.841395 | -1.562624 | -1.029674 | -0.590157 | -1.188829 | -0.851926 |
| -1.670229 | -0.209888 | -0.284281 | -2.571572 | -0.444261 | -0.958283 | -2.215167 | -0.440066 | -1.288109 |
| -0.986119 | 0.0688193 | -0.337662 | -3.069440 | -0.329710 | -0.622735 | -0.659570 | -0.922380 | -0.337132 |
| -1.573532 | 0.2461967 | 0.0226188 | -5.235421 | -3.625905 | -2.430884 | -3.602115 | -2.038833 | -2.124978 |
| -1.600491 | -0.225595 | -0.472461 | -2.259714 | -0.431555 | -0.753894 | -0.884693 | -0.426118 | -0.696482 |
| -1.664448 | -0.407986 | -1.463118 | -3.090999 | -0.790681 | -1.529193 | -1.658621 | -0.503056 | -2.304156 |
| -0.924615 | 0.2846006 | 0.0903317 | -2.476601 | -1.757944 | -1.611748 | -1.070841 | -1.660522 | -1.037842 |
| -2.017293 | -1.074917 | -0.602844 | -2.037408 | -1.259787 | -1.474710 | -2.406324 | -1.025491 | -1.283695 |
| -0.173928 | -0.470182 | -0.359547 | 0.1811589 | -0.377285 | -0.482467 | -0.353629 | -0.461730 | -0.414760 |
| -2.246719 | -1.232316 | -2.275350 | -1.539814 | -0.277412 | -1.772828 | -3.484038 | -0.373693 | -2.526792 |
| -0.410548 | -0.006857 | -0.133920 | -1.486046 | -1.163423 | -0.848162 | -0.132715 | -0.947600 | -0.634035 |
| -1.135185 | -0.392919 | -0.693008 | -1.256951 | -1.028694 | -0.745886 | -1.522180 | -0.947537 | -0.520409 |
| -0.451859 | -0.484860 | -0.841156 | -1.272557 | -0.521322 | -0.563405 | 0.6448598 | -0.603708 | -0.262299 |
| -1.156009 | -0.265558 | -0.207118 | -1.524743 | -0.636019 | -0.573315 | -2.431144 | -0.307377 | -0.610560 |
| -1.103970 | -0.615722 | -0.796875 | -2.573631 | -1.127678 | -0.509502 | -0.894161 | -0.812584 | -0.525838 |
| 0.7545963 | 0.1642910 | 0.2586904 | -0.071279 | -0.213908 | 0.4393372 | 1.4298594 | -0.089685 | 0.5120272 |
| 0.9266082 | -0.090295 | 0.5657722 | -0.133206 | -1.707135 | -1.145411 | 0.6736604 | -1.583509 | -0.755699 |
| -0.329616 | -0.686230 | -0.425213 | -1.586284 | -0.253126 | -0.061324 | -0.604616 | -0.303256 | -0.237353 |
| -0.688440 | -0.024277 | -0.278014 | -2.628280 | -1.714114 | -1.023419 | -1.939542 | -0.729838 | -0.988755 |
| -0.336905 | -0.001748 | -0.169287 | -0.710802 | 0.3965617 | -0.044010 | 0.7758426 | -0.007563 | -0.075417 |
| -1.085934 | -0.491016 | -0.672528 | -2.034036 | -0.368640 | -0.537797 | -0.808793 | -0.372686 | -1.017541 |
| -0.734552 | 0.0226843 | -0.034324 | -1.457051 | -0.282020 | -0.672020 | -1.634233 | -0.629531 | -0.201890 |
| -1.363848 | -0.615659 | -0.936887 | -3.645666 | -1.041509 | -0.887688 | -2.381532 | -0.553426 | -1.002874 |
| -2.074304 | -1.021159 | -1.543234 | -1.923512 | -0.619003 | -0.938170 | -1.892228 | -0.543141 | -1.244560 |
| -0.276443 | -0.306426 | -0.211152 | -0.040268 | -0.019355 | 0.0388382 | -0.217530 | -0.039581 | -0.269501 |
| 1.1281207 | -0.774040 | 0.2543072 | 1.0283671 | -0.300976 | 0.5124823 | 0.3268593 | -0.760494 | 0.4941744 |
| -0.169084 | -0.115631 | -0.053067 | -1.244358 | -0.806981 | -0.703386 | -1.037479 | -0.566723 | -1.110767 |
| -0.222031 | 0.1382819 | -0.037976 | -2.025434 | -2.833882 | -1.551327 | -1.153007 | -2.324625 | -0.628678 |
| -2.049839 | -0.711520 | -1.241337 | -4.214281 | -1.275717 | -0.803990 | -2.199124 | -0.733931 | -0.762087 |
| -1.048091 | -0.420492 | -0.960395 | -0.978831 | -0.540308 | -0.489585 | -0.557562 | -0.507935 | -0.749875 |
| -1.133889 | 0.3038613 | -0.147602 | -1.873746 | -1.637969 | -1.087536 | -1.193678 | -1.129040 | -0.716484 |
| 0.1468735 | -0.578167 | -0.354023 | -0.968126 | -0.937649 | -1.224787 | -0.951829 | -0.621183 | -0.789439 |
| -0.851407 | -0.367295 | -0.861888 | -2.044544 | -2.545166 | -2.039575 | -2.968622 | -1.602739 | -2.246542 |
| -0.899365 | -0.011404 | -0.128722 | -1.313462 | -0.848118 | -1.090941 | -0.803711 | -0.587873 | -1.063321 |
| -1.010398 | -0.716026 | -1.949334 | -3.015851 | -1.235129 | -1.119389 | -1.300947 | -1.230206 | -1.025702 |
| -0.773467 | -0.975821 | -0.467718 | -0.937980 | -0.941133 | -1.034876 | -0.640035 | -0.993021 | -1.034955 |
| -1.393215 | -0.929786 | -1.262725 | -1.867184 | -0.879212 | -1.110981 | -1.955214 | -0.813502 | -1.207526 |

| | | | | | | | | |
|---|---|---|---|---|---|---|---|---|
| 0.0715544 | -0.2095776 | 0.2207106 | -0.6664667 | -0.2204055 | -0.4784765 | -0.2634666 | -0.2353965 | -0.3354165 |
| 0.3144647 | -0.1961037 | -0.3452403 | -0.5216158 | -0.3514916 | -0.6605887 | -0.2132025 | -0.6939458 | -0.2521917 |
| -0.1385951 | -0.4130860 | -0.3675320 | -1.0722926 | -0.7734590 | -0.5215924 | -0.2864892 | -0.8065555 | -0.4691974 |

| PG_426 | PG_427 | PG_428 | PG_429 | PG_430 | PG_431 | PG_432 | PG_433 | PG_434 |
|---|---|---|---|---|---|---|---|---|
| -1.660689 | -0.016421 | -0.596639 | -1.377391 | -0.184588 | -0.756806 | -0.936908 | -0.376620 | -0.463782 |
| -1.452951 | -0.503233 | -0.929929 | -1.495951 | -0.648043 | -0.850701 | -1.344138 | -0.424374 | -0.767458 |
| -0.171133 | -0.469839 | -0.748700 | -3.063052 | -0.594284 | -1.268690 | -2.952855 | -0.843458 | -0.573058 |
| -1.551990 | -0.293400 | -0.615835 | -0.109999 | -0.199516 | -0.720168 | -0.265313 | -0.537308 | -0.461659 |
| -1.549739 | -0.200538 | -0.317815 | -1.230792 | -0.083348 | -0.164499 | -1.355088 | -0.002463 | 0.0092181 |
| -3.169011 | -0.068841 | -0.685773 | -1.588186 | -0.170790 | -0.679097 | -1.793567 | -0.217154 | -0.804930 |
| -2.006279 | -0.337992 | -1.964307 | -1.209247 | -0.386812 | -1.858259 | -1.363805 | -0.223839 | -0.678790 |
| -2.039949 | -0.253390 | -0.727462 | -2.125720 | -0.188409 | -0.801164 | -2.355848 | -0.375861 | -0.219178 |
| 0.4382274 | -0.172670 | -0.146898 | 0.5010693 | -0.186797 | -0.019844 | 0.4690100 | -0.314653 | -0.300470 |
| -0.619610 | -0.718139 | -1.624777 | 0.1479316 | -0.945415 | -1.602251 | 0.2919781 | -0.571206 | -0.519056 |
| -1.553900 | -0.238296 | -0.415839 | -0.546065 | -0.237613 | -0.499970 | -0.599602 | -0.196582 | -0.242125 |
| -1.356950 | -0.143419 | -0.370742 | -0.357294 | -0.175042 | -0.638974 | -0.537649 | -0.213850 | -0.327373 |
| -3.371395 | -0.534231 | -1.519001 | -5.398118 | -0.479505 | -1.304850 | -4.778125 | -0.652187 | -0.716472 |
| 4.2325028 | -1.288793 | 4.1204943 | 5.6113885 | -0.837198 | 5.3016688 | 5.6144792 | -0.943360 | 2.2248118 |
| -1.876265 | 0.0649717 | -0.154285 | 0.5248483 | 0.1858813 | -0.164986 | 0.6559545 | -0.136922 | -0.309632 |
| 2.9386585 | -0.143076 | 2.5256959 | 3.6651739 | 0.4757683 | 2.7878773 | 3.6698435 | 0.1335058 | 1.2495677 |
| -2.377712 | -1.031108 | -2.350459 | -2.768678 | -0.951294 | -2.712205 | -2.614460 | -1.273102 | -2.056804 |
| -0.228841 | -0.166758 | -0.110282 | -0.559116 | -0.008221 | 0.0252073 | -0.194831 | -0.279147 | -0.177952 |
| -0.376705 | 0.1863916 | -1.374258 | -2.398843 | -0.350044 | -1.870026 | -2.169502 | -0.250332 | 0.0397761 |
| -0.789971 | -0.910849 | -0.585033 | -0.564786 | -0.792903 | -0.548481 | -0.791211 | -0.942677 | -0.589967 |
| -0.461289 | -0.130444 | 0.5612438 | 0.5138562 | 0.0477727 | 0.4113015 | 0.4428972 | -0.555271 | 0.2478691 |
| -0.260017 | -0.385008 | -0.421307 | -0.605349 | -0.648457 | -0.276485 | -0.464187 | -0.118870 | -0.569430 |
| 2.3732568 | 0.1530731 | 1.1263187 | 1.9810201 | 0.0942664 | 1.1600991 | 1.8607177 | -0.396025 | 0.2489986 |
| -2.837614 | -0.556237 | -1.238496 | -1.691102 | -0.676391 | -1.716266 | -2.118986 | -0.678041 | -0.742001 |
| -2.295777 | -0.601713 | -1.398062 | -1.556768 | -0.761945 | -1.572998 | -1.777246 | -0.163889 | -0.983638 |
| 1.5557652 | -0.189515 | 1.0744861 | 1.7726259 | -0.027971 | 1.0407068 | 1.7059320 | -0.278702 | 0.6764342 |
| -1.709975 | -0.040859 | -0.317916 | -0.329060 | 0.0006902 | -0.196449 | -0.286158 | 0.1253892 | -0.132478 |
| -1.875495 | -0.681681 | -1.394137 | -2.825581 | -1.057123 | -1.797177 | -2.983646 | -1.501318 | -1.614517 |
| -2.034577 | -0.412784 | -1.515630 | -2.023194 | -0.555050 | -1.957355 | -2.003472 | -0.023021 | -0.536233 |
| -1.285140 | -0.002657 | -0.299573 | -0.756951 | -0.010730 | -0.390396 | -0.723074 | -0.318659 | -0.524719 |
| -0.949025 | -0.884361 | -0.711233 | -0.704588 | -0.399010 | -0.561099 | -0.639503 | -0.057538 | -0.330560 |
| 0.1244853 | 0.2229762 | -0.204315 | 0.0974561 | -0.398727 | -0.190147 | 0.6911533 | -0.230748 | -0.211756 |
| -0.774062 | 0.0463006 | -0.021194 | -3.874834 | 0.1879337 | 0.2999099 | -3.139818 | -0.114406 | -0.239158 |
| -2.348516 | -0.384407 | -0.988386 | -3.868599 | -0.736336 | -1.057777 | -3.894307 | -0.411584 | -0.763901 |
| -0.301466 | -0.465969 | -0.526921 | -0.489628 | -0.316853 | -0.428034 | -0.458077 | -0.055288 | -0.133613 |
| -0.326163 | -0.256435 | -0.664463 | -0.205378 | -0.524987 | -1.182847 | -0.096921 | -0.476496 | -0.995160 |
| -1.886669 | 0.3786090 | -0.473634 | -1.729188 | 0.0135684 | -0.308646 | -1.651083 | -0.011445 | -0.518607 |
| 1.1206533 | -0.342116 | 4.3654330 | 0.9960605 | -0.080340 | 4.7660783 | 0.8199185 | 0.1228175 | 2.1340983 |
| -1.203757 | 0.4989242 | 0.9977697 | 0.5170932 | 0.5144471 | 0.5879468 | 0.7352361 | -0.262472 | -0.583282 |
| -1.265475 | -0.756281 | -1.231395 | 0.3950629 | -0.970202 | -1.010043 | 0.7325902 | -0.504126 | -1.259003 |
| 0.5399037 | 0.3270114 | 0.6295009 | 0.8684772 | 0.3981106 | 0.5599446 | 0.9391134 | 0.0910803 | 0.2233781 |
| -2.222222 | -0.311322 | -0.985462 | -2.098668 | -0.308957 | -1.293864 | -1.987025 | -0.737968 | -0.678786 |
| -0.950476 | -0.672091 | -1.056145 | -0.200091 | -0.845309 | -1.110091 | -0.005083 | -0.393349 | -0.463169 |
| -2.271272 | -0.051702 | -0.419211 | -1.216161 | -0.052668 | -0.535426 | -1.095184 | -0.063412 | -0.108240 |
| 1.9014523 | 0.2713171 | 0.7456303 | 1.2646165 | 0.1426354 | 0.8522782 | 1.2469688 | -0.075816 | 0.3218765 |
| 1.0474827 | -2.184581 | -2.325294 | 2.0291590 | -2.173235 | -1.928866 | 2.8992320 | -0.046282 | 0.2976746 |
| -0.480451 | -0.140244 | -0.172547 | -0.136427 | -0.483851 | -0.502797 | -0.163044 | -0.303533 | 0.1836542 |

| | | | | | | | | |
|---|---|---|---|---|---|---|---|---|
| -0.492479 | -0.446452 | -0.183098 | -0.051247 | -0.438512 | 0.0718623 | 0.2355618 | -0.265864 | -0.318543 |
| -3.067166 | -0.145644 | -0.583078 | -2.744611 | -0.165425 | -0.744656 | -2.478296 | -0.767845 | -0.870658 |
| 0.1320322 | -0.465980 | 0.3008620 | 0.6433310 | -0.298076 | 0.1100980 | 0.5278620 | -0.513189 | -0.309719 |
| -0.287799 | -0.576754 | -0.460627 | 0.0398780 | -0.394777 | -0.441938 | -0.360983 | 0.0377391 | -0.214616 |
| -2.679250 | -0.795808 | -2.110240 | -2.176324 | -1.382588 | -2.697539 | -1.703953 | -0.291361 | -0.298040 |
| 0.3483867 | -0.342803 | -0.275037 | -0.158024 | -0.360827 | -0.306297 | 0.3619355 | -0.382851 | 0.0231032 |
| -2.004060 | -0.021564 | -0.786611 | -1.833101 | -0.283333 | -0.892599 | -1.684032 | -0.138163 | -0.765871 |
| 2.1038050 | -0.476760 | 3.5940401 | 2.4959243 | 0.3237541 | 3.4207805 | 2.5126668 | -0.675916 | 1.3146981 |
| -0.567219 | -1.066460 | 1.2088526 | -1.573873 | -1.545715 | 0.8907575 | -1.235000 | -1.294373 | 3.3012339 |
| -0.136839 | -0.373076 | -0.206208 | -0.476470 | -0.503434 | -0.547820 | -0.478306 | -1.369844 | -0.964292 |
| -2.158270 | -0.608773 | -1.236864 | -1.485469 | -0.636178 | -1.630616 | -1.246661 | -0.406998 | -0.942491 |
| 2.8788226 | 0.1913865 | 2.9123755 | 3.8848642 | 1.0758079 | 3.2903206 | 3.9769550 | 0.0045005 | 2.5231796 |
| 1.8363211 | -0.340463 | 3.0103346 | 3.2715248 | 1.0795876 | 3.1030376 | 3.3784686 | -0.530266 | 1.4374479 |
| -1.533879 | -0.568629 | -0.528421 | -0.838340 | -0.630927 | -0.704879 | -0.796331 | -0.742364 | -1.181811 |
| -1.455375 | -0.687869 | -2.133126 | -2.386772 | -1.473133 | -2.231840 | -2.314162 | -1.371215 | -1.806829 |
| -0.822899 | -0.361605 | -0.760892 | -0.145823 | -0.602721 | -0.614748 | -0.334844 | -0.541394 | -1.292532 |
| -1.971262 | -0.262655 | -0.623520 | -0.792285 | -0.347001 | -0.992021 | -0.915348 | -0.297925 | -0.372554 |
| -0.620642 | -0.042392 | -0.458377 | -0.589708 | -0.247035 | -0.598554 | -0.570739 | -0.218995 | -0.279592 |
| -1.154629 | -0.110123 | -0.510963 | -0.475953 | -0.208462 | -0.545456 | -0.328423 | -0.437644 | -0.559332 |
| -2.458889 | 0.4475711 | -0.108500 | 0.2410911 | 0.4458940 | -0.149568 | 0.0673954 | 0.3487404 | -0.005095 |
| -0.613202 | 0.0045054 | -0.093369 | -0.023020 | -0.052658 | -0.236364 | 0.1916899 | 0.0538205 | 0.0537484 |
| -1.507899 | -0.553750 | -1.162209 | -2.744061 | -0.338815 | -1.088305 | -2.648912 | -0.478162 | -0.805519 |
| 0.8282019 | 0.1375486 | 0.3241814 | 0.3265369 | 0.1639175 | -0.132818 | -0.000389 | -0.091019 | -0.781486 |
| -2.719910 | 0.6209752 | 0.4460495 | 0.6052915 | 0.6701199 | 0.5674850 | 0.3809906 | 0.5054538 | 0.4650155 |
| -2.112068 | -0.721033 | -1.324387 | -1.364673 | -0.816480 | -0.754745 | -1.700752 | -0.857507 | -0.882904 |
| -0.493278 | 0.2781567 | -0.015482 | 0.3038014 | 0.2480190 | -0.112095 | 0.3939357 | -0.281520 | -0.437548 |
| -0.770077 | -1.086836 | -0.938500 | -0.656544 | -0.665733 | -1.113464 | -0.244186 | -0.634562 | -1.611406 |
| 3.4869744 | 0.3786722 | 2.0923197 | 3.5943173 | 0.5333618 | 1.8009194 | 3.3599718 | -0.184839 | 0.5049990 |
| 3.2006489 | 0.3702232 | 2.1622712 | 3.2200246 | 1.0078405 | 2.5287776 | 3.1417979 | -0.268947 | 2.3030864 |
| -0.938986 | -0.027306 | -0.674169 | -0.642467 | -0.135555 | -0.856701 | -0.399343 | -0.184624 | -0.608965 |
| -0.013428 | -0.041760 | -0.058408 | -0.129622 | 0.2010671 | -0.226831 | -0.005730 | 0.3377741 | 0.5401486 |
| -1.628603 | 0.0631908 | -0.143680 | 0.1897975 | 0.1313033 | -0.268791 | 0.2224479 | -0.210196 | -0.162758 |
| -0.980489 | -0.174156 | -1.006159 | -0.872453 | -0.355453 | -1.385157 | -0.742795 | -0.219502 | -1.123178 |
| -1.638799 | -0.086631 | -0.637672 | -0.625933 | -0.089036 | -0.644951 | -0.860230 | -0.231973 | -0.338268 |
| 1.2697315 | -0.115202 | 1.8094007 | 2.1937626 | 0.3809437 | 1.9250713 | 2.2204691 | -0.353091 | 0.5067870 |
| -1.668164 | -0.104563 | -0.613304 | -1.201960 | -0.114732 | -0.739736 | -0.911726 | -0.456984 | -0.378561 |
| -0.166420 | -0.275831 | -0.544882 | -0.367806 | -0.212886 | -0.242071 | -0.562477 | -0.816472 | -0.400384 |
| -2.638668 | -0.708698 | -1.299852 | -1.532664 | -0.676150 | -1.729872 | -1.594913 | -0.391611 | -0.516708 |
| -2.050843 | -0.042204 | -0.182841 | -1.164148 | -0.176624 | -0.446647 | -1.452843 | -0.323473 | -0.575131 |
| -1.722760 | -0.615418 | -1.292038 | -1.633869 | -0.208326 | -1.297711 | -1.721420 | -0.489032 | -1.592193 |
| -1.115288 | -0.480967 | -1.026219 | -1.590627 | -0.684624 | -1.206957 | -1.487204 | -1.055579 | -0.678598 |
| -1.136980 | -0.554548 | -2.834245 | -1.836511 | -0.975074 | -3.362537 | -1.699782 | -0.276509 | -0.221500 |
| 0.2780156 | 0.8881504 | 5.0255725 | 2.9494031 | 1.9204430 | 5.1221900 | 2.8338079 | 0.3280875 | 2.9417340 |
| -0.788740 | -0.201960 | -0.340870 | -0.469524 | -0.340501 | -0.492733 | -0.399287 | -0.208418 | -0.126045 |
| -1.864760 | -0.235591 | -0.397266 | -1.283930 | -0.361162 | -0.432940 | -1.088089 | -0.070952 | -0.418261 |
| -0.465964 | -0.123796 | -0.915560 | -0.286782 | -0.211169 | -0.665250 | -0.219191 | -0.565805 | -0.415091 |
| 5.5479729 | -0.345511 | 2.9369361 | 5.6940014 | 0.0443716 | 3.9416199 | 6.4795399 | -0.034602 | 5.2899156 |
| 1.8969391 | 0.0314169 | 2.6926467 | 2.7190882 | 0.3419172 | 3.0879897 | 2.6960741 | 0.2140200 | 1.1026963 |
| -3.374744 | -0.715693 | -2.478990 | -1.447007 | -1.222817 | -3.170646 | -0.651710 | -0.847299 | -0.172240 |
| -0.792326 | -0.040539 | -0.080007 | -0.085153 | -0.137486 | 0.1317050 | -0.350065 | -0.557601 | -0.375882 |

| | | | | | | | | |
|---|---|---|---|---|---|---|---|---|
| -2.947638 | -0.583369 | -1.587575 | -5.025559 | -0.599302 | -1.732997 | -4.504314 | -0.441779 | -0.445406 |
| 0.5796241 | -0.301887 | -0.309865 | 0.0211772 | -0.576285 | -0.261050 | 0.1714570 | -0.324186 | -0.361385 |
| -0.056109 | -0.484022 | -0.384966 | -0.181153 | -0.448899 | -0.711234 | -0.394680 | -0.757754 | -1.442461 |
| 0.3409578 | -0.550722 | -0.423332 | -0.250557 | -0.412078 | -0.360248 | -0.186504 | -0.113503 | 0.0668789 |
| -2.237268 | -0.302290 | -1.202743 | -1.395361 | -0.364967 | -1.616364 | -1.511440 | -0.133858 | -0.917714 |
| -0.661834 | -0.725183 | -0.018783 | -1.530592 | -0.569696 | -0.310878 | -1.198440 | -0.980010 | -0.602030 |
| -0.768646 | -0.217466 | 0.5507127 | 0.5127891 | -0.085388 | 0.2846961 | 0.5620314 | -0.314549 | -0.158167 |
| -0.146648 | -0.078263 | -0.088687 | 0.0453682 | -0.012766 | 0.0706780 | -0.031340 | 0.2956532 | 0.6244434 |
| -0.128817 | -0.048022 | -0.201879 | -0.009058 | -0.142067 | -0.051123 | -0.750416 | -0.100610 | -0.310542 |
| 1.1184613 | 0.2385241 | 1.0767032 | 1.1503697 | 0.4850094 | 0.9159128 | 1.1338062 | -0.070504 | 0.0701800 |
| -0.816487 | -0.042699 | -0.120807 | -1.001789 | -0.061600 | 0.0987250 | -0.855964 | -0.539554 | -0.691898 |
| -3.030826 | -0.419078 | -0.418367 | -1.657453 | -0.276948 | -0.755076 | -1.583612 | -0.960321 | -0.516182 |
| -4.602480 | -0.681337 | -1.362574 | -3.901515 | -0.688188 | -1.199803 | -3.631897 | -0.233041 | -0.551040 |
| -1.468084 | -0.625570 | -1.024080 | -2.622395 | -0.524859 | -1.042665 | -1.939614 | -0.315876 | -0.754595 |
| -1.126948 | -0.208782 | -0.279100 | -0.500707 | -0.269256 | -0.313232 | -0.489802 | -0.045555 | -0.602789 |
| 0.1126965 | 0.0504137 | -0.236391 | -0.258046 | -0.201174 | -0.168056 | -0.084746 | -0.123012 | -0.226823 |
| -1.333657 | -0.271181 | -0.970396 | -1.662552 | -0.700298 | -1.599883 | -1.411734 | -0.818180 | -1.149005 |
| 0.2900960 | -0.345125 | -0.288272 | -0.047921 | -0.082454 | -0.563280 | -0.530590 | -0.016375 | 0.1215222 |
| -1.619029 | -0.232489 | -0.549382 | -0.163143 | -0.453826 | -0.774327 | -0.077916 | -0.252369 | -0.576598 |
| 1.5096630 | -0.234631 | 0.3479400 | 1.6528486 | -0.170628 | 0.1776496 | 1.9221808 | -0.794880 | -0.669248 |
| -1.641576 | -0.075877 | -0.272053 | -0.772710 | -0.131279 | -0.284816 | -0.787291 | -0.312978 | -0.394388 |
| -0.664318 | -0.786838 | -1.144272 | -0.728033 | -0.747819 | -1.320371 | -1.003605 | -0.415867 | -0.469689 |
| 0.1605002 | -0.571194 | -0.286363 | -0.161342 | -0.340116 | -0.203692 | -0.211214 | -0.110344 | -0.172417 |
| -1.827531 | -0.364652 | -0.909829 | -2.183586 | -0.431250 | -0.914660 | -2.184455 | -0.143025 | -0.197143 |
| -3.405067 | -0.980333 | -2.190797 | -0.179980 | -1.386640 | -2.398323 | -0.043808 | -0.395617 | 0.2320557 |
| -0.948402 | -0.598550 | -0.905785 | -0.581345 | -0.522943 | -1.057935 | -0.502127 | -0.280181 | -0.472689 |
| -0.544631 | 0.2197288 | 0.0037386 | 0.0322779 | 0.1197233 | -0.046063 | 0.1004267 | 0.0154335 | -0.325260 |
| 1.0833903 | -0.627469 | -0.409581 | -0.359345 | -0.705389 | -0.576970 | -0.313983 | -0.174389 | -0.105865 |
| -1.655068 | -0.358460 | -1.419632 | -1.259599 | -0.425718 | -1.803367 | -1.016166 | -0.353945 | -0.427082 |
| -1.214181 | -0.470152 | -1.209016 | -2.876481 | -0.253771 | -0.578518 | -2.727531 | -0.115117 | -0.290189 |
| -0.256535 | 0.8561280 | 0.1880927 | 0.9223742 | 1.1531282 | 0.1115774 | 1.1250416 | -0.129441 | 0.3098228 |
| -1.874453 | -0.060528 | -0.571443 | -0.813508 | -0.040952 | -0.664671 | -0.839821 | -0.058827 | -0.396347 |
| -2.827028 | -0.622479 | -1.550619 | -3.435793 | -1.031598 | -1.768092 | -2.898645 | -0.497685 | -0.133664 |
| -0.833417 | 0.5705494 | 0.4179908 | 0.1267608 | 0.6326751 | 0.3584494 | 0.0064435 | 0.4040386 | 0.3354282 |
| -1.606647 | -0.142038 | -0.412116 | -1.596636 | -0.070476 | -0.641900 | -1.846160 | -0.678818 | -0.262374 |
| -0.701563 | -0.141704 | -0.535014 | -0.221273 | -0.118344 | -0.907042 | -0.319971 | -0.173157 | -0.554093 |
| -1.335371 | -0.099578 | -0.469899 | -0.142138 | -0.228617 | -0.754503 | -0.188598 | -0.100276 | -0.229916 |
| -1.865310 | -0.282554 | -0.380330 | -1.813736 | -0.178654 | -0.168370 | -1.874837 | -0.235400 | -0.448660 |
| -0.294707 | -0.143387 | -0.338129 | 0.0811571 | -0.524190 | -0.336508 | -0.111611 | -0.555870 | -0.700205 |
| -1.606061 | -0.602550 | -1.024926 | -2.678992 | -0.428862 | -0.691163 | -2.634327 | -0.893963 | -0.965044 |
| -2.350932 | -1.225579 | -1.519021 | -1.624904 | -1.507067 | -1.784074 | -1.239120 | -0.367360 | -0.184007 |
| -0.553323 | 0.0383949 | -0.075838 | -2.429540 | 0.0004815 | 0.0827292 | -1.973537 | 0.0503848 | -0.056714 |
| -0.501070 | 0.4096100 | -0.268491 | -0.543631 | 0.3997237 | -0.231749 | -0.472315 | 0.0298840 | 0.0497538 |
| -1.359237 | -0.450932 | -0.669979 | -0.474153 | -0.509441 | -0.713931 | -0.503582 | -0.584824 | -0.686491 |
| -0.238027 | -0.228569 | -0.094637 | -0.280984 | -0.123383 | -0.215505 | -0.232821 | -0.190285 | 0.0242054 |
| 0.2826869 | -0.402798 | -0.005354 | -0.081228 | -0.423324 | -0.045843 | -0.258296 | -0.399255 | 0.2142561 |
| -1.193877 | -0.451958 | -1.323887 | -2.101309 | -0.624416 | -1.293842 | -1.704019 | -0.493283 | -0.643429 |
| 1.2891414 | -0.451354 | 0.2214582 | 1.5659163 | -0.315867 | 0.4324357 | 1.9741105 | -0.971071 | -0.211929 |
| -1.552140 | -0.499933 | -1.095606 | -0.926466 | -0.505561 | -0.779182 | -0.958126 | -1.055638 | -1.091376 |
| -2.098761 | -0.972458 | -1.096947 | -1.869543 | -0.795383 | -1.207057 | -1.682155 | -1.135261 | -0.928484 |

| | | | | | | | | |
|---|---|---|---|---|---|---|---|---|
| 0.1024684 | 0.3844717 | 0.2751703 | 0.2555415 | 0.2776598 | 0.2144919 | 0.3537934 | 0.2470871 | 0.1434688 |
| -2.241552 | -0.698305 | -1.867599 | -2.252141 | -0.628504 | -2.268962 | -2.717016 | -0.655424 | -1.371833 |
| 0.1950882 | -0.478182 | 0.1513066 | 0.1692376 | -0.362873 | -0.026933 | 0.1471029 | -0.454162 | -0.606701 |
| -0.440723 | -0.992172 | -0.057272 | 0.3097425 | -0.601795 | 0.0129699 | 0.6134179 | -1.101220 | 0.0881946 |
| -1.800629 | -0.439092 | -1.453682 | -2.717316 | -0.458135 | -1.868248 | -2.492200 | -0.158748 | -0.504380 |
| 0.7232448 | 0.4589276 | 2.9639473 | 1.8012269 | 1.7251189 | 3.0599236 | 2.1663962 | -0.058808 | 1.9873447 |
| -1.281137 | 0.1753681 | -0.026562 | -0.519328 | 0.1501377 | -0.114645 | -0.391756 | -0.339773 | -0.544008 |
| -0.893421 | -0.022420 | -0.316217 | -0.820446 | -0.361955 | -0.851899 | -1.074586 | -0.440586 | -0.844674 |
| -2.503865 | -0.287941 | -1.140472 | -1.432138 | -0.521977 | -0.902649 | -1.638410 | -0.305141 | -0.698475 |
| -0.126656 | -0.752936 | -0.679282 | -0.463585 | -0.689375 | -0.756299 | -0.467459 | -0.555714 | -0.798487 |
| 0.5260807 | 0.1195355 | 0.3471957 | 0.6550174 | 0.2078579 | 0.3494905 | 0.5943266 | -0.014219 | 0.1094034 |
| -0.966600 | -0.337661 | -1.102494 | -1.203536 | -0.414982 | -1.175286 | -1.193967 | -0.604884 | -0.909588 |
| 0.9184292 | -0.710547 | -0.649019 | -0.146541 | -0.505412 | -0.286842 | -0.364640 | -1.286857 | -0.917753 |
| 0.4635789 | -0.354112 | -0.210948 | -0.180588 | -0.450101 | -0.420922 | 0.3745356 | -0.645833 | -0.689315 |
| -0.756716 | -0.161058 | -0.498519 | -0.579355 | -0.084315 | -0.252524 | -0.423521 | -0.376741 | -0.189536 |
| -1.945665 | 0.0218159 | -0.736882 | -3.105049 | -0.102096 | -1.070595 | -2.449506 | -0.418205 | -0.152159 |
| 0.8755881 | -0.331531 | -0.446088 | -0.301983 | -0.607353 | -0.150756 | -0.015373 | -0.354562 | -0.298155 |
| -1.650907 | -0.485506 | -1.165806 | -1.446494 | -0.622319 | -1.846824 | -1.582761 | -0.229801 | -0.643512 |
| -0.655318 | -1.037828 | -1.336873 | -1.284358 | -1.000413 | -1.559312 | -1.563245 | -0.141572 | -0.424236 |
| -2.125638 | -0.640182 | -1.657479 | -1.867777 | -0.764563 | -1.740009 | -1.675112 | -0.534969 | -0.762341 |
| 0.9732578 | -0.311701 | 0.0503305 | -0.235317 | -0.202803 | 0.1695754 | -0.024432 | -0.154825 | 0.2181179 |
| -0.667352 | -0.138582 | 0.6215867 | -0.023697 | 0.3947518 | 0.2114730 | -0.069962 | -0.143335 | -0.556317 |
| -1.706576 | -0.699632 | -1.208511 | -1.550446 | -0.745687 | -1.399202 | -1.665957 | -0.807625 | -0.922698 |
| -1.622346 | -0.332362 | -1.047227 | -0.911481 | -0.729603 | -0.610464 | -1.286676 | -0.513569 | -0.376835 |
| -1.537322 | -0.007183 | -1.003585 | -1.352690 | 0.0362063 | -1.177631 | -1.267302 | -0.170577 | -0.520712 |
| -0.553197 | -0.265979 | -0.345318 | -0.753196 | -0.393604 | -0.444011 | -0.831783 | -0.613943 | -0.575354 |
| -1.186321 | -0.653202 | -1.120266 | -1.196627 | -0.752007 | -1.433429 | -1.453686 | -0.561477 | -0.943924 |
| 0.4808427 | -0.084047 | -0.175088 | 0.3432105 | -0.153727 | -0.265670 | 0.4756820 | -0.138339 | -0.426913 |
| -2.175483 | -0.602344 | -1.341659 | -0.933033 | -0.919012 | -1.574807 | -1.152249 | -0.929945 | -1.103198 |
| 1.8663250 | -0.006739 | 0.9655071 | 1.6144235 | -0.018519 | 0.9420696 | 1.5737939 | 0.0856855 | 0.5853180 |
| 3.8329326 | -0.071896 | 1.5175390 | 2.8831290 | -0.044813 | 1.9161330 | 3.1880058 | -0.298391 | 0.7406660 |
| -0.474171 | -0.810918 | -1.078710 | -1.333982 | -0.656531 | -0.962799 | -1.205408 | -0.858747 | -0.977868 |
| 0.2398120 | -0.042435 | -0.131579 | 0.4876896 | -0.140277 | -0.015596 | 0.5399125 | 0.0531790 | 0.0936017 |
| -0.368490 | 0.2367757 | 0.4645428 | 1.1796339 | 0.2130919 | 0.6497910 | 1.1174785 | 0.1138320 | 0.2865675 |
| 0.8460122 | 0.9049645 | 1.6680620 | 1.8124244 | 0.9314341 | 1.7039036 | 1.9265363 | 0.2068583 | 0.6694788 |
| -1.968130 | -0.263297 | -1.248928 | -1.596937 | -0.302647 | -0.959593 | -1.594695 | -0.580503 | -0.978086 |
| -1.412633 | 0.4884773 | 0.9665183 | 0.8091111 | 0.4408233 | 1.2427925 | 1.4541187 | -0.107292 | 0.4234435 |
| 0.4574890 | -0.103291 | 0.5247042 | 0.9064760 | -0.124631 | 0.5849852 | 0.8997867 | -0.282187 | 0.0876472 |
| 0.4741004 | -0.315126 | -0.209898 | 0.0036444 | -0.555633 | -0.315235 | -0.100656 | -0.823891 | -0.542166 |
| -2.039345 | 0.0405360 | -0.416652 | -1.159819 | 0.0375825 | -0.609675 | -1.171278 | -0.179131 | -0.466496 |
| -2.020307 | -0.307037 | -2.298558 | -2.097527 | -0.559666 | -2.661550 | -1.384198 | -0.917138 | -0.254008 |
| 0.6722667 | -0.081024 | 0.1751347 | 0.4953473 | 0.0368089 | 0.2240022 | 0.4552211 | -0.290332 | -0.119337 |
| 0.4392184 | -0.336452 | 0.4242468 | 1.1483659 | 0.0535168 | 0.4217246 | 1.1231027 | -0.145848 | 0.5699297 |
| 0.4113658 | 0.0261001 | 0.2660225 | 1.0518134 | 0.0756489 | 0.3579115 | 1.0102015 | -0.166409 | 0.2124085 |
| -0.415094 | -0.239227 | -0.498977 | -0.507983 | -0.262709 | -0.736889 | -0.384251 | -0.258266 | -0.384464 |
| -0.388918 | -0.414805 | -0.163148 | 0.1814504 | -0.407878 | -0.108467 | 0.2106877 | -0.244322 | -0.351134 |
| -1.031844 | -0.323847 | -0.463227 | -0.780650 | -0.446062 | -0.531856 | -0.758643 | -0.470290 | -0.799932 |
| -1.578521 | -0.622718 | -1.535526 | -1.226892 | -0.713810 | -1.749857 | -1.279349 | -0.236732 | -0.962732 |
| -1.673463 | -0.354409 | -0.856108 | -1.656922 | -0.324280 | -1.132890 | -1.506346 | -0.198535 | -1.018432 |
| -1.096266 | -0.031673 | -0.034678 | -0.100495 | -0.129121 | -0.184879 | -0.141850 | -0.248802 | -0.465541 |

| | | | | | | | | |
|---|---|---|---|---|---|---|---|---|
| -1.7200653 | 0.2973432 | 0.1890660 | 1.0865889 | 0.2148522 | 0.3784053 | 0.8925048 | 0.0409501 | -0.6674069 |
| -1.7488235 | -0.6447430 | -0.7403462 | -1.9627129 | -0.5179360 | -0.9651764 | -2.4071677 | -0.3127799 | -0.1056062 |
| -1.2996134 | -0.7114687 | -0.3078402 | -2.5136286 | -0.4428667 | 0.1676794 | -1.8730679 | -0.6986691 | -0.2670096 |
| -1.8279731 | -0.8046228 | -2.3249009 | -3.6880965 | -0.6232096 | -2.8062852 | -4.0073865 | -0.3934542 | -1.4760010 |
| -1.8528193 | -0.7199933 | -1.1877691 | -2.0342034 | -0.6049950 | -1.5412066 | -1.8551124 | -0.8019413 | -1.4633409 |
| -0.8694993 | -0.2464520 | 0.1502300 | 0.0327605 | -0.1303848 | -0.1808390 | 0.2127198 | -0.4461600 | 0.1766972 |
| 0.6006001 | -0.0449314 | 1.4968373 | 1.0585031 | 0.1059589 | 1.4245113 | 0.6534046 | -0.2447275 | 0.7531860 |
| 0.1445115 | -0.4068708 | -0.5679961 | -0.4307661 | -0.7115170 | -0.2477410 | -0.1124289 | -0.4642914 | 0.0440611 |
| -2.7938197 | 0.1481836 | -0.6388739 | -1.1970759 | 0.0312839 | -0.7883544 | -0.8764194 | -0.2520134 | -0.3680946 |
| -1.6050245 | -0.2219828 | -1.1035441 | -1.1838781 | -0.2665547 | -0.8575551 | -1.3463834 | -0.8173531 | -1.0686243 |
| -0.6683538 | -1.1568983 | -0.6570337 | -0.0807892 | -1.5731676 | -0.7546508 | -0.2026169 | -0.4443331 | -0.7874183 |
| -0.4880042 | -0.8253246 | -1.2434597 | -1.2153835 | -1.1360316 | -1.1862561 | -1.0946848 | -0.8265331 | -1.4325909 |
| -2.5034488 | 0.2053273 | -0.0734197 | -0.8196431 | -0.1675243 | -0.2411570 | -1.5740672 | 0.0570518 | -0.0254276 |
| -0.8582467 | 0.2728396 | 0.0820834 | 0.3396806 | 0.0494608 | -0.0905075 | 0.2828327 | -0.4187155 | -0.4187302 |
| -2.4902276 | -0.4235849 | -0.7943311 | -1.9270081 | -0.3558132 | -1.2416787 | -2.0570625 | -0.2535146 | -0.5112038 |
| -0.4204670 | 0.0519870 | -0.2179244 | -0.6776989 | -0.0783557 | -0.3067316 | -0.6701065 | -0.1401585 | -0.6570966 |
| -3.6628969 | -0.0167394 | -0.5571357 | -1.2320341 | -0.1606762 | -0.7231762 | -1.2488708 | -0.3508702 | -0.3544603 |
| -0.6199771 | -0.5062948 | -0.8998127 | -1.9465420 | -0.4801560 | -0.8927348 | -1.5580410 | -0.6913100 | -0.6737381 |
| -2.2772009 | -0.0040461 | -1.1290293 | -1.5700126 | -0.0735885 | -1.4626079 | -1.5337666 | 0.1098354 | -0.4798913 |
| -1.2246327 | 0.2757473 | 0.3354940 | 0.9227860 | 0.4382463 | 0.7986889 | 0.5612256 | 0.1001252 | 0.1593582 |
| -2.2472676 | -0.3868100 | -1.4220931 | -2.0195081 | -0.5222083 | -1.1712893 | -2.1848775 | -0.9470578 | -0.3677728 |
| -0.4507290 | -0.3385636 | -0.1701048 | -0.4282646 | -0.2585012 | -0.1410739 | -0.3194409 | -0.2888100 | -0.2362725 |
| -2.9346156 | -0.6337212 | -2.4419607 | -3.4549171 | -0.7492637 | -2.9047329 | -3.7324666 | -0.3910425 | -1.1725447 |
| -0.4206151 | -0.0757758 | 0.0732697 | 0.7565085 | -0.2149500 | 0.0070524 | 0.7840729 | -0.3186667 | -0.2776766 |
| -1.2866959 | -0.1990982 | -0.6477699 | -0.9005074 | -0.4636173 | -0.8053517 | -1.2216942 | -0.1549175 | -0.4378363 |
| 0.3376566 | -0.2045452 | -0.2562837 | 0.2286624 | -0.5182227 | -0.4576217 | 0.0521639 | -0.4556662 | -0.4953809 |
| -2.0337392 | 0.0194410 | -0.4098360 | -2.3612820 | 0.0225467 | -0.5663938 | -1.9880339 | -0.1264284 | -0.1490390 |
| -0.5157361 | -0.7476334 | -0.8943520 | -0.5884779 | -0.7165567 | -0.8440965 | -0.4611913 | -0.3489993 | -0.3839823 |
| 1.3705871 | -0.1352453 | 0.4958621 | 0.7462969 | 0.0571840 | 0.3450115 | 0.6555245 | -0.1265451 | -0.0583676 |
| 0.6903160 | 0.1368616 | 0.2123508 | 2.0645791 | -0.1185683 | 0.3201506 | 1.8461952 | -0.2917621 | -0.1226484 |
| -0.6585274 | -0.6330837 | -0.7570119 | -1.2497832 | -0.9168316 | -1.0237968 | -1.3238715 | -0.5834584 | -0.6290086 |
| -1.7273181 | 0.1847958 | -0.2535073 | -1.0830053 | 0.0889675 | -0.2868275 | -0.9560814 | -0.3375579 | -0.3189454 |
| -0.4156325 | -0.4912967 | -0.2729111 | -0.3178667 | -0.5070485 | -0.3162530 | -0.8078134 | -1.1836917 | -0.6683497 |
| -1.3637870 | -0.1619549 | -0.4101667 | -0.7551522 | -0.0492413 | -0.5620391 | -0.8422314 | -0.2585772 | -0.2749597 |
| -1.3522388 | -0.1270641 | -0.2677040 | -0.1067887 | -0.0138453 | 0.1284058 | -0.1348657 | -0.0072652 | -0.0350459 |
| -2.8699614 | -0.1098494 | -0.6314411 | -1.2557167 | -0.2537979 | -0.9739763 | -1.2784333 | -0.5351533 | -1.0116784 |
| -1.9674259 | -0.2602989 | -1.4449151 | -1.5601545 | -0.4839807 | -1.3401833 | -1.8185992 | -0.6413871 | -2.0516050 |
| -0.2247118 | -0.1502567 | -0.1589581 | -0.1938196 | -0.1641748 | -0.2131378 | -0.2805880 | -0.7080108 | -0.4413598 |
| -0.0415129 | -0.3351430 | 0.5508575 | 0.2176735 | -0.2949207 | 0.5220052 | 0.0098845 | -0.2578473 | -0.1561639 |
| -1.8635770 | 0.0759143 | -0.2275631 | -0.4224126 | 0.1083216 | -0.1954286 | -0.7424419 | -0.3658758 | -0.2604467 |
| -0.7748582 | -0.1152257 | 0.7319409 | 0.7013160 | 0.0870595 | 0.7968742 | 0.8567697 | -0.4021242 | -0.2593075 |
| -2.3620214 | -0.5243902 | -0.5948706 | -1.6975108 | -0.5718355 | -0.5938753 | -1.4527336 | -0.3065453 | -1.0147442 |
| -0.4244254 | -0.3246504 | -0.9521862 | -0.9113336 | -0.7714441 | -1.1305205 | -0.9476537 | -0.2670690 | -1.2120843 |
| -0.8377051 | -0.0885702 | -0.3627871 | -0.3498899 | -0.2383197 | -0.4650714 | -0.3601800 | -0.6471165 | -0.5841484 |
| -0.5076808 | -0.8991171 | -1.0648002 | -0.9752909 | -0.9403409 | -0.9412545 | -0.9339977 | -0.6380123 | -0.2219577 |
| -2.4558452 | -0.6500396 | -0.6980459 | -0.7589062 | -0.7158308 | -0.7604441 | -0.8702035 | -0.4194569 | -0.9947333 |
| -1.1742970 | -0.1993711 | -0.6804848 | -0.5959908 | -0.1923190 | -0.5242154 | -0.5200592 | -0.3611905 | -0.2939863 |
| -1.7780029 | -1.2443499 | -1.1496250 | -1.0882145 | -1.4619794 | -1.0205907 | -0.7333740 | -0.5692160 | -0.9081054 |
| 0.6216561 | -0.3281982 | 0.1020617 | 0.6521982 | -0.2065834 | 0.3185253 | 1.1784843 | -0.4305922 | -0.1420026 |
| -1.6826592 | -1.0350091 | -1.5526821 | -1.2551595 | -1.0765294 | -1.4411465 | -1.1662823 | -0.9917796 | -0.9845872 |

| | | | | | | | | |
|---|---|---|---|---|---|---|---|---|
| 1.0735534 | -1.034599 | -0.311126 | 0.7027278 | -0.984505 | 0.0174330 | 0.9093187 | -0.370337 | -0.177539 |
| 0.3721530 | -0.161579 | -0.294598 | 0.3397950 | 0.0868018 | -0.056626 | 0.5933099 | -0.537365 | -0.292185 |
| -0.487106 | -0.214214 | -0.035266 | -0.040234 | -0.186213 | -0.071540 | 0.1425827 | -0.315801 | -0.085511 |

| PG_435 | PG_436 | PG_437 | PG_438 | PG_439 | PG_440 | PG_441 | PG_442 | PG_443 |
|---|---|---|---|---|---|---|---|---|
| -2.3417534 | -0.6688603 | -1.0226717 | -2.2577444 | -0.6899872 | -1.2512427 | -0.6271349 | -1.4454666 | -1.1455848 |
| -1.1278673 | -0.7441317 | -0.9021322 | -1.1514839 | -0.4008689 | -1.9721661 | -1.5801309 | -1.3790022 | -2.3157076 |
| -1.2161294 | -0.4011570 | -0.1256791 | -0.9038347 | -0.5215816 | 0.1728640 | -1.2928772 | 0.4596831 | -0.0516170 |
| 0.7171981 | -0.2834667 | -0.7883984 | 0.6179368 | 0.5167408 | -3.0839325 | -1.6238320 | -2.4720273 | -3.8472189 |
| -0.5520095 | 0.0424234 | 0.1077704 | -0.255214 | 0.1634874 | -1.9117049 | -1.7158009 | -2.2950917 | -1.9195631 |
| -1.0681302 | -0.3021065 | -1.0855312 | -1.3950780 | -0.3280700 | -1.3947008 | -1.8801686 | -1.1653701 | -2.9325253 |
| -0.7809575 | -0.1039264 | -0.9844076 | -0.3516016 | -0.2198266 | -1.6941097 | -0.4342986 | -1.2333703 | -1.4680341 |
| -1.2460788 | -0.4364071 | -0.4398582 | -1.5650242 | -0.1575081 | -1.1242846 | -1.7687989 | -0.9247988 | -1.152037 |
| 0.3015038 | -0.5028225 | -0.2809964 | 0.4457287 | -0.3617849 | -0.2312313 | -0.4629868 | -0.4768784 | -0.2323259 |
| -0.1925206 | -0.5634225 | -0.5443394 | 0.1312512 | 0.5712887 | -0.6566723 | -1.0922393 | 0.5797404 | -1.2902596 |
| -0.8120554 | -0.1871514 | -0.5375693 | -0.8973158 | -0.0645364 | -1.5433000 | -1.3391946 | -1.3434211 | -2.1457335 |
| -0.3248119 | -0.2565544 | -0.6798614 | -0.4067176 | 0.0081880 | -2.1916738 | -1.1254670 | -1.5887320 | -2.6268869 |
| -3.5880336 | -0.6046186 | -0.9258383 | -3.7324173 | -0.3444444 | -2.0280817 | -1.8228781 | -1.2013573 | -3.4203863 |
| 4.8305899 | -0.8216106 | 3.4625863 | 5.3009132 | -0.2977861 | 0.7206781 | 4.0128203 | -0.1331751 | 2.5253676 |
| 0.1094367 | -0.2341802 | -0.4992027 | 0.7334040 | -0.0715112 | -2.7999769 | -1.6600473 | -2.5601041 | -2.8728080 |
| 1.9129581 | -0.0849696 | 2.0428609 | 2.2860605 | -0.5410362 | 0.4137558 | 1.8120778 | -0.2401431 | 1.7873696 |
| -2.8735370 | -1.8056544 | -2.6138899 | -2.7964864 | -0.6220968 | -2.0831385 | -1.2866729 | -0.5890292 | -2.2987120 |
| -0.5177841 | 0.0236022 | -0.5761278 | -0.4396817 | 0.5093801 | -0.4488011 | -0.7320175 | 1.1135048 | -0.7289252 |
| -0.4120504 | -0.4993237 | -0.5753690 | -0.2154150 | -0.7771351 | -0.5671581 | -0.3496483 | -0.5377078 | -0.0983674 |
| -0.7009036 | -0.7033162 | -0.5711597 | -0.6333457 | -1.4023788 | -0.8648416 | -1.2585837 | -1.3642729 | -0.1376053 |
| 0.6368254 | -0.3614677 | 0.2004552 | 0.3525802 | -0.3352385 | -1.5582489 | 0.0508042 | -1.5020068 | -1.2854802 |
| -0.2796444 | -0.4046511 | -0.1583026 | -0.5814302 | -0.4725771 | -0.2152527 | -0.3670319 | -0.4763539 | -0.0965903 |
| 1.4109705 | -0.3327733 | 0.4182677 | 1.3152908 | -0.3804158 | 1.1750340 | 2.2515520 | 0.3304614 | 1.4348195 |
| -2.0216804 | -0.5053465 | -1.9105425 | -1.7818461 | -0.4327137 | -1.6061022 | -1.8982082 | -1.2263124 | -2.0123235 |
| -1.4815030 | -0.3045170 | -1.3529457 | -2.0324194 | -0.4067508 | -1.4742626 | -3.0547340 | -1.2353028 | -2.2629950 |
| 1.3957006 | -0.3741438 | 0.2604825 | 1.0675620 | -0.2116242 | 0.7110726 | 1.2504931 | 0.3954781 | 0.8130078 |
| -0.6020402 | 0.2429405 | -0.0679770 | -0.5337320 | 0.2933986 | -2.3627778 | -1.7884112 | -2.3345847 | -2.8138885 |
| -1.4280389 | -0.8563129 | -1.6698003 | -1.3281548 | -0.7708324 | -1.8746297 | -2.6013654 | -2.0235742 | -1.9806275 |
| -2.0463012 | -0.1069807 | -1.4726741 | -0.9148224 | 0.1636159 | -0.4881497 | -1.7452004 | -0.4678572 | -0.9768905 |
| -0.2621951 | -0.2714573 | -0.7385547 | -0.1302612 | 0.1797073 | -1.7836367 | -1.6915019 | -1.4964360 | -2.2520790 |
| -1.8857871 | -0.0314462 | -0.7541413 | -1.6488864 | -0.4102554 | -0.6694622 | -0.4910208 | -0.5560568 | -0.4295678 |
| -0.5750354 | 0.2478643 | 0.0791027 | 1.2247070 | -0.1178896 | -0.0841636 | 0.1355916 | -0.2044065 | 0.3132296 |
| -0.8522409 | -0.1478315 | 0.3476977 | -0.8833536 | -0.2798723 | -1.6961539 | -0.6833207 | -1.1600359 | -1.5715081 |
| -4.2006254 | -0.5554056 | -1.1088161 | -4.3368114 | -0.3958145 | -0.6122154 | -2.8421631 | -0.3970299 | -1.0711605 |
| 0.0716651 | -0.0720270 | -0.0763398 | 0.0844503 | -0.0340502 | 0.0218160 | -0.0555041 | -0.0328838 | 0.0236359 |
| -1.2275718 | -0.7427337 | -0.9993375 | -1.0919519 | 0.0191777 | -1.7447195 | -0.4696737 | -0.9102963 | -2.5447629 |
| -2.3962954 | -0.2184811 | -0.6620399 | -1.8663548 | -0.2478682 | -0.6827144 | -2.0257060 | -0.6705746 | -1.1269775 |
| 1.6980580 | 0.5395629 | 2.6373959 | 1.0082098 | -0.4924484 | 2.6288217 | -0.3377167 | 1.5779946 | 3.4973379 |
| 0.0628125 | -0.5483028 | -0.6098169 | 0.7980926 | -0.4539104 | -2.0088798 | -0.8116377 | -1.1491264 | -1.7995266 |
| -1.7254843 | -0.7541140 | -1.3573365 | -1.0550624 | 0.2194682 | -1.8179089 | 0.0060535 | -1.4088026 | -1.5968453 |
| 1.1548413 | -0.0488114 | 0.2344177 | 1.4145509 | -0.4383591 | 0.4280028 | 2.2151930 | 0.5864191 | 0.9547618 |
| -2.8352302 | -0.6373059 | -1.1492581 | -2.7693903 | -0.5765266 | 0.0124403 | -2.5412240 | -0.2485478 | -0.5645598 |
| -0.5492239 | -0.9415844 | -0.7351582 | -0.6386841 | 0.0431276 | -1.5580221 | -1.3991204 | -1.3513842 | -2.0099409 |
| -0.3867515 | -0.0640336 | -0.2839785 | -0.5413964 | 0.1937774 | -2.6059530 | -3.1412542 | -1.9724948 | -2.8434003 |
| 1.5334514 | -0.1190443 | -0.3950429 | 1.4853599 | -0.0100344 | 1.5281276 | 2.3423725 | 0.9921236 | 1.4758200 |
| -0.2615256 | -0.2604172 | -0.4890154 | 1.7498789 | -0.8707888 | -0.7098398 | -0.0144682 | -0.6064949 | -0.6149265 |
| 0.0199881 | 0.1551220 | -0.1445376 | 0.0285741 | -0.5671766 | 0.5087053 | 0.2008700 | 0.3362833 | 0.4343977 |

| | | | | | | | | |
|---|---|---|---|---|---|---|---|---|
| 0.3371419 | -0.1197554 | 0.0587753 | 0.2583750 | -0.3751094 | -1.8668515 | -0.7935145 | -1.5567704 | -1.7461450 |
| -2.4898286 | -0.8126267 | -1.0741935 | -1.3603124 | -0.4327666 | -1.5843757 | -1.8522633 | -1.2987863 | -1.5877100 |
| 0.3505853 | -0.6012062 | -0.0376618 | -0.0827120 | -0.5907581 | -1.5237628 | -0.2596122 | -1.1851193 | -1.4767264 |
| -0.1217868 | -0.0628682 | -0.1875833 | -0.1950249 | -0.1762655 | 1.2376820 | 3.8391057 | 0.5820480 | 3.9256752 |
| -0.1343836 | -0.2282606 | -0.4179529 | -0.3084466 | -0.4128925 | -0.1416089 | -0.0757822 | -0.6480849 | -0.2608110 |
| 0.0561129 | -0.2565806 | -0.2292347 | 0.0801831 | -0.2145084 | 0.4379687 | 0.3670361 | 0.4558430 | 0.5817914 |
| -1.6519700 | -0.4269931 | -0.7470040 | -1.6372986 | -0.5666639 | -0.8691519 | -0.9880852 | -1.3573993 | -1.8327394 |
| 3.1388158 | -0.5216444 | 1.8440785 | 3.0915330 | -0.6993999 | 0.5197807 | 1.4625968 | 0.0766077 | 0.6881979 |
| 1.8504493 | -1.2702499 | 3.3726849 | 1.2105934 | 2.1715489 | 1.3249121 | -2.1264655 | 2.1576724 | 2.0228466 |
| -0.4403975 | -0.9327430 | -0.5758049 | -0.5984984 | -0.6099616 | -1.2309548 | -0.4825214 | -1.4902830 | -1.0057640 |
| -1.7521158 | -0.6859293 | -0.8041335 | -1.8477834 | -0.6465121 | -2.5574437 | -2.0643962 | -1.8966422 | -2.2439631 |
| 3.2498399 | 0.2040656 | 3.2428610 | 3.5599034 | -0.1693437 | 0.5296546 | 2.6052377 | -0.5201329 | 2.4173201 |
| 1.8634762 | -0.5660876 | 2.7492766 | 2.3822425 | -0.2070143 | -1.1923936 | 0.8111659 | -1.4826616 | 0.0584711 |
| -2.0093087 | -0.6505495 | -1.7061465 | -1.0735913 | -0.2710219 | -0.8696578 | -1.2032354 | -1.0973499 | -1.2014707 |
| -2.1710515 | -0.9105826 | -2.0937124 | -2.2885438 | -0.5098502 | -2.0508907 | -1.6022186 | -1.4727580 | -2.3376606 |
| -0.7365763 | -0.3897313 | -1.5126185 | -0.9376057 | -0.6294778 | -1.5206538 | -1.4083337 | -0.9023485 | -1.7563355 |
| -1.9529034 | -0.2001782 | -0.8676421 | -2.0950915 | -0.2494877 | -1.4798201 | -2.6748341 | -1.2131862 | -2.1532076 |
| -0.8934435 | -0.1448344 | -0.6827548 | -0.8889004 | -0.2398667 | -0.8657552 | -1.0737787 | -0.7876161 | -1.2422330 |
| -1.2630699 | -0.2937506 | -0.7399833 | -1.2145569 | -0.4491120 | -2.1661089 | -2.1937058 | -1.5132132 | -2.3925473 |
| 0.3255351 | 0.2563245 | 0.2780691 | 0.1353391 | 0.5176804 | -4.0154627 | -4.0766746 | -3.6208318 | -3.6801169 |
| -0.1600109 | -0.4052543 | -0.1090840 | 0.0298176 | -0.7641836 | -0.7150758 | 0.0926564 | -0.6626219 | -0.7478545 |
| -3.0473255 | -0.5123450 | -1.2199778 | -2.5219841 | -0.4910561 | -0.7221568 | -2.0361583 | -1.1329562 | -1.5707459 |
| 0.2505874 | -0.0075896 | -0.3693184 | -0.3240436 | -0.4569453 | 1.0761845 | 1.0188427 | 0.9674136 | 0.9146570 |
| 1.1095416 | 0.6485185 | 0.5392545 | 0.8139782 | 1.1787939 | -3.5424913 | -3.1252352 | -3.3889041 | -3.5987590 |
| -1.2122648 | -0.9058761 | -1.6253951 | -2.5663595 | -0.3034175 | -1.5140615 | -2.4226961 | -1.2740982 | -2.2945577 |
| -0.5341500 | -0.3556885 | -0.3993136 | -0.7117556 | -0.1029051 | -1.1416142 | -0.4226444 | -1.4146602 | -1.6718678 |
| -1.5963083 | -0.5439280 | -2.1245957 | -1.5329945 | -0.6187063 | -0.6890875 | -2.2968070 | 0.1654357 | -1.5775958 |
| 4.1276193 | -0.4010688 | 0.5030357 | 3.5717947 | -0.1785673 | 0.8251687 | 4.3322088 | 0.2920402 | 1.2748216 |
| 4.4546340 | 0.5741858 | 2.6389522 | 4.7805819 | -1.6363970 | -0.6811639 | 2.3189760 | -1.8179525 | 0.2442534 |
| -1.3565369 | -0.3105028 | -0.9175267 | -1.2022394 | 0.0890368 | -0.6491364 | -1.7685619 | -0.3863148 | -0.6954797 |
| 0.1653956 | 0.0380178 | 0.1093964 | 0.2500720 | -0.2438188 | -0.6513073 | -1.3581186 | -0.7396555 | -1.3970834 |
| -0.1884949 | -0.1776358 | -0.3081707 | -0.1367855 | 0.2476530 | -4.2170207 | -1.8189208 | -2.4653758 | -3.8182358 |
| -1.5738843 | -0.2606359 | -1.5297298 | -1.3892254 | -0.6199423 | -1.6974150 | -1.5519622 | -1.1708717 | -1.8663674 |
| -1.2458573 | -0.2461120 | -0.5915667 | -1.5402024 | -0.3470815 | -2.0072678 | -2.2537198 | -1.8662968 | -2.8475689 |
| 1.7742120 | -0.3160731 | 0.5284117 | 1.8229322 | -0.4075926 | 0.9988412 | 2.1109268 | 0.0145105 | 1.1847092 |
| -0.3937666 | -0.3019632 | 0.1025179 | 0.6663130 | -0.1272222 | -0.7438356 | -0.9592396 | -0.4309730 | -0.7550484 |
| -1.5165055 | -0.2568887 | -0.6918495 | -1.0740786 | -0.2330711 | -0.0879553 | -0.1304011 | -0.1142674 | -0.1765694 |
| -1.3447043 | -0.3033800 | -1.3613867 | -1.1832870 | -0.1585795 | -0.8319085 | -1.6595377 | -0.4760272 | -1.3955461 |
| -1.5268548 | -0.2881066 | -1.1554882 | -1.4773603 | -0.1276338 | 0.3414460 | -1.6244820 | 0.3140630 | -0.0116698 |
| -3.1717347 | -0.6129350 | -1.8778367 | -2.9443496 | -0.5985527 | -0.3635188 | -1.6994671 | -0.1381193 | -0.4067564 |
| -0.6690923 | -0.8238616 | -0.6407460 | -0.6853552 | -1.0574224 | -1.5923182 | -1.5459758 | -1.5643124 | -1.8308952 |
| -0.4146564 | -0.8056973 | -0.5904147 | -0.6565036 | -1.4541484 | -1.2782888 | -1.5629321 | -1.4207696 | -1.3245988 |
| 3.5877062 | 0.5225883 | 3.5728831 | 3.2243705 | -0.5069379 | 0.9838807 | 1.4700950 | 0.3157635 | 2.8078517 |
| -1.3929602 | -0.0856625 | -0.1580890 | -1.3889165 | 0.0784343 | -1.2191884 | -0.5029204 | -1.1091590 | -1.7512886 |
| -1.3355409 | 0.0476244 | -0.2294689 | -1.1354016 | 0.0459565 | -2.1876748 | -2.3510145 | -2.0553629 | -1.8167050 |
| -0.3874499 | -0.5445045 | -0.4220481 | -0.3159991 | -0.2660167 | -0.4829898 | -0.4421779 | -0.4349183 | -0.6245350 |
| 7.1178276 | 0.1860495 | 7.5586540 | 8.4259983 | -0.0884464 | 1.4459570 | 5.7868507 | 0.1777618 | 5.9934564 |
| 2.1491975 | 0.3020739 | 1.7969183 | 2.4702749 | -0.0111059 | -0.5947649 | 1.5514041 | -1.0265141 | 0.7876053 |
| -4.5147293 | -1.3180536 | -0.3338520 | -2.9628458 | 0.3741290 | -1.4855168 | -2.5812943 | -0.6203605 | -0.8522355 |
| 0.6074621 | -0.5823974 | -0.3808259 | 0.4410074 | -0.1836354 | -0.3474158 | -0.8578104 | -0.6368581 | -0.6458533 |

| | | | | | | | | |
|---|---|---|---|---|---|---|---|---|
| -0.511873 | -0.250001 | -0.563420 | -0.189293 | 0.0393605 | -1.831275 | -2.017150 | -1.128210 | -2.154774 |
| -0.137487 | -0.115013 | -0.588080 | -0.179698 | -0.396838 | -0.506067 | 0.0881278 | -0.462236 | -1.482562 |
| -0.080431 | -0.634256 | -1.646357 | -0.599560 | -0.205515 | -0.478093 | -0.314554 | -0.290195 | -0.883211 |
| -0.372048 | -0.035367 | 0.0794070 | -0.151353 | -0.182413 | -1.071647 | 0.0657264 | -0.771960 | -1.012045 |
| -1.446382 | -0.064377 | -1.259268 | -1.352889 | -0.390440 | -2.190062 | -2.271037 | -1.305784 | -2.767868 |
| -1.557622 | -0.894493 | -0.275803 | -0.517071 | 1.3137588 | -0.592227 | -0.501516 | 0.2672672 | -1.212183 |
| -0.414578 | -0.582476 | -0.038427 | -0.311383 | -0.152980 | -2.479681 | -0.930681 | -2.224732 | -1.429194 |
| 0.2171814 | 0.0117304 | -0.126960 | 0.1406451 | -0.152474 | -0.224830 | 0.1496089 | 0.3076569 | 0.0487344 |
| -0.209494 | 0.0067156 | -0.295645 | -0.286710 | -0.322435 | 0.4032674 | 0.1208088 | 0.3343266 | 0.7461644 |
| 1.2262246 | -0.111454 | 0.3036752 | 1.1433933 | -0.302671 | -0.054106 | 1.0321788 | -0.419749 | -0.014917 |
| -1.436061 | -0.394765 | -1.169285 | -0.720469 | -0.001313 | -0.984454 | -1.690059 | -1.123781 | -1.335945 |
| -2.336227 | -0.386309 | -0.696856 | -2.394499 | -0.253178 | -3.021792 | -3.181083 | -2.100915 | -3.404593 |
| -4.268823 | -0.229591 | -1.012227 | -3.558600 | -0.184973 | -0.177988 | -3.044139 | -0.160854 | -1.780391 |
| -2.186950 | -0.460281 | -0.547354 | -3.144025 | -0.606044 | -1.458352 | -1.846642 | -0.998780 | -2.055531 |
| -0.340882 | -0.370894 | -1.246317 | -0.631519 | -0.129470 | -1.249289 | -1.138733 | -0.887414 | -1.656485 |
| 0.1322706 | -0.020729 | -0.162009 | -0.222228 | -0.254671 | 0.0332404 | -0.024931 | -0.106608 | -0.025192 |
| -0.836007 | -1.042903 | -1.276543 | -0.847463 | -0.790606 | -1.536749 | -1.108656 | -1.337883 | -2.113801 |
| 0.1317809 | -0.221378 | -0.440349 | 0.4332919 | -0.390260 | -0.206756 | 0.0088294 | 0.0473288 | 0.1134632 |
| -0.661032 | -0.467496 | -0.602242 | -0.577161 | -0.480160 | -0.985151 | -0.673919 | -1.104278 | -1.558706 |
| 0.3880699 | -1.218475 | -0.156612 | 0.3674659 | -0.885617 | 0.9135166 | 1.9898116 | 0.7871520 | 1.3725629 |
| -0.477756 | -0.335763 | -0.783746 | -0.323312 | -0.062278 | -0.793545 | -1.277901 | -0.391564 | -0.736718 |
| -0.283474 | -0.532921 | -0.766226 | -0.331411 | -0.543105 | -1.681980 | -1.646608 | -1.108903 | -1.322160 |
| -0.375527 | -0.208968 | 0.0452666 | -0.443167 | -0.365719 | -0.088859 | -0.135350 | -0.203637 | 0.1336611 |
| -1.237663 | -0.242663 | -0.431789 | -1.397576 | -0.530768 | -1.507192 | -1.163866 | -1.218905 | -1.563012 |
| -0.202105 | -0.138796 | -0.415961 | -0.331562 | -0.363393 | -0.450253 | -0.296203 | -0.270835 | 0.0720491 |
| -1.132185 | -0.423180 | -0.977926 | -1.358149 | -0.656120 | -1.524730 | -1.064652 | -1.006297 | -2.471906 |
| 0.1790564 | 0.1011896 | -0.546340 | 0.1138751 | -0.004226 | -1.376369 | 0.0687765 | -0.946315 | -1.574964 |
| 0.3099078 | -0.611978 | -0.230626 | 0.1683171 | -0.754289 | 0.6894557 | 0.6320909 | 0.2471998 | 0.6932661 |
| -0.286703 | -0.307354 | -0.391389 | -0.319527 | -0.179908 | -0.551033 | -1.357226 | -0.328257 | -1.152032 |
| -2.128564 | -0.084017 | 0.1295170 | -2.872264 | -0.459000 | -1.862342 | -0.368924 | -1.126496 | -1.502162 |
| -0.672452 | 0.0782617 | -0.169154 | 0.0667048 | -0.536403 | -0.590651 | -1.473048 | -0.676775 | -0.661399 |
| -0.485399 | -0.199964 | -0.558639 | -0.285575 | -0.177572 | -1.606227 | -2.145266 | -1.558880 | -2.322822 |
| 0.3529077 | -0.405532 | -0.354632 | 0.7856244 | -0.771700 | -1.143394 | -0.745593 | -1.077813 | -0.725567 |
| 0.4451477 | 0.3298709 | 0.2202374 | 0.3106592 | 0.0816567 | -0.913396 | -0.742465 | -0.069645 | -0.871484 |
| -1.547468 | -0.349652 | -0.760528 | -1.944609 | -0.607442 | -0.634163 | -2.503104 | -0.203467 | -0.733182 |
| -1.069872 | -0.303107 | -1.031716 | -1.465248 | -0.623132 | -1.908277 | -1.743389 | -1.301454 | -3.190604 |
| -0.246205 | -0.110128 | -0.522305 | -0.268116 | -0.445718 | -1.635095 | -0.139180 | -1.106347 | -1.345139 |
| -3.145299 | -0.228489 | -0.516665 | -2.745400 | 0.0198231 | -3.340359 | -1.862855 | -2.134959 | -3.331158 |
| -0.275044 | -0.516964 | -0.877906 | -0.681484 | -0.323881 | -0.710659 | -0.604651 | -0.558984 | -0.803198 |
| -3.363640 | -1.006013 | -1.132916 | -3.404125 | -0.527235 | -0.965845 | -0.906776 | -0.532975 | -1.505206 |
| -0.249642 | -0.457430 | 0.1810292 | -0.100109 | -0.409026 | -1.521693 | 0.6539644 | -1.875683 | -1.026855 |
| -1.573553 | 0.0618607 | 0.0080508 | -2.207333 | 0.0204728 | -1.805214 | -1.162069 | -1.319094 | -1.765993 |
| -0.442289 | 0.0702651 | -0.094287 | -0.395268 | -0.156102 | -2.283315 | -1.242570 | -1.413032 | -2.506541 |
| -1.686842 | -0.678131 | -0.845299 | -1.642368 | -0.363065 | -1.587296 | -1.362464 | -0.735131 | -1.657830 |
| -0.227798 | -0.270874 | -0.225691 | 0.3614462 | -0.239268 | 0.0718767 | -0.245389 | -0.016824 | -0.086845 |
| 0.6483217 | -0.256436 | 0.5538330 | 0.6728643 | -0.188152 | -0.787226 | -0.272076 | -1.104852 | -0.913075 |
| -1.162983 | -0.561565 | -1.064668 | -1.297233 | -0.451580 | -1.380542 | -1.025588 | -1.227902 | -1.180739 |
| 0.6806688 | -0.770491 | 0.0893732 | 0.7251136 | -1.046613 | -1.244212 | -0.322985 | -1.321941 | -1.336082 |
| -2.625010 | -0.612440 | -1.669867 | -2.775573 | -0.616462 | -1.157282 | -2.184497 | -1.253541 | -1.990014 |
| -2.236548 | -1.155434 | -1.431457 | -2.409916 | -0.912219 | -1.740674 | -1.654288 | -1.395657 | -1.631476 |

| | | | | | | | | |
|---|---|---|---|---|---|---|---|---|
| -0.8708299 | 0.2473789 | -0.0202909 | -0.9600735 | -0.0203934 | -2.0709134 | -0.7739900 | -1.9078067 | -2.2838977 |
| -3.1037908 | -0.8761646 | -1.7201073 | -3.4368793 | -1.0452137 | -2.3006239 | -2.2618309 | -2.2460274 | -2.5019583 |
| 0.1639151 | -0.3726107 | -0.6354049 | -0.5583437 | -0.4712373 | -1.0480614 | -0.1816125 | -0.8787594 | -1.7367344 |
| 0.7355509 | -1.0709424 | 0.5582476 | 0.4841851 | -1.0644077 | 0.4226643 | 1.3912975 | -0.3010286 | 1.1765377 |
| -2.1592765 | -0.4497805 | -1.2503685 | -1.7238769 | -0.4934591 | -2.0287101 | -2.4508089 | -1.5499654 | -1.1397105 |
| 3.3736001 | 0.6538437 | 2.3760118 | 3.2799611 | -0.5230626 | 2.0482986 | 1.4063962 | 0.1097718 | 2.6132763 |
| -0.9148578 | -0.3136691 | -0.6070878 | -0.8129269 | -0.4229074 | -1.7109733 | -1.0132457 | -0.8957922 | -2.1645961 |
| -1.0235028 | -0.7754150 | -0.8501114 | -1.3290530 | -0.7237051 | -0.5791268 | -0.9785857 | -0.6173819 | -1.4675876 |
| -1.6783656 | -0.2051828 | -1.4831475 | -1.5766842 | -0.5683457 | -0.6513225 | -1.9997520 | -0.8105289 | -1.6040476 |
| -0.4531763 | -0.6958649 | -1.0570552 | -0.4242998 | -0.4743147 | -1.4121251 | 0.3689203 | -0.9761551 | -1.4200502 |
| -0.2348898 | -0.0386653 | -0.0549432 | -0.2216597 | -0.0505737 | -0.7108198 | 0.2662745 | -0.7313695 | -0.8417174 |
| -0.2748005 | -0.5982982 | -1.2596713 | -0.2750801 | -0.7668353 | -0.9022316 | -1.6529915 | -0.9902246 | -1.6074790 |
| -1.7158032 | -1.3881764 | -1.6880849 | -1.1241404 | 0.1126504 | -0.7081559 | -1.7385130 | -0.6493419 | -1.5803181 |
| -0.2025111 | -0.7320226 | -1.1298717 | 0.1442271 | -0.3733031 | -1.3457814 | -0.8683532 | -0.8409872 | -0.8105751 |
| -0.5987313 | -0.5391780 | -0.7996067 | -0.6228886 | -0.1497371 | -0.2319175 | -0.0743189 | -0.5244171 | -0.3831102 |
| -0.5304556 | -0.4312556 | -0.3834276 | -0.2467109 | -0.4423723 | -0.8728206 | -0.9559388 | -0.4303066 | -1.2199044 |
| -1.0719583 | -0.8490578 | -0.7051768 | -0.7083748 | -0.8046045 | 1.2924641 | 0.6333971 | 0.8198129 | 0.7805751 |
| -0.9542444 | -0.4512647 | -1.2254650 | -1.6914552 | -0.1942592 | -1.1890876 | -2.0914529 | -1.1301412 | -3.1985830 |
| -0.3136816 | -0.4683063 | -0.6460926 | -0.2832176 | -0.4464726 | -1.1591231 | -0.8451275 | -1.1084880 | -0.8913526 |
| -1.1639129 | -0.5018067 | -1.4768665 | -1.0636701 | -0.4764317 | -2.4177204 | -1.7407072 | -1.9595376 | -2.8829510 |
| 1.5970381 | -0.2781052 | 0.3420696 | 1.5668153 | -0.2546303 | 0.2937690 | 0.1766842 | 0.1823036 | 0.3206888 |
| -0.0686897 | 0.0498592 | -0.2516222 | -0.3381367 | -0.2010105 | -1.4011390 | -0.9582530 | -1.8746205 | -1.1588801 |
| -1.7842217 | -0.6725590 | -1.4289288 | -1.9441777 | -0.5259085 | -0.9691748 | -1.6569467 | -0.7806765 | -1.7312510 |
| -1.2184628 | -0.7815396 | -1.1168831 | -1.2460206 | -0.1971315 | -1.7358321 | -1.0466951 | -0.9596669 | -2.0254278 |
| -1.4224931 | -0.2887260 | -1.5032438 | -1.6225447 | -0.1792619 | -0.7507817 | -1.6132707 | -0.1204210 | -1.1346310 |
| -0.6873550 | -0.6256348 | -0.8616410 | -0.4655266 | -0.1759911 | -0.6857854 | -1.1026945 | -0.6382632 | -1.5317919 |
| -0.6746401 | -0.6894422 | -1.1212746 | -0.8469709 | -0.3318177 | -1.4206579 | -1.4096736 | -0.8650898 | -1.6297597 |
| -0.5354951 | -0.2032256 | -0.7415313 | -0.3604719 | -0.7762903 | -1.3145145 | -0.3885744 | -1.3633517 | -1.6448558 |
| -1.3320907 | -0.8077262 | -1.6125588 | -2.1164750 | -1.0418064 | -1.0957740 | -1.6047178 | -0.9748084 | -1.5130382 |
| 1.2797955 | -0.0123537 | 0.5964535 | 1.0461856 | -0.0930787 | -0.5793477 | 1.2440557 | -0.5104928 | -0.5097712 |
| 3.4097609 | -0.4345583 | 0.9833812 | 3.7107910 | -0.9756915 | 1.1370774 | 3.5209209 | 0.3272420 | 1.8535449 |
| -1.2403442 | -0.7272012 | -1.4001180 | -0.6212109 | -0.9280882 | -1.0046483 | -0.4813129 | -1.1580938 | -0.8330519 |
| 0.2759791 | 0.1829311 | 0.1205623 | 0.0747590 | -0.0883717 | -0.7287130 | 0.2923219 | -0.4662961 | -0.7075769 |
| 0.8134702 | 0.1891235 | 0.2484990 | 0.6461477 | 0.0758577 | -1.1459382 | 0.0321891 | -0.7735973 | -1.0482843 |
| 1.1200183 | 0.1930562 | 0.4961513 | 1.0676906 | -0.0368434 | 1.2779868 | 1.5658937 | 0.8452043 | 1.3699204 |
| -1.1363674 | -0.7462387 | -0.8616787 | -1.0731677 | -0.8200955 | -1.5829302 | -2.1193743 | -1.5301739 | -2.5326423 |
| 0.7604627 | 0.2776048 | 0.7666876 | 0.9975880 | -0.0977410 | -1.0714823 | -0.8356983 | -0.8977145 | -1.0871697 |
| 0.3744606 | -0.2149135 | 0.4347489 | 0.7232615 | -0.3645585 | -1.9621654 | -0.6204373 | -2.1085031 | -1.5344081 |
| 0.3385782 | -0.4654207 | -0.2922350 | 0.5131084 | -0.1995166 | -1.2985912 | -0.2294119 | -1.2869565 | -1.5704938 |
| 0.0613579 | -0.4073309 | -0.5238178 | -0.1466695 | 0.0599869 | -2.0207602 | -2.3107032 | -1.6456174 | -1.9331717 |
| -0.8592773 | -0.6779477 | -0.6264559 | -0.7036269 | -0.6996964 | -1.0424528 | -1.9058675 | -0.4370247 | -1.3806285 |
| 0.5787399 | -0.2302002 | 0.0760434 | 0.5456768 | -0.5917268 | -0.8329667 | 0.4153508 | -1.4070326 | -1.1924646 |
| 1.9108756 | -0.1526776 | 0.8651788 | 1.8360197 | -0.8770632 | -2.0336673 | 0.8676425 | -1.3897877 | -1.3884437 |
| 0.6143520 | -0.0732172 | 0.3268782 | 0.3307980 | -0.3285126 | -1.5855479 | -0.0126844 | -2.0068134 | -2.0328052 |
| -0.5288076 | -0.3273532 | -0.7062927 | -0.2647437 | -0.0825816 | -0.7242607 | -0.5486375 | -0.5911173 | -0.8343513 |
| 0.1697575 | -0.2916365 | -0.1137047 | 0.3004479 | -0.3189047 | -1.4218590 | -0.0176084 | -0.9399954 | -0.7990840 |
| -1.1092310 | -0.5980341 | -1.1726721 | -1.0335325 | -0.4626244 | -0.8672125 | -1.8246475 | -0.2962796 | -1.2130488 |
| -1.2560440 | -0.2410277 | -0.8861040 | -1.1838239 | -0.9335680 | -1.8992776 | -1.7760296 | -1.4490915 | -2.4318716 |
| -1.2721221 | -0.2896417 | -1.5522146 | -1.1748679 | -0.2305304 | 0.1643835 | -1.9201773 | -0.0309289 | -0.3434474 |
| -0.2332555 | -0.2319045 | -0.7377150 | -0.3094613 | -0.2461361 | -2.8638286 | -0.9034080 | -2.6735198 | -3.5061844 |

| | | | | | | | | |
|---|---|---|---|---|---|---|---|---|
| 0.4444300 | 0.0892786 | -0.7182650 | 0.5120869 | 0.3647086 | -4.2327471 | -2.8440300 | -3.8954700 | -4.1146778 |
| 0.2326734 | -0.3069662 | -0.0296589 | 0.3089100 | -1.2225099 | -0.8641926 | -1.1769126 | -0.5502567 | -1.7577350 |
| -1.9769041 | -0.7494835 | -0.3244033 | -2.2065988 | -0.6511824 | -0.9934794 | -1.0788269 | -1.0531918 | -1.4623279 |
| -2.6052850 | -0.5196820 | -1.9979144 | -2.3102785 | -0.5069374 | -0.9688686 | -2.2174149 | -0.6827157 | -2.0168487 |
| -1.1914780 | -0.9028642 | -1.8688828 | -1.5308660 | -0.5830189 | -1.7739512 | -1.4985076 | -1.2704544 | -1.9688790 |
| 0.6466659 | -0.1905789 | -0.1025818 | 0.2895660 | -0.6024683 | -0.4893588 | -1.2900122 | -0.9354122 | -0.5479035 |
| 2.4805283 | 0.0771351 | 0.7486437 | 2.0125557 | -0.5629987 | 0.8341015 | 3.0196471 | 0.1024510 | 1.5314816 |
| -0.2948364 | 0.0411483 | -0.4248493 | -0.3590225 | -0.2055071 | 0.5303140 | 0.6111650 | 0.2041051 | 0.0678021 |
| -1.7407948 | -0.2613744 | -0.6962906 | -1.4693528 | 0.1747602 | -3.9549820 | -2.5689196 | -3.3778231 | -3.9202407 |
| -0.7121680 | -0.7934220 | -0.8665867 | -0.7852076 | -0.7213195 | -1.0140253 | 0.1406914 | -0.9531530 | -1.8052677 |
| 0.5780081 | -0.6461888 | -0.5906516 | 0.3115312 | 0.7902047 | -0.8972310 | -0.3955993 | -0.1298067 | -0.5277680 |
| -0.6622851 | -0.6402150 | -1.0824095 | -0.5121082 | -0.2234155 | -2.2114052 | -0.9337162 | -1.6865760 | -2.1043513 |
| 0.0407689 | -0.0908451 | -0.2449603 | -0.0776776 | -0.2284789 | -0.3703136 | 0.2688828 | 0.0339522 | 0.6212974 |
| -0.2145167 | -0.1033106 | -0.7438396 | 0.0420195 | -0.0161171 | -0.6798136 | -1.1898674 | -0.5203457 | -1.1289634 |
| -2.0588850 | -0.2529920 | -0.8728594 | -1.7192053 | -0.1870056 | -0.8194894 | -3.2366374 | -0.6270434 | -1.9140019 |
| -0.1616941 | -0.1274238 | -0.4446214 | -0.0020851 | -0.3863712 | -0.3334404 | -1.2229814 | -0.2567809 | -1.1482710 |
| -0.9873789 | -0.1803080 | -0.7251308 | -1.0619138 | 0.3995955 | -4.6984215 | -3.6121754 | -3.7628455 | -4.4407781 |
| -0.7206191 | -0.5042804 | -0.8748084 | -0.8589801 | -0.3007004 | -1.3108821 | -1.3769330 | -1.1218727 | -2.0066942 |
| -1.9545517 | 0.0037438 | -1.0391796 | -2.1153079 | -0.4070843 | -1.9307999 | -3.0184879 | -1.1383645 | -3.4006510 |
| 0.9795510 | 0.2942004 | 0.2545092 | 0.6607378 | 0.6594198 | -1.8494086 | -1.2248679 | -1.4635299 | -2.2192912 |
| 1.3340581 | -0.7229131 | -0.1250472 | 1.4355632 | -0.2894170 | -1.5011373 | -2.1873392 | -1.4552918 | -2.2672927 |
| -0.3663794 | -0.3907450 | -0.4078867 | -0.3051526 | -0.0561862 | 0.2616689 | -0.9899128 | -0.0925963 | 0.1438588 |
| -3.9590870 | -0.6880696 | -1.9001605 | -3.3657465 | -0.6968142 | -0.7362749 | -3.0383104 | -0.6517179 | -1.3234275 |
| 0.3184351 | -0.2732544 | -0.5075325 | 0.3006838 | -0.2471836 | -1.5209011 | -0.3384878 | -1.4141490 | -2.0503680 |
| -0.5661188 | -0.5047167 | -0.5726816 | -0.6535892 | -0.1821217 | -0.4896787 | -1.4064426 | -0.6389914 | -1.1076932 |
| -0.1024303 | -0.5600059 | -0.5011322 | -0.0221624 | -1.0516600 | -0.9464167 | -0.2056670 | -1.0207379 | -1.2425508 |
| -1.7194222 | -0.2183495 | -0.3839209 | -1.8466503 | 0.0993518 | -0.6438401 | -1.2435288 | -0.6230588 | -1.2286316 |
| -0.8668201 | -0.4975447 | -0.5042417 | -0.9450955 | -0.4583324 | -1.0680494 | -0.8237973 | -1.1044972 | -1.2184670 |
| 0.5540638 | -0.0538349 | -0.1734272 | 0.8887725 | -0.4229059 | -0.8479551 | 0.5604063 | -0.9027767 | -1.0485482 |
| 0.8576399 | -0.2309009 | -0.0671320 | 1.0675253 | 0.1890982 | -2.0419078 | -0.2855839 | -1.6843372 | -1.4006307 |
| -1.0051460 | -0.6373073 | -0.8189894 | -0.9223123 | -0.4989173 | -0.1488269 | -0.8149950 | -0.3215607 | -0.3902894 |
| -0.6254818 | -0.3342219 | -0.3692791 | -0.5171504 | -0.0921180 | -2.7699187 | -1.7627390 | -2.0537675 | -2.8313612 |
| 2.6458565 | -0.7786174 | -0.7922231 | 2.2398093 | -0.8816591 | 0.2623882 | 1.0401816 | -0.0425726 | 0.5935468 |
| -1.5238995 | -0.2106358 | -0.6423490 | -1.4197985 | -0.1900364 | -0.4242966 | -0.8555457 | -0.4043796 | -0.7597586 |
| -0.0013718 | -0.2393656 | -0.1233976 | 0.1753171 | -0.4237880 | -0.2931669 | -0.5666708 | -0.3571691 | 0.0054881 |
| -0.5845296 | -0.5930013 | -1.7598963 | -0.7857341 | -0.4993245 | -1.3805791 | -1.3418938 | -1.3948826 | -2.4828146 |
| -2.1231094 | -0.9579435 | -2.2622967 | -2.1504489 | -0.1971872 | -0.9670222 | -2.4892596 | -0.8261008 | -1.6097805 |
| -0.6057021 | -0.7337405 | -0.6036653 | -0.6102897 | -0.2192015 | 0.1334080 | -0.1746121 | -0.1946748 | -0.0660008 |
| 1.2248058 | -0.3323572 | 0.1112978 | 0.9063611 | -0.8001265 | 0.3173099 | 0.6665095 | 0.0583319 | 0.3221927 |
| -0.2354411 | -0.4193482 | -0.7916464 | -1.0223697 | -0.1763405 | -1.3525611 | -1.2631173 | -0.5021580 | -1.2206969 |
| 1.0784367 | -0.4907738 | -0.3875059 | 1.3100552 | 0.4085453 | -3.7572613 | -2.0427016 | -2.8310962 | -3.7886620 |
| -1.5858351 | -0.4408348 | -1.2815811 | -2.0189321 | -0.3608266 | -2.2759842 | -1.8966563 | -1.5119060 | -2.5611300 |
| -0.5399234 | -0.3025188 | -0.7808646 | -0.8181573 | -0.5730541 | -0.6663799 | -0.8126977 | -0.6416111 | -0.9311255 |
| -0.2010687 | -0.5926928 | -0.9131137 | -0.1644629 | 0.0250778 | -1.0283999 | -1.6785623 | -0.7207232 | -1.4546289 |
| 0.2453074 | -0.6941717 | -0.1160839 | 0.1645110 | -0.7248722 | -1.7571209 | -0.3569480 | -1.6570350 | -1.6838558 |
| -0.9964118 | -0.4953580 | -1.0580343 | -1.2320080 | -0.3439351 | -2.8126584 | -1.7481767 | -2.3864756 | -3.5303408 |
| -0.3340290 | -0.1067195 | -0.5189223 | -0.2877450 | -0.3582614 | -1.1755608 | -1.1343670 | -0.7399448 | -1.2238420 |
| 0.0772968 | -0.6634580 | -0.5823231 | -0.0599154 | -1.3589848 | -1.5760593 | -0.7921279 | -2.1367197 | -1.4370241 |
| 0.3413171 | -0.3569530 | 0.3839152 | 0.3616712 | -0.1475755 | -1.4377111 | 0.0945697 | -1.9659398 | -1.3317998 |
| -1.9780670 | -1.1348178 | -1.5749840 | -2.3801824 | -0.6855587 | -1.8005902 | -2.0379848 | -1.2133375 | -2.3133773 |

| | | | | | | | | |
|---|---|---|---|---|---|---|---|---|
| 0.4037581 | -0.1228512 | 0.0563532 | 0.6553541 | -0.3014233 | -0.2659437 | 0.6876521 | -0.5746083 | -0.2624952 |
| -0.3856009 | -0.5010937 | -0.2967221 | -0.5407431 | -0.4844002 | -0.8134728 | 0.643517 | -0.8130933 | -0.2995810 |
| -0.0369626 | -0.3502401 | -0.5693037 | -0.0991003 | -0.6387392 | -0.8418891 | 0.0988774 | -0.8084216 | -0.9544105 |

| PG_444 | PG_445 | PG_446 | PG_447 | PG_448 | PG_449 | PG_450 | PG_451 | PG_452 |
|---|---|---|---|---|---|---|---|---|
| -1.1486991 | -0.4947539 | -0.5044410 | -1.0988478 | -0.4234899 | -0.7978831 | -0.8467752 | -0.0852988 | -0.0448525 |
| -1.9391368 | -0.5793352 | -0.2624081 | -0.9544218 | -0.5968510 | -0.3445310 | -0.6826924 | -0.0722420 | -0.3823684 |
| -0.8837838 | -0.5321162 | -0.7156123 | -1.1752490 | -0.7513500 | -1.0260000 | -1.5106985 | -0.4361003 | -0.3019152 |
| -2.1500155 | -0.3611797 | -0.3853879 | -0.5383381 | -0.3033310 | -0.5574650 | -0.6506224 | -0.3346700 | -0.3447375 |
| -1.7085595 | -0.0410000 | -0.3724750 | -1.0813190 | -0.0355373 | -0.3348290 | -0.9383010 | -0.1128670 | 0.2275044 |
| -2.4206025 | -0.2105509 | -0.9060425 | -2.9973617 | -0.3950843 | -1.3691890 | -2.9434723 | -0.5864625 | -0.8414957 |
| -0.5882221 | -0.2385761 | -1.9382980 | -1.2377288 | -0.2231280 | -1.8368497 | -0.8056702 | -0.2032167 | -0.8254534 |
| -2.2584470 | -0.1701167 | -0.3207033 | -1.9014085 | -0.0254413 | -0.5603569 | -2.0241420 | -0.3394287 | -0.4895098 |
| -0.1691957 | -0.5070710 | 0.1160018 | -0.4239320 | -0.5302492 | 0.0922252 | -0.6548231 | -0.0692611 | -0.1076311 |
| -1.4124519 | -0.5697317 | -1.0223743 | 0.1614229 | -0.7623530 | -0.9474568 | -0.2355740 | -0.4295210 | -0.4663351 |
| -1.6061893 | -0.1998505 | -0.5082594 | -1.3529390 | -0.1606940 | -0.6719252 | -1.5128348 | -0.1430423 | -0.3811707 |
| -1.5025135 | -0.2885545 | -0.6484813 | -1.2149523 | -0.2958122 | -0.8901150 | -1.3110573 | -0.3292539 | -0.6238390 |
| -3.1038130 | -0.2505038 | -0.7864972 | -1.5370544 | -0.3995560 | -1.3506525 | -2.0887754 | -0.5432215 | -1.1132010 |
| 4.6001814 | -0.3619338 | 2.9423954 | 3.0261788 | -0.0334368 | 3.6051278 | 2.9264457 | -0.3741330 | 2.7562500 |
| -2.04273 | -0.2962110 | -0.1539659 | -0.8103328 | 0.2113454 | 0.1573513 | -0.6958315 | 0.1711757 | 0.3620127 |
| 2.5570707 | -0.3862160 | 2.4073602 | 2.2384806 | 0.0195244 | 2.5253743 | 2.3049034 | -0.4755381 | 1.6517537 |
| -1.0222429 | -1.0717462 | -0.8590790 | -1.1728708 | -1.0316829 | -0.8778227 | -1.2264441 | -0.5720350 | -2.2180799 |
| -1.2213670 | -0.0226925 | -0.1797303 | -0.0939290 | -0.3033130 | -0.4598977 | -0.1966672 | -0.2229333 | -0.0703659 |
| -0.3159217 | -0.3210080 | -1.5794807 | -0.8254474 | -0.9826855 | -1.4213049 | -1.8293014 | -0.4760424 | -0.8912933 |
| -1.0540481 | -0.4555787 | -0.5388302 | -0.7923153 | -0.6694463 | -0.5276423 | -0.2870747 | -0.8024245 | -0.9055119 |
| -0.4855719 | -0.2526542 | -0.1255409 | -0.1233027 | -0.4997922 | -0.0428348 | -0.0867450 | -0.0348815 | 0.8888814 |
| 0.0439062 | -0.1986337 | -0.2686340 | -0.2216440 | -0.3557150 | -0.4651654 | -0.1261640 | -0.3310144 | -0.3834125 |
| 1.8910123 | -0.5690472 | 0.4246621 | 0.8415764 | -0.4356372 | 0.1926271 | 0.5789244 | 0.0809096 | 0.6478318 |
| -2.3399395 | -0.4030954 | -1.1069753 | -2.2908420 | -0.3724938 | -1.8106367 | -2.6334590 | -0.5225657 | -1.4588652 |
| -3.0538753 | -0.4478755 | -1.7706120 | -2.4064877 | -0.6960050 | -2.2785559 | -2.9631734 | 0.0469949 | -0.5010041 |
| 0.9129532 | -0.1897639 | 0.3150875 | 0.5060059 | 0.0150988 | 0.1418193 | 0.0269625 | 0.1559986 | 0.0904464 |
| -2.0037418 | -0.0513189 | -0.2277414 | -0.0783040 | 0.0356362 | -0.1623011 | -0.1147159 | 0.2203579 | 0.0838848 |
| -2.6577328 | -1.0153925 | -1.0962861 | -2.6501413 | -1.0492574 | -1.4624488 | -2.4678938 | -0.5613474 | -1.1649014 |
| -2.1400605 | -0.1507478 | -1.2124563 | -1.9864158 | -0.3586980 | -1.6556043 | -2.2428018 | -0.0735555 | -0.9123190 |
| -2.1843823 | -0.2552562 | -0.4082260 | -0.5982105 | -0.0999591 | -0.3376579 | -0.4718600 | 0.0722658 | -0.3213090 |
| -0.3312633 | -0.5673031 | -0.7027797 | -0.6687435 | -0.6633322 | -0.5863820 | -0.8119224 | -0.0422797 | -0.3176247 |
| 0.1240652 | 0.3805759 | -0.2913634 | -0.1737514 | -0.1823210 | 0.0705727 | -0.2427180 | 0.2163259 | 0.1923705 |
| -1.4166067 | -0.4262481 | -0.0347111 | 0.7482540 | -0.2762964 | 0.5573724 | 1.0973033 | 0.1299711 | -0.0137967 |
| -3.7441073 | -0.2372278 | -0.8159855 | -1.1667901 | -0.1632918 | -0.7840104 | -1.6318920 | -0.6189339 | -0.7128584 |
| 0.0298753 | -0.1582570 | -0.0735374 | 0.0133074 | -0.2012603 | -0.1724634 | -0.0607350 | -0.1913069 | -0.0914757 |
| -0.8314890 | -0.7224051 | -1.2230729 | -1.0215312 | -0.9144454 | -1.9025230 | -1.4364959 | -0.2755273 | -0.6326901 |
| -2.4412443 | 0.0770603 | -0.8111964 | -0.9577984 | -0.1243487 | -1.0837282 | -1.3804220 | -0.2121382 | -0.7499011 |
| -0.3147011 | 0.0819670 | 3.2731996 | 1.1339953 | 0.4997144 | 3.6564838 | 0.6891121 | -0.0475499 | 2.1667294 |
| -0.5178444 | -0.1458405 | -0.3838278 | -0.3433711 | -0.2652849 | 0.2226237 | -0.7730741 | -0.0756787 | 0.1836881 |
| -0.0782779 | -0.7078373 | -1.3991582 | -0.2628131 | -1.2684479 | -1.4851619 | -1.2056650 | -0.2520027 | -0.3591004 |
| 2.6886821 | -0.1484030 | -0.0330520 | 0.2707569 | 0.0108451 | 0.2703384 | 0.3405212 | 0.3003263 | 0.3026167 |
| -2.5177613 | -0.4158991 | -0.9193267 | -2.0671991 | -0.4339798 | -1.2686770 | -2.5429989 | -0.7197289 | -0.9741887 |
| -1.7021157 | -0.8021789 | -0.9035895 | -0.3918862 | -0.7251543 | -0.7644071 | -0.4952578 | -0.0741960 | -0.6498099 |
| -3.0136657 | -0.0368844 | -0.4155568 | -0.7192808 | -0.1046960 | -0.5434142 | -0.9084642 | -0.6669300 | -0.4791160 |
| 2.3425670 | -0.2397845 | 0.1478077 | 1.1361893 | 0.0945578 | -0.1783281 | 0.3967000 | -0.0163329 | 0.3497496 |
| 0.3336406 | -0.7654482 | -0.7755270 | 2.3301885 | -0.5433438 | -0.3144778 | 3.1054598 | -0.8825759 | -1.1530377 |
| 0.2645248 | -0.4521422 | -0.4308634 | -0.4414305 | 0.2089239 | -0.3761891 | -0.8011850 | -0.2202461 | -0.0906488 |

| | | | | | | | | |
|---|---|---|---|---|---|---|---|---|
| -0.9363857 | -0.4063597 | -0.1574240 | -0.2763413 | -0.2301688 | -0.0083017 | -0.0582794 | -0.9290372 | 0.2184467 |
| -2.0667429 | -0.4745523 | -0.9464131 | -2.4336425 | -0.3299818 | -0.9355691 | -2.3187972 | -0.1311740 | -0.4060836 |
| -0.3155248 | -0.4350079 | -0.2052506 | 0.0532492 | -0.4346459 | -0.0626458 | 0.1797660 | -0.3118716 | 0.0971307 |
| 5.4046521 | -0.3468924 | -0.3871500 | -0.1269259 | -0.2895580 | -0.1117442 | -0.4939582 | -0.6376690 | -0.2732873 |
| -0.1993595 | -0.5684456 | -0.6210828 | -0.2207417 | -0.6541112 | -0.4755272 | -0.5464356 | -0.0634333 | -0.0802681 |
| 1.0256423 | -0.3009060 | -0.3274408 | 0.1046858 | -0.3704116 | -0.2276257 | -0.0641501 | -0.4438176 | -0.0357962 |
| -1.5116689 | -0.1995018 | -0.6257443 | -0.7791934 | -0.4309324 | -1.0942229 | -1.0003091 | -0.2272739 | -0.8006805 |
| 1.6352463 | -0.1982979 | 1.7966968 | 2.4039144 | 0.2352677 | 1.9749788 | 2.4689775 | -0.3008972 | 2.6873635 |
| -1.1980626 | -0.3520980 | 3.0325542 | 0.9744896 | 0.1954272 | 2.6874813 | 0.4214160 | -0.0450722 | 1.8001985 |
| -0.6642246 | -1.0652457 | -0.2952611 | -0.3903946 | -1.2340904 | -0.6766805 | -0.8909570 | -0.6377333 | -0.3556850 |
| -2.5825410 | -0.6620484 | -1.4062787 | -1.5491642 | -1.0614555 | -2.1589376 | -2.2160620 | -0.7491890 | -0.7435017 |
| 3.5509597 | 0.1098127 | 3.2666447 | 3.0965732 | 0.9602200 | 3.6207273 | 3.2951032 | -0.2760740 | 1.8926591 |
| 1.2381782 | -0.7163608 | 2.0895539 | 2.3572366 | -0.3890923 | 2.3591704 | 2.1752868 | -0.1269022 | 2.0061219 |
| -1.7751322 | -0.5784637 | -0.3412034 | -1.4321243 | -1.1249693 | -0.3833577 | -1.6433934 | -1.1044680 | -1.1444593 |
| -1.9892852 | -0.8777852 | -0.8500472 | -1.9321025 | -1.3255161 | -1.7644334 | -1.7058469 | -0.7314633 | -0.9316885 |
| -1.6221426 | -0.5224235 | -0.8560428 | -0.9564851 | -0.5555690 | -1.5213827 | -1.5862361 | -0.4671779 | -1.0485675 |
| -3.4090121 | -0.1018624 | -0.7239206 | -1.7097861 | -0.1894303 | -1.2014075 | -1.9984903 | -0.4936593 | -0.8495135 |
| -1.4545071 | -0.0913424 | -0.5790011 | -0.9329009 | -0.2692362 | -0.8558145 | -0.9841768 | -0.2336345 | -0.2318064 |
| -2.1360237 | 0.0367768 | -0.3708062 | -0.6135474 | -0.0320372 | -0.4174028 | -0.6755829 | 0.1013891 | -0.0457577 |
| -4.0093381 | -0.3039451 | -0.3102341 | -0.1530377 | -0.2939527 | 0.1065687 | 0.2246238 | 0.0291933 | -0.1296615 |
| 0.5088852 | 0.1869309 | -0.1016547 | -1.4752775 | -0.0048782 | -0.1636329 | -2.0608953 | -0.4838357 | -0.2338795 |
| -3.1125613 | -0.3880318 | -0.7039677 | -0.8132065 | -0.3724578 | -0.8988835 | -0.9199570 | -0.4406317 | -1.0020560 |
| 1.3850147 | 0.1203702 | -0.1309368 | 0.1842988 | 0.1263097 | -0.0491607 | -0.1838940 | 0.1558915 | 0.0390370 |
| -3.2465111 | 0.0055210 | -0.3121087 | 0.1071906 | 0.1854513 | -0.1270798 | -0.3201709 | 0.5459086 | 0.7219997 |
| -2.6747824 | -1.3898712 | -1.4382480 | -2.3258755 | -1.2899203 | -1.5188594 | -2.0619739 | -0.1715756 | -0.9934398 |
| -0.8803059 | -0.3271099 | -0.4743864 | -0.0653348 | -0.1909019 | -0.8212039 | -0.2048820 | -0.0327848 | -0.2853881 |
| -2.7576723 | -0.4441064 | -0.5973535 | -0.5729864 | -0.5477849 | -0.5509263 | -0.8572065 | -0.0756870 | -0.7763093 |
| 4.2222028 | 0.3844145 | 1.1064117 | 2.7138479 | 0.3373747 | 1.1346411 | 1.9714412 | 0.3702997 | 1.8005511 |
| 2.1718419 | -0.2457935 | 1.8900694 | 3.6771992 | -0.0178009 | 2.5013047 | 2.9150137 | 0.5567402 | 1.2738952 |
| -1.9568612 | -0.3693906 | -0.3462794 | -1.0214282 | -0.4680830 | -0.4459833 | -1.1653230 | -0.2441517 | -0.6676877 |
| -1.3136079 | -0.0298234 | -0.1872552 | -0.1819932 | 0.0211596 | 0.0499373 | -0.1137562 | -0.0456536 | -0.2006373 |
| -2.3512013 | -0.2214636 | -0.7246865 | -0.5360952 | -0.2435091 | -0.6435808 | -0.5708540 | 0.1522569 | -0.0229957 |
| -1.9118899 | -0.5801332 | -1.3914488 | -0.9752890 | -0.6126043 | -1.6628347 | -1.0923971 | -0.4520483 | -0.9151455 |
| -2.4390551 | -0.4156871 | -0.4871250 | -1.2489965 | -0.4209905 | -0.6547291 | -1.3940114 | -0.2768450 | -0.9781277 |
| 2.0652329 | -0.0877408 | 0.4060304 | 1.0920157 | -0.1211444 | 0.4207136 | 0.8957884 | -0.7003516 | 1.0139859 |
| -0.3864230 | 0.3057052 | -0.3400633 | -0.7533546 | 0.2598316 | -0.4690025 | -0.7792101 | -0.6427779 | -0.7363797 |
| -0.0696392 | -0.5967772 | -0.4005923 | -1.0356472 | -0.5877477 | -0.2470100 | -0.5645525 | 0.1528320 | -0.1730930 |
| -1.4319721 | -0.3688695 | -1.2570919 | -2.3051386 | -0.3289719 | -1.7064766 | -2.9499932 | -0.0444016 | -0.4012574 |
| -2.4133945 | -0.3981551 | -0.5378954 | -1.9991655 | -0.5703247 | -1.1618314 | -2.8541032 | -0.9010089 | -0.6123309 |
| -2.1145595 | -0.2257048 | -1.0350295 | -1.0459751 | -0.3804422 | -1.2484828 | -1.8794554 | -0.2072569 | -0.5270453 |
| -1.9875738 | -0.5010979 | -0.6844240 | -1.3534362 | -0.6693007 | -0.7917060 | -1.5437300 | -0.6048553 | -0.9950392 |
| -2.3257792 | -0.3619291 | -0.9957026 | -0.5893551 | -0.5415769 | -1.4186625 | -1.4686906 | -0.2598006 | -0.5906571 |
| 0.9103971 | 0.1094077 | 1.9715385 | 1.2172823 | 0.5595781 | 3.1802080 | 1.0449725 | -0.1523539 | 2.0916534 |
| -0.6515132 | -0.1087068 | -0.1770298 | -0.5247982 | -0.0312446 | -0.2272888 | -0.4618025 | 0.0691593 | 0.0024268 |
| -2.2098263 | -0.1569025 | -0.3683769 | -0.4705151 | -0.0756075 | -0.1777572 | -0.6208360 | -0.0233556 | 0.0043195 |
| -0.4618848 | -0.6608167 | -0.7160003 | -0.6872198 | -0.6635262 | -0.9103166 | -0.8711476 | -0.6291411 | -1.4727645 |
| 8.0903189 | -0.1418189 | 0.2740160 | 0.8477022 | -0.0710021 | 1.4742544 | 2.9550307 | -0.0136260 | 1.4168387 |
| 2.4133440 | -0.0078169 | 1.9223968 | 2.1701869 | 0.0182666 | 2.2285972 | 2.2458354 | -0.4183139 | 1.6798510 |
| -1.1405956 | -0.3301044 | -1.4767066 | -0.1951379 | -0.7761500 | -0.9426004 | -0.7048918 | -0.1423018 | -0.3055920 |
| -1.0121569 | -0.1689843 | -0.1572426 | -1.3604999 | -0.1907364 | -0.3648047 | -1.0698636 | -0.0884353 | 0.2198883 |

| | | | | | | | | |
|---|---|---|---|---|---|---|---|---|
| -1.254717 | -0.368018 | -0.731094 | -0.802146 | -0.834976 | -0.791534 | -0.236500 | -0.846871 | -1.393746 |
| 0.0327074 | -0.323124 | -0.431033 | -0.046687 | -0.269005 | -0.389667 | -0.323409 | -0.508749 | -0.430596 |
| -0.785863 | -0.423306 | -1.031144 | -1.454868 | -0.555250 | -1.572711 | -1.575715 | -0.335049 | -0.891959 |
| 0.1220202 | -0.228533 | -0.332503 | 0.3953214 | -0.131179 | -0.053414 | 0.6195353 | -0.012341 | 0.0048578 |
| -3.113101 | -0.112303 | -1.698747 | -1.198792 | -0.302355 | -1.954410 | -1.818941 | -0.308567 | -0.625349 |
| -0.383695 | -0.482081 | 0.6737396 | 0.5590667 | -0.514362 | 0.5136688 | 0.3459030 | -0.588550 | -0.635996 |
| -1.027815 | -0.430146 | -0.559949 | -0.921172 | -0.468959 | -0.555948 | -1.025184 | -0.116064 | -0.290883 |
| 0.1599789 | -0.030034 | -0.105648 | -0.019808 | -0.083703 | 0.0650366 | 0.0385949 | -0.262093 | -0.101714 |
| 0.1541786 | -0.124303 | -0.445025 | -0.417635 | -0.418463 | -0.013110 | -0.627000 | -0.410855 | -0.241413 |
| 0.5161282 | 0.3304819 | 0.8669602 | 0.9089939 | 0.3302746 | 0.8532483 | 0.7328791 | -0.315701 | -0.478328 |
| -1.974339 | 0.0212476 | -0.056175 | -0.094167 | -0.259746 | -0.135565 | -0.138059 | -0.037081 | -0.142595 |
| -3.761225 | -0.432546 | -0.723329 | -1.537089 | -0.697892 | -1.422675 | -1.705814 | -0.103677 | -0.028340 |
| -3.996593 | 0.0484089 | -0.638710 | -2.072134 | -0.020815 | -1.064833 | -2.460725 | -0.533019 | -1.189003 |
| -2.869648 | -0.110860 | -0.591989 | -0.398378 | -0.222054 | -0.704076 | -0.820775 | 0.0694814 | -0.283584 |
| -1.298638 | -0.411371 | -0.274572 | -1.506959 | -0.327497 | -0.450619 | -1.660204 | -0.072098 | -0.317552 |
| -0.105467 | -0.025508 | -0.103654 | -0.058039 | -0.035464 | 0.1508253 | 0.1330742 | -0.229427 | 0.0951713 |
| -1.111619 | -1.487718 | -1.918137 | -1.581596 | -0.821006 | -1.887428 | -1.628065 | -1.055674 | -1.337829 |
| 0.3060916 | 0.1430611 | -0.098508 | -0.348769 | 0.1812534 | -0.238922 | 0.1822667 | -0.215183 | -0.306897 |
| -0.997628 | -0.475874 | -0.354016 | -0.464011 | -0.956483 | -0.550917 | -0.562544 | -0.033871 | -0.901082 |
| 2.1666019 | -0.521253 | -0.204890 | 1.3774306 | -0.825586 | -0.221111 | 0.3240467 | -0.151927 | 0.0182048 |
| -0.944419 | -0.303029 | -0.540854 | -2.038832 | -0.249677 | -0.677928 | -2.308273 | -0.611722 | -0.817286 |
| -1.692512 | -0.368863 | -0.622246 | -1.069201 | -0.255571 | -0.672873 | -1.001767 | -0.364625 | -0.508359 |
| -0.153018 | -0.099053 | -0.272961 | -0.115454 | 0.1197850 | -0.139128 | -0.140683 | -0.102740 | -0.262923 |
| -1.442275 | -0.221283 | -0.546028 | -1.764802 | -0.170271 | -0.733723 | -1.561243 | 0.0292826 | 0.4542485 |
| -0.393637 | -1.072866 | -1.919360 | -2.112683 | -0.507908 | -2.212901 | -2.335523 | -0.643448 | -0.614807 |
| -1.364460 | -0.332448 | -0.915791 | -0.595543 | -0.481068 | -0.960846 | -0.768380 | 0.1179497 | -0.111954 |
| -0.227029 | 0.1419999 | -0.552778 | -0.156388 | 0.1328068 | -0.308768 | -0.309242 | 0.0842735 | -0.134999 |
| 0.9062897 | -0.226965 | 0.2901668 | -0.397305 | -0.195149 | -0.419729 | -0.957605 | -0.124829 | -0.471144 |
| -1.494729 | -0.333206 | -1.553250 | -1.739654 | -0.296102 | -1.504246 | -1.649806 | 0.0454882 | -0.574485 |
| -1.558852 | -0.438181 | -0.275013 | -0.034271 | -0.390598 | -0.108496 | 0.0879541 | -0.332434 | -0.220937 |
| -2.142595 | -0.457419 | -0.812743 | -1.137483 | -0.828028 | -0.961874 | -1.224739 | -0.656918 | -1.192546 |
| -3.089855 | -0.267660 | -0.778700 | -2.036262 | -0.353299 | -1.051208 | -2.923349 | -0.173258 | -0.395713 |
| -0.014134 | 0.0231882 | -2.181223 | -3.621806 | -0.623596 | -2.529686 | -2.809535 | -1.244154 | -1.231125 |
| -0.775541 | 0.2895713 | -0.104353 | -0.068527 | 0.4173109 | 0.1127925 | 0.0419787 | 0.0491762 | 0.1540115 |
| -2.094131 | -0.106988 | -0.744181 | -1.392144 | -0.065352 | -0.812832 | -2.090002 | -0.591243 | -0.633083 |
| -1.870022 | -0.448076 | -0.781609 | -0.734678 | -0.360038 | -1.411277 | -1.168275 | -0.041669 | -0.482298 |
| -0.774050 | -0.268166 | -0.356145 | -0.397907 | -0.126984 | -0.581007 | -0.440507 | 0.0811818 | -0.235058 |
| -2.713499 | -0.288389 | -0.429422 | -1.334781 | -0.370729 | -0.491409 | -1.114593 | 0.0267966 | -0.162771 |
| -0.880176 | -0.381103 | -0.145641 | -0.667455 | -0.450657 | -0.405448 | -0.449807 | -0.613935 | -0.838666 |
| -1.272028 | -0.573096 | -0.427923 | -1.207319 | -0.770399 | -0.531889 | -1.277108 | -0.749757 | -0.842297 |
| 0.4659419 | 0.2038754 | 0.4457106 | 0.3226718 | 0.4136017 | 0.5755173 | -0.153758 | 0.4298577 | 1.7038406 |
| -1.248305 | -0.229719 | -0.034959 | -0.446319 | -0.056699 | 0.1112112 | -0.414057 | -0.156248 | -0.230317 |
| -1.895486 | -0.182153 | -0.482317 | 0.1758869 | -0.176540 | -0.537234 | 0.3127939 | -0.038761 | -0.144637 |
| -1.533590 | -0.452375 | -0.544007 | -0.249279 | -0.496162 | -0.550511 | -1.202076 | 0.4284344 | 0.3516570 |
| -0.073071 | 0.0436612 | -0.152095 | -0.123746 | -0.119279 | -0.145504 | -0.187081 | -0.222506 | -0.091007 |
| -0.181527 | -0.529908 | 0.2608280 | -0.072555 | -0.411441 | 0.0897254 | -0.283418 | -0.185398 | -0.006226 |
| -0.988337 | -0.456626 | -0.682262 | -1.365723 | -0.404545 | -0.791950 | -1.578381 | -0.413014 | -0.592072 |
| -0.461420 | -0.205124 | 0.3224242 | 1.0699417 | -0.347224 | 0.3620650 | 0.8535933 | -0.384850 | 0.1067557 |
| -2.457710 | -0.220931 | -1.355311 | -1.227387 | -0.405150 | -1.152005 | -1.518387 | -0.308880 | -1.265855 |
| -1.888043 | -0.840681 | -1.062503 | -2.133044 | -1.131904 | -1.146699 | -2.094738 | -0.667969 | -1.380735 |

| | | | | | | | | |
|---|---|---|---|---|---|---|---|---|
| -1.240919 | -0.123081 | -0.133194 | 0.6500693 | 0.0208779 | -0.079744 | 0.7355661 | 0.1002445 | -0.125868 |
| -1.916715 | -0.592713 | -1.608185 | -2.310520 | -0.965007 | -1.812226 | -2.181118 | -0.377127 | -0.983333 |
| -0.486131 | -0.445722 | -0.200649 | 0.3177398 | -0.335053 | -0.394424 | -0.274064 | -0.494705 | -0.314396 |
| 1.7151463 | -0.115995 | 0.5416697 | 0.6564625 | -0.422415 | 0.7942920 | 0.4366323 | 0.4369500 | 0.8402593 |
| -1.893857 | -0.144891 | -1.475270 | -1.835692 | -0.165840 | -1.337866 | -1.562716 | -0.250819 | -0.693042 |
| 1.2781687 | 0.0944374 | 0.4591304 | 0.2152325 | 0.1106659 | 0.9187663 | 0.0986314 | 0.1131946 | 1.5736716 |
| -1.065733 | -0.230661 | -0.669533 | -0.811296 | -0.214099 | -0.650683 | -0.935217 | -0.412553 | -0.477270 |
| -1.299264 | -0.627426 | -1.126363 | -0.962092 | -0.667809 | -1.786919 | -1.258196 | -0.569431 | -0.370477 |
| -1.764487 | -0.212970 | -1.029053 | -1.665594 | -0.425716 | -1.413001 | -2.369150 | -0.274267 | -0.547069 |
| 0.2595307 | -0.496896 | -0.621414 | -0.245373 | -0.582919 | -0.642693 | -0.254727 | -0.460843 | -0.262917 |
| -0.051638 | -0.147062 | 0.3862451 | 0.5019543 | -0.144728 | 0.3829806 | 0.5344353 | 0.2551408 | 0.2140981 |
| -1.941299 | -0.394054 | -1.324435 | -2.418866 | -0.370454 | -1.548431 | -2.752854 | -0.587641 | -1.508610 |
| -1.568856 | -0.607415 | -0.880862 | -0.841548 | -0.728496 | -0.778664 | -0.860509 | 0.2622698 | -0.300405 |
| -0.437951 | -0.530697 | 0.1059980 | 0.1759807 | -0.396437 | -0.072490 | 0.1729742 | -0.252715 | -0.474640 |
| -0.102039 | -0.594164 | -0.517221 | -0.725058 | -0.650240 | -0.456313 | -0.497927 | -1.313709 | -1.778583 |
| -0.558599 | 0.0562309 | 0.0576700 | -2.656425 | 0.2543173 | 0.0443898 | -2.187250 | -1.455953 | -1.550245 |
| 0.3092488 | -0.520578 | 0.0893428 | -0.112651 | -0.244149 | 0.1078363 | 0.0114331 | -0.392155 | -0.534829 |
| -3.040850 | -0.531812 | -1.587101 | -2.191326 | -0.842071 | -2.626200 | -2.489737 | -0.570661 | -1.446384 |
| -0.992440 | -0.634713 | -1.122752 | -0.105519 | -0.858603 | -1.337700 | -0.808184 | -0.399626 | -1.007921 |
| -2.207510 | -0.750418 | -1.753594 | -1.339120 | -1.137417 | -2.213740 | -1.380566 | -0.478619 | -0.985702 |
| 0.7594572 | -0.269889 | -0.294835 | 0.2793863 | -0.222277 | -0.234285 | 0.2589949 | -0.286908 | -0.229767 |
| -0.804581 | -0.344760 | 0.0078214 | -0.119438 | -0.302793 | -0.085225 | -0.315037 | -0.119296 | 0.1154634 |
| -2.120779 | -0.464184 | -1.261085 | -1.374846 | -0.681605 | -1.608735 | -1.928396 | -0.440166 | -0.548803 |
| -1.190807 | -0.548635 | -0.516593 | -1.164647 | -0.483563 | -0.549194 | -1.543804 | -1.245448 | -0.915612 |
| -1.808075 | -0.335560 | -0.889178 | -1.351249 | -0.328563 | -0.901770 | -1.561100 | -0.570842 | -1.095444 |
| -1.749679 | -0.463688 | -0.769427 | -0.960800 | -0.384401 | -1.095816 | -1.042954 | -0.856826 | -1.082457 |
| -1.840556 | -0.542489 | -0.366033 | -0.267936 | -0.861785 | -0.527709 | -1.330883 | -0.133300 | -0.755135 |
| -0.353476 | -0.051336 | -0.166724 | 0.3437999 | -0.173322 | -0.278265 | 0.1857546 | 0.0594672 | -0.388730 |
| -1.807959 | -0.803074 | -1.385626 | -1.354909 | -0.921540 | -1.847388 | -1.504799 | -0.374236 | -0.799134 |
| 0.7411549 | -0.296305 | 0.5992663 | 0.9350035 | -0.502570 | 0.6133944 | 0.8550263 | -0.049672 | 0.4408064 |
| 3.7727858 | -0.115360 | 1.8829628 | 3.3072255 | 0.4532947 | 2.5018721 | 3.3499973 | 0.3852943 | 1.4210141 |
| -0.470272 | -0.211962 | -0.391811 | -0.123320 | -0.374023 | -0.517917 | -0.509286 | -0.520762 | -0.449028 |
| 0.1396012 | -0.139683 | -0.343293 | 0.4388492 | -0.025643 | -0.468386 | 0.1852298 | 0.1303059 | 0.2230939 |
| -0.056135 | -0.049888 | -0.282559 | 0.2685837 | -0.119613 | -0.031825 | 0.0723572 | 0.1134088 | 0.4448537 |
| 1.3084709 | -0.023238 | 0.4874491 | 0.3921673 | 0.0201863 | 0.2440092 | 0.1046858 | 0.3090827 | 0.8326010 |
| -2.447848 | -0.629155 | -1.189340 | -1.450944 | -0.857719 | -1.438729 | -1.799030 | -0.636973 | -0.774838 |
| -0.903706 | 0.5423120 | -0.401405 | 0.1114599 | 0.3832747 | -0.011382 | -0.012952 | 0.5173498 | 0.8391300 |
| -0.237609 | -0.276774 | 0.3041467 | 0.4821361 | -0.257476 | 0.697586 | 0.1013939 | 0.1020972 | 0.6899627 |
| -0.466909 | -0.249692 | -0.215362 | 0.2388699 | -0.488062 | -0.192844 | 0.2206073 | -0.226413 | 0.1154104 |
| -2.350532 | -0.454738 | -0.539154 | -1.216380 | -0.431314 | -0.332978 | -1.332846 | 0.1634797 | -0.071307 |
| -2.430621 | -0.939271 | -2.203989 | -0.994392 | -1.089076 | -2.127234 | -1.578813 | -0.484594 | -1.452966 |
| 0.2240955 | -0.377727 | -0.037076 | -0.019261 | -0.374889 | 0.0216860 | 0.0379673 | -0.298738 | -0.017679 |
| 0.6283580 | -0.021794 | -0.109155 | 1.0203047 | -0.309997 | -0.019428 | 1.2163942 | 0.0801730 | 1.4990994 |
| -0.712441 | -0.473453 | 0.2236633 | 0.7013167 | -0.521349 | 0.0408905 | 0.8552524 | 0.0580042 | 0.2965814 |
| -0.417196 | -0.330396 | -0.565557 | 0.0116523 | -0.128030 | -0.588097 | -0.082273 | -0.208019 | -0.678577 |
| -0.035741 | -0.192221 | -0.402238 | -0.064892 | -0.300451 | -0.171495 | -0.347323 | 0.0524225 | 0.1145573 |
| -2.070234 | -0.294943 | -0.569565 | -1.360447 | -0.236325 | -0.641361 | -1.878166 | -0.577203 | -1.089938 |
| -1.550193 | -0.354790 | -0.686135 | -0.227715 | -0.299986 | -0.461612 | -0.166302 | -0.368685 | -0.275726 |
| -1.910335 | 0.1380045 | -0.408455 | -1.516187 | 0.0116739 | -0.742591 | -2.279712 | -0.072803 | -0.565297 |
| -1.396767 | -0.302279 | -0.452781 | -0.207921 | -0.173550 | -0.313739 | -0.255224 | -0.366024 | -0.160885 |

| | | | | | | | | |
|---|---|---|---|---|---|---|---|---|
| -3.417805 | -0.496261 | 0.3894967 | 0.6502722 | -0.637179 | 0.6855909 | 0.7337023 | 0.7975480 | 0.6104339 |
| -1.827354 | -0.745679 | -1.099494 | -0.911488 | -0.866684 | -1.342144 | -1.143539 | -1.448186 | -1.254421 |
| -1.658436 | -0.829687 | -0.528696 | -1.020986 | -0.508443 | -0.310116 | -1.217331 | -0.715446 | -0.209645 |
| -3.004380 | -0.438577 | -1.710354 | -2.962483 | -0.508492 | -1.818862 | -3.021425 | -0.786720 | -1.649278 |
| -1.660346 | -0.549096 | -0.675544 | -1.853156 | -0.630549 | -1.415791 | -1.825426 | -0.204304 | -0.882637 |
| -1.711290 | -0.381910 | -0.653533 | -0.279324 | -0.252811 | -0.807577 | -0.949858 | -0.134893 | 0.0640196 |
| 2.6011828 | -0.261176 | 0.3319890 | 0.4554514 | 0.0142167 | 0.5360024 | -0.185029 | -0.452770 | 0.8891726 |
| 0.6554218 | -0.268149 | -0.345558 | 0.4268123 | -0.550745 | -0.501713 | -0.264063 | -0.121656 | -0.294211 |
| -2.693747 | -0.362037 | -0.562785 | -1.398236 | -0.259008 | -0.501264 | -1.711379 | -0.121859 | -0.635042 |
| 0.5106154 | -0.215895 | -0.597278 | -2.575090 | -0.238176 | -1.042664 | -2.308391 | -0.567744 | -0.674482 |
| -0.663789 | -0.523684 | 0.0873464 | -0.358192 | -0.098154 | -0.098897 | -0.942014 | 0.0375264 | 0.9133942 |
| -1.368705 | -1.105770 | -1.144938 | -0.933463 | -1.572551 | -0.827034 | -0.910206 | -0.446982 | -0.920695 |
| 0.2694508 | -0.342860 | 0.0236263 | -1.342931 | -0.230484 | 0.4314680 | -1.807010 | -0.366539 | -0.194633 |
| -1.250163 | -0.054419 | -0.526807 | -0.197098 | 0.0888872 | -0.285531 | -0.521475 | -0.115667 | -0.331194 |
| -3.283588 | -0.183289 | -0.996624 | -3.568827 | -0.270397 | -1.526377 | -3.532736 | -0.629944 | -1.003758 |
| -1.509519 | -0.211975 | -0.332358 | -0.332439 | -0.307094 | -0.633984 | -0.682724 | -0.006352 | -0.172208 |
| -4.159670 | -0.219145 | -0.656422 | -2.019713 | -0.257852 | -0.677562 | -2.024347 | -0.004647 | -0.064973 |
| -1.185024 | -0.291282 | -0.769338 | -0.954622 | -0.233127 | -1.015725 | -1.189654 | -0.247789 | -0.538021 |
| -3.076541 | -0.158687 | -1.291961 | -2.131590 | -0.091994 | -2.012041 | -2.896629 | 0.0707132 | -0.697541 |
| -1.088615 | -0.256223 | -0.445845 | -0.264057 | -0.259246 | 0.3643322 | -0.557327 | 0.3816450 | 0.3132555 |
| -2.773841 | -0.938466 | -1.202751 | -2.460539 | -1.477479 | -1.620512 | -2.407710 | -0.733590 | -1.345472 |
| 0.2108112 | -0.638176 | -0.590293 | -0.652689 | -0.555439 | -0.540493 | -0.611191 | -1.256453 | -2.445595 |
| -3.670602 | -0.337376 | -1.447099 | -3.690708 | -0.544746 | -2.238524 | -3.743861 | -1.953643 | -3.440975 |
| -0.521718 | -0.283348 | -0.355329 | -0.221149 | -0.217622 | -0.426679 | -0.133995 | -0.159966 | -0.291366 |
| -1.614440 | -0.179642 | -0.245129 | -1.510097 | -0.354588 | -0.217685 | -2.200015 | -0.302573 | -0.285235 |
| -0.307086 | -0.637347 | -0.197752 | -0.228611 | -0.623273 | -0.543668 | -0.557257 | -0.185112 | -0.263215 |
| -1.284803 | -0.329486 | -0.573967 | -1.849560 | -0.112218 | -0.670441 | -1.596789 | -0.097387 | -0.330578 |
| -0.941547 | -0.523653 | -0.674081 | -0.666002 | -0.543413 | -0.957236 | -1.022221 | -0.169084 | -0.331082 |
| 0.9804039 | -0.166101 | 0.4904498 | 1.1888155 | -0.474188 | 0.4957571 | 0.9323825 | 0.1735608 | 0.1768254 |
| -0.128942 | -0.216663 | -0.059718 | 1.0683731 | -0.176426 | 0.4319629 | 0.9355122 | 0.1075808 | 0.5180300 |
| -1.337417 | -0.575292 | -0.340317 | -1.332500 | -0.517784 | -0.744936 | -1.893218 | -0.942159 | -0.784400 |
| -1.886924 | -0.123957 | -0.428517 | -0.676745 | -0.194115 | -0.734203 | -0.555628 | -0.082660 | -0.285442 |
| 0.9971515 | -0.540966 | -0.117427 | 0.4438848 | -0.537975 | 0.0454210 | -0.202512 | 0.0895865 | 0.3861186 |
| -0.975338 | -0.392266 | -0.843721 | -1.717221 | -0.271464 | -1.167320 | -1.602655 | -0.086395 | -0.475912 |
| -0.709918 | -0.747892 | -0.013126 | -0.820855 | -0.930257 | 0.1707100 | -0.917764 | -0.055289 | -0.021971 |
| -1.695932 | -0.535073 | -0.879493 | -2.950808 | -0.491084 | -0.950334 | -2.533551 | -0.339563 | -0.526670 |
| -2.703476 | -0.657469 | -1.598109 | -2.775585 | -1.131149 | -2.357107 | -2.524228 | -0.381353 | -1.355091 |
| -0.246113 | -0.171022 | -0.245155 | 0.1102882 | -0.287128 | -0.185238 | -0.070713 | -0.191006 | -0.042587 |
| 0.1600048 | -0.153238 | 0.1526824 | -0.139268 | -0.286828 | 0.1002325 | -0.621715 | -0.295059 | 0.4415299 |
| -1.323965 | -0.228045 | -0.776555 | -2.267028 | -0.448665 | -0.943900 | -2.567165 | -0.506355 | -0.537164 |
| -2.156096 | -0.077989 | -0.169063 | 0.3850473 | 0.0214573 | 0.0253750 | 0.5506076 | -0.337691 | -0.095324 |
| -2.486081 | -0.612749 | -0.810098 | -1.729840 | -0.352415 | -0.887304 | -1.574552 | -0.031780 | -0.390087 |
| -0.930036 | -0.294980 | -0.457797 | -0.496471 | -0.318068 | -0.385420 | -0.504271 | -0.355333 | -0.240247 |
| -2.109387 | -0.606550 | -0.802375 | -0.555872 | -0.553134 | -0.439850 | -0.875925 | -0.642498 | -1.044131 |
| -0.747606 | -0.620687 | -0.502654 | -0.294876 | -0.561730 | -0.260602 | -0.215338 | -0.333378 | -0.452434 |
| -2.376418 | -0.619225 | -1.022482 | -1.030457 | -0.521393 | -1.253355 | -1.231236 | -0.098030 | -0.151462 |
| -1.000579 | -0.388792 | -0.672668 | -0.439597 | -0.413308 | -0.686317 | -0.923913 | -0.377396 | -0.507249 |
| -1.550008 | -0.855466 | -0.006631 | -1.801583 | -1.274790 | -0.340533 | -2.645090 | -0.410419 | -0.546440 |
| -0.172463 | -0.814802 | -0.647245 | 1.2834794 | -0.521158 | -0.445523 | 1.6819839 | -0.592397 | -0.779810 |
| -2.130014 | -1.010567 | -1.011286 | -2.096430 | -0.914986 | -1.446381 | -1.995283 | -0.641577 | -1.364209 |

| | | | | | | | | |
|---|---|---|---|---|---|---|---|---|
| 0.4424330 | -0.3486602 | -0.0685209 | 0.9167729 | -0.3546387 | -0.2224289 | 1.2712987 | -0.4113575 | -0.4176965 |
| 0.5939340 | -0.0205184 | -0.1964312 | 0.7538740 | -0.3675597 | -0.2766987 | 0.5613016 | 0.0877856 | 0.2189722 |
| -0.2417998 | -0.4782237 | -0.4134742 | -0.7655217 | -0.1532529 | -0.7308742 | -0.6128688 | -0.1174376 | -0.2580127 |

| PG_453 | PG_454 | PG_455 | PG_456 | PG_457 | PG_458 | PG_459 | PG_460 | PG_461 |
|---|---|---|---|---|---|---|---|---|
| -0.337332 | -0.305265 | 0.0929168 | 0.1273975 | -0.609569 | -0.631162 | -0.363656 | -1.073114 | -1.153792 |
| -0.971009 | -0.042123 | 0.0029166 | -0.756037 | -0.117172 | -0.029541 | 0.1205072 | 0.0022101 | -0.389285 |
| -0.603453 | -0.234327 | -0.218184 | -0.089938 | -0.864126 | -0.627197 | -1.286921 | -0.369483 | -0.189505 |
| -0.499890 | -0.296270 | -0.181564 | -0.920895 | -0.456683 | -0.439471 | -0.442189 | -0.943947 | -0.328058 |
| -0.045134 | 0.1961922 | 0.0886213 | -0.267197 | -0.078372 | -0.521483 | -0.565930 | -0.304672 | -0.413736 |
| -2.380634 | -0.636673 | -0.589755 | -1.983819 | -0.231554 | -0.283787 | -0.213053 | -0.280670 | -0.448878 |
| -0.324482 | -0.337712 | -0.788413 | -0.211940 | -0.272254 | -0.685387 | -0.163937 | -0.868152 | -0.298399 |
| -2.686694 | -0.413419 | -0.412882 | -2.649359 | -0.590632 | -0.789795 | -0.650040 | -0.941891 | -0.157308 |
| 0.3450107 | -0.226187 | 0.1510129 | 0.6012035 | -0.306207 | -0.145675 | -0.363539 | -0.296275 | -0.436507 |
| 0.5959794 | -0.382908 | -0.406951 | 0.7338653 | -0.267675 | -1.008530 | -0.421623 | -1.302630 | -0.088120 |
| -0.882329 | -0.311022 | -0.262758 | -1.079738 | -0.320132 | -0.544759 | -0.282126 | -0.490349 | -0.234115 |
| -0.419463 | -0.390818 | -0.537383 | -0.602226 | 0.0435460 | -0.356543 | -0.006227 | -0.671631 | 0.0223519 |
| -2.382739 | -0.446367 | -0.441723 | -2.903631 | -0.121262 | -0.289106 | -0.058484 | -0.421695 | -0.533929 |
| 3.2902196 | 0.6762576 | 2.6075200 | 3.1420446 | 0.5289331 | 4.8477247 | 1.8651023 | 5.1153994 | 0.1280862 |
| 0.4835157 | 0.3847120 | 0.0040228 | 0.7965232 | 0.1293992 | -0.538843 | -0.104767 | -0.589096 | 0.3982036 |
| 2.3858803 | -0.206786 | 1.5152109 | 2.6105156 | -0.361807 | 1.7909713 | -0.244346 | 1.8227011 | -0.287416 |
| -0.630731 | -0.896386 | -1.182081 | -0.303868 | -0.515702 | -2.246559 | -0.694833 | -2.430864 | -0.644924 |
| -0.184127 | -0.142011 | -0.225551 | 0.0167192 | -0.884582 | -0.381495 | -0.761437 | -0.398976 | -0.301507 |
| -0.481261 | -0.312952 | -0.448847 | -0.597189 | -0.081440 | 0.0328408 | -0.228218 | 0.3791899 | 0.0048473 |
| -0.462757 | -0.829821 | -0.568964 | -0.739329 | -1.276391 | -1.988888 | -1.474065 | -1.828914 | -1.127103 |
| 1.1448119 | -0.024306 | 0.5861471 | 0.4481548 | 0.0219405 | 0.3428865 | -0.163775 | 0.5069308 | -0.523017 |
| -0.176328 | -0.612239 | -0.123445 | -0.296680 | -0.325168 | -0.558499 | -0.646385 | -0.573622 | -0.417131 |
| 1.1947035 | 0.0748518 | 0.4505282 | 1.0518856 | -0.153850 | 0.9114054 | -0.050918 | 0.7348006 | -0.326724 |
| -2.291470 | -0.710295 | -0.626241 | -2.334326 | -0.742632 | -1.133290 | -0.652506 | -1.635275 | -0.632404 |
| -1.435311 | -0.273279 | -0.358867 | -1.786287 | -0.462419 | -0.823685 | -0.651823 | -1.068994 | -0.480126 |
| 0.3219583 | 0.1234920 | 0.0578764 | 0.6384167 | -0.096188 | 0.2564399 | -0.110810 | 0.1083465 | -0.210123 |
| 0.2156261 | 0.2192232 | 0.1500020 | 0.0452154 | 0.2438514 | -0.076891 | -0.008737 | 0.1382674 | 0.0662978 |
| -0.238477 | -0.360918 | -0.902462 | -0.520352 | -0.354999 | -0.366091 | -0.043592 | -0.591875 | -1.233123 |
| -0.046516 | -0.318983 | -1.126733 | 0.0021507 | -1.149462 | -0.435058 | -1.432221 | -0.704852 | -0.035974 |
| -1.154876 | -0.093998 | -0.087083 | -1.009881 | 0.0583518 | -0.349230 | 0.0794509 | -0.417141 | -0.139230 |
| -1.283315 | -0.148939 | -0.234052 | -1.443509 | -1.179499 | -0.682039 | -1.228356 | -0.787613 | 0.2923612 |
| 0.0579312 | 0.0914516 | 0.2428150 | -0.516665 | -0.008213 | 0.6181040 | 0.0760236 | 0.1883904 | -0.204303 |
| 1.5353633 | 0.1102175 | 0.2021433 | 1.3352616 | -0.185050 | 0.2924975 | -0.144442 | 0.7121018 | -0.377006 |
| -3.115019 | -0.657498 | -0.464509 | -3.006630 | -0.103344 | -0.388857 | -0.166411 | -0.382657 | -0.502334 |
| -0.191708 | -0.190496 | -0.054821 | -0.146491 | -0.098397 | -0.174737 | -0.149911 | -0.181037 | -0.010324 |
| -0.785729 | -0.867568 | -0.685130 | -1.000822 | -0.234404 | -0.709914 | -0.358700 | -1.393712 | -0.175460 |
| -2.176812 | -0.726945 | -0.401361 | -2.571478 | 0.3239574 | -0.224649 | 0.5714107 | -0.193771 | 0.0171005 |
| 1.1259842 | 1.6211054 | 1.3242173 | 0.6955506 | 0.3216158 | 5.1426076 | 1.3865032 | 5.4973455 | 0.3379617 |
| 0.5231936 | -0.091477 | -0.258319 | 1.1836570 | 0.1580814 | -0.326755 | 0.3046689 | -0.276127 | -0.528536 |
| 0.9623854 | -0.743202 | 0.4381981 | 1.0704136 | -0.842918 | -1.115844 | -0.772409 | -1.436008 | 0.1929529 |
| 0.5529723 | 0.2224684 | 0.3202319 | 0.3208466 | -0.086277 | -0.041801 | -0.029458 | 0.2549772 | -0.237070 |
| -2.428877 | -0.814067 | -0.559825 | -2.779244 | -0.290012 | -1.134993 | -0.497623 | -1.664576 | 0.1245806 |
| 0.0071914 | -0.485057 | -0.426713 | 0.2591704 | -1.115742 | -0.334445 | -0.356695 | -0.763454 | -0.263955 |
| -1.367265 | -0.476523 | -0.675191 | -1.872332 | 0.3522734 | 0.0161080 | 0.0594155 | -0.035240 | 0.0120088 |
| 0.7997649 | -0.133122 | -0.005387 | 1.1081565 | -0.117517 | 0.0779941 | -0.243563 | 0.1693519 | 0.1206381 |
| -2.261598 | -1.030043 | -0.859332 | -1.324969 | -0.119443 | -0.140134 | -0.212947 | -0.218146 | 0.2679989 |
| -0.218488 | -0.184169 | -0.109312 | -0.322661 | -0.014209 | -0.173553 | -0.312390 | -0.224680 | 0.1664882 |

| | | | | | | | | |
|---|---|---|---|---|---|---|---|---|
| 0.0161566 | -0.6382147 | 0.0475394 | -0.0135736 | -0.5669006 | -0.0679672 | -0.3885947 | 0.0318524 | -0.4239878 |
| -1.4026031 | -0.1655628 | -0.2687209 | -1.3082141 | -0.4724477 | -1.0182875 | -0.6356863 | -1.2101378 | -0.7671482 |
| 0.6831154 | -0.4095813 | 0.0728748 | 0.7768139 | -0.6863743 | -0.4537528 | -0.4830781 | -0.3674974 | -0.6157406 |
| 0.1195636 | -0.4185267 | -0.2478178 | 0.1644237 | -1.0292296 | -0.1899921 | -1.1307029 | -0.1496678 | -0.2370487 |
| -0.3810679 | -0.1302056 | -0.2284306 | -0.2819531 | -0.3951185 | -0.1622763 | -0.7932926 | -0.5658815 | -0.3759952 |
| 1.0840081 | -0.2755145 | -0.4159046 | 1.1766945 | -0.3783646 | -0.4972863 | -0.0136627 | -0.1971338 | -0.2156122 |
| -1.6831509 | -0.4415915 | -0.5933242 | -1.9739373 | 0.1164644 | -0.2391831 | 0.1312311 | -0.4941860 | -0.4705037 |
| 2.2401778 | 0.9821480 | 1.8614376 | 2.2356976 | -0.6680894 | 1.9407513 | 0.3540729 | 1.8858330 | -0.6473719 |
| 0.7306232 | 1.2022703 | 0.6119293 | -0.2502990 | -1.1176564 | -0.0218634 | -1.4161726 | 0.4229251 | 0.3663871 |
| -0.1471754 | -0.6758734 | -0.1558932 | -0.5879871 | -0.8117023 | -0.3086421 | -0.8834283 | -0.4365710 | -0.7118013 |
| -1.1997883 | -0.6326745 | -0.4470164 | -1.0810547 | -0.5566232 | -1.0220324 | -0.0787407 | -1.2051636 | -0.3815179 |
| 2.8715449 | 0.1896086 | 1.4527613 | 3.1769902 | 0.1193008 | 3.5963171 | 1.2302691 | 4.0347924 | -0.1250107 |
| 1.2920017 | 0.1650905 | 1.3459462 | 1.1459277 | 0.0886211 | 2.6536890 | 1.1798933 | 2.7496341 | -0.3324901 |
| -0.5691203 | -1.0825471 | -0.6262358 | -0.4176873 | -1.1414798 | -0.5633813 | -1.5816912 | -0.7201046 | -0.6438808 |
| -1.2941977 | -0.4664620 | -0.1880445 | -1.2783612 | -0.9886863 | -1.4923448 | -0.8386922 | -1.8593983 | -0.8788078 |
| -0.4794238 | -0.6016217 | -0.7731167 | -0.0437263 | -0.9375845 | -1.2027782 | -0.7780455 | -1.6501542 | -0.3977308 |
| -2.7737662 | -0.6240534 | -0.6453570 | -3.1193867 | -0.3501230 | -0.9944178 | -0.4544511 | -1.3957032 | -0.3363028 |
| -0.8415633 | -0.4593722 | -0.3611993 | -1.1152688 | -0.1039629 | -0.6964131 | -0.0779376 | -0.8865384 | -0.1712713 |
| -1.0870241 | -0.1016532 | 0.0896257 | -1.3403577 | -0.2328280 | -0.3245417 | -0.3269450 | -0.4539065 | -0.1960218 |
| -0.2196691 | -0.0638263 | 0.0044915 | -0.0021657 | 0.0099790 | -0.2080986 | -0.4290817 | 0.0114338 | -0.0962001 |
| 0.1474397 | -0.6542070 | -0.1725687 | -0.0686834 | 0.5212744 | -0.4438329 | 0.4274457 | -0.9459428 | 0.2291623 |
| -2.0673832 | -0.6204503 | -1.0404238 | -1.7645143 | -0.6181249 | -0.7795454 | -0.3716466 | -0.8319291 | -0.8797444 |
| 0.2641941 | 0.1416183 | 0.3105389 | 0.2776748 | 0.0646048 | 0.0850089 | -0.1223769 | -0.0696388 | -0.5016059 |
| 1.0106431 | 0.5719204 | 0.2106787 | 0.3699802 | 0.6957987 | 0.8640633 | 0.6341436 | 0.8529611 | 0.3262515 |
| -1.1296653 | -0.4433268 | -0.6513955 | -1.0019919 | -1.0404425 | -0.8955353 | -0.5871580 | -0.4541602 | -1.2703648 |
| -0.6651906 | 0.0155989 | -0.3844937 | -0.5028817 | -0.1685007 | -0.1882363 | 0.1763552 | -0.3483311 | -0.5074462 |
| -1.2906989 | -0.4443757 | -0.3059297 | -1.6237918 | -0.9634220 | -0.7668167 | -0.7714189 | -0.1737648 | -0.6673948 |
| 3.4893714 | 0.5744050 | 1.0078030 | 2.9813069 | 0.2139895 | 2.8110989 | 0.8794666 | 2.6961942 | 0.4699958 |
| 3.2113402 | 0.6764366 | 1.1082453 | 3.2205467 | -0.3670010 | 1.8825846 | 0.3460846 | 2.0324492 | -0.5792663 |
| -1.1776362 | -0.2987651 | -0.4764795 | -0.8420294 | -0.3715081 | -1.1742951 | -0.3650055 | -1.8298092 | -0.3093359 |
| -0.2379803 | -0.1482964 | -0.0597323 | 0.2013671 | -0.5737787 | -0.5582175 | -0.3949467 | -0.5478771 | 0.3340883 |
| -0.2017710 | 0.0567755 | 0.1813237 | -0.1440321 | -0.0477142 | -0.3666163 | -0.1364591 | -0.3763095 | -0.1127400 |
| -1.4279337 | -0.6812633 | -0.8113514 | -1.5577892 | -0.5586829 | -1.0476879 | -0.5974357 | -1.4715971 | -0.6306029 |
| -1.9527134 | -0.5113355 | -0.6652125 | -1.8184163 | -0.2219973 | -0.6445214 | -0.0982318 | -1.4712529 | -0.6456414 |
| 2.5552071 | -0.4402464 | 0.5362736 | 2.3457438 | -0.5133338 | 0.5469316 | -0.3769870 | 0.4375792 | -0.4759547 |
| -0.5648516 | -0.4146294 | -0.5577815 | -0.2228896 | -0.8593680 | -0.4189677 | -0.2166092 | -0.1347428 | -0.6272815 |
| -1.2539111 | 0.3161865 | 0.2003231 | -0.7954168 | -0.2979806 | -0.1264691 | -0.3295845 | -0.3091525 | -0.0323426 |
| -2.0238548 | -0.2611628 | -0.2229173 | -1.8331005 | -0.1575669 | -1.1083585 | -0.2071775 | -1.6092200 | -0.4920591 |
| -1.0878816 | -0.5825702 | -0.7667401 | -1.3342246 | -0.0953431 | 0.1474425 | -0.1977790 | -0.2813924 | 0.2627095 |
| -1.0504775 | -0.1877292 | -0.0228746 | -0.9988073 | -0.1654221 | -0.4742272 | -0.3860553 | -0.9101402 | -0.6963921 |
| -1.1173271 | -0.7491414 | -0.5947218 | -1.2602359 | -0.5638725 | -0.7285477 | -0.5474239 | -1.4087508 | -0.4407775 |
| -0.8804818 | -0.6984328 | -0.6377745 | -0.8667218 | -0.9201517 | -1.2142723 | -0.2619225 | -0.8015093 | -1.5766831 |
| 1.8434530 | 0.8196139 | 1.8735260 | 1.6213897 | -1.2716721 | -0.4783746 | -0.8469408 | -0.9193897 | -0.3080294 |
| -0.2133984 | 0.0936641 | 0.1545076 | -0.2449865 | -0.0190678 | -0.1742688 | 0.0469292 | -0.2427058 | -0.0756831 |
| -0.0521184 | 0.2292735 | 0.1697803 | -0.0740442 | 0.2104664 | -0.0769228 | -0.0971103 | 0.2256615 | 0.0718717 |
| -1.4226005 | -0.4959670 | -1.0812209 | -0.7931503 | -0.9278465 | -1.0025362 | -0.6231307 | -1.0625280 | -0.6992161 |
| 6.0687685 | -0.0706906 | 2.3660192 | 7.6570518 | -0.1922342 | 2.8700518 | -0.0254262 | 3.7469764 | -0.1410538 |
| 1.8486448 | -0.3261786 | 1.3833782 | 2.0772753 | -0.1301610 | 2.0523931 | -0.0270423 | 2.4671721 | -0.2593083 |
| -2.6862377 | -0.6469244 | -0.4683961 | -2.6020082 | -0.4322943 | -1.4530396 | -0.5988839 | -1.5076144 | 0.1625759 |
| -0.5628940 | -0.1237928 | 0.2716055 | -0.9991080 | -1.1721932 | -1.2791095 | -1.4481060 | -1.2331591 | -0.2252396 |

| | | | | | | | | |
|---|---|---|---|---|---|---|---|---|
| -1.0868959 | -0.9645054 | -1.2633027 | -0.6200652 | -1.3719726 | -0.8931390 | -1.4084349 | -0.8361821 | -0.7591853 |
| -0.6113593 | -0.4374972 | -0.3747738 | -0.7905455 | 0.0119147 | -0.3665838 | -0.3894233 | -0.3155481 | -0.5122939 |
| -1.4336034 | -0.5271293 | -0.3594888 | -1.7375553 | -0.2595120 | -1.0297984 | -0.0181923 | -1.0719557 | -0.8317379 |
| -0.2332359 | 0.0229544 | -0.0089944 | -0.4489229 | 0.1840244 | 0.0796260 | 0.1485849 | 0.1750189 | -0.4327139 |
| -1.4663281 | -0.3995164 | -0.5847539 | -2.1460391 | -0.0550070 | -0.6040752 | -0.3108921 | -0.6513782 | -0.2556440 |
| -0.3399713 | -0.4315471 | -0.5939933 | -0.4986897 | -0.6282108 | -0.3142508 | -0.5472252 | -0.6065182 | 0.6894555 |
| -0.8014942 | -0.3406275 | -0.4219002 | -0.3105766 | -0.2103840 | -0.0260788 | -0.0352579 | 0.2030070 | -1.0948937 |
| -0.0215324 | -0.4153396 | -0.1958712 | 0.0054375 | -0.2340757 | -0.3280958 | -0.0072819 | -0.0808814 | -0.1535717 |
| -0.0417315 | -0.3577806 | -0.0490773 | -0.1869879 | -0.5371353 | -0.4978374 | -0.3454574 | -0.6003161 | -0.0170877 |
| -0.2062485 | -0.5421729 | -0.8185259 | -0.3753776 | 0.1918953 | 0.4886206 | 0.2320903 | 0.5308944 | -0.1950735 |
| -0.3457996 | -0.1473579 | -0.1011281 | -0.3171814 | -0.3685580 | -0.2596745 | -0.3807652 | -0.3439951 | -0.3402659 |
| -1.9383994 | -0.2444924 | 0.0491521 | -2.3699612 | -0.0357282 | -0.3861542 | -0.0187513 | -0.4711737 | -0.6647756 |
| -5.0410425 | -0.7270770 | -0.8435123 | -4.7811767 | -0.0406322 | -0.4508814 | -0.0861172 | -0.4373630 | -0.2354591 |
| -0.7679422 | -0.0546896 | -0.4186767 | -1.4240420 | -0.4785939 | -0.6668372 | 0.1344689 | -0.6213498 | -0.5650523 |
| -0.3590289 | -0.3271481 | -0.1478981 | -0.6137723 | -0.2247543 | -0.7536648 | -0.2565040 | -1.0415668 | -0.3906914 |
| -0.1861062 | -0.1594916 | 0.0267185 | -0.1736792 | -0.2838898 | 0.1949250 | -0.1882450 | 0.0986207 | 0.0499401 |
| -1.8388315 | -1.1879932 | -0.7714261 | -2.1080336 | -0.4695079 | -0.7626981 | -0.0614910 | -0.9915343 | -0.9096278 |
| -0.3063566 | -0.2756588 | -0.0514913 | -0.3480723 | -0.4377133 | -0.2434972 | -0.3081150 | -0.1387606 | 0.0041349 |
| -0.7221674 | -0.2765668 | -0.9788581 | -0.6936773 | 0.3267174 | -0.9098346 | -0.2186021 | -1.2261614 | -0.8132247 |
| 0.7665526 | -0.1164319 | 0.5522341 | 0.7701319 | 0.0054779 | -0.1969373 | -0.5707506 | 0.1195940 | -0.5455210 |
| -2.2949581 | -0.6980822 | -0.8998947 | -2.3433707 | -0.3465740 | -0.7481864 | -0.4299812 | -1.0639498 | -0.0782635 |
| -0.5787666 | -0.4545294 | -0.4408007 | -0.9269625 | -0.1534906 | -0.3103366 | -0.1619439 | -0.5055792 | -0.0197969 |
| 0.0279787 | -0.1880322 | -0.1298480 | -0.0331219 | -0.3323551 | -0.0122436 | -0.0623171 | 0.0349323 | -0.2348221 |
| -0.2611430 | 0.0869003 | 0.2582184 | -0.3740615 | -0.2520127 | -1.0672859 | -0.4256197 | -1.0060624 | 0.0413455 |
| -0.8781498 | -0.8040326 | -0.6246788 | -0.6537763 | -0.4133063 | -0.3326653 | -0.0882124 | -0.4732191 | -0.3418240 |
| -0.6228934 | -0.1248848 | -0.1330685 | -0.8819185 | -0.0288075 | -0.4983594 | -0.6229129 | -0.7935003 | -0.3224725 |
| 0.1985542 | -0.0275432 | -0.2299484 | 0.1236381 | -0.0143401 | -0.4686886 | -0.1797717 | -0.2503254 | -0.1512688 |
| 0.3882409 | -0.1382626 | -0.3179464 | 0.2020447 | -0.2826131 | -0.5693225 | -0.0098938 | -0.1273353 | 0.2378994 |
| -0.1905870 | -0.2467296 | -0.1133111 | 0.0033288 | -0.7863768 | -0.2327907 | -0.6605118 | -0.2097863 | -0.1338258 |
| 0.728054 | -0.3469164 | -0.2045481 | 0.2953814 | -0.0310575 | 0.0408982 | -0.2233482 | 0.1821308 | -0.8930423 |
| -3.0274558 | -1.0547180 | -1.0154218 | -2.3372958 | -0.8703694 | -1.2039981 | -1.0823213 | -0.6274239 | -0.7076202 |
| -0.9354147 | -0.5013895 | -0.2954209 | -0.5693916 | -0.5684684 | -0.6254519 | -0.2276036 | -0.8498140 | -0.2100359 |
| -0.5941324 | -1.4480869 | -1.0121812 | -1.1719277 | -1.1016222 | -0.9322806 | -0.8954875 | -0.3103897 | -0.2741909 |
| 0.0702036 | -0.0852577 | 0.0131645 | -0.0961857 | 0.3961290 | 0.2301260 | 0.3148214 | 0.1780738 | 0.0924640 |
| -2.4356688 | -0.5824815 | -0.5637480 | -2.6568260 | -0.1189626 | -0.7772315 | -0.1959183 | -0.9205053 | -0.0629345 |
| -1.0630992 | -0.1278111 | -0.3273415 | -1.4624804 | -0.0888448 | -0.7823393 | -0.2517151 | -1.0673667 | -0.8781546 |
| -0.1116038 | 0.0252804 | -0.2376006 | -0.2078501 | -0.3299651 | -0.3474125 | -0.4758107 | -0.3903901 | -0.3245748 |
| 0.6363804 | 0.0779245 | -0.1088477 | 0.3795378 | -0.0505205 | -0.1847723 | 0.0641322 | -0.1952566 | -0.3682112 |
| -0.6795559 | -0.7961070 | -0.6913428 | -0.9113286 | -0.5237991 | -0.5814234 | -0.4727896 | -0.5760367 | -0.8284053 |
| -0.2673865 | -0.9256453 | -0.6675381 | -0.1688672 | -0.8438292 | 0.2418435 | -0.7666894 | 0.1450195 | -0.1332893 |
| 2.0590587 | 0.8720067 | 1.3086210 | 2.0442113 | -0.2290664 | -0.4623796 | -0.3294487 | -0.6460806 | -0.1946898 |
| -0.5227192 | -0.1086137 | -0.3109482 | -0.9862481 | 0.1162983 | 0.2567350 | 0.0165501 | 0.3248692 | -0.0880023 |
| 0.2859999 | -0.1833108 | -0.3255817 | -0.1264971 | -0.0499788 | 0.1352423 | 0.0322276 | 0.1116795 | -0.4058885 |
| -0.6886252 | 0.3930944 | 0.3001092 | -0.5801906 | -0.5942792 | -0.2969625 | -0.7039156 | 0.1001176 | -0.4514662 |
| -0.2213339 | -0.1231743 | -0.0657600 | -0.1893948 | -0.2502605 | -0.1535824 | -0.2838501 | -0.0980352 | -0.1763059 |
| 0.1784875 | -0.1434208 | -0.0563103 | 0.2817209 | -0.5112165 | 0.8657808 | 0.0343403 | 0.6975503 | -0.4304959 |
| -0.6922943 | -0.2392992 | -0.2643132 | -0.8187966 | -0.3480247 | -0.5515536 | 0.0751325 | -0.6637213 | -0.5938468 |
| 0.4548909 | -0.0951720 | 0.0230948 | 0.9491987 | -0.1678311 | -0.0215549 | 0.1172298 | -0.0718557 | -0.3973171 |
| -3.6692240 | -0.4980311 | -0.6402331 | -3.2032076 | 0.5259053 | -0.7252537 | 0.4405192 | -1.5718441 | -0.4488728 |
| -2.0260915 | -0.9031758 | -1.4353985 | -1.9816057 | -0.7740908 | -1.0827951 | -1.1980913 | -1.0219714 | -0.9155934 |

| | | | | | | | | |
|---|---|---|---|---|---|---|---|---|
| 0.0975408 | -0.0374707 | -0.2702932 | 0.1308485 | 0.4744189 | 0.3506039 | 0.4487669 | 0.5097435 | 0.0704732 |
| -1.8719402 | -0.7050002 | -0.2593389 | -2.0798838 | -0.5358451 | -1.6606755 | -0.8714072 | -2.0930759 | -1.057840 |
| 0.2836224 | -0.6319974 | -0.4985759 | 0.1432157 | -0.3479064 | -0.3856789 | -0.2439699 | -0.1170007 | -0.557144 |
| -1.1250093 | 0.4736701 | 0.7365356 | -1.3615841 | -0.615518 | -0.2261047 | -0.4451799 | -0.5019074 | -0.524456 |
| -0.8632944 | -0.2700089 | -0.4748764 | -1.0053294 | 0.1709060 | -0.7739807 | -0.2841909 | -1.0299468 | 0.0395248 |
| 1.3681209 | 1.1378212 | 0.8746079 | 1.1556049 | -0.9058661 | 0.1047121 | -0.0656887 | 0.1897389 | -0.1397621 |
| -0.6095859 | -0.4940287 | -0.4895233 | -0.796365 | -0.3459422 | -0.4525815 | -0.4824931 | -0.5586037 | -0.2998467 |
| -0.8218753 | -0.8268051 | -0.9017478 | -1.5200383 | -0.5179128 | -0.9176074 | -0.583818 | -0.8397027 | -1.244550 |
| -1.3402342 | -0.3216151 | -0.1716305 | -1.5426242 | -0.1893474 | -0.8887168 | -0.5818031 | -1.027582 | -0.5707167 |
| 0.3766602 | -0.238313 | -0.2790027 | 0.5616648 | 0.0919974 | 0.1569906 | -0.202353 | 0.2295865 | -0.1769253 |
| 0.4822282 | 0.1281368 | -0.012119 | 0.5594899 | -0.1812811 | 0.1817931 | 0.2014715 | 0.1454319 | -0.1538587 |
| -2.0915583 | -0.6478158 | -0.8420169 | -1.9694714 | -0.650847 | -2.0417393 | -0.7680303 | -2.7407157 | -0.3138292 |
| -1.3840011 | 0.1805007 | 0.0449420 | -0.6060534 | -0.9254413 | -0.4276929 | -0.1300582 | -0.3955243 | -0.361120 |
| 0.2231330 | -0.412684 | -0.6750204 | 0.7455493 | -0.3423809 | -0.0263202 | -0.272097 | 0.2213450 | -0.816917 |
| -3.6572263 | -1.4513389 | -1.2897801 | -3.6611181 | -0.3335101 | -0.1731862 | -0.376098 | -0.331220 | -0.2599155 |
| -1.2635852 | -0.9607203 | -1.243934 | -0.8894684 | -1.1074088 | -1.0333622 | -0.8714204 | -0.9131694 | 0.5281284 |
| -0.315247 | -0.2312449 | -0.3173821 | -0.5116187 | -0.6231321 | -0.6954669 | -0.315947 | -0.2373197 | -0.6742657 |
| -2.2152087 | -0.9340082 | -1.0447571 | -3.2522629 | -0.5825522 | -1.0891404 | -0.682478 | -1.913795 | -1.2391263 |
| -1.1321317 | -0.7017002 | -0.5607668 | -0.7725367 | -0.2368225 | -0.5241765 | -0.5027677 | -1.1645862 | -0.6560771 |
| -1.2947704 | -0.8232201 | -0.9235154 | -1.6702707 | -0.3966093 | -0.8435971 | -0.3020369 | -1.279433 | -0.5301831 |
| 0.1743973 | -0.1576823 | -0.1164672 | 0.4815207 | -0.2977648 | -0.1543648 | -0.1542444 | -0.0183542 | -0.0516949 |
| 0.2495449 | -0.2289319 | -0.0616285 | -0.0956868 | 0.1367457 | 0.4810959 | 0.1700679 | 0.2443876 | -0.277528 |
| -1.7639838 | -0.5860245 | -0.6362981 | -1.6002549 | -0.6769175 | -0.8885435 | -0.4331259 | -1.3458879 | -0.3487417 |
| -1.424512 | -1.2281975 | -0.6923972 | -1.340065 | -0.1901512 | -0.1791053 | -0.314808 | -0.1183455 | -0.1359653 |
| -1.4643625 | -0.7772313 | -0.6508037 | -1.4897415 | -0.2909677 | -0.9745878 | -0.4297548 | -0.8761644 | -0.1761629 |
| -1.0666281 | -0.8407847 | -0.9630408 | -1.2576394 | -0.4685105 | -0.9741131 | -0.3961367 | -1.204133 | -0.2629205 |
| -0.7831532 | -0.2078707 | -0.441520 | -1.0498403 | -1.0154414 | -1.1558181 | -1.2365378 | -1.204541 | -0.531811 |
| -0.8782133 | -0.1398519 | -0.3439632 | -1.0260945 | -0.0407248 | -0.3265065 | 0.0148126 | -0.3419653 | -0.1733152 |
| -1.5418217 | -0.4261957 | -0.423798 | -1.7313438 | -0.5187738 | -0.825201 | -0.8460392 | -1.3966175 | -0.6602354 |
| 1.0402671 | 0.0653600 | 0.1472523 | 1.0872086 | -0.3481221 | 0.4243698 | 0.2331743 | 0.6143761 | -0.466411 |
| 3.6272730 | 0.8074773 | 1.4814688 | 3.5896876 | -0.392241 | 1.5948527 | 0.2206813 | 1.9156885 | -0.2303948 |
| -0.6170395 | -0.3247981 | -0.769859 | -0.6320595 | -0.4259353 | -0.5552748 | -0.2138618 | -0.8194958 | -1.042736 |
| 0.3454602 | 0.1632690 | 0.1824631 | -0.0380323 | 0.0063996 | -0.0276723 | -0.1982862 | -0.0807701 | -0.043198 |
| 0.5182948 | 0.1009423 | 0.0968137 | 0.0904634 | 0.5638095 | 0.3938211 | 0.2102146 | 0.4768976 | 0.0467912 |
| 1.1192651 | 0.4023903 | 0.5816304 | 0.8447171 | 0.3883292 | 1.0015745 | 0.2340947 | 1.0321697 | 0.0934347 |
| -0.5556468 | -0.380354 | -0.7703898 | -0.246686 | -0.1613495 | -0.4581348 | -0.2089911 | -0.692714 | -0.4289269 |
| 1.5596442 | 0.5752049 | 0.2867368 | 1.6861507 | 0.4761262 | 0.7892291 | -0.141407 | 0.6577972 | -0.395304 |
| 0.9473548 | 0.3574080 | 0.2496128 | 0.6690001 | -0.2640653 | 0.5915735 | -0.3107667 | 0.9342761 | -0.3669134 |
| 0.2647622 | -0.1344211 | -0.0607429 | 0.2409050 | -0.0350797 | 0.2558923 | 0.1125001 | 0.0617452 | -0.244754 |
| -0.0388182 | -0.0419399 | 0.0254853 | -0.2610913 | -0.745752 | -0.7547504 | -0.5126495 | -0.7269014 | -0.2782914 |
| -0.4316171 | -0.5327675 | -0.7091297 | -0.6730565 | -1.3703302 | -0.629080 | -0.5009781 | -0.8937113 | -0.606939 |
| 1.0630018 | -0.4118235 | -0.3056408 | 0.9834777 | -0.6114758 | -0.0894168 | -0.2873222 | 0.0955283 | -0.8896219 |
| 2.0152849 | 0.6752123 | 0.8921561 | 1.6742177 | 0.2103329 | 0.3559374 | 0.1108729 | 0.5083353 | -0.6768442 |
| 1.1763710 | 0.0942953 | 0.0609847 | 1.1994571 | -0.1792494 | 0.2691493 | -0.1727505 | 0.1873305 | -0.6974742 |
| -0.9568911 | -0.324026 | -0.5259781 | -0.931677 | -0.1992272 | -0.7959831 | -0.2641383 | -0.850094 | -0.0075544 |
| 0.5344174 | 0.1807155 | -0.1148558 | 0.3069079 | 0.0362436 | -0.0883778 | -0.0327919 | -0.2646454 | -0.2311738 |
| -2.1828861 | -0.7916968 | -0.7512228 | -2.429920 | -0.5795627 | -1.011158 | -0.5418428 | -1.038241 | -0.1518554 |
| -0.5653215 | -0.509575 | -0.2486707 | -0.2581207 | -0.7484174 | -0.784028 | -0.6854601 | -0.6332213 | -0.4522447 |
| -2.5661069 | -0.2119675 | -0.245282 | -2.4286673 | -0.2806687 | -0.2012832 | -1.1732414 | -0.0151697 | 0.1046151 |
| -0.1361292 | -0.416678 | -0.1947908 | -0.502763 | -0.1455143 | -0.002816 | -0.0453297 | 0.1123547 | -0.1539748 |

| | | | | | | | | |
|---|---|---|---|---|---|---|---|---|
| 1.1102107 | 0.7960801 | 0.6464092 | 1.2984753 | 0.2290365 | 0.4066120 | -0.111249 | 0.3020107 | 0.2356173 |
| -1.522947 | -1.390702 | -1.196167 | -1.225959 | -0.302405 | -0.180081 | 0.0841488 | -0.399155 | -0.260695 |
| -0.102416 | -0.652911 | -0.343534 | -0.607374 | -0.806690 | -0.131629 | -0.361672 | 0.2209447 | -0.806271 |
| -1.307321 | -0.922215 | -1.497818 | -1.282106 | -0.528023 | -1.269106 | -0.745147 | -1.543312 | -0.509715 |
| -1.458333 | -0.375431 | -0.652252 | -1.690916 | -0.331968 | -0.767472 | -0.675223 | -0.804503 | -0.782939 |
| -0.705936 | -0.272084 | -0.166719 | -1.080114 | 0.0640048 | 0.2235117 | 0.2877883 | 0.3685756 | -0.585586 |
| 1.5437621 | 0.2097468 | 0.4124412 | 1.1096556 | -0.868523 | -0.206794 | -0.732181 | -0.120235 | -0.701618 |
| -0.001217 | 0.0213296 | -0.110361 | -0.384145 | -0.135856 | -0.086113 | -0.547311 | -0.344850 | -0.555423 |
| -1.204117 | -0.084373 | -0.261818 | -0.964311 | 0.3024618 | -0.191037 | 0.2120773 | -0.178695 | -0.179020 |
| -0.050123 | -0.528085 | -0.366707 | -0.008812 | -0.957174 | -0.948191 | -1.249551 | -1.167014 | -0.034004 |
| 0.0252196 | 0.7671628 | 0.7042560 | 0.0446013 | -0.526642 | -1.377756 | -0.254900 | -2.325534 | 0.0780784 |
| -0.181561 | -0.542779 | -0.473841 | -0.556458 | -0.648497 | -0.228246 | -0.567058 | -0.423270 | -0.820872 |
| -0.292240 | -0.157234 | -0.343246 | -0.146322 | -0.597540 | -0.320274 | -0.284235 | -0.259480 | 0.1483030 |
| -0.520621 | -0.367736 | -0.356925 | -0.880947 | -0.233144 | -0.745609 | -0.571654 | -0.484501 | -0.479390 |
| -2.074270 | -0.666471 | -0.775900 | -1.958015 | -0.344482 | -1.062317 | -0.272594 | -1.843436 | 0.2302406 |
| 0.0439321 | -0.146913 | -0.170338 | -0.177029 | -0.171793 | -0.100223 | -0.319163 | 0.3558 | -0.450717 |
| -1.452078 | -0.149527 | -0.109529 | -1.810809 | 0.0334352 | -0.482503 | -0.071706 | -0.753972 | -0.164838 |
| -0.677507 | -0.229524 | -0.235345 | -0.628550 | -0.025052 | -0.403072 | -0.189875 | -0.557813 | -0.376932 |
| -2.229129 | -0.208270 | -0.449975 | -2.114327 | -0.291309 | -0.774545 | -0.502490 | -1.324189 | -0.264448 |
| 0.9974484 | 0.2672703 | 0.4167454 | 1.2321256 | -0.396818 | -0.023915 | -0.183236 | 0.0954847 | -0.244351 |
| 0.0067996 | -0.794022 | -1.039680 | 0.3070237 | -0.235845 | -0.543907 | -0.321096 | -0.678609 | -0.557129 |
| -5.424011 | -1.678396 | -0.798224 | -4.956863 | -0.120043 | -0.491025 | -0.443592 | -0.630918 | -0.238265 |
| -3.855560 | -1.773543 | -2.633911 | -3.267612 | -1.268953 | -1.274938 | -1.368499 | -1.194058 | -0.566268 |
| -0.087667 | -0.271545 | -0.251045 | -0.025794 | -0.186552 | -0.204508 | -0.207965 | -0.185482 | -0.348874 |
| -0.812961 | -0.020997 | -0.294613 | -0.446423 | 0.4296155 | 0.4431266 | 0.3587226 | 0.2455164 | -0.344102 |
| 0.0970432 | -0.326066 | -0.283658 | 0.1833468 | -0.444074 | -0.157469 | -0.642678 | -0.227462 | -0.601327 |
| -1.044985 | -0.203177 | -0.337257 | -1.012832 | -0.154921 | -0.371583 | -0.027445 | -0.411036 | -0.214965 |
| -0.958775 | -0.170670 | -0.224523 | -0.750170 | -0.352938 | -0.492308 | -0.401419 | -0.440968 | -0.408053 |
| 1.0816360 | 0.2979998 | -0.230301 | 1.0062519 | -0.596823 | 0.1995468 | -0.348493 | -0.076598 | -0.638730 |
| 1.9426422 | -0.050504 | 0.2080893 | 1.8109891 | 0.0014779 | 0.2560252 | 0.0550690 | 0.5806664 | 0.0139034 |
| -1.237789 | -0.997173 | -0.463614 | -1.409030 | -0.662163 | -0.507114 | -0.572605 | -0.931941 | -1.055646 |
| -0.548473 | -0.268652 | -0.245448 | -0.679129 | -0.237026 | -0.360781 | -0.104915 | -0.368997 | -0.249785 |
| 4.7246710 | 0.2113762 | 0.3550513 | 3.9576096 | -1.036681 | -0.387754 | -1.234974 | -0.364687 | -1.068874 |
| -1.415117 | -0.191954 | -0.247454 | -1.877660 | -0.207127 | -0.756937 | -0.396044 | -1.406804 | -0.487357 |
| 0.4177672 | -0.130763 | 0.0848023 | 0.5668815 | -0.080744 | -0.083174 | 0.1746255 | 0.1159683 | -0.205611 |
| 0.3951138 | -0.279054 | -0.447407 | 0.5855142 | -0.674189 | -0.733331 | -0.299319 | -0.910125 | -0.347152 |
| -0.940611 | -0.762278 | -0.543302 | -0.573287 | -0.543338 | -1.773106 | -0.906465 | -2.319567 | -0.674074 |
| -0.140312 | 0.1221573 | -0.236519 | -0.172590 | -0.106270 | -0.145616 | -0.245857 | -0.320673 | -0.181725 |
| 0.0648230 | -0.420654 | 0.3424929 | -0.350575 | -0.239914 | 0.3753019 | -0.143361 | 0.2936882 | -0.251831 |
| -1.818684 | -0.591731 | -0.586803 | -2.446952 | -0.095594 | -0.526519 | -0.145027 | -0.652668 | -0.597430 |
| 0.5839301 | -0.451409 | -0.501236 | 0.3574697 | -0.229144 | -0.172824 | -0.167721 | -0.197442 | -0.020057 |
| 0.1042513 | -0.031780 | -0.067745 | -0.187189 | -0.341945 | -0.696488 | -0.346067 | -0.670233 | -0.209307 |
| -0.466786 | -0.449636 | -0.398306 | -0.375209 | -0.374042 | -0.550044 | 0.0771492 | -0.467073 | -0.228841 |
| -0.833060 | -0.645456 | -1.385959 | -1.023512 | 0.2250422 | 0.2742654 | -1.026067 | -0.204165 | -0.137081 |
| -0.286975 | -0.062073 | -0.540858 | -0.402260 | -0.159058 | -0.274390 | -0.089135 | -0.030800 | -0.555104 |
| -0.979627 | -0.211221 | -0.376803 | -0.739884 | -0.271142 | -0.872479 | -0.088476 | -1.239346 | -0.755911 |
| -0.484527 | -0.676784 | -0.470500 | -0.516386 | 0.2727377 | -0.275675 | 0.2404468 | -0.352056 | -0.394567 |
| -1.007317 | -0.546722 | -0.117988 | -1.130098 | -0.790417 | -1.320801 | -0.883322 | -1.966186 | -0.994538 |
| -0.652087 | -0.534470 | -0.872221 | -0.470359 | 0.7330539 | 0.8274065 | 0.3849392 | 1.1784432 | -0.592809 |
| -2.104182 | -0.706563 | -0.580243 | -1.698154 | -0.744421 | -1.424823 | -1.028435 | -1.179423 | -0.903384 |

| | | | | | | | | |
|---|---|---|---|---|---|---|---|---|
| 0.1273022 | -0.2563029 | -0.5377904 | 0.3296692 | 0.1423544 | -0.0109882 | 0.3307883 | 0.5797502 | -0.5356072 |
| 0.6273288 | 0.1817825 | 0.2105876 | 0.8333292 | 0.1280497 | -0.1652659 | 0.0628235 | -0.0589653 | -0.6881094 |
| -0.1950039 | -0.1363088 | -0.2376361 | -0.2585488 | -0.0333596 | -0.0540238 | 0.1046085 | -0.0564166 | -0.3184741 |

| PG_462 | PG_463 | PG_464 | PG_465 | PG_466 | PG_467 | PG_468 | PG_469 | PG_470 |
|---|---|---|---|---|---|---|---|---|
| -0.9599339 | -1.2941465 | -0.8472691 | -0.4197146 | -2.1805606 | -0.9285105 | -1.4912586 | -2.4064301 | -1.5125755 |
| -0.7025184 | -0.4938426 | -0.6433975 | -0.8024309 | -1.0094332 | -0.2845304 | -1.1631158 | -1.8863198 | -1.5595020 |
| -0.2741438 | -0.5677800 | -0.2349758 | -0.3030319 | -0.9293386 | 0.1781341 | 0.8646812 | -0.8912441 | 0.6730930 |
| -0.0805741 | -0.2032869 | -0.1803063 | -0.3110096 | -0.9168278 | 0.5411925 | -2.0250373 | -0.7567721 | -3.0188916 |
| -0.3896343 | -0.4836746 | -0.2963637 | -0.2417983 | -0.8347664 | 0.0079161 | -1.4833218 | -1.6316622 | -2.3083432 |
| -0.7312726 | -0.3364981 | 0.0049996 | -0.9985432 | -1.9955107 | -0.1161684 | -1.5666942 | -2.5407606 | -1.7656496 |
| -0.3734006 | -0.3015293 | -0.0982823 | -0.6173937 | -0.4130889 | -0.0826926 | -1.6106082 | -1.2033387 | -1.0772677 |
| 0.2769427 | -0.5671275 | 0.3743127 | -0.0135173 | -1.4548725 | 0.5639850 | 0.0720150 | -0.4690262 | -0.2439011 |
| -0.1601262 | -0.7238987 | -0.3323550 | -0.1011152 | -1.0404243 | -0.4571075 | -0.3405357 | -0.0708343 | -0.1760312 |
| -0.1960979 | -0.5260645 | -0.2612550 | -0.8188254 | -0.9481053 | -0.3703145 | -0.3063895 | -0.9364382 | -0.3895545 |
| -0.2670611 | -0.4103364 | -0.2029596 | -0.4499875 | -0.8716889 | -0.0726995 | -1.0478781 | -1.2021748 | -1.8283321 |
| -0.0858957 | 0.0730338 | -0.0395005 | -0.2348474 | -0.2543148 | 0.0913682 | -1.8417241 | -1.4144778 | -2.2299441 |
| -0.3060868 | -0.2241935 | -0.4618898 | -0.5718492 | -0.2388250 | -0.6122506 | -1.5863874 | -2.7367265 | -1.6029268 |
| -0.0034351 | -0.2104771 | 0.2670493 | 0.9175771 | 1.9835345 | -1.0686587 | 1.3364915 | 3.7631057 | -0.1902460 |
| -0.5211050 | -0.321437 | 0.2711896 | 0.0198382 | 0.0940758 | 0.6385027 | -2.5818025 | -1.1047004 | -2.6385146 |
| 0.4660603 | -0.0377189 | -0.4568673 | 2.6478040 | 1.7680660 | -0.4329653 | 1.5782008 | 2.5891124 | 0.4225259 |
| -0.7930773 | -1.4159226 | -0.4296966 | -1.0771102 | -1.1783902 | -1.1534794 | -1.1050878 | -1.1677914 | -1.1882588 |
| -0.6650524 | -0.6696742 | -0.5507832 | -1.5818547 | -1.4736709 | -1.6328859 | -1.7079778 | -1.6673107 | -2.1907249 |
| -0.1978300 | 0.5267242 | -0.2193979 | -0.2444540 | -0.2013679 | -2.3137475 | -1.7381432 | -1.8605104 | -2.1054509 |
| -0.7626976 | -0.5005155 | -0.9175311 | 0.3335732 | -0.4398937 | -1.4002655 | -0.4818371 | 0.4149126 | -1.9932863 |
| -0.4602763 | -0.1310301 | -0.5233438 | -0.1720718 | 0.4648973 | 0.0904369 | -0.8591007 | 0.4791995 | -1.5665264 |
| -0.5552009 | -0.5514868 | -0.4158054 | -0.4730337 | -0.4525358 | -0.4654604 | -0.6763963 | -0.4213732 | -0.2504750 |
| -0.1457360 | -0.0670544 | -0.2755110 | 0.4228257 | 0.6534939 | -0.5010984 | 0.8909923 | 1.2849338 | 0.5177881 |
| -0.2988261 | -0.6028827 | -0.3585025 | -0.4571386 | -0.6633087 | -0.7416453 | -1.3290875 | -2.4541699 | -1.2615426 |
| -0.3678133 | -0.6310657 | -0.4645253 | -1.1832061 | -1.7785068 | -0.3241510 | -0.9549453 | -1.4994288 | -1.3099346 |
| 0.1841111 | -0.0518195 | 0.0790893 | 0.4103984 | 0.2669561 | -0.2753641 | 0.8268212 | 1.1367261 | 0.2566167 |
| 0.0025785 | 0.0041454 | -0.0448472 | -0.0524135 | 0.0498608 | 0.3115806 | -2.2704175 | -2.0400742 | -2.8397171 |
| -0.7906211 | -1.1311267 | -1.0105426 | -1.0626000 | -1.0078052 | -0.9593907 | -1.6149474 | -2.6356518 | -1.9133631 |
| -0.1984546 | -0.3390265 | -0.1254358 | -0.5007877 | -1.3776471 | -0.1162453 | -0.5765583 | -2.5028480 | -0.5454754 |
| -0.2137722 | -0.1279339 | -0.1648645 | -0.3759030 | -0.2538142 | 0.2692677 | -2.0556767 | -1.2711951 | -2.2508629 |
| -0.0507583 | -0.1624798 | 0.4928683 | -0.1140896 | -0.6300125 | 0.3298357 | -0.4084273 | -1.5281865 | -0.3764578 |
| -0.0119417 | -0.5637015 | -0.4065686 | -0.0959122 | -0.3121728 | 0.3516907 | 0.5495483 | 1.3712067 | 0.6797356 |
| -0.6317856 | -0.5273485 | -0.3691725 | -0.4548084 | -0.2588226 | -0.3512874 | -1.1678831 | -1.8264614 | -1.2441789 |
| -0.4178454 | -0.3516736 | -0.3262545 | -0.4090356 | -0.9102175 | -0.8493642 | -0.7080150 | -1.6491569 | -1.0114907 |
| -0.0339746 | 0.0446094 | -0.0010922 | 0.0436656 | 0.1858409 | -0.8676020 | -1.0031501 | -2.1458905 | -1.1474540 |
| -0.1919166 | -0.0853698 | -0.1707783 | -0.1131477 | -0.0590705 | -0.5982363 | -1.5737725 | -1.0940333 | -2.0570655 |
| -0.1944071 | -0.1162303 | -0.5257377 | -0.3728536 | -0.3755183 | 0.2473426 | -0.8566932 | -1.0008068 | -0.6548376 |
| -0.0706811 | -0.4204075 | -0.0914397 | 1.4561689 | 0.8062981 | 0.1997011 | 2.8890831 | 1.3825180 | 2.4935644 |
| -0.1926473 | -0.5807447 | -0.4590752 | 0.1808121 | -0.6267463 | -1.5351767 | -1.3704056 | -0.4967154 | -1.4175382 |
| -0.5150259 | 0.1574004 | -0.2241442 | -1.0610848 | 0.4120602 | -1.5729246 | -2.6745462 | -1.6671505 | -2.8419269 |
| -0.2084191 | -0.0045196 | -0.2005521 | -0.1869559 | 0.2937050 | -0.4831494 | 0.4693169 | 0.8384995 | 0.8212896 |
| -0.2652799 | -0.7007482 | -0.2374160 | -0.7908313 | -1.9454052 | -0.3127389 | -0.4658362 | -2.2452673 | -0.1300664 |
| -0.4096915 | -0.4698649 | -0.4901350 | -0.3985927 | -0.7562704 | -0.1766061 | -1.3014001 | -0.9958432 | -1.6037606 |
| -0.3685486 | -0.4207940 | -0.3543873 | -0.2873621 | -0.7733913 | 0.0234782 | -2.0160189 | -2.0461381 | -2.2760048 |
| 0.0750140 | -0.2005594 | 0.0823633 | 0.4611015 | 0.1702313 | -0.2598942 | 1.6482002 | 1.8285222 | 0.9591045 |
| 0.0194061 | -0.0428238 | 0.1367899 | -0.1764625 | -0.2328405 | -0.3614526 | 0.2508678 | 5.2605032 | 0.0918666 |
| -0.0598520 | -0.1485656 | -0.1309524 | -0.0933033 | 0.1456564 | -0.0582395 | 0.4098382 | 0.2733676 | 0.4325236 |

| | | | | | | | | |
|---|---|---|---|---|---|---|---|---|
| -0.3598470 | -0.2238211 | -0.3258511 | -0.2429823 | -0.0960139 | -0.3132058 | -1.0443721 | -0.3052767 | -1.8531478 |
| -0.6867032 | -0.8798180 | -0.7397491 | -0.9293532 | -1.8867544 | -0.3613354 | -0.7634899 | -2.0739046 | -0.9503706 |
| -0.5132009 | -0.6277057 | -0.6319126 | -0.5910682 | -0.8899995 | -0.5604270 | -0.8046692 | -0.4388116 | -1.2776982 |
| 0.0579129 | -0.3068001 | -0.3749643 | 0.7326291 | -0.1400904 | -1.8647341 | 1.7112425 | 3.8945142 | -1.0555230 |
| -0.3200015 | -0.2756067 | -0.4758479 | -0.4751518 | 0.1113626 | -0.3484033 | -0.6345498 | -0.5955307 | -0.5938480 |
| -0.0726378 | -0.0836009 | -0.2261219 | -0.1078480 | 0.3548371 | -0.0603177 | 0.6511348 | 1.1580582 | 0.7249103 |
| -0.2687016 | -0.0377271 | -0.3607677 | -0.4180610 | -0.4627700 | -0.1165458 | -0.9738829 | -1.4260165 | -0.8877058 |
| -0.3886688 | -0.3972384 | -0.7398218 | 0.0647818 | 0.7197666 | -1.0634979 | 1.3639586 | 2.2866029 | 0.9818164 |
| 0.2410510 | -1.1246706 | 0.3544534 | 2.8166584 | -0.0774009 | 0.3653260 | 3.6179387 | 0.9437809 | 2.8320683 |
| -0.4608262 | -0.6737561 | -0.6030056 | -0.2389767 | -0.2636798 | -0.8185125 | -0.6246264 | 0.0281068 | -1.2303652 |
| -0.5466109 | -0.7493289 | -0.5189783 | -0.9817155 | -1.7955446 | -0.6085822 | -1.4807677 | -1.2765144 | -1.8664025 |
| 1.1755595 | 0.2111279 | 0.2505174 | 2.9136495 | 1.7291697 | 0.4466697 | 1.9656268 | 3.2598985 | 0.8827999 |
| 0.3953261 | 0.3578653 | -0.1811907 | 1.8971066 | 1.5245196 | -0.7082909 | -1.4359659 | 0.3822865 | -2.2189888 |
| -0.7813229 | -0.7512953 | -0.8654966 | -0.7524344 | -0.6456781 | -0.7913650 | -1.0418634 | -1.8282299 | -1.3058110 |
| -1.2674022 | -0.8324962 | -1.0833284 | -2.5271922 | -1.8896413 | -0.4961671 | -0.7198005 | -0.8185394 | -0.6307032 |
| -0.8126467 | -0.5673218 | -0.4457274 | -0.2854482 | -0.4396628 | -0.7701090 | -1.6128571 | -1.3813709 | -1.3048011 |
| -0.2532405 | -0.3982979 | -0.2553671 | -0.4529121 | -1.6309060 | 0.2607125 | -0.8321267 | -1.7780760 | -1.0847182 |
| -0.3933604 | -0.3283085 | -0.3012919 | -0.6125272 | -0.5035078 | -0.1723726 | -0.7249619 | -0.3915096 | -0.5860212 |
| -0.5906162 | -0.4099835 | -0.3593538 | -1.2642295 | -1.0754701 | -0.3856136 | -0.4052198 | -0.7041582 | -0.2949810 |
| -0.1436197 | -0.1526250 | -0.0390315 | -0.3066594 | -0.4928385 | 0.5545271 | -3.3014494 | -2.4674691 | -2.9092690 |
| 0.2612874 | -0.0378292 | 0.0661887 | 0.2436004 | 0.1597781 | -0.5025672 | -0.2244078 | 0.8293308 | -0.4730576 |
| -0.7683695 | -0.5410590 | -0.7520564 | -0.8308804 | -0.7240269 | -0.9846642 | -1.0957346 | -2.2640348 | -0.9770472 |
| -0.0733132 | 0.2433985 | -0.3115828 | -0.4398883 | 0.3104949 | -0.5305332 | 0.9626863 | 1.3014206 | 1.0046536 |
| 0.3842251 | 0.4405506 | 0.2520767 | 0.2494428 | -0.0898600 | 0.4549174 | -2.4126606 | -2.3503105 | -3.0430665 |
| -0.7971823 | -1.1980200 | -0.8449538 | -1.0677425 | -2.3533635 | -0.0234474 | -0.9064412 | -0.5622306 | -1.3633747 |
| -0.3394957 | -0.2944291 | -0.2674575 | -0.4974533 | -0.7894868 | -0.0108808 | -0.8264367 | -0.5522819 | -0.6913606 |
| -0.9214514 | -1.1063645 | -0.8118729 | -1.7076014 | -1.7335492 | -1.2767097 | -2.0428732 | -2.0742696 | -0.9195160 |
| 0.4608373 | 1.3461558 | 0.4214871 | 1.6668116 | 3.0759668 | -0.0617393 | 0.9504730 | 2.9553818 | 0.0265545 |
| 0.1450768 | 0.2363767 | -0.3630113 | 0.3345573 | 0.9595403 | -0.6201233 | -0.0178093 | 1.7215391 | -0.9450124 |
| -0.0613347 | -0.6372265 | -0.0254003 | -0.4989653 | -1.2051577 | -0.3880070 | -0.9337999 | -2.6974689 | -0.8659931 |
| -0.0041456 | 0.1060752 | 0.0117346 | -0.1824661 | -0.2268162 | -0.6298432 | -0.7522737 | -0.4569970 | -0.4721815 |
| -0.2675143 | -0.3091068 | -0.0468472 | -0.3061358 | -0.4923251 | 0.2822782 | -2.2140423 | -1.3169006 | -3.7355959 |
| -0.5837439 | -0.5448137 | -0.6474662 | -0.8302860 | -0.7841557 | -0.9514202 | -1.3113492 | -1.5879796 | -1.4114046 |
| -0.3938025 | -0.5226776 | -0.3107323 | -0.5652354 | -1.7766914 | -0.2170623 | -1.5328666 | -1.8113509 | -1.8993949 |
| 0.0153725 | 0.5305464 | -0.2953487 | 0.3825154 | 1.4514502 | -0.6396464 | 0.6959329 | 1.8332393 | 0.3334246 |
| -0.3406592 | -0.4455645 | -0.4616339 | -0.3376435 | -0.7267677 | -0.4348341 | 0.5883138 | -0.6199513 | 0.8553831 |
| -0.2907495 | -0.4719788 | -0.4015438 | -0.1761037 | -0.6258733 | -0.1642572 | -0.1923924 | -0.2360764 | -0.4336008 |
| -0.0700881 | -0.4076382 | -0.1082849 | -0.4065347 | -1.1472709 | -0.0523107 | -1.0776569 | -2.0450073 | -1.1617692 |
| 0.3239752 | 0.0366763 | 0.3452619 | -0.5985672 | -0.7664717 | 0.1480378 | 0.3209743 | -0.1618847 | 0.2175129 |
| -0.7874688 | -0.6829795 | -0.3721122 | -0.6676393 | -0.9705390 | -0.1844789 | -0.5403358 | -1.3703693 | -0.0517351 |
| -0.5208537 | -0.7051006 | -0.6456794 | -0.6023978 | -1.0459914 | -0.4652980 | -0.9186892 | -0.8117726 | -1.0837085 |
| -1.3316874 | -1.5180050 | -1.2638855 | -1.3720584 | -2.4213444 | -1.0188347 | -1.2154712 | -1.0121322 | -1.0011005 |
| 0.4909282 | 0.7357057 | 0.3144200 | 1.5733559 | 1.4890137 | 0.0187860 | 3.4302796 | 2.7876753 | 2.8455339 |
| -0.1728551 | -0.2525635 | -0.0991575 | -0.1220254 | -0.1158671 | 0.0712021 | -0.8059101 | -0.8078673 | -1.1113165 |
| -0.2276208 | -0.1633540 | -0.1049934 | -0.2012050 | -0.4475816 | -0.0381148 | -1.6976532 | -2.4629914 | -2.5597107 |
| -0.7668300 | -1.0398829 | -0.7339398 | -0.7055791 | -1.0902211 | 0.4900878 | -0.0563029 | 0.4948675 | 0.5639536 |
| 3.0056814 | 0.0270265 | 0.2688244 | 6.6336576 | 2.0913190 | 0.1791309 | 6.0033421 | 7.8525810 | 3.8636433 |
| 0.1870713 | 0.3026601 | -0.2170254 | 1.7691092 | 1.3058929 | 0.0802329 | 0.6245785 | 1.7636242 | -0.3470392 |
| -0.3277274 | -0.5569257 | -0.0277362 | -1.0884138 | -0.5644894 | 0.1948408 | 0.2019097 | -1.5834968 | -0.2064458 |
| -0.0620776 | -0.6423254 | 0.1385903 | 0.2088249 | -0.9743207 | 0.6505777 | 0.5408075 | 0.0079188 | 0.4607039 |

| | | | | | | | | |
|---|---|---|---|---|---|---|---|---|
| -0.757931 | -0.708513 | -0.713273 | -1.440046 | -3.010632 | -0.323854 | -1.448115 | -2.096919 | -1.715141 |
| -0.307454 | -0.042675 | -0.285934 | -0.382522 | 0.1179772 | -0.103278 | -0.179838 | 0.1192303 | -0.568748 |
| -0.279114 | -0.679955 | -0.178015 | -0.908956 | -1.042358 | 0.1280694 | -0.006811 | 0.0882094 | -0.027582 |
| -0.214411 | -0.293632 | -0.132740 | -0.239605 | -0.094925 | -0.228651 | -0.654785 | -0.212014 | -0.930637 |
| -0.529067 | -0.378782 | -0.479948 | -1.023618 | -0.875312 | -0.279764 | -1.412488 | -1.604427 | -1.127830 |
| 0.0267655 | -0.883990 | 0.6123059 | 0.4015763 | -0.407718 | -0.133428 | -0.441487 | -1.218373 | -0.860427 |
| -0.559135 | -0.446443 | -0.744537 | -0.467788 | -1.361051 | 0.2238637 | -2.057016 | -1.385754 | -2.393960 |
| -0.185763 | -0.042474 | 0.2850572 | 0.1822702 | 0.4226909 | -0.097962 | 0.0797058 | 0.0491896 | 0.1134022 |
| -0.446916 | -0.850822 | -0.080687 | -0.071924 | 0.1114478 | -0.134313 | 0.3886540 | -0.255878 | 0.3220353 |
| -0.089921 | -0.170098 | -0.138402 | 0.2527367 | 0.4490068 | -0.096075 | 0.4015608 | 1.3587943 | -0.039282 |
| -0.002837 | -0.262323 | -0.145655 | -0.306195 | -0.665666 | 0.2119213 | -0.539129 | -1.443018 | -0.846476 |
| -0.499731 | -0.320264 | -0.841899 | -1.020472 | -0.522285 | -0.445003 | -2.703999 | -2.086647 | -2.672980 |
| -0.118421 | -0.201424 | -0.209974 | -0.393712 | -0.826391 | -0.038532 | -0.726439 | -4.922261 | 0.0172413 |
| -0.217497 | -0.485302 | -0.528651 | -0.719166 | -0.993839 | -0.049226 | -0.911023 | -1.573068 | -0.770714 |
| -0.319250 | -0.107411 | -0.152801 | -0.358791 | -0.386563 | -0.241089 | -1.188600 | -0.560943 | -1.121581 |
| -0.053327 | -0.050524 | 0.1183726 | -0.097118 | 0.1024815 | -0.245162 | 0.1794243 | -0.159538 | 0.2754850 |
| -1.302021 | -0.970991 | -0.708663 | -1.284838 | -0.964414 | -0.338217 | -1.591873 | -1.298871 | -1.775864 |
| -0.136981 | -0.168665 | 0.1307786 | -0.005355 | -0.012163 | -0.129983 | 0.2520464 | 0.4033984 | 0.7418787 |
| -0.275823 | -0.662957 | -0.450249 | -0.349057 | -0.745517 | -0.775039 | -1.338267 | -1.999845 | -2.076984 |
| -0.160389 | -0.168454 | -0.373797 | -0.239880 | 1.1902796 | -1.331952 | 1.1488738 | 2.0496344 | 0.8703233 |
| -0.210403 | -0.286407 | -0.061855 | -0.151961 | -0.754647 | -0.298536 | -0.657488 | -1.385566 | -0.929731 |
| -0.180386 | 0.2210460 | -0.092252 | -0.238618 | 0.1721415 | -0.308363 | -0.965521 | -0.776570 | -1.038568 |
| -0.275588 | -0.355404 | -0.141577 | -0.349368 | -0.237277 | 0.1171039 | -0.270319 | -0.020338 | -0.059269 |
| -0.235603 | -0.523110 | -0.203753 | -0.653993 | -0.970812 | -0.546833 | -1.087662 | -1.322089 | -1.363698 |
| -0.219319 | -0.265065 | -0.392307 | 0.0484463 | 0.0939970 | -0.905101 | -1.027417 | -2.207795 | -1.480347 |
| -0.289759 | -0.113006 | -0.423449 | -0.631313 | -0.590486 | -0.720336 | -1.338729 | -0.808927 | -1.342158 |
| -0.000290 | 0.0208111 | -0.064645 | -0.199197 | -0.067351 | 0.2615765 | -0.637579 | -0.484211 | -1.030622 |
| -0.050492 | -0.703334 | -0.120575 | -0.078488 | -0.262434 | -0.310496 | 1.2826970 | 1.1582440 | 1.1966793 |
| -0.301069 | -0.538915 | -0.229198 | -0.546150 | -0.362012 | -0.201022 | -1.287225 | -1.407433 | -0.996110 |
| -0.727162 | -0.434252 | -0.764033 | -0.552536 | -0.254985 | -0.701256 | -1.413746 | -1.701644 | -1.939404 |
| -0.627367 | -0.742324 | -0.648177 | -0.903642 | -1.715153 | -0.557845 | -1.052971 | -2.417953 | -0.973226 |
| -0.085085 | -0.187996 | -0.068330 | -0.177720 | -1.135042 | -0.297560 | -1.960920 | -1.578972 | -2.176477 |
| -0.598080 | -0.411693 | -0.104476 | -0.350488 | 0.0627857 | -0.027315 | -0.280155 | -0.826598 | -0.160509 |
| 0.1909038 | 0.0711332 | 0.1784619 | 0.1788123 | -0.277868 | 0.0250694 | -0.147183 | 0.2907165 | -0.521985 |
| -0.023860 | -0.466819 | -0.156520 | -0.672154 | -1.529064 | -0.625458 | -0.086346 | -1.810512 | -0.269202 |
| -0.447121 | -0.410298 | -0.313103 | -0.632749 | -0.694441 | -0.713856 | -1.690601 | -1.300630 | -2.113596 |
| -0.110531 | -0.108949 | -0.136792 | -0.296742 | -0.370158 | 0.2918017 | -0.789329 | -0.563765 | -1.129905 |
| -0.428593 | -0.253733 | -0.356352 | -0.326202 | 0.0004300 | -0.605156 | -2.179080 | -2.510604 | -2.777134 |
| -0.527458 | -0.553440 | -0.386618 | -0.526154 | -0.958475 | -0.363268 | -0.729815 | -0.889183 | -0.514020 |
| -0.665386 | -0.560231 | -0.177787 | -0.776919 | -0.664698 | -0.743094 | -0.865839 | -0.589666 | -0.997531 |
| -0.081863 | 0.0020660 | -0.642450 | -0.283029 | 0.3960071 | -0.953811 | -1.676157 | -0.409713 | -2.521318 |
| -0.153762 | 0.1666224 | -0.030112 | -0.046413 | -0.063740 | -0.805934 | -1.492782 | -1.670913 | -1.892996 |
| -0.223623 | -0.251526 | -0.194012 | -0.398427 | -0.587544 | -0.473207 | -1.575307 | -1.762977 | -2.141185 |
| -0.490988 | -0.558293 | -0.446979 | -0.562704 | -0.787888 | -0.397035 | -0.922501 | 0.2530503 | -1.039480 |
| -0.038592 | -0.022254 | -0.130127 | -0.107473 | 0.0602443 | -0.152746 | -0.055436 | -0.452311 | 0.0717110 |
| -0.098876 | -0.081937 | -0.427671 | 0.2620736 | -0.087554 | -0.239683 | -0.915729 | -0.526765 | -1.613278 |
| -0.520359 | -0.468529 | -0.459293 | 0.0176639 | -0.032536 | -0.848229 | -1.226468 | -0.808937 | -1.654619 |
| -0.524810 | -0.485850 | -0.583232 | -0.147831 | -0.371464 | -0.253320 | -0.611638 | 1.0737709 | -1.054445 |
| -0.345429 | -0.733504 | -0.251160 | -0.522708 | -0.621532 | 1.3826680 | -0.136329 | -1.946919 | -0.103739 |
| -0.941299 | -0.627373 | -0.725383 | -1.014734 | -1.251467 | -0.817275 | -0.882736 | -1.129155 | -1.265152 |

| | | | | | | | | |
|---|---|---|---|---|---|---|---|---|
| 0.0459873 | 0.1161343 | 0.0259276 | 0.0318367 | 0.1596033 | -0.018275 | -2.140635 | -1.466086 | -2.537462 |
| -0.511340 | -0.860699 | -0.921811 | -1.754598 | -1.705010 | -0.915786 | -0.957933 | -1.256530 | -0.828104 |
| -0.353353 | -0.333474 | -0.497223 | -0.646494 | -0.133633 | -0.494533 | -0.482559 | 0.0816512 | -0.946172 |
| -0.190859 | -0.060676 | -0.471065 | 0.0501903 | -0.385516 | 0.2013864 | 1.5240034 | 1.7426837 | 1.1443664 |
| -0.548935 | -0.548776 | -0.206093 | -0.908096 | -0.764175 | -0.513999 | -0.903624 | -1.670679 | -1.121417 |
| -0.194980 | -0.123996 | -0.004471 | -0.080698 | -0.025675 | 0.0813356 | 2.7099264 | 2.3821933 | 2.8447743 |
| -0.229764 | -0.310601 | -0.319460 | -0.474075 | -0.518827 | -0.362507 | -0.954476 | -0.885882 | -1.338088 |
| -0.639631 | -0.361603 | -0.850307 | -1.191127 | -1.011696 | -0.820685 | -0.195651 | -0.802128 | -0.824947 |
| -0.559381 | -0.846480 | -0.653845 | -1.124967 | -1.588956 | -0.322785 | -1.074303 | -1.420246 | -0.421956 |
| -0.605828 | -0.721067 | -0.455221 | -0.196203 | -0.472933 | -0.640598 | -1.335614 | 0.4827689 | -1.092483 |
| 0.0299515 | -0.166993 | 0.0247821 | 0.3085471 | 0.6885936 | -0.285602 | -0.504004 | 0.0073356 | -1.012405 |
| -0.616668 | -0.886209 | -0.340254 | -0.539486 | -0.744740 | 0.0547579 | -0.384174 | -0.059474 | -0.509735 |
| -0.391751 | -0.331543 | -0.326245 | -0.752038 | -0.368519 | -0.418142 | -1.523111 | -3.110391 | -1.103465 |
| -0.388818 | -0.562827 | 0.0050551 | -0.178833 | -0.545233 | -0.343531 | -0.668844 | -0.546714 | -0.897010 |
| -0.327746 | -0.181619 | -0.103381 | -0.356965 | -0.474194 | 0.2238415 | -0.483071 | -0.266428 | -0.188317 |
| 0.1552994 | -0.146596 | 0.4373866 | 0.1508043 | -0.721921 | -0.729268 | -0.888040 | -1.741182 | -0.714730 |
| -0.748224 | -0.652346 | -0.395979 | -0.777635 | -0.117008 | -0.573974 | 0.8359883 | 0.2674123 | 1.0494714 |
| -0.527557 | -0.697955 | -1.123272 | -1.713612 | -1.929845 | -0.416633 | -0.936494 | -1.075452 | -1.246976 |
| -0.565421 | -0.266139 | -0.312807 | -0.517956 | -0.240204 | -0.616038 | -1.096770 | -0.310327 | -1.695851 |
| -0.417322 | -0.509434 | -0.403730 | -0.732666 | -0.922483 | 0.1154963 | -1.506725 | -1.785314 | -2.670989 |
| -0.196596 | -0.353665 | -0.070496 | 0.0047263 | 0.0754315 | 0.2532263 | 1.0336444 | 1.9315665 | 0.8622968 |
| -0.289876 | -0.430528 | -0.397118 | -0.496884 | -0.155878 | -0.607812 | -1.033278 | -0.774275 | -1.031042 |
| -0.395325 | -0.499379 | -0.288723 | -0.851715 | -1.518776 | -0.913224 | -1.560293 | -2.274350 | -0.728136 |
| -0.299078 | -0.265213 | 0.0192665 | -0.598959 | -0.811078 | -0.419833 | -1.100098 | -1.028870 | -1.325405 |
| -0.295203 | -0.184422 | -0.194689 | -0.669772 | -1.060650 | -0.050389 | -0.736366 | -0.683212 | -0.334169 |
| -0.392879 | -0.119636 | -0.174420 | -0.590358 | -0.004870 | -0.285590 | -1.100000 | -0.997910 | -0.859413 |
| -0.697118 | -0.653695 | -0.547865 | -0.862121 | -0.763982 | -0.158911 | -0.551604 | -0.329484 | -0.695264 |
| -0.328115 | -0.414682 | -0.364071 | -0.645331 | -0.482352 | -0.103688 | -0.964697 | 0.2756138 | -1.280595 |
| -0.273292 | -0.853256 | -0.376708 | -0.973471 | -1.389810 | -1.536111 | -1.519260 | -1.959192 | -1.717851 |
| -0.002273 | -0.643452 | -0.077931 | 0.1422190 | -0.166564 | -0.245114 | 0.0457897 | 1.4309026 | -0.414252 |
| 0.3004397 | 0.1099368 | -0.140855 | 0.8405126 | 1.4694176 | -1.093114 | 1.3503887 | 3.2600610 | 0.7344600 |
| -0.774306 | -0.735501 | -0.959912 | -1.055339 | -0.815572 | -0.576895 | -0.700959 | -0.227611 | -1.108038 |
| -0.078591 | 0.0440398 | -0.079952 | -0.260863 | -0.029082 | -0.159862 | -0.337543 | 0.2108614 | -0.596265 |
| 0.0348873 | 0.3393691 | -0.157586 | -0.034224 | 0.3084450 | -0.227193 | -0.517151 | 0.2392202 | -0.912127 |
| 0.2988498 | 0.7564350 | 0.0968202 | 0.4030054 | 0.7300002 | -0.013008 | 1.2594271 | 1.4702648 | 1.1831078 |
| -0.713908 | -0.358694 | -0.413340 | -0.893350 | -0.748645 | 0.2859720 | -0.790823 | 0.0531028 | -1.176253 |
| -0.002120 | -0.387405 | -0.211379 | 0.3130507 | 0.6853427 | -0.237144 | -1.215577 | -1.151741 | -1.227196 |
| -0.351573 | -0.433863 | -0.369669 | -0.226866 | -0.576767 | -0.439342 | -1.439396 | -0.758284 | -2.498882 |
| -0.284985 | -0.311354 | -0.347892 | -0.390062 | -0.135654 | -0.295834 | -1.083560 | -0.448169 | -1.715724 |
| -0.310806 | -0.230627 | -0.247822 | -0.531314 | -0.674437 | 0.0388404 | -1.630818 | -1.640271 | -1.957718 |
| -0.533774 | -0.380223 | -0.841746 | -0.835955 | -1.101453 | -0.634140 | -1.008965 | -1.437324 | -0.858877 |
| -0.409757 | -0.528478 | -0.553994 | -0.434746 | -0.252957 | -0.515885 | -0.566858 | 0.7649209 | -0.363295 |
| -0.093296 | 0.0045113 | -0.305686 | -0.099952 | 1.0527258 | -1.246127 | -1.636969 | -0.454943 | -2.404396 |
| -0.398933 | -0.235343 | -0.374181 | -0.156856 | -0.104683 | -0.059463 | -0.697141 | 0.2335765 | -1.164907 |
| -0.113287 | -0.227375 | -0.146596 | -0.332111 | -0.273185 | 0.1576709 | -0.574239 | -0.648879 | -0.244846 |
| -0.382166 | -0.454648 | -0.605656 | -0.523170 | -0.478499 | -1.058311 | -1.219440 | -0.982666 | -1.952996 |
| -0.324919 | -0.410896 | -0.361485 | -0.645670 | -1.011966 | -0.165238 | -0.383727 | -0.984057 | -0.493468 |
| -0.556187 | -0.507560 | -0.405499 | -1.058629 | -1.335917 | -0.103900 | -1.064916 | -1.007992 | -0.730391 |
| -0.012285 | -0.273209 | 0.0812104 | -0.277557 | -1.196801 | 0.1968130 | -0.198193 | -1.059171 | 0.1317780 |
| -0.271791 | -0.094224 | -0.258753 | -0.293956 | -0.343662 | -0.205147 | -1.797777 | -0.752925 | -2.568204 |

| | | | | | | | | |
|---|---|---|---|---|---|---|---|---|
| -0.066399 | 0.1125726 | 0.1066773 | -0.041819 | 0.3432884 | 0.0951426 | -3.093055 | -1.642552 | -3.178150 |
| -0.758268 | 0.1658219 | -1.145408 | -1.149566 | -1.606227 | -0.994482 | -0.876480 | -1.327163 | -1.296678 |
| -0.546909 | -0.780000 | -0.285573 | -0.475997 | -0.761452 | -1.043349 | -0.934131 | -1.522006 | -1.240750 |
| -0.720176 | -0.757110 | -0.580908 | -1.485780 | -2.029942 | -0.992094 | -1.173797 | -1.880020 | -0.611268 |
| -0.649711 | -0.899433 | -0.666306 | -1.051364 | -1.102639 | -0.666553 | -1.397788 | -2.041497 | -1.646536 |
| -0.261258 | -0.274464 | -0.493368 | -0.326450 | -0.601063 | -0.296234 | -0.422835 | -0.419423 | -0.225947 |
| 0.1508656 | 1.0885103 | -0.362964 | 1.0703746 | 2.1956960 | -0.137822 | 0.8428197 | 1.6733361 | 0.3484726 |
| -0.666612 | -0.465627 | -0.656387 | -0.750106 | -0.217256 | -0.089189 | 0.2451811 | 0.0453361 | 0.7621346 |
| -0.205571 | -0.357118 | -0.190783 | -0.341010 | -0.877262 | -0.022690 | -3.051522 | -3.627536 | -3.699718 |
| -0.275591 | -0.137108 | -0.328636 | -0.160258 | 0.0909891 | -0.819077 | -0.770177 | 0.3795827 | -1.081835 |
| -0.219088 | -0.334884 | -0.084912 | -0.057275 | -0.291334 | -1.228144 | -1.165571 | -0.687516 | -1.741813 |
| -1.019483 | -1.081896 | -0.749781 | -1.161065 | -0.643444 | -0.531791 | -2.096241 | -0.464949 | -2.636044 |
| 0.1691923 | -0.003202 | 0.0699461 | 0.1242203 | -0.118494 | -0.034706 | 0.2166073 | -0.844941 | 0.2165643 |
| -0.267198 | 0.1313147 | -0.327234 | -0.257979 | -0.160600 | -0.449829 | -1.430133 | -1.583370 | -1.674852 |
| -0.086442 | -0.279959 | 0.0021474 | -0.320959 | -1.513779 | 0.3643410 | -0.555441 | -2.777548 | -0.331799 |
| -0.340509 | -0.276957 | -0.223367 | -0.331886 | -0.288882 | -0.568602 | -0.723342 | -0.855653 | -0.554557 |
| -0.193620 | -0.433753 | -0.225655 | -0.499930 | -1.254024 | 0.1292026 | -3.409320 | -3.355669 | -4.254052 |
| -0.322544 | -0.295170 | -0.293317 | -0.236810 | -0.240214 | -0.202936 | -0.976498 | -0.325282 | -0.596726 |
| -0.082224 | -0.258581 | 0.0233334 | -0.784383 | -1.505755 | 0.0337664 | -0.433559 | -0.593755 | -0.670476 |
| -0.008919 | -0.203875 | -0.007781 | -0.232575 | -0.442737 | 0.4660020 | -1.746166 | -1.176636 | -1.563208 |
| -0.684377 | -0.669762 | -0.318719 | -0.847616 | -1.225256 | -0.230027 | -1.479376 | -1.780449 | -1.830597 |
| -0.071633 | -0.144658 | -0.211545 | -0.187389 | -0.117773 | -0.269363 | -0.255204 | -0.149544 | -0.235067 |
| -0.904848 | -0.967052 | -0.436203 | -1.933524 | -3.024244 | -0.524910 | -1.066155 | -4.003610 | -0.618724 |
| -0.240969 | -0.202189 | -0.367047 | -0.153499 | 0.1666367 | -0.096325 | -0.824390 | -0.155220 | -1.326908 |
| 0.0003037 | -0.145064 | 0.0061163 | -0.351982 | -1.027701 | -0.121067 | 0.0040742 | -0.593590 | 0.1764504 |
| -0.575685 | -0.859274 | -0.549713 | -0.561902 | -0.302588 | -1.377617 | -0.980064 | -0.244821 | -1.170441 |
| -0.205634 | -0.197346 | -0.093446 | -0.222678 | -0.566303 | 0.1784246 | -0.565153 | -1.475348 | -0.905392 |
| -0.353986 | -0.570452 | -0.409642 | -0.537039 | -0.721371 | -0.286319 | -0.807868 | -0.513502 | -0.947229 |
| -0.109229 | -0.467964 | -0.474513 | -0.096155 | 0.7198826 | -0.363995 | -0.956755 | 0.4311845 | -1.040419 |
| -0.213069 | 0.1237525 | 0.0356512 | -0.085834 | 0.8659709 | -0.373346 | -1.985304 | -0.710119 | -2.650572 |
| -0.583725 | -0.676314 | -0.515222 | -0.276210 | -0.709251 | -0.418337 | -0.439517 | -0.389975 | -0.459371 |
| -0.137123 | -0.066700 | -0.058040 | -0.257728 | -0.182227 | 0.3764951 | -1.284120 | -1.079004 | -2.197159 |
| -0.611009 | -1.005919 | -0.770310 | -0.199851 | -0.120071 | -1.139171 | -0.078380 | 2.2083349 | -0.018545 |
| -0.314307 | -0.495051 | -0.410250 | -0.561913 | -1.079502 | -0.088284 | -0.281363 | -1.219943 | -0.332303 |
| -0.191882 | -0.149897 | -0.249980 | -0.224715 | -0.496496 | -0.125346 | -0.507948 | -0.495295 | -0.315204 |
| -0.466067 | -0.511166 | -0.355643 | -0.762908 | -0.978335 | -0.932598 | -1.409899 | -0.955000 | -2.227133 |
| -0.896606 | -0.859240 | -0.896977 | -0.774214 | -1.444877 | 0.0052635 | -0.711593 | -0.902316 | -0.304625 |
| -0.164053 | -0.106266 | -0.168276 | -0.202955 | -0.090743 | -0.077283 | -0.345048 | -0.118538 | -0.263647 |
| -0.158818 | 0.0966758 | -0.273789 | -0.092115 | 0.0306785 | -0.255209 | 0.6537457 | 0.6834675 | 0.3770021 |
| -0.152759 | -0.515030 | -0.308389 | -0.386243 | -1.738683 | -0.134345 | -0.807169 | -0.588679 | -0.710950 |
| -0.108123 | -0.000267 | -0.185566 | -0.177605 | 0.4290633 | 0.0095216 | -2.192548 | -0.493570 | -3.723246 |
| -0.395840 | -0.513559 | -0.314629 | -0.974869 | -1.098176 | 0.1724882 | -1.076060 | -0.806613 | -1.899470 |
| -0.406971 | -0.807711 | -0.612703 | -1.074921 | -1.024598 | -0.277097 | -0.779522 | -0.526343 | -0.746715 |
| -0.744137 | -0.486317 | -0.080599 | -0.492402 | -0.746668 | -0.620978 | -1.306200 | -1.234514 | -1.518881 |
| -0.514159 | -0.550010 | -0.580925 | -0.455690 | -0.380051 | -0.397200 | -0.931280 | -0.374519 | -1.272204 |
| -0.571428 | -0.898745 | -0.685196 | -0.835355 | -0.996776 | -0.441204 | -2.174659 | -1.823159 | -2.526309 |
| -0.025064 | -0.288421 | 0.0077116 | -0.597493 | -0.708309 | -0.494082 | -1.277341 | -1.371518 | -1.041080 |
| -0.772702 | -0.999400 | -0.921514 | -0.982923 | -1.144699 | -1.096164 | -1.537422 | -1.625596 | -2.220764 |
| -0.707073 | -0.666369 | -0.370880 | -0.428896 | -0.554033 | -0.294413 | -1.878543 | -0.945276 | -1.740842 |
| -0.649747 | -0.955985 | -0.786110 | -0.839045 | -1.491666 | -0.747843 | -1.588290 | -1.475515 | -1.262046 |

| | | | | | | | | |
|---|---|---|---|---|---|---|---|---|
| -0.7842349 | -0.5767309 | -0.4471487 | -1.1929704 | -0.3496867 | -0.5703044 | -0.3464598 | 0.2378463 | -0.6646246 |
| -0.3438434 | -0.9587569 | -0.6243823 | -0.5120564 | 0.0444243 | -0.9852736 | -0.7305429 | -0.5594038 | -0.8768089 |
| -0.2265712 | -0.0744185 | -0.3972981 | -0.1812360 | 0.0134359 | -0.4809668 | -0.4107213 | 0.2119244 | -0.5407372 |

| PG_471 | PG_472 | PG_473 | PG_474 | PG_475 | PG_476 | PG_477 | PG_478 | PG_479 |
|---|---|---|---|---|---|---|---|---|
| -1.733595 | -1.902765 | 0.0156203 | -0.207479 | -1.197113 | -0.184321 | -0.009701 | -0.948249 | -0.169201 |
| -1.214063 | -1.749956 | -0.301679 | -1.086298 | -1.548443 | -0.581573 | -0.797353 | -1.659858 | -0.440120 |
| 0.6086349 | -1.345745 | -0.660989 | -0.490963 | -1.329186 | -1.027439 | -0.652250 | -1.390529 | -0.221782 |
| -2.217984 | -0.787508 | -0.445005 | -0.345550 | 0.3516294 | -0.353371 | -0.393984 | 0.3285494 | -0.116891 |
| -1.890396 | -1.069404 | 0.0767620 | -0.176909 | -0.918699 | -0.026461 | 0.0389326 | -0.882296 | 0.1477420 |
| -1.578057 | -2.432560 | 0.0913196 | -0.424211 | -0.849038 | 0.0354002 | -0.467394 | -1.231426 | -0.675684 |
| -1.583260 | -0.963003 | -0.125900 | -0.422746 | -0.153954 | -0.317166 | -0.858065 | -0.205665 | -0.174560 |
| -0.059215 | -0.339675 | 0.2363404 | -0.071077 | -0.434063 | 0.0537848 | -0.021423 | -0.598415 | -0.507650 |
| -0.259575 | 0.3889758 | -0.431839 | -0.205964 | 0.0816417 | -0.579056 | 0.1898381 | 0.2021574 | 0.0230127 |
| -0.771289 | -0.896122 | -0.380356 | -0.670879 | -0.650582 | -0.495994 | -0.793497 | -0.446059 | 0.0179353 |
| -1.551839 | -0.936712 | -0.247142 | -0.329931 | -0.506231 | -0.102311 | -0.298688 | -0.781872 | -0.114932 |
| -2.136265 | -1.231905 | 0.0126395 | -0.205561 | -0.507553 | -0.012600 | -0.668104 | -0.687186 | -0.210822 |
| -1.980216 | -2.926483 | -0.733742 | -0.643386 | -0.854318 | -0.831855 | -1.318724 | -1.811137 | -0.929305 |
| 1.6668291 | 3.8337056 | -0.162322 | 1.2356275 | 3.1044646 | -0.208660 | 2.6650323 | 3.7512522 | 0.0976428 |
| -2.788030 | -0.623403 | -0.282799 | -0.537073 | 0.2934997 | -0.113449 | -0.374882 | 0.6481690 | 0.0731919 |
| 2.1909643 | 2.7272956 | -0.090992 | 1.6803825 | 2.1394523 | -0.063020 | 2.2882065 | 2.3180324 | -0.083232 |
| -0.300486 | -1.176469 | -2.142950 | -2.131801 | -1.987527 | -1.885292 | -2.883653 | -2.477639 | -0.976364 |
| -2.312138 | -2.073016 | 0.0573222 | -0.075903 | -0.193112 | -0.081280 | -0.449692 | -0.303716 | -0.456720 |
| -1.901950 | -2.170734 | -0.362071 | -1.716270 | -2.479855 | -0.507420 | -2.047135 | -2.728107 | -1.235469 |
| -1.295750 | 0.1801539 | -0.757125 | 0.1646934 | -0.212212 | -0.879592 | -1.090913 | -0.570666 | -0.621770 |
| -0.688139 | 0.5435622 | -0.819940 | -0.029224 | 0.1066573 | -0.703156 | 0.0575892 | 0.1964875 | -0.866607 |
| -0.606105 | -0.431616 | -0.496653 | -0.132997 | -0.676016 | -0.604551 | -0.478710 | -0.506015 | -0.440972 |
| 1.0493107 | 1.0207661 | -0.284911 | 0.3256839 | 0.7887322 | -0.390659 | 0.7510426 | 0.7066380 | -0.551627 |
| -2.475609 | -2.705936 | -0.211267 | -0.521290 | -1.440969 | -0.251619 | -1.095788 | -1.448757 | -0.533381 |
| -1.610404 | -1.870332 | -0.109049 | -0.721894 | -2.051195 | -0.326228 | -1.275679 | -2.211693 | -0.135271 |
| 0.9226017 | 0.9320019 | -0.271017 | 0.5800469 | 0.4672344 | -0.231959 | 0.6580710 | 0.5701337 | 0.1147311 |
| -2.543014 | -1.673869 | 0.0549723 | -0.035090 | -0.142148 | -0.032988 | -0.421464 | -0.080584 | 0.1840353 |
| -1.965936 | -2.312590 | -0.889170 | -0.893706 | -1.160994 | -0.794366 | -0.965973 | -1.544164 | -1.051779 |
| -1.054567 | -2.596370 | 0.0168587 | -0.487924 | -1.171303 | -0.011828 | -0.886383 | -2.050792 | -0.754460 |
| -2.338745 | -1.321283 | 0.0553379 | -0.226132 | -0.519334 | -0.055693 | -0.790548 | -0.672116 | 0.1439047 |
| -0.591604 | -2.122669 | -0.734248 | -0.383944 | -0.747479 | -0.689186 | -0.529584 | -0.499294 | 0.6683279 |
| 0.6363168 | 0.7984830 | -0.126790 | -0.344439 | -0.518286 | -0.110867 | 0.2495719 | -0.431644 | -0.242453 |
| -1.188111 | -1.803316 | -0.332241 | -0.085476 | -0.169191 | -0.286668 | 0.2608842 | -0.246943 | -0.459541 |
| -1.212272 | -2.068647 | -0.392123 | -0.732655 | -1.429806 | -0.570049 | -1.246572 | -2.178435 | -0.811094 |
| -1.234248 | -2.147026 | -0.084666 | -0.074881 | -0.066637 | -0.011870 | -0.134221 | -0.168602 | 0.1112736 |
| -1.790802 | -1.402051 | -0.230852 | -0.882443 | -0.097228 | -0.789320 | -0.897316 | -0.353339 | -0.442220 |
| -1.364409 | -1.635517 | -0.170450 | -0.491499 | -0.970154 | -0.104735 | -0.976712 | -1.740320 | -0.511981 |
| 3.2611999 | 1.3687039 | -0.213288 | 1.9844149 | 1.9948225 | 0.0610000 | 3.4469001 | 1.0701497 | -0.094772 |
| -1.279204 | -0.383312 | -0.399358 | -0.289465 | -1.021549 | -0.238454 | 0.0930011 | 0.3481962 | -0.406934 |
| -2.610830 | -1.458488 | 0.3024881 | -0.766746 | -1.618655 | 0.0645253 | -1.055656 | -1.453460 | -0.438586 |
| 0.8217076 | 0.5447458 | 0.1497746 | -0.014924 | 0.3551874 | -0.264491 | 0.2558300 | 0.2309089 | -0.347830 |
| -0.672238 | -2.286620 | -0.272326 | -0.696706 | -1.860647 | -0.340490 | -1.081703 | -1.743491 | -0.746777 |
| -1.406286 | -0.898152 | 0.1640501 | 0.0233658 | 0.0817235 | -0.057970 | 0.2891443 | 0.2845969 | -0.685464 |
| -2.259832 | -1.963296 | 0.1931606 | -0.067325 | -0.265167 | 0.2414280 | -0.263345 | -0.386965 | -0.098788 |
| 1.9329253 | 1.6121659 | -0.331425 | 0.6376711 | 1.1425104 | -0.259976 | 0.9148632 | 1.1487732 | -0.239353 |
| 1.4321982 | 5.6514297 | -0.334674 | -0.079678 | 2.7971528 | 0.3624021 | 0.4498606 | 5.3565929 | -0.422023 |
| -0.051525 | 0.0825435 | -0.035434 | 0.5157440 | -0.244879 | 0.1199531 | 0.1147084 | 0.2688332 | 0.1360964 |

| | | | | | | | | |
|---|---|---|---|---|---|---|---|---|
| -0.948307 | -0.177071 | -0.125967 | -0.184135 | 0.3275258 | -0.279019 | -0.381586 | 0.2804232 | -0.463753 |
| -1.006387 | -1.569440 | -0.453723 | -0.406762 | -2.152390 | -0.524452 | -0.596557 | -2.700540 | -0.350121 |
| -1.310395 | -0.228586 | -0.668158 | -0.454607 | -0.406820 | -0.571503 | -0.214576 | -0.076113 | -1.100157 |
| 2.1229149 | 3.6238043 | 0.5254236 | 1.3741452 | 4.4585104 | 0.2890800 | 1.1997630 | 4.8362229 | -0.216892 |
| -0.260781 | -0.334655 | -0.533091 | -0.347488 | 0.0365820 | -0.359824 | -0.056214 | -0.105747 | -0.726534 |
| 0.5869820 | 0.6782199 | -0.394588 | -0.234851 | -0.111410 | -0.279946 | -0.314013 | -0.020133 | -0.831734 |
| -0.976496 | -1.662699 | -0.318696 | -0.899639 | -0.932831 | -0.756439 | -1.715033 | -1.730231 | -0.668780 |
| 1.5316097 | 2.0495037 | -0.189771 | 0.5796662 | 2.7501533 | -0.138148 | 1.4903954 | 2.8269050 | -0.464244 |
| 3.8443572 | 0.9950069 | -0.952741 | 0.9807643 | 0.7622413 | -0.842362 | 1.8864348 | -0.038395 | 0.1894949 |
| -0.310316 | -0.209835 | -0.583999 | -0.634251 | -0.712311 | -0.779785 | -1.062965 | -0.620732 | -0.771196 |
| -1.254425 | -1.440674 | -0.540279 | -0.929206 | -1.275210 | -0.505339 | -1.271126 | -1.346172 | -0.418950 |
| 2.4742926 | 3.5573832 | 0.2435854 | 2.0972608 | 1.9309802 | 0.2754127 | 2.5505716 | 2.3138148 | 0.2257146 |
| -1.194920 | 0.5647435 | 0.3161983 | 1.6525144 | 2.0201662 | 0.1989602 | 2.1759978 | 1.7245508 | -0.476163 |
| -1.557555 | -1.474014 | -0.831117 | -0.677763 | -1.269917 | -0.547381 | -0.781662 | -1.193287 | -0.375836 |
| -0.551915 | -1.220731 | -0.377193 | -1.012268 | -1.248660 | -0.463045 | -1.836232 | -1.791930 | -0.416181 |
| -1.400538 | -1.374146 | -0.640227 | -0.925923 | -0.436638 | -0.539160 | -1.773973 | -0.357697 | -0.657468 |
| -1.559947 | -1.920820 | -0.184223 | -0.293117 | -1.467208 | -0.372756 | -0.890903 | -1.553057 | -0.395481 |
| -0.733829 | -0.427046 | -0.193845 | -0.410960 | -0.403612 | -0.259494 | -1.004122 | -0.362859 | -0.382892 |
| -0.571470 | -1.108175 | -0.451989 | -0.725739 | -0.697626 | 0.0910430 | -0.934158 | -1.395865 | -0.006494 |
| -2.600951 | -1.892587 | 0.0184743 | 0.2057492 | 0.1769812 | -0.099787 | 0.2545930 | 0.1284160 | 0.3374800 |
| 0.0136711 | 0.5055368 | 0.1739967 | -0.205782 | 0.1785118 | -0.116547 | 0.1172215 | 0.0349227 | -0.325729 |
| -1.226262 | -2.638337 | -0.487068 | -0.735027 | -1.071411 | -0.610531 | -1.123454 | -1.532256 | -0.645572 |
| 1.2250596 | 1.0310000 | -0.056679 | -0.232414 | -0.002519 | -0.133398 | 0.1194799 | -0.139293 | -0.173643 |
| -2.924404 | -2.147233 | 0.4448432 | 0.6404316 | 0.9705426 | 0.4039934 | 0.0438266 | 0.8572868 | 0.6329252 |
| -1.446714 | -0.860020 | -0.963773 | -0.888517 | -1.099830 | -1.064398 | -1.067492 | -1.552863 | -1.073174 |
| -0.738666 | -0.313970 | -0.292849 | -0.464620 | -0.536885 | -0.514442 | -0.627692 | -0.308558 | -0.413640 |
| -2.315287 | -2.564933 | -0.250846 | -1.664908 | -1.802366 | -0.614725 | -1.626200 | -1.362465 | -0.433180 |
| 0.7663031 | 2.8612455 | 0.0246733 | 0.6691249 | 1.3077696 | 0.1714417 | 0.9117364 | 1.5093417 | -0.110838 |
| 0.2638319 | 1.7696617 | 0.0924167 | 0.1547137 | 1.6964463 | -0.112145 | 0.2674062 | 2.0213438 | -0.491431 |
| -1.129829 | -2.291269 | -0.202366 | -0.540532 | -1.668250 | -0.202779 | -0.958017 | -1.679864 | -0.100514 |
| -1.039419 | -0.562532 | -0.407092 | -0.392033 | 0.0082216 | -0.229918 | 0.1412107 | 0.0985384 | -0.714701 |
| -2.838373 | -0.803332 | -0.215956 | -0.184733 | -0.492818 | -0.167315 | -0.167751 | -0.215581 | 0.1363669 |
| -1.657239 | -1.165326 | -0.314002 | -0.729145 | -0.916633 | -0.363713 | -1.225434 | -0.827196 | -0.620474 |
| -1.503714 | -1.388802 | -0.229344 | -0.490441 | -0.908755 | -0.425526 | -0.798689 | -1.336502 | -0.572099 |
| 1.1403694 | 1.5542086 | -0.379174 | 0.7627899 | 1.1037078 | -0.167618 | 0.8190956 | 1.0251097 | -0.524530 |
| 0.5445994 | -0.444421 | 0.4481644 | 0.4162712 | -0.678719 | 0.3785644 | 0.0107672 | -0.777077 | -0.787824 |
| -0.146473 | -0.065348 | -0.629222 | -0.642016 | -1.025929 | -0.450243 | -0.829069 | -0.461589 | 0.1189981 |
| -1.635151 | -2.098282 | -0.141710 | -0.265231 | -1.352045 | -0.184141 | -0.682072 | -1.764912 | -0.148892 |
| 0.2803066 | -0.498394 | 0.2132225 | -0.254399 | -1.536500 | 0.0664750 | -0.807131 | -1.531460 | -0.055861 |
| -0.787364 | -1.583956 | -0.521998 | -0.825860 | -1.004369 | -0.584429 | -1.110443 | -1.282301 | -0.602935 |
| -0.653593 | -0.712779 | -0.825050 | -0.666897 | -1.035213 | -0.679696 | -0.576064 | -1.140423 | -0.654640 |
| -1.184873 | -1.195276 | 0.3958670 | -0.064882 | -0.142726 | 0.3747342 | -0.739277 | -0.860308 | 0.3988232 |
| 4.0600438 | 2.6578663 | 0.0043518 | 2.2676469 | 2.3668558 | 0.5717658 | 2.6967582 | 1.7763428 | -0.491869 |
| -1.079957 | -0.787300 | -0.175202 | -0.256426 | -0.478860 | -0.179995 | -0.370118 | -0.410721 | -0.174767 |
| -2.351506 | -1.741904 | -0.081734 | -0.482242 | -0.235198 | -0.005778 | -0.492408 | -0.661556 | -0.222054 |
| 0.1759951 | 0.2056184 | -0.740874 | -0.642288 | -0.522967 | -0.429126 | -0.669272 | -0.626355 | -1.152457 |
| 6.9431678 | 8.5987694 | -0.106129 | 6.6656790 | 7.2342206 | -0.147229 | 8.2803190 | 8.2458990 | -0.062506 |
| 1.1611311 | 1.9250337 | 0.1043631 | 0.9405703 | 1.5535941 | 0.0945596 | 1.4321656 | 1.5722521 | -0.115607 |
| 0.3773887 | -0.141447 | -0.017935 | -0.285489 | -1.333579 | -0.157290 | -0.503512 | -1.200624 | -0.054167 |
| 0.1966437 | -0.170432 | -0.345008 | -0.043403 | 0.6373908 | -0.297191 | 0.1509073 | 0.4449798 | -0.409222 |

| | | | | | | | | |
|---|---|---|---|---|---|---|---|---|
| -1.981604 | -1.307500 | -0.402307 | -0.282684 | -0.982770 | -0.252564 | -0.102925 | -0.816991 | 0.1515863 |
| -0.611678 | -0.018728 | -0.267754 | -0.460347 | 0.0032367 | -0.269130 | -0.389288 | 0.2627915 | -0.804208 |
| -0.309553 | -0.062687 | -0.520504 | -0.617165 | -0.235925 | -0.381522 | -0.583165 | -0.178971 | -0.101449 |
| -0.803097 | -0.017411 | -0.127411 | -0.024883 | -0.020749 | -0.178230 | -0.024704 | 0.0855427 | -0.293958 |
| -1.898573 | -1.516417 | -0.150131 | -0.676132 | -0.941193 | -0.190197 | -1.360348 | -1.127232 | -0.308092 |
| -0.669158 | -1.090780 | -0.452189 | -0.457179 | -0.063890 | -0.432693 | -0.658696 | 0.4120996 | -0.751148 |
| -2.505458 | -1.136387 | -0.759363 | -0.217065 | -0.606196 | -0.888527 | -0.200540 | -0.673192 | -0.078133 |
| -0.048943 | -0.018739 | -0.053149 | 0.1957060 | 0.1125192 | 0.1625334 | -0.030144 | 0.2183968 | -0.165217 |
| 0.4749521 | 0.0029343 | -0.313716 | -0.658338 | -0.210894 | -0.339570 | -0.101235 | -0.424522 | -0.237173 |
| 0.7786379 | 1.4704776 | -0.483621 | 0.0449109 | 0.4615983 | -0.402998 | 0.3429392 | 0.8221654 | -0.102656 |
| -0.682348 | -1.028180 | -0.045836 | -0.293402 | -0.840308 | -0.129848 | -0.512404 | -0.637386 | -0.548236 |
| -2.492047 | -2.076293 | -0.325782 | -1.002148 | -0.300495 | -0.522203 | -1.260014 | -1.443145 | -0.867954 |
| -1.356885 | -4.819516 | -0.183869 | -1.063496 | -1.233033 | -0.481890 | -1.840498 | -2.859824 | -1.129582 |
| -1.139121 | -1.707245 | -0.284313 | -0.481902 | -1.181947 | -0.670259 | -0.759102 | -1.619312 | -0.858170 |
| -1.311526 | -0.512059 | -0.068296 | -0.535663 | -0.457498 | -0.052232 | -0.546468 | -0.599757 | -0.269246 |
| -0.006484 | -0.176082 | -0.239797 | -0.129367 | 0.0040735 | -0.011928 | -0.097442 | -0.201656 | -0.207311 |
| -1.210559 | -1.464459 | -0.534734 | -0.830181 | -1.235813 | -0.914619 | -0.979032 | -1.127555 | -0.989215 |
| 0.3029148 | 0.2851970 | -0.244526 | -0.367306 | 0.1494742 | -0.423659 | -0.218171 | -0.023505 | -0.119274 |
| -1.850524 | -1.890660 | -0.517926 | -0.550723 | -0.657568 | -0.287115 | -1.041032 | -0.434558 | -0.134387 |
| 1.8773343 | 1.5696898 | -0.541057 | -0.653408 | 0.5815261 | -0.713940 | 0.2077183 | 1.3921515 | -0.507526 |
| -1.127525 | -1.282248 | -0.060865 | -0.124375 | -0.775141 | 0.1563841 | -0.092272 | -0.946150 | -0.513615 |
| -1.062742 | -0.758336 | -0.632959 | -0.437258 | -0.108166 | -0.527989 | -0.607642 | -0.018913 | -0.132260 |
| -0.252413 | -0.064994 | 0.0292143 | -0.196458 | -0.395510 | -0.085394 | -0.242758 | -0.354271 | -0.091490 |
| -1.401195 | -1.148928 | -0.323645 | -0.404671 | -0.820313 | -0.457837 | -0.337349 | -0.707802 | -0.374555 |
| -1.594175 | -1.918764 | -0.149508 | -0.094248 | 0.2164098 | -0.212351 | -0.357468 | -0.394062 | -1.069533 |
| -1.753065 | -1.362566 | -0.043326 | -0.297560 | -0.543718 | -0.513620 | -0.586448 | -0.390555 | -0.204443 |
| -1.051026 | -0.042728 | 0.0790970 | -0.056935 | 0.2489766 | 0.1324845 | -0.196554 | 0.3968363 | 0.2226463 |
| 1.3781280 | 0.7771338 | 0.0355374 | -0.329674 | -0.321171 | -0.340204 | 0.1494760 | 0.2748467 | -0.225831 |
| -2.266009 | -1.827764 | 0.3641249 | -0.038080 | -1.481797 | 0.3674068 | -0.565952 | -1.534951 | -0.242252 |
| -1.364507 | -1.848828 | -0.381408 | -0.227641 | -0.607700 | -0.337846 | 0.0550911 | -0.830444 | -0.444833 |
| -1.535823 | -2.190315 | -0.265622 | -1.017461 | -2.443112 | -0.756488 | -1.455781 | -2.530132 | -0.494503 |
| -2.139335 | -1.301489 | 0.0912269 | -0.398922 | -0.353219 | -0.073330 | -0.404033 | -0.456323 | -0.428327 |
| -0.583362 | -0.523258 | -0.907395 | -1.129860 | -0.960588 | -1.227600 | -0.763591 | -0.589440 | -1.542071 |
| -0.440962 | 0.2444213 | 0.3875593 | 0.5243705 | 0.3641523 | 0.3957902 | 0.2393791 | 0.0783057 | 0.4400993 |
| -0.538544 | -1.956152 | -0.147785 | -0.300147 | -0.815836 | -0.206308 | -0.389556 | -0.889852 | -0.274816 |
| -2.439086 | -1.413415 | -0.208671 | -0.345944 | -0.567925 | -0.424566 | -0.779789 | -0.767022 | -0.297945 |
| -1.193272 | -0.519215 | -0.346390 | -0.017356 | -0.477940 | -0.481301 | -0.129779 | -0.311424 | -0.406825 |
| -2.020486 | -1.944584 | -0.237757 | -0.453157 | -0.914672 | -0.373735 | -0.358284 | -1.079634 | -0.363758 |
| -0.479970 | -0.530938 | -0.628613 | -0.445351 | -0.241961 | -0.533539 | -0.335748 | -0.455593 | -0.203096 |
| -0.697906 | -0.550628 | -0.180229 | -0.956920 | -0.613719 | -0.465280 | -0.577416 | -0.924643 | -1.460418 |
| -1.755983 | -0.370488 | 0.0864066 | -0.239703 | 1.2404125 | 0.0433386 | -0.156651 | 1.6206060 | 0.0806229 |
| -1.720779 | -1.632065 | -0.157791 | -0.028406 | -0.254136 | -0.175264 | 0.0385267 | -0.558691 | -0.491067 |
| -1.568405 | -2.181187 | 0.0885714 | 0.0785973 | -0.160374 | 0.0580984 | -0.302189 | -0.444520 | -0.236246 |
| -0.776555 | 0.1213338 | -0.200171 | -0.992122 | -0.470828 | -0.441147 | -1.259317 | -0.495451 | -1.014576 |
| -0.077920 | -0.524672 | -0.379810 | -0.558743 | -1.245317 | -0.299839 | -0.454610 | -1.099279 | -0.282396 |
| -1.003159 | -0.532925 | -0.329926 | -0.246136 | -0.729120 | -0.265852 | -0.126674 | -0.829017 | -0.196201 |
| -1.466240 | -0.666323 | -0.597474 | -0.424666 | -0.481120 | -0.510846 | -0.338550 | -0.735825 | -0.170500 |
| 0.0563403 | 1.0828740 | -0.479081 | -0.116647 | -0.387087 | -0.813808 | 0.2833497 | 0.2145309 | -0.488958 |
| -1.024420 | -2.232507 | 0.6124775 | -0.569574 | -2.931672 | 0.2467919 | -1.600922 | -3.120293 | -0.707927 |
| -0.962247 | -1.344692 | -0.829744 | -0.976286 | -1.170302 | -1.212154 | -0.570377 | -2.468537 | -1.159267 |

| | | | | | | | | |
|---|---|---|---|---|---|---|---|---|
| -2.1825137 | -1.4773678 | 0.4030127 | 0.2819161 | 0.3613040 | 0.3628401 | -0.2085874 | 0.3175334 | -0.135696 |
| -1.1093878 | -1.2781868 | -0.7698317 | -1.4925407 | -2.0998539 | -0.8826827 | -1.5448937 | -2.3063404 | -1.0654293 |
| -0.4441938 | -0.1161498 | -0.2524897 | -0.5518104 | -0.1071183 | -0.1217554 | -0.7977396 | -0.3289926 | -0.4942728 |
| 1.4820657 | 1.6004537 | -0.0733358 | -0.6259206 | -0.4577779 | -0.0661531 | 0.0030386 | -0.5783161 | -0.4377870 |
| -1.3444295 | -1.8883657 | 0.0176944 | -0.5270741 | -1.2886660 | 0.0369429 | -0.8989527 | -1.6583621 | -0.2242769 |
| 2.7407644 | 1.6819616 | -0.6235943 | 1.8285882 | 1.0681564 | 0.1614896 | 2.1672684 | 0.8238247 | -0.1510318 |
| -1.1190328 | -0.7204366 | -0.2628725 | -0.3016174 | -0.4552853 | -0.4786220 | -0.3737210 | -0.4360879 | -0.3610862 |
| -0.6447718 | -0.8385704 | -0.4232205 | -0.3125096 | -0.2880745 | -0.9257502 | -0.8213281 | -0.8895946 | -0.6956194 |
| -1.0073519 | -1.8581901 | -0.5322842 | -1.3862351 | -1.6050015 | -0.3579093 | -1.6927557 | -1.8091958 | -0.2825284 |
| -1.0911923 | 0.3608151 | -0.5018832 | -0.6377937 | -0.5767997 | -0.8122473 | -0.6498628 | -0.1073953 | -0.6367849 |
| -0.3570251 | 0.1669116 | 0.0795048 | 0.1526926 | 0.3335285 | -0.0080272 | -0.0260366 | 0.2650785 | -0.1132649 |
| -0.7727596 | -0.1496867 | -0.8379278 | -0.7643513 | -1.4039676 | -0.6806488 | -0.9496347 | -1.0248563 | -0.9992448 |
| -2.4704441 | -2.7572350 | 0.3163546 | -0.0719301 | -0.5048137 | 0.4063268 | -0.4459524 | -0.1142502 | -0.5143861 |
| -0.9934824 | -0.4174055 | -0.4470141 | -0.4448769 | -0.7846286 | -0.6429976 | -0.5926995 | -0.7270552 | 0.1781331 |
| -0.4309731 | -0.1210435 | -0.3324517 | -0.3209179 | -0.3271679 | -0.1598178 | -0.3061871 | -0.0435137 | -0.4087447 |
| -1.0109680 | -1.4721713 | 0.1003140 | 0.2026644 | -1.5192253 | 0.0545863 | 0.1986739 | -0.8380694 | 0.0401070 |
| 1.2443084 | 0.3798916 | -0.5383116 | -0.3616292 | -0.4605360 | -0.6022165 | -0.3432006 | -0.1241342 | -0.2834081 |
| -0.8461695 | -1.1682437 | -0.3872897 | -0.4735278 | -0.2300117 | -0.5989069 | -1.1220014 | -0.1192745 | -0.0632458 |
| -0.8278883 | -0.5178319 | -0.5760151 | -0.5599344 | -0.1628003 | -0.4700165 | -0.6553320 | -0.4610053 | -0.7331378 |
| -2.1354632 | -1.5603294 | 0.0326170 | -0.4337009 | -0.4814403 | -0.3129771 | -0.6610326 | -0.7950065 | -0.5371323 |
| 1.5712671 | 1.9087890 | -0.1689387 | 0.4255451 | 1.0960067 | -0.2513692 | 1.4883695 | 1.4275810 | -0.2582399 |
| -1.3112645 | -0.5872952 | 0.0557475 | -0.2933340 | 0.3177887 | 0.0681600 | 0.1445951 | 0.0032742 | -0.1940584 |
| -1.7920511 | -2.8956758 | -0.3012226 | -0.7983092 | -1.1865547 | -0.5125338 | -1.4346288 | -1.0884951 | -0.4769373 |
| -0.7842606 | -0.8903859 | -0.5011615 | -0.7712477 | -0.3298299 | -0.2504836 | -0.5362005 | -0.1272992 | -0.4348890 |
| -0.8306828 | -0.6539827 | -0.1093379 | -0.8261413 | -1.1687419 | -0.1156519 | -0.8919982 | -1.8765851 | -0.2820158 |
| -1.2168287 | -1.1589790 | -0.6195157 | -1.0385898 | -0.7068778 | -0.5762631 | -1.5532893 | -0.5766882 | -0.5464654 |
| -1.2013599 | -0.6875111 | -0.7151576 | -0.7947013 | -0.6390255 | -0.5937391 | -0.9072787 | -0.6070953 | -0.3079707 |
| -1.0503176 | 0.2210395 | -0.2167482 | -0.6417993 | -0.9141720 | -0.2979284 | -1.2283246 | -0.7816300 | -0.3124602 |
| -1.5213957 | -2.0226382 | -0.2526618 | -0.5975386 | -1.3176820 | -0.5334770 | -1.3417890 | -1.5780281 | -0.8853817 |
| 0.6868625 | 1.4329343 | -0.1844476 | 0.7254171 | 0.8890760 | -0.2786143 | 0.5358470 | 1.2596806 | -0.1728332 |
| 1.4784580 | 3.1848243 | -0.2299541 | 0.9725528 | 1.9350551 | -0.1172800 | 2.5230028 | 2.5740479 | -0.1087100 |
| -1.1642145 | -0.4181892 | -0.6143417 | -1.1050047 | -1.3556247 | -0.8855297 | -1.7053064 | -1.5065160 | -0.6265864 |
| -0.4984074 | 0.1830349 | -0.0880884 | -0.0651421 | 0.2774621 | -0.1548829 | 0.0761161 | 0.3019974 | -0.1308626 |
| -0.8950413 | 0.2513438 | -0.0258658 | 0.2527882 | 0.2482056 | 0.0287799 | 0.2330806 | 0.1857195 | 0.0415394 |
| 1.2247331 | 1.2863518 | -0.1097868 | 0.2141396 | -0.5549523 | 0.0574973 | 0.6014289 | -0.6610385 | 0.4346064 |
| -0.6947582 | 0.0957481 | -0.4829507 | -0.6169747 | -0.5438751 | -0.3734835 | -0.5787283 | -0.4871886 | -0.8494473 |
| -1.1912539 | -0.9759220 | 0.2219262 | 0.5761694 | 0.9314522 | 0.1520072 | 0.8729476 | 0.7997835 | 0.1333964 |
| -1.3670571 | -0.4070206 | -0.1702051 | 0.1770012 | -0.1699791 | -0.2341177 | 0.2468474 | -0.0602948 | -0.4153553 |
| -1.2826658 | -0.5602015 | -0.4891018 | -0.2947732 | -0.0748278 | -0.5458509 | -0.1442105 | -0.1901284 | -0.3712839 |
| -1.9593417 | -1.7698831 | -0.3384951 | -0.4452587 | -0.7999996 | -0.4456427 | -0.4749743 | -0.8835005 | -0.0883805 |
| -0.8518191 | -1.3815362 | 0.3064472 | -0.0739580 | -0.6973920 | -0.0042129 | -0.2641292 | -0.7665256 | -0.1983332 |
| 0.0556057 | 0.6453322 | -0.5969798 | -0.3297575 | 0.3085656 | -0.7701791 | -0.1144030 | 0.3879206 | -0.8560744 |
| -1.7721733 | -0.3544771 | -0.1497289 | 0.1556415 | 0.8449383 | -0.0700532 | 0.2899763 | 1.2553484 | -0.2651507 |
| -0.7693457 | 0.2853740 | -0.1936041 | -0.2183706 | 0.2119178 | -0.2388975 | -0.2218346 | 0.5406784 | -0.3199982 |
| -0.8837057 | -0.7843890 | -0.1690514 | -0.3555394 | -0.6579656 | -0.1911254 | -0.6618882 | -0.3972421 | -0.2076085 |
| -1.5244271 | -0.6583623 | -0.6114892 | -0.2510346 | 0.2051059 | -0.1069393 | -0.0087974 | 0.0653618 | -0.2286656 |
| -0.9035816 | -1.4647346 | -0.3798831 | -0.7093348 | -0.9514743 | -0.3897801 | -0.6906449 | -1.0330105 | -0.6097476 |
| -0.8434904 | -1.0541033 | -0.3083837 | -0.8618067 | -1.1232794 | -0.2784544 | -1.0118904 | -0.9232065 | -0.5051353 |
| -0.4667372 | -1.1767284 | -0.1859670 | -0.3629601 | -1.0246163 | -0.0568624 | -0.7292828 | -0.8594477 | -0.3320704 |
| -1.9091749 | -0.6537041 | -0.1059172 | -0.1279633 | -0.5448597 | -0.0284333 | -0.1693468 | -0.8689999 | -0.2047233 |

| | | | | | | | | |
|---|---|---|---|---|---|---|---|---|
| -3.048395( | -1.180950 | -0.116762 | -0.162361 | -0.029023 | -0.111639 | -0.915174 | 0.2079678 | 0.0489197 |
| -0.482817 | -1.032341 | -0.385828 | -0.387862 | -0.543199 | -0.356569 | -0.244650 | -0.348216 | -0.035319 |
| -0.952184 | -1.421354 | -1.150494 | -0.654147 | -0.688783 | -0.853861 | -0.158459( | -0.925853( | -1.115009 |
| -1.461510 | -1.962508 | -0.893995 | -1.025264 | -1.076187 | -0.825916 | -0.730943 | -0.961526( | -1.428342 |
| -2.028449( | -2.357366( | -0.469314 | -0.845527 | -0.893026 | -0.308420( | -1.387508 | -1.039976( | -0.747176 |
| -0.602627( | -0.647972 | -0.250898 | -0.161500 | -0.330994 | -0.328588 | -0.359196 | -0.090394 | -0.464112 |
| 0.9753705 | 1.3261351 | -0.360484 | 1.2603254 | 1.9066354 | -0.235488( | 1.6250036 | 1.4415693 | -0.566791 |
| 0.0861823 | 0.0851718 | -0.041589 | -0.557530( | -0.504085( | -0.520062 | -0.748387 | -0.624258 | -0.174571 |
| -3.585873( | -3.337397 | -0.509327 | -0.218046( | -0.851905 | -0.401993 | -0.570638 | -1.255383 | 0.2265281 |
| -0.739875 | -0.030170 | -0.354342 | -1.012991 | -0.013255 | -0.322197 | -0.458717( | -0.013620 | -0.781529 |
| -1.234788 | -0.824834 | 0.1617046 | -0.245984 | 0.4092986 | -0.104220 | -0.026615( | 0.5441630 | -0.382568 |
| -1.636528 | -0.423593 | -0.700895 | -0.852970 | -0.618022 | -0.654553( | -0.539216 | -0.831196 | -1.155050 |
| 0.1707786 | -0.789615 | -0.105772 | 0.0012605 | 0.0678084 | 0.0441414 | -0.233615( | 0.0708610 | -0.015262 |
| -1.992652 | -1.712718 | 0.1282224 | 0.1471167 | -0.075814 | -0.147408 | -0.181553 | -0.009537 | -0.117481( |
| -0.751989 | -2.333051 | 0.1508157 | -0.124752 | -1.204351( | 0.0115115 | -0.465067( | -1.334219 | -0.263868 |
| -0.555597 | -0.664703 | -0.281849 | -0.514282( | -0.775742 | -0.306482( | -0.258302 | -0.316685 | 0.0517665 |
| -3.971648( | -3.035839 | 0.0611672 | 0.0114813 | -0.602537 | 0.0775126 | -0.161505 | -0.987233 | -0.091163 |
| -0.936710 | -0.465457 | -0.488088 | -0.769022 | 0.3835961 | -0.434900 | -0.826082 | 0.3760238 | -0.784632 |
| -0.823897 | -0.883938 | -0.298118 | -0.752538 | -1.479884 | -0.319003( | -1.472869 | -1.135617 | -0.146428 |
| -1.666516( | -1.261418( | 0.0922909 | 0.3670897 | 0.3168276 | 0.3009811 | 0.1738649 | 0.6456760 | 0.0935691 |
| -1.363369 | -1.743485 | -0.425267 | -0.265413 | -0.415891 | -0.421372( | -0.066793 | -0.732763 | -0.784295 |
| -0.159089 | -0.319684( | 0.6072490 | -0.789430 | -4.840226 | 0.4041242 | -1.593134 | -4.895938( | -0.366909 |
| -1.530138 | -3.785285 | -0.072744( | -0.860606 | -2.906178( | -0.208533 | -1.338642 | -3.733948( | -0.893631( |
| -1.288050 | -0.147952 | -0.130659 | -0.094434( | 0.2241235 | -0.305763 | -0.278113 | 0.0844228 | -0.299232 |
| -0.131006 | -0.408735 | -0.145594 | -0.272125( | -0.509111 | -0.211491 | -0.402741 | -0.998356 | -0.477145 |
| -0.773942 | -0.019560 | -0.532992 | -0.589013( | -0.867966 | -0.483952 | -0.509608 | -0.665751 | -0.540453 |
| -0.953243( | -1.371859 | -0.108872 | -0.253602 | -1.780027( | -0.119414 | -0.366691 | -2.048925 | -0.428040 |
| -1.098529 | -0.658672 | -0.408203 | -0.533467 | -1.150422 | -0.297478 | -0.340939 | -0.981041 | -0.284762 |
| -1.036943( | 0.2727221 | -0.201677( | 0.2571745 | 0.5209740 | -0.289185( | 0.1051842 | 0.5794792 | -0.354892 |
| -1.832353 | -0.479890 | 0.1487432 | -0.019048( | 0.0024281 | 0.2102359 | 0.1926090 | 0.1412346 | 0.1683049 |
| -0.403298 | -0.799498( | -0.562728 | -0.651177 | -0.854837 | -0.755627 | -0.918780 | -0.787168 | -0.859869 |
| -1.674152 | -0.592733 | -0.009521 | -0.339710 | -0.267719 | -0.035099( | -0.318093( | -0.324228 | -0.439119 |
| -0.102990 | 1.6681978 | -0.333665( | -0.913552 | 0.5664837 | -0.339193 | -0.809020 | -0.144922 | -0.765248( |
| -0.766809 | -1.478181( | -0.324596 | -0.393704 | -1.043025 | -0.321492 | -0.664145 | -1.117080 | -0.549932 |
| -0.275204 | -0.658981( | -0.150162 | -0.159878( | -0.489080 | -0.050663 | -0.110763 | -0.583166 | -0.331586 |
| -2.250510 | -0.690641 | -0.179821 | -0.541188 | -1.571501 | -0.238156 | -0.590746 | -1.543857 | -0.263282 |
| -0.725361( | -0.548624 | -1.168904 | -1.575233 | -1.642169 | -0.834695 | -1.775102 | -2.189502 | -0.770131 |
| 0.0096966 | -0.205323 | -0.144421 | -0.067652( | -0.137150 | -0.114701 | -0.078627 | -0.124067 | -0.119403( |
| 0.6097279 | 0.6196714 | 0.0088156 | 0.2048400 | 1.1047800 | 0.1025837 | 0.3946218 | 0.9419513 | -0.246712( |
| -0.971062 | -0.795268 | -0.567824 | -0.479404( | -0.328781 | -0.307064 | -0.590868 | -0.443872 | -0.801433 |
| -2.809479 | -0.390993 | -0.045073( | 0.1940648 | 0.7253644 | -0.053546 | 0.3980096 | 0.8901130 | -0.253050 |
| -1.555831 | -0.714232 | -0.455180 | -0.918635 | -0.925493 | -0.482216( | -1.008307 | -1.035763 | -0.615059 |
| -0.592716 | -0.807564 | -0.400492 | -0.902896 | -0.500937 | -0.208895 | -0.941152( | -0.761333 | -1.046713 |
| -1.476903 | -1.383560 | -0.418117 | -0.611942 | -0.570828 | -0.724128 | -0.485279 | -0.896116 | -0.551917 |
| -0.905398 | 0.0185054 | -1.033083 | -0.434242( | -0.577375( | -0.662830( | -0.348705 | -0.302630( | -0.689221 |
| -2.236517( | -1.638477( | -0.612919 | -1.261392( | -0.603188( | -0.770624 | -1.220281 | -0.369535( | -0.008051 |
| -1.452528( | -1.406385 | -0.257551 | -0.511374 | -0.420039( | -0.193201 | -0.801414 | -0.653196 | -0.334577 |
| -2.060776( | -1.828995( | -0.492219 | -0.841365( | -1.563610 | -0.855430 | -0.976221 | -1.333280( | -0.984891 |
| -1.369933 | -0.590665 | -0.671848 | -0.322603 | -1.057921 | -0.605326( | -0.732714 | -0.549534 | -0.534370 |
| -1.422623 | -1.527624 | -0.966841 | -1.295810 | -1.604382 | -1.293084 | -1.339552 | -1.748713 | -0.544762 |

| | | | | | | | | |
|---|---|---|---|---|---|---|---|---|
| -0.5360643 | 0.0212658 | -0.7622289 | -0.1448353 | -0.7657317 | -0.5235294 | -0.4540312 | -0.1477844 | -0.2666895 |
| -0.6855087 | -0.5185256 | -0.4266479 | -0.1008926 | -0.0951679 | -0.7063033 | -0.0762151 | 0.0089638 | -0.2490445 |
| -0.3429192 | 0.2183477 | -0.4643304 | -0.2177014 | 0.1962060 | -0.4128926 | -0.0518543 | 0.2384036 | -0.4473374 |

| PG_480 | PG_481 | PG_482 | PG_483 | PG_484 | PG_485 | PG_486 | PG_487 | PG_488 |
|---|---|---|---|---|---|---|---|---|
| 0.0185729 | -1.432671 | 0.0036671 | -0.174478 | -1.335965 | -0.537949 | -0.528787 | -0.820612 | -0.307688 |
| -1.512504 | -1.365244 | -0.758964 | -1.861771 | -2.110932 | -0.358992 | -0.573566 | -0.516507 | -0.479566 |
| -0.411542 | -1.425566 | -0.130659 | -1.307270 | -1.720133 | -0.968404 | -1.366239 | -0.383891 | -0.842794 |
| -0.119918 | 0.1566510 | -0.093566 | -0.405760 | -0.463024 | -0.247141 | 0.1596928 | -0.169156 | 0.0065451 |
| 0.0464301 | -0.252830 | 0.3050406 | 0.1163623 | -0.501002 | -0.105563 | 0.0307427 | -0.641591 | -0.072947 |
| -0.378703 | -1.245441 | -0.686652 | -0.779791 | -2.279135 | -0.243686 | -0.463188 | -0.812557 | -0.311399 |
| -0.430121 | -0.356657 | -0.096330 | -0.284941 | -0.430171 | -0.339762 | -0.138419 | -0.204170 | -0.238469 |
| -0.402742 | -1.409273 | -0.426497 | -0.541799 | -2.282048 | -0.383332 | -0.255989 | -0.366841 | -0.361899 |
| 0.4055116 | -0.377983 | -0.221130 | 0.4665484 | 0.7238725 | -0.712269 | -0.307398 | -0.794369 | -0.324521 |
| -0.468630 | -0.191093 | -0.023971 | -0.923654 | -0.575020 | -0.289368 | -0.296822 | -0.525886 | -0.141351 |
| -0.138423 | -0.458562 | -0.075914 | -0.403772 | -1.016191 | -0.396904 | -0.187029 | -0.222127 | -0.308875 |
| -0.103335 | 0.3075570 | -0.296980 | -0.190922 | -0.099109 | -0.245525 | -0.280989 | -0.173342 | -0.286935 |
| -1.257185 | -1.520988 | -1.092130 | -1.689112 | -1.851437 | -0.626604 | -0.753347 | -0.642693 | -0.382221 |
| 0.6276387 | 0.8983474 | 0.0784075 | 1.1034712 | 1.9549119 | -0.839884 | -0.153401 | 2.1175876 | -0.985586 |
| -0.182862 | -0.395328 | 0.1661989 | -0.232599 | -0.090342 | -0.334551 | -0.020588 | 0.1169974 | -0.233646 |
| 2.5568937 | 1.3730434 | 0.2124776 | 2.8249026 | 2.4380843 | -0.157999 | 0.3548144 | -0.037257 | -0.056802 |
| -1.772993 | -1.552873 | -0.837660 | -1.879234 | -1.394505 | -1.624596 | -2.113008 | -0.688590 | -1.004778 |
| -1.057759 | 0.7823382 | -1.100040 | -1.062969 | 0.2904582 | -0.224399 | -1.649200 | 0.3193407 | -0.504656 |
| -1.487017 | -1.094417 | -0.979644 | -1.597397 | -1.033997 | -0.665007 | -0.703519 | -0.300348 | -0.400583 |
| -0.102394 | 0.8841330 | -0.954244 | 0.0866774 | 0.8591540 | -0.647895 | -0.689905 | -0.487041 | -0.408722 |
| 0.1973549 | 0.6562221 | -0.578346 | 0.1637070 | 0.2688727 | -0.492344 | -0.003172 | -0.119461 | -0.193494 |
| -0.403657 | -0.163621 | -0.126321 | -0.267476 | -0.381909 | -0.302775 | -0.784723 | -0.342262 | -0.630813 |
| 0.6467311 | 0.9587164 | -0.169244 | 0.7822843 | 1.6646355 | -0.593581 | 0.6140860 | 1.1317726 | -0.066143 |
| -0.574606 | -0.485766 | -0.520047 | -0.805757 | -1.336023 | -0.241362 | -0.437840 | -0.773921 | -0.310580 |
| -1.109909 | -1.371539 | -0.741465 | -1.587085 | -2.011076 | -0.352269 | -0.604944 | -1.061726 | -0.401934 |
| 0.7750697 | 0.8937178 | 0.2527169 | 0.8501065 | 1.1286628 | -0.387367 | 0.6650323 | 0.3548171 | -0.088193 |
| -0.170022 | -0.362512 | 0.1030676 | -0.184165 | -0.322937 | -0.023712 | 0.3328509 | -0.295107 | 0.1058214 |
| -1.005968 | -1.506618 | -1.344888 | -1.491009 | -1.507767 | -0.969710 | -1.076189 | -0.861780 | -1.192163 |
| -1.318352 | -1.765770 | -0.924152 | -1.585676 | -2.622300 | -0.213005 | -0.421649 | -0.358420 | -0.263337 |
| 0.0980054 | 0.0355246 | 0.1322157 | -0.052151 | -0.060959 | -0.363634 | -0.220513 | -0.054419 | -0.335354 |
| 0.2892128 | 0.6845262 | 0.5413885 | -0.099730 | 0.0365634 | -1.787141 | -1.709902 | -1.703462 | -1.652499 |
| -0.317422 | -0.767672 | 0.3904121 | -0.226346 | -0.653488 | -0.093986 | 0.1804745 | 0.0627895 | 0.2240872 |
| -0.328671 | -0.698396 | -0.363074 | 0.1506862 | -0.290840 | -0.227210 | -0.000392 | -0.868240 | -0.101657 |
| -1.196842 | -1.693961 | -0.698095 | -1.360231 | -3.849286 | -0.614633 | -0.599619 | -0.986347 | -0.509028 |
| -0.075927 | 0.0299624 | 0.0472354 | -0.207126 | -0.230316 | -0.106847 | -0.358227 | -0.173649 | -0.085511 |
| -0.512182 | -0.011959 | -0.918348 | -0.526927 | 0.3090835 | -1.089265 | -0.933816 | 0.1359001 | -1.167190 |
| -0.976650 | -1.902670 | -0.881248 | -1.264622 | -3.024682 | -0.742254 | -0.356766 | -0.669198 | -0.347438 |
| 1.2235103 | -0.252172 | 0.2447692 | 2.2080155 | -0.045655 | 0.1602432 | 0.9059142 | 0.8163913 | -0.291788 |
| -0.777010 | -0.783255 | -0.684811 | -0.568922 | -1.046952 | -0.452767 | -0.077293 | 0.3478769 | -0.238515 |
| -0.397320 | 1.3610664 | -0.860792 | -0.235431 | 1.7694096 | -0.511220 | -1.326052 | 1.3594146 | -0.830624 |
| -0.139167 | 0.8109429 | -0.191616 | 0.0190643 | 0.6235539 | -0.181757 | 0.1821873 | 0.2663641 | 0.2601602 |
| -0.970603 | -1.651660 | -0.644658 | -1.088496 | -2.506586 | -0.538087 | -0.431894 | -1.613292 | -0.490466 |
| -0.797532 | -0.527177 | -0.822982 | -0.865750 | -0.631869 | -1.360260 | -0.894983 | 0.0049393 | -0.813862 |
| -0.368384 | -0.499948 | -0.292423 | -0.310327 | -0.984896 | -0.147104 | 0.0423108 | -0.342259 | -0.143690 |
| 0.8882531 | 0.6747138 | -0.071955 | 0.6073009 | 1.3871309 | -0.421443 | 0.0329639 | 0.1072559 | 0.0140478 |
| -0.521707 | 0.5573635 | -0.327106 | 0.3347690 | 3.9719686 | -0.056425 | 0.0011961 | 0.0355432 | -0.479605 |
| -0.018348 | 0.2035334 | -0.089602 | 0.1002090 | -0.380733 | 0.0176466 | -0.229253 | -0.034199 | -0.076786 |

| | | | | | | | | |
|---|---|---|---|---|---|---|---|---|
| -0.5017195 | -0.7387336 | -0.6966438 | -0.6156361 | -0.3739849 | -0.3378475 | -0.1131784 | 0.1026802 | -0.1400631 |
| -0.2901218 | -1.3836789 | -0.3639978 | -0.4293476 | -1.8872683 | -0.5450353 | -0.5502721 | -0.3711360 | -0.4581047 |
| -0.3582685 | -0.1569098 | -0.7572466 | -0.2446661 | 0.0828833 | -0.5233266 | -0.3520826 | -0.2627717 | -0.4559879 |
| 0.9350742 | 0.6198911 | -0.4356688 | 2.0607673 | 1.0361487 | -0.6018490 | 0.0800856 | 1.0419775 | -0.9438073 |
| -0.5616051 | -0.2922916 | -0.5975877 | -0.4414949 | -0.2096253 | 0.3365458 | -0.2277436 | -0.2446601 | -0.2250242 |
| -0.5326922 | 0.6622266 | -0.5153655 | -0.8373837 | 1.6001523 | -0.5132212 | -0.0431369 | -0.0206336 | -0.2640988 |
| -1.1609307 | -1.3562653 | -1.0209084 | -1.8262418 | -1.7838238 | -0.4510320 | -0.2807268 | -0.4072497 | -0.4408479 |
| 0.2014401 | 0.3374000 | -0.0952666 | 1.0795887 | 1.7139548 | -0.5412149 | 0.4939238 | 1.0551716 | -0.3779955 |
| 1.9194047 | 0.3003199 | 0.3914247 | 2.0383345 | 0.4610453 | -0.9498476 | 0.2772370 | 0.1900007 | -0.9017175 |
| -0.7799896 | -0.6637596 | -0.5934727 | -0.4916687 | -0.0666221 | -1.1973616 | -0.5004420 | -0.4300588 | -0.7263571 |
| -1.0826394 | -0.9623688 | -0.5556540 | -1.4831669 | -1.5416683 | -0.8955867 | -0.7769471 | -0.5354193 | -0.8745415 |
| 2.1967578 | 1.0153335 | 1.1415256 | 2.7686024 | 2.4668324 | -0.1990001 | 0.9621361 | -0.0579382 | 0.2503413 |
| 2.0032703 | 0.4065302 | 0.2969171 | 3.1156663 | 1.7603051 | -0.3544549 | 0.3769778 | 0.1896219 | -0.0714626 |
| -0.1248517 | -1.7365424 | -0.3962505 | -0.3083433 | -0.8869819 | -0.3113358 | -0.4356791 | -0.7272178 | -0.2825674 |
| -1.3931520 | -2.1480612 | -0.4237364 | -2.2823682 | -2.6330604 | -0.8002355 | -1.1207001 | -0.4672855 | -0.9609653 |
| -0.9892264 | -0.9165625 | -0.7889601 | -0.8811097 | -0.4448766 | -0.5929937 | -0.2933311 | -0.1551285 | -0.4811823 |
| -0.4362201 | -0.9539127 | -0.4746795 | -0.8801002 | -1.7665966 | -0.2021508 | 0.0295594 | -0.4959663 | -0.1426729 |
| -0.3738250 | -0.0683887 | -0.4535776 | -0.6850864 | -0.5047517 | -0.2594649 | -0.3202587 | -0.8426572 | -0.1932910 |
| -0.4267560 | -0.8618188 | 0.2234197 | -0.9295721 | -1.8829888 | -0.5384893 | -0.3176401 | -0.9975002 | -0.4060326 |
| 0.2194886 | -0.2997850 | 0.3582781 | 0.2065984 | -0.1546619 | -0.1031937 | 0.2358221 | -0.5427740 | 0.0895850 |
| 0.1118049 | -0.2537327 | -0.5259819 | -0.0738501 | -0.3793628 | -0.4736149 | -0.2082354 | -0.2110821 | -0.4048177 |
| -0.9494260 | -1.3756280 | -0.6409073 | -0.8826075 | -2.2696795 | -0.6577301 | -0.4854574 | -0.8489791 | -0.4541627 |
| -0.0635978 | 0.2745232 | -0.3785026 | -0.4498322 | 0.1372392 | -0.1438942 | -0.1070908 | -0.2862723 | -0.1049619 |
| 0.8044866 | 1.3316188 | 0.6002474 | 0.7237250 | 1.2171086 | -0.0831679 | 0.8500969 | 0.2119331 | 0.3028900 |
| -0.9871778 | -2.3846264 | -0.7798626 | -1.3051453 | -2.9135105 | -1.1872418 | -0.6772325 | -1.2337530 | -1.0068064 |
| -1.0262739 | -0.6207869 | -0.4650818 | -1.0165116 | -0.2933390 | -0.1983997 | -0.1382243 | -0.1238045 | -0.2036515 |
| -1.4655354 | -1.3880372 | -0.8434120 | -2.3904901 | -2.5847178 | -0.3620506 | -1.4289891 | -0.9819999 | -0.5163611 |
| 1.7952164 | 3.5283652 | 0.2228811 | 2.1371958 | 4.1456921 | 0.1428990 | 0.8790934 | 2.0528188 | 0.2398274 |
| -0.1258172 | 0.6604728 | -0.7808890 | -0.0687852 | 1.1155622 | -0.2792475 | 0.4873225 | 1.0690096 | -0.0980488 |
| -0.1252600 | -1.6703124 | -0.0261973 | -0.1948668 | -1.8782903 | -0.4140295 | -0.2160368 | -1.0407529 | -0.4999414 |
| -0.6400576 | -0.9974364 | -0.3588340 | -0.8450725 | 0.0050398 | -0.2380076 | -0.0071603 | -0.0469899 | 0.0053995 |
| -0.1503753 | 0.1994515 | 0.1771443 | -0.1290474 | 0.5460457 | -0.5365833 | 0.0234313 | -0.4849628 | -0.4204400 |
| -0.8701223 | -0.6900477 | -0.6530584 | -1.2707733 | -1.1953499 | -0.5587488 | -0.7382344 | -0.8206836 | -0.5222318 |
| -0.4300464 | -1.2735327 | -0.6172880 | -0.4877409 | -1.1763043 | -0.7068155 | -0.3749043 | -1.1596917 | -0.3925103 |
| 0.3058158 | 1.3589770 | -0.2298499 | 0.7226992 | 1.1826800 | -0.2466284 | -0.1648103 | -0.0368618 | -0.1450096 |
| -0.2248547 | -0.3648593 | -0.7016792 | -0.4016902 | -0.5312194 | 0.2039378 | 0.2226434 | 0.3098004 | 0.1735162 |
| -0.8446660 | -1.9659023 | -0.0683791 | -0.7724153 | -0.3993943 | -0.0116422 | -0.2637494 | -0.2452723 | -0.1881645 |
| -0.3120215 | -0.8927596 | -0.3464320 | -0.6752754 | -1.0120447 | -0.1899854 | -0.3309496 | -0.5159251 | -0.1899776 |
| -0.1138229 | -0.4720576 | -0.0979675 | -1.0111509 | -1.9625530 | -0.3253062 | -0.1493130 | -0.7127918 | -0.0860824 |
| -1.8487364 | -1.2058243 | -0.9041367 | -1.6415652 | -2.1612562 | -0.4838670 | -1.1048442 | -1.1056495 | -0.5334985 |
| -0.5775704 | -1.1861760 | -0.7761518 | -0.9609793 | -0.6850623 | -0.7743446 | -0.9266423 | -1.4712297 | -0.5956238 |
| -0.0643303 | -1.4472278 | 0.3664662 | -0.8063375 | -1.8040936 | -0.4201938 | -0.5901642 | -0.2946862 | -0.5199598 |
| 0.2972188 | 0.5481831 | -0.0839240 | 1.1817399 | 1.6412550 | 0.2737238 | -0.1306144 | -0.3131798 | 0.4375474 |
| -0.3055398 | -0.1817088 | -0.2127928 | -0.3687403 | -0.2003784 | -0.1133626 | -0.1594324 | -0.1331272 | -0.1072935 |
| -0.4996396 | -0.6470627 | -0.1426337 | -0.5974661 | -0.9648137 | 0.0221036 | -0.1537564 | -0.2230916 | -0.1418112 |
| -1.2598197 | -1.4024119 | -1.2410953 | -1.5023800 | -1.6182441 | -1.1513792 | -1.3385056 | -0.9617481 | -0.9641119 |
| 7.6955985 | 5.5466422 | 3.2828930 | 8.8626494 | 8.5646043 | -0.1488776 | 0.4508042 | -0.0185580 | 0.2378198 |
| 1.7006927 | 0.9164992 | 0.1151362 | 2.4852463 | 1.8890402 | 0.1206682 | 0.4161982 | 0.1069152 | 0.3003197 |
| -0.4260450 | -0.1718397 | -0.4095787 | -0.5401174 | -0.6061138 | -0.2573102 | -0.5642748 | -0.5433308 | -0.2701795 |
| -0.0743854 | -0.5726318 | -0.3991330 | 0.0177991 | -0.1922318 | -0.1824276 | -0.1548408 | 0.2265402 | -0.2283396 |

| | | | | | | | | |
|---|---|---|---|---|---|---|---|---|
| -0.298052 | 0.5364751 | -0.013111 | -0.658027 | -0.007767 | -0.589024 | -1.056837 | -0.756256 | -0.597661 |
| -0.614176 | -0.268967 | -0.591001 | -0.537516 | -0.260082 | -0.350387 | -0.332250 | 0.0102230 | -0.186044 |
| -0.396825 | -0.029160 | -0.347183 | -0.597940 | -0.233180 | -0.852219 | -0.765034 | 0.0391693 | -0.510652 |
| -0.339896 | -0.285400 | -0.257460 | -0.332481 | -0.271814 | -0.158536 | 0.0440381 | -0.297378 | -0.089885 |
| -0.920583 | -0.431053 | -0.409935 | -1.396153 | -1.349039 | -0.249034 | -0.796292 | -0.344551 | -0.258560 |
| -0.728548 | -0.707591 | -0.641095 | -0.274573 | 0.3892061 | -0.878971 | -0.338635 | -1.512958 | -0.466691 |
| 0.3777881 | -0.273844 | -0.086703 | -0.068738 | -0.050347 | -0.718262 | 0.2586636 | 0.0290536 | -0.038204 |
| 0.1114620 | -0.144030 | -0.199004 | -0.023057 | 0.4210473 | 0.0104403 | 0.2369792 | 0.0372030 | 0.0333820 |
| -0.260245 | -0.324231 | -0.373904 | 0.0329475 | -0.097192 | -0.154687 | -0.257270 | -0.004154 | -0.106427 |
| 0.5903001 | 0.5260660 | 0.1403728 | 0.7453864 | 0.8597115 | -0.153019 | 0.4090704 | -0.124311 | -0.097546 |
| -0.717826 | -1.584234 | -0.458614 | -0.744103 | -1.081835 | -0.516234 | 0.0419259 | -0.692568 | -0.361810 |
| -1.065692 | -1.717947 | -1.186828 | -1.265104 | -2.138446 | -0.556799 | -0.912880 | -0.233590 | -0.365398 |
| -1.630195 | -1.863420 | -1.108627 | -2.269525 | -4.401806 | -0.196041 | -0.390543 | -0.782250 | -0.298845 |
| -1.016937 | -1.699184 | -1.051287 | -1.512325 | -2.475489 | -0.352418 | -0.339256 | -0.179294 | -0.189207 |
| -0.071181 | 0.0722521 | 0.0311539 | -0.355465 | -0.483119 | -0.254204 | -0.278038 | -0.645722 | -0.362247 |
| -0.016860 | -0.185301 | -0.294232 | -0.078857 | -0.174355 | -0.166283 | -0.270171 | 0.0153339 | -0.170241 |
| -0.939254 | -1.739925 | -0.976582 | -2.223510 | -1.415057 | -0.618576 | -0.826267 | -0.042153 | -0.745566 |
| -0.111911 | 0.2044532 | -0.464836 | 0.1689587 | 0.0080825 | -0.104748 | -0.078944 | -0.117903 | -0.169166 |
| -0.420805 | 0.0718188 | -0.314587 | -1.189546 | 0.0386047 | -0.470559 | -0.657381 | 0.0123220 | -0.527877 |
| -0.272000 | 0.0426526 | -0.574064 | -0.452798 | 0.8508031 | -0.643844 | 0.1424048 | 0.6626343 | -0.477733 |
| -0.457452 | -0.434136 | -0.697772 | -0.576840 | -0.555336 | -0.327795 | -0.593908 | -0.500517 | -0.373229 |
| -0.179014 | 0.5490594 | -0.077447 | -0.208565 | 0.2679128 | -0.267567 | -0.361144 | -0.309082 | -0.348320 |
| -0.424275 | -0.331330 | -0.226083 | -0.372775 | -0.269158 | -0.084434 | -0.095081 | -0.234099 | -0.263036 |
| -0.721437 | -0.906025 | -0.496936 | -0.912472 | -1.355855 | -0.442760 | -0.636483 | -0.501795 | -0.442779 |
| -0.665959 | -2.083230 | -1.366203 | -1.637162 | -0.954730 | -0.509327 | -0.628327 | -0.286633 | -0.798818 |
| -0.094121 | 0.1007568 | -0.097469 | -0.154279 | -0.142061 | -0.581008 | -0.306124 | -0.250960 | -0.217366 |
| 0.0010202 | 0.0630521 | 0.1327389 | -0.090603 | 0.3564590 | -0.120154 | 0.0066125 | -0.351647 | -0.059903 |
| -0.708637 | 0.3365329 | -0.693250 | -0.709920 | -0.079554 | -0.688193 | -0.202038 | -0.438160 | -0.401829 |
| -0.681834 | -0.358864 | -0.531039 | -0.688522 | -0.357036 | -0.734452 | -0.769608 | -0.312003 | -0.895815 |
| -0.516577 | -1.309737 | -0.347927 | -0.265904 | -1.541203 | -0.413239 | -0.020290 | -0.901937 | -0.203405 |
| -1.099958 | -1.640282 | -0.670988 | -1.406529 | -1.830208 | -0.547019 | -0.975528 | -1.281477 | -0.600905 |
| -0.500031 | -0.549530 | -0.612353 | -0.505524 | -0.977092 | -0.104890 | -0.451960 | -0.325317 | 0.0037286 |
| -1.659037 | -0.069145 | -2.007252 | -1.325714 | 0.1695435 | -0.646037 | -1.061463 | -0.371888 | -0.368771 |
| 0.5012491 | 0.6234474 | 0.5761240 | 0.5410277 | 0.3534621 | 0.0111015 | 0.4313151 | 0.1908366 | 0.1157889 |
| -0.552668 | -1.106707 | -0.376690 | -0.623878 | -1.909351 | -0.580379 | -0.199498 | -0.704483 | -0.200289 |
| -0.151304 | -0.396350 | -0.258923 | -0.516935 | -0.755024 | -0.782686 | -0.616497 | -0.568765 | -0.585459 |
| -0.342731 | -0.212166 | -0.402319 | -0.489739 | -0.505954 | -0.337923 | -0.074244 | -0.178467 | -0.217767 |
| -0.394974 | -0.706445 | -0.255813 | -0.607828 | -1.112803 | -0.341493 | -0.069685 | -0.711697 | -0.238804 |
| -0.291931 | 0.0831212 | -0.567324 | -0.466414 | -0.182494 | -0.539311 | -0.441454 | -0.374722 | -0.179480 |
| -2.728652 | -1.967620 | -1.478516 | -1.741784 | -1.411759 | -0.858495 | -1.084024 | -1.171014 | -0.190453 |
| -0.302608 | 0.7021331 | -0.649284 | -0.377114 | 0.3234992 | 0.1282007 | -0.362018 | 0.2591053 | 0.1316866 |
| -0.188436 | -1.587040 | -0.328876 | -0.044042 | -1.727502 | -0.212571 | 0.0033480 | -0.305467 | -0.145677 |
| -0.313252 | -0.893068 | -0.198847 | -0.492917 | -1.523143 | -0.277483 | -0.001133 | -0.240106 | -0.223400 |
| -0.866301 | -1.021705 | -0.972741 | -0.962101 | -1.598310 | -0.545484 | -0.328879 | -0.306492 | -0.223823 |
| -0.137211 | -0.272134 | -0.265563 | -0.227587 | -0.095743 | 0.1356943 | 0.2099636 | -1.038169 | 0.2107322 |
| 0.2476739 | -0.176752 | -0.158862 | 0.2733351 | 0.0605890 | -0.450956 | -0.124592 | -0.003718 | -0.196789 |
| -0.043277 | -0.251257 | -0.026959 | -0.031707 | -0.634161 | -0.786749 | -0.579368 | -0.817595 | -0.525497 |
| -0.169913 | 0.0289257 | -0.592732 | 0.2441323 | 0.7677897 | -0.603971 | -0.193058 | 0.1118971 | -0.478750 |
| -0.248937 | -0.398187 | -0.199465 | -0.236552 | -0.172820 | -0.449962 | -0.693737 | -1.103918 | -0.394031 |
| -0.650559 | -1.817575 | -0.802646 | -1.005559 | -2.072007 | -1.426304 | -0.761572 | -1.753029 | -1.089008 |

| | | | | | | | | |
|---|---|---|---|---|---|---|---|---|
| -0.1230898 | -0.0931705 | -0.0053474 | -0.0540729 | 0.0226703 | -0.0052271 | 0.3314736 | -0.0934441 | 0.0943671 |
| -1.2224038 | -1.5389948 | -1.1015383 | -1.4558930 | -1.4685865 | -1.1845419 | -1.4975987 | -0.9409488 | -0.8380458 |
| -0.4362070 | -0.2169887 | -0.4606380 | -0.5499249 | -0.3881842 | -0.4753570 | -0.6082211 | -0.4203269 | -0.3912543 |
| -0.2250251 | 0.1218094 | -0.3582251 | -0.7370550 | -0.4933963 | -0.3972075 | -0.2343458 | -0.0658835 | -0.5841744 |
| -1.1404181 | -0.8154507 | -0.6857882 | -1.3236138 | -2.3468811 | -0.4302858 | -0.4774771 | -0.4665062 | -0.0463326 |
| 1.5235394 | 0.1854791 | 0.8297999 | 1.8870221 | 0.4477156 | -0.5530723 | 0.3486427 | -1.2005771 | -0.0672400 |
| -0.5834290 | -0.5871049 | -0.4796063 | -0.9430582 | -0.9826252 | -0.4197339 | -0.4270408 | -0.4993593 | -0.3149716 |
| -0.6512058 | -0.3273202 | -0.7975562 | -1.1743077 | -0.8973414 | -0.6380842 | -0.7767574 | -0.2207594 | -0.5178473 |
| -0.6439470 | -1.2533066 | -0.2122839 | -1.1126301 | -2.0810871 | -0.6996868 | -0.6844380 | -1.1530512 | -0.4323507 |
| -0.9000178 | -0.4098690 | -0.5158030 | -0.6931114 | -0.4281768 | -0.8926183 | -0.3970749 | -0.5771076 | -0.4619076 |
| -0.1048298 | -0.0649470 | -0.1969505 | 0.0746860 | 0.6858370 | -0.2674453 | 0.0668112 | -0.1175697 | -0.0530190 |
| -0.9272402 | -1.3194687 | -0.9555143 | -1.0619329 | -1.1448388 | -0.7544265 | -1.0695526 | -0.9030331 | -1.0656191 |
| -0.5723260 | -1.6318020 | -0.4864063 | -0.7493741 | -1.4139030 | -0.2205859 | -0.4949819 | -0.5432409 | -0.1131042 |
| -0.0873259 | -0.4414778 | -0.3555371 | -0.3089633 | 0.3331807 | -0.6998340 | -0.3036563 | -0.8405132 | -0.3585410 |
| -0.3590420 | -0.5347952 | -0.2343756 | -0.1109788 | -0.4907548 | -0.0969920 | -0.3132702 | -0.2042863 | -0.1725785 |
| -0.2143274 | -0.8099470 | -0.0041103 | -0.2637702 | -0.0635681 | -0.6939821 | -0.7674229 | -1.0905406 | -0.6152894 |
| -0.4315682 | -0.8910298 | -0.5463010 | -0.7103383 | -0.7396831 | -0.7050871 | -0.3829749 | -0.6670523 | -0.3018804 |
| -0.3036880 | -0.5290891 | -0.5995158 | -1.8077129 | -1.3711959 | -0.4733770 | -0.4807292 | -0.6348181 | -0.3843785 |
| -0.6243095 | 0.0029648 | -1.0728390 | -0.8418925 | -0.4332730 | -0.3136298 | -0.6655195 | -0.7147362 | -0.5952252 |
| -0.6926564 | -0.7204677 | -0.7113179 | -1.1314477 | -1.0291470 | -0.8390414 | -0.6483503 | -0.7906582 | -0.5386763 |
| -0.1817386 | 0.0313069 | -0.3834234 | 0.5136339 | 0.5795580 | -0.6901581 | -0.2401536 | -0.1718070 | -0.3344777 |
| -0.3113679 | 0.1129535 | -0.2218609 | -0.0291087 | -0.1019654 | -0.6861897 | 0.1514185 | -0.0641719 | -0.4750551 |
| -0.8842099 | -0.8450094 | -0.5841580 | -1.3784871 | -0.6041171 | -0.5529442 | -0.5895880 | -1.0656413 | -0.3085422 |
| -0.3307500 | -0.3057181 | -0.5884499 | -0.4833062 | -0.5424152 | -0.7628649 | -0.6762891 | -0.7363799 | -0.3069259 |
| -0.8014923 | -0.7962905 | -0.4345117 | -0.9383306 | -1.3629335 | -0.3225292 | -0.4123541 | -0.4035985 | -0.2852210 |
| -0.6008983 | 0.3174448 | -0.5737177 | -0.8165367 | 0.1805294 | -0.5362508 | -0.2444191 | -0.1556794 | -0.5335961 |
| -0.5293020 | -0.4113462 | -0.4562822 | -1.0992414 | -0.6942280 | -0.3857020 | -0.4507235 | -0.4833865 | -0.1691569 |
| -0.5513232 | -0.6375908 | -0.5873723 | -0.6970792 | -0.4363228 | -0.2309366 | -0.4715023 | -0.5927614 | -0.2669779 |
| -0.7464800 | -1.8181872 | -0.8461514 | -2.0235772 | -1.9375654 | -1.2043925 | -1.0054421 | -0.1657545 | -0.6909384 |
| 0.8173463 | 0.7516117 | 0.0920413 | 0.7041117 | 0.9542547 | -0.2734552 | 0.4615388 | 0.1844262 | 0.0265661 |
| 0.8949759 | 1.3598722 | 0.0299607 | 1.3474262 | 2.3029923 | -0.4510362 | -0.1316140 | 0.9542619 | -0.2635996 |
| -1.0787681 | -0.8664173 | -0.9147232 | -1.2627730 | -0.5575621 | -0.8174620 | -0.7120071 | -1.1724806 | -0.4579163 |
| -0.1747415 | 0.3132576 | -0.1278361 | -0.1475791 | 0.1985265 | -0.0391779 | -0.0260855 | -0.0513290 | 0.0125631 |
| 0.2425778 | 0.6500380 | 0.1346825 | 0.3193207 | 0.4778321 | -0.1281829 | 0.1970729 | 0.7942390 | -0.0534928 |
| 0.9348222 | 1.0656235 | 0.4262325 | 0.8428151 | 0.7408938 | 0.2931805 | 0.5241617 | 0.8614536 | 0.3083988 |
| -1.0417423 | -1.0909168 | -0.9115410 | -1.0828349 | -1.1408950 | -0.5014787 | -0.3484616 | 0.4460120 | -0.4487194 |
| 0.4615231 | 0.3125011 | 0.7585062 | 0.3858039 | 0.6286914 | -0.4164788 | 0.6000198 | -0.1663921 | 0.2355495 |
| -0.0528486 | -0.4374890 | -0.4306507 | 0.1673730 | -0.0822598 | -0.3957614 | -0.3366076 | -0.6217390 | -0.2524194 |
| -0.1284206 | 0.0438915 | -0.3624243 | -0.1225452 | 0.3358015 | -0.8014147 | -0.4646290 | -0.1140210 | -0.3206661 |
| -0.2286688 | -0.4209523 | -0.0956570 | -0.4197673 | -0.7116503 | -0.1142381 | 0.0836441 | -0.2872838 | 0.0020766 |
| -0.7321002 | -1.6873278 | -0.3542167 | -1.3409602 | -2.0242315 | -0.8332665 | -1.4422358 | -0.0457035 | -0.8034383 |
| -0.0400772 | 0.0164635 | -0.6344169 | -0.0824998 | 0.6691123 | -0.7312535 | 0.0315937 | -0.3675438 | -0.3466251 |
| 0.2638236 | 0.3385146 | -0.0348386 | 0.4596287 | 0.1778985 | -0.0580637 | 0.3905413 | 0.1433969 | -0.0895096 |
| -0.0553610 | 0.1625796 | -0.1631461 | 0.1167729 | 0.3053092 | -0.3011194 | 0.0508271 | 0.2847455 | -0.2362944 |
| -0.5115665 | -0.4725098 | -0.3334724 | -0.7121735 | -0.6276690 | -0.2982475 | -0.2733043 | -0.3934157 | -0.2699727 |
| -0.3182711 | -0.0557628 | -0.1623596 | -0.3680803 | 0.1654876 | -0.6261209 | -0.3483481 | -0.5602289 | -0.3917633 |
| -0.7774352 | -0.4506768 | -0.6590074 | -1.2504718 | -1.0350936 | -0.6138750 | -0.6184459 | -0.5301415 | -0.4329918 |
| -1.0251821 | -1.4846155 | -0.6778915 | -1.8664790 | -2.1185064 | -0.3163737 | -0.5739474 | -0.6104231 | -0.4281060 |
| -0.9169387 | -0.9501203 | -0.2994913 | -1.2162972 | -1.2577668 | 0.2350171 | 0.1254103 | -0.5761786 | -0.0664859 |
| -0.1887505 | 0.5088914 | -0.2080386 | -0.2204538 | 0.1595830 | -0.2791704 | -0.3351206 | -0.4634708 | -0.1872476 |

| | | | | | | | | |
|---|---|---|---|---|---|---|---|---|
| 0.3022027 | 0.9511279 | -0.126169 | 0.4489050 | 1.0331456 | -0.075624 | 0.4403233 | 0.2172152 | -0.048231 |
| -0.015182 | -0.594417 | 0.0320771 | 0.0908081 | -0.092730 | -0.880440 | -0.511992 | -1.158521 | -1.032189 |
| -1.072843 | -0.790303 | -0.715365 | -0.679659 | -1.053204 | -0.768783 | -0.208902 | -0.420881 | -0.601454 |
| -1.641207 | -1.143620 | -1.441244 | -1.867726 | -1.093545 | -0.193438 | -0.873506 | -0.077565 | -0.148301 |
| -0.931129 | -1.470560 | -0.853491 | -1.419990 | -1.517603 | -0.691068 | -0.813546 | -0.887968 | -0.646590 |
| 0.1242912 | 0.0095627 | -0.323852 | -0.086652 | -0.283749 | -0.324454 | -0.089553 | -0.446578 | -0.203240 |
| 0.5839612 | 1.0644648 | -0.119328 | 0.8619139 | 0.6734772 | -0.234766 | 0.0703153 | 0.3197477 | -0.024974 |
| -0.263204 | -0.583112 | -0.329006 | -0.463346 | -0.573948 | 0.1389438 | -0.314233 | -0.053839 | -0.067654 |
| -0.081239 | -0.752133 | 0.2096966 | -0.364266 | -0.455203 | -0.157429 | 0.0026842 | 0.0282791 | 0.0508094 |
| -0.687960 | 1.2209515 | -1.110725 | -0.668889 | 1.4606043 | -0.750065 | -0.771592 | -0.129284 | -0.342393 |
| -0.276581 | -0.008744 | -0.607577 | -0.026339 | 0.4156889 | -0.565827 | -1.126911 | 0.5712144 | -0.761710 |
| -1.124547 | -0.760230 | -0.879572 | -1.600285 | -1.007219 | -0.982616 | -1.088092 | -0.878282 | -0.818633 |
| -0.090103 | 0.2790367 | -0.036055 | 0.1240363 | 0.0924938 | -0.652982 | -0.562271 | -0.320232 | -0.432310 |
| 0.0395171 | -0.046289 | -0.100539 | -0.313643 | -0.269496 | -0.099052 | 0.1681289 | 0.0023438 | -0.017915 |
| -0.363271 | -1.041776 | -0.299768 | -0.523925 | -2.147215 | -0.657642 | -0.437701 | -1.467271 | -0.467404 |
| -0.247032 | -0.951178 | -0.219077 | -0.370815 | -1.099888 | -0.287426 | -0.407833 | -0.044077 | -0.156674 |
| -0.250443 | -0.587402 | -0.155476 | -0.237174 | -1.147696 | -0.266053 | 0.0194521 | -0.354211 | -0.158162 |
| -1.022713 | -0.824384 | -0.625700 | -1.459531 | -0.949349 | -0.496367 | -0.173750 | -0.130362 | -0.256435 |
| -0.798966 | -0.899951 | -0.196068 | -1.657218 | -1.253018 | -0.520535 | -0.436403 | -0.710705 | -0.195579 |
| -0.133987 | 0.2599123 | 0.2537673 | -0.037496 | 0.3964982 | -0.368775 | 0.7988246 | -0.172761 | 0.3049811 |
| -0.663984 | -0.623775 | -0.831216 | -0.773552 | -0.764836 | -1.231074 | -0.614893 | -0.960930 | -0.677546 |
| -0.259174 | -0.406312 | -0.262919 | -0.401689 | -0.227928 | -0.188220 | -0.242236 | -0.388113 | -0.239064 |
| -1.086181 | -2.187999 | -1.024547 | -1.816443 | -2.887381 | -0.385513 | -1.032764 | -2.176686 | -0.497965 |
| -0.257968 | -0.450148 | -0.347617 | -0.303871 | 0.0525139 | -0.211837 | 0.0487827 | -0.072294 | -0.239982 |
| -0.300760 | -0.731880 | -0.382787 | -0.701278 | -0.865088 | -0.112409 | -0.028704 | -1.006000 | 0.1040719 |
| -0.690268 | -0.930456 | -0.498695 | -0.957512 | -1.200850 | -0.594276 | -0.635963 | -0.722961 | -0.447410 |
| -0.249023 | -1.424636 | -0.281127 | -0.507377 | -1.455993 | -0.189600 | -0.159619 | -0.591083 | -0.325410 |
| -0.455346 | -0.646378 | -0.342751 | -0.785787 | -0.917425 | -0.360777 | -0.584918 | -0.599110 | -0.229817 |
| -0.041000 | -0.189557 | -0.333800 | -0.060427 | 0.2205038 | -0.253946 | -0.072278 | -0.008351 | 0.0282809 |
| -0.093578 | 0.6865753 | 0.1121980 | 0.3342304 | 1.1140441 | -0.247429 | 0.0527047 | 0.3544801 | -0.039584 |
| -0.324075 | -0.788795 | -0.874422 | -0.479329 | -1.215794 | -0.330604 | -0.259248 | -0.362537 | -0.303088 |
| -0.399242 | -0.308085 | -0.394545 | -0.422773 | -0.440881 | -0.183805 | -0.213360 | -0.229692 | -0.137992 |
| -0.548335 | 0.0352892 | -0.735360 | -0.417915 | -0.092165 | -1.345486 | -0.839153 | 0.1197109 | -0.764257 |
| -0.453608 | -0.688267 | -0.509573 | -0.811100 | -1.248072 | -0.413302 | -0.301285 | -0.985095 | -0.343851 |
| -0.677478 | -0.262689 | -0.452566 | -0.202991 | -0.405318 | -0.365583 | -0.422276 | -0.648571 | -0.226966 |
| -0.249253 | -0.458540 | -0.267126 | -0.393187 | -1.095157 | -0.379994 | -0.324996 | -0.602917 | -0.262216 |
| -1.021367 | -2.013901 | -0.779347 | -1.734733 | -2.888945 | -0.362197 | -1.237910 | -1.370910 | -0.387014 |
| -0.079009 | -0.131271 | -0.238559 | -0.161975 | -0.158193 | -0.083520 | -0.214251 | -0.264185 | -0.210177 |
| 1.0617967 | 1.4507895 | -0.149247 | 1.2772443 | 1.3046759 | -0.396765 | -0.395430 | 0.0865693 | -0.304297 |
| -0.807141 | -0.339667 | -0.533245 | -1.052750 | -0.943120 | -0.812130 | -0.296954 | -0.144527 | -0.389719 |
| 0.2821747 | 1.0479679 | -0.208770 | 0.3077685 | 1.2070419 | -0.280955 | 0.1294028 | 0.0558627 | -0.211702 |
| -1.033763 | -1.128889 | -0.670296 | -1.503351 | -1.869288 | -0.468008 | -0.647737 | -0.610934 | -0.367226 |
| -0.997470 | -0.301891 | -0.837880 | -1.069246 | -1.503773 | -0.691412 | -0.740055 | -0.779710 | -0.486369 |
| -0.747762 | -0.348513 | -0.568791 | -1.207162 | -0.587318 | -0.334500 | -0.853774 | 0.6142155 | -0.444149 |
| -0.418096 | -0.834731 | -0.487852 | -0.548663 | -0.739741 | -0.619590 | -0.406056 | -0.702020 | -0.495540 |
| -0.456497 | -1.262569 | -0.355319 | -0.903682 | -0.986617 | -1.080987 | -0.707353 | -0.289427 | -0.573789 |
| -0.391240 | -0.146235 | -0.352249 | -0.669906 | -0.700777 | -0.489613 | -0.228774 | -0.350390 | -0.457775 |
| -0.898962 | -0.912171 | -1.055416 | -0.837582 | -0.566132 | -0.989874 | -1.232648 | -0.621327 | -0.969191 |
| -0.663423 | -0.475372 | -0.156988 | -0.334555 | 0.1353872 | -1.054570 | -0.019637 | -0.628941 | -0.641538 |
| -0.974680 | -1.553214 | -0.654648 | -1.053525 | -1.604633 | -0.921684 | -0.896024 | -1.057962 | -0.722354 |

| | | | | | | | | |
|---|---|---|---|---|---|---|---|---|
| -0.9526377 | 0.1570372 | -0.5209786 | -0.2081905 | 1.1954696 | -0.8811046 | -0.3321197 | -0.5355678 | -0.5787031 |
| -0.6106212 | -0.7943897 | -0.5428227 | -1.0684803 | -1.1457727 | -0.6255059 | -0.4700294 | -0.1198328 | -0.4219905 |
| -0.2242195 | 0.3380146 | -0.3903299 | -0.3715251 | 0.3123700 | -0.3873396 | -0.2353444 | -0.4486201 | -0.3452450 |

| PG_489 | PG_490 | PG_491 | PG_492 | PG_493 | PG_494 | PG_495 | PG_496 | PG_497 |
|---|---|---|---|---|---|---|---|---|
| -0.4614474 | -1.4852530 | -2.0795630 | -0.5664252 | -1.0286752 | -0.0107163 | -1.1942190 | -0.6496807 | -1.2556548 |
| -0.6725131 | -1.5768601 | -2.0811370 | -0.6972360 | -1.6723645 | -0.8917121 | -1.3258575 | -0.6115495 | -1.0472605 |
| -1.3171852 | -0.7344945 | 0.4787952 | -1.7173453 | 0.0748647 | 0.0725690 | -1.5473489 | -0.2205840 | -1.1856445 |
| 0.0817607 | -0.0905304 | -2.9296033 | -1.2040828 | -2.7249970 | -1.0901615 | -1.6170784 | -0.1147465 | -0.3171151 |
| 0.0815867 | -0.8742785 | -1.7950085 | -1.1535418 | -1.6099193 | -0.5199078 | -1.6720059 | 0.0071289 | -0.1666936 |
| -0.7458989 | -2.6240611 | -0.5353547 | -1.1733824 | -0.7953272 | -0.8092085 | -2.0707635 | -0.4351500 | -0.7032697 |
| -0.4335555 | -0.7366929 | -0.8616279 | -0.9448141 | -1.1797857 | -0.9289838 | -0.5401392 | -0.1115228 | -0.5147957 |
| -0.3322503 | -1.6895706 | -0.8166184 | -1.1698766 | -0.4291749 | -0.3214451 | -1.9533711 | -0.0672454 | -0.2072341 |
| -0.2778811 | -1.2511007 | -0.9449261 | -0.1042460 | -0.7032328 | 0.4422240 | -0.4292733 | -0.2007608 | -0.1918837 |
| -0.1253467 | 0.1709650 | -0.1959676 | 0.0177954 | -0.4003764 | -0.1483091 | 0.1727840 | -0.4681079 | -1.3498595 |
| -0.3122354 | -0.6991086 | -1.8384041 | -1.3034521 | -1.2808078 | -0.7982877 | -1.9737941 | -0.0757751 | -0.2396686 |
| -0.5606083 | -0.3521129 | -1.7992260 | -0.8871469 | -1.9676156 | -0.8210667 | -1.0229321 | -0.1875481 | -0.4368434 |
| -0.6457443 | -1.3050353 | -1.4424744 | -1.6017402 | -0.7961670 | -1.4620729 | -2.9440885 | -0.7861493 | -0.9059308 |
| 2.100505 | 5.7055434 | -1.3161650 | 2.8615687 | 0.1306776 | 2.7235562 | 2.7221091 | 0.3706372 | 4.3419416 |
| 0.0210248 | 0.1239574 | -2.1136898 | -0.8684554 | -2.3720563 | -0.9308424 | -1.2183809 | 0.1049518 | -0.3280455 |
| 1.1523320 | 1.0358813 | -0.7637218 | 2.2292182 | -0.2012124 | 1.9417289 | 2.4332148 | -0.4377262 | 2.4722916 |
| -2.5396885 | -1.4461276 | -1.7052883 | -0.1942513 | -0.9235043 | -1.1268325 | -0.1695503 | -1.0463236 | -1.1421019 |
| -2.0836909 | -1.1180106 | 0.2151295 | 0.2035514 | 0.3415225 | 0.1670556 | 0.3882105 | -0.7870039 | -0.6772394 |
| -0.6959476 | -0.2037594 | 0.0693964 | 0.1505294 | -0.0150703 | -0.0846727 | 0.2502791 | -0.2498250 | -1.6313345 |
| -0.7062659 | -0.6277914 | -0.6525809 | -0.2557915 | -0.4636928 | 0.6603504 | -0.5839603 | -0.5166998 | -0.6234778 |
| 0.0494813 | -0.8016165 | -1.7684755 | 0.1504139 | -2.3226891 | -0.2677613 | -0.2023281 | -0.2389505 | 0.3805283 |
| -0.6817328 | -0.4125597 | -0.0607983 | -0.4691810 | -0.3911031 | -0.7178062 | -0.5979824 | -0.5287332 | -0.2533638 |
| 0.6762242 | 0.9880331 | 0.0355629 | 0.7824896 | 0.5474134 | 0.9002086 | 0.5436899 | -0.0781471 | 0.5525486 |
| -1.1950741 | -1.8975408 | -1.3224534 | -2.1356148 | -0.7713020 | -1.1722582 | -2.2326515 | -0.2951965 | -1.1452645 |
| -1.7858474 | -2.5194685 | -0.8238229 | -2.3171643 | -0.9957708 | -1.3549925 | -2.7120433 | -0.1578338 | -0.8889785 |
| 0.3601389 | -0.0841172 | 0.2723618 | 1.1833842 | 0.2258542 | 0.7630139 | 0.9764524 | -0.1617460 | 0.2716358 |
| 0.2325812 | -0.5642689 | -2.2803915 | -1.3146840 | -2.6504114 | -1.1261958 | -1.9645146 | 0.1510326 | -0.1025473 |
| -1.2328421 | -1.9203784 | -2.9924835 | -1.5670140 | -2.6309631 | -1.4030136 | -2.4484095 | -0.4966219 | -0.4482649 |
| -0.8735422 | -1.4011419 | -0.6053619 | -0.1944163 | -0.4169612 | -0.6854909 | -0.2831647 | -0.3114130 | -1.0684198 |
| -0.5074340 | -0.3915891 | -1.7656825 | -1.1373878 | -2.1072304 | -1.5685132 | -1.4201762 | -0.0809478 | -0.1935528 |
| -2.4695045 | -2.1358354 | -0.4328528 | -1.5979394 | -0.3520349 | -0.1389673 | -2.0096679 | 0.6486726 | 0.1873897 |
| 0.0647031 | -0.8701674 | 0.5152458 | 0.3209339 | 0.3065481 | -0.1341476 | 0.3304955 | 0.7895862 | 0.2871773 |
| 0.5483412 | 0.0719774 | -1.5837589 | -0.0656022 | -1.4069509 | -0.2871178 | -0.2710768 | -0.0842574 | -0.1840038 |
| -0.7970980 | -2.3279369 | -1.1603524 | -2.0836600 | -0.4604048 | -1.6137707 | -2.9014627 | -0.8537738 | -1.0509252 |
| -0.3216559 | -0.0326658 | -0.0340548 | -0.0395609 | -0.1088525 | 0.0119891 | 0.1619265 | 0.2368353 | -0.2970989 |
| -0.9298115 | -0.1180548 | -1.9788360 | -0.1140840 | -1.6687942 | -1.5939127 | -0.1868834 | -0.3368379 | -0.5865921 |
| -0.4530370 | -1.5390313 | -0.5577851 | -1.8197662 | -0.6665087 | -1.1794343 | -1.9172905 | -0.3517007 | -0.6293498 |
| 3.0154070 | 0.3030625 | 0.5656802 | 2.0194371 | 1.3104806 | 3.3294592 | 0.8817606 | 1.1641195 | 3.0277790 |
| -0.2356539 | 0.1397042 | -1.2883109 | -0.0679972 | -0.8231541 | 0.2127949 | -0.0046763 | -0.3995119 | 0.3484262 |
| -0.9220974 | 1.4896010 | -1.3032647 | -0.4978474 | -1.1461199 | -0.5857803 | -0.1257841 | -0.6584742 | -0.6567144 |
| 0.4473227 | -0.2727060 | 0.8860006 | 1.2429682 | 0.8077032 | 0.8247789 | 1.6284542 | -0.1113480 | 0.0828699 |
| -0.7650126 | -2.0556906 | -0.1910973 | -1.5580382 | -0.2593429 | -0.9450282 | -2.3594896 | -0.4926210 | -0.9565976 |
| -0.9624755 | -0.3481185 | -1.4151438 | -1.1965342 | -1.1057380 | -0.4751160 | -1.5558611 | -0.5936212 | -0.8708100 |
| -0.1608775 | -1.0240633 | -2.0378501 | -1.7931149 | -2.1200641 | -1.0524678 | -2.2529938 | 0.2264457 | 0.1170801 |
| 0.1809183 | 0.1833056 | -0.0059577 | 1.7496124 | 0.8226940 | 1.1250146 | 2.0200222 | 0.0945358 | 0.1590448 |
| 0.0021014 | 4.2833868 | 0.6805177 | -0.0731991 | 0.0542763 | 0.5929355 | -0.1266931 | -0.1224031 | 0.7590106 |
| 0.0542511 | 0.1569984 | 0.2193435 | -0.3874532 | 0.0060953 | 0.0190409 | -0.5856268 | -0.3793494 | -0.5897559 |

| | | | | | | | | |
|---|---|---|---|---|---|---|---|---|
| 0.2134929 | -0.3241663 | -1.4967985 | -0.9063944 | -2.0665585 | -0.7988844 | -1.0294603 | -0.2220964 | 0.2080471 |
| -0.5537388 | -1.3470887 | -1.4340779 | -1.7330571 | -1.0300134 | -1.0746299 | -2.6468614 | -0.5953506 | -0.8669603 |
| -0.0632314 | -0.0899908 | -2.2931874 | 0.0477547 | -1.5326062 | -0.6166926 | -0.2729312 | -0.4188602 | 0.0770927 |
| 1.0669178 | 1.4377690 | -0.1819483 | -0.0717845 | -0.2043047 | -0.0506683 | 0.0910314 | -1.2454569 | 0.7610249 |
| -0.1711423 | -0.1716784 | -0.3888413 | -0.1271564 | -0.2191683 | -0.3181991 | -0.0336870 | -0.5830859 | -0.6676265 |
| -0.1104834 | -0.1172201 | -0.0104839 | 0.2211000 | -0.0509268 | -0.3015067 | 0.7605438 | -0.2025115 | -0.2525781 |
| -0.7508978 | -1.3302048 | -0.5020396 | -0.6004896 | -0.5969369 | -1.3459389 | -0.9807819 | -0.4717909 | -0.7959524 |
| 2.222092 | 3.5472939 | 0.3214364 | 2.3455168 | 0.6720199 | 1.8905047 | 2.3448681 | 0.3286851 | 2.5722233 |
| 0.8748022 | -0.2324244 | -0.3716158 | 0.0783142 | 0.6659777 | 1.9155223 | -0.1175858 | 0.8561887 | 3.7819745 |
| -0.3587911 | -1.5657198 | -1.2240856 | -0.6608466 | -1.5802136 | -1.3985707 | -1.0220512 | -0.5565739 | -0.1568364 |
| -1.3610107 | -1.7610206 | -1.7934604 | -1.4429193 | -1.4057768 | -1.5195361 | -2.0864544 | -0.5778281 | -0.8574956 |
| 2.0141462 | 1.5019694 | -0.9747204 | 1.6597285 | -0.1728111 | 1.9612031 | 2.3617663 | 0.0760751 | 3.2220902 |
| 1.6287244 | 0.5995272 | -0.4498649 | 0.0931451 | -0.4534510 | 0.8859088 | -0.2144710 | -0.0437493 | 2.9133775 |
| -0.5973865 | -1.2534606 | -1.9061477 | -0.5543144 | -1.0496740 | -1.0297205 | -1.0450310 | -0.8786691 | -0.8764900 |
| -1.9498462 | -2.1417824 | -1.7804664 | -2.8219744 | -2.3268979 | -2.2487434 | -3.4467302 | -0.8356755 | -1.2775805 |
| -0.5527763 | -0.7942721 | -0.5795064 | -0.7050632 | -0.9545203 | -1.3728371 | -0.9536283 | -0.3433492 | -0.6549981 |
| -0.4119656 | -1.1880608 | -1.1397074 | -2.6535666 | -1.3733751 | -1.2700795 | -3.1483929 | -0.5307630 | -0.8665797 |
| -0.5629769 | -1.4008679 | -1.5159344 | -1.0186283 | -1.5578170 | -0.9461211 | -1.4809546 | -0.2445462 | -0.5196137 |
| -0.7002675 | -2.0651133 | -2.2388316 | -1.6210280 | -1.9145575 | -1.4741145 | -2.2408021 | -0.4410907 | -0.5437158 |
| 0.1650005 | -1.6045916 | -2.5140148 | -2.0569102 | -3.0077507 | -1.3907948 | -2.3891271 | 0.2781859 | 0.2084498 |
| -0.1963779 | -0.2194315 | -0.4302360 | 0.1593040 | -0.2363308 | 0.0034451 | 0.1642159 | -0.3698042 | -0.1325453 |
| -0.7639087 | -1.7047408 | -1.0368738 | -1.4903867 | -0.9336123 | -1.3707268 | -1.6238482 | -0.7100173 | -0.8558061 |
| -0.2741791 | -0.9931993 | 1.0560916 | 1.0138767 | 0.8669800 | 0.2380518 | 0.3812899 | 0.2479175 | -0.1270520 |
| 0.6992100 | -0.0228801 | -2.8627148 | -1.8596456 | -2.7295793 | -1.9268030 | -2.5155421 | 0.5599039 | 0.7825644 |
| -0.6889135 | -2.2588847 | -1.5086506 | -2.1574343 | -1.3411905 | -1.1511432 | -2.8761607 | -0.2988578 | -0.4905163 |
| -0.4628528 | -1.0028782 | -1.1082209 | -0.2110721 | -1.3229848 | -0.8118218 | -0.1806721 | 0.0906095 | -0.2494027 |
| -2.4635401 | -1.8471539 | 0.2228152 | -0.7621583 | 0.1823324 | -0.8776201 | -0.5795478 | -0.7291731 | -2.1957418 |
| 1.3271280 | 3.7510500 | -0.1083262 | 2.4739512 | 0.3866509 | 0.9347488 | 1.8575758 | 0.4732443 | 2.4401431 |
| 1.2460974 | 2.7711771 | -0.9645681 | 1.2075750 | -0.1389926 | 1.1551291 | 1.8154081 | 0.2082131 | 1.7670187 |
| -0.4731099 | -1.6787897 | 0.0136542 | -1.2226766 | -0.6467646 | -1.3384621 | -1.3121749 | -0.2573408 | -0.4797251 |
| -0.4056155 | 0.2424804 | 0.1417249 | 0.3089034 | 0.3709512 | 0.1525381 | 0.2636247 | -0.3872813 | -0.5635567 |
| -0.2761150 | -0.5952787 | -3.1070987 | -1.7188350 | -3.3278529 | -1.4363519 | -2.5629796 | -0.0029547 | -0.4057171 |
| -1.5888450 | -1.6677651 | -1.1160333 | -1.8492868 | -1.2698415 | -1.8872043 | -2.1026526 | -0.5048842 | -1.3428503 |
| -0.6959417 | -2.0680084 | -0.7469329 | -1.4196843 | -0.8457266 | -0.9976742 | -1.7999314 | -0.3658566 | -0.8329238 |
| -0.1662359 | -0.1564994 | -0.1610670 | 2.0158143 | -0.2114826 | 1.4348395 | 2.1387239 | -0.2660395 | 0.8732868 |
| 0.0877831 | -0.4987157 | 0.1737922 | -0.0615468 | 0.4544885 | 0.0658444 | 0.5182927 | -0.1854454 | -0.3304124 |
| -0.2605791 | -0.2288679 | 0.2065093 | -0.2727318 | -0.3141124 | -0.1200070 | -0.0240446 | 0.0407045 | -0.5089333 |
| -0.9512844 | -1.5199104 | 0.3164659 | -1.6096838 | -0.4363278 | -0.7449144 | -2.7559756 | -0.2439382 | -0.5947665 |
| -0.8451955 | -1.8357277 | -0.0403387 | -1.4695860 | 0.1814230 | -0.5067156 | -2.9303464 | -0.0572491 | -0.4580286 |
| -1.3636480 | -2.3443359 | -0.1157122 | -0.7138849 | -0.0855886 | -0.8037799 | -1.1928444 | -0.4752268 | -1.5847437 |
| -0.6866452 | -1.9003131 | -0.9011588 | -0.8351720 | -0.9716396 | -0.7658404 | -1.8226643 | -0.8462098 | -1.6865435 |
| -0.6956157 | 0.5366240 | -0.8955770 | -1.1409818 | -0.6431708 | -0.1311027 | -1.2154695 | -0.2796213 | -1.4725570 |
| 0.0823256 | -0.4308533 | -0.7156033 | 2.9309023 | 0.3778941 | 2.7520347 | 2.1855890 | -0.1743631 | 1.8640137 |
| -0.1716050 | -0.0973405 | -1.0487763 | -0.5768365 | -0.8973016 | -0.5725167 | -0.6254732 | -0.2295353 | -0.1316048 |
| -0.4133825 | -0.6751762 | -2.0128789 | -1.2034207 | -1.9126104 | -0.8614559 | -1.6187966 | 0.0970660 | -0.0824222 |
| -1.3739952 | -1.9207564 | -0.4745559 | -0.5096637 | -0.3637970 | -0.6111356 | 0.0019278 | -0.4264681 | -0.9032369 |
| 2.7525362 | 4.0930440 | -0.0672606 | 3.9201597 | 0.0131241 | 4.4579298 | 6.4038794 | -0.2682460 | 5.0257253 |
| 0.9097637 | 0.6550535 | -0.3305799 | 1.3378747 | -1.2601978 | 1.0233227 | 1.7488244 | -0.1650345 | 1.5587060 |
| -0.7785437 | -2.1287849 | -2.0164018 | -1.4355626 | -1.2941712 | -0.4258623 | -0.8693185 | -0.1418271 | -0.3897340 |
| -0.2823004 | -0.7591278 | -0.4951074 | -0.9636630 | -0.2680482 | -0.2966479 | -1.2684225 | -0.3708512 | -0.2168501 |

| | | | | | | | | |
|---|---|---|---|---|---|---|---|---|
| -1.6554579 | -2.0079417 | -0.3471402 | -0.4646678 | -0.4216657 | 0.5505102 | -1.6087071 | 0.1104004 | -0.3626848 |
| -0.3081573 | 0.0205499 | -0.5093438 | 0.0550381 | -0.0693888 | -0.2510953 | -0.1594653 | -0.1558490 | -0.1896372 |
| -0.8035224 | -0.9059242 | -0.1600258 | -0.4806213 | -0.5523664 | -0.5519158 | -1.0387552 | -0.1704934 | -0.3958814 |
| 0.0760785 | -0.0870442 | -0.9140234 | -0.2649724 | -0.7579392 | -0.0490543 | -0.2229154 | -0.1049147 | 0.0161707 |
| -1.6961716 | -1.7888430 | -2.1054942 | -1.6925268 | -1.1647161 | -1.8529370 | -2.3171913 | -0.2198792 | -1.1679134 |
| 0.1539963 | -1.2610354 | -1.4546975 | -0.5751575 | -1.4470692 | -1.0284299 | -0.0942596 | -0.5467163 | -0.4575977 |
| 0.4091937 | 0.0231987 | -2.3862853 | -1.4139430 | -2.2962187 | -1.2330172 | -2.0237205 | -0.2318654 | -0.1357812 |
| -0.0772548 | 0.3093321 | -0.1142902 | 0.1490575 | 0.1485036 | -0.1514853 | 0.1518996 | -0.0478635 | -0.1527218 |
| -0.2456035 | -0.2718966 | 0.7641590 | -0.3916577 | 0.6611141 | -0.3792698 | -0.1291348 | -0.3053154 | -0.4079376 |
| 0.7002018 | 0.3556158 | -1.2479944 | 0.5684171 | -0.7940460 | 0.7881064 | 0.7851817 | 0.0072945 | 0.6205715 |
| -0.2826311 | -0.8857207 | -0.8427612 | -1.6434659 | -0.7345844 | -1.1677385 | -1.9078830 | -0.2268519 | -0.2775027 |
| -0.8362766 | -1.5396818 | -1.8349910 | -1.4906965 | -1.8024497 | -1.3780401 | -2.7332281 | -0.1769328 | 0.1317898 |
| -0.5482739 | -1.7319155 | -0.5568472 | -2.0471392 | 0.0992881 | -0.9491019 | -2.6701434 | -0.6558004 | -1.1154863 |
| -0.2083123 | -0.7715870 | -0.5912962 | -0.7248171 | -1.0010537 | -0.9178989 | -1.0209142 | -0.5449918 | -0.4407852 |
| -0.5612838 | -1.2637592 | -1.0009218 | -0.2137144 | -0.8651824 | -0.3967844 | -0.5719452 | -0.1176995 | -0.3089986 |
| -0.1328726 | -0.3073335 | -0.0480247 | 0.0623971 | 0.2917615 | -0.1403844 | -0.1265989 | -0.1457358 | -0.0221759 |
| -0.9295132 | -0.5510182 | -0.3649253 | -0.9939566 | -1.4450644 | -1.1759504 | -1.6175797 | -0.3357157 | -0.2629602 |
| -0.1984383 | -0.0571610 | 0.0897129 | 0.1467331 | 0.1312526 | 0.1379613 | 0.0977925 | -0.1138999 | -0.1898682 |
| -0.6267660 | -0.9031802 | 0.1066503 | -0.4070028 | -0.2728107 | -0.0226085 | -1.1838455 | 0.0758617 | -0.1486807 |
| 0.6742118 | 1.0220236 | 0.8625004 | 1.4219080 | 1.0710044 | 0.7823979 | 0.9842095 | -0.1191457 | 0.6016260 |
| -0.5683848 | -1.2260950 | -1.7187185 | -2.2016391 | -1.5638628 | -1.3598246 | -3.4846630 | -0.3478206 | -0.7239982 |
| -0.6964096 | -1.2403860 | -0.6830999 | -0.9767380 | -1.1705452 | -0.9094434 | -1.4760026 | -0.0082276 | -0.1646087 |
| -0.1771712 | -0.1631581 | -0.0734740 | -0.0607740 | -0.1620261 | 0.0209050 | -0.1301429 | -0.2182456 | -0.2132854 |
| -1.0138515 | -1.6688001 | -0.5398573 | -0.9304428 | -0.4362472 | -0.2369312 | -1.7332702 | -0.1867309 | -0.4629061 |
| -0.6948975 | -0.1398737 | -0.2274190 | -0.3591004 | -0.4193885 | -0.5554477 | -0.2678247 | -0.0729096 | -0.8566726 |
| -0.6338725 | -0.6609024 | -0.6419367 | -0.8863814 | -1.4823541 | -1.3836370 | -1.5025285 | -0.1477151 | -0.0630453 |
| -0.1860814 | -0.2982843 | -0.4143955 | -0.0338467 | -1.9350262 | -0.7766662 | -0.7005744 | 0.0121189 | -0.1182525 |
| -0.0281258 | -0.5326590 | 0.3311948 | 0.8789719 | 0.3753655 | 0.7804687 | 0.5636490 | -0.4868936 | 0.0569393 |
| -0.8995735 | -0.2001640 | 0.1480614 | -1.2643016 | -0.4928100 | -1.0598017 | -1.3249212 | -0.3714487 | -1.1568877 |
| -0.0582673 | -1.1481830 | -0.8979942 | -0.4125414 | -1.4527030 | -0.6612694 | -0.6610111 | -0.4879074 | -0.4502655 |
| -0.7760829 | -2.1923852 | -0.1645105 | -1.2608132 | -0.1526918 | -1.3010087 | -2.0975784 | -0.8670834 | -1.6199822 |
| -0.9879684 | -1.6318658 | -1.5903605 | -0.9935721 | -1.3764639 | -0.6226232 | -1.5781638 | -0.2769381 | -0.5504757 |
| -1.0697931 | -0.6957436 | -0.6526360 | -0.2244740 | -0.8187085 | -0.3440186 | 0.0059613 | -0.7827785 | -0.8194923 |
| 0.3489231 | -0.2886103 | -0.9916568 | -0.2215719 | -0.6402516 | 0.0593454 | -0.8914780 | 0.4791152 | -0.0666842 |
| -0.4125971 | -1.3956753 | -0.7870796 | -1.2814607 | -0.6377829 | -0.6656262 | -2.0227227 | -0.2142077 | -0.3626785 |
| -1.1871781 | -1.6781062 | -1.2366893 | -1.5678772 | -1.4791027 | -1.3285052 | -2.1418163 | -0.0316902 | -0.4975954 |
| -0.3811274 | -0.5701975 | -1.5247629 | -1.0777314 | -1.4950141 | -0.5468793 | -1.7858318 | -0.1632261 | -0.2965513 |
| -0.1238615 | -1.0261444 | -2.2537737 | -0.8266564 | -2.1287381 | -0.8067439 | -1.3873076 | -0.1589298 | -0.2628189 |
| -0.5955884 | -0.8659110 | -0.6198451 | -0.9282050 | -0.8763782 | -0.6973889 | -1.0224783 | -0.4158819 | -0.2564391 |
| -0.2350923 | -1.0762529 | -0.8813242 | -0.9822983 | -0.2470442 | -0.7466866 | -1.2638492 | -0.3998477 | -0.712951 |
| 0.4933594 | 1.0043361 | -1.9252069 | 0.5262682 | -1.2209511 | 0.1884083 | 0.8987049 | -0.1926332 | 0.9885488 |
| 0.1804076 | -0.6268844 | -1.1406765 | -0.4382523 | -1.317782 | -0.1805478 | -0.8958168 | -0.2115505 | -0.0259714 |
| -0.1619314 | -1.2336879 | -1.6869003 | -0.4976103 | -1.4986264 | -0.9331137 | -1.0891041 | 0.1209863 | -0.1565148 |
| -0.5613568 | -0.8733002 | -0.8131674 | -1.5849205 | -0.4536946 | -0.5923772 | -2.5014143 | -0.6362480 | -0.0233937 |
| -0.0600140 | -2.0439010 | 0.0125963 | -0.0034663 | -0.1351059 | -0.2184128 | -0.1182878 | -0.2227933 | -0.1403108 |
| 0.2168345 | 0.3555647 | -0.7921481 | 0.2946735 | -0.9184346 | 0.1966025 | 0.1384545 | -0.2150028 | 0.4949827 |
| -0.4332696 | -0.9900484 | -0.8644305 | -0.3610195 | -0.7217258 | -0.3081714 | -0.7240665 | -0.4909775 | -0.5491449 |
| 0.3055634 | 0.4705101 | -1.1122350 | 0.1927821 | -1.1137607 | 0.0210889 | -0.2907768 | -0.1631102 | 0.4549494 |
| -0.7861960 | -1.4163456 | -1.3227949 | -2.4605899 | -0.9271274 | -1.2763373 | -2.6419827 | -0.1840513 | -1.1302678 |
| -0.8607862 | -2.8663973 | -0.8022361 | -1.7483346 | -1.3837969 | -1.1114624 | -1.7940574 | -0.6554958 | -0.6019134 |

| | | | | | | | | |
|---|---|---|---|---|---|---|---|---|
| 0.3668116 | -0.190972 | -2.235061 | -0.983204 | -2.213562 | -1.245372 | -1.684767 | 0.3889634 | 0.7274966 |
| -1.889923 | -1.813691 | -0.904743 | -1.384175 | -0.490633 | -1.747335 | -1.572216 | -0.796328 | -1.152355 |
| -0.585994 | -0.866088 | -1.518581 | -0.178268 | -1.103856 | -0.890857 | -0.871837 | -0.759468 | -0.600678 |
| -0.007611 | -0.103220 | 0.0321399 | -0.181012 | 0.2152447 | -0.444850 | -0.016209 | -0.752523 | -0.201634 |
| -1.099716 | -1.870389 | -1.941550 | -2.369572 | -1.522419 | -1.542724 | -2.225793 | -0.157305 | -0.828362 |
| 0.7001090 | -0.907658 | 0.6804929 | 1.3071016 | 0.5816184 | 2.0765143 | 0.6387884 | 0.0896630 | 1.4437266 |
| -0.458490 | -0.859297 | -1.249603 | -0.778806 | -0.960651 | -0.485808 | -1.263383 | -0.321522 | -0.308645 |
| -0.863799 | -1.520563 | -1.107151 | -1.058000 | -1.197869 | -1.038162 | -1.899910 | -0.445055 | -0.533920 |
| -1.287121 | -2.027636 | -1.271111 | -1.311667 | -0.899545 | -1.476866 | -1.824118 | -0.724705 | -1.338053 |
| -0.743239 | -0.352144 | -1.450152 | -0.146168 | -0.895879 | -0.366340 | -0.334095 | -0.291495 | -0.442885 |
| 0.0700992 | -0.374154 | -0.880073 | 0.2899465 | -0.756681 | -0.180937 | 0.1171148 | -0.030597 | -0.000782 |
| -1.513962 | -1.814131 | -0.208562 | 0.0087397 | -0.371542 | -0.679190 | -0.000432 | -0.538517 | -1.863585 |
| -1.012010 | 0.3511165 | -0.005263 | -0.729079 | -0.119724 | -0.101037 | -0.985182 | 0.1019353 | -0.685560 |
| 0.0740625 | -0.761532 | -1.012343 | -0.315853 | -0.466530 | -0.628408 | -0.668153 | -0.345089 | -0.038229 |
| -0.290947 | -0.120841 | -0.006083 | -0.259446 | -0.374570 | -0.528925 | -0.081417 | -0.277389 | -0.079240 |
| -0.593325 | -1.034994 | -0.794357 | -0.488094 | -0.620765 | -0.606981 | -0.433877 | 0.3011414 | -0.279381 |
| -0.186997 | -0.770395 | 1.0506351 | 1.0227091 | 1.4005210 | 0.2266859 | 0.7396488 | -0.886505 | -0.784481 |
| -1.343787 | -3.032019 | -0.656444 | -1.616100 | -0.795780 | -2.021082 | -2.855379 | -0.341827 | -1.043238 |
| -0.547749 | -1.023238 | -1.115882 | -0.893684 | -0.922643 | -1.429607 | -1.493881 | -0.549296 | -1.005175 |
| -1.029698 | -2.156375 | -1.887133 | -1.818673 | -2.143998 | -1.529830 | -2.397640 | -0.134056 | -0.630765 |
| -0.158579 | 0.0843775 | 0.5923965 | 0.5022391 | 0.5025638 | 0.2911313 | 0.8194985 | -0.482446 | 0.2554003 |
| 0.3401698 | -0.693108 | -0.644869 | -0.246402 | -0.885714 | -0.513921 | -0.567283 | -0.025798 | 0.5523084 |
| -1.133457 | -1.494173 | -0.401482 | -1.148879 | -0.348209 | -1.397001 | -1.817998 | -0.423030 | -1.221315 |
| -0.254959 | -1.285881 | -1.324931 | -0.572329 | -1.211552 | -0.969148 | -0.881007 | -0.407847 | -0.602434 |
| -0.744967 | -1.305353 | -0.276133 | -1.870194 | -0.333971 | -0.996637 | -2.260757 | -0.137545 | -0.421939 |
| -0.563981 | -0.145321 | -0.019255 | -1.029555 | -0.997113 | -2.108153 | -1.708756 | -0.464211 | -0.676393 |
| -0.748698 | -0.655835 | -0.429463 | -0.952899 | -0.479469 | -0.794148 | -1.627354 | -0.425738 | -0.576338 |
| -0.858213 | -0.505734 | -0.731699 | -0.863326 | -0.836652 | -0.787319 | -0.938287 | -0.122168 | -0.236443 |
| -1.348286 | -1.651636 | -1.546283 | -0.994605 | -1.040780 | -1.382282 | -2.391256 | -0.534566 | -0.850174 |
| 0.6311734 | 0.1476611 | -1.506268 | 0.5297125 | -0.834029 | 0.1909307 | 0.0470843 | -0.059364 | 0.6991223 |
| 1.6302680 | 3.3898414 | -0.175934 | 1.7800386 | 1.0105948 | 2.4527987 | 2.4777491 | 0.1306295 | 1.7893179 |
| -1.093041 | -0.955866 | -0.435221 | -0.717942 | -0.932144 | -1.289656 | -0.256471 | -0.622398 | -0.753597 |
| -0.026455 | -0.540068 | -0.805979 | 0.3010303 | -0.530678 | 0.0146725 | -0.136964 | -0.072865 | -0.060363 |
| 0.2977006 | 0.4448239 | -1.689229 | 0.1499254 | -1.281205 | 0.0803366 | -0.097841 | 0.1834929 | 0.4017024 |
| 0.7051404 | 0.6037479 | 0.0486933 | 1.2210129 | 0.8826897 | 1.1607583 | 1.0914327 | 0.1960532 | 0.4486916 |
| -0.709270 | -1.357373 | -0.586047 | -0.840832 | -0.689409 | -0.549504 | -0.928226 | -0.496699 | -1.281296 |
| 1.3641946 | -0.054613 | -1.434087 | -0.630168 | -1.345639 | -1.319668 | -1.518405 | 0.7574177 | 1.5658736 |
| 0.1331416 | -0.221027 | -1.419101 | -0.427020 | -1.805290 | -0.018597 | -0.673965 | -0.008255 | 0.5987636 |
| -0.235123 | -0.412336 | -1.200916 | -0.525872 | -1.430560 | -0.747087 | -0.873662 | 0.0321315 | 0.3201307 |
| -0.231077 | -0.997936 | -1.356273 | -0.941691 | -1.652470 | -1.039143 | -1.728682 | -0.365068 | -0.344820 |
| -1.634549 | -1.797117 | -0.268195 | -0.361891 | -0.193766 | -0.273716 | 0.7057756 | -0.306285 | -0.675131 |
| 0.1907992 | -0.332018 | -0.606766 | 0.6495572 | -0.809804 | -0.083834 | 0.2840376 | -0.456211 | 0.2161522 |
| 0.6579020 | 0.1729677 | -1.614272 | -0.539791 | -2.103391 | -0.673896 | -1.069832 | -0.058615 | 0.5937298 |
| 0.1280707 | 0.2105551 | -1.606359 | 0.1394791 | -1.369081 | -0.373993 | -0.121123 | 0.0533435 | 0.1480657 |
| -0.508291 | -0.330643 | -0.827329 | -1.191522 | -0.560291 | -0.660569 | -0.986727 | -0.088283 | -0.498357 |
| -0.317296 | -0.788758 | -1.372693 | -0.403625 | -1.207261 | -0.706327 | -0.663979 | 0.0234717 | -0.088447 |
| -0.987901 | -1.312222 | -0.558465 | -0.661697 | -0.103351 | -0.660933 | -1.180451 | -0.367522 | -0.876119 |
| -0.779228 | -1.021379 | -1.405990 | -1.032107 | -1.224511 | -1.205210 | -1.096398 | -0.472147 | -0.485365 |
| -0.443543 | -1.370887 | 0.4412535 | -1.946166 | 0.3110656 | -0.388165 | -2.390387 | -0.168602 | -1.103862 |
| -0.410260 | -0.971615 | -2.483761 | -0.913018 | -2.335945 | -0.855414 | -1.492295 | -0.121691 | -0.078919 |

| | | | | | | | | |
|---|---|---|---|---|---|---|---|---|
| 0.6043671 | 0.0214350 | -2.5690169 | -1.9161880 | -2.9956401 | -1.9494761 | -2.6598404 | 0.3507318 | 0.8612810 |
| -0.7899784 | -2.3063187 | -0.0164274 | -1.2013269 | -0.0318933 | -0.5055512 | -1.5429596 | 0.1322761 | 0.1195969 |
| -0.1242454 | -1.1167823 | -2.6042239 | -0.8753718 | -1.9005146 | -0.6264344 | -1.6881552 | -0.7121537 | -0.0871741 |
| -1.2131012 | -2.2125830 | 0.6525062 | -1.2308316 | -0.3186792 | -1.3165060 | -2.3033127 | -0.8779948 | -1.0591851 |
| -1.5404252 | -1.9024834 | -1.0113445 | -1.9355660 | -1.9223268 | -1.9196678 | -2.0015237 | -0.3136602 | -0.3044926 |
| -0.3664129 | -0.7397979 | -0.2263165 | -0.5278954 | 0.0067640 | -0.3356457 | -0.9746708 | -0.2363523 | 0.0579712 |
| 0.0928922 | -0.4948876 | 0.2044696 | 1.2566583 | 0.5515394 | 1.9394500 | 0.5471752 | -0.0964450 | 1.5766073 |
| -0.3366965 | -0.1264423 | 0.5702041 | 1.1179908 | 1.3859567 | -0.2717047 | -0.2625319 | -0.0241137 | -0.3014983 |
| -0.1103082 | -0.8199900 | -3.8962480 | -1.3289674 | -3.4855982 | -1.8740305 | -2.4757946 | 0.0029991 | -0.2801652 |
| -0.4121055 | -0.8559690 | -1.2978132 | -0.8777302 | -0.9847651 | -0.8184802 | -1.2696383 | 0.1358476 | -0.2701733 |
| -0.9493729 | 0.2576862 | -1.1453760 | -0.0330855 | -0.6179372 | -0.4227330 | 0.3322888 | -0.3800679 | 0.3038333 |
| -1.2753366 | -1.5586319 | -1.4826183 | -0.5425584 | -1.3322435 | -1.2481439 | -0.8987958 | -0.6737322 | -0.3133336 |
| -0.6643949 | -0.2392782 | 0.0620049 | -0.1465311 | 0.0648899 | -0.1981464 | 0.7621749 | -0.2793162 | -0.2238077 |
| 0.0983521 | -0.9288482 | -0.9904583 | -1.3332562 | -0.9806641 | -1.1598468 | -2.2173927 | 0.1592558 | -0.1786691 |
| -0.8179213 | -2.2734885 | -0.8323111 | -2.2241485 | -0.7865916 | -1.2505486 | -3.1715984 | -0.3516247 | -0.6569217 |
| -0.8066637 | -0.9845346 | -0.9120873 | -0.2499436 | -0.3743837 | -0.1391785 | -0.5715261 | -0.2580237 | 0.0409715 |
| -0.2086357 | -1.6102886 | -2.7719696 | -3.1841350 | -3.9025867 | -1.7635047 | -4.6814303 | 0.0156164 | -0.2882885 |
| -0.6704719 | -1.1667281 | -0.9712250 | -1.0291325 | -1.0178992 | -1.5836950 | -1.6376844 | -0.0468413 | -0.5349178 |
| -1.1038777 | -1.9603596 | -0.2056011 | -0.9980785 | -0.4208429 | -1.1335480 | -1.6359148 | 0.0687209 | -1.0479489 |
| 0.7773992 | -0.4151022 | -0.8614706 | -0.8990605 | -1.6376036 | -1.3102554 | -0.8431625 | 0.4025369 | 0.4577505 |
| -0.6112936 | -1.6196286 | -1.2033239 | -1.2240455 | -1.4437879 | -1.1527436 | -1.4187330 | -0.3318182 | -0.9207930 |
| -0.2823386 | -0.3912036 | 1.1571347 | -1.8321038 | 0.4850303 | -0.0363274 | -1.9742435 | -0.2700998 | -0.3509730 |
| -1.7065638 | -3.2019361 | -0.2467083 | -1.7710364 | 0.1041832 | -0.6035671 | -2.9153134 | -1.1314604 | -2.0685029 |
| -0.1147595 | 0.0324325 | -0.4961346 | -0.8929143 | -0.7597575 | -0.5921814 | -0.8421403 | -0.1146839 | -0.1191097 |
| -0.0635352 | -1.0155058 | -0.1569096 | -0.5389622 | -0.2003166 | -0.3134623 | -0.2476279 | -0.3209256 | -0.1008028 |
| -0.6453044 | -0.9656836 | -0.7511843 | -0.5530838 | -0.6123772 | -0.4020918 | -1.1270573 | -0.4916997 | -0.3025561 |
| -0.3117954 | -0.8147351 | -0.8063589 | -1.2094111 | -0.6880778 | -0.6027517 | -1.6497232 | -0.2163827 | -0.4898707 |
| -0.8010617 | -0.9787232 | -0.2531075 | -1.1098656 | -0.9295111 | -0.7787471 | -1.5553250 | -0.5426855 | -0.7970470 |
| -0.0899962 | 0.2798993 | -0.8674026 | 0.1068406 | -1.0334767 | -0.2392900 | -0.2600971 | -0.2532256 | 0.0234556 |
| 0.1798846 | 0.6188796 | -1.4149019 | 0.2412317 | -1.7319916 | -0.5449025 | -0.2104036 | 0.0847207 | 0.4596169 |
| -0.4964315 | -1.6099567 | -0.0895976 | -0.7560479 | -0.2208736 | -0.6561667 | -1.0238104 | -0.7559699 | -0.5164881 |
| -0.1901507 | -0.1458953 | -1.1791968 | -1.0902176 | -1.6721419 | -0.9683155 | -1.8594697 | -0.2089378 | -0.3781219 |
| -0.8044609 | 0.1107294 | -0.4235802 | 0.5278267 | -0.3068941 | -0.2496357 | -0.4359970 | -0.7514733 | -0.1595451 |
| -0.6343432 | -1.3958014 | -0.6549610 | -1.5720352 | -0.6258788 | -0.7368525 | -2.0625351 | -0.0766864 | -0.3852407 |
| -0.2386470 | -0.7955166 | -0.9234392 | 0.1435718 | -0.8399757 | -0.2898538 | -0.1980622 | -0.0245729 | -0.0198411 |
| -0.7955694 | -1.6277458 | -1.5203583 | -0.9323144 | -1.1518832 | -0.7350854 | -1.0719232 | -0.4526700 | -1.0483821 |
| -1.8085361 | -2.5779497 | -0.8944643 | -1.5908601 | -0.9757432 | -1.9018313 | -2.1061129 | -0.9383132 | -2.2897775 |
| -0.2837171 | -0.2699578 | -0.0007816 | -0.1516641 | -0.1952105 | -0.1602848 | -0.2239371 | 0.0722334 | -0.1283443 |
| -0.0781723 | -0.4292693 | 0.1062108 | 0.9541298 | 0.2196339 | 0.5935072 | 0.0154430 | -0.0728111 | 0.4650121 |
| -0.5213372 | -1.4412462 | 0.1842172 | -1.3400331 | -0.2302027 | -0.6830929 | -3.0338864 | -0.2167187 | -0.5714308 |
| 0.3179743 | 0.3039983 | -2.8116675 | -1.1532474 | -3.1255570 | -1.0460124 | -1.4152603 | 0.1735158 | 0.1797801 |
| -1.0608088 | -1.2774889 | -2.2788251 | -1.2574517 | -1.6165141 | -1.1932894 | -1.8559007 | -0.4111505 | -0.8549878 |
| -0.8305817 | -1.2541310 | -0.8669727 | -1.0547983 | -0.1372469 | -1.1977234 | -0.8280298 | -0.2261728 | -0.9020429 |
| -0.9156101 | -0.5115326 | -0.3245185 | -0.3626535 | -0.4263890 | -0.8026200 | -0.7951639 | -0.2598730 | -0.6540420 |
| -0.1865841 | -0.3768555 | -2.0724746 | -0.2381646 | -1.5325885 | -0.6710131 | -0.7341887 | -0.4893333 | -0.4814027 |
| -1.0471129 | -0.9514497 | -1.8087091 | -1.0135300 | -2.2523056 | -1.1597798 | -2.4749967 | -0.3634554 | 0.0105351 |
| -0.5179230 | -0.5939299 | -1.1419036 | -1.5060906 | -1.2864752 | -1.0925935 | -1.8310786 | -0.1409120 | 0.0728948 |
| -1.1926635 | -1.4629732 | -0.5751559 | -1.0770355 | -1.1025549 | -1.0300997 | -1.2899836 | -0.7562593 | -0.3921713 |
| 0.3554581 | 0.2477682 | -1.8215560 | -0.9543554 | -1.5154983 | -0.8995606 | -0.8075693 | -0.2243904 | -0.3082542 |
| -0.9332002 | -1.3092080 | -0.9187505 | -0.9903661 | -1.0013282 | -1.3767425 | -1.8345520 | -0.6552928 | -1.0807439 |

| | | | | | | | | |
|---|---|---|---|---|---|---|---|---|
| -0.9721317 | 0.0876728 | -0.5343599 | -0.5612186 | -0.9886101 | -0.5804199 | -0.2370741 | -0.1547904 | -0.1613270 |
| 0.0482455 | -0.0724953 | -1.0533529 | -0.0716919 | -0.7613662 | 0.1576242 | -0.3017306 | -0.0201863 | -0.0151288 |
| -0.3629635 | -0.3365896 | -0.9866088 | -0.1028043 | -0.7075627 | -0.1742394 | -0.0824368 | -0.1658509 | -0.2072624 |

| PG_498 | PG_499 | PG_500 | PG_501 | PG_502 | PG_503 | PG_504 | PG_505 | PG_506 |
|---|---|---|---|---|---|---|---|---|
| -2.0662495 | -0.8337303 | -1.4152517 | -1.7239309 | -0.3310430 | -0.5163351 | -2.2946555 | -0.2184468 | -0.6801026 |
| -2.0928118 | -0.6584199 | -1.0405791 | -1.6635836 | -0.6713351 | -0.8364778 | -1.4986857 | -0.8840439 | -1.0500739 |
| -2.5428229 | -0.3388740 | -1.4218506 | -1.5738569 | -0.2578305 | -0.6838495 | -0.9958496 | -0.2957955 | -1.3559372 |
| -0.3124137 | -0.2609994 | -0.4714350 | -0.4365289 | -0.2257992 | -0.2822257 | 0.4467220 | -0.2208320 | -0.4277019 |
| -0.5792793 | -0.1555809 | -0.1709828 | -0.9715004 | 0.0626049 | 0.2844103 | -1.1568203 | 0.0808302 | 0.1120800 |
| -1.3089730 | -0.3524510 | -0.6457023 | -1.4872838 | -0.2949547 | -0.3403549 | -2.3601733 | -0.2407706 | -0.4973024 |
| -0.1406769 | -0.1731941 | -0.6995271 | -0.2677393 | -0.3552418 | -0.6659791 | -1.1076272 | -0.0731554 | -1.1532263 |
| -0.8301082 | 0.0057818 | -0.2465850 | -0.5961530 | -0.4915806 | -0.5362716 | -1.9325641 | -0.4075813 | -0.6965617 |
| 0.0335161 | -0.3849281 | -0.1092356 | -0.0281357 | -0.4691543 | -0.4855709 | -0.1387402 | -0.4676057 | -0.4810459 |
| -1.6011583 | -0.9310334 | -1.4701155 | -1.1430477 | -0.1560569 | -0.4323854 | -1.6132170 | -0.1568979 | -0.9758464 |
| -0.6129202 | -0.1563094 | -0.5738240 | -0.7555188 | -0.0240062 | 0.0314410 | -0.0091109 | 0.0212817 | -0.0190820 |
| -0.2966273 | -0.3321054 | -0.5495415 | -0.3632395 | -0.0590343 | -0.4925733 | -0.3599696 | -0.1496453 | -0.6824609 |
| -1.8046503 | -0.9288618 | -1.1153050 | -1.7433901 | -0.7493209 | -1.3720411 | -4.7243455 | -0.6509560 | -1.8461741 |
| 3.5860794 | 2.2020117 | 4.4697532 | 3.9218420 | -0.0083163 | 1.7328980 | 4.1009062 | 0.2191940 | 3.3090572 |
| 0.0203806 | -0.1385469 | -0.0056799 | 0.1504383 | 0.2431134 | -0.4759729 | -0.2332756 | 0.5136538 | -0.5538317 |
| 2.9868359 | 0.7223323 | 3.1921283 | 2.9611575 | -0.3650463 | 2.7899520 | 3.1439787 | 0.3401747 | 3.3064526 |
| -0.9024169 | -1.0566313 | -1.0258517 | -0.7605399 | -0.7306448 | -1.7090105 | -2.0659500 | -0.9224473 | -2.5343203 |
| 0.5358925 | -0.7976638 | -0.9717165 | 1.0079563 | -0.0703635 | -0.0935262 | -0.0293808 | -0.2972641 | 0.3212162 |
| -2.6694443 | -1.1109630 | -2.4003354 | -2.7175564 | -0.3990898 | -0.3032003 | -0.4477101 | -0.3499204 | -0.4090651 |
| -0.7122496 | -0.8690561 | -0.7658867 | -0.4704081 | -1.8738625 | -2.2059423 | -0.9455916 | -2.1071592 | -2.4949045 |
| 0.5798983 | -0.2736772 | 0.5307008 | 0.1691136 | -0.7147829 | 0.8665389 | 0.9079643 | -0.1042372 | 1.1375312 |
| -0.1001059 | -0.2912394 | -0.1343555 | -0.4080895 | -0.2541854 | -0.5910958 | -0.4374486 | -0.4496854 | -0.1947245 |
| 0.8073700 | 0.2199970 | 0.5347746 | 0.8969352 | -0.5207621 | 0.2888671 | 1.3765835 | -0.3847354 | 0.2482643 |
| -2.5421490 | -0.4398048 | -1.8490596 | -2.1207738 | -0.4167468 | -0.6070071 | -1.9190546 | -0.4396909 | -1.5345794 |
| -1.8038061 | -0.5020425 | -1.5750526 | -1.7524404 | -0.2843889 | -0.9906304 | -1.9493993 | -0.5576262 | -1.3154045 |
| 0.5606824 | -0.1870659 | 0.1886529 | 0.4609970 | -0.3420090 | 0.4376329 | 1.0120666 | -0.1449860 | 0.4001206 |
| -0.2726574 | 0.1007000 | -0.0421082 | -0.2618360 | 0.0074196 | -0.0641561 | -0.5709239 | 0.1164196 | -0.0977504 |
| -1.2542426 | -0.2503503 | -0.8565374 | -1.6843375 | -0.5618182 | -1.0709366 | -1.1689595 | -0.6128012 | -1.3745664 |
| -1.6929865 | -0.5023456 | -1.3446434 | -1.7428865 | -0.8180980 | -1.5556620 | -1.9082427 | -0.6771324 | -1.8170366 |
| -0.2302317 | -0.1477303 | -0.2399993 | -0.4150862 | 0.0594043 | -0.1398131 | -0.2740596 | 0.0000524 | -0.4221335 |
| -1.1375303 | 0.5745393 | -0.0498530 | -1.1576009 | 0.1502387 | -0.0919787 | -1.0680288 | 0.0656971 | -0.4969720 |
| -1.2366338 | 0.8517639 | 0.0827463 | -0.9936520 | -0.1070242 | -0.1367302 | 0.5630775 | -0.4042207 | -0.3798922 |
| -0.6958092 | -0.1284170 | 0.4503727 | -0.5279411 | 0.4865274 | 0.2489477 | -3.1437102 | 0.3928936 | 0.0293818 |
| -2.5003594 | -0.9729758 | -1.4320819 | -2.9618779 | -0.3302072 | -0.4952338 | -4.5214954 | -0.3468745 | -0.5906362 |
| -2.4579946 | 0.1551198 | -0.5569215 | -2.3797039 | 0.8711061 | 0.7925095 | -1.5628044 | 0.7101392 | 0.4757678 |
| -0.3390247 | -0.5942228 | -1.0936984 | -0.1789768 | -0.5669532 | -0.3745175 | -0.8188633 | -0.9255002 | -0.7390684 |
| -1.5572675 | -0.4601189 | -0.6902489 | -1.9724193 | -0.0023651 | -0.7079796 | -2.3706495 | -0.1784259 | -1.4527722 |
| -0.0006604 | 3.0269881 | 2.5383384 | -0.2956073 | -0.1920936 | 3.3286868 | 2.8416176 | -0.0340414 | 4.5186126 |
| 0.6446710 | -0.2058319 | 1.0941513 | 1.0626669 | -0.5384106 | -0.3890400 | 0.1428123 | -0.4843133 | -0.1697718 |
| -0.3366142 | -1.0019218 | -0.5748977 | -0.2946599 | -0.9086399 | -1.8082684 | -0.5293494 | -1.7127995 | -1.8482199 |
| 0.8282462 | -0.4419909 | 0.2828851 | 0.9918551 | -0.0561490 | 0.3196886 | 1.0152367 | 0.1028715 | 0.6679351 |
| -1.5805265 | -0.6793059 | -1.4126441 | -1.8796570 | -0.5277676 | -0.5557433 | -2.1097486 | -0.4335786 | -0.8155316 |
| -1.2067465 | -0.6534682 | -0.7048847 | -0.7396709 | -0.1608981 | -0.6824778 | -1.5540641 | -0.4852530 | -0.9309756 |
| -0.2907813 | -0.0262685 | 0.1147203 | -0.4352664 | 0.2773688 | 0.0547468 | -0.4355291 | 0.0783910 | -0.0121301 |
| 1.0946524 | 0.2285931 | 0.3333297 | 0.9722506 | -0.0653118 | 0.4064970 | 1.1272754 | 0.0500332 | 0.5869820 |
| 2.8028697 | -0.2260780 | 1.6531523 | 3.8684181 | 0.0626778 | 0.2230349 | 0.5311573 | 0.1357929 | 0.6138712 |
| -0.7108364 | -0.5485146 | -0.4824522 | -0.6337050 | -0.1959471 | -0.2386635 | -0.2420081 | -0.4569392 | -0.1899141 |

| | | | | | | | | |
|---|---|---|---|---|---|---|---|---|
| 0.2003162 | -0.198898 | 0.3086352 | -0.174803 | -0.323675 | -0.191650 | 0.5756737 | -0.201332 | -0.019133 |
| -2.101451 | -0.792287 | -0.955955 | -1.926013 | 0.0063043 | -0.052848 | -2.373257 | 0.0038856 | 0.0379416 |
| 0.4088372 | -0.504628 | 0.1191846 | 0.2796109 | -0.327507 | -0.090217 | 0.1512702 | -0.320803 | -0.055173 |
| -0.540539 | -0.771913 | -0.039393 | -1.307039 | -0.219480 | 0.0676728 | -0.609718 | -0.526376 | -0.466341 |
| -0.428732 | -0.621806 | -0.394400 | -0.354085 | -0.320716 | -0.172304 | -0.114360 | -0.321334 | -0.108933 |
| 0.2527140 | -0.440679 | -0.005646 | 0.7186917 | -0.495752 | -0.437301 | -0.004014 | -0.528690 | -0.789811 |
| -1.633966 | -0.584520 | -1.465930 | -1.745535 | -0.144110 | -0.962903 | -3.546123 | -0.069021 | -1.319118 |
| 1.9747513 | 2.0103099 | 2.4191748 | 1.9431163 | -0.575203 | 1.9074363 | 3.2229626 | -0.255771 | 2.5337914 |
| 0.4356808 | 3.2392955 | 3.5786931 | 0.8573597 | -1.191732 | 3.2157222 | 2.0916665 | -0.889146 | 4.4805177 |
| -0.331839 | -0.981925 | -0.116806 | -0.456863 | -0.621981 | -0.170953 | 0.0127850 | -0.765088 | -0.597652 |
| -1.040071 | -0.821910 | -1.001129 | -1.669905 | -0.258489 | -1.230363 | -1.812911 | -0.557517 | -1.309215 |
| 3.4033169 | 1.3917285 | 3.9341473 | 3.7179102 | 0.0421226 | 2.9757017 | 2.8659456 | 1.6314518 | 3.1675263 |
| 2.6865666 | 0.9910179 | 3.2757975 | 2.7228694 | -0.560679 | 2.0125309 | 1.5624203 | -0.194063 | 2.1690855 |
| -0.990198 | -0.972122 | -0.906918 | -0.837554 | -0.785776 | -0.499966 | -2.462843 | -1.006445 | -0.389620 |
| -2.244446 | -0.953849 | -1.682553 | -2.904102 | 0.0151762 | -0.731944 | -1.589926 | 0.0110710 | -1.055245 |
| -0.172961 | -0.620870 | -0.737505 | 0.3926024 | -0.347536 | -0.873675 | -1.401390 | -0.585229 | -0.987785 |
| -2.297293 | -0.634143 | -1.636547 | -2.287118 | -0.111758 | -0.492289 | -2.066035 | -0.159927 | -0.930353 |
| -0.834938 | -0.319881 | -0.990938 | -0.971756 | -0.409529 | -0.325569 | -0.915173 | -0.345118 | -0.619732 |
| -1.499925 | -0.543816 | -0.801727 | -1.461146 | -0.196297 | 0.1218265 | -1.262615 | -0.057037 | -0.255410 |
| -0.195925 | -0.065893 | 0.4396892 | 0.0791323 | 0.2259705 | -0.066394 | -0.340523 | 0.1448145 | -0.181033 |
| -0.316734 | -0.564162 | -0.825656 | -0.480036 | 0.0460649 | -0.222648 | 0.0522355 | -0.061745 | 0.0618969 |
| -1.896981 | -0.870062 | -1.607563 | -2.099776 | -0.402292 | -0.841540 | -3.611874 | -0.445590 | -1.046586 |
| 0.0127648 | -0.018844 | 0.0694713 | -0.354386 | -0.609144 | -0.030997 | 0.6211116 | -0.477788 | -0.312996 |
| 0.6389213 | 0.6144205 | 0.6268504 | 0.4987344 | 0.5076540 | 0.8615705 | 1.3037471 | 0.8065577 | 0.7587142 |
| -1.017520 | -0.609097 | -0.858670 | -1.776150 | -0.857531 | -1.174093 | -0.486240 | -0.712967 | -1.433703 |
| -0.023964 | -0.066216 | -0.573960 | -0.043050 | -0.200777 | -0.310473 | -0.523942 | -0.023642 | -0.476137 |
| -3.510495 | -1.761017 | -2.889379 | -3.859520 | -0.800565 | -1.766734 | -1.875639 | -0.841098 | -2.050907 |
| 4.3168727 | 0.9907759 | 2.4198019 | 4.1811452 | -0.555026 | 1.5408057 | 4.6769859 | -0.002265 | 1.6056727 |
| 2.9353545 | 1.0073616 | 2.0344441 | 2.8768632 | -0.463598 | -0.211174 | 2.5506747 | -0.683567 | 0.3496581 |
| -1.683534 | -0.218797 | -0.869602 | -1.926813 | -0.600792 | -0.857814 | -1.166763 | -0.482737 | -1.204744 |
| -0.162185 | -0.301384 | -0.596376 | -0.256907 | -0.291554 | -0.308643 | -0.335408 | -0.825404 | -0.573709 |
| -0.547082 | -0.239238 | -0.639600 | -0.555616 | 0.1995190 | 0.0862184 | -0.307674 | 0.2242086 | -0.055637 |
| -1.081835 | -0.930385 | -1.457021 | -0.987719 | -0.485581 | -0.783639 | -1.732192 | -0.478974 | -1.074767 |
| -1.381168 | -0.490736 | -1.176755 | -1.585048 | -0.240696 | -0.589123 | -1.139446 | -0.346280 | -0.829696 |
| 1.5228966 | 0.0338699 | 1.0227723 | 1.3874364 | -0.334603 | 0.7862976 | 1.1476974 | -0.120357 | 0.8644402 |
| -1.265876 | -0.293572 | -1.028590 | -0.845027 | 0.1412971 | 0.0736585 | -0.501309 | 0.0718779 | -0.133540 |
| -0.767883 | -0.657652 | -0.452455 | -0.415478 | 0.1884915 | -0.210257 | -2.112772 | -0.116320 | -0.122816 |
| -1.125469 | -0.402459 | -0.916470 | -0.985124 | -0.065075 | -0.501445 | -2.452044 | -0.244904 | -0.785576 |
| -1.797785 | -0.125775 | -0.690425 | -1.793513 | -0.427764 | -0.432033 | -0.351143 | -0.178911 | -0.840731 |
| -2.615857 | -0.876909 | -1.583200 | -2.837407 | -0.571303 | -0.821514 | -2.395209 | -0.753454 | -0.895658 |
| -2.447609 | -1.256466 | -2.068792 | -2.838685 | -0.779023 | -1.729259 | -2.597585 | -1.310220 | -2.025238 |
| -1.855449 | -0.710962 | -1.900715 | -1.597344 | -0.175703 | -0.489008 | -2.035071 | -0.067534 | -1.411192 |
| 0.6486869 | 0.5732847 | 2.9062720 | 1.0732531 | -0.244325 | 2.1618063 | 3.6925714 | 0.7605847 | 3.1320098 |
| -0.328543 | -0.233199 | -0.239648 | -0.213453 | -0.128122 | -0.103813 | -0.180823 | -0.247744 | -0.147507 |
| -0.260162 | 0.0042893 | 0.0766488 | -0.184911 | 0.0542459 | 0.0014343 | -1.188283 | 0.1010009 | -0.034914 |
| -0.529525 | -0.954708 | -0.721413 | -0.633544 | -0.836034 | -0.806606 | -0.999035 | -0.949100 | -0.941659 |
| 7.5997965 | 0.0240820 | 7.2877304 | 8.4736145 | -0.449618 | 6.2524645 | 6.9790267 | 0.9265921 | 6.8195549 |
| 2.2868187 | 0.0149400 | 2.3306805 | 2.5110502 | -0.127802 | 1.8183094 | 1.7092814 | 0.0967640 | 2.2945667 |
| -1.386080 | -0.429265 | -0.725615 | -0.548681 | -0.282335 | -0.343764 | 0.6568622 | -0.251484 | -0.418680 |
| -1.219417 | -0.444021 | -0.139924 | -1.410585 | -0.302767 | -0.252978 | 0.4462162 | -0.288287 | 0.0323037 |

| | | | | | | | | |
|---|---|---|---|---|---|---|---|---|
| -2.088083 | -0.145626 | -0.791990 | -1.821004 | 0.0772159 | -0.648610 | -2.715146 | -0.241117 | -1.102292 |
| 0.0853807 | -0.349970 | -0.325543 | 0.0458237 | -0.477390 | -0.360296 | 0.2854378 | -0.295317 | -0.485509 |
| -0.340504 | -0.066597 | -0.901801 | -0.061734 | 0.0036920 | -0.440524 | 0.2768118 | -0.139641 | -0.906196 |
| 0.0167923 | -0.037784 | 0.2067499 | 0.0353273 | 0.2089707 | 0.1691108 | -0.067586 | 0.1563409 | 0.1470170 |
| -1.400184 | -0.854859 | -1.902593 | -1.792385 | -0.430785 | -0.758082 | -1.601479 | -0.465133 | -1.116184 |
| -1.363165 | -0.472537 | -0.496699 | -0.830331 | -0.224759 | -0.033198 | -0.222703 | 0.1528674 | 0.0596796 |
| -0.596643 | -0.456974 | -0.286908 | -0.452473 | -0.107798 | -0.511599 | 0.0143358 | -0.384647 | -0.315292 |
| 0.0368378 | -0.379598 | -0.243101 | -0.070292 | -0.060772 | 0.0267347 | -0.001212 | -0.135075 | 0.3850234 |
| -0.129016 | -0.045192 | -0.424527 | -0.354265 | -0.474348 | -0.283548 | 0.1275995 | -0.286888 | 0.2494606 |
| 1.0141094 | 0.2979579 | 0.8951412 | 0.9302495 | -0.131387 | 0.5944640 | 0.9209233 | 0.1996551 | 0.6866034 |
| -0.572599 | -0.067625 | -0.302564 | -0.258100 | 0.0062530 | 0.2651084 | -0.455851 | 0.0512988 | 0.3465953 |
| -1.294308 | -0.245514 | -0.139987 | -1.013630 | -0.461961 | -1.189295 | -3.218606 | -0.218824 | -0.639446 |
| -3.479752 | -0.907324 | -1.497161 | -2.995464 | -0.638720 | -1.149091 | -5.147424 | -0.558687 | -1.829632 |
| -1.094535 | -0.568680 | -0.454413 | -1.023778 | -0.279768 | -1.309753 | -2.841041 | -0.402078 | -1.483214 |
| -1.026775 | -0.310796 | -0.619363 | -1.048792 | -0.074867 | -0.371532 | -0.079322 | -0.034044 | -0.729846 |
| 0.0982423 | -0.055794 | -0.099691 | -0.250475 | -0.125729 | -0.050585 | 0.0090132 | -0.166841 | -0.079193 |
| -0.659233 | 0.0287850 | -0.400714 | -0.461861 | -1.068164 | -1.214519 | -0.698377 | -0.967633 | -1.553482 |
| -0.097436 | -0.409087 | -0.014097 | 0.3103213 | -0.261699 | -0.106994 | -0.289307 | -0.306044 | -0.363675 |
| -0.708671 | -0.601615 | -0.419339 | -1.173585 | -0.207770 | -0.487660 | -0.709826 | -0.367734 | -0.748639 |
| 1.1408235 | 0.2808283 | 0.6846380 | 1.5050687 | -0.342156 | 0.2025779 | 1.7861542 | -0.476269 | 0.3053251 |
| -1.270489 | -0.462698 | -0.989464 | -1.196605 | -0.623534 | -0.810982 | -0.871016 | -0.487270 | -1.019571 |
| -0.572281 | -0.066080 | -0.450319 | -0.584238 | -0.404749 | -0.641113 | 0.4501838 | -0.331642 | -0.683976 |
| -0.865452 | -0.419334 | -0.505852 | -0.570373 | -0.147212 | -0.374660 | -0.569190 | -0.257853 | -0.282418 |
| -1.754936 | -0.275665 | -0.635085 | -2.255871 | -0.452547 | -0.649064 | -1.077094 | -0.494693 | -0.387210 |
| -2.529669 | -0.435828 | -1.259579 | -2.801813 | -0.598687 | -0.555646 | -0.588161 | -0.374054 | -0.408267 |
| -0.582285 | -0.188523 | -0.666607 | -0.662117 | -0.104016 | -0.330357 | -0.237079 | -0.150270 | -0.372602 |
| 0.3349883 | -0.034575 | -0.160364 | 0.1901293 | 0.1102707 | 0.2846367 | 0.5592611 | 0.1320317 | 0.3208060 |
| 0.1130326 | -0.571496 | -0.117321 | -0.060958 | -0.582823 | -0.151164 | 0.2719756 | -0.645484 | -0.469077 |
| -1.747595 | -0.463881 | -1.410221 | -2.020948 | -0.222830 | -0.553967 | -0.964450 | -0.254182 | -0.979691 |
| -0.641899 | -0.504591 | -0.360308 | -0.988554 | -0.081426 | -0.139907 | -4.054029 | 0.0599899 | -0.695125 |
| -2.226563 | -1.040042 | -1.485025 | -2.336462 | -0.092981 | -0.367329 | -0.371471 | -0.705542 | -0.691419 |
| -0.696688 | -0.365901 | -0.762029 | -0.923616 | -0.323982 | -0.502504 | -1.283043 | -0.374405 | -0.695289 |
| -0.636318 | -0.648850 | -0.472746 | -0.691528 | -0.637373 | -1.744526 | -1.710598 | -0.824492 | -1.541975 |
| 0.0662059 | -0.000295 | 0.3210484 | 0.0493895 | 0.4232454 | 0.4397266 | 0.2940730 | 0.2321830 | 0.3994535 |
| -1.748209 | -0.491652 | -0.368131 | -1.819536 | -0.291575 | -0.159363 | -1.095445 | -0.407253 | -0.384281 |
| -0.799804 | -0.196265 | -1.072432 | -1.090062 | -0.372092 | -0.354026 | -0.770990 | -0.541689 | -0.739063 |
| -0.238320 | -0.166972 | -0.420926 | -0.223275 | -0.152713 | -0.373667 | -0.182072 | -0.098756 | -0.434969 |
| -0.727770 | -0.070100 | -0.204147 | -0.703962 | -0.104349 | -0.045507 | -1.324381 | 0.0634110 | -0.305565 |
| -0.436816 | -0.488048 | -0.321636 | -0.418504 | -0.352923 | -0.486520 | -0.184979 | -0.527553 | -0.552402 |
| -0.091261 | -0.409712 | -0.280790 | 0.3172085 | -0.493265 | -0.841969 | -4.043722 | -0.273087 | -1.248096 |
| 0.9131255 | 0.3195583 | 0.5577408 | 0.7164879 | -0.171502 | -0.777563 | -0.592460 | -0.526713 | -0.943793 |
| -0.499897 | -0.096670 | 0.1292730 | -0.798524 | -0.115130 | 0.0896270 | -2.162943 | 0.0594406 | 0.0952439 |
| -0.645413 | 0.0305561 | -0.136051 | -0.645814 | 0.0553881 | 0.2415629 | -1.192530 | 0.1479009 | 0.0210386 |
| -0.790811 | -0.146925 | 0.0006560 | -0.531512 | -0.874959 | -0.917119 | -0.218631 | -0.399222 | -0.478994 |
| -0.334031 | -0.372224 | -0.199630 | -0.326803 | -0.221232 | -0.004873 | -0.211517 | -0.140094 | -0.048904 |
| 0.2914365 | 0.3084938 | 0.5824330 | 0.1743604 | -0.295782 | -0.238085 | 0.1085440 | -0.106741 | -0.064337 |
| -1.360977 | -0.613293 | -0.557793 | -1.060375 | -0.580897 | -1.161546 | -1.249491 | -0.522046 | -1.220984 |
| 0.5703892 | 0.1709064 | 0.6143176 | 0.4163761 | -0.318678 | 0.1385692 | 0.9960975 | -0.290039 | 0.2928172 |
| -2.207529 | -0.305892 | -1.344070 | -2.048669 | -1.033106 | -1.477994 | -3.418779 | -0.832479 | -2.373845 |
| -1.286672 | -0.861839 | -0.751993 | -1.593403 | -0.587164 | -0.879628 | -1.414371 | -0.901489 | -1.053693 |

| | | | | | | | | |
|---|---|---|---|---|---|---|---|---|
| 1.1978546 | 0.3936715 | 0.7092233 | 1.1332127 | 0.0444307 | 0.3595179 | -0.0561510 | 0.1304456 | 0.2091622 |
| -2.1246662 | -0.6203658 | -1.5002413 | -1.6701347 | -0.3896588 | -1.0393932 | -2.0739167 | -0.2945850 | -1.0826086 |
| -0.5001906 | -0.6262836 | -0.5490095 | -0.5829071 | -0.4525804 | -0.8898405 | -1.2548142 | -0.4727508 | -0.6893399 |
| -0.4113741 | -0.5784463 | -0.4165211 | -0.3490984 | -1.0857216 | -0.1971150 | 0.8611176 | -0.9361690 | 1.0697934 |
| -1.7386661 | -0.4445620 | -0.9619630 | -1.5877895 | -0.3873216 | -0.3714939 | -1.3100047 | -0.1412875 | -1.1401598 |
| 1.1102945 | 0.7223253 | 1.7159782 | 0.7320555 | -0.1619666 | 1.0432259 | 1.1862260 | 0.0549077 | 1.0328797 |
| -0.6903739 | -0.5045171 | -0.6525184 | -0.7117193 | -0.2781385 | -0.3841190 | -0.3313774 | -0.2435902 | -0.3224292 |
| -0.7434607 | -0.5212790 | -1.0267847 | -0.8227475 | -0.7930426 | -0.9567895 | -1.1128973 | -0.9596145 | -1.4103456 |
| -1.8990043 | -0.7505773 | -1.0685211 | -1.6288710 | -0.0387109 | -0.2069086 | -1.3053627 | 0.1025327 | -0.1832625 |
| -0.2388721 | -0.3150714 | -0.4222808 | -0.1198742 | -0.7966722 | -0.4171508 | 0.2460629 | -0.7941435 | -0.3205741 |
| 0.4585810 | -0.0818723 | 0.0864848 | 0.3684113 | -0.1467004 | 0.0477040 | -0.2196242 | -0.0259639 | 0.1397060 |
| -2.7654079 | -0.8299073 | -2.4341664 | -2.7527651 | 0.3424460 | 0.3773929 | -2.0917350 | 0.4138691 | -0.1112443 |
| -0.3644162 | -0.0832505 | -0.7223204 | -0.1521334 | 0.5140966 | -0.0138396 | -0.9433023 | 0.5596863 | -0.4668732 |
| 0.4738224 | 0.0557078 | 0.1670725 | 0.8457896 | -0.1286428 | -0.0553964 | -0.2105574 | -0.1192733 | 0.0525846 |
| -0.1713678 | -0.2333218 | -0.2827676 | -0.2035834 | -0.2912765 | -0.2654520 | -0.0744731 | -0.5253299 | -0.3941234 |
| -0.8738380 | 0.0599723 | -0.1635358 | -0.4571148 | 0.3253236 | 0.0831331 | -1.9133880 | 0.2785241 | -0.1212948 |
| -0.9023889 | -0.8362709 | -0.6899140 | -1.1555203 | -0.4943017 | -0.5617094 | -0.5706482 | -0.4099442 | -0.2352349 |
| -1.3186448 | -0.7608729 | -2.1820024 | -1.9924121 | -0.4343557 | -0.9875291 | -0.7417933 | -0.8250208 | -1.3328339 |
| 0.0566752 | -0.6185355 | -0.6371431 | -0.5360950 | -0.5783280 | -1.0945521 | -0.9068775 | -0.6748841 | -0.8099434 |
| -0.9990395 | -0.3163632 | -1.0609491 | -0.9870564 | -0.4414767 | -0.7365285 | -1.3144756 | -0.4349596 | -0.9831043 |
| 1.0969452 | -0.3216567 | 1.2196864 | 1.2752231 | -0.4493826 | 0.0806867 | 0.5065758 | -0.3150238 | 0.4083506 |
| 0.1343164 | 0.0328685 | 0.3897349 | -0.1428446 | 0.1968445 | 0.3597762 | 0.5714682 | 0.3736690 | 0.4521302 |
| -1.6072122 | -0.7042248 | -1.9325065 | -1.6665242 | -0.3082753 | -1.3357520 | -2.5622836 | -0.4044132 | -1.5647877 |
| -0.5115759 | -0.4416027 | -0.4093288 | -1.0489333 | -0.5494131 | -0.3925357 | -0.1500635 | -0.5909849 | -0.5867830 |
| -0.8490510 | -0.3695324 | -0.6365564 | -0.9680382 | -0.4794138 | -1.0245539 | -0.9175832 | -0.5828019 | -1.0951440 |
| -1.5392588 | -0.5032879 | -1.3334304 | -1.6820765 | -0.4722653 | -0.5223887 | -0.1765857 | -0.6412433 | -1.0482839 |
| -1.4555141 | -0.4104626 | -0.7146553 | -1.5132183 | -0.5917423 | -0.8509928 | -0.8344588 | -0.7043545 | -1.1004905 |
| -0.2301916 | -0.1318449 | -0.2327583 | -0.2909909 | -0.2790353 | -0.4645563 | -0.6434096 | -0.4821052 | -0.6481786 |
| -0.7442188 | -0.4907256 | -0.7653109 | -0.7908433 | -0.3062392 | -0.7331369 | -0.8841847 | -0.5251462 | -1.1340401 |
| 0.7948743 | -0.0324218 | 0.6106223 | 0.9744504 | -0.1071887 | 0.5040416 | 1.4473288 | -0.0077819 | 0.6074900 |
| 3.3685666 | 0.9192819 | 2.5883216 | 3.6959003 | -0.3036705 | 0.6318701 | 2.5641048 | -0.0422221 | 1.4813783 |
| -1.2033656 | -0.3861129 | -0.9182244 | -0.7784395 | -0.5080940 | -0.8574197 | -0.3051983 | -0.6065140 | -1.1880009 |
| 0.3334310 | -0.0923122 | 0.0326254 | 0.3062056 | 0.0774753 | 0.2247121 | 0.2789449 | 0.1030779 | 0.1796988 |
| 0.6025385 | 0.1581951 | 0.3699894 | 0.3132275 | 0.1922783 | 0.4504607 | 0.7884970 | 0.2210604 | 0.4305584 |
| 0.6582596 | 0.1580013 | 0.5331545 | 0.6045100 | 0.2157781 | 0.6605681 | 0.8137278 | 0.1609874 | 0.6246922 |
| -0.5518402 | -0.4151575 | -1.3420405 | -0.5992186 | -0.5367944 | -1.1107789 | -1.3594625 | -0.5094849 | -1.4434644 |
| 2.0592878 | 1.3284398 | 1.7705470 | 2.1763473 | 0.0662286 | 0.7818369 | 1.5043605 | 0.3717882 | 1.2113933 |
| 1.2352226 | 0.1081212 | 0.8597136 | 0.9110825 | 0.0558668 | 0.3132156 | 0.5655537 | 0.1175416 | 0.6066379 |
| 0.0915846 | 0.1483192 | 0.3341744 | 0.0190625 | -0.5211892 | -0.4313590 | 0.1348118 | -0.5038415 | -0.3120281 |
| -1.0537149 | -0.3781765 | -0.4250134 | -1.1440204 | 0.1739599 | -0.1942062 | -0.9954272 | 0.1733501 | -0.2558240 |
| -0.6086254 | -0.5463359 | -0.8268886 | -0.5286979 | -1.2466920 | -1.5790697 | -0.9677403 | -1.1490569 | -1.2796840 |
| 0.4076450 | -0.3092301 | 0.0405365 | 0.2847686 | -0.0114607 | 0.0680532 | 0.6082133 | -0.2518214 | 0.3802150 |
| 1.1451268 | 0.1691640 | 0.8673263 | 1.4585206 | -0.0237440 | 0.9329530 | 1.7584104 | 0.3389109 | 0.8732001 |
| 0.4027303 | 0.1723434 | 0.3397921 | 0.2120425 | -0.0986445 | -0.0700061 | 0.0972423 | -0.0795478 | -0.0670771 |
| -0.3703549 | -0.1876687 | -0.6252253 | -0.4316279 | -0.1604345 | -0.2182025 | -0.7210914 | -0.0877006 | -0.5204485 |
| -0.1596074 | -0.1530242 | -0.2663660 | 0.0885827 | -0.0644363 | -0.0719649 | -0.2461765 | 0.0520177 | 0.1603545 |
| -2.0036376 | -0.6400365 | -1.2568555 | -1.7893602 | -0.4965985 | -0.6362734 | -1.1981112 | -0.4776292 | -0.9189228 |
| -0.9457503 | -0.7557811 | -0.8673070 | -1.1641148 | -0.3624810 | -0.6595063 | -1.9749998 | -0.3876228 | -0.6553394 |
| -2.3863371 | -0.4995586 | -2.0665234 | -2.2368934 | 0.3329128 | -0.0287052 | -1.0708139 | 0.1992708 | -0.4212408 |
| -0.0893069 | -0.2155393 | -0.1031496 | -0.3897462 | -0.0661443 | 0.0496122 | 0.3177610 | 0.0109475 | -0.0067878 |

| | | | | | | | | |
|---|---|---|---|---|---|---|---|---|
| 1.3584380 | 0.2255319 | 1.3952343 | 0.7622365 | 0.4515456 | 0.4331107 | 0.7985723 | 0.7038132 | 0.4959962 |
| 0.4404125 | 0.1660191 | 0.2687483 | 0.4596441 | -0.2253225 | -0.1748415 | -0.0613705 | -0.1896938 | 0.0049850 |
| -0.9543439 | -0.3129971 | 0.1322129 | -0.7709838 | -0.7235510 | -0.6434689 | -2.3066313 | -0.5147451 | -0.5315816 |
| -3.1621615 | -0.6049318 | -1.4947688 | -2.9076726 | -0.0165765 | -0.0401487 | -1.8996834 | 0.0086333 | -0.5681695 |
| -1.0786041 | -0.2324842 | -0.9201838 | -1.2253588 | -0.8132183 | -1.0699125 | -1.4718343 | -0.9123307 | -1.5557387 |
| -0.1816348 | -0.1541149 | -0.1997440 | -0.5397226 | -0.1861740 | 0.0962739 | -0.1112166 | 0.0179764 | -0.1271457 |
| 1.2611081 | 0.1879308 | 1.7075136 | 0.7986050 | 0.0674549 | 1.6314430 | 2.3274367 | 0.0242457 | 1.6550469 |
| -0.3891694 | -0.2065640 | -0.1422627 | -0.3031323 | -0.1805281 | -0.5954624 | 0.0466449 | -0.2422970 | -0.1062971 |
| -0.4828840 | -0.3340779 | -0.2719502 | -0.2596864 | 0.2152398 | -0.7057294 | -0.8818032 | -0.0258574 | -0.6633945 |
| -0.0767016 | -0.2609406 | 0.2807179 | 0.4614398 | -0.7820029 | -1.1685241 | 0.9480820 | -1.0784116 | -0.8840884 |
| -0.9532314 | -0.1690043 | -0.0611193 | -0.4247616 | 0.3324570 | 0.8246509 | 1.1669377 | 0.4669572 | 0.7965758 |
| -0.0630971 | -0.5088531 | -0.4511440 | -0.3392370 | -0.7432303 | -1.1106433 | -0.4161466 | -1.1158731 | -0.7303760 |
| -0.0910761 | -0.1496707 | -0.1390389 | -0.3535381 | -0.1661119 | -0.0565772 | -0.2568617 | -0.0895538 | 0.0261867 |
| -0.3113044 | -0.0488549 | -0.3608481 | -0.2021772 | -0.1036448 | -0.6609876 | -0.5619943 | -0.0696238 | -0.6811940 |
| -1.7680023 | -0.3372462 | -0.9162216 | -1.5584153 | -0.7871179 | -0.8327637 | -2.2531823 | -0.7722556 | -1.2173982 |
| -1.0466575 | -0.1077601 | -0.3817562 | -0.8847819 | -0.1284690 | -0.3546391 | -1.0127626 | -0.1830309 | -0.3079731 |
| -1.4000676 | -0.2697290 | -0.4920434 | -1.3636626 | -0.2228305 | -0.1845033 | -0.8627834 | -0.0109062 | -0.2129351 |
| -0.3851888 | -0.3794083 | -1.2724802 | -0.4975997 | -0.3063582 | -0.2140288 | -1.0425893 | -0.0980133 | -0.4603544 |
| -2.0753885 | -0.3293992 | -1.6842950 | -2.3328613 | 0.0177944 | -0.8021286 | -2.3826873 | -0.2241463 | -1.4130054 |
| 0.9829357 | 0.3179998 | 0.5995095 | 0.7941875 | 0.2509556 | 0.1423147 | 1.5217567 | 0.3413000 | 0.2039598 |
| -1.5326746 | -0.5381221 | -0.7495013 | -1.2013357 | -1.0335325 | -1.0688138 | -2.0964256 | -0.6429943 | -1.2958340 |
| -0.5919427 | -0.6083125 | -0.4930091 | -0.4410144 | -0.9418014 | -0.8152238 | -1.1292027 | -0.9251444 | -0.8486435 |
| -4.1566728 | -1.1924730 | -2.7782524 | -4.0203476 | -0.7162794 | -1.2543502 | -2.9502998 | -0.7549704 | -1.9868072 |
| 0.0852409 | -0.1081548 | -0.3572718 | -0.0337862 | -0.1292645 | -0.1074322 | -0.2824357 | -0.1785482 | -0.1646677 |
| -0.8130699 | -0.1191316 | 0.0341593 | -0.7065860 | -0.9372906 | -0.3675375 | -0.8552808 | -0.6826109 | -0.5384857 |
| -0.4622488 | -0.4661862 | -0.6028839 | -0.8343707 | -0.2942713 | -0.3704367 | -0.3700561 | -0.5587518 | -0.1260650 |
| -1.0404211 | -0.2332591 | -0.4842323 | -0.8324227 | 0.0066842 | -0.1434886 | -2.1833025 | -0.0055241 | -0.5011075 |
| -1.3882634 | -0.6106094 | -0.8725064 | -1.2303110 | -0.5455464 | -0.8019748 | -0.9101228 | -0.5747977 | -0.9520962 |
| 0.6431532 | -0.0943626 | 0.0071440 | 0.8199858 | 0.0321587 | 0.0535497 | 0.7912437 | 0.1284731 | -0.0934339 |
| 0.8086025 | 0.3843841 | 0.5645003 | 0.8804362 | 0.2992583 | 0.6511580 | 1.3453939 | 0.2280846 | 0.8418402 |
| -1.5662648 | -0.6393321 | -1.1433777 | -2.0082136 | -0.3987673 | -0.5064635 | -0.9197487 | -0.4463282 | -0.8968351 |
| -0.7114649 | -0.3380444 | -0.5187489 | -0.7673978 | -0.3419005 | -0.5341863 | 0.0332353 | -0.2756027 | -0.6127555 |
| 1.0181474 | -0.7200127 | -0.0618704 | 0.5370975 | -0.7817331 | -0.6114524 | 1.9703027 | -0.7715138 | -0.3232605 |
| -0.8977140 | -0.1884373 | -0.8176369 | -0.8338774 | -0.0309853 | -0.0934603 | -0.9353014 | -0.1753874 | -0.3321228 |
| -0.9598002 | 0.0210275 | 0.1585457 | -0.8633662 | -0.3532825 | -0.7427158 | -0.5069962 | -0.4107449 | -0.5488510 |
| -1.8068957 | -0.7185869 | -1.2273703 | -1.7130664 | -0.0538462 | -0.5577512 | -0.8105438 | -0.0794416 | -1.0521128 |
| -2.6428144 | -1.3779244 | -2.5904527 | -2.5804947 | -1.1524657 | -1.4175869 | -2.2522763 | -1.6670329 | -1.3855119 |
| -0.1331226 | 0.0695437 | -0.1131778 | 0.0374945 | -0.1534080 | -0.2196589 | -0.0547013 | -0.2257047 | -0.2775786 |
| 0.0635085 | -0.3157331 | 0.4914707 | -0.0870319 | -0.4293315 | 0.6822504 | 1.0164999 | -0.5275502 | 0.9234632 |
| -1.2084364 | -0.2699407 | -0.8060319 | -1.0650280 | -0.7529349 | -1.6468008 | -1.0957713 | -0.4826851 | -1.3212591 |
| 0.8091000 | 0.1190351 | 0.2894104 | 0.5137315 | 0.0377592 | 0.5029370 | 1.2285149 | 0.0719558 | 0.4737374 |
| -1.5574439 | -0.5839252 | -0.9461881 | -1.8988824 | 0.0208615 | -0.5297341 | -0.7138731 | -0.2450936 | -0.8302654 |
| -1.0236036 | -0.8790762 | -1.1832625 | -0.8968058 | -0.6344572 | -0.6592049 | -0.9046630 | -0.7089417 | -1.3082386 |
| -0.8190046 | -0.2400024 | -1.0078583 | -0.7439353 | -0.8030043 | -0.8734106 | 0.4500470 | -0.7738422 | -0.6777047 |
| -0.6139861 | -0.8383437 | -0.4170437 | -0.8398399 | -0.4244792 | -0.7854999 | -0.7903068 | -0.5423373 | -0.7747194 |
| -0.5317717 | -0.3226349 | -0.2077331 | -0.7855534 | -0.1581811 | -0.0305477 | -0.1370192 | -0.3375457 | -0.4866608 |
| -0.1313462 | 0.1116019 | -0.0137545 | -0.3397401 | -0.5325288 | -0.3114883 | -0.2644258 | -0.3502592 | -0.6592484 |
| -1.5942197 | -0.5848331 | -0.6026400 | -1.4313967 | -0.9073344 | -0.3100114 | -0.6616721 | -0.8881031 | -0.5247765 |
| 0.0894957 | -0.2283577 | -0.2322791 | 0.3265629 | -0.2304567 | -0.5838424 | -1.1321205 | -0.2158501 | -0.1974858 |
| -1.0933658 | -0.8429907 | -1.0669517 | -1.1847562 | -0.6786626 | -0.8269184 | -1.2347056 | -0.9228361 | -0.8241362 |

| | | | | | | | | |
|---|---|---|---|---|---|---|---|---|
| 1.0923841 | 0.2015364 | 0.2721405 | 1.4645480 | -0.6665564 | 0.0384474 | 0.6135869 | 0.0320034 | 0.0380878 |
| 0.1100056 | 0.0721366 | 0.3202941 | -0.1291877 | -0.5415405 | -0.5070979 | -0.9130564 | -0.3162261 | -0.4813424 |
| -0.0161516 | -0.4009845 | -0.2778680 | -0.1841882 | -0.1528626 | -0.0374394 | 0.1877786 | -0.1044220 | 0.1540284 |

| PG_507 | PG_508 | PG_509 | PG_510 | PG_511 | PG_512 | PG_513 | PG_514 | PG_515 |
|---|---|---|---|---|---|---|---|---|
| -2.1284334 | -0.5236336 | -0.3068480 | -1.2769788 | -0.6500610 | -0.4264155 | -1.0954703 | -0.6080006 | 0.1188229 |
| -1.3779672 | -0.4426647 | -1.1810263 | -1.3706704 | -0.4146854 | -1.0265448 | -1.5106212 | -0.4866758 | -0.7538279 |
| -1.2319179 | -0.7492698 | -1.3356750 | -2.0443587 | -0.5652993 | -1.6079444 | -1.6784318 | -0.6039628 | -0.7458675 |
| 0.2150422 | -0.2629573 | -0.4216169 | 0.0542862 | -0.1842971 | -0.6183510 | -0.0986604 | -0.3352626 | -0.2934565 |
| -1.0427500 | -0.1263068 | -0.3471742 | -1.0938184 | -0.2101233 | -0.2329401 | -0.7741411 | 0.0470509 | -0.2013394 |
| -1.7568351 | -0.0850593 | -0.4373562 | -1.6714275 | -0.0059724 | -0.5546033 | -1.6065741 | -0.3709951 | -0.6702284 |
| -0.6157452 | -0.2302761 | -0.6907872 | 0.3347263 | -0.1281560 | -0.8721442 | 0.2013236 | -0.2295172 | -0.7577856 |
| -2.2357613 | 0.6491432 | 0.1946648 | -2.0253730 | 0.6544709 | -0.0720948 | -1.5926620 | -0.2110184 | -0.1397043 |
| -0.0161645 | -0.4696237 | -0.2681154 | -0.1586297 | -0.5018421 | -0.0861741 | -0.0906153 | -0.4876719 | -0.3166461 |
| -1.6541297 | -0.5928972 | -1.5473749 | -1.5210434 | -0.8315943 | -1.8380732 | -2.1014666 | -0.4553304 | -0.5269892 |
| -0.1142492 | -0.1240606 | -0.2804744 | -0.2614482 | -0.1899603 | -0.4464845 | -0.4059756 | -0.2707028 | -0.3194416 |
| -0.5007077 | -0.0362946 | -0.3152774 | -0.3366161 | -0.0676010 | -0.3141457 | -0.5367260 | -0.2505791 | -0.2083708 |
| -6.3361836 | -0.8733897 | -0.8813784 | -1.5609449 | -0.8586077 | -1.2035474 | -1.9996327 | -1.1458864 | -0.6376729 |
| 4.1430185 | 0.0648943 | 2.4681165 | 4.1431909 | 0.4353085 | 4.0048013 | 4.6513624 | -0.7102528 | -0.3083474 |
| -0.1646271 | 0.2010354 | -0.6933149 | -0.8370398 | 0.1584133 | -0.8547524 | -0.8262991 | -0.1267108 | -0.0019222 |
| 3.1444508 | -0.4807817 | 2.8086076 | 2.8477440 | -0.2980197 | 3.1956487 | 3.2201905 | -0.3662718 | 1.9137801 |
| -1.5510983 | -0.7484516 | -2.1349313 | -1.4329115 | -1.2938416 | -2.0823865 | -1.5172248 | -0.9313089 | -1.7488250 |
| -0.1451916 | -0.2548257 | -2.0444916 | -2.5105410 | -0.3999089 | -1.7732426 | -3.1012570 | 0.6869681 | -0.0292875 |
| -0.2972622 | -0.4826166 | -0.9609076 | -1.5891047 | -0.8948327 | -0.8829825 | -1.8593275 | -0.1256588 | -0.2011020 |
| -1.2050481 | -0.7510735 | 1.1451650 | 0.9963046 | -0.9919840 | 0.6435669 | 0.0233029 | -1.0657448 | -0.8700888 |
| 1.1447587 | -0.3506927 | 0.6951688 | 1.1578230 | -0.4244152 | 0.7759240 | 1.4071433 | -0.4057511 | -0.2296662 |
| -0.3233116 | -0.4695047 | -0.3175947 | -0.3982114 | -0.4002535 | -0.0943228 | -0.2513060 | -0.2707664 | -0.5311182 |
| 1.3300253 | -0.3905334 | 1.0201214 | 1.4452374 | -0.2122612 | 1.3990748 | 1.5913122 | -0.2771277 | 0.1287438 |
| -2.3220794 | -0.1224174 | -0.6336016 | -1.7691248 | -0.3174853 | -1.2170089 | -2.4456933 | -0.2068724 | -0.4470978 |
| -2.1847409 | -0.1758176 | -1.3233476 | -2.4277105 | -0.5889549 | -1.7266970 | -2.7084680 | -0.4660419 | -1.3281552 |
| 0.8972656 | -0.1386433 | 0.6202546 | 0.8027560 | 0.0684999 | 0.5886750 | 0.9617435 | -0.3397875 | 0.4528763 |
| -0.4793375 | 0.1167304 | -0.0676161 | -0.3484824 | 0.1699535 | -0.0940990 | -0.3659921 | -0.0105556 | -0.0522044 |
| -1.2942391 | -0.8159638 | -1.0866649 | -1.8913233 | -0.9290973 | -1.3572497 | -1.6314197 | -0.9366231 | -0.9905579 |
| -2.1580923 | -0.1728068 | -0.5232135 | -1.8424120 | -0.1109095 | -0.8068149 | -1.8743094 | 0.3010096 | 0.4139747 |
| -0.3804216 | -0.0543728 | 0.1609371 | -0.0613920 | -0.0165523 | -0.2148707 | -0.2813425 | -0.2327338 | -0.5008069 |
| -1.2901914 | 0.1165598 | -0.5898672 | -2.3882233 | 0.1873811 | -1.0676577 | -2.6625631 | 0.8872468 | 1.0609713 |
| 0.1042939 | -0.4332589 | 0.6349916 | 0.5360898 | 0.0842558 | -0.0047937 | -0.0569714 | 0.4499783 | 0.4781952 |
| -2.9000691 | -0.2234763 | -0.4314864 | 0.6305183 | -0.1958042 | 0.0770564 | 0.7711301 | -0.2573493 | -0.2630729 |
| -4.7749922 | -0.5241421 | -1.0087590 | -2.5739109 | -0.5445794 | -1.3215985 | -3.3454949 | -0.5271850 | -0.4150876 |
| -1.5235811 | -0.0753477 | -0.2572683 | -0.3296530 | -0.2674692 | -0.1543079 | -0.2831252 | -0.1330177 | -0.1629966 |
| -0.6596514 | -0.3797017 | -0.9490276 | -0.4904950 | -0.8986867 | -1.0099038 | -0.7341969 | -0.4457633 | -0.2440331 |
| -2.7020058 | -0.4047998 | -0.6814696 | -1.2711548 | -0.7045338 | -0.9925628 | -1.7236473 | -0.5819139 | -0.2934742 |
| 2.4450702 | 0.3210768 | 2.6299924 | 1.1425178 | 0.4115964 | 3.6255714 | 0.8773816 | 0.2251992 | 0.4781903 |
| -0.4668125 | -0.4614949 | -1.0797297 | 0.3404747 | -0.5805257 | -0.5380635 | -0.2356140 | -0.4500235 | -0.5059974 |
| 0.0356079 | -0.3917967 | -0.7662060 | 0.2192973 | -1.0037758 | -0.5031969 | -0.5682135 | -0.1774598 | -0.9522538 |
| 1.1342382 | -0.1779437 | -0.0817537 | 0.7592382 | -0.3070721 | 0.3064002 | 1.1557966 | 0.0256552 | 0.1018177 |
| -2.0098640 | -0.1101137 | -0.8798434 | -2.1794119 | -0.2633766 | -1.2622096 | -2.2979467 | -0.4887929 | -0.7886288 |
| -1.3593415 | -0.4327888 | -0.4481397 | -1.0030002 | -0.5414473 | -0.6202650 | -1.0508567 | -0.3244451 | 0.1970706 |
| -0.4571010 | 0.2375015 | -0.205336 | -0.7012515 | 0.1029941 | -0.1501708 | -0.9422433 | -0.2150033 | -0.0521652 |
| 0.9801483 | -0.0240963 | 0.5343759 | 1.1521115 | -0.1128882 | 1.0319702 | 1.5172197 | -0.1621510 | -0.2159357 |
| 1.1186775 | 0.1551222 | 0.0049276 | 1.4704020 | 0.3994506 | 0.7442299 | 3.1915037 | 0.0670821 | 0.6290454 |
| -0.2171296 | -0.1214610 | -0.2213807 | -0.2545244 | -0.2236762 | -0.2922434 | -0.7256665 | -0.1261137 | -0.0239152 |

| | | | | | | | | |
|---|---|---|---|---|---|---|---|---|
| 0.5157359 | -0.1560342 | -0.3518048 | 0.0803886 | -0.1544370 | -0.0010717 | 0.2399790 | -0.1002418 | -0.0424767 |
| -2.1281521 | -0.5168527 | -0.6095305 | -1.9205234 | -0.3158862 | -0.6786413 | -1.9370023 | -0.5258089 | -0.8427183 |
| 0.0742355 | -0.6686766 | -0.4533076 | 0.4663100 | -0.5370488 | -0.0605910 | 0.7200678 | -0.4312142 | -0.4659478 |
| -0.2641078 | 0.2448853 | 0.8832531 | 3.4682089 | 0.0058069 | 2.1515261 | 3.0691899 | -0.4336893 | -0.3664388 |
| -0.0156691 | -0.0226703 | -0.1952614 | -0.0389532 | -0.2487154 | -0.2196638 | -0.0745722 | -0.1389834 | -0.3036315 |
| 0.2646902 | -0.2661308 | 0.1002211 | 0.0939400 | -0.5551752 | 0.0222604 | 0.2901056 | -0.2042052 | -0.3911267 |
| -2.9806386 | -0.7652868 | -0.7808202 | -0.9266336 | -0.8421229 | -1.3502384 | -1.2112688 | -0.7644355 | -0.5064819 |
| 2.7216485 | -0.3228461 | 0.6206684 | 2.5156712 | -0.0943302 | 0.7985887 | 2.7767327 | -0.4452978 | 0.2019600 |
| 1.7181824 | 1.0366801 | 2.9713843 | 0.0190883 | 0.8901299 | 3.6398826 | 0.7090976 | -0.4273317 | 1.0903321 |
| -0.0051893 | -0.4764824 | -0.2392024 | -0.5387922 | -1.3009297 | -0.4200114 | -0.5692836 | -1.0607508 | -0.4036327 |
| -1.8054930 | -0.8548941 | -1.4603270 | -2.0312109 | -0.8444555 | -1.9910399 | -2.4553258 | -0.7840544 | -0.8145805 |
| 2.9023227 | 0.0301551 | 2.6702041 | 2.5884250 | 0.5007849 | 3.3248939 | 2.9295337 | -0.1598254 | 2.4908443 |
| 1.7992770 | -0.2817031 | 3.3562613 | 2.4769091 | 0.2137290 | 3.6550596 | 2.6161655 | -0.1277872 | 1.8509224 |
| -1.8417230 | -0.4371595 | -0.6214381 | -1.5397907 | -0.5872181 | -0.5253310 | -1.1653448 | -0.5242687 | -0.5354866 |
| -1.8024351 | -1.0261968 | -2.1512178 | -2.4759593 | -1.1937817 | -2.5272802 | -2.6979842 | -0.7503189 | -1.6148746 |
| -1.2050969 | -0.5766156 | -1.0627404 | -1.5157786 | -0.6955024 | -0.8582057 | -1.1310188 | -0.4254270 | -0.4962505 |
| -2.1686135 | -0.2266499 | -0.4324351 | -2.1521805 | -0.2404289 | -1.0050522 | -2.3999534 | -0.3289550 | -0.3836724 |
| -0.7752935 | -0.0376502 | -0.4467686 | 0.0174027 | -0.2197137 | -0.6069758 | -0.3049454 | -0.2310407 | -0.5037553 |
| -0.7115094 | -0.2912174 | -1.1154415 | -1.0774903 | -0.6030466 | -1.1206386 | -0.9962253 | -0.1763754 | -0.8897516 |
| -0.4409553 | 0.2085420 | -0.5533833 | -1.5022543 | 0.2758033 | -0.5153905 | -1.8541602 | -0.2865788 | -0.0911513 |
| 0.0497040 | -0.7450286 | -0.3611413 | 0.1479071 | -0.6203370 | -0.1915896 | 1.2030221 | -0.3308589 | 0.2328008 |
| -3.9047594 | -0.5368608 | -1.0773362 | -1.4696336 | -0.7490369 | -1.5150962 | -1.8975345 | -0.6652361 | -0.7731621 |
| 0.5269848 | -0.3012842 | -0.4754746 | 0.3751976 | -0.2261954 | -0.1438405 | 0.6301560 | -0.0044384 | 0.0318777 |
| 1.1776184 | 0.3143349 | 0.6528251 | 1.0637286 | 0.3538735 | 0.6827716 | 0.3725597 | 0.1757641 | 0.4345810 |
| -0.9471131 | -0.8397223 | -1.1690382 | -1.6174221 | -0.9828178 | -1.4781376 | -1.7923426 | -0.7103774 | -0.9052952 |
| -0.1812257 | -0.2180867 | -0.6666155 | 0.2964984 | -0.2784846 | -0.7762103 | 0.3557797 | -0.2600214 | -0.5490826 |
| -1.8431979 | -0.9740580 | -2.0412307 | -2.3439093 | -1.3474923 | -2.1646280 | -2.8225619 | -0.2462409 | -1.3398393 |
| 4.3640108 | 0.5701756 | 2.7527172 | 4.4902897 | 0.9334136 | 3.2170964 | 4.5809315 | 0.7532116 | 1.5360130 |
| 2.9112453 | -0.7385020 | 0.6252344 | 2.4545207 | -0.6204612 | 0.8941412 | 2.2424438 | -0.2337372 | 0.0086346 |
| -1.3359288 | 0.1013933 | -0.2603835 | -1.6774897 | 0.1103246 | -0.4910366 | -1.7947483 | -0.6937434 | -0.9600809 |
| -0.3141857 | -0.0153277 | -0.0189917 | 0.3317618 | -0.1510032 | -0.0575865 | 0.2429901 | -0.2017456 | -0.1342585 |
| -0.1172287 | -0.0396147 | -0.3512067 | 0.1236290 | 0.0189230 | -0.3720835 | -0.3240485 | -0.1428903 | -0.0070309 |
| -1.3021164 | -0.4553974 | -0.8901441 | -1.1324760 | -0.4331810 | -1.1249358 | -1.0823803 | -0.4807686 | -0.8089260 |
| -1.1570273 | -0.2898573 | -0.3748764 | -1.3098542 | -0.2012819 | -0.8502575 | -1.5635507 | -0.4467146 | -0.6013214 |
| 1.0111220 | -0.1829146 | 1.2425824 | 2.1146571 | 0.3038678 | 1.7194781 | 2.2041002 | -0.3633527 | 0.4632914 |
| -0.5791903 | 0.5529616 | -0.3018722 | -1.9470983 | 0.1759245 | -0.8284681 | -1.8448709 | 0.8464044 | 0.8149939 |
| -1.7551332 | -0.3230172 | -1.5840511 | -2.7301511 | -0.9967605 | -1.6563263 | -2.5582485 | -0.1776756 | -0.3474890 |
| -2.0442461 | -0.1133036 | -0.5168223 | -1.7846664 | -0.1932750 | -0.7504352 | -1.9793494 | -0.2058990 | -0.6072229 |
| -0.6904849 | 0.3772854 | -0.3678771 | -1.3208830 | 0.4253705 | -0.3748285 | -1.3755912 | -0.0516748 | -0.3737663 |
| -2.6946296 | -0.2895799 | -0.9615325 | -1.2588575 | -0.4721643 | -1.0599479 | -1.6994789 | -0.6814348 | -1.0202019 |
| -2.7115513 | -0.7951827 | -0.5971902 | -1.7262689 | -1.0263301 | -0.6950315 | -1.5001361 | -1.1010151 | -0.7385393 |
| -2.6382732 | -0.6601794 | -1.9082701 | -2.3717562 | -0.4638173 | -1.8737373 | -2.7617024 | -1.7703071 | -2.3930040 |
| 3.7500942 | 0.7024469 | 2.5371237 | 1.7392329 | 1.0606789 | 3.6378705 | 1.8909630 | 0.2614059 | 0.7931711 |
| -0.2685495 | -0.0735193 | -0.2201806 | -0.1802043 | -0.0710742 | -0.2630138 | -0.1931927 | -0.1420231 | -0.1136628 |
| -1.0758465 | -0.1572184 | -0.7647703 | -1.4063713 | -0.1257081 | -0.4810423 | -1.3409034 | -0.1094713 | -0.2776469 |
| -0.7805661 | -0.3707173 | -0.1372722 | -0.7318106 | -0.6057784 | -0.2298529 | -0.3624293 | -0.4180387 | -0.8451338 |
| 7.1167841 | -0.0745450 | 6.8306542 | 5.0250088 | 0.3693263 | 7.8429475 | 7.3474814 | -0.0499973 | 6.2153687 |
| 2.0207448 | 0.0232874 | 2.2548444 | 2.2287490 | 0.0082359 | 2.5489200 | 2.5745205 | -0.2833895 | 1.2391545 |
| 0.8709621 | 0.2023951 | -0.1898544 | -1.0578957 | -0.0822899 | 0.2940286 | -0.6115487 | 0.5109958 | -0.0409528 |
| 0.2146554 | 0.3640615 | -0.0156826 | -1.1724455 | 0.1279388 | -0.1519552 | -1.1614570 | -0.4829032 | -0.4650188 |

| | | | | | | | | |
|---|---|---|---|---|---|---|---|---|
| -2.8763245 | -0.0252465 | -0.1154666 | -0.3050345 | -0.2996088 | 0.0706510 | -0.0657003 | 0.3001517 | -0.2618454 |
| 0.3669534 | -0.1347555 | -0.5044455 | 0.0550874 | -0.1893109 | -0.1521846 | 0.0703240 | -0.2181102 | -0.1886060 |
| -0.0517087 | 0.0623494 | -0.8438748 | -0.6764757 | -0.2340364 | -1.1250532 | -0.5785516 | -0.5345612 | -0.8401534 |
| 0.1933288 | -0.4797042 | -0.3688019 | 0.0387122 | -0.2529609 | -0.2748944 | 0.2519365 | -0.2496401 | -0.1767196 |
| -1.7019129 | -0.1988605 | -1.3977814 | -2.0385940 | -0.2935074 | -1.6580083 | -2.2698900 | -0.2656483 | -1.0438667 |
| 0.1784675 | 0.8509719 | 0.1971418 | -0.1232665 | 0.8005311 | 0.1038845 | 0.1822342 | -0.2170489 | -0.4550032 |
| -0.6051256 | -0.5134772 | 0.2830921 | -0.5544261 | -0.7986183 | -0.1122033 | -0.1423838 | -0.4363210 | -0.7425941 |
| -0.1301577 | -0.0092822 | -0.1685775 | -0.1500720 | -0.2033052 | 0.0843798 | -0.1768408 | -0.0770941 | -0.2081928 |
| 0.3712141 | -0.1617418 | -0.081341 | -0.3589089 | -0.5494143 | -0.2000217 | -0.4487393 | 0.0553327 | -0.3264251 |
| 0.7061203 | -0.2984169 | 0.6196541 | 1.2427211 | -0.4346512 | 0.5071456 | 1.1600158 | -0.3285993 | 0.2396864 |
| -0.0196831 | -0.0031118 | -0.4271122 | -0.8586339 | -0.0832460 | -0.5847795 | -0.8856411 | 0.2748347 | 0.1135567 |
| -3.1497543 | -0.8729812 | -0.7699395 | -0.9942096 | -0.9407760 | -1.0300712 | -1.2723627 | -0.6434081 | -0.6821984 |
| -5.9847601 | -0.5161976 | -0.9864807 | -2.2639478 | -0.4406580 | -1.0763855 | -2.2233845 | -0.5478811 | -0.2237168 |
| -3.0904978 | -0.5126316 | -0.7825257 | -1.0304124 | -0.6998107 | -1.2711084 | -1.4768335 | -0.6111302 | -0.4215288 |
| -0.2153033 | -0.3447919 | -0.6674958 | -0.2004920 | -0.3648420 | -0.8842281 | -0.4217614 | -0.2081653 | -0.0974474 |
| -0.1808150 | 0.1081557 | 0.0377057 | 0.0286641 | -0.1439592 | 0.1038983 | -0.0705130 | -0.2039639 | -0.2097324 |
| -0.4657551 | -1.2818811 | -1.3171080 | -1.9772880 | -0.6986962 | -1.9279420 | -2.0108076 | -0.7079896 | -0.9930553 |
| -0.3658008 | -0.0173124 | 0.1164265 | 0.0594500 | -0.3217199 | 0.1623742 | 0.0870633 | -0.2046627 | -0.3524662 |
| -1.2241365 | -0.4931657 | -1.1469365 | -0.5808972 | -0.6163050 | -0.9835051 | -0.7184982 | -0.6195566 | -0.6356647 |
| 1.7553469 | -0.4422405 | -0.3345651 | 1.2938710 | -0.5427955 | 0.5027682 | 1.4808177 | 0.0660802 | -0.1722774 |
| -0.8858921 | 0.1365089 | -0.3210800 | -1.1550328 | -0.0816149 | -0.3190970 | -1.3343900 | -0.3534457 | -0.0616692 |
| 0.5460980 | 0.0530957 | -0.3503028 | -0.2588092 | 0.1699831 | -0.3138014 | -0.2356744 | -0.3189215 | -0.4271899 |
| -0.3973220 | -0.2436467 | -0.0951897 | -0.1627739 | 0.0832545 | -0.0383494 | -0.0017693 | -0.2271764 | -0.0386180 |
| -1.5990254 | -0.2707306 | -0.8029694 | -2.1275674 | -0.1771509 | -0.6153482 | -1.6050166 | -0.2463515 | -0.5744746 |
| -0.7416464 | -1.0566076 | -1.4884596 | -1.9145925 | -1.2941774 | -1.7544318 | -1.4426282 | -0.2141784 | -0.3123483 |
| -0.3491181 | -0.2201679 | -0.7329555 | -0.1633863 | -0.1928662 | -0.4835624 | -0.6697187 | -0.0812483 | -0.2460348 |
| 0.3641308 | 0.0585321 | -0.2589415 | 0.3535503 | -0.0115389 | -0.1188321 | 0.1864583 | 0.0308010 | -0.0270476 |
| 0.2315152 | -0.4249096 | -0.1305655 | -0.2370038 | -0.4464173 | 0.1573938 | -0.1718986 | -0.0065374 | -0.0583661 |
| -1.1103768 | -0.2574962 | -1.0029106 | -1.0504034 | -0.2788430 | -0.9936504 | -1.7110539 | 0.0391580 | 0.0224773 |
| -4.3124326 | -0.5203795 | -0.5293587 | 0.0860888 | -0.7593059 | -0.4801218 | 0.1033418 | -0.5433996 | -0.5138698 |
| 0.0465695 | -0.3040121 | -1.0663123 | -3.2016438 | -0.1815820 | -1.2762311 | -2.7971032 | -0.6926407 | -0.7769927 |
| -1.4403302 | 0.1095221 | -0.2767329 | -1.1017364 | -0.0242397 | -0.4382761 | -1.4923182 | -0.2875192 | -0.2799469 |
| -1.6722270 | -0.2678467 | -1.4465913 | -0.7586047 | -1.0305616 | -1.1174029 | -0.5409991 | 0.0709439 | -0.8275633 |
| 0.1477003 | 0.1522917 | -0.1649709 | -0.3521585 | 0.2369183 | 0.2559500 | -0.4479218 | 0.0260389 | 0.2634361 |
| -1.4003513 | -0.1522796 | -0.6618202 | -1.8684390 | -0.0611723 | -0.8060735 | -1.7484043 | -0.4432470 | -0.6255680 |
| -1.0151665 | -0.2965034 | -0.2623318 | -1.1225290 | -0.2822872 | -0.6910227 | -1.4617203 | -0.4651412 | -0.4880594 |
| -0.0972819 | -0.2542794 | -0.1414881 | -0.2449057 | -0.1956232 | -0.2902118 | -0.4335635 | 0.0495995 | 0.0588514 |
| -1.3193283 | -0.1651560 | -0.2955042 | -0.2955184 | -0.0819041 | -0.3077293 | -0.3968338 | -0.1957962 | -0.3053623 |
| -0.7247255 | -0.3849329 | -0.1147909 | -0.6645219 | -0.4959986 | -0.5082887 | -0.6213895 | -0.5508546 | -0.3985597 |
| -4.3719931 | -0.4077769 | -1.0720769 | -0.5809272 | -0.4064884 | -1.3709581 | -0.5949280 | 0.0509137 | -0.2725919 |
| -1.3504982 | -0.1871431 | -0.3184949 | 1.3380759 | -0.2526053 | -0.5369143 | 0.3585552 | -0.0616710 | -0.3441957 |
| -2.7508600 | 0.0030510 | -0.2725547 | -0.3218385 | -0.0796791 | -0.0277859 | -0.1974294 | -0.1349325 | -0.1266124 |
| -1.4561351 | -0.1251388 | -0.1346800 | -0.5199448 | 0.0403432 | -0.2589619 | -0.6280274 | -0.1898474 | 0.0155337 |
| -0.7648576 | -0.0433667 | -0.8276707 | -0.9605456 | 0.0109968 | -0.4504102 | -1.2882190 | -0.1899571 | -0.1940889 |
| -0.1445678 | -0.1920581 | 0.0064145 | -0.0927261 | -0.0855535 | -0.0185704 | -0.0289048 | -0.2103726 | -0.0639230 |
| -0.0234310 | -0.3893014 | 0.3050777 | -0.0272415 | -0.1135807 | 0.2215394 | 0.1376599 | -0.5078212 | -0.0189032 |
| -1.6935318 | -0.6022596 | -0.6950939 | -0.7868025 | -0.9987756 | -0.5671212 | -0.8120708 | -0.5806098 | -0.6452311 |
| 1.0404643 | -0.4434720 | 0.1749800 | 0.6716002 | -0.5751081 | 0.1540367 | 0.9754130 | -0.5264411 | -0.1542232 |
| -3.4951152 | -0.4623321 | -0.8246661 | -1.8250980 | -0.3772907 | -0.9014759 | -1.8377611 | -0.5666683 | -0.9425459 |
| -1.7765140 | -0.7259913 | -1.1601436 | -2.0688604 | -0.8065072 | -0.6169436 | -1.9004340 | -1.0057341 | -0.4234457 |

| | | | | | | | | |
|---|---|---|---|---|---|---|---|---|
| 0.2181791 | 0.3270608 | 0.2071217 | 0.9260760 | 0.3625834 | 0.6085647 | 0.7151563 | 0.2434526 | 0.0124438 |
| -2.0432594 | -0.8987059 | -1.9494922 | -2.9009369 | -1.4431101 | -2.3118412 | -2.9878387 | -0.9264455 | -1.2463842 |
| -1.7114856 | -0.4902757 | -0.0600642 | -0.1883683 | -0.4477865 | -0.4480705 | -0.0285255 | -0.6542715 | -0.6961177 |
| 1.7058225 | -0.5491294 | -0.2653436 | 0.2182087 | -0.1360123 | 0.0779335 | 0.6637975 | -0.2525852 | -0.4684353 |
| -1.1994360 | -0.1876089 | -1.2102265 | -1.7577603 | -0.3368948 | -1.5032770 | -2.1078839 | -0.6604852 | -0.5007882 |
| 0.8523328 | 0.4318277 | 1.6941086 | 0.9846405 | 0.5272570 | 2.1482558 | 1.6947588 | -0.3943609 | -0.3142649 |
| -0.4817131 | -0.3814601 | -0.5990912 | -0.7314546 | -0.4733676 | -0.6329020 | -0.8319746 | -0.3512305 | -0.4031096 |
| -1.8456331 | -0.8228847 | -0.2626599 | -0.8975936 | -0.7299332 | -1.0503463 | -0.8825122 | -0.6555904 | -0.8549636 |
| -1.7022739 | -0.5585522 | -1.6789359 | -2.449978 | -0.5698885 | -1.6058822 | -2.3845742 | -0.4001609 | -0.7918970 |
| 0.2060206 | -0.3205403 | -0.5511631 | 0.5283078 | -0.3360686 | -0.3232774 | 0.6344299 | -0.6377495 | -0.4210463 |
| 0.2849518 | -0.0061393 | -0.0739361 | 0.0252557 | -0.1097735 | -0.0012062 | 0.3151539 | -0.2539739 | -0.1148957 |
| -2.0295035 | -0.4429628 | -1.0320524 | -1.6337383 | -0.6756590 | -0.9653786 | -1.3025129 | -0.7263454 | -0.8153866 |
| -0.8886514 | -0.1850072 | -0.6618496 | -1.2438182 | -0.0580986 | -0.8034110 | -1.1402897 | -0.1937838 | -0.4630374 |
| 0.2024672 | -0.6089689 | -0.6882011 | -0.6124133 | -0.3130681 | -0.5168618 | -0.5452727 | -0.4109320 | -0.1909668 |
| 0.3287091 | -0.5736235 | -0.3149692 | -1.3865551 | -0.2647178 | -0.6946646 | -1.0128729 | -0.0950594 | -0.4758928 |
| -1.4698872 | 0.4830971 | 0.0774374 | -2.1274912 | 0.2173267 | 0.0269820 | -1.6512569 | 0.3684209 | 0.6134438 |
| -1.1294945 | -0.3135245 | -0.6876084 | -0.4413593 | -0.4431128 | -0.5978807 | -0.081884 | -0.4406353 | -0.6166027 |
| -0.9198168 | -0.3980252 | -1.1490509 | -1.1683499 | -0.7684637 | -2.2215268 | -1.6964796 | -0.6223860 | -1.2909203 |
| -0.8585936 | -0.3433501 | -0.6653945 | -0.6596704 | -0.2128218 | -0.7149119 | -0.5864046 | -0.6014647 | -0.8124831 |
| -1.0617472 | -0.2109608 | -0.5953219 | -0.5923156 | -0.2760819 | -0.7892050 | -1.0021906 | -0.3519102 | -0.5202820 |
| 0.5831232 | -0.5723352 | 0.2677925 | 0.0481263 | -0.8840707 | 0.3513894 | 0.4297511 | -0.0863896 | -0.3864984 |
| 0.2468376 | -0.4457662 | -0.1158593 | 0.5756743 | -0.5666730 | -0.0160223 | 0.3436128 | -0.2709713 | 0.3498674 |
| -2.2162619 | -0.2366981 | -1.0406702 | -2.0690214 | -0.0853963 | -1.4015276 | -2.3419856 | -0.4313673 | -0.7665083 |
| -0.6674285 | -0.1393522 | -0.2924303 | -0.3621968 | -0.4847917 | -0.1305073 | 0.0711042 | -1.3863543 | -0.6042793 |
| -1.4360255 | 0.1314914 | -0.6303986 | -2.3577208 | 0.0796740 | -0.9111202 | -2.0775025 | -0.2070308 | -0.6649456 |
| -0.2528734 | -0.1609408 | -0.7093539 | -0.6301047 | -0.2282220 | -1.0093534 | -0.9796646 | -0.4507603 | -0.6142797 |
| -0.9653640 | -0.5809927 | -1.2761993 | -1.1174051 | -0.8150300 | -1.4828609 | -1.9919035 | -0.5449900 | -0.5364090 |
| -0.3004987 | -0.2932534 | -0.5079957 | -0.1548793 | -0.3229662 | -0.4482639 | -0.0088938 | -0.5268676 | -0.6838547 |
| -1.3487696 | -0.6532078 | -1.6786819 | -1.4099559 | -0.7167756 | -1.5138376 | -1.6628704 | -0.5336659 | -0.4047466 |
| 1.4134886 | -0.1971420 | 1.2605206 | 1.7347850 | 0.0197218 | 0.7449929 | 1.8965452 | -0.2957249 | 0.2232955 |
| 3.2352112 | -0.8049155 | 0.5797500 | 2.6594614 | -0.5339037 | 1.2638100 | 3.1073290 | -0.0802421 | 0.5759657 |
| -0.3430333 | -0.5463359 | -1.1066169 | -0.5941108 | -0.3711318 | -1.3785995 | -0.9685206 | -0.8667669 | -1.3356936 |
| 0.1979415 | -0.1484297 | -0.1352647 | 0.1220154 | -0.0005173 | -0.0136079 | 0.0123864 | -0.1901025 | -0.0970985 |
| 0.6185671 | 0.1241045 | 0.2195278 | 0.5464565 | 0.2357256 | 0.3737593 | 0.5654116 | -0.0295505 | 0.1668436 |
| 0.6234038 | -0.0130089 | 0.6627574 | 0.2484988 | -0.0326356 | 0.8982973 | 0.3994945 | 0.0261960 | 0.3567121 |
| -1.4626730 | -0.1635338 | -0.4529690 | -1.0298368 | 0.0157223 | -0.4846693 | -1.0800103 | -0.9086433 | -0.7980665 |
| 1.3015530 | -0.2445416 | 0.6938514 | 0.3523568 | -0.2180559 | 0.8745018 | 0.5889425 | -0.0150327 | 0.3913120 |
| 0.8985835 | -0.1267086 | 0.3283218 | 0.9472122 | -0.2607231 | 0.5971242 | 1.7549436 | -0.1460421 | 0.0093366 |
| 0.3653419 | -0.1505074 | -0.4009889 | 0.2192228 | -0.3719325 | -0.2822975 | 0.4255046 | -0.2542814 | -0.3091595 |
| -0.9871604 | -0.2769783 | -0.3507570 | -0.9522965 | -0.3270811 | -0.5832987 | -1.2309248 | -0.1242677 | -0.1299693 |
| -0.8786125 | -0.6610842 | -1.0825212 | -1.0203383 | -0.7703202 | -0.7443004 | -1.0206111 | -0.1571542 | -0.8395948 |
| 0.6840464 | -0.8881519 | -0.0914319 | 0.6308947 | -0.6671994 | 0.0337752 | 0.8398029 | -0.7606803 | -0.6457820 |
| 2.1265024 | -0.4228861 | 0.1895884 | 1.5391926 | -0.1538021 | 1.0275962 | 1.4528741 | -0.4581754 | 0.0151529 |
| 0.3854572 | -0.3265000 | -0.0666161 | 0.7103127 | -0.1111035 | 0.1000771 | 0.3847831 | -0.3027852 | -0.1016731 |
| -0.7270854 | -0.1412167 | -0.2822604 | -0.7298196 | -0.0874853 | -0.5208339 | -0.6373665 | -0.1786101 | -0.3139840 |
| -0.0813103 | -0.1810819 | -0.1233586 | 0.4158926 | -0.5315718 | 0.1495059 | 0.5466002 | -0.2355996 | -0.3489796 |
| -1.3258467 | -0.4657876 | -1.1490957 | -2.6000005 | -0.8434008 | -1.5158950 | -2.5799187 | -0.6117706 | -0.5913306 |
| -1.9109490 | -0.4376660 | -1.3258350 | -1.3832436 | -0.6651917 | -1.4926397 | -1.6253534 | -0.2260528 | -1.0146685 |
| -1.2668763 | 0.1040642 | -0.4253689 | -1.8589441 | 0.0727909 | -0.6495621 | -2.1078192 | 0.1285828 | -0.1132918 |
| 0.2033586 | -0.1408824 | -0.4749891 | -0.0884901 | -0.1567834 | -0.2924516 | -0.2385546 | -0.2971909 | -0.2620653 |

| | | | | | | | | |
|---|---|---|---|---|---|---|---|---|
| 0.8279020 | 0.1627668 | 0.1590554 | 0.4952523 | 0.1756989 | -0.0353685 | 0.1305774 | -0.1076151 | 0.0187122 |
| -0.2368555 | 0.1941352 | 0.0955841 | -0.3848503 | 0.3592649 | 0.4777421 | 0.1347804 | -0.8900214 | -0.3907435 |
| -1.7702247 | -0.8386985 | -0.4110722 | -0.3062318 | -0.8861061 | -0.3514314 | -0.3488051 | -0.8528837 | -0.4569703 |
| -2.4683677 | -0.4736604 | -1.1849024 | -1.5705740 | -0.8285824 | -0.7582553 | -1.6056580 | -0.3664529 | -0.8820135 |
| -1.4029824 | -0.4307028 | -0.9506625 | -1.3870810 | -0.5160012 | -0.9473729 | -2.0366644 | -0.5524182 | -0.5016550 |
| -0.4012307 | -0.3267804 | 0.2787428 | -1.1136763 | -0.1058435 | 0.0278344 | -1.1643480 | -0.2692609 | -0.2272950 |
| 1.9419057 | -0.1600857 | 1.4169330 | 1.7910179 | 0.2221600 | 1.7910491 | 1.3902662 | -0.1112482 | 0.8025731 |
| -0.1067025 | -0.2751518 | -0.2201481 | -0.1675235 | -0.1845559 | 0.1288106 | -0.2371078 | -0.1309058 | -0.2362736 |
| -1.1217763 | -0.4856830 | -0.3252926 | -1.1297683 | -0.2183362 | -0.4885241 | -1.2419928 | -0.3654233 | -0.3041540 |
| 0.5907354 | -0.3601224 | -0.7763019 | -0.5232318 | -0.3998467 | -1.0047507 | -0.1424543 | -0.5941734 | -0.4596455 |
| 0.9276031 | -0.0378703 | -0.6144858 | -0.8545664 | 0.0122202 | -0.8777488 | -1.2624408 | 0.6697377 | 0.1955739 |
| -0.6192963 | -0.4884840 | -0.4759955 | -0.5258927 | -1.3038004 | -0.4027077 | -0.2855781 | -0.7321652 | -0.5768506 |
| -0.1197393 | 0.1720651 | -0.1066795 | -0.2184359 | -0.0134920 | 0.0325036 | -0.1315595 | -0.4208459 | -0.0853490 |
| -0.3921461 | 0.0330795 | -0.3892706 | -0.5875735 | -0.0984427 | -0.1387017 | -0.7548616 | 0.0511933 | 0.1085298 |
| -2.4203719 | -0.1602386 | -0.2900612 | -1.2551980 | 0.0128067 | -0.7316267 | -1.3947777 | -0.1386326 | -0.1690724 |
| -1.2002536 | -0.5149134 | -0.8053099 | -1.7171877 | -0.5300336 | -0.8731189 | -1.7573700 | -0.0420522 | 0.0934676 |
| -0.9454116 | 0.1718419 | -0.0746908 | -1.2560819 | 0.2122326 | -0.3196822 | -1.3231392 | -0.2103606 | -0.2343232 |
| -0.6452424 | -0.4116863 | -0.9429013 | -0.5784786 | -0.2210236 | -0.6134411 | -0.6975307 | -0.3258207 | -0.1439012 |
| -2.5843141 | -0.1011730 | -1.3275473 | -2.2584458 | -0.2838036 | -1.5989649 | -2.5041236 | -0.2986123 | -0.3412812 |
| 1.4025392 | 0.1629699 | 0.1804531 | 0.1425356 | 0.2393663 | 0.0667884 | -0.1985656 | 0.0241917 | 0.2179951 |
| -1.7637355 | -0.5355802 | -0.8433973 | -1.2429105 | -0.7323881 | -0.7079426 | -1.5799040 | -0.7524164 | -1.3190683 |
| -0.9128859 | 0.1116065 | -0.6440742 | -3.1247457 | -0.0523061 | -0.6602233 | -2.9048926 | -0.2417648 | -0.2010659 |
| -3.0834082 | -0.3002355 | -1.1652323 | -2.7161430 | -0.4285633 | -1.7618198 | -2.5247422 | -0.5742624 | -0.8940029 |
| -0.1879312 | -0.1120425 | -0.1513155 | 0.3018439 | -0.2156829 | -0.1222858 | 0.2396133 | -0.2734997 | -0.1874824 |
| -0.9839695 | -0.3554568 | -0.5343371 | -0.6755967 | -0.4733390 | -0.2045258 | -0.9190386 | -0.5196405 | -0.4622420 |
| -0.1214274 | -0.5321484 | -0.7721040 | -0.8746574 | -0.6768995 | -0.5764792 | -0.7744958 | -0.5354367 | -0.6187645 |
| -2.2840753 | -0.0576244 | -0.0321157 | -0.9968703 | -0.0145399 | -0.2976688 | -0.7648386 | -0.3201851 | -0.3498722 |
| -0.9487348 | -0.2040755 | -0.2942155 | -0.7876698 | -0.3014620 | -0.4193660 | -0.9589196 | -0.7251187 | -0.6626747 |
| 0.9070851 | -0.2427455 | 0.3236291 | 1.0817371 | -0.0497831 | 0.1886303 | 1.0463672 | -0.2511258 | 0.0496320 |
| 1.1642765 | 0.2855948 | 0.2856578 | 1.2926872 | 0.1663683 | 0.9859773 | 1.2884782 | 0.2246093 | 0.2390942 |
| -1.4258323 | -0.7418807 | -0.4275841 | -1.3134558 | -0.8324313 | -0.7305131 | -2.6282229 | -0.7603888 | -0.5211209 |
| -0.0436879 | 0.0549657 | -0.2704392 | -0.2065468 | 0.0679453 | -0.3912086 | -0.1639498 | -0.1081477 | -0.1979858 |
| 1.1865440 | -0.7482219 | -0.7709496 | 1.6215411 | -0.7433528 | -0.6210259 | 1.2082978 | -0.8339220 | -0.5378011 |
| -0.9487497 | -0.3833069 | -0.3762444 | -1.6617721 | -0.3012502 | -0.8173563 | -1.5977681 | -0.3822360 | -0.3300049 |
| -0.7599545 | 0.0289542 | -0.6249392 | -0.7625828 | 0.1805126 | -0.2808395 | -0.8310419 | -0.2207084 | -0.4312739 |
| -0.9208911 | -0.2438604 | -0.4085087 | -0.4646258 | -0.3392809 | -0.4426420 | -0.5186654 | -0.2363859 | -0.4878892 |
| -2.3313730 | -0.8296887 | -1.3783020 | -1.9775052 | -1.3120662 | -1.5742826 | -2.0914936 | -0.8972844 | -0.9901773 |
| -0.0061672 | -0.2459406 | -0.0486148 | -0.4774810 | 0.1160205 | -0.2513765 | -0.0128413 | -0.2276898 | -0.2052651 |
| 0.8893615 | -0.2949839 | 0.9435276 | 0.7919003 | -0.4035280 | 1.4929921 | 0.6535354 | -0.6313410 | -0.3572340 |
| -1.0795684 | -0.0456434 | -0.6391766 | -0.8764334 | -0.0885436 | -0.6294065 | -0.982149 | -0.2527895 | -0.4432833 |
| 1.3642010 | 0.0524897 | 0.2924615 | 1.0499420 | 0.1095677 | 0.4598160 | 1.0014429 | 0.0146681 | 0.1637315 |
| -1.1493595 | -0.2521462 | -0.7862026 | -1.0381469 | -0.3476922 | -0.9831073 | -1.5264412 | -0.1963964 | -0.5326050 |
| -1.1951111 | -0.0641801 | -0.4841316 | -0.5933609 | -0.6591443 | -0.6728938 | -1.0479026 | -0.5531936 | -0.6653893 |
| 0.0554958 | -0.0207898 | -0.4395357 | -0.1412027 | -0.3545174 | -0.3333551 | -0.1482121 | 0.1199188 | -0.0893608 |
| -0.6285110 | -0.6780861 | -0.6959423 | -0.2807944 | -0.5411879 | -0.4890016 | -0.0239931 | -0.7953810 | -0.4021648 |
| -0.1780423 | -0.1088328 | -0.8348342 | -0.7824410 | -0.5351993 | -0.8599409 | -1.1145166 | -0.2886570 | -0.6406178 |
| -0.2323280 | -0.3696498 | -0.4122970 | -0.6570079 | -0.2508635 | -0.4261998 | -0.6494173 | -0.1534394 | -0.4307079 |
| -0.7097377 | -0.9598423 | -0.6299355 | -1.1717850 | -1.1781450 | -1.0179717 | -2.1222048 | -1.0405160 | -1.1514641 |
| -0.7485151 | -0.7237649 | 0.1503870 | 0.6282801 | -0.5112935 | 0.1917879 | 1.1398503 | -0.5158749 | -0.8085434 |
| -1.1948089 | -0.7716078 | -1.2037161 | -1.8533353 | -1.2280561 | -1.7507110 | -1.3850641 | -1.2078346 | -1.2765780 |

| | | | | | | | | |
|---|---|---|---|---|---|---|---|---|
| 0.4955377 | -0.420067 | -0.170166 | 0.7521709 | -0.775255 | -0.009287 | 1.3797914 | -0.058801 | -0.608687 |
| -0.555267 | -0.632682 | -0.975967 | -0.026967 | -0.576404 | -0.811449 | 0.4286044 | -0.182555 | -0.365343 |
| 0.3627836 | -0.173063 | -0.240246 | 0.4536598 | -0.061182 | -0.017581 | 0.2617230 | -0.402122 | -0.265371 |

| PG_516 | PG_517 | PG_518 | PG_519 | PG_520 | PG_521 | PG_522 | PG_523 | PG_524 |
|---|---|---|---|---|---|---|---|---|
| -1.034971 | -0.610593 | 0.3388718 | -1.627391 | 0.0099421 | -0.134313 | -0.831512 | 0.2624666 | -0.262089 |
| -0.527290 | -0.617055 | -0.855075 | -0.984218 | -0.533006 | -0.991220 | -0.911631 | -0.496503 | -1.603605 |
| -0.823132 | -0.359556 | -0.586578 | -0.993840 | -0.920903 | -1.578726 | -1.758072 | -0.958392 | -2.140624 |
| -0.341614 | -0.380319 | -0.589164 | -0.700426 | -0.247256 | -0.101548 | 0.0251637 | -0.191428 | -0.398633 |
| -0.373145 | -0.008791 | -0.103031 | -0.491288 | -0.074462 | -0.115615 | -0.768158 | -0.123859 | -0.216495 |
| -1.900832 | -0.526728 | -0.800659 | -2.462331 | -0.325476 | -0.083448 | -1.099976 | -0.420372 | -0.443650 |
| -0.738912 | -0.154718 | -1.008409 | 0.1098118 | -0.361148 | -0.968535 | -0.351127 | -0.604012 | -0.711428 |
| -0.752191 | -0.257206 | -0.061797 | -1.182917 | -0.562498 | -0.661530 | -1.572104 | -0.579236 | -0.502201 |
| -0.918726 | -0.766098 | -0.110203 | -1.122740 | -0.341255 | 0.0297130 | -0.493944 | -0.149805 | 0.2496750 |
| -0.758007 | -0.461391 | -0.459453 | -0.711307 | -1.073720 | -1.273333 | -0.868092 | -1.327691 | -1.407375 |
| -0.702226 | -0.245286 | -0.474156 | -1.296498 | -0.315930 | -0.202706 | -0.644432 | -0.313497 | -0.379089 |
| -0.531639 | -0.221493 | -0.542507 | -0.733722 | -0.545738 | -0.591004 | -0.400003 | -0.613657 | -0.611768 |
| -0.490384 | -1.017773 | -1.305234 | -0.634093 | -0.970405 | -0.926634 | -0.721625 | -1.196791 | -1.030337 |
| 1.5503846 | -0.561777 | 1.6236658 | 3.2760713 | -0.706957 | 0.9972614 | 2.4456014 | -0.047176 | 2.1606544 |
| 0.1472044 | -0.021272 | 0.1216663 | 0.9332690 | 0.0443645 | -0.056755 | 0.6572032 | 0.2054711 | 0.2816211 |
| 1.4820904 | 0.1203327 | 2.4311761 | 2.2040303 | -0.212863 | 1.9532340 | 0.4026719 | 0.9760276 | 2.3402584 |
| -1.251692 | -0.612065 | -1.181798 | -1.481166 | -1.054964 | -2.214619 | -1.557479 | -1.242229 | -2.343601 |
| -0.049870 | 0.1507556 | -0.127773 | -1.303460 | -0.054230 | -2.079108 | -0.095819 | -0.965927 | -1.608082 |
| -0.092217 | 0.3311316 | 0.2086039 | 0.3021150 | -1.497655 | -1.950292 | -2.422136 | -2.099733 | -2.073222 |
| -0.336056 | -1.069518 | -1.097011 | -0.553925 | -1.325027 | -1.284701 | -1.094989 | -1.237598 | -1.205145 |
| 0.1052907 | -0.337384 | -0.368412 | 0.4056109 | -0.166759 | 0.3452460 | 0.2930706 | -0.124283 | 0.3938855 |
| -0.388988 | -0.329655 | -0.439391 | -0.365627 | -0.218387 | -0.425670 | -0.519790 | -0.474347 | -0.267342 |
| 0.6487287 | -0.061451 | 0.4431282 | 0.9826611 | -0.000155 | 1.1088096 | 1.3764735 | 0.1963922 | 1.2862633 |
| -0.514697 | -0.435779 | -1.054774 | -1.189029 | -0.278002 | -0.791553 | -1.224889 | -0.706781 | -1.183137 |
| -2.681341 | -0.769666 | -1.966741 | -3.025277 | -0.335181 | -1.355546 | -2.136930 | -0.869594 | -1.655991 |
| 0.5297343 | -0.247476 | 0.2986553 | 0.6835974 | 0.2012039 | 1.0737968 | 1.1710818 | 0.4490248 | 0.4527372 |
| 0.2340151 | 0.0500792 | -0.002502 | 0.2108094 | -0.012604 | -0.024983 | -0.132445 | -0.077965 | -0.083268 |
| -1.696879 | -1.339774 | -1.522006 | -1.085663 | -0.641324 | -0.544631 | -1.324551 | -0.256672 | -1.057387 |
| -0.585886 | 0.1841387 | 0.0357611 | -1.722127 | -0.413056 | -0.992537 | -1.261129 | -0.495773 | -1.301526 |
| -0.015181 | -0.254089 | -0.609118 | 0.1603756 | -0.212104 | -0.373988 | -0.087142 | -0.303400 | -0.536879 |
| -0.260592 | 0.7985091 | 0.7832740 | -1.046210 | -0.872000 | -1.331627 | -0.988936 | -0.935681 | -1.539786 |
| -0.494413 | 0.3607784 | 0.2802075 | -0.586630 | -0.282941 | -0.557898 | -1.274551 | -0.393634 | -0.957353 |
| -0.012591 | -0.162633 | 0.1331634 | 0.9434335 | -0.263604 | -0.269598 | -1.052318 | -0.126239 | 0.1335902 |
| -1.045068 | -0.661186 | -0.853436 | -1.300731 | -0.848182 | -0.905711 | -1.978566 | -1.119018 | -1.320322 |
| -0.125485 | -0.152553 | -0.128593 | -0.114845 | -0.356589 | -0.401487 | -0.175323 | -0.415216 | -0.308952 |
| -0.308574 | -0.369562 | 0.0575726 | 0.3088961 | -0.326019 | -0.521685 | 0.1100931 | -0.866112 | -0.253014 |
| -0.141513 | -1.012124 | -1.063917 | -0.752685 | -0.505675 | -0.986175 | -0.931889 | -1.011264 | -1.744717 |
| -0.084683 | -0.037479 | 2.9591390 | 0.2040553 | 0.1935364 | 2.3070043 | 1.0658953 | 1.9248941 | 3.2773007 |
| -0.172163 | -0.762424 | 0.2338936 | 0.4269739 | -0.354294 | -0.245388 | 0.2213883 | -0.478775 | -0.022168 |
| 0.2737738 | -0.850098 | -0.642910 | 0.0522928 | -0.621887 | -0.849805 | 1.1105395 | -1.017401 | -0.456156 |
| 0.3744409 | -0.139044 | 0.2741464 | 0.1580222 | -0.098973 | 0.2261375 | 0.7945888 | 0.0642586 | 0.1659863 |
| -1.920050 | -0.486447 | -0.998993 | -2.210781 | -1.011686 | -1.281966 | -2.220008 | -1.111959 | -1.250192 |
| -0.031939 | -0.322807 | 0.1616575 | -0.054836 | -0.389919 | -0.059499 | 0.7824774 | -0.185784 | 0.1269580 |
| -0.324402 | 0.0738690 | -0.148475 | -0.916357 | -0.130781 | -0.434378 | -0.504159 | -0.250550 | -0.174059 |
| -0.204650 | -0.204590 | -0.212063 | 0.1642139 | -0.144596 | 0.5882581 | 0.6920237 | 0.0759253 | 0.7500403 |
| 0.2168757 | 0.1153152 | 0.6113829 | 0.1883192 | -0.280071 | 0.0458206 | 2.4123073 | -0.040260 | 1.7672220 |
| -0.020483 | -0.025551 | 0.0295083 | 0.2805029 | -0.092992 | -0.186536 | -0.020816 | -0.342292 | -0.317449 |

| | | | | | | | | |
|---|---|---|---|---|---|---|---|---|
| 0.0918561 | -0.0763461 | 0.1500277 | -0.0146951 | -0.3892351 | -0.1979811 | -0.0834963 | -0.6109283 | -0.0160156 |
| -1.3501059 | -0.5592567 | -0.9359609 | -1.9747481 | -0.8284976 | -0.9438175 | -1.8156376 | -0.8787182 | -1.2743574 |
| -0.1796245 | -0.4206795 | -0.2733673 | -0.6334662 | -0.4248734 | -0.1482189 | 0.0033300 | -0.2789233 | -0.0260592 |
| -0.1600165 | -1.0332495 | -0.0063883 | 0.5024709 | -0.5908853 | 0.8423382 | 1.9405406 | -0.1019171 | 0.7355953 |
| -0.1173476 | -0.3672755 | -0.2887983 | 0.2193993 | -0.1911343 | -0.5676691 | -0.4051853 | -0.4445480 | -0.3707436 |
| -0.0667654 | -0.2703967 | -0.3355646 | -0.1555342 | -0.0472279 | -0.2000186 | -0.2749703 | -0.2418785 | -0.1750165 |
| -0.5384170 | -0.9181532 | -1.1627931 | -0.9255222 | -0.3653572 | -0.7633120 | -0.6153956 | -0.4621348 | -1.0758071 |
| 0.6745756 | -0.2921448 | 0.7934704 | 1.2982385 | -0.5694614 | 0.4066000 | 1.7831787 | -0.1407541 | 0.6707277 |
| -0.4246271 | 0.2146417 | 4.0296319 | 0.4518030 | -0.4333467 | 2.4073249 | 0.4282646 | 2.4324321 | 2.9537773 |
| -0.2036189 | -0.8744862 | -0.3300072 | -0.2833924 | -0.6400745 | -0.2173724 | 0.1416339 | -0.9512842 | -0.4746966 |
| -1.0703537 | -0.7898291 | -0.9114311 | -1.2753261 | -0.6050987 | -1.0671279 | -1.3535686 | -0.7153852 | -1.4537287 |
| 1.3898954 | 0.8384517 | 2.6786045 | 2.1291383 | -0.0751811 | 2.4340753 | 1.2883301 | 2.0720144 | 2.9042358 |
| 1.8743927 | 0.4198369 | 2.3099201 | 2.3070860 | -0.5820040 | 2.2871605 | 1.8872470 | 0.5666474 | 3.4120294 |
| -0.3027280 | -0.5304593 | -0.5040073 | -0.4360012 | -0.7757836 | -0.9083264 | -1.4554159 | -0.7810225 | -0.8338094 |
| -1.2049327 | -0.8826732 | -2.0735298 | -2.3102085 | -0.5444449 | -1.2310707 | -1.4548960 | -0.8699322 | -2.4044170 |
| -0.4582156 | -0.3933772 | -0.1711772 | -0.4760321 | -0.5656131 | -0.8444381 | -0.4363166 | -0.7266227 | -1.3195678 |
| -1.7022975 | -0.2884737 | -0.6261198 | -2.4805024 | -0.5536834 | -0.8259909 | -1.3959891 | -0.6327216 | -1.3082877 |
| -0.8053732 | -0.3812960 | -0.9094663 | -0.8860792 | -0.1801222 | -0.5310245 | -0.5528373 | -0.3536549 | -0.9648928 |
| -1.3573072 | -0.3797614 | -1.1516373 | -0.6352484 | -0.1036287 | -0.2865012 | -0.7334115 | -0.1910583 | -0.3895752 |
| 0.1158532 | -0.1303683 | -0.3900303 | -0.4777255 | 0.0374221 | -0.0365904 | -0.2438390 | 0.1413874 | -0.0473218 |
| -0.4736282 | -0.5022365 | -0.1071983 | -0.3469807 | -0.6230172 | -0.5768973 | -0.6912209 | -0.5134227 | -0.2600943 |
| -0.7845403 | -0.7693006 | -0.8453333 | -0.9683534 | -1.0489738 | -0.6801487 | -0.6071165 | -1.0326352 | -0.8105888 |
| 0.3076922 | -0.2650675 | -0.4732620 | -0.1058541 | -0.0327100 | -0.2501670 | -0.2109283 | -0.0563305 | -0.5004230 |
| 0.4736318 | 0.3245718 | 0.4332049 | 0.2624737 | 0.4908076 | 0.6871413 | 1.1412132 | 0.2427079 | 0.8099532 |
| -1.5283266 | -0.5908791 | -1.1119819 | -1.2153633 | -0.7525207 | -1.1680342 | -1.9709001 | -0.8645078 | -1.5554290 |
| -0.5436246 | -0.2271169 | -0.8347461 | -0.6149479 | 0.1845353 | -0.2884321 | -0.2880748 | -0.0435010 | -0.7135730 |
| -2.0448571 | -0.7219685 | -2.2679394 | -2.4065625 | -1.0823151 | -2.3983741 | -2.4223686 | -1.8294152 | -3.0211591 |
| 4.0008847 | 1.0195064 | 2.2878779 | 4.6226745 | 0.3952268 | 2.3170675 | 3.7253137 | 0.6893297 | 2.6934288 |
| 0.5242192 | -0.2501207 | 0.5196208 | 2.4016063 | -0.3164846 | 0.2213209 | 1.6642075 | -0.1379953 | -0.0133002 |
| -1.1032893 | -0.4480713 | -0.8864048 | -1.7844813 | -0.1941454 | -0.4755477 | -1.1900597 | -0.3504000 | -0.8414005 |
| -0.6407510 | -0.4176946 | -0.2193105 | -0.1936752 | 0.0138576 | 0.0755201 | 0.0510950 | 0.0017117 | -0.1410494 |
| 0.1609562 | -0.1683842 | -0.1265482 | 0.0055744 | 0.1579181 | -0.1364573 | -0.1126415 | -0.0073133 | -0.4204515 |
| -0.9825576 | -0.7980274 | -1.2736769 | -0.9952727 | -0.5041046 | -0.9444392 | -1.1864637 | -0.7409544 | -1.2897014 |
| -1.7180690 | -0.3956936 | -0.938174 | -2.3776328 | -0.3978324 | -0.4089073 | -0.8718786 | -0.2571081 | -0.3053971 |
| 1.5055917 | -0.1353061 | 0.3791221 | 1.7305621 | -0.1706196 | 1.4219413 | 2.1857545 | 0.2508341 | 1.6144171 |
| 0.4674897 | 0.5757994 | 0.6248096 | 0.1222166 | 0.1758505 | 0.0469294 | -0.9032620 | -0.0096256 | 0.0081223 |
| -0.6184533 | -0.0550065 | -0.2238804 | -0.3696974 | 0.3952280 | 0.1067864 | -0.7083827 | 0.1412006 | -0.3380330 |
| -0.9480854 | -0.2187768 | -0.7389127 | -1.6818597 | -0.1412358 | -0.1116581 | -0.8930306 | -0.2923707 | -0.3039751 |
| -1.4255458 | -0.2229500 | -1.8161427 | -1.4206600 | -0.944478 | -0.9518578 | -1.8485209 | -0.9094106 | -1.5499616 |
| -1.3325954 | -0.5698166 | -1.5211084 | -0.9381389 | -0.4533751 | -0.9927022 | -1.5867627 | -0.5815404 | -1.4805779 |
| -0.6273795 | -0.8684628 | -0.6843067 | -0.5216473 | -0.6881870 | -0.6146289 | -1.1359322 | -0.6945985 | -0.9280982 |
| -1.5424162 | -1.6002527 | -2.5330059 | -1.7014594 | 0.0417151 | -1.6234565 | -1.2364748 | -0.5936940 | -2.5544968 |
| 1.9994504 | 0.7094411 | 1.1940748 | 1.6225418 | -0.2947026 | 2.6555554 | 4.3377326 | 0.9073759 | 3.6422548 |
| -0.0022234 | -0.1665621 | -0.0376584 | 0.1405194 | -0.0244036 | -0.0043132 | -0.0748652 | -0.0129862 | 0.0191697 |
| -0.3028037 | -0.0542647 | -0.0474762 | -0.2230853 | -0.0113644 | -0.2628155 | -0.5103862 | -0.1717437 | -0.2297507 |
| -1.0119113 | -0.7202306 | -0.7988383 | -0.4070588 | -0.9874588 | -2.3130262 | -0.7020855 | -1.8807100 | -2.5634537 |
| 0.3786492 | 1.1863896 | 7.1030074 | 2.6799325 | -0.1855614 | 5.8741221 | 1.8021890 | 4.2778557 | 8.0489461 |
| 1.2623830 | -0.0398360 | 1.9555555 | 1.6637081 | 0.0324567 | 1.2268628 | 0.5706857 | 0.4026667 | 2.0989962 |
| -0.0674646 | 0.3395329 | -0.2079235 | -0.3674781 | 0.1837633 | -0.1163233 | -0.6069428 | 0.1619069 | -0.4587158 |
| -0.7908013 | -0.4656208 | -0.0072803 | -0.3887743 | -0.4256297 | -0.4662600 | -0.1548902 | -0.4016396 | -0.4013543 |

| | | | | | | | | |
|---|---|---|---|---|---|---|---|---|
| -3.585232 | 0.0744283 | -1.015548 | -3.566034 | -1.883406 | -2.001173 | -1.618388 | -1.841761 | -2.072302 |
| 0.4795095 | -0.153225 | -0.078910 | 0.6285137 | -0.377727 | -0.182359 | -0.089700 | -0.387215 | 0.0329153 |
| -0.470069 | -0.541125 | -0.660547 | -0.503055 | -0.437630 | -0.572729 | -0.211062 | -0.276619 | -1.167474 |
| -0.101011 | -0.190233 | -0.255805 | 0.1546389 | 0.0779257 | 0.1832955 | -0.233267 | 0.0900772 | 0.1538178 |
| -1.209574 | -0.480939 | -1.324275 | -1.454606 | -0.653511 | -0.984036 | -1.387868 | -1.005284 | -1.787845 |
| -1.448387 | -0.302380 | -0.624343 | -0.537333 | -0.803160 | -0.481815 | 0.9847632 | -0.625243 | 0.4163847 |
| -0.490023 | -0.625548 | -0.491515 | -0.891720 | -0.645610 | -0.633956 | -0.406492 | -0.301152 | -0.657908 |
| -0.074899 | -0.183779 | 0.1520824 | 0.0603977 | -0.124720 | -0.016193 | 0.1992202 | -0.218600 | 0.3371501 |
| -0.350976 | -0.133114 | -0.142487 | -0.528733 | -0.127361 | -0.025927 | -0.095345 | -0.370166 | 0.1792578 |
| 0.5309475 | -0.299742 | 0.1612253 | 0.7131951 | 0.1366052 | 0.6557414 | 0.7616508 | 0.5610799 | 0.7295835 |
| -0.572722 | 0.0458774 | -0.100633 | -0.687442 | -0.604208 | -0.692411 | -1.140461 | -0.653747 | -0.677199 |
| -0.228755 | -0.800372 | -1.214879 | -0.900261 | -0.680007 | -0.857194 | -0.312066 | -1.467805 | -0.923605 |
| 0.1487026 | -0.697679 | -1.293739 | -1.414794 | -0.808577 | -1.136385 | -1.540200 | -0.957921 | -1.810814 |
| -0.699124 | -0.791941 | -1.232577 | -0.942537 | -1.183586 | -1.193573 | -1.185352 | -1.226355 | -1.979589 |
| -0.384557 | -0.121302 | -0.371722 | -0.088245 | -0.364573 | -0.067531 | -0.099955 | -0.329095 | -0.434299 |
| 0.0639962 | 0.0190518 | -0.198049 | 0.2430465 | -0.098755 | -0.176216 | -0.083589 | -0.012782 | -0.185665 |
| -0.984589 | -1.045498 | -1.532882 | -1.384981 | -0.644476 | -1.108730 | -1.271882 | -0.622035 | -1.942619 |
| -0.315161 | -0.017636 | -0.079776 | -0.269476 | -0.488396 | -0.393259 | -0.131468 | -0.189058 | -0.328346 |
| -0.147918 | -0.680245 | -0.769542 | -0.539889 | -0.102379 | -0.578632 | 0.2262839 | -0.423046 | -1.247156 |
| 1.0210567 | -0.206138 | 0.3082602 | 1.3828376 | -0.254502 | 0.0032611 | 0.9196687 | -0.419277 | 0.4163365 |
| -0.319270 | -0.309045 | -0.142248 | -0.884648 | -0.525552 | -0.798239 | -1.026288 | -0.673470 | -1.146013 |
| -0.062214 | -0.203812 | -0.312381 | -0.014598 | -0.371937 | -0.545053 | 0.1877317 | -0.572514 | -0.584086 |
| -0.138320 | -0.001581 | -0.274211 | -0.013623 | 0.0002228 | -0.039766 | -0.038651 | -0.084122 | -0.168834 |
| -1.010880 | -0.396143 | -0.638977 | -1.634613 | -0.115146 | -0.236352 | -0.767575 | -0.138606 | -0.493582 |
| -0.470971 | -0.342647 | -0.499288 | -0.375190 | -0.777888 | -0.652754 | -0.670132 | -0.958754 | -0.667743 |
| -0.479051 | -0.287015 | -0.537259 | -0.358654 | -0.052227 | -0.210004 | -0.374238 | -0.245586 | -0.426250 |
| 0.2940848 | -0.189609 | -0.146929 | 0.0724864 | -0.013871 | -0.136574 | 0.0823731 | -0.227072 | -0.206956 |
| -0.526414 | -0.398095 | -0.312894 | -0.199312 | -0.260626 | -0.014650 | 0.2747657 | 0.0863215 | 0.2007740 |
| -0.186783 | -0.212580 | -0.504834 | -0.262931 | -0.329273 | -0.866434 | 0.0506643 | -0.498543 | -1.261262 |
| -0.450219 | -0.546653 | -0.080453 | 0.4500176 | -0.890077 | -0.187505 | -0.845596 | -0.345133 | -0.055018 |
| -0.954405 | -0.920284 | -0.608982 | -1.033485 | -0.021257 | -1.153083 | -0.387609 | -0.082056 | -1.161706 |
| -0.390010 | -0.221877 | -0.338102 | -1.017602 | -0.459086 | -0.352870 | -1.127152 | -0.568708 | -0.746933 |
| -0.379183 | -0.267679 | -0.235399 | -0.222070 | -0.907307 | -1.508294 | -0.699890 | -0.947642 | -1.353478 |
| -0.180003 | 0.1320603 | 0.0355035 | -0.672381 | 0.3973026 | 0.2855749 | 0.3556752 | 0.3334054 | 0.499771 |
| -1.853053 | -0.297565 | -0.653801 | -2.591543 | -0.064069 | -0.313231 | -1.374410 | -0.228273 | -0.392436 |
| -0.948606 | -0.626369 | -0.892527 | -0.889969 | -0.082736 | -0.235715 | -0.391426 | -0.257864 | -0.665494 |
| -0.222824 | -0.044131 | -0.000894 | -0.327767 | 0.0967073 | -0.045597 | 0.1728184 | -0.001137 | -0.107915 |
| -0.076355 | -0.202986 | -0.091010 | 0.0082556 | -0.236578 | -0.105535 | -0.513614 | -0.202535 | -0.187883 |
| -0.771912 | -1.000273 | -0.835730 | -1.089908 | -0.697150 | -0.854929 | -0.764854 | -0.704078 | -0.930492 |
| -0.368827 | -0.070246 | -0.185443 | 0.0401386 | -1.005960 | -1.090521 | -1.623565 | -1.067681 | -0.944216 |
| 0.2821371 | -0.543308 | -0.210736 | 0.0777314 | 0.4678103 | 0.1219645 | 0.2212937 | 0.0317905 | 0.2846003 |
| -0.087163 | -0.094763 | -0.279118 | 0.1045395 | -0.290042 | -0.404267 | -0.652347 | -0.224567 | -0.158079 |
| -0.003728 | 0.0059644 | -0.107750 | -0.529130 | 0.1494877 | 0.1103858 | -0.163526 | 0.0953213 | -0.118645 |
| -0.626527 | -0.338510 | -0.141423 | -0.907680 | -0.534354 | -0.104292 | 0.0445746 | -0.231517 | -0.114493 |
| -0.084986 | -0.090588 | -0.034450 | -0.178592 | -0.590487 | -0.777261 | -2.157151 | -0.479392 | -0.954162 |
| -0.025146 | -0.506556 | 0.2447211 | 0.5080816 | -0.258737 | 0.1287972 | 0.1656053 | 0.1092070 | 0.2618702 |
| -0.484694 | -0.517156 | -0.325842 | -0.636915 | -0.181938 | -0.398673 | -0.742407 | -0.345923 | -0.721621 |
| 0.1097273 | -0.485935 | -0.026143 | 0.5828086 | -0.100644 | 0.1515439 | 0.4313820 | 0.0702683 | 0.2864015 |
| -1.128522 | -0.597274 | -1.238636 | -1.121588 | -0.720339 | -2.229975 | -2.284064 | -0.971760 | -3.151209 |
| -0.925945 | -0.761605 | -0.632256 | -1.861516 | -0.784768 | -0.472339 | -1.404721 | -0.614138 | -0.797509 |

| | | | | | | | | |
|---|---|---|---|---|---|---|---|---|
| 0.3917081 | 0.2289755 | 0.0968012 | 0.6020049 | 0.2709152 | 0.2309565 | 0.1703914 | 0.0562408 | 0.3324879 |
| -1.3339338 | -1.2267103 | -1.6505796 | -1.9723764 | -0.7340954 | -0.7655990 | -0.6841448 | -0.4821875 | -1.5344324 |
| -0.3557654 | -0.7008391 | -0.6653181 | -0.2419376 | -0.4879812 | -0.8998655 | -1.0066374 | -0.5201507 | -1.0462774 |
| -0.3174776 | -0.0039366 | 0.2609764 | -0.1048722 | 0.0937918 | 0.6705776 | 0.8639650 | 0.3403186 | 1.4565702 |
| -0.9467395 | -0.2040096 | -0.5777563 | -1.0429910 | -0.2958151 | -0.7341259 | -1.2333134 | -0.7061902 | -1.1378064 |
| -0.1969713 | -0.2976915 | -0.2827358 | -0.0499180 | 0.0312222 | 1.4421726 | 1.6441618 | 0.1564728 | 1.5914526 |
| -0.8112883 | -0.3658637 | -0.5380872 | -0.9214493 | -0.2094939 | -0.4207134 | -0.4609672 | -0.2961291 | -0.5811145 |
| -1.0676528 | -1.0146769 | -1.3232219 | -1.7287287 | -0.3481920 | -0.4015849 | -0.4805028 | -0.7824955 | -1.3710239 |
| -1.8966168 | -0.2385256 | -0.9334661 | -2.0392616 | -0.4963085 | -0.8873208 | -1.6159890 | -0.5661734 | -1.3814658 |
| -0.0406385 | -0.4797887 | -0.3319071 | 0.0077877 | -0.7075709 | -0.7510802 | -0.2130106 | -0.8211200 | -0.7673474 |
| 0.3115616 | -0.2870850 | 0.0174609 | 0.9267781 | -0.2224903 | -0.0212488 | -0.1353278 | 0.0154115 | 0.0772326 |
| -0.7481013 | -0.7801410 | -0.9469784 | -0.8100544 | -0.2908239 | -0.4383015 | -1.0624464 | -0.5306427 | -0.8456025 |
| -0.0037823 | -0.2479944 | -0.5583921 | -0.3452110 | -0.5245605 | -1.5304771 | -0.2335337 | -0.5700886 | -1.4917415 |
| -0.2407467 | -0.3344697 | -0.0939311 | -0.3636429 | -0.1051649 | -0.6601083 | -0.4602499 | -0.135887 | -0.5858688 |
| -0.5146083 | -0.2460605 | -0.2783447 | -0.3448016 | -0.4372688 | -0.2192380 | -0.3701701 | -0.5108571 | -0.2634160 |
| -0.1223544 | 0.3699251 | 0.7645481 | 0.0496527 | 0.5333422 | 0.5434470 | -0.3381576 | 0.7289035 | 0.4715252 |
| -0.0446807 | -0.7978732 | -0.3243757 | -0.0646237 | -0.5468056 | -0.4625513 | -0.6114233 | -0.0588315 | -0.4054564 |
| -1.9215474 | -1.2508482 | -2.3034255 | -2.0565856 | -0.3327371 | -0.7651149 | -0.9609444 | -0.8130844 | -2.1567185 |
| -0.6483192 | -0.7336919 | -0.4507879 | 0.2466221 | -0.6019753 | -0.6044371 | -0.7006976 | -0.6639965 | -0.6407237 |
| -1.1085015 | -0.3763901 | -0.7403314 | -1.2626453 | -0.4510155 | -0.8982922 | -0.7693719 | -0.8400248 | -1.2009169 |
| 0.2395459 | -0.1765738 | -0.0240539 | -0.1486666 | -0.1279286 | 0.0797927 | 0.2462736 | -0.1522814 | 0.8260557 |
| 0.3781781 | 0.0828859 | -0.3607773 | 0.5784433 | 0.0482230 | 0.0436676 | 0.1829467 | -0.0587558 | 0.0981415 |
| -1.3908481 | -0.3667147 | -0.8948432 | -2.1508054 | -0.5899090 | -0.8845722 | -1.0263251 | -0.5532834 | -1.1764128 |
| -0.6735467 | -0.8028454 | -0.7226238 | -0.6428393 | -1.0052748 | -0.5871701 | -0.9702598 | -0.5981220 | -0.7304785 |
| -1.0153986 | -0.4279990 | -1.1011855 | -1.6520523 | -0.3768159 | -0.8630338 | -0.7896248 | -0.5760566 | -1.0295309 |
| -0.6104316 | -0.5286971 | -0.7496435 | -0.3373653 | -1.2225451 | -0.8207682 | -0.1515790 | -0.8167101 | -1.6665167 |
| -1.0817050 | -0.6666242 | -0.9258817 | -1.3903624 | -0.7679459 | -1.0860663 | -0.9080135 | -0.7406652 | -1.6773981 |
| -0.8499363 | -0.6628764 | -1.1132789 | 0.3613275 | -0.3649386 | -0.7154119 | -0.4612823 | -0.6476770 | -0.9309723 |
| -0.7891249 | -0.6267007 | -0.8407405 | -0.9491543 | -0.5961783 | -1.0930347 | -0.8717863 | -0.6535724 | -0.9848008 |
| 0.2555172 | -0.1730092 | 0.1713062 | 1.0607683 | -0.3983485 | -0.0937451 | 0.0823873 | -0.0624838 | 0.0467701 |
| 0.9535762 | -0.1832706 | 0.9173131 | 2.2668635 | -0.4527144 | 0.3228537 | 1.5033156 | 0.0150654 | 1.2456095 |
| -1.5315176 | -1.2615189 | -1.9390291 | -0.6667164 | -0.2064266 | -1.0389245 | -1.0513917 | -0.4194351 | -1.4059839 |
| 0.1118708 | -0.1490579 | -0.1153490 | -0.0173638 | 0.1265729 | 0.1937433 | 0.3357471 | 0.1354784 | 0.1500197 |
| 0.3317316 | 0.0129971 | 0.1656911 | 0.1914762 | 0.0987109 | 0.2768567 | 0.5712489 | 0.0666796 | 0.4681116 |
| 0.8325845 | -0.0439117 | 0.3377640 | 0.2722050 | 0.4875155 | 0.9016922 | 0.8459780 | 0.5943478 | 0.8355371 |
| -0.6406146 | -0.8510169 | -1.1679296 | -0.8136846 | -0.5324582 | -0.6907831 | -1.2763678 | -0.2824801 | -0.7651927 |
| 1.2219794 | 0.1225822 | 0.4367524 | 2.2383444 | 0.7188873 | 0.3676223 | 1.1955190 | 0.0667376 | 0.8758960 |
| -0.4732099 | -0.1718526 | 0.0916797 | 0.2883491 | -0.2389948 | -0.0969254 | -0.3790291 | -0.1001103 | 0.4550068 |
| -0.0086536 | -0.4379150 | -0.3743887 | 0.6896552 | -0.4078799 | -0.1394093 | -0.2002828 | -0.2887591 | -0.1396205 |
| -0.0558284 | -0.0863507 | -0.3329396 | -0.1539174 | -0.2639217 | -0.4201115 | -0.0797281 | -0.2795986 | -0.6060062 |
| -0.3647130 | -0.2816797 | -0.3904203 | -1.5098899 | -1.2060624 | -1.7090928 | -1.0395869 | -1.2146448 | -2.7681311 |
| 0.1377417 | -0.5955270 | -0.2395711 | 0.2615319 | -0.2328537 | -0.0958492 | 0.3086314 | -0.0560271 | 0.4040596 |
| 0.8534822 | -0.1336489 | 0.2446994 | 1.4397532 | -0.3116921 | -0.0643176 | 1.2771937 | -0.0784945 | -0.0398795 |
| 0.2886966 | -0.2871220 | -0.0152846 | 0.5963388 | -0.0631117 | 0.4076227 | 0.3497227 | 0.1589417 | 0.5240803 |
| -0.6253044 | -0.2462067 | -0.4197384 | -0.4203266 | -0.1620482 | -0.2636071 | -0.3540844 | -0.1964284 | -0.4585822 |
| -0.1332521 | -0.4432838 | -0.6658029 | 0.0491828 | 0.1025744 | -0.0693539 | -0.2425072 | -0.0946775 | -0.3900881 |
| -1.2246841 | -0.5852738 | -0.9780392 | -1.9893151 | -0.8905652 | -1.2918373 | -1.2642339 | -1.0282920 | -1.6219677 |
| -1.2583921 | -0.3845553 | -1.0713877 | -1.8228671 | -0.4254043 | -0.9501587 | -1.3598289 | -0.7783759 | -1.5467090 |
| -1.2610781 | 0.0341904 | -0.4218885 | -1.9076806 | -0.7847723 | -1.1405080 | -1.7506504 | -0.9611984 | -1.3859313 |
| 0.0585171 | -0.2489342 | -0.2888430 | -0.0153810 | -0.1207889 | -0.0214893 | 0.3957333 | -0.1989406 | -0.0527744 |

| | | | | | | | | |
|---|---|---|---|---|---|---|---|---|
| 0.4313906 | 0.0520199 | -0.0005628 | 0.8796020 | -0.3097743 | -0.4602388 | 0.1978675 | -0.3830797 | -0.4434582 |
| -1.8862444 | -0.6047545 | -0.4020389 | -1.8184475 | -0.1478874 | -0.4390655 | -1.9469840 | -0.2625480 | -0.1803514 |
| -0.7042826 | -0.7732045 | -0.5388197 | -0.3016905 | -0.9448606 | -0.7389663 | -0.6705390 | -0.9562397 | -0.6061401 |
| -1.3838752 | -0.5443940 | -1.4962385 | -2.4665579 | -1.0306736 | -1.6946316 | -2.0281169 | -1.2216218 | -2.7368140 |
| -0.7992310 | -0.4099017 | -1.1118686 | -1.2241046 | -0.4790352 | -0.8330954 | -0.8774903 | -0.6012963 | -0.8034930 |
| -0.5465504 | -0.2650684 | -0.3665446 | -1.0338963 | -0.5853775 | -0.4312868 | -0.2300050 | -0.5152124 | -0.3384929 |
| 2.5052581 | 0.0871282 | 0.7177913 | 2.2386444 | 0.0526486 | 1.3773214 | 2.7215684 | 0.3084886 | 1.6673935 |
| -0.2828636 | 0.0541893 | -0.7353699 | -0.0868528 | 0.1274693 | -0.4857049 | -0.3158302 | -0.2793695 | -0.6654670 |
| -0.626211 | -0.3839101 | -0.4221325 | -1.3315544 | 0.3156204 | 0.3692088 | -0.3440022 | 0.5791811 | -0.1345724 |
| -0.4886471 | -0.6140703 | -0.5125779 | -0.8474828 | -0.1445564 | -0.2634563 | 0.8492349 | -0.0950568 | -0.3007652 |
| 0.1631700 | 0.2772079 | 0.3187769 | -0.3090905 | -0.4477746 | -0.3508389 | 0.1666857 | -0.6844365 | -0.4420590 |
| -0.3096072 | -0.8032052 | -0.1400984 | 0.3992944 | -0.7526724 | -0.6313126 | -0.2915497 | -0.7222674 | -0.5909493 |
| 0.0082570 | -0.0156698 | -0.0600202 | -0.0485891 | -0.3738077 | -0.5179241 | -0.1845559 | 0.0051659 | -0.0581919 |
| -0.0313615 | 0.0259805 | -0.2034003 | -0.2617219 | -0.2188445 | -0.4576833 | -0.5691558 | -0.4978457 | -0.6751734 |
| -1.3720472 | -0.1321818 | -0.3476994 | -1.7228288 | -0.5236094 | -0.5541251 | -1.4654469 | -0.5402278 | -0.8687223 |
| -0.0356084 | 0.0253122 | -0.1344902 | -0.5213897 | -0.6722601 | -0.8173304 | -0.9511564 | -0.6199658 | -1.0079839 |
| -1.2605372 | -0.1377611 | -0.4139877 | -2.0540700 | -0.2794801 | -0.2714733 | -1.0555798 | -0.4343900 | -0.1948362 |
| 0.3617155 | -0.3660517 | 0.0754832 | 0.5303276 | -0.0302378 | -0.2989968 | 0.1006803 | -0.1904743 | -0.1681653 |
| -2.0360956 | -0.4366712 | -1.1294535 | -2.1791678 | -0.0354366 | -0.4488978 | -1.1992312 | -0.1380047 | -1.2551252 |
| 0.0093730 | 0.1538650 | 0.0296936 | -0.1286855 | 0.0586771 | 0.1290620 | 0.5164756 | 0.1546663 | 0.2319182 |
| -1.4687590 | -0.8439288 | -1.3915973 | -1.5699476 | -0.9605613 | -0.6961988 | -0.8628451 | -1.0100933 | -0.7313306 |
| -0.2040556 | -0.1838150 | -0.2706161 | -0.2262044 | -0.2424554 | -0.3019276 | -0.7783006 | -0.2943973 | -0.4116393 |
| -2.7168142 | -0.5014339 | -1.4793266 | -3.1985145 | -0.7740884 | -1.5229188 | -2.3814383 | -0.9245140 | -2.4147611 |
| 0.1479407 | -0.2259649 | -0.1329014 | 0.3002670 | -0.0591897 | 0.0507484 | 0.1546190 | -0.0101443 | 0.2677146 |
| -0.8393239 | -0.4923418 | -0.5036302 | -1.6693984 | -0.2081890 | -0.4893867 | -0.7567376 | -0.0891292 | -0.8237491 |
| -0.4437390 | -0.5390853 | -0.7719373 | -0.4359835 | -0.1400457 | -0.2366114 | -0.5070418 | -0.1364920 | -0.3526904 |
| -0.6677043 | -0.3214642 | -0.4505875 | -0.5121347 | -0.0764532 | -0.2919486 | -1.2581938 | -0.1043211 | -0.3510080 |
| -1.0103910 | -0.7554224 | -1.1143434 | -1.1392272 | -1.0084146 | -1.0508837 | -1.6415037 | -0.7358031 | -1.5958870 |
| 0.5863712 | -0.2676869 | 0.0383149 | 1.6814067 | -0.2749663 | -0.2432093 | -0.0447912 | -0.0309010 | 0.0866404 |
| 0.9616362 | 0.0754911 | 0.418194 | 1.0994861 | -0.0798257 | 0.0366356 | 0.7755466 | -0.1839555 | 0.2137548 |
| -1.0592369 | -0.5952359 | -0.1680674 | -0.9538003 | -0.7948148 | -0.4397197 | -0.6334810 | -0.7129777 | -0.6827809 |
| -0.2418956 | -0.1592712 | -0.2145796 | -0.2589997 | -0.3359462 | -0.6147722 | -0.3194401 | -0.4870588 | -0.6915228 |
| -0.1692900 | -0.7648447 | -0.3821500 | -0.3119034 | -0.8236450 | -0.4949834 | 2.1214702 | -0.7415962 | -0.3737769 |
| -1.3575591 | -0.3284006 | -0.6397466 | -1.8368713 | -0.2801798 | -0.3162197 | -0.8834276 | -0.2361594 | -0.6559860 |
| -0.4655231 | -0.1717767 | -0.4845589 | -0.8016654 | -0.1065421 | -0.3763235 | -0.0619203 | -0.0312042 | -0.6098962 |
| -0.5811744 | -0.2551365 | -1.1650274 | -1.1413887 | -0.5842828 | -0.5052706 | -0.9921067 | -0.4587886 | -1.0616582 |
| -1.3533191 | -1.2076755 | -2.0690270 | -2.1372026 | -0.6333561 | -1.3144117 | -1.6936804 | -1.1517478 | -2.3608782 |
| -0.2322006 | -0.0801043 | -0.2109237 | -0.1120520 | 0.0756069 | -0.3794921 | -0.1457972 | -0.0717266 | -0.2359478 |
| 0.1713028 | -0.5349864 | -0.0342508 | -0.3780916 | -0.4682147 | 1.0010506 | 0.5215015 | -0.3342001 | 1.0560009 |
| -0.8696089 | -0.3487602 | -0.5955028 | -1.8404217 | -0.2986691 | -0.2294126 | -0.5834988 | -0.3096230 | -0.3789332 |
| 0.8887185 | 0.0727048 | 0.3098827 | 1.1427511 | -0.3198966 | -0.2283201 | 0.2939047 | -0.6853767 | -0.3425120 |
| -1.1716703 | -0.2546600 | -1.1726357 | -1.1132531 | -0.5832273 | -1.0743893 | -0.8191300 | -0.5598893 | -2.0393342 |
| -0.4973986 | -0.5112705 | -0.9558985 | -0.8415075 | -0.6852735 | -0.9954510 | -0.9626125 | -0.8434425 | -1.4625964 |
| 0.0962475 | 0.2311166 | -0.2038512 | -0.2528568 | -0.8675737 | -0.6186888 | -0.2666841 | -0.3393781 | -0.0227566 |
| -0.5275484 | -0.7801500 | -0.6580393 | -0.3493001 | -0.4407281 | -0.8192868 | -0.5016970 | -0.4071988 | -0.4882679 |
| -0.8908345 | -0.4834080 | -1.1054727 | -1.0716502 | -0.4231671 | -0.5469257 | -0.3683794 | -0.6978111 | -0.8800198 |
| -0.3766064 | -0.1999868 | -0.4626877 | -0.9038026 | -0.1607297 | -0.2125769 | 0.0037237 | -0.4291601 | -0.1683414 |
| -1.5889941 | -1.2650084 | -1.2919314 | -2.1429526 | -1.0103392 | -1.0109912 | -0.6086537 | -1.1656342 | -0.8878030 |
| -0.6704611 | -0.5278120 | -1.0402777 | 0.1378936 | 0.0255875 | -0.1649589 | -0.0242959 | -0.0805508 | -0.0218629 |
| -1.6928967 | -1.3514025 | -1.4250532 | -1.9638784 | -0.9678638 | -1.2347542 | -1.9013667 | -0.9593793 | -1.6021252 |

| | | | | | | | | |
|---|---|---|---|---|---|---|---|---|
| -0.2557207 | -0.3966875 | -0.6603176 | 0.2141105 | -0.1590370 | -0.0885978 | 0.5832301 | -0.1339739 | 0.3150171 |
| -0.3230551 | -0.3638110 | -0.2355580 | 0.6514806 | -0.1862664 | -0.6765534 | -0.2939272 | -0.2572769 | -0.7268082 |
| -0.0585147 | -0.3148781 | -0.3404345 | 0.0760676 | 0.0057604 | 0.0654772 | 0.2075612 | 0.0055240 | 0.0287539 |

| PG_525 | PG_526 | PG_527 | PG_528 | PG_529 | PG_530 | PG_531 | PG_532 | PG_533 |
|---|---|---|---|---|---|---|---|---|
| -0.328685 | -1.155930 | -1.597422 | -1.382881 | -1.744101 | -1.044806 | -0.182435 | -0.804454 | -1.757614 |
| -0.984663 | -0.314490 | -0.463492 | -1.426552 | -0.468308 | -0.626254 | -0.441222 | -1.196690 | -1.233567 |
| -3.607306 | -0.374887 | -0.117750 | 0.2744168 | -0.799364 | -0.435849 | 0.2705036 | -1.056741 | -1.829627 |
| -0.149791 | -0.109254 | -0.629792 | -0.320610 | -0.191890 | 0.0758127 | -0.539852 | -0.316262 | -0.352402 |
| -1.174920 | 0.0338405 | -0.633783 | -1.096702 | -0.098054 | -0.595226 | -0.809112 | -0.168191 | -0.638462 |
| -1.938604 | 0.1291316 | -0.542098 | -1.232006 | -0.000830 | -0.211488 | -0.375677 | -0.845859 | -1.060674 |
| 0.5651427 | -0.563074 | -1.390163 | -1.018994 | -0.715681 | -0.390042 | -0.428204 | -0.687867 | -0.543011 |
| -2.645803 | -0.057466 | -0.145824 | -1.201086 | -0.209579 | -0.465010 | -0.670636 | -0.736045 | -2.228306 |
| -0.320498 | -0.486596 | -0.358121 | -0.849036 | -0.568878 | -0.062327 | 0.2394658 | -0.168018 | 0.4142451 |
| -1.295941 | -0.596479 | -1.193883 | -1.413988 | -0.967089 | -0.918317 | -0.787322 | -0.869705 | -1.604412 |
| -1.368010 | -0.252174 | -0.594224 | -0.930328 | -0.397021 | -0.233116 | -0.664217 | -0.494526 | -1.014945 |
| -0.830649 | 0.0181544 | -0.797641 | -0.556184 | 0.0690780 | -0.257559 | -0.381966 | -0.383786 | -0.525635 |
| -1.740560 | -0.468741 | -1.273250 | -1.817182 | -0.554631 | -0.326368 | -0.247631 | -0.790766 | -1.475881 |
| 4.3996129 | -0.193151 | 3.9133418 | 3.7177524 | 1.0071810 | 1.8592560 | -0.706753 | 0.6250308 | 2.6777072 |
| 0.7031519 | -0.519414 | -0.470925 | -0.415774 | -0.698602 | 0.2893923 | -0.357644 | -0.649464 | -0.349051 |
| 1.0980759 | -0.142692 | 3.6395051 | 3.5581398 | 1.3925867 | 1.3412790 | -0.347360 | 1.6765903 | 2.2957616 |
| -1.832448 | -0.541299 | -0.271788 | -2.307161 | -0.558460 | -0.828386 | -0.422599 | -2.469412 | -1.878558 |
| 0.1482811 | -0.285145 | 0.1440525 | -0.292847 | -0.331766 | -0.368345 | -0.127724 | -0.087500 | -0.245914 |
| -2.892745 | 0.6438463 | 0.1231133 | -0.508004 | 0.4716434 | 0.0947313 | 0.3416096 | -1.420809 | -1.984066 |
| -1.047795 | -0.475029 | -0.481219 | -1.618880 | -1.097989 | -0.657504 | -0.311927 | -0.224378 | -1.548911 |
| -0.250862 | -0.153540 | -0.419812 | -0.754590 | -0.230560 | -0.009254 | -0.448360 | 0.1235680 | 0.3221166 |
| -0.704099 | -0.417762 | -0.443215 | 0.3760801 | -0.290849 | -0.557484 | -0.791344 | -1.248614 | -2.280930 |
| 1.4817219 | 0.2186066 | 0.8677559 | 0.9062088 | 0.3075206 | 0.5156022 | -0.026758 | 0.4092098 | 0.9051026 |
| -1.852544 | -0.038420 | -1.794455 | -1.436582 | -0.320950 | -0.809890 | -0.351185 | -0.687948 | -1.302734 |
| -2.451728 | -0.135660 | -1.058961 | -1.393630 | -0.561300 | -0.154737 | -0.787404 | -1.687720 | -2.175546 |
| 1.0488825 | -0.137592 | 0.4145608 | 0.6524890 | -0.088409 | 0.6185232 | 0.0620858 | 0.5005137 | 0.9245174 |
| 0.0909679 | 0.0818659 | 0.0271314 | -0.316819 | -0.026644 | 0.1394813 | -0.678377 | 0.0667006 | -0.104345 |
| -2.052252 | -0.614121 | -1.213647 | -2.220845 | -0.657967 | -1.027016 | -1.269733 | -1.378684 | -2.290535 |
| -2.177500 | -0.440953 | -1.640616 | -1.922518 | -0.655039 | -1.114657 | -0.433326 | -0.630519 | -1.891144 |
| -0.055749 | -0.229158 | -0.743270 | -1.121246 | -0.352809 | -0.412294 | -1.042288 | -0.420639 | -0.530562 |
| -2.012816 | -0.822272 | -0.000493 | 0.1730302 | -0.752744 | 0.5004802 | -1.339147 | -1.274409 | -3.413464 |
| -1.348831 | 1.0311611 | 0.6833468 | -0.768319 | 0.6721067 | -0.193579 | -0.824356 | 0.0177124 | -1.325487 |
| -1.122794 | -0.248961 | 0.4619001 | -0.695421 | 0.0018108 | -0.404181 | -0.221322 | -0.217080 | 0.0272531 |
| -3.504658 | -0.782300 | -1.728621 | -1.905089 | -0.913557 | -0.630381 | -0.532849 | -0.036035 | -1.218516 |
| -0.175462 | -0.217304 | -0.114575 | 0.2113778 | -0.194418 | -0.203372 | -1.403488 | -1.118765 | -3.053000 |
| 0.9125472 | -0.423012 | -1.123090 | -0.784342 | -0.674765 | -0.368596 | -0.049977 | -0.761070 | -0.455989 |
| -2.223793 | -0.376410 | -1.351102 | -1.825519 | -0.763695 | -0.326388 | -0.565701 | -0.630778 | -1.348056 |
| 0.3232339 | -0.075748 | 2.4445482 | 0.4070293 | 1.1864694 | 2.2298436 | -0.156459 | 2.0886692 | 0.7473844 |
| -0.100464 | -0.403242 | -0.375848 | -1.367107 | -0.513771 | -0.254357 | -0.263228 | 0.2455982 | -0.197671 |
| 2.3743257 | 0.2943985 | 0.0461173 | -0.597269 | 0.3423078 | 0.1619658 | -0.620476 | -0.902414 | -1.032102 |
| 1.3629754 | 0.2128658 | 0.2236451 | -0.009067 | 0.1310345 | -0.071904 | 0.2276772 | 0.0839717 | 0.8687747 |
| -2.653219 | -0.425978 | -0.945181 | -1.883746 | -0.384259 | -0.380517 | -0.233735 | -0.690736 | -1.615889 |
| 0.4872563 | 0.8055601 | 0.5671987 | -1.341705 | 0.7121879 | 0.4392060 | -0.258701 | -0.291630 | -1.036463 |
| -1.620402 | 0.0591109 | -0.221845 | -0.747092 | 0.0534633 | -0.468021 | -0.813197 | -0.233653 | -1.159008 |
| 1.1739207 | -0.269149 | 0.0362972 | 0.4020930 | -0.025417 | 0.3630558 | 0.2150157 | 0.1631313 | 1.2975601 |
| 5.7357289 | -0.139407 | -0.218177 | 0.7976625 | -0.655276 | 0.4793578 | -0.627561 | -0.053319 | 1.0660545 |
| -0.170690 | -0.017472 | -0.322968 | -0.548195 | -0.208648 | -0.818233 | 0.7117713 | 0.2308577 | 0.3281309 |

| | | | | | | | | |
|---|---|---|---|---|---|---|---|---|
| 0.0796328 | -0.7631262 | -0.0730360 | -0.9002111 | -0.7995527 | 0.1829367 | -0.7214060 | -0.3909075 | -0.3965142 |
| -2.4479063 | -0.2955957 | -1.2207549 | -1.5538515 | -0.4020739 | -0.6883645 | -0.1393212 | -0.3361449 | -1.4147368 |
| -0.3245956 | -0.5970380 | -0.5031762 | -0.4874195 | -0.5931366 | -0.4109383 | -0.4637931 | -0.2388131 | 0.0384807 |
| 1.6129718 | -0.3156333 | -0.4589125 | -0.0044900 | -0.5972900 | 1.8643883 | 0.2942003 | 1.6366346 | 2.1576066 |
| -0.1407068 | -0.4736657 | -0.5120846 | -0.3111965 | -0.5246628 | -0.3845448 | 0.5713167 | -0.6087052 | -0.8233446 |
| 0.0656587 | -0.3362644 | -0.8659381 | 1.0316404 | -0.3683679 | -1.7525850 | 0.0560023 | -0.0810090 | 0.8009804 |
| -0.9750039 | -0.3762760 | -1.0584060 | -1.3236448 | -0.4704899 | -0.1021508 | -0.4423817 | -1.1348884 | -1.0177579 |
| 2.1217869 | -0.1745641 | 2.9326097 | 3.1352994 | 1.6639124 | 1.1812901 | -0.0957964 | 0.6152617 | 2.2258822 |
| 0.6081585 | 0.9408125 | 2.6349153 | 0.9470143 | 2.5175950 | 1.0568603 | -1.5255483 | -0.4258899 | -1.0677244 |
| 0.0626754 | -0.3815889 | -0.2971706 | -1.1130565 | -0.5900652 | -0.3907815 | -0.8466815 | -0.5358359 | -0.2476434 |
| -1.4527553 | -1.2357808 | -1.1666907 | -1.7020281 | -2.1435762 | 0.3297014 | -0.1016636 | -0.9419582 | -1.2317498 |
| 1.8865390 | 0.3175836 | 3.8479151 | 3.2920825 | 2.8735943 | 2.0724488 | -0.3996183 | 1.8309465 | 2.3383402 |
| 2.6758891 | 0.1905493 | 2.8907875 | 2.4656781 | 1.8418712 | 2.2978974 | -0.6888102 | 1.8215631 | 1.4955563 |
| -1.4804272 | -0.3966158 | -1.3218264 | -1.6966390 | -0.3257622 | -0.3479682 | -0.2079013 | -0.7630546 | -0.4111151 |
| -2.1746062 | -1.1230887 | -1.5098223 | -2.0838636 | -1.2620233 | -0.9707877 | 0.0130148 | -0.6341160 | -1.0484051 |
| -0.1876588 | -1.1059623 | -2.2094958 | -2.1889981 | -1.4779029 | -0.4947118 | -0.5448548 | -0.3426032 | -0.3581876 |
| -1.6927370 | -0.1229588 | -1.2530442 | -1.3002220 | -0.1992870 | -0.4367341 | -0.4861461 | -0.6101170 | -1.3113003 |
| -0.7012127 | -0.3354293 | -0.7807179 | -0.8798813 | -0.3924513 | -0.1657813 | -0.2746723 | -0.4663347 | -0.7290458 |
| -0.8291326 | -0.5337616 | -1.3527157 | -1.4436334 | -0.7707708 | -0.4794711 | -0.8307990 | -0.4579407 | -0.5988386 |
| -0.2013588 | -0.1414483 | -0.0009154 | -1.5846309 | -0.1735945 | -0.0585706 | -1.2504791 | -0.4008576 | -0.8640799 |
| -0.9643887 | -0.1110880 | -0.7218312 | -0.5476042 | 0.1578326 | -1.0770929 | -0.7845296 | -0.4737581 | -1.0244421 |
| -1.3270003 | -0.6341824 | -1.2606392 | -2.5106955 | -1.0473094 | -0.5700166 | -0.3506485 | -0.6588421 | -0.5635120 |
| -0.6438165 | 0.2597522 | 0.4194241 | 0.0577016 | 0.3472983 | -0.1338681 | 0.5400962 | -0.2600622 | -0.1202296 |
| 0.5423961 | 0.4316712 | 0.6689973 | 0.2167632 | 0.4425145 | 0.4675832 | -1.3670442 | 0.7022242 | 0.3720789 |
| -2.1745242 | -0.8357889 | -1.1349604 | -1.1449211 | -1.2707161 | -0.8416630 | -0.6336904 | -1.1386505 | -2.0819842 |
| -0.3194469 | -0.3761215 | -0.2177339 | -0.9606102 | -0.3651771 | 0.4702754 | -0.1768493 | -0.7233263 | -0.3245518 |
| -2.7320030 | -0.7482594 | -1.2928802 | -0.9954199 | -0.8025494 | -0.6406375 | 0.4064579 | -1.5989486 | -2.4271069 |
| 4.0939713 | -0.5151052 | 1.6391130 | 3.7306329 | -0.0996141 | 1.0093051 | -0.2282368 | 0.6253725 | 2.7150536 |
| 2.2039002 | -0.0407269 | 1.1132598 | 1.6642850 | 0.1116912 | 0.3684050 | -0.1976459 | 0.3352654 | 1.3591874 |
| -1.1002834 | -0.6574648 | -2.5955517 | -1.8960888 | -0.8641009 | -0.1033261 | 0.1311030 | 0.0901043 | -1.0384812 |
| 0.5696586 | 0.1537081 | 0.1269120 | 0.4003879 | 0.1243638 | -0.0972350 | -0.1954253 | -0.7016837 | -0.7645181 |
| -0.1956043 | 0.0200232 | -0.0556759 | 0.4691750 | 0.0233930 | -0.6528610 | -1.2199849 | -0.0557807 | -0.3168666 |
| -1.3771742 | -0.7768860 | -1.3758326 | -1.2874753 | -1.0864248 | -0.6141278 | -0.3930627 | -0.7321901 | -1.1329635 |
| -1.4229679 | -0.3163816 | -1.0047956 | -1.5972410 | -0.5378788 | 0.0529509 | -0.8122900 | -0.6326700 | -1.1447678 |
| 2.3186678 | -0.1504302 | 0.3424553 | 0.8021962 | -0.1116627 | -0.4844300 | -0.3256675 | 0.8209984 | 2.3143445 |
| -1.1800486 | -0.8798498 | -0.4554542 | -0.3092633 | -0.7653889 | -0.6216935 | 0.0895582 | 0.1995232 | -0.7547441 |
| -0.2080447 | -0.3830248 | -0.4959665 | -0.3925443 | -0.4945933 | -0.5956159 | 0.0304894 | -0.0513960 | -1.4749903 |
| -1.2526605 | 0.1385752 | -0.9666495 | -1.4590021 | 0.0063073 | 0.2391025 | -0.4661098 | -0.6713300 | -1.0815041 |
| -1.9892521 | -0.1925092 | -0.3137634 | -1.5837970 | -0.4486151 | -0.1811928 | 0.5730514 | -0.2783420 | -2.0786606 |
| -2.3629391 | -0.6723895 | -0.9723394 | -1.1051998 | -0.5992412 | -0.6000509 | -0.7440321 | -1.7324291 | -2.6981349 |
| -1.0059565 | -0.6378355 | -0.3068806 | -1.4630123 | -0.8642411 | -0.6699720 | -0.2535213 | -0.5336206 | -0.9527269 |
| -2.7256543 | -1.3348336 | -0.8983193 | 0.0661952 | -0.8446623 | -0.8300612 | 0.8643654 | -0.0929561 | -0.5772140 |
| 2.4732566 | 1.3501290 | 3.1873330 | 3.9401499 | 2.4905642 | -0.2126796 | 1.9850796 | 3.6578693 | 3.9483166 |
| -0.3161975 | -0.0789018 | -0.2507246 | -0.7576777 | 0.0159404 | -0.1400545 | -0.0086294 | 0.0544524 | 0.0347575 |
| -0.3741328 | -0.1614031 | 0.0549355 | -0.6732464 | -0.2816797 | 0.2129666 | -0.0717767 | -0.2484986 | -0.1853315 |
| -0.2530230 | -0.6884574 | -0.5335881 | -0.0590668 | -0.9969825 | -0.9294006 | -0.5195227 | -1.5921630 | -1.4565866 |
| 5.1180714 | 0.3777585 | 7.9301785 | 8.4525266 | 5.8562992 | -0.7314991 | -0.0643224 | 3.5215867 | 6.3449719 |
| 1.0816712 | -0.1940675 | 2.4905683 | 2.2434513 | 0.4495669 | 0.9192753 | -0.2966487 | 1.0867453 | 1.3971154 |
| -1.2300692 | -0.0692044 | 1.4533763 | -1.7212956 | -0.4880695 | -0.0929445 | -2.2009428 | -1.7589405 | -1.2210755 |
| -0.6540780 | -0.8857575 | -0.2295134 | 0.7190501 | -1.1731292 | 0.1214800 | -0.7928531 | -0.8678073 | -1.1934663 |

| | | | | | | | | |
|---|---|---|---|---|---|---|---|---|
| -1.7781837 | 0.3099712 | -1.4581833 | -1.1371123 | 0.1285271 | -0.6204369 | -0.8171575 | -0.2999690 | -2.8450687 |
| 0.0871396 | -0.1578595 | 0.0580325 | 0.5230555 | -0.1456121 | 0.2597448 | -0.4177402 | -0.3291608 | 0.2200383 |
| -0.4857199 | -0.5372129 | -1.0055776 | -0.7348875 | -0.5082177 | -0.2042936 | -0.6225582 | -0.8727335 | -0.6990722 |
| 0.0471973 | -0.0441013 | 0.0699417 | -0.0922897 | -0.0528645 | -0.0312111 | -0.3167989 | -0.1653060 | -0.1613841 |
| -1.8698988 | -0.2501175 | -0.9904707 | -0.9949497 | -0.5766135 | -0.6821241 | -0.7015322 | -1.1710536 | -1.1933801 |
| 3.1153744 | -0.3052842 | -0.0459032 | -1.2471007 | 0.0952148 | -1.0828811 | -0.8475233 | -0.9829026 | -0.8428408 |
| -0.8584013 | -0.7592895 | -0.1522833 | -1.9215066 | -0.9470079 | -0.4231710 | -1.0356874 | -0.6985324 | -0.6889419 |
| -0.3000730 | -0.1931242 | 0.1170996 | -0.1191632 | 0.0157005 | -0.3348391 | 0.2184444 | -0.1295761 | 0.1349990 |
| -0.3401698 | -0.2275842 | -0.3314147 | 0.1553935 | 0.1251103 | -0.4695247 | -0.2296812 | -0.2452035 | -0.2069642 |
| 1.0609544 | 0.3059831 | 0.7328386 | 0.2316641 | 0.6178654 | -0.3392154 | -0.9142242 | -0.2502837 | 0.1930841 |
| -1.5080731 | -0.1291981 | -0.5534102 | -1.6289820 | -0.2551329 | -0.2905872 | -0.9511153 | -0.6114599 | -1.8820231 |
| -0.7375518 | -0.6293467 | -0.4529421 | -1.9625152 | -0.9819167 | 0.4132115 | -0.4321275 | -0.3801325 | -0.8581293 |
| -3.4518876 | -0.8676623 | -2.7336461 | -3.0215660 | -1.1799453 | -0.4684269 | -0.7435735 | -0.8173179 | -2.1551930 |
| -1.7262436 | -0.6687920 | -1.6627363 | -2.1716717 | -1.0552003 | -0.7610735 | -0.0132751 | -0.5849798 | -1.1863479 |
| -0.1999360 | -0.6214996 | -0.5479494 | -1.4072830 | -0.4770545 | 0.3094928 | -0.2817977 | -0.4258646 | -0.9067338 |
| -0.2385833 | -0.0652238 | -0.0792305 | -0.5056114 | -0.2203990 | -0.1616925 | -0.0757929 | -0.1413407 | 0.0358011 |
| -1.4195068 | -0.1109914 | -0.5719004 | -1.1303111 | -0.3994205 | -0.9665494 | -0.9250333 | -1.0606061 | -0.9057945 |
| -0.1393459 | -0.0345117 | -0.3471760 | -0.2737470 | -0.5759834 | -0.4466919 | 0.0712394 | -0.0544458 | 0.0529988 |
| -0.1840283 | -0.3769412 | -0.4833785 | -0.5048870 | -0.5638764 | -0.4236896 | -0.0868224 | -0.1630976 | 0.1410419 |
| 1.3410919 | 0.0947963 | 0.5953011 | 0.7741327 | -0.1905730 | 0.5871590 | -0.0303328 | 0.0907198 | 1.1658793 |
| -2.0915743 | -0.2954123 | -0.3372635 | -1.1651137 | -0.1454641 | -1.2076115 | -0.6989335 | -0.5511550 | -0.9940940 |
| -0.0413913 | 0.1703975 | -0.2649256 | -0.1678348 | 0.3535945 | -0.3005676 | -0.5315556 | -0.8387605 | -0.6578248 |
| -0.0741400 | -0.2156042 | -0.3065265 | 1.1941187 | -0.3494335 | -0.4611970 | -0.0838806 | -0.1995060 | -0.2700906 |
| -1.6020566 | -0.4803975 | -0.4758606 | -1.1733310 | -0.4985635 | -0.7530864 | 0.1347359 | -0.3344846 | -1.4829010 |
| -0.7838418 | -0.6513033 | -0.7983404 | -0.6343620 | -0.4573990 | -0.5501835 | -1.3139113 | -1.6489278 | -2.8099866 |
| -0.7200241 | -0.1389795 | -0.2107040 | -0.5581041 | -0.3159858 | 0.0519266 | -0.5796317 | -0.7803099 | -0.3748528 |
| -0.2560276 | -0.1145730 | -1.0329570 | -0.6397981 | -0.3090833 | -0.6698659 | -0.5696556 | 0.3857402 | 0.5260543 |
| 0.5687917 | -0.2339996 | -0.4933503 | 0.0577509 | -0.7938201 | -0.4010178 | 0.1157690 | 0.3491966 | 0.3164274 |
| 0.6448005 | 0.1932779 | -1.5022923 | -1.6185621 | -0.0455315 | -0.3436920 | -0.9542572 | -0.7255108 | -1.6742710 |
| -1.6305818 | -0.3484030 | -0.2654151 | -1.7206763 | -0.3900288 | -0.1147934 | -0.0807231 | -0.2457626 | -0.5394127 |
| 0.4759752 | -0.9984960 | -1.7549164 | -1.9617149 | -1.2302112 | 1.0966231 | -0.7043238 | -1.3048475 | -1.9691903 |
| -2.1514337 | -0.7776699 | -0.0154438 | -0.7869886 | -0.8726037 | 0.6655966 | -0.1608847 | -0.4176182 | -0.7805880 |
| -0.7079937 | -0.7861021 | -0.9139407 | -1.0984034 | -0.8110496 | -0.9388653 | -0.7686552 | -0.9166701 | -1.2345883 |
| -0.1899047 | 0.4777969 | 0.0656861 | 0.0373331 | 0.5120139 | 0.4171686 | -1.0165063 | 0.2913820 | -0.0407272 |
| -2.5434287 | -0.1218327 | -0.6979837 | -1.6968064 | -0.3218692 | -0.3000422 | -0.1604618 | -0.1430993 | -1.4686417 |
| -0.7080471 | -0.4955363 | -0.6105111 | -0.8830117 | -0.6282853 | -0.3272634 | -0.6530638 | -0.6275913 | -0.9231905 |
| -0.0066354 | -0.4180659 | -0.6174500 | -0.5219129 | -0.4011304 | 0.3366401 | -0.0286396 | -0.1320856 | 0.2111742 |
| -1.0649613 | -0.2350389 | -0.3378145 | -1.6214553 | -0.1630866 | 0.1433055 | -0.6334068 | -0.5410445 | -1.0697374 |
| -1.2397530 | -0.4423450 | -0.4718998 | -0.3665921 | -0.5000295 | -0.0539151 | -0.1729285 | -0.2191359 | -0.7054949 |
| -2.2967937 | -1.1582427 | -1.0174167 | -1.8827037 | -1.4579192 | -1.1791176 | -0.6254877 | -1.4491105 | -1.6107925 |
| 0.5848038 | 0.1831730 | 0.6339394 | -0.1260358 | 0.1761586 | -0.5868558 | 0.2210075 | 0.3172884 | 0.4260350 |
| -0.9020912 | -0.0254227 | -0.1952768 | -0.9692316 | 0.0817516 | -0.0215944 | -0.1604865 | -0.1809363 | -0.4192497 |
| -0.9148493 | 0.1457378 | -0.3009524 | -1.1634476 | 0.2622683 | -0.2582915 | -0.3586962 | 0.0350237 | -0.1862592 |
| -0.7761162 | -1.6893547 | -0.3930489 | -0.4525135 | -1.0462663 | 0.7223221 | 0.3323602 | 0.2200065 | -0.6339129 |
| -1.0855269 | -0.3978187 | -0.2319587 | 0.0549879 | -0.3983804 | -0.2155360 | -0.5302941 | -0.2073102 | -2.3248295 |
| 0.6005210 | -0.5209104 | 0.0782141 | -0.2508562 | -0.3560231 | 0.1744481 | -0.3763379 | 0.0088298 | 0.2260771 |
| -0.7222984 | -0.4841929 | -0.5323444 | -0.8358271 | -0.4181865 | -0.8322502 | 0.2384990 | -0.2294424 | -0.8596333 |
| 0.6361881 | -0.5205967 | 0.3164659 | 0.0108567 | -0.2335480 | 0.1716530 | -0.2792217 | -0.3046691 | 0.0690143 |
| -2.6369694 | -0.2640858 | -1.7181887 | -1.8551973 | -0.6769078 | -0.9993059 | -1.4299672 | -1.1805377 | -2.4501020 |
| -3.2577459 | -1.0067495 | -1.1655971 | -1.7490947 | -0.8467508 | -1.2221888 | -0.7939487 | -0.8282418 | -1.7623828 |

| | | | | | | | | |
|---|---|---|---|---|---|---|---|---|
| 0.1916133 | 0.1452568 | 0.0841785 | -0.3761016 | 0.1216747 | 0.1683016 | -0.5870401 | 0.4550097 | 0.3797664 |
| -0.0618565 | -0.8617244 | -1.0126224 | -1.7340839 | -1.0172393 | -0.3076431 | -1.0656507 | -0.9697429 | -1.4665824 |
| -0.9775406 | -0.5234111 | -0.0141219 | -0.0427308 | -0.5300223 | -0.3981643 | -0.3367334 | -0.2628354 | -0.0600397 |
| 0.4886747 | -0.2942685 | -0.1190674 | -0.5095835 | -0.3281109 | -0.5318268 | -0.4106912 | 0.7480661 | 1.1967964 |
| -1.7009074 | -0.2900967 | -0.9857949 | -0.8146914 | -0.4902707 | -0.7073456 | -0.8472807 | -0.4349922 | -1.3526012 |
| 1.7879833 | -0.5134198 | 0.8793811 | 1.2740221 | -0.1536542 | 0.6715649 | -0.2295141 | 0.3025554 | 1.3119020 |
| -0.8114599 | -0.2493116 | -0.1520383 | -0.4425336 | -0.1439666 | -0.2601194 | -0.5496278 | -0.6087756 | -0.5379545 |
| -1.1860631 | -0.3308907 | -0.5566389 | -0.5327578 | -0.6025974 | -0.3841505 | -0.7074177 | -1.0894163 | -0.6157536 |
| -2.7540051 | -0.0379504 | -0.9594343 | -1.2728300 | -0.1492275 | -0.2823643 | -0.2816541 | -0.6558255 | -1.5291464 |
| 0.4093749 | -0.9583658 | -1.5271849 | -0.9649346 | -1.0723079 | -0.7135396 | -0.1480017 | -0.1898029 | -0.4544650 |
| 0.3320773 | -0.2315263 | -0.2036325 | -0.5031838 | -0.0483559 | 0.0182736 | -0.0874952 | -0.2079019 | 0.6610759 |
| -1.1292475 | -0.6986976 | -0.7245787 | -0.9039414 | -1.1373714 | 0.3097889 | -0.9759743 | -1.2622900 | -1.1903169 |
| -0.3751034 | -0.0124023 | -1.0278552 | -1.4705602 | -0.3666091 | -0.8805700 | 0.3367740 | -0.0693305 | -1.0268346 |
| -0.0199863 | -0.1288406 | -0.1529289 | -0.8554744 | -0.1898285 | 0.0134856 | 0.0034231 | -0.4773496 | -0.2212186 |
| -0.4622362 | 0.0228813 | -0.1772297 | 0.1878899 | -0.1762865 | -0.1545331 | -1.5682203 | -1.2038284 | -2.2538597 |
| 1.0795592 | -0.5596718 | -1.0950649 | -2.2929903 | -0.6967714 | -1.4218944 | -0.0815848 | 0.2729473 | -1.3842750 |
| -0.5868642 | -0.6736032 | -0.2736266 | -0.8016185 | -0.8502916 | -0.4200391 | 0.8345138 | -0.4363226 | -0.1519305 |
| -2.2239160 | -0.1242577 | -0.7038287 | -1.2353080 | -0.3856187 | -0.5949442 | -0.6588405 | -1.2522237 | -1.3820263 |
| -0.5928342 | -0.5485245 | -0.5928247 | -0.6171688 | -0.9321677 | -0.1301468 | -0.6402028 | -0.6309447 | -0.3347563 |
| -0.6645818 | -0.5205415 | -0.8800726 | -2.0271353 | -0.6002452 | -0.0433589 | -0.8684460 | -0.6433927 | -0.8694584 |
| 0.3082962 | -0.1987136 | 0.0614195 | 1.4001704 | -0.0382519 | -0.1907735 | -0.9200965 | -0.6508549 | 0.3222545 |
| -0.1300786 | -0.2932611 | 0.6664240 | -0.4193227 | 0.0697825 | 0.3222984 | -0.8590219 | -0.0279950 | -0.1025326 |
| -1.5610747 | -1.0405608 | -1.4135070 | -2.1792927 | -1.0950642 | -0.6724604 | -0.7278336 | -1.4534978 | -2.2077314 |
| -1.3757214 | -0.2789020 | -0.5720011 | -1.0276965 | -0.0616280 | -0.8041881 | 0.0140502 | -0.0541651 | -0.8655880 |
| -1.3351577 | 0.2128093 | -0.4611803 | -0.6672422 | -0.1613693 | -0.0436861 | -0.2816592 | -0.5972217 | -1.3726502 |
| -0.0244753 | -0.3032998 | -0.5421953 | -1.0155428 | -0.4577853 | -0.5137144 | -1.2444994 | -1.3939391 | -1.1123819 |
| -0.7738618 | -1.5828794 | -1.4898586 | -1.1684354 | -1.2989434 | 1.1093519 | -0.4286298 | -0.9543756 | -1.0908321 |
| 0.1035122 | -0.1192483 | 0.0196669 | -0.3478442 | -0.1281767 | 0.0974629 | 0.0211148 | -0.5596897 | -0.4237933 |
| -1.5470641 | -0.1318527 | -0.3516596 | -0.8298910 | -0.2188469 | -0.7235354 | 0.0893274 | -0.3282317 | -0.6011331 |
| -0.1365125 | -0.5554445 | 0.4522359 | 0.5031352 | -0.1362695 | -0.1409933 | 0.1696094 | 0.2822585 | 0.7076042 |
| 2.6767535 | -0.3020008 | 1.0654038 | 2.6140207 | -0.0929939 | 2.0631872 | 0.4634584 | 0.9223572 | 2.4047233 |
| -0.0491670 | -0.6660405 | -1.3159621 | -1.9330592 | -0.8731195 | -0.9884965 | -0.9129919 | -1.7152736 | -1.0182155 |
| 0.3573486 | 0.0761567 | 0.0385620 | -0.1299525 | 0.0514617 | 0.2878309 | -0.1984367 | 0.1766871 | 0.7839777 |
| 0.2688333 | 0.4198737 | 0.2476130 | 0.3188068 | 0.3974804 | 0.2575734 | -0.6674527 | 0.3628879 | 0.3684578 |
| 0.3581684 | 0.4977127 | 0.6700511 | 0.7202569 | 0.4972467 | 0.3919511 | 0.4927565 | 0.4485963 | 0.8171793 |
| -1.1484331 | -0.7855382 | -0.8227628 | -1.1835688 | -0.9463521 | -0.6443184 | -1.0238127 | -1.0043167 | -0.8705854 |
| 1.4265132 | 0.4226991 | 1.8530073 | 1.0488365 | 0.5697556 | 0.0870754 | -1.2753577 | 0.9335546 | 1.4728945 |
| 0.5022097 | -0.4954332 | 1.0594126 | 0.5385660 | -0.2431243 | 0.2802067 | -0.1094874 | 0.0224150 | 0.2940368 |
| 0.7560340 | -0.1701115 | -0.0175667 | -0.3683303 | -0.0117149 | 0.0531398 | -0.2027439 | -0.2587843 | -0.3711401 |
| -0.3846976 | -0.8348226 | -0.6009367 | -1.0096352 | -0.8891462 | 0.5848836 | -0.7646701 | -0.2393291 | -1.1589394 |
| -2.2057967 | -1.8675686 | -1.6063435 | -0.6667427 | -2.0238834 | -1.5382731 | -0.2543289 | -0.8646348 | -1.6490689 |
| 0.5157307 | -0.4075115 | -0.0330884 | 0.1165134 | -0.2540563 | -0.2483800 | -0.5058950 | -0.5665814 | -0.1598018 |
| 2.0936490 | -0.3062597 | 0.3459939 | 0.7812078 | -0.1606765 | -0.0243742 | -1.2510705 | 0.0955612 | 0.6508217 |
| 0.7424366 | -0.2115657 | -0.1115839 | 0.0074071 | -0.1709834 | 0.3521373 | -0.3827239 | 0.1796267 | 0.6875979 |
| -0.0533124 | -0.2040264 | -0.5480175 | -0.4972647 | -0.3828984 | -0.3629181 | 0.0475035 | -0.4377633 | 0.0621844 |
| -0.5367875 | -0.0047083 | 0.2053004 | 0.0497168 | -0.0867115 | -0.6072465 | -0.7631133 | -0.2369893 | 0.1136000 |
| -1.9005252 | -0.5668781 | -0.9535089 | -1.0189130 | -0.5459779 | -0.5731030 | -0.3198353 | -1.0483396 | -1.6759228 |
| -1.4046963 | -0.6376267 | -0.6968201 | -0.9739547 | -0.8213804 | -0.7142349 | 0.2123420 | -0.7436390 | -0.8986813 |
| -2.1961440 | -0.3202221 | -0.2943554 | -0.1311844 | -0.1408905 | 1.3838203 | -0.2989448 | -0.1197660 | -1.5094028 |
| 0.0192386 | -0.0170718 | -0.2358285 | -0.1213784 | -0.0714847 | -0.6021382 | -0.7349898 | -0.0350651 | 0.0275056 |

| | | | | | | | | |
|---|---|---|---|---|---|---|---|---|
| -0.0463542 | -0.2697513 | -0.3960150 | -1.6880904 | -0.5687554 | 0.0058529 | -0.8614232 | -0.0589327 | -0.3199771 |
| -2.3799815 | -0.9674715 | -0.8600573 | -0.6442116 | -1.3438296 | -0.3521946 | -0.4603923 | -0.3013909 | -1.2470020 |
| -1.6543592 | -0.6375048 | -0.4631603 | -1.5589376 | -0.3523798 | -0.4669859 | -0.9815953 | -0.4695598 | -0.9662882 |
| -3.4508800 | -0.7281290 | -0.7581082 | -1.7767065 | -0.7635689 | -1.2183726 | -1.1126717 | -1.1767783 | -2.3327842 |
| -1.5315410 | -0.3861317 | -0.7924836 | -1.4409645 | -0.5907837 | -0.7616522 | -0.3283650 | -0.0977982 | -0.8107916 |
| -0.1837210 | -0.3924962 | -0.1943979 | -0.3376668 | -0.5209129 | -0.3439851 | 0.0045780 | 0.1674939 | 0.1721794 |
| 2.8146188 | 0.5998016 | 2.2934291 | 3.3549823 | 0.8187437 | 0.1916091 | -0.1157664 | 0.7200309 | 1.4278313 |
| -0.1842160 | -0.3867740 | -0.0982113 | 0.3513872 | -0.1869256 | -0.5413000 | -0.2075351 | -0.4339044 | 0.2309573 |
| -0.7751870 | 0.4441210 | 0.0427229 | -0.6829794 | 0.3513352 | -0.4232638 | -0.6132653 | -0.9242482 | -1.9199099 |
| 0.5857383 | -0.8983308 | -0.5402286 | -0.3302717 | -1.1186658 | 0.2490049 | 0.0197574 | -0.5778512 | 0.1070262 |
| 0.7963875 | -0.5665913 | -0.2808508 | -0.4009883 | -0.8429904 | 0.9436706 | 0.1230520 | -0.4125104 | -0.1464643 |
| 0.1330599 | -0.6866607 | -0.4876522 | -1.2199498 | -0.8095929 | -0.4421259 | -0.6608941 | -1.0340660 | -0.5082665 |
| -0.2675993 | -0.6179663 | -0.7141925 | -0.4271268 | -0.7609023 | -1.2429106 | 1.2730595 | 1.2241475 | -0.3932414 |
| -0.6104600 | 0.2722453 | -0.4413690 | -1.1895526 | -0.0001747 | -0.3202683 | -0.7235357 | -0.0547919 | -0.3061191 |
| -2.8750741 | -0.1240704 | -0.9696682 | -1.8863130 | -0.3920143 | -0.1211820 | -0.2520364 | -0.3638540 | -1.6086281 |
| -1.6800463 | -0.3326183 | -0.8344744 | -2.4103398 | -0.8942984 | -0.2467849 | -0.1712509 | -0.3644206 | -0.7373700 |
| -2.0128314 | 0.1709418 | -0.2942986 | -0.5506714 | 0.1554134 | -0.2409368 | -1.0173468 | 0.0773608 | -0.7832663 |
| 0.0804339 | -0.0495166 | 0.0656715 | -0.3157756 | -0.4312565 | -0.7526510 | -0.7730480 | -1.0134938 | -1.0100473 |
| -1.3752075 | -0.5288345 | -1.7413360 | -2.7035848 | -0.6400479 | -0.4105578 | -0.0770137 | -1.0779898 | -1.4771663 |
| 0.5460248 | -0.0278858 | 0.3307356 | -0.4986665 | -0.1263319 | -0.2905060 | -1.3556470 | -0.0529562 | -0.5890890 |
| -1.5882164 | -1.0912611 | -1.9310323 | -2.6434459 | -1.4017552 | -0.2043732 | -0.3739484 | -0.9531305 | -1.8681126 |
| -0.6321930 | -0.3067140 | -0.3782745 | -0.3846375 | -0.4427139 | -0.2035288 | -0.8212550 | -1.5756086 | -3.3270834 |
| -4.0107790 | -0.3557564 | -0.4708895 | -1.2942847 | -0.4810424 | -0.9603963 | -0.1210579 | -0.9458400 | -1.8759626 |
| 0.1396359 | -0.1794265 | -0.3505975 | -0.2236568 | -0.1569040 | -0.1597615 | -0.3148688 | -0.3726276 | 0.1648753 |
| -1.1905280 | -0.5266456 | -0.5060633 | -0.5064130 | -0.6362084 | -0.0991698 | 0.7125049 | -0.6270433 | -1.4694303 |
| -0.2158810 | -0.1511122 | -0.1215497 | -0.9049119 | -0.2593698 | -0.2690702 | -0.2413369 | -0.7406633 | -0.2606576 |
| -1.5785732 | -0.0311939 | -0.5548856 | -0.8181684 | -0.0997326 | -0.0443898 | -0.6827132 | -0.6120087 | -1.1631791 |
| -1.8429679 | -0.4431466 | -0.7430306 | -1.1314206 | -0.4008364 | -0.2618952 | -0.0461041 | -0.3338983 | -0.0847064 |
| 0.5607932 | -0.6277684 | -0.3776082 | -0.9000758 | -0.5636952 | -0.2173431 | 0.1158973 | 0.1455829 | 0.6104083 |
| 0.9002172 | -0.0192156 | 0.9789815 | 1.2284746 | -0.0738213 | -0.4686396 | -1.0977529 | 0.0743392 | 0.5002732 |
| -0.6055962 | -0.8024993 | -0.7281729 | -1.4862373 | -0.8770976 | -0.8907452 | 0.2329061 | -0.2066068 | -0.4917879 |
| -0.1359576 | -0.2446704 | -0.4655364 | -0.8651635 | -0.2438829 | -0.2740676 | -0.4853125 | -0.6051534 | -0.8567894 |
| 1.3241895 | -0.2330441 | 0.9159039 | 2.1173968 | 0.0459694 | -0.8967437 | -0.4254839 | -0.4791837 | 1.5634754 |
| -1.0359162 | 0.0303514 | -0.6618920 | -0.9452486 | -0.0794972 | -0.2796922 | -0.6380979 | -0.5531881 | -0.7464354 |
| 0.1791123 | -0.2741567 | 0.2430885 | -0.9913381 | -0.2382162 | 0.7329949 | 0.0949206 | -0.4730797 | -0.9854151 |
| -1.8846694 | -0.5937155 | -1.4836105 | -1.4665047 | -0.3731128 | -0.8970107 | -0.3776428 | -0.5588499 | -1.8065348 |
| -2.7068078 | -1.3197670 | -1.7931976 | -2.2948195 | -1.5372104 | -0.4352200 | -0.4505793 | -1.8171832 | -2.3389351 |
| -0.2233524 | -0.1366692 | -0.1271533 | -0.1547826 | 0.1228572 | -0.2802585 | -0.3521447 | -0.2979761 | -2.5598527 |
| -0.1488389 | -0.3149940 | 1.3989388 | 0.8831256 | 0.0495547 | 0.1023407 | -0.2793592 | 0.5029852 | 0.2082599 |
| -1.6054675 | -0.1668224 | -0.1643539 | -0.5354765 | -0.0618823 | -0.2217236 | -0.3645008 | -0.3854685 | -1.0733859 |
| 0.1919361 | 0.0286148 | 0.1886378 | 0.1655393 | -0.0262820 | -0.2055062 | -2.0693428 | -0.4149931 | -0.1980149 |
| -1.2842080 | -0.3773040 | -1.1661161 | -1.6883505 | -0.5868119 | -0.1266609 | 0.0304174 | -0.2484104 | -1.0546505 |
| -1.5524836 | -0.1777528 | -0.5776316 | -0.7569250 | -0.4528710 | -0.6285393 | -0.5727934 | -0.6620163 | -0.4290857 |
| -0.5476123 | -0.1350424 | -0.7005275 | -0.6115451 | -0.1012057 | -1.5014735 | -1.6348575 | -0.6765366 | -1.3407859 |
| -0.0063181 | -0.3924957 | 0.2265405 | -0.1239352 | -0.2412685 | -0.4914668 | -0.1095642 | -0.6531623 | -0.5515348 |
| -0.8472366 | -0.7724906 | -0.8905543 | -1.6957751 | -0.8355694 | -0.5792789 | -1.0094562 | -0.1792032 | -1.0508528 |
| -0.3331585 | 0.0665303 | 0.1408684 | -0.4656038 | -0.3451642 | 0.0464366 | -0.8214131 | -0.4144661 | -0.7677810 |
| -1.1573036 | -0.9390549 | -1.511236 | -2.1560366 | -1.0382887 | -0.9746276 | -1.0132805 | -1.4386698 | -1.7127930 |
| 0.4010636 | -0.3460783 | 0.2630828 | -1.0575837 | -0.1242680 | 0.2912425 | -1.3503951 | -0.7364301 | 0.1996695 |
| -1.7445262 | -0.9436939 | -1.0759012 | -0.7286761 | -0.9169292 | -0.2466224 | -0.4542716 | -1.5107555 | -1.3891363 |

| | | | | | | | | |
|---|---|---|---|---|---|---|---|---|
| 1.7609110 | -0.7378584 | 0.0962332 | 0.0367371 | -0.8151060 | -0.4830505 | -0.2138974 | -0.2847963 | 0.4753033 |
| -0.5610032 | -0.1813367 | 0.2190678 | -0.4190429 | -0.1238456 | -0.3640897 | -0.0246366 | -0.2568286 | 0.0598002 |
| -0.0256817 | -0.1680751 | -0.0472396 | 0.2367462 | -0.2444117 | 0.1151028 | -0.2311597 | -0.2039671 | 0.4682274 |

| PG_534 | PG_535 | PG_536 | PG_537 | PG_538 | PG_539 | PG_540 | PG_541 | PG_542 |
|---|---|---|---|---|---|---|---|---|
| -0.7171478 | -0.8449153 | -2.2231179 | -0.3037140 | -0.5653053 | -1.3127583 | -0.4594055 | -0.5903845 | -1.4466936 |
| -0.7478757 | -1.3394514 | -1.4167010 | -0.5713565 | -0.4196060 | -1.3200338 | -0.7359902 | -0.5729269 | -1.5032261 |
| -0.6144376 | -1.2071128 | -1.7213961 | -1.3267752 | -1.4398817 | -2.0177525 | -1.2460501 | -1.3995311 | -1.7772492 |
| -0.2150525 | -0.0712283 | -0.7289888 | 0.0666074 | 0.3513423 | -0.1880598 | -0.0870467 | -0.1013961 | -0.1300998 |
| -0.1195593 | -0.0521103 | -0.5989607 | -0.1360934 | -0.1277427 | -0.5081404 | 0.0133735 | -0.0855084 | -0.5770767 |
| -0.4583318 | -0.6688710 | -1.7120129 | -0.5006831 | -0.6817443 | -2.0807585 | -0.5080851 | -1.2946693 | -2.5163647 |
| -0.1551353 | -0.9619690 | -0.6843600 | -0.2575799 | -0.2320969 | 0.3813898 | -0.2420309 | -0.5591241 | 0.4899206 |
| -0.7644378 | -0.7482365 | -2.7819770 | -0.6652560 | -0.6414310 | -1.9113705 | -0.7326757 | -0.7932314 | -1.8710936 |
| -0.1144934 | 0.2729943 | 0.5072275 | -0.6207430 | -0.2806375 | -0.9202139 | -0.6831554 | -0.3017170 | -0.6598556 |
| -0.7779942 | -1.8080720 | -1.7931595 | -0.6484239 | -0.8520067 | -0.9574200 | -0.8267764 | -0.8507106 | -0.9466650 |
| -0.5287522 | -0.6076551 | -1.2568918 | -0.1454177 | -0.4465537 | -1.1402324 | 0.0004273 | -0.4180339 | -1.2260438 |
| -0.3769445 | -0.3044626 | -0.6971286 | -0.2264221 | -0.4194625 | -0.3998932 | -0.2099464 | -0.6708524 | -0.5518963 |
| -0.7006295 | -0.9944928 | -2.5415796 | -0.5575228 | -0.6507695 | -1.1617483 | -0.7137669 | -0.9253546 | -1.4714661 |
| -0.6494587 | 1.9082477 | 3.4038413 | -0.6008553 | 1.2224059 | 3.2030940 | -0.3356940 | 3.2279602 | 3.5266882 |
| -0.1653549 | -0.2944480 | 0.0935492 | -0.1423281 | 0.2727818 | 0.7331783 | 0.2139542 | 0.4959046 | 0.5404876 |
| -0.1607162 | 2.6420190 | 2.5628534 | -0.6750590 | 1.0957037 | 2.5286855 | -0.5674652 | 2.3017401 | 2.8337080 |
| -1.9730151 | -2.6498273 | -1.4003558 | -0.6087298 | -1.3698453 | -1.7207825 | -0.9211898 | -1.3562237 | -1.2714635 |
| -0.8885270 | -0.2250342 | -0.2539743 | -0.2457318 | -0.7718727 | -0.7864645 | -0.4501749 | -0.5407268 | -1.1273406 |
| -1.0269004 | -1.8094154 | -1.9517739 | -0.4408775 | -0.6785530 | -0.2687928 | -0.6726193 | -0.5801341 | -0.5468957 |
| 0.5625024 | -0.2647116 | -2.679995 | -0.0856767 | -0.7652236 | -0.1555160 | -0.7332843 | -0.7216085 | -0.1134625 |
| -0.1082879 | 0.0036520 | 0.1105162 | -0.2856006 | 0.1133386 | 0.1652895 | -0.2365716 | 0.1739416 | -0.0728628 |
| -1.7357838 | -1.5729244 | -3.0593154 | -0.3901501 | -0.4462846 | -0.4738591 | -0.3883484 | -0.3352596 | -0.3001617 |
| -0.2009342 | 0.7104055 | 0.8641229 | -0.1802151 | 0.5551387 | 1.4550298 | -0.2769921 | 0.8785303 | 1.3573124 |
| -0.5070798 | -1.3313230 | -1.8115082 | -0.2446565 | -0.3071348 | -1.2014175 | -0.4052411 | -1.1228158 | -1.5186081 |
| -1.1544179 | -1.8829305 | -2.5978144 | -0.2719533 | -0.9789678 | -2.9836628 | -0.530261 | -1.4372135 | -2.6679737 |
| -0.1528421 | 0.3749981 | 0.6043327 | -0.3169291 | 0.4549384 | 0.2443806 | -0.2309834 | 0.3705689 | -0.2090968 |
| 0.1859629 | -0.0132440 | -0.0298179 | 0.2626401 | 0.0699093 | -0.1893783 | 0.1262761 | 0.1709205 | -0.3201708 |
| -1.2116128 | -1.5633151 | -2.6649933 | -0.2103534 | -0.4849069 | -2.1206183 | -0.7599692 | -0.7798107 | -1.9921638 |
| -0.3986286 | -1.0666254 | -2.8122660 | -0.2502273 | -0.6316861 | -2.5089394 | -0.4271566 | -0.8695719 | -2.2641294 |
| -0.2889381 | -0.4274872 | -0.6236736 | 0.0648827 | -0.0003805 | 0.3292658 | 0.0533393 | -0.0541155 | -0.0104332 |
| -1.1426532 | -1.8160943 | -4.2822456 | -0.5100076 | -0.4976470 | -2.5081948 | -0.3349054 | -0.8258127 | -2.4126319 |
| -0.1132943 | -0.5993444 | -1.6913150 | -0.0366587 | -0.0792107 | -0.3620085 | 0.1826740 | -0.0187275 | -0.1121623 |
| -0.1451085 | -0.1419641 | 0.2119588 | 0.0126223 | 0.0115005 | 0.4359068 | 0.0398972 | 0.7200809 | 0.7828663 |
| -0.3553508 | -0.2109722 | -2.2490329 | -0.5577142 | -0.8329650 | -1.7987580 | -0.2561823 | -0.9910269 | -1.7963463 |
| -1.3175764 | -1.3680448 | -3.0834319 | -0.2546340 | -0.0975968 | -0.2389375 | -0.2373951 | -0.2761093 | -0.3228804 |
| -0.9244202 | -0.7938472 | -0.1985544 | -0.2591229 | -0.5416226 | 0.2455233 | -1.2356638 | -0.6526652 | 0.4947421 |
| -0.3177969 | -0.9972474 | -1.4656740 | -0.4624461 | -0.8222389 | -1.6010327 | -0.3769389 | -0.9331148 | -1.8786271 |
| 0.8924224 | 2.7586593 | 0.7675183 | -0.1878735 | 1.9311160 | -0.2239272 | -0.2538028 | 3.2796165 | -0.4249544 |
| -0.0293551 | -0.1966823 | 0.1653955 | -0.4277091 | -0.6623391 | 0.7242462 | -0.2578757 | -0.5121336 | 0.9104264 |
| -0.9931768 | -1.0296062 | -0.9017369 | -0.2500478 | -0.7451339 | -0.3242624 | -0.9357753 | -0.2028861 | -0.2819926 |
| 0.3041944 | 0.3487260 | 1.6313689 | -0.1581103 | -0.2435842 | 0.4616959 | 0.1251096 | 0.1730302 | 0.5314367 |
| -0.5260101 | -0.9488389 | -1.9506233 | -0.4886497 | -0.7339492 | -1.6362303 | -0.3554559 | -0.8790838 | -1.3056657 |
| -0.2477409 | -0.2048320 | -1.0923641 | -0.1397282 | -0.3197636 | -0.6254641 | -0.1618774 | -0.2628180 | -0.5290826 |
| -0.3229714 | -0.1420503 | -1.5664804 | -0.2645788 | -0.1385719 | -1.1309115 | -0.2561653 | -0.3168413 | -1.5483374 |
| -0.2244403 | 0.0062035 | 1.0284946 | -0.1057232 | 0.6057131 | 0.9162527 | -0.0066312 | 0.2064570 | 0.1206806 |
| -0.5361903 | 0.4945716 | 1.8452083 | -0.5498613 | -0.2673470 | 0.2154793 | -0.0659357 | -0.3182805 | 1.3956957 |
| 0.1364812 | -0.2140096 | 0.1086022 | -0.2604131 | -0.2471013 | -0.5192695 | -0.2369453 | -0.1812610 | 0.2644069 |

| | | | | | | | | |
|---|---|---|---|---|---|---|---|---|
| -0.3980948 | -0.0395321 | -0.1825519 | -0.2163293 | -0.2075739 | 0.0833244 | -0.3181895 | -0.2174181 | 0.1625256 |
| -0.3452544 | -0.6152586 | -1.9500981 | -0.2614244 | -0.2913189 | -1.3214075 | -0.1810999 | -0.6559412 | -1.4923179 |
| -0.4272712 | -0.0891600 | 0.1274652 | -0.4206542 | -0.5290474 | -0.4381121 | -0.3431852 | -0.0931365 | -0.5754056 |
| 0.7514365 | 2.8231123 | 1.8747668 | -0.9139848 | 0.2723049 | 0.2464145 | -0.9071801 | 1.3334960 | 1.0763924 |
| -0.3729040 | -0.8233024 | -0.9715300 | -0.4615100 | -1.3771623 | -1.4486323 | -1.4183109 | -0.8728811 | -0.8356098 |
| -0.2374337 | -0.2067267 | 0.5332429 | -0.2316693 | -0.3468367 | 0.1009617 | -0.2029020 | -0.1669920 | -0.1303626 |
| -0.8091172 | -1.1643303 | -1.5193665 | -0.3176382 | -0.6038538 | -1.2541831 | -0.4531286 | -0.9319544 | -1.1278689 |
| -0.1763785 | 0.9835963 | 2.4676409 | -0.3172068 | 0.5197195 | 1.3572215 | -0.1012104 | 1.3358691 | 1.6828952 |
| -1.3165109 | -0.2616761 | -1.5892445 | -1.0564434 | 2.0246917 | -0.0096052 | -0.6424707 | 4.0558259 | 1.6776482 |
| -0.8383280 | -0.4703973 | -0.6582639 | -0.5595779 | -0.0498404 | -0.2663964 | -0.9066226 | -0.3185081 | -0.8958681 |
| -0.3615919 | -1.3132615 | -1.3332498 | -0.7561011 | -0.7104910 | -1.5680659 | -0.9712488 | -0.9561185 | -1.7277098 |
| 0.2633750 | 2.7963529 | 3.0872703 | 0.0338579 | 2.7921147 | 3.9434060 | 0.3433582 | 4.3665422 | 4.3178196 |
| 0.5054522 | 2.6177914 | 1.8961174 | 0.2608843 | 1.4869403 | 1.5960305 | 0.1899582 | 2.0767791 | 1.3994171 |
| -0.7855628 | -0.8253001 | -0.5646088 | -0.6577685 | -1.0035392 | -0.8275244 | -0.6435100 | -0.8725023 | -0.7860974 |
| -0.3108084 | -1.3243796 | -1.5245835 | -0.7573956 | -1.3854901 | -2.0911150 | -0.9372256 | -1.9736970 | -2.4391724 |
| -0.5078026 | -0.3805806 | -0.0645056 | -0.5166578 | -0.5187034 | -0.9149957 | -0.8785789 | -0.6470606 | -0.5071504 |
| -0.3279849 | -0.9488411 | -1.6977782 | 0.0655002 | -0.2256414 | -2.0639485 | 0.0390659 | -0.7957314 | -1.7991505 |
| -0.2474027 | -0.8766184 | -0.7662009 | -0.2953100 | -0.6197382 | -1.0353440 | -0.0447490 | -0.8554124 | -1.0060552 |
| -0.2559095 | -0.7236408 | -0.3551724 | -0.2606237 | -0.5413153 | -0.6857829 | -0.2606106 | -0.5889455 | -0.4519518 |
| -0.0600201 | -0.4451748 | -0.9175009 | -0.0140997 | -0.2575640 | -0.9736501 | 0.0300973 | 0.0184229 | -0.6977165 |
| -0.9250846 | -0.3796497 | -1.6983425 | -0.3536281 | -0.3130183 | -0.4362358 | -0.4633546 | -0.6320823 | -0.3882239 |
| -0.6379776 | -0.7636952 | -1.1357832 | -0.5709311 | -0.8908215 | -1.4851573 | -0.7257636 | -0.8494208 | -1.7378398 |
| -0.1277106 | -0.8047635 | -0.2315249 | -0.4529338 | -0.2237509 | -0.7378923 | -0.1312913 | -0.2964328 | -0.7876122 |
| 0.4777094 | 0.6924790 | 0.3841979 | 0.4642359 | 0.7969701 | 0.3269023 | 0.6531617 | 0.6512875 | 0.0547744 |
| -0.9334441 | -1.5279292 | -3.0189744 | -0.4717424 | -0.6314894 | -2.2926149 | -0.7668592 | -1.0945388 | -2.1797339 |
| -0.6856079 | -1.3982783 | -0.4145769 | -0.3699104 | -0.4919429 | -0.6722488 | -0.4469484 | -1.1534625 | -1.0996977 |
| -0.3726450 | -2.5196748 | -3.3598308 | -0.6682251 | -1.8866225 | -3.8279025 | -0.6277807 | -2.6982912 | -3.5665730 |
| -0.2575415 | 0.7206307 | 2.9479490 | 0.3986518 | 2.2152386 | 4.1657814 | 0.6767970 | 2.3389125 | 3.6525218 |
| -0.1851393 | 0.6567020 | 1.1681629 | 0.1206917 | 0.5173204 | 1.3169591 | 0.0991493 | 1.2465466 | 1.1865565 |
| 0.1954190 | 0.0841670 | -1.8670062 | -0.4176665 | -0.4052333 | -1.9714253 | -0.4921052 | -0.8417951 | -2.1840157 |
| -0.5902160 | -0.5865874 | -0.9357357 | -0.1427408 | -0.4594719 | -0.3482230 | -0.1574180 | -0.4540495 | -0.2363332 |
| -0.1749343 | -0.2722726 | -0.0671129 | -0.1602129 | -0.1385783 | -0.2487294 | -0.0949216 | -0.3586739 | -0.6522956 |
| -0.5808896 | -1.3614330 | -0.5477942 | -0.6161946 | -1.2972378 | -1.0672864 | -0.5877736 | -1.9781672 | -1.1263751 |
| -0.4904540 | -0.5928664 | -1.5039497 | -0.3908882 | -0.5055729 | -1.2730081 | -0.5813377 | -0.8108093 | -1.3320553 |
| 0.0879601 | 0.9599826 | 2.1333656 | -0.6729466 | -0.1356079 | 1.1731750 | -0.6543665 | -0.3677697 | 0.8020245 |
| 0.0345636 | 0.1787954 | -0.4773717 | -0.2363999 | -0.1371596 | -0.3304988 | -0.2407869 | -0.5391726 | -0.1648646 |
| 0.0131809 | -0.2276062 | -2.0462840 | -0.3203352 | -0.3520550 | -0.2203679 | -0.4240830 | -0.5288065 | -0.2699829 |
| -0.4868715 | -0.9759440 | -1.0348012 | 0.0038786 | -0.3062442 | -1.3230136 | -0.1272559 | -0.9078424 | -0.9076596 |
| 0.1236210 | -0.7730565 | -2.9633723 | -0.5249766 | -0.7964174 | -2.4746440 | -0.4619340 | -1.3725566 | -2.6653333 |
| -1.3906648 | -2.3274124 | -2.5700912 | -0.3563315 | -1.0965933 | -2.5364569 | -0.4277620 | -1.4389188 | -2.9746676 |
| -0.5722031 | -0.6495473 | -1.3617241 | -0.3978350 | -0.6645370 | -0.7857279 | -0.6618041 | -0.5276751 | -0.8092498 |
| 0.5140298 | -0.7753887 | -1.3310840 | -0.6226787 | -1.9164900 | -1.5959183 | -0.9695493 | -1.5839512 | -2.2538181 |
| 2.8097525 | 4.1729250 | 3.3469219 | -0.4100586 | 1.7060572 | 0.8263948 | -0.0122000 | 2.5066264 | 0.3832369 |
| 0.0091843 | -0.0284475 | -0.1498010 | -0.0614241 | 0.1139699 | 0.1113580 | -0.0444842 | 0.0242067 | 0.0385304 |
| 0.1107632 | -0.2684082 | 0.1813886 | 0.0030891 | -0.0801951 | -0.6415114 | 0.1220543 | 0.1447412 | -0.6334839 |
| -1.3522894 | -1.9776863 | -0.6728013 | -0.6471463 | -1.2756446 | -1.2174295 | -0.8610401 | -1.2002984 | -1.0910955 |
| 0.2693899 | 6.6150609 | 8.4658273 | -0.2046287 | 4.6413840 | 8.0118843 | -0.0909463 | 7.9361695 | 8.9188093 |
| -0.0318227 | 2.0025011 | 1.9700128 | 0.0373610 | 1.2974630 | 2.2003368 | -0.0493197 | 2.4306636 | 2.3636663 |
| -2.3371270 | -2.2613363 | -0.8048433 | -0.3812204 | -0.3196607 | 0.6263664 | -0.2087666 | -0.2455552 | 1.9146823 |
| -0.8316485 | -0.9787726 | -1.0332825 | -0.6038560 | -0.7331205 | -1.2181495 | -0.7578723 | -0.7116504 | -1.1389093 |

| | | | | | | | | |
|---|---|---|---|---|---|---|---|---|
| -0.6292854 | -0.5275272 | -3.7312323 | 0.1225853 | -0.3279055 | -0.7763878 | -0.0006694 | -0.4249693 | -0.3260330 |
| -0.5019082 | -0.5225598 | 0.5127238 | -0.2029187 | -0.1862797 | 0.2309986 | 0.0226411 | -0.2719486 | 0.0972644 |
| -0.6945148 | -1.3009405 | -1.0804322 | -0.5513437 | -0.6608753 | -0.7240350 | -0.6204696 | -1.0075991 | -1.2234438 |
| -0.0925787 | -0.1066946 | -0.0938720 | -0.0173906 | 0.1797203 | 0.4067514 | -0.0206764 | 0.1368893 | 0.3488963 |
| -0.6576987 | -1.9980545 | -1.6686448 | -0.3459238 | -0.9987633 | -2.9723416 | -0.3480731 | -1.5467001 | -2.9429756 |
| -0.7949723 | -1.0859334 | 0.0601173 | -0.5362952 | -0.0828300 | -0.7783103 | -0.3858893 | 0.0341998 | -0.3509243 |
| -0.6545375 | -0.7440386 | -0.6765988 | -0.3306267 | -0.2034480 | -1.3550593 | -0.5174211 | -0.2184645 | -1.1971443 |
| -0.1171279 | 0.0346219 | -0.1092484 | 0.1116661 | -0.1061362 | -0.0574383 | -0.0082821 | -0.0865087 | 0.1389363 |
| -0.3264707 | -0.1579937 | 0.0826356 | -0.5851784 | -0.1699401 | -0.2256554 | -0.4725377 | -0.2618479 | -0.2358239 |
| -0.4990434 | -0.1515867 | -0.0004296 | -0.0650030 | 0.3323293 | 0.3402091 | 0.0512757 | 0.4825737 | -0.0129922 |
| -0.3907046 | -0.7341794 | -2.0473439 | -0.0647159 | -0.1490204 | -0.5375951 | -0.1531523 | -0.2959949 | -0.4187594 |
| -0.5106380 | -0.4472264 | -1.4477763 | -0.0426426 | -0.3816407 | -1.7136058 | -0.4304913 | -0.4850702 | -2.0882936 |
| -0.8987016 | -1.1324320 | -3.9325469 | -0.4320001 | -0.4778652 | -2.8383560 | -0.3590932 | -0.8193649 | -2.6658276 |
| -0.4236134 | -0.9086062 | -2.6345289 | -0.0849241 | -0.3011428 | -1.9473440 | -0.2051520 | -0.4787171 | -1.5309766 |
| -0.4112652 | -0.8731580 | -1.6136112 | -0.2115295 | -0.4187401 | -0.4663076 | -0.2232597 | -0.5222519 | -0.7602967 |
| 0.0687471 | -0.0101695 | 0.1513134 | 0.0427593 | -0.1259458 | 0.0376753 | 0.0797378 | -0.0521017 | 0.0279277 |
| -0.7485833 | -1.4287600 | -0.6164084 | -1.0445377 | -0.6726047 | -1.1858189 | -0.7063290 | -1.5056991 | -1.4663883 |
| 0.1662964 | 0.1490525 | 0.2286741 | -0.1469515 | -0.4196424 | 0.0077553 | -0.1817855 | -0.4678133 | 0.7960862 |
| -0.0574111 | -0.6332090 | 0.2543119 | -0.0051341 | -0.6403429 | -0.6455615 | -0.3160222 | -0.0823342 | -0.6761834 |
| -0.3772937 | 0.2543462 | 1.3694671 | -0.1921307 | -0.0566596 | 0.6499714 | -0.2622297 | 0.0899135 | 0.6220077 |
| -0.6314272 | -0.5376549 | -1.2304349 | -0.6844189 | -0.6586782 | -2.3043621 | -0.6254975 | -1.0605545 | -2.2252680 |
| -0.6070539 | -0.5673171 | -0.6208078 | -0.4973209 | -0.4809608 | -0.6144277 | -0.5166088 | -0.7583139 | -1.0054864 |
| -0.0718064 | -0.2527714 | -0.0831298 | -0.3071772 | -0.3594310 | -1.1448877 | -0.3323780 | -0.3682324 | -1.1236166 |
| -0.2143496 | -0.5099863 | -2.0187337 | -0.3410988 | -0.8533963 | -1.6015037 | -0.3748919 | -1.0179120 | -1.8182655 |
| -1.3314564 | -1.9349865 | -2.4244305 | -0.7799453 | -0.5858336 | -0.5668851 | -0.4289898 | -0.6891140 | -0.2129868 |
| -0.4582744 | -0.8377812 | -0.2280596 | -0.0887836 | -0.1341516 | -0.7143874 | -0.1612562 | -0.5436586 | -0.5733156 |
| 0.3753902 | 0.1616545 | 0.5313629 | 0.0202062 | -0.1728018 | 0.2474047 | 0.1599963 | -0.0705104 | 0.0587239 |
| -0.2635974 | 0.5197541 | 0.3869096 | -0.4829731 | -0.4164237 | -0.1490594 | -0.7883853 | -0.4022350 | -0.7020321 |
| -0.5188425 | -1.2494416 | -2.0640521 | -0.1137506 | -0.3189173 | -1.4089697 | -0.2196293 | -0.8519880 | -1.3932714 |
| -0.1761352 | -0.0053293 | -0.6694506 | -0.0753391 | -0.0670058 | -0.0632482 | -0.1010569 | 0.2538029 | -0.2930209 |
| -1.4061116 | -1.6475528 | -1.8295029 | -0.2675746 | -0.9874017 | -1.0606242 | -0.6204567 | -0.8143189 | -0.7678942 |
| -0.4808708 | -0.8310075 | -1.2825876 | -0.3026963 | -0.6251054 | -1.1014271 | -0.4400062 | -0.6164085 | -1.3964080 |
| -1.0646903 | -0.6972286 | -0.9447164 | -0.6852888 | -0.9906181 | 0.2859968 | -0.8295789 | -0.5580493 | 0.1724671 |
| 0.4094102 | 0.2230918 | -0.2860032 | 0.2423412 | 0.1862306 | 0.1388106 | 0.3239869 | 0.1074007 | 0.2196379 |
| -0.1376762 | -0.1790625 | -1.9794075 | -0.2199794 | -0.3680158 | -1.3234458 | -0.1034973 | -0.6128194 | -1.1495062 |
| -0.5694061 | -1.0197369 | -1.0270569 | -0.1705561 | -0.0681641 | -0.9888865 | -0.2083476 | -1.0052739 | -1.4199589 |
| -0.0174881 | -0.2645376 | 0.1394269 | -0.1115566 | -0.0879096 | -0.0505359 | -0.1813871 | -0.2679635 | -0.3301503 |
| -0.3739182 | -0.5249993 | -1.0276738 | -0.0564071 | -0.1346872 | 0.0412089 | -0.1019180 | -0.1321412 | -0.1955944 |
| -0.4758958 | -0.4453551 | -0.6763925 | -0.4209646 | -0.5829178 | -1.0230877 | -0.4835881 | -0.7497416 | -0.9167670 |
| -1.0231625 | -1.7681030 | -1.6639285 | 0.0401738 | -0.6844887 | -0.5037823 | -0.1419124 | -0.5302242 | -0.2186264 |
| 0.2350218 | -0.1355891 | -0.0134687 | -0.3061152 | -0.0927695 | -0.3328296 | -0.6557535 | 0.2294974 | -0.6107318 |
| -0.3160597 | -0.1089036 | -0.7440285 | 0.0204109 | 0.1076749 | -0.0404912 | 0.0156870 | 0.0455044 | -0.0102085 |
| 0.1255612 | -0.2491071 | -0.6175528 | 0.0295099 | 0.0323765 | -0.1517648 | -0.1268316 | -0.3298559 | -0.4986408 |
| 0.1774411 | 0.2442378 | -0.8118907 | -0.1618409 | -0.2363047 | -1.3650152 | -0.2302875 | -0.3874516 | -1.4786408 |
| -0.6331813 | -0.4342667 | -2.5746686 | -0.3705221 | -0.3722054 | -0.5068208 | -0.3503998 | -0.5160027 | -0.1135348 |
| -0.1987412 | 0.0993482 | 0.1701378 | -0.3918577 | 0.5188766 | 0.2239814 | -0.1889809 | 0.7681681 | 0.0657330 |
| 0.1874889 | -0.2285649 | -1.4401517 | -0.5435156 | -0.5169637 | -0.6512760 | -0.3726046 | -0.2755641 | -0.3202437 |
| -0.4762305 | 0.0985578 | 0.1175772 | -0.2613402 | 0.1020272 | 0.5685219 | -0.5309495 | 0.4353506 | 0.4653059 |
| -1.0220455 | -1.9759051 | -2.6889777 | 0.0260461 | -0.7042415 | -2.3740495 | -0.0322299 | -1.6280023 | -2.1951925 |
| -1.0844136 | -0.8141370 | -1.5376203 | -0.9956173 | -1.0050708 | -1.8590854 | -0.8421935 | -0.8818244 | -1.6873414 |

| | | | | | | | | |
|---|---|---|---|---|---|---|---|---|
| 0.4703471 | 0.4746236 | 0.5796549 | 0.2688945 | 0.1324571 | 0.7262212 | 0.3239479 | 0.1866081 | 0.3808925 |
| -1.185499 | -1.605013 | -1.749183 | -1.032372 | -1.635710 | -1.621494 | -0.957996 | -1.229167 | -1.557604 |
| -0.451963 | -0.505922 | -0.284807 | -0.188295 | -0.206493 | -0.180442 | -0.417974 | -0.302675 | -0.600585 |
| 0.0079168 | 1.5915725 | 1.7063829 | -0.371623 | -0.398604 | 0.3047871 | -0.176343 | 0.3098322 | 1.1584160 |
| -0.180422 | -1.141916 | -1.267059 | -0.072347 | -0.713077 | -0.878070 | -0.062667 | -1.148859 | -1.484854 |
| -0.399367 | 0.1118844 | 0.9183355 | -0.586670 | 0.8099342 | 0.4470250 | -0.310518 | 1.0239356 | 0.2801775 |
| -0.214393 | -0.814764 | -0.805755 | -0.376634 | -0.328990 | -1.023998 | -0.511515 | -0.688676 | -1.132690 |
| -0.831958 | -0.932403 | -0.626924 | -0.612872 | -0.913490 | -1.503795 | -0.734111 | -1.163967 | -1.715367 |
| -0.589757 | -1.107479 | -1.487575 | -0.373518 | -0.824323 | -1.794037 | -0.301386 | -1.156121 | -1.945991 |
| -0.075484 | -0.497716 | -0.363095 | -0.281892 | -0.505715 | -0.285406 | -0.292730 | -0.709506 | -0.467437 |
| -0.037413 | -0.096299 | 0.9073744 | -0.230568 | 0.1101957 | 0.4074994 | -0.322350 | 0.2521294 | 0.3120351 |
| -0.801984 | -1.462895 | -1.558130 | -0.812619 | -1.132713 | -1.494655 | -0.964594 | -1.881312 | -1.466218 |
| 0.1653782 | -0.353051 | -1.314436 | -0.205835 | -0.715676 | -0.640767 | -0.276800 | -1.250767 | -0.627263 |
| -0.183508 | -0.331749 | 0.4524538 | -0.596628 | -0.424022 | -0.773760 | -0.462426 | -0.227637 | -0.478163 |
| -1.530499 | -1.359309 | -1.938396 | -0.305290 | -0.323966 | -0.196523 | -0.458741 | -0.377918 | -0.257736 |
| 0.3436618 | 0.3665695 | -1.640023 | 0.2776098 | 0.0164291 | -0.914355 | 0.0251414 | -0.020819 | -0.273374 |
| -0.332618 | -0.227680 | 0.0798896 | -0.897712 | -1.059627 | -0.938631 | -0.760755 | -0.725846 | -1.014411 |
| -1.419981 | -2.201618 | -2.193916 | -0.517815 | -1.139516 | -2.206078 | -1.066324 | -2.161789 | -3.426645 |
| -0.514870 | -0.478053 | -0.797293 | -0.592564 | -0.746359 | -0.584839 | -0.729674 | -1.125308 | -0.867673 |
| -0.692075 | -0.691523 | -0.302120 | -0.653716 | -1.155836 | -1.657303 | -0.966677 | -1.237796 | -1.356741 |
| -1.114682 | -0.127798 | 1.3305398 | -0.160897 | -0.088460 | 0.4509314 | -0.381109 | -0.016789 | 1.2434009 |
| -0.106086 | 0.2442241 | 0.2871024 | -0.091698 | -0.068266 | 0.1754941 | -0.139139 | 0.1193523 | 0.0073217 |
| -1.062764 | -1.964051 | -2.192952 | -0.155799 | -0.735291 | -1.804666 | -0.401168 | -1.290950 | -1.756892 |
| -0.328689 | -0.165840 | -1.560238 | -0.425302 | -0.695772 | -0.745276 | -0.497434 | -0.515395 | -1.490441 |
| -0.503123 | -0.500287 | -1.564343 | -0.203604 | -0.544292 | -1.349214 | -0.171311 | -0.927392 | -1.343786 |
| -1.214603 | -1.447291 | -1.197903 | -0.462990 | -0.374324 | -0.623265 | -0.501326 | -0.585223 | -0.790170 |
| -0.783809 | -1.099696 | -1.158268 | -0.572253 | -0.698889 | -2.313089 | -0.404882 | -0.915803 | -2.218943 |
| -0.323700 | -0.598348 | -0.139560 | -0.203913 | -0.491209 | 0.2139996 | -0.180681 | -0.458181 | 0.1637171 |
| -0.325215 | -0.908310 | -1.035524 | -0.429152 | -1.014775 | -1.931620 | -0.767011 | -1.250552 | -2.200416 |
| -0.002871 | 0.5894170 | 1.1060720 | 0.1012549 | 0.7128314 | 1.1310001 | 0.1850272 | 0.8695822 | 0.6662475 |
| 0.5190785 | 1.5758881 | 2.8004752 | -0.063352 | 0.6054117 | 2.7457829 | 0.1101857 | 1.1938498 | 3.2893494 |
| -0.979665 | -1.623838 | -1.363069 | -0.732727 | -0.703720 | -0.271027 | -0.815816 | -0.785059 | -0.075774 |
| 0.1820223 | 0.1575652 | 0.7682278 | 0.0275550 | 0.065743 | 0.1115142 | -0.051369 | -0.076698 | -0.050839 |
| 0.2298937 | 0.4208122 | 0.3230232 | 0.1131425 | 0.3172762 | 0.3894481 | 0.2395564 | 0.2355282 | 0.3642299 |
| 0.2959144 | 0.5740722 | 0.7790818 | 0.1159894 | 0.2967984 | 0.5335240 | 0.2173875 | 0.7267462 | 0.6746239 |
| -0.540272 | -0.882912 | -0.657871 | -0.613792 | -0.570418 | -0.434553 | -0.524910 | -0.490048 | -0.822409 |
| 0.6526546 | 1.0823629 | 1.5969798 | 0.4112435 | 0.5986673 | 1.3276016 | 0.3472709 | 1.1402621 | 0.4491626 |
| 0.0045456 | 0.0761827 | 0.2326511 | -0.131503 | 0.0737115 | 0.7270270 | 0.0832493 | 0.3467093 | 0.6673473 |
| -0.313572 | -0.161922 | -0.250842 | -0.292328 | -0.102324 | 0.7425014 | -0.325910 | 0.2402981 | 0.7018064 |
| -0.306267 | -0.284787 | -1.753641 | -0.159492 | -0.365351 | -1.124345 | -0.203465 | -0.307796 | -1.224238 |
| -0.707615 | -1.365564 | -1.961257 | -0.544876 | -1.001210 | -1.603980 | -0.879528 | -1.393088 | -1.513142 |
| -0.667876 | -0.314610 | -0.190561 | -0.175892 | 0.0985737 | 0.1281511 | -0.493457 | 0.0224796 | 0.1238362 |
| -0.787997 | 0.1492841 | 0.5395580 | -0.263467 | 0.3017938 | 1.8063838 | -0.337743 | 0.2828717 | 1.5795174 |
| -0.030028 | 0.4758788 | 0.6112734 | 0.1453412 | 0.3927605 | 0.8622062 | -0.245920 | 0.4208362 | 0.4304566 |
| -0.071594 | -0.714194 | 0.3190769 | -0.219200 | -0.215620 | -0.424171 | -0.109301 | -0.639285 | -0.318703 |
| -0.015400 | -0.370627 | 0.0153592 | -0.179712 | 0.0024321 | -0.060263 | -0.253244 | -0.039866 | 0.0439603 |
| -0.690186 | -1.462857 | -2.004215 | -0.765808 | -0.982020 | -2.452587 | -0.702955 | -1.155181 | -2.582340 |
| -0.290461 | -1.690462 | -0.947510 | -0.408425 | -1.115697 | -2.447832 | -0.455621 | -1.424323 | -2.401652 |
| -0.023930 | -0.642872 | -1.954398 | -0.068823 | -0.261068 | -1.911621 | 0.0540188 | -0.575365 | -1.677647 |
| -0.079084 | -0.017601 | 0.0300161 | -0.141731 | -0.165821 | -0.134058 | -0.077926 | -0.150740 | -0.301527 |

| | | | | | | | | |
|---|---|---|---|---|---|---|---|---|
| 0.2001483 | 0.1234622 | -0.7599129 | 0.0693510 | 0.3716691 | 1.0001826 | -0.1046592 | 0.5016438 | 0.7276923 |
| -0.4268375 | -0.5537203 | -1.0663660 | -0.4370340 | -0.3989322 | -1.3152229 | -0.9147305 | -0.5737904 | -0.9908441 |
| -0.7910780 | 0.0117903 | -1.4686704 | -0.5545887 | -0.4229662 | -0.4208841 | -0.4055358 | -0.6519587 | -0.2219767 |
| -1.0939287 | -1.6361663 | -3.2320285 | -0.6810960 | -0.9501418 | -2.8469468 | -0.6583002 | -1.2208370 | -2.4497519 |
| -0.3429591 | -0.8384679 | -0.7949804 | -0.3261680 | -0.5926071 | -1.0526951 | -0.5341880 | -1.0665348 | -1.4076963 |
| -0.2765800 | -0.1134054 | 0.3457537 | -0.2030270 | 0.2124107 | -0.7572809 | 0.0143381 | 0.0533802 | -0.6674258 |
| 0.3089377 | 0.7928683 | 0.5450953 | -0.4358958 | 0.7065272 | 1.1390828 | -0.1608034 | 1.1409814 | 0.6841991 |
| -0.1477150 | -0.2462633 | 0.0032452 | -0.2937578 | -0.2516135 | -0.0857053 | -0.3607964 | -0.4048338 | -0.2740388 |
| -0.6094544 | -0.9188054 | -2.0458911 | 0.0039400 | 0.0930360 | -0.6538797 | -0.0358204 | -0.2696374 | -0.9441960 |
| -0.2492494 | -0.6974260 | -0.1013479 | -0.3252533 | -0.7130304 | -0.6534719 | -0.6463475 | -0.5773569 | -0.9443163 |
| -0.1803219 | -0.7190260 | -0.2221220 | -0.4989660 | -0.8665813 | -0.3145020 | -0.4255537 | -0.8828080 | -0.3562482 |
| -0.9293599 | -0.6968194 | -0.6121430 | -0.6999921 | -0.4867780 | 0.0465136 | -0.7135059 | -0.6482044 | 0.1495520 |
| 0.8559198 | 0.5274107 | -0.6676810 | 0.1977457 | -0.2614748 | -1.5623340 | 0.1469301 | -0.3640777 | -1.0212211 |
| 0.1085766 | -0.3110689 | -0.2460440 | 0.0482944 | -0.3474218 | -0.4307479 | 0.1520029 | -0.3803604 | -0.7060496 |
| -0.0779009 | -0.7262291 | -2.2783290 | -0.1606854 | -0.2432763 | -1.6590764 | -0.1624650 | -0.8257255 | -1.2526435 |
| -0.2927343 | -0.2263868 | -0.9260910 | -0.1086170 | -0.2337206 | -1.8074280 | -0.0178845 | -0.2395576 | -1.7268320 |
| 0.1595235 | 0.0439101 | -0.8962983 | 0.0809779 | -0.1958036 | -0.9055531 | 0.1701898 | -0.2051339 | -0.7153138 |
| -0.8567530 | -0.9806790 | -1.3206765 | -0.3567753 | -0.6358920 | -0.0976668 | -0.3310397 | -0.3319829 | -0.0007079 |
| -0.3120402 | -1.5093771 | -1.4341750 | -0.1626173 | -0.5786770 | -1.3458530 | -0.1645575 | -0.9346761 | -1.3738181 |
| 0.1276324 | 0.3702164 | -1.1037585 | 0.2352909 | 0.2375519 | -0.3945611 | 0.1811987 | 0.1860965 | -0.5628511 |
| -0.7007802 | -1.1330928 | -2.3556309 | -0.6079119 | -0.6730408 | -1.7107945 | -0.7770248 | -0.9162455 | -1.5484911 |
| -0.9197045 | -2.5213420 | -4.1424043 | -0.2472494 | -0.3282513 | -0.2477101 | -0.2518482 | -0.2968413 | -0.3088246 |
| -0.7811857 | -1.6265574 | -2.1279567 | -0.8694920 | -1.5276190 | -3.6242742 | -0.7539931 | -1.6582250 | -3.7140279 |
| -0.2911405 | -0.3680877 | 0.2464767 | -0.1330852 | -0.2615543 | 0.1512620 | -0.1302110 | -0.4535445 | 0.0852308 |
| 0.4492308 | -0.2479404 | -1.6616943 | -0.6103055 | -0.4982225 | -1.7236955 | -0.6475990 | -0.5553413 | -1.6132686 |
| -0.6494569 | -0.9445010 | -0.1366395 | -0.4533927 | -0.5598210 | -1.1868557 | -0.4998335 | -0.8340362 | -1.5041465 |
| -0.5280232 | -0.6926278 | -1.0724839 | -0.0826239 | -0.2932892 | -0.3487870 | -0.0754082 | -0.2669504 | -0.2380000 |
| -0.3418064 | -0.2960637 | -0.0829138 | -0.5768929 | -0.7490478 | -1.5305807 | -0.5229853 | -0.9542291 | -1.5879960 |
| 0.2581002 | 0.2701205 | 0.8605364 | -0.1604633 | 0.4955880 | 1.4410171 | -0.1669567 | 0.4039732 | 1.3478001 |
| -0.2961395 | 0.1549523 | 0.7276126 | -0.1647243 | -0.1667629 | 0.9149165 | -0.1301697 | 0.2625295 | 0.8798136 |
| 0.0015287 | -0.1367441 | -0.7432202 | -0.6040068 | -0.0520945 | -1.2806657 | -0.7825405 | -0.2367673 | -1.7588321 |
| -0.5404742 | -0.7389576 | -0.9262479 | -0.2531915 | -0.4483943 | -0.7489586 | -0.1913614 | -0.5747109 | -1.0105688 |
| -0.6755658 | -0.1591483 | 1.2658163 | -0.6647454 | -0.9674705 | -0.3058398 | -0.6372596 | -0.3320013 | -0.0862671 |
| -0.5128714 | -0.9683309 | -0.5575565 | -0.1532531 | -0.2515715 | -0.7537975 | -0.1552770 | -0.7154317 | -0.4826162 |
| -0.2751809 | -0.2381354 | -1.0033759 | -0.4352520 | -0.4410462 | -0.9032553 | -0.2251041 | -0.4042047 | -1.4665729 |
| -0.6461784 | -0.9813412 | -2.6822268 | -0.5540169 | -0.6428440 | -1.9248033 | -0.5032933 | -0.9417791 | -1.7963410 |
| -1.1810691 | -2.0542218 | -2.8360847 | -0.7918899 | -1.4168410 | -1.7770824 | -0.8289370 | -1.6448627 | -1.7184841 |
| -0.3029670 | -0.6153480 | -5.2335205 | -0.0725638 | 0.0674858 | -0.3978049 | -0.3120328 | -0.3068490 | -0.3602574 |
| -0.1925889 | 0.8269949 | 0.0367669 | -0.4549529 | -0.0940493 | -0.3798915 | -0.7003121 | -0.2255461 | -0.6229680 |
| -0.4152633 | -0.6417162 | -1.4768527 | -0.3505978 | -0.6290499 | -1.5557568 | -0.3743310 | -0.6527905 | -1.4876361 |
| -0.4061532 | -0.3007402 | -0.3502915 | 0.1824886 | 0.0302274 | 0.4832593 | 0.2042583 | 0.1749567 | 0.3685133 |
| 0.0561792 | -0.5947395 | -1.8294190 | -0.2716219 | -0.3096220 | -1.3486007 | -0.2076358 | -0.5176231 | -1.2628135 |
| -0.4528198 | -0.6332274 | -0.7171320 | -0.5403807 | -0.9737151 | -0.9538134 | -0.3052657 | -0.7445754 | -1.0712622 |
| -0.8658419 | -0.9459715 | -1.4447569 | -0.7026640 | -0.9411603 | -1.3536921 | -0.7414024 | -1.0863520 | -1.2264744 |
| -0.6274384 | -0.3373239 | -0.3171948 | -0.4791433 | -0.4449763 | -0.1730049 | -0.5932292 | -0.3694314 | -0.0105924 |
| -0.0320098 | -0.5602257 | -1.8619310 | -0.0991107 | -0.4053988 | -1.6013222 | -0.3735540 | -0.7344955 | -2.5454792 |
| -0.3898430 | -0.9076285 | -0.8624815 | -0.2330069 | -0.2514409 | -0.5384083 | -0.0267014 | -0.4017380 | -0.7334122 |
| -1.1823973 | -1.4562482 | -2.2170709 | -0.6047765 | -0.9011500 | -1.6091604 | -1.1659695 | -1.5035451 | -2.1769785 |
| -0.4040784 | -0.6158270 | 0.3533815 | -0.4029757 | -0.4684351 | -0.4043428 | -0.3953379 | -0.7918627 | -0.9778675 |
| -1.3145143 | -1.3423549 | -2.0484124 | -0.6156843 | -1.3276869 | -1.7426470 | -0.8415587 | -1.2010509 | -1.9191755 |

| | | | | | | | | |
|---|---|---|---|---|---|---|---|---|
| -0.1325613 | -0.2956807 | 1.2407772 | -0.0381397 | -0.4556653 | 0.0738487 | -0.2990727 | -0.6792111 | 0.1427752 |
| -0.3026853 | -0.3771948 | 0.3299750 | -0.5425533 | -0.0536539 | 0.2988660 | -0.4106247 | -0.0580603 | 0.2046845 |
| -0.2553419 | -0.2488415 | 0.2275531 | -0.3130661 | -0.1645702 | -0.0800310 | -0.3104488 | -0.6029326 | -0.3550589 |

| PG_543 | PG_544 | PG_545 | PG_546 | PG_547 | PG_548 | PG_549 | PG_550 | PG_551 |
|---|---|---|---|---|---|---|---|---|
| -0.884235 | -0.822848 | -1.222588 | -0.481022 | -0.594734 | -1.066592 | -0.877326 | -0.596513 | -0.243635 |
| -0.739802 | -1.339143 | -1.053013 | -1.251549 | -1.264849 | -1.411932 | -0.531583 | -0.946871 | -1.975861 |
| -0.915407 | -1.206247 | -0.186770 | -0.718636 | -0.795427 | -0.334306 | -0.545860 | -0.506099 | -1.884018 |
| -0.405871 | -0.389063 | 0.0097878 | -0.190209 | -0.110363 | 0.1173483 | -0.244301 | -0.985470 | -0.872387 |
| -0.316387 | -0.428892 | -0.785981 | -0.349823 | -0.496260 | -0.696283 | -0.381095 | -0.724718 | -1.648479 |
| -0.194747 | -0.872435 | -1.417660 | -0.332571 | -1.131685 | -1.819862 | -0.325508 | -0.676072 | -2.756533 |
| -0.437980 | -1.034501 | -0.737137 | -0.491798 | -1.198191 | -0.797281 | -0.383779 | -1.392793 | -1.048721 |
| 0.1239392 | 0.1469497 | -0.364277 | 0.1029033 | -0.028774 | -0.458248 | 0.1263432 | -0.235369 | -1.698729 |
| -0.287168 | -0.426003 | -0.889835 | -0.614611 | -0.204627 | -0.392513 | -0.368601 | 0.4329767 | -0.226292 |
| -0.399113 | -1.678594 | -0.562714 | -0.925490 | -1.801870 | -0.253017 | -0.535041 | -1.428331 | -1.074824 |
| -0.189787 | -0.259220 | -0.080761 | -0.214567 | -0.394440 | -0.321776 | -0.433858 | -0.689614 | -1.019878 |
| -0.146033 | -0.229299 | -0.426263 | -0.152096 | -0.541782 | -0.552163 | -0.249149 | -0.729707 | -1.073106 |
| -0.261384 | -1.252609 | -0.314152 | -0.361135 | -1.431403 | -2.046207 | -0.407468 | -0.938965 | -2.578737 |
| -0.193350 | 1.0640337 | 2.3589689 | 0.1128332 | 1.8996457 | 2.7829183 | -1.129268 | 1.9877205 | 4.3903702 |
| -0.138979 | -0.186828 | 0.0835762 | -0.000248 | 0.1534778 | 0.4964700 | -0.257649 | -1.174112 | -0.117632 |
| -0.316904 | 1.9894785 | 1.3403534 | 0.5433241 | 2.3464554 | 1.8045913 | -0.403168 | 2.5691674 | 2.4585666 |
| -1.358130 | -2.016735 | -1.917562 | -1.429980 | -1.467396 | -1.982279 | -0.916094 | -1.561367 | -0.803591 |
| -1.140730 | -2.172276 | -1.388436 | -1.080984 | -1.848066 | -1.744128 | -1.088133 | -2.720609 | -1.389271 |
| -1.465890 | -1.530198 | -1.571749 | -1.697542 | -1.791553 | -1.437347 | -0.692390 | -0.675728 | -1.028217 |
| -0.655708 | -2.188043 | -2.958856 | -1.214540 | -3.115242 | -3.936222 | -0.708998 | -0.546559 | -1.071506 |
| -0.015814 | 0.4167616 | 0.2045875 | 0.2185066 | 0.3547983 | 0.2984390 | -0.403602 | 0.0096467 | 0.3601950 |
| -0.111954 | -0.241129 | -0.287866 | -0.047835 | -0.632192 | -0.299195 | -0.350374 | -0.426535 | -0.393822 |
| 0.1351176 | 0.3897159 | 1.4181143 | 0.1477312 | 0.8309086 | 1.3753902 | -0.062510 | 1.2447552 | 1.1210683 |
| -0.330226 | -0.493567 | -1.825814 | -0.460413 | -1.007162 | -2.570924 | -0.402868 | -0.728693 | -2.116054 |
| -0.251783 | -1.654341 | -1.877586 | -0.796442 | -2.501488 | -2.344931 | 0.0433345 | -0.834836 | -3.499944 |
| -0.068752 | 0.8040655 | 0.9187429 | 0.1767918 | 0.6941291 | 1.3174376 | -0.022106 | 0.6221544 | 1.3976872 |
| -0.090556 | -0.253787 | -0.480343 | -0.147291 | -0.322244 | -0.440579 | -0.115438 | -1.125134 | -0.547743 |
| -1.024986 | -1.389466 | -1.834407 | -1.411247 | -1.420210 | -2.302476 | -1.229786 | -1.204231 | -2.670551 |
| 0.1014411 | -0.146724 | -0.973023 | 0.0426836 | -0.346983 | -2.327181 | -0.715688 | -1.546809 | -3.558135 |
| -0.200844 | -0.266733 | -0.203631 | -0.195641 | -0.346201 | -0.270876 | -0.000061 | -0.938543 | -1.028444 |
| -0.163136 | -0.546533 | -0.111336 | -0.207052 | -0.356648 | -0.917433 | 0.9720595 | 0.1083798 | -2.947439 |
| 0.7278565 | -0.128434 | -1.588222 | 0.2118735 | -0.496170 | -1.781765 | 0.2271251 | -0.629275 | -0.707487 |
| -0.496199 | -0.042118 | -0.392221 | -0.506095 | 0.3912564 | -0.239924 | -0.808545 | -0.965207 | 0.5401470 |
| -0.070417 | -0.637601 | -1.852581 | -0.204621 | -1.027317 | -3.523053 | -0.366623 | -1.737015 | -2.969560 |
| -0.281373 | -0.247968 | -1.251373 | -0.403660 | -0.685959 | -0.833892 | -0.123646 | -0.234534 | -0.081389 |
| -0.895007 | -1.262159 | 0.1522486 | -1.690405 | -1.232373 | -0.056441 | -0.114103 | -0.787273 | -0.801636 |
| 0.0767954 | -0.951693 | -0.742406 | -0.362290 | -1.088328 | -1.569418 | 0.1391041 | -1.126115 | -2.227000 |
| 0.2105913 | 2.0444028 | 0.1571209 | 0.8690977 | 2.5683285 | -0.001304 | -0.184095 | 3.2504517 | -0.137030 |
| -0.764401 | -0.734960 | -0.445964 | -0.684626 | -0.634854 | -0.567088 | -0.426190 | -1.422938 | 0.4790939 |
| -1.129946 | -2.514929 | 0.0276231 | -1.342539 | -1.522673 | -0.286806 | -1.473896 | -1.827464 | -0.553365 |
| -0.139179 | -0.020918 | 0.1775171 | -0.067899 | 0.1452546 | 0.0190423 | -0.129395 | -0.126657 | 0.8645275 |
| -0.154087 | -0.591425 | -2.021326 | -0.327415 | -0.951129 | -2.591227 | -0.440619 | -0.959644 | -2.179228 |
| -0.157032 | -0.101076 | -0.469634 | -0.158015 | -0.080253 | -0.577464 | -0.255913 | -0.703167 | -1.163301 |
| -0.025650 | 0.1684690 | -0.370377 | 0.0192014 | -0.207928 | -0.592767 | -0.166788 | -1.539249 | -1.766799 |
| -0.001024 | 0.4550790 | 0.5562036 | 0.1476309 | 0.7240233 | 1.0930172 | -0.039485 | 0.8795281 | 1.0186151 |
| -0.408598 | -0.526417 | 1.0865354 | 0.0510979 | -0.310267 | 2.6036234 | -0.218886 | 0.5125582 | 2.6437361 |
| -0.167766 | 0.0971165 | -0.147360 | 0.2560017 | -0.075179 | -0.422700 | 0.0797482 | -0.368031 | 0.3976731 |

| | | | | | | | | |
|---|---|---|---|---|---|---|---|---|
| -0.248688 | -0.307748 | -0.029907 | -0.316974 | 0.1043458 | 0.0689419 | -0.392187 | -0.811413 | -0.571693 |
| -0.847317 | -0.945201 | -2.526860 | -0.704468 | -0.784149 | -3.496356 | -0.628342 | -0.794737 | -2.814484 |
| -0.523174 | 0.1986102 | 0.1484730 | -0.264420 | 0.4044930 | 0.4292334 | -0.599105 | -0.324118 | 0.4281605 |
| 0.3843646 | 2.4633423 | 0.7320075 | 0.9006251 | 3.8388796 | -0.837806 | -0.504773 | 3.0739321 | 2.4778292 |
| -1.167288 | -1.834643 | -1.316881 | -1.646743 | -1.217437 | -1.248809 | -1.070091 | -1.136066 | -1.247577 |
| -0.137162 | -0.237754 | -0.073874 | -0.231388 | -0.396342 | 0.4168707 | -0.370038 | 0.1191506 | 0.3182309 |
| -0.452757 | -0.656660 | -0.549678 | -0.473814 | -0.752900 | -1.071414 | -0.118203 | -0.773032 | -1.324216 |
| 0.0573179 | 0.4258515 | 2.3526046 | -0.113944 | 0.5954731 | 2.7897612 | -0.833918 | 1.5221045 | 2.0939728 |
| -0.555644 | 2.9879722 | -0.294030 | 0.2142221 | 3.3970112 | -1.340448 | 0.9618782 | 3.2585525 | -1.808344 |
| -0.785743 | -0.396478 | 0.4025490 | -0.644701 | -0.159933 | 0.6038922 | -0.883670 | -0.097905 | 0.0997926 |
| -1.182778 | -1.941315 | -1.243944 | -1.057285 | -1.687219 | -1.375126 | -0.413950 | -1.423660 | -1.105242 |
| 0.1824159 | 2.9469663 | 2.2154734 | 1.4246781 | 2.8686815 | 2.4572232 | -0.149915 | 3.2055257 | 2.6794104 |
| -0.050259 | 2.2891191 | 1.9866170 | 0.9662320 | 2.7132376 | 1.7177084 | -0.607353 | 1.6074158 | 0.9814083 |
| -0.415792 | -0.487407 | -0.890065 | -0.316714 | -0.338408 | -0.776687 | -0.370941 | -0.461341 | -1.224271 |
| -1.176010 | -1.760680 | -0.858720 | -1.455820 | -1.935228 | -1.546376 | -0.955991 | -2.237024 | -2.219568 |
| -0.454320 | -1.189959 | -0.672339 | -0.834459 | -1.389516 | -0.249545 | -0.201079 | -1.289132 | -0.883048 |
| -0.493715 | -0.452228 | -1.595886 | -0.474232 | -0.892871 | -2.245581 | -0.292177 | -0.628327 | -2.089843 |
| -0.161671 | -0.431622 | -0.719734 | -0.283310 | -0.974366 | -0.955335 | -0.419392 | -0.746170 | -0.551453 |
| -0.356356 | -0.405052 | -1.021741 | -0.258707 | -0.494873 | -1.456016 | -0.647350 | -1.063848 | -0.838312 |
| -0.022061 | -0.263047 | -0.525762 | -0.274970 | 0.1190316 | -0.705639 | 0.0384509 | -1.185657 | -1.359938 |
| -0.034161 | 0.1979755 | -1.093207 | -0.143139 | 0.2379310 | -1.106280 | 0.1778722 | -0.249446 | 0.8832498 |
| -0.628535 | -0.920435 | -0.802849 | -0.812423 | -1.072935 | -1.624980 | -0.549436 | -1.207892 | -1.500517 |
| 0.0537574 | -0.352850 | 0.1274451 | 0.0971522 | -0.484333 | -0.170659 | 0.0288806 | -0.061002 | -0.304590 |
| 0.1308960 | 0.4984089 | 0.5976315 | 0.0202504 | 0.3506737 | 0.3553956 | -0.021597 | -1.953373 | -0.717112 |
| -0.858024 | -1.206273 | -1.927564 | -1.140260 | -1.529348 | -1.977414 | -0.903742 | -1.350518 | -2.118837 |
| -0.218374 | -0.755417 | -0.245027 | -0.519369 | -1.066206 | -0.076288 | -0.096183 | -0.792779 | -0.885351 |
| -1.050942 | -1.606591 | -1.600522 | -1.229675 | -2.027671 | -1.821066 | -0.778819 | -2.478566 | -1.264176 |
| 0.7403592 | 2.0789949 | 3.0318301 | 1.0668387 | 2.1037800 | 3.2574963 | 0.1866223 | 1.4567513 | 3.3588007 |
| 0.2597309 | 0.8699117 | 2.1171451 | 0.3514673 | 1.0685971 | 2.4914500 | -1.058950 | 1.0354378 | 3.3103734 |
| 0.1618698 | -0.226422 | -1.001935 | -0.001686 | -0.083302 | -1.461877 | -0.055421 | -0.466886 | -2.517983 |
| -0.409633 | -1.084594 | 0.2285168 | -0.509969 | -1.299554 | 0.0950061 | -0.332009 | -1.383691 | -1.743747 |
| -0.212668 | -0.180012 | 0.1310998 | -0.189425 | -0.324716 | 0.1040946 | -0.305806 | -1.483413 | -0.826000 |
| -0.514139 | -1.328696 | -1.297594 | -0.802175 | -1.489388 | -1.443100 | -0.666272 | -1.471082 | -1.135535 |
| -0.208172 | -0.627088 | -0.983032 | -0.396199 | -0.917421 | -1.304225 | -0.615603 | -0.938730 | -2.784947 |
| -0.085847 | 0.7206318 | 0.8034229 | 0.1928406 | 0.5359009 | 0.6243621 | -0.225166 | 0.9423250 | 2.2602039 |
| -0.275612 | -0.230382 | -1.413485 | -0.158825 | -0.380664 | -1.500939 | 0.1039020 | -0.306064 | -1.063335 |
| 0.0533552 | -0.172704 | -0.335985 | -0.107787 | -0.161164 | -0.033985 | -0.222831 | -0.188016 | -0.176342 |
| -0.137659 | -0.621230 | -1.898020 | -0.399124 | -1.209715 | -2.770092 | -0.033173 | -0.436046 | -2.784799 |
| 0.0333058 | -1.108892 | -0.630806 | -0.326467 | -1.064435 | -0.981265 | -0.087497 | -0.475858 | -2.966413 |
| -1.001445 | -1.662078 | -1.178969 | -1.008219 | -1.267794 | -1.449046 | -0.686050 | -1.177456 | -2.220401 |
| -0.811023 | -1.061216 | -1.372210 | -0.869931 | -0.812034 | -1.549602 | -0.708646 | -0.607104 | -2.087617 |
| -0.141245 | -2.619367 | -0.201382 | -0.864838 | -2.160469 | -0.520469 | -0.289804 | -2.319070 | -2.711518 |
| 0.7247668 | 3.5251655 | 1.9744168 | 2.7294158 | 4.5131256 | 1.2902406 | 0.0992648 | 3.3306044 | 2.3260253 |
| -0.115800 | -0.066599 | -0.381495 | -0.008597 | -0.184949 | -0.542368 | -0.172843 | -0.182703 | -0.452681 |
| -0.310296 | -0.689743 | -0.967108 | -0.300185 | -0.561121 | -1.038729 | -0.295735 | -1.289680 | -0.404727 |
| -0.587668 | -0.671589 | -0.820860 | -0.722976 | -0.729552 | -0.559801 | -1.132180 | -1.508414 | -1.543962 |
| -0.065979 | 5.5591902 | 3.4427903 | 1.9130581 | 7.0987362 | 5.1105666 | 0.0803330 | 8.3727990 | 8.1306561 |
| -0.176389 | 1.5006822 | 1.2589056 | 0.2687026 | 1.8251308 | 1.5319483 | -0.287895 | 1.2947106 | 1.4406184 |
| -0.131846 | -0.163410 | -3.089873 | -0.152599 | 0.1315623 | -3.880070 | -0.870474 | -1.941222 | -1.170089 |
| 0.0152459 | -0.092612 | -0.123146 | 0.0423445 | 0.0916505 | -0.004816 | 0.1349301 | 0.1245397 | -1.407758 |

| | | | | | | | | |
|---|---|---|---|---|---|---|---|---|
| -0.0081492 | -0.2183504 | -2.8427402 | -0.0102309 | -0.2017268 | -2.6676354 | -1.1658561 | -1.5626984 | -4.0366279 |
| -0.4183318 | -0.0756018 | 0.2031694 | -0.2035679 | -0.2246023 | 0.2597956 | -0.3114197 | -0.3618767 | 0.1790291 |
| -0.3362105 | -0.9016249 | -0.1359905 | -0.2885976 | -1.5211661 | -0.5571247 | -0.0923519 | -0.7208160 | 0.2496364 |
| -0.2112674 | -0.0553611 | -0.3143566 | -0.1618235 | -0.1329413 | -0.2677680 | -0.3209211 | -0.4585294 | 0.3081941 |
| -0.5749360 | -1.4799420 | -1.5835119 | -0.8443740 | -2.0251926 | -1.4050907 | -0.6136522 | -2.254605 | -1.1983365 |
| -0.0841373 | -0.7989392 | -1.0485040 | -0.2022252 | -0.7554763 | -1.0606909 | 0.4553373 | 0.5456232 | -1.8976587 |
| -0.5375936 | -0.1453913 | -0.3218349 | -0.3318257 | -0.6259207 | -0.2574444 | -0.3165108 | -0.9076040 | -0.7803267 |
| -0.2554595 | 0.1107590 | -0.1408555 | -0.1861061 | 0.0465102 | -0.2129125 | 0.3187824 | -0.0480966 | 0.4221398 |
| -0.0143980 | -0.2559376 | -0.1912138 | -0.4433618 | -0.2604503 | -0.3803579 | -0.0906924 | -0.3445477 | -0.3871638 |
| 0.4767468 | 1.1619323 | 0.5177124 | 0.5217348 | 1.2848032 | 1.1127220 | -0.1103718 | 0.3893107 | 1.5321489 |
| -0.0361413 | -0.3104597 | -0.7975294 | -0.1155792 | -0.4057503 | -0.4974472 | 0.1159388 | -0.8840093 | -2.2850950 |
| -0.0403027 | -0.9642755 | -0.2368291 | -0.4956201 | -1.2986280 | -1.1259135 | -0.7361452 | -1.1969095 | -1.4538642 |
| 0.0638913 | -0.8666543 | -1.5280947 | 0.0255301 | -1.3160648 | -3.8625601 | -0.0380839 | -0.7574191 | -3.8178197 |
| -0.3754701 | -0.9410638 | -0.8743866 | -0.5137607 | -1.2682855 | -2.1122864 | -0.2776709 | -0.6064417 | -1.4463874 |
| -0.4503283 | -0.8171046 | -0.1295495 | -0.3440731 | -0.6554065 | -0.3000089 | -0.1810449 | -0.6861893 | -0.7413988 |
| -0.1762392 | 0.1040769 | -0.1185228 | 0.0445656 | -0.1413190 | -0.1625116 | -0.1985666 | 0.0448752 | -0.2582946 |
| -0.0304976 | -1.4971742 | -1.1642170 | -0.5560733 | -1.6480260 | -1.2756771 | -0.5825696 | -1.0332189 | -2.0965321 |
| -0.1109801 | -0.1837555 | -0.2060323 | -0.0747627 | -0.2481584 | -0.0182005 | -0.3679513 | -0.0108491 | -0.0557865 |
| -0.5785602 | -0.9260462 | -0.5093162 | -0.4866542 | -1.2862476 | -0.5686861 | -0.8026909 | -1.4880463 | -1.5412838 |
| -0.4033794 | -0.5705224 | 1.1266936 | -0.5117626 | -0.3869716 | 1.2411444 | -0.4968403 | 0.0388506 | 1.4291367 |
| -0.3163562 | -0.1000286 | -0.5427403 | -0.1519632 | -0.3100005 | -0.5966778 | -0.4497793 | -0.1379952 | -0.0308384 |
| -0.6395713 | -0.8632923 | -0.4006405 | -0.3528195 | -0.8473787 | -0.3875497 | -0.1993058 | -0.5792019 | -1.1638103 |
| 0.5490704 | 0.3963342 | -3.8138740 | 0.5926484 | 0.1865787 | -4.3509933 | -0.0372340 | -0.6429362 | -4.8060469 |
| -0.6686842 | -1.1740296 | -1.6261113 | -0.5199631 | -0.9717071 | -1.6448005 | -0.4837328 | -0.8471592 | -1.9419423 |
| 0.0707129 | -1.6334114 | -3.1238254 | -0.4163207 | -1.2822603 | -2.7854274 | -0.7084074 | -0.9269453 | -0.7606335 |
| -0.4273426 | -0.6126450 | -0.7712094 | -0.4705070 | -1.1523661 | -1.4274324 | -0.2443584 | -1.4929399 | -0.7781969 |
| 0.0943428 | -0.0737959 | -0.2620819 | 0.0864709 | -0.4371916 | -0.2426203 | 0.1859908 | -1.2262737 | 0.0561824 |
| -0.5065991 | -0.2378012 | -0.3273718 | -0.3937093 | -0.3256534 | -0.0666055 | 0.0603079 | 0.5831879 | -0.1883805 |
| 0.0811256 | -0.9278369 | -0.9229604 | -0.1447153 | -1.3893804 | -1.5247919 | 0.1130412 | -0.8947391 | -2.6887613 |
| -1.1271306 | -0.8602753 | -1.1386049 | -1.6002777 | -0.4478814 | -1.5881431 | -1.3068631 | -1.4833349 | -0.1849957 |
| -0.8683511 | -1.7543163 | -1.7161874 | -0.9988647 | -1.5826694 | -1.5238342 | -0.8466559 | -1.1190527 | -2.6363959 |
| -0.1387750 | -0.5925999 | -1.0473454 | -0.1601214 | -0.8232801 | -1.2464041 | -0.1678979 | -0.0411346 | -0.1835691 |
| -1.5662880 | -1.5137747 | -1.4296325 | -1.2228684 | -1.4030064 | -1.2065981 | -1.0368993 | -0.6994744 | -0.4813939 |
| 0.3688641 | 0.5231003 | -0.2635764 | 0.3199397 | 0.4379032 | -0.6258209 | 0.0960044 | -1.3340888 | -0.3448012 |
| -0.1543052 | -0.4812844 | -1.0150316 | -0.3249348 | -0.6118336 | -1.8719134 | -0.1127229 | -0.8087826 | -2.3414328 |
| -0.2000898 | -0.6058758 | -0.8085058 | -0.5749461 | -0.7374336 | -0.6883559 | -0.5799614 | -0.8659174 | -1.3000849 |
| -0.2619507 | -0.3897223 | -0.2148735 | -0.3646889 | -0.6426427 | -0.3336392 | -0.2628983 | -0.6198904 | -0.2796041 |
| -0.7394674 | -0.9243931 | -0.7523350 | -0.6378991 | -0.5867612 | -0.6904470 | -0.6525342 | -0.9657740 | 0.0483475 |
| -0.5941583 | -0.7101331 | -0.0532985 | -0.9086071 | -0.9119082 | -0.3494352 | -0.4575763 | -0.7476006 | -0.5879019 |
| -0.3537635 | -0.7745637 | -0.7003717 | -0.2883476 | -0.4568931 | -0.9236191 | -0.6982624 | -1.4195418 | -0.8160534 |
| -1.0086773 | -0.8306065 | 0.0623334 | -1.2347660 | -1.2641889 | -0.4927650 | -1.0742097 | -0.6602927 | 0.9543943 |
| -0.1141977 | -0.1667359 | -0.4112434 | -0.0235829 | -0.1088193 | -0.7485096 | -0.2194506 | -0.3661071 | -1.1393246 |
| -0.2171118 | -0.2638761 | -0.2823059 | -0.2421839 | -0.3826377 | -1.0642870 | -0.3555257 | -1.1077718 | -0.9075002 |
| -0.4103019 | -0.8441472 | -0.6272290 | -0.4100791 | -0.8453022 | -0.8867826 | -0.5793590 | -0.7422216 | -1.5760407 |
| -0.1317837 | -0.3156380 | -2.8284452 | 0.0442898 | -0.4528288 | -2.7170168 | 0.0876091 | -0.2088144 | -1.9536060 |
| -0.2442872 | 0.0242530 | -0.2321819 | -0.1558895 | 0.2133793 | -0.1826078 | -0.3331207 | 0.4054424 | 0.4905802 |
| -0.5542465 | -0.8510748 | -1.4500589 | -0.5294682 | -0.7061092 | -1.6303973 | -0.2301588 | -0.6792204 | -0.7961551 |
| -0.7582708 | 0.0541013 | 0.1660344 | -0.7624351 | 0.5947299 | 0.2380819 | -0.3083462 | 0.1241894 | -0.1828745 |
| -0.3830284 | -1.5072797 | -1.6181913 | -0.5528738 | -2.5389437 | -2.5574803 | 0.1206616 | -1.1914549 | -4.6399349 |
| -0.9762682 | -1.3182967 | -1.7037112 | -1.3403054 | -1.0929314 | -2.1067664 | -1.0346482 | -1.1088241 | -1.9466127 |

| | | | | | | | | |
|---|---|---|---|---|---|---|---|---|
| 0.0408796 | 0.2580312 | 0.0616354 | 0.1625625 | 0.1492288 | 0.0623394 | 0.1137025 | -1.558752 | 0.2722388 |
| -1.350402 | -1.910626 | -1.456270 | -1.355666 | -2.153241 | -2.035347 | -1.088987 | -1.922666 | -2.540919 |
| -0.274079 | -0.223723 | -0.286925 | -0.282895 | -0.412434 | -0.186044 | -0.647907 | -0.667797 | 0.2714369 |
| 0.5800720 | 0.9592245 | 0.6009845 | 0.8957551 | 1.1926056 | 0.2024437 | 0.9558365 | 1.2286080 | 0.2501627 |
| -0.334097 | -1.249449 | -1.559682 | -0.310492 | -1.154286 | -1.764639 | -0.905966 | -1.630725 | -1.875068 |
| 0.0985348 | 1.7114518 | 0.3396489 | 0.8558274 | 1.5960394 | 0.7042109 | 0.1113306 | 1.3882164 | 2.2425784 |
| -0.578619 | -0.635775 | -0.638477 | -0.519552 | -0.865255 | -0.478999 | -0.374941 | -1.121280 | -0.899338 |
| -0.657503 | -1.069392 | -0.696431 | -0.809840 | -1.545858 | -0.745497 | -0.799056 | -0.993515 | -1.554062 |
| -0.282846 | -1.334915 | -1.697680 | -0.612639 | -2.056163 | -1.842688 | -0.597541 | -2.008105 | -2.168340 |
| -0.716532 | -0.539245 | -0.704847 | -1.057181 | -0.519011 | -0.700271 | -0.375927 | -0.691353 | -0.184529 |
| -0.347243 | 0.1452551 | 0.2938199 | -0.155800 | 0.2291209 | 0.6462885 | -0.407894 | -0.038061 | 1.1176918 |
| -0.603685 | -1.133656 | -0.853439 | -0.950196 | -1.310208 | -0.876659 | -0.718257 | -0.815113 | -2.077591 |
| -0.456096 | -1.355297 | -0.861174 | -0.646864 | -1.137827 | -0.641076 | -0.233561 | -0.607759 | -2.635088 |
| -0.839838 | -0.657575 | -0.879953 | -0.727226 | -0.426422 | -0.089709 | -0.355658 | -0.407807 | -0.163453 |
| -0.245617 | -0.333120 | -0.357344 | -0.455256 | -0.291174 | -0.119410 | -0.339289 | -0.542293 | 0.0737136 |
| -0.066372 | -0.387706 | -2.393106 | -0.181578 | -0.265245 | -2.567042 | -0.502258 | 0.1984903 | -0.180261 |
| -0.536286 | -0.411512 | -0.084240 | -0.556020 | -0.512641 | -0.025820 | -0.275584 | 0.4890218 | 0.8951389 |
| -0.217164 | -1.678248 | -0.604865 | -1.182152 | -2.737397 | -1.142457 | -0.574294 | -1.419255 | -2.711316 |
| -0.625628 | -0.995735 | -0.205604 | -0.772029 | -0.972500 | -0.740238 | -0.435885 | -0.975380 | -1.125322 |
| -0.319010 | -0.600931 | -0.780611 | -0.351973 | -0.920726 | -1.470738 | -0.195610 | -1.330350 | -2.281512 |
| -0.238635 | -0.141436 | -0.125308 | -0.142566 | -0.335242 | 0.0457591 | -0.335093 | 0.8700921 | 1.5637794 |
| -0.403112 | 0.1315743 | -0.174471 | 0.1082558 | -0.210984 | -0.361497 | -0.710448 | -0.806605 | -0.683002 |
| -0.612746 | -0.947003 | -0.959221 | -0.687540 | -1.131804 | -1.620803 | -0.011915 | -0.476385 | -1.991069 |
| -0.225915 | -0.166293 | -0.142078 | -0.274597 | -0.080026 | -0.199067 | -0.219994 | -0.713056 | -1.858929 |
| 0.1265061 | -0.882428 | -1.096645 | -0.232903 | -1.001105 | -1.599076 | -0.094206 | -0.697907 | -1.944102 |
| -0.306173 | -0.682959 | 0.0780764 | -0.291277 | -0.669203 | -0.144325 | 0.0378319 | -0.893331 | -1.489183 |
| -0.643199 | -1.287032 | -1.044567 | -0.772623 | -1.346409 | -1.252903 | -0.614970 | -0.735762 | -1.711037 |
| -0.428597 | -0.811667 | -0.323143 | -0.791744 | -1.166603 | -0.298825 | -0.290044 | -0.693712 | -0.988157 |
| -1.088947 | -1.356916 | -0.161598 | -1.201380 | -1.725747 | -1.074113 | -1.171294 | -1.343497 | -2.087676 |
| -0.081597 | 0.7806369 | 0.8472095 | 0.1732470 | 0.8153117 | 1.1676873 | -0.470371 | 0.2667675 | 0.9653453 |
| -0.014262 | 1.2389998 | 2.0871629 | 0.3483603 | 1.8169999 | 2.3838421 | -0.482377 | 1.0393882 | 3.4169513 |
| -0.305401 | -0.872946 | -1.739257 | -0.471940 | -1.449205 | -1.587062 | -0.842432 | -0.613914 | -1.536784 |
| -0.299425 | -0.150052 | 0.0801889 | -0.315917 | -0.248851 | 0.0053073 | -0.304825 | -0.733173 | 0.4316060 |
| 0.1085901 | 0.2666137 | 0.0445140 | 0.1533205 | 0.1569977 | -0.019880 | -0.091396 | -0.613572 | 0.3254480 |
| 0.3571262 | 0.6293736 | 0.8932653 | 0.5219783 | 0.8620296 | 0.8459572 | 0.2123777 | 0.3572879 | 1.0386060 |
| -0.506743 | -0.613888 | -0.464028 | -0.413859 | -0.563970 | -0.202076 | -0.592280 | -1.440661 | -0.978827 |
| -0.188029 | 0.2600596 | 0.6012840 | 0.1824698 | 0.1632760 | 0.7998433 | -0.175416 | -0.813397 | 0.3086177 |
| -0.230162 | -0.054571 | -0.769727 | -0.125338 | -0.010471 | -0.126367 | -0.676517 | -0.123306 | 0.8279037 |
| -0.355555 | -0.487937 | -0.538819 | -0.313562 | -0.492552 | -0.799456 | -0.512576 | -0.504953 | -0.953799 |
| -0.471570 | -0.618876 | -1.295459 | -0.491833 | -0.661170 | -1.586916 | -0.474104 | -1.590222 | -1.849791 |
| -0.768274 | -1.121764 | -0.598727 | -0.863297 | -0.291345 | -0.730379 | -0.497428 | -1.509098 | -1.755670 |
| -0.500240 | 0.1957387 | 0.3979302 | -0.375517 | 0.3519960 | 0.4479461 | -0.265395 | 0.0634829 | 0.4912506 |
| -0.140560 | 0.4791107 | 0.7217961 | 0.2257483 | 0.1801118 | 0.8478239 | -0.554984 | -0.686371 | 0.9808079 |
| -0.239006 | 0.2010034 | 0.7714856 | -0.136554 | 0.3108497 | 0.6779003 | -0.690852 | -0.314353 | 0.2480704 |
| -0.239732 | -0.359577 | -1.044185 | -0.337989 | -0.688358 | -0.801874 | -0.266343 | -0.423230 | -0.250212 |
| -0.312078 | -0.291642 | -0.401110 | -0.223656 | -0.277942 | -0.121502 | -0.347604 | -0.663647 | -0.507622 |
| -0.416171 | -0.817197 | -1.009045 | -0.427042 | -1.167526 | -1.593892 | -0.159663 | -0.825727 | -1.867506 |
| -0.695559 | -1.581059 | -1.394732 | -0.922229 | -1.753143 | -1.326687 | -0.663762 | -1.703809 | -0.477060 |
| -0.124800 | -0.473588 | -1.538789 | -0.119341 | -0.822332 | -1.811893 | 0.1600330 | -0.235488 | -2.158033 |
| -0.429223 | -0.284748 | -0.258743 | -0.356158 | -0.537419 | -0.585068 | -0.452794 | -1.204817 | -0.754483 |

| | | | | | | | | |
|---|---|---|---|---|---|---|---|---|
| -0.0685713 | -0.0739422 | 0.5261568 | -0.1707564 | 0.0013996 | 0.5900388 | -0.3209940 | -1.2214141 | 0.1855861 |
| -0.6180715 | -0.5932300 | -0.6565576 | -0.6816763 | -0.7325287 | -0.6178290 | -0.5018442 | -0.6779757 | -2.2430556 |
| -0.9355667 | -0.6242143 | -0.8939142 | -0.8153625 | -0.2582249 | -1.9265474 | -0.7362433 | -1.3117948 | -1.4611017 |
| -0.5719830 | -1.4882096 | -1.4606957 | -0.7742612 | -1.5352038 | -1.2429446 | -0.3897342 | -1.4254481 | -3.0384486 |
| -0.5569796 | -0.6058548 | -0.8579818 | -0.6353902 | -0.9559672 | -1.4451494 | -0.1734865 | -0.7900041 | -2.3478131 |
| -0.0099355 | 0.0306098 | -1.2740328 | -0.0418871 | -0.4865821 | -1.3688300 | -0.1057343 | -0.1890926 | -0.4102602 |
| 0.0187437 | 1.1740562 | 1.6641740 | 0.2845877 | 1.2196812 | 1.3514626 | 0.1222141 | 1.6977128 | 1.8835995 |
| -0.0990328 | -0.5302300 | -0.3480582 | -0.3816646 | -0.4128047 | -0.2356940 | -0.3105535 | -0.3814005 | 0.0680708 |
| -0.4778038 | -0.6832220 | -0.9557206 | -0.5410047 | -0.1849090 | -1.2353993 | -0.0624720 | -0.8083289 | -1.9677666 |
| -0.3781354 | -0.8506440 | -0.2900449 | -0.3385576 | -0.6931708 | 0.0623757 | 0.0794774 | -0.5481216 | -0.0765961 |
| -0.7010083 | -0.4650305 | 0.1100380 | -0.6855761 | 0.0939697 | -0.4329064 | -0.2022486 | -1.4326694 | 0.4916891 |
| -0.6626785 | -1.0802386 | -0.4100781 | -0.8577329 | -1.2489564 | -0.8292038 | -0.6673027 | -1.2162469 | -1.2964857 |
| -0.4945094 | -0.3529751 | -0.3112639 | -0.1821835 | -0.4369426 | -0.4534935 | -0.0972227 | 0.6996707 | -4.0194789 |
| 0.0362398 | -0.3039178 | -0.8376096 | -0.1687436 | -0.6002125 | -0.9165458 | -0.1061826 | -1.1567862 | -0.6427718 |
| -0.0349019 | -0.3428438 | -1.3804638 | -0.2456424 | -0.6630044 | -1.7008289 | -0.3158471 | -0.5637017 | -2.9763551 |
| -0.3812695 | -0.9735385 | -1.0014093 | -0.4242772 | -0.9984070 | -1.4191225 | -0.1982129 | -0.5563467 | -0.8104380 |
| -0.2134833 | -0.2967832 | -0.6715821 | -0.2100992 | -0.5717102 | -1.3505603 | 0.0014069 | -1.5569005 | -2.3786101 |
| -0.2833168 | -0.8493284 | -0.5232459 | -0.4591680 | -0.3542878 | -0.2733065 | -0.3131055 | -0.9608123 | -1.0540369 |
| -0.5595773 | -1.3364573 | -1.5279227 | -0.8735864 | -1.8427782 | -2.6163503 | 0.3596030 | -0.7306258 | -1.5814033 |
| -0.1708777 | -0.0027187 | 0.3876649 | -0.0039042 | 0.2560115 | 0.0853477 | 0.1528537 | -1.6097716 | -0.3205297 |
| -0.8225129 | -1.3577075 | -1.8883649 | -1.0824679 | -1.1403495 | -1.3806071 | -0.7451551 | -0.7388654 | -1.7250437 |
| -0.5428900 | -0.4731542 | -1.0352803 | -0.7423398 | -0.5685259 | -0.9052592 | 0.5087586 | -0.5081137 | -3.2461921 |
| -0.5386931 | -1.8267872 | -2.4758891 | -0.7731363 | -2.0413727 | -2.3336597 | -0.1300150 | -0.1264706 | -2.6403463 |
| -0.1141599 | -0.1503963 | -0.2097066 | 0.0131181 | -0.2302188 | -0.2186436 | -0.0459290 | -0.5800907 | 0.3805243 |
| -0.2262492 | -0.3052680 | -0.8829862 | -0.2033208 | -0.9790204 | -0.8215588 | -0.3604753 | -0.1760882 | -1.6920535 |
| -0.7249456 | -0.9911774 | -0.3365411 | -0.6495383 | -0.8792139 | -0.1453375 | -0.7550709 | -0.4904269 | -0.8459192 |
| 0.0317083 | -0.1161320 | -1.6262916 | 0.0076799 | -0.3432752 | -2.0064208 | -0.0431642 | -0.3242526 | -1.1067759 |
| -0.7032903 | -0.8467576 | -1.2995134 | -0.6548950 | -0.9929635 | -1.1241390 | -0.3363319 | -0.5724747 | -0.8710006 |
| 0.1241319 | 0.0422942 | -0.0291388 | -0.0457181 | -0.0513415 | -0.0087657 | -0.3310154 | -0.0546221 | 0.3536877 |
| -0.0066422 | 0.1323986 | 0.7924646 | -0.0477671 | -0.1652737 | 0.4662836 | 0.1487328 | -0.6562362 | 0.2592649 |
| -0.5155893 | -0.2539301 | -1.0987269 | -0.5754246 | -0.2787336 | -1.2481935 | -0.2079365 | 0.2579351 | -0.5707318 |
| -0.0756442 | -0.2826781 | -0.4184553 | -0.1037571 | -0.3488825 | -0.2424466 | -0.0033670 | -0.5214336 | -1.1910417 |
| -1.0636602 | -0.7842854 | 0.7365588 | -0.9764786 | -0.8354234 | 0.5524137 | -0.5926473 | -0.3646632 | 0.4989609 |
| -0.2274815 | -0.4808336 | -1.4022086 | -0.4510456 | -0.8094998 | -1.7430280 | -0.2065617 | -0.6132021 | -1.1897096 |
| -0.5007824 | -0.7685348 | -0.6636615 | -0.5516769 | -0.6232206 | -0.3353295 | -0.0979552 | -0.6315800 | -1.1428029 |
| -0.5754428 | -0.4521627 | -0.6867283 | -0.4431788 | -0.6065433 | -1.0362264 | -0.6580089 | -1.0731577 | -1.1339996 |
| -0.6980399 | -1.4068064 | -1.108308 | -1.2429381 | -1.1958553 | -1.5234068 | -0.7296793 | -1.3600919 | -2.6190112 |
| -0.5102931 | -0.8484206 | -1.4041399 | -0.4616591 | -0.7090989 | -1.4156780 | -0.7283868 | -0.6990362 | -3.1013126 |
| -0.7549381 | -0.2943306 | 0.3968951 | -0.3870061 | -0.3446606 | -0.1178011 | -0.4113567 | -0.2231784 | -0.5949758 |
| -0.0016492 | -0.1029744 | -0.0359561 | 0.0663159 | -0.3762798 | -0.5263993 | 0.1648469 | -0.5197008 | -1.5603844 |
| 0.0630650 | 0.1436554 | 0.3708728 | 0.1732172 | 0.1396739 | 0.3257540 | -0.0566752 | -1.3053320 | 0.0806322 |
| -0.4467082 | -1.1083608 | -1.2204885 | -0.5669164 | -0.8912241 | -1.4743403 | -0.5422519 | -1.2078317 | -1.3572481 |
| -0.5675533 | -0.7149438 | -0.4672497 | -0.7445866 | -0.6205938 | -0.7786137 | -0.6286889 | -0.7313527 | -1.0840417 |
| -0.2554750 | -0.7448673 | -0.2701962 | -0.4324115 | -0.5314970 | -0.5452323 | -0.1508048 | -1.8904406 | -1.2687325 |
| -0.6173385 | -0.5861877 | -0.0976486 | -0.6700004 | -0.1322417 | 0.2669292 | -0.6746031 | -0.0849196 | -0.2931337 |
| -0.6472605 | -1.0831521 | -0.3387408 | -0.7461731 | -1.4350938 | -0.3009327 | -0.4847989 | -1.7194175 | -1.4088138 |
| -0.1629435 | -0.4682717 | -0.5699311 | -0.4096509 | -1.0064566 | -0.9180919 | -0.1468722 | -1.1455494 | -1.5266826 |
| -0.9353350 | -1.4491788 | -0.6749201 | -1.6546344 | -1.5559553 | -1.1371216 | -0.9343199 | -1.1337529 | -2.0482159 |
| -0.5277864 | -0.0774822 | 0.3989320 | -0.3661318 | -0.2407858 | 0.7586466 | -0.7417943 | -1.2278567 | -0.0393857 |
| -0.9422796 | -1.5232068 | -1.2741669 | -0.9354663 | -1.2741955 | -0.8563380 | -1.0049742 | -1.2140615 | -0.9830701 |

| | | | | | | | | |
|---|---|---|---|---|---|---|---|---|
| 0.0151021 | -0.1143593 | -0.0866112 | -0.0927968 | -0.2957268 | 0.3303794 | -0.2691625 | -1.0771651 | 1.0726913 |
| -0.5232772 | -0.4583559 | 0.1003912 | -0.3803762 | -0.2178104 | 0.3964172 | -0.4137002 | -0.3249985 | 0.3375052 |
| -0.3515880 | 0.0701763 | 0.3601743 | -0.1210944 | -0.0025502 | 0.5009430 | -0.3762399 | -0.7697035 | 0.4102626 |

| PG_552 | PG_553 | PG_554 | PG_555 | PG_556 | PG_557 | PG_558 | PG_559 | PG_560 |
|---|---|---|---|---|---|---|---|---|
| -1.015429 | -1.021810 | -0.320895 | -1.480247 | -1.118484 | -1.232534 | -1.223626 | -1.081851 | -0.333304 |
| -0.527233 | -0.487488 | -1.881706 | -1.320903 | -1.490029 | -1.804994 | -1.192464 | -2.124244 | -0.386067 |
| -0.940257 | -1.135320 | -1.774479 | 0.9891801 | 1.1330919 | 0.4003703 | 0.5307199 | 0.6081524 | -0.933100 |
| -0.297591 | -0.523720 | -1.452009 | -2.446087 | -1.966709 | -2.112408 | -2.023536 | -2.342184 | -0.161037 |
| -0.350412 | -0.040921 | -1.352075 | -1.383153 | -1.653449 | -3.073087 | -1.611000 | -2.440045 | -0.103999 |
| -0.228434 | -1.002538 | -2.408173 | -1.090070 | -1.208026 | -2.653254 | -1.673286 | -2.066775 | -0.403771 |
| -0.401890 | -1.178728 | -1.308503 | -0.717918 | -0.833835 | -0.852722 | -0.964726 | -1.185596 | -0.378498 |
| 0.1242646 | -0.248839 | -1.716888 | -1.447243 | -1.241094 | -2.666609 | -1.496267 | -2.792874 | -0.019773 |
| -0.509374 | 0.0848867 | -0.380910 | -0.004823 | 0.0657264 | 0.0416471 | -0.047537 | 0.3148656 | -0.356603 |
| -0.499434 | -1.392582 | -0.794207 | -0.247326 | -0.470491 | 0.0472864 | -0.669582 | -0.038330 | -0.548766 |
| -0.509247 | -0.692229 | -1.089033 | -1.483563 | -1.450872 | -2.269501 | -1.643097 | -2.244888 | -0.155702 |
| -0.133936 | -0.499234 | -1.109316 | -1.525075 | -1.234670 | -1.998955 | -1.566855 | -2.052264 | -0.285596 |
| -0.426948 | -1.184860 | -3.597780 | -1.331744 | -1.315261 | -1.889189 | -1.856427 | -2.937719 | -0.224678 |
| -0.971529 | 3.2526450 | 4.1015726 | -0.701009 | 1.7075936 | 3.8301792 | 3.1793280 | 4.2152026 | -0.512461 |
| -0.038871 | 0.0465422 | -0.191735 | -2.560261 | -2.390407 | -2.416567 | -2.433636 | -2.916171 | 0.0452515 |
| 0.2441330 | 2.7342538 | 2.6579096 | -0.529369 | 1.0118051 | 2.3333225 | 1.7090552 | 2.4844763 | -0.219816 |
| -1.041771 | -1.803510 | -0.690122 | -1.385145 | -1.410533 | -0.993070 | -1.087892 | -1.203360 | -0.813857 |
| -0.868998 | -2.452826 | -0.952589 | -0.913553 | -1.171382 | -0.460979 | -1.121647 | -0.327302 | -0.522888 |
| -1.113874 | -0.966469 | -0.851126 | 0.3122345 | -0.612018 | -0.536756 | -0.569673 | -0.204628 | -0.482267 |
| -0.603630 | -1.338448 | -1.050673 | -1.255978 | -0.886738 | -1.070191 | -1.311035 | -1.371023 | -0.857828 |
| -0.544832 | 0.0547061 | 0.0605303 | -2.002525 | -0.878689 | -1.615629 | -1.234941 | -1.941891 | 0.0240343 |
| -0.721756 | -0.656459 | -0.036726 | -0.479542 | -0.554360 | -0.491281 | -0.580786 | -0.547352 | -0.483548 |
| 0.1032934 | 1.1561271 | 0.9496943 | -0.011625 | 0.3017244 | 1.0078036 | 0.5614514 | 0.4971163 | 0.0867082 |
| -0.250061 | -0.941012 | -2.182404 | -0.791370 | -0.663142 | -1.841173 | -1.232022 | -2.217993 | -0.261832 |
| -0.146085 | -1.372943 | -3.753555 | -0.347581 | -0.702697 | -1.885139 | -1.188531 | -1.509097 | -0.490245 |
| -0.109475 | 0.4023161 | 0.8105671 | -0.330088 | 0.5736312 | 0.4958482 | 0.5260357 | 0.0789009 | 0.2363331 |
| -0.174331 | 0.0653538 | -0.555479 | -2.324345 | -2.363916 | -2.204093 | -2.367430 | -2.643597 | -0.003720 |
| -1.149180 | -1.283144 | -3.075327 | -1.438060 | -1.335721 | -2.233193 | -1.669824 | -2.544743 | -0.702139 |
| -0.788206 | -1.977736 | -3.726969 | -0.553888 | -1.564634 | -1.991493 | -1.927683 | -2.417987 | -1.011792 |
| 0.0234094 | -0.134706 | -1.275461 | -1.098975 | -0.852390 | -1.013880 | -0.945595 | -1.286603 | 0.1087683 |
| 0.9115624 | -0.006445 | -2.792986 | -0.681178 | -0.824072 | -1.065751 | -0.819835 | -0.814124 | -0.342840 |
| 0.5457947 | -0.305704 | -0.417290 | 0.3187222 | 0.1435808 | -0.478663 | 0.0790725 | -0.814216 | -0.014837 |
| -0.848665 | 0.4508399 | 0.6889608 | -1.375779 | -0.972431 | -0.589793 | -0.610151 | -0.422519 | -0.214421 |
| -0.505305 | -1.480717 | -2.984853 | -1.620033 | -1.243909 | -2.455159 | -1.429301 | -2.850241 | -0.275149 |
| -0.234555 | -0.595671 | -0.048764 | -0.015484 | -0.050553 | 0.0447489 | -0.202068 | 0.0208668 | -0.303870 |
| -0.318332 | -1.200687 | -0.843693 | -1.854845 | -1.413772 | -1.428527 | -1.818481 | -1.284854 | -0.370409 |
| -0.115754 | -0.986717 | -1.807079 | -0.821156 | -1.417722 | -2.199734 | -1.901112 | -2.156732 | -0.051092 |
| -0.064383 | 4.0754479 | 0.4286380 | 0.2001527 | 1.2424527 | 1.2586427 | 3.3268495 | 1.5992232 | -0.181800 |
| -0.653011 | -0.596293 | 1.1042623 | -0.791235 | -0.297007 | -1.812915 | -0.529140 | -1.378354 | 0.2039546 |
| -2.043237 | -1.187426 | -0.463533 | -1.011056 | -2.365621 | 0.4462856 | -1.958167 | 0.3445095 | -1.765201 |
| 0.1189835 | 0.0161872 | 0.9107617 | 0.3516027 | 0.3817663 | 0.2874342 | -0.084691 | 0.7789344 | -0.192247 |
| -0.463200 | -1.502575 | -2.118937 | 0.4245461 | 0.1218482 | -1.079061 | -0.053595 | -1.096543 | -0.731523 |
| -0.332442 | -0.466556 | -1.181029 | -1.210836 | -1.363386 | -1.022260 | -1.012377 | -1.158114 | -0.718062 |
| -0.022425 | -0.601505 | -1.989509 | -1.846326 | -1.600892 | -2.203543 | -1.625113 | -2.148510 | -0.131841 |
| -0.041128 | 0.4502968 | 1.0535047 | 0.0731599 | 1.0408385 | 1.7376535 | 1.2423746 | 1.8699577 | 0.4204066 |
| -0.028428 | 0.5332873 | 3.5050853 | 1.2867265 | 1.5520979 | 3.7315877 | 1.5456867 | 4.3060153 | -0.878788 |
| 0.0340889 | -0.058442 | 0.2511108 | 1.4238126 | 1.1386534 | -0.327602 | 0.4608996 | 0.3403992 | -0.208711 |

| | | | | | | | | |
|---|---|---|---|---|---|---|---|---|
| -0.296849 | 0.2067111 | -0.645164 | -0.547475 | -1.643927 | -0.849303 | -0.932760 | -1.401615 | -0.065973 |
| -0.633994 | -0.880922 | -2.949137 | -1.233246 | -0.990482 | -2.085960 | -1.105069 | -2.360316 | -0.552613 |
| -0.663245 | -0.136590 | 0.3227334 | -1.362973 | -0.984549 | -0.634249 | -0.490798 | -0.611867 | -0.304792 |
| -0.386852 | 3.9954017 | 1.4143911 | -0.572423 | 0.2921026 | 0.0095690 | -0.267322 | 0.4547695 | -0.876568 |
| -1.184220 | -1.258980 | -1.496408 | 0.4412187 | -1.055397 | -1.084436 | -0.613355 | -0.798660 | -1.369475 |
| -0.240338 | -0.112352 | 0.9294010 | 0.3202314 | 0.4058533 | 0.5408120 | 0.3059916 | 0.8758507 | -0.270535 |
| -0.284826 | -1.516629 | -1.383863 | -1.283770 | -0.991186 | -1.642796 | -1.306919 | -2.259479 | -0.516791 |
| -0.323214 | 1.6950482 | 2.4694963 | -0.107187 | 1.2519926 | 2.3807119 | 2.1725567 | 2.6006713 | -0.117992 |
| 1.5029908 | 3.0805580 | -0.917930 | -0.933199 | 1.1938851 | -0.186411 | 2.6581532 | -0.368892 | -0.573445 |
| -1.231819 | -0.124379 | -0.499223 | -1.683890 | -1.001289 | -1.053981 | -1.105264 | -1.350393 | -0.418755 |
| -0.665529 | -1.358155 | -0.723944 | -0.992956 | -1.189392 | -1.659501 | -1.462172 | -1.591531 | -0.713477 |
| 1.7538355 | 4.3389482 | 3.2234086 | -0.659858 | 0.5292779 | 2.3362509 | 1.2665452 | 2.6172357 | 0.7499782 |
| 0.4522808 | 2.9388394 | 1.2623957 | -1.892885 | -0.869451 | -0.089437 | -0.176535 | 0.0701392 | 0.2048021 |
| -0.571787 | -0.846917 | -1.566442 | -0.579981 | -0.495967 | -1.069178 | -1.100699 | -1.147158 | -0.547174 |
| -1.232814 | -2.254831 | -2.499710 | -1.779287 | -1.646666 | -2.069853 | -1.504300 | -1.950634 | -0.585155 |
| -0.538319 | -1.547690 | -0.871536 | -1.025650 | -1.019754 | -0.887009 | -0.753077 | -0.529165 | -0.499750 |
| -0.264883 | -1.030183 | -2.106955 | -0.902379 | -1.206813 | -1.865904 | -1.092987 | -1.881288 | -0.340516 |
| -0.325516 | -0.945045 | -0.400000 | -0.503312 | -0.468563 | -1.407738 | -0.796947 | -1.291595 | -0.400081 |
| -0.411941 | -1.008869 | -0.776155 | -0.251306 | -0.358699 | -1.393474 | -0.262453 | -1.063010 | -0.188916 |
| -0.402345 | -0.185846 | -1.233896 | -3.075938 | -2.470826 | -2.581132 | -2.229229 | -2.849173 | -0.033630 |
| -0.207422 | -0.234221 | 0.5823224 | 0.2551766 | -0.066885 | 0.5240633 | 0.1894267 | 0.4602965 | 0.1639863 |
| -0.841074 | -1.238576 | -1.600374 | -1.013098 | -0.940131 | -1.296374 | -1.065660 | -1.866277 | -0.504651 |
| 0.0059378 | -0.163925 | -0.292266 | 0.2156455 | 0.6121963 | 0.0873867 | 0.0695376 | -0.138486 | -0.040914 |
| 0.2717130 | 0.5099225 | -0.941824 | -3.476411 | -3.640355 | -3.036053 | -3.848241 | -3.376955 | 0.4366433 |
| -0.899788 | -1.609402 | -1.954034 | -1.534121 | -1.379314 | -2.012284 | -1.394490 | -1.679797 | -0.484701 |
| -0.196769 | -0.930710 | -1.292608 | -1.465941 | -0.989847 | -1.626723 | -1.884155 | -1.345338 | -0.039354 |
| -1.126315 | -2.668855 | -1.285785 | -0.283489 | -0.767477 | -0.943708 | -1.575391 | -1.280944 | -0.287830 |
| 0.5091528 | 1.9745251 | 2.9054983 | -0.369648 | 0.6523065 | 2.5787344 | 0.9216172 | 2.3471248 | 1.1858593 |
| -1.088461 | 1.6663195 | 3.0093677 | -0.858770 | -0.214014 | 3.0187433 | 1.0528418 | 2.8516156 | 0.0718640 |
| -0.200808 | -0.624069 | -3.062724 | -1.048695 | -1.158458 | -1.759462 | -1.355538 | -1.885441 | -1.361491 |
| -0.474081 | -1.205613 | -1.727529 | -0.209118 | -0.184610 | 0.1689541 | -0.226386 | -0.191121 | -0.530337 |
| -0.318679 | -0.406063 | -0.953992 | -3.724188 | -2.216436 | -2.460834 | -2.837714 | -2.592800 | 0.0923442 |
| -0.828768 | -1.632282 | -1.240931 | -0.683559 | -0.803555 | -1.487657 | -1.435469 | -1.306559 | -0.567826 |
| -0.428758 | -1.584854 | -2.616775 | -1.566305 | -1.428156 | -2.278124 | -1.539657 | -2.276231 | -0.435426 |
| 0.1246907 | 1.2547773 | 1.9186851 | -0.288701 | 0.9516540 | 1.3446027 | 0.9513382 | 1.5253279 | 0.1125544 |
| 0.2692950 | -1.066765 | -1.209391 | 0.5888153 | 0.5565990 | 0.3115109 | -0.100305 | -0.093662 | -0.247187 |
| -0.515427 | -0.439097 | 0.0895713 | -0.850134 | -0.878440 | -1.411189 | -1.069198 | -1.284460 | -0.329548 |
| -0.059844 | -1.236605 | -1.993405 | -0.566309 | -0.371312 | -1.735945 | -0.906659 | -2.288505 | 0.0043966 |
| -0.160507 | -0.631713 | -3.161950 | 0.4540925 | 0.3145436 | -0.858869 | -0.280626 | -1.089476 | -0.391453 |
| -0.780229 | -1.977521 | -2.267402 | 0.0240783 | -0.449781 | -1.583806 | -0.796977 | -2.164485 | -0.643674 |
| -0.668832 | -0.635173 | -2.427891 | -1.121087 | -1.405749 | -1.142527 | -1.062679 | -0.961495 | -0.499788 |
| -0.848874 | -2.439500 | -2.711689 | -1.040256 | -2.063282 | -0.906741 | -2.281590 | -0.790417 | -0.621206 |
| 2.3093141 | 4.5151406 | 3.0898522 | 0.0104072 | 1.1214164 | 2.0513574 | 2.8649586 | 2.0944447 | 1.4187186 |
| -0.171406 | -0.176461 | -0.421444 | -1.263125 | -0.932534 | -1.127562 | -0.986517 | -1.468740 | 0.1679159 |
| -0.364101 | -0.217509 | -0.033052 | -1.818015 | -1.754695 | -1.864268 | -1.718557 | -2.037493 | -0.034784 |
| -1.258574 | -1.717105 | -1.289177 | -0.982895 | -1.102814 | -1.616803 | -1.322995 | -1.299617 | -0.838773 |
| 4.3034440 | 8.8717071 | 8.9500887 | 0.1653632 | 0.5197359 | 5.6733232 | 1.3576127 | 7.3209930 | -0.239180 |
| -0.002997 | 2.2663245 | 1.8467087 | -0.995758 | -0.852201 | 1.1493699 | 0.2365846 | 1.4099486 | -0.198280 |
| -0.835277 | -1.476988 | -0.234992 | -1.892040 | -1.451557 | -2.702293 | -1.307787 | -2.168022 | 0.1994683 |
| 0.0206384 | -0.199941 | -1.252554 | -0.715099 | -0.615358 | -0.821349 | -0.486064 | -1.368229 | -0.061532 |

| | | | | | | | | |
|---|---|---|---|---|---|---|---|---|
| -1.2285088 | -1.3688163 | -3.8092128 | -0.8020371 | -0.7675871 | -0.9392296 | -1.0359229 | -0.8973324 | -1.2704886 |
| -0.4908972 | -0.2554879 | 0.0169059 | -0.5743082 | -0.3079848 | -0.1177217 | -0.3287895 | -0.2453381 | 0.0171314 |
| -0.0509550 | -0.5601279 | 0.1108165 | -0.8330377 | -0.8159531 | -0.4408919 | -1.1984134 | -0.9260611 | -0.5097281 |
| -0.2553197 | 0.0547334 | 0.3388604 | -1.0579388 | -0.5305009 | -1.0682078 | -0.5920048 | -1.0928330 | -0.0192526 |
| -0.7765999 | -2.2279349 | -1.2526214 | -0.6488269 | -1.1898801 | -2.5387821 | -1.5634089 | -3.2370432 | -0.2054643 |
| 0.6388851 | 0.5787062 | -1.8726765 | -0.9324875 | -0.6165692 | -1.0391032 | -0.4623306 | -1.0043779 | -0.8971251 |
| -0.3850869 | 0.4901464 | -0.5381756 | -2.4700186 | -2.1896731 | -1.6731014 | -1.2556733 | -1.6215056 | 0.0284487 |
| -0.0280597 | -0.0987205 | 0.3322890 | 0.2671246 | 0.0664458 | -0.1473983 | -0.0570756 | -0.1345726 | 0.0092789 |
| 0.0587895 | 0.1943587 | -0.4111648 | 0.1248694 | 0.1614590 | 0.1897108 | -0.1459714 | -0.2278913 | -0.2155764 |
| 0.1185502 | 1.0604864 | 1.2720917 | -0.5684591 | 0.0055923 | 0.5004640 | 0.4997221 | 0.3351079 | 0.1705102 |
| 0.0641410 | -0.4362230 | -1.3237880 | -1.0258043 | -0.7662750 | -2.3237719 | -0.9501803 | -2.1575237 | -0.0584355 |
| -0.6923197 | -1.1777990 | -2.2008235 | -2.2608312 | -2.4843886 | -2.5464189 | -2.1316003 | -3.3458741 | -0.1617449 |
| -0.1764926 | -1.1047049 | -4.6396118 | -0.1907800 | -0.5825849 | -2.6381058 | -0.9481773 | -3.0431367 | -0.0050308 |
| -0.2457087 | -0.4671170 | -1.9604643 | -1.2164225 | -0.8663009 | -1.5309639 | -0.9662751 | -1.8666412 | -0.2553572 |
| -0.2087736 | -0.4464046 | -0.7881331 | -1.1924862 | -1.1033649 | -1.9878152 | -1.2601318 | -2.7805829 | -0.3182257 |
| -0.2450612 | -0.0180661 | -0.3191207 | -0.1790938 | -0.0249231 | 0.0934130 | 0.1753006 | -0.1083529 | 0.2041227 |
| -0.1662417 | -1.0578277 | -1.6090554 | -0.9781964 | -1.2287370 | -0.5500952 | -1.0926648 | -1.3242430 | -0.3544299 |
| -0.0930350 | -0.2365528 | 0.1667472 | 0.0977872 | 0.2332972 | 0.4219401 | -0.0471902 | 0.1403716 | -0.1038825 |
| -0.7829532 | -0.8965490 | -1.900025 | -1.7986225 | -1.6446069 | -1.6649591 | -2.2652117 | -1.0609900 | -0.2012645 |
| -0.1932845 | 0.6174560 | 1.5104454 | 0.1593886 | -0.1525350 | 0.5398717 | -0.1760008 | 0.4156701 | 0.0687897 |
| -0.0040193 | -0.7514084 | -0.1497949 | -0.3833159 | -0.5243981 | -1.0033034 | -0.5848964 | -1.2401028 | -0.2440181 |
| -0.2518767 | -0.6937682 | -1.2641356 | -0.6513901 | -0.7689411 | -1.3819894 | -1.1623413 | -1.3833086 | -0.3867256 |
| -0.2482004 | -0.4513874 | -4.6624800 | -0.5296785 | -0.5157738 | -0.6447356 | -0.6284017 | -0.7048070 | -0.1035411 |
| -0.5170949 | -0.7438239 | -1.7544461 | -0.7320174 | -0.4359239 | -1.3078931 | -0.8603522 | -1.8317751 | -0.3267857 |
| -0.7805709 | -0.7972313 | -0.9368055 | 0.1360763 | 0.3040896 | -0.7937947 | -0.3275039 | -1.1331468 | -0.3751184 |
| -0.3070972 | -0.7927868 | -0.8513953 | -0.8422191 | -1.3157991 | -1.4001075 | -1.6546713 | -1.5499021 | 0.0147667 |
| -0.0426297 | -0.2037173 | -0.0307367 | -0.3060712 | -0.8244697 | -0.7069838 | -0.4707376 | -0.5995662 | 0.2003572 |
| -0.2069872 | 0.3128182 | -0.0651498 | 0.8835725 | 0.5584546 | 0.8904522 | 0.5898940 | 0.6461945 | -0.3942002 |
| 0.0840316 | -1.0010021 | -2.4304332 | -0.1680448 | -0.8184224 | -0.3568355 | -0.5635930 | -0.5464575 | -0.2505000 |
| -1.4767548 | -0.1791388 | -0.0341480 | -0.7244514 | -0.9901187 | -1.1614534 | -0.6062002 | -1.4797161 | -0.7702831 |
| -0.8558612 | -1.8470199 | -2.3268943 | -0.6567593 | -0.9525072 | -1.0059683 | -0.5673435 | -0.7011511 | -0.6509365 |
| -0.0967536 | -0.4239229 | -0.3089016 | -0.9987125 | -1.0620928 | -2.4414507 | -1.7665302 | -1.7827917 | -0.3130799 |
| -0.9891528 | -0.8713120 | -0.5303686 | 0.9694763 | -0.0065157 | -2.1010066 | 0.4918098 | -1.7417577 | -0.5756161 |
| 0.2077490 | 0.1807069 | -0.4076604 | -0.6457296 | -0.4242621 | -0.5353816 | -0.2055823 | -0.7085368 | 0.3514058 |
| -0.1596098 | -0.5114798 | -2.7144156 | -0.3227225 | -0.5030964 | -1.3757337 | -0.4754142 | -2.2330273 | -0.4252392 |
| -0.6705184 | -1.3056756 | -1.8013693 | -1.7258289 | -1.0660347 | -1.5370325 | -1.5965487 | -2.2272793 | -0.3245514 |
| -0.2991158 | -0.7759510 | -0.3324166 | -1.5755415 | -1.1212815 | -0.9944295 | -1.1047363 | -0.9162338 | -0.1804361 |
| -0.4331526 | -0.4561449 | 0.0133837 | -1.7564465 | -1.3397647 | -2.7574130 | -1.4024665 | -3.4941786 | -0.0894531 |
| -0.4975686 | -0.6627657 | -0.8238368 | -0.5666789 | -1.2393112 | -1.0207835 | -0.5158081 | -1.0707931 | -0.4617494 |
| -0.5174861 | -1.3325218 | -0.9578840 | -0.6365505 | -0.4025095 | -0.6446722 | -0.5944696 | -0.5927373 | -0.7577575 |
| -1.1536581 | -0.3204084 | 1.0383030 | -2.1462813 | -1.5659266 | -1.3293342 | -1.8726377 | -1.2119585 | -0.4051494 |
| -0.3311020 | -0.2702123 | -1.0762792 | -1.0044675 | -0.6209135 | -1.0685181 | -0.8984789 | -2.2810434 | -0.0901786 |
| -0.3461750 | -0.5462410 | -1.0459224 | -1.8878116 | -1.2038986 | -0.9482286 | -1.9470487 | -1.7119486 | -0.0277677 |
| -0.3165715 | -0.4231695 | -1.8972285 | -0.7424374 | -0.9354567 | 0.0618739 | -0.8057030 | 0.1655777 | -0.2479939 |
| 0.1296181 | -0.6953201 | -1.6627289 | -0.4833635 | -0.3554765 | -0.4316939 | -0.3437971 | -0.3428657 | -0.1201030 |
| -0.0842705 | 0.5451497 | 0.5093302 | -1.2621538 | -0.8089056 | -0.6287326 | -0.4209577 | -0.7542890 | -0.2958313 |
| -0.5113242 | -0.3713722 | -0.8872723 | -1.0200842 | -1.0001052 | -1.9199523 | -1.3407881 | -1.8064162 | -0.8681377 |
| -0.1221646 | 0.6098776 | 0.1458868 | -0.8547785 | -0.2870952 | -0.0587400 | -0.3488114 | 0.4550009 | -0.0199354 |
| 0.1121471 | -1.2042022 | -4.1738356 | -0.7862127 | -0.8240465 | -1.5076285 | -1.2706111 | -1.6032151 | -0.9032083 |
| -0.9665312 | -1.1781904 | -2.1503584 | -0.7614276 | -1.3196732 | -1.5477643 | -0.4768159 | -1.6655646 | -0.7061610 |

| | | | | | | | | |
|---|---|---|---|---|---|---|---|---|
| 0.1314595 | 0.2892393 | 0.0426459 | -1.685104 | -1.117595 | -0.579408 | -1.196775 | -0.945223 | 0.0650820 |
| -1.412001 | -2.015640 | -2.624805 | -1.507193 | -1.876141 | -2.256340 | -2.189282 | -2.401475 | -0.593702 |
| -0.703694 | -0.352794 | 0.3106508 | -0.749360 | -0.524005 | -0.191078 | -0.508215 | -0.392336 | -0.412418 |
| 0.9109263 | 1.5150934 | 0.6687539 | 0.2477674 | 1.6341366 | 2.0702181 | 2.3199002 | 2.4685420 | -0.468141 |
| -0.917016 | -1.313825 | -1.909872 | -1.526131 | -1.804351 | -2.250019 | -1.638272 | -1.865280 | 0.0511927 |
| 0.4125668 | 1.7787953 | 2.1644849 | -0.047292 | 1.7383066 | 1.7256451 | 1.9142169 | 1.4940192 | 0.5967768 |
| -0.447516 | -0.548381 | -0.661469 | -0.717860 | -0.719642 | -1.553928 | -0.946201 | -1.305225 | -0.275565 |
| -0.994650 | -1.508198 | -1.629173 | -0.971526 | -0.544411 | -0.708355 | -0.955291 | -0.829138 | -0.456930 |
| -0.782748 | -1.548116 | -1.907600 | -0.618193 | -1.336896 | -2.216378 | -1.465168 | -2.377615 | -0.532211 |
| -0.266010 | -0.685189 | -0.194041 | -0.866844 | -1.045068 | -0.801111 | -0.911906 | -1.154667 | -0.007967 |
| -0.322155 | 0.4585393 | 1.2696255 | -0.707486 | -0.545524 | -0.182962 | -0.468115 | -0.185372 | -0.070043 |
| -0.804263 | -1.571451 | -2.413224 | -1.451174 | -1.262892 | -1.435520 | -1.696032 | -1.494096 | -0.377136 |
| -0.127994 | -0.837577 | -2.893243 | -0.697418 | -1.420499 | 0.3068535 | -1.876231 | 0.3228420 | -1.324472 |
| -0.282176 | -0.457257 | -0.239124 | -1.660655 | -0.583913 | -0.205409 | -0.880017 | 0.0892427 | -0.308184 |
| -0.244028 | -0.668357 | -0.071820 | -0.356781 | -0.280525 | -0.043234 | -0.218082 | -0.093878 | -0.214644 |
| -0.534962 | -0.909167 | -0.297794 | -0.556419 | -0.827392 | -1.379641 | -0.658272 | -1.494294 | 0.0527777 |
| -0.231492 | -0.376353 | 0.6294791 | 1.0009555 | 1.5328315 | 0.9413786 | 1.0658381 | 0.6053529 | -0.418306 |
| -1.093281 | -1.964507 | -3.248506 | -1.358215 | -1.794033 | -2.674102 | -2.397359 | -2.459857 | -0.689268 |
| -0.774518 | -0.598543 | -1.486604 | -1.363757 | -1.233459 | -1.257989 | -1.292777 | -1.500230 | -0.815090 |
| -0.248941 | -1.054448 | -2.842244 | -2.303321 | -2.407417 | -3.346574 | -2.241361 | -3.315596 | -0.496326 |
| -0.152071 | 0.7855183 | 2.4325707 | 0.2982516 | 0.8791499 | 2.4460133 | 1.0091450 | 2.7079403 | -0.319573 |
| -0.287106 | -0.193295 | -1.196020 | -1.653956 | -1.343472 | -1.746583 | -1.901764 | -1.971316 | 0.0908271 |
| -0.275536 | -1.197590 | -2.137976 | -0.580414 | -0.742045 | -0.859892 | -0.868186 | -0.693024 | -0.688531 |
| -0.425017 | -0.582160 | -2.251397 | -0.760134 | -0.429096 | -1.107825 | -0.800618 | -1.100735 | -0.418065 |
| -0.123042 | -0.809404 | -2.068111 | -0.110097 | -0.211702 | -1.524577 | -0.934392 | -1.389416 | -0.318401 |
| -0.129805 | -0.433134 | -1.427383 | -0.741653 | -1.862038 | -0.419236 | -0.462932 | -0.672604 | -0.306350 |
| -0.582913 | -0.864853 | -1.794755 | -0.578836 | -0.529270 | -0.735068 | -0.769527 | -1.555607 | -0.801460 |
| -0.509092 | -0.734971 | -1.052956 | -0.922138 | -0.813237 | -0.688404 | -0.628094 | -0.479132 | -0.253964 |
| -0.896733 | -1.402094 | -2.315100 | -1.026743 | -1.398516 | -2.199813 | -1.469439 | -1.659230 | -0.254743 |
| -0.235361 | 0.6799471 | 0.4994423 | -1.082810 | -0.049483 | 0.6597736 | 0.2149703 | 0.2583434 | 0.0223375 |
| -0.201462 | 1.7644700 | 3.3673293 | 0.5073534 | 1.2541596 | 3.0085173 | 1.9917000 | 3.2520458 | -0.057887 |
| -0.631927 | -1.257730 | -1.478223 | -0.863460 | -1.431706 | -1.118276 | -1.437348 | -1.353153 | -0.595214 |
| -0.279297 | -0.413515 | 0.2090236 | -0.538483 | -0.129238 | 0.2047561 | -0.108550 | -0.164275 | 0.0042375 |
| -0.118524 | 0.0878037 | 0.2436824 | -1.336758 | -0.714098 | -0.317724 | -0.636147 | -0.458679 | 0.2062807 |
| 0.2910408 | 0.8382948 | 1.0205097 | 0.6112685 | 1.0068755 | 1.2719187 | 1.1425577 | 1.1846807 | 0.6070874 |
| -0.475480 | -0.410742 | -0.041250 | -0.493062 | -2.075922 | -1.215810 | -1.906918 | -1.053281 | -0.434824 |
| 0.1267157 | 1.3061787 | 0.1364792 | -1.236904 | -1.467305 | -1.517216 | -1.261484 | -1.358368 | 0.6920933 |
| -0.619816 | 0.4441304 | 1.0214554 | -1.814714 | -1.388595 | -1.656780 | -1.152361 | -2.091554 | -0.277536 |
| -0.283972 | -0.275541 | -0.900525 | -1.667998 | -1.517533 | -1.598379 | -1.183941 | -1.923706 | -0.117268 |
| -0.444225 | -0.721599 | -2.372416 | -2.093844 | -2.455395 | -2.493554 | -2.214337 | -2.207562 | -0.336769 |
| -0.487955 | -1.319939 | -1.139388 | 0.7002896 | 0.3050303 | 0.6212902 | 0.2532630 | 1.0645557 | -0.785939 |
| -0.276572 | 0.2237517 | 0.3267787 | -1.367420 | -0.908852 | -0.523007 | -0.799790 | -0.436576 | -0.324812 |
| -0.341251 | 0.2385790 | 0.8650960 | -1.640137 | -1.089165 | -0.127326 | -1.410031 | -0.796609 | 0.0123314 |
| -0.469586 | -0.170729 | -0.019221 | -1.912252 | -1.135887 | -0.161619 | -0.987232 | -1.189496 | -0.009867 |
| -0.203317 | -0.992722 | -0.124013 | -0.645539 | -0.327884 | -0.805829 | -0.488865 | -0.760109 | -0.112655 |
| -0.543307 | -0.092645 | -0.518052 | -1.142444 | -1.144105 | -1.151318 | -0.907570 | -0.918916 | 0.0952773 |
| -0.282342 | -0.857443 | -1.881807 | -0.343362 | -0.144675 | -1.430390 | -0.934508 | -1.493867 | -0.441947 |
| -0.854262 | -1.237407 | -0.224742 | -0.917784 | -1.232566 | -1.959151 | -1.080944 | -1.129471 | -0.729025 |
| 0.0755241 | -0.613087 | -2.132585 | -0.011591 | -0.049867 | -1.541075 | -0.128118 | -1.699438 | -0.335582 |
| -0.511271 | -0.423139 | -1.280387 | -2.106590 | -1.876614 | -1.836357 | -1.846481 | -1.683248 | -0.002171 |

| | | | | | | | | |
|---|---|---|---|---|---|---|---|---|
| -0.178150 | 0.2935203 | -0.302326 | -2.710073 | -2.956065 | -2.676744 | -2.789007 | -2.848190 | -0.129114 |
| -0.627214 | -0.674197 | -2.081205 | -0.539753 | -0.484843 | -0.329129 | 0.0773848 | -0.234479 | -0.422099 |
| -0.746983 | -0.402896 | -1.515270 | -1.752196 | -0.586461 | -1.240117 | -1.185053 | -1.968744 | -0.874635 |
| -0.069763 | -1.094696 | -1.194342 | 0.3192100 | -0.989333 | -1.892984 | -0.830963 | -1.677832 | -0.717002 |
| -0.432863 | -1.039752 | -2.516884 | -0.813681 | -0.901612 | -1.731545 | -1.347951 | -2.085316 | -0.284489 |
| -0.244809 | -0.312229 | -0.697510 | -0.263006 | 0.1661690 | -0.258508 | -0.365875 | -0.270608 | -0.060354 |
| 0.1766469 | 1.5312798 | 0.8656458 | -0.010406 | 0.6696981 | 1.0076537 | 0.9593688 | 0.1782207 | 0.2698521 |
| -0.238635 | -0.673046 | -0.024374 | 0.5000787 | 0.2463519 | 0.3965681 | -0.089626 | 0.3123729 | 0.0250393 |
| -0.183560 | -0.341392 | -2.482147 | -2.583787 | -2.432729 | -3.787642 | -3.047907 | -3.157164 | 0.1788419 |
| -0.062571 | -0.559143 | -0.294054 | -1.335482 | -1.551375 | -1.316663 | -1.228785 | -1.984257 | -0.011653 |
| -0.274267 | -0.349476 | 0.4740350 | -0.775794 | -0.769865 | -0.894534 | -0.493508 | -0.978665 | -0.520030 |
| -0.776231 | -0.871505 | -1.378655 | -1.734227 | -1.488851 | -0.939677 | -1.669706 | -1.859286 | -0.930685 |
| -0.310556 | -0.132229 | -3.400947 | 0.3723916 | -0.281105 | -0.681299 | 0.0421561 | 0.3793011 | -0.245587 |
| 0.0372366 | -0.024097 | -0.469692 | -1.822473 | -0.793262 | -2.754708 | -1.371319 | -2.722305 | -0.079897 |
| -0.122946 | -1.198178 | -2.847314 | -0.710742 | -0.595256 | -2.845433 | -0.946934 | -3.390810 | -0.157030 |
| -0.178842 | -0.187401 | -0.888844 | -0.578943 | -0.459847 | -0.857435 | -0.608034 | -1.061227 | -0.150884 |
| -0.200657 | -0.366588 | -2.328607 | -3.779847 | -3.056794 | -5.173282 | -3.688176 | -5.262369 | 0.0961069 |
| -0.212051 | -1.254030 | -0.912575 | -0.978278 | -0.565426 | -1.594863 | -0.868617 | -1.857622 | -0.205088 |
| 0.1280111 | -1.621860 | -2.383699 | -0.125812 | -0.052562 | -2.357137 | -0.884196 | -2.107750 | -0.220753 |
| -0.009892 | 0.7060398 | -0.835017 | -1.950519 | -1.553844 | -1.221604 | -1.864341 | -2.485936 | 0.2564887 |
| -1.038106 | -0.759398 | -1.267316 | -1.699130 | -1.613756 | -2.102646 | -1.536991 | -2.537744 | -0.916296 |
| 0.2057154 | -0.983824 | -3.191761 | -0.166489 | -0.167663 | -0.153677 | -0.039233 | -0.184008 | -0.245012 |
| -0.328013 | -0.752044 | -2.454392 | 0.1368987 | -0.468783 | -2.205394 | -1.164267 | -1.907424 | -0.826936 |
| -0.121743 | -0.416162 | 0.2796702 | -1.401564 | -0.844885 | -0.577764 | -1.061581 | -0.399286 | -0.117812 |
| -0.086497 | -0.692952 | -1.658275 | -0.410229 | -0.528031 | -1.075644 | -0.828839 | -1.279572 | -0.371385 |
| -0.679592 | -0.829147 | -1.349694 | -0.660682 | -0.784803 | -0.108085 | -1.183588 | -0.740013 | -0.625483 |
| -0.160604 | -0.470355 | -0.951451 | -0.819921 | -0.921934 | -1.816515 | -1.080859 | -2.471743 | -0.219624 |
| -0.385162 | -0.511359 | -0.964842 | -0.604349 | -0.775630 | -0.827998 | -0.688513 | -0.821455 | -0.574237 |
| -0.183643 | -0.125223 | 0.1082857 | -0.963163 | -0.693112 | 0.2672900 | -0.530507 | 0.2103456 | 0.0188775 |
| 0.0681793 | 0.4243032 | 0.4702673 | -1.363098 | -1.390157 | -0.254873 | -1.273428 | -0.124234 | -0.265681 |
| -0.165783 | -0.253909 | -0.924787 | -0.389875 | -0.123995 | -0.340749 | 0.1448476 | -0.865841 | -0.678882 |
| -0.061788 | -0.534562 | -1.288887 | -1.310845 | -0.660575 | -2.388419 | -1.451300 | -2.471137 | -0.053608 |
| -0.660954 | -0.751578 | -0.161682 | 0.5038960 | 0.9942931 | 2.3406627 | 1.1763204 | 2.1847845 | -0.373075 |
| -0.264613 | -1.167383 | -1.504293 | -1.166590 | -1.080959 | -1.765243 | -1.185750 | -1.786093 | -0.099997 |
| -0.193794 | -0.376972 | -0.977484 | -0.273469 | -0.113061 | -0.044861 | -0.461519 | -0.212305 | -0.359296 |
| -0.483024 | -0.808404 | -1.205530 | -0.829109 | -0.926938 | -2.238317 | -1.215208 | -2.959692 | -0.257921 |
| -0.920606 | -1.993537 | -2.656243 | -0.102703 | -0.597846 | -1.177176 | -1.063037 | -0.969597 | -0.858294 |
| -0.678876 | -1.675342 | -2.923451 | -0.288385 | -0.143514 | -0.441797 | -0.245964 | -0.342797 | -0.002885 |
| -0.187256 | 0.2095824 | -0.526110 | 0.9123709 | 0.6576263 | 1.0672503 | 0.9870653 | 1.2001249 | -0.330228 |
| -0.156856 | -0.746296 | -1.583521 | -0.485045 | -0.063542 | -1.226869 | -0.706693 | -0.849099 | -0.145142 |
| -0.025670 | 0.6974988 | 0.1350671 | -3.733469 | -2.906226 | -2.764317 | -2.427855 | -2.505309 | 0.2441961 |
| -0.761298 | -1.085613 | -1.635651 | -1.977588 | -2.087381 | -2.538579 | -2.006491 | -2.618894 | -0.216029 |
| -0.745884 | -0.754690 | -0.992279 | -0.000360 | -0.442699 | -0.901387 | -0.904985 | -1.342937 | -0.493786 |
| -0.129894 | -0.456759 | -0.319323 | -0.173070 | -1.649780 | -0.923515 | -0.563549 | -0.823460 | -0.249856 |
| -0.766925 | -0.085282 | -0.050846 | -1.159096 | -1.452345 | -1.403810 | -1.435644 | -1.286273 | -0.383253 |
| -0.398655 | -0.785127 | -1.202466 | -3.026729 | -2.573840 | -2.624795 | -2.488400 | -3.279326 | -0.253923 |
| -0.212023 | -0.589480 | -1.722294 | -0.983264 | -1.092785 | -1.178639 | -1.319111 | -1.141889 | 0.0207036 |
| -1.496668 | -1.339684 | -2.999655 | -1.257882 | -1.802103 | -1.214328 | -0.916370 | -1.366697 | -1.237927 |
| -0.351823 | -0.336224 | 0.3280532 | -2.063297 | -1.180829 | -0.298030 | -0.911908 | -1.031484 | -0.194237 |
| -1.048045 | -1.176162 | -0.879453 | -1.120272 | -1.218317 | -1.669407 | -1.256983 | -1.833992 | -0.683274 |

| | | | | | | | | |
|---|---|---|---|---|---|---|---|---|
| -0.400920 | -0.153756 | 1.6368813 | -1.156677 | -0.716368 | 0.7359402 | -0.280105 | 0.5592386 | -0.017924 |
| -0.522830 | -0.187408 | 0.2983718 | -0.187780 | -0.006251 | 0.2501440 | 0.2786757 | 0.1199409 | -0.218124 |
| -0.474514 | -0.599039 | 0.0690864 | -1.180965 | -0.475529 | -0.952258 | -0.425815 | -1.079865 | -0.186858 |

| PG_561 | PG_562 | PG_563 | PG_564 | PG_565 | PG_566 | PG_567 | PG_568 | PG_569 |
|---|---|---|---|---|---|---|---|---|
| -0.729735 | -1.483475 | -0.224703 | -0.771810 | -1.782870 | -0.076803 | -0.393195 | -1.437311 | -0.178040 |
| -0.716657 | -1.169808 | -0.483476 | -0.736515 | -0.969999 | -0.449074 | -1.025484 | -0.874603 | -0.465043 |
| -0.682854 | -1.028086 | -0.778507 | -1.109379 | -1.219932 | -0.870309 | -0.973151 | -1.344952 | -0.920526 |
| -0.582665 | -0.352286 | -0.123252 | -0.556579 | -0.462450 | -0.119687 | -0.564317 | 0.1252529 | 0.0367313 |
| -0.262510 | -1.166048 | -0.039510 | -0.149727 | -0.899744 | -0.484833 | -0.641627 | -1.591764 | -0.634603 |
| -0.866097 | -2.052464 | -0.433467 | -0.832521 | -1.995093 | -0.237859 | -0.897731 | -2.525119 | -0.619910 |
| -1.456482 | -1.075389 | -0.400893 | -1.339408 | -0.951332 | -0.144782 | -1.606169 | -1.358471 | -0.309624 |
| -0.333017 | -1.028254 | -0.099335 | -0.396991 | -1.509063 | 0.2606448 | -0.063614 | -1.000067 | 0.1268177 |
| 0.1728659 | 0.4776848 | -0.364856 | 0.1587051 | 0.5197295 | -0.479774 | -0.272752 | 0.2022512 | -0.542121 |
| -0.983071 | -0.334971 | -0.582215 | -1.061206 | -0.387906 | -0.040954 | -0.474520 | 0.7269675 | -0.039359 |
| -0.248099 | -0.785455 | -0.087103 | -0.373408 | -0.856964 | -0.208446 | -0.399088 | -0.973441 | -0.204721 |
| -0.605725 | -0.979385 | -0.344417 | -0.810144 | -1.048456 | -0.068518 | -0.339797 | -0.603895 | -0.014998 |
| -0.922924 | -3.289963 | -0.216827 | -1.388032 | -2.923218 | -0.597497 | -1.394507 | -2.454550 | -0.649723 |
| 2.1348248 | 3.4846407 | -0.072091 | 3.0743614 | 3.7413318 | -0.291377 | 3.0977000 | 4.2676549 | 0.2009108 |
| -0.120692 | 0.1090386 | 0.1016208 | 0.1354929 | 0.3869960 | 0.0142200 | 0.1216819 | 0.0723448 | 0.0641944 |
| 2.0081195 | 2.5169028 | 0.6849005 | 2.2448210 | 2.4232109 | -0.454681 | 2.5849558 | 3.0089156 | -0.065145 |
| -1.640504 | -1.942629 | -0.698894 | -1.542786 | -1.502916 | -1.006496 | -1.873314 | -2.300935 | -1.334232 |
| -0.536585 | -0.239281 | -0.745146 | -0.475842 | -0.364156 | -0.426984 | -0.474727 | 0.8760721 | -0.370602 |
| -1.826872 | -2.382746 | -0.947673 | -1.376143 | -2.458409 | -1.209560 | -1.929059 | -2.003791 | -0.847990 |
| -0.445097 | -0.192685 | -0.653332 | -0.339540 | -0.235051 | -0.636573 | -0.444654 | -0.525742 | -0.672964 |
| 0.5286257 | 0.0974750 | 0.0462926 | 0.3561072 | -0.249829 | -0.560510 | -0.318859 | -0.668416 | -0.345381 |
| -0.385632 | -0.336350 | -0.485233 | -0.288223 | -0.250783 | -0.473836 | -0.396493 | -0.513943 | -0.402077 |
| 0.8532422 | 1.1549143 | -0.047534 | 0.5553135 | 0.9326993 | -0.116785 | 0.6551037 | 1.5539630 | -0.113005 |
| -0.684228 | -1.046766 | -0.137459 | -0.791839 | -1.046913 | -0.384620 | -0.950557 | -1.725419 | -0.530568 |
| -1.564399 | -2.148664 | -0.774030 | -1.824440 | -2.586129 | 0.0590277 | -0.789121 | -1.413461 | -0.263029 |
| 0.9006312 | 0.8721710 | 0.2752983 | 0.6078928 | 0.4651504 | -0.284166 | 0.6253348 | 1.1097818 | -0.083034 |
| -0.120286 | -0.147435 | 0.1033406 | -0.131287 | -0.043484 | 0.1318899 | -0.211699 | -0.265480 | 0.1819030 |
| -1.691535 | -2.238535 | -0.744125 | -1.304471 | -2.331580 | -1.048767 | -1.587959 | -1.937633 | -1.211855 |
| -1.791700 | -1.864063 | -1.085868 | -1.879865 | -2.109834 | -0.309048 | -1.015783 | -1.597051 | -0.651252 |
| -0.026238 | -0.179508 | 0.1089161 | 0.0507814 | -0.207936 | 0.0685287 | -0.188982 | -0.238104 | -0.114181 |
| -0.204354 | -0.322928 | -0.266074 | -0.501128 | -0.262337 | -0.345709 | -0.344541 | -0.053632 | -0.294302 |
| 0.2483884 | -0.375929 | -0.056096 | 0.3283521 | 0.0404920 | -0.267454 | 0.5762151 | -0.513002 | -0.020953 |
| -0.210148 | -1.136969 | -0.117622 | 0.3501899 | -0.763200 | -0.484712 | -0.072678 | 0.1645642 | -0.405332 |
| -0.949506 | -3.409772 | -0.265281 | -1.263052 | -3.037248 | -0.379074 | -0.981666 | -3.014890 | -0.637102 |
| -0.212988 | -0.081086 | -0.271328 | -0.234315 | -0.101951 | -0.215298 | -0.142419 | -0.406518 | -0.150909 |
| -0.981765 | -0.199531 | -0.890981 | -1.256833 | -0.351805 | 0.2504916 | -0.719115 | 0.2905753 | -0.647933 |
| -1.048544 | -1.892406 | -0.593890 | -1.393055 | -2.083690 | 0.0945368 | -0.426849 | -1.463452 | -0.026939 |
| 2.7954215 | 0.9635812 | 0.0479447 | 3.3020686 | 0.5238201 | 0.1407439 | 3.6205501 | 1.7405948 | 0.8241992 |
| 0.1714521 | -0.094512 | 0.1252175 | -0.036343 | 0.0130057 | 0.1240738 | 0.0419915 | -2.281765 | -0.180467 |
| -1.487162 | 1.2461267 | -1.994703 | -0.947268 | 1.2391231 | -0.621644 | -1.396732 | 0.9806965 | -1.482961 |
| 0.0979166 | -0.137678 | -0.200049 | -0.058288 | 0.0218043 | 0.1549922 | 0.1855882 | 0.2089646 | 0.0063822 |
| -1.025962 | -1.331485 | -0.734868 | -1.198343 | -1.329335 | -0.713029 | -1.117959 | -1.700460 | -0.774953 |
| -0.940387 | -0.900334 | -0.568746 | -0.738795 | -0.738373 | 0.5003378 | 0.5714930 | 0.4329825 | 0.6010448 |
| -0.333953 | -0.528975 | -0.052136 | -0.481540 | -0.995312 | -0.271676 | -0.551007 | -1.077766 | -0.133804 |
| 0.8168860 | 1.3852506 | 0.2867541 | 0.3527879 | 1.1103116 | -0.376257 | 0.4331137 | 1.5096888 | -0.103221 |
| -0.610097 | 4.1141597 | -0.788546 | -0.633899 | 6.0048253 | -1.275318 | -0.962866 | 3.6173056 | -1.257975 |
| -0.363978 | -1.092588 | -0.346885 | -0.772683 | -0.934969 | 0.1139389 | -0.333519 | -0.221820 | -0.020716 |

| | | | | | | | | |
|---|---|---|---|---|---|---|---|---|
| -0.2971889 | 0.2418689 | -0.2589906 | -0.0372912 | 0.2675910 | -0.7406140 | -0.5437907 | -0.0895841 | -0.7975744 |
| -0.5977305 | -2.2592948 | -0.4648557 | -0.7010596 | -2.3758239 | -0.8914357 | -1.0064827 | -2.8394168 | -0.5734799 |
| -0.0019883 | 0.0350309 | -0.2137517 | -0.0541007 | -0.1433053 | -0.6421707 | -0.0881477 | -0.0190457 | -0.6997023 |
| -0.4368583 | -0.3151739 | -0.5245331 | -0.5309328 | -0.5926968 | -0.7275057 | -0.6720582 | 0.8777627 | -1.0165744 |
| -0.6276919 | -1.2285346 | -1.2045099 | -0.6987417 | -1.2897209 | 0.1797506 | -0.8312264 | -2.1300237 | 0.1557396 |
| -0.3485327 | -0.0739969 | -0.1967617 | -0.1401492 | -0.2617593 | 0.1117372 | -0.2907058 | 0.1576084 | -0.1014391 |
| -1.0096583 | -1.7731240 | -0.4831171 | -0.8228726 | -1.3445714 | -0.3827961 | -1.2066305 | -2.2448106 | -0.8003209 |
| 1.8089000 | 2.5280177 | -0.0117299 | 2.1075124 | 2.3460867 | -0.4139371 | 2.7940922 | 3.2573216 | 0.5284740 |
| 0.7031936 | -1.4687370 | -0.2891472 | 0.2960265 | -2.1697373 | -0.2197336 | 1.0118939 | 0.4610955 | -0.1135932 |
| -0.0016859 | 0.3870435 | -0.2460706 | -0.0383901 | 0.0653397 | -0.9113765 | -0.9472550 | 0.1068391 | -1.0467808 |
| -1.8457470 | -1.4952183 | -0.9118068 | -1.5648906 | -1.6321765 | -0.6113388 | -1.3368352 | -2.0611300 | -0.9390131 |
| 3.0999459 | 3.5476364 | 1.9174056 | 3.5945146 | 3.6383286 | 0.0139100 | 2.6512372 | 2.7407461 | 0.9076922 |
| 2.6903116 | 3.1569784 | 1.5354165 | 3.2302963 | 3.4570658 | -0.4629673 | 2.2536013 | 2.9451305 | 0.0095306 |
| -0.4004736 | -1.3907141 | -0.4291107 | -0.6595010 | -1.4174854 | -0.3252653 | -0.3058033 | -0.7170427 | -0.3411232 |
| -0.6872773 | -0.9835264 | -0.9600821 | -0.9263154 | -1.0269314 | -1.0349794 | -1.7723617 | -1.7672188 | -1.2411745 |
| -0.7119757 | -0.4784935 | -0.4746533 | -0.2923060 | -0.1025949 | -0.4133209 | -0.1681037 | 1.0086924 | -0.3178605 |
| -0.7920172 | -1.5007511 | -0.4090796 | -1.0653847 | -1.4328259 | -0.0579901 | -0.5978821 | -1.1343024 | -0.1925797 |
| -0.3992834 | -0.0690459 | -0.4334642 | -0.4171704 | -0.2796150 | -0.2694018 | -0.3262182 | -0.5342882 | -0.1507271 |
| -0.4564991 | -0.5491954 | -0.3966303 | -0.8839455 | -0.4777926 | -0.6905688 | -0.5299358 | -1.4492377 | -0.6603166 |
| -0.4620593 | -0.0693132 | -0.2482746 | -0.2684196 | -0.1013562 | 0.8597883 | 0.5723958 | -0.0653872 | 0.5878949 |
| -0.0050189 | 0.3918319 | -0.1420213 | -0.0619412 | 0.7193701 | -0.1431099 | 0.0243597 | -0.0521405 | -0.1887211 |
| -1.0448418 | -2.5792197 | -0.3620591 | -1.0929577 | -2.4443794 | -0.9102824 | -1.2787564 | -2.2776260 | -0.8641645 |
| 0.0362802 | 0.7784548 | -0.1248019 | 0.0688783 | 0.3438170 | -0.0085910 | -0.1297286 | 0.7767782 | 0.0440329 |
| 0.1657522 | 0.2566217 | 0.3380406 | 0.1674770 | -0.3336283 | 0.5285525 | 0.2875984 | 0.7781964 | 0.4448632 |
| -1.211389 | -1.9739425 | -0.3447575 | -1.3297806 | -2.3468804 | -1.2306325 | -1.3635592 | -1.6795811 | -1.4034910 |
| -0.6365734 | -0.3858348 | -0.1279020 | -0.7745797 | -0.2221257 | -0.0101638 | -0.5374145 | -0.3621587 | -0.0435172 |
| -0.2358166 | -0.1531633 | -0.1500379 | -0.4578607 | 0.0698031 | -0.5576096 | -0.9284452 | -1.4612758 | -0.7971341 |
| 2.0367936 | 3.3459148 | 0.9935284 | 1.4648586 | 3.0385231 | 0.2668288 | 0.9602751 | 2.9465630 | 0.3636302 |
| 0.5110798 | 1.3960893 | -0.0698620 | 0.5717246 | 1.0267687 | 0.5748675 | 1.8355372 | 3.2125738 | 0.9328594 |
| -1.6409821 | -1.5920744 | -1.2660134 | -1.5716835 | -1.8205550 | -0.2579178 | -0.7668734 | -1.4299931 | -0.4125937 |
| -0.6315100 | -0.1573267 | -0.3325626 | -0.4160697 | -0.3062273 | 0.0839401 | 0.4116617 | -0.1774437 | 0.4776594 |
| 0.0242172 | 0.6840707 | 0.1276023 | 0.1757686 | 0.5732321 | -0.0850040 | -0.2173860 | 0.2173717 | -0.0838114 |
| -1.0186191 | -0.8803016 | -0.6563142 | -1.5201734 | -1.1862027 | -0.6084051 | -1.1739959 | -1.0644699 | -0.5050573 |
| -0.7103645 | -1.0453303 | -0.4324065 | -0.7521495 | -1.2486946 | -0.2990227 | -0.6959447 | -0.6166521 | -0.4638138 |
| 0.9564191 | 1.4549895 | 0.2287078 | 0.9964723 | 1.7482828 | -0.5061031 | 0.1691039 | 0.6010861 | -0.3366892 |
| -0.6493411 | -0.6018555 | -0.2432889 | -0.7829747 | -0.3479359 | -0.2524236 | -0.6564178 | -0.8751042 | -0.2526463 |
| -0.3723655 | -0.5581227 | -0.3920561 | 0.0216610 | -0.3907819 | -0.2026996 | -0.5892549 | -1.4228894 | -0.3476227 |
| -0.6989577 | -1.4795096 | -0.3274314 | -0.9292220 | -1.3110199 | -0.3729032 | -1.0171912 | -1.8947696 | -0.3462424 |
| -0.7948051 | -1.5286120 | -0.6266596 | -1.2115005 | -2.1296910 | -0.5756393 | -0.5045467 | -1.5845029 | -0.5198320 |
| -1.1390132 | -1.8980347 | -1.1123959 | -1.1375183 | -1.8538637 | -0.9455720 | -1.5182465 | -1.5966403 | -0.2043382 |
| -1.0442991 | -1.3503296 | -0.6622518 | -0.7949431 | -0.8331380 | -0.4243099 | -1.1215886 | -1.6135287 | -0.5342354 |
| -0.8970279 | -1.0294405 | -0.7533741 | -1.2318159 | -1.2580809 | 0.7443739 | 0.3716912 | 0.5050303 | 0.6671381 |
| 3.4304891 | 1.9892601 | 2.2662856 | 3.1615445 | 1.6740850 | -0.3501692 | 1.6244623 | 1.7247958 | 0.5327588 |
| 0.0545001 | -0.2557775 | 0.1125959 | -0.0507687 | -0.2024701 | -0.2692915 | -0.3265264 | -0.5588483 | -0.2232952 |
| -0.3213110 | -0.8998095 | -0.0756183 | -0.2872250 | -0.6665622 | -0.3854146 | -0.7565353 | -1.7276919 | -0.5556221 |
| -0.6419692 | -0.8485881 | -0.9015811 | -0.7476378 | -0.5703357 | -0.7912105 | -0.6700801 | -0.8041573 | -0.5974755 |
| 1.7785402 | 6.0251652 | 0.1801614 | 4.7898800 | 7.8461414 | 0.0402140 | 0.1184847 | -0.3384703 | 0.0274330 |
| 2.1964390 | 2.6413102 | 0.5608211 | 2.8419154 | 2.7393234 | -0.2290898 | 1.5318939 | 1.7752476 | -0.0579821 |
| -0.8392961 | -1.4335389 | -0.3124801 | -1.4728037 | -1.4676636 | -0.3824207 | -1.0717503 | -0.7932947 | -0.2652022 |
| -0.0612923 | -0.3100913 | 0.0983120 | 0.0863185 | -0.4650972 | -0.5447181 | -0.4905704 | -0.1757599 | -0.5086287 |

| | | | | | | | | |
|---|---|---|---|---|---|---|---|---|
| -1.3624531 | -1.7980730 | -1.2033660 | -1.3456061 | -1.9084934 | -0.6104955 | -1.2926885 | -0.7675086 | -0.5977499 |
| 0.1384054 | 0.8230121 | 0.1644705 | 0.1339231 | 0.8233838 | -0.4434347 | -0.5714267 | 0.0565750 | -0.3307183 |
| -0.5642057 | 0.0189781 | -0.5784378 | -0.7470036 | -0.0552942 | -0.5572970 | -0.2081802 | 0.2192575 | -0.2824745 |
| -0.0618358 | -0.0655334 | -0.0483035 | 0.0456628 | 0.1198382 | -0.1774296 | -0.1045590 | -0.1746613 | -0.1582439 |
| -1.3311237 | -1.4856816 | -0.5011242 | -1.5226388 | -1.7892376 | -0.6110594 | -1.5096543 | -1.6606562 | -0.5761767 |
| -0.6178470 | -0.3657481 | -0.9070383 | -0.3352380 | 0.2623470 | -0.7204232 | -0.4821263 | -0.6251849 | -1.0048149 |
| -0.3684150 | -0.3414425 | 0.0889782 | -0.3934516 | -0.9073465 | -0.1664604 | -0.2632686 | -0.1940448 | -0.4109305 |
| -0.1579330 | -0.0791082 | 0.0863134 | -0.2568178 | -0.2000931 | -0.1074238 | -0.0737061 | 0.1965565 | -0.0023890 |
| -0.1422514 | -0.1997922 | 0.0710352 | -0.1496831 | -0.2097793 | -0.3488872 | -0.0947529 | -0.4652327 | -0.0365434 |
| 0.3022329 | 0.5184034 | 0.2858509 | 0.3689220 | 0.2422680 | 0.0521708 | 1.1990351 | 0.9035005 | 0.2672260 |
| -0.1778264 | -0.4601279 | -0.0338248 | 0.1591551 | -0.1307565 | 0.1058705 | -0.0806842 | -0.7938260 | 0.0567418 |
| -0.3342369 | -1.9322161 | -0.4735698 | -0.6621112 | -2.2115826 | -0.5619851 | -1.1781970 | -2.0159613 | -0.7709783 |
| -0.9750798 | -3.5863434 | 0.0409858 | -1.2891484 | -2.6631258 | -0.2077526 | -1.0527365 | -3.6455344 | -0.4798488 |
| -0.5841187 | -2.0996152 | -0.3089581 | -0.8074867 | -2.1923666 | -0.2790390 | -0.5571419 | -2.1299905 | -0.2574987 |
| -0.4086440 | -0.6620113 | -0.2297692 | -0.5816299 | -0.8583704 | -0.5055562 | -0.7676392 | -0.9842567 | -0.6725688 |
| -0.0754816 | -0.1407720 | -0.0215840 | 0.0251821 | -0.0436389 | -0.1811198 | -0.1312065 | 0.0290506 | -0.1609900 |
| -1.5860290 | -1.0977326 | -0.5460943 | -2.1887321 | -0.7943235 | -0.3573753 | -1.1715440 | -1.2938722 | -0.6444763 |
| -0.1297500 | -0.1590982 | -0.1973842 | -0.3026728 | 0.0953110 | -0.2903152 | -0.0806707 | 0.1385873 | -0.2842642 |
| -0.9018347 | -0.3873943 | -0.3030360 | -0.9086323 | -0.3775600 | -0.2642427 | -0.5411809 | -0.1053534 | -0.3615196 |
| 0.2518137 | 1.9142030 | -0.0647604 | 0.2385060 | 1.8084628 | -0.0723510 | -0.1837063 | 1.5727936 | -0.4061557 |
| -0.4281343 | -0.1972969 | -0.2792348 | -0.4199407 | -0.3918920 | -0.3718261 | -0.2850131 | -0.6334530 | -0.3375431 |
| -0.2246527 | -0.1139322 | -0.2805345 | -0.3371294 | 0.1506844 | -0.7982851 | -0.7317097 | -0.5950747 | -0.5669258 |
| -0.1595102 | -0.1618307 | -0.3374351 | -0.1837354 | -0.0971674 | -0.1844626 | -0.1559667 | 0.0240123 | -0.1163979 |
| -0.6999645 | -1.3659575 | -0.3135917 | -0.6073435 | -1.2946658 | -0.3325735 | -1.0239922 | -1.6804913 | -0.1318927 |
| -1.0753833 | -0.2874037 | -0.3953330 | -0.9186663 | -0.1381050 | 0.1566640 | -0.3999348 | -0.1503434 | -0.0430658 |
| -0.3521683 | -0.2754077 | -0.1946025 | -0.4960127 | -0.2606166 | -0.1884326 | -0.7955060 | -0.3775711 | -0.4601634 |
| -0.1650156 | 0.0296966 | 0.0873508 | 0.0462661 | 0.1896894 | 0.1321169 | -0.1021170 | -0.2933351 | 0.0460885 |
| -0.4580048 | -0.4052816 | -0.2067874 | -0.3291849 | -0.1468824 | -0.1586758 | -0.021635 | -0.2714164 | -0.0245987 |
| -0.8504740 | -0.6099829 | -0.3026365 | -0.9635822 | -0.6689474 | 0.2695862 | -0.7230631 | -0.6009578 | 0.0400390 |
| -0.6546887 | -2.5899145 | -0.3912949 | -0.1414413 | -2.6397168 | -0.6348755 | -0.7555965 | -1.8822452 | -0.8924471 |
| -1.3422441 | -0.5156386 | -0.7776890 | -0.9124235 | 0.0615278 | -0.2057080 | -1.0595520 | -2.1424665 | -0.3402498 |
| -0.6375334 | -0.8874878 | -0.1437288 | -0.3363924 | -0.9699353 | 0.0165455 | -0.7804273 | -1.5800948 | -0.4456149 |
| -0.8257363 | -2.3122329 | -1.2448656 | -0.6901949 | -2.0563412 | -0.4022621 | -1.5996259 | -3.7237805 | -0.7788069 |
| 0.2523464 | 0.4316750 | 0.3092711 | 0.3136071 | 0.4253574 | 0.2619283 | 0.1319015 | -0.0827117 | 0.3222336 |
| -0.4392467 | -1.2102587 | -0.3670332 | -0.4920430 | -1.3192443 | 0.0322964 | -0.0479394 | -1.2048294 | -0.0743176 |
| -0.5510559 | -0.1615565 | -0.4115401 | -0.9723901 | -0.2385069 | -0.3868398 | -1.0073538 | -0.5916115 | -0.6875839 |
| -0.2438296 | -0.2572542 | -0.0613009 | -0.4222759 | -0.4869313 | -0.2798523 | -0.4264366 | -0.4116104 | -0.2620648 |
| -0.2422064 | -1.4966578 | -0.0986128 | -0.1351197 | -1.3542090 | -0.3073151 | -0.4538921 | -1.2979792 | -0.3141850 |
| -0.5886033 | -0.2520428 | -0.2309374 | -0.5755304 | -0.3710691 | -0.2840156 | -0.1406506 | -0.1759091 | -0.3302080 |
| -0.9966341 | -2.1934370 | -0.7426979 | -0.5779447 | -1.7925972 | -0.2370221 | -0.4094956 | -1.9525693 | -0.3948026 |
| -0.5296254 | -0.8875298 | -0.9804240 | -0.9449545 | -1.0120360 | -0.1867070 | 0.0244389 | -0.4016880 | -0.4951322 |
| -0.2358270 | -2.1167904 | -0.1249415 | -0.1565623 | -1.7872526 | -0.3973188 | -0.3026387 | -1.4169353 | -0.3028731 |
| -0.2496301 | -0.9012766 | -0.1600660 | -0.4381910 | -1.0456394 | -0.2486319 | -0.6257906 | -1.0830415 | -0.4565251 |
| -0.1000895 | -0.2214043 | -0.3073920 | -0.1300859 | -0.4803893 | -0.6450316 | -0.5447327 | -0.0735995 | -0.5967327 |
| -0.0095732 | -0.4127371 | 0.1974773 | -0.1608151 | -0.4914237 | -0.7747132 | -0.8189975 | -1.9173888 | -0.6622447 |
| -0.2810288 | -0.2515734 | -0.3716464 | -0.4233170 | -0.2632804 | -0.2516206 | -0.0153055 | -0.0328600 | -0.5056611 |
| -1.2238504 | -1.4115909 | -1.2532548 | -1.3360435 | -1.1589113 | -0.5214877 | -0.7865511 | -1.9123967 | -0.6634304 |
| -0.0375552 | 0.9417593 | -0.1331641 | 0.2789569 | 1.1949382 | -0.3989193 | -0.5570503 | 0.1015389 | -0.5494955 |
| -1.2113453 | -1.2087998 | -0.8179764 | -1.2241720 | -1.3311203 | -0.0087370 | -1.2426444 | -1.9464689 | -0.3736208 |
| -0.9824238 | -1.9016664 | -0.7503902 | -0.9689651 | -2.2570420 | -1.2535934 | -1.6176684 | -1.8657674 | -1.5140108 |

| | | | | | | | | |
|---|---|---|---|---|---|---|---|---|
| -0.061534 | -0.153698 | 0.0302044 | -0.102613 | -0.001763 | -0.090260 | -0.308446 | -0.400614 | -0.156856 |
| -1.433584 | -1.989220 | -0.759036 | -1.426063 | -1.927669 | -0.643560 | -1.907960 | -2.244590 | -1.522806 |
| -0.301446 | -0.035862 | -0.444813 | -0.294501 | 0.0372451 | -0.336572 | -0.830697 | -0.770391 | -0.637466 |
| 0.9083100 | 1.9643721 | -0.538828 | 1.0767554 | 2.1020490 | -0.490148 | 0.4873093 | 0.3171435 | -0.425326 |
| -0.896006 | -2.084333 | -0.362923 | -1.031626 | -2.438761 | -0.228479 | -1.049646 | -2.389172 | -0.594738 |
| 1.8002874 | 1.2238071 | 1.1649548 | 1.1165842 | 0.5456633 | -0.319896 | 0.6043462 | 0.2605922 | 0.4098829 |
| -0.175103 | -0.325008 | -0.287552 | -0.373440 | -0.496111 | -0.516384 | -0.698925 | -0.821897 | -0.493853 |
| -0.803821 | -1.285019 | -0.794020 | -0.942767 | -1.277459 | -0.560794 | -0.983051 | -0.277463 | -0.786144 |
| -0.770042 | -1.520218 | -0.589653 | -0.810098 | -1.830852 | -0.687210 | -0.775662 | -1.598733 | -0.551923 |
| -0.619602 | -0.851944 | -0.250992 | -0.796896 | -0.530503 | -0.189926 | -0.632376 | -1.208611 | -0.072521 |
| -0.032715 | -0.320337 | -0.049979 | -0.035084 | -0.002805 | -0.465926 | -0.135330 | -0.424050 | -0.365154 |
| -0.985388 | -0.865605 | -0.551232 | -1.028604 | -0.860817 | -0.330519 | -0.916742 | -1.106215 | -0.621910 |
| -1.150661 | 0.9790255 | -1.087652 | -0.949219 | 1.0376336 | -1.331406 | -2.159958 | -0.653762 | -1.796120 |
| -0.078149 | -0.363022 | -0.185258 | -0.099374 | -0.061660 | -0.390760 | -0.543005 | -0.628894 | -0.323722 |
| -0.312252 | -0.261062 | -0.267920 | -0.299667 | -0.300292 | -0.034093 | -0.461677 | -0.226885 | -0.165914 |
| -0.135991 | -1.474754 | 0.1653123 | -0.187866 | -0.810689 | 0.6482031 | 0.2426505 | -0.571356 | 0.5799325 |
| -0.528689 | -0.859294 | -0.367178 | -0.820508 | -0.689055 | -0.435676 | -0.390569 | -0.330469 | -0.462854 |
| -1.676814 | -1.462031 | -0.764281 | -1.942442 | -2.025950 | 0.0224105 | -0.863552 | -1.031705 | -0.337008 |
| -0.817933 | -0.536422 | -0.509434 | -0.887731 | -1.038647 | -0.596470 | -0.794408 | -1.005325 | -0.823717 |
| -1.117324 | -1.045346 | -0.724170 | -1.151585 | -0.907044 | -0.414579 | -1.163086 | -1.094724 | -0.603573 |
| 0.3418057 | 1.2037620 | -0.142940 | 1.4459745 | 1.9672804 | -0.003320 | 0.3432775 | 0.5751084 | 0.0089177 |
| 0.2649392 | 0.3277135 | 0.0860418 | 0.2835461 | 0.3363230 | -0.400775 | -0.283393 | -0.160260 | -0.604590 |
| -1.223423 | -1.357808 | -0.678181 | -1.286908 | -1.068732 | -0.578490 | -1.221254 | -1.977291 | -0.666310 |
| -0.785347 | -0.901137 | -0.361196 | -0.980095 | -1.319341 | -0.257468 | -0.298228 | -0.936036 | -0.246040 |
| -0.896892 | -1.296746 | -0.360522 | -1.127273 | -1.277962 | -0.305969 | -0.947853 | -1.084445 | -0.365849 |
| -0.631671 | -0.503536 | -0.250426 | -0.551488 | -0.140847 | -0.269734 | -0.782473 | 0.2785758 | -0.462960 |
| -1.498857 | -1.006229 | -0.935448 | -1.942871 | -1.403304 | -0.997917 | -1.252723 | -0.557866 | -1.268725 |
| -0.517393 | -0.144335 | -0.425984 | -0.435509 | 0.2742828 | -0.259367 | -0.219904 | -0.406807 | -0.346194 |
| -1.424217 | -1.214819 | -0.381540 | -1.257360 | -1.339692 | -0.279596 | -1.455443 | -0.572117 | -0.657144 |
| 0.5465055 | 1.2737518 | 0.3960184 | 0.3831532 | 1.0362782 | 0.0950917 | 0.8497998 | 1.4081261 | 0.0466107 |
| 0.5456631 | 2.10927 | -0.146853 | 0.9391794 | 2.4552300 | 0.2540385 | 2.7108527 | 3.7484315 | 0.4537262 |
| -1.310591 | -0.992669 | -0.824783 | -1.277700 | -1.312492 | -0.821813 | -0.856691 | -0.797929 | -0.947056 |
| 0.0440329 | 0.4616212 | -0.015041 | -0.015730 | 0.3297676 | -0.349012 | -0.313137 | 0.1225536 | -0.190903 |
| 0.2869113 | 0.5917093 | 0.1158734 | 0.2817682 | 0.1445448 | -0.027974 | 0.1212229 | 0.5777476 | 0.2100066 |
| 0.8455409 | 0.7227907 | 0.5400692 | 0.7029846 | 0.6030117 | 0.2895215 | 0.8869456 | 0.9913336 | 0.4647857 |
| -0.608857 | -0.013770 | -0.488725 | -0.542680 | -0.000164 | -0.095994 | -0.291163 | -1.376345 | -0.307339 |
| 0.7442011 | 1.0400112 | 0.8117947 | 1.0350358 | 1.2899459 | 0.4601226 | -0.005887 | 0.2420148 | 0.5245184 |
| -0.063700 | -0.437565 | -0.300412 | -0.163176 | -0.429567 | -0.775245 | -0.252088 | -0.355959 | -0.822382 |
| -0.542935 | -0.564242 | -0.280001 | -0.561885 | -0.403765 | -0.260166 | -0.233678 | -0.051693 | -0.404622 |
| -0.808089 | -1.442812 | -0.357808 | -0.955083 | -1.767475 | 0.0281601 | -0.498339 | -1.041825 | -0.066872 |
| -0.827306 | -0.743307 | -0.929806 | -0.919538 | -0.758373 | -0.551988 | -0.549787 | -1.145259 | -0.024054 |
| -0.115285 | 0.3839789 | -0.059363 | 0.2499384 | 0.3273680 | -0.327837 | -0.329772 | 0.0981265 | -0.383126 |
| 0.2775470 | 1.4401511 | 0.1193590 | 0.5262346 | 1.3749817 | -0.683276 | -0.040958 | 0.6642277 | -0.698609 |
| 0.3401078 | 0.5931679 | 0.1648561 | 0.2464155 | 0.2142511 | -0.470505 | -0.198073 | 0.7547908 | -0.616843 |
| -0.431617 | -0.295659 | -0.162258 | -0.648462 | -0.257302 | -0.445780 | -0.776396 | -0.976879 | -0.392935 |
| 0.0914337 | 0.3321854 | 0.0601379 | 0.1253605 | 0.0660506 | -0.211046 | -0.514910 | -0.423550 | -0.155613 |
| -0.792194 | -1.421239 | -0.609225 | -0.868553 | -1.234303 | -0.308926 | -0.546341 | -1.076178 | -0.255770 |
| -1.481231 | -1.258129 | -0.869202 | -1.617124 | -1.203611 | -0.320749 | -1.112258 | -1.640122 | -0.382294 |
| -0.575934 | -1.375246 | -0.446911 | -0.767830 | -1.131631 | -0.182211 | -0.504255 | -1.223969 | -0.310268 |
| 0.0247385 | -0.141759 | -0.085563 | -0.012337 | -0.166378 | -0.118330 | -0.163326 | -0.554069 | 0.0398701 |

| | | | | | | | | |
|---|---|---|---|---|---|---|---|---|
| -0.1972153 | 0.3633274 | -0.0023557 | -0.1966362 | 0.3916148 | -0.2220444 | -0.0421598 | 0.8476139 | -0.2459611 |
| -0.0049337 | -0.6114434 | -0.4046779 | -0.3128535 | -0.4211886 | -0.0234789 | -0.4389648 | -1.3456690 | -0.0785222 |
| -0.5519081 | -2.1265599 | -0.6690719 | -0.7267949 | -2.0736311 | -0.7753256 | -0.7453083 | -1.9320507 | -1.0483350 |
| -1.8785246 | -3.2935378 | -0.9102137 | -1.9068971 | -2.9910846 | 0.2507094 | -1.1879021 | -3.3916519 | 0.1020732 |
| -0.5295088 | -1.3581924 | -0.4430169 | -0.7218988 | -1.6455721 | -0.4965530 | -0.8852385 | -1.1256701 | -0.6044924 |
| -0.2481005 | -0.4103620 | -0.1154798 | -0.5427614 | -0.4423860 | -0.0665068 | 0.0570166 | 0.2280296 | -0.1070892 |
| 0.8675906 | 0.5538997 | 0.3041822 | 0.5290545 | -0.5892162 | -0.6522425 | 0.0141740 | 0.8072630 | -0.6640201 |
| -0.1329903 | 0.1688410 | 0.0391090 | 0.1586767 | -0.0023999 | -0.6437442 | -0.3150684 | -0.1392881 | -0.2576649 |
| -0.3891289 | -0.6841664 | 0.0823692 | -0.3273292 | -0.3586399 | -0.0728595 | -0.6168472 | -1.7702887 | -0.1183576 |
| -0.5740739 | -1.4578080 | -0.0305809 | -0.7370625 | -1.5207678 | -0.0871387 | -0.5327754 | -1.6787061 | -0.0193427 |
| -0.9696329 | -0.9852218 | -1.3378116 | -1.2943614 | -1.2749890 | -0.3335235 | 0.2703862 | 0.3455077 | -0.6933090 |
| -0.6212083 | -0.5511091 | -0.9542940 | -0.7423751 | -0.5409495 | -1.0075716 | -1.0708554 | -0.3092147 | -1.6450355 |
| -0.1626669 | -0.3781480 | -0.1339078 | -0.2039933 | -0.4569674 | -0.0680442 | 0.0651066 | -0.1111823 | 0.1753999 |
| -0.1070242 | 0.3822719 | -0.2002727 | -0.1788171 | 0.4938897 | 0.0897141 | -0.1108806 | 0.0299642 | -0.0032242 |
| -0.4554996 | -1.1247160 | -0.0885586 | -0.5583209 | -1.2691358 | -0.1852455 | -0.6169439 | -1.7372350 | -0.3486635 |
| -0.5797832 | -0.8456321 | -0.3518929 | -0.6946002 | -0.6664063 | -0.0473697 | -0.1027973 | -0.4170717 | -0.1341220 |
| -0.1771973 | -0.5799431 | 0.0186172 | -0.1814239 | -0.6359529 | 0.0831569 | -0.1702188 | -0.6087811 | 0.2002814 |
| -0.5499443 | -1.1101965 | -0.2348716 | -0.4770429 | -0.5526396 | -0.3321313 | -0.9762508 | -1.9325256 | -0.4286225 |
| -1.1599502 | -1.1889076 | -0.1867378 | -1.5292381 | -1.0943198 | -0.1992826 | -0.9467164 | -1.9802752 | -0.4859527 |
| 0.0819640 | -0.0667461 | 0.1367550 | 0.1943664 | -0.4445838 | 0.3638228 | 0.3473357 | 0.3940359 | 0.6520611 |
| -1.2281010 | -2.2683425 | -0.9895034 | -0.9974403 | -2.3932591 | -1.1073239 | -1.5864027 | -1.7494062 | -1.2205965 |
| -0.2890594 | -0.3567756 | -0.3529863 | -0.3016767 | -0.3367782 | -0.3221579 | -0.2439536 | -0.6473187 | -0.2497085 |
| -2.1230960 | -2.5682246 | -1.0954303 | -2.4164169 | -2.3224026 | -0.4838914 | -1.6594402 | -3.3122735 | -0.7018432 |
| -0.0844870 | 0.2485489 | -0.1156901 | 0.1327458 | 0.4379635 | -0.2932692 | -0.2808946 | 0.3757654 | -0.2419500 |
| -0.4779342 | -1.0211271 | -0.4341419 | -0.7284044 | -1.4497989 | -0.2354739 | -0.5779698 | -1.6004872 | -0.6734263 |
| -0.6849768 | -0.5195569 | -0.6203864 | -1.0371517 | -0.8705272 | -0.8005298 | -0.8978845 | -0.1915235 | -0.7890303 |
| -0.6603601 | -2.5284107 | -0.2333162 | -0.6191644 | -2.0854331 | -0.1240674 | -0.5542851 | -1.9107163 | -0.2738887 |
| -0.6653044 | -0.4098182 | -0.6251510 | -0.7070811 | -0.3550389 | -0.3568345 | -0.6077722 | -0.5358051 | -0.2537402 |
| 0.3237677 | 0.7363301 | 0.1612443 | 0.3542464 | 0.8405621 | -0.2253018 | 0.2502897 | 0.7875854 | -0.1627600 |
| 0.1939379 | 1.7550696 | -0.3044821 | 0.5042644 | 1.8317678 | -0.0271846 | 0.4718082 | 1.8531494 | 0.0452671 |
| -0.6218296 | -1.3185866 | -0.5227156 | -1.3138447 | -1.7945478 | -0.9300688 | -1.1818151 | -1.4992680 | -1.0766445 |
| -0.2920937 | -1.1139738 | 0.0737722 | -0.2262962 | -1.0499124 | -0.1054272 | -0.2788527 | -0.5527651 | -0.1441268 |
| -0.3808767 | 0.1221021 | -0.4690272 | -0.5117708 | -0.2909692 | -0.9249389 | -0.2599998 | 0.4204473 | -0.7676664 |
| -0.2240107 | -0.8397823 | -0.1604519 | -0.5431482 | -0.7125162 | -0.3113723 | -0.4910486 | -1.1634471 | -0.3897156 |
| -0.5139707 | -0.8610090 | -0.3735209 | -0.4332324 | -1.0602652 | -0.3565529 | -0.5554139 | -0.9024531 | -0.4807574 |
| -0.5451839 | -1.8034021 | -0.3348099 | -0.5727051 | -1.9711651 | -0.1579121 | -0.4004514 | -1.9030043 | -0.1514981 |
| -1.5969803 | -1.3691361 | -0.9276549 | -1.4810410 | -0.5174962 | -0.9308980 | -1.3324694 | -1.7783383 | -0.8632729 |
| -0.0874357 | -0.0671924 | -0.1781107 | -0.1566538 | -0.0329262 | -0.2369917 | -0.1966993 | -0.4813658 | -0.1164221 |
| 0.2112068 | 0.4842849 | -0.3506237 | 0.0560189 | -0.3643959 | -0.3652315 | 0.5393933 | 1.0607258 | -0.3730137 |
| -0.2014922 | -1.0301056 | -0.0408638 | -0.3355038 | -1.2169063 | 0.1024317 | -0.1426080 | -0.5833649 | 0.0907340 |
| 0.3924944 | 1.0425987 | 0.1548913 | 0.5379072 | 1.0661133 | 0.1034905 | 0.1917951 | 0.6475579 | -0.0645266 |
| -0.6612604 | -1.6281616 | -0.3311991 | -1.0685408 | -1.8669499 | -0.1905273 | 0.0361063 | -1.1837434 | -0.1675454 |
| -0.8260048 | -0.8996505 | -0.6792723 | -0.8063245 | -1.0677922 | -0.4103462 | -1.0548579 | -0.7126024 | -0.7927877 |
| -0.1286992 | 0.6902340 | -0.2580283 | 0.0926010 | 0.8075910 | -0.4254048 | -0.6139716 | 0.0511617 | -0.4181359 |
| -0.5052841 | -0.3224652 | -0.3920040 | -0.5908604 | -0.2020682 | -0.2120240 | -0.6731971 | -1.0388577 | -0.4800544 |
| -0.4325697 | -0.6764586 | -0.2229866 | -0.3636363 | -1.2133478 | -0.6593402 | -0.8651996 | -0.6807557 | -0.6543690 |
| -0.4898959 | -0.0491260 | 0.0177764 | -0.5834756 | -0.1179997 | -0.0740783 | -0.4597039 | -0.6460517 | -0.3161667 |
| -1.6461527 | -2.0999100 | -1.7607219 | -1.7345910 | -2.1189565 | -0.5504568 | -0.9214634 | -0.8702490 | -1.4774051 |
| -0.2566248 | 0.1467944 | -0.1610239 | -0.1331470 | 0.6444279 | 0.0254530 | -0.4039418 | 0.0562550 | -0.0204033 |
| -1.0850048 | -1.8197121 | -0.8404159 | -1.0136526 | -1.7270386 | -0.4762216 | -1.1906068 | -1.5661016 | -0.8028334 |

| | | | | | | | | |
|---|---|---|---|---|---|---|---|---|
| 0.3459771 | 0.9241805 | 0.1804819 | 0.6015757 | 1.6057904 | -0.2990773 | -1.2036509 | -0.913610 | -0.4482047 |
| -0.2987915 | 0.0552722 | -0.105552 | 0.0127710 | 0.0938075 | -0.0533215 | -0.2100322 | 0.2085512 | -0.1701566 |
| 0.0561509 | -0.3546940 | -0.1238778 | -0.2996177 | -0.4481143 | -0.3379383 | -0.4074408 | -0.4088725 | -0.3335143 |

| PG_570 | PG_5703 | PG_571 | PG_572 | PG_5728 | PG_573 | PG_574 | PG_575 | PG_576 |
|---|---|---|---|---|---|---|---|---|
| -0.656676 | -0.295023 | -0.991595 | -0.625613 | 0.3333697 | -1.142987 | -1.117673 | -0.368371 | -0.976153 |
| -0.927403 | -0.154888 | -0.911689 | -0.688449 | 0.3480480 | -0.733497 | -1.065494 | -0.813801 | -0.552593 |
| -1.306920 | 0.1260501 | -1.499468 | -0.678759 | -0.802480 | -1.301918 | -1.946053 | -0.957440 | -2.163464 |
| -0.555063 | -0.003753 | -0.652995 | -0.247136 | 0.4962701 | -0.130817 | 0.4394499 | -0.160369 | -0.365112 |
| -0.675405 | 0.0183718 | -1.164075 | 0.0286774 | -0.466075 | -0.092569 | -1.095365 | -0.122958 | -0.529990 |
| -1.143161 | -0.344436 | -2.535837 | -0.096521 | 0.4818697 | -0.199171 | -1.321845 | -0.347063 | -0.495196 |
| -1.647126 | -0.389008 | -1.184717 | -0.441752 | -0.715184 | -0.923549 | -0.156687 | -0.230647 | -1.237038 |
| -0.114752 | -0.084397 | -1.658177 | -0.263054 | 0.1506182 | -0.512293 | -1.995455 | -0.253240 | -0.486466 |
| -0.170060 | 0.0003258 | 0.3840142 | -0.605879 | -0.140999 | -0.644698 | -0.592554 | -0.346907 | -0.666437 |
| -0.321060 | -0.510484 | 0.6834359 | -0.585921 | -1.069428 | -1.237259 | -0.891435 | -0.936087 | -1.636387 |
| -0.619522 | -0.070425 | -0.890523 | -0.475059 | 0.7224657 | -0.191382 | -0.531793 | -0.447183 | -0.505234 |
| -0.682702 | 0.0761671 | -0.758880 | -0.096929 | -0.456149 | -0.147814 | -0.263770 | -0.133649 | -0.241930 |
| -1.619727 | -0.520442 | -2.136477 | -0.539482 | -1.433988 | -0.475734 | -0.806176 | -0.443538 | -1.155588 |
| 3.6078306 | 2.7830808 | 4.6303198 | -1.464273 | 0.7723467 | 0.4645355 | 4.4452794 | -1.777552 | 3.2232117 |
| 0.0509580 | 0.3475978 | 0.1262006 | 0.0509753 | -1.327414 | -0.135963 | 0.2737975 | 0.1779738 | -0.003108 |
| 2.9876483 | 2.1072978 | 3.1154596 | -0.333859 | 1.1084414 | 2.4744911 | 2.1491366 | 0.3488054 | 3.1112099 |
| -2.059216 | -1.322762 | -1.832914 | -1.640138 | 0.4865566 | -2.234576 | -1.667627 | -1.691309 | -1.984262 |
| -0.528596 | -1.060356 | 1.2315824 | 0.1103407 | -0.653169 | -1.882791 | -0.020192 | -0.881084 | -1.403724 |
| -2.378353 | -1.183503 | -1.373985 | -1.736110 | -1.379285 | -1.985438 | -2.084206 | -1.735244 | -1.874976 |
| -0.740205 | -0.675524 | -0.795356 | -0.759063 | -1.082327 | -0.666713 | -0.659854 | -0.663723 | -0.780782 |
| -0.320475 | 0.3718957 | -0.373696 | -0.446830 | 1.1710054 | 0.0177807 | -0.022827 | -0.456816 | -0.245386 |
| -0.383430 | -1.024591 | -0.506344 | -0.439240 | -0.713907 | -0.140732 | -0.402228 | -0.338394 | -0.529958 |
| 0.8154520 | 0.9051496 | 1.5538933 | -0.315952 | 1.0519831 | 0.7977914 | 1.8300900 | 0.2998166 | 1.0100293 |
| -1.539752 | -0.162797 | -1.690418 | -0.560270 | 0.0203378 | -0.854558 | -1.716947 | -0.607209 | -1.262683 |
| -1.284161 | -0.196261 | -1.434310 | -0.465044 | 0.3334295 | -1.382159 | -1.152089 | -1.031361 | -1.880195 |
| 0.3314077 | 0.1411180 | 1.1875054 | -0.100244 | 0.7103576 | 0.1274194 | 0.6390597 | -0.051998 | 0.2751640 |
| -0.073277 | -0.000381 | -0.341885 | -0.013209 | 0.1943656 | -0.198730 | -0.258823 | -0.091074 | -0.071767 |
| -1.523862 | -0.405159 | -2.479741 | -1.310438 | 0.5256300 | -1.227216 | -1.666799 | -1.439750 | -1.283107 |
| -1.323501 | -0.542484 | -1.733620 | -0.189699 | -0.942917 | -0.815170 | -2.079636 | -0.203337 | -1.312068 |
| -0.161167 | -0.169082 | -0.302906 | -0.127266 | 1.1352846 | -0.103253 | -0.136582 | -0.055161 | -0.409872 |
| -0.273489 | -0.223888 | -0.024578 | 0.0876023 | -0.547045 | -0.621652 | 0.0376197 | 0.0577276 | -0.696946 |
| -0.024672 | -1.460577 | -0.237348 | -0.204710 | 0.7167956 | -0.471204 | -1.517488 | -0.166964 | -0.729832 |
| 0.3967925 | 0.2830900 | 0.5396976 | -0.101172 | -0.608366 | -0.321392 | -0.142168 | -0.189869 | 0.4289892 |
| -1.155396 | -0.684432 | -2.823706 | -0.437364 | -1.831799 | -0.309419 | -1.908065 | -0.316313 | -0.999511 |
| -0.200012 | -2.144896 | -0.259767 | -0.247450 | 0.8564445 | -0.541486 | -0.897739 | -0.293172 | -0.444592 |
| -0.592412 | -0.750400 | -0.088635 | -0.305788 | 0.8393637 | -1.445749 | -0.698022 | -0.949655 | -1.265747 |
| -0.541017 | -0.697262 | -1.832108 | -0.060011 | -2.469930 | -0.375823 | -0.498084 | -0.340668 | -0.670559 |
| 3.5050690 | 3.2474488 | 1.4989044 | -0.632195 | 2.0636229 | 1.7822467 | 0.7549458 | -0.212511 | 3.9534159 |
| 0.0627016 | -0.296452 | -1.829704 | 0.0711088 | 0.2073930 | -0.290959 | 0.1937930 | -0.096179 | 0.6218293 |
| -0.875418 | -1.073824 | 0.8690647 | -0.924899 | 0.2566913 | -1.547433 | 0.8471170 | -1.444568 | -0.397350 |
| -0.249678 | -0.011232 | 0.3015443 | -0.202625 | 0.6024427 | -0.203713 | 0.2439561 | -0.034791 | 0.4083658 |
| -1.257630 | -0.712374 | -1.332410 | -0.339118 | -1.508238 | -1.190851 | -2.272342 | -0.517514 | -1.279438 |
| 0.5812266 | -0.413412 | 0.7503410 | -0.421863 | 0.2602437 | -0.276560 | -0.287377 | -0.359773 | -0.136987 |
| -0.568383 | 0.2496298 | -1.596790 | -0.342587 | 0.2848335 | -0.594982 | -1.119151 | -0.434938 | -0.971301 |
| 0.2860992 | 0.9433824 | 1.3302287 | -0.304180 | 0.8590078 | 0.4548031 | 0.4662398 | -0.115457 | 0.2812822 |
| 0.1244155 | 0.3099339 | 4.3076010 | -0.912879 | 3.4074787 | 0.0850158 | 2.8918408 | -0.907440 | 1.6553554 |
| -0.242072 | 0.3163019 | -0.374734 | -0.427889 | -0.956040 | 0.0045682 | -0.358767 | -0.565092 | -0.318095 |

| | | | | | | | | |
|---|---|---|---|---|---|---|---|---|
| -0.359280 | -0.002944 | -0.300137 | -0.470171 | 0.2769554 | -0.359918 | 0.2794911 | -0.457978 | 0.1360191 |
| -1.140731 | 0.3355488 | -3.076130 | -0.532112 | 0.2860527 | -0.678195 | -1.198276 | -0.653933 | -0.727145 |
| 0.0154749 | 0.1893377 | 0.1833932 | -0.606690 | 0.1689077 | -0.062488 | -0.095109 | -0.470084 | 0.0464878 |
| -1.270084 | 1.3237734 | -0.016768 | -1.362330 | 1.5418298 | -0.434004 | 2.7256634 | -0.970275 | 1.1278583 |
| -0.536234 | -0.182269 | -2.044505 | -0.824905 | 0.0406846 | -0.959059 | -1.133159 | -0.808704 | -1.089778 |
| -0.242817 | -0.643437 | 0.0879607 | -0.365763 | 0.0695832 | -0.042639 | -0.164163 | -0.279769 | -0.194467 |
| -0.961309 | -0.565575 | -1.977648 | -0.238720 | -0.431953 | -0.426660 | -0.616541 | -0.547792 | -1.018560 |
| 2.9528685 | 2.3070975 | 3.3422815 | -0.669811 | 0.7661237 | 0.4735552 | 1.6394586 | -1.021795 | 1.9697054 |
| 0.7383910 | 3.4404143 | -0.257033 | -0.617402 | -1.216226 | 3.0953726 | -0.728012 | -0.744053 | 4.8486402 |
| -0.713852 | -0.100246 | -0.657702 | -0.726574 | -0.715602 | -0.656107 | -0.571153 | -0.743631 | -0.652951 |
| -1.346547 | -0.585425 | -1.518903 | -0.870560 | -1.575613 | -1.359444 | -1.383468 | -0.811265 | -1.323143 |
| 2.8108728 | 2.2687826 | 2.8107898 | 0.1122613 | 0.9638260 | 2.9319954 | 3.1058671 | 1.1794739 | 3.8048400 |
| 2.9589670 | 2.2851278 | 2.8731857 | -0.598042 | -0.123099 | 1.5882352 | 1.5636758 | -0.020980 | 2.4998486 |
| -0.300222 | 0.0751920 | -0.723204 | -0.957055 | -0.313787 | -0.435230 | -0.805601 | -0.505124 | -0.882736 |
| -1.863427 | -0.475464 | -1.727737 | -1.232402 | 0.2598516 | -1.716985 | -1.713648 | -1.416221 | -2.191757 |
| -0.010586 | -0.102326 | 0.7599760 | -0.677944 | -0.694993 | -0.638253 | -0.621078 | -0.456079 | -0.389283 |
| -1.275924 | 0.1725426 | -1.433150 | -0.537903 | -0.447647 | -0.729348 | -1.623882 | -0.550893 | -1.159481 |
| -0.541667 | -0.272196 | -0.704161 | -0.353014 | 0.9301774 | -0.398564 | -0.669943 | -0.346923 | -0.675473 |
| -0.686118 | -0.645052 | -1.511921 | -0.354273 | 0.1743244 | -0.631796 | -0.922255 | -0.100418 | -1.337027 |
| 0.6642743 | -0.033876 | -0.127228 | 0.0723971 | -0.627680 | -0.038905 | 0.0692949 | 0.2708691 | 0.1745608 |
| -0.104866 | -1.332866 | 0.2172679 | -0.275920 | -0.283340 | -0.375135 | -1.682492 | -0.259860 | -0.422116 |
| -1.488581 | -0.782337 | -2.014990 | -0.481322 | -2.736538 | -0.528849 | -0.872299 | -0.353799 | -0.882446 |
| -0.006690 | -0.168251 | 0.8168396 | -0.325000 | 0.0166174 | -0.923034 | 0.1589948 | -0.615384 | -0.380355 |
| 0.4608137 | 0.3084076 | -0.234410 | 0.5591763 | -0.158419 | 0.1598024 | 0.9727561 | 0.3397146 | 0.7203785 |
| -1.466791 | -0.256259 | -1.728341 | -0.922973 | 0.4678693 | -1.364127 | -1.816533 | -1.046578 | -1.263062 |
| -0.626626 | -0.182435 | -0.270443 | 0.0284339 | 0.6514505 | -0.645965 | -0.020467 | 0.0738298 | -0.395056 |
| -1.230592 | -1.177559 | -1.065252 | -0.387486 | 0.9696477 | -0.909790 | -0.338538 | -0.811616 | -0.325016 |
| 1.1657910 | 1.4198918 | 2.6663971 | 0.0471659 | 1.4631550 | 0.5567957 | 3.3768262 | 0.1637383 | 1.2332534 |
| 2.4103608 | 1.8567664 | 3.2451551 | -0.473422 | 1.8018697 | 0.8849627 | 3.8619889 | -0.444149 | 1.6722320 |
| -0.809233 | -0.406306 | -1.512329 | -0.522998 | -1.066088 | -0.673879 | -1.125259 | -0.548242 | -0.878357 |
| 0.2667269 | -0.065289 | -0.191480 | -0.321071 | -0.154930 | -1.434233 | 0.7569629 | -0.134917 | -1.369478 |
| -0.389911 | -0.070202 | -0.264494 | 0.0828638 | 0.1259973 | -0.313739 | -0.484106 | -0.110377 | -0.086652 |
| -1.669477 | -0.874722 | -1.364483 | -0.655282 | 0.0810075 | -0.778700 | -0.863366 | -0.578478 | -1.215744 |
| -0.764503 | -0.072158 | -0.982763 | -0.269277 | -0.385392 | -0.405401 | -0.924945 | -0.006585 | -0.837673 |
| 0.3526681 | 0.4747321 | 0.5968752 | -0.277592 | 2.6900241 | 1.2342802 | 1.7204785 | -0.023953 | 1.2599808 |
| -1.052037 | -1.436633 | -1.293634 | -0.015533 | -0.013572 | -0.159459 | -1.492659 | -0.142116 | -0.422960 |
| -0.624461 | 0.0394561 | -1.315293 | -0.330216 | -2.045815 | -0.739464 | -1.874347 | 0.3071794 | -0.777697 |
| -1.356146 | -0.167881 | -2.267578 | -0.335455 | 0.1781397 | -0.530446 | -1.165198 | -0.363013 | -1.022614 |
| -0.991013 | -0.471512 | -1.226217 | -0.216818 | -0.220738 | -0.722262 | -1.970728 | -0.282143 | -1.385879 |
| -1.855113 | -0.278452 | -1.234412 | -0.255004 | -2.220255 | -0.875638 | -0.729588 | -0.300283 | -0.892846 |
| -1.015409 | -0.062650 | -1.385618 | -1.093817 | 0.3230077 | -1.023617 | -1.316206 | -0.911232 | -1.009234 |
| 0.3337775 | 0.0180686 | 0.6148289 | 0.0548981 | -0.374074 | -0.657246 | -0.568277 | -0.272805 | -0.689232 |
| 1.9360560 | 2.0408125 | 1.4987778 | -0.505084 | 0.7536490 | 1.8306800 | 4.6736516 | 0.2094568 | 3.2554485 |
| -0.449996 | 0.0327370 | -0.609809 | -0.299902 | 0.6294138 | -0.051218 | -0.352033 | -0.309558 | -0.053678 |
| -0.909926 | -0.521229 | -1.456754 | -0.284224 | 0.1925713 | -0.643160 | -0.852112 | -0.441542 | -0.575142 |
| -0.549207 | 0 | -0.668334 | -0.614613 | 0.6524008 | -0.898260 | -1.026915 | -0.541420 | -0.641772 |
| 0.3832105 | 4.6298940 | 2.6717938 | 0.0058073 | 1.8963897 | 3.1810631 | 1.2480734 | 0.0600363 | 6.6691648 |
| 1.9727639 | 1.5026755 | 1.6844752 | -0.680783 | 0.9490779 | 2.0241965 | 1.5368198 | -0.152018 | 2.6946145 |
| -1.651217 | -0.125934 | -2.011240 | -0.365024 | -0.255733 | -0.695628 | -1.630188 | -0.568950 | -0.139171 |
| -0.403487 | -0.147137 | -0.367424 | -0.726930 | 1.4276639 | -0.780463 | -1.160940 | -0.602091 | -0.508990 |

| | | | | | | | | |
|---|---|---|---|---|---|---|---|---|
| -1.058884 | -0.677560 | -0.880212 | 0.4703248 | -3.331503 | 0.0021911 | -2.674983 | 0.1791811 | -0.419465 |
| -0.587851 | 0.0523705 | -0.014414 | -0.345557 | 0.1894448 | -0.528232 | -0.313132 | -0.440707 | -0.452674 |
| -0.498761 | -0.009759 | -0.305825 | -0.550422 | -0.494367 | -1.344961 | -0.598220 | -0.709092 | -1.210103 |
| -0.069087 | -0.118177 | -0.092838 | -0.178267 | 0.7932040 | -0.205402 | -0.076328 | -0.310785 | -0.172449 |
| -1.927086 | -0.547503 | -1.826990 | -0.651932 | -0.692573 | -1.413225 | -1.069349 | -0.905889 | -1.784027 |
| -0.231641 | -0.147149 | -0.194360 | -0.555087 | -0.276230 | -0.340352 | -1.016044 | -0.299684 | -0.269025 |
| -0.393318 | 1.1679984 | -0.573263 | -0.519094 | -0.381883 | -0.354861 | -0.203970 | -0.250975 | 0.0058475 |
| 0.0102009 | 0 | 0.0057527 | -0.095397 | 0 | 0.0863103 | 0.0438813 | -0.077760 | 0.2178279 |
| -0.298025 | 0.2893230 | -0.364556 | -0.324249 | -0.401084 | -0.528824 | 0.0226406 | -0.643048 | -0.187006 |
| 1.3189924 | 0.5862309 | 0.9218659 | -0.050790 | 0.9070764 | 0.9202374 | 1.2619090 | 0.3126828 | 0.8184117 |
| -0.176783 | 0.1292107 | -0.741014 | -0.277868 | -1.549441 | -0.752301 | -1.709415 | -0.430793 | -0.827478 |
| -0.958042 | -0.511517 | -1.725156 | -1.032588 | 0.0730713 | -0.769640 | -1.032829 | -0.660657 | -1.186442 |
| -1.205820 | -0.625242 | -3.039820 | -0.539955 | -2.401945 | -0.584088 | -2.285378 | -0.459277 | -1.088405 |
| -0.527962 | -0.279594 | -1.850164 | -0.083257 | -1.462266 | -0.240908 | -0.936765 | -0.326478 | -0.312876 |
| -0.918955 | 0.1815684 | -1.536143 | -0.161784 | 0.4866251 | -0.325654 | -0.192660 | -0.092101 | -0.383694 |
| 0.1355728 | 1.0551263 | 0.0505637 | -0.090608 | -2.388469 | -0.410389 | -0.209391 | 0.0820506 | -0.067104 |
| -1.106234 | -0.128255 | -1.577767 | -0.773005 | -0.478426 | -1.283576 | -1.122357 | -0.845901 | -1.747045 |
| -0.309668 | -0.629169 | 0.2029715 | -0.131607 | -1.193522 | 0.1032657 | -0.147318 | -0.250508 | -0.182800 |
| -0.697381 | -0.355677 | -0.601692 | -0.056993 | 0.3265733 | -0.347130 | -0.595219 | -0.008825 | -1.008003 |
| -0.088326 | 0.0051361 | 1.5164279 | -0.173989 | 0.3209351 | -1.121566 | 1.0243827 | -0.502757 | 0.1716978 |
| -0.449132 | -0.399708 | -1.094704 | -0.554473 | 0.2057993 | -0.834080 | -2.147330 | -0.692424 | -1.128940 |
| -0.981960 | 0.2744712 | -0.793554 | -0.498458 | 1.7096412 | -1.022560 | -1.563362 | -0.704803 | -1.034214 |
| -0.129744 | -0.809803 | -0.265450 | -0.435502 | 0.8799971 | -2.282350 | -2.700814 | -0.543318 | -2.705986 |
| -1.239260 | -0.423659 | -1.538039 | -0.296558 | -1.819609 | -1.247985 | -1.208779 | -0.423846 | -1.432675 |
| -0.245373 | -0.669185 | 0.1839976 | -0.384148 | -1.418783 | -1.592413 | -2.133856 | -0.500506 | -1.214764 |
| -1.014684 | 0.0966749 | -0.917464 | -0.686557 | -0.227192 | -0.807784 | -0.601266 | -0.273676 | -0.708940 |
| -0.257087 | -0.155624 | -0.463777 | -0.165722 | 1.1151832 | -0.390384 | 0.0199458 | -0.087716 | -0.299478 |
| 0.0689037 | 0.6581002 | -0.119734 | -0.353350 | 0.7585389 | -0.410357 | -0.654468 | -0.144986 | 0.1247212 |
| -1.067410 | -0.960203 | -0.611403 | -0.098481 | -0.519047 | -1.211561 | -0.479816 | -0.113086 | -1.277771 |
| -0.455344 | -0.504699 | -1.005968 | -0.148758 | -1.043399 | -0.282594 | 0.0060895 | -0.121477 | 0.3271458 |
| -1.233572 | -1.084943 | -1.086785 | -0.698929 | -0.360866 | -1.252837 | -2.687704 | -0.689008 | -1.375450 |
| -1.151437 | 0.0361683 | -1.855545 | -0.372681 | -0.342074 | -0.940123 | -1.160181 | -0.067674 | -0.316563 |
| -2.055787 | -0.217106 | -2.819675 | -0.481891 | -0.326381 | -1.466295 | -1.342896 | -0.466394 | -1.267900 |
| 0.0875728 | 0.2462697 | -0.506047 | 0.1478874 | 0.6371247 | -0.287636 | -0.154460 | 0.0512517 | 0.0758427 |
| -0.399245 | -0.015616 | -1.522283 | -0.332878 | -0.083778 | -0.704217 | -1.748903 | -0.588567 | -0.962129 |
| -1.258732 | -0.177486 | -0.948366 | -0.585258 | 0.3116090 | -0.486219 | -0.937591 | -0.700610 | -1.367212 |
| -0.678515 | -0.229540 | -0.632685 | -0.448059 | 1.2903882 | -0.397007 | -0.166554 | -0.357651 | -0.310446 |
| -0.317086 | -0.021179 | -1.330357 | -0.051200 | 0.0675644 | -0.267939 | -0.590715 | -0.134599 | -0.266301 |
| -0.244130 | -0.912575 | -0.418407 | -0.270926 | -0.538521 | -0.611240 | -0.559672 | -0.508577 | -0.831891 |
| 0.0462504 | -0.413829 | -1.366781 | -0.014745 | -0.932861 | -1.190249 | -1.364101 | -0.143842 | -0.847109 |
| 0.3303181 | 0.4620415 | -0.204950 | -0.538297 | 1.6545776 | 0.3766867 | 2.0532976 | -0.529769 | 0.3282855 |
| -0.182750 | 0.0189336 | -1.152089 | 0.0888039 | -1.760146 | -0.096170 | -0.259844 | 0.2188049 | -0.045983 |
| -0.434468 | -0.029781 | -0.635933 | 0.0975929 | -0.599196 | -0.105506 | -0.181116 | -0.002240 | -0.231857 |
| -0.488479 | 0.5061700 | -0.306882 | -0.908767 | -0.834114 | -1.460172 | -0.811896 | -0.895029 | -0.937203 |
| -0.709088 | -0.768612 | -2.261141 | -0.594582 | -0.510268 | -0.630742 | -0.466556 | -0.574482 | -0.626585 |
| 0.1170545 | 0.3406709 | -0.265009 | -0.360276 | -0.156508 | 0.1147148 | -0.146259 | -0.101417 | 0.2733995 |
| -1.236575 | -0.390400 | -1.480945 | -0.408784 | 0.5037220 | -0.596556 | -0.880190 | -0.506654 | -0.033276 |
| -0.211031 | 1.1086377 | 0.1391857 | -0.508808 | 0.0934797 | -0.112915 | 0.3624825 | -0.430817 | 0.4422885 |
| -1.761872 | 0.0753709 | -1.799843 | -0.226350 | -0.393289 | -1.484298 | -2.218956 | -0.466107 | -2.131481 |
| -1.890020 | -0.341160 | -2.342511 | -0.927479 | -0.722348 | -1.221481 | -1.450638 | -0.885178 | -0.951129 |

| | | | | | | | | |
|---|---|---|---|---|---|---|---|---|
| -0.1935763 | 0.0660111 | -0.3769737 | 0.4633278 | 0.4599334 | -0.1130491 | 0.4834239 | 0.3228136 | 0.2906473 |
| -1.9521561 | 0.0302028 | -1.8367082 | -1.0981272 | -0.4820468 | -1.6884304 | -1.4733777 | -1.0781035 | -1.5852119 |
| -0.6721428 | -0.0271703 | -0.6320997 | -0.5025621 | 0.7445071 | -0.5438454 | -0.0931182 | -0.6380271 | -0.5331018 |
| 0.4902738 | 0.6554667 | 0.1907779 | -0.8227548 | 0.6508809 | 0.5647666 | 2.4243048 | -0.4323323 | 2.9426484 |
| -1.4434592 | -0.1447247 | -2.5782878 | -0.6158411 | -1.0297591 | -1.0686107 | -1.2681717 | -0.8806651 | -1.4236148 |
| 0.5949142 | 1.8026113 | 0.5572598 | -0.2762110 | 2.0292288 | 0.7248454 | 1.7741215 | 0.1762601 | 1.6543952 |
| -0.8248860 | 0.0532934 | -1.0053060 | -0.6163365 | 0.6318238 | -0.8560063 | -0.7922031 | -0.9364889 | -0.9687734 |
| -1.2302680 | -0.2468261 | -0.9887167 | -0.0075190 | -0.0332887 | -0.0252932 | -0.6317368 | -0.2693473 | -0.6616984 |
| -1.0251225 | -0.5671508 | -1.8128079 | -0.4647670 | -0.2809278 | -0.9480752 | -1.5553607 | -0.4601608 | -0.8101094 |
| -0.5689238 | -0.0961955 | -0.8477542 | -0.7220835 | 0.5696580 | -1.2818417 | 0.2277050 | -0.7762816 | -0.5191390 |
| 0.1149822 | 1.2686409 | 0.0112561 | -0.0770037 | -1.8929134 | 0.3347400 | 0.5622772 | 0.1202761 | 0.6058721 |
| -0.9961589 | -1.0654406 | -0.9375344 | -0.8826442 | -0.0261185 | -1.2337419 | -1.1485728 | -1.0955927 | -1.1959398 |
| -1.8715773 | -1.2543616 | -0.4820973 | -0.2924137 | 0.6480332 | -0.9544710 | -0.1968885 | -0.4126923 | -1.1196552 |
| -0.4702300 | -0.1069630 | -0.0771368 | -0.1433783 | -0.5792685 | -0.2909007 | 0.2113838 | -0.3015037 | 0.3592041 |
| -0.2677989 | -0.0559639 | -0.4603115 | -0.4476193 | -2.0623636 | -0.2299429 | -0.4332788 | -0.6901234 | -0.7329431 |
| 0.0866568 | -0.8522893 | -1.0133492 | -0.6548426 | -0.6792161 | -0.0860030 | -1.2817967 | -0.8137525 | -1.0119247 |
| -0.6451942 | -0.0857369 | 0.2728692 | -0.3648817 | -1.3077256 | -0.6702757 | -0.3008541 | 0.0762036 | 0.1509997 |
| -1.6151758 | -1.0805154 | -1.5205139 | -0.4732712 | 0.1135924 | -1.0484199 | -0.8146859 | -0.7693653 | -2.4180458 |
| -1.0424355 | -0.7334721 | -0.8741172 | -0.4342672 | 1.1254198 | -0.3424950 | -0.6631252 | -0.3723002 | -0.4885634 |
| -1.1397249 | -0.5823017 | -1.4148102 | -0.3809695 | 0.3739194 | -0.7177604 | -0.9907846 | -0.4593155 | -0.8481985 |
| 0.7867056 | 1.9667589 | 0.8062218 | -0.3843756 | -1.3155272 | 0.1166603 | -0.1140274 | -0.3357481 | -0.0178263 |
| -0.0809535 | 0.1173464 | -0.7264657 | 0.1031300 | -0.0423444 | -0.1814073 | 0.2170207 | -0.4080240 | 0.3601121 |
| -1.8687779 | -0.6422284 | -1.9329509 | -0.3936986 | 0.1133654 | -1.1877108 | -1.2431555 | -0.5700096 | -1.4085404 |
| -0.2022569 | 0.1531324 | -1.0996924 | -0.6204911 | -0.6701068 | -0.5973736 | -0.6371540 | -0.4593653 | -0.6359933 |
| -1.2106684 | -0.3343742 | -1.2927028 | -0.3974169 | -1.6952600 | -0.7084772 | -1.0749484 | -0.3969680 | -0.9606054 |
| -1.0068216 | -0.2370250 | -0.0350081 | -0.4455503 | -1.6800116 | -0.9034684 | -0.3845813 | -0.5166672 | -1.0861359 |
| -1.3253826 | 0.0173374 | -0.8180811 | -0.4110180 | -0.5859793 | -0.5415606 | 0.0209368 | -0.3799951 | -0.5245899 |
| -0.3958031 | -0.1026889 | -0.0463894 | -0.2868503 | -0.0463342 | -0.1297243 | -0.2785332 | -0.5233385 | -0.5798969 |
| -1.1805300 | -0.9575559 | -1.0266760 | -0.5037311 | 0.1784714 | -1.5418150 | -1.2178716 | -0.8508050 | -1.7122351 |
| 0.8221439 | 0.6475742 | 1.2780652 | 0.1746042 | 1.0953212 | 0.8187442 | 0.7506074 | 0.2357683 | 0.6258227 |
| 3.4717682 | 1.4647466 | 4.1909040 | -0.2963427 | 0.8681323 | 0.2446994 | 2.5013665 | 0.0438552 | 1.4964218 |
| -0.7988276 | -0.3473845 | -1.4127595 | -0.4849095 | -0.2506152 | -1.2152318 | -0.0743269 | -0.2770371 | -1.0676322 |
| -0.3416264 | -0.0907052 | -0.0449954 | -0.3972426 | 0.5696165 | -0.2166445 | 0.3341994 | -0.6054555 | -0.2902366 |
| 0.2667478 | 0.1752745 | 0.5118720 | 0.0116129 | 0.0579111 | 0.2905016 | 0.7934181 | 0.1082490 | 0.1791258 |
| 0.7577429 | 0.8177310 | 0.8005677 | 0.5021397 | 0.4977682 | 0.7814293 | 1.3252721 | 0.5523237 | 1.0317426 |
| -0.4135828 | -0.7290114 | -2.0441054 | -0.1161245 | 0.0682451 | -0.3220205 | -0.5426673 | -0.3107797 | -0.1346062 |
| 0.5781162 | -0.3941500 | -0.4421860 | -0.4078029 | 0.3746690 | 0.3370233 | 1.8146291 | 0.2405219 | 0.9016752 |
| 0.1228005 | 0.8663945 | -0.0733786 | -0.5960193 | 0.8432114 | 0.0280193 | 0.3404293 | -0.3601886 | 0.2823069 |
| -0.3805848 | -0.0214597 | -0.2548196 | -0.3744675 | -0.0531853 | -0.4585305 | 0.0594125 | -0.3724203 | -0.1513888 |
| -0.6685602 | 0.0112378 | -1.0795086 | -0.1665885 | -1.1020676 | -0.4157115 | -0.6256403 | -0.2533170 | -0.2945617 |
| -0.9975527 | -0.2613877 | -0.9031481 | -0.6259655 | -0.4685245 | -1.9051120 | -1.4782654 | -0.7273002 | -1.3123105 |
| -0.1808467 | 0.2331174 | -0.0774646 | -0.4775796 | 0.8871439 | -0.0857461 | 0.2964606 | -0.0284793 | 0.6601022 |
| -0.1506210 | 0.3293071 | 0.3644909 | -0.4711275 | 1.0567275 | 0.2275485 | 1.1158270 | -0.3213188 | 0.8200249 |
| -0.0499066 | 0.6764587 | 0.3313197 | -0.1546672 | -0.1502906 | 0.2068360 | 0.3915217 | 0.0012962 | 0.2717123 |
| -0.9762079 | -0.2835094 | -0.7736921 | -0.8255690 | 0.0084481 | -0.8140258 | -0.9057975 | -0.7040183 | -1.0296044 |
| -0.5285650 | 0.1796930 | -0.4271084 | -0.0143576 | 0.3746724 | -0.0541740 | 0.7886244 | -0.0156434 | 0.0915640 |
| -0.7298393 | -0.2663286 | -1.0547430 | -0.5874060 | -0.8744726 | -1.1190612 | -1.6466221 | -0.5113768 | -1.3210122 |
| -1.2695861 | -0.9865575 | -1.2559874 | -0.3686630 | -0.4926379 | -0.9737345 | -0.4300521 | -0.5623749 | -0.8214485 |
| -1.0341658 | -0.3577523 | -1.3447237 | -0.1242329 | 0.4578164 | -0.4539275 | -1.1715190 | -0.1068243 | -0.9415173 |
| -0.2523879 | 0.2010307 | -0.8306934 | -0.3435646 | 0.6596408 | -0.2196312 | -0.0972309 | -0.3239946 | -0.1905226 |

| | | | | | | | | |
|---|---|---|---|---|---|---|---|---|
| 0.2567965 | -0.477527 | 0.5447858 | -0.204736 | 0.5473410 | -0.538858 | 0.5346452 | -0.100235 | -0.095960 |
| -0.397122 | 0.0809011 | -1.212189 | -0.647997 | -0.860436 | -1.394631 | -1.256824 | -0.222304 | -1.127199 |
| -0.470252 | 0.5032872 | -2.158113 | -0.443144 | -1.254734 | -0.135783 | 0.0168550 | -0.709630 | 0.1960683 |
| -1.719418 | -0.658123 | -3.432833 | 0.2203067 | -0.570411 | -0.734603 | -1.754475 | -0.161898 | -0.849832 |
| -0.907711 | -0.084682 | -1.489603 | -0.395860 | -0.170389 | -0.949932 | -1.160026 | -0.678880 | -1.161791 |
| -0.249279 | 0.1443531 | 0.3566534 | -0.394362 | -0.258780 | -0.058912 | -0.081773 | -0.236797 | 0.0758555 |
| 0.1830637 | 0.9309411 | -0.236891 | -0.656404 | 3.1006722 | 0.5342763 | 1.9014447 | -0.611948 | 0.3387904 |
| -0.450908 | NA | 0.2341823 | -0.662935 | NA | -0.538699 | -0.390938 | -0.579249 | -0.783178 |
| -0.877916 | -0.492274 | -1.738374 | -0.546958 | -0.451744 | -0.403537 | -1.081806 | -0.388602 | -0.854819 |
| -0.468616 | -0.353645 | -1.753300 | -0.330568 | -0.990866 | -0.696819 | -0.394595 | -0.231637 | -0.188097 |
| 0.1959021 | -0.106490 | 0.0578853 | -0.290459 | 0.2152850 | -0.248991 | 0.3012047 | -0.349312 | 0.1593219 |
| -0.777185 | -1.042164 | -0.594057 | -0.679969 | 0.4552837 | -0.984309 | -0.353901 | -1.019076 | -1.202606 |
| -0.067508 | -1.729042 | -0.186536 | 0.0673125 | -0.625156 | -0.183476 | -0.532562 | 0.0398134 | 0.1369573 |
| -0.271855 | 0.1775017 | -0.292095 | -0.460459 | 0.1736122 | -1.039026 | -0.783134 | -0.482852 | -0.903086 |
| -0.882326 | -0.329648 | -1.965680 | -0.358976 | -0.795306 | -0.660818 | -2.080693 | -0.313729 | -1.290525 |
| -0.011986 | -0.134968 | -0.227335 | -0.201505 | -0.196419 | -0.557052 | -0.301656 | 0.0136071 | -0.116373 |
| -0.427044 | -0.224573 | -1.249886 | -0.274653 | 0.3292863 | -0.293799 | -0.390931 | -0.203838 | -0.591060 |
| -1.023826 | -0.589757 | -1.739047 | -0.207410 | -0.093833 | -1.382356 | -1.049357 | -0.353774 | -1.217501 |
| -1.366886 | -0.358322 | -1.781265 | -0.098340 | -0.062245 | -0.813502 | -0.990670 | -0.356052 | -1.508297 |
| 0.5175987 | NA | -0.292929 | -0.109113 | NA | -0.140254 | 0.6784401 | -0.403310 | 0.3987760 |
| -1.314272 | -0.305532 | -2.268030 | -0.117944 | -0.527387 | -0.715157 | -0.475057 | -0.318223 | -0.379752 |
| -0.373450 | -2.833027 | -0.576102 | -0.900566 | -1.414840 | -0.546845 | -2.374360 | -0.867222 | -0.693167 |
| -2.129397 | -1.346503 | -3.157458 | -1.215519 | -0.185950 | -1.746606 | -3.331004 | -1.188643 | -2.312327 |
| -0.312110 | 0.1098269 | 0.3458873 | -0.152557 | 0.5774106 | -0.190991 | 0.2588059 | -0.049623 | -0.127017 |
| -0.760368 | -0.113906 | -1.395300 | 0.1329878 | -0.048507 | -0.662183 | -1.370827 | -0.013905 | -0.466409 |
| -0.867394 | -0.139705 | -0.246376 | -0.659199 | 0.6879356 | -0.758643 | -0.485266 | -0.584982 | -0.980647 |
| -0.843576 | 0.0908948 | -1.631597 | -0.002615 | -1.205581 | 0.0840397 | -0.656199 | -0.044103 | -0.180136 |
| -0.973410 | NA | -0.170341 | -0.494466 | NA | -0.319888 | -0.376595 | -0.519718 | -0.468764 |
| 0.3914981 | 0.4929547 | 0.7235700 | -0.389834 | 1.2127480 | -0.033174 | 0.6333721 | -0.428563 | 0.2773698 |
| 0.7675992 | 0.2667858 | 1.6624456 | -0.003382 | -0.134753 | -0.274582 | 0.9640513 | -0.039902 | 0.7425420 |
| -1.363853 | 0.0511977 | -1.379417 | -0.797314 | 0.3863259 | -0.249835 | -0.836014 | -0.527085 | -0.284352 |
| -0.285932 | -0.145956 | -0.548311 | -0.254979 | -1.497327 | -0.398368 | -0.717885 | -0.214484 | -0.525867 |
| -0.104129 | -0.043250 | 0.2210687 | -0.867125 | -0.937325 | -0.192469 | 2.9725826 | -0.710770 | 0.0909566 |
| -0.787075 | 0.0752829 | -1.302591 | -0.460449 | 0.2674175 | -0.181029 | -0.595841 | -0.394557 | -0.840586 |
| -0.228102 | -0.686924 | -0.564250 | 0.0569303 | -0.193075 | -0.242383 | -0.384479 | 0.1039899 | -0.019168 |
| -0.632090 | -0.067565 | -2.068534 | -0.307317 | 0.1009940 | -0.607780 | -0.954657 | -0.532276 | -0.752436 |
| -1.888335 | -0.497506 | -1.733652 | -0.804987 | -1.308935 | -0.863838 | -1.433908 | -0.697170 | -1.991239 |
| -0.293714 | 0 | -0.664822 | -0.509165 | -1.706785 | 0.0153202 | -0.307299 | -0.211016 | -0.302846 |
| 0.4792253 | 0.1234031 | 0.8280422 | -0.759277 | 1.2124979 | -0.364394 | 0.1827403 | -0.682113 | -0.138009 |
| -0.385797 | -0.338683 | -1.290412 | -0.383108 | 0.7047439 | -0.792999 | -0.791056 | -0.291459 | -0.797556 |
| 0.1957142 | 0.1309112 | 0.1278782 | -0.121323 | 0.3949297 | -0.294993 | 0.5927277 | -0.332710 | -0.078071 |
| 0.0668734 | -0.471407 | -1.562772 | -0.251132 | -1.564005 | -0.383218 | -0.436027 | -0.534424 | -1.068966 |
| -1.019164 | -0.009062 | -0.536860 | -0.657880 | -0.165225 | -0.805559 | -0.621146 | -0.979813 | -1.149412 |
| -0.584738 | -0.392669 | -0.152911 | -0.553637 | -0.079493 | -0.624729 | 0.0345189 | -0.733976 | -0.009223 |
| -0.816195 | -0.033341 | -0.751863 | -0.634623 | -0.098247 | -0.487492 | -0.201885 | -0.680734 | -0.484316 |
| -0.833811 | 0.0721406 | -0.699210 | -0.822719 | -1.208714 | -0.883904 | -0.705956 | -0.440904 | -1.018683 |
| -0.373207 | 0.2823915 | -0.825921 | -0.096041 | -0.030665 | -0.637261 | -0.356658 | -0.335252 | -0.457859 |
| -0.835027 | -0.136666 | -0.858947 | -1.088040 | 0.0082932 | -0.929024 | -0.705428 | -1.250242 | -1.259601 |
| -0.122713 | 0.5343250 | 0.7728774 | -0.888712 | -0.665468 | 0.0412796 | 0.1097166 | -0.350320 | -0.105714 |
| -1.359051 | -0.364976 | -1.131212 | -0.868749 | -2.288634 | -0.765961 | -1.315626 | -1.002990 | -0.943971 |

| | | | | | | | | |
|---|---|---|---|---|---|---|---|---|
| -0.7583209 | -0.1706942 | -0.0372854 | -0.4202956 | -0.5820377 | -0.3234532 | 0.6164717 | -0.5910934 | 0.3088571 |
| 0.1940557 | -0.1297056 | 0.8072316 | -0.3943388 | -0.1525076 | -0.1788789 | 0.3015740 | 0.0042672 | 0.1341459 |
| -0.4562843 | 0.1562637 | -0.2463654 | -0.1853820 | 0.1697425 | -0.5498542 | -0.0604355 | -0.2269171 | -0.4330634 |

| PG_577 | PG_578 | PG_579 | PG_580 | PG_581 | PG_582 | PG_583 | PG_584 | PG_585 |
|---|---|---|---|---|---|---|---|---|
| -1.172933 | -2.030954 | -2.551554 | -0.920725 | -1.640744 | -1.898460 | -0.137076 | -0.727858 | -0.853986 |
| -1.019707 | -2.728305 | -2.708189 | -2.051402 | -2.949975 | -3.516043 | -0.649625 | -0.595615 | -1.174381 |
| -3.572854 | 0.7735163 | -1.700481 | 0.8734055 | 0.5352698 | -1.532836 | -1.073054 | -1.611943 | -2.535385 |
| -0.046103 | -4.147379 | -3.644300 | -3.336152 | -4.710836 | -2.655474 | -0.035928 | -0.425548 | -0.417172 |
| -0.947380 | -1.954239 | -2.956460 | -2.053343 | -2.738032 | -1.672986 | 0.0994282 | 0.0225414 | -0.341761 |
| -1.650238 | -2.167205 | -2.374331 | -1.840642 | -2.104239 | -3.204692 | -0.758881 | -1.396086 | -2.131738 |
| 0.2939171 | -2.168124 | -1.901815 | -1.683185 | -2.392561 | -2.057983 | -0.152066 | -1.423159 | -0.340231 |
| -2.675311 | -1.292178 | -1.459680 | -0.608101 | -1.187134 | -2.182780 | -0.619461 | -0.911735 | -2.369486 |
| -0.488005 | -0.794921 | -0.219375 | -0.575670 | -0.306379 | -0.876784 | -0.497329 | -0.026500 | -0.064527 |
| -1.674147 | -1.007543 | -0.952890 | -1.152895 | -1.000852 | -1.078428 | -0.653642 | -1.448224 | -2.272791 |
| -0.797785 | -1.939536 | -2.321458 | -1.894541 | -2.961399 | -2.433552 | -0.257964 | -0.489255 | -0.759623 |
| -0.575993 | -2.007831 | -2.253146 | -2.182847 | -2.585865 | -1.597507 | -0.065453 | -0.477533 | -0.507532 |
| -1.591399 | -2.084706 | -1.632195 | -2.304225 | -2.806443 | -2.696024 | -0.160886 | -0.886621 | -1.942536 |
| 6.1117959 | -0.646182 | 0.9132019 | -2.051274 | 1.2592582 | 2.2786330 | -0.757972 | 4.2574947 | 3.3277603 |
| 0.3441204 | -2.292628 | -2.369335 | -1.893567 | -2.363271 | -1.583932 | -0.196863 | -0.839580 | 0.0961319 |
| 2.7084704 | 0.6352714 | 0.6306847 | -0.013782 | 1.7960960 | 1.9359691 | 0.0838051 | 2.2697575 | 2.1921667 |
| -2.218681 | -2.097274 | -2.324567 | -1.044463 | -2.455353 | -2.054238 | -1.001148 | -3.057005 | -1.578773 |
| -0.281424 | -1.218261 | -1.182976 | -0.998241 | -1.734783 | -1.395166 | -0.435902 | -0.011480 | 0.1131485 |
| -2.320731 | 0.5106905 | -0.030129 | 0.0496862 | -0.098562 | 0.5088273 | -0.863180 | -1.420370 | -2.074266 |
| -0.869611 | -1.102989 | -0.060738 | -1.102236 | -0.913507 | -1.002402 | -0.598166 | -0.401176 | -1.458245 |
| -0.041328 | -1.606775 | -1.062081 | -1.949083 | -1.603930 | -0.384833 | -0.231497 | 0.4034089 | 0.0920249 |
| -0.404113 | -0.286664 | 0.2335661 | -0.421920 | -0.297770 | 0.0779987 | -0.308951 | -0.594140 | -0.398073 |
| 1.7007740 | 1.1761910 | 1.7615613 | 0.7662026 | 1.6660971 | 1.4264194 | -0.240102 | 0.8755239 | 1.0163427 |
| -2.000551 | -1.604753 | -1.750038 | -1.227442 | -1.873441 | -3.117736 | -0.210442 | -1.335553 | -1.686014 |
| -2.158814 | -1.585316 | -2.427915 | -1.434718 | -1.645599 | -2.510227 | -0.215363 | -1.130741 | -2.713394 |
| 0.6538276 | 0.8555988 | 1.0956939 | 0.4067434 | 0.8273193 | 1.2164836 | -0.003323 | 0.9124771 | 1.2539014 |
| -0.142504 | -2.598829 | -2.448707 | -2.701527 | -2.860464 | -2.148612 | 0.1553393 | 0.0213896 | -0.185611 |
| -1.976673 | -2.388001 | -2.357935 | -2.151234 | -2.582930 | -2.676518 | -1.493658 | -2.327906 | -3.050794 |
| -3.444217 | -0.996778 | -2.362020 | -0.594807 | -1.387550 | -2.447109 | -0.049351 | -0.639906 | -2.225575 |
| -0.197242 | -1.767693 | -1.721716 | -2.003098 | -1.887839 | -2.056590 | -0.148701 | -0.502716 | -0.057001 |
| -1.140423 | -0.462156 | -0.254188 | -0.078203 | -0.888314 | -2.282653 | -0.460582 | -1.448513 | -2.534830 |
| -2.152587 | 0.6860572 | 0.8957097 | 0.5205175 | 0.6543022 | 0.5137342 | 0.2965331 | -0.104017 | -1.619070 |
| 0.3828005 | -2.264096 | -1.248498 | -1.525012 | -1.328611 | -1.054852 | -0.359954 | 0.2100202 | 0.7511052 |
| -3.639748 | -0.804965 | -1.309675 | -0.903482 | -1.390623 | -2.015398 | -0.417978 | -1.196839 | -2.903394 |
| -1.042058 | 0.0702015 | 0.1271197 | 0.1309260 | 0.0207936 | 0.0875342 | -0.226097 | -0.212752 | -0.284118 |
| -0.537274 | -2.493041 | -2.567603 | -2.030325 | -2.898615 | -2.267719 | -0.416257 | -1.093657 | -1.142175 |
| -2.285016 | -1.084408 | -1.770527 | -1.067056 | -1.798509 | -2.815359 | -0.425528 | -0.490474 | -1.395314 |
| 0.4631897 | 0.9643114 | 0.5509359 | 0.2072016 | 3.4703413 | 0.5524618 | 0.0802484 | 4.5386655 | 1.2412770 |
| 0.2983794 | -2.065931 | -1.482732 | -1.912082 | -1.907874 | -0.531751 | -0.530583 | 0.0015713 | 0.9836303 |
| 1.2786745 | -3.283076 | -1.770432 | -3.298279 | -3.088884 | -1.949797 | -0.459524 | 0.3708383 | 0.2108984 |
| 0.2281045 | 0.9326517 | 2.3470175 | 0.9006952 | 0.7865941 | 2.0297427 | -0.161261 | 0.2032485 | -0.045853 |
| -2.240705 | -0.338228 | -2.016968 | -0.471197 | -0.784123 | -2.383786 | -0.365286 | -1.013960 | -2.441308 |
| -0.254627 | -2.178624 | -2.715796 | -1.987641 | -2.450595 | -2.408131 | 0.2397577 | 0.2878377 | -0.588771 |
| -1.452670 | -3.317402 | -3.726891 | -2.161685 | -3.258625 | -2.635550 | -0.424927 | -0.532462 | -1.718270 |
| 0.4082046 | 1.7125590 | 2.0243718 | 1.2008995 | 1.5793704 | 2.0726925 | 0.2163239 | 0.4579650 | 0.6055685 |
| 5.4093158 | -0.118196 | -0.286338 | -0.078519 | -0.328200 | 0.2130372 | -0.489428 | 0.7878126 | 2.0768797 |
| -0.091883 | 0.3233784 | 0.0283629 | 0.5190172 | 0.9527440 | 0.0044868 | -0.212815 | -0.225307 | -0.187726 |

| | | | | | | | | |
|---|---|---|---|---|---|---|---|---|
| 0.6187495 | -2.162411 | -1.377177 | -2.227312 | -2.654493 | -2.285949 | -0.444268 | 0.0014577 | 0.2045227 |
| -1.534779 | -1.372050 | -1.993041 | -1.321507 | -1.641066 | -1.931901 | -0.274041 | -0.673710 | -1.442997 |
| 0.0008917 | -1.694320 | -1.209644 | -1.926762 | -2.256962 | -0.710220 | -0.750585 | 0.1299097 | 0.2891547 |
| 3.3211979 | 0.4246529 | 0.9453287 | -1.090300 | 1.5685765 | 1.3095228 | -0.546901 | 2.2245999 | 1.2338869 |
| -0.880952 | -0.035600 | -0.018993 | -0.181041 | -0.073743 | 0.5617871 | -1.153190 | -1.060018 | -1.419149 |
| -0.157309 | 0.4237941 | 0.4047692 | 0.6859682 | 1.0264060 | 1.0983613 | -0.233339 | -0.188570 | 0.1356634 |
| -1.933878 | -1.926656 | -1.445343 | -1.598796 | -1.663431 | -2.176952 | -0.176904 | -0.671879 | -1.189325 |
| 2.4388264 | 0.6204780 | 1.9490348 | -0.186809 | 1.8283077 | 2.1710943 | -0.907649 | 1.3237647 | 2.1907931 |
| 0.1290292 | 1.5180978 | 1.1119529 | 0.1780188 | 4.3554814 | 1.7241844 | 0.1246517 | 3.8916589 | -1.963116 |
| -0.615447 | -1.337596 | -1.099235 | -0.876919 | -1.109099 | -0.947403 | -0.956819 | -0.206590 | -0.637290 |
| -1.517143 | -2.525971 | -3.008290 | -2.177724 | -3.027860 | -3.558012 | -0.810357 | -1.348156 | -1.095340 |
| 4.0019801 | 0.7255269 | 1.1847524 | 0.0015864 | 1.8458763 | 2.4900470 | 0.4038478 | 2.6929265 | 2.6723697 |
| 1.6357790 | -1.552767 | -1.554761 | -2.559916 | -1.463600 | -1.632939 | 0.1420830 | 2.3048609 | 0.9211656 |
| -0.850369 | -1.393399 | -1.912390 | -1.044553 | -1.444917 | -1.275734 | -0.574914 | -0.933285 | -0.520525 |
| -2.560996 | -2.165453 | -2.507625 | -2.871198 | -2.789655 | -2.040994 | -1.100435 | -1.751416 | -3.129360 |
| -0.336561 | -1.243971 | -1.718235 | -1.032223 | -0.758600 | -2.169929 | -0.560221 | -0.520645 | 0.2409978 |
| -2.643270 | -1.344641 | -2.966704 | -1.421267 | -2.227325 | -4.082524 | 0.0763097 | -0.554612 | -1.986012 |
| -0.842646 | -0.631911 | -1.014346 | -0.545869 | -1.440636 | -1.570571 | -0.450611 | -0.718110 | -0.513368 |
| -1.199090 | -2.371182 | -2.159861 | -1.837333 | -2.927496 | -3.217255 | -0.159139 | -1.046524 | -0.529690 |
| 0.1402460 | -3.620699 | -3.388389 | -3.937590 | -3.701093 | -3.389314 | -0.043232 | -0.173401 | -0.433259 |
| -1.733633 | -0.526667 | -0.020545 | -0.735967 | -0.986486 | -0.498058 | 0.1349830 | 0.0546158 | -0.464823 |
| -1.567697 | -1.733846 | -1.374524 | -1.240799 | -1.733153 | -2.112581 | -0.570859 | -1.084725 | -0.726387 |
| -0.343478 | 0.8618158 | 1.4654837 | 0.9950088 | 0.9030917 | 0.5675127 | 0.0494022 | -0.215309 | -0.201548 |
| 0.6087272 | -3.271401 | -3.654529 | -3.737164 | -3.207855 | -3.579475 | 0.4682553 | 0.6497245 | 0.4669941 |
| -1.869320 | -2.560853 | -2.768161 | -1.998566 | -2.054487 | -2.706614 | -0.587440 | -1.197177 | -1.520875 |
| 0.2529463 | -1.518180 | -2.015896 | -1.392536 | -1.594527 | -1.793632 | -0.253738 | -0.787869 | -0.407098 |
| -0.071373 | -1.111777 | -3.630496 | -0.323658 | -2.636014 | -4.200470 | -0.438373 | -1.699251 | -1.479466 |
| 3.3549790 | 0.8642471 | 2.4559964 | 0.8647823 | 1.4827384 | 3.4934628 | 0.2650346 | 2.8516668 | 3.7069051 |
| 4.3540497 | -0.433540 | 0.1384739 | -1.741675 | -0.113800 | 1.1259147 | -0.168450 | 2.6252690 | 2.2835428 |
| -1.568116 | -1.012931 | -1.297483 | -0.815999 | -0.593502 | -2.813912 | -0.413968 | -0.811255 | -2.089496 |
| 0.6100799 | -0.795364 | -0.338432 | 0.0656060 | -0.819735 | -0.267332 | 0.1930878 | -0.411637 | -0.150773 |
| -0.305977 | -4.483599 | -3.887226 | -3.814621 | -4.599876 | -3.092408 | -0.269129 | -0.373458 | -0.493216 |
| -1.242293 | -1.947408 | -1.710714 | -1.743186 | -2.421597 | -2.552770 | -0.548687 | -1.766991 | -0.945054 |
| -1.433955 | -2.209727 | -2.797623 | -2.518309 | -3.396491 | -3.053328 | -0.439249 | -1.003395 | -1.514836 |
| 1.4699189 | 1.0347369 | 2.6448465 | 0.3958432 | 1.4724573 | 2.0098255 | -0.417057 | 0.8914532 | 1.7158233 |
| -0.635972 | 0.6174675 | 0.6302214 | 0.1212663 | -0.011347 | -1.145166 | 0.3756972 | -0.064001 | -0.730509 |
| -2.103622 | -0.570657 | -0.786494 | -0.346490 | -0.704983 | -0.649901 | -0.315356 | -0.164676 | -0.442033 |
| -1.684988 | -1.707996 | -1.507465 | -0.402620 | -2.157660 | -2.646608 | -0.252242 | -1.036185 | -2.155437 |
| -2.779004 | 0.6540971 | -1.198956 | 0.4735379 | 0.2445429 | -1.866396 | 0.0462501 | -0.850764 | -2.021656 |
| -1.372952 | -0.446508 | -0.574320 | 0.0184060 | -0.686206 | -1.529148 | -0.896840 | -1.854633 | -1.876537 |
| -1.973962 | -1.502759 | -1.801884 | -1.730356 | -1.829060 | -1.715660 | -0.831265 | -0.857832 | -1.037334 |
| -1.021319 | -1.038859 | -0.883061 | -0.344437 | -1.766284 | -0.656980 | -1.015408 | -2.083408 | -1.257411 |
| 3.9258539 | 1.4125708 | 2.2797846 | 1.1817687 | 2.8624462 | 2.4756267 | -0.060099 | 2.0011155 | 1.9022534 |
| -0.211782 | -1.562095 | -1.313025 | -1.584079 | -2.091945 | -0.294764 | -0.217304 | -0.297443 | -0.101467 |
| -1.122447 | -3.378394 | -2.809864 | -2.383517 | -2.793614 | -2.953846 | -0.143668 | -0.642565 | -0.556357 |
| -0.890025 | 0.0082509 | -0.019232 | -0.143823 | -0.466283 | -0.491819 | -0.706299 | -0.643808 | -0.764094 |
| 5.9575266 | 1.1828736 | 0.6174198 | 0.2241194 | 4.4271634 | 5.1755439 | 0.1891353 | 5.0916929 | 7.2660082 |
| 2.3427027 | -0.177189 | 0.1536835 | -1.647384 | 0.9548893 | 0.6754190 | -0.264196 | 1.8054576 | 1.9335292 |
| -1.028296 | -1.894951 | -2.596947 | -1.821137 | -2.540808 | -1.416789 | -0.204165 | -1.112488 | -2.248381 |
| -1.205762 | -0.652162 | -0.648375 | -0.465280 | -0.926731 | -1.165646 | -0.813376 | -0.461687 | -0.894533 |

| | | | | | | | | |
|---|---|---|---|---|---|---|---|---|
| -2.3605408 | -1.9053369 | -3.4817670 | -1.7809317 | -2.3637959 | -2.8151955 | -0.6866233 | -0.9903620 | -0.7103923 |
| 0.0660216 | -0.4951149 | 0.2552410 | -0.7672908 | -0.5776551 | -0.1248348 | -0.2959703 | -0.3251372 | 0.1224230 |
| -1.0580690 | -0.3965366 | -0.2424339 | -0.3866908 | -0.5943939 | -0.9857573 | -0.6598976 | -1.1783126 | -0.3892756 |
| 0.0662509 | -1.4024051 | -1.0910192 | -1.1339847 | -1.6575259 | -0.7028020 | -0.3190818 | -0.2725906 | 0.0680552 |
| -2.4934532 | -1.5406221 | -2.0236590 | -1.1660318 | -2.6334044 | -2.4541048 | -0.5292386 | -1.3541061 | -1.4649818 |
| -0.7168656 | -0.9652595 | -1.0168334 | -0.9843651 | -1.1452326 | -0.4625552 | -0.7312430 | -1.6829401 | -2.1513912 |
| -0.4745662 | -1.9717352 | -1.4755006 | -1.5789197 | -1.6265356 | -0.9465468 | 0.1300563 | 0.1962813 | 0.0686353 |
| 0.3534861 | 0.1582433 | 0.5932408 | 0.3318146 | 0.3807758 | 0.6058636 | -0.3968220 | -0.0179692 | 0.0558114 |
| -0.7553402 | 0.5886304 | 0.8261231 | 1.0269911 | 1.0757480 | 0.8518590 | -0.4876428 | -0.5876494 | -0.8438552 |
| 0.8573160 | -0.2545073 | -0.0466872 | -0.6181178 | -0.1757952 | 0.5533997 | -0.0218513 | 0.7947053 | 1.1063075 |
| -1.4304921 | -0.7833595 | -1.5917948 | -0.7965988 | -1.2501663 | -2.0689160 | -0.0921921 | -0.4765807 | -0.8587117 |
| -2.3914354 | -3.7262974 | -2.9415859 | -3.1875370 | -4.3786447 | -3.9634326 | -0.1331380 | -0.2295945 | -1.4348676 |
| -4.8480997 | -0.6359680 | -0.5618920 | -0.7427456 | -1.0065312 | -2.7043457 | -0.3045812 | -0.8400804 | -3.3264442 |
| -1.9288949 | -1.6031310 | -1.5516812 | -1.3630477 | -2.5991221 | -2.1433245 | -0.1994290 | -0.4778506 | -1.5515809 |
| -0.4345615 | -1.3963981 | -2.1568195 | -1.1920778 | -1.7002747 | -2.0466784 | -0.0295676 | -0.1028378 | -0.4798329 |
| -0.0470964 | -0.2284815 | 0.0835802 | 0.0928919 | 0.3033439 | 0.0355462 | -0.3454578 | -0.0434580 | -0.0611883 |
| -1.3279580 | -1.6741632 | -1.7805914 | -1.1192045 | -1.5200954 | -1.7981633 | -0.4059658 | -1.4418120 | -1.6259379 |
| -0.3032042 | 0.4798115 | 0.8825370 | 0.8153493 | 0.5931453 | 2.8954061 | -0.4392316 | -0.1499782 | -0.0734644 |
| -1.1861835 | -1.3649774 | -1.8263327 | -1.5479132 | -1.3641340 | -1.6122041 | -0.0965849 | -0.6048507 | -0.1309968 |
| 0.8583524 | 1.0014424 | 2.0807303 | 0.7088284 | 1.4158473 | 1.3229031 | -0.0560989 | 0.7093095 | 1.4848055 |
| -2.8692154 | -0.7528614 | -1.5888724 | -0.5219681 | -0.9827432 | -2.3101493 | -0.4612364 | -0.7911667 | -2.4951971 |
| -1.4578607 | -1.9773807 | -1.3155670 | -1.1566536 | -1.8172491 | -1.7494582 | -0.5484409 | -0.7726527 | -2.0126041 |
| -3.6196975 | -0.0213844 | 0.1567306 | 0.1300442 | -0.3630365 | 0.1073009 | -0.1395704 | -0.3242862 | -0.5899029 |
| -2.0179821 | -1.0047437 | -1.5834212 | -0.7009237 | -1.5406900 | -2.3652212 | -0.5848120 | -0.9617011 | -2.0976107 |
| -2.0254160 | 0.2621049 | -0.2186449 | -0.3477123 | 0.2301419 | -0.0749418 | 0.1729315 | -0.6927590 | -2.2751338 |
| -0.9468579 | -1.9196579 | -1.9238348 | -2.6302001 | -2.5223371 | -2.2376984 | -0.2002209 | -0.6977058 | -0.7904977 |
| 0.1936750 | -1.1041519 | -0.7648890 | -1.0726672 | -1.2515214 | -1.5515944 | 0.1157519 | -0.3535228 | 0.5906050 |
| 0.0002761 | 0.8081338 | 0.8877773 | 0.5368725 | 0.8989244 | 0.4699421 | -0.3334979 | -0.2450530 | -0.2314872 |
| -0.4396093 | -0.9318030 | -1.4403698 | -0.7258866 | -1.8799243 | -2.6152413 | -0.0882485 | -0.9239387 | -1.8146508 |
| 0.3495431 | -2.3237175 | -1.3606033 | -1.7187992 | -2.5092482 | -0.9894297 | -0.4255508 | -0.1013540 | 0.5486993 |
| -2.2530077 | -0.8312366 | -2.6389997 | -1.0605291 | -1.1631670 | -2.1325611 | -1.0343316 | -1.8433977 | -2.0288093 |
| -1.5568797 | -1.9589160 | -2.1088008 | -1.3797353 | -2.2655323 | -3.0950577 | -0.0723366 | -0.8335695 | -0.8884343 |
| -0.9218122 | 0.1631857 | 0.1346311 | 0.0167863 | 0.5424053 | -0.2407627 | -0.5936022 | -0.6095881 | -0.5998046 |
| -0.1126887 | -0.7427136 | -0.8954206 | -0.6397067 | -1.2677472 | -0.7948349 | 0.2500630 | 0.1794444 | -0.1637460 |
| -2.0319817 | -0.2059840 | -1.7361324 | -0.5604908 | -0.5109970 | -2.9270413 | -0.3661049 | -0.5326311 | -1.8275357 |
| -1.7504060 | -2.2279548 | -2.3143043 | -2.3200026 | -4.2312879 | -2.6692008 | -0.4633477 | -1.1643280 | -0.9875228 |
| -0.6082749 | -1.5788107 | -0.8852242 | -1.7840877 | -1.5530093 | -1.1751169 | -0.1920516 | -0.5417031 | -0.2354108 |
| -0.1558785 | -3.2399458 | -3.4405939 | -2.7638907 | -3.7418807 | -2.4994868 | -0.4032956 | -0.7630431 | -0.2337860 |
| -0.8835499 | -0.1529307 | -0.8116865 | -0.3776524 | -0.2595529 | -0.3361717 | -0.0474588 | -0.1779937 | -0.4865587 |
| -0.9374876 | -1.4508170 | -2.0559963 | -1.0881409 | -1.7830454 | -2.0794304 | -0.0132913 | -0.4462893 | -0.3311864 |
| 2.0201225 | -2.2919701 | -1.2307215 | -2.4923933 | -2.1155743 | -0.3398841 | -0.8217190 | 1.1819209 | 0.6310331 |
| -0.0438642 | -2.1958143 | -1.7209651 | -1.7178538 | -2.3891014 | -0.8729278 | -0.0962356 | 0.0374089 | -0.4512065 |
| -0.2672702 | -2.3915653 | -1.6967123 | -2.3325768 | -2.2240092 | -2.3331982 | -0.0237067 | -0.3818636 | -0.0216522 |
| -0.8600615 | -1.8272029 | -3.2939732 | -1.7056319 | -2.9601318 | -3.4005960 | -0.3699986 | -0.9325656 | -0.6807009 |
| -0.5424125 | -0.1873852 | 0.2036643 | -0.0016545 | 0.0405964 | 0.1856156 | -0.7251588 | -1.1987389 | -4.4917596 |
| -0.0362374 | -1.3509330 | -1.3235435 | -1.5198559 | -1.3823569 | -0.5928131 | -0.0860957 | 1.1365255 | 0.4982207 |
| -0.4186091 | -1.2273524 | -1.2995611 | -1.1180631 | -1.1010894 | -0.6667545 | -0.4630952 | 0.3706656 | -0.3480475 |
| 0.9149177 | -1.9196740 | -1.5706360 | -2.0574484 | -1.1931679 | -1.1534322 | -0.2509157 | 0.5782829 | 0.5266698 |
| -2.6653729 | -0.5194488 | -0.3310683 | -0.6348627 | -0.7796419 | -0.8095454 | -0.0714424 | -1.9891700 | -3.2742309 |
| -2.1502732 | -1.7902972 | -2.4691575 | -1.6443086 | -1.9586623 | -2.1943977 | -0.9537611 | -0.8785315 | -1.2131327 |

| | | | | | | | | |
|---|---|---|---|---|---|---|---|---|
| 0.7633613 | -2.7374148 | -2.5714991 | -2.6796403 | -2.9434219 | -1.8315866 | 0.4073582 | 0.3106708 | 1.1464400 |
| -1.6463072 | -1.7440884 | -1.6400570 | -1.0505397 | -1.3739639 | -1.5844211 | -0.9882258 | -1.5981251 | -1.7899753 |
| -0.4602464 | -1.2132889 | -1.4032813 | -1.2379526 | -1.4797780 | -1.2649034 | -0.7463384 | -0.1802704 | 0.4405973 |
| 3.0937606 | 0.0065019 | 0.4274894 | -0.2716487 | 0.4306351 | 1.1552159 | -0.1084499 | 0.9223516 | 1.4793408 |
| -1.7160712 | -1.2929574 | -1.8547556 | -1.7842065 | -1.5970799 | -1.2158467 | -0.8833663 | -1.3369713 | -1.8332175 |
| 1.2945070 | 2.2898533 | 1.4793950 | 1.5885528 | 2.1714755 | 1.2543612 | -0.1448570 | 1.3598064 | 0.8375082 |
| -1.0965739 | -1.5849904 | -1.1131911 | -1.4108513 | -2.3645818 | -1.3883862 | -0.5245781 | -0.6736624 | -0.9226509 |
| -1.4313869 | -0.9196897 | -0.8955404 | -1.1954229 | -1.5832533 | -2.3219854 | -0.6056977 | -0.5163209 | -0.4784927 |
| -1.9531966 | -1.6749958 | -1.9468725 | -0.9014970 | -1.6179524 | -1.9101975 | -0.5947894 | -1.2150861 | -1.5202173 |
| 0.3236419 | -1.2580993 | -1.2247919 | -1.0410754 | -1.2630147 | -0.2210717 | -0.6537717 | -0.6066680 | -0.0795033 |
| 0.7259675 | -1.2833964 | -0.6559057 | -1.1128101 | -1.1086518 | -1.0446581 | -0.2906656 | -0.0618766 | 0.9434194 |
| -1.3889064 | -1.5734395 | -1.0247819 | -1.1194405 | -1.6187996 | -2.1175858 | -1.0327704 | -1.7739380 | -1.3309268 |
| 0.0225332 | -1.3434953 | -2.8910454 | -0.7377740 | -1.8158380 | -2.8275946 | -0.2155291 | -0.6975307 | -0.9758744 |
| 0.5419198 | -1.4488510 | -0.6971965 | -1.7869431 | -1.0619867 | -1.3948850 | -0.0089935 | -0.4215339 | 0.4590296 |
| -0.5311838 | -0.4054374 | -0.0403992 | -0.5038122 | -0.3825242 | -0.1607395 | -0.0882081 | 0.0196166 | -0.2124982 |
| -0.5834438 | -1.5782176 | -1.5719783 | -1.3190069 | -1.2913583 | -2.4235528 | 0.3979656 | 0.0833339 | -1.4304045 |
| 0.0183373 | 1.6707633 | 1.6697124 | 1.7132339 | 1.5966844 | 1.6753045 | -0.3190687 | -0.2966677 | -0.3154476 |
| -1.5600368 | -1.2388068 | -1.6662228 | -0.8289781 | -2.1462147 | -3.1786930 | -0.7208617 | -0.8348141 | -2.2931786 |
| -0.9017932 | -0.8244436 | -0.9738238 | -0.4457368 | -0.8755657 | -0.4250728 | -0.6074511 | -0.5453648 | -0.2250543 |
| -1.3415499 | -3.5751282 | -3.2240781 | -3.5638613 | -3.8265869 | -3.4076852 | -0.5819801 | -1.2803226 | -1.3784157 |
| 0.1703090 | 0.5430547 | 0.7777889 | 0.4982949 | 1.5171793 | 1.6726746 | -0.4052638 | -0.3408089 | 0.5174796 |
| 0.4650725 | -1.0100889 | -1.2240804 | -1.6271722 | -1.4025024 | -1.5211810 | -0.6944286 | 0.0997059 | -0.0592875 |
| -1.4276659 | -0.9331288 | -2.0613263 | -1.0925230 | -1.7779749 | -3.2104616 | -0.4187653 | -1.3208802 | -1.6339222 |
| -1.3442814 | -1.6293077 | -1.8641372 | -1.1245962 | -1.3168724 | -1.4516122 | -0.4476847 | -0.3712816 | -0.3947937 |
| -1.4432539 | -0.3228039 | -0.8797213 | -0.5324173 | -0.8986227 | -1.4347345 | -0.3722162 | -0.8149069 | -1.1146215 |
| -0.5369204 | -1.0696618 | -1.3148470 | -1.1740000 | -1.4450949 | -1.7438428 | -0.0826504 | -0.2510444 | -1.0336471 |
| -0.7723058 | -1.0339101 | -2.1908551 | -2.1634243 | -2.7465867 | -2.6673269 | -0.4023604 | -0.5490101 | -1.3647422 |
| 0.3101389 | -1.7688687 | -1.5628574 | -1.7100847 | -2.5220785 | -1.5999066 | -0.5533076 | -1.0585489 | -0.5691878 |
| -2.0989948 | -1.2227898 | -1.4792882 | -1.0059637 | -1.1982075 | -1.0652858 | -0.6194155 | -0.9372750 | -1.8603790 |
| 1.4795993 | -0.7875785 | -0.4733300 | -0.5611789 | -0.4099872 | 0.0380658 | 0.0538227 | 0.8039484 | 1.1710031 |
| 3.8299873 | 1.5221976 | 2.6140219 | 0.0984225 | 1.9170115 | 3.9029155 | -0.0128519 | 1.9814873 | 3.7497706 |
| 0.4114425 | -1.5483511 | -1.8198038 | -0.9889718 | -1.1180407 | -1.4478754 | -0.7315273 | -0.5090912 | -0.5984966 |
| 0.0671320 | -0.6888996 | -0.3051621 | -0.7810899 | -0.8676085 | -0.5131578 | -0.2287473 | 0.0380275 | 0.1127978 |
| 0.4522117 | -1.1879615 | -0.7112372 | -1.2021105 | -1.7965188 | 0.2726957 | 0.0323464 | 0.3269780 | 0.3394425 |
| 0.9405743 | 1.2896863 | 1.7015129 | 0.9947324 | 1.4379524 | 1.1346434 | 0.3644114 | 0.8333173 | 0.7733120 |
| -0.7005874 | -1.8988619 | -2.0489931 | -2.0736675 | -2.1723688 | -3.0126422 | -0.2699723 | -0.7582744 | -0.3524917 |
| 1.7191641 | -0.9833735 | -1.1902885 | -1.2984403 | -1.0456437 | -1.0832955 | 0.3046739 | 0.9357405 | 1.3372538 |
| 1.0459750 | -2.2968362 | -2.1390697 | -2.6498922 | -2.6741222 | -0.9502952 | -0.2718345 | 0.215002 | 0.3124596 |
| 0.5254455 | -2.1643567 | -1.9393729 | -2.3610559 | -2.1735604 | -1.6783050 | -0.3645413 | -0.2547225 | -0.2786734 |
| -0.9368233 | -2.1591246 | -2.4261650 | -2.3051230 | -2.3491256 | -2.8532088 | 0.1676408 | -0.0074140 | -1.0577296 |
| -1.9271795 | -0.8060691 | -1.4792869 | -0.4873453 | -0.7190341 | -2.2783017 | -0.0141386 | -0.5672631 | -0.9342853 |
| 0.1589285 | -1.1018991 | -0.6473929 | -0.4049289 | -1.3806196 | -0.9556908 | -0.1983666 | 0.4314242 | 0.6632739 |
| 1.3743742 | -2.4660499 | -1.0960813 | -3.1471813 | -2.0403755 | -0.3644921 | -0.1715561 | 0.7765078 | 1.3115364 |
| 0.4913649 | -1.9625766 | -1.9899249 | -2.3681257 | -2.3009748 | -1.6244515 | -0.1734699 | 0.1675791 | 0.5685113 |
| -0.6671346 | -0.7941167 | -1.5743007 | -0.7048364 | -1.6324432 | -1.0647328 | -0.3703088 | -1.2540773 | -0.8597428 |
| 1.2003184 | -1.5580062 | -0.8477838 | -1.5222657 | -1.2652543 | -0.3774221 | -0.2510550 | 0.1664623 | 0.2450226 |
| -2.4176817 | -0.8695449 | -1.6639511 | -0.7887686 | -1.1604319 | -2.3763661 | -0.2842545 | -0.7346259 | -1.7923074 |
| -0.9966668 | -1.6683679 | -2.0421770 | -1.8498858 | -2.0970573 | -2.5707813 | -0.4835268 | -0.8183144 | -0.9840565 |
| -2.0352089 | 0.0521346 | -1.4897692 | 0.2369533 | -0.1436515 | -1.5746103 | 0.0358273 | -0.5658815 | -2.2635868 |
| -0.2330326 | -3.0201969 | -2.7993329 | -2.7425852 | -3.8118420 | -2.0034191 | -0.1168785 | -0.0819969 | -0.1127415 |

| | | | | | | | | |
|---|---|---|---|---|---|---|---|---|
| 0.8273108 | -3.572658 | -3.344542 | -3.538493 | -3.474114 | -3.217189 | -0.104016 | 0.3807680 | 1.1102634 |
| -1.205046 | -0.760179 | -0.357368 | -0.515920 | -0.301778 | -0.444255 | -0.933520 | -0.997467 | -1.881999 |
| -0.315471 | -1.452768 | -0.897346 | -2.219375 | -1.006342 | -1.174159 | -0.682963 | -0.170228 | -0.728942 |
| -3.361387 | -0.627172 | -1.989286 | -0.974164 | -2.001908 | -3.477937 | -0.522584 | -1.150498 | -2.771364 |
| -0.959347 | -1.983385 | -3.259857 | -1.425811 | -2.866134 | -2.526884 | -0.589153 | -0.945151 | -0.972309 |
| -0.091433 | -0.290681 | -0.748201 | -0.817441 | -0.512119 | -1.360177 | 0.0726820 | 0.1196126 | -1.052529 |
| 1.0595600 | 0.9664912 | 2.4735910 | 0.5765766 | 1.3407146 | 1.4192365 | -0.374097 | 1.2475319 | 1.1031899 |
| -0.660857 | -0.116643 | 0.6088589 | 0.6570289 | -0.111196 | 0.4553033 | -0.302357 | -0.769613 | -0.202759 |
| -1.231340 | -4.218812 | -4.189179 | -4.411501 | -4.087687 | -3.456574 | -0.159274 | -0.743787 | -1.307869 |
| -0.088874 | -1.230860 | -0.730805 | -0.456363 | -1.588408 | -1.116909 | -0.383087 | -1.056743 | -0.262800 |
| 0.2778507 | -1.687595 | -1.265791 | -1.575509 | -1.508580 | -0.971697 | -0.476687 | -0.170898 | -0.874569 |
| -0.738314 | -1.979224 | -2.223955 | -2.154437 | -1.413726 | -2.430655 | -1.065777 | -1.375878 | -1.138278 |
| -0.503060 | 0.5154813 | 0.8447723 | 0.3561013 | 0.4975419 | 0.5560338 | -0.063971 | -0.066131 | -0.474787 |
| -1.243756 | -1.142229 | -0.409683 | -1.539796 | -1.188665 | -1.833409 | -0.010538 | -0.287150 | -0.599168 |
| -2.546479 | -0.807456 | -2.257808 | -0.795700 | -1.535879 | -3.488866 | -0.208481 | -1.169748 | -2.609367 |
| -0.749721 | -0.818681 | -0.986915 | -0.412954 | -0.819504 | -2.830620 | -0.413406 | -0.819678 | -0.859378 |
| -0.846736 | -3.852996 | -4.218258 | -4.770765 | -4.284426 | -4.502255 | -0.042581 | -0.262225 | -1.548442 |
| -0.912848 | -1.932022 | -1.311185 | -1.545123 | -2.028387 | -1.655024 | -0.630128 | -1.431679 | -0.522127 |
| -1.570730 | -1.589098 | -2.668052 | -1.705411 | -3.127862 | -3.221091 | 0.0183847 | -1.075004 | -1.565516 |
| 0.3182670 | -1.915973 | -2.828541 | -1.966444 | -1.543182 | -2.583009 | 0.0854736 | 0.3282902 | -0.444892 |
| -1.056009 | -1.930761 | -2.113881 | -1.871079 | -2.003935 | -2.824603 | -0.649172 | -0.650282 | -1.277677 |
| -2.186651 | 0.0121482 | 0.9400925 | 0.0014141 | -0.060209 | -0.101944 | 0.8333144 | -0.322774 | -1.187187 |
| -3.383941 | -0.559452 | -2.734830 | -0.266724 | -1.023734 | -3.762314 | -0.608798 | -1.363774 | -3.849575 |
| 0.4984011 | -1.756777 | -1.440101 | -1.756488 | -2.442210 | -1.369573 | -0.125035 | -0.508416 | 0.4992815 |
| -1.229409 | -1.492483 | -1.628575 | -1.470447 | -1.698314 | -1.617536 | 0.1113680 | -0.468072 | -1.716906 |
| -0.452228 | -0.806643 | -1.011885 | -1.129947 | -2.090484 | -1.186809 | -0.713379 | -0.834629 | -0.515525 |
| -0.926785 | -0.862758 | -1.268841 | -0.675237 | -1.151137 | -1.251595 | -0.045960 | -0.333785 | -0.824623 |
| -0.480766 | -1.660588 | -2.178669 | -1.676815 | -1.883234 | -2.757481 | -0.571676 | -0.766941 | -0.911454 |
| 1.3558035 | -0.441885 | 0.1786494 | -0.838934 | -0.546134 | 0.2775268 | -0.078174 | 0.2009104 | 0.7758424 |
| 0.9795453 | -1.735716 | -1.550380 | -2.537509 | -1.988889 | -0.990169 | 0.0048436 | 0.3883277 | -0.216357 |
| -1.198855 | -0.229828 | -0.805435 | -0.484028 | -0.142585 | -1.277834 | -0.259089 | 0.2050250 | -1.089245 |
| -0.824665 | -2.490732 | -2.634298 | -2.192785 | -3.572470 | -2.612993 | -0.270625 | -0.763011 | -1.412647 |
| 2.1729207 | 0.9990008 | 1.5679500 | 0.7612254 | 1.2049635 | 1.5340574 | -0.482442 | -0.162125 | 0.5310528 |
| -0.950645 | -0.713498 | -1.169638 | -0.309900 | -0.942070 | -3.166571 | -0.353670 | -0.860593 | -1.369309 |
| -0.329656 | -0.564683 | -0.848843 | -0.379299 | -0.324869 | -1.927943 | -0.671736 | -0.187350 | -0.955976 |
| -1.360775 | -1.716498 | -2.067058 | -1.304102 | -2.571444 | -1.845304 | -0.361167 | -0.788845 | -1.541878 |
| -2.703229 | -1.108819 | -1.996877 | -0.398621 | -1.684601 | -2.507033 | -0.311236 | -1.726561 | -1.846930 |
| -0.310543 | -0.296802 | 0.0621004 | -0.136425 | -0.231607 | -0.035970 | -0.844356 | -1.062657 | -1.919614 |
| -0.003890 | 0.1906819 | 0.5955387 | -0.004989 | 0.6566856 | 0.2098526 | -0.768694 | 0.4436609 | -0.486845 |
| -1.475091 | -0.859010 | -0.974399 | -1.094126 | -1.282766 | -1.559904 | -0.465943 | -0.502750 | -1.547660 |
| 0.5812737 | -3.795802 | -3.414014 | -3.960454 | -3.803840 | -2.651366 | -0.212319 | 0.3931799 | 0.8877853 |
| -1.313420 | -3.152211 | -3.868420 | -1.986892 | -3.731699 | -2.934096 | -0.479374 | -1.300599 | -2.121424 |
| -1.144795 | -0.836187 | -0.635137 | -0.018272 | -0.905214 | -0.640269 | -1.070243 | -1.042730 | -0.821425 |
| -0.289154 | -1.224358 | -1.283256 | -1.123272 | -1.102021 | -2.158888 | -0.316054 | -0.322361 | -0.122443 |
| -0.277265 | -2.051182 | -2.033650 | -1.899454 | -2.236667 | -0.887526 | -0.402491 | -0.530188 | -0.356259 |
| -1.369382 | -2.872871 | -2.242401 | -2.889262 | -2.965309 | -1.633647 | -0.710835 | -1.428294 | -1.884151 |
| -0.256330 | -1.098627 | -1.014750 | -0.779730 | -0.909364 | -1.408918 | -0.223075 | -0.396461 | -0.717984 |
| -1.332821 | -1.766164 | -2.117985 | -2.076529 | -1.956187 | -2.802543 | -1.079460 | -0.699637 | -1.607008 |
| 1.2997061 | -1.839991 | -1.908722 | -1.770838 | -2.363243 | -1.837256 | -0.691739 | -0.447993 | 0.4841817 |
| -1.606340 | -1.451091 | -1.597337 | -1.206389 | -1.596743 | -2.298652 | -0.952120 | -1.158450 | -1.187450 |

| | | | | | | | | |
|---|---|---|---|---|---|---|---|---|
| 1.9931371 | -1.250598 | -1.536892 | -1.399156 | -1.519857 | -1.156113 | -0.729779 | -0.343073 | 0.4698615 |
| 0.6474507 | -1.452327 | -0.951335 | -1.045081 | -0.769299 | -0.807480 | -0.555650 | -0.248311 | 0.5155333 |
| -0.240852 | -1.438158 | -0.673225 | -1.369812 | -1.550249 | -0.788715 | -0.369882 | -0.626491 | -0.195259 |

| PG_586 | PG_587 | PG_588 | PG_589 | PG_590 | PG_591 | PG_592 | PG_593 | PG_594 |
|---|---|---|---|---|---|---|---|---|
| -0.3312139 | -1.0161853 | -0.8118977 | -0.5812351 | -0.4785618 | -0.9567542 | -0.3447567 | -0.3532697 | -0.5252049 |
| -0.5221835 | -0.3483535 | -0.9634898 | -0.8092130 | -1.0442545 | -1.8065315 | -1.0293271 | -1.0476138 | -1.7991177 |
| -0.8801736 | -1.3635520 | -2.5720985 | -1.6811418 | -1.7666495 | -3.3618840 | -1.2736852 | -1.7875328 | -3.2856776 |
| -0.0752358 | -0.4170801 | -0.6700940 | -0.0982211 | 0.2498582 | 0.2199673 | -0.2211935 | -0.0255817 | -0.1340879 |
| -0.1040518 | 0.2205649 | -0.1853279 | 0.0710014 | 0.2222114 | -0.8008900 | 0.1939146 | 0.3718074 | -0.7567075 |
| -1.4378626 | -1.5737563 | -2.6687560 | -0.4778325 | -0.6464010 | -1.8982458 | -0.6837549 | -0.9989726 | -2.4504747 |
| -0.4357813 | -1.4341242 | 0.0596590 | -0.3238421 | -1.1213008 | -0.2054292 | -0.3033657 | -1.7587708 | -0.4959295 |
| -0.8869448 | -1.0538682 | -2.8263566 | -0.3811805 | -0.2931201 | -1.8594548 | -0.3724550 | -0.3077873 | -2.3453481 |
| -0.3681989 | -0.2480846 | -0.2661190 | -0.4891231 | -0.5848800 | -0.6772018 | -0.4707897 | -0.3918281 | -0.4884142 |
| -1.0613708 | -1.5158477 | -2.1964214 | -0.5300969 | -0.9994387 | -0.8022764 | -0.6370907 | -1.3321108 | -0.7390518 |
| -0.2263336 | -0.4393509 | -1.2450860 | 0.0644978 | 0.0531666 | -0.2126397 | -0.0838348 | 0.0087556 | -0.4501087 |
| -0.1393297 | -0.8354715 | -0.3877712 | -0.1484768 | -0.1901399 | -0.5251603 | -0.2120761 | -0.5436076 | -0.7639837 |
| 0.0229653 | -0.9976860 | -2.0178154 | -0.3805102 | -0.5474302 | -3.1749437 | -0.3894001 | -1.8994426 | -3.4908424 |
| 0.2922699 | 5.1645831 | 4.1438897 | -1.2179026 | 0.4598837 | 3.6131391 | -1.2011758 | 2.3147810 | 3.4858606 |
| -0.3668581 | -0.4817235 | 0.4537402 | -0.0830394 | -0.0821857 | 1.2768222 | 0.3515584 | 0.3735906 | 1.4172404 |
| 1.0668000 | 2.5919104 | 2.4388984 | -0.2961137 | 2.1581150 | 3.3057190 | 0.2276296 | 3.1546666 | 3.4950090 |
| -1.7860851 | -3.0050059 | -1.4579992 | -0.8751714 | -2.4287342 | -1.7192777 | -0.9082912 | -2.7396751 | -0.8964522 |
| -1.2393855 | 0.1509288 | -0.1775037 | -0.2160919 | -1.6807334 | -1.6024087 | -0.7129294 | -3.0106320 | -1.0201553 |
| -1.4584776 | -1.4432608 | -2.0176659 | -0.8641330 | -1.6128457 | -1.8459964 | -1.4903588 | -2.0944966 | -2.1043231 |
| -0.9422159 | -0.4286921 | -1.4255751 | -0.8612217 | -2.0914134 | -2.3588507 | -1.9845519 | -2.2823409 | -2.5770063 |
| -0.1216815 | 0.3282871 | -0.0871565 | -0.3666248 | 0.5042912 | 0.5205316 | -0.2281163 | 0.8030675 | 0.4294668 |
| -0.3211654 | -0.4399938 | -0.4290687 | -0.1015596 | -0.9681878 | -2.0468967 | -0.1183352 | -1.5636209 | -1.6179391 |
| 0.0345888 | 1.1664926 | 0.8423789 | -0.4117973 | 0.5787072 | 1.1796654 | -0.3196460 | 0.8500004 | 1.1019170 |
| -0.7846073 | -2.0108187 | -1.8217764 | -0.2605640 | 0.0137619 | -2.0270512 | -0.4298435 | -1.2060534 | -2.7276989 |
| -0.7942957 | -1.5215179 | -2.5227016 | -0.0557223 | -0.7258100 | -2.6676731 | -0.3193540 | -1.6542079 | -2.9795637 |
| 0.2402246 | 1.1064142 | 0.6793820 | -0.6413518 | 0.4769067 | 0.7550920 | -0.3207208 | 0.4577364 | 0.5049599 |
| 0.1119675 | 0.2304002 | -0.0701177 | 0.2913159 | 0.3277066 | -0.0615149 | 0.2369239 | 0.2994145 | -0.0690274 |
| -1.3207942 | -2.3156873 | -3.2311235 | -1.0379890 | -0.5294340 | -1.5129377 | -0.9124522 | -0.7388339 | -1.5187641 |
| -0.0666491 | -1.0012199 | -1.940668 | -0.1662257 | -0.4290765 | -3.0460389 | -0.3070299 | -1.0074402 | -3.5642154 |
| -0.1302636 | -0.7054865 | 0.1434136 | 0.0220180 | 0.2611774 | -0.0848800 | -0.0463312 | 0.0216822 | 0.0735568 |
| -0.8294713 | -1.7785612 | -2.7531535 | -0.2510713 | -0.4432001 | -3.4350046 | -0.4707856 | -0.8580265 | -3.5621465 |
| -0.0040275 | -0.4805587 | -1.8855737 | -0.0066058 | -0.1015592 | -1.3964511 | 0.1308579 | -0.2123423 | -1.3396780 |
| -0.2568987 | 0.9287079 | 1.3441171 | -0.4773593 | -0.7829075 | 0.7593933 | -0.6253546 | -0.0577886 | 0.9564887 |
| -0.7443322 | -1.2467236 | -2.7339660 | -0.3294874 | -0.6629328 | -3.6202365 | -0.4295628 | -1.4905964 | -3.8006839 |
| -0.2060958 | -0.2603234 | -0.0899013 | -0.2025385 | -0.2169011 | -0.4297710 | -0.2432365 | -0.2269161 | -0.3477059 |
| -0.9582618 | -1.6099787 | -0.6892951 | -0.3979538 | -1.3970300 | -1.1694266 | -1.5416821 | -2.7097143 | -0.9282101 |
| -0.6950971 | -0.9965696 | -1.3102403 | 0.1818880 | -0.4123132 | -1.7930092 | 0.0315685 | -1.2345663 | -1.7564671 |
| 2.5036234 | 4.4110646 | 0.7588736 | -0.4536674 | 1.5540492 | -0.6891327 | -0.4728616 | 3.2853036 | -0.4509124 |
| -0.2974720 | 0.0454511 | 1.0578524 | -0.1335886 | -0.5884346 | 0.1436820 | -0.2392844 | -0.4127964 | 0.2823064 |
| -0.9118195 | 0.6370476 | 0.5363134 | -0.4459466 | -2.0082854 | -1.6426482 | -2.0995042 | -2.1818587 | -2.1220128 |
| -0.3654405 | 0.0028465 | -0.2049258 | 0.0391058 | 0.2124122 | 1.2407507 | 0.0784522 | 0.4061521 | 1.2564578 |
| -0.4623764 | -1.1011342 | -2.3165699 | -0.5302238 | -0.8251585 | -2.6790987 | -0.5144053 | -1.0748766 | -2.8098899 |
| 0.2390851 | 0.3384286 | -0.3226285 | -0.0612393 | -0.0233194 | -0.7958200 | -0.1002445 | -0.1423241 | -0.7558324 |
| -0.7775921 | -0.6016531 | -1.6743781 | -0.2376454 | -0.1216229 | -0.9949027 | -0.3111635 | -0.2852998 | -1.4012518 |
| -0.0270202 | 0.5618660 | 0.3366982 | -0.2323360 | 0.7560655 | 1.1613164 | 0.0929075 | 0.9175655 | 1.3406557 |
| -0.0698199 | 2.1459862 | 3.2409823 | 0.2300130 | 0.8413196 | 2.5553462 | 0.1431084 | 1.7432673 | 3.2365293 |
| -0.2790602 | 0.0686579 | 0.0458320 | 0.0319632 | -0.1178462 | -0.3311018 | -0.0544665 | -0.0850107 | -0.0854777 |

| | | | | | | | | |
|---|---|---|---|---|---|---|---|---|
| -0.6637004 | 0.2394143 | 0.4864527 | -0.2806252 | -0.1348620 | -0.0155417 | -0.0577308 | 0.2029635 | 0.0605549 |
| -0.3969026 | -0.8281708 | -1.3753872 | -0.3584104 | -0.2652913 | -1.8339869 | -0.4351651 | -0.7290240 | -2.3993895 |
| -0.6704598 | 0.4583826 | 0.2038544 | -0.3456341 | 0.2958757 | 0.4764235 | -0.2142640 | 0.6048430 | 0.6875345 |
| 0.2134816 | 1.7157023 | 1.9178490 | -1.0310944 | -0.4451448 | 2.5207391 | -1.0215630 | 0.5041015 | 1.4940529 |
| -1.7844832 | -0.6442449 | -1.9242962 | -0.1755868 | -2.2565106 | -3.4440148 | -1.2023621 | -2.5714706 | -2.6875907 |
| -0.0759647 | -0.4244275 | 0.1231170 | -0.2527501 | -0.4033941 | -0.1950432 | -0.4421642 | -0.2574283 | 0.0539716 |
| -0.3714414 | -1.0836690 | -1.1294945 | -0.0168422 | -0.2261934 | -0.6426212 | -0.5494105 | -0.8771936 | -0.9371551 |
| 0.2336961 | 1.3690731 | 2.3684595 | -0.6133488 | 0.9876849 | 2.3171866 | -0.3263829 | 2.2165521 | 2.0919980 |
| 2.5618578 | 2.9212606 | -1.3845600 | -0.8592005 | 2.6722364 | 0.2205601 | -0.6201826 | 4.1939674 | -0.2546285 |
| -0.7718486 | 0.0419738 | -0.6296943 | -0.5353985 | -0.1875433 | 0.6487032 | -0.8245314 | 0.0129954 | 0.3201452 |
| -1.1003500 | -1.2570951 | -1.1824156 | -0.7830018 | -0.9970800 | -1.7107540 | -1.0105820 | -1.3746842 | -1.6028298 |
| 1.1068068 | 3.1228047 | 2.6919130 | -0.0424967 | 2.8269890 | 3.7955716 | 0.5101662 | 3.9198537 | 3.9642265 |
| 1.2786663 | 2.4399784 | 1.0817085 | 0.4990304 | 1.3782478 | 1.3707601 | 0.5579548 | 1.8203896 | 1.2631077 |
| -0.5478152 | -1.0316180 | -0.7627567 | -0.4647954 | -0.4375713 | -1.0045802 | -0.4390257 | -0.5442428 | -0.7033911 |
| -1.7322621 | -1.8521512 | -3.6251843 | -0.7159552 | -0.9984028 | -0.9211142 | -0.6108470 | -1.1070727 | -1.3144446 |
| -0.8422674 | -0.5777534 | 0.3953248 | -0.3657881 | -0.7039168 | -0.9555435 | -0.5471241 | -1.1937737 | -0.3577438 |
| 0.1517647 | -1.1920991 | -2.0557388 | -0.1252690 | -0.2037268 | -2.4505956 | -0.1479014 | -0.8803682 | -2.3222890 |
| -0.5357854 | -0.9527360 | -0.5570179 | -0.2753287 | -0.5467773 | -1.0918063 | -0.4629247 | -0.7402956 | -1.3245689 |
| -0.7521501 | -1.6012887 | -0.5742880 | 0.0274973 | -0.5519044 | -1.0211499 | -0.2624345 | -0.8594400 | -0.9315739 |
| -0.2201422 | 0.1817085 | -0.4507311 | -0.1120123 | -0.2799503 | -0.1533697 | 0.0081713 | -0.3672616 | -0.1920689 |
| -0.1889568 | -0.1255474 | -1.0322674 | -0.0477771 | 0.2959125 | -1.1701470 | -0.2538470 | 0.1243431 | -1.7717442 |
| -0.5797750 | -1.3379936 | -0.8656410 | -0.5594808 | -0.8883202 | -1.1928277 | -0.7166364 | -1.4224796 | -1.8263200 |
| -0.5656493 | -0.1193459 | -0.9158680 | 0.0614340 | -0.2620902 | 0.0765776 | -0.0347772 | -0.3373691 | -0.1326970 |
| -0.0172880 | 0.2262082 | 0.1722746 | 0.5933857 | 0.7472683 | 1.0234601 | 0.6215278 | 0.9480744 | 0.7766044 |
| -0.690405 | -1.1422276 | -1.8118908 | -0.7942982 | -0.9394602 | -2.4129744 | -0.9522095 | -1.2909517 | -2.2663384 |
| -0.6337757 | -1.1125196 | -0.3669640 | -0.5482809 | -0.3901038 | -0.6935933 | -0.5740742 | -0.8764151 | -0.8841467 |
| -1.4218222 | -2.0331516 | -1.7675825 | -0.2116451 | -1.7408942 | -1.9490073 | -0.5476794 | -2.6168160 | -1.9325622 |
| 0.7697212 | 2.6630788 | 3.7467351 | 0.1195784 | 1.8609242 | 4.0507054 | 0.5078388 | 2.1513980 | 4.0745076 |
| 0.3065765 | 2.7687824 | 2.9005094 | -0.8304786 | -0.3520029 | 2.1426976 | -1.0606747 | 0.2935447 | 1.2149218 |
| -0.3900578 | -1.2064679 | -1.9109173 | -0.3180893 | -0.2037207 | -1.4995656 | -0.2226182 | -0.5528379 | -1.7622817 |
| -0.5282390 | -0.6040293 | 0.1270821 | 0.0227520 | -0.9420565 | -0.2943974 | -0.3300600 | -1.7873189 | -1.1707345 |
| -0.5066994 | -0.3643042 | -0.5348603 | -0.0819630 | 0.0527835 | -0.2453857 | -0.1325622 | 0.0913822 | -0.4237130 |
| -1.2861520 | -2.3041488 | -1.0386694 | -0.5274489 | -1.2092793 | -1.0941504 | -0.7341645 | -1.8098308 | -1.0301141 |
| -0.6471018 | -1.4698854 | -1.5985694 | -0.3626158 | -0.4003117 | -0.8526709 | -0.4380418 | -0.8350608 | -1.0019690 |
| 0.0566098 | 0.8565853 | 1.5611641 | -0.5837976 | 0.9820045 | 2.4953252 | -0.1045134 | 1.3465970 | 2.5035595 |
| 0.2673787 | -0.8969519 | -0.5727923 | 0.1381017 | -0.1267035 | -1.0633114 | -0.0341682 | -0.5202843 | -0.6387760 |
| -0.1814822 | -0.4174835 | -0.1001451 | -0.0761664 | -0.1898564 | -0.1157177 | -0.2438218 | -0.3425684 | -0.0974250 |
| -0.5806631 | -1.6386371 | -2.0657006 | 0.0454749 | -0.3556665 | -2.7109741 | -0.0712776 | -1.2621960 | -2.6180373 |
| -0.3936485 | -1.3755591 | -2.5188278 | 0.0865484 | -0.4451633 | -1.9538780 | -0.1907897 | -0.6331692 | -2.9982450 |
| -1.2344318 | -1.9835293 | -2.3003015 | -0.5396821 | -0.9622556 | -1.8214475 | -0.5259276 | -1.1336635 | -1.9541936 |
| -0.8178097 | -0.7611044 | -0.9417431 | -0.6401725 | -0.6049573 | -1.5441088 | -0.7520209 | -1.1117535 | -1.1141198 |
| -2.1623417 | -2.3385929 | -1.5600564 | -0.0261717 | -2.9372598 | -1.0715625 | -1.0277041 | -3.4998856 | -1.6514107 |
| 1.1621387 | 2.4898212 | 1.4744249 | 0.5292122 | 3.9384301 | 4.7345654 | 2.4102620 | 5.4138712 | 4.3062218 |
| -0.1430024 | -0.3990385 | -0.0738306 | -0.0949954 | 0.1171286 | 0.0928036 | -0.1889717 | -0.0537975 | 0.1875362 |
| -0.3645718 | -0.1684108 | -0.5437733 | 0.0229446 | -0.3073112 | -0.7512102 | -0.1238424 | -0.3107381 | -0.4367778 |
| -0.4357745 | -0.4867622 | -0.2178847 | -0.8222589 | -1.2945534 | -1.7219313 | -0.7352619 | -1.5945244 | -1.4991237 |
| 3.7179434 | 6.7485836 | 7.8231906 | -0.1232709 | 4.9094556 | 7.6417853 | 0.1017488 | 7.2607523 | 9.0981215 |
| 0.1043699 | 2.0754000 | 2.0249118 | 0.0583023 | 1.8708657 | 3.2044800 | 0.1701241 | 2.8404206 | 3.3638888 |
| -0.2160618 | -1.7508914 | -2.2151172 | -0.4875736 | -0.7134932 | -1.4024773 | -0.5996208 | -0.2381885 | -0.6780940 |
| -0.6144377 | -0.4900333 | -0.9780551 | -0.9272372 | -0.7502812 | -1.1085415 | -1.0411070 | -0.6666758 | -1.5881477 |

| | | | | | | | | |
|---|---|---|---|---|---|---|---|---|
| -0.597629 | -1.100160 | -0.307751 | 0.3476644 | 0.3471245 | -2.174650 | 0.2819981 | 0.2988173 | -2.118548 |
| -0.446103 | -0.538301 | 0.0226266 | -0.290335 | -0.079676 | -0.154640 | -0.261429 | -0.034160 | 0.0537207 |
| -0.839631 | -1.614744 | -1.072977 | -0.169052 | 0.0439733 | -0.170839 | -0.170957 | -0.525718 | -0.493253 |
| -0.300645 | -0.244609 | 0.4065590 | -0.088693 | 0.0925804 | -0.165224 | -0.019642 | 0.1166636 | 0.0203691 |
| -1.077347 | -1.995919 | -1.982857 | -0.528079 | -1.450542 | -1.803488 | -0.822277 | -2.281593 | -1.986575 |
| -0.251632 | -1.179239 | -1.521403 | -0.309144 | 0.0303637 | -1.245904 | -0.182287 | -0.198575 | -1.203885 |
| 0.3096193 | 0.0643525 | -0.453777 | -0.267612 | 0.0413573 | -0.503125 | -0.123821 | -0.111212 | -0.279951 |
| -0.073962 | -0.012712 | 0.1295335 | -0.163048 | 0.1207877 | -0.035866 | 0.0183950 | -0.194510 | -0.085071 |
| -0.536446 | -0.477695 | -0.444031 | -0.182310 | -0.284459 | -0.263603 | -0.360912 | -0.111011 | -0.490305 |
| -0.294502 | 0.8167535 | 0.9671623 | -0.089169 | 0.8706049 | 1.4377668 | 0.2156762 | 1.2825268 | 1.4434960 |
| -0.251626 | -0.629562 | -0.762666 | 0.3916438 | 0.1004711 | -1.553878 | 0.2093156 | -0.300269 | -1.334365 |
| 0.0957826 | -0.479030 | -1.535502 | 0.0643692 | -0.417693 | -1.335443 | -0.167349 | -1.537659 | -1.116181 |
| -0.382845 | -1.437426 | -2.796561 | -0.247831 | -0.294222 | -4.846279 | -0.374723 | -1.194237 | -5.264312 |
| -0.105621 | -0.502372 | -1.451174 | -0.170830 | -0.208780 | -1.683216 | -0.324837 | -0.869473 | -2.026492 |
| -0.190317 | -0.254384 | -0.399818 | -0.351299 | -0.317028 | -0.638581 | -0.284822 | -0.407769 | -1.122940 |
| -0.116701 | -0.325358 | -0.242101 | -0.052318 | 0.0888802 | -0.039221 | -0.000194 | -0.018577 | -0.018326 |
| -0.602997 | -1.564093 | -1.838621 | -0.769784 | -1.005548 | -1.686066 | -0.690615 | -1.518739 | -1.909431 |
| 0.2366890 | -0.289749 | 0.0749975 | -0.219458 | -0.359257 | -0.362041 | -0.132605 | -0.351835 | -0.044943 |
| -0.703699 | -0.696749 | -0.232209 | -0.421504 | -0.575259 | -0.446895 | -0.374975 | -0.777168 | -0.808292 |
| -0.475539 | 0.7143376 | 1.0695669 | 0.1462638 | 0.3855998 | 1.4156267 | 0.0784836 | 0.8576589 | 1.2911805 |
| -0.640728 | -1.294976 | -2.939363 | -0.265207 | -0.377274 | -1.659741 | -0.321284 | -0.450920 | -2.145874 |
| -0.726628 | -1.020221 | -2.075882 | -0.288063 | -0.220636 | -0.281545 | -0.375372 | -0.296690 | -0.760487 |
| 0.1439965 | -0.221241 | -0.373856 | -0.114402 | -0.378106 | -3.909858 | -0.165519 | -0.613669 | -3.904560 |
| -0.569357 | -0.879356 | -2.128955 | -0.428627 | -0.809885 | -1.309474 | -0.542929 | -1.015865 | -1.835494 |
| -0.621262 | -0.942606 | -1.613500 | -0.491501 | -0.826139 | -1.592974 | -0.666874 | -0.909942 | -1.384269 |
| -0.702716 | -1.024410 | -0.821466 | -0.015116 | -0.178298 | -0.871130 | -0.350148 | -0.796740 | -0.915685 |
| -0.237469 | -0.509483 | 0.3890852 | 0.0433493 | 0.1319614 | 0.6319846 | 0.0052016 | -0.065893 | 0.7195306 |
| -0.452824 | -0.233396 | -0.375773 | -1.038456 | -0.737777 | -0.278414 | -0.887804 | -0.388045 | -0.331894 |
| -0.304965 | -1.102313 | -1.703472 | 0.0072535 | -0.540343 | -1.480053 | -0.168068 | -2.002298 | -1.327122 |
| -0.369745 | 0.3359465 | 0.7936464 | -0.692204 | -0.965095 | 0.4129148 | -0.857472 | -0.510644 | 0.3872941 |
| -1.145747 | -2.316549 | -1.389756 | -1.132809 | -1.211509 | -1.806010 | -1.307734 | -1.695166 | -1.115677 |
| -0.464385 | -1.354743 | -0.978932 | -0.384522 | -0.360715 | -1.170480 | -0.540726 | -0.917213 | -1.146073 |
| -0.467101 | -0.270513 | -0.223806 | -0.841973 | -1.131071 | -0.515430 | -0.796274 | -0.544916 | -0.377080 |
| -0.299626 | -0.251032 | -0.178766 | 0.3127275 | 0.4436847 | -0.098817 | 0.3238249 | 0.2728201 | -0.422960 |
| -0.602541 | -0.840402 | -1.799795 | -0.218627 | -0.180521 | -1.937707 | -0.032738 | -0.284227 | -2.264033 |
| -0.838870 | -1.788955 | -1.208795 | -0.321598 | -0.349673 | -1.136913 | -0.379483 | -1.186484 | -1.216454 |
| -0.381186 | -1.184380 | -0.845410 | -0.126609 | -0.204510 | -0.146627 | -0.272333 | -0.625829 | -0.091119 |
| -0.460006 | -0.531158 | -0.172608 | -0.537030 | -0.603472 | 0.0330886 | -0.567796 | -0.633753 | -0.070031 |
| -0.527249 | -0.102790 | -0.295483 | -0.306725 | -0.283523 | -0.218724 | -0.416468 | -0.226170 | -0.370234 |
| 0.0871179 | -0.265915 | 0.3560985 | -0.700361 | -1.341432 | -1.627096 | -0.623339 | -1.731353 | -1.788019 |
| -0.299637 | 1.1017302 | 0.4153934 | -0.815143 | -0.564905 | 0.4827133 | -1.736521 | -0.633490 | -0.221395 |
| -0.132915 | 0.0943309 | -0.400944 | -0.021201 | 0.1047870 | -0.031266 | 0.0952670 | 0.0627161 | -0.185532 |
| -0.257380 | -0.442594 | -0.268905 | -0.087769 | 0.0513581 | 0.0191882 | -0.087142 | -0.338015 | -0.728309 |
| -0.535459 | -0.819346 | -0.483547 | -0.341331 | -0.356891 | -0.984813 | -0.401203 | -0.693042 | -0.778039 |
| -0.733093 | -1.606697 | -3.618456 | -0.737759 | -0.641184 | -0.643283 | -0.637451 | -0.672950 | -0.683979 |
| 0.4456344 | 1.2691170 | 0.4263866 | -0.422822 | 0.1550783 | 0.1560604 | -0.368487 | 0.3937778 | 0.2783784 |
| -0.570266 | 0.4887184 | -0.341084 | -0.456902 | -0.573250 | -1.204294 | -0.476226 | -0.170248 | -1.176922 |
| 0.1120520 | 0.5118978 | 0.6542306 | -0.271739 | 0.3838432 | 0.5246338 | -0.219887 | 0.9922495 | 0.7530133 |
| -0.399266 | -2.333562 | -2.937274 | 0.3006535 | -0.057309 | -5.463514 | 0.1803722 | -1.495622 | -5.570986 |
| -0.953472 | -1.253342 | -2.085354 | -0.965528 | -1.344467 | -2.223775 | -1.119345 | -1.262163 | -2.084919 |

| | | | | | | | | |
|---|---|---|---|---|---|---|---|---|
| -0.189754 | 0.4695111 | 1.2637114 | 0.2489235 | 0.3059750 | 0.7163209 | 0.2164837 | 0.2686517 | 0.8272216 |
| -0.916463 | -1.195908 | -1.615764 | -0.742955 | -1.187329 | -2.045385 | -1.205603 | -1.343116 | -1.924328 |
| -0.474813 | -0.168751 | 0.1883065 | -0.728287 | -0.494208 | -0.667313 | -0.603429 | -0.542784 | -0.414029 |
| 0.3468496 | 0.7624791 | 1.4904019 | -0.224169 | 0.6151977 | -0.232224 | -0.062798 | 1.4521510 | -0.076160 |
| -0.930968 | -1.373420 | -1.870614 | -0.530564 | -0.977002 | -1.260203 | -0.580695 | -1.456336 | -0.828813 |
| 0.1000938 | 1.8672139 | 0.5943192 | 0.0064135 | 0.7132445 | 0.6864668 | -0.352477 | 0.8598195 | 0.9717254 |
| -0.813655 | -1.111783 | -1.534770 | -0.500941 | -0.303563 | -0.804415 | -0.402846 | -0.465383 | -0.994663 |
| -0.927224 | -1.419872 | -0.599546 | -0.613355 | -0.935921 | -1.396133 | -0.994141 | -1.263744 | -1.799164 |
| -0.927771 | -1.934294 | -1.468990 | -0.358484 | -0.709037 | -1.686680 | -0.397997 | -1.138635 | -1.696828 |
| -1.146052 | -0.538603 | 0.1279663 | -0.608655 | -1.004116 | -0.426828 | -0.731679 | -0.973700 | -0.257938 |
| -0.068726 | 0.0824725 | 1.1583847 | -0.422779 | 0.0526510 | 0.8286254 | -0.160984 | 0.2054755 | 1.0984489 |
| -1.387108 | -1.734809 | -1.303210 | -0.811344 | -0.715009 | -1.171859 | -0.815766 | -1.388000 | -1.176814 |
| -0.470804 | -0.915465 | -0.681833 | -0.211162 | -0.777363 | -2.713640 | -0.236424 | -1.985765 | -2.305652 |
| -0.391605 | -0.404378 | 0.4853071 | -0.267865 | -0.114462 | -0.531659 | -0.281585 | -0.039778 | 0.1532350 |
| -0.025573 | -0.074168 | -0.218178 | -0.553928 | -0.387167 | -0.193197 | -0.621173 | -0.432631 | -0.198775 |
| 0.4667668 | 0.1920268 | -0.822064 | -0.462037 | -0.453977 | -1.122245 | -0.214583 | -0.160482 | -0.419274 |
| -0.268427 | 0.2666987 | -0.323821 | -0.719884 | -0.409050 | -0.697382 | -0.582723 | 0.0165940 | -0.365785 |
| -0.878357 | -1.927063 | -2.806150 | -0.649867 | -0.809136 | -1.993816 | -0.959411 | -1.255258 | -2.767204 |
| -0.845953 | -0.852728 | -0.054921 | -0.686919 | -0.945238 | -1.037265 | -1.093286 | -1.115927 | -0.954817 |
| -0.964891 | -1.607541 | -0.977016 | -0.224154 | -0.647301 | -1.500395 | -0.561069 | -1.543554 | -1.589988 |
| -0.023175 | -0.106693 | 1.0600848 | -0.286071 | -0.445765 | 0.0994934 | -0.363483 | -0.212657 | 0.6907738 |
| -0.601664 | 0.0040378 | -0.416857 | -0.212857 | -0.286602 | -0.451068 | -0.120349 | -0.310669 | -0.209762 |
| -0.692795 | -1.859309 | -1.873815 | -0.277604 | -0.584850 | -2.432075 | -0.248538 | -1.096011 | -2.079774 |
| -0.318509 | -0.729936 | -0.857623 | -0.526582 | -0.297147 | -0.769277 | -0.425597 | -0.310416 | -1.354640 |
| -0.513008 | -1.003479 | -1.425849 | -0.341999 | -0.899056 | -1.488701 | -0.556387 | -1.054829 | -1.465876 |
| -0.213301 | -0.360720 | -0.871681 | -0.271702 | -0.221248 | -0.540510 | -0.314734 | -0.269227 | -0.575785 |
| -0.555827 | -0.889812 | -1.688388 | -0.692225 | -0.871230 | -1.836226 | -0.663887 | -1.098692 | -1.971292 |
| -0.919980 | -1.133769 | 0.0294732 | -0.149463 | -0.764203 | -0.237672 | -0.637356 | -1.115084 | -0.382413 |
| -0.819641 | -1.350113 | -1.536181 | -0.377054 | -1.113365 | -1.736607 | -0.978602 | -1.216785 | -1.397860 |
| 0.3047659 | 1.1128263 | 0.9821854 | -0.020762 | 0.7246936 | 1.2983959 | 0.2254971 | 1.1243094 | 1.2548492 |
| 0.5585891 | 2.8011708 | 4.3980748 | -0.020979 | 0.9354632 | 2.8575253 | 0.2803699 | 1.6362809 | 3.3553642 |
| -0.992309 | -0.717842 | 0.1556091 | -0.818427 | -0.679103 | -0.308258 | -0.925305 | -1.069868 | -0.096364 |
| -0.217017 | -0.156221 | 0.0811090 | -0.186531 | -0.023050 | 0.2499896 | -0.262079 | -0.070628 | 0.1986344 |
| -0.035273 | -0.015944 | 0.2238990 | 0.2034133 | 0.4929126 | 0.6744774 | 0.2491875 | 0.5166500 | 0.5105278 |
| 0.2762282 | 0.8917663 | 0.6496545 | 0.2363534 | 0.9739574 | 1.4283348 | 0.4932343 | 1.1841407 | 1.6355452 |
| -0.571251 | -0.598781 | -0.668869 | -0.556052 | -0.430467 | -0.622301 | -0.519578 | -0.707318 | -0.507424 |
| 0.1416295 | 1.3757273 | 1.4538119 | 0.3298527 | 1.1103766 | 1.9371378 | 0.2615989 | 1.4317456 | 2.2847140 |
| 0.0174428 | 0.3017722 | 0.5178125 | -0.637480 | 0.0725172 | 0.2165724 | -0.294051 | 0.1831498 | 0.7709749 |
| -0.527079 | -0.392046 | 0.1864105 | -0.264837 | -0.054871 | 0.1580530 | -0.417572 | -0.145548 | 0.0990355 |
| 0.0826769 | -0.143193 | -1.222087 | -0.047320 | 0.1139571 | -0.136973 | -0.027788 | 0.0855918 | -0.408991 |
| -0.366695 | -0.422740 | -0.398557 | -0.308159 | -1.138903 | -1.848190 | -0.671589 | -1.622639 | -2.223448 |
| -0.268053 | 0.2905952 | 0.7496073 | -0.311042 | 0.4142668 | 0.6673222 | -0.158167 | 0.5210012 | 1.0396174 |
| -0.198405 | 0.5801942 | 1.4412054 | -0.025876 | 0.4828137 | 1.2328624 | -0.114125 | 0.1552614 | 0.9182523 |
| -0.347375 | 0.0964260 | 0.4014167 | -0.463681 | 0.3712311 | 0.3664134 | -0.554894 | 0.2988778 | 0.2609877 |
| -0.437731 | -1.650607 | -0.718937 | -0.362114 | -0.217807 | -1.029367 | -0.243546 | -0.872276 | -0.536223 |
| -0.132334 | 0.0038098 | 0.0728596 | -0.276186 | -0.001439 | 0.1577553 | -0.259378 | -0.018198 | 0.0258350 |
| -0.526634 | -0.695155 | -1.938179 | -0.189489 | -0.333365 | -1.906918 | -0.081041 | -0.527792 | -1.762704 |
| -0.640358 | -0.991748 | -1.029979 | -0.668367 | -1.338635 | -1.311597 | -0.700820 | -1.607814 | -1.379032 |
| -0.134471 | -1.025277 | -2.355325 | 0.1714242 | -0.210142 | -2.898271 | 0.1571381 | -0.564501 | -2.843601 |
| -0.251747 | -0.317231 | -0.169609 | -0.167830 | 0.1738295 | 0.0897092 | -0.158408 | 0.1764995 | -0.292604 |

| | | | | | | | | |
|---|---|---|---|---|---|---|---|---|
| -0.298261 | 0.5112839 | 1.3575997 | -0.054608 | 0.0530284 | 0.5458167 | -0.060281 | 0.1076255 | 0.7600675 |
| -1.024411 | -1.118766 | -1.741639 | -0.510435 | -0.384505 | -1.244422 | -0.433831 | -0.786291 | -1.803333 |
| -0.594105 | -0.171249 | -0.603073 | -0.539423 | 0.0001873 | -0.835290 | -0.499714 | -0.075128 | -0.883683 |
| -0.538203 | -1.454625 | -3.175863 | -0.855645 | -1.009850 | -2.542271 | -0.581622 | -1.440294 | -3.149325 |
| -0.972064 | -1.466754 | -1.275450 | -0.505442 | -0.429062 | -1.535458 | -0.527720 | -1.040079 | -1.494244 |
| -0.136996 | -0.282614 | -1.133968 | -0.346412 | 0.3199333 | -1.007150 | -0.059110 | 0.0473346 | -0.671098 |
| 0.2173100 | 1.3548075 | 0.2624813 | 0.0685814 | 1.5143673 | 2.4401087 | 0.4193599 | 1.7741508 | 1.7908188 |
| -0.271027 | -0.221336 | -0.470942 | -0.217496 | -0.362153 | -0.558705 | -0.246809 | -0.653191 | -0.402211 |
| -0.079796 | -0.627199 | -1.427667 | 0.2989478 | -0.134806 | -1.797893 | 0.2210745 | -0.727577 | -1.336484 |
| -0.790573 | -0.742853 | -0.371097 | -0.854339 | -1.118803 | -0.315375 | -0.879735 | -0.971983 | -0.546528 |
| -0.524369 | -0.912299 | -0.915222 | 0.0259051 | -1.095715 | 0.3515654 | -0.581436 | -0.636020 | -0.144019 |
| -1.406255 | -1.425682 | -0.631641 | -0.683509 | -1.244105 | -0.320200 | -0.825704 | -0.991158 | -0.591143 |
| 0.4165527 | 0.2432136 | 0.1308406 | 0.5335386 | 0.9023566 | -1.412112 | 0.6776130 | 0.6672883 | -1.263264 |
| -0.387244 | -0.632944 | -0.654726 | 0.2363124 | 0.3828668 | -0.381384 | 0.1007645 | 0.0149243 | -0.432556 |
| -0.542402 | -1.784972 | -2.468438 | -0.559061 | -0.603901 | -3.431324 | -0.466430 | -1.553862 | -3.759616 |
| -1.125753 | -0.571925 | -1.174580 | -0.191219 | -0.436475 | -0.628806 | -0.004237 | -0.240376 | -0.338307 |
| -0.130952 | -0.637433 | -1.698153 | 0.1940005 | 0.2614924 | -1.449521 | 0.1740945 | 0.1268482 | -1.685460 |
| -0.745011 | -0.942481 | -0.270335 | 0.0982485 | -0.235381 | -0.843643 | -0.047509 | -0.272576 | -0.596728 |
| -0.679262 | -1.391153 | -1.527628 | -0.497269 | -1.165503 | -1.885348 | -1.024972 | -2.257788 | -2.179212 |
| 0.0099565 | -0.041271 | -0.073894 | 0.2414896 | 0.2403192 | 0.3631911 | 0.1773484 | 0.2952499 | 0.1924102 |
| -0.660140 | -0.824789 | -1.319611 | -0.603363 | -1.264005 | -2.214271 | -0.916756 | -1.207953 | -2.738783 |
| 0.4262930 | -0.157273 | -0.717684 | -0.978552 | -0.991548 | -0.771389 | -0.991089 | -1.011917 | -0.819919 |
| -0.186160 | -1.775955 | -3.804820 | -0.477512 | -1.048750 | -3.759316 | -0.514260 | -1.718699 | -3.880388 |
| -0.486533 | -0.673031 | 0.4844645 | -0.102110 | -0.034389 | 0.6458459 | -0.066675 | -0.259293 | 0.6849822 |
| -0.542417 | -0.633795 | -1.468865 | -0.437707 | -0.391937 | -1.130461 | -0.550355 | -0.576442 | -1.129243 |
| -0.665700 | -0.995944 | -0.805310 | -1.264886 | -1.026722 | -0.561514 | -1.105104 | -0.662413 | -0.819374 |
| 0.0327348 | -0.601774 | -0.596122 | -0.047579 | -0.173643 | -1.007466 | -0.159471 | -0.383119 | -0.996842 |
| -0.485620 | -0.658970 | -0.885827 | -0.711094 | -0.670273 | -1.539072 | -0.537218 | -0.983314 | -1.215120 |
| -0.139366 | -0.219433 | 1.3465705 | -0.043265 | 0.3873957 | 0.7492114 | -0.032411 | 0.1286179 | 0.9353379 |
| -0.251897 | 0.5169787 | -0.283388 | 0.1758430 | 0.3445828 | 0.8114839 | 0.2202000 | 0.6429985 | 0.7112223 |
| -0.096445 | 0.1241461 | -1.390088 | -0.376324 | -0.094398 | -1.368036 | -0.298009 | -0.216024 | -0.988089 |
| -0.327739 | -0.965008 | -0.993905 | -0.187900 | -0.281220 | -0.487127 | -0.168758 | -0.433839 | -0.627117 |
| -0.614775 | -0.219694 | 0.1222640 | -0.557916 | -0.667184 | 2.0025647 | -0.520999 | -0.385007 | 0.7077495 |
| -0.335478 | -1.723439 | -1.350264 | -0.247972 | -0.297874 | -1.533036 | -0.204277 | -0.873820 | -1.453963 |
| -0.743542 | 0.1902419 | -0.503789 | -0.272201 | -0.722601 | -0.922278 | -0.272742 | -0.565748 | -0.996174 |
| -0.710766 | -1.268668 | -1.885599 | -0.591813 | -0.440414 | -1.269281 | -0.417109 | -0.803510 | -1.946537 |
| -0.575177 | -1.635419 | -2.014232 | -0.674694 | -1.463734 | -2.678143 | -0.721194 | -2.359137 | -2.711160 |
| -0.898847 | -0.953759 | -1.667625 | 0.5007892 | -0.241589 | -3.660910 | 0.4566191 | -0.779831 | -4.075747 |
| -1.033006 | 0.1637274 | -0.822759 | -0.192835 | 0.0384232 | -0.121909 | -0.617904 | 0.3831459 | -0.652535 |
| -0.529004 | -1.149101 | -1.870414 | -0.195254 | -0.325751 | -1.313620 | -0.228806 | -0.551999 | -2.206202 |
| -0.234543 | 0.1765096 | 0.7959743 | 0.2302780 | 0.2966076 | 0.0120490 | 0.0745867 | 0.4574989 | 0.1856205 |
| -0.688299 | -0.986178 | -2.150125 | -0.542260 | -0.762299 | -1.165686 | -0.549818 | -1.042730 | -1.634925 |
| -0.484035 | -0.668416 | -0.829158 | -0.678594 | -0.971352 | -1.037056 | -0.757858 | -0.971741 | -1.279680 |
| -0.522028 | -0.472595 | 0.1105328 | -0.379789 | -0.690764 | -1.066650 | -0.437680 | -0.832180 | -1.097122 |
| -0.338503 | -0.302420 | -0.292527 | -0.551619 | -0.366489 | -0.031182 | -0.348010 | -0.124396 | 0.2552225 |
| -0.955785 | -1.801484 | -2.188443 | -0.264099 | -0.218840 | -1.203182 | -0.359860 | -0.411386 | -1.657582 |
| -0.679444 | -1.057209 | -0.932225 | -0.262598 | -0.119367 | -0.912624 | -0.160114 | -0.378788 | -1.130116 |
| -1.203365 | -1.234993 | -1.512752 | -0.749923 | -1.985785 | -1.725570 | -1.936600 | -2.363273 | -2.001662 |
| -0.555988 | -0.535940 | 0.7921201 | -0.271817 | 0.4130230 | 0.3494255 | -0.015461 | -0.369752 | 0.8373701 |
| -0.857499 | -1.160259 | -1.250603 | -1.037540 | -1.074025 | -1.493190 | -0.914034 | -1.138394 | -1.608255 |

| | | | | | | | | |
|---|---|---|---|---|---|---|---|---|
| -0.0597547 | -0.3319491 | 1.229991 | -0.3589923 | -0.5615734 | 0.1308272 | -0.2715755 | -0.5175369 | 0.8160451 |
| -0.0736953 | -0.290570[illegible] | 0.7756469 | -0.5371269 | -0.3438393 | 0.5022169 | -0.6460929 | -0.0263692 | 0.5228701 |
| -0.8091657 | -0.882499[illegible] | -0.4800644 | -0.3638471 | -0.1548934 | 0.0824520 | -0.4499922 | -0.1903914 | -0.0926168 |

| PG_595 | PG_596 | PG_597 | PG_598 | PG_599 | PG_600 | PG_601 | PG_602 | PG_603 |
|---|---|---|---|---|---|---|---|---|
| -0.243355 | -0.247422 | -1.748965 | -0.089703 | -0.561265 | -0.295758 | -0.479155 | -1.573003 | -0.101367 |
| -0.517841 | -0.629376 | -1.054149 | -0.421356 | -0.328480 | -0.543868 | -0.473071 | -1.206814 | -0.450389 |
| 0.1530059 | 0.0494980 | 0.2020548 | 0.3704470 | 0.2925946 | -0.480127 | -0.595986 | 0.0179849 | -0.420788 |
| 0.1017064 | -0.044812 | -0.141543 | -0.282507 | -0.147612 | -0.040403 | -0.537063 | -0.292869 | -0.016539 |
| -0.021447 | -0.250101 | -0.683255 | -0.161602 | -0.191523 | 0.0114026 | -0.013239 | -0.596073 | 0.0425958 |
| -0.321845 | -0.279373 | -1.231669 | -0.441536 | -0.160890 | -0.384322 | -0.830637 | -1.512733 | -0.510399 |
| -0.379520 | -0.968735 | -1.431760 | -0.455351 | -1.500796 | -0.071422 | -0.667763 | -0.461369 | -0.196336 |
| 0.6347554 | 0.5024781 | -0.097982 | 0.4187769 | 0.5218437 | 0.0946460 | -0.189793 | -1.703381 | -0.039570 |
| -0.291989 | -0.481815 | -0.983177 | -0.269761 | -0.209297 | -0.396882 | -0.237331 | -0.193531 | -0.146913 |
| 0.0442357 | 0.0934640 | 0.4422197 | 0.0027164 | 0.0525373 | -0.391376 | -0.398699 | 0.4004485 | -0.446858 |
| 0.1303934 | 0.0904243 | -0.186777 | 0.0347542 | 0.0563630 | -0.188456 | -0.550778 | -0.494174 | -0.454732 |
| 0.1114818 | -0.061767 | -0.048008 | -0.150743 | -0.324167 | -0.112170 | -0.550101 | -0.138842 | -0.345337 |
| -0.567557 | -0.811906 | -1.635738 | -0.632181 | -0.938149 | -0.686538 | -1.070020 | -1.186320 | -0.480975 |
| 0.4542475 | 0.6923817 | 0.7389254 | 0.6084614 | 1.1394140 | -0.074210 | 4.2037015 | 4.5352535 | 2.4959349 |
| 0.0106232 | -0.306886 | 0.0712683 | 0.1858012 | 0.0960620 | -0.031943 | -0.359455 | 0.3433119 | 0.0022117 |
| -0.272041 | 2.2412899 | 1.2403163 | 0.2817694 | 2.5710621 | 0.1980802 | 1.7735098 | 3.5853629 | 1.2326413 |
| -0.720597 | -2.511574 | -3.234596 | -0.906606 | -3.185805 | -1.159027 | -2.507430 | -1.492651 | -1.451080 |
| 0.2228816 | -0.235247 | 0.1368467 | -0.163807 | -0.535528 | -0.615978 | -0.261645 | -0.099363 | -0.382370 |
| 0.1725453 | -0.492958 | 0.2626069 | -0.114006 | 0.3235628 | -0.214342 | 0.0829712 | 0.1617323 | -0.467118 |
| -1.032730 | -1.419860 | -1.712410 | -1.133186 | -2.644246 | -0.953051 | -1.311330 | -0.565080 | -1.064666 |
| -0.614498 | -0.008703 | -0.026083 | -0.739015 | 0.0534912 | -0.218987 | 0.1617669 | 0.2969804 | -0.248091 |
| -1.002757 | -0.480222 | -0.212790 | -0.505950 | -0.487496 | -0.435351 | -0.350661 | -0.260246 | -0.270119 |
| -0.359340 | 0.6141679 | 0.5673468 | 0.0193737 | 0.7655107 | 0.0970989 | 0.7693779 | 1.8060416 | 0.1395388 |
| -0.325728 | -0.445021 | -1.182921 | -0.480721 | -1.002652 | -0.204108 | -1.183001 | -1.767603 | -0.719952 |
| -0.436798 | -0.897662 | -0.801054 | -0.740968 | -0.752843 | -0.696516 | -1.347018 | -1.857761 | -1.235263 |
| -0.057540 | 0.2806093 | 0.6275973 | 0.1797705 | 0.4754039 | -0.024247 | 0.5215791 | 0.8293983 | 0.0188426 |
| 0.1545216 | 0.0844858 | -0.199022 | 0.0384027 | -0.013824 | 0.1025011 | 0.0583397 | -0.121811 | 0.1216564 |
| -0.857506 | -0.823352 | -2.336119 | -0.827639 | -0.983098 | -0.363241 | -1.237736 | -1.245980 | -0.399432 |
| -0.007493 | -0.739684 | -1.382388 | -0.191460 | -0.931677 | -0.590816 | -1.758960 | -0.383612 | -0.745281 |
| -0.252837 | -0.363922 | -0.715053 | -0.409096 | -0.596140 | 0.0087775 | -0.436153 | -0.261086 | -0.194422 |
| -0.107615 | -0.089803 | 0.1213120 | -0.164005 | 0.0924684 | 0.1195866 | -0.688417 | -1.648319 | -0.032394 |
| -0.180222 | 0.3974164 | -0.088727 | 0.7474951 | -0.394403 | -0.407240 | -0.212687 | -0.061783 | -0.400961 |
| -0.233582 | -0.529189 | -1.528356 | -0.347624 | -0.066496 | 0.0291315 | 0.3955263 | 0.7672865 | 0.2082353 |
| -0.149275 | -0.518812 | -1.894397 | -0.252944 | -1.084700 | -0.465911 | -0.906870 | -1.021748 | -0.789466 |
| -0.164175 | -0.163157 | 0.0218725 | -0.117964 | -0.104639 | -0.199171 | -0.070698 | -0.079741 | -0.222581 |
| -0.584193 | -0.826846 | -0.388050 | -1.288299 | -1.334964 | -0.565277 | -0.785952 | 0.1228483 | -0.564346 |
| -0.053452 | -0.612892 | -1.248677 | -0.464018 | -0.834462 | -0.324667 | -0.820023 | -1.448581 | -0.754751 |
| 0.0625548 | 1.8495536 | 0.7597581 | 1.1919284 | 2.5830351 | 1.5732722 | 4.5028362 | 1.9927776 | 4.0484712 |
| -0.326695 | -0.479576 | -1.320189 | -0.248738 | -0.437131 | 0.1838574 | -0.112205 | -0.354816 | 0.1144835 |
| -0.858086 | -1.027227 | -1.234071 | -1.391958 | -1.388513 | -0.722342 | -0.584301 | 1.1781569 | -0.297529 |
| 0.1723249 | 0.2960754 | -0.638094 | 0.3896399 | 0.7241515 | -0.006205 | -0.164857 | 0.5514879 | -0.029816 |
| -0.420009 | -0.931816 | -0.985298 | -0.725933 | -1.066586 | -0.578584 | -1.289988 | -2.023115 | -0.525721 |
| -0.313011 | -0.194808 | -0.336978 | -0.558955 | -0.180695 | -0.554154 | -0.692992 | -0.498889 | -0.531897 |
| 0.2349343 | -0.148593 | -0.501756 | -0.053131 | -0.343177 | 0.1168153 | -0.621732 | -1.406570 | -0.135230 |
| 0.0599875 | 0.3769633 | 0.8203050 | -0.023168 | 0.4555244 | -0.234284 | 0.6761731 | 0.8212724 | -0.090191 |
| -0.190584 | -0.263564 | 0.7248328 | -0.237446 | -0.240222 | -0.159251 | -0.329777 | 0.4407910 | -0.173712 |
| -0.232060 | -0.294764 | -0.302242 | -0.258548 | -0.065852 | -0.027280 | -0.067514 | -0.629715 | -0.128131 |

| | | | | | | | | |
|---|---|---|---|---|---|---|---|---|
| -0.020392 | 0.2225265 | 0.0412032 | -0.032527 | 0.5396239 | -0.329465 | -0.250888 | 0.1002505 | -0.270784 |
| -0.663397 | -0.668858 | -1.190291 | -0.574758 | -1.281677 | -0.514710 | -0.501644 | -1.996441 | -0.535826 |
| -0.158089 | -0.289253 | 0.4237018 | -0.190910 | -0.277872 | -0.282005 | 0.0058523 | -0.283193 | -0.365897 |
| -0.056817 | -0.017581 | 0.1126488 | -0.499586 | -0.135357 | -0.225426 | 0.2485874 | 1.1850760 | 0.1217141 |
| -0.206470 | -0.272404 | 0.1103741 | -0.201981 | -0.137066 | -0.402565 | -0.664389 | -0.148106 | -0.621231 |
| -0.877544 | -0.746728 | 0.1903238 | -1.476911 | -0.578033 | -0.310007 | -0.322643 | -0.049564 | -0.478218 |
| -0.727715 | -0.737847 | -1.428996 | -0.739490 | -0.858924 | -0.275333 | -0.796363 | -1.012629 | -0.436666 |
| -0.390226 | 0.9306577 | 2.2863984 | 0.0084298 | 1.6738593 | 0.0850705 | 1.8321237 | 2.7429656 | 1.1712361 |
| 0.0077042 | 0.9021378 | -0.493130 | 0.5982568 | 1.3260035 | 1.1984696 | 5.0219089 | 3.7333667 | 4.1826453 |
| -0.793746 | -0.382546 | -0.369103 | -0.790167 | -0.370041 | -0.359828 | -0.427451 | -0.352705 | -0.392593 |
| -0.861489 | -1.593897 | -2.108072 | -1.200062 | -1.846006 | -0.433102 | -1.020326 | -1.321157 | -0.646744 |
| 0.1672181 | 3.0759083 | 2.4842928 | 1.2978940 | 3.6292695 | 1.1349557 | 2.4733062 | 3.3812719 | 2.0652364 |
| -0.367475 | 2.5485252 | 2.4412008 | 0.4432511 | 2.8602682 | 0.4704812 | 1.6372406 | 2.6738656 | 1.3812410 |
| -0.659329 | -0.941936 | -1.210888 | -0.578563 | -1.139009 | -0.473511 | -0.691649 | -1.469814 | -0.563419 |
| -0.484003 | -1.412301 | -1.908670 | -0.625126 | -1.648516 | -0.976960 | -1.471325 | -2.582442 | -1.081124 |
| -1.111457 | -1.015270 | -2.087496 | -1.297006 | -1.635748 | -0.854068 | -1.515685 | -0.075283 | -0.600121 |
| -0.257015 | -0.501740 | -0.770095 | -0.468153 | -1.121138 | -0.136574 | -1.004578 | -1.600693 | -0.438097 |
| -0.307650 | -0.604735 | -0.707122 | -0.402030 | -1.004805 | -0.189268 | -0.512088 | -0.559682 | -0.367254 |
| -0.301199 | -0.547460 | -0.716414 | -0.281501 | -0.768595 | -0.000205 | -0.840282 | -1.129738 | -0.537825 |
| -0.193725 | -0.424904 | -0.886675 | -0.124553 | -0.187521 | -0.021328 | -0.085654 | -0.743027 | -0.018998 |
| 0.0513834 | 0.3070797 | 0.1541890 | 0.1392410 | -0.108663 | -0.167920 | 0.0764729 | -0.022223 | -0.228820 |
| -0.932700 | -1.278130 | -2.300525 | -0.966008 | -1.458320 | -0.578878 | -0.972825 | -1.140803 | -0.732918 |
| 0.2904099 | 0.0603723 | 0.4137995 | 0.4200556 | 0.3612007 | -0.030989 | 0.0298490 | -0.146381 | -0.018047 |
| 0.6066399 | 0.3648354 | 0.2454882 | 0.0101250 | 0.4957820 | 0.5618989 | 0.1983187 | 0.8486632 | 0.3784709 |
| -0.398967 | -0.757607 | -1.296921 | -0.472819 | -1.072256 | -0.285216 | -1.268478 | -2.029844 | -0.544808 |
| -0.058235 | -0.478375 | -0.351327 | -0.282379 | -0.360159 | 0.0498822 | -0.394672 | -0.578125 | -0.083499 |
| -0.189821 | -0.992163 | -1.187664 | -0.214392 | -0.765983 | -0.834293 | -1.797041 | -2.022421 | -1.614163 |
| -0.226916 | -0.328071 | 1.3398669 | -0.391113 | -0.255977 | 0.6873052 | 2.1293004 | 4.6604159 | 0.5856815 |
| -0.442275 | 0.7782864 | 2.5776488 | -0.131454 | 1.0653262 | -0.488483 | 1.0406743 | 1.7454095 | 0.3129503 |
| -0.331854 | -0.340662 | -0.392497 | -0.135597 | -0.527698 | -0.282751 | -0.826165 | -1.677227 | -0.293276 |
| -0.091249 | -0.746008 | -0.659127 | -0.378443 | -0.494953 | -0.177782 | -0.285277 | -0.223172 | -0.213983 |
| -0.302282 | -0.442067 | -0.229894 | -0.248287 | -0.484065 | 0.1491322 | -0.489485 | -0.492943 | -0.092264 |
| -0.686095 | -1.020512 | -1.202223 | -0.896904 | -1.436868 | -0.746830 | -1.282106 | -1.077643 | -1.054576 |
| -0.397696 | -0.535557 | -1.138424 | -0.672296 | -0.614573 | -0.265748 | -1.202723 | -1.439041 | -0.427239 |
| -0.440504 | 0.0182775 | 0.7459487 | -0.360611 | 0.1864771 | 0.0345683 | 0.3970887 | 1.2088026 | 0.0110978 |
| -0.015302 | -0.013189 | -0.005579 | 0.0373274 | -0.031313 | -0.348747 | -0.041911 | -0.056294 | -0.318467 |
| 0.0047373 | 0.0861180 | 0.2865550 | 0.2284866 | 0.0042816 | -0.112657 | 0.1183617 | -0.053671 | -0.081261 |
| -0.044779 | -0.138089 | -0.681852 | -0.006525 | -0.755714 | -0.170314 | -1.053075 | -1.762070 | -0.449599 |
| 0.3595885 | 0.3867267 | -0.238993 | 0.3346063 | 0.0709062 | -0.138175 | -1.225955 | -0.327661 | -0.348668 |
| -0.653443 | -0.971086 | -1.514042 | -0.681078 | -0.974089 | -0.664287 | -1.034520 | -1.996294 | -0.842761 |
| -0.687837 | -0.658083 | -1.608470 | -0.733623 | -1.081533 | -0.308889 | -1.047101 | -1.408946 | -0.592888 |
| -0.189255 | -0.360413 | -0.027429 | -0.242271 | -0.118635 | -0.251474 | -2.410176 | -1.714829 | -0.842035 |
| 0.7786131 | 3.2080022 | 2.9875947 | 1.9301092 | 4.0811857 | -0.378515 | 2.3901055 | 2.7694621 | 0.9432757 |
| -0.240153 | -0.232564 | -0.422520 | -0.208379 | -0.363669 | -0.015491 | 0.1256636 | -0.098073 | -0.001482 |
| -0.119092 | -0.281459 | -0.594950 | -0.097732 | -0.510049 | -0.051984 | 0.1078338 | -0.351391 | -0.079136 |
| -0.598485 | -0.668775 | -0.694149 | -0.760029 | -0.643882 | -1.086028 | -1.848293 | -1.251237 | -1.344122 |
| -0.002195 | 6.1685247 | 5.1560822 | 0.5516857 | 7.0723263 | -0.202666 | 0.5236534 | 6.7708519 | -0.025092 |
| -0.167136 | 1.5864019 | 0.9112518 | -0.039036 | 2.2233597 | 0.0820926 | 0.9723163 | 1.8502733 | 0.1621149 |
| 0.6505539 | 0.5498307 | 0.3680343 | 0.6567709 | 0.4847202 | -0.806232 | -0.967660 | -2.514688 | -1.361271 |
| -0.860241 | -0.545881 | 0.1494275 | -0.805798 | 0.0581721 | -0.272895 | 0.0785912 | 0.2061491 | -0.300043 |

| | | | | | | | | |
|---|---|---|---|---|---|---|---|---|
| -0.4210684 | -0.5309340 | -0.3531671 | -0.4278063 | -0.4642525 | -1.0661454 | -0.1472819 | -0.2303322 | -0.9133276 |
| -0.0333496 | 0.1042503 | 0.1704423 | 0.0968460 | 0.0612250 | -0.0869674 | 0.0399733 | 0.3356473 | -0.1862474 |
| -0.1554687 | -0.2294710 | -0.0230551 | -0.5477397 | -0.8494104 | -0.1295991 | -1.1243230 | -0.0158036 | -0.5910827 |
| 0.0633559 | -0.1065194 | -0.4703874 | 0.0189128 | -0.2083136 | 0.1197950 | -0.0087211 | -0.0126627 | 0.0366052 |
| -0.4842184 | -1.2172139 | -1.2750519 | -0.6601460 | -1.8784832 | -0.3370012 | -0.8095103 | -1.8274663 | -0.7266159 |
| -0.8013665 | -0.9686690 | -0.5104379 | -0.5958569 | -0.6526106 | -0.6027939 | -0.4217260 | 0.1701033 | -0.2663300 |
| -0.4834551 | -0.2629699 | -0.9682550 | -0.5275837 | -0.4752871 | -0.0836544 | 0.0943836 | 0.3905960 | -0.2066559 |
| 0.2381829 | -0.2147939 | -0.1741421 | 0.0317569 | -0.0919608 | -0.0443596 | 0.1199725 | -0.0824971 | -0.2547010 |
| -0.1329354 | 0.0961439 | -0.1563059 | 0.2361504 | 0.2667369 | -0.0650989 | 0.1406373 | -0.2836166 | 0.0439734 |
| 0.0293048 | 0.1274269 | 0.6199247 | 0.3706155 | 0.4663648 | 0.1751122 | 0.4058563 | 0.9106032 | 0.2976278 |
| 0.2345034 | 0.3842866 | 0.0619583 | 0.1679432 | 0.2986877 | -0.1603016 | -0.8903055 | -0.8031451 | -0.2855483 |
| -0.3281436 | -0.5057970 | -1.6497691 | -0.5831199 | -1.0604984 | -0.2279181 | -0.6232866 | -0.9954684 | -0.2701689 |
| 0.0207167 | -0.4889587 | -1.3844184 | -0.0933938 | -0.9215465 | -0.3216005 | -0.9622027 | -2.2192986 | -0.5384991 |
| -0.8729616 | -1.1471542 | -2.2421217 | -0.8642909 | -1.5954487 | -0.4292614 | -0.9140823 | -0.5099189 | -0.4710422 |
| 0.0400723 | -0.5066461 | -0.8356424 | -0.2100409 | -0.5225773 | -0.0988546 | -0.5970772 | -0.3014400 | -0.1520162 |
| 0.1703510 | -0.1206122 | -0.3661448 | 0.0066506 | -0.0042172 | -0.1102932 | 0.0518940 | 0.1077957 | -0.0642628 |
| 0.0536696 | -0.3578110 | 0.0549353 | 0.1837614 | -0.6819714 | -0.6040014 | -1.3503376 | -1.1527636 | -0.5465439 |
| -0.3550288 | -0.0865236 | 0.2845597 | -0.3078499 | -0.0565363 | -0.5295986 | -0.4222352 | -0.1326069 | -0.2392208 |
| -0.1620398 | -0.2283520 | -0.2376071 | -0.3910279 | -0.5276775 | -0.2580894 | -0.7798740 | -0.6826389 | -0.4509825 |
| -0.1988201 | -0.1588962 | 1.2533592 | -0.1151495 | 0.3705193 | 0.1210358 | 0.1034800 | 1.4419690 | 0.2279103 |
| -0.3133032 | -0.2198162 | -0.1891621 | -0.2455224 | -0.3387270 | -0.1981525 | -0.6058298 | -1.9587716 | -0.2477754 |
| -0.1639487 | -0.2638068 | 0.0294411 | -0.2991702 | -0.2532730 | -0.3398692 | -0.6719847 | -0.1875414 | -0.2636077 |
| -0.2699404 | -0.2153378 | 0.2010744 | -0.1492924 | 0.0141799 | -0.0689290 | -0.2633019 | -0.0966468 | -0.2590891 |
| -0.0729620 | -0.3743532 | -0.8647514 | -0.3467014 | -0.0776448 | -0.3261063 | -0.8172195 | -1.5705520 | -0.5104837 |
| -0.2640662 | -0.3172725 | -0.2171649 | -0.0591925 | -0.2152599 | -0.3667980 | -0.3379724 | -0.3504191 | -0.3937366 |
| -0.1856202 | -0.3487496 | 0.0152018 | -0.3428641 | -0.5034793 | 0.0525192 | -0.8636052 | -0.2820080 | -0.2879918 |
| 0.0865306 | -0.0560344 | -0.0944006 | 0.1109837 | -0.0907373 | 0.0314865 | -0.2012666 | 0.7542902 | -0.3070001 |
| 0.0329454 | -0.5291401 | -0.3877027 | -0.1012222 | -0.0389547 | -0.0824302 | -0.3741497 | -0.1490353 | -0.0641654 |
| -0.1957641 | -0.1836290 | 0.1314825 | -0.0800766 | 0.0568072 | -0.2162228 | -1.5566536 | -0.5815447 | -0.4696631 |
| -0.1484427 | -0.4325790 | -1.6911193 | -0.2887512 | -0.0354732 | -0.1685966 | -0.0450158 | 0.0386839 | -0.0421027 |
| -0.2909424 | -0.8733368 | -1.6742664 | -0.7534731 | -0.9956037 | -0.4562554 | -1.5303851 | -0.1598017 | -0.6120404 |
| -0.3467161 | -0.2076632 | -0.6476382 | -0.5927414 | -0.1083982 | -0.0883952 | -0.6387141 | -0.8113123 | -0.3109954 |
| -0.2404889 | -0.2053671 | -0.3096909 | -0.3888656 | 0.4602818 | -0.5398034 | -0.1923223 | -0.1435356 | -0.4006977 |
| 0.3996544 | 0.1237166 | -0.3584050 | 0.1596717 | 0.1192969 | 0.4227153 | -0.1094461 | -0.0718664 | 0.2437806 |
| -0.0938346 | -0.2312738 | -0.5868132 | 0.0098821 | -0.5609588 | -0.2752851 | -0.6017798 | -2.1179128 | -0.2621449 |
| -0.3999729 | -0.4613646 | -0.5481744 | -0.2853902 | -0.6658934 | -0.2928324 | -0.6787750 | -1.1042779 | -0.4140760 |
| -0.3361982 | -0.4266797 | -0.9125592 | -0.3960721 | -0.6019907 | -0.0016752 | -0.5702006 | -0.4733967 | -0.1216355 |
| -0.4303292 | -0.5009666 | -1.0981037 | -0.4451233 | -0.3626221 | 0.1580192 | -0.0371487 | -0.4841261 | 0.1597114 |
| -0.2500615 | -0.4616623 | -0.3080794 | -0.2017150 | -0.1897730 | -0.3413561 | -0.5655330 | -0.8356804 | -0.5771688 |
| 0.3260169 | -0.2936430 | -1.7931772 | -0.0538856 | 0.1081443 | -0.4590179 | -1.2351703 | -0.2518362 | -0.5421867 |
| -0.3529016 | -0.5468025 | -0.5035186 | -0.6526511 | -0.6067428 | 0.3505517 | 1.1372706 | 0.2638885 | 0.8647431 |
| -0.0615342 | -0.2411163 | -1.0033114 | -0.0785470 | -0.2028167 | 0.0735112 | 0.1285105 | -0.2743813 | 0.0605228 |
| -0.1109844 | -0.2831427 | -1.7152052 | -0.4683038 | -0.3312802 | 0.2423460 | -0.2890039 | -0.2122307 | -0.0027685 |
| -0.2335839 | -0.0346812 | -0.0348402 | -0.0451478 | 0.4312525 | -0.2044225 | -0.1472928 | 0.0642480 | -0.1528346 |
| -0.1526956 | -0.0996847 | 0.1706337 | -0.1934377 | 0.1371454 | -0.3039856 | -0.2472752 | -0.2232398 | -0.3623523 |
| -0.3036183 | -0.3837870 | -0.4731704 | -0.3719457 | -0.3100244 | -0.0374181 | 0.6655402 | 0.5528671 | 0.3677855 |
| -0.1800329 | -0.3956918 | -0.3772100 | -0.1095492 | -0.5886528 | -0.4681728 | -0.7204002 | -1.0156410 | -0.3858066 |
| -0.3332878 | -0.2533285 | -0.1135661 | -0.5927751 | -0.0159613 | -0.0286905 | -0.0206965 | 0.5852516 | 0.0451539 |
| 0.3732037 | -0.3359657 | -0.4286032 | 0.1575531 | -1.1596710 | -0.3884755 | -1.3610798 | -1.7828667 | -0.7498063 |
| -1.3220343 | -1.4482367 | -1.5399477 | -1.0585252 | -1.4911262 | -0.7596547 | -1.2025984 | -1.8062615 | -0.6667106 |

| | | | | | | | | |
|---|---|---|---|---|---|---|---|---|
| 0.0898706 | -0.0301985 | -0.5290513 | -0.2144200 | -0.0655250 | 0.6285482 | 0.3605780 | 0.6946695 | 0.4738221 |
| -0.8643910 | -1.2324278 | -0.6330826 | -1.0904202 | -1.4588028 | -0.7518587 | -1.0133437 | -1.5210738 | -0.9110096 |
| -0.4571463 | -0.3544304 | -0.7777908 | -0.4623695 | -0.4817954 | -0.6508612 | -0.4079840 | -0.1301907 | -0.3142826 |
| -0.2100657 | -0.3166002 | -0.2543927 | 0.0047856 | -0.0540001 | -0.6802048 | -0.1648566 | -0.3400158 | -0.4406301 |
| -0.4281914 | -0.5146728 | -0.6027147 | -0.3148939 | -0.6331023 | -0.1548955 | -1.0271523 | -1.3968555 | -0.5340835 |
| -0.6600605 | 0.0725202 | 0.4833402 | -0.5104323 | -0.0561360 | -0.3019607 | 0.9359677 | 1.1625013 | 0.1030949 |
| -0.6121900 | -0.8635449 | -1.0321772 | -0.5964516 | -0.8423030 | -0.0851300 | -0.2180936 | -0.6552060 | -0.2893714 |
| -0.6658935 | -0.4729573 | -1.0464567 | -0.6031377 | -0.6531682 | -0.5728517 | -1.1741461 | -1.1003311 | -0.8368441 |
| -0.0817688 | -0.8954052 | -1.3329710 | -0.4208488 | -1.0783566 | -0.4028739 | -1.1490887 | -1.2180496 | -0.6649187 |
| -0.3663954 | -0.6425834 | -0.7269330 | -0.6150753 | -0.7874704 | -0.2362328 | -0.4218291 | 0.3338847 | -0.1994780 |
| -0.4336283 | -0.3113929 | -0.5693422 | -0.5142405 | -0.2424808 | 0.0155760 | 0.0519204 | 0.2308903 | 0.1649496 |
| -0.3540891 | -0.7078356 | -0.5870608 | -0.5254586 | -0.6261285 | -0.3655193 | -0.8398305 | -0.2738787 | -0.5894156 |
| 0.5463205 | -0.2043554 | 0.1428611 | -0.0609298 | -0.4795359 | -0.4935593 | -0.3361294 | 1.4219588 | -0.5570674 |
| -0.5219710 | -0.3167538 | -1.5722435 | -0.3640227 | -0.1735418 | -0.4936289 | -0.5271763 | -0.7169923 | -0.5136108 |
| 0.0087050 | -0.0231725 | -0.5383577 | 0.0890398 | -0.2128019 | -0.4131292 | -0.4644843 | -0.4592175 | -0.4150361 |
| -0.0417905 | 0.0684942 | -0.1860807 | -0.0540362 | 0.0561928 | 0.3200710 | -0.0018887 | -1.6035660 | 0.3910068 |
| -0.3943191 | -0.8253312 | -0.8650915 | -0.2879409 | -0.5813950 | -0.6463223 | -0.3286055 | -0.3644369 | -0.2352546 |
| -0.2608835 | -0.6805926 | -0.7616873 | -0.4536466 | -1.0416805 | -0.8160688 | -1.8194082 | -2.3585994 | -0.7566617 |
| -0.4482255 | -0.7829920 | -0.1837526 | -1.0373354 | -0.7218160 | -0.4491744 | -1.1129476 | -0.4090241 | -0.6181233 |
| -0.6063746 | -0.7696113 | -1.0019803 | -0.9202882 | -1.0186609 | -0.1451534 | -0.7815054 | -0.7230199 | -0.5368250 |
| -0.2781937 | -0.1678152 | 0.9142435 | -0.2858427 | 0.0756606 | -0.3348691 | 0.0239562 | 0.3360428 | -0.1740798 |
| 0.1540028 | 0.3518704 | -0.2753881 | -0.0962593 | 0.3161202 | 0.1775400 | 0.3043707 | 0.2930065 | 0.2850308 |
| -0.5690865 | -1.4474864 | -1.0980136 | -0.9733610 | -1.9265830 | -0.6068443 | -1.8514106 | -1.2602975 | -0.6307352 |
| -0.0014262 | -0.2199947 | -0.6768374 | -0.0810312 | -0.1722330 | -0.3278915 | -0.6346404 | -0.7994993 | -0.2286265 |
| -0.1995425 | -0.3038724 | -0.7984104 | -0.2866740 | -0.4176386 | -0.2815167 | -0.9450196 | -1.2767600 | -0.3982019 |
| -0.2576223 | -0.7631081 | -0.6681100 | -0.3698987 | -1.2512195 | -0.3380389 | -1.3748844 | -0.4935717 | -0.4672791 |
| -0.7665827 | -1.1065436 | -1.0081403 | -0.6821090 | -1.2879717 | -0.5179864 | -1.0812990 | -0.8838333 | -0.6340647 |
| -0.1787195 | -0.6021825 | -0.5893274 | -0.3515136 | -0.5157236 | -0.1324321 | -0.5978391 | -0.4843054 | -0.2894587 |
| 0.0622933 | -0.4863070 | -0.6067041 | -0.3502755 | -0.7458765 | -0.3148694 | -0.7683302 | -0.9223461 | -0.6836794 |
| -0.2463205 | 0.1830770 | 0.2920290 | -0.0493905 | 0.4003225 | 0.1494209 | 0.2395523 | 1.0146454 | 0.4867664 |
| 0.0152774 | 0.4494116 | 1.3966287 | 0.5086373 | 1.0350858 | 0.1143963 | 2.1385032 | 3.3353155 | 1.0037775 |
| -0.6964806 | -1.4248027 | -1.3237650 | -0.8102770 | -1.4886430 | -0.4735110 | -0.8850253 | -0.7207363 | -0.4459597 |
| 0.0860826 | -0.3015151 | -0.6663079 | -0.2013531 | -0.3927619 | 0.0717131 | -0.1480022 | -0.3449519 | -0.0293093 |
| 0.2119434 | 0.3794611 | 0.5175535 | 0.2653175 | 0.4091673 | 0.1715426 | 0.3171943 | 0.8698420 | 0.1111926 |
| 0.3914210 | 0.6063825 | 0.4089758 | 0.4007553 | 0.7987944 | 0.3057965 | 0.7264032 | 1.0452334 | 0.2919709 |
| -0.3266860 | -0.4830287 | -1.2634685 | -0.5327214 | -0.2216369 | -0.2104598 | -0.6771903 | -0.8931951 | -0.2414421 |
| 0.0437011 | 0.6226363 | -0.2156914 | 0.5776559 | 0.1988065 | 0.6184091 | 0.2190463 | 1.6324712 | 0.6639181 |
| -0.4218446 | -0.1532101 | -0.7370104 | -0.4345967 | -0.0645557 | -0.2775087 | 0.2042928 | 0.2189920 | 0.0708957 |
| -0.1475956 | -0.5979162 | -0.9150414 | -0.4994889 | -0.5809221 | -0.1105518 | -0.0505925 | 0.3148604 | -0.0218722 |
| 0.4214748 | 0.0820243 | -0.2160563 | 0.1477366 | 0.1169362 | -0.3062418 | -0.3978052 | -0.0335076 | -0.2656608 |
| -0.1277469 | -0.2278162 | 0.0705862 | -0.2085290 | -0.0493824 | -1.0515162 | -3.3121363 | -1.3973457 | -1.3822979 |
| -0.7547060 | -0.6217816 | -0.5467189 | -0.7194247 | -0.1660840 | 0.0164552 | -0.2465992 | 0.7388898 | -0.2230805 |
| 0.3057743 | 0.8271920 | 1.2312302 | 0.4043886 | 1.0083946 | 0.2689416 | -0.4326478 | 0.5624667 | 0.0314873 |
| -0.2911210 | 0.2090956 | -0.1438120 | -0.4311553 | 0.4816130 | 0.1886784 | 0.0368719 | 0.7779952 | 0.1904132 |
| -0.2219319 | -0.4825063 | -0.4266653 | -0.2534851 | -0.7783836 | -0.0911803 | -0.5130012 | -0.2263895 | -0.2693791 |
| -0.0919594 | -0.3231163 | -0.2868636 | -0.0902167 | -0.0166773 | -0.0664471 | -0.2822853 | 0.2538482 | -0.1916655 |
| -0.1375274 | -0.7665458 | -0.6176501 | -0.2523225 | -1.1199938 | -0.5439967 | -1.0942973 | -1.7871243 | -0.8601292 |
| -0.5790599 | -1.1019406 | -1.1541983 | -0.5882880 | -1.2125255 | -0.5591821 | -0.7029682 | -1.1599188 | -0.6554389 |
| 0.0635770 | -0.1810513 | -0.2993263 | 0.0566504 | -0.6389743 | -0.7338618 | 0.3453110 | 0.2925844 | -0.5506368 |
| -0.0396290 | -0.1076031 | -0.0914187 | -0.1465319 | -0.1476656 | -0.1929089 | -0.1392399 | -0.4553383 | -0.4748832 |

| | | | | | | | | |
|---|---|---|---|---|---|---|---|---|
| -0.1235441 | -0.3295722 | -0.2559339 | -0.2519021 | -0.3093222 | 0.0567669 | -1.0142016 | 0.1968850 | 0.0387058 |
| -0.3686274 | -0.1598227 | -0.4995926 | -0.3091265 | -0.1617065 | -0.7829453 | -1.1541835 | -1.3153517 | -0.3943254 |
| -0.3304239 | -0.5698299 | -1.6528775 | -0.4499862 | -0.5614386 | -0.7238469 | -0.0750518 | -0.1611957 | -0.4729558 |
| 0.0082558 | -0.3877474 | -0.3842496 | -0.0290292 | -0.2269132 | -0.3933537 | -0.8464116 | -1.0739214 | -1.0263235 |
| -0.4835032 | -0.4393151 | -1.0733249 | -0.4631639 | -0.6799845 | -0.5274615 | -0.6977799 | -0.8923521 | -0.7061446 |
| -0.2368629 | -0.2630465 | -0.1645510 | -0.2357564 | -0.3426881 | -0.0151195 | -0.2246816 | -0.4631775 | -0.2195495 |
| -0.2603722 | 1.4895932 | 3.3795744 | 0.3395875 | 1.7950853 | 0.1581379 | 1.2770362 | 1.9495962 | 0.3717002 |
| -0.5718596 | -0.3649386 | -0.1739069 | 0.0652024 | -0.6175977 | -0.0820131 | -0.5289582 | -0.0969864 | -0.3165747 |
| -0.1439527 | -0.2795077 | -0.9615498 | 0.1393207 | -0.5793812 | -0.0213322 | -0.0481901 | -1.0914770 | -0.0039438 |
| -0.2353119 | -0.0595522 | -0.2756493 | 0.1172260 | 0.1172140 | -0.1870436 | -0.3603891 | 0.5222746 | -0.0931146 |
| -0.4099877 | -1.0115955 | -0.2164565 | -0.7383153 | -1.3908150 | -0.2275061 | -0.1314895 | -1.0390352 | -0.3511226 |
| -0.7896703 | -0.6770602 | -1.6391731 | -1.0010857 | -0.7308376 | -0.6162651 | -1.1881532 | -0.8684515 | -0.8241481 |
| 0.0566875 | 0.2671275 | -0.0339813 | -0.1576845 | -0.0560470 | -0.3859849 | -0.2752302 | -0.1565157 | -0.3881943 |
| 0.0162983 | -0.1880563 | -0.3020057 | 0.0578759 | -0.2893141 | 0.0091034 | -0.6071928 | -0.3363685 | -0.2083473 |
| -0.4896226 | -0.6366893 | -0.9915411 | -0.3851817 | -1.1967262 | -0.2304118 | -1.1545744 | -1.9928671 | -0.3962225 |
| -0.5275139 | -0.5900282 | -1.3772962 | -0.3610953 | -0.4806832 | -0.0138493 | -0.7468082 | -0.2994398 | -0.5708029 |
| 0.1743630 | -0.0391592 | -0.1221060 | 0.0408272 | -0.1426033 | 0.1425823 | -0.3382183 | -0.9085562 | -0.0567483 |
| -0.6641050 | -1.0140989 | -0.9700856 | -0.8519605 | -1.4298670 | -0.2207394 | -0.7405558 | -0.6122638 | -0.2916479 |
| -0.2001938 | -0.8824064 | -0.9555594 | -0.5538486 | -1.5248408 | 0.0593699 | -1.7155315 | -1.6043349 | -0.3757231 |
| 0.4463194 | 0.1345222 | -0.5810502 | 0.2830374 | 0.1991179 | 0.2587006 | 0.2228216 | 0.5964143 | 0.3866828 |
| -0.4095287 | -0.9090050 | -1.4933502 | -0.6238477 | -1.0369316 | -0.3305677 | -0.7221571 | -1.3553865 | -0.1792028 |
| -0.4833566 | -1.1430285 | -1.9878077 | -1.0551799 | -2.3076199 | -0.3715663 | -0.8572420 | -1.5185629 | -0.7058491 |
| -0.4149986 | -0.7493871 | -0.7156023 | -0.5341293 | -0.7445048 | -0.5310915 | -1.5376688 | -3.0863983 | -0.7317062 |
| -0.4939579 | -0.3441954 | -0.2845369 | -0.3613865 | -0.3055388 | -0.1423516 | -0.3670017 | 0.3832641 | -0.2445754 |
| -0.1538072 | -0.2700690 | -0.8369848 | -0.4616629 | -0.3287006 | 0.0269538 | -0.6544821 | -1.0836280 | 0.2203651 |
| -0.5309377 | -0.6698795 | -1.2586493 | -0.4465736 | -0.5127686 | -0.3923181 | -0.3206274 | -1.1112608 | -0.4672583 |
| -0.2081095 | -0.3197297 | -0.7618811 | -0.1082302 | -0.4412703 | -0.1159730 | -0.3898821 | -0.8369580 | -0.2231326 |
| -0.3881235 | -0.4114775 | -0.6222365 | -0.3489521 | -0.4638080 | -0.4563417 | -0.7538686 | -1.3101136 | -0.5731069 |
| -0.3716321 | -0.0193236 | -0.0987399 | -0.2698680 | 0.2505539 | 0.0194376 | -0.1334993 | 1.5059295 | -0.2030740 |
| 0.0015813 | -0.3542995 | 0.5102199 | -0.1181525 | -0.4340951 | -0.1035199 | -0.0384912 | 1.6643377 | -0.1339485 |
| -0.7521724 | -0.7414974 | -0.7510466 | -0.8797929 | -0.8778821 | -0.6386726 | -0.9078505 | -1.2200353 | -0.6054512 |
| -0.0440861 | -0.4569069 | -0.5407206 | -0.2073778 | -0.5962729 | -0.0723746 | -0.5063520 | -0.6942162 | -0.2041222 |
| -0.6000337 | -0.4395342 | 0.2474300 | -0.6289567 | -0.3009062 | -0.6851275 | -0.4533915 | 2.0170862 | -0.7011598 |
| -0.1410473 | -0.1633754 | -0.4512138 | -0.0547511 | -0.6631838 | -0.2359275 | -0.8546302 | -1.3303600 | -0.4483914 |
| -0.4241953 | -0.5744595 | -0.2825050 | -0.4909667 | -0.1510658 | -0.1294649 | 0.0493576 | -0.2638396 | -0.0493248 |
| -0.0655332 | -0.7186589 | 0.1957913 | -0.4341921 | -0.4269363 | -0.4705761 | -0.6767176 | -1.4943364 | -0.4989031 |
| -0.6052904 | -0.7656109 | -0.7491760 | -0.3168881 | -0.9908802 | -0.9381186 | -1.6463929 | -2.2916777 | -1.3203001 |
| -0.2855720 | -0.0395822 | -0.2284157 | 0.1084241 | 0.1049260 | -0.2350252 | -0.2594496 | -0.2059565 | 0.0712385 |
| -0.4946372 | 0.1337400 | 0.3314114 | -0.3431798 | 0.4548496 | -0.1356997 | 0.4148481 | 0.5851080 | -0.3605450 |
| 0.0514252 | -0.3920732 | 0.1111066 | -0.1038964 | -0.5258230 | -0.1056908 | -0.8321981 | -2.2826944 | -0.3218090 |
| 0.3291125 | 0.1689487 | 0.2074266 | 0.0848897 | 0.2095056 | 0.1090834 | -0.0321839 | 1.3702073 | 0.0102480 |
| -0.1627493 | -0.6621158 | -1.0210892 | -0.2265400 | -0.9883697 | -0.2272171 | -0.5046920 | -1.2901947 | -0.4032555 |
| -0.4796344 | -0.4935451 | -0.9167080 | -0.6302759 | -0.4085609 | -0.3287593 | -0.5645430 | -0.6908359 | -0.6312062 |
| 0.1671894 | -0.1471568 | -0.0936676 | -0.1528193 | 0.2576897 | -0.2758716 | -0.5154867 | 0.0336741 | -0.3747613 |
| -0.4010897 | -0.5768924 | -0.6473428 | -0.4334071 | -0.4416937 | -0.4728595 | -0.3901116 | -0.4592680 | -0.3012986 |
| -0.1185581 | -0.4365070 | -0.4327149 | -0.0181162 | -0.7226885 | -0.5260670 | -0.9492660 | -0.9076495 | -0.6882366 |
| 0.0724563 | -0.5209441 | -0.4230715 | -0.0897593 | -0.3512381 | 0.0433873 | -0.3780707 | -0.1914364 | -0.2214586 |
| -0.7681632 | -1.1346328 | -1.4445704 | -1.2961864 | -1.4150533 | -0.8912260 | -1.1002753 | -1.4896661 | -0.6016406 |
| -0.5634350 | -0.4822833 | -1.2288289 | -0.5752546 | -0.2320725 | 0.0875919 | -0.4244290 | 1.2006245 | 0.8414691 |
| -0.5531789 | -1.0112006 | -1.0471408 | -0.3873091 | -1.0142036 | -0.8899287 | -0.8814113 | -1.6166452 | -1.0073182 |

| | | | | | | | | |
|---|---|---|---|---|---|---|---|---|
| -0.4996797 | -0.5952031 | -0.409426 | -0.1593957 | -0.0910867 | -0.2265901 | -0.563823 | 1.2161575 | -0.1196135 |
| -1.0696352 | -0.5671499 | -0.0355291 | -0.7862083 | -1.082680 | -0.1985449 | -0.180096 | 0.3848116 | 0.1679552 |
| -0.2717262 | -0.2479321 | -0.1071534 | -0.371564 | -0.1570937 | -0.113775 | -0.0865853 | 0.1436352 | -0.1240487 |

| PG_604 | PG_605 | PG_606 | PG_607 | PG_608 | PG_609 | PG_610 | PG_611 | PG_612 |
|---|---|---|---|---|---|---|---|---|
| -0.4868527 | -1.6909207 | -0.1497102 | -1.4694019 | -2.3187513 | -0.6239089 | -2.2432781 | 0.0157867 | -0.2642093 |
| -0.3497247 | -1.1363167 | -0.5015184 | -0.8560575 | -1.5206214 | -0.6815359 | -1.4551808 | -0.6556623 | -0.7767939 |
| -0.3520634 | 0.2244450 | -0.8993270 | -1.2479780 | -1.8967839 | -0.5523980 | -2.0711680 | -0.0114220 | -0.7071113 |
| -0.4053890 | -0.3570534 | -0.0044350 | -0.1000580 | -0.0681211 | -0.1337088 | -0.6339713 | 0.1700594 | -0.0790418 |
| -0.1123862 | -0.7402193 | -0.3366462 | -0.3244852 | -0.6016318 | -0.2718081 | -0.3307170 | -0.3324228 | -0.4489567 |
| -0.4109159 | -1.4632512 | -0.2634770 | -0.9585395 | -1.7446698 | -0.6481551 | -1.8107069 | 0.0792414 | -0.2524771 |
| -0.3492665 | -0.0595598 | -0.1853608 | -1.3025955 | -0.0328654 | -0.4879130 | -0.3558319 | -0.1893279 | -0.7670114 |
| -0.4035791 | -1.6338934 | 0.0868637 | -0.5520178 | -2.2202338 | -0.0855538 | -2.1747240 | 0.4440697 | 0.2028721 |
| -0.0133264 | -0.0375133 | -0.6085764 | 0.1320135 | -0.3032780 | -0.5000969 | -0.3596503 | -0.3249490 | 0.0342566 |
| -0.2322571 | 0.4414427 | -0.4852162 | -0.6013254 | -0.3753109 | -0.5590050 | -0.3707445 | 0.3421421 | 0.0299392 |
| -0.8032930 | -0.8090110 | -0.1062579 | -0.3490741 | -0.6109291 | -0.1491062 | -0.9271348 | -0.1181952 | 0.0135854 |
| -0.5083052 | -0.1360150 | 0.0648158 | -0.3423350 | -0.3986851 | -0.0475399 | -0.5979162 | -0.1308921 | -0.2779940 |
| -0.7944898 | -1.0379980 | -0.0138764 | -0.8847714 | -2.3807070 | -0.2365032 | -2.0026377 | -0.4678092 | -0.8923233 |
| 4.8924328 | 4.5831022 | -0.2747841 | 3.1042589 | 3.8731634 | 1.8549969 | 3.8511509 | -0.3820392 | 1.8310549 |
| 0.1969126 | 0.4600687 | -0.4113739 | -1.1153777 | -0.3861269 | -0.3714478 | -0.5998433 | 0.1293142 | -0.6554869 |
| 2.4919835 | 3.4532791 | -0.2265512 | 2.7357853 | 3.7170232 | 0.8273933 | 3.8016016 | -0.5573528 | 2.8898368 |
| -2.8154361 | -1.6817281 | -1.8465227 | -2.9113643 | -2.9997115 | -2.5212419 | -2.7803537 | -0.9521784 | -1.9673152 |
| -0.5669362 | 0.0573981 | -0.2950735 | -0.5374512 | -0.7389980 | -0.1269033 | -0.5626605 | -1.2227395 | -1.5265190 |
| -0.2666368 | 0.2959878 | -0.5928510 | -0.5310233 | -0.9425412 | -0.3579158 | -0.7027028 | -0.3961707 | -0.3966030 |
| -1.0503609 | -0.3682813 | -0.6807554 | -0.8337392 | -0.6895685 | -0.8091225 | -0.6957162 | -0.5565413 | -0.5677547 |
| 0.1920325 | -0.0868323 | -0.0774847 | 1.4107746 | 1.3011890 | 0.4920628 | 1.1321698 | -0.4872028 | 0.0895668 |
| -0.3451277 | -0.3562182 | -0.2428680 | -0.2941542 | -0.3577768 | -0.3424573 | -0.2133701 | -0.3462394 | -0.7704131 |
| 0.7253739 | 1.5866796 | -0.2792522 | 0.9853781 | 0.8252917 | -0.0098023 | 0.5460172 | -0.4186500 | 0.7130133 |
| -1.6166019 | -1.6620226 | -0.3069359 | -1.2091511 | -2.2904281 | -0.4090969 | -3.0038279 | -0.4222229 | -0.8957860 |
| -1.4273719 | -2.1068800 | -0.3448332 | -1.0042350 | -1.4963770 | -0.7001673 | -1.4805905 | -0.2376680 | -1.0637985 |
| 0.3428317 | 0.3757619 | 0.2201583 | 1.3919742 | 1.0233340 | 0.5292165 | 0.7668480 | -0.5083740 | 0.3587300 |
| 0.3344676 | -0.0454110 | -0.1361717 | -0.4684882 | -0.5037682 | -0.1957769 | -0.5512030 | -0.0065218 | -0.1483681 |
| -1.1310474 | -1.3439717 | -1.1090365 | -1.0347123 | -1.8439309 | -1.0787850 | -2.0931288 | -0.8320339 | -0.9373385 |
| -1.5719545 | -0.0742963 | -0.6289463 | -0.6690133 | -0.7764630 | -0.6442460 | -1.0172821 | -0.4635672 | -1.0371992 |
| -0.3730884 | -0.2462299 | 0.1381400 | -0.2248030 | -0.1759894 | -0.0323799 | -0.3056927 | -0.1174923 | -0.2349721 |
| -0.7966959 | -1.1840742 | 0.8327088 | 0.0666531 | -1.4228640 | 0.8117079 | -1.2794037 | -0.3883714 | -1.1215475 |
| -0.2603925 | 0.1611074 | 0.5712110 | 0.0023157 | 0.1740760 | 0.0260904 | -0.0599432 | 0.1310254 | -0.4903209 |
| 1.1376081 | 0.9824595 | -0.3472297 | -0.6930078 | -1.2600797 | -0.2140497 | -0.6070950 | -0.0069923 | 0.1348557 |
| -1.1340222 | -0.9563226 | -0.3975834 | -0.7494061 | -2.8891210 | -0.6166713 | -2.8264370 | -1.0952828 | -0.9621834 |
| -0.1571708 | -0.1376390 | -0.2300070 | -0.1879728 | -0.2823632 | -0.2002222 | -0.2620541 | 0.1616011 | -0.2909218 |
| -0.6272993 | 0.3543774 | -0.2667000 | -0.2969503 | -0.7977718 | -0.3717420 | -0.9894864 | -0.3653753 | -0.3505595 |
| -0.8308864 | -1.1171984 | 0.2630669 | -0.3556213 | -1.3161513 | -0.2754801 | -1.5048064 | -0.4270162 | -0.6957670 |
| 4.2503753 | 1.1918888 | 0.8899158 | 3.1035941 | 0.3200684 | 3.3133035 | 0.1663445 | -0.5280038 | 3.1989759 |
| 0.0461505 | -0.4325754 | -0.7474362 | -0.9508643 | -1.3866240 | -0.7109367 | -1.1799790 | -0.0564224 | -0.3723118 |
| 0.6061820 | 1.7262295 | 0.5804702 | -1.4222189 | -2.3003630 | -0.4209160 | -2.2477168 | -1.2905054 | -1.7445642 |
| -0.0222837 | 0.2684617 | -0.0307090 | 1.2901178 | 1.4793187 | 0.0147216 | 1.0703174 | -0.4050334 | 0.0757385 |
| -1.3272420 | -2.2077063 | -0.3004357 | -1.3008260 | -2.3961503 | -0.4773665 | -2.0820172 | -0.9145063 | -1.2898939 |
| -0.6127090 | -0.5241020 | -0.0564409 | -0.3245843 | -1.7435573 | -0.0582278 | -1.6555549 | -0.4279970 | -0.5465798 |
| -0.3616350 | -1.3060877 | 0.1255907 | -0.0715628 | -0.0053747 | -0.0277078 | -0.0371875 | 0.0560411 | -0.0817372 |
| 0.3180296 | 0.8201173 | -0.5073464 | 0.9885008 | 1.1155272 | 0.0284214 | 0.8009042 | -0.1802915 | 0.4776260 |
| -0.3166015 | 1.0920621 | -0.5939273 | 0.9010374 | 6.3682493 | -0.5653322 | 7.2096790 | -0.5717957 | -0.4080601 |
| 0.0679343 | -0.6613957 | -0.1812891 | -0.1809710 | -0.3462242 | -0.2458208 | -0.3895631 | -0.4367944 | -0.1467128 |

| | | | | | | | | |
|---|---|---|---|---|---|---|---|---|
| 0.2179613 | -0.0553764 | -0.3968144 | 0.3171467 | 0.0306403 | -0.2718638 | -0.0640775 | -0.4406667 | -0.3288016 |
| -0.9259930 | -2.0492515 | -0.6460356 | -0.9723489 | -1.2847076 | -0.6553933 | -1.0790311 | -0.8810247 | -0.7692366 |
| -0.0004912 | -0.4400235 | -0.7613675 | 0.2089004 | 0.4418747 | -0.4852855 | 0.2838522 | -0.3693985 | -0.1099770 |
| 0.8217428 | 1.6438184 | -0.0760358 | -0.1814127 | 1.8690359 | -0.4450550 | 1.7839873 | -0.4337628 | 1.5361287 |
| -0.5652746 | -0.1505866 | -0.3597371 | -0.2820777 | -0.2692335 | -0.3402307 | -0.4010327 | -0.7195452 | -0.7392783 |
| -0.2454295 | 0.0201352 | -0.1111187 | -0.0868517 | 1.7850824 | 0.0483811 | 1.8981522 | -0.1181290 | -0.1754877 |
| -0.7747569 | -0.9790491 | -0.5056230 | -0.9555433 | -1.1151883 | -0.4453170 | -1.1221308 | -0.3359225 | -1.0087999 |
| 1.6059522 | 2.8377378 | 0.1445692 | 3.1189457 | 3.5405955 | 1.9737842 | 3.3447466 | -0.8600430 | 0.9897083 |
| 4.8131825 | 3.0141542 | -0.4104361 | 2.7732097 | 0.7897340 | 1.9470866 | 1.2383355 | -0.3520270 | 2.7638929 |
| -0.6071572 | -0.1783234 | -0.7161325 | -0.4731905 | -0.0429282 | -0.1500263 | -0.5090516 | -0.4616026 | -0.2990877 |
| -1.0926482 | -1.3524170 | -0.8932958 | -1.7764574 | -2.1433831 | -1.4322474 | -2.0686717 | -0.8655511 | -1.2236890 |
| 2.8575835 | 3.2803562 | 0.2046926 | 3.7294736 | 4.0182144 | 2.5631857 | 4.4664514 | -0.0646454 | 2.9271448 |
| 2.3679878 | 2.3445919 | 0.5100942 | 4.1233815 | 4.3006321 | 2.8633933 | 4.5840047 | -0.5302325 | 2.3150471 |
| -0.9676100 | -1.3330555 | -0.8195356 | -1.2861313 | -3.0596756 | -1.0749552 | -2.5898038 | -0.0827788 | -0.0924926 |
| -1.4566007 | -2.9583218 | -0.5909526 | -1.6913651 | -1.5548504 | -0.8101099 | -2.1001357 | -0.9931564 | -1.9954001 |
| -0.8498200 | -0.2411532 | -0.7859569 | -1.2868267 | -1.1495756 | -0.9629710 | -1.9463696 | -0.7842746 | -1.0447094 |
| -1.3007689 | -1.4546281 | 0.2633226 | -0.4815174 | -1.1554160 | 0.1164574 | -1.4043231 | -0.3238776 | -0.7504407 |
| -0.8425819 | -0.6241716 | 0.0065621 | -0.1114331 | 0.0442558 | 0.0156222 | -0.3039309 | -0.3416301 | -0.5691455 |
| -0.8339644 | -1.1741245 | -0.4356334 | -0.7176152 | -1.2291726 | -0.9904777 | -1.7443337 | -0.2905554 | -0.4869934 |
| 0.1299885 | -0.7064711 | -0.4034121 | -0.1446207 | -0.6503534 | -0.3462207 | -0.2899599 | 0.3383787 | -0.0357022 |
| -0.1464944 | -0.1366597 | -0.2042132 | -0.1485239 | -0.6955416 | -0.3345802 | -0.8814225 | -0.0835616 | -0.3070905 |
| -0.9293423 | -1.0916927 | -0.6771190 | -1.2861922 | -2.1743087 | -0.5958505 | -1.9013928 | -0.9733469 | -1.3452438 |
| -0.0929909 | -0.0592373 | -0.0461471 | 0.3016516 | 0.3290830 | 0.0005468 | 0.3885685 | 0.0240852 | -0.1670950 |
| 0.4535190 | 0.3471518 | 0.4325044 | 0.8889759 | 0.8680194 | 0.5669541 | 0.7300483 | -0.0046776 | 0.5613777 |
| -1.4986676 | -2.3466890 | -0.7370124 | -1.7913066 | -1.4797688 | -0.8225149 | -1.6813313 | -0.4989396 | -0.9658841 |
| -0.4709797 | -0.3558720 | -0.0760613 | -0.1663327 | 0.0940301 | 0.0461186 | 0.1195568 | -0.0257946 | -0.2758994 |
| -1.8598802 | -1.5534747 | -0.2184604 | -2.0294547 | -2.7908630 | -1.0365841 | -3.1999137 | -0.6826660 | -2.3494735 |
| 2.4475013 | 3.9159513 | 0.7196136 | 2.4901481 | 3.8099340 | 0.9755790 | 3.3639516 | 0.0460514 | 1.7857704 |
| 1.5899101 | 2.0488390 | 0.8404643 | 2.9231599 | 5.1192896 | 1.4033295 | 4.6852597 | -0.6220198 | 0.5707720 |
| -0.8366035 | -1.7732135 | -0.4841230 | -1.8912738 | -2.1809106 | -0.6790827 | -2.2312139 | 0.2854704 | 0.1210864 |
| -0.2063076 | 0.0726048 | -0.1285459 | 0.0415173 | -0.1364917 | -0.0294854 | 0.2228521 | -0.1031126 | -0.1404805 |
| -0.4701395 | -0.3812839 | 0.1160355 | -0.3298503 | -0.3318378 | -0.1001719 | -0.5246089 | -0.1753858 | -0.3405971 |
| -1.5555051 | -1.0275490 | -0.8675183 | -1.3894094 | -1.6661600 | -1.2510826 | -1.9012071 | -0.4397445 | -1.1431580 |
| -1.0835219 | -1.6619353 | -0.3342884 | -0.7692226 | -0.5690415 | -0.4907843 | -0.7776219 | -0.3729850 | -0.5863427 |
| 0.7005871 | 1.0202384 | -0.4244946 | 0.9036294 | 1.5134436 | -0.1542059 | 0.8463198 | -0.6129164 | 0.6731416 |
| -0.2041674 | 0.0640031 | -0.0290999 | -0.9534168 | -1.9509397 | -0.2357583 | -1.6212423 | 0.1712078 | -0.0737554 |
| 0.0284808 | -0.0265609 | -0.5116019 | -1.0138923 | -0.8423019 | -0.5981131 | -0.7321175 | 0.6326569 | 0.0153115 |
| -1.5294380 | -1.6487305 | 0.0740650 | -0.8127268 | -1.3716429 | -0.1888421 | -1.4269650 | -0.1533585 | -0.7445323 |
| -0.6340499 | -0.9110969 | 0.1561239 | -0.3512546 | -1.6344076 | -0.0213337 | -1.5624385 | -0.0138856 | -0.5157519 |
| -1.0439429 | -2.0670176 | -0.8253029 | -2.1753069 | -3.8046377 | -1.7206692 | -4.2467787 | -0.4784441 | -0.8399794 |
| -0.6714843 | -1.5244744 | -0.4228803 | -0.5389510 | -0.8696894 | -0.6226377 | -0.9740431 | 0.0078602 | -0.2687277 |
| -2.6270074 | -1.7963790 | -0.2901029 | -0.5885797 | -0.9790535 | -0.6359646 | -1.2614229 | 0.8087360 | 0.0789210 |
| 3.0047447 | 2.7104465 | -0.2603340 | 2.3198073 | 2.6578714 | 0.5264290 | 2.7452297 | 0.3611351 | 3.0302657 |
| -0.1048205 | -0.1113384 | -0.1399461 | -0.1972775 | -0.2142350 | -0.0984314 | -0.1919692 | -0.1092345 | -0.1014217 |
| 0.1984209 | -0.5628623 | -0.4567263 | -0.8211054 | -1.5738132 | -0.4169399 | -1.3407786 | -0.3943026 | -0.5349030 |
| -1.8028181 | -1.2917156 | -0.6740992 | -0.3595419 | -0.5845329 | -0.3742576 | -0.7371278 | -0.4079245 | -0.6750219 |
| 2.5955243 | 7.7121978 | -0.0795301 | 6.5911502 | 7.1993426 | 3.5007742 | 8.2631434 | 0.0608697 | 7.4517710 |
| 1.6290798 | 1.9312655 | -0.5543329 | 3.0910955 | 3.4734442 | 0.8821723 | 3.518005 | 0.0165876 | 1.9420310 |
| -1.3067827 | -2.4140417 | -0.7019948 | -0.6522819 | -2.7944183 | -0.5630621 | -2.0719258 | -0.5810065 | -1.2079588 |
| 0.0648160 | 0.1543561 | -0.2812891 | -0.4747289 | -0.3615647 | -0.6650905 | -0.5983832 | 0.0755188 | 0.2182111 |

| | | | | | | | | |
|---|---|---|---|---|---|---|---|---|
| -0.6830504 | 1.9082652 | -0.6546214 | -0.7291679 | -0.8211652 | -0.5967696 | -0.9158840 | 0.2258425 | -0.7506682 |
| -0.0399948 | 0.2489063 | -0.4227946 | -0.4884100 | 0.0049763 | -0.3895345 | -0.0625279 | -0.3383021 | -0.1758519 |
| -1.4469061 | -0.5011825 | -0.0948882 | -0.3771502 | 0.2859377 | -0.3295012 | -0.0474901 | -0.2448581 | -0.3311435 |
| 0.0567536 | 0.1547062 | -0.1376828 | 0.0006037 | 0.1613911 | -0.1276042 | 0.2069504 | -0.1680635 | -0.2241948 |
| -1.0603260 | -1.8732599 | -0.4682016 | -1.5642575 | -1.9571076 | -1.0485639 | -2.1849414 | -0.3789802 | -1.1377261 |
| -0.1003295 | 0.7160881 | -0.6719003 | -1.4818650 | -2.0144448 | -0.6417388 | -1.7196997 | -0.0753240 | -0.4342110 |
| 0.0953868 | 0.1517007 | -0.0976871 | 0.2158449 | 0.0355142 | -0.0260544 | 0.2654456 | -0.1178283 | 0.1807012 |
| -0.2243322 | -0.0591091 | -0.1461350 | 0.0167094 | 0.3829738 | 0.0543200 | 0.7339478 | -0.1880099 | -0.1367819 |
| -0.3884598 | -0.5832090 | -0.2368509 | -0.4442187 | -0.0370486 | -0.2292255 | -0.0237069 | -0.4921029 | -0.3677246 |
| 0.6273551 | 0.6759322 | -0.1000399 | 1.1142928 | 0.9549424 | 0.4410039 | 0.6418308 | -0.4332686 | 0.4991808 |
| -0.8695181 | -0.6256705 | -0.3879425 | -0.8734997 | -1.8136273 | -0.4643817 | -1.5272432 | -0.0860015 | -0.4460379 |
| -0.2899720 | -1.2512058 | 0.0047700 | -0.1281856 | -0.7531064 | -0.0909106 | -0.3364114 | -1.4548741 | -1.3212293 |
| -0.9841389 | -1.6721941 | -0.3587945 | -1.1398821 | -2.7319699 | -0.6575077 | -2.4261890 | -0.6707228 | -1.4101121 |
| -0.7730884 | -0.7030599 | -0.0657948 | -0.0655252 | -0.4093179 | 0.1296545 | 0.0405666 | -0.4130800 | -0.6394408 |
| -0.4917322 | -0.1088236 | 0.1382719 | -0.3163934 | -0.6314743 | -0.0248511 | -0.7455836 | -0.7724289 | -0.8868422 |
| -0.3773221 | -0.0195143 | -0.0629154 | -0.1301367 | -0.2081098 | -0.2641727 | -0.3596840 | -0.0492379 | 0.0753934 |
| -1.1624403 | -1.3481884 | -0.5023496 | -0.7503117 | -0.6754125 | -0.7473774 | -1.0286539 | 0.0638192 | -0.6802051 |
| -0.3779104 | -0.0152857 | -0.0881112 | -0.2317300 | -0.3509733 | -0.2923315 | -0.1770158 | -0.2499201 | -0.1592685 |
| -0.2787937 | -0.9723899 | -0.0219693 | -0.5051191 | -0.5992702 | -0.3102171 | -0.3721331 | -0.2987664 | -0.5568908 |
| 0.6961379 | 1.3907638 | 0.0377497 | 0.1800030 | 0.8872379 | 0.0945951 | 0.8587604 | -0.5775334 | -0.1705678 |
| -0.7889984 | -1.9231176 | -0.2611329 | -0.7246703 | -1.0603677 | -0.4285314 | -0.9752864 | -0.3417178 | -0.4573069 |
| -0.5604960 | -0.2015790 | -0.5609737 | -0.9306307 | -0.3711925 | -0.5160834 | -0.6631142 | -0.5826606 | -0.8678021 |
| -0.2180423 | 0.1624677 | -0.2078160 | -0.2543491 | -0.2049149 | -0.1586421 | 0.0084382 | -0.2430919 | -0.2326529 |
| -0.8363021 | -1.6004010 | -0.3414125 | -0.6975633 | -1.1463880 | -0.3191042 | -1.0378263 | -0.5293008 | -0.8763562 |
| -0.3932571 | -0.3193043 | -0.3752325 | -0.3097670 | -0.5399235 | -0.1720433 | -0.3615814 | 0.6156635 | -0.7935089 |
| -0.7928775 | -0.4868304 | 0.0478072 | -0.1721592 | -0.0689604 | -0.2063188 | -0.2026499 | -0.6113432 | -0.5736226 |
| -0.3049658 | 0.4473202 | -0.4003490 | -0.3680898 | 0.2657362 | -0.4521251 | -0.4443070 | -0.0176303 | -0.0641134 |
| -0.1232407 | 0.0903144 | -0.3994575 | -0.2602533 | -0.1660932 | -0.3932108 | -0.1100428 | -0.3122092 | -0.1502452 |
| -1.4521618 | -0.5842589 | -0.6037041 | -0.3538141 | -0.7146427 | -0.4741580 | -0.7036193 | -0.0021794 | -0.9922629 |
| 0.3800738 | 0.1135184 | -0.4505154 | -0.7219186 | 0.0063050 | -0.3352107 | 0.1990246 | -0.0604278 | -0.2689130 |
| -0.8114622 | -0.4189929 | -0.6817645 | -1.1853104 | -1.9913523 | -0.8561595 | -1.6208128 | -0.3432913 | -0.8068940 |
| -0.5649397 | -1.1141415 | -0.1399127 | -0.5948616 | -1.542974 | -0.4157676 | -1.4395422 | -0.3473039 | -0.5712575 |
| -0.2465408 | -0.3012953 | -0.9084681 | -0.5483203 | -0.1476087 | -0.3414767 | 0.2048670 | -0.7245471 | -0.5272858 |
| -0.0808078 | -0.1172901 | 0.1456206 | 0.1647332 | -0.0116208 | -0.1475039 | -0.2703208 | 0.1343579 | 0.2275657 |
| -0.9357403 | -2.1746601 | -0.1616358 | -0.5893538 | -1.6209773 | -0.5248945 | -1.7109765 | -0.0464085 | -0.2086824 |
| -0.9384694 | -1.1434704 | -0.3429109 | -0.6204596 | -0.8102715 | -0.6496249 | -1.2287175 | -0.5569022 | -0.6346943 |
| -0.4986735 | -0.4148311 | -0.2773556 | -0.5254804 | -0.6180206 | -0.2319302 | -0.8222978 | -0.5829335 | -0.8347157 |
| 0.1332197 | -0.3818199 | -0.3682249 | -0.8212133 | -0.7767060 | -0.3584638 | -0.6249783 | -0.0487421 | -0.2141855 |
| -0.9098500 | -0.7414517 | -0.6944117 | 0.2206070 | -0.7821006 | -0.7253303 | -1.0842006 | -0.6091407 | -0.6510062 |
| -0.5520602 | 0.1417632 | -0.6115905 | -1.5547313 | -1.4669022 | -0.5825803 | -1.0960849 | -0.1479518 | -0.8846823 |
| 1.4560564 | -0.2152639 | -1.0208332 | 0.8695795 | 0.8634816 | -0.3031835 | 0.0097010 | -0.3771972 | -0.0798799 |
| 0.1512532 | -0.0076896 | -0.3482288 | -0.0878568 | -0.5376665 | -0.1608336 | -0.2975445 | -0.1176725 | 0.0664973 |
| -0.1212968 | -0.4196711 | 0.2628705 | -0.1840804 | -0.8946285 | 0.1927158 | -1.0732215 | 0.1192266 | 0.0722173 |
| 0.1291015 | -0.2387560 | -0.3490749 | -0.4385971 | -0.4639701 | -0.3985059 | -0.4209008 | -0.6310429 | -0.8234119 |
| -0.2663519 | -0.1556843 | -0.4380822 | -0.4775900 | -0.4873553 | -0.3961310 | -0.5932059 | 0.9556653 | 0.5844954 |
| 0.8773482 | 0.5406064 | -0.2119727 | 0.1444314 | 0.6069994 | 0.2526464 | 0.6468279 | -0.1276975 | 0.0860515 |
| -0.8016203 | -0.8722749 | -0.8530557 | -0.7077961 | -0.3974510 | -0.7795175 | -0.6634455 | -0.4990694 | -0.6735015 |
| 0.0848199 | 0.7766955 | 0.1544578 | 0.6256708 | 1.2978051 | 0.6808333 | 1.3716171 | -0.7715165 | -0.5638434 |
| -1.2496160 | -1.4843286 | -0.4078664 | -1.1253010 | -1.4670076 | -0.6936591 | -1.8854548 | -0.4794420 | -0.8656054 |
| -0.9959882 | -1.9037527 | -0.8821142 | -0.7692634 | -2.0043821 | -0.7941722 | -2.0720559 | -1.1707542 | -1.2167876 |

| | | | | | | | | |
|---|---|---|---|---|---|---|---|---|
| 0.5706215 | 0.6183569 | 0.0668440 | -0.162664 | 0.2662005 | 0.0544119 | 0.0415714 | -0.082179 | 0.2396994 |
| -1.273443 | -1.488531 | -0.704726 | -2.046423 | -1.900422 | -1.356333 | -1.955414 | -1.068950 | -1.576427 |
| -0.224368 | -0.080492 | -0.364445 | -0.794051 | -0.175111 | -0.424405 | -0.436522 | -0.293808 | -0.137892 |
| -0.171780 | -0.253606 | 0.3433415 | 0.7825085 | 0.4569725 | 0.4385693 | 0.7274762 | -0.030770 | -0.276680 |
| -1.044863 | -1.050963 | -0.527842 | -1.461044 | -1.889495 | -0.935614 | -2.057025 | -0.124745 | -0.690434 |
| 0.8743121 | 0.4720207 | 0.6235782 | 2.7333150 | 2.0863275 | 1.9471086 | 1.6596135 | 0.3397523 | 0.9873460 |
| -0.487051 | -0.753942 | -0.471374 | -0.668805 | -0.989916 | -0.521365 | -1.208669 | -0.551728 | -0.645373 |
| -1.499127 | -1.282590 | -0.449661 | 0.0417166 | -0.676840 | -0.325196 | -1.232963 | -0.574660 | -0.703529 |
| -1.214681 | -1.355478 | -0.576500 | -1.094389 | -2.073888 | -0.939871 | -1.898457 | -0.646620 | -0.969904 |
| -0.243473 | 0.4869952 | -0.478253 | -0.722579 | -0.547595 | -0.487743 | -0.532659 | -0.741920 | -0.708221 |
| 0.2291816 | 0.4831892 | -0.151115 | -0.059190 | 0.1898198 | -0.197728 | 0.0590695 | -0.154518 | -0.028919 |
| -1.099392 | -0.281665 | -0.567411 | -1.222081 | -1.183365 | -1.048233 | -1.489201 | -0.349752 | -0.741943 |
| -0.851161 | 2.1159636 | -0.397644 | -1.562091 | -0.889453 | -0.704789 | -1.319172 | 0.0894647 | -0.490845 |
| -0.566793 | -0.316615 | -0.017706 | 0.1568217 | 0.1728007 | -0.026407 | 0.3301273 | 0.1569155 | 0.0924829 |
| -0.272253 | -0.466137 | -0.539283 | -1.130470 | -2.954092 | -0.198281 | -2.042795 | -0.107148 | -0.622220 |
| 0.0163100 | -1.003485 | -1.001720 | -1.382654 | -2.626969 | -1.148622 | -2.409502 | 0.0495460 | -0.214080 |
| -0.396641 | -0.613016 | -0.287659 | -0.608875 | -0.116181 | -0.211392 | -0.067925 | -0.508005 | -0.665261 |
| -1.848240 | -2.849159 | -0.306212 | -0.816327 | -1.338077 | -0.600455 | -1.766623 | -0.051514 | -0.375346 |
| -1.085206 | -0.631281 | -0.305283 | -0.538148 | 0.0826172 | -0.734679 | -0.000910 | 0.2768599 | -0.211973 |
| -0.889775 | -0.869437 | -0.284771 | -0.551099 | -0.855737 | -0.440332 | -0.765987 | -0.217291 | -0.694706 |
| -0.078034 | 0.6459893 | -0.195137 | -0.096323 | 0.9259647 | -0.105324 | 2.1070965 | -0.222502 | -0.003147 |
| 0.5282137 | 0.2228639 | -0.066183 | 0.5558111 | 0.4738299 | 0.0009531 | 0.2917835 | -0.362830 | -0.411227 |
| -1.413290 | -0.946980 | -0.074959 | -1.454813 | -2.022122 | -0.453193 | -2.246406 | -0.438935 | -1.403753 |
| -0.437966 | -0.538374 | -0.394961 | -0.722723 | -1.625205 | -0.574538 | -1.866756 | -0.149994 | -0.215470 |
| -0.518684 | -1.385677 | -0.289718 | -1.087845 | -2.370117 | -0.608892 | -2.605609 | -0.286743 | -0.742130 |
| -1.183608 | -0.591900 | -0.286368 | -0.346169 | -0.852200 | -0.287026 | -0.972991 | -0.376624 | -0.421471 |
| -0.793972 | -0.951821 | -0.968901 | -1.505676 | -2.010118 | -1.245040 | -2.398897 | -0.643887 | -0.959617 |
| -0.592780 | -0.262972 | -0.321801 | -0.754561 | 0.0501806 | -0.589614 | 0.0788075 | -0.345189 | -0.674291 |
| -1.089993 | -1.138828 | -0.030902 | -0.667493 | -0.727868 | -0.279102 | -1.438786 | 0.1187698 | -0.590442 |
| 0.7865259 | 0.8361439 | 0.0568194 | 1.1537262 | 1.7568108 | 0.5464889 | 1.4755115 | -0.172270 | 0.5616266 |
| 2.9010385 | 3.4620792 | -0.366944 | 0.8938111 | 2.5569839 | 0.2712265 | 2.6532752 | -0.873013 | 1.0264262 |
| -0.548697 | -0.264061 | -0.565142 | -0.424202 | 0.6054604 | -0.539638 | 0.4745575 | -0.458521 | -1.170202 |
| -0.082847 | -0.504005 | -0.450517 | 0.2094532 | 0.1829764 | -0.393781 | 0.0551454 | -0.657088 | -0.545883 |
| 0.3486311 | 0.5135837 | -0.109749 | 0.4362790 | 0.7445756 | -0.002451 | 0.7016983 | -0.204541 | 0.0880572 |
| 0.5082438 | 0.8971280 | 0.1629248 | 1.5101751 | 1.2422068 | 0.6069105 | 1.2208877 | 0.5054877 | 0.9756152 |
| -0.419462 | -1.004007 | -0.216506 | -1.444324 | -0.943119 | -0.614222 | -0.808298 | -0.258363 | -0.778090 |
| 1.1190776 | 1.7294877 | 0.2429015 | 0.8902405 | 1.3653688 | 0.9091884 | 1.3093318 | 0.1429752 | 0.3019755 |
| 0.7537260 | 0.6154114 | -0.095013 | 0.7507633 | 1.6948005 | 0.3109710 | 1.9374174 | -0.541986 | -0.037719 |
| 0.1931266 | 0.3125138 | 0.0649121 | 0.1623241 | 0.8259183 | 0.2604517 | 0.7021827 | -0.916267 | -0.585911 |
| -0.241178 | -0.149149 | -0.033722 | -0.191079 | -0.434997 | -0.076445 | -0.363045 | -0.414673 | -0.714866 |
| -2.821177 | -1.455339 | -0.970034 | -1.454737 | -2.252835 | -0.882615 | -1.476715 | -0.151036 | -0.967216 |
| 0.2322671 | 0.6347074 | -0.338481 | 0.3109396 | 0.7298257 | -0.219290 | 0.6579583 | 0.1246646 | 0.2480063 |
| -0.014822 | 0.6139825 | 0.5468774 | 1.8660907 | 2.9380490 | 0.8531129 | 2.1602162 | -0.168989 | 0.9205945 |
| 0.4813943 | 0.9191420 | -0.328527 | 0.2018837 | 0.7778729 | -0.392194 | 0.6328984 | -0.219095 | 0.1654110 |
| -0.605080 | -0.120302 | -0.243231 | -0.580830 | -0.968481 | -0.228225 | -1.079198 | -0.513374 | -0.909289 |
| -0.084526 | 0.2451161 | -0.305793 | -0.130297 | 0.1407333 | -0.069444 | 0.1651359 | -0.464604 | -0.330294 |
| -1.053713 | -1.747261 | -0.341333 | -0.856152 | -2.097678 | -0.564787 | -2.151674 | -0.505556 | -1.138235 |
| -0.694289 | -1.129345 | -0.728846 | -0.975805 | -1.364553 | -1.055069 | -1.271483 | -0.511490 | -1.301384 |
| 0.3683414 | 0.4902044 | 0.1554995 | -0.781126 | -1.580047 | -0.065295 | -1.757411 | -0.196204 | -0.748351 |
| -0.232213 | -0.675919 | -0.034699 | 0.1787625 | 0.2636822 | -0.131344 | 0.0243911 | -0.136282 | -0.131300 |

| | | | | | | | | |
|---|---|---|---|---|---|---|---|---|
| -0.126430 | 0.1787540 | 0.3189458 | 0.0950860 | 0.6245725 | 0.3538119 | 0.2379208 | -0.077487 | -0.439787 |
| -0.769310 | -0.765662 | 0.2451483 | -0.107553 | -0.402059 | 0.0913306 | -0.216942 | -0.660204 | -1.070439 |
| 0.1036040 | -0.289737 | -0.710561 | 0.0573952 | -0.186003 | -0.295197 | 0.0999378 | -0.402272 | 0.0662869 |
| -0.794040 | -0.866394 | -0.283566 | -1.245440 | -1.938030 | -0.674582 | -1.967844 | -0.500252 | -1.172085 |
| -0.426972 | -0.737194 | -0.296841 | -0.750728 | -1.387055 | -0.331975 | -1.383539 | -0.985187 | -0.819253 |
| -0.458115 | -0.782339 | 0.0030878 | 0.3073446 | -0.438505 | -0.063973 | -0.623477 | -0.576562 | -0.130306 |
| 1.3554956 | 1.3215035 | -0.230471 | 2.0499814 | 2.2783729 | 0.6826497 | 1.9007701 | 0.0482165 | 1.1924863 |
| -0.709871 | -0.013811 | -0.394988 | -0.360784 | -0.325620 | -0.504888 | -0.148815 | -0.351246 | -0.634532 |
| -0.125464 | -1.293834 | -0.000547 | -0.457882 | -1.580052 | -0.079408 | -1.103677 | 0.1578901 | 0.0227425 |
| -0.339125 | 0.6856927 | -0.696925 | -0.930410 | -1.137568 | -0.518665 | -1.428545 | 0.0512334 | -0.655617 |
| -0.261472 | -0.984523 | -0.680191 | 0.3749992 | 0.6822199 | -0.812855 | -0.001299 | -0.085829 | -0.446902 |
| -0.565474 | -0.754094 | -0.973922 | -1.102054 | -0.602370 | -1.318727 | -1.065921 | -1.128497 | -0.705128 |
| -0.438795 | 0.0046577 | -0.329053 | -0.062451 | -0.435632 | 0.1165426 | -0.392451 | -0.485780 | -0.151028 |
| -0.582896 | -0.450253 | -0.097198 | -0.365409 | -1.229370 | -0.264556 | -1.442012 | -0.157074 | -0.485476 |
| -1.502531 | -1.955611 | -0.014073 | -1.035274 | -1.550369 | -0.244167 | -1.844440 | -0.272711 | -0.552111 |
| -0.242428 | -0.715574 | -0.338780 | -0.501815 | -1.498364 | -0.600865 | -1.576291 | -0.255252 | -0.748627 |
| -0.589695 | -1.129496 | -0.110497 | -0.193771 | -0.978753 | -0.183875 | -1.094449 | -0.282773 | -0.271354 |
| -0.710582 | -0.632176 | -0.425742 | -1.004316 | -0.556166 | -0.657691 | -0.579033 | -0.287663 | -0.815798 |
| -1.569938 | -1.722547 | 0.1075320 | -1.200142 | -1.231107 | -0.304598 | -1.696628 | -0.470132 | -1.194370 |
| 0.6476838 | 0.4762902 | 0.2172068 | 0.5916646 | 0.9444807 | 0.1344422 | 0.6602698 | 0.0507976 | 0.1907995 |
| -0.236944 | -1.521572 | -0.211591 | -0.913549 | -0.169997 | -0.445200 | -0.573973 | -0.048044 | -0.321661 |
| -0.739067 | -1.430055 | -0.071387 | -1.149628 | -1.995498 | -0.842848 | -1.960912 | 0.7100345 | -0.090830 |
| -1.872822 | -2.875454 | -0.170060 | -1.716933 | -4.196893 | -0.398135 | -3.406567 | -0.241504 | -1.280548 |
| -0.538726 | 0.3125950 | -0.129136 | -0.413468 | 0.2555439 | -0.190587 | 0.0026852 | -0.170781 | -0.124630 |
| -0.192827 | -0.869257 | 0.1462862 | -0.232313 | -1.254867 | 0.3904013 | -0.957851 | -0.133513 | -0.029676 |
| -0.535975 | -1.391637 | -0.581067 | -0.545722 | -0.238127 | -0.685259 | -0.400569 | -0.089984 | -0.226659 |
| -0.696890 | -0.742981 | 0.0095998 | -0.431822 | -0.645560 | -0.050875 | -0.589688 | -0.170997 | -0.189875 |
| -0.844284 | -1.316159 | -0.295893 | -0.444302 | -1.058205 | -0.365478 | -0.953572 | -0.339921 | -0.583130 |
| -0.139816 | 1.6375222 | 0.1251017 | 0.2448899 | 1.8649359 | 0.3062011 | 1.5855431 | -0.186760 | 0.2194560 |
| 0.1908637 | 1.3696480 | -0.380092 | 0.5180274 | 0.9608956 | -0.552236 | 0.7533919 | -0.262187 | 0.3764413 |
| -0.851448 | -1.294592 | -0.533408 | -0.383027 | -0.792775 | -0.396108 | -1.176591 | -0.613709 | -0.697210 |
| -0.472819 | -0.615206 | -0.019791 | -0.383741 | -0.349587 | -0.133952 | -0.392346 | -0.109793 | -0.463167 |
| 0.0095076 | 1.5459866 | -0.734655 | -0.674940 | 2.3290287 | -0.456490 | 2.2496019 | -0.419696 | 0.1756464 |
| -1.449417 | -1.244822 | -0.231677 | -0.650820 | -1.343897 | -0.300508 | -1.591764 | -0.401240 | -0.422483 |
| 0.3887558 | -0.688975 | -0.216839 | -0.110543 | -0.740600 | -0.108736 | -0.747628 | -0.301333 | -0.496386 |
| -0.820529 | -1.581108 | -0.499294 | -0.260643 | -0.241928 | -0.572190 | -0.861654 | -0.156103 | -0.574027 |
| -1.974090 | -2.241828 | -0.583130 | -1.392887 | -1.919186 | -0.752084 | -1.926018 | -0.869303 | -1.522976 |
| -0.195651 | -0.057240 | -0.136000 | -0.154636 | -0.630198 | -0.253821 | -0.464617 | 1.0403630 | -0.035933 |
| 0.5584487 | 0.0336307 | -0.012810 | 1.6341672 | 0.9465030 | 0.1690700 | 0.8706011 | -0.205865 | 1.1367072 |
| -1.099700 | -2.348684 | 0.1148229 | -0.338091 | -0.667615 | -0.130067 | -0.676231 | -0.229480 | -0.496261 |
| 0.0255435 | 1.2850909 | -0.004740 | 0.4014775 | 0.8182701 | 0.1489266 | 0.5883673 | -0.205588 | 0.0056652 |
| -0.470818 | -1.251299 | -0.680326 | -1.393957 | -1.926618 | -0.904938 | -1.749042 | -0.460493 | -0.804436 |
| -0.415117 | -0.730863 | -0.582671 | -0.867802 | -1.282877 | -0.651709 | -1.681451 | -0.248335 | -0.307067 |
| -0.655732 | -0.125197 | -0.732760 | -0.674650 | -0.313856 | -0.711909 | -0.423107 | 0.0252225 | 0.5025068 |
| -0.178530 | -0.032997 | -0.406001 | -0.571124 | -0.130357 | -0.117331 | 0.2354493 | -0.639684 | -0.661092 |
| -0.882311 | -1.384788 | -0.132536 | -0.526309 | -1.591225 | -0.336231 | -2.072337 | -0.978196 | -1.379899 |
| -0.396293 | -0.354993 | -0.192070 | -0.449183 | -0.355794 | -0.158206 | -0.493682 | -0.171604 | -0.090009 |
| -0.717565 | -1.661434 | -0.595280 | -0.741274 | -1.164641 | -0.672368 | -1.691894 | -0.874550 | -0.633030 |
| 0.4555391 | 1.4981244 | -0.191304 | -0.612453 | -0.250454 | 0.0148916 | 0.0098734 | -0.504217 | -0.574598 |
| -1.130082 | -1.370413 | -0.975400 | -1.663930 | -1.974106 | -1.383634 | -1.984596 | -0.871988 | -1.139811 |

| | | | | | | | | |
|---|---|---|---|---|---|---|---|---|
| -0.259572 | 1.3419108 | -0.239995 | -0.670000 | 1.0084891 | -0.112968 | 1.1269234 | -1.358183 | -0.998850 |
| 0.0731538 | 0.4764170 | -0.252658 | -0.369040 | 0.2036633 | 0.0759700 | 0.0210120 | -0.279317 | -0.372910 |
| -0.024272 | 0.1258276 | 0.0440579 | -0.116121 | -0.047243 | 0.1001478 | -0.054828 | -0.131267 | 0.0048606 |

| PG_613 | PG_614 | PG_615 | PG_616 | PG_617 | PG_618 | PG_619 | PG_620 | PG_621 |
|---|---|---|---|---|---|---|---|---|
| -1.388141 | 0.0919134 | -0.357242 | -2.342810 | -0.386785 | -0.467480 | -0.800276 | -0.270415 | -0.754601 |
| -0.910409 | -0.783342 | -0.396031 | -3.377343 | -0.324457 | -0.609300 | -1.055408 | -0.424720 | -0.358863 |
| -1.959025 | -0.369334 | -0.789752 | -2.052448 | -0.550222 | -1.292823 | -2.123646 | -0.454146 | -1.632808 |
| 0.0808900 | 0.0890050 | -0.252289 | -4.204340 | -0.156027 | -0.098861 | -0.380959 | -0.235398 | -0.265687 |
| -0.534575 | -0.139469 | -0.704631 | -2.685796 | 0.0351231 | -0.086181 | -1.126789 | -0.005344 | -0.084436 |
| -1.130153 | -0.263117 | -0.193286 | -2.163460 | -0.520651 | -1.034764 | -2.117549 | -0.800182 | -1.335424 |
| -1.066358 | -0.275475 | -1.160059 | -1.578451 | -0.376767 | -0.895732 | -0.811699 | -0.584632 | -1.214231 |
| -1.185552 | 0.3154282 | -0.022034 | -2.562433 | -0.172715 | -0.075387 | -1.180136 | -0.211235 | -0.190392 |
| -0.411185 | 0.1440790 | -0.168728 | -0.162512 | -0.392360 | -0.456365 | -0.406267 | -0.388261 | -0.316190 |
| -1.314006 | 0.2749104 | -0.539633 | -1.448428 | -0.554220 | -1.510080 | -2.212803 | -1.092024 | -2.053482 |
| -0.508433 | -0.102012 | -0.239119 | -1.937624 | -0.113793 | -0.153738 | -0.626926 | -0.200992 | -0.374531 |
| -0.692142 | -0.143778 | -0.511614 | -1.976844 | -0.208533 | -0.023655 | -0.180408 | -0.163142 | -0.303643 |
| -1.990664 | -0.505556 | -1.355690 | -4.147819 | -0.059543 | -0.696345 | -1.286939 | -0.325072 | -1.011497 |
| 2.5103570 | -0.112543 | 2.6442216 | 1.2135894 | -0.952885 | 2.4801489 | 3.6892208 | 0.1003818 | 4.8831231 |
| 0.6840032 | -0.034594 | -0.625322 | -1.679350 | -0.121923 | -0.134264 | 0.0492617 | -0.004650 | 0.2709641 |
| 3.1691539 | 0.3167145 | 3.4145007 | 2.8226935 | -0.229597 | 1.0446799 | 1.7246504 | 0.4127003 | 2.0534098 |
| -1.485372 | -1.056759 | -1.909046 | -1.374009 | -0.522255 | -1.631915 | -1.077781 | -1.123536 | -1.566658 |
| -1.423642 | -1.357462 | -1.330372 | -0.994532 | -1.213326 | -2.372741 | -1.786911 | -1.770653 | -2.333362 |
| -0.445308 | -0.595226 | -0.326516 | 0.2405466 | -0.950111 | -1.011586 | -1.123238 | -0.993554 | -0.751767 |
| -1.707309 | -1.071432 | -0.545701 | -2.599958 | -0.748758 | -0.660692 | -0.880702 | -0.600326 | -0.890839 |
| 0.1592347 | -0.370385 | 0.0765868 | -1.017768 | -0.216969 | -0.046257 | -0.146687 | -0.201326 | -0.005840 |
| -0.633575 | -0.422857 | -0.491865 | -0.205158 | -0.589467 | -0.711831 | -1.076017 | -0.652087 | -0.778273 |
| 0.6458874 | -0.364645 | 0.8517072 | 1.1950281 | -0.088293 | 0.4284044 | 2.1493336 | 0.0068148 | 0.7102076 |
| -2.409536 | -0.424295 | -1.597404 | -2.732648 | -0.070792 | -0.207139 | -1.042312 | -0.253514 | -1.404868 |
| -2.777090 | -0.401950 | -1.456378 | -2.696228 | -0.440140 | -1.062223 | -1.444945 | -1.035697 | -1.840321 |
| 0.7209655 | -0.201657 | 0.3774379 | 0.2357269 | -0.205408 | 0.3629326 | 1.0430379 | -0.100361 | 0.2888371 |
| -0.172377 | -0.041602 | -0.172173 | -3.398345 | 0.0425406 | 0.0630095 | -0.523339 | 0.0169748 | 0.0863389 |
| -1.785972 | -0.651437 | -1.208955 | -2.933367 | -1.017895 | -1.588305 | -0.978257 | -1.245097 | -1.239177 |
| -2.595461 | -0.560635 | -1.688750 | -3.841544 | 0.0087250 | -0.300598 | -2.385401 | -0.056329 | -0.623743 |
| -0.697602 | -0.126824 | -0.748259 | -2.717768 | -0.044111 | -0.059751 | -0.122110 | -0.091934 | -0.322298 |
| -3.752304 | -0.470786 | -1.512100 | -4.064681 | -0.604263 | -0.857889 | -3.593973 | -0.747239 | -1.449882 |
| -2.020949 | 0.0349290 | -0.780742 | -2.524214 | 0.5620473 | 0.6050623 | -0.851145 | 0.5443837 | 0.2745654 |
| 0.9603392 | -0.236619 | 0.9155311 | 0.3855653 | -0.484560 | -0.322138 | -0.770888 | -0.330252 | 0.5465976 |
| -2.481629 | -1.120389 | -1.450347 | -4.135248 | -0.097874 | -0.351143 | -2.885293 | -0.212391 | -0.806658 |
| -2.038153 | 0.3391717 | -0.403395 | -1.764402 | -0.327558 | -0.439666 | -0.148884 | -0.494232 | -0.289361 |
| -1.116569 | -0.674051 | -0.370568 | -1.996000 | -0.814215 | -0.718580 | -0.097066 | -1.109318 | -0.596960 |
| -1.479191 | -0.469795 | -1.396170 | -2.431253 | -0.001921 | -0.535800 | -1.859094 | -0.398728 | -0.821650 |
| 1.2070021 | 0.2118921 | 3.2357500 | 0.9584348 | -0.010425 | 4.3580009 | 1.2470356 | 3.6005388 | 5.0038683 |
| -0.411563 | -0.017522 | -0.335274 | -1.980180 | -0.086729 | 0.2086789 | -0.178420 | -0.114082 | -0.003901 |
| -2.828895 | -1.826181 | -1.778230 | -3.439006 | -0.717851 | -0.731972 | 0.8073112 | -1.030574 | 0.4319936 |
| 0.8781506 | -0.473125 | 0.1213274 | 0.7737799 | -0.121243 | 0.3699500 | 0.6457841 | 0.1856569 | 0.2104972 |
| -2.720315 | -0.881855 | -1.447821 | -2.371673 | -0.470390 | -0.637607 | -1.890701 | -0.478025 | -1.036496 |
| -1.269950 | -0.508192 | -0.256505 | -2.336971 | -0.096036 | -0.082851 | -1.094202 | -0.056591 | -0.244516 |
| -0.167538 | -0.021442 | -0.003725 | -3.363076 | -0.306834 | 0.0813292 | -1.138777 | -0.296586 | -0.057430 |
| 1.3150589 | 0.1426008 | 0.8901150 | 2.2654810 | -0.139625 | 0.5850924 | 1.4410035 | 0.1699237 | 0.4400826 |
| 2.0963234 | -0.528706 | 0.3238526 | 2.1630031 | 0.4010174 | 0.5452457 | 3.3535148 | 0.7930727 | 1.5771357 |
| 0.0483125 | -0.202801 | 0.3655017 | 0.9578686 | -0.151173 | -0.192185 | 0.1505890 | -0.171788 | -0.315835 |

| | | | | | | | | |
|---|---|---|---|---|---|---|---|---|
| 0.0804042 | -0.5218465 | -0.1610531 | -1.4854888 | -0.4563361 | -0.1943363 | -0.1096432 | -0.4621219 | 0.2650602 |
| -2.2792313 | -0.6912067 | -1.1579757 | -3.4604683 | -0.2415707 | -0.3451169 | -0.7369006 | -0.1387802 | -0.4699601 |
| -0.0382888 | -0.3554880 | 0.0789879 | -1.4829398 | -0.2396746 | -0.0935470 | 0.3998892 | -0.1526855 | 0.1744169 |
| 2.9171226 | 0.0476278 | 1.8730584 | 2.5685561 | -0.8748813 | 0.7252076 | 1.0536843 | 0.2213311 | 1.7590084 |
| -0.2266637 | -0.3304104 | -0.6189341 | -0.0575513 | -1.3814871 | -2.0131684 | -2.5112514 | -2.0970550 | -1.2976472 |
| -0.2376082 | -0.2106513 | 0.0174326 | 0.3556564 | -0.4798263 | -0.3267151 | -0.1663851 | -0.3866165 | -0.4955369 |
| -1.8605986 | -0.4650558 | -1.1976834 | -2.1948657 | -0.1144510 | -0.3305844 | -1.1240918 | -0.5284378 | -0.8493825 |
| 1.9797842 | -0.4631081 | 1.3586906 | 2.4785874 | -0.4638551 | 0.9759857 | 2.1010367 | 0.2954124 | 2.0154860 |
| 0.8528427 | 0.6637338 | 2.6203801 | 0.6554672 | 0.0200589 | 4.2563524 | 0.6781331 | 3.8944083 | 5.1944073 |
| -0.1942319 | -0.6481389 | -0.2821374 | -1.3136924 | -0.8993209 | -0.1480166 | 0.5083942 | -0.8332469 | -0.1608070 |
| -1.9931687 | -0.8634515 | -1.6362367 | -2.1638174 | -0.8052548 | -1.0287047 | -1.1997810 | -0.7728695 | -1.0768771 |
| 3.2568186 | 1.2755997 | 3.2297509 | 2.2065147 | -0.0693757 | 1.8050506 | 1.8443604 | 1.2772925 | 2.8861212 |
| 1.3891155 | 0.4609118 | 2.6070163 | 0.2557471 | -0.2892890 | 1.1337159 | 1.0078751 | 0.3895239 | 2.2199625 |
| -0.8846250 | 0.0901937 | -0.3013642 | -1.3784399 | -0.4106795 | -0.6658115 | -0.2644998 | -0.4210521 | -0.8573696 |
| -2.3242291 | -0.8845513 | -2.4243610 | -3.8218984 | -0.6532136 | -1.5884716 | -1.7726724 | -1.2946979 | -1.9982015 |
| -1.3555219 | -0.7718182 | -1.4621124 | -1.7311312 | -0.5368694 | -0.6072739 | -0.3966107 | -0.6843313 | -0.0923774 |
| -2.6100522 | -0.3920993 | -1.4888783 | -3.5570195 | 0.0071413 | 0.0351473 | -1.1795989 | -0.1533165 | -0.5756001 |
| -0.9469334 | -0.5901806 | -0.8495662 | -1.2651481 | -0.3635987 | -0.4424965 | -0.5263338 | -0.4487859 | -0.6834877 |
| -1.0605353 | -0.3005157 | -0.7831774 | -1.9185799 | 0.0490754 | -0.5234655 | -1.1436782 | -0.2201246 | -0.9675934 |
| -0.3021911 | 0.3677835 | -0.0607221 | -3.7538740 | -0.0794505 | -0.3626596 | -1.5326899 | -0.0296486 | -0.2216880 |
| 0.0499201 | 0.2064753 | 0.1416132 | -0.6152323 | -0.0871215 | 0.184616 | -0.4031365 | -0.3145154 | 0.0515606 |
| -1.8649443 | -0.8807959 | -1.4283261 | -2.1724302 | -0.1309725 | -0.4448089 | -0.9886138 | -0.5748175 | -0.9147346 |
| 0.5660432 | -0.0117171 | 0.1667008 | 1.6249387 | -0.0269590 | -0.5792709 | 0.2198724 | -0.2677516 | -0.2857869 |
| 0.3389411 | -0.0526223 | 0.4393912 | -3.8020652 | 0.0899203 | 0.5997296 | 0.4450004 | 0.2577319 | 0.5993928 |
| -2.2872861 | -0.6604921 | -0.9969614 | -2.6631907 | -0.4948404 | -0.3872606 | -1.4916450 | -0.5908102 | -1.0006455 |
| -0.4968842 | -0.0602833 | -0.2539667 | -0.7347734 | -0.1706593 | -0.4716179 | -0.5385271 | -0.3676839 | -0.5584951 |
| -2.9675996 | -1.0472591 | -3.1593384 | -2.5116552 | -0.5406095 | -2.4861340 | -2.0161342 | -1.3719778 | -3.4259769 |
| 2.6681761 | 0.2774394 | 1.5862440 | 2.3439405 | 0.1463534 | 1.8089180 | 3.7155931 | 0.4691335 | 2.0347430 |
| 0.8705870 | -0.4976846 | 0.8289467 | 0.1031651 | -0.3068531 | 1.0154556 | 2.2818572 | 0.0458982 | 1.9136011 |
| -1.5074391 | 0.2235497 | -0.0975361 | -1.4643927 | -0.5248033 | -0.4033184 | -1.8637510 | -0.6453888 | -0.8070287 |
| -0.7459112 | -0.3585728 | -0.3224112 | -0.2825607 | -0.0181897 | -1.0083291 | -0.5632732 | -0.5018639 | -0.9315019 |
| -0.3942463 | -0.3025468 | -0.6433204 | -4.0743249 | 0.1167475 | 0.1889009 | -0.2293811 | 0.2536869 | -0.0472044 |
| -1.4121035 | -0.8175346 | -1.7071259 | -1.4093482 | -0.5007210 | -0.9220741 | -0.8793154 | -0.8516786 | -1.8307366 |
| -1.9693293 | -0.3365284 | -0.9143907 | -2.9824281 | -0.4171680 | -0.4695921 | -1.1723569 | -0.5848670 | -0.8912111 |
| 1.3501882 | -0.3845611 | 0.8258823 | 1.3375126 | -0.0917360 | 0.8617782 | 1.7770342 | 0.2237274 | 0.9925587 |
| -1.5768798 | 0.2638035 | -0.6385390 | -0.7173929 | 0.0249868 | 0.1631925 | 0.0775928 | -0.0519092 | -0.4709661 |
| -2.0909375 | 0.5543464 | -0.0199528 | -3.1770253 | -0.6227474 | -0.4540636 | -0.5411942 | -0.3374431 | -0.4107513 |
| -3.2683875 | -0.2972814 | -1.2623893 | -2.6071105 | -0.1571570 | -0.2615859 | -1.6615952 | -0.3530544 | -0.9363052 |
| -1.9129314 | -0.0554160 | -1.0411636 | -1.8323619 | -0.3889123 | -0.7120698 | -1.8679371 | -0.6053492 | -1.2717181 |
| -1.8248275 | -1.0194108 | -0.6952151 | -1.7445735 | -0.6103930 | -1.3508593 | -2.0113309 | -0.7371758 | -0.8409528 |
| -1.2663706 | -0.1407213 | -0.3858758 | -0.6482967 | -0.4605627 | -0.5611211 | -1.2278878 | -0.8666683 | -0.4538673 |
| -0.3899580 | 0.6554566 | -0.7442020 | -1.6615333 | -1.0268278 | -3.2205996 | -3.0617021 | -2.3092899 | -3.5054954 |
| 4.2888728 | 0.9144968 | 3.8327596 | 4.1382410 | 0.5372850 | 3.3406500 | 3.2100990 | 2.5280359 | 4.6444832 |
| -0.4056008 | -0.0560771 | -0.0914814 | -1.2079670 | -0.0126636 | -0.0153128 | -0.1528092 | 0.0268242 | -0.0966599 |
| -0.6978107 | -0.4547081 | -0.6516929 | -3.5624888 | 0.0339978 | -0.1498223 | -0.8800779 | -0.0093501 | -0.0847911 |
| -1.9792759 | -0.4769401 | -0.9475220 | -0.9190551 | -0.7175321 | -1.1398865 | -1.0448282 | -1.0600830 | -1.2528028 |
| 8.1158071 | 3.3127543 | 7.9554248 | 8.7591722 | -0.1729767 | 4.1448592 | 5.9867978 | 2.6583234 | 7.4726968 |
| 2.2560823 | 0.2871984 | 2.4318970 | 1.8473640 | -0.0910292 | 0.5447796 | 0.9753409 | 0.0960141 | 1.3624864 |
| -2.3598393 | -1.3253219 | -1.4886994 | -2.6898175 | 0.3266526 | -0.2220316 | -3.2037494 | 0.1395904 | -0.1853205 |
| -0.4539329 | 0.0805896 | 0.1095006 | -1.0619330 | -0.7500941 | -0.3104937 | -0.1752308 | -0.8377312 | -0.2157979 |

| | | | | | | | | |
|---|---|---|---|---|---|---|---|---|
| -3.549996 | 0.0920742 | -1.717589 | -4.053493 | 0.5005172 | 0.1151452 | -1.395330 | 0.3402498 | -0.072497 |
| 0.6766423 | -0.320445 | -0.101331 | 0.7346553 | -0.138909 | -0.000751 | 0.5163433 | -0.153517 | -0.041422 |
| -0.251599 | -0.352207 | -0.839970 | -0.691222 | -0.136001 | -0.338437 | 0.5254384 | -0.339470 | -0.900467 |
| -0.464898 | -0.223886 | -0.222973 | -1.379439 | 0.0200444 | -0.046959 | -0.241235 | -0.017958 | -0.043994 |
| -1.902275 | -0.479376 | -1.722510 | -3.308720 | -0.303425 | -0.981970 | -1.487403 | -0.483137 | -1.295717 |
| -1.652400 | 0.0414247 | -0.087149 | -1.765127 | -0.397074 | -0.479927 | -1.762682 | -0.580018 | -0.677870 |
| 0.0205794 | -0.169544 | -0.250981 | -1.218165 | 0.0609776 | -0.055474 | 0.1407912 | -0.165739 | -0.171104 |
| -0.017052 | -0.278034 | -0.169802 | 0.3464872 | -0.414430 | 0.1806999 | -0.164693 | -0.328470 | -0.129667 |
| -0.251017 | -0.373155 | -0.505719 | 0.6987143 | -0.253569 | -0.074728 | -0.415598 | -0.356902 | -0.221376 |
| 1.0786283 | -0.133177 | 0.6399836 | -0.225315 | 0.2279002 | 0.5708663 | 0.9305542 | 0.2182803 | 0.7677104 |
| -1.136205 | -0.243031 | -0.690520 | -1.900315 | -0.123020 | -0.138269 | -1.788780 | -0.216639 | -0.211053 |
| -2.075135 | -1.244271 | -1.307624 | -3.921104 | -0.244682 | -0.572636 | -0.950224 | -0.702102 | -0.515327 |
| -3.749675 | -0.854408 | -2.404638 | -4.572073 | 0.1069296 | -0.257485 | -3.237294 | -0.035886 | -0.691807 |
| -2.249467 | -0.283027 | -1.001700 | -3.434097 | 0.1178438 | -0.166372 | -1.021345 | 0.0481989 | -0.364506 |
| -0.709750 | -0.717936 | -0.962854 | -2.508984 | -0.312276 | -0.549296 | -0.539912 | -0.491350 | -0.529105 |
| 0.0208498 | -0.223348 | -0.102578 | -0.331235 | -0.067754 | 0.1495995 | -0.178642 | -0.111362 | -0.076581 |
| -1.085533 | -0.100080 | -0.984885 | -1.713587 | -0.523102 | -1.229031 | -0.632798 | -0.923615 | -1.292852 |
| 0.0329273 | -0.067269 | -0.012547 | 0.9496159 | -0.310903 | -0.214205 | -0.118370 | -0.427918 | -0.230402 |
| -0.662999 | -0.625524 | -0.951184 | -1.700345 | -0.098368 | -0.344005 | 0.6140136 | -0.555274 | -0.476883 |
| 1.3760066 | -0.702143 | 0.0654649 | 1.9942535 | -0.131797 | 0.4762801 | 1.2831275 | -0.067696 | 0.7997590 |
| -1.250163 | -0.388951 | -0.754857 | -3.533889 | -0.490120 | -0.334758 | -1.558686 | -0.494019 | -0.580793 |
| -0.919229 | -0.479523 | -0.741736 | -2.250652 | -0.509136 | -0.376491 | -0.171683 | -0.559773 | -0.511564 |
| -0.118946 | -0.389276 | 0.1295346 | 0.0501644 | 0.3367755 | 0.1879472 | -1.602640 | 0.6526183 | 0.3455116 |
| -1.074253 | -0.507307 | -0.792555 | -1.736887 | -0.156990 | -0.670722 | -1.975422 | -0.370926 | -0.875811 |
| -2.240633 | 0.5118154 | -1.492718 | -2.749837 | -0.075401 | -0.460175 | -3.769495 | -0.219923 | -1.238188 |
| -1.424603 | -0.693770 | -0.856836 | -3.012556 | -0.150168 | 0.1620505 | 0.0712213 | -0.299211 | -0.484709 |
| 0.3288557 | -0.026490 | -0.515498 | -1.198949 | -0.024850 | -0.331653 | -0.130190 | -0.115990 | -0.331971 |
| 0.2364187 | -0.243657 | 0.0461329 | 0.9992803 | -0.438786 | -0.444733 | -0.470603 | -0.489305 | -0.465178 |
| -1.537701 | -0.161907 | -1.776804 | -1.900419 | 0.0174970 | -0.103485 | -1.688457 | -0.219641 | -0.694941 |
| 0.2369049 | -0.140172 | 0.1317496 | -0.970022 | -0.923186 | -0.799982 | -0.933356 | -0.992836 | -0.547392 |
| -2.034039 | -0.233052 | -0.569262 | -0.987149 | -0.686981 | -1.618487 | -1.974747 | -1.144593 | -1.626632 |
| -0.817821 | -0.514505 | -0.621857 | -1.576816 | -0.260051 | -0.138177 | -0.710626 | -0.386579 | -0.723837 |
| -0.990088 | -0.459034 | -0.094877 | -1.988352 | -0.782375 | -1.222517 | -0.769409 | -0.910330 | -0.703237 |
| 0.2290798 | 0.0641321 | -0.123267 | -2.061220 | 0.3882993 | 0.1818172 | -0.595467 | 0.3614393 | 0.1780733 |
| -1.539567 | -0.354013 | -0.620902 | -3.976333 | -0.286871 | -0.503466 | -1.756410 | -0.336690 | -0.510432 |
| -1.905949 | -0.659117 | -1.040383 | -1.985703 | -0.570539 | -0.713970 | -0.733907 | -0.799080 | -1.406871 |
| -1.173186 | -0.549836 | -0.945568 | -2.325324 | -0.327016 | -0.219713 | -0.204332 | -0.329458 | -0.450160 |
| -0.236982 | -0.156523 | -0.298908 | -2.752955 | -0.341320 | -0.287347 | -0.882951 | -0.353925 | -0.344430 |
| -0.222377 | -0.540522 | -0.697803 | -0.476534 | -0.571527 | -0.772818 | -0.398234 | -0.976540 | -0.713942 |
| -1.530792 | -0.058814 | -1.031147 | -1.605709 | -0.234833 | -0.994187 | -0.802449 | -0.163692 | -0.793784 |
| 0.6186515 | -0.362737 | -0.222657 | -2.044282 | -0.854604 | 0.2357823 | 0.9845355 | -0.687934 | 0.7175591 |
| -0.192169 | -0.069340 | -0.049541 | -1.834017 | 0.0255667 | -0.080140 | -0.509704 | 0.0274280 | 0.0472992 |
| -0.117243 | 0.0070810 | -0.172518 | -1.999734 | -0.137055 | -0.029088 | -0.410705 | -0.250118 | -0.219557 |
| 0.0093774 | -0.575914 | -0.383236 | -2.461466 | -0.161834 | -0.431556 | -0.769681 | -0.126302 | -0.566742 |
| -0.391231 | 0.9584647 | 0.2722820 | -0.555355 | 0.4411479 | 0.3679735 | -2.688727 | 0.4626388 | 0.0093199 |
| -0.065972 | 0.0198890 | 0.3289549 | -1.399610 | -0.293845 | 0.4786037 | 0.7003352 | 0.0532329 | 0.9505613 |
| -0.466179 | -0.407776 | -0.274827 | -1.872406 | -0.147862 | -0.456790 | -0.654607 | -0.219554 | -0.385985 |
| -0.875165 | -0.815371 | -0.210467 | -0.837855 | -0.340939 | 0.2551323 | 0.5037456 | -0.360341 | 0.6248027 |
| -2.550099 | -0.454564 | -1.402000 | -2.329904 | -0.194697 | -0.836224 | -2.505331 | -0.399706 | -1.460093 |
| -1.974192 | -1.065461 | -1.387347 | -1.951288 | -1.026414 | -0.726524 | -1.187813 | -1.086172 | -0.795970 |

| | | | | | | | | |
|---|---|---|---|---|---|---|---|---|
| 0.7465706 | -0.1074949 | 0.1863009 | -1.2894689 | -0.1538927 | 0.1328675 | -0.0212261 | -0.1811660 | 0.1174544 |
| -1.9531305 | -1.2707049 | -1.7330960 | -1.7953356 | -0.9365696 | -0.9402697 | -1.3873801 | -1.0244165 | -1.3449122 |
| -0.0128871 | -0.3953941 | -0.0832970 | -1.1787842 | -0.4822178 | -0.4413072 | 0.2172418 | -0.5172911 | -0.2175069 |
| -0.5193238 | 0.1611045 | -0.1711447 | 0.3963844 | 0.0358717 | 1.4207143 | 1.7528759 | 0.2213083 | 1.9127644 |
| -1.1337178 | -0.3527461 | -1.2676841 | -1.4805643 | -0.3882422 | -0.8535855 | -1.4761183 | -0.3344110 | -0.9628656 |
| 0.9299356 | 0.3667155 | 1.0087096 | 1.3780825 | 0.2007229 | 1.7807636 | 2.2206245 | 0.8006391 | 1.7433959 |
| -0.5728726 | -0.5633490 | -0.7850761 | -2.4144123 | -0.3841776 | -0.4703692 | -0.8603488 | -0.4936134 | -0.6384100 |
| -1.8489568 | -0.4216632 | -0.8300267 | -1.7944496 | -0.4521346 | -0.6712266 | 0.0245881 | -0.7865101 | -0.7042070 |
| -1.4221683 | -0.6979779 | -1.3389498 | -1.7626403 | -0.5997247 | -0.8195505 | -2.3832653 | -0.5061624 | -1.6921383 |
| -0.3936432 | -0.7851546 | -0.9121252 | -0.4796373 | -0.4730407 | -0.4880956 | -0.2153649 | -0.5413621 | -0.4204670 |
| 0.1464645 | -0.0657715 | 0.1868317 | -0.7038024 | -0.4069336 | -0.3219968 | 0.4179581 | -0.4008741 | -0.2501637 |
| -1.4518895 | -0.6776042 | -0.8543610 | -1.4221140 | -0.6120729 | -0.7051295 | -1.4705733 | -0.8749082 | -1.2319246 |
| -1.1401898 | 0.0365942 | -0.6925934 | -1.4006450 | 0.0289269 | -0.3659394 | -0.9664598 | -0.0734504 | -0.6948056 |
| -0.4120123 | -0.0281438 | 0.2028920 | 0.5176877 | -0.3422859 | -0.2897429 | -0.3613344 | -0.4455996 | -0.7021936 |
| -3.5221837 | -0.0108673 | -0.356617 | -3.1831199 | -0.4482937 | -0.3171575 | -0.2455979 | -0.3808368 | -0.3430492 |
| -2.4447633 | 0.0717601 | -0.4155288 | -3.5128078 | 0.6904615 | 0.3564654 | -1.4636464 | 0.7047427 | 0.3112451 |
| -0.0626799 | -0.3844171 | -0.4372497 | 1.1644947 | -0.2180970 | -0.5937497 | -0.3429879 | -0.1613115 | -0.5418610 |
| -0.7108362 | -0.3662869 | -0.8947651 | -2.5408011 | -0.4476851 | -0.9786116 | -1.2451962 | -1.1495640 | -1.5387575 |
| -0.5981754 | -0.1878698 | -0.1168957 | -1.0240859 | -0.2086676 | -0.8390547 | 0.2788137 | -0.7842305 | -0.3789764 |
| -1.4339021 | -0.3218990 | -0.9087586 | -3.1309000 | -0.5640560 | -0.6593030 | -1.3635229 | -0.9762301 | -1.5448390 |
| -0.1304117 | -0.1833063 | -0.0846739 | 0.9121828 | -0.1500490 | 0.1654045 | 0.1176245 | -0.0650709 | 0.1119888 |
| -0.0915796 | -0.3913230 | -0.1997670 | -0.9189082 | -0.3785436 | 0.0748162 | -0.3426477 | -0.3205025 | -0.0530104 |
| -2.7271223 | -0.5621981 | -1.5341990 | -2.2431413 | -0.6600510 | -0.7568089 | -1.4833198 | -0.8314506 | -1.3952582 |
| -0.1957394 | -0.1160854 | -0.3895346 | -0.8089544 | -0.4002038 | -0.6561138 | -0.2067707 | -0.4152523 | -0.4749033 |
| -1.6708757 | -0.4149548 | -1.0219319 | -1.6750327 | -0.3947430 | -0.5605592 | -0.6367475 | -0.6410761 | -0.5059387 |
| -0.8821984 | -0.3354584 | -0.9381247 | -1.9532516 | -0.4414984 | -0.7029084 | -0.5822696 | -0.7021142 | -0.7889130 |
| -1.6422370 | -0.6549596 | -0.9261723 | -2.6975335 | -0.6958993 | -1.1386054 | -0.4872530 | -0.9888084 | -1.1726627 |
| -1.7296283 | -0.5036798 | -0.7959330 | -1.7303036 | -0.3155401 | -0.7004840 | -0.1784796 | -0.7119414 | -0.9245508 |
| -1.3720944 | -0.5235136 | -1.0904561 | -1.7158945 | -0.5404425 | -0.3350663 | -0.0970837 | -0.8028780 | -0.6202135 |
| 1.3680944 | 0.2522279 | 0.9089755 | 0.1146616 | -0.1496483 | 0.5108425 | 1.0221681 | -0.0614152 | 0.7072805 |
| 2.5614806 | -0.3125894 | 1.4553897 | 2.5221335 | 0.0151452 | 0.9956539 | 3.1344616 | 0.7651714 | 1.9574639 |
| -1.3407422 | -0.7489139 | -1.0581305 | -0.8424377 | -0.7883312 | -0.9039525 | -0.3783950 | -0.8508684 | -1.2132739 |
| -0.0527757 | -0.5593791 | -0.5406489 | -1.8207625 | -0.1193913 | -0.0831157 | -0.0991641 | -0.2652607 | -0.1713494 |
| 0.6671756 | -0.1393603 | -0.0174217 | -0.7502056 | 0.1883829 | 0.1134926 | 0.0962621 | 0.2683018 | 0.1809007 |
| 1.5194256 | 0.5632598 | 0.9821885 | 1.5578938 | 0.4437388 | 0.4283798 | 0.7231358 | 0.4881659 | 0.5243108 |
| -0.2377024 | -0.2495619 | -0.5619868 | -3.5709913 | -1.0075386 | -0.7737291 | -0.7077538 | -0.8695401 | -0.4732434 |
| 0.1895796 | -0.0612531 | 0.2917017 | -0.9047766 | 0.1623078 | 0.2209272 | 0.4035758 | 0.1043081 | 1.3263734 |
| -0.0872549 | -0.3790683 | 0.1054805 | -1.5699281 | -0.4169695 | -0.1501233 | 0.0473422 | -0.2601981 | 0.4204261 |
| -0.8769741 | -0.9873763 | -0.6695250 | -1.9753453 | -0.3875491 | -0.3947949 | -0.0655275 | -0.6028390 | -0.1743049 |
| -1.3844841 | -0.5790636 | -0.8648455 | -3.0991249 | -0.0994455 | -0.3455883 | -1.2909095 | -0.2564434 | -0.4524696 |
| -1.1179271 | -0.2446125 | -0.7568698 | -0.7498602 | -0.7192940 | -1.0771374 | -1.5883824 | -0.7771132 | -1.4043778 |
| 0.8585883 | 0.0475107 | 0.5012215 | 0.4015127 | -0.3575967 | 0.1407184 | 0.6959814 | -0.2992234 | 0.2871518 |
| 1.1727715 | -0.1373398 | 0.8526833 | -0.2527971 | -0.3841433 | 0.4401702 | 1.1802429 | -0.1832961 | 0.5986104 |
| 0.5460806 | -0.2420522 | 0.1634133 | -0.4588641 | -0.2723759 | 0.0916778 | 0.7131229 | -0.2403736 | 0.2570763 |
| -1.7443439 | -0.5943042 | -1.3048224 | -1.3017163 | -0.2632002 | -0.4145776 | -0.5410180 | -0.2800027 | -0.8669664 |
| -0.2588511 | -0.6788476 | -0.5512059 | -1.4114279 | -0.1403298 | -0.0686740 | -0.2589380 | -0.1930898 | 0.0698152 |
| -2.2830993 | -0.5427001 | -1.6664906 | -2.5997061 | -0.2473546 | -0.4916698 | -1.1912254 | -0.2998592 | -0.5865921 |
| -1.8843017 | -0.5610109 | -1.7644355 | -2.2657519 | -0.4744967 | -0.9790996 | -1.6018753 | -0.5477671 | -1.3645853 |
| -2.7068238 | -0.3501948 | -1.8337895 | -2.4519704 | 0.0196701 | -0.1173799 | -1.9125230 | -0.0775752 | -0.5388689 |
| -0.3120977 | -0.1912647 | -0.3480103 | -3.2442953 | -0.0809749 | -0.1798655 | 0.1687497 | -0.0650023 | -0.1178536 |

| | | | | | | | | |
|---|---|---|---|---|---|---|---|---|
| -0.612248 | -0.124467 | -0.644873 | -3.007322 | -0.249480 | 0.0162093 | 0.6749213 | -0.416707 | 0.3370143 |
| -0.741569 | -1.048664 | -0.787691 | -0.820781 | -0.903019 | -0.428031 | -1.066678 | -0.990615 | -0.916050 |
| -0.341204 | -0.519212 | 0.2343103 | -1.266081 | -0.672474 | -0.687376 | -1.020042 | -0.482919 | -0.190513 |
| -2.736226 | -0.595598 | -1.621138 | -2.340378 | -0.762524 | -1.413495 | -1.282489 | -0.879943 | -1.117357 |
| -1.829976 | -0.696553 | -1.174941 | -1.991933 | -0.333078 | -0.779823 | -0.638971 | -0.738819 | -1.071107 |
| -1.661306 | -0.455194 | -0.526902 | -2.398983 | -0.337750 | 0.0402507 | -0.352900 | -0.307274 | -0.385836 |
| 1.5061652 | 0.1608144 | 1.1676596 | 0.9017892 | 0.2360954 | 1.4571066 | 2.4032846 | 0.4925483 | 1.6526841 |
| -0.483265 | -0.184728 | -0.497836 | 0.3859444 | -0.402537 | -0.620042 | 0.0652955 | -0.312815 | -0.452873 |
| -0.989283 | 0.2644067 | -0.087052 | -4.187583 | 0.1315324 | -0.048870 | -0.738984 | 0.1368885 | -0.212262 |
| -0.304214 | -0.043935 | -0.325268 | -0.454556 | -0.244652 | -0.269764 | 0.0512953 | -0.309325 | -0.224329 |
| -0.525163 | -0.275455 | -0.877683 | -2.905711 | -0.243287 | -0.675247 | 0.0185773 | -0.150477 | -0.231114 |
| -0.899384 | -1.081620 | -0.659929 | -2.576958 | -0.854696 | -1.041161 | -0.488101 | -1.217364 | -0.895222 |
| 0.1154927 | -0.464044 | -0.034978 | -0.596841 | -0.355824 | -0.398594 | -0.929554 | 0.1108597 | -0.208145 |
| -0.795214 | -0.213026 | -0.708346 | -2.522113 | -0.134906 | -0.119515 | -0.898725 | -0.355045 | -0.546205 |
| -2.121944 | -0.197396 | -1.077873 | -2.714577 | -0.579866 | -0.406543 | -2.886625 | -0.490342 | -1.034668 |
| -1.060967 | -0.204557 | -1.064666 | -2.450286 | -0.191278 | -0.605532 | -0.762805 | -0.349522 | -0.755494 |
| -1.458831 | -0.338217 | -0.650557 | -5.499648 | -0.229147 | -0.069333 | -0.997277 | -0.217578 | -0.251982 |
| -0.795090 | -0.301401 | -0.886667 | -1.602975 | -0.433125 | -0.716466 | -1.008273 | -0.538595 | -0.747226 |
| -2.619155 | -0.498398 | -1.734388 | -2.609554 | -0.235658 | -0.533851 | -1.183594 | -0.620453 | -1.141747 |
| 0.1036329 | 0.0145267 | -0.006791 | -2.781582 | -0.217754 | 0.0118598 | -0.424333 | -0.140779 | 0.3142915 |
| -1.019344 | -0.238928 | -0.316178 | -1.255996 | -1.031403 | -0.817617 | -1.463181 | -1.269671 | -0.485452 |
| -1.962929 | 0.6564305 | -0.172963 | -1.291718 | -0.047244 | -0.207019 | -0.994549 | -0.271013 | -0.444955 |
| -2.511424 | -0.344063 | -1.814034 | -2.370698 | -0.369633 | -1.019813 | -3.557017 | -0.381695 | -1.481958 |
| 0.2688204 | -0.063605 | -0.047604 | -0.552679 | -0.300724 | -0.076076 | 0.2690054 | -0.293246 | -0.304542 |
| -0.541548 | 0.2129068 | -0.081062 | -0.631353 | -0.122948 | -0.142168 | -1.096184 | -0.343519 | -0.269683 |
| 0.0815946 | -0.253156 | -0.163880 | 0.1513616 | -0.362031 | -0.474790 | -0.427517 | -0.608978 | -0.385921 |
| -0.930036 | -0.150199 | -0.270015 | -1.450968 | -0.113618 | -0.108395 | -1.337422 | -0.063417 | -0.405500 |
| -1.290201 | -0.433931 | -0.716305 | -2.741488 | -0.481297 | -0.755711 | -0.705467 | -0.491483 | -1.267072 |
| -0.017101 | -0.064902 | 0.0959974 | -0.135969 | -0.292312 | 0.1912403 | 0.7729319 | -0.232536 | 0.1441046 |
| 1.0481064 | -0.260931 | 0.4861370 | -1.345946 | -0.027112 | -0.182239 | -0.100288 | -0.112142 | 0.0395166 |
| -2.325405 | -0.516414 | -0.527918 | -1.193704 | -0.603220 | -0.182795 | -0.364354 | -0.618616 | -0.295173 |
| -0.425810 | -0.132505 | -0.390756 | -1.736752 | -0.253104 | -0.261963 | -0.878590 | -0.244784 | -0.412837 |
| 1.9839979 | -0.364257 | 0.4199207 | 2.3919696 | -0.349350 | -0.329145 | 1.6947308 | -0.316622 | 0.2326064 |
| -2.260834 | -0.366970 | -1.039971 | -2.939709 | -0.271278 | -0.382040 | -0.948818 | -0.434241 | -0.878912 |
| -0.630704 | -0.291782 | -0.133055 | -1.092421 | -0.012011 | -0.265496 | -0.209465 | -0.207873 | 0.1545841 |
| -0.938060 | -0.221182 | -0.953872 | -2.554148 | -0.194396 | -0.547549 | -1.256262 | -0.410433 | -0.986897 |
| -2.406468 | -0.732862 | -2.250143 | -1.909519 | -0.345508 | -1.456457 | -2.109483 | -0.984164 | -1.851113 |
| -2.523818 | 0.9413828 | -0.655242 | -2.656331 | -0.194456 | -0.097461 | -0.158354 | -0.234801 | -0.104612 |
| 1.1742932 | -0.160845 | 1.0836821 | 0.5492017 | -0.087668 | 0.1739277 | 0.7818383 | -0.130953 | 0.2747497 |
| -0.423374 | -0.246986 | -0.554533 | -0.666417 | -0.377675 | -0.231604 | -0.595791 | -0.483428 | -0.417951 |
| 0.1373499 | -0.088704 | -0.257330 | -4.160608 | -0.041812 | 0.1446558 | 0.4155412 | -0.026642 | 0.4984019 |
| -1.364210 | -0.518851 | -1.185070 | -4.040388 | -0.274928 | -0.893312 | -2.003886 | -0.427229 | -0.977037 |
| -0.511737 | -0.729811 | -0.385687 | -0.841365 | -0.730337 | -0.678382 | -0.789267 | -0.774081 | -0.771808 |
| -0.373431 | 0.3700069 | -0.113443 | -0.932333 | 0.1483207 | -0.211995 | 0.1533093 | 0.0231655 | -0.279554 |
| -0.659230 | -0.631893 | -0.555752 | -1.102702 | -0.247481 | -0.082421 | -0.222929 | -0.295959 | -0.194625 |
| -1.953554 | -0.597133 | -1.743099 | -4.160722 | -0.447362 | -0.575964 | -1.484390 | -0.762491 | -0.977785 |
| -0.935261 | 0.0454168 | -0.383013 | -1.915275 | -0.364031 | -0.242007 | -0.500582 | -0.438277 | -0.491925 |
| -1.599430 | -0.859728 | -0.609327 | -2.105900 | -1.019329 | -1.374458 | -0.915766 | -1.529718 | -0.656153 |
| -0.172910 | -0.500803 | -0.676305 | -1.115066 | -0.623213 | -0.436828 | -0.401671 | -0.790479 | -0.434193 |
| -1.203290 | -0.832179 | -1.062655 | -1.398316 | -0.442467 | -1.059753 | -1.126341 | -0.662907 | -1.330274 |

| | | | | | | | | |
|---|---|---|---|---|---|---|---|---|
| -1.0573772 | -1.2115933 | -1.0776134 | -1.1905416 | -0.4315678 | -0.5345512 | -0.2796152 | -0.5138004 | -0.3478202 |
| 0.6443118 | -0.0244440 | 0.0740139 | 0.9048170 | -0.4276734 | -0.2495164 | 0.1432203 | -0.1582612 | 0.4513768 |
| 0.1992922 | -0.1560364 | -0.0334158 | -0.7061213 | -0.2743675 | -0.2622669 | -0.3367711 | -0.2865909 | -0.4922711 |

| PG_622 | PG_623 | PG_624 | PG_625 | PG_626 | PG_627 | PG_628 | PG_629 | PG_630 |
|---|---|---|---|---|---|---|---|---|
| -0.7573694 | -0.4487511 | -0.6147073 | -1.1745814 | -0.2218731 | -0.7268185 | -0.7172306 | -0.8746070 | -0.9756814 |
| -1.1295025 | -0.3477182 | -0.8562933 | -1.3502830 | -0.5126141 | -0.5048121 | -1.3553164 | -0.6249293 | -1.6434896 |
| -2.7187524 | -0.8005249 | -0.4369645 | -1.0223960 | -0.7320583 | -0.3463242 | -1.0929335 | -0.7844131 | -0.7974209 |
| -0.2879824 | -0.1306668 | -0.2896727 | 0.1535816 | -0.2768708 | -0.4502605 | -0.0405184 | -0.1205058 | -0.2075394 |
| -0.5635136 | -0.3383314 | -0.2624570 | -0.9100104 | -0.1658144 | -0.3945977 | -0.5889527 | -0.1639840 | -0.3651348 |
| -2.3548920 | -0.2276761 | -0.7838320 | -2.2704793 | -0.5429001 | -1.2218302 | -2.4155462 | -0.3422678 | -0.9260188 |
| -0.6086490 | -0.2435532 | -1.3310476 | -0.3035120 | -0.3686125 | -1.5811612 | -0.4536162 | -0.4712250 | -0.9804716 |
| -1.4758519 | -0.0697240 | -0.2504339 | -1.5502825 | -0.1204157 | -0.5413108 | -1.8914594 | -0.4453454 | -0.3997115 |
| -0.5287642 | -0.6051457 | -0.2162328 | 0.0673971 | -0.5526055 | -0.1179262 | 0.4328451 | -0.4721148 | 0.1240992 |
| -2.2346307 | -0.4060516 | -0.7818919 | -0.6602271 | -0.3408990 | -0.6495050 | -0.5276715 | -0.3643672 | -0.9112394 |
| -0.8831413 | -0.1724197 | -0.2250075 | -0.6702446 | -0.0228928 | -0.4620558 | -0.7167346 | -0.1363805 | -0.1793750 |
| -0.4050228 | -0.1069913 | -0.3215832 | -0.3078021 | -0.1549635 | -0.6081324 | -0.4533817 | -0.1909200 | -0.2441970 |
| -1.6051371 | -0.6080625 | -0.9260362 | -4.0121389 | -0.6570400 | -1.3239663 | -3.6369490 | -0.1335549 | -0.7463401 |
| 4.8177232 | -0.3408844 | 2.2860870 | 4.2537534 | -0.0859621 | 4.5524029 | 4.7235294 | -0.3371275 | 1.2946638 |
| 0.6009864 | -0.1943398 | 0.0878563 | 0.2174175 | 0.0445262 | 0.0416260 | 0.6054376 | -0.1680130 | -0.0745255 |
| 2.2511909 | -0.6787537 | 2.4800145 | 3.6789605 | -0.2286591 | 3.3347608 | 3.9570379 | -0.2219314 | 1.9393407 |
| -1.6625306 | -0.7686081 | -1.7908096 | -2.2506378 | -0.8724222 | -1.9304071 | -2.3126403 | -1.1291863 | -2.1906004 |
| -1.8119352 | -0.4037482 | -0.4536793 | 0.1810609 | -0.5422810 | -0.1590559 | 0.1504292 | -0.0047855 | 0.0243068 |
| -1.2237741 | 0.0203070 | -1.0195861 | -3.0066752 | -0.5627831 | -1.8177350 | -2.8445850 | -0.6846707 | -1.4624295 |
| -0.6453177 | -0.6684051 | -0.7350628 | -1.0771859 | -0.7520980 | -0.5893327 | -0.6695103 | -0.6954944 | -0.6100549 |
| -0.1273901 | -0.5016492 | 0.5087178 | 0.3953612 | -0.1290339 | 0.5461475 | 0.3634681 | -0.3826239 | 0.5333371 |
| -0.9970728 | -1.0237792 | -0.4788342 | -0.3639074 | -1.0774413 | -0.5309978 | -0.2308993 | -0.5267848 | -0.4008343 |
| 1.7084949 | -0.2530671 | 0.6557156 | 1.2240340 | -0.3057384 | 0.4935019 | 1.0156738 | -0.1745940 | 0.8742137 |
| -1.4921334 | -0.1504800 | -0.6435750 | -1.9080756 | -0.4020930 | -1.3864914 | -2.1697333 | -0.4123786 | -0.5058858 |
| -2.1127099 | -0.2169632 | -0.8034870 | -2.6641800 | -0.1923571 | -1.6362127 | -2.5252225 | -0.0990650 | -0.5493956 |
| 0.8219510 | -0.3208097 | 0.5317733 | 1.0743912 | -0.0816380 | 0.6254214 | 0.8236997 | -0.0951371 | 0.6658231 |
| -0.1608304 | 0.0121976 | -0.0465274 | -0.2211613 | 0.0795192 | -0.0452340 | -0.0892929 | 0.2511211 | -0.0260465 |
| -1.7136096 | -0.7515459 | -1.1064958 | -2.0538376 | -0.7473369 | -1.2987227 | -2.4068629 | -1.0235501 | -0.8560816 |
| -2.7234343 | -0.4643133 | -1.3402240 | -1.5949550 | -0.5274638 | -1.9856679 | -2.4049375 | 0.2867806 | -0.3972649 |
| 0.2097348 | -0.1076425 | -0.1772846 | -0.1564524 | -0.0317247 | -0.3851059 | -0.2212720 | 0.0257933 | -0.0207058 |
| -2.9874067 | -0.7435795 | -0.1546687 | -0.1504683 | -0.8081183 | -0.0505629 | -0.0496977 | -0.5531133 | -0.5878248 |
| -0.8561607 | 0.0916755 | 0.1801246 | -0.8847482 | 0.0067683 | 0.0448971 | -0.4906490 | 0.0907816 | 0.2059548 |
| -0.0885132 | -0.3047731 | -0.0230745 | -2.5902528 | -0.1904011 | 0.6473024 | -1.2703118 | -0.2175712 | -0.0999119 |
| -2.9998378 | -0.5609354 | -0.7225603 | -3.4455352 | -0.5813326 | -1.0236618 | -3.0536127 | -0.0124838 | -0.9068342 |
| -0.4753710 | -0.3745018 | -0.1571312 | -0.0306609 | -0.4432513 | -0.1989263 | -0.0581190 | -0.1863128 | -0.1228480 |
| -0.1639691 | -0.0155369 | -0.7639414 | 0.0357305 | -0.5942358 | -0.8823292 | -0.0915188 | -0.3052985 | -0.7605762 |
| -2.3284359 | -0.1899379 | -0.6408797 | -1.9636439 | -0.2231282 | -0.9111535 | -1.7710026 | 0.2561968 | -0.5141870 |
| 0.1686797 | -0.3696873 | 1.5884298 | -0.1316772 | -0.2927503 | 3.5187016 | -0.0601230 | -0.0345740 | 1.2843093 |
| -0.1531363 | -0.0500753 | 0.3621262 | -0.9120754 | 0.1635679 | 0.0161751 | -0.6240292 | 0.0507158 | -0.3149093 |
| 1.2258846 | -0.6056101 | -1.8816505 | 0.9302244 | -1.5545161 | -1.7351178 | 0.2555164 | -0.9693020 | -0.5448155 |
| 0.7383476 | -0.2097548 | -0.0748968 | 0.3727595 | -0.2131218 | 0.0408935 | 0.2913280 | 0.0171672 | 0.3681934 |
| -1.9301546 | -0.4549445 | -0.8048184 | -1.7790165 | -0.4892880 | -1.1071035 | -1.6508776 | -0.4055205 | -0.7495424 |
| -0.8370575 | -0.4762112 | -0.5858136 | -0.1386469 | -0.3696165 | -0.5541300 | -0.2661350 | -0.5879023 | -0.7337388 |
| -1.0113941 | -0.0685812 | -0.0813696 | -0.7891734 | -0.0920198 | -0.4071528 | -1.0681476 | -0.0221006 | -0.1944412 |
| 1.5494383 | -0.1889508 | 0.6005600 | 1.5511167 | -0.0999559 | 0.2205658 | 1.2278239 | -0.0867334 | 0.7709413 |
| 5.6287827 | 0.2353094 | 0.3183934 | 2.4495261 | 0.5046146 | 0.7701610 | 3.1120758 | 0.2384885 | 0.3376007 |
| -0.1408354 | -0.1405864 | -0.3904251 | -0.4485391 | -0.1159585 | -0.4179075 | -0.5034738 | -0.0630338 | -0.2432520 |

| | | | | | | | | |
|---|---|---|---|---|---|---|---|---|
| 0.2065069 | -0.3733608 | -0.2710820 | 0.3172027 | -0.2495315 | -0.2989361 | 0.1702300 | -0.2256813 | -0.3665695 |
| -1.0008702 | -0.2615902 | -0.7177993 | -2.4250057 | -0.4236025 | -0.9998691 | -2.5391194 | -0.1481925 | -0.3640994 |
| 0.2964013 | -0.6491981 | 0.1512722 | 0.6443087 | -0.4149240 | 0.3431771 | 0.3745483 | -0.3522358 | -0.2038686 |
| 2.2530724 | -0.6385958 | -0.1326348 | -0.5885199 | -0.2151719 | -0.3802822 | -0.4716075 | -0.5123846 | -0.4943471 |
| -2.3369286 | -0.8464307 | -1.6964128 | -1.0683008 | -1.3649305 | -1.5598446 | -1.3586328 | -0.3810779 | -0.8934752 |
| -0.0453333 | -0.2245180 | -0.1387284 | -0.2734070 | -0.2504779 | -0.2401646 | -0.2598513 | -0.2594818 | -0.2235747 |
| -1.6043540 | -0.3335866 | -1.0330783 | -1.2652466 | -0.7116700 | -0.8877229 | -1.0967659 | -0.4109804 | -0.9878598 |
| 2.1839933 | -0.6484927 | 1.7128110 | 2.7542723 | -0.2846125 | 2.5066936 | 2.3628134 | -0.1779710 | 1.2054914 |
| 0.4181115 | -0.7318026 | 1.0125363 | 0.2448794 | -1.1308303 | 1.8422253 | -0.0939712 | -1.0202214 | 0.2045734 |
| 0.2279035 | -0.7058453 | -0.4076871 | 0.2182349 | -0.7621287 | -0.5395897 | -0.1281135 | -1.2249305 | -0.2511017 |
| -1.3444730 | -0.6959823 | -1.3138066 | -1.4472026 | -0.8011505 | -1.7310229 | -1.2513909 | -1.1296983 | -1.8300742 |
| 2.6203760 | -0.0184992 | 3.1014080 | 3.4651511 | 1.1430533 | 3.5781347 | 3.6521827 | 0.2908489 | 2.7199656 |
| 1.3406772 | -0.2217662 | 2.6773997 | 2.9120623 | 0.4507531 | 3.0041332 | 2.7779531 | 0.1801346 | 3.3352866 |
| -0.7506427 | -0.4839654 | -0.6100007 | -1.0134101 | -0.4979399 | -0.5480832 | -0.9236162 | -0.2756275 | -0.3026624 |
| -1.8745766 | -0.7378304 | -1.8112881 | -1.9662123 | -0.8743169 | -1.8359587 | -1.8769570 | -1.1936511 | -1.4982814 |
| -0.1642854 | -0.5873407 | -0.6094351 | 0.1084214 | -0.9118157 | -0.2173794 | 0.4134914 | -0.4468790 | -0.5877130 |
| -1.5070889 | 0.0544372 | -0.3203243 | -1.6348446 | 0.0150854 | -0.8278376 | -1.5140963 | 0.0132918 | -0.1612459 |
| -0.6205528 | -0.4022308 | -0.5555312 | -0.4143102 | -0.3780143 | -0.7498690 | -0.4838633 | -0.1021782 | -0.5344370 |
| -0.8003166 | -0.2663533 | -0.6110090 | -0.3231721 | -0.1997156 | -0.7621592 | -0.4780904 | -0.1785967 | -0.4904336 |
| -1.1214896 | -0.4054061 | -0.1587945 | -0.0944463 | -0.1410472 | -0.1310611 | 0.2481967 | 0.0315120 | -0.3908773 |
| -0.4908419 | -0.3269853 | -0.2287345 | -0.5079134 | -0.1268618 | -0.5425412 | -0.0638689 | -0.0215930 | -0.1000465 |
| -1.5160266 | -0.5880656 | -0.8849756 | -2.4300701 | -0.6523079 | -0.7024871 | -2.5738427 | -0.5637569 | -0.6486926 |
| 0.1391273 | -0.0300970 | 0.1073910 | 0.7631051 | -0.1977232 | 0.0402496 | 0.7386590 | -0.0302952 | -0.2950531 |
| 0.5813396 | 0.1301443 | 0.6553570 | 1.0140767 | 0.2993953 | 0.4809329 | 0.6860323 | 0.7010651 | 0.7809225 |
| -1.4049029 | -0.4552755 | -0.9582657 | -1.6181824 | -0.4950130 | -1.0452841 | -1.6057146 | -0.5022612 | -0.8865374 |
| -0.5806398 | -0.2341058 | -0.4982323 | -0.2375303 | -0.3459286 | -0.9790129 | -0.3289785 | -0.4486254 | -0.5769955 |
| -2.8978286 | -0.4444044 | -0.6497402 | -0.4124229 | -0.6573559 | -0.7660540 | -0.1952630 | -0.5288663 | -0.8913066 |
| 3.7362865 | 0.3316419 | 1.8083816 | 3.7472307 | 0.8546507 | 1.6733582 | 3.2493598 | 0.6374966 | 2.4990114 |
| 2.8143386 | 0.0607274 | 0.8482026 | 2.5301598 | 0.1666390 | 1.2181859 | 2.1649771 | 0.2900338 | 0.9825053 |
| -1.9030866 | -0.3247106 | -0.9016860 | -0.9976176 | -0.4190536 | -1.0727594 | -0.9222506 | -0.1930936 | -0.2131348 |
| -0.8315099 | 0.1881884 | -0.1529706 | 0.2124747 | -0.1573099 | 0.0664956 | 0.2355100 | -0.0663804 | 0.0829957 |
| -0.0372254 | 0.0354760 | 0.1264435 | 0.3655750 | 0.0380853 | -0.0644583 | 0.2009925 | -0.0039989 | -0.0729702 |
| -1.0414143 | -0.5805000 | -1.1117223 | -0.9685471 | -0.5563313 | -1.4962243 | -1.0953630 | -0.5427999 | -0.8752167 |
| -1.3540003 | -0.2195058 | -0.5198424 | -0.7567132 | -0.4473367 | -0.8302357 | -1.1202808 | -0.5382856 | -0.3632314 |
| 1.7516259 | -0.5451880 | 0.7420945 | 1.5054101 | -0.1295330 | 0.9188830 | 1.3776137 | -0.1383688 | 1.1665293 |
| -0.4422627 | -0.1276283 | -0.7744330 | -1.0760451 | -0.3390995 | -1.0543899 | -1.2644009 | 0.5050200 | 0.2869754 |
| -0.5240694 | -0.4629948 | -0.5519504 | -0.9102468 | -0.8437886 | -0.2262225 | -0.7740251 | -0.1108122 | -0.2602190 |
| -1.7821677 | -0.2772982 | -0.6367440 | -1.4992163 | -0.5232999 | -1.3302697 | -1.6371603 | -0.3121047 | -0.6066369 |
| -2.1199390 | -0.1990332 | -0.2926747 | -1.7508174 | -0.2689514 | -0.4936726 | -1.8752597 | -0.1302409 | -0.3788994 |
| -2.6610192 | -0.3352294 | -1.2796264 | -1.6630982 | -0.2141570 | -0.8229885 | -1.0339562 | -0.5297227 | -0.8277639 |
| -1.0206661 | -0.5455246 | -0.9655227 | -1.3562541 | -0.7211818 | -0.9710322 | -1.1855666 | -0.6665010 | -0.9710419 |
| -3.2328936 | -0.2442708 | -1.1323398 | -2.2258181 | -0.3192030 | -1.6101011 | -2.0350681 | -0.7172714 | -1.2531055 |
| 2.7444263 | -0.1407697 | 3.9282084 | 4.2739579 | 0.9635633 | 4.5066527 | 3.4522270 | 0.3243607 | 3.9132026 |
| -0.1768303 | 0.0017851 | -0.1039703 | -0.1438159 | -0.0145082 | -0.2651389 | -0.1824025 | 0.0095757 | -0.0129550 |
| -0.6338636 | -0.2660818 | -0.2864137 | -1.0685717 | -0.1212820 | -0.1634300 | -0.8558914 | -0.2326550 | -0.8309161 |
| -1.1081969 | -0.9891415 | -1.2012140 | -1.2823980 | -0.9480247 | -1.3939847 | -1.1833076 | -0.7443405 | -0.9196391 |
| 8.1332516 | -0.0943811 | 0.6951100 | 0.3115761 | -0.1065556 | 2.7883579 | 3.5425189 | -0.2561658 | -0.0708479 |
| 1.4405928 | -0.0399253 | 1.6837770 | 2.1521609 | 0.1806913 | 2.3689685 | 2.3816481 | 0.0295121 | 1.6015968 |
| -3.3819814 | -0.0514923 | 0.1066626 | -1.7546768 | 0.0540494 | -0.3191960 | -0.5632962 | 0.0948536 | -0.3954951 |
| -0.8881167 | -0.2629307 | -0.3417227 | -0.2167467 | -0.1586228 | -0.3384295 | -0.4693785 | -0.5059674 | -0.6526385 |

| | | | | | | | | |
|---|---|---|---|---|---|---|---|---|
| -0.828766 | -0.620294 | -0.588665 | -2.787893 | -0.832948 | -0.849199 | -2.354067 | 0.7173639 | 0.4324206 |
| 0.8636814 | -0.103127 | -0.179876 | 0.5243841 | -0.108304 | -0.300893 | 0.5612550 | 0.1131402 | 0.0220598 |
| 0.4034246 | -0.502655 | -0.370281 | 0.2386727 | -0.480882 | -0.668540 | -0.257794 | -0.560823 | -0.374987 |
| 0.1755057 | -0.096129 | -0.084078 | -0.156483 | -0.058792 | -0.025839 | 0.0316090 | -0.127630 | -0.013168 |
| -1.567685 | -0.257938 | -0.889419 | -1.191006 | -0.281260 | -1.262030 | -1.495197 | -0.284301 | -1.148781 |
| -1.325515 | -0.380925 | -0.408990 | -1.038503 | -0.547118 | -0.210363 | -0.998738 | -0.601196 | -0.057736 |
| -0.041158 | -0.419163 | -0.384666 | -0.220458 | 0.0246192 | -0.292332 | -0.263604 | -0.426561 | -0.100550 |
| -0.257936 | -0.142834 | -0.042815 | -0.310320 | -0.081810 | -0.018596 | -0.012182 | -0.210708 | -0.207544 |
| -0.527454 | -0.229416 | 0.0126337 | -0.318627 | -0.192436 | -0.049025 | -0.161088 | -0.430992 | -0.326524 |
| 1.3253244 | -0.230684 | 0.8949121 | 1.1478672 | -0.161792 | 1.1950601 | 0.9393312 | -0.121698 | 1.0960995 |
| -1.296827 | -0.062423 | -0.019728 | -1.259400 | -0.197328 | -0.068260 | -0.890009 | -0.139255 | 0.0316764 |
| -1.323079 | -0.486566 | -1.034334 | -1.988702 | -0.845284 | -0.718814 | -1.594646 | 0.0622740 | -0.647477 |
| -3.988450 | -0.417963 | -1.090668 | -3.557585 | -0.639972 | -1.453611 | -2.625377 | 0.0233076 | -0.401815 |
| -1.362277 | -0.115725 | -0.828242 | -1.580996 | -0.342502 | -0.964061 | -1.648109 | -0.060071 | -0.722320 |
| -0.492162 | -0.381740 | -0.292967 | -0.321793 | -0.532363 | -0.323679 | -0.463371 | -0.368362 | -0.593113 |
| -0.058117 | -0.105975 | -0.095914 | 0.1523500 | -0.065026 | 0.1412082 | -0.025260 | 0.0708206 | -0.058450 |
| -0.981374 | -0.855095 | -0.998907 | -1.222330 | -1.054681 | -1.747751 | -1.463444 | -1.021218 | -1.232924 |
| 0.0087888 | 0.0803780 | 0.0737542 | -0.196470 | -0.054218 | -0.131347 | -0.140610 | -0.004373 | -0.383671 |
| -0.056018 | -0.017237 | -0.108996 | -0.305816 | 0.0663210 | -0.545100 | -0.251845 | -0.225097 | -0.590057 |
| 1.1881637 | 0.1460815 | 0.2921522 | 1.9968733 | -0.149273 | 0.6037774 | 2.1206755 | -0.387102 | 0.3154188 |
| -1.945613 | -0.150071 | -0.295332 | -0.881111 | -0.143619 | -0.543558 | -1.430660 | -0.298879 | -0.456669 |
| -0.122944 | -0.055795 | -0.292446 | 0.2120966 | -0.133640 | -0.486494 | 0.1118398 | -0.178443 | -0.380478 |
| -1.374390 | -0.524449 | -0.431044 | -0.768362 | -0.655266 | -0.228314 | -0.698271 | 0.4571243 | 0.0755713 |
| -2.120859 | -0.248852 | -0.921192 | -1.740408 | -0.245089 | -0.993683 | -1.763141 | -0.376902 | -1.288955 |
| -2.907852 | -0.948069 | -1.305356 | -0.756972 | -0.679641 | -1.820054 | -0.555584 | -0.637697 | -0.517440 |
| -0.298264 | 0.0132645 | -0.206088 | -0.360093 | -0.143356 | -0.635647 | -0.449155 | 0.0566179 | -0.080772 |
| 0.3539729 | -0.191526 | -0.087357 | 0.1231793 | -0.092135 | -0.219951 | 0.0980210 | 0.1170992 | -0.077383 |
| -0.313291 | -0.359928 | -0.536571 | -0.138385 | -0.519033 | -0.696609 | -0.439295 | -0.368440 | -0.074141 |
| -1.374523 | -0.208301 | -0.799067 | -0.838653 | -0.355204 | -1.404556 | -0.799044 | -0.269215 | -0.607148 |
| -0.649455 | -0.474854 | -1.052501 | -2.322266 | -0.572929 | 0.0849722 | -2.159055 | -0.232883 | -0.422629 |
| -1.600696 | -0.479626 | -1.262376 | -0.969502 | -0.665431 | -1.312106 | -0.701037 | -0.940889 | -1.573274 |
| -1.078877 | -0.239564 | -0.922845 | -1.452024 | -0.293939 | -1.317320 | -1.494608 | -0.491010 | -0.638620 |
| -0.501970 | -0.516159 | -1.904109 | -3.016606 | -0.880019 | -2.499265 | -2.686465 | -0.540938 | -1.872196 |
| -0.449662 | 0.2067623 | 0.2734609 | -0.292033 | 0.3566018 | 0.1726301 | -0.232210 | 0.3711809 | 0.5731108 |
| -1.935578 | -0.134604 | -0.271047 | -1.784077 | -0.096994 | -0.447020 | -1.834254 | -0.315048 | -0.340902 |
| -0.889503 | -0.142631 | -0.370008 | -0.249891 | -0.493788 | -0.740470 | -0.498335 | -0.544728 | -0.406824 |
| -0.591187 | -0.053144 | -0.220889 | -0.391709 | -0.181037 | -0.505519 | -0.408051 | -0.272402 | -0.159591 |
| -0.771565 | -0.062179 | -0.240840 | -1.450834 | 0.0013553 | 0.2321654 | -1.217544 | -0.171468 | -0.476961 |
| -0.655125 | -0.746412 | -0.513048 | -0.111183 | -0.649213 | -0.476278 | -0.080574 | -0.677356 | -0.536580 |
| -0.767310 | -0.727559 | -1.149164 | -2.924764 | -0.670898 | -0.536245 | -2.303809 | -0.179049 | -0.977804 |
| 0.8868754 | -0.291591 | -0.618079 | -0.943121 | -0.616649 | -0.690411 | -1.167871 | -0.821162 | -0.436843 |
| -0.382727 | -0.401028 | -0.134562 | -2.328273 | -0.168848 | -0.029043 | -1.561784 | 0.1757835 | 0.0919231 |
| -0.507853 | -0.008275 | -0.277523 | -0.738855 | 0.0898968 | -0.388882 | -0.698336 | -0.004053 | -0.133918 |
| -0.989255 | -0.023468 | -0.509650 | -0.225301 | -0.042897 | -0.239597 | -0.041664 | 0.2644304 | -0.359152 |
| -2.371035 | -0.634447 | -0.096082 | -0.412163 | -0.503174 | -0.236329 | -0.192444 | 0.2175828 | 0.1846997 |
| 0.7597577 | -0.232862 | -0.100135 | 0.1198463 | -0.227903 | 0.0850515 | 0.0530173 | -0.354115 | -0.148260 |
| -0.510065 | -0.729555 | -1.024581 | -1.006018 | -0.735808 | -1.330779 | -1.311547 | -0.447513 | -0.907462 |
| 0.6532490 | -0.047726 | -0.287533 | 0.6092534 | -0.118398 | -0.085840 | 0.8818503 | -0.366175 | 0.1691512 |
| -2.421640 | -0.382522 | -0.155895 | -0.224202 | -0.606472 | -0.385195 | -0.415789 | 0.0440099 | -1.427023 |
| -1.462005 | -0.562544 | -0.852079 | -1.457986 | -0.749110 | -1.077469 | -2.034728 | -1.061844 | -0.714248 |

| | | | | | | | | |
|---|---|---|---|---|---|---|---|---|
| 0.5167988 | -0.0356732 | 0.1030552 | 0.5144698 | -0.0115284 | 0.0907518 | 0.5262055 | 0.3687867 | 0.2176418 |
| -1.4804714 | -0.1538282 | -1.1989973 | -1.8134638 | -0.6173730 | -1.5144533 | -1.9351908 | -0.5385094 | -1.5859961 |
| 0.0966237 | -0.5050946 | -0.6102369 | -0.5180129 | -0.6296856 | -0.0423872 | -0.1580226 | -0.6213340 | -0.6595545 |
| 1.3000819 | 0.0006713 | 0.7038565 | 1.4373338 | -0.2877409 | 1.4968576 | 1.3569957 | 0.1282881 | 0.6418996 |
| -1.1002371 | -0.2253964 | -1.0074250 | -2.1941368 | -0.1077512 | -1.2104079 | -2.1442137 | -0.0288313 | -1.1626981 |
| 1.5548468 | 0.0942377 | 2.6345879 | 2.2763601 | 1.3188077 | 2.5485579 | 1.7160007 | 0.5584891 | 2.8360282 |
| -0.9047779 | -0.4169228 | -0.2746541 | -0.4849323 | -0.3381578 | -0.3185928 | -0.4677582 | -0.1188962 | -0.3725922 |
| -0.4478860 | -0.6943443 | -0.8887745 | -0.5878266 | -1.0049158 | -1.1856984 | -0.6068274 | -0.6657896 | -0.6968724 |
| -2.4255014 | -0.3669915 | -0.9806162 | -1.5047944 | -0.4407223 | -0.9723212 | -1.3997923 | -0.2747198 | -1.0394876 |
| -0.0630609 | -0.3077850 | -0.5375582 | -0.2888289 | -0.1841836 | -0.8111657 | -0.2718791 | -0.7144355 | -0.8681281 |
| 0.5488333 | -0.4201939 | -0.1884614 | 0.3706362 | -0.2394089 | 0.0690355 | 0.3695395 | -0.4954549 | -0.0937773 |
| -1.3400307 | -0.6231612 | -1.0428440 | -0.6840743 | -1.0701069 | -1.2360752 | -0.6544501 | -0.6115988 | -0.5654778 |
| -0.8125841 | -0.6142809 | -0.6103264 | 1.3214766 | -0.9751203 | -0.6259846 | 1.5660902 | -0.8460505 | -1.1806911 |
| -0.2230784 | -0.3039077 | -0.3124617 | 0.0998446 | -0.7635001 | -0.0207599 | 0.5097280 | -0.3327923 | -0.2397468 |
| -0.1896650 | -0.7689967 | -0.3164072 | -0.6186640 | -1.0049324 | -0.5838325 | -0.5793249 | -0.2603083 | -0.1907985 |
| -0.8635534 | -0.3197962 | -0.2637342 | -2.8252862 | -0.1029501 | -0.3818236 | -2.1050925 | 0.5669543 | 0.2263178 |
| -0.5828861 | -0.4222155 | -0.6016930 | -0.4358909 | -0.3907761 | -0.4749580 | -0.3159146 | -0.9696813 | -0.5768658 |
| -1.8089486 | -0.5002009 | -0.9484877 | -1.1652823 | -0.8560792 | -1.5645640 | -2.2293797 | -0.9659452 | -1.2506162 |
| 0.2309412 | -0.3599222 | -1.0217258 | -0.5455821 | -0.7098215 | -1.3322186 | -0.8120856 | -0.8550512 | -0.6833700 |
| -1.2596871 | -0.3400206 | -0.7803793 | -0.4942086 | -0.7107156 | -1.1634280 | -0.8979144 | -0.4973974 | -0.8190948 |
| 0.5523733 | -0.2867641 | -0.1796890 | 0.2572024 | -0.0813095 | 0.5146204 | 0.2206220 | -0.0986256 | -0.1204869 |
| -0.2262920 | -0.3676470 | 0.1962540 | 0.3799867 | -0.1320217 | 0.0550273 | 0.2068067 | -0.0007512 | 0.2553505 |
| -1.6646043 | -0.4040996 | -1.0359448 | -1.7721247 | -0.4704275 | -1.6535603 | -1.3281115 | -0.5242423 | -0.7793803 |
| -0.1430024 | -0.3557475 | -0.5426835 | -0.2781961 | -0.4123449 | -0.5798647 | -0.5645306 | -0.5126054 | -0.3466176 |
| -0.8132165 | -0.1485588 | -0.8706786 | -1.3125913 | -0.3442953 | -1.3225089 | -1.7189346 | -0.2275099 | -0.8583027 |
| -0.5755841 | -0.3220289 | -0.6014276 | -0.2126927 | -0.3280620 | -0.7231466 | -0.5036432 | -0.4016329 | -0.5017189 |
| -1.0586307 | -0.6858981 | -0.6584402 | -0.9798284 | -0.7267925 | -1.1963594 | -0.9843749 | -0.4802061 | -0.9438796 |
| -0.0618734 | -0.1436581 | -0.3534261 | -0.3386169 | -0.1145690 | -0.6176933 | -0.2262860 | -0.3211533 | -0.6115063 |
| -0.5624291 | -0.1728049 | -0.5487362 | -0.9014017 | -0.3020772 | -0.6850393 | -0.8386008 | -0.3785568 | -1.3473109 |
| 1.1564112 | -0.0569677 | 0.4980348 | 1.1364591 | 0.1953362 | 0.6785221 | 1.0127337 | 0.0082026 | 1.1963064 |
| 3.9748187 | -0.1709210 | 0.8369502 | 3.0688208 | -0.0407315 | 1.7455137 | 3.1880957 | 0.3087799 | 1.2727824 |
| -0.3894742 | -0.5059437 | -1.1979979 | -1.3756127 | -0.8482443 | -1.8816108 | -1.1282557 | -0.7677280 | -0.8298136 |
| -0.0921843 | -0.1712094 | -0.1667112 | 0.4997062 | -0.0334284 | -0.2535810 | 0.4927803 | 0.0828894 | 0.0299448 |
| 0.0895503 | -0.0991513 | 0.3610384 | 0.7538759 | 0.0712120 | 0.2809881 | 0.5596815 | 0.2364484 | 0.4895968 |
| 0.7886022 | 0.0952762 | 0.8462432 | 1.2416661 | 0.4140717 | 0.8342218 | 1.1733956 | 0.1412115 | 0.7882051 |
| -0.6318046 | -0.4619297 | -0.8256100 | -0.9900399 | -0.9506075 | -1.3714443 | -1.0857397 | -0.2798342 | -0.6235543 |
| 1.1443329 | 0.0811382 | 1.0479156 | 1.0603815 | 0.2335612 | 0.9434714 | 1.0229952 | -0.0725952 | 0.7547799 |
| 0.4442490 | -0.3089161 | 0.2521596 | 0.2651857 | -0.0930737 | 0.5230805 | 0.5282647 | -0.3637910 | 0.0064958 |
| 0.4781744 | -0.3032955 | -0.0267305 | 0.4498538 | 0.0074280 | -0.0013203 | 0.2905056 | -0.4298453 | -0.3631564 |
| -1.4055716 | -0.1338614 | -0.3084563 | -0.7173735 | -0.1138880 | -0.5355902 | -0.8486138 | -0.2413538 | -0.4887717 |
| -2.5498172 | -0.6162691 | -2.0811357 | -0.4583563 | -0.8861117 | -2.0030282 | -1.2025978 | -0.3114948 | -2.2088380 |
| 0.7347723 | -0.3758678 | -0.2272146 | 0.4461361 | -0.3319951 | -0.0637483 | 0.4530465 | -0.4546752 | 0.3240653 |
| 1.2212406 | -0.3584358 | 0.1294410 | 1.2157694 | -0.3143932 | 0.1296031 | 1.0496104 | -0.3445907 | 0.1120870 |
| 0.5542161 | -0.0190577 | 0.1257571 | 0.8248664 | -0.2927302 | 0.2627730 | 0.6241763 | -0.3941594 | 0.1576530 |
| -0.3965056 | -0.2149191 | -0.2083778 | -0.1690519 | -0.1128416 | -0.3996661 | 0.0928084 | -0.1750664 | -0.2446993 |
| -0.0444039 | -0.3871804 | -0.1130915 | 0.1201840 | -0.3839006 | -0.0787428 | 0.2382328 | -0.0912770 | -0.0927633 |
| -1.5742661 | -0.5292265 | -0.7048720 | -1.1737879 | -0.3633395 | -0.9752880 | -1.0799893 | -0.2935580 | -0.5057166 |
| -1.7109871 | -0.4305148 | -0.9394712 | -1.2750146 | -0.4809954 | -1.4181409 | -1.2152543 | -0.4876922 | -1.3747695 |
| -1.9724682 | -0.0057450 | -0.1616187 | -1.7180935 | -0.0971907 | -0.7645381 | -1.5971262 | -0.0827580 | -0.3493761 |
| 0.1525278 | -0.2956721 | 0.0146305 | 0.3578569 | -0.0995302 | -0.1302262 | 0.0791039 | -0.0893489 | -0.1691376 |

| | | | | | | | | |
|---|---|---|---|---|---|---|---|---|
| 1.0320771 | 0.0110618 | -0.1089909 | 0.5456442 | -0.1331288 | -0.0545966 | 0.5649513 | 0.0567658 | 0.3427781 |
| -1.1133719 | -0.1626690 | 0.0289377 | -1.9279352 | -0.4227884 | -0.1119432 | -1.1314245 | -0.4215973 | -0.6622458 |
| -0.6704001 | -0.7397120 | -0.5259304 | -2.2219246 | -0.6590798 | -0.3613584 | -1.5166642 | -0.4093193 | -0.3834527 |
| -1.8745264 | -0.1526335 | -1.5726584 | -3.2196692 | -0.3046360 | -2.1883793 | -3.3277337 | -0.2240794 | -1.2938550 |
| -1.1132026 | -0.1864775 | -1.0299983 | -1.2500745 | -0.3846224 | -1.4181664 | -1.4065529 | -0.2343465 | -0.6663549 |
| -0.8854661 | -0.0367642 | -0.0505315 | -0.7623187 | -0.0240202 | -0.2574924 | -0.6730978 | 0.1712854 | 0.2753263 |
| 1.5255138 | -0.1139417 | 0.9987955 | 1.2440991 | -0.0041537 | 0.9804071 | 0.5180719 | 0.1138870 | 1.2175223 |
| -0.2981832 | -0.1028137 | -0.3587241 | -0.1557374 | -0.3763577 | -0.3510810 | -0.2491218 | -0.2369636 | -0.3426614 |
| -0.7077700 | -0.1089843 | -0.2033637 | -0.3961781 | -0.0820097 | -0.5207333 | -0.2999223 | 0.0005441 | -0.4632523 |
| 0.5182161 | -0.3793381 | -0.8591667 | -1.1790250 | -0.3435319 | -1.0120197 | -0.9965380 | -0.6417880 | -1.0514034 |
| -0.1776497 | -0.4931606 | -0.2734496 | 0.0489471 | -1.0600330 | -0.0033403 | -0.2671964 | -0.7541458 | -1.4629852 |
| -0.5658297 | -0.4630841 | -1.6280034 | -0.6790054 | -1.1522383 | -1.1261487 | -0.9905924 | -0.8640027 | -0.9964249 |
| -0.8166138 | 0.2754934 | 0.0342644 | -2.3022386 | 0.2930514 | -0.1385364 | -1.7236603 | -0.0430091 | -0.2270298 |
| -0.6947849 | -0.0535516 | 0.1345974 | -0.0366772 | 0.1031433 | -0.2199182 | 0.0280377 | 0.1903077 | 0.2616208 |
| -3.2306828 | -0.1125575 | -0.3560692 | -1.4832987 | -0.2266679 | -0.8704316 | -1.8074043 | -0.3690958 | -0.3915751 |
| -0.5528092 | -0.3483938 | -0.3455910 | -1.1588776 | -0.2365708 | -0.4766468 | -0.9344053 | -0.3508170 | -0.4697988 |
| -1.2509719 | -0.0432174 | -0.0127356 | -0.7519852 | 0.1358673 | -0.2875962 | -0.9259686 | 0.1624316 | -0.1772160 |
| -0.8611490 | -0.2605249 | -0.6953376 | -0.8769238 | -0.3088359 | -0.6360855 | -0.8243282 | -0.1166525 | -0.5301031 |
| -1.5638641 | 0.2053539 | -0.7937145 | -2.1691487 | 0.0695715 | -1.5181679 | -1.9349459 | -0.1751000 | -1.0519221 |
| -0.2276110 | -0.1353444 | 0.4479054 | 0.7403973 | 0.0914013 | 0.3995422 | 0.6728851 | 0.5775450 | 0.5043761 |
| -1.6544233 | -0.8605915 | -0.9653814 | -1.4777544 | -0.7866514 | -0.7701914 | -1.4104105 | -0.8079138 | -1.0901966 |
| -1.0104748 | -0.7381043 | -0.2647096 | -0.2852199 | -0.6916242 | -0.2688675 | -0.2447978 | -0.2684006 | -0.2454495 |
| -3.4121325 | -0.6169381 | -1.4622927 | -2.4286297 | -0.6493156 | -2.0059642 | -2.4326484 | -0.5908751 | -1.6701751 |
| 0.4263197 | -0.1953574 | -0.2949753 | 0.6276734 | -0.1844968 | -0.2285905 | 0.6938102 | -0.0535542 | -0.1606454 |
| -0.9969807 | -0.1362094 | -0.3764676 | -0.2669524 | 0.0049064 | -0.4424921 | -0.4736349 | -0.3230965 | -0.5912883 |
| -0.5523116 | -0.7628132 | -0.9864517 | -0.0938608 | -0.6969301 | -0.9638932 | -0.2521014 | -0.4959787 | -0.7427153 |
| -0.8868277 | -0.2006141 | -0.3989785 | -1.7676853 | -0.2096941 | -0.5686984 | -1.6291335 | -0.2820334 | -0.1535757 |
| -0.6336654 | -0.4472734 | -0.3402195 | -1.2143524 | -0.2545624 | -0.5442409 | -0.8857885 | -0.6057251 | -0.6841634 |
| 1.0579536 | -0.0506228 | 0.1672330 | 0.7960093 | -0.0947977 | 0.1262177 | 0.7704157 | 0.1319918 | 0.4187827 |
| 0.1837796 | -0.4243372 | -0.2453246 | 1.8848808 | -0.0280085 | -0.1914934 | 1.5190629 | 0.0660849 | 0.0514399 |
| -0.8184072 | -0.4202850 | -0.6028625 | -1.5464706 | -0.5786426 | -0.7313197 | -1.5744255 | -0.6586407 | 0.0245014 |
| -0.9788459 | -0.0243911 | -0.3292138 | -0.4244482 | -0.0786417 | -0.4693386 | -0.4320774 | -0.0157067 | -0.3022719 |
| 0.8806173 | -0.5333905 | -0.5358840 | -0.0903537 | -0.6086858 | -0.2392422 | -0.5215288 | 0.3571570 | 0.3837039 |
| -1.0876994 | -0.2409658 | -0.3696813 | -1.0859894 | -0.3786277 | -0.7659783 | -1.1655425 | -0.2039247 | -0.1558874 |
| -0.3284056 | -0.2757150 | -0.2099319 | -0.4272051 | -0.2514639 | -0.0588314 | -0.2860086 | -0.5714843 | -0.8420996 |
| -1.6745779 | -0.4350612 | -0.9099625 | -1.3600599 | -0.3932821 | -1.2546604 | -1.5062955 | -0.3183376 | -0.3514170 |
| -2.0233007 | -0.6839750 | -1.2314462 | -1.3064789 | -0.7608229 | -1.7899156 | -1.3718691 | -1.0611879 | -1.5631529 |
| -0.0504189 | -0.3660067 | 0.1456162 | -0.0849308 | -0.4998664 | -0.1653768 | -0.0009910 | -0.1077699 | -0.2170908 |
| 0.1643391 | -0.4090584 | 0.1914199 | 0.5685833 | -0.2344627 | 0.4515099 | 0.4394153 | -0.4481867 | 0.3150623 |
| -0.9658778 | -0.2568531 | -0.4281244 | -0.8974286 | -0.3683309 | -0.6869797 | -1.4671735 | -0.0335066 | -0.2749604 |
| 1.1501474 | -0.0268234 | 0.2463399 | 1.0080994 | 0.0127245 | 0.2301500 | 0.9050382 | 0.0621872 | 0.4070346 |
| -1.7345557 | -0.4230355 | -0.5966400 | -1.7863997 | -0.2820974 | -0.6326442 | -1.8269202 | -0.3182318 | -0.9065526 |
| -0.9079310 | -0.2403703 | -0.6924161 | -0.7597628 | -0.4409811 | -0.9070945 | -0.9852194 | -0.1384041 | -1.1344379 |
| -0.1448747 | -0.4070256 | -0.2767155 | 0.0561558 | -0.4138377 | -0.4303180 | -0.1240268 | 0.2695700 | 0.0132658 |
| -0.2954467 | -0.4255726 | -0.4860628 | -0.5329575 | -0.3606930 | -0.4085422 | -0.2687256 | -0.2910318 | -0.3405328 |
| -1.5715622 | -0.5104076 | -0.3302051 | -0.2070443 | -0.4880760 | -0.6256585 | -0.3722788 | -0.4072305 | -0.4300178 |
| -0.4503357 | -0.3066800 | -0.2334953 | -0.2977153 | -0.3190176 | -0.5914809 | -0.3754736 | -0.1375418 | -0.2046026 |
| -1.4331783 | -0.8177299 | -1.6274684 | -0.8209519 | -1.2927031 | -1.5453298 | -1.3922858 | -1.1460464 | -1.7609515 |
| -0.0502050 | -0.6557479 | -0.0117844 | 0.3954066 | -0.0886062 | 0.6802700 | 0.8567576 | -0.2393359 | 0.0582618 |
| -1.0987207 | -0.8587202 | -1.0960503 | -1.6764625 | -0.8025492 | -1.0418909 | -1.9348907 | -1.1626563 | -1.4464971 |

| | | | | | | | | |
|---|---|---|---|---|---|---|---|---|
| 0.4308627 | -0.4313378 | 0.2782946 | 0.4621349 | -0.3132233 | 0.7102094 | 1.1420280 | 0.1162626 | -0.0155720 |
| 0.4347120 | -0.5968675 | -0.3214361 | -0.4676821 | -0.3361239 | 0.0497017 | -0.1374912 | -0.6866197 | -0.5557991 |
| -0.3287629 | -0.2115486 | 0.0222810 | 0.1542710 | -0.1349388 | -0.0052071 | 0.2333003 | -0.3982785 | -0.1652496 |

| PG_631 | PG_632 | PG_633 | PG_634 | PG_635 | PG_636 | PG_637 | PG_638 | PG_639 |
|---|---|---|---|---|---|---|---|---|
| -1.952707 | -0.536557 | -0.950673 | -2.006137 | -0.600627 | -1.443553 | -1.598912 | -1.392259 | -1.085652 |
| -1.392697 | -0.943301 | -1.614404 | -2.584265 | -0.175737 | -2.476529 | -1.183841 | -1.778622 | -1.594700 |
| -0.305545 | -0.570490 | -0.767022 | -0.873216 | -0.830347 | -0.421897 | -2.090248 | -0.463355 | -0.651862 |
| 0.1531136 | -0.092050 | -0.347303 | 0.2081971 | 0.5219560 | -4.404138 | -0.949525 | -3.416258 | -3.299250 |
| -1.372825 | -0.090086 | -0.211475 | -1.043127 | 0.3359476 | -2.714859 | -0.453107 | -2.241678 | -1.986295 |
| -2.907629 | -0.485120 | -1.100135 | -3.022565 | -0.252469 | -1.807248 | -1.446957 | -1.214689 | -1.186822 |
| -0.316470 | -0.407430 | -0.991386 | -0.120129 | -0.032592 | -1.558838 | -0.499170 | -0.888640 | -1.009253 |
| -0.682907 | -0.392232 | -0.367252 | -1.454730 | 0.1340465 | -1.028009 | -0.972693 | -0.688674 | -0.843886 |
| -0.126795 | -0.345223 | 0.1776388 | -0.261063 | -0.549444 | -0.601906 | -0.139925 | -0.745047 | -1.040370 |
| 0.2806793 | -0.641778 | -0.894014 | 0.1047676 | -0.309777 | -0.935395 | -1.901495 | -0.618595 | -0.410824 |
| -0.449030 | -0.114483 | -0.288593 | -0.633016 | -0.089531 | -2.045609 | -0.313085 | -1.630505 | -1.955651 |
| -0.308722 | -0.310826 | -0.489261 | -0.305885 | 0.1053394 | -2.548179 | -0.820396 | -2.024305 | -2.462577 |
| -3.940464 | -0.211829 | -1.364515 | -4.509743 | -0.557477 | -2.598167 | -1.905628 | -2.459281 | -1.207322 |
| 4.0531463 | 0.0410779 | 3.2067931 | 6.0624072 | -0.144124 | 0.8227251 | 2.9019723 | -0.366250 | -0.584637 |
| -0.503383 | -0.076434 | -0.056946 | -0.283329 | 0.0834489 | -2.253403 | -1.177299 | -1.985286 | -2.793513 |
| 2.3781720 | 0.0772177 | 2.5550785 | 2.8076435 | -0.442259 | 1.2898408 | 2.6649498 | -0.106532 | -0.346904 |
| -1.310115 | -1.068351 | -2.097931 | -2.173027 | -1.215067 | -2.258817 | -1.730458 | -1.441756 | -0.939809 |
| -0.420527 | -0.193106 | -0.178316 | -0.238190 | -0.439231 | -1.431935 | -0.910396 | -0.504183 | -0.466653 |
| -1.435102 | -0.744373 | -1.588540 | -2.437963 | -0.498756 | -2.026732 | -2.918590 | -1.627314 | -0.580291 |
| -1.094167 | -0.494643 | -0.615170 | -0.516239 | -1.261088 | -1.760153 | -2.041886 | -3.543604 | -1.833504 |
| 0.4452387 | -0.009203 | 0.4985046 | 0.3920480 | -0.385861 | -2.589042 | -0.019868 | -1.980215 | -2.878349 |
| -0.241825 | -0.504854 | -0.375931 | -0.388423 | -0.947253 | -0.700784 | -1.024589 | -0.915095 | -0.825455 |
| 2.1657024 | 0.1096286 | 1.1461030 | 1.8466975 | -0.305287 | 1.0529592 | 1.5075436 | 0.5998740 | 0.5833571 |
| -1.220998 | -0.328257 | -0.894848 | -2.169158 | -0.557545 | -1.520082 | -2.313932 | -1.244616 | -1.298565 |
| -1.621096 | -0.215573 | -0.943409 | -1.897128 | -0.363406 | -1.852587 | -2.224467 | -1.282616 | -1.241650 |
| 1.0822428 | -0.018303 | 0.6295434 | 1.3769115 | -0.395968 | 1.1397193 | 1.4415998 | 0.0108732 | 0.0856771 |
| -0.326891 | 0.1344544 | -0.187692 | -0.356146 | 0.7172560 | -2.871474 | -1.069711 | -2.534767 | -2.669181 |
| -1.886258 | -0.502640 | -1.290930 | -2.977461 | -0.552585 | -2.665135 | -1.669704 | -2.265054 | -2.184066 |
| 0.6323595 | 0.2261416 | -0.685245 | -1.320152 | -0.005903 | -0.911384 | -1.542654 | -0.779884 | -0.603225 |
| -0.047014 | 0.1674165 | -0.130188 | -0.258689 | 0.4302124 | -2.108018 | -0.843168 | -1.931787 | -1.966859 |
| -0.495639 | -0.678677 | -0.573774 | -0.449978 | 0.4886427 | -1.024085 | -2.051307 | -0.581898 | -0.462935 |
| 0.0139238 | 0.2056397 | 0.2957705 | 0.0916336 | -0.058972 | -0.152608 | -0.971190 | 0.1271095 | -0.237582 |
| -2.223170 | -0.126995 | 0.4552530 | -1.266377 | -0.212360 | -1.859673 | -0.879660 | -0.882509 | -1.034229 |
| -3.732278 | -0.237692 | -1.138849 | -3.511072 | -0.541100 | -0.902029 | -1.607291 | -0.684683 | -0.541272 |
| -0.166064 | -0.222660 | -0.141914 | -0.136632 | 0.1669273 | -0.521502 | -0.921702 | -0.491246 | -0.478249 |
| -0.221641 | -0.831938 | -0.585440 | -0.305426 | -0.114762 | -1.855662 | -1.190197 | -1.606316 | -1.422036 |
| -2.358469 | -0.426361 | -1.149855 | -2.339685 | -0.088819 | -1.481493 | -1.379044 | -1.174505 | -1.166917 |
| -0.252412 | 0.0953866 | 3.7087297 | -0.438220 | 0.0788333 | 2.6857075 | 1.3658380 | 0.2263519 | 0.3341831 |
| -1.558949 | -0.062220 | -0.478639 | -1.565246 | -0.099933 | -0.744365 | -0.602525 | -0.537721 | -0.854715 |
| 2.6613226 | -1.402281 | -0.529920 | 0.4684662 | 0.2267960 | -3.331258 | -1.389554 | -1.833081 | -1.351869 |
| 0.7004623 | 0.0490997 | 0.2360848 | 0.8287788 | -0.434571 | 0.9697514 | 0.7144504 | 0.9457117 | 0.6720731 |
| -1.941074 | -0.564538 | -1.069262 | -1.560136 | -0.812467 | -0.518429 | -1.984715 | -0.719852 | -0.340388 |
| -0.596576 | -0.495910 | -0.569918 | -0.769616 | 0.0413555 | -1.984582 | -0.985443 | -1.351611 | -1.349628 |
| -0.226572 | -0.136906 | -0.450345 | -0.817671 | 0.2104802 | -2.854183 | -1.705633 | -2.166926 | -2.790794 |
| 0.8552675 | 0.3052600 | 0.7358948 | 1.4844138 | -0.415400 | 1.5866094 | 1.6494538 | 0.3649789 | 0.5307654 |
| 2.9347796 | 0.4544224 | 0.6372079 | 3.8295847 | 0.1615035 | -0.388463 | 2.2426011 | -0.272267 | -0.205996 |
| -0.035504 | 0.3700825 | -0.382651 | -0.246923 | -0.248920 | 0.8497227 | -0.086604 | 0.4100451 | 0.6792843 |

| | | | | | | | | |
|---|---|---|---|---|---|---|---|---|
| 0.1226380 | -0.2155012 | -0.3990924 | -0.1368155 | -0.1957269 | -2.3619457 | -0.3947013 | -1.4731667 | -2.0121557 |
| -2.2485369 | -0.2900163 | -0.4035975 | -3.4848557 | -0.3634129 | -1.4526140 | -1.2431370 | -0.9961555 | -1.1675779 |
| -0.2485533 | -0.4930316 | -0.0482023 | 0.2375671 | -0.5711885 | -1.6329185 | -0.3061527 | -1.6078252 | -1.5620811 |
| -0.0085067 | -0.2242975 | -0.4556499 | -0.1946677 | -1.3893043 | -1.4604468 | 1.5337246 | -1.3033679 | -1.0962756 |
| -0.4414236 | -0.6339960 | -0.8073199 | -0.2766951 | -0.4948149 | -0.7402661 | -1.5877660 | -1.1005453 | -0.8801687 |
| -0.0382861 | -0.2529230 | -0.1541737 | 0.2837275 | -0.1128508 | 0.4489583 | 0.0983441 | 0.3490196 | 0.1300363 |
| -2.0589219 | -0.6047327 | -1.2203347 | -2.6018594 | 0.0350527 | -1.5961890 | -0.9383190 | -1.3358863 | -0.6997646 |
| 3.7333308 | 0.1075451 | 2.4596354 | 3.6775978 | -0.5358791 | 1.8804653 | 3.0490341 | 0.0034503 | 0.1953481 |
| -1.5185405 | -0.5619870 | 0.9037596 | -2.5318538 | -0.3945215 | 2.6891993 | 2.2405535 | -0.3949927 | -0.0168874 |
| 0.9695146 | -0.5612114 | -0.4284644 | 0.5581505 | -0.7464247 | -1.0926741 | -0.3839185 | -1.6957100 | -1.3507987 |
| -1.9947413 | -1.4071391 | -2.2735889 | -2.8760318 | -0.3998979 | -2.4037497 | -2.4732540 | -1.3989173 | -1.2990528 |
| 2.4360934 | 1.1780317 | 3.2814948 | 2.9982495 | 0.1295308 | 1.0738106 | 3.1826899 | -0.3758919 | -1.0987335 |
| 4.4626978 | 1.3138733 | 3.9336936 | 4.5589877 | 0.6279317 | -1.2683646 | 1.3785834 | -1.2857091 | -1.8855462 |
| -1.3188170 | -0.1321171 | -0.4116799 | -1.5917352 | -0.5955176 | -1.6176992 | -1.0530689 | -1.4081997 | -1.4322384 |
| -1.7670695 | -1.5178860 | -1.8317072 | -2.1759068 | -0.4971786 | -2.3645260 | -1.8043490 | -1.7164207 | -1.5177371 |
| -1.0835661 | -0.3792291 | -0.2526018 | -1.2881321 | -0.2912711 | -1.8351483 | -1.5691692 | -1.6569711 | -1.1957089 |
| -0.7834830 | 0.1650985 | -0.5339765 | -1.2451480 | -0.0355055 | -1.8822524 | -2.0983106 | -1.6177144 | -1.4135282 |
| -0.2735380 | -0.2632742 | -0.8394411 | -0.3824398 | -0.1009674 | -1.2009795 | -0.9441431 | -1.0095039 | -0.9877091 |
| -0.5522345 | -0.2884038 | -0.6881064 | -0.9227045 | -0.1119801 | -2.8392941 | -1.4967263 | -1.6212603 | -2.1216998 |
| -0.4490247 | -0.0551624 | -0.1688263 | -0.6244531 | 0.5043296 | -2.7527098 | -1.8103726 | -2.6050468 | -3.0042715 |
| 0.0719802 | -0.2253280 | -0.0779236 | -0.0775581 | -0.3360159 | -0.7786247 | -1.0231124 | -0.9029990 | -0.7796671 |
| -3.0147340 | -0.7933956 | -1.0751079 | -2.9914401 | -0.8110197 | -1.1408389 | -1.6239199 | -0.9798150 | -0.8689422 |
| 0.4619882 | -0.2532663 | -0.3225699 | 0.6007674 | -0.3369421 | 0.8160317 | 0.2738781 | 0.7313742 | 0.6675892 |
| 1.1557775 | 0.7386364 | 0.5305251 | 1.0456172 | 0.9893636 | -2.8813117 | -1.1364414 | -2.5021751 | -2.7405290 |
| -1.4051320 | -0.4038174 | -0.9921731 | -2.3689615 | -0.8234965 | -2.7279653 | -1.6267714 | -2.3806318 | -1.6034885 |
| -0.8386293 | -0.4797701 | -0.8970273 | -1.5533304 | -0.3698133 | -1.2410533 | -0.4104398 | -0.9508775 | -0.6593753 |
| -0.0865266 | -0.6795899 | -0.6414313 | 0.0259393 | -0.4709787 | -1.8201933 | -2.6007463 | -0.0065537 | -0.2050493 |
| 4.5648699 | 1.1329318 | 2.4619511 | 4.8093074 | -0.2771597 | 0.3833891 | 2.6907418 | -0.0448958 | -0.4513296 |
| 3.2288972 | 0.5645226 | 1.3736057 | 3.7843771 | 0.0692709 | -0.3261373 | 1.3677861 | -1.2085292 | -1.0861272 |
| -1.2519198 | -0.1033130 | -0.1833942 | -2.1872685 | -0.0862414 | -0.5288313 | -1.1004773 | -0.4408182 | -0.4313129 |
| -0.0754986 | -0.2291466 | -0.3305065 | 0.0251565 | -0.1882680 | -0.0921060 | -0.8143096 | -0.7918603 | -0.4481452 |
| 0.2711526 | -0.0247580 | -0.2502901 | -0.0870227 | 0.3232966 | -4.5031957 | -0.7655472 | -3.1521215 | -3.4126803 |
| -0.7957302 | -0.6884084 | -1.5794968 | -1.2026084 | -0.5219423 | -1.9023379 | -1.6793645 | -1.1739765 | -1.3432244 |
| -0.6196012 | -0.3910142 | -0.7293440 | -1.7280344 | -0.3737245 | -2.8693633 | -0.8788841 | -2.2655324 | -1.9792594 |
| 1.7350990 | 0.2804751 | 1.2928718 | 1.8349082 | -0.9147253 | 0.9969214 | 1.4553683 | -0.6845666 | -0.0264411 |
| -0.0325842 | 0.4689947 | 0.0604109 | -0.5197406 | -0.1696486 | -0.7302983 | -1.5932585 | -0.7353012 | -0.5410055 |
| -0.1023905 | -0.0396386 | -0.4519870 | 0.3465811 | -0.0376670 | -1.4774191 | -0.9628308 | -0.3709798 | -0.7556497 |
| -1.1293638 | -0.3726186 | -0.8966534 | -1.9908837 | -0.1320716 | -0.9284477 | -2.0113046 | -0.8856645 | -0.9023749 |
| -1.7501901 | -0.2955502 | -0.7232964 | -2.4503082 | -0.8300008 | 0.1681465 | -1.1926840 | 0.1058560 | 0.1214698 |
| -1.5037135 | -0.6102317 | -0.3149506 | -2.1787274 | -0.6176273 | -0.9176778 | -1.5416999 | -0.1873948 | -0.2349507 |
| -1.1869131 | -0.8578266 | -0.9143490 | -1.5516841 | -0.5452657 | -1.3709739 | -1.2056447 | -1.3794945 | -1.0629181 |
| -2.1067009 | -0.8145054 | -1.5517137 | -3.2531379 | 0.3094174 | -1.4950422 | -1.4903165 | -0.3014558 | -0.5105696 |
| 3.2154681 | 1.9151390 | 4.4610419 | 3.1909201 | -0.7197917 | 2.7079453 | 3.2404583 | 0.7272085 | 0.6017317 |
| -0.2890495 | 0.0872965 | -0.1304213 | -0.5085092 | -0.0094460 | -1.5213269 | -0.4952649 | -1.4149748 | -1.0783655 |
| -1.3863980 | -0.2964016 | -0.8103244 | -1.5536041 | 0.3102819 | -3.2240244 | -1.7041674 | -2.1680416 | -2.1265336 |
| -0.7467982 | -0.6680006 | -0.9255546 | -1.1295246 | -0.7381613 | -1.7482455 | -2.5291840 | -1.0715488 | -1.0026418 |
| -0.5364234 | -0.0166006 | -0.0839963 | -0.1416420 | -0.4002535 | 0.9074955 | 2.9568658 | 0.0980271 | -0.1445859 |
| 2.0258632 | 0.2294611 | 2.4612398 | 2.6591838 | 0.3434289 | 0.1932075 | 1.5147617 | -0.7580866 | -1.0531923 |
| -3.2924752 | -0.1078883 | -0.9097347 | -3.6293886 | 0.0047209 | -2.6729776 | -1.1734923 | -1.7692140 | -1.7390508 |
| -0.4690871 | -0.4384091 | -0.4230727 | -1.1979530 | -0.4024082 | -1.2333165 | -1.0727437 | -0.6683373 | -0.7459939 |

| | | | | | | | | |
|---|---|---|---|---|---|---|---|---|
| -3.411566 | 0.8181132 | 0.4838824 | -3.152477 | -0.279697 | -2.007617 | -1.178891 | -1.799257 | -1.919534 |
| 0.8356429 | 0.0357660 | 0.1240782 | 1.1765882 | -0.272579 | -0.904705 | -0.288428 | -0.563842 | -1.015168 |
| 0.7934008 | -0.602614 | -0.557040 | 0.5624527 | -0.638555 | -1.341832 | -0.867525 | -0.492311 | -0.680105 |
| -0.220875 | -0.046763 | 0.0410769 | 0.1472700 | -0.053965 | -1.603849 | -0.078921 | -1.076727 | -1.301829 |
| -1.467576 | -0.654120 | -1.556297 | -2.106191 | -0.147650 | -2.199517 | -1.971977 | -1.101299 | -1.188626 |
| -0.492379 | -0.356965 | 0.0648669 | -1.313835 | -0.786139 | -1.538977 | -1.994823 | -1.525102 | -1.354469 |
| -0.232755 | -0.289539 | -0.016664 | -0.513856 | -0.001397 | -2.181491 | -0.733511 | -1.677708 | -2.690946 |
| -0.182055 | -0.057690 | 0.0197834 | 0.0832009 | -0.017063 | -0.341509 | -0.133597 | -0.127119 | -0.251062 |
| -0.204802 | -0.409717 | 0.0154014 | 0.0419928 | -0.835363 | 1.4749215 | -0.178823 | 0.9179586 | 0.6954858 |
| 0.5520453 | 0.5057934 | 0.9302849 | 0.6800920 | -0.260605 | 0.1504300 | 1.3415672 | -0.584696 | -0.380335 |
| -2.193178 | 0.1677751 | -0.102172 | -1.638735 | -0.011995 | -1.278087 | -1.288277 | -1.152249 | -1.111693 |
| -2.694011 | -0.212541 | -1.007094 | -3.287412 | -0.001338 | -3.026997 | -1.242756 | -3.201359 | -2.349020 |
| -4.478051 | -0.118829 | -0.694400 | -4.909296 | -0.645848 | -0.122511 | -1.316468 | -0.251397 | 0.1062001 |
| -2.586199 | 0.0119579 | -1.065196 | -2.539160 | -0.111365 | -1.795035 | -1.196737 | -0.997340 | -0.938493 |
| -0.497835 | -0.349114 | -0.815879 | -0.336380 | -0.015356 | -1.872913 | -0.934252 | -1.139486 | -1.065962 |
| -0.149723 | -0.279606 | -0.020692 | -0.087595 | -0.254966 | -0.090845 | -0.253865 | 0.1225977 | -0.152504 |
| -0.992149 | -0.695578 | -1.687734 | -1.138601 | -1.168421 | -1.135358 | -1.127344 | -1.550552 | -1.638613 |
| -0.163406 | -0.268164 | -0.156958 | -0.207806 | 0.5169351 | 0.2146593 | -0.238932 | 0.5156938 | 0.3778634 |
| -0.253765 | -0.449701 | -0.448649 | -1.199643 | -0.273393 | -0.684091 | -0.987713 | -1.031351 | -1.390174 |
| 2.1714762 | -0.256921 | 0.7637356 | 2.4087521 | -1.126040 | 1.2006325 | 1.4376833 | 0.7509245 | 0.9626469 |
| -0.908079 | -0.328404 | -0.377944 | -1.416939 | -0.637286 | -1.186245 | -1.379847 | -0.999818 | -1.123520 |
| 0.1477664 | -0.140202 | -0.466857 | -0.187321 | -0.489715 | -1.786208 | -0.787121 | -0.905036 | -1.890281 |
| -3.546390 | 0.1761216 | 0.2547985 | -3.590971 | -0.088326 | -0.186284 | -0.439875 | -0.248426 | -0.240614 |
| -2.020683 | -0.458562 | -1.545317 | -2.886587 | -0.600883 | -0.984905 | -1.187620 | -1.099585 | -1.056332 |
| -0.351009 | -0.359782 | -0.801354 | -0.320426 | -0.393622 | -1.801080 | -2.643578 | -0.465924 | -0.700305 |
| 0.4322735 | -0.152908 | -0.316355 | 0.1559458 | -0.299889 | -1.757113 | -0.890971 | -1.734768 | -0.997426 |
| -0.386575 | 0.1034534 | -0.285026 | -0.106771 | 0.3769732 | -0.897700 | -0.199479 | -0.834798 | -1.080450 |
| -0.293955 | -0.426263 | -0.121098 | 0.0961202 | -0.596145 | 0.9631604 | 0.1338555 | 0.4243380 | 0.8409079 |
| -0.007443 | -0.232557 | -0.937601 | -0.468889 | -0.171776 | -1.161836 | -2.067102 | -0.560414 | -0.358784 |
| -2.224532 | -0.401156 | -0.127471 | -1.852520 | -0.012363 | -2.402689 | -0.449350 | -1.341553 | -1.671883 |
| -2.136928 | -0.771660 | -1.489656 | -1.478653 | -0.711883 | -0.648620 | -1.277352 | -0.244345 | 0.3767070 |
| -1.502217 | -0.302236 | -0.784233 | -2.725173 | -0.092674 | -1.835810 | -1.556899 | -1.939998 | -1.288201 |
| -2.642203 | -1.251347 | -1.632428 | -2.513607 | -0.206053 | -1.947954 | -1.537534 | -0.177746 | -0.874042 |
| 0.0444174 | 0.2100679 | 0.4085597 | -0.054057 | 0.0361909 | -1.327286 | 0.1026241 | -0.251398 | -0.848403 |
| -1.038362 | -0.466440 | -0.489953 | -2.127291 | -0.462459 | -0.620515 | -1.173822 | -0.211225 | -0.319473 |
| -0.157002 | -0.450387 | -0.798292 | -0.622574 | -0.353270 | -2.750255 | -1.858616 | -2.059013 | -1.920128 |
| -0.027526 | -0.213460 | -0.165371 | -0.530646 | -0.144048 | -2.160281 | -0.418804 | -1.491407 | -1.667208 |
| -1.694022 | -0.225876 | -0.509814 | -1.914014 | 0.0745945 | -3.687729 | -1.160358 | -3.001657 | -2.900292 |
| -0.075156 | -0.598787 | -0.743370 | -0.598867 | -0.391677 | -0.963449 | -0.680297 | -0.750971 | -0.833913 |
| -2.620331 | -0.365693 | -0.368078 | -3.095569 | -0.379710 | -2.027153 | -1.963924 | -0.895912 | -0.577237 |
| 0.0380051 | -1.387789 | -0.769777 | -0.362088 | 0.0610069 | -2.250291 | 0.0944090 | -1.849993 | -1.818802 |
| -1.972287 | 0.1086579 | 0.2608798 | -2.187663 | -0.312860 | -2.305922 | -0.426018 | -1.577834 | -1.926009 |
| -1.297581 | 0.0742383 | -0.482333 | -1.968282 | 0.1646308 | -2.454241 | -1.431483 | -1.832194 | -2.229247 |
| -0.427040 | 0.1510273 | -0.452599 | -0.902361 | -0.046942 | -1.637335 | -0.637275 | -1.655913 | -0.943420 |
| -1.788382 | 0.4816560 | 0.1501411 | -1.468115 | -0.480138 | -1.122569 | -1.522275 | -0.671699 | -0.537458 |
| 0.0543649 | -0.374493 | -0.191795 | 0.2727166 | -0.058189 | -1.600818 | -0.430925 | -1.612832 | -1.285513 |
| -1.733704 | -0.806388 | -1.403432 | -1.802081 | -0.323462 | -2.244724 | -1.337567 | -1.267147 | -1.742777 |
| 0.6548339 | -0.215779 | 0.4030447 | 0.7377704 | -0.401015 | -1.577211 | 0.3686848 | -1.026342 | -1.370109 |
| -2.979770 | -0.252986 | -1.841735 | -2.952228 | -0.413381 | -1.340017 | -2.917334 | -1.101097 | -1.204952 |
| -1.549058 | -0.474487 | -0.536028 | -2.313452 | -0.821204 | -1.581106 | -1.824942 | -1.631286 | -1.638551 |

| | | | | | | | | |
|---|---|---|---|---|---|---|---|---|
| 0.0805349 | 0.3661262 | 0.1819145 | 0.3001947 | 0.8084284 | -2.900624 | -0.822734 | -2.371345 | -2.014730 |
| -1.366926 | -1.094770 | -1.659022 | -2.146672 | -0.652364 | -1.290882 | -1.678647 | -1.523292 | -0.875534 |
| -0.883406 | -0.804949 | -0.777527 | -1.034940 | -0.390463 | -1.611411 | -0.726602 | -1.371409 | -1.335722 |
| 1.5430200 | -0.008650 | 1.9113969 | 1.6378423 | -0.563441 | 0.1875712 | -0.096246 | -0.130396 | -0.436524 |
| -1.948126 | -0.214407 | -1.495991 | -2.248135 | 0.0482058 | -1.476153 | -1.714694 | -1.020592 | -1.137460 |
| 3.1109247 | 1.6652671 | 2.4806935 | 2.7647427 | -0.212186 | 3.1607314 | 2.4970637 | 0.9279693 | 0.0945409 |
| -0.673028 | -0.155707 | -0.492564 | -0.893038 | -0.365294 | -1.957564 | -0.953926 | -1.557616 | -1.858085 |
| -0.515390 | -0.754099 | -1.101709 | -1.045706 | -0.401767 | -0.829324 | -0.832534 | -0.602375 | -0.378704 |
| -2.068984 | -0.329664 | -1.268568 | -2.344850 | -0.472977 | -1.558214 | -1.727768 | -1.111287 | -1.279353 |
| -0.562957 | -0.860715 | -1.262961 | -1.116565 | -0.938036 | -1.050599 | -0.725996 | -0.997887 | -0.973047 |
| -0.206676 | -0.284486 | -0.084265 | 0.3648783 | -0.251397 | -1.144285 | 0.0198371 | -0.999380 | -0.584509 |
| -0.742378 | -0.581492 | -0.819597 | -0.942618 | -0.731514 | -1.240180 | -1.060961 | -0.762612 | -1.169926 |
| -1.064374 | -0.872514 | -1.440237 | -1.228728 | -0.124235 | -1.015195 | -1.146812 | -0.561410 | -0.494287 |
| -0.601816 | -0.245247 | -0.296542 | -0.053043 | -0.356957 | -1.669527 | -0.436570 | -1.795532 | -0.885599 |
| -0.144387 | -0.376770 | -0.093420 | -0.034931 | -0.404780 | -0.090967 | -0.245526 | -0.418993 | -0.298203 |
| -2.581043 | 0.5134057 | 0.2912253 | -1.761455 | 0.6950390 | -1.654118 | -1.165755 | -1.260076 | -1.201006 |
| -0.105515 | -0.475170 | -0.469478 | 0.0717087 | -0.875076 | 1.2259853 | -0.012976 | 1.0402253 | 1.2694047 |
| -0.586629 | -1.111177 | -2.089095 | -2.124392 | -0.663509 | -1.433581 | -1.032284 | -0.707344 | -0.324693 |
| 0.0972745 | -1.147171 | -1.035966 | -0.582268 | -0.356401 | -0.828450 | -1.197313 | -0.495814 | -0.872857 |
| -0.670646 | -0.484928 | -1.060001 | -1.919772 | -0.066045 | -3.598269 | -1.659447 | -2.621232 | -2.476845 |
| -0.604622 | 0.0237508 | 0.2780306 | -0.176465 | -0.064036 | 0.7442290 | 0.6353761 | 0.3098546 | 0.2816576 |
| 0.1927070 | -0.317487 | 0.0262476 | -0.454590 | -0.258470 | -0.325792 | -0.428973 | -0.412151 | -0.632010 |
| -0.691067 | -0.238411 | -0.933355 | -1.826551 | -0.388837 | -1.440479 | -1.809036 | -1.023403 | -0.926739 |
| -0.721989 | -0.419889 | -0.310654 | -1.133220 | -0.463663 | -1.680204 | -0.805702 | -1.131430 | -1.047063 |
| -0.711135 | -0.349583 | -1.151135 | -1.413951 | -0.225050 | -1.018060 | -1.433748 | -0.448161 | -0.493711 |
| -0.185089 | -0.220155 | -0.765540 | -0.513921 | -0.283391 | -1.452152 | -0.552683 | -1.207494 | -0.950100 |
| 0.5052877 | -0.526586 | -1.338011 | -0.257349 | -0.729913 | -1.883537 | -1.071713 | -1.389327 | -1.257509 |
| -0.627057 | -0.498735 | -0.737108 | 0.4353380 | -0.120254 | -1.625415 | -0.769694 | -1.058774 | -1.000683 |
| -0.629114 | -0.940627 | -1.085809 | -1.325105 | -1.059095 | -1.454278 | -1.641932 | -0.909390 | -1.211082 |
| 1.5967453 | 0.3413230 | 0.9166222 | 1.7726303 | 0.0460305 | -1.064859 | 1.2521008 | -0.518181 | -0.846126 |
| 2.8066098 | 0.5076326 | 2.2484004 | 3.6193503 | -0.424242 | 2.0525111 | 2.8944565 | 0.9116761 | 0.8929739 |
| 0.0535332 | -0.891930 | -1.252967 | 0.0405443 | -0.587704 | -1.296955 | -0.695006 | -0.841651 | -0.645026 |
| 0.2271177 | 0.1426589 | 0.0707343 | 0.1569704 | 0.0952330 | -1.042569 | 0.1933787 | -0.390596 | -0.650873 |
| 1.0305317 | 0.2421895 | 0.5995004 | 0.8941058 | 0.0931494 | -1.533206 | 0.2508213 | -0.974284 | -1.558262 |
| 1.0546626 | 0.3981937 | 0.9468945 | 0.8673901 | -0.177897 | 1.3995666 | 1.4616507 | 1.0331427 | 0.7669388 |
| -0.815616 | -0.156283 | -0.599063 | -1.150288 | -0.027342 | -2.005646 | -1.826910 | -1.324587 | -0.871815 |
| 1.3371408 | 0.5040698 | 0.8658774 | 1.6883812 | 0.2035338 | -1.695017 | -0.323351 | -0.852558 | -1.265796 |
| 0.0376499 | -0.219251 | 0.3351728 | 0.7911058 | -0.141727 | -1.866212 | 0.4627559 | -1.941739 | -2.205858 |
| 0.2681014 | -0.369808 | -0.286888 | 0.5960929 | -0.133833 | -2.096063 | -0.676449 | -1.830297 | -1.706046 |
| -0.446289 | -0.321059 | -0.677937 | -0.438246 | 0.2596172 | -2.084054 | -1.325361 | -1.679990 | -2.062348 |
| -1.073550 | -0.748057 | -2.249720 | -3.312588 | -0.512604 | -0.908459 | -1.394769 | -0.277118 | -0.137624 |
| 0.6868506 | 0.0843796 | 0.6576270 | 0.9535209 | -0.384645 | -1.239001 | 0.6739153 | -0.737568 | -0.361362 |
| 2.0853738 | -0.214772 | -0.085472 | 1.6795612 | -0.619411 | -2.623833 | -0.418066 | -2.443798 | -2.280255 |
| 0.6923891 | 0.0014450 | 0.1515200 | 0.4410842 | 0.1208745 | -1.734244 | 0.1945450 | -1.416750 | -1.369583 |
| -0.340015 | -0.287248 | -0.619751 | -0.124858 | -0.124868 | -1.173205 | -0.904639 | -0.913105 | -0.789712 |
| 0.1845690 | -0.197016 | -0.124806 | 0.0909253 | -0.553940 | -1.722190 | 0.0156143 | -1.045811 | -1.661456 |
| -0.710854 | -0.410930 | -0.618158 | -0.870990 | -0.653957 | -0.932058 | -1.551311 | -0.610741 | -0.625709 |
| -1.281103 | -0.895902 | -1.590378 | -1.749490 | -0.252793 | -1.813590 | -1.640416 | -1.229280 | -1.206509 |
| -1.134726 | -0.246064 | -0.565684 | -1.365153 | -0.142369 | -0.117322 | -1.425572 | -0.087517 | -0.240085 |
| -0.139941 | -0.209988 | -0.135103 | -0.073580 | 0.1243498 | -2.996100 | -0.419750 | -2.543807 | -2.452922 |

| | | | | | | | | |
|---|---|---|---|---|---|---|---|---|
| 0.4276685 | 0.4555516 | 0.2648705 | 1.0812219 | 0.5241745 | -3.820303 | -1.272373 | -3.118742 | -3.449975 |
| -1.737979 | -0.727820 | -0.660048 | -2.163306 | -0.618931 | -0.035572 | -0.565654 | -0.279644 | -0.386973 |
| -2.143971 | -0.386065 | -0.414830 | -1.877976 | -0.456102 | -1.913856 | -0.675400 | -1.171259 | -1.757765 |
| -3.316310 | -0.329712 | -1.819847 | -4.231992 | -0.252081 | -1.600492 | -2.184417 | -0.785667 | -0.873693 |
| -0.801333 | -0.388574 | -1.014380 | -1.497521 | -0.473610 | -2.950816 | -1.744334 | -1.497512 | -1.760756 |
| 0.2139757 | 0.1255263 | 0.2260542 | 0.1662552 | -0.276849 | -0.468601 | -0.444450 | -0.862023 | -0.916518 |
| 3.1399641 | 0.3757595 | 0.9809096 | 1.7646099 | -0.275784 | 1.0364459 | 1.6501658 | 0.1447352 | -0.029450 |
| -0.050223 | -0.207681 | -0.340875 | 0.0134580 | -0.377266 | 0.3047636 | -0.287703 | 0.8727794 | 0.1009346 |
| -1.540241 | 0.0646460 | -0.751728 | -2.677948 | 0.4420471 | -4.166905 | -1.427225 | -4.283947 | -4.358435 |
| -2.015933 | -0.699255 | -1.356271 | -1.561745 | -1.387706 | -1.854703 | -0.814748 | -1.707844 | -1.120287 |
| -0.147030 | -1.690695 | -1.302208 | -0.815284 | 0.2256461 | -2.194373 | -0.331238 | -1.175570 | -1.097611 |
| 0.2628145 | -0.934148 | -0.725903 | -0.218295 | -0.477422 | -2.019222 | -1.126286 | -1.196565 | -1.725437 |
| -0.946161 | -0.259242 | -0.313343 | -0.608353 | -0.527596 | 0.3619527 | -0.723722 | 0.2169851 | 0.3000122 |
| 0.0941654 | 0.1690937 | 0.1331281 | -0.046196 | -0.258771 | -1.233685 | -1.019433 | -0.590563 | -0.992192 |
| -1.642229 | -0.224145 | -0.690311 | -2.446865 | -0.374916 | -1.307512 | -1.824312 | -0.995141 | -1.180274 |
| -0.596959 | -0.181089 | -0.293962 | -0.834593 | -0.501316 | -1.141803 | -1.248932 | -0.609136 | -0.455389 |
| -0.197046 | 0.0597201 | -0.173110 | -0.785956 | 0.4676972 | -5.584004 | -1.896251 | -5.005867 | -5.453178 |
| -1.252517 | -0.099491 | -0.588442 | -1.898434 | -0.352866 | -1.338920 | -0.965107 | -1.010668 | -1.095491 |
| -1.361867 | -0.260738 | -1.542898 | -1.986463 | -0.513433 | -2.238514 | -2.111410 | -1.001700 | -1.629217 |
| 0.9534215 | 0.6512707 | 0.5777907 | 0.7309298 | 0.6848849 | -1.482556 | -1.093732 | -1.250954 | -1.141532 |
| -1.901025 | -0.784484 | -0.772107 | -2.048448 | -0.517815 | -2.562462 | -2.079815 | -2.301712 | -1.517777 |
| -0.271958 | -0.278635 | -0.305331 | -0.335264 | 0.4575646 | -1.321482 | -3.797320 | -0.237857 | -0.213349 |
| -3.253368 | -0.782194 | -2.432952 | -3.462722 | -0.676795 | -1.252816 | -2.817762 | -0.797344 | -0.674764 |
| 0.2601625 | -0.137409 | -0.148607 | 0.6096226 | -0.203395 | -2.453107 | -0.662979 | -1.929081 | -1.661984 |
| -0.667154 | -0.019319 | -0.440396 | -1.149313 | -0.248989 | -0.904035 | -0.956517 | -0.873867 | -0.243867 |
| -0.690432 | -0.360613 | -0.693573 | -0.470310 | -0.606938 | -1.023907 | -0.027803 | -1.006975 | -1.157101 |
| -1.499448 | -0.103992 | -0.223562 | -1.727335 | 0.0545055 | -1.226013 | -1.180701 | -0.793172 | -0.984163 |
| -1.194369 | -0.597100 | -0.801718 | -0.820925 | -0.446613 | -1.322883 | -0.937175 | -1.156236 | -1.036077 |
| 0.8919054 | 0.3321055 | 0.4398226 | 0.8212230 | -0.183187 | -1.108465 | 0.1708320 | -1.383406 | -0.764888 |
| 1.0446823 | -0.034988 | 0.2552605 | 0.8693004 | 0.2248239 | -2.394294 | -0.418537 | -2.025029 | -2.329678 |
| -0.095613 | -0.331843 | -0.241474 | -0.837861 | -0.439336 | -0.582313 | -0.881320 | -0.580005 | -0.464946 |
| -0.882536 | -0.123584 | -0.509953 | -0.931027 | -0.033032 | -2.911771 | -0.828445 | -2.114171 | -2.341625 |
| 2.0458323 | 0.2143473 | 0.1572442 | 0.7936727 | -0.894564 | 0.5010669 | 1.0044151 | 0.0876274 | -0.002348 |
| -0.719852 | -0.236123 | -0.389880 | -1.119400 | -0.293317 | -0.912753 | -0.809468 | -0.564659 | -0.822388 |
| -0.750126 | -0.633998 | -0.527240 | -0.977255 | -0.474592 | -0.150942 | -0.812348 | -0.248101 | -0.242247 |
| -1.798093 | -0.285637 | -0.506067 | -2.007748 | -0.280234 | -1.565653 | -0.546401 | -1.619162 | -1.338648 |
| -1.526252 | -0.598048 | -1.649977 | -1.575800 | -0.826313 | -1.348565 | -1.601129 | -1.000096 | -0.966865 |
| -0.232068 | -0.243615 | -0.068077 | -0.124832 | -0.617561 | -1.676441 | -3.641265 | -1.065049 | -1.046249 |
| 1.0018218 | -0.546446 | 0.5723777 | 0.6165123 | -0.482483 | 0.5055622 | 0.5307581 | 0.1397619 | 0.0780591 |
| -0.030883 | -0.223842 | -0.290541 | -0.334642 | -0.394591 | -1.274580 | -0.821294 | -0.574979 | -0.832600 |
| 1.1289642 | 0.2013789 | 0.6244095 | 1.3972376 | 0.4140471 | -4.151637 | -0.627465 | -3.800524 | -4.216783 |
| -1.613346 | -0.387782 | -1.128014 | -2.153402 | -0.081606 | -2.731373 | -1.329591 | -1.925766 | -2.008494 |
| -0.728241 | -0.723205 | -1.111988 | -0.819619 | -0.413609 | -1.046699 | -0.683741 | -0.708544 | -0.574018 |
| 0.8718451 | 0.1138413 | 0.0298184 | 0.6231660 | 0.3913562 | -1.593598 | -1.747838 | -0.884581 | -0.664976 |
| -0.758391 | -0.092666 | -0.426890 | -0.919859 | -0.509983 | -1.520453 | 0.2677390 | -1.560754 | -1.344879 |
| -0.322438 | -0.380938 | -0.556016 | -0.583024 | -0.299123 | -4.248467 | -1.429661 | -2.983546 | -3.482885 |
| 0.0982138 | -0.094264 | -0.427384 | -0.105649 | -0.333739 | -1.428162 | -1.436193 | -0.980141 | -1.187943 |
| 0.2073140 | -1.509332 | -1.869281 | -2.010253 | -0.766055 | -1.677371 | -1.083945 | -1.318182 | -1.305031 |
| -1.002772 | -0.206540 | 0.1015281 | 0.4067094 | -0.517550 | -1.619302 | -1.009619 | -1.946847 | -2.142672 |
| -1.742801 | -1.272797 | -1.364211 | -1.859179 | -0.667624 | -1.215955 | -0.883593 | -0.931792 | -1.007851 |

| | | | | | | | | |
|---|---|---|---|---|---|---|---|---|
| -0.3863683 | 0.1503945 | 0.1459472 | 1.0470638 | -0.6863100 | -0.2670075 | -0.5041669 | -0.5894669 | -1.0939063 |
| 0.1641944 | -0.3759258 | -0.0474596 | 0.6645655 | -0.7683386 | -0.7288318 | -0.5336178 | -1.0222981 | -0.6811588 |
| 0.0836046 | -0.5039561 | -0.1647178 | 0.2615252 | -0.7031656 | -1.4575897 | -0.3042594 | -0.8877972 | -1.5667350 |

| PG_640 |
|---|
| -2.665278415 |
| -2.336969763 |
| -2.572565763 |
| -1.232316961 |
| -0.83435278 |
| -1.253154965 |
| -0.176722104 |
| -2.379223858 |
| -0.050431276 |
| -2.601510036 |
| -0.482704347 |
| -1.193638517 |
| -3.767977896 |
| 3.396736102 |
| -1.444220945 |
| 3.223208925 |
| -2.116414422 |
| -0.732240931 |
| -3.276753287 |
| -3.655108059 |
| -0.212935839 |
| -0.653343616 |
| 1.8320528 |
| -2.268013161 |
| -2.680878883 |
| 1.333029058 |
| -1.931094292 |
| -2.057748874 |
| -2.597703162 |
| -0.667463954 |
| -3.753836115 |
| -1.788841665 |
| -1.565965259 |
| -4.505037899 |
| -1.296190464 |
| -1.142521982 |
| -1.73032339 |
| 0.572500844 |
| -0.629311147 |
| -1.764724972 |
| 1.416475953 |
| -2.598515468 |
| -1.207860463 |
| -2.335598267 |
| 1.689314978 |
| 2.571573137 |
| -0.053336747 |

-0.24964366
-2.198852284
-0.304902567
1.912567348
-1.363565773
0.265683167
-1.545390403
3.633497858
0.713673586
-0.636787446
-2.205237411
3.422194356
1.281191915
-0.848530615
-2.021830045
-1.34150015
-2.690843984
-0.796123819
-1.626050724
-2.47584614
-1.692140335
-2.660443165
0.396732093
-2.49054709
-2.333442286
-0.370818472
-3.054991439
3.441594365
1.465676148
-1.889960188
-0.637809871
-1.222278956
-1.333213449
-0.842261306
2.085359473
-1.378896605
-1.038407821
-2.201267253
-1.623060253
-1.843375351
-1.425352968
-1.553299157
2.23530045
-0.531585846
-2.061933335
-2.228156261
5.103417007
1.908876546
-1.809227956
-0.886315401

-2.472679838
0.070543043
-0.652807218
0.075421956
-1.94557407
-1.838795402
-0.883963383
0.086867123
0.217527213
1.346991013
-1.394209604
-2.761126084
-4.102908822
-2.827698765
-1.052827283
0.162378969
-1.731035047
0.296892763
-1.226176717
2.2697354
-1.6051107
-0.476714874
-0.465469885
-1.878234391
-3.37054643
-1.661104221
-0.044942168
0.835951793
-1.787023233
-1.666068834
-1.071788074
-1.392435657
-1.632614345
-0.260849536
-1.586468269
-1.906950267
-0.751535977
-2.206783727
-0.428010528
-2.376376394
-0.441927409
-1.455626525
-1.759067921
-1.237142898
-2.435541957
-0.689419392
-2.035431466
0.982228356
-2.826186419
-2.518180271

-0.596157275
-1.949505605
-0.607667907
0.869134815
-1.640760625
2.514384941
-0.843420125
-1.173182188
-2.168499326
-0.772420159
-0.131845774
-1.086030872
-1.520401251
-0.004477442
-0.372804834
-2.097681101
0.385958289
-1.499645956
-1.349675781
-1.60985835
1.472879385
-0.533467786
-2.175081595
-0.751637876
-1.415975991
-0.604629407
-1.693477736
-0.586846361
-1.996563704
1.554275252
3.659266767
-0.438519661
-0.056770402
0.010451231
1.413666788
-2.039800523
-1.006559307
0.689603714
-0.662169114
-1.767672739
-1.737421147
0.928847824
0.00916186
-0.024856031
-0.983457527
-0.336787958
-1.589593484
-1.986934325
-1.420212473
-0.991191308

-2.436123328
-1.981631447
-1.885058427
-1.929653182
-2.01303869
-0.883112897
1.242981711
0.008718054
-2.998352413
-0.758652216
-0.306219411
-0.956048109
-0.389136912
-0.505835682
-2.257133145
-0.860869392
-3.29013283
-0.816388082
-1.775287458
-1.182701927
-3.076311485
-4.365848659
-4.218366437
-0.155366664
-1.085651541
0.163526589
-1.980723964
-1.182744552
0.692157481
-0.586388464
-1.267499285
-0.999778434
0.866445084
-1.036395307
-1.219122481
-1.086071911
-2.046427095
-5.705307195
0.383613347
-1.077059953
-0.790210079
-2.744653923
-0.727737246
-1.864554011
0.209191071
-1.852779381
-1.411933269
-1.643722265
-0.635635804
-0.950008263

-0.21697831
-0.355881905
-0.246757623
