# Supplementary material for: Integrated transcriptomics- and structure-based drug repositioning identifies drugs with proteasome inhibitor properties
Source: Sci Rep. 2024 Aug 13;14:18772. doi: 10.1038/s41598-024-69465-6 (PMC11322189; doi:10.1038/s41598-024-69465-6)
Supplement: Supplementary file 12 — Supplementary Table S2. [file 41598_2024_69465_MOESM12_ESM.pdf]

Supplementary Table 2. Top 250 differentially regulated genes (log2) for MG-132 in the CMap dataset

Gene Symbol	CPC002_HA1E_6H.BRD.K60230970.001.04.3.10	CPC002_HCC515_6H.BRD.K60230970.001.04.3.10	CPC002_HCC515_24H.BRD.K60230970.001.04.3.1[illegible]	CPC002_PC3_6H.BRD.K60230970.001.04.3.10	CPC002_PC3_24H.BRD.K60230970.001.04.3.10	CPC002_VCAP_6H.BRD.K60230970.001.04.3.10	CPC002_VCAP_24H.BRD.K60230970.001.04.3.10	CPC017_A375_6H.BRD.K60230970.001.04.3.10	CPC017_A549_6H.BRD.K60230970.001.04.3.10	CPC017_A549_24H.BRD.K60230970.001.04.3.10	CPC017_ASC_24H.BRD.K60230970.001.04.3.10	CPC017_HEPG2_6H.BRD.K60230970.001.04.3.10	CPC017_HT29_6H.BRD.K60230970.001.04.3.10	CPC017_MCF7_6H.BRD.K60230970.001.04.3.10	CPC017_MCF7_24H.BRD.K60230970.001.04.3.10	CPC017_NPC_24H.BRD.K60230970.001.04.3.10	CPC017_PHH_24H.BRD.K60230970.001.04.3.10	CPC017_SKB_24H.BRD.K60230970.001.04.3.10	NMH002_FIBRNPC_24H.BRD.K60230970.001.09.[illegible]	MUC.CP002_MCF7_6H.BRD.K60230970.001.10.0.[illegible]	MUC.CP002_MCF7_6H.BRD.K60230970.001.10.0.[illegible]	MUC.CP002_MCF7_6H.BRD.K60230970.001.10.0.[illegible]	MUC.CP002_MCF7_6H.BRD.K60230970.001.10.0.[illegible]	[illegible]
ABCB6	-0.92	1.6	3.41	0.03	-1.46	-1.71	-7.77	-1.37	-3.66	0.11	-3.49	-0.89	-1.17	0.46	-2.16	2.83	-2.55	-0.98	-2.29	0.69	1.64	2.81	0.04	[illegible]
ABHD4	3.71	-0.32	4.76	2.04	5.38	3.32	7.11	5.53	3.03	1.46	0.37	6.47	9.61	6.87	7.83	1.8	1.82	-0.61	0.14	4.34	2.09	3.69	1.2	[illegible]
ADGRE5	1.65	-0.85	-3.39	-2.73	-5.04	-0.97	2.97	-3.77	-5.03	-2.59	-0.17	2.37	-4.94	-0.81	2.69	2.84	0.13	0.61	-1.06	0.98	0.37	2.77	-0.48	[illegible]
ADI1	-2.68	-5.27	-8.14	-1.74	-2.21	-1.15	-8.81	-2.67	-6.77	-0.97	-5.42	-1.96	-1.77	-2.91	-5.91	-1.84	-3.41	-0.77	-1.47	-0.85	-0.1	0.49	0.15	[illegible]
ADO	0.22	-2.13	-6.49	-3.75	-9.02	1.93	-6.78	-3.56	-0.22	-1.69	-0.45	-0.61	-4.24	0.04	1.71	-7.35	-0.54	-1.98	-4.38	-0.51	-0.72	-0.03	5.52	[illegible]
AKAP8L	3.62	3	5.19	2.42	2.34	0.61	-1.31	0.63	4.17	4.56	0.72	2.57	2.22	3.02	4.36	0.24	0.96	1.16	4.02	2.29	0.83	0.61	-0.46	[illegible]
AKR1C3	1.19	-1.03	-3.18	0.23	-0.02	0.09	-0.23	0.15	-1.86	-3.05	-2.17	0.39	0.31	0.3	-1.91	10	-0.34	-1.71	-2.11	-0.39	-0.95	-0.07	-0.59	[illegible]
AKR7A2	-2.02	-1.24	-5.91	-1.05	-4.9	-2.57	-8.94	-0.84	-0.8	-5.38	-6.53	-0.31	-2.17	-1.31	-7.19	-7.81	-0.21	-5.64	-7.31	-1.06	-1.2	-3.32	0.02	[illegible]
ALDH1A1	0.36	0.1	-2.08	0.95	0.32	0	0.58	0.2	-4.5	-5.5	-0.38	-0.96	-0.07	-0.11	-1.77	-1.31	-0.34	-1.33	-0.31	-0.56	0.23	0.21	-0.69	[illegible]
ALDOC	-0.54	1.99	4.96	0.25	-0.43	-0.15	5.76	1.04	0.38	-1.45	-5.94	-0.05	1.44	1.29	6.55	4.39	-1.27	0.64	7.22	1.18	-0.53	0.52	0.17	[illegible]
ANXA7	0.5	-1.71	-0.69	-2.49	-6.03	0.01	0.01	-0.68	2.29	1.38	0.56	1.76	1.39	2.63	-0.01	-2.53	-0.28	-3.92	-5.84	1.58	2	2.19	0.87	[illegible]
ARHGAP1	-1.18	-3.73	-1.36	-1.39	-3.24	-1.77	-1.12	-2.04	-4.33	-2.55	-8.12	-4.53	-3.86	-4.67	-3.45	0.11	0.18	-5.06	-8.05	-2	-2.41	0.19	-0.83	[illegible]
ARHGEF2	2.27	0.84	0.97	0.09	3.89	3.58	10	1.67	4.46	5.48	1.46	5.09	8.48	8.03	9.67	-0.83	1.49	-3.6	-2.4	5.46	3.64	0.58	0.18	[illegible]
ARID4B	2.07	6.97	6.4	2.47	8.2	2.14	7.72	-0.01	3.75	1.3	3.29	1.47	0.96	5.36	3.47	1.84	-0.25	2.25	2.9	1.45	0.77	0.62	-5.43	[illegible]
ASPH	-0.26	-0.6	-0.27	-2.08	-5.68	-1.72	-1.94	-0.72	-2.28	-2.15	-2.43	-1.29	-1.53	-0.74	1.25	-1.02	-3.51	-2.03	-1.18	-0.71	-0.21	0.08	-0.43	[illegible]
ATF5	-3.21	-10	-6.18	-4.07	2	-3.43	-1.23	-3.33	-0.06	-0.81	0.35	1.69	-4.21	-6.52	-4.32	0.32	-0.65	-1.69	-0.38	-6.28	-3.57	0.06	-0.11	[illegible]
AURKB	-3.55	-3.14	0.05	-5.1	-3.39	0.9	-0.98	0.02	-2.31	-6.55	-1.15	-2.34	0.22	-4.08	-2.25	-0.37	0.08	-0.23	0.1	-2.15	-2.24	-0.28	-0.45	[illegible]
B4GAT1	-3.52	-8.48	-8.4	-5.32	-1.31	-5.21	-8.71	-2.78	-6.92	-5.21	-3.23	-7.03	-4.18	-8	-5.24	-8.11	-1.19	-1.57	-2.59	-5.01	-4.37	-0.03	-1.86	[illegible]
[illegible]	[illegible]	[illegible]	[illegible]	[illegible]	[illegible]	[illegible]	[illegible]	[illegible]	[illegible]	[illegible]	[illegible]	[illegible]	[illegible]	[illegible]	[illegible]	[illegible]	[illegible]	[illegible]	[illegible]	[illegible]	[illegible]	[illegible]	[illegible]	[illegible]
