# Supplementary material for: Integrated transcriptomics- and structure-based drug repositioning identifies drugs with proteasome inhibitor properties
Source: Sci Rep. 2024 Aug 13;14:18772. doi: 10.1038/s41598-024-69465-6 (PMC11322189; doi:10.1038/s41598-024-69465-6)
Supplement: Supplementary file 13 — Supplementary Table S3. [file 41598_2024_69465_MOESM13_ESM.pdf]

Supplementary Table 3. Top 250 differentially regulated genes (log2) for MLN-2238 in the CMap dataset

Gene Symbol	CPC006_A375_6H.BRD.K78659596.001.01.3.10	CPC006_A549_6H.BRD.K78659596.001.01.3.10	CPC006_A549_24H.BRD.K78659596.001.01.3.10	CPC006_A673_6H.BRD.K78659596.001.01.3.10	CPC006_AGS_6H.BRD.K78659596.001.01.3.10	CPC006_CL34_6H.BRD.K78659596.001.01.3.10	CPC006_CORL23_6H.BRD.K78659596.001.01.3.10	CPC006_COV644_6H.BRD.K78659596.001.01.3.10	CPC006_DV90_6H.BRD.K78659596.001.01.3.10	CPC006_EFO27_6H.BRD.K78659596.001.01.3.10	CPC006_HA1E_6H.BRD.K78659596.001.01.3.10	CPC006_HA1E_24H.BRD.K78659596.001.01.3.10	CPC006_HCC15_6H.BRD.K78659596.001.01.3.10	CPC006_HCC515_6H.BRD.K78659596.001.01.3.10	CPC006_HCC515_24H.BRD.K78659596.001.01.3.1	CPC006_HCT116_6H.BRD.K78659596.001.01.3.10	CPC006_HEC108_6H.BRD.K78659596.001.01.3.10	CPC006_HEPG2_6H.BRD.K78659596.001.01.3.10	CPC006_HT115_6H.BRD.K78659596.001.01.3.10	CPC006_HT29_6H.BRD.K78659596.001.01.3.10	CPC006_HT29_24H.BRD.K78659596.001.01.3.10	CPC006_JHUEM2_6H.BRD.K78659596.001.01.3.10	CPC006_LOVO_6H.BRD.K78659596.001.01.3.10	CPC006_MCF7_6H.BRD.K78659596.001.01.3.10	CPC006_MCF7_24H.BRD.K78659596.001.01.3.10	CPC006_MDST8_6H.BRD.K78659596.001.01.3.10	CPC006_NCIH1694_6H.BRD.K78659596.001.01.3.1	CPC006_NCIH1836_6H.BRD.K78659596.001.01.3.1	CPC006_NCIH2073_6H.BRD.K78659596.001.01.3.1	CPC006_NCIH508_6H.BRD.K78659596.001.01.3.10	CPC006_NCIH596_6H.BRD.K78659596.001.01.3.10	CPC006_NOMO1_6H.BRD.K78659596.001.01.3.10	CPC006_OV7_6H.BRD.K78659596.001.01.3.10	CPC006_PC3_6H.BRD.K78659596.001.01.3.10	CPC006_PC3_24H.BRD.K78659596.001.01.3.10
ABL1	0.18	0.03	0.46	0.75	3.09	-4.17	1.12	1.43	1.1	2.33	-0.06	1.22	-0.59	0.75	0.74	1.04	2.28	1.96	-0.01	0.56	-0.97	-1.65	0.21	1.86	1.28	1.85	0.92	1.79	0.42	-0.03	-1.32	1.82	-1.1	1.03	0.85
ADGRE5	-3.27	-0.83	-3.41	1.23	-1.36	-3.05	-2.83	1.44	-1.23	-3.21	1.29	1.13	-3.42	-0.72	-5.28	-1.38	-1.03	0.98	-1.3	-3.71	-6	1.28	-2.37	-0.67	1.44	0.3	-0.13	1.44	-2.4	-0.1	-2.95	-1.32	-2.85	-3.72	-5.84
ADO	-3.22	-0.75	-3.44	-1.28	-1.27	-0.6	-0.26	0.67	-1.98	3.84	0.75	-2.96	1.02	-1.93	-4.87	-1.84	-1.59	-0.97	-1.56	-2.85	-7.41	4.41	-0.56	-0.07	0.7	-1.57	-0.22	-1.74	-0.65	-0.36	-2.43	0.12	-2.16	-5.08	-8.5
ADRB2	-1.83	-1.31	0.36	1.34	-1.28	2.93	-1.09	1.05	4.94	0.37	1.36	-2.09	-2.55	-0.47	0.41	6.7	0.31	-0.43	-1.61	0.27	-1.58	-0.71	-2.48	-0.33	0.83	-0.32	-0.51	0.39	0.57	0.02	5.24	-0.68	1.01	-2.85	-1.91
AGR2	0	-0.08	-1.64	0.86	-0.41	-0.08	3.94	0.09	0.08	0.09	1.27	0	3.03	-0.44	-0.54	0	0	0.23	0.25	0.04	-1.19	0	-0.7	-0.94	-2.79	4.86	1.35	-0.53	-1.01	0.25	-1.51	5	5	-0.68	-0.8
AKR7A2	-0.8	-0.44	-5.36	-2.73	-2.54	-5.5	0.21	-10	-0.44	-1.13	-1.46	-7.09	0.52	-0.77	-6.91	-0.66	-1.8	-1.52	-0.67	-2	-7.51	-2.44	-3.39	-2.65	-7.55	-1.1	-1.47	-1.72	-0.81	-2.6	-0.59	-7.15	-1.43	-0.39	-5.84
ARHGDIB	-0.74	0.24	0.44	0.03	-0.59	0.98	-1.4	-1.26	1.17	-0.43	0.29	0.09	-1.4	-0.59	-0.88	-0.84	-0.82	-0.06	-0.73	-1.04	-1.35	-0.24	-1.3	-1.54	-1.06	-0.33	-0.93	0.02	-0.25	-1.41	2.1	-10	0.5	-0.99	-0.3
ARHGEF2	-0.03	0.93	1.44	0.68	2.97	2.06	2.63	1.94	2.11	1.2	0.69	1.28	0.48	0.69	2.21	0.18	1.69	1.55	2.78	3.96	3.21	0.86	4.69	7.03	8.21	1.41	-3.02	2.03	1.36	6.59	0.74	-1.6	-0.22	0.12	3.49
ARL4C	-0.69	-2.62	1.68	0.43	0.35	2.42	-0.92	-0.32	0.62	-0.44	-1.12	2.44	-5.82	-2.87	-1.52	0.1	-1.03	-0.14	0.98	-1.37	1.5	1.19	-0.42	-0.06	1.91	-4.5	-0.75	0.35	-1.54	-0.56	0.54	1.45	-0.45	0.78	-0.77
ARPP19	-1.4	-0.6	-2.26	0.6	0.16	-3.51	0.46	-0.88	0.7	0.49	-3.01	-10	1.24	2.33	-7.96	-1.02	0.86	2.09	0.21	-0.12	2.35	-1.53	-1.38	-1.99	1.62	0.62	0.62	-1.93	0.89	2.13	0.65	-1.11	0.68	-2.66	5.43
ASS1	0.79	0.8	-0.59	0.66	0.11	-10	-0.25	-10	0.29	0.62	0.68	1.58	1.5	1.53	-0.95	0.43	-0.36	0.44	-2.5	-0.11	0.53	0.88	0.06	0.23	0.2	2.49	0.85	0.16	-0.05	0.34	0.86	2.35	0.52	0.68	0.37
ATF5	-3.34	-0.91	-2.23	-0.16	-0.83	0.32	-0.91	-0.61	0.06	-0.24	-5.02	1.52	-1.12	-5.23	-4.14	-2.3	-1.83	0.79	-0.03	-2.84	0.34	-2.37	-0.39	-3.01	-1.58	-8.52	1.69	0.47	-1.51	-1.62	-1.19	0.47	-1.1	-0.53	1.67
ATP6V1D	-1.03	0.59	1.28	-4.25	1.13	0.59	-0.06	0.63	1.15	0.13	-0.83	0.69	-5.51	1.82	1.86	-1.06	-3.03	0.85	0.98	0.4	-1.22	1.34	0.11	-1.14	-0.57	1.42	1.24	0.29	1.52	0.41	0.74	-1.28	1.22	0.46	2.19
AURKB	-1.42	-2.46	-2.75	-4.56	-4.17	1.98	-0.52	-0.05	-1.74	-2.37	-1.99	-6.19	-0.63	-1.61	-4.53	-0.08	0.21	-1.14	-2.83	-1.6	-7.18	-0.47	0.27	-1.85	-7.49	-1.1	-1.41	0.69	-3.79	-0.29	1.7	-0.37	0.56	-1.86	-8.15
BACE2	-0.68	0.43	0.81	-2.99	-0.58	-1.9	-0.42	-10	1.07	0.6	-1.34	-4.69	0.93	0.79	-0.2	3.26	-0.42	0.66	-0.47	-0.27	0.41	1.26	0.5	-0.21	0.53	-0.91	-0.7	0.16	0	-0.44	-0.73	5.15	-0.86	-0.67	-3.01
BAD	-2.34	-3.18	-0.68	-0.38	-0.29	-1.84	-4.21	-7.1	0.46	-0.18	-0.55	1.01	-5.19	-0.36	-1.04	-2.59	0.03	1.11	-0.07	-0.08	-0.32	-1.06	-0.3	-0.94	-0.06	-0.44	0.07	-1.9	-1.3	-0.49	-4.7	-0.6	-0.43	-3.01	-1.85
BAG3	5.2	7.08	4.9	4.6	8.43	4.65	5.93	3.41	5.22	4.39	6.49	7	5.49	8.06	8.02	1.89	4.32	3.4	3.53	8.29	9.65	2.77	2.28	3.7	1.94	7.88	2.03	3.03	7.45	5.59	4.33	1.53	5.05	3.54	7.37
BAMBI	0.62	1.51	-0.16	3.09	-1.22	2.67	2.21	0.61	1.88	0.4	0.58	2.65	2.78	2.74	4.22	4.4	-0.29	-0.02	1.52	1.9	0.86	0.36	1.89	-1.27	-5.84	1.98	-0.26	0.61	1.52	0.51	-0.08	-0.08	-0.07	3.29	8.29
BDH1	-1.75	-0.73	0.83	-2.19	2.77	-2.08	-0.69	0.36	-1.72	-2.52	-0.8	-3.41	-0.16	0.32	-0.78	-1.6	0.64	-2.63	-0.86	-2.62	-7.4	-0.9	-2.62	-3.82	-4.75	-1.12	-0.42	-0.83	-1.14	-3.27	-1.59	-0.79	1.28	-1.84	-3.12
BHLHE40	-0.6	-0.11	0	1.78	1.96	-0.51	0.93	0.98	-0.56	-0.81	-0.31	-2.65	1.55	1.9	2.36	-0.5	-0.11	-0.04	0.56	-0.01	0.41	-0.6	-0.37	1.25	3.46	6.3	0.2	1.34	-0.53	-0.28	-1.11	0.99	-0.36	-1.61	-1.2
BIRC5	-0.66	-1.82	-3.55	-1.94	-5	-1.46	-0.67	0.58	-2.04	-2.7	-1.63	-5.08	-1.05	-0.5	-5.51	-0.65	-3.73	-2.24	-2.34	-2.96	-7.19	-2.37	-2.12	-0.99	-9.02	-2.46	-1.26	-0.47	-3.3	-1.41	-0.88	-0.43	-2.3	-2.57	-5.77
BMP4	0.35	0.49	0.26	0.26	0.21	-5.38	-1.03	1.54	-0.44	0.35	0.55	1.16	-1.65	-1.36	-0.69	-0.41	0.27	-2.72	-2.94	-7.98	-7.88	-1.97	-2.62	0.13	1.18	0.1	-0.38	0.01	-0.36	-1.01	0.3	0.98	0.87	0.66	3.48
BNIP3	-0.17	1.27	0.93	-1.26	-0.45	5.48	-0.37	0.27	-0.74	-2.04	-0.43	-4.24	-0.14	1.95	2.23	-1.4	-1.43	-0.51	-0.9	0.11	1.66	-0.23	-0.6	-0.3	2.9	0.11	0.19	-0.86	-0.74	-0.39	-0.41	1.72	0.47	-0.56	-4.49
C2CD5	-0.76	-1.26	-4.69	-4.25	-2.56	-6.18	-3.55	-10	-1.87	-1.99	-2.92	-4.78	-2.14	-4.13	-6.79	-1.43	-1.32	-3.04	-1.68	-4.31	-8.26	-2.13	-2.18	-1.66	-7.35	-1.93	-1.43	-2	-2.68	-4.1	-1.14	-5.14	-2.86	-1.75	-8.2
CADM1	0.01	1.31	1.74	-0.94	1.49	1.06	0.49	-2.2	2.11	0.89	0.82	-0.6	1.02	0.17	-0.71	0.83	1.42	-0.79	0.54	1.9	8.16	0.14	0.46	0.51	1.1	-0.26	-0.53	1.06	-1.07	0.45	0.26	0.62	1.39	2.05	8.88
CANT1	-1.65	-1.32	-0.03	-2.11	-0.48	-5.79	0.11	-1.95	-1.99	-2.05	-3.65	-0.07	0.19	-1.54	-4.3	-5.49	-3.31	-0.54	-6.73	-3.76	-3.78	-0.66	-2.85	-2.65	-3.7	-2.87	-0.7	-1.95	-0.74	-0.68	-1.25	-0.44	-0.83	-2.53	-3.92
CAPN2	0.4	-0.98	-0.28	0.42	-0.2	-0.93	-4.88	0	0.38	0.07	-0.11	-0.13	0	-1.18	-0.45	-1.04	-0.04	0.35	0.98	0.47	2.13	-0.33	-1.05	0.92	2.66	-0.22	0.74	-0.36	0.95	1.48	-0.05	-1.3	0.44	0.52	-0.25
CASP2	0.69	-0.6	0.45	0.28	0.78	1.27	0.35	-0.39	-0.25	0.73	0.54	-0.63	-0.13	-0.04	-1.19	10	0.11	-0.26	-0.85	1.26	0.26	0	5.08	-0.05	-0.16	-0.09	-0.1	-0.14	-0.91	-1.24	-0.13	0.19	-0.56	-0.29	2.7
CBR3	-1.34	1.97	-1.05	-0.59	0.13	0.83	0.89	1.51	-0.1	0.63	-2.41	-8.7	0.93	1.56	3.04	0.51	0.41	1.04	-0.39	4.53	4.25	-0.22	1.77	1	5.31	-0.77	0.23	1.86	1.9	5.32	0.22	-2.7	2.51	0.59	-2.07
CCDC85B	-3.98	-2.98	-4.17	-5.89	-2.25	0.48	-6.71	-0.32	-1.77	-1.71	-2.66	-3.53	-2.28	-1.07	-5.64	0.29	-2.67	-3.62	-0.59	-2.84	-3.92	-2.21	-5.95	-2.56	-4.35	-3.35	-0.92	0.64	-2.85	-0.38	-3.18	0.06	-3.2	-6.88	-5.89

[illegible]
