# Supplementary material for: Integrated transcriptomics- and structure-based drug repositioning identifies drugs with proteasome inhibitor properties
Source: Sci Rep. 2024 Aug 13;14:18772. doi: 10.1038/s41598-024-69465-6 (PMC11322189; doi:10.1038/s41598-024-69465-6)
Supplement: Supplementary file 14 — Supplementary Table S4. [file 41598_2024_69465_MOESM14_ESM.pdf]

Supplementary Table 4. Perturbagens (compounds or gene knock-down) for MG-132 with CMap *tau* -scores ≥ 95

Rank	Score	Type	ID	Name	Description
1	99.98	Compound	BRD-K60230970	MG-132	Proteasome inhibitor
3	99.89	Compound	BRD-K78659596	MLN-2238	Proteasome inhibitor
4	99.89	Compound	BRD-K15935639	z-leu3-VS	Proteasome inhibitor
6	99.79	Compound	BRD-A28970875	puromycin	Protein synthesis inhibitor
8	99.75	Compound	BRD-K74402642	NSC-632839	Ubiquitin specific protease inhibitor
10	99.68	Compound	BRD-K33551950	radicicol	HSP inhibitor
11	99.65	Compound	BRD-K78599730	manumycin-a	Farnesyltransferase inhibitor
12	99.65	Compound	BRD-K07303502	arachidonyl-trifluoro-methane	Cytosolic phospholipase inhibitor
13	99.65	Compound	BRD-A55484088	BNTX	Opioid receptor antagonist
14	99.65	Compound	BRD-A11007541	BCI-hydrochloride	Protein phosphatase inhibitor
15	99.61	Compound	BRD-A20697603	thiostrepton	FOXM1 inhibitor
20	99.44	Compound	BRD-K80970344	pyrrolidine-dithiocarbamate	NFkB pathway inhibitor
22	99.33	Compound	BRD-K51290057	SA-792709	Retinoid receptor agonist
24	99.29	Compound	BRD-K36737713	AG-957	Protein tyrosine kinase inhibitor
25	99.19	Compound	BRD-A28105619	cucurbitacin-i	JAK inhibitor
26	99.08	Compound	BRD-K31238592	devazepide	CCK receptor antagonist
29	98.87	Compound	BRD-U08759356	EI-346-erlotinib-analog	EGFR inhibitor
31	98.78	Compound	BRD-K14821540	FCCP	Mitochondrial oxidative phosphorylation uncoupler
32	98.76	Compound	BRD-K89930444	AG-592	Tyrosine kinase inhibitor
33	98.73	Compound	BRD-K44432556	VU-0418946-1	HIF modulator
36	98.7	Compound	BRD-K76907295	VU-0418947-2	HIF modulator
37	98.7	Compound	BRD-K24132293	piperlongumine	Glutathione transferase inhibitor
38	98.7	Compound	BRD-K22010301	JLK-6	Gamma secretase inhibitor
39	98.7	Compound	BRD-K17705806	JTC-801	Opioid receptor antagonist
40	98.7	Compound	BRD-K13169950	NSC-3852	HDAC inhibitor
41	98.7	Compound	BRD-A52193669	withaferin-a	IKK inhibitor
42	98.67	Compound	BRD-A83326220	brazilin	Nitric oxide production inhibitor
43	98.66	Compound	BRD-A50737080	CGK-733	ATR kinase inhibitor
45	98.62	Compound	BRD-K05396879	15-delta-prostaglandin-j2	PPAR receptor agonist
46	98.59	Compound	BRD-K83988098	alvespimycin	HSP inhibitor
47	98.59	Compound	BRD-K31912990	CGP-71683	Neuropeptide receptor antagonist
49	98.48	Compound	BRD-K73395020	SA-1478088	-
50	98.48	Compound	BRD-K26669427	WR-216174	PFMRK inhibitor
52	98.45	Compound	BRD-K17075857	chloroxine	Opioid receptor antagonist
54	98.41	Compound	BRD-K51730347	diphencyprone	Immunostimulant
55	98.41	Compound	BRD-K03109492	NSC-663284	CDC inhibitor
56	98.38	Compound	BRD-K38477985	malonoben	Protein tyrosine kinase inhibitor
58	98.31	Compound	BRD-K64517075	heliomycin	ATP synthase inhibitor
59	98.31	Compound	BRD-A78360835	cercosporin	Photoactivated toxin
62	98.27	Compound	BRD-K74305673	IKK-2-inhibitor-V	IKK inhibitor
63	98.24	Compound	BRD-K28907958	CD-437	Retinoid receptor agonist
66	98.07	Compound	BRD-K40255344	tyrphostin-A9	Protein tyrosine kinase inhibitor
67	98.06	Compound	BRD-M86331534	pyrvinium-pamoate	AKT inhibitor
68	98.06	Compound	BRD-A38030642	cyclosporin-a	Calcineurin inhibitor
69	97.99	Compound	BRD-K17497770	butein	EGFR inhibitor
71	97.96	Compound	BRD-K51967704	BIIB021	HSP inhibitor
72	97.96	Compound	BRD-K20755323	SA-792728	Sphingosine kinase inhibitor
74	97.94	Compound	BRD-K67844266	MLN-4924	Nedd activating enzyme inhibitor
76	97.85	Compound	BRD-K10573841	tunicamycin	GLCNAC phosphotransferase inhibitor
77	97.83	Compound	BRD-K98548675	parthenolide	NFkB pathway inhibitor
78	97.78	Compound	BRD-K03406345	azacitidine	DNA methyltransferase inhibitor
79	97.74	Compound	BRD-K39120595	bithionol	Autotaxin inhibitor
80	97.74	Compound	BRD-K35960502	niclosamide	DNA replication inhibitor
81	97.74	Compound	BRD-K15616905	CCCP	Mitochondrial oxidative phosphorylation uncoupler
82	97.67	Compound	BRD-K15409150	penfluridol	T-type calcium channel blocker
84	97.64	Compound	BRD-K72895815	SSR-69071	Leukocyte elastase inhibitor
85	97.6	Compound	BRD-K82135108	elesclomol	Oxidative stress inducer
86	97.6	Compound	BRD-K39111395	BCL2-inhibitor	BCL inhibitor
87	97.57	Compound	BRD-K88677950	PD-198306	MAP kinase inhibitor
88	97.5	Compound	BRD-K88868628	iodoacetic-acid	Cysteine peptidase inhibitor
89	97.5	Compound	BRD-K32744045	disulfiram	Aldehyde dehydrogenase inhibitor
90	97.5	Compound	BRD-K21672174	RO-28-1675	Glucokinase activator
91	97.38	Compound	BRD-A34205397	suloctidil	Adrenergic receptor antagonist
92	97.29	Compound	BRD-K66792149	quinoclamine	Algicide
93	97.29	Compound	BRD-A58564983	selamectin	Nematocide
95	97.15	Compound	BRD-K17140735	SCH-79797	Proteasome inhibitor
97	97.11	Compound	BRD-K41859756	NVP-AUY922	HSP inhibitor
98	97.08	Compound	BRD-K98490050	amsacrine	Topoisomerase inhibitor
99	97.08	Compound	BRD-A98283014	calmidazolium	Calcium channel blocker
100	97.04	Compound	BRD-K59469039	AG-879	Angiogenesis inhibitor
101	96.93	Compound	BRD-K94325918	kinetin-riboside	Apoptosis stimulant
102	96.91	Compound	BRD-K15600710	obatoclax	BCL inhibitor
104	96.9	Compound	BRD-K78122587	NNC-55-0396	T-type calcium channel blocker
105	96.9	Compound	BRD-K08417745	SID-26681509	Cathepsin inhibitor
106	96.9	Compound	BRD-K06426971	ryuvidine	Histone lysine methyltransferase inhibitor
107	96.86	Compound	BRD-K78126613	menadione	Mitochondrial DNA polymerase inhibitor
108	96.83	Compound	BRD-K47150025	KI-8751	VEGFR inhibitor
109	96.83	Compound	BRD-A62809825	thapsigargin	ATPase inhibitor
111	96.55	Compound	BRD-K74133369	oligomycin-a	ATP synthase inhibitor
112	96.55	Compound	BRD-K21806131	tegaserod	Serotonin receptor partial agonist
114	96.5	Compound	BRD-A79465854	auranofin	NFkB pathway inhibitor
115	96.49	Compound	BRD-A56020723	CA-074-Me	Cathepsin inhibitor
116	96.31	Compound	BRD-K06593056	LE-135	Retinoid receptor agonist
119	96.01	Compound	BRD-K03816923	rottlerin	MAP kinase inhibitor
120	95.98	Compound	BRD-A17065207	brefeldin-a	Protein synthesis inhibitor
122	95.95	Compound	BRD-K24681473	YM-155	Survivin inhibitor
124	95.81	Compound	BRD-K62289640	lylamine	Cannabinoid receptor agonist
125	95.81	Compound	BRD-K19295594	gossypol	BCL inhibitor
126	95.81	Compound	BRD-A82371568	clofarabine	Ribonucleoside reductase inhibitor
129	95.67	Compound	BRD-A08003242	rhodomyrtoxin-b	sodium fluorescein uptake inhibitor
130	95.64	Compound	BRD-K15025317	BAY-11-7821	NFkB pathway inhibitor
131	95.63	Compound	BRD-K37865504	LY-2183240	FAAH inhibitor
132	95.62	Compound	BRD-K30296925	flavokavain-b	Antineoplastic
133	95.59	Compound	BRD-K37691127	hinokitiol	Tyrosinase inhibitor
134	95.5	Compound	BRD-K66175015	afatinib	EGFR inhibitor
137	95.38	Compound	BRD-K36198571	WAY-170523	Metalloproteinase inhibitor
138	95.36	Compound	BRD-A19500257	geldanamycin	HSP inhibitor
139	95.35	Compound	BRD-K88625236	nonoxynol-9	Membrane integrity inhibitor
142	95.1	Compound	BRD-K44849676	capsazepine	TRPV agonist
144	95.07	Compound	BRD-K68143200	SA-792541	CDC inhibitor
145	95.07	Compound	BRD-A06352418	terfenadine	Histamine receptor antagonist
27	98.98	Gene knock-down	CGS001-5682	PSMA1	Proteasome subunits
61	98.27	Gene knock-down	CGS001-5684	PSMA3	Proteasome subunits
64	98.18	Gene knock-down	CGS001-5707	PSMD1	Proteasome subunits
73	97.95	Gene knock-down	CGS001-5690	PSMB2	Proteasome subunits
94	97.25	Gene knock-down	CGS001-3309	HSPA5	Heat shock proteins / HSP70
103	96.9	Gene knock-down	CGS001-7316	UBC	-
117	96.15	Gene knock-down	CGS001-8892	EIF2B2	-
121	95.95	Gene knock-down	CGS001-5693	PSMB5	Proteasome subunits
127	95.77	Gene knock-down	CGS001-7415	VCP	ATPases / AAA-type
128	95.77	Gene knock-down	CGS001-11331	PHB2	-
136	95.45	Gene knock-down	CGS001-5709	PSMD3	Proteasome (prosome. macropain) subunits
141	95.23	Gene knock-down	CGS001-8894	EIF2S2	Serine/threonine phosphatases / Protein phosphatase 1. regulatory subunits
