# Supplementary material for: Integrated transcriptomics- and structure-based drug repositioning identifies drugs with proteasome inhibitor properties
Source: Sci Rep. 2024 Aug 13;14:18772. doi: 10.1038/s41598-024-69465-6 (PMC11322189; doi:10.1038/s41598-024-69465-6)
Supplement: Supplementary file 15 — Supplementary Table S5. [file 41598_2024_69465_MOESM15_ESM.pdf]

Supplementary Table 5. Perturbagens (compounds or gene knock-down) for MLN-2238 with CMap *tau* -scores ≥ 95

Rank	Score	Type	ID	Name	Description
2	99.97	Compound	BRD-K78659596	MLN-2238	Proteasome inhibitor
3	99.93	Compound	BRD-K60230970	MG-132	Proteasome inhibitor
4	99.93	Compound	BRD-K15935639	z-leu3-VS	Proteasome inhibitor
5	99.89	Compound	BRD-K74402642	NSC-632839	Ubiquitin specific protease inhibitor
7	99.86	Compound	BRD-K78599730	manumycin-a	Farnesyltransferase inhibitor
9	99.82	Compound	BRD-K07303502	arachidonyl-trifluoro-methane	Cytosolic phospholipase inhibitor
11	99.75	Compound	BRD-A28970875	puromycin	Protein synthesis inhibitor
12	99.72	Compound	BRD-A20697603	thiostrepton	FOXM1 inhibitor
13	99.72	Compound	BRD-A11007541	BCI-hydrochloride	Protein phosphatase inhibitor
15	99.65	Compound	BRD-A55484088	BNTX	Opioid receptor antagonist
17	99.53	Compound	BRD-K33551950	radicicol	HSP inhibitor
20	99.3	Compound	BRD-K24132293	piperlongumine	Glutathione transferase inhibitor
21	99.3	Compound	BRD-A28105619	cucurbitacin-i	JAK inhibitor
22	99.26	Compound	BRD-K13169950	NSC-3852	HDAC inhibitor
23	99.22	Compound	BRD-K36737713	AG-957	Protein tyrosine kinase inhibitor
25	99.19	Compound	BRD-K51290057	SA-792709	Retinoid receptor agonist
26	99.12	Compound	BRD-K51730347	diphencyprone	Immunostimulant
27	99.12	Compound	BRD-K31238592	devazepide	CCK receptor antagonist
31	99.05	Compound	BRD-K80970344	pyrrolidine-dithiocarbamate	NFkB pathway inhibitor
32	99.04	Compound	BRD-A83326220	brazilin	Nitric oxide production inhibitor
34	98.77	Compound	BRD-K98548675	parthenolide	NFkB pathway inhibitor
36	98.73	Compound	BRD-U08759356	EI-346-erlotinib-analog	EGFR inhibitor
37	98.72	Compound	BRD-K17705806	JTC-801	Opioid receptor antagonist
38	98.7	Compound	BRD-K67844266	MLN-4924	Nedd activating enzyme inhibitor
39	98.7	Compound	BRD-A78360835	cercosporin	Photoactivated toxin
42	98.66	Compound	BRD-K51967704	BIIB021	HSP inhibitor
43	98.66	Compound	BRD-K22010301	JLK-6	Gamma secretase inhibitor
44	98.66	Compound	BRD-A52193669	withaferin-a	IKK inhibitor
45	98.56	Compound	BRD-K89930444	AG-592	Tyrosine kinase inhibitor
48	98.46	Compound	BRD-K03109492	NSC-663284	CDC inhibitor
49	98.45	Compound	BRD-K88868628	iodoacetic-acid	Cysteine peptidase inhibitor
50	98.45	Compound	BRD-K82135108	elesclomol	Oxidative stress inducer
51	98.45	Compound	BRD-K41859756	NVP-AUY922	HSP inhibitor
52	98.45	Compound	BRD-A50737080	CGK-733	ATR kinase inhibitor
53	98.41	Compound	BRD-K39111395	BCL2-inhibitor	BCL inhibitor
54	98.41	Compound	BRD-K05396879	15-delta-prostaglandin-j2	PPAR receptor agonist
57	98.27	Compound	BRD-K76907295	VU-0418947-2	HIF modulator
58	98.27	Compound	BRD-K74305673	IKK-2-inhibitor-V	IKK inhibitor
59	98.27	Compound	BRD-K73395020	SA-1478088	-
60	98.27	Compound	BRD-K44432556	VU-0418946-1	HIF modulator
61	98.27	Compound	BRD-K17075857	chloroxine	Opioid receptor antagonist
63	98.24	Compound	BRD-K94325918	kinetin-riboside	Apoptosis stimulant
64	98.24	Compound	BRD-K83988098	alvespimycin	HSP inhibitor
65	98.24	Compound	BRD-K64517075	heliomycin	ATP synthase inhibitor
66	98.24	Compound	BRD-K40255344	tyrphostin-A9	Protein tyrosine kinase inhibitor
67	98.24	Compound	BRD-K17497770	butein	EGFR inhibitor
68	98.2	Compound	BRD-K14821540	FCCP	Mitochondrial oxidative phosphorylation uncoupler
69	98.17	Compound	BRD-K66792149	quinoclamine	Algicide
70	98.17	Compound	BRD-K15025317	BAY-11-7821	NFkB pathway inhibitor
71	98.13	Compound	BRD-A34205397	suloctidil	Adrenergic receptor antagonist
74	98.1	Compound	BRD-K31912990	CGP-71683	Neuropeptide receptor antagonist
75	98.06	Compound	BRD-K38477985	malonoben	Protein tyrosine kinase inhibitor
76	98.02	Compound	BRD-M86331534	pyrvinium-pamoate	AKT inhibitor
79	97.85	Compound	BRD-K35960502	niclosamide	DNA replication inhibitor
80	97.82	Compound	BRD-K30296925	flavokavain-b	Antineoplastic
81	97.82	Compound	BRD-A56020723	CA-074-Me	Cathepsin inhibitor
82	97.78	Compound	BRD-K17140735	SCH-79797	Proteasome inhibitor
83	97.78	Compound	BRD-A62809825	thapsigargin	ATPase inhibitor
84	97.77	Compound	BRD-K20755323	SA-792728	Sphingosine kinase inhibitor
86	97.74	Compound	BRD-K26669427	WR-216174	PFMRK inhibitor
87	97.71	Compound	BRD-K03406345	azacitidine	DNA methyltransferase inhibitor
88	97.57	Compound	BRD-A79465854	auranofin	NFkB pathway inhibitor
89	97.53	Compound	BRD-K61829047	7b-cis	Exportin antagonist
92	97.46	Compound	BRD-A98283014	calmidazolium	Calcium channel blocker
93	97.43	Compound	BRD-K47150025	KI-8751	VEGFR inhibitor
94	97.43	Compound	BRD-K10573841	tunicamycin	GLCNAC phosphotransferase inhibitor
95	97.42	Compound	BRD-K21672174	RO-28-1675	Glucokinase activator
96	97.35	Compound	BRD-K36529613	PU-H71	HSP inhibitor
98	97.32	Compound	BRD-K06426971	ryuvidine	Histone lysine methyltransferase inhibitor
99	97.3	Compound	BRD-K15409150	penfluridol	T-type calcium channel blocker
101	97.29	Compound	BRD-K32744045	disulfiram	Aldehyde dehydrogenase inhibitor
102	97.29	Compound	BRD-A19500257	geldanamycin	HSP inhibitor
105	97.15	Compound	BRD-K28120222	parthenolide	NFkB pathway inhibitor
106	97.13	Compound	BRD-K72895815	SSR-69071	Leukocyte elastase inhibitor
107	97.11	Compound	BRD-K56700933	phenethyl-isothiocyanate	Antineoplastic
108	97.11	Compound	BRD-K33583600	isoliquiritigenin	Guanylate cyclase activator
109	97.11	Compound	BRD-K28907958	CD-437	Retinoid receptor agonist
110	97.11	Compound	BRD-A58564983	selamectin	Nematocide
114	96.86	Compound	BRD-K21806131	tegaserod	Serotonin receptor partial agonist
116	96.83	Compound	BRD-K24681473	YM-155	Survivin inhibitor
117	96.83	Compound	BRD-K08417745	SID-26681509	Cathepsin inhibitor
118	96.8	Compound	BRD-K44849676	capsazepine	TRPV agonist
119	96.8	Compound	BRD-K28296557	AKT-inhibitor-IV	AKT inhibitor
121	96.72	Compound	BRD-K15616905	CCCP	Mitochondrial oxidative phosphorylation uncoupler
125	96.62	Compound	BRD-K39120595	bithionol	Autotaxin inhibitor
126	96.59	Compound	BRD-K78122587	NNC-55-0396	T-type calcium channel blocker
127	96.58	Compound	BRD-K78126613	menadione	Mitochondrial DNA polymerase inhibitor
128	96.58	Compound	BRD-A64228451	terreic-acid	BTK inhibitor
130	96.51	Compound	BRD-A25775766	securinine	GABA receptor antagonist
132	96.48	Compound	BRD-A38030642	cyclosporin-a	Calcineurin inhibitor
133	96.26	Compound	BRD-A17065207	brefeldin-a	Protein synthesis inhibitor
134	96.21	Compound	BRD-K68143200	SA-792541	CDC inhibitor
135	96.17	Compound	BRD-K03816923	rottlerin	MAP kinase inhibitor
138	96.07	Compound	BRD-K88625236	nonoxynol-9	Membrane integrity inhibitor
139	96.05	Compound	BRD-K36198571	WAY-170523	Metalloproteinase inhibitor
140	96.05	Compound	BRD-A41451487	PK-11195	Benzodiazepine receptor antagonist
141	96.02	Compound	BRD-U68942961	JW-7-24-1	LCK Inhibitor
142	95.99	Compound	BRD-K66175015	afatinib	EGFR inhibitor
143	95.94	Compound	BRD-K04853698	LDN-193189	Serine/threonine kinase inhibitor
144	95.93	Compound	BRD-A15079084	phorbol-12-myristate-13-acetate	PKC activator
145	95.9	Compound	BRD-A08003242	rhodomyrtoxin-b	sodium fluorescein uptake inhibitor
146	95.88	Compound	BRD-K74133369	oligomycin-a	ATP synthase inhibitor
147	95.88	Compound	BRD-K65503129	HSP90-inhibitor	HSP inhibitor
148	95.84	Compound	BRD-K88677950	PD-198306	MAP kinase inhibitor
149	95.84	Compound	BRD-A89434049	sarmentogenin	ATPase inhibitor
152	95.62	Compound	BRD-A82371568	clofarabine	Ribonucleoside reductase inhibitor
153	95.59	Compound	BRD-K55420858	mirin	MRE11A exonuclease inhibitor
156	95.53	Compound	BRD-K68336408	tyrphostin-AG-1478	EGFR inhibitor
157	95.53	Compound	BRD-K35573744	erbstatin-analog	EGFR inhibitor
161	95.42	Compound	BRD-K06593056	LE-135	Retinoid receptor agonist
162	95.42	Compound	BRD-A43150328	penicillic-acid	other antibiotic
169	95.21	Compound	BRD-K37865504	LY-2183240	FAAH inhibitor
171	95.17	Compound	BRD-K28168037	fenretinide	Apoptosis stimulant
172	95.17	Compound	BRD-K14618467	IKK-16	IKK inhibitor
173	95.17	Compound	BRD-A73741725	exemestane	Aromatase inhibitor
174	95.16	Compound	BRD-A71459254	cymarin	ATPase inhibitor
177	95.14	Compound	BRD-K54095730	CMPD-1	p38 MAPK inhibitor
178	95.14	Compound	BRD-K37798499	etoposide	Topoisomerase inhibitor
179	95.14	Compound	BRD-K15600710	obatoclax	BCL inhibitor
180	95.14	Compound	BRD-A06352418	terfenadine	Histamine receptor antagonist
185	95	Compound	BRD-A15914070	4-hydroxy-2-nonenal	Cytotoxic lipid peroxidation product
16	99.61	Gene knock-down	CGS001-5682	PSMA1	Proteasome subunits
24	99.19	Gene knock-down	CGS001-5707	PSMD1	Proteasome subunits
29	99.08	Gene knock-down	CGS001-5690	PSMB2	Proteasome subunits
30	99.08	Gene knock-down	CGS001-5684	PSMA3	Proteasome subunits
41	98.66	Gene knock-down	CGS001-5709	PSMD3	Proteasome (prosome. macropain) subunits
47	98.49	Gene knock-down	CGS001-5693	PSMB5	Proteasome subunits
62	98.24	Gene knock-down	CGS001-7415	VCP	ATPases / AAA-type
78	97.99	Gene knock-down	CGS001-5689	PSMB1	Proteasome subunits
90	97.46	Gene knock-down	CGS001-3309	HSPA5	Heat shock proteins / HSP70
91	97.46	Gene knock-down	CGS001-27243	CHMP2A	Charged multivesicular body proteins
97	97.32	Gene knock-down	CGS001-7316	UBC	-
103	97.22	Gene knock-down	CGS001-5708	PSMD2	Proteasome subunits
104	97.21	Gene knock-down	CGS001-8764	TNFRSF14	Tumour necrosis factor (TNF) receptor family
111	96.9	Gene knock-down	CGS001-11331	PHB2	-
115	96.83	Gene knock-down	CGS001-1054	CEBPG	basic leucine zipper proteins
120	96.72	Gene knock-down	CGS001-8891	EIF2B3	-
129	96.55	Gene knock-down	CGS001-10972	TMED10	-
150	95.78	Gene knock-down	CGS001-146540	ZNF785	Zinc fingers. C2H2-type
155	95.53	Gene knock-down	CGS001-23451	SF3B1	-
158	95.49	Gene knock-down	CGS001-8894	EIF2S2	Serine/threonine phosphatases / Protein phosphatase 1. regulatory subunits
159	95.49	Gene knock-down	CGS001-1314	COPA	Endogenous ligands
160	95.42	Gene knock-down	CGS001-5688	PSMA7	Proteasome subunits
163	95.39	Gene knock-down	CGS001-64241	ABCG8	ATP binding cassette transporters / subfamily G
164	95.38	Gene knock-down	CGS001-6818	SULT1A3	Sulfotransferases. cytosolic
165	95.38	Gene knock-down	CGS001-5411	PNN	-
166	95.38	Gene knock-down	CGS001-23244	PDS5A	-
167	95.31	Gene knock-down	CGS001-4664	NAB1	-
168	95.28	Gene knock-down	CGS001-10482	NXF1	-
170	95.17	Gene knock-down	CGS001-6605	SMARCE1	-
181	95.07	Gene knock-down	CGS001-5947	RBP1	Fatty acid binding protein family
183	95.03	Gene knock-down	CGS001-9318	COPS2	-
