# Supplementary material for: Integrated transcriptomics- and structure-based drug repositioning identifies drugs with proteasome inhibitor properties
Source: Sci Rep. 2024 Aug 13;14:18772. doi: 10.1038/s41598-024-69465-6 (PMC11322189; doi:10.1038/s41598-024-69465-6)
Supplement: Supplementary file 16 — Supplementary Table S6. [file 41598_2024_69465_MOESM16_ESM.pdf]

Supplementary Table 6. Molecular docking score to β5 site for known PIs (black) and identified virtual PIs (blue).

**Compounds**	**Docking score**
Mln-2238	-6.728
Kinetin-Riboside (Brd-K94325918)	-6.398
Bortezomib	-6.387
Epoxomicin	-5.866
Bcl2-Inhibitor (Brd-K39111395)	-5.812
Carfilzomib	-5.779
Thapsigargin (Brd-A62809825)	-5.19
Oprozomib	-5.025
Mg-132	-4.900
Delanzomib	-4.790
Mln-9708	-4.622
Manumycin-A (Brd-K78599730)	-4.613
Heliomycin (Brd-K64517075)	-4.553
Ag-592 (Brd-K89930444)	-4.408
Puromycin (Brd-A28970875)	-4.338
Tegaserod (Brd-K21806131)	-4.016
Pi-1840	-3.965
Celastrol	-2.487
V3-23	-1.166
